# Interspecific and intraspecific gene variability in a 1-Mb region containing the highest density of NBS-LRR genes found in the melon genome

**DOI:** 10.1186/1471-2164-15-1131

**Published:** 2014-12-17

**Authors:** Víctor M González, Núria Aventín, Emilio Centeno, Pere Puigdomènech

**Affiliations:** Molecular Genetics Department, Center for Research in Agricultural Genomics CRAG (CSIC-IRTA-UAB-UB), Campus UAB, Edifici CRAG, Bellaterra (Cerdanyola del Vallès), 08193 Barcelona, Spain; Bioinformatics Core Unit, Center for Research in Agricultural Genomics CRAG (CSIC-IRTA-UAB-UB), Campus UAB, Edifici CRAG, Bellaterra (Cerdanyola del Vallès), 08193 Barcelona, Spain

## Abstract

**Background:**

Plant NBS-LRR -resistance genes tend to be found in clusters, which have been shown to be hot spots of genome variability. In melon, half of the 81 predicted NBS-LRR genes group in nine clusters, and a 1 Mb region on linkage group V contains the highest density of R-genes and presence/absence gene polymorphisms found in the melon genome. This region is known to contain the locus of *Vat*, an agronomically important gene that confers resistance to aphids. However, the presence of duplications makes the sequencing and annotation of R-gene clusters difficult, usually resulting in multi-gapped sequences with higher than average errors.

**Results:**

A 1-Mb sequence that contains the largest NBS-LRR gene cluster found in melon was improved using a strategy that combines Illumina paired-end mapping and PCR-based gap closing. Unknown sequence was decreased by 70% while about 3,000 SNPs and small indels were corrected. As a result, the annotations of 18 of a total of 23 NBS-LRR genes found in this region were modified, including additional coding sequences, amino acid changes, correction of splicing boundaries, or fussion of ORFs in common transcription units. A phylogeny analysis of the R-genes and their comparison with syntenic sequences in other cucurbits point to a pattern of local gene amplifications since the diversification of cucurbits from other families, and through speciation within the family. A candidate *Vat* gene is proposed based on the sequence similarity between a reported *Vat* gene from a Korean melon cultivar and a sequence fragment previously absent in the unrefined sequence.

**Conclusions:**

A sequence refinement strategy allowed substantial improvement of a 1 Mb fragment of the melon genome and the re-annotation of the largest cluster of NBS-LRR gene homologues found in melon. Analysis of the cluster revealed that resistance genes have been produced by sequence duplication in adjacent genome locations since the divergence of cucurbits from other close families, and through the process of speciation within the family a candidate *Vat* gene was also identified using sequence previously unavailable, which demonstrates the advantages of genome assembly refinements when analyzing complex regions such as those containing clusters of highly similar genes.

**Electronic supplementary material:**

The online version of this article (doi:10.1186/1471-2164-15-1131) contains supplementary material, which is available to authorized users.

## Background

The survival of plants in any given environment relies on the ability of the populations to develop appropriate responses to biotic and abiotic stresses. For this reason, complex coordinated systems of responses to these stresses have evolved in different plant species. Understanding of these systems is one of the main goals of plant biology and this information can be crucial for breeding genes related to stress resistance in crop plants.

From this perspective, the species of the family Cucurbitaceae are of special interest for a number of reasons. They form a group of plant species with genomes of intermediate size (between 300 and 450 Mbases), they are mostly diploid, and they have not undergone large genome duplications apart from those which occurred with the original diversification of flowering plants. They also have a particular system of vascular development that has been related to defense mechanisms specific to these species. In addition, they are of significant economic interest and breeding of the cucurbit species is active within the seed industry.

Most disease resistance genes in plants encode nucleotide-binding site leucine rich repeat (NBS-LRR) proteins, a populated family that can be encoded by hundreds of genes per genome [1]. One of the features of cucurbit genomes that has been revealed by sequencing three representative genomes is that the number of defense-related genes, particularly those belonging to the NBS-LRR protein family, appears to be significant lower when compared to other plant species [2–4]. It has recently been proposed that the content of NBS-LRR genes in plant species is correlated with that of miRNAs directed to them as a mechanism to control their levels of expression [5, 6]. It has also been shown that these genes tend to be present in clusters, which appear to be hot spots of genomic variability due to the high level of presence/absence gene variation (PAV), detected when comparing genomes of related species or genome sequences within the species, which make these regions good candidates for comparative genomics and phylogeny analysis [7–14]. In melon, half of the 81 predicted NBS-LRR genes group in nine clusters, with a region that spans 1 Mb of linkage group V, containing the highest density of R-genes (28 NBS-LRR amongst them) and also the highest concentration of PAV polymorphisms found in the melon genome [2, 14–18]. This region is also known to include the *locus* of the agronomically important *Vat* gene that confers aphid resistance to some melon cultivars [19, 20].

While recent studies have produced genome-wide analysis of NBS-LRR genes in cucurbits, these efforts have been limited by the quality and completeness of the available sequences [21, 22]. The presence of multiple duplicated genes in NBS-LRR clusters makes these regions challenging sequencing targets. Indeed, it has been proposed that the available annotation of a high proportion of melon R-genes is likely wrong, due to a combination of sequence quality issues and automatic gene annotation [22].

This article presents a detailed analysis of the structure of the largest NBS-LRR gene cluster found in melon, together with comparisons with the syntenic regions in cucumber and watermelon and also between different melon varieties. As a required preliminary step, however, a comprehensive refinement step was used to close unsequenced gaps and to correct sequencing errors in order to improve the available sequence and gene annotations.

## Results and discussion

### Sequence refinement of a 1 Mb fragment of the melon genome

The aim of this research was to carry out a detailed analysis of the genomic variability of a cluster of gene sequences putatively involved in resistance to pathogens (R-genes). Found in melon linkage group 5, they are known to be highly polymorphic at the intra- and interspecific levels [14]. However, the presence of multiple, highly similar genes in a relatively small region makes it difficult to obtain an accurate and complete sequence by high-throughput new-generation technologies. Indeed, a cursory view of the reference genome sequence and the annotation of genes in this region shows multiple sequence gaps, many of which are in ORFs causing partial annotations, fragments from the same gene annotated as independent genes, etc. Also, by aligning re-sequencing reads to the reference genome, small indels and homopolymer errors on coding regions are visible.

A recent study on R-genes in cucurbits concluded that a large proportion of R-genes are pseudogenes caused by large deletions, frameshift indels, and nonsense mutations [22]. The same study reported that the annotations of nearly half the melon R-genes were likely wrong, often due to the forced annotation of extra introns to avoid premature stop codons or frameshift mutations. However, several of these putative melon pseudogenes could be just partially annotated due to sequence gaps or single nucleotide errors resulting in false stop codons. Considerable refinement of the one-megabase fragment that contains the melon R-cluster was clearly necessary before further studies could be done on this region.

The sequence improvement was in two stages. First, re-sequencing by Illumina paired-end reads was used to close gaps and correct false SNPs and small indels; secondly, a PCR experimental approach was used to close still unresolved gaps, particularly those affecting ORFs.

About thirty million pairs of Illumina reads have been previously obtained from DHL92, the double haploid line source of the melon reference assembly [2]. It was our aim to evaluate how these reads could help to improve the reference sequence (which had been assembled using a different set of 454 and Illumina reads) by aligning them to the reference sequence in order to extract information regarding gaps and sequence errors. Few publicly available software utilities address either gap closing or sequence correction using mapping alignments. The PAGIT toolkit does in fact deal with both aspects but, being designed primarily to be used with small size genomes such as that of bacteria, it is not directly applicable to larger genomes [23]. Therefore, a specific method of applying PAGIT to our particular needs was devised. A detailed description of the procedure is given in the Methods section and a schematic representation in Additional file 1: Figure S1.

It is important to note that, although the final goal was the refinement of *ca*. 1 Mb sequence, it was deemed necessary to work with the complete reference genome and whole set of Illumina reads. Working only with those reads that map to the selected region would filter out reads that map to absent sequences (particularly those in gaps) and, therefore, essential for closing gap purposes. But mapping the whole set of re-sequencing reads to only a small fraction of the genome normally results in a high number of misalignments, because reads that map to different but highly similar regions align to the same region when only one of those regions is available.

The metrics of the melon reference assembly (CM3.5) and the improved, post-PAGIT, version are compared in Table 1. While the number of scaffolds remained unchanged, many contigs (32%) were shown to be redundant based on identity to sequences in other, larger, contigs or scaffolds, and they were removed from the final version. The processed assembly contains *ca*. 1.2 Mb of additional sequence, roughly equal to the number of uncertain nucleotides (hereafter referred to as ‘Ns’) removed from it. In all, only 0.3% of the original amount of Ns was deleted while *ca*. 8,000 stretches of Ns (henceforth, referred to as ‘N-stretches’) were solved (28%), which means that, as expected, mainly short gaps were closed. Although the percentage reduction of N-stretches was not impressive, the number of estimated corrected errors was high (54,000 SNPs and 168,000 1-3 bp insertion-deletion errors). Also, the number of Illumina reads remaining unmapped after aligning reads against the reference genome was reduced by 25% in the refined sequence, a quality improvement indicator.

**Table 1:** **Melon genome sequence improvement metrics**

	Whole genome		
	v3.5		Post-PAGIT^1^
**Scaffolds**	1,599		1,599
**Contigs**	29,865		20,406
**Length (bp)**	375,485,313		375,516,019
**Length, no Ns (bp)** ^**2**^	336,097,046		337,325,315
**Stretches of Ns** ^**3**^ **(#N > 1)**	28,306		20,295
**Stretches of Ns** ^**3**^ **(#N = 20)** ^**4**^	9,133		2,581
**Number of Ns**	39,388,267 (10.5%)		38,190,704 (10.2%)
**Length of scaffolds (bp)**	361,983,232		362,182,953
**Length of scaffolds, no Ns (bp)** ^**2**^	322,595,151		323,992,316
**Length of contigs (bp)**	13,502,081		13,333,066
**Length of contigs, no Ns (bp)** ^**2**^	13,501,895		13,332,999
**Illumina unmapped reads (%)** ^**5**^	18.3		13.9
**Corrected sequence errors** ^**6**^			
** 1 bp substitution errors**			53.771
** 1-3 bp insertion errors**			46.659
** 1-3 bp deletion errors**			121.178
	**4235-4331 fragment**		
	**v3.5**	**Post-PAGIT** ^**1**^	**Final**
**Length (bp)**	1,118,599	1,120,734	1,066,373
**Stretches of Ns (#N > 1)**	120	84	27
**Number of Ns**	202,018 (18%)	192,656 (17.1%)	57,617 (5.4%)
**Average N-stretch size (bp)**	1,656	2,007	1,859

^1^Immediately after prinseq processing step performed following iCORN/redundancy removal steps.

^2^Only A, T, C, and G, not N, are counted.

^3^Contiguous strings of Ns.

^4^Most 20 bp-long gaps produced by the assembler while building the reference assembly mark adjacent contigs that overlap but for the presence of short, low quality/wrong sequences at their ends. These gaps are good targets for IMAGE, the software responsible for closing gaps in the PAGIT toolkit, which removes end-sequence from contigs while attempting to extend them with re-sequencing data and to overlap adjacent contigs.

^5^Unmapped reads after aligning the set of DHL92 high-quality Illumina PE (see Methods section).

^6^Based on the output reports of the iCORN software.

At this stage, the 1 Mb genomic fragment comprising genes MELO3C004235 to MELO3C004331 was selected for further improvement. This region includes the cluster of NBS-LRR genes [MELO3C004258-MELO3C004324] together with other genes putatively involved in defence responses, and has been shown to be a hot-spot of genomic variability across several melon cultivars [14]. The procedure to close unresolved gaps and correct any remaining sequence errors detected is described in detail in the Methods section and included the use of previously available BAC sequence information [14, 15, 17, 18], PCR-aided gap closing, and manual correction of the final sequence by visually inspecting the mapping-alignments of the re-sequencing reads. The final, improved sequence comprising genes MELO3C004235 to MELO3C004331 is given in Additional file 2: File S1.

**Figure 1:**
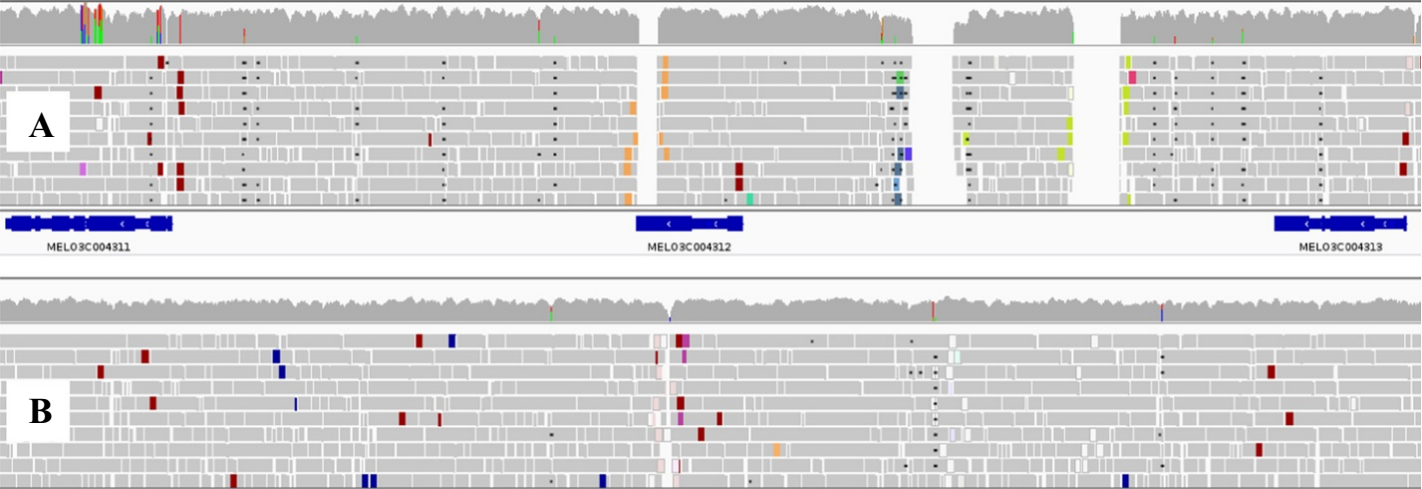
**A graphical view of the melon genome sequence improvement. A** Map of the DHL92 Illumina PE reads against the reference genome (DHL92, CM3.5 version) on the region containing genes MELO3C004311 to MELO3C004313. Three major gaps mark the position of stretches of Ns in the reference genome, one of which causes gene MELO3C004312 to be partially annotated. Colored vertical lines above the coverage track signal discrepancies between the aligned reads and the reference genome. **B** Mapping of DHL92 Illumina PE reads against the improved reference genome. The main gaps were closed and the number of discrepancy lines greatly reduced. Figure produced using IGV software.

The metrics of the refined sequence as compared to CM3.5 and the post-PAGIT versions are given in Table 1. The number of Ns in the original 1 Mb sequence was 18%, nearly twice the average value in the melon genome, which points to the complexity of this region as a sequencing target. This figure decreased to 17% in the post-PAGIT version but dropped to just 5% in the final sequence, due to the successful outcome of the PCR approach. The number of N-stretches was also significantly reduced (from 120 to just 27). Figure 1 gives a graphical view of mapped alignments against CM3.5 and the refined sequence, with some examples of gap closing and SNP correction.

Once the final 1 Mb sequence was available, the CDS of all NBS-LRR in that region were re-annotated as described in the Methods section. Table 2 summarizes how the changes affected gene annotation. Only five sequences of a total of 23 genes remained unchanged. Gap closing resulted in additional coding sequence for ten genes, while nucleotide corrections occurred in exons of four genes. As expected, most of these changes were related to homopolymer corrections. Other important changes were that the previous genes MELO3C004302 and MELO3C004310 do in fact belong to the MELO3C004303 ORF and MELO3C004309 ORF, respectively, and MELO3C004304 probably represents a 5′ fragment of gene MELO3C004302-4303, although no satisfactory ORF could be found joining both fragments. Apart from the 23 annotated NBS-LRR ORFs, five previously predicted genes (MELO3C004261, MELO3C0042665, MELO3C004295, MELO3C004304, and MELO3C004319) were probably pseudogenes, due to their short lengths, and were not analyzed further. The protein sequences of the newly annotated genes can be found in Additional file 3: File S2.

**Table 2:** **Effects of sequence improvement on the annotation of R-genes**

Gene Id^1^	Class^2^	Sequence changes^3^	Notes
**MELO3C004258**	TN	No	3′-partial
**MELO3C004259**	TNL	(T)_14_ -- > (T)_11_ (I)	
		(T)_10_ -- > (T)_9_ (I)	
**MELO3C004260**	TN	No	3′-partial
**MELO3C004262**	TNL	(T)_7_ -- > (T)_8_ (I)	Additional a.a.s (gap closed)
		(T)_4_ -- > (T)_3_ (I)	
		1 T deleted (I)	
		20 N - > [559 bp (I + E) + 30 N (in poly(AT), I)]	
**MELO3C004266**	TNL	(N)_5054_ gap closed (E)	Additional a.a.s (gap closed)
		(CTATAATTG) -- > (CATTG) (E)	
		(TGTCGTTTA) -- > (T_9_GCGTTA) (I)	
		(TTATA_9_T) -- > (TA_9_T) (I)	
		(A_6_CA_5_G) -- > (A_7_CA_6_G) (I)	
**MELO3C004288**	N	No	5′- and 3′-partial
**MELO3C004289**	TNL	(N)_767_ gap closed (E)	Additional a.a.s (gap closed)
		(A)_20_ -- > (A)_26_ (I)	
		(A)_16_ -- > (A)_24_ (I)	
		(T)_7_(A)_11_ -- > (T)_8_(A)_1o_ (I)	
**MELO3C004290**	TN	(N)_20_ - > (N)_1_ (E)	3′-partial
**MELO3C004291**	TN	(N)_2153_ gap closed (E)	
		(CA_5_CCT_12_) -- > (CAAT_10_) (I)	3′-partial
		(CTTTTA_13_) -- > (CTTTA_11_) (I)	Additional a.a.s (gap closed)
		(GAAAG) -- > (GAG) (E)	
**MELO3C004292**	TNL	(A)_14_ -- > (A)_7_ (I)	
		(AA) deleted (I)	
		(A)_21_ -- > (A)_15_ (I)	
**MELO3C004294**	TNL	(AA) deleted (I)	
**MELO3C004301**	TNL	(N)_73_ - > (N)_28_ (I)	(N)_28_ in poly(TA) (intron)
		(TACCA_28_GAAA) -- > (TCCA_28_GA) (I)	
		(A_10_CCAAAG) -- > (A_13_CCAAAAG) (I)	
		(AAAAGTTTA_6_) -- > (AAAAGTTTCA_6_) (I)	
		(GAAAGTATA_5_TCA) -- > (GAAAGTATAAATCA) (I)	
		(TTA_10_TTTGAAAA) -- > (A_11_TGAAAA) (I)	
**MELO3C004302-4303** ^**4**^	NL	(CTTGAATGAAACTTA) -- > (CTTGAAC) (E)	5′-partial^5^
		(GTGAAACTTA_11_CATGG) -- > (GTA_8_CATGG) (I)	
		(TACAATT) -- > (TACTT) (E)	
		(CTA_11_CAT) -- > (CTA_8_CAT) (E)	
**MELO3C004309-4310** ^**6**^	TNL	(N)_78_ gap closed (I)	
		(GTTCCGTATTCTAATTAT_4_ACT_3_) -- > (GTCCGATCAATAT_3_ACT) (I)	
		(TAT_6_C) -- > (TATTC) (E)	
**MELO3C004311**	TN	(N)_30_ gap closed (E)	3′-partial
		(GA_4_TTTACT) -- > (GA_5_TACT) (I)	
**MELO3C004312**	TN	(N)_468_ gap closed (introns + exons)	3′-partial
			Additional a.a.s (gap closed)
**MELO3C004313**	TN	(N)_20_ gap closed (E)	3′-partial
			Additional a.a.s (gap closed)
**MELO3C004317**	CNL	(N)_1414_ gap closed (E + I)	Additional a.a.s (gap closed)
		(N)_4442_ gap closed (exons + introns)	
**MELO3C004318**	CN	(N)_4962_ gap closed (exons + introns)	3′-partial
			Additional a.a.s (gap closed)
**MELO3C004320**	NL	(N)_4489_ -- > (N)_2460_ (introns + exon)	Add. a.a.s (gap partially solved)
**MELO3C004321**	CNL	(N)_3747_ gap closed (E)	Additional a.a.s (gap closed)
**MELO3C004323**	CN	No	3′-partial
**MELO3C004324**	NL	No	

^1^MELO3C004261, MELO3C004265, MELO3C004295, MELO3C004304, MELO3C004319 are ORFs that code for, respectively, 141-, 220-, 98-, 109-, and 233-aa peptides with homology to TNL proteins. Due to their short length, they are most probably not R-genes and, therefore, were not considered for further analysis.

^2^TN: TIR-NBS; TNL: TIR-NBS-LRR; N: NBS; NL: NBS-LRR; CNL: CC-NBS-LRR; CN: CC-NBS.

^3^Sequence changes between assemblies CM3.5 and CM3.6.1 in each R-gene region (start to stop codon, including introns). I, intron; E, exon.

^4^Previous MELO3C004302 gene does belong to the MELO3C004303 ORF, as deduced by sequence comparison with other TNL R-proteins.

^5^Gene MELO3C004304 codes for a 109-aa peptide with a TIR domain, and may represent a 5′ fragment of gene MELO3C004302-4303.

^6^Previous MELO3C004310 gene does belong to the MELO3C004309 ORF, as deduced by sequence comparison with other TNL R-proteins.

To assess sequence improvement, specifically that related to error correction, the number of genetic variants (SNPs and indels) found when mapping DHL92 re-sequencing reads to CM3.5 and the improved region was calculated. Re-sequencing reads and reference sequences belonged to the same cultivar (DHL92) so the expected number of variants was low, all the more since DHL92 is a double haploid line. Therefore, and as a contrasting control, the variant analysis was also performed using previously available re-sequencing data from the distant melon variety cultivar C-836, a *Cucumis melo* ssp. *agrestis* accession from Cape Verde [14]. These results are shown in Table 3.

**Table 3:** **Genetic variants and their effects on genes in melon cultivars DHL92 and C-836**

	DHL92^1^				C-836^2^			
	Whole genome		R-genes region		Whole genome		R-genes region	
	CM3.5	Improved	CM3.5^a^	Improved^b^	CM3.5	Improved	CM3.5^a^	CM3.6.1^b^
**Changes/kb**	1	0.5	4.1	0.25	12.2	12	8.9	9.5
**Variants**	365,012	193,146	3,085	180	4,482,184	4,455,971	6,761	7,219
**SNPs**								
** Homo**	18,606	15,867	2,106	21	3,463,344	3,488,405	5,311	5,631
** Hetero**	77,841	63,846	261	112	424,469	416,645	522	685
**Insertions**								
** Homo**	90,809	45,563	254	31	244,983	242,498	390	415
** Hetero**	1,992	2,115	12	2	11,240	12,037	16	23
**Deletions**								
** Homo**	172,138	62,162	429	7	323,714	271,219	510	442
** Hetero**	3,570	3,576	23	7	14,380	15,136	12	23
**Effects in genes**								
** Missense**	1,469	-^c^	125^d^	2^d^	62,321	-^c^	393^d^	520^d^
** Nonsense**	54	-^c^	4^d^	0^d^	1,461	-^c^	2^d^	6^d^
** Silent**	934	-^c^	97^d^	5^d^	62,200	-^c^	210^d^	249^d^
** Exons**	4,317	-^c^	232^d^	7^d^	131,515	-^c^	625^d^	800^d^
** Introns**	50,688	-^c^	118^d^	12^d^	526,990	-^c^	309^d^	496^d^
** Splice sites**	700	-^c^	5^d^	0^d^	8,809	-^c^	8^d^	15^d^

^1^DHL92 Illumina PE reads mapped either to the melon reference assembly (CM3.5) or to the improved sequence.

^2^C-836 Illumina PE reads mapped either to the melon reference assembly (CM3.5) or to the improved sequence.

^a^Region comprising MELO3C004258 to MELO3C004324 genes (CM3.5, scaffold0003: 5,478,521 - 6,236,414).

^b^Region comprising MELO3C004258 to MELO3C004324 genes (improved scaffold0003: 5,478,926 - 6,186,068).

^c^Gene annotation not available.

^d^Only TIR-NBS-LRR and CC-NBS-LRR genes are considered.

The number of changes (SNPs and indels) predicted when mapping DHL92 Illumina reads against CM3.5 was 1/kb, a figure reduced by half when mapping was against the improved sequence. As a reference, mapping of C-836 reads against CM3.5 produced 12 changes/kb. Interestingly, the number of changes in the 1 Mb region using CM3.5 as a reference was 4/kb, four times higher than the average genome value, which may be a result of the bad quality of this particular region compared to the average assembly quality. This figure dropped to 0.25 changes/kb in the improved 1 Mb region. Significantly, the number of changes found in exons of NBS-LRR genes in the 1 Mb regions dropped from 232 in CM3.5 (of which 130 cause missense and nonsense mutations) to only seven in the improved region. It is clear that, even without gap closing, the refined version is a much better source for gene prediction than the original one.

### Analysis of gene sequences in the NBS-LRR cluster and comparison with other cucurbits

**Figure 2:**
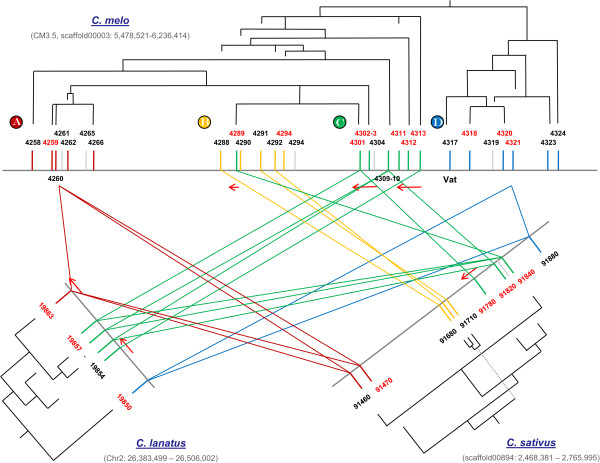
**Clusters of R-genes in the MELO04258-MELO3C004324 region, phylogeny relationships, and synteny with cucumber and watermelon.** Melon genes, represented by vertical lines accompanied by gene Ids, are grouped in four sub-clusters based on proximity: TIR-NBS-LRR genes in clusters **A** (red lines), **B** (yellow lines), and **C** (green lines); CC-NBS-LRR genes in cluster **D** (blue lines). Syntenic genes in cucumber and watermelon are in the same colour as their melon orthologues. Colored lines connect pairs of genes in two different species showing the highest protein identity/blastp e-values; however, when the genes in a cluster are so similar as to unequivocally establish one-to-one orthology relationships, a colored line connects a gene to a cluster of genes instead. ORFs with homology to NBS-LRR R-genes but too short as to be considered true genes are in grey. Gene Ids in red indicate putatively expressed genes (on the basis of information from EST databases). Direction of transcription of all genes is left to right, but for the few genes marked with red arrows. For each species, phylogeny relationships based on the alignment of the conserved NBS regions are also shown. Note that branch lengths have no phylogeny meaning due to the original trees having been distorted to project them on the actual disposition of genes on the genome.

The highly conserved NBS regions of the annotated R-genes were used for phylogenetic analysis. Their sequences are given in Additional file 4: File S3. A phylogeny tree was built and drawn alongside a schematic representation of the genomic distribution of the NBS-LRR genes (see Figure 2). Based on proximity criteria, the cluster of R-genes can be subdivided into four regions where the one marked D in Figure 2 corresponds to a cluster of coiled-coil NBS-LRR while A-C clusters contain TIR-domain NBS-LRR proteins. There is good correlation between the topology of the phylogeny tree and the clustering and distribution of genes along the genome, indicating a pattern of local gene amplifications. The transcription direction of the genes was also consistent with this picture (Figure 2).

In order to investigate further the generation of gene duplications within the Cucurbitaceae family, the published genome sequences of two other cucurbits, cucumber and watermelon, were analyzed for the correspondent syntenic regions [4, 24, 25]. The sizes of the three sequenced cucurbit genomes were comparable but significantly different, with that of melon being the largest (450 Mb), followed by watermelon (425 Mb) and cucumber (360 Mb). It has already been reported that the region containing the NBS-LRR cluster in cucumber is shorter than the homologous region in melon [14].Phylogeny trees of the cucumber and watermelon R-genes were generated and drawn alongside the correspondent genomic distribution of genes, and orthologous relationships established between melon, cucumber, and watermelon R-genes. As shown in Figure 2, clusters A, C and D are present in the three cucurbits, although they appear to have undergone different amplification processes. In melon, there are six genes in cluster D, but only one in the other two species. Similarly, while melon has five genes in cluster A, there are only one and two in, respectively, watermelon and cucumber, and cluster C contains six genes in melon but 3–4 in the other genomes. Finally, cluster B appears to be absent from watermelon, while melon contains six genes and cucumber only three in this cluster.

The CC NBS-LRR cluster appears to have multiplied in melon from a common single gene present in all cucurbits. In the other cases, complex duplication events have occurred, although it seems clear that major amplification processes have taken place in melon since its divergence from the other species. Similarly, R-gene duplications probably occurred after the divergence of the *Citrullus* and *Cucumis* genera. Similar analyses in species in the *Rosaceae* family, relatively close to cucurbits in evolutionary terms, show that the syntenic regions contain only one (strawberry, cluster A) or two genes (peach, cluster C) [6]. Therefore, a general pattern of NBS-LRR duplications in the analyzed region seems to have occurred at different stages since the divergence of the ancestral cucurbit and through speciation and diversification within the family.

Finally, expression data from cucurbit unigene databases show that at least 50% of the annotated R-genes are probably expressed in some circumstances, with all four clusters A-D containing at least one such gene (Table 4) [24].

**Table 4:** **Expression data support of R-genes**

Gene	ICUGI Gene^a^	ICUGI Unigene	Cultivar	Library
**Cucumber**				
91460.1	Csa2M022790.1	-		
91470.1	Csa2M022270.1	CU122153	Vlaspik	Fruit (mixed, 1–50 DAP)
		CU171360	WI 1983H	
91680.1	Csa2M021540.1	-		
91690.1	Csa2M021520.1	-		
91710.1	Csa2M021510.1	-		
91780.1	Csa2M020940.1	CU141826	WI 1983H	Hermaphrodite flower
		CU173837	WI 1983H	Hermaphrodite flower
91820.1	Csa2M020890.1	CU134688	WI 1983G	Gynoecious flower
91840.1	Csa2M020870.1	CU162592	WI 1983H	Hermaphrodite flower
91880.1	Csa2M014830.1	-		
**Melon**				
MELO3C004258		-		
MELO3C004259		MU66045	T-111	Callus
MELO3C004260		-		
MELO3C004262		-		
MELO3C004266		-		
MELO3C004288		-		
MELO3C004289		MU63434	PI161375	Callus
MELO3C004290		-		
MELO3C004291		-		
MELO3C004292		-		
MELO3C004294		MU53509	pat81	Root (healthy)
				Root (infected, *M. cannonballus*)
MELO3C004295		-		
MELO3C004301		MU66172	T-111	Callus
MELO3C004302-3		MU53927	PI161375	Callus
MELO3C004309-10		-		
MELO3C004311		MU43621	PI161375	Callus
			Cantaloupe C-35	CMV Cotyledon infected
		MU48550	PI161375	Callus
MELO3C004312		MU52721	PI161375	Callus
			Dulce	Mixed Fruit (4 devel. stages)
		MU54890	T-111	Fruit (15 DAP)
MELO3C004313		MU67511	Védrantais	Callus
MELO3C004317		-		
MELO3C004318		MU61424	Cantaloupe C-35	Healthy leaf
MELO3C004319		-		
MELO3C004320		MU55681	T-111	Fruit (46 DAP)
MELO3C004321		MU63490	PI161375	Callus
MELO3C004323		-		
MELO3C004324		-		
**Watermelon**				
Cla019863		WMU41608	97103	Fruit (10 DAP)
		WMU77867	97103	Fruit (34 DAP)
		WMU45091	97103	Fruit (18 DAP) & Fruit (26 DAP)
Cla019857		WMU48640	97103	Fruit (10 DAP)
Cla019856		-		
Cla019855		-		
Cla019854		-		
Cla019850		WMU79003	Illinwake Red	Norm. and subst. library
				(Mix 12, 24, 36 DAP flesh fruit; driver: leaf)

^a^Cucumber gene annotation used is taken from Phytozome (Gy14 cultivar draft genome) while the genome assembly deposited at ICUGI is that of the cucumber 9930 inbred line.

### Comparison of the NBS-LRR gene sequences in melon varieties

It has already been shown that clusters of R-genes are hotspots of presence/absence of gene variability [11]. The melon NBS-LRR cluster region here analyzed, which shows the highest density of melon stress-response genes, also contains the highest concentration of PAV polymorphisms, detected by comparing the genome sequences of five melon cultivars [14]. In particular, while clusters B and D had very high levels of PAV, with nearly all genes in these regions probably absent from at least one of the cultivars analyzed, clusters A and C were relatively less affected by this kind of variability.

**Figure 3:**
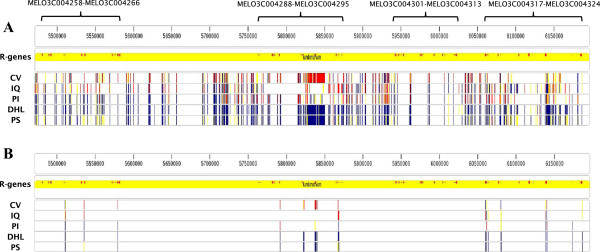
**Density of SNPs in the cluster of R-genes across five melon cultivars. A** Vertical colored lines mark the position where a SNP is detected in any of the following melon cultivars: CV: C-836, IQ: C-1012, PI: PI 161375, DHL: DHL92, PS: T-111 “Piel de Sapo”. Reference: DHL92 MELO3C004258-MELON3C004324 improved region. Blue lines: same nucleotide as in the reference; red lines: alternative nucleotide (two alleles); yellow lines: alternative nucleotide (one allele only). The “R-Genes” track shows the position of the TIR- and CC-NBS-LRR genes. **B** As in **A**, but only SNPs in the coding sequences of the R-genes are shown. Figure produced with SVAMP software.

It is interesting to note that, when computing the variability in terms of SNPs, a similar correlation with variability being higher in some groups was found. This is shown in Figure 3 where, for all five analyzed cultivars, the presence of SNPs is plotted against the clusters as total number of SNPs (Figure 3A) or SNPs in the coding regions (Figure 3B). It appears that cluster C has the lowest variability while cluster B has the highest. The comprehensive list of SNPs and small indels can be found in the Additional file 5: Table S1.

**Figure 4:**
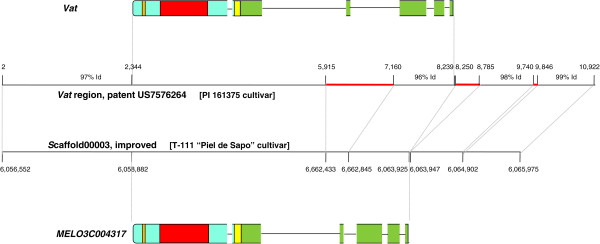
**Sequence comparison between the** ***Va*** **t region of the PI 161376 variety and gene MELO3C04317.** Dissimilar DNA regions are marked with horizontal red lines. Putative protein products are also shown: Orange sectors, CC domains; red sectors, NBS domains; yellow sectors, LRR domains; green sectors, LRR C-terminus.

One gene in the cluster is of special interest. It is the only gene to which a particular function can tentatively be attached on the basis of published information. This is the gene responsible for aphid resistance, *Vat*, an economically important gene cloned in PI 161375, one of the parentals of the DHL92 melon line, and shown to be a CC-NBS-LRR gene syntenic to those found in cluster D [19, 20, 26]. However, the unambiguous identification of the *Vat* orthologue in the CM3.5 assembly was not possible, because protein sequences of all six genes in cluster D are highly similar and due to the multiple sequence gaps in the cluster. Also, it is important to note that the published *Vat* was cloned in PI 161376 while the 1 Mb region analyzed was from T-111 “Piel de Sapo”, the other parental of DHL92. However, gene MELO3C4317, which was only partially annotated in CM3.5 due to several sequence gaps, was completely annotated in the refined version. The availability of the region immediately upstream of the gene, also missing in CM3.5, allowed its identification as the orthologue of *Vat* in DHL92 and “Piel de Sapo”, demonstrating the importance of genome draft sequence refinement. It is important to note that T-111, unlike PI 161376, is sensitive to aphid colonization. Figure 4 shows the comparison of the corresponding genome regions while Additional file 6: File S4 shows the BLASTP alignment of the protein sequences. It can be observed that the overall structure of the two genes is very similar but with a major change in the length of the third intron. There are also a number of SNPs that have an effect on the protein sequence: a total of 60 amino acid changes were observed in the 1,019 amino acid-long protein, and some of them cause significant changes in the amino acid type. Therefore both point mutations and changes in gene structure were observed and could be responsible for the lack of aphid resistance in DHL92.

## Conclusions

Important sequence changes were observed in one of the most variable regions of the melon genome, which contains the highest defense-related gene density found in this species. However, good as the quality of the current melon genome assembly is, the complexity of this region as a sequencing target, due to a high degree of internal duplication, made necessary a refinement of the available sequence prior to any detailed analysis of the cluster of resistance genes. In this work, an approach combining the use of re-sequencing Illumina data with PCR-based gap closing allowed us to reduce the amount of N-stretches by 80% and to correct about 3,000 SNPs and small indels in the region of interest.

Phylogeny analysis of the melon NBS-LRR gene cluster in the refined sequence and comparison with its syntenic counterparts in other cucurbit species allow us to conclude that new resistance genes have essentially been produced by sequence duplication in adjacent genome locations since the divergence of cucurbits from other close families, and through speciation processes within the family. Comparison of different melon cultivars indicates that diverse mechanisms that generate variability are at work in the melon genome. Presence/absence variation of genes previously described, but also single nucleotide mutations that appear to be diversely represented in different parts of the analyzed sequence, indicate that some genes may be important for specific functions but other groups may allow a higher degree of variability. These sequence changes are present in functional genes such as the *Vat* resistance gene, where a high number of point mutations and a change in the gene structure are observed between the functional and non-functional genes.

The attribution of specific functions to members of the highly populated NBS-LRR gene family is an open question in plant biology. Analysis of the different mechanisms acting on these highly variable genes may help to understand how plants adapt to different environments and provide useful strategies for plant breeding.

## Methods

### Genome improvement: Source data and pre-processing steps

The sources of the *C. melo DH*L92 genome draft assembly (CM3.5) and the Illumina resequencing read sets of DHL92 can be found in [2]. The 152-bp resequencing reads were processed using standard Illumina quality filtering followed by trimming to obtain the longest contiguous segment for which quality values were greater than 15 and to remove Illumina adapters, with all resulting reads shorter than 40 bp removed; deletion of non-paired single reads and PCR duplicates produced two final sets of paired high-quality reads. Details on the quality filtering procedures can be found in [14].

A blastn analysis using the CM3.5 assembly as subject, its contig sequences as query, and dust filtering, revealed the presence of contigs that could be considered redundant because at least 99% of each sequence was found to be at least 98% identical to sequences in other, larger, contigs or scaffolds. A total of 6,807 contigs were discarded on this basis.

### Genome improvement: Gap closing using resequencing reads

Assembly CM3.5 minus redundant contigs and the set of DHL92 high-quality paired-end sequences were used as inputs for the IMAGE software of the PAGIT toolkit [23]. As IMAGE only processes sequences with a contiguous segment of at least 300 bp without ‘N’, a total of 8,331 small contigs were saved to be used in a later step. IMAGE was launched with parameter *-kmer 61* and left to perform six iterations.

The stretches of Ns on CM3.5 had been produced during pair-end scaffolding processes and therefore their lengths represent an estimation of actual genome distances. However, an undesired effect of IMAGE is that the length of N-stretches in the processed sequences no longer bears any relation to true genome distances. To correct this, the ABACAS software in the PAGIT toolkit was used. The 14,727 contig sequences in the *Res.Image.fa* file produced during the last IMAGE iteration were saved as they did not contain stretches of Ns, while the scaffold sequences had to be split into their constituent contigs before proceeding to resize the N stretches. For this, the file *New.fa*, also produced during the last IMAGE iteration and containing the start and end coordinate positions of N-stretches in every scaffold, together with the scaffold sequences from *Res.image.fa*, were processed using a perl script to produce, for every scaffold, a multifasta file containing its contig sequences. ABACAS was then fed with the CM3.5 assembly minus redundant contigs, a multifasta file, and parameters *-p nucmer -d -n 20* to produce a scaffold sequence file with resized stretches of Ns. This was repeated for every scaffold in the assembly.

Finally, all previously unprocessed contigs (8,331 small contigs and 14,727 post-IMAGE contigs) were added to the scaffold sequences. As gaps had been closed and sequences extended, the presence of redundant contigs was again checked using the same criteria as above and 2,178 additional contigs were removed.

### Genome improvement: SNP and small indel correction using resequencing reads

The iCORN software of the PAGIT toolkit enables errors in the consensus sequence to be corrected by mapping resequencing reads to the assembly. However, as every contig/scaffold must be processed separately, it is first necessary to produce local alignments of the Illumina reads against every contig/scaffold to obtain the subset of reads that maps on the selected sequence, because mapping the whole set of reads against a particular sequence produces a high number of mapping errors.

The complete set of DHL92 Illumina high-quality sequence reads were aligned to the IMAGE-ABACAS modified assembly using the Burrows-Wheeler aligner (v0.6.1) [27]. The reference sequence was indexed using ‘bwa index’ with the ‘-a bwtsw’ flag. The two fastq files were then aligned to the reference genome and a .sam file generated using *bwa aln* and *bwa sampe* with the default parameters. The corresponding *bam* file was produced using the samtools software [28] (*samtools view -bS mapping_file.sam -T ref_genome.fa -o mapping_file.bam*) and the resulting files sorted and indexed using *samtools sort* and *samtools index*. Sub-bam files were then generated by feeding each contig or scaffold fasta header to *samtools view*, and a pair of fastq pair-end files was obtained for each contig/scaffold using the sub-bam files as input for the bam2fastq software [29]. As Illumina reads used as inputs for iCORN software must be of uniform length, a perl script was used to extend reads shorter than 152 bp (the maximum read length) by adding the required amount of Ns at 3′ to obtain reads of 152 bp in length. The quality values of the added positions were set to ‘#’ in the appropriate quality tracks.

For every contig and scaffold, the corresponding pair of fastq files, together with the IMAGE-ABACAS modified assembly, were used as input for iCORN, running four iterations with the parameters ‘insert range’ 152,645 and ‘mean insert size’ 509. After this step, contig redundancy was again checked and 476 additional contigs were removed. Lastly, prinseq software [30] was used to trim any stretch of Ns found at the end of contigs/scaffolds.

### Genome improvement: Further refinement using previously available sequences

The region on the modified scaffold00003 that contains the genes MELO3C004235 to MELO3C004331, was selected for additional improvement. As a first step, data from previously sequenced BACs that span genes MELO3C004258 (partially) to MELO3C004290 (partially) was analyzed to close unresolved gaps. Two BACs, Cm60_K17 and Cm13_J04, had been completely Sanger-sequenced but for a small gap of 32 bp which is, in fact, solved in CM3.5 [15, 17]. Cm13_J04, together with two additional BACS, Cm43_H20 and Cm14_M22, had been sequenced as part of a group of 32 BACs using 454 technology and a BAC pooling strategy [18]. In all, a contiguous sequence of ca. 300 kb containing only seven small gaps, of which two were already solved in the improved melon assembly, was available. It was considered higher quality than that of the melon genome assembly, because it had been obtained by Sanger sequencing or 454 sequencing of a small number of BACs to a high confidence degree, based on high coverage values. Therefore, the corresponding sequence in the improved assembly was replaced with the 300-kb fragment, where only five small gaps remained to be closed.

### Genome improvement: Gap closing by PCR

The MELO3C004235-MELO3C004331 region was further analyzed in search of still unresolved sequence gaps, and pairs of primers were designed flanking the selected regions using the Primer3 software in an attempt to close gaps by PCR [31].

DNA was extracted from young tender leaves of a five-week old DHL92 plant using the Nucleo-Spin Plant II (Macherey-Nagel) to give *ca*. 30 ng/μl DNA per sample. RANGER DNA Taq polymerase (Bioline) was used for PCRs. The amplified bands were analyzed on agarose gels, then purified with a QIAquick Extraction Kit (Qiagen) and sequenced. The results were added to the scaffold00003 sequence, deleting the corresponding N-stretches or reducing their lengths when the gaps could not be completely solved.

DNA from BAC clones known to span the selected gaps was used if PCR resulted in no amplification or complex band patterns when using genomic DNA, and to confirm positive results. DNA from BAC clones was obtained as described in [32]. The source of the BAC information is as follows: a minimal tiling path of eight BAC clones spanning 713 kb of CM3.5_scaffold00003, and comprising genes MELO3C004287 to MELO3C004347, previously obtained using BAC-end sequence data, a BAC-based physical map, and the melon genome reference sequence [14]. Additionally, the previously mentioned Sanger-sequenced BAC clones Cm60_K17 and Cm13_J04 span the region comprising genes MELO3C004258 to MELO3C004290, but for a gap of *ca*. 3 kb between the BACs.

### Genome improvement: Manual SNP correction

The DHL92 assembly modified up to this point was used as reference to map the complete set of DHL92 Illumina high-quality sequence reads, using the Burrows-Wheeler aligner and samtools, as described above. The sorted *.bam* file was loaded into the IGV v2.3 software [33] and mapping in the region with the MELO3C004235 to MELO3C004331 genes was inspected in detail to search for discrepancies between the reference sequence and the aligned reads. The reference was only changed when more than 75% of the reads in a given position with a coverage of four or more reads supported a different nucleotide.

### Gene annotation and R-gene phylogeny

TIR- and CC-NBS-LRR ORFs in the MELO3C004235-MELO3C004331 improved region were re-annotated using AUGUSTUS and FGENESH software tools with Arabidopsis as the model species [34, 35]. For each gene, an ORF was decided upon based on the output of both programs, the annotation of CM3.5, information from other species’ R-genes in GenBank, and the analysis of the structure of R-genes in *Cucurbitaceae* published in [22]. A GenBank annotation file was produced using Sequin [36] and, from this, a gff3 file using genbank2gff [37]. Finally, a gtf file was produced from the gff3 file using the gffread program of the cufflinks software [38].

The coordinates of the TIR, CC, NBS, and LRR domains of the predicted protein sequences were calculated using HMMER [39]. The sequences of the NBS domains were used to build a phylogeny tree of the annotated R-genes with the “One click” option of the Phylogeny.fr web service, which includes alignment with MUSCLE, curating using Gblocks, phylogeny analysis with PhyML, and tree rendering using TreeDyn [40].

### Identification of cucumber and watermelon syntenic regions

A file with the predicted proteins of the cucumber and watermelon genome assemblies and their .gff3 annotation files were downloaded, respectively, from Phytozome [25] (data as of October 23rd 2012) and ICUGI [24] (July 2nd 2013). In order to detect syntenic regions among the genomes, the MCScanX toolkit was employed [41]. The genomes were processed according to the software instructions. Briefly: for all pair of species (A, B), BLASTP was used with e-value 1e-10, database, species A proteins, and query, species B proteins. The results from these A *vs*. B and B *vs*. A analyses were combined to produced a single A-B blast file. The gff3 files were modified to comply with the program’s format specifications and then combined to produce A-B gff3 files. The combined blast and gff3 files were processed by MCScanX in search of putative homologous regions.

The nucleotide and protein sequences of genes in the cucumber and watermelon selected regions were blastn/blastp’ed against gene and protein sequences of the syntenic melon R-genes to establish orthologous relationships between all three species.

### Expression data support of R-genes

The Melon v4.0, Cucumber v3.0, and Watermelon v2.0 unigene collections in the ICUGI website were searched for expression data support for the annotated ORFs in the analyzed melon region and its syntenic counterparts in cucumber and watermelon [24]. In the case of melon, the availability of re-sequencing data of the PI161375 cultivar helped to confirm positive blastn results of melon unigenes from this variety [14]: A sorted bam file, generated as described above, was loaded into IGV tools so that the PI161375 sequence could be inferred from the aligned reads in any area of interest.

### Genetic variant annotation and effect prediction on R-genes

The whole set of DHL92 high-quality re-sequencing reads was mapped to the improved reference sequences as described above. The sorted *.bam* file was then processed with SAMtools and BCFtools to produce a variant file with detected SNPs and indels [28]:

1. samtools mpileup -Q20 -D -B -S -uf improved_reference.fa file.sorted.bam | bcftools view -N -bvcg - > file.var.raw.bcf
2. bcftools view file.var.raw.bcf | vcfutils.pl varFilter -D 42 > file.var.flt.vcf

The filtered vcf file, the improved reference sequence, and the gtf file with annotation of R-genes were processed by the SnpEff software [42] to produce an html file with statistics regarding the distribution of SNPs and indels and their effects on genes, such as amino acid changes or silent mutations. Note that only effects on the annotated R-genes were obtained as no general annotation of the improved reference was available.

For comparison, the previous procedure was repeated using CM3.5, its gene annotation file, and a filtered vcf file obtained by mapping the high-quality re-sequencing reads to CM3.5 and processed using SAMtools and BCFtools.

As a contrasting control, the variant analysis was also performed using previously available re-sequencing data from a distant melon variety: cultivar C-836, a *Cucumis melo* ssp. *agrestis* accession from Cape Verde [14]. The C-836 re-sequencing reads, subjected to the same quality filters as DHL92, were mapped to both CM3.5 and the improved sequence, and variant analysis performed as described previously.

### Density of SNPs in the cluster of R-genes across five melon cultivars

The distribution of SNPs along the clusters of NBS-LRR genes (MELOC004258-MELO3C004324 region) was visualized using the SVAMP software [43]. For comparison, previously available resequencing data of four additional melon cultivars was also included [14]. Sorted *.bam* files of all five varieties were obtained as explained above, and a multi-sample variant file also generated as above, except that all five *.sorted.bam* files were listed in the command line.

Finally, the improved reference sequence, the gff3 annotation file of R-genes in the analyzed region and the *.flt.vcf* file were loaded into SVAMP.

## Availability of supporting data

The improved melon genome sequence as well as the protein sequences of the predicted TIR- and CC-NBS-LRR genes in the improved region can be found in the supplementary material (Additional files 2 and 3).

## Electronic supplementary material

Additional file 1: Figure S1: A schematic representation of the strategy followed for sequence improvement. (PDF 280 KB)

Additional file 2: File S1: Scaffold00003:5,189,390-6,307,985 (CM3.5 coordinates), improved sequence. (PDF 1 MB)

Additional file 3: File S2: Protein sequences of the predicted TIR- and CC-NBS-LRR genes in the improved MELO3C004258-MELO3C004324 region. (PDF 29 KB)

Additional file 4: File S3: NBS domains of the predicted TIR- and CC-NBS-LRR proteins in the improved MELO3C004258-MELO3C004324 region. (PDF 18 KB)

Additional file 5: Table S1: Variant call file (vcf) with SNP and small indel distribution on the MELO3C004258-MELO3C004324 region from five melon cultivars. (XLS 4 MB)

Additional file 6: File S4: BLASTP alignment of the Vat (PI 161376 melon variety) and MELO3C004317 (DHL92 melon variety) protein sequences. (PDF 21 KB)
