# Supplementary material for: Interspecific and intraspecific gene variability in a 1-Mb region containing the highest density of NBS-LRR genes found in the melon genome
Source: BMC Genomics. 2014 Dec 17;15(1):1131. doi: 10.1186/1471-2164-15-1131 (PMC4378003; doi:10.1186/1471-2164-15-1131)
Supplement: Supplementary file 1 — Additional file 1: Figure S1: A schematic representation of the strategy followed for sequence improvement. (PDF 280 KB) [file 12864_2014_6878_MOESM1_ESM.pdf]

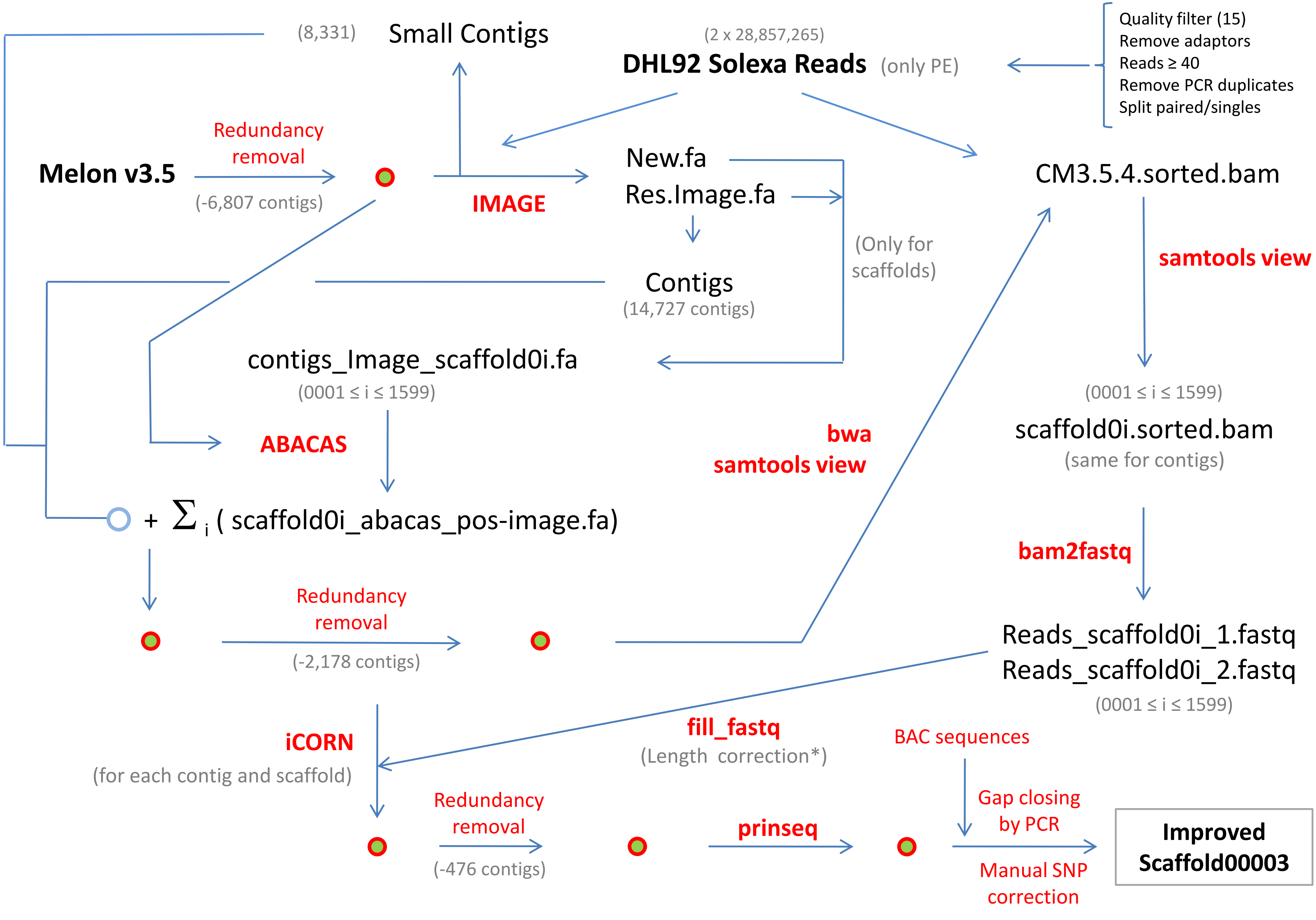

*Reads must be of uniform length. Therefore, Ns are added to a maximum length of 152 bp if needed, and the qualities are modified accordingly (adding '#').
