# Supplementary material for: Interspecific and intraspecific gene variability in a 1-Mb region containing the highest density of NBS-LRR genes found in the melon genome
Source: BMC Genomics. 2014 Dec 17;15(1):1131. doi: 10.1186/1471-2164-15-1131 (PMC4378003; doi:10.1186/1471-2164-15-1131)
Supplement: Supplementary file 2 — Additional file 2: File S1: Scaffold00003:5,189,390-6,307,985 (CM3.5 coordinates), improved sequence. (PDF 1 MB) [file 12864_2014_6878_MOESM2_ESM.pdf]

Coordinates [Scaffold00003, Assembly CM3.5] : 5,189,390-6,307,985
Coordinates [Scaffold00003, improved]: 5,190,204-6,256,576

>Genes_MELO3C004235_to_MELO3C004331_improved
CTACTTTGATAAAATCATGTATCCTCTAGGTAGTCTTATTGCAAGTGTATTCATAGCATCCCACAATAGG
GTAAGTGGAACCCAGTGAAAAGGATATTTGAAACGTGCAACATCCAAAATGAGAACCATGTCTTTACCAG
CGTGATAACCTCCAATTGGAGAAAAATGACCAGCTCCAGTTTGCTTGAGAACTTCTCTATGGTAGGAAGC
GATCACATGACGGTCATTAGAAGATGAACATGATATAACATGCTTACGAAAATCATCCACAGTGCTCTCG
TTTGTTCTTATAGCTTTCACTTTAGCACCATTGCAGCGAGCCAAATTCGCTGCCTCATCAAATGTAATAC
CGTTAGTTTTGATCTTTGCTAAAAGTTCACAACAATCTAGCATAGTCTCATCAAACCATCTCCACGGTCC
TTTCCATTTTCTACCGGGATCAATAGAAAGAGCGTTTAGAACAATAGCCAGGGTTGTGAGTCCACAATAA
GTAAGTTCTGATTGGGTTTGATGGCAAGAAATCAATTTACAAAACCCTTTCATAGTTCCATCTCCAAGCG
CCTCTTCCAAGAGACATTGGCCATCGAAAGAAGTAAAATCTATAGCGGGAGGAGAAGGAAGAACCCTCCG
ATAAAATCCTGCACCATTATGGGAATTAAGAATTGTCAAGATTTGAAATTTGAAAGACCAATCAATGGGA
GGAGAAAGTAGAAGGAAAATTCTACATAATTTGAATGTTTTTAATGGAAGTCTCCTCCTTTTGTTTCTCC
CATATTCCACCATAGTGGAAATCTTAACATAGATCGATTGAATTATCAACTTAATTACAAATAGTCTTTA
TATGTTGAATTATATTAGGTTCGAATTCACAAAAAGTATGCCTTGGCATGAACTTCAACATGTTTAACCG
CTTTAAAAAAATATATATGCGAGACAACACTTTATCTTTTTTTCTTTTTGTTGTCTTAAATCAAATCTTA
TGTGACTACTATGGACTCATTTCTTCTAAGACTTCTATTATTTATTTGGCCATAACGGTCAGATGTATAC
AAAGAACAAAGTGAGAATCAAGTGAGGGAAAAATATAAAGCTTTCTGTGAGTCAAACCTATTATATATGC
TTTAGTTAAACAAAAACTAACTAAGAAAACCTGCATTGTAAATGTACTTGAGAGAGAAACTAAGAATCAC
CGGTTACAGGAGGAGGAGTGGGATGAGACTGAGGAGGAGAGAATGGGTTGCCACCAAAGCTGTCCGTCCC
AGCTTGTTCTCTGTCATGGACTCGCGTTTCCGCCCTATCCTCTTCATTGTCCCCATCCCAAGAACCAAAA
CAACTCCCAACACTTCTCGCCATTCTCGGAATCCTCTTCCCCACCACCGAAAGATCAGATCCTGAGAGGG
CTCCCATCATATTGATCCACTGAACACCGATCAGGGGCCGTGATTTAGGAATTTGGTGGAGGATTAAAGT
GAAATAGGGTTTCCATGGATTGAGAATTGGAGTTGATGGACTTCTTAGAAGCATTATTGAAAGATGGAAA
TCTGTGAGTGAGACGAAGAAGAAGAAACAGTTATTTGTGAGTGGTTTGGAATTTACACGAAGAGAGAAAG
TTTCGGAGTATTTTAAAAAATATAGTCTTTTTAAAATTTAAATTACCATGCACATAAAAAAAAGGAAATT
TATGGTTTTGAAATTTACGAGAATTTTAGTCAAAATTAAGATTTCAAATCATAAATTTTAAATTTATTGA
TTTTATTTTTTATCCAAACATGGAAGTTCATTTAAAATCGTAATTATTTTTTCTTTAATATTTTTCTTCC
TATTTAACTATTAAACAAATTTGGCAATTTAACACTTTTTTTATTTTAATTCGATTTAATACACATTTTT
TAAACACATTAAATTTGGAAATTTTGTAGGTCTTTTTTTCCTTAATACTAACTAAGGTTTTTTCTTCCCA
TCTAACACATTTAATATAATTATTTTAGCATTTTTTTAGATATTTTGTTAATGTCATATTTTGTTAATAT
ATATCTTCCTAGCAAATTGATGAATGAAAAAAATTTGTATAATTTTGATTGACAAATAATCAAATTAAAA
TAAAAAATAAATTTAATAAGAAAAATTGAAAATTGAGAATCATAGTATTTAATATATATATATATAAAGA
AAAAAATAAAAAAATACAAGTTTGTTAATAAGGAAATAATTAAAATTAGAAAGGTGAGGTTAATTCAATA
GGAAAGATATATTTTTAGGATCGAAATTTAAAAATTGAGATATTTATTATATGTATATATATATATATAA
AATGTATGGAATCCACGGGCTAACTCATTCAAATAGTTGACCCATCTTCGGTTGTCAACACAGTTGACTT
CGCCTTCACTATCATATTTTTTTTACAATACTTTTCTCATATCTTATTTTTTACATTTACTTTTAAAATA
ACATTATTAAAAATAACTTATTTTTTTCTAACGTCATTTCGTTCCTTTTCTTTTTAATTATACTTATTTT
CTTTTGTTGCCGTACAATAATTTTCATTTTTTTAATACATTGAAATTCTACCAATTTTTAAAAAATAAAA
ATAATTATTTAAAATTGTCTTTTAAAAAACATAACTAAATATTTAGATCCAAAAGATGACTAAACTTTGT
AAAAAGCATAGTAAAATTTGATTTTAAACACATAGTAAAAAGCACTTTTATCATGTGTTCGAAAAATCAA
ATTTCTACCTATAAGTGCTTTTACACATGTTTAAGAAACATTTTAAGCACGTAGATATTATTTCGCACAT
TCTAAACTTCTACTATAGATTCCAAAATCATACATTTCAAACTTTTACTATGCGTTCCAAAATCAAACTT
TTACCTCTTCTAACTCTTTTGGAGTGCTTTTACGCATATTAGGAGTTGTAAAATAGGTATGAGGTGGTAG
CACGATAAAAAGTTGTAATTAAATCATGTTTTAAACCATCTAAATTACTTTTAAAATCACCCAACATAAA
ACCAACCAATAACAATATTCTCAAAAAGTAAAGACAAAAAAATAATCAACTATTCCATTTCGGAGCTAAG
ATCTAGTTATTAATTAAAAACTCTCTATCAGTGTTACAAATTCAAAATTTTGCTATATTTTATAAAGATC
TTTTAACTTATTTTACTATAAATGTCCTAATTTGTTTTTAAGGTAAAAGTATGAAATTGAAGTGTAATTT
TATAAATAATAATAATTATAAAAATTATGGTTGCACATTGTGCATAAAATATATATAATACATTCTAAGA
TAATGAAGGGGTTTGAGTGCAATTTACGAAGAGAAGTTGGAAAACTTTGTAAATCAAACATCCTTAGTGG
TGAATAGTGCAGTTTGAGTGTCACTTTGGCATCAAAAAGATTGCCGTGGAAGTTTCCATTATTTACACCA
TCTAAAAAGGCTTCTCCACCGCGCCATTTTCTTGCGCCAAAAAAATCACACAAACAAAAAGCAAATTGCT
CTTCGTTCTGGGACACCAATTCAAATTCACCTACTCATCATCAATGGCTTTTCGTTCTTTCCTTCTCGTG
GTTAATGATTCTACATCTTTGTATTTGTTCCGGTGAATTGGAAGCAAGGCAATGGGGGATTCGCAAGCGG
AGTCGTGTGTCTTCAGTGTTCGGGTCGTTTCCATTGATTACTATCTTGCCCCTCCAATTCCAGGCCTCGA
CATATCCTACAGTAGCTTTCAAGGTAAAGTTCACTTCTCTTTCGTACTTTGTTATGGTTTGCTTAGTTTA
CCCACTGTCGTGACGACAATGGGGTTCTTCTGATTTTCAGTTTTGGTAATCAAGGGCAGGTTTAGGATTC
TTTATGCAATTCACAATCTTAAGTCAATGGTAGAATGATAGTGGGAGATTCTATACTCTGTTTTTATTTG
GTCGTTCTCCATATGGGTACTACTTGAACTTGACCAAGGAGAGTGTAAAACATGAATGTAGATATTATTT
GCTATATGTATTTATTACCCCCTTTATTTTTTATCGAGTATGAACTTAGCTCTGGGGTTGACTAATGTAT
TATTTCGTATCATTTAATTTGAAGGCGGGAAGGTGAACGAGGTCCCTGTATTGAGAATATATGGTTCCAC

TCCAGCTGGTCAGAAAACATGCGTTCATGTTCATGGAGTAAGTCTTGTTACTGTGTCTTGAATAGGCATG
TGGTTATGTAAATGTAATGTTAGAAAATCAAGCTTTTCATAAACATTTTGATTTGTTTTTTTAGGTTTTA
CCCTATTTATATGTGCCGTGCTCAGAGATTTTATTGCTGTCCAACGACAAAGGTATGCAACTCATGAATG
TGATTATGGTTTTATTTTGGTTATCAAGCCTATTTAATGAGCATGTTACTGTTATATTTCATATTATTTG
TTTTACTTTACTTTCCTGATGACAATTTAATCTATATTGTCTGTATATAACGTTAATAACATTTATTATA
AATATCCTCTAAAGTTAATACTAACGTAAAGGTTAAATGTTTTACATGGAAAAACTAGTGAAAATATTTA
TAAATATAGCAAATAAAAAAAAATGATGGTGGGTTTTTGACATATGTTTGGTGTTAAATCATATATCGAC
CGAAGAACTTGAGTTGATGCGTGAAGGTAAATTTAATATTATATCATCTAACACTTTTCCTCACTGTGGG
TTTGAAATAGATAGAAGATTCAACAAGTGGAAATCAATAATAATTAGGGAGGAAATAATATTTTAAGGGT
TTGAATATAATACTTCTTGGACCACCTAAGGGTAAGATCAACTTGGACCACCTAAGAATTGATTCAACAT
GAGCTAAGATCAACTGGTATCTGCATGTACCCGAGGATAAGAGGTCTTTGGTTCAAATCCCATCACTCTC
AAGTTATATTACAATACATTAAAAAAAAAAAAAGAATTATAAGACTTCTTGGACCACCTAAGGGTGAGAA
GGGTTATTCGGCCATGTAGAAATCTAAGTGTGCAAGCGCATTGACGCTTCCTCTTGGTGCACACTTTACA
AGCCTTTCGCCGATTTCTCTTTACAAGATCTAAGTCTTAATTGGAAGTCCTTGTTTTCTTTTGTATTGGA
GTTCGTATTGTATTAGTTTGTATTAGTCTATTTAGTTCAGTTAGTCTTCTGCTTTGTTTTTGGCTTTGTA
TTGCTTAGGTTTTATTTTTGGAACGAAAACGAGACTATTAATTGATCTAATGAAAGAGCCTAATCCTCAA
ATTACATGAGAGGAAACAAAGAAAAAACTTCAAGCATGCCAATACAAAGCTAGAACCAAATTACACCATA
AAGTCCAAAACTTTGAAGAAAAAGTAAAAAAATTCAATTAAACACATAGCTGAAGAGAATCCCAAATTCC
TAAGACTTAGGCTCAAACAAGCTTGAAACAAATAGAAGAAAACTTCCACCACAATATCAAATGTTGATGA
GGAAAGAGCCCCAAACTTTGAGCTTGATAGAACAAGAAACCATTGTTGAGGACCAAAAAATACAACTTTG
AAGTTTGCCAAAGAAATAAAAACTCACGACATGGAATCCCAAGCCCAAGATAAGAAAATGCTCCCACATG
CAATTCTTGATCCTTAGGTTTTAGTTATTGGTGTATCCCTTTTATACCTTGACATTAGTCTTATTTCATT
GCATCAATGAAGAGACTCGTTTCTTTTCAAAAAGAAAAAAGTGTCCAAGTGCATCATGATCAATTCATTA
AATAGCCATTGATTATTTGGGTGGTAAGGTGGTTTATTCAGCCATGTGGAAGTCTAAGAGGCTGTTTTGG
GTGTTGAGTTAGTTACTATAGTCAGTGGCTTATAATAGTCAATGAATTATAATAAATTGTGTTTTGATTG
CATACTATTTTAGCATGGGTTATAATAGTTTGTGTTTAGAGTGCACCAAATTTTTTTAGTCTGGGTTAGT
CAGTAATCTTGGGTCTTTGTATCTTAGTTTTGAGCTGTTTGTATTTGGCATTTCTATGTTTTTTTTTTAA
TTTGATTTTGGGTATCTCTATTGATCTAAGTTCGGATATGATGAGAGTGCTAAGAGGGTCAACCTAGTTG
AGATGTTCGGGTGCACTTGCTTATCCTAAGGGCTTTGATCTTGTCTCTTCTTTGTATTAGCCTTTGAAAT
GTCTATTCATTTTCTTAATGAAGAGGTTCATTTCCTTTTTTTTTTTTTAAAAAAAGTCTGGGTTACAGAT
GATGATAGAGGAATTGTGGACGAGGTAGACAATGAGGATTTTCAAATAGTTTTATTGTAGCTAAGTGTGA
GTTATAACAATTGGGAACTTCAACTATTATATTGGAAGACCAAACATTGAGTGGGGCAAGTTGGAGGCCA
AATGTCCAAGTGTCTCAAGCGCATCAATACTCGTATTTGGGTGATGATCAGTAGCTCATTAAATAGCTTT
GAAGTCTTTCAAAGAAAAAACCCTTCTCATTGCATTTTCTCATCAGTTTGGTCGTGTTGTATGTCAGACA
ATGATACTTTGCAACATTTAATCTCTGATTGCTCATTTTCAAGCCATTGATGATGGGATTTGTTCTCAAT
TTTTAGTTTTCAGTGGGTGTTTCCAAACTTGTTCAGGGACAACATTCTTCATTTTGTGGCTGGTCCTTTG
CGTACAAATCTAAGCTTTTGTGGCACAGTGAAAACTCTTGTATCAGAAATTTGGTCTGAAAGGAATCAGA
GGATCTTTCACAATAAATCACTTCCTTGGTTCAATTGTTTCAGTTTAGCTCGCCTCAAAGGCCAAAGCTT
CCACTTGACATTCTCGTTCTAAACATTGCAAACTTTTCTATTCTAGACTTATATCTGAATTGGAGTGGTT
TTATAACTTAAGTTTGTTCTGTGCTGTCTTTTTATATTTTTATGCCTTTTATTTTCTTACCCTTCTAGGG
TTACATGTCCTTTTGACTACTAGCTTCTTTTCATTGTTTCAAAAATTTGTTTCCTCTAAAAAGGTTATAG
GGTTTCTGGGTTTGTTTGTTTTGTTTTGTATTTTTTAATGACTATTCCTGCATGCTATATTTTAATTTTC
GTGCACTTGCTTGAATCTTTGTTGAATCTATAAGATAGTTGCCAAAATGAGCTTGCTTTAGTGGTATCGA
TCTGGTCGGACCTTTGTCTCTAGAGGTTGGAGGTTTAGTCCTCATCCCTCATTTGTTGTACTGGAAAAAA
AAATCTATGACACTGTTGTGTTATGGTGCTACTGTAGCTTAAGATTTTTATTTTGCACAGGGGAAGCTTT
GGGGAATAATGTAGCTCTCGCACTTGAAAAAGCCTTAAAGGTATGCTTCATGAGAACAACTAAGGCTTAT
GCAATGTATCAGTTACGTGTTTATCCCTCTTATTCTTTCATTGGCAATGGCATTATTTTGGTTCTTTTTG
AGCTTAGCTGAAAGGCAATTCTGGTTCAAAGAGGCAGCATGTGCATGGTTATAATCTTGTTCGAGCAAAG
AAGCTTTATGGTTACCATTCATCAGAAGAGCTTTTTATGAAGATATATTTGTATCCAAAATGAAACTGAG
TGTTTTTTTTGTTTAAATTTAAATTTATTATATAAAAATATGAATTAGTTAAAAATTAATTTATCTATCT
GCTATACTGTCTTTATAGGCTTTTGTGTCCATATTAACAGTTTGCAACCTGTGATTGAATGCCTTGACAA
TAATTTAGATATTATCCACAAGATATCACCCGTGCTGCAAATCTTCTGCTGGTATAATTTTTGAAGTTTC
ACTTTGCAGCCATATTTCTTATTTTCCTTGTGATTTACATGCTAAATTGGATGGCCAATGGTGGACATTG
CAACGACATCTGTTTCTCTGTAACAGATGATGATAGTGTTAGCTGATATGTAACTAAATTCGTTTTTACC
CATAAGCTTACGTTTGGGTTAATTGGTGCTTTAAGATGACATCAGAGCGGGTAGCAGGTGACCAATGAGG
TCATGTGTCCAAGTCCCTGCAATGTTATTTCCTCCTCATTTAAAATTAATTTTCACTTGTTGGGTCTTTT
TCATATTTCGAGCTCTCAAAAGCGAGGGGGAGTGTTAGATGATATATAATAAATTTGCATTCACCGATAA
GCTTAAGCTTTTGGATCAATCGGTGATTTAAGAATTTGCTTAACGTAATGTTTTGTTGATGTATACTATA
TGGAGAATTTGCAGTGAAAATGTGTGTCTTATGTTAGAAATTCAAATTACAAACTATATCAAGGGCTTGA
ACTTCTGAGATTGATGCTGTAAATGAGACGAATTTTGAGAATAACTTTTGGTTATTTTTTAAGCCCTATT

TTTAATGAAGTCTACAGCAATCACTCTCTCATCATTTTCATCTGCTGTTATAATCCAAAACTAGTTATAA
TTAAAAGTTCTTTCATTGATTGATATATTGTGTATTATTTTCTACTTATTTTAAGATTTTAAATACTTTC
TCGTTTTCAGCTTTTATTAATTAGTTGAGTTCTTAATATTTGATTCAAAACATCCAATTGATCTAAATGC
ACTATGAGGATTCTAGCTATATAATTTTGCACTTCTCGAGAAATATTTAAATTGATTTCCAGCCTACTTT
TTTTGGATAGGAATACTTTTATTGTCTAAGTTTTGAAAATTCCTTTTGAGAATATATTTATATTCCTATT
TTTTTTAAAAAATAATTTAAATAACTTTTCTACTTTCCCTCCTCTCCAATTGGAAAGGTTTTTTGTAATT
ATCACACGAGGTGTATTTATTTATTTTTATTTTTATAATTTCATACTATCAAATTTCTTATTAAAAAAAC
AATTTTTAAGTTAAAATATTTGATTTCTAATGTTGATTTCAAATATCTGTGTTATTCTTCTATTTAATGT
TTGTTTCTGTTTCATCCAGACCTCAGGTTTGGTCCTGTCACCACATCATAACTTTAACCAAAATGTTCAA
TTGTACCATGTGGATGTTTTGCTTGTGCCATTTGTAGGCAGCTTGGAAGATCTGTGTTTAGTGACTGTAT
TTACTCCTTTTCTTTCTCCAGAATGGTGCTGTTCTCGAGAAGAGTTTACAGCCATATGAATCCCACATAC
CATTTGTCCTGCAGTTCCTTGTGAGTAGCTTTATGAGTTTCAACATAGTTATAAATAAGAGATACACTCA
TCTACTTTGTAATTGGTAGACTAATGGATTTCATCCAGCTTCCTGATCTAGTTGCAATTTAGTTTCTATG
TTTATTTGCTCCCTTGTTCTCTTTCTCGTTCTTTTGGGCGTATGTGCATGTGCATCTCTTTCATGTGGTA
TTTGTGAACGGGTAAGCTTAGTTTTCACTCCTTTTAGTTATTGTGGTGGATGTGCATGTCATCCTGGATA
ATCCACTAGAGGTATAAGCTCACTGTCAATTAAATTTTACCATTAGTCTTTTTTGTCTTGGTCTCATTAA
GGCTCAATAGTGGAATTATAGTAACAATTCACTATAAAGCTAATTGAATGAAGTCTAAGCTAGCTGTTAA
CTTGATTTTAGTATTAGCCTTTTATCTTGCTCTCATCAAGGCTGGAGAATAGTTCTATAACATAACTTAG
TCAAATTTACGTTGAAGTATTAGGTACTTAACTTGCTTTTGTTTAATATTGCACCTTTCGCAATTATCCT
TGATTTTATTACTTACTCTTAGCACTTTGCTTATCACATATATAAAATCTTATATTACGTGGGTTATTTA
AGGTACCCATCTTCAATTAGTGTATCTCCATTAGTGTGTGGCTCCTATTTTGCTGCGGTCAAAGCCATTT
TTCTTCTTGAGGCTATCATACTCTGTTTTAGAAATGCAGGTCTCTACATTGCTTTTCCCTTCGTTCTGTA
TCATAAATATCTTCTAGTAAACAGTTAAGCATTCACTTCATTTGGTACTTTGGAAGCTTTATTTTTTTAA
TCTTTATTTGTGTAGCGCTTGACCTTTTTGATCTGAAGTACTGTTTCTGCTAGGTTGACTATAATTTGTA
TGGTATGGGTCTTCTGCATCTAGTGAAATTGAAGTTCCGTCTTCCAGTACCAGATGCTCCGGGGAAGAAA
TTAGATGAGATTTTTCGGTACATGCATGAGAATCATGCAATGGATAATCCAACTTACATGCCATCTGATC
CTCAGGTTTCCATTGTTCTTTTGCTGCCCCCACCCGTAGAAAAGTATAACAATATGCGTAATTGAATGTA
TTGATTAGGAAAGTAATGGGTGTTCTCTTGGACATTTCATTAGGCAGATACAAGTAATGAAGCAGCATCA
ACTTCACCAGTATGGATATCTTCTAAAATTCCAACAGATTGGATGTGGAAATTTCCTACTTCAATGGATA
CCTTAGATGACAATGGAATCAATTTCTGTAAACGTCAAAGTGTTTGTGAACTTGAGGGAGATGTTACTGT
AGAAGGTTCATTCGGCTTCTTAATATTTGACTTGAAAATAATTTCTTTTAACAGGTTCTGCTAGAGGGGT
TCCTTATAACTCATTTATTTCCTTGTGATGTTAAGGCTTTTTAAAATTTTGTTCTCCAGATATTCTCAAT
CAACATTCTAAACTGTACACATCTTTTTCTCAAAACCATTCGGACGTGAAAATGGTTCAATCACTTGTAT
CAATTTGGGAGGTCTGACATTATCCTTTTTTCCTCCTATAGTGTTGTATTTATTGCATTCATGTGCAAAA
ACTGTATGATGGATTATTTAGCTGTAATACGCAATTGGTACATATACATGCTTGTTCAGTGAAGTTGCTT
CTTGTCTGTATCCCAGGAGTATAGGAGAACTGGCGTGCAGGAGGCACCACTGCCACCTGATCCTGGAAAA
CCATTGGCGAAAGAAGTTTTGGAGACTTTCTCACCTGATATGGACTATGAGAAAAAATTGACTGAACTAT
ATGAGAGACCAAAAAGTCCATCAGTGTTAACTCCACTTGAAAAAGATGAAAGGTTGGTGCAGTCCTTGAC
ATCCTCAGTTAATGAAGCAAATATAACTAGAGTAGGATGTTCTGAGGGCGAGTCTCTGAAACATGTTGAG
GAGACAGGCAGAACTAATTCAGACTTGTTTTTCGCTTCTTCATTTGAAGATCATGACAAAATGCTGACTG
AAGGAGAGGATCTTGTCCCAAGATTGTCTATGGATGAAGTTCAAGTAACTCCCAAGGTAGCTAATTGCTG
TTCTTGTTGGGGTTCTCTCTCTCTTTTGCTTGGATGTCAATTGTCACGCTGATTCTGCTCTTGATATATA
AATTTTTATGTTACCTTATGCTCTAATACTACGTTAAATTGTTATTTCACCTACTTTAGGGATGTGGTTT
ATCAAGTGTTTTTGGCAGTAAATGACACAACCTGACAAGGATCGATAAATTGCTCTTAGTCTTGACTGTT
TCCTTCCTTCACTACAGTTTGGGGGGGGGGGGGGGGGGGAGGGTATGGGTGTAATTTGAGGATCATTCTT
CTTTCACACTCTATCTCAGTTTCTTTTCCTTGATCATATGTTCTTATCAGCCCCTTGATCTACTCAAACA
ATTCTTCGATGTCTATAAATTATTTTAGAGAATGTTGTTGATGAGAAAATTGATGCATATTCCTCTTTGC
TTTATCCTTAGGAGTTTTTCTTTTCAATATTAATAGGCAAATTTCCAGGGCATGGATTTTTCAAATCTTC
TCAGTATTTTCTCTCATTTGTTTAAAAAATAATTGAAGTGCATATGATGAATTATATTAAAGACATTAAC
CATAGCTGGAATGCTTTTTAAAACGCTTGTTAGTTTGCTGTTTTGGTTTTATGTTTCTCTTTGGTATTCT
GTTTTGTTTATGTTTTTGGTACAATTATGTCTCTAAGGTTTGTCTTTTTAGCAAATTATTAGCCTCTTTC
ATTTATTTAATGATAAGTCTTGGAATTCTTTTCAAAAAAAAAAATAAAAAAATAAAAAAAAATAAAAAAA
AAGAAAGAAAGAAAGAAAGAAAGAAGAAGAAAATAGGTTTAGTAAGGTGAAGTTTAGTGGGAACTGAATT
CTATTTGGTTAGTGGTGAAAAATGAGAAGATAAAAAAGTGATCATAACAGTGGGTTTTAGCCCATTTCGA
GTTGAACCAAATGTTTGATTTTATTCCAACGTCAGTCATGTTTGGGTTTGAAAGCTTATTTTGGAATATT
CATCTTTTCAGGCTGTCGACGAGGAGGCACTAGGACTCTTGAGATGGCTAGCTACTTCTCACGCGGCACA
AGATATTAATTCTGATGACGAACTTCTTTGCGAGACAATTTTGGGGCCCTTGTTGCCTGCAGCAAACATG
GATCAAGTGCTAGAGAGAGCTAGCCAGGATTATGGTTCTGAGTCTCAAAAGGAGTGTCAAGACATTCTTG
ATTCAGTTGAAGATTTAGATGGATTTGAGGGGTTTAACAAGACAAAATGTTGTCCAGATGATGAGCATTT
TTTCAGGTCTTCGTCTGAAGAAACTATTCCCCAACTTGATGGTGCTGCAGATGATATGTTCTCTTCTTCT

GGAGGGTCGACTGAAAACTCACCTGATAGAGATCTTAATGTTGAGAATGAAAGATCTTCCAAACTTGCAA
TATTATTGCATGGCATTGATTCAGGTTCTTGCAGTCACAAAAAAGAGAAATCATTTTGGGGATCTTTACC
TTTTCATGAGGCTGAGAAAGTGAATACAGATTCGACGTGTGTCAATTCTTGCAGGCCTGACATATGGACA
AGTTCGACCAAAGATTCTGAGTTCATTAGTTGCTTTTCAAGTGAAGATGGAGGGCAGGTGGATGTTACAT
TACGAAATGCAGGTACAGGCACACATAACTCCAGGGAAGGCCGTTCTGTAAGTGATTTAATGAGGAGGAA
GCGTAACTCTCGAAATGAGCCACTTGACTGTGGATATGGTAAAGCTCAAAATTTTACTGTTGATAGCAGG
CAAAAAAAAGTATGGTCAAGAGACTTGAATTCTGGAGTGCTGCGGTCCAATGAGAACAGCTTGAGATTTC
GGGATTCTTCCCATTTGATGCCATGTTTAACTAATCCAAATGCAAGCGTTAATGTGTTTTATGAAAATAA
GCCTGCTTATTCTAATAGTTCCATGTATGGTAGACTGCCGCTTGTAGATGTGTGTGATGGCTTTGAACAG
GCTAGCTTACCCAATGTAGGAGAAATCCCTGGATCTGAGACAGTTTCAGGTCCTTCACAAGTGTGCTTTG
ATCCTTGGCTTTCTGAGGCAGAGACCCCAGGCGTAGGTCCTGCTTCTCTTGGTGGTTGTGAAATCTTGGC
TAGCAAGAAATCTAATTCTGGTGTGTGTAATGCTGATGCTCATGACAGCACACCTTCCATGCAATGTGCA
GACGGGGATTATTTCTCTCCAAGTACAAAAAGAAGATTTCTTTTGGGTAATCAGAATAGTAATGATAGAA
AACAAAAAGATGATGCCGTTTTGCCTGGGCTTGGTCAGTCTATGTCTATGGTTACCAATTTTGATGGTGA
GCAAATATTATCAATTGGTTTGACTACCTGCAGAAAGCCACCAAATGCAGATTTGGTGCACAAGGAGCCC
TTTGCTTCAACTTCGTCTACCATGTCCTGGAAACGTGCTTTTTTGAAGCAAAAAGATGTTGAAGGGGAAA
CAGGTTAAATTCTATTTTCTTTATGCACGTGGTATTGGTACTTGTCTACCTTTCTGGATTAATTTTTGCA
TGAAGACATTTATCCTTCGGGGTAGAAATGCAGGGTTTATGATTTTTATATTTTATTTATTATTTCTGTT
GGGAAATTGAAGTGTCACTGATCTTATGATTCCCTTCCTTGCAAGTGATAAATTTAAATAACAAGAACTT
TGGATGAACTTTTTCTTGCTGAAATGGTACTGACTAAAATAACCTTGATTTCTCCAGGGAGAGCTTTGGA
TGATCTTTTGCCATTTTTTCTAGACAGAGATAAGAATGATATTTTTGAAGATCATGGTTACAGTTCTCCT
AAAGAAGCTGCCATGGGTGTTCCCATTCACTATCGAAATGATGGCTCCTTCATGTATATTTTAACACCTG
TAAACTCACCTCCTTCTACAGACAGTGTTCGTCAGTGGGTAACTACTGCACAAGGAGGTGATAACCAGAA
CTCAACAGGGCATATATTGTTTTTTTTTTTTGTTTCTGTTCTCTTGTTCGTGTGTATTTTTGTTTTTTTT
TTTAAATTTTTGGGATGTCTAGAAACACCAAGTTGACAAGTCTAATACATGTAGATCCTTCATGTTAATT
GGAAAATTACTCTTTAAGCTTTATGTGGGCAGATGAACCTTTGAAGGACTTGTTAAATATTTCATTTGCA
GCTCTCAGGATCAACTCAGGAAAAACATTAGTTGATGATCGGAACAAGCCTCTGCCACAACCTGCTTCTT
CATCTCATACTAGTCATACAGTAAATCATGGAGGTCTGCCTAACTCATCTGCAGATGAAACATCTGTTCC
TGAAAATTTAGAACCAGTTAAAAGTGGAGTTACAGGAGAAGTAAGAGCTTGTGCAAGTTTGTCACAAGAT
GCTTCTCAAATTTCTGGCCCAGATGGGATATCAAAAGCTACTCCTCTCAGCCAAATTGGGTTTCGTGATC
CTGCAAGTGTTGGAGGCATTCAGCAGTTAACATTGTTGAGCATAGAGGTATTCATTACATTATATCTCAT
TTTTATTTCACGTGCTTAATACAGGTAGCAACGGATATTAAAATACTGTTTGATACTGTTCACTTTATGT
AATGGAACTTGAAATACCTTTTCTTTGCATGGAGTTTTGAATACCTTTTCTTCTGGAAATTCAGTGGGTC
CTGATAGCCTCTTCGTTTTGATATGGTTTCTTGTACTTGTCTATTGGGATGCATTATTGAGATTTTGGCT
CAATGTTTTTACTAGCTTAGAAACTTGCTCGTATATGAGGGATTTATCTCTCTCTCTCTCTCTCTCTCTC
TCTCTCTTTTAAATTCAAAATTTACAACCTTTTCTTATAAGGAATATTTTATTGATAAAATGAAAAATTA
CAACATAGGAAACGTAGAGGTAGATCATAAATAAAGCTAACCAAAAACACCAAAAGAATTCAACAATACA
TGAATACCCCAAATAGCTTTGGAAAAAAACTCAACCACTAACCCTGATTTCTCAGAAAGGAATTTCAAGT
ATAAATATCAGAAAAGAATAATTGCTGAAACTTATAGAAAGACTACACCGTAATCTGCTGTTAATCAAAA
TTTCAAATCACTCTAAGCTTCTCTGGAAATTCTAGAGTCTCTTTCACCCTCAAATGACCCACTTCATAGC
TCTGATTGAGTTAATCCTACATTATTTTAGCCTTCTTACCATAGGAAAAAGATCTGATCTTGCTTTGTTT
TAGGAACTTGTGCTTAATATGACACTGTTCATGTTTTAATTCATTACCTCATCCTTGTCTCTGGTTGTCA
AGGTTCAAGCAGAATGTAGGGGTGATCTCAGGCCCGATCCTCAACTTGATGCTGTCAAGATGATAGCTCT
TGCTATTCAGACTGATAGTGGCCCCGTTGTTGAAGTTGTTTTAATCTTATGTACTAACATTGATTCATCT
AAGAGGTATGGTATTTATGGCCACAATGAAAAAGTTTGATCTTGAACCATTGCCTTTGTTGATTTTTTGA
GTTTATGGTGAATTTACCCTGGTGATCCTGAGATTTTTGCTGCATTCAAGGATTTTATTAGAACGTTGAT
TTATAACTAATGGAGTCCTTCAAGTATGGATATTACAAATTTTATGCTATCTATTTGTTAAGATAGGTGC
ACATATGCATATCTCCTTTTTATCCTAATTTGTTCTTGATTTGGTTTTGTATTTGTGAGACATTTTGTTT
TTGTTTTTGCTCTTTTGGGTGCTTGTGAGAAATTTCTGGTGCATGCCTTGCTGTCCAGTCATTATTACTT
TATGTACAAGGGAGTATTATAGTAATTAAATGTCCGAAATTATAAAATCGGCCAATTTCTAGAAGTAAAA
AATAAAAATGATATAGTTTAAATGTCTTCTTCTTTATTGACACACTTTTTTTTGGTGTGATAATGTATCA
TTTCTTTTCCAGAAACTGCTATGGGATAGGCTACAAAGTTTTGGTTCATCACGAAGAGAAGTGTTTATTT
CAAAGTTTTATGAAAATTATATATTTAACTGACCCAGATATTTTGATTGGCTGGGATATTCAAGGTAGTT
CTCTTGGATATTTGGCAGAAAGAGCTTCTCAGCTTGGTATAAATCTACTAAATAAGATATCTAGGACACC
AGATGAAGCCAAGATGTTGGATGGAGATTCAAAAACTCACACAGAAATTCCAGAAAACTTGGTTTCCGAG
TTGGTAGATTCTGATTCTACTGTAGTAGAAGATATGATAATTGAGGATGAATGGGGTCGAACACATGCCA
GTGGAATCCATATTGGTGGTAGGATTGTTCTAAATCTATGGAGGCTTATGCGCAATGAAGTTAAGCTTAA
TATCTACACGCTTGAAGCTGTTGCTGAAGCTGTTTTGAGACGAAAATTACCTTACATCCATCATCGGGTA
TTAACACAATGGTTTAACAGTGGTCCTCGACAAGCAAGATTTCGGTGCATTGAATACGTGATGGAAAGAG
CAAAGTTGAACCTTCAATTAATGAGTCAGCTCGATATGGTATTTTCATATTATTTGTCATAAAATATGAT

AGTTTTTTGTAAACATATGGGTGTAGGTTACTGTATCTGCTGGCCAAGTAACTTTCATTTTCTAATACTT
CCAAGTTTTTAGAGTTCCTTTTCGCATGCATTTGAAAAGGGGTGACACTCAAAACACTTCATACTTTCAT
GGATCATGTAGCATGCATATCCTATACCATGGTGGACAACTATCCATCACAAAGAGTTTCCATCTTTGTA
ACTGAAAGTGGAAACATTGGAAAAATATTTTTTCCCATTGGCAGGATAAGTTATTTTGTATTAGAAGTAG
ATTTGGTAGAGGCTAGAGAGCCCCACCTTTTAATTATTTTGCCTCTCTTCATCTTGCAATAATTTTGATG
TCTGAATTATATGAAAATATTAATTAGCTAAACCGAAATATATTGTAAAAATAAGATTTTTAGAAAGAAA
ATATAATGTAAGTGCTTTATTTTTTTGAATGAGAATTTATCACCTTAGAGTAGGAGACTTGACCTTTTTT
TGTTTATTTTTTCTTGTTTTATTTTGTCATAATATGTTAATGGATAAATTTTAGCCCTCCTTTTGAGATT
AATGGTGCTTCCTTTGGTGAATGGTTTCTCGATAATTTATGGAATCGGAGGAGTTGTTAGTTCGTAGAAG
GCAATTGGGATAATGACTATAATCTTTCTTATTGAGTTCTTCCCTTGGGGTTTTTGCTTCTAAGATTTTT
TGAGCGGATTTCTTTTTTCTTTGATTAAGAATAATTGGATCATTTCTTAAATTAATTTGTTGCACTTTTC
TTTTTGGAATTCAATGCACACTGCAAAAGATATTGGCAAAACAGGAGTATTTTCTTTTTAAAGAATCTAT
TAGATGATATAATATTGAATTTGCCTTCACCTATTAGCTTTAGCTTTTTGGGTAGTCTGTGAACTAACAG
AACTTTTCATTGACAAAATTAAAAGACTCTATCGTTCAAATTTAAACAGAAGAGTGTTAAAAGTTTGGCT
TTGTAGGTTGGTATTTTGGTTTGGGTTCAGCATTATCTTTTCCTCCCTTCTTCATCCTATATACTTTTGG
TTTGTATCTCTAAATTGTACTCTCTTATGGATATATTTATTTACATAAGGTAATTCTTTCATTCACATGA
TGACTACTATATCACATTGTGTACCTTGCAGATAAATAGAACTTCAGAACTTGCTCGTGTGTTTGGCATT
GAATTTTTTTCTGTTCTATCTCGAGGCTCACAATATCGTGTTGAATCTATGTTACTAAGATTGGCTCATA
GTCAAAATTATCTGGCAGTCTCCCCCGGGAATTTGCAGGTATGAATGGATGGTCATTTGCTCTTCCCTGA
ATAATTTTGATAATGTAGGAAATTGTACTACGGCTTCTTTTATGTTTATGTTGTCTACTTTGTTAATGTT
TTTATCCAAGGTGTACTTTGTGATAATTTGTTTGCATGCATCTCATGTATATCACCATTTAATGATTATT
TTTCACAATATATTAGCAAGAAATTTAGTTGGAGAAGACAAGATTGAAGCACCCTTCTCTTTTTTTGTGA
TCGGTATACCAGGTTGGGGCTGGGGGACAAATTTAATCCTGATGATATCACCCAGAGGTCTATTTTCATT
TATATTCCCTACAATGAATATTAGTAAGAGAGAAGGGTGCATTATTTTGTCAATTATTTTTCAATAATGG
TATACCAGGTTGGGGGTAGGTGTTATGCTTTCTTTCAAGATTATTACTTGTGTGAATTTGATGTCTGTTA
TCATACATAATTTTGTCAGTTACTTTTCATGATCATTACGTTGTTTTTCCGGCGAAATCGTATCTATGAT
CAATTTCCTAGTGTGTGTTGTTTTTATCTTGCTGTGAATATGTGTCTGTGTGAGTCTCCTTTTTTAGGCT
GTTATGGGTCATGGGTTTATGCTACTTCTTGAGCTTATATGCATCTGCCCTTTTGAATAGCAGTTTTGCT
ATGGTTCTTCCTATAGACATAATTGGTTGTTCAAACCGAGGACCTTCAATAATATATGGCTGTCTTAAAA
AAAAAGTTGGAAATTTTGTATAAGAATCAATTGATTGGTGGAATGTGTAAATACTGTATTTGTAGTTTGA
GTATTTCTCTTTACATACTTGATGCTTGATGATTTATCTGTTTACATAATCTCTGACTATGTATTATGTG
TATTTTTTTCTTCTTTTTCTCTCACCTTTCTTTAAATTTTTCATTCAAGGATATCAAGGTGAATTTCATT
ACATAATTATAACCCAAAAGTTTTTCAATGCTTTGTGGCCTTTATGCCTTTTCAGTTTTTTTTTTATCAT
CTAAATCTAGCTTTTGGTTTTTCCTTTTGTTTATTATATTTATGATTCCATTATGATAATATTGTCTATC
TTGGCATGTTTGTTTTTTTGTTAGGTCGCATCTCAACCTGCAATGGAATGCCTGCCACTTGTGATGGAAC
CTGAGTCTGGCTTTTATGCTGATCCTGTGGTCGTTTTAGATTTCCAGTCTCTTTATCCATCCATGATAAT
TGCTTACAATCTTTGCTTTTGTACATGCTTGGGAAAAGTAGCCCCTTCAAAGGTCAATACACTAGGGGTT
ATTTCATATTCACCAGAACAACAGGTTGTCAACGAACTGAAAGATCAGATACTGTTCACTCCTAATGGTG
CAATGTACGTAACACCAAAGGTAGTTCTCTGAAACTAGGTCTTTATACGATAATATGGAATATAAAGAGT
GACAATATGAAAAATGAAGAATATTGCAGCGGATTCTACAGGCCTAGCTAATAAATTTTAGCTGATAGGC
ATTAATATATGACTACAACACCAAAATAGTTTTATGTTGTAGGTAGTGATTCTATTAGCTTTTGGTTTAG
TGTTTTCTTTCCTACCAAGAAGGAGGTTATTGACAATTGTTTTTCGACCTTAGCAAGTGTCTATTTTTCT
TCCTTTTTTTTCTGTCTTGTTTTTCTTCCCTTTACAATGATCTTTTTTTCTCATTTACTTGGTAGACATG
AGAATAAGGAGAATCAGAATGTATTAATGAAGATGACACTAAAACATGTATTATATTTCTAATTTTCTAG
TCGACCATTAAAAGAATTTTAGAGTACAGTTGTTAGTTCTATTAGATTTTGGTTTAATGTCTTCCTTTCT
ACCATGAACGAGGTTATTGATAGAAATTCCTTTTGATGTTAGCAAGCATCAATTTGTTGTTTTTTTTTTT
ATTTAATTTCTTTCTGATGATATTTAAGGTCTTGTTTACTTAGTAGGTTCGGGGCTGAGAGCATTGTTAA
GGTCTTCTTTTGGAACCAATGATGGAGAGGAACCATCAGGCGTTCAATAAAACATAATCAACTTTTTTTC
CTAAAGAGGAGACAAGCTTCTATATTATTAATTAACTCAAAGTACAAGAGAGTTATACATTGAAAATAAT
AGAGAAGCCTAGAGATGGAGGGGAGAGAGGATCAGTAGATGCACCCAAACATCTCAACTAGGTTGACACC
CCCGTAGCACACTCATCATATCCAAAATACAAGACCAATACTAAGGTCATGAAAAGACCAATGTAACAAC
CAAAATAACAATATAAGCTACCTACAAAACCATTGTTATCAATGTCTTGTTAATGGCTGTGTATTTTTGT
AAATATTTACCCCCTTTTTTATTATGGTTTTACCATATTTTTGGCTAAATGGATCACCCTTTTATAACTC
CATTTCTGGACTGTGGGGTCTTCACACCATGTCTTTCGTAGTATTTCACATATCAACTTTTTAATGAAGA
AATGGAATAAAAATTTTAATATTGCACATACTAATATTATGTTAGAGTAGGAATAAAGAAAAAGGCATCT
TATCATTATTTGTGTTGTTATTGCTGCTATTTATTTATTTATTATACTAATCTTTTTGTTCACAAACTAG
ATTCGGTCTTAATTAACTATGTGACCTATTGGGTGGGATCAAATTAGAACTAATGAAATGCATCCCATAA
AAAAAGTTAGGAGACCCATTGCAAAAGTAATTAATTAAGGAAGGATGATAAAAAATTGTAGAAACAAACTG
TGGGATCGATGAATTTGAAGATTGGAATGAGATTAGGGGATCTCTCTCTTTTGATCTTGGCCTACAGATT
TTCTAATCATCGTTCTGATGAGTAAATCCTCCCGTAGGTTTTTTGTCTTATCACTAATGGTAGAAAACAC

TAAAAATCCTATGGTGTAGTGGTATCAAAATGATGACATCAGTGCTTTGTTCCCTATTATTTCTCAGGTT
CGTAAAGGTATATTACCTCGTCTTCTGGAGGAGATATTATTGACTAGAATCATGGTAAAACAGGAAATGA
AAAAGCTGGCTCCATCACAGAAAGTTCTTCAGAGGGTACGTCTATTTCAGACTACAGTAGTCTTAGTCTT
TTAGCAAATCATTGCTGTTGGTAATAACAATAATTTGCAAATGTATTGATGTTTTTGTTTTCATCACATG
ATATTAATTTTGGAAAACTATTTTATATATGAACAAGATAAGAGTTTATAGATGTTATTCGATTATATAA
TGCATAACATGCATTTGATTTTTCCAATATGTGATCCGTGACATACATGAAGAACAAATGTGATCGGAAA
CCTTTAATTTCAGTTTTAGTTAATATTTTATAAATGTACATTTAACCTTGTTCGTTTGTGTGTTTTTACT
TGAAACTAGTGTAGGAAAAGTAGCATGACTAGTTCTAGCCCCGTTTTGTAGCCAATTGGTTTTTTAAGTT
TTTATTCCTAAAATTGAGTTTATGATTACTATCTCTATCTATAACTTATTTGTTTTCTTATCTATTTTCC
ACCTACATTTTCAACAACCAAGTCAAAAAATTTGAAAACTAAAAAAAAAAAAGTTTTCAAAAACTTGTTT
TTGTTTTTAGAATTTGACTAGAATTCAAGTGTTTCTTTCTTAAATGTGAAAACCATAGTGGGAAATCTAG
GAGAAAACCAACCCAAATTTCAAAAACGGGAAACTAAAAACGAAGTCTTTTCCAAATGGGGCCTTGGTAA
TTGAAATTACCCAAATACATTAAAATTTAACTATTACTTTGTAAAAGATTTCTAACTTTGTACTTGACAC
ATATTGAGCGTCTTTTATCGAATAAATAATATATGACAGAATACATTAAAAATTTTCACGTCAAACAATG
TTTTTATAGATGTTTGTTGTAGATTTTCTTCTTCTTGGCCTGTGTTCTTCCTTAAATGTGTTTGTCTCTT
ATTTTTTCTTCTATTTGGAAATCTTGTATTTTGGAGCAGTATTGTCTTTTCATTATATCAATGAAATATT
TGTTTCCTAAAAAAACAATGTTTTTAAAGATTACTGGTACCTGAAAAAGGACTTTTTTTTTTTTTTTGTC
TCTCTATATTTTAAATATTTATTTAAAGAATATCCCTGTGTTTTTTTTTGTTTGTTTGTTTTGTGTTCCG
CAACTAAGTTGAACATTTCCTTCGTGACATTCAGTAAAGTGTACATAAAATTCCTTGTTTCCTTGGATTA
TCTTATTTCATTAGTGCCTCTTGATCATGCCCTGGATCACAGATTCACACTATTACAAGTGTATTGCTGA
GTCTGATTTATTACGCTTCCCAGGTATTTAATGCTAGACAACTTGCTTTGAAGCTAATAGCAAATGTAAC
ATATGGCTACACAGCAGCTGGTTTTAGTGGTCGTATGCCTTGTGCTGAGCTTGCAGATAGTATTGTTCAG
TGTGGCCGCCGTACACTAGAAAGTGCAATCTCATTTGTTAATTCACAGGAGAAATGGAAGGCTAAAGTTA
TTTATGGGGATACTGACAGGTATGATATCTAATTAATTTTGACCAGTATGAGTTAAAACTGTGTTGAGCT
GACAGCGTAGTAAACAAAAATTTAACATTCTAAATTTCTTACCTTTGGGCATTTTAGAGTGGGGAAATGG
GGTTTGGTTGAGGACCTTTTAGATTTAAGTGCGTGCTTTTCCCTTCTCATGCAGCATGTTTGTTCTTCTA
AAGGGACGTACAGTCGAACAGGCTTTTGGCATTGGACAGGAGATTGCATCTGCAATCTCTGCAATGAATC
CCAATCCAGTCACACTTAAGATGGAGAAAGTTTATTCCCCTTGCTTCCTTCTCACTAAGAAGCGTTATGT
TGGCTACAGTTTTGAAAGCCCTGAACAAATTGAACCTATCTTTGATGCTAAAGGCATTGAGACTGTACGG
AGAGATACCTGTGCAGCTGTGGCAAAGACAATGGAGCAATCATTGAGGCTTTTCTTTGAACATCAGGATA
TTTCGGAGGTGCTTTCTCTATCTGTCGCTTCCTTTAAAATTAGTTGTCAGATAATGTAACACTCGAGAAT
GTAAAAGAAGTTGTCAGATAATGTAACTATTAGATCATATATATTTATAGCTTTTAACTGCTGTTGACTT
GAGACCATTACATTGTTTGGTCTGAGGGAACGGAATATCTAAAACTTCAAGGAAGCTTAGAATTACTTGT
TTTGCTAATTCTTAATGTTGAATTTCTCAATACCTGCTTGGGTGATTGAATCAGTTTTTCAATGCACAGA
TCAAAACATATTTGCAGCGTCAATGGAAGCGGATTCTTTCTGGAAGGGTTTCAATTCAAGATTTTATCTT
TGCAAAGGAGGTACGGCTGGGTACATATCGCACAAGGGGTCCATCCGCACTTCCACCCGCAGCAATTGTG
GCCACTAAAGCAATGAGAATTGATCCGAGGGCAGAACCACGCTATGCTGAGCGAATTCCTTATGTTGTAA
TTTATAGGGAGCCTGGAGCTCGCCTTGCGGATATGGTTGTTGATCCAATGGATCTTTTGGCTGTGGATTC
CCCCTACAGATTGAACACTCTATATTACATCAACAAACAAATAATACCCGCCCTGCAAAGAGTTTTCAGT
CTTGTTGGAGCCAACTTAAATCAGTGGTTTTTGGAGATGCCTCGTCCTGTCAGGGAGGTGTTTTTTAAAC
AACCTGTATCAGCCGCAAATCCTAACCGAACCCGGATTGATTATTACTACCTATCCAAGCATTGTATACT
ATGTGGTGAGTTAGTCCAAACCTCTTCTAATTTATGCAACCAGTGTCTACAGAATGAAGCTGCATCTACC
ACTGCGATAATCAGAAGAACTTCAAAGTTAGAGAGTGAAATGCAACACCTTGCTGCCGTAAGTACCATTG
CAATATTTCTTCTATTTTATTAGGTTTCTTGGTTTCTTTGGTTACACCAATCTTCTTTCAACAGAATCTG
ATGCCCTTGTAGCATGCTCTTTGATTTACTTGATCCGTTTGGTGATTACCAAAGTTTTATTGACGAACAT
ATATGATCCATTTTATTGGAAAATTTGCAAAAACCATCCATACAATATTACTAAGCATAAAATGTAATAT
GGATTAGTGTTTGATTTTAGTTTGTTGGTTGGTTGGTAGGCTTAAATCTATTGTATTGGTACACTTTTGA
ATCATAATAAAGATTTGTCTAGTTTTAGACTCTTTGATACCTGTCTGCATCACAGTTGCACCCTCAAACT
ACCCTTTTTAAAGTTCCAATACCGTAATAACTAGATACTACCATCAGAACTCCATTAGTGGTTGGCTGCA
TTACCGTTGCACCCACAAACTAGCCTTTAAAGTTTCAAAGCCATTATAACTGTAATCGACTTTTTTAGGG
AAGTTCCTTCCCTTAAAGTCTTCCTCTTGGTATTCTTTTACCCTAGGATTCAAGGGTTTTCGTTTCAAGA
TATTAATTGTAGTTGGGTGAATTTTATTGTTCCTTATTCATCTTTTATTGCTCTGTTACTTTTAACCATT
TGTTGTTGTTCTGTTAAATCTGTGTGGATCTGTTCTTTGTATACTTTCTCTGTTAATGTTCTATCTTGGA
GCATTAATCTCTTTTCATTCTCATGAAAAGTTCGGTAACATTTTCCGAAAAATGGTAAAATTTAGTTAGC
TTAGGAGCAGTTAATTTCACATGTTAATATAATTTTTCCAGTTACAACTAAAAAATCCCTTACGGTACGT
ATGTTTTGCTTGCAATGAATTGATAAGTATCATCTCTTTACAGATTTGCCAGCATTGTGGAGGAGCAGAT
GGGATTGTGGAATTTGGTGTGAAATGTACTTCATTCGCATGTTCAGTTTTCTATGAGAGGCTGAAAACTC
AAAAAGAATTGCGTGGTCTTGTTGCTGTTGCTGCACACAAAGATCTATACCCAAAATTGCCAGTTGAATG
GTTCTGATTTCTGGCTTCTCAAAGCATCATCACAAAATCTTCCACTTTTTCAAAGGCAGTCTTTCAGCCT
CAGGTACCCTACATTTGTTGGTCTCCATCCCTCAGCCTTTAGAAGTGCCTACTAGTCACCACGTCATATA

CAAGCCCTCACCCTGTGTTGCGACTCTAACTTATTCCACAGTAACCACTAACCATATAAGCAAACCACAC
TATGACAGAGAAGACTGGCAAAGGGGTGTCCCTACAAACGAGTCCCGCAACCCATGCACATGGGCGGGCG
GGCGGGTGGGCGACCTAGAGAACTCCTTGGTGCCACCACGAGCAAGACAAAAGATTATGGTTTGGAATTA
GCCCCATGATTCCTTGTACCATATGTTTTATACCTACTGATAAGAAAGGGGCTTAATAGTCATATTCTGA
TACATATATTTTTATTTCACCAAATGTATAAATCAATATAGAAAGTTCGTTTTTGATTTTGATTAGGTAA
ATTTGCGTTTATCTGGATCTAAGTCCATTGACAAATAAAGAATTAAGCTGAAAGTTCTTTACTTTTATTG
AATTGATGTGAATGCAAGGTTTTGATACAAATTAGAAAGGCAGGTTCAATTGTTATATGTTAAAAGGCTA
CAAATGAATTAGTAATAGGTGAGTATGTTTCATCTCCAACAGGGTTTAATTGATTTTTATTGAAGTGAAA
TGAAGGCAAGTTTTTCTTACAAACATGAAAGAAAGGTTAATTTATAATGCATATAAGATTAATTCATGGA
TCATGCCAATCAGATCACTTCTTTTAAATTTGAATGGAAATGAAATTTTTAGTTAAAAAAAAACTCATTG
AAAAGAAAAAAGGATCTTTTTTTTTATATCAGCGAAGCAAAAACCTCTTGAATTAGAAAATAGAAACAAA
GCAGAAAAGAAATTTATGGTGGAATTAGAAATAGAAACAAAGCAGAAAAGAAATTTGTGGTGGAAGAGGA
TCTTGACTTAGTTCTCTCAAACCATACAACAAATCTTCAAGAAGACTCTATGGAGTAGATTTGGAAAAAC
TATAAATAAAAAGCAATCAAAGAAACAGATGGATGCAAAGATTGATTTATTCCAAAAAAGACATTTTCGG
TTTTAATGGAGATGGAATGAATCCTTATCCTTAAATAAAAAACTTTCAATAACATCAAAATAATATATGA
TTTTAGGCTACAAAATGATTACCAATGGATTTAATTTCGTTGATTCAAGGTCTATGAAATGTATGATTGG
ACATGCAATTTTTCAATACACTTATAACTTAATTTGTTAAAGCATTTTAGAACATTAGGTATCTTAGACC
GACATTTGTTCATGATATTCTTAGATAAAAAACTCACTCAAGGTTCAATTAACTAAAAGAAATAAAAACT
ATTCTCGTGATATTTTAGAAATAATGCTAAAAGATCATTTGAATTAAAGTTAGAAATTTCAATCTCCCGT
CGTAGTTTATAAAAATTTAAGAATTACACGTACACTACTATAACATTTAAAATAAAATTTTTAATTTCAT
CCTTTTATTTAACCTAACTAAGAAAGCTCACTAAAACTTGAAAATATGAATGGAGTTAAAAAAAGGACTT
AAAAAGCACTCTCATTTTTAGAATATAACGAATTTAAAAGAGATGTTTTTTTAAGAATATAATAAGATAT
GATTTAATTGATAGGTTATACTCAATAGTCATTAAGCCTATATTAACTGCGATTTATAAATTAATTAAAT
ATACATTAAAAAAAACTTAGTTAAGTGAAATGAAAACTAAAGAAAATAGTTCTGTAAAAATAAAATAAAA
AGGAAAATAAGAAAGAAATAGTTTCCCCCTCCCTCTCGTTGTTTAGTATTGTGGGGCTTTTCAGGGTTTA
AGCTCTCACCTTCTTGTTCAGTCCTCCCATGGAGGATTTTAGGATTTCATCTCTAAAATAGTCCCCTCTC
TTCGACAATGACTTACTCCCCCTTTTCTCTCCTCTTCCTTAAGCGTCTTCCATTTTCCTCTAAAACCTCC
AATGCCCCCCTTTCTTCCCCCCTCCTATCTCATCTCCTTGCAATCGGAACATCCAAGCGATTTTTCACCT
CTCAAATCTCTTCAACTCCCTCACCACCTTCCTCTGATCCAAAACCCAATTCCCTTTCAGCCCGGATGAG
CTTTATCTTCGATCAAATCGATGCCATTGAAAAAGAACGCTCTCAGAAGGACCAAACTTTGCAGAGAATT
CGAGCCTGGCGTCAATCCAAAACCACCCAGAATCCTCCCCTTGGTGATAATGTTAATAATTCGGAGCCTG
CTTTGGAAATTGACGAAGGACATGAGGTGGAAATTGTGGAAAAGAAAAAGAATGAAATTGAATTGGTGCA
TCCTTGGCGGGAGTGGATTGAGTTGATGGAAAGGTTAGTTCAGCAGAATTACTTTGATCATAAGAGGAGA
AATGAGGGCAAAATGGTGGAGAAAATGGGATTTAGTGCCTCTGACGTTGCTTTGGAAGAGGATGTTGGGT
TGGATTTGTCAAAGGATTTTACGGCAGTTTACACTGCGTGCCTGAATTTTGGGAAGGACCGGTTCGACAT
TATGAGGTTAGTACCTCTGCATTTCGACTTCAGTATGTGAAAAAAGTTTGACAATACTTGTTTTGTGAAT
GCACTTGATTAATGTTGATGCAACAATAATGGTAGGTTGGTATGAGGATGTAGATGTTAATATTATCTAT
CTTAGATTACATTTCGTTTTAAATTTGGGTAATTTTGGATTGGTTTAAGCTTCTTGTTATGAGTGGGATG
TTTTTCAGACAGTTTTCTGGTGCATGTACCTTCTTTTAAACTGAATGTTCTGTTTTCTCTTTGAAAAATG
TTATGAGTTCTACAATTCAAATTGAACTTTCCACTTGATTTAAATTTTTTTACTCATTGATCTGCTTGCC
CTTTTTAATCCGAGCGAAAGTGTGGAACATATCTGACTAAAATTCTAATATATATTTTTTTTCTTTTCCT
TTCCAACCGTATATTGTGTTAATTTATTTTTGTATTTGGGTTAATCAACCATACTGATCAATTTTCATGT
ATATCTAATCCTTGTTTTGGAACTGTGTATGTAAATCTCCGGAATAGATGAATTTTTTTTTATAAATATA
CTCGTCTAAGGTCTAAAAAGCTTTTATTTTAGGTCGTTGTCAAAAAAAGACATTCAAATGTTGGTAGGTT
TTGGATGCCCAAGTCCAGACAAGAAGGTTGTTTTCTCTGCAAAACTCTTGAGGAAGCTTGTTCACCTTGA
TGAAGGAGACGTGAGTACTTCTTTGCTTGTTGTATTTGTTCCTACTTCTTTAGCATGTCATGAAGTAGTG
GAAATGGTTTTAATACTGAAAATACTTTATTTTTACTTTTAACTTGTTTATCATCTTTCTCCTTTTACAG
GTTTTTGCTTATGTAGTTTTTTGGTCTCACTTTTAACTTTCAAGTTCAACTAGCTCTGTATTGAATTCTA
TGTTAGGTACAAATCTTGATTAGGGTTTAGAGTTCGTTATCTTTCTACATTGTAATAAAAATTATTTTCT
GGTAGTAACAAACTGCCATAAATTTGGATAATTTTCAGATATTGTTTTCCTCTTAGGGTAGGTATTAATA
ATCAAATAATATGATTTTTGTCCAAATAATAACATTTAGTCTTAATATCCATTTGAAATGTTATTTTATG
CCACAAGCTTATTTTCTGCTGCTCGTTTGACCTATCACCTACATAATCTAATTTACTTTTGTTTTTATGC
TCTGCATTAATTCTGTCTTCAGGTCTGCAGTTCCTGCAGTTTAAGAAATTCTTGTGAAAGAGCATATTTG
GTGACTAATAAAGAGGATGAGGCACGCACAATCGATATAATGCGAGTTTTATTTACTTTTGGTTTTGATC
ACATGAACGGAACTGTTGTCAACGATTCCCTTCTGAAGCGGAAAACTGTTAAAGCAGTAGTTCGCAAATT
GCTTCATCAGGTTGTCAAGTTGGGTTCAGTCCCAATAGATCCTACTCTCACTCCTCCTGTAATCAAAAAG
CCTCCACCAAAAGTGAAAAAGCCTCCACCTCCTCCAAGGAGGCGAGTAGGACGTGATGATGTTGAGATGA
AAAAAGGGGATTGGCTTTGTTCTAAGTATGTATTTCTTCCTATCAATTTATCGTCCAAAGTAGTCAACTT
TTTATTGCCGGAACCATATTATGCTTTCTTTGAAGTTATAACCTTATGGAAATGGTTTATTGTTCCATAT
GTAGAGCTCATATGGTGCTCTAGAAAATCAAAACCTAGAAACTTATAATATCATAATTTTTTATATTAAT

ATGTACTTATAATGATCACTAGCGTTAAACTACTGTACTTTTGCCTTTTGCCTTGTGTAAATGGTCTGGT
AATGGCAGTTGCCAAAATTGTATCATATCTGGAGTTTCCTCAATATTCACTTCTTCAGTTGTCAGATTGT
GTTTGATTTCTGTGATGTAATGCAAACTAACAAATTACTTTCAGGTGCGACTTCCTGAACTTTGCAAAAA
ATACGGCGTGCTTACAGTGTGATGCCAAACGTCCCAAACGGCAACTACTTCCAGGAGAATGGGAATGCCC
ACAGTAAGAATTTTCATATCTACTTAATTGTTTCTCCATTTAGATACTTGAACCCTATCTGTATGATTAT
TAATGGATGCCATAGTTATTCTTTCTACAGTATAGCTAAATTGCAGAGAATAGGTAAGAGGGTCAGCTTT
TATTGGTCTGGATAGTTGCACATTTTCTTGGGTTAAATCTGGGTCTGCTGGAGCGTTCTTGGACATAATT
GGTTTGAGGTCGAATTATTTTTGACAGATAAAAGGGAAAGATTATTACTTTATTTTCTAATATAAGAAAT
AAAATTAGGAAGAAAAAGGAGGGATCGATGGAAGCATGGTTGAACTTACAATTGTGAGCGACTTCACTTG
ACTTTTTGTTATCGTCATTAATTTGCAGTCACTGCTTGTGCTTACTATTATCATTCTAAAACTAGCTAGG
ATTTTTCGAAGAAGATGCCTGATGAGAAATAAAATATATTATAAAAAAATTAAAGTGGCTAAATCTGAAA
AAACTACTTTTGAAGTAACGAAAGGCTAAGATCATAGTGAGTGTGAGAGTTGATAAGAGCATTTCTTGCT
TCTTTCTCATTTAAGACCCCATACAGGAAACTTTTATCTAACATGAAAATATATCTTCTCAGTGGTAACT
GGTTTGATTGTTCAAACTTCAAACCACAGCACTTGATGATATTGTATAGAATAAAAAAAAGATTATGATT
TTGTGTTATGATACATCAGTCAGTTCCTAATTCTTTTTACCTTTTGTTATCTGTGGCAGATGCAACTTTT
TAAATTATAGGAGAAATATGGCATGTTTCCATTGTGAATGCAAGCGACCAGCTGAAACATTTTTAGATAA
TGAAGAACAAGAAAAACCGCATGCTCCGTCGAAGTTTGAAAAGATTGCCAATAGGCAGGAGGTCTCTAAT
GCCTGGAATTTTGACTTTGATGATGATGAGTCAGATGGGGCAGATGTTGCAGCTTTTGAGCATGCAGATT
CATCGATTGCAGCCGAAGGATCTCGTTCAGATTTTCAAGCAAGAGGAAGCAAACTTGGCTGGGATCTTCC
GGAAAGACAGCACTCTGATGCTGGAAATAGAGGTCCAGGCGTTGGATTTGACGACTTTGTCGACGAGGAG
GACGATATTGACAGTTATGAACTAGATAGCTCCAATTCAGCAAGTAAACTTCATTCAGTCAATTTTTCTG
ACATTGAAAGGGACTCGGGGTCAGAAGATGGTAATGAGGACCATAGACCTGGCGTCCGAGCTTATAATAA
GCGTCCAGCACGTCAACAAGCTGGTTTCTCTGGTTCTGAGGACGATGAACTTGATTTTGTCTCTGATGAC
AATATTTCTGTTAAGGAAAATTGGAAATCTAGTCATGTGGCTGGTCCAAGGCAGAAAAGTAGAGACAGGG
GCTTGACAGGTCGATCAAGGAGGGGATTAAGCTATGGTTCTGATGAAGAATTTGGCCTTGATTCCGACCT
TGATGAGGATGTAGATGAAGGTCCTCGATCTAGATTGAGTAAAGGAAAGGATTTTGATTCAGGTAGGAAG
CTATTTCAAAGGAGAGGAAGTTCTCGAATGGAAAGCGGCCTATCTTCAGGATCAGATTTTGATGATTTTG
ATAGAGGTATGCATAGACAAAAATTGAGAGGGAATCAAAGAGAAAACCATAGATGGGAAAGCGACCAGCG
ATCTCGTGGCGATAAATTCACAAAAGGGAGTGGGTTTCAATCTAGCAATTTCAGGAATGATAGAAAGAAC
ACATTTCAAAGAGAAAATAATAGACGGGGAAGCGACCACCGAGCTCGTGGCGATAAATTCACAAAAGGGA
GTGAGTTTCAATCTAACAATTTCAGAAATGGTAGAAAGAATACATTTGATGACGACTTTGACGACTTTGA
CGACAAGCCCCATCGGTCTCGTGGTGCTAATTCAAAATTTCATGGGAACAATCATGGTGGACGAGGAACT
TATGGCCCAAGAGCTAGCAAGCCAGACTTTCGGGGTTCCAAGGAAGGCTACAAGAAACATCGACCAGAAA
GGTATAATGAATATGATACAGCCACGAATAAGGATAATGTTCAGTTTAGAAACAGTAGACGTGTTATTGA
AAGATGAAGCCTAAACGAAGCTTGATTCATAACTTGGGTTCGGGTTCGATCTTCAGTGACCCTTTGTTAA
GAATTATATTCAATAGATTTCATATTCATATCACTGCTAACCAAAGATCTCTTGTGCCGTTGCTGTAAAA
TCAATGCTGTTTGATTATGTAGCGTTTCACTTAAATCACACATAATCTCTAGAGAGCGAGGCATTTGAGT
TTTTGTATCAACTTTGGTTATATGAGTGGGTTCTTAATTCCCACTATCGTTTTTTTTTAAAATCTGTTTT
GTCAATTTGTACGTCTTCTTTCTGTATTTTATTTTAAAAATTTAAATAATACAAACAAATCTTCAAAATT
TGAGAAGATATAAAAGAGCTTTTATAGAGGCATTGCTCATAGCATTTTTCTATATTATATGAAAATTGCA
AAAACTAACCTAAAGAAGGGTAGGGTGACGATTGCAATTACACTCTTGAATTGTCCATAAGAAAAGTGGA
TCTAAACTTAGAGTTCGTTTGAATTGCCTATAAAAAAAAGTTTTTTTTAAAAATATATTTTTGAAAAAAT
ATTTTTGTAAAATGAGTTTAAAATCTAAATACTCTTTATAAATGTTTATATATAAAATTGTATTTGGTTT
GTAAATTTATGAACAAATTTCATAAAAAAAAATACACAATTTTTGAATTTTTTTTATAAATATATTTTAAA
ATTAATTGTACGTTATCTTGAAATTATTATTTTTTTGGTTACTTGAAATTAAACATTAATTTCATTTTCT
GTATTAATAGTTTTTAAAAATTTAATTTTAAAAACACAAATATATCTTGAAATGTAAATATATAATTATA
ATTTTTTTTGATTTCTATCAACAAAATATAAATAAGATAAATAATTTTTTTCAAGACTATAATATCTACT
AGACAAAAAGATGTGCAATTCGATCATGTTCTTCATTCATCTTTATTTCACGAACCGAACGATCATGTTC
TTCATTGACGTCTTCATGATTTTTTTAATCACAGATGTTAAATCGAAATAAATTTGACTGAGAGAGAAAT
ATTTGTTAATCTCTATACTTAAATGTGTTCATATACATTGAAAAAAATATTCAAAGTATGTTCATATACA
TTAAAAAAAATATTCAAAGTATTTAAAAATAGAAAAAAAAAAAAACAAAAAGAACGATCGAGTAAAAAACA
CATAGTAAAAAGGAATAAAAAAAATAAATGAAGAAAAATAGATATTTGGAAGGGAATTCTCTTTAAACAT
AAAATAAAAATTTATTCTAAAATTTGGTTGCTTATGTTTTCTCTAAAATGATTTATTTTATAATCTCATT
TTTCCAAAAATTATTTCAAACGAAATGCCAAACATCTTAATTTATCTTAATATGAATTATTTTAAAAATG
AAACACTTCAAAAATCAATCCAAACACATTCTTAATCAAACCGTTTCTAACTATTCAAAATCAAGAAAAA
AAAAATATCATTCTAATCACTTAAATTTCCCTCTAAATACATTAACATTATTGAAAATATGACAATCTCT
TGAATTAAGCAAAATAAAAACGTGATAGGGAGGGCCTATCTCCCTACCTGCTTTTCCATTTTTGTCCTCT
CCAATCCAACGCTTTAATAAAATCAAGTTTTAGCAAAAACAAATTGACCTTTAAATCATCTTATGGCTAA
TAATCCAAACACCCTTCTAAACTCATTTTCTAAATGTTAGGTAGAAAGTAATTAAGCAATGTTGTTAAGT
TATAAGTCCTCATGCTATGTATGGGTTCCATCCATCCATCCATGGTCATTTTAAACGTGGAACCACTTGA

TAAAAAGGACTAAAACAAGAATCAAATAATTGAGAGATCGATTGTATAATATTTATACGCAAGTTTTTGG
TTTTCGAGTTTCTATTTCGAAGAGACAAAAACCATCAAAGTCTTGTTTGGGTAATTTTGTTTTTATTTTT
TGCTTTTGGAAATTAAGTTTATTGACACTATTTCTACATCTAAATTTATATTTTTATTGTTGTTATTTAC
TTTTCATTAATAATTTGAAAAATTAAACTAAATTTGTTGCATCATATTTAAAAAATGTACAAATTATTGT
AAGAAATGGTCTTGATTTAAAAAATAAGATCAACAAAAAAACAAAATGGTTGTCAAACGGAATTTTTAGT
TTTGAGAGACGAAACAAAAAAATATGTTTTCGAGGATTGTTTTGAGTTTTTTTTTTCTTAAAACAAAATG
GACAGTTAGTTGTGTTTTTCTTTTCCATTTTTTTAAAAAATCAAAACAAGTTTTTGGTGTCTTTTTCTTA
GTTCTATATATTTTATTAAAGAATAAAATCAAGTTATTTTGACAATAATTCTCTATCTAATATAAACATA
TAAAATTGTATATTATATGTTTCTTTTTTGAGTACTATAAAGTTACATTTTTGTTTTAACTTTTTTTTTT
TTTTAAAATATGTATATATAAAACATTATAAATATTGACAATGATCACGAATGAAATGAAGAAAAAATTA
TTGACAAAATACAAGAAAATAATGTCAATAATACTTTTAATTTATTGATATATCCTTGAGGAAATACATA
TCATACCATTGGTGGGTCATTTATTCGTAAACACTCTCTAATGAAAAGTTTGAATGAAATTGTTTATTCA
TATTTTTCAGAGGCTTTTTATTTTGTTTACGATTGCAAGATTGATAAAAACATTCGAAAAAATTAGGCCT
ATTGTTCAAAAGTTTGATTTTTTTTCTTTCTGTAAAAACAACAATTTTGAAGTTCAATCCAATTTATATG
ATGAATTGAAATGGAAAAGTCAAATACTTTCGTTAATTTACGAACTTGCAGATAAATTTGAATGCTATAT
GTGATTGTTATCTGATTGTCATTTGGTAACCTTCTATATCTCTTTGTTTTTTTCTTTTTCTTTTTTATTA
TTATTTTAGTTTTAGTTAGCTTTGTGAGATAATCATTTGATTACTTTCTATTTTTTTCTAATTTTATTTA
ATTTTAATTCTGAGATAGTTATTTGATTATTTTCTGATTGTCATAGTAATTTTTTTATTGTTTGATTGTC
TTTTGATTACCATCTAATTGGTTTCTATTCTTTGACACGTATAATTTTTATTTGATATTGATTGTTATCT
AATTTCACTATCGTATATACATTTATTTGTTTCCTTAGCTTCATGTGACTAAGGTTGTATGTTTATTGTA
CCAGTGAATATTTTGGGAATTGTGTACCCAACACCTATTAAATTATTGTACCATTCCTTGTCACTACACC
ACAAAGCCAACTTTAGTTTCAAAAGAACACCACAAAATATTTTTGGAGTACTTTAGGGTTTTGAGATGTG
GGTTTTCCATTTGCTAACAAGACCAAGGATTTTTTTTAGAAAAATTTATTTAATAATGTTGACATGACAA
ATTATCCCATGACTTCAAATATATACATTTCTTTAAAAATTATCAAAAACATTAAGCCCTATAATCGGTG
TTAACATTAAGCAAGCTAGCAAGGGAATATGTTAAAATTAATGAAGATGTCTCTAAATTTCATCCACATT
TTATAAAAGCATGGAAATTAATTAAACCTAAGCATGAAAAACACATTTAACCTACCAACATAATTGAGCA
TGACGTGAATGGGTGTGACAACATCACTTCGATCAACAACTTAATTTCAAATACTAAACAAGTTCTCAAT
AAGGAGGTGTTATTAGGGTCAGCAACCTTAAGTTTATTAAAGTATAATTTTATTTCCTATTAAATCGAAT
TTATATTATTATATAATTAGCGATTTACATCAACACAAAACTAAATCAAGAGTGCAAAAAACTAAACAAA
AATTTGTAGATGGTTGAGATAGGATAACATGAGGTTGTAGATGATTAATATGTGGCCATCAATTTTCTCT
ATCCTCAATTTTAATAACCTTACTACTAAGTACTTAATTAAGTACTTAATTTTCTCTATCCTCAAAAGTA
AAATTAAGTACTTAACATTACATTGTGTTTTTAAATGTAATAAAAAGCATTAAAATAAAAAATATGGATT
TAAACAATACTTTACTTAATTTAGACACTTAAAAGAAGTTTTGGTAATAAGCATATATTTGTTACACACT
TTTCAAGATTTTGTCTAGAGAAATATTTACAAACATCCTCTTTTCCTACAAACAAGAGTTTTCTATACTC
TCCTAATATTGTGGATTTTCTCATTCACATTTTTCTTTAATTTTTTAAATTTTATATACGATTTTATTTC
AAATATGGTTAATTAATTAATTAATATTAGTTTAAACAAAGTAAAATAATTATTTGTTTTAGTGAATCAA
TACTAATTCACTAAAATATTTAGTTAATTTATTTCTTCATATAAAATTTATTAAATGAATGCAATTACTT
ATTCACATAAATTATTAGTTAATTTGCTCACAATATTTTAAATATTTTTAATTAATAATGTAGGATTAGT
TAACTTTAGGATAAAAAATCATTTTTTCAATTATCATAATTAGATTATTTAGTGTTAATAATAATTTGTT
TATATAAAATCACTCATTATATTATCATTATTTTGATTACGGTGTTTCGATAGGCGTATACCTCTCTTTG
TCTTTTGATTGTATTGAGCTTTCCTTTCCTCTAGCTCTTGATGTACTCTTTGCCTAGCTTATTTTATTGT
ATGAATATTTTGATAAGAGGTCAACAAATATATCAACCTAATTGAGATGTTCTGATGCACCTTTAACATA
CTAGTTAGTCGCCCTTCAGTATTTCTTTAAAATAAAAAAAATTAGATAGTAATTATGCAAAGCCCATTTT
TAGCTTTGAGTTTTGTTATTTGTTGTTGGTTTTGGACTTTGTGTGAAGTTTTATAATTAATTTAGCTGAG
TTTATGGGTTAAATTTGGTGTTTGTGATTATGGTTTTCTTCTTTTTCATGTGGATGAATTTTGTAGATTT
CATTTTTAGTGATGAAATTGTGCATTGTACTTAAACAGTAACATAATTTTGAGACCCGTATATGTAAGGC
TTTCCATTTGAGTACCCTCTACATTTCAATTACCAAACTTAAAAGTTATAGAAAAGAACAATCCACTGCC
AAGCTTAAAAGTTACTATTGTTGAAGTGTGTATGTGCTGTGTTGTTGAAGTGAGCGTGTTGTGTTGTTGA
AGTGAATGTGCTGTCTTGTTGAATCAATTTTTTTCCTTTTCTTTTCTTACTAGGTTTTCCCAGAATCTACT
GGAGCAGTGAAACCATATTTGCGGCAATTCATTGATGTATAGGCCTTTTTGTAGGTTTGGTAGGAATATT
TTTTGTTCAAATGTACTTATTTAAATAGCTTGTTCGTATAAAAATATATATGTACCAACAATTATACAAA
TATTAGAAAGTATATACTTCTTTCTATTAAAGCGTTCACCGTTTCTTTTCATATGTGATTGTATTAGTCT
GTGCAATGGTATGTGTAATGGCAGGGTAATAAGAAAAACAGGATTCAATAAAAAAAATAGGGCAAGAAAA
ACAGGGACTGAAAACATAGTTATGATATTTAAATCTAAAAAACACTGTTATTGAAAACGTTTTCTTGACA
GTTAATTAAAGTCATGATAAGTGAAATGATGACAGTTAATAAGTGTCATAAAAGATAAACAATGACATTT
AATATCTGTCAAACAATATTAACAATGACCATTATTCGCTGTCATAAAATGTAAACAATGACGTTATATG
TTGTCATAAAATATAAACAATGATAGTTATTATCTGTCATCGAAAATAAATAATGACAGTTATAATCTGC
CATAAAATAATGAATTTGTGACATTTATTCTTTGTCATGAAAAACACATTTCGTGACAGTTTTGTAGAAT
TGTCATAAAATACTTTCCATAACTGCGATATTAATGACTGCAAATAACTGTCACGAAAGCTTTCTATGAC
AACATTTAATTGTCATCGTAGACCCCTTTTCTACTAGTGTAGTCGGTTTAATTTTAGTATTAGTTTCAA

CAAAAAAGAAATTTAGTATTAGGTATTTTAATTTAGTATGAACATTCATTTTTAATAGCACTTGTAATTT
TGATCGTCTGATTAAAAATGATTTCTCATTGTATTAGTTAATTAATATTTCTATCGATTGATCTATATTT
TAAAGGGTATTTTAAAAAAAAAGCAAATATCTAAACTATTAACAAAATATAGCGAAATCCATTAAATTTC
TTTTTTTCTCTCCATATTTTGTTAATAGTTTCATTTTCTTGCTATCCATAACAATTCTCTTATTGAGATT
AAATTTTATCAAAACATATTCAAATCCTAATAACATTTATGATTTCCATTAACAAACCAAGCACATCAAT
CTTACTTTCACACACACATAAATATGTATATATTCTGATATCCATAAAACCTTAAACCAAATAGTTCTTA
AAGAGATGTGAAGTTGTGAAATTCATAGAATAAAAAGTAAAAGAATTATCTAAGATTAATGTTTTTTTAA
TTAAAATATAATTTATATTCAATAATTGACCAAACATCCCATATGATTTTGTTTGATAACACCATAAAAT
CTTTTGAGATTATGTTGTTAAGAGTGATGTTGATAGATACACTCATTTAGGTCATAGTAGGTTAAATATA
ATGTTTTGGATATCAATCAAAGACCTCACTGTTCTTTTGTTTCAAATGAGAACCACAAAGTGTATTCAAA
TAATTATTGGTACTATATTTGACCTCATATGATTAATATGTATGTATGTATGTATGTATGTATGTATGTA
TGTATGTATGTATGTATGTATGTATGTATGTATGTATGTATGTATGTATGTATGTATGTATGTATGTATG
TATGTATGTATGTATGTATGTATGTATGTATGTATGTATGTATGTATGTATGTATGTATGTATGTATGTA
TGTATGTATGTATGTATGTATGGACAATTATTGATGGACTTTGGATTAAATGATAACTCTTATCCCGTTG
GTTTCACCTTTCTTTTTTTTTGTTGTAATTTTAGACATACATTTATTGTGTTTGTTTCGCAAAATTCACA
TTTAAAACCAACAATATTTATTATCTAGAGAACTTTAAAATAATCCATCCACTAAGGATTTTTCAATTCT
CTGAAGTATTTGGACAGAGAGAAACAATAGAATTTTTGGTACTTTCACCTATCAAAAATCTCAGTTAAAC
ATATAGAAAAAGTGTAGAAATTTCATAGACAATTGGTGTAGTAGAGATCCTTGTTTTTGTAACAATAGCC
TTAAATCTTAATGTCTTTTGTAATTAGCTATCGGGCTTTTCTCTAGCCCCTTTTATCAATAATATTTAGC
GTTGTGTTCGGTCCTAGGCTTCTATTGGAAGTCCTAAGTATCCCACTTATGTCGTTTCTCTTCAAAAAAT
AAAAAAAAAAAGAAATGCAAAAGTGTGAGTTTTCTATGTCGGGGAGATTGAAAAGAAAAGAATTTAGCTA
AGTTAATTAATAAATTATCAAGTATTCATTATTATTTATTTTAATGAATGGAAAACTACATTTTTATTTC
TCAAGTTTTGATGAATTTGTGCTTTTGGTCTTTGAGTTTTAAAAATGGACATTTTTGGTCTTCAGGTTTA
TAAAAATAGATCCATTTGATCATCGATTTTTCAAAATGTACATTTTTAGTTATTGAGTTTTAAAAAATTA
GGTTTATAAAGTCCTGAAAGTATTTTATTTTTGATTTTTTTTTAGAAATAATTATATGATATTTAAAATT
GAAAAATAGTTTTTACAGAGAATATGATTTTTAGAAATTATATACTTCTAATTTAAAATGTCATATAGTA
TAAAGTTGTTTGAAGGGTTTTTTAAACCTATTTTTAAAAATTCAGGACTAAGAAGGTACATTTTGAAAAC
TTAAAACCAAATAAAATCTATTTTTATGAACATGGGAACTAAAATGTTGATTTTTAAAACTTAGGGACCA
AACACACGTATTCATTAAAAACCTAAAGACTAGAAAGGTTTATTTTCCTTTTAGTTAATTGTCAAATATA
AATAAATTATACAGACTTAATAAATTTAAAGAAATATCTTTACTCGCTATTTTAACTATTTAATATAAAT
TATTATCGTTAATTATCAATATTATAACTAATTTATATTAGTTTAATTGTTTTTATAAGTAATTAATTTA
ATTATATTTAATTTAAAAGTATTCATTTATTTTATTTATTAAATAATTAATTACTAAGGATTTCTTAAAA
ATAATAAAGTAACAAAATATTTACGCTTTCACAGAAAAATCACTTAAGAATAAAAATCTATTACATTTAT
TTTTCAGTGAGGGATAAATATTTTGTTTATTTTACTATTTTTGATAATTTTATTTAACTATTACGAAAAT
AAATAAATTTAATTTATTGATCTTTTGTATTTAAATAATTAATTATTTAATTTTATTTTCTATATATAAA
TTATTGAATTAAATATTTAACTTAGATATTAATCAAAACATTTAAAATCCAAACATTCAAATCTCATCTA
TTTAATATTTATATTCATGATGCAAATCATTTGATTAGTCGTAACTCCTCTCCCATGTATGGCCAATAAT
TTTCCATGTCACCTAAATTTTGTACCCCAATTTTTGTGAATATTTACTTATATTACACTATTTTAAAAAG
TTCTAGGGTGTGTTTGGGGTGAGGTTTTAGGGGGAAAATGATTAGAAAATTGGGCCAAACCGTGTTTGGC
CTAAGGATTATGATACATGGGGATTAGGATTATCTTAATCCCTAAATAAACCTATCTTATTCTCTCTTTT
ACTTTCTTACAACCCTACATTACCCTATCAAACTAACCAAATCCATATTCTATTATTATTTTTTAAATTA
CTACAATCATAACCTTCCCCCGAACACATATTATTATAACACTACTATCATAATCCCTCCTCCAAACACA
TACTATCATAACACTACCTTTTATATTCTTTCCCCCAAAAACATATTACCATAACAATTCCAATAATAAC
CTTTCTCTCAAATACATATTATCATAACACTAAGATTATCATAATCTTAGGATTATTATAATCATTTTCC
CTCATAACTCTTTCTTTTTCCCAAACGCATCCAAAATGTTAATGCACGAGGGCAGAAGTTGGAATTTCGA
ACAAATCTATAAAGAAAAGGGTAGGGGTCCCACACAAAATTCAAATACAATTTCATTGATCCGTCAACCT
TTCGATTTCATTCAGATTACATTAGTTTATCGTGGGTTTTGAAATGGGACCTCACTTGTAATCCGAACAA
ATACAAAAAGCGAATAAACAATGACGATTGTAATTAAATAGGTCCACTTTTCAAATAACGGTACATTTTT
TGCTTTACACCATCATTAACATGACTTAAGTGTTAAATTTTAGAGAGAAAATTTTGGTTAAATTACAAAA
AAAAAAAAAAAAAAGTTTATGAACTTTGTAGTTTGTGTAAAAAATACTAATTTTCAAAAGTTTCAAATAT
GTTCTTAAACTTAAAAAAAAAAAAATTAAAAAAACACCATTACTGTTAGTTATGGATGAAAGCCGTTAAAA
TTTTGTTTCAAAAATGCCCTTGAACTATTGAAAAATTTCATAAATGTCGTTGAACTTAAAAAAAAGTACA
AAAATACCCTTATTGATCCAAAATGTACACCAATTTTCTCACCAACCAAAATTTTAAATTAAAACCATAG
TGTTAAATTTATTTAACTATTAACAGATCATGAACACAACCAATCTCAATAATTACTGTTGTAATTAACA
TACAATTAATTGTCAAATAAAAAAATGTATTTGACTATTAACACACAGTAATATTCTCATAGATCTATAA
GTATGAGATTTTTTTATTTGACTAATTATTGTTTTAGTATGTTCCAAGAAGAAATTTTTATTTTGAAATT
TGTGTAAGATTTTATGTTTTAACCAAGGTTTAAAATATCGATGTTGATTGGTATATCAAAATCTTAATCT
TACATAAATTTAGATTTTGATGGATATTTCTTGAAAAATTATAAAAAAATAAAAAATAAAAAATAAATTT
AAATTAGTTAATATATATATATATATATATATATATATTATTTTCAAATAACCAAACATGTTTGCTATTTAT
ATTATATTTATATTAGTGCCATTTAGATGTTTATTTCTTGTGATTCGTGGGGTTTTTGTACGATATGTAA

TGGAAATATCGGTTCACCCATTCTATCAATATTGAACCATGGAAATATAAAATTTAACTATGATTTTAAC
TTAAAAGTTTGGTTTTTATTCTAGGCGTTAAAAAGTTTGATGTAAAAATTGAATTAATGAAATTATTTTT
TTTCTAATTTTTTTTTAAAGTTTAAAGGTATTTATTAAATTTTTTGATAGTTTTCCATGCAAAACTAACA
GTAAGAGTACTTTTGAACAATTTTCAAAAATTTAAGAATATTTTTAAAAGTTTGAAAATTTAAGGATATT
TTTGACACAGGCCACAAAGTTATTTATAATTGTGTTTAAAATCAATAGTTTGAGCTCTACTTTGAATTAA
ACGAAGATGAGAGAGAGTGAGTATATGAAACAAAGCAAAAGCATTATTTTAATTTTAAAAAAAATGAAAA
TATGTTATTGAGAAAGAGAGAAATATATTTTTTAAAATTGGACGAAGAAGACAAAAGTACTATTGCCTTA
ATTTGGCTATTTTTTATTTCGTGATTAACCAACTTTTATAAAAACAAATATTATTTAATTACTTTTAAAG
TTTTGTGGTCAGTTTTGGCTAAAGTAACTAGACTATTTAGTTAGAGTAACAAAGGTGGGGGAGAAATACT
CAATATGGTAACTTTTTGAAATTAGTTTTTTAATAAAGGCTCTATGCAATTCTGAGATGTATGCGAAAGC
TAGTATTATTTTTAAAAGCAAAAAATCTCACCATTTTAAAGAAATGATTTGGCTATAAATAATCTCTTCT
TTTTCATCCACACAAAACAACATAAAGAAAAGGCCACACAAAAAACCCTTGAAATTTTTACAACTTTCAC
ATCCTTTTTTCTTCATCTAAAATCTCTAACCTCCATATCTTCTTAAAAAAAAAACAAAATATCTACAACA
TCCATTTTATCTTCTCTTCTAGCAACCCCTAAATTTTCATCTTTCTTCCTACAATGCCAAGAATCCAACA
TAAAAGAAAAAACTTTAACTTAGTTGGAGGTATATGGTAAGATTTTGGTCCTAATGATCCGCACCACAAA
CAATAAATGAGTTTAGGGTCTTGGGATTTCAATCTTTCTTTTTTCTTCTTCTTATTGTTTTGGTTATTTT
TCACTCAAAGTTCTTTATTTTTTCTTATTCTTATTATTCTCATTATTTTATTCATCTTTCTCCAAGCTTC
CATAAAAAATGTAAAAAAAAAAAAAGAAAGAGAAATAAAAGATTAAATGATTAAATATTTGCTTATTATT
CGTGTTCTTATTTCTCTACTTTTCTCTCTTATAATTATTTATTTATTATCTTGCGAGTTTTTTTATGTAT
ATATAGTGTTATTTATTACATGTTTCTTCAGATTGTTTATCTCTTATTTATTTATTATTATGTATTTCCT
TTGTTAATTTATTTCTAAACTTTATTCTCTATATATTGTATCTTTAATTTTTTATTATTAGTATTTTTAT
TTATTTATTTAATTTTTTTCTTTTTATAGTATTGATTATTGTTTCTTTAATATTTTCTCATCTTGATTAT
TGCTCTTTTAATATGTTCTCGTCTTTATATGTTATTTAATATTTTATTTTTTTCTTTCTTTTACGGTCAA
TTTAATATATTATTGCTAATAATTGTTTAAATTTGTTAACATTTTAAATTATTATTTTTTTTAAAAAAAA
ATCCGACGACGGAATCTAGGATATTTGAGAGTCCAATTTCTTAGAATTCTTGAGGTGAGGAAATCGATTG
TTTCGAAGTCCGAGATCGATCTCCAATATGTTTTTGTTAAAATCTCAAATGTTATTTTATGTTTGCATAA
TTTATTATGATGCTTCTTTGTTCTTGTTTCTACTTTATTTAATTTTCCTACTAAGTCTCATTTGTTTCTT
ATTTAAAATTTTCAACTCTTTCTAATTAAAATTCTCAATATTTTAAAATTCTTTCTGATTTAAAATTCTC
CAAGTTTTAAAATATTTCACTAAAGTTTTTTTTTTTTTTTAAAAAATCATTTAGAATCTTTTCTTTTAAA
GTTATAACTTTTTTTTTAATGTTTTTCAAACATTAAAAATTTAATATATGATTTCATAAGCTTTCTTAAT
CAATCTTTTCTAATAACTTATACCGTTTTTCTCAAACAAAATCCTACCGGCGGAATCTAGAAAATTTGAG
ATTTCGATTCTCTAGAATTCTTGAGATGAGGGATCACATCTTTTAGGAGTCCAAGATTTGATTAAAAAAA
ATGATTTTAATTAATGTTTTTTTAAAAAAATCTCTTTATTAGAAAGCTATTTCAATGATGGATTTTGAGA
AATTGTACTAATTTCTCACTTTTTTGAGGTGAGAAGATTTTCTTTAATATAGTATAATTGATGGAAAGCT
CTCAACTTATTAATGAGATCATTGCTTCAAATATTGGAATAATGAGGGGTAAAATAAGACATTAGTTTTA
AAATAAATTAAAGAATATTTTTAATTCAAGATTTGAAATTTGGTCATCGTTTTCTAAATGGGTGTTGTGA
GGTGGTAACACCTTCCTCATACACAAATAACTCCCGAACTCAAGTATTAAATGACACTCTTTAAATGCTG
TTGGTAACTCGGGGGGTATCCTGATTATGTGGGATGATCTCAGGTTCAATGTGACAGATTTTATAGGTCT
CTTCTCCATCTCCATCAAAATTAATGTTCCAAATGGGCCTCCTAATTCTGCTTGGTGGCTCTCGACTATT
TATGGGCCTGCAAATCATAGAAATAGAAACAACTTTTGGTCTGAACTCGAAGACCTCAATCCAATATGCT
CTCCAAACTGGCTTTTAGCTAGCGATTTCAATGTAGTTAGATATACAGTTGAGGCTTCGGCTCAAAATTC
GAGCACTTACAGTATGAGAAAGTTTAATGCCTTCATTACAAACAACAACCTTATTGATCCTTCACTCACA
AATGCCAAATATACTTGGTCTAATCTCAGAGTTCAGCGGGTGCTTTCTAGAATCGACAGATTTCTATACA
CTGCCGGATGGGAAAATATGTTCTCCTTGCACTATTCCAAAGCTCTCTCTAGAATCACTTCGGACCACTT
TCCGCTTCTCCTTGAGTCTTCAAATATAAGTTGGGGACCTCCTCCATACAAATTTATAAACTAGCACTTA
AAGAAAAATTGGTTTAAACACAACATTGACTCGTGGTGGAAAAATTCGAGGCAGACCAGTCATCCGGGTT
ACTCTTTTATGTAGAAGCTAAAAAATTTATCCTTCTCCATAAAGGTTGAGTACAAAAAGAACAAAAAAGT
CAATGAAGAAGATAAAAGAGCTTGGATTAAAGAAATTGACAAAATTGATAAACTTGAAGCAGCAAGCCAA
ATTTCTGAAGCTCAGAGAAGGCGCAGAACCTCCTTAAAAGCTGAGATCAACCAACACGACTATAGAGAAG
CTCAAATTTGGTCCCAGAAATGTAAGAGACTATGGACCTTAGAAGGAGATGAAAACTCATCCTTTTTCCA
CAGAATCTGTATTACAAGGCAAAAAAGAAAAAGCTACATATCAAATATTATATCTAAAAATGGAGATCCA
TGTACTACCAATGAGGATATATAAAAAGCTTTCTTGGATCATTTTGAAGAAATTTACAACGGCAGCGTCT
CTGAATCTCCTTGGCTCATAGACAACCTTCATTTATCTCCAATATCCTTTAGACATCTAGAAAATCTGTG
CTCTCTGTTCACTGAAGGGGAAATTCATTCAACACTTTCTTCTTTCACAAATAACAAAAGTCCATGGCTA
GACGATTTTACTATGGAATTCTACAAATCAACTTGGCATCTCATAAAAGGCGATATTTGAAATATCTTCA
AATCAACTTGCATCAGCAGTGTCCAATACTCCATCATTATAAATGGCAGACCAAGAGGCAAAATCCAACC
TTCCCGTGACATTCGGCAAGGAGACCCAATCTCCCCTTTTATTTTTTGTCCTTGCTATGGACTATCTTAG
CAGGCTAATTGAATCATTGGGCGATAAAATTGAAGGAGTAAGGATGAACAACAATCTCAACCTCACTCAC
CTCCTTTTTGCAGACGACATCCTTCTCTTTGTAGAAGACAATGAAGACTCCCTCAACAATCTAAAAATTG
TCATTCAACTCTTTCAACTGGCTTCGGGTCTAAATGTAAATTTAAACAAATCCACCATTACTCCTATAAA

TGTTGATGCTGCTAGAACAAACCAAGTTGCTTCATCATGGGGTATTTCGACACAATTTCTTCCCACAAGC
TACCTGGGAGTGCCTCTCGGTGGAAAGGCGCCCATTTTAACAAGCTTCTGGAAAAATATTGAAGAAAAAG
TCAGCAAAAAATTGTCCAGCTAGAACTACTTTATGCTTTCCAAGGAGGAAAAATTACTTTGATAAATTCC
ACACTCTCCGGCCTCTCCACTTACCAGCTTTCGGTTTTTGAAGCTCCTTCCTCCATCTACAAAAGTGTGG
AAAAAACCTGGGGAAAATTCCTTTGGAATAGTCCAAATGAATTCCAAAAGTTGCACCTGGTTAGATGGTC
GGTGGCCACTTCTCCAAAAATGAAAGGGGGTCTTGGTATCAGCCGCATCAAAGATACTAACTTCGCGCTC
TTAAATAAGTGGTTATGGAGATTCATTCAAGAAGAAAACCTCATGTGGAAAAGATTTATAGCAACAAAAT
ATGATAGCTCAGTTTCGGGAGATATTCCAAACAGAATACAGTAGCAGCAGATCCCCATGGCACTCTATCA
TTCAAGGCCTGGACTGGTTTCGATCTCAGATTACCTGGAAAATTAAAAATGACAGCAACTTTTCCTTTTG
GCATAGTCATGCATTGGCACTCAAACAACCCTGTCTCTACGCACTATCCGAGATTATTTTCTTTATCTAC
AAAACAAAATAGCTCCATAAGAGACATGTGGAATTCTGAAGAGCTTGATTGAGGTTTTAGCCCAAGAAGA
CCTATGAGGGAATGTGATGTATCTTTGTGGACAGAGCTTAAAGCCTCAATAAATGCTCAAATTTCTGCCG
TTGGTAGTGACACTCCAATTTGGAACCTTAATGCTGATGGCATATTTTCGATTGCTTCGGTTAAAAAAGC
AATCCACTTAAGCGACCACGGAGCTATACCTTTTCCAGACCATCATATTTTTATAAATCTTTGGAAATCC
AGCTTGCCCAAAAAGTGCAAATTCTTTATATGAACCCTGCTATATGATAGTATAAACACAGCAGATCAAC
TTGTTAAAAGGATCCCTAATCTGTGTTCAAGGCCAAACTGGTATGTCCTTTGCAGAAGAAATGAAGAAGA
CATGAATCATCTTTTTATCTTTTGTTCCTTTGCAAAAATTATTTGGACCCACATTGCCGCCCTCTTTAAT
AGAAGTTTAATGTTCCTCAGTCCCAAAGAAATATGTCTACATATCTGCGATTGGAAACAAAAGAGAAAAA
AAGAACATCATTCTCTTCAACACCATTGCATCTGCTCTTTGGAATATTTGGCTGGAAAGGAATGAGAGAA
TCTTCAATGGCAATGAAAAGACTGCAAAGGAAACTTGGGAAAACATAAAAGCAATATCTGGGCTTTGGAC
TAGTAGATCAAATCTATTTTCCAGTTATCCAACTTCCTCCATAGCCCTGAACTTACATGCTTTTATTTAG
CTTATGTGCTTTTCTTTTCTTTTGGATTCAGGGCTTATCTCCAGCCCTTTTCTTTGGTGTTTTCTTTGTA
GCGCTTAGCATTTTCTACCTTCTTCCTTGTACTGTTTTGCCGATTATTTAATGAAGCGGGAATGATGAGG
GTGCTAAGGGGGTGTTCACCTAGTGGAGATGTTCGGGTGCACCTACTGACCCATAGTATCATTTTTCTAA
AAAAAACTCCCGAACTCAACTCTAGTTTTTGCAGACAAGTTTTTTAATGACTTTATTTTAAAATTGTTTA
CTTTATTTCGGTGCTCAATCACACCGTAAAAAGAATTGATAACGACTCATATTTTGTTTCAAAACTAACC
CATTTTTTAGGATGTCGGCCGTTGTGCGTTGTCTCGGTCACATGGTGACACGGTTTATGTTTGTTCTGGA
ATTAATTTATGATTTTTGATATTAATTATGGAATTTTAAGTTTTTCTATGAGTTTCAATTTTTTAATAAT
TTATAAAATATATGATTATATATATGCATGATTTCGGAGAGCTTGATATGATAATTTCATGAATTGTTCA
TGAATTTAAGCAATGTCTTGTTTTAGGATTGTTTGCTGGTTGATTTTAATAAATAAACATGGATAAATCC
AACCTAGAATTGAATTGCATGAATATTTAGTCAGACGCTTGAAAACTAAATACTCAACGCTTTACTCGTC
CTAGATCGTAATACAATTGATAAGAAAACCCATTTTGACCGGCCAAGCTAACTGGGATCCAATTTTACTT
TTAGCAATTTTATTTCATTTGCAATTAACACTCAAATTCTCCCCATTTGGTTACTTTGGTTGGAAACAGT
TATCTTGAGAAATTCATCGTGCTTACTGTGTTCGACCTAAACTTACTGTTAAAAGTAGTTCTTGTGAAAT
ATTTCAATTTATTTATTTGGTCCTTGGATTTAGGTGGCGAAATCCGTCCGAGGAATCAACTTTATTTTAC
ACATTAGGTTTTTGCTACTTCAATTTCATTATAGCACAATTTCACATATATTTGGATAAAAAAGAAAAGC
TTAGCCACTCATAATCAAAAAAATAAATGATTAAAATTGAGAGATGAATAAAATTTCATGGTGTACTTAA
CGAGTTGATGAACTAATAATGATTAACTAACAATGCCTAAAAGAAAAGTTTAAGTTAGAATGAACATTTC
AATAAAATTAGAACTTACTGTTTGAGGAAAGAACTGCTAGAAATACAAATGAAATAAATAACACAAAACG
GAAGCAAATGGTTACGGAATCAATTACAAGTTAGAAAGAATGCCTAAATCGATTATTTAAACTGTTACAG
AATTGACAAATCGATTACTCCTAAGATCCCACCACAAAAATTGTGGTTCTAGTTTTTTCTCTTCACTAAT
AATATTTTTTCAAGTTTTTATACACAATAATCTGTTTGTTTATTTATTATTTTTTTCAGCTATAAATACC
TGATGAAATTCACATCTTCAAATCATCCATTCTTCAGTTCAGTTAGTAAAATAGTGAGAAAAAAATGATT
GGGAAGATTATTCTAGGAGATGCCCTTAAAATCACTGGCAACGTTCTTAGAACGACCGGGGACGTTGCCG
GGTCCATCGTAAATGCTGGAGGGAATTTTATCGACCGTGCTAGCGATATCGGTCAGCTCGGGAAGAAGAA
GATCAAAGGGAAGGTGATTCTGATTAGAAGCAATGTCTTGAACATCACTGAGTTTCATTCTAGTATTCTT
GATGGTTTCACTGAGCTGTTGGGCAGTGGAATTGTAATGCAACTCGTGAGTGCAACTGAGATTGATAGTC
ATTGTAAGTTTCTCTTTTTAATAAATTACTTTTACTATTCTTGATTAGAATAATGGAACAAACCTACTCC
TATCTTTTTACAATGGTATGGTATATATTGAAAATGCATGGACCCATGACACATCATTATTTTTTGTGGA
TGAAAATATATACGTGGGTAAAAAGGTTGTGTAATTTAGTGTACCGACCTTTTACATCTTTAAACCAATG
AAAATATCATATAATTGTCATCAATTATACCTACGGCTACTTTCTTCTCTCTTACTTATTATGTATGTAA
CACTGTTTGTGTAGTCAGAACAATTATTATCATTATTATATTATTCTTATCGATTAGACTATAACAAATA
TTCTTGAACTTTTCAACTTTCCATTTTAACTATTGGAACGTGAGTTACTATTATTCCTTTCTTTCCTGAT
CAAACACTTAATTTTATAAAAAAGAAATTGTGTCTACTTACAAGTTTTCAAAGTTCAAGGATATAATTAT
TGAAACCAACCCCACAATATGTGTATGTATATTTGCCACTGCAGTCATATTATTTTGTACTTGAGCGTTT
TTTAATTCTTTTGTTAGATCACTGATATAATATTAAATCTGTCGTAATTTGTTGCATGATTTTTACACCA
TGCACGGTGTGTTCAAATTCTAATAAAAAGTGATTAAACCATTAACTATTAAGTTAAGTACTACAATTAC
TTCTATACGAATTAAACGTTAAAAATGAATTATTTAGTTAACACTACTCTACTTCTAACGTTGAAGCGTC
GCTTCAATGATCCAAATGAAACTTATATGTCGAAGCGCCACAGTGTTTGCTACGATGCTCTCTTTCTTCT
TCTAAGTGCAAACATTTATTAAAAAACAAACTATAATTTAAACCTAAGGGACATATATATAATCCTATCA

AGTTAAAGCTTTTGATTTGATTGATAATTTAATGCATCATGTGTTTGAATATAGCGAACGAGAGACAAGG
GAAAGTCGGAAGGCGAGCTTATTTGGAGAAGTGGTTGACTTCATTCCCACCAATATTTGCTGAAGAGTCG
GTGTTTGAAATAAACTTTGAATGGGAAGATGATTTTGGATATCCAGGAGCTTTCTATATAAGAAATGGAC
ATACAAGTGAATTTTTTCTCAAATCCCTCACTCTTGAAGATGTTCCTAACTATGGAAAAGTCCATTTTGA
TTGCAACTCATGGGTTTACCCTCAAAGAAGATACAATAAAGATAGAATTTTTTTTGCCAACAAGGTATAT
TATGTAGGTATAGTCTTCATAATAATTTTATTTTTCGGGTGTATGATGATGTTAAAATTGTGCCAACCTA
ATTGAGATGTCTACATGCATCTTTTTGTACCTTTTTTTGAACTCTAATATCTTGCTAAAAGAATAAAGTA
GGATGATAAGGATGCTATGGAGGTGGCAATCGAATCGAGATTGTCTAAGAAATTTTTTTTATTTAATTTC
TTATTTATGCTCATTTTCTTTCTTGCTTTTTAATTATGACTTTCACATTTCTTAAAAACCCACTTCAACT
TCAGTAAAGAAAAACAAACTTGAACCGGAGAGACCATCATTGTGTTATAACTTTCAAAAACTATCGTTTT
AAAAATTTAGTTTAGATTTTGAAAAGATTAGTAGAGAGTAAAATATAAAACATAGAAACTTGTGAGTAAA
AGTAGTGCTTGAAACTAAGACTCACGTTTAGTAATCATAGTTGAAAAATGTCGCAAAACGCAATAAAAAA
TACTTATCAAACAACTTTCGAATTTTTCATCATCATCATTATTTTAATCTATCTTGTAATTTATCATACT
AATTTTTGTTTTGGCAGACATATCTCCCAAATGAAACACCAAAAGCACTTCGTAAGTACAGAGAAGAAGA
GCTATTAAATCTAAGAGGAGATGGAAAAGGAGAGCATCAAGAATGGGATAGAATTTATGATTATGATGTT
TACAACGACATTGCTGATCCTGATGCAGGTCACCAATTTGTTCGTCCTATTCTTGGAGGGAGTACTAGTT
ATCCTTATCCTCGCAGAGGAAGAACAGGAAGACCACGATCAAGAAGAGGTTCAAATTAATTATAATAATA
TTTTTCACCAACTTCATTAGTTCTTAGGGCTCAGTTTTGGAGTCATTTAGTTTTTAATTTTTGACGATAA
TTAAGCCTATAAACACTTATTTATGATCTGTCCCGTTGGATAAAAATCTGAAATTTGTTTTATATTTATA
AATTCAGCGAGTTCGGGAAATTAATTGTTCCACCGACCATTTCGATTTTGTTTCGAAAAAGTTAATTTTG
GGTTTTGTGTTTCGAATAGTGAAAATTCTGAAAATAATAATACTTTTGTGCTTTCATTTTAACTGGATTA
TGAACCGTAAATTTTAAAATGTAGAATTATATTAAAGAAATTAGAATAAAATGTTTAGAAATGTGGTATG
TCGGGTTATAGACTGGATTCATTTTCTTTTTAAATTGAAAGAGATTATGTGATCTATCATAAATTATCTA
GTATTACGAAGCAATAGTTATAATTTTTTTCTAACATATAATTTGTTAATAAAAAGTTAATAATAGTTTA
CCTTGTCCGTGATATTTTGTGGAGTAATTACAACTATATAATATATAGTATCAAACACATTTAGTCAATA
CGGTGTTTTGGGATGGGTTTAAGACGAGGAAACTAAGGATTTGCCAACTTAGTTGACATTCTTGGATGAA
TATCAAACAAATTTACGAGATCATGAAAAATAGTTTTGTTTTCGCGTTAAAATCATTAGGTTCAAATTCC
CTACCGAACATAACCTAAACTTCTTGATTCTTTATCTATTTAATTTATTTTGAATGTTTGAGTCAATTCT
TGAAAAGAAAATATTAGTTTCTAAAATAGGAGAATTTGTATGATCATTTGTATGACCATGAATTTGTATC
TATTTAATTTGTATGATCATGAATAGAAAACAAAATTGAAACTATTTACAAAATATAATAAAAAAAAACA
TAAAATTAGACTATAGAGAGTTTAATGGATTTTCCATACTTTGTGAATAGTTTTAAAATTTTGTTATTTT
TGAAAATGCCCAAAACTTATTTTTATTTTTAAAATTTGGTTACAAATTCAATTCTTTTACTTAGTAAAGA
TGCAAATTTCTATAAACAAAACATTGAGAGAGCATGTTTTGACATTTTCAAGAAAAAAAAAAATAAAATG
GTTGCAAAACGAAGTCTGAAATATAAAGTGATATAGAGTTCGACTTTTTAAAATTAAGTATTTTTTTTTT
TGAACAGGATACTGGAGATGTGAGATCTATAGATGCACTAAAATATATCTCAACTAGGTTAACAAATCCT
TAATTAACATCCTCAGCACATCCCGATACAATAAAGATATATATTTTTAAAATTAAGCTTATAAACATGC
CCACGATCTATTTTAATTTACAATTTCATCTTAATTTTGACCATTGACATGTATTGCATGTTAATTATTA
TTGCAGATGTCAACTACGAGGAGAGATTAAAAAGTGTCATAAAAAGCATTTACGTTCCTAGAGATGAATC
AATCTTTGGCAAGAAGATATCCGAGATATTTGCTTATCAATTGAAATCAATTGCCCAATCTCTTCAACAT
AAATTTGGAGTGCTTTTTAATAGATACCCCAAAGAATTTGGGTCCTTTAAAGATGTACTTAAACTCTACA
ATGGTGGATTTCCTTTGCCAACACATTTTCTTTCAACACTTGGGAGCTTTGTTAAAGAACCATTTCTAAA
GGAACTTCTTAGAACTGATGGTGAACAGTTGCTCAAATTCCCATTGCCCCAGCTGATTCAAGGTACGTAA
TGTTGAGGATTGTGCATTGAAAAGATCGTGAACAGTGAGACTTTATAAAACTTGAGTTATTTGTATTTTA
TATATGATATCAGAGCCTATGAAATCGAAACGAATATGGATCCCACTATGAAAAAGCTGATTAAACCTCG
TAATATTGGGCTTTTACATGAAAAATATAGTGTAATATAAAGGGACCGGTCAATAATAGACCATCTTAAC
AAATGTCTCGAACACTCTTCTTATTTTTTTTTTGAAAAATGATATTAGGGTCCAAGGGTGATATTGAACA
TTTCCACAAGATGGATATTCTCAAAATACTCTCATCCATTGTTAGAATATATTTATTAAGCACGAAGTAC
ATGAGTTTAGGGCTAGAGATAAGTCCAGAAGCATTTACATATTACAAAAAGTTGGTTGCAATATTGGATA
CTCTTTTTTTTACCATTTAATTTTTGAGATAGGATATGAAGTACTGTCTCAAAAACCAATGGACACAAAG
AGAAGTAACCTATCTATATATATCTTATATATGTAAGGTCATTCCTTGATTTTTTTTAGTGTGAGATCTT
CAACAAATGAAATCTATATGGGTAAGTCTTTTGACTTGCAATCCGAATACGCAAAATGATAAAATAAAGG
AGTACAATACAGAAAAATTGCACCAAGGACACATCTTGAGACTTGAATATGAAGTTTCTTACCATTGCAT
AGATGGATTTATACATCAAAAAGGATTATTGTTCTTAAAAAAAAAAAAGATTATTGTGTAGGCTGAATTT
TTGGTTATGATTAATTGAAAAATTAAGTTTACTAAACTCATTTGTTATGGGTAAATTAAACAGATAATTA
TTCAGGATGGAAGACCGATGAAGAGTTCGCTAGAGAAATGTTGGCTGGAGCAAACCCAGCCGTCATTTGT
GGTCTCCAAGTAATTTTCTTTCAAATCAAAATGGTTCAAAATATTTATAAATACGATTTTTTTTTTTAAT
CTTTGATAGACTTATCATAGATAAAAGAAAAAATAAAATTTTACTGTGTTTGCAAATAAGTCTAACTAAT
TATTTTGTTATATTCTTTATTATTATTACTGTTTTTTTCTTCTCTAAATTTAACTATAGGAATTCCCACC
ATCAAGCAAGTTGGACCCTAACATTTATGGAGATCAAAAGAGTAAGATAACCGAAGAACACATCATGAAT
AATTTGGATGGATTCACCGTAAATGAGGTAAAATTTTTAACTATAATATAGTTATCCATTTATAATTTTA

GAAGTTATATCAATTGAAGGACGAATACTTTTAAGTATTTAAATTTATGCAATTTTTTAAATTTTATATG
AAAAAATGATCTGTAAAACTTATGATTAATTTGCGTATGAATTAAACGTCTAAAGTTATAAAAATGAATC
AATTTAGACTCATAGTAATAAATGAAATTTGAGAATCGTTAGTCATATATCTAATCTAATAATTTATTTT
GCAAAACCAGATTTTGAAAGAGCCTATACACATTTGAGACCAAATGCATGACTGGTTTTTAAATAATCGT
AATAAAATGTTTAAATTGATATAAACCTCAAAATTTAAAAGGGTAAAAGCTAATATTTTCTCCTAACCAC
TAAAGACGGTGGACATGTCAACTACTGCTAAACTATACTAATTTCGTTTTTCTTTTTAATTATGTGATAA
TAGGCAATGAAGCAAAACAAACTCTATATATTGGATCACCATGATTATGTGATTCCATTTCTTAGAAGAA
TAAACACAACAAGTTCTACCAAGATTTATGCAACAAGAACAATTCTATTCCTAAAGAAAGATGGGACATT
GAAGCCTCTGGCGATTGAACTAAGTTTGCCACACCCTCAACATAACAAGTATGGTGTCATTAGTAGAGTG
CTGTTACCAGCTACAAAAGGAGTTGCTGCCTCACTCTGGCAACTCGCTAAGGCTTATGTTGCTATCAATG
ATAATGGTCATCACCAACTTATTAGTCATTGGTAAATTTCTTAATTAATTAAAAAGATAAGTTTGGATAT
ATAATATGTAAAATAAAAGACCAAAATGTTTAAAAAATATAGTAAAATTTAATGATCTATCAATAATAGA
TATAATAAAAGATGGACATTGATAACTAGATTTTTATTAGTGTTTATCATTGACGACATTGATAAACATT
GATATATAAACTTCTATATCATTGTTTGTGAGCAACATCTATCATTTTACTATATCTATTATTTATAGAT
TCTATTTTTTTTATTTTATTTTGTAAACGTTTTTTTCATTTGAAATAATTGTAGAGGTTGTGTTAAAAAA
TGGTTTAATATATTAATTATTTATAAAATATATATTTTCTCTTTTAACATTAGGAAAACTTGATATTGGA
AAACAACAATACTCCTTGAAATTAAATTAGCATGGACTAATCAATTGTAAGATCTAATCAACCATCATGC
ATGCATATATATCATGTTAGTACTAAAGTTTTTTTTTTAAAGGAAACAGCTAGATAGGACTTAGGGATGA
GCCTAAGACCACTCTAGACACACAGATTTATTAATGAAGGGCTAGAGAAAAGCCCAAGAGTTAATTACAG
AAGTTGCTAAGGTTTAAAGCAATTGTAGTAGCCGAGTAATTTTTAAAGTAAGAGTCCCTACTGCACCAAT
TGCCTATAAGGATTTTGCAATCCTCCCACATATTGGCAGTTGTTTTATGGGAGCTAGGGGATCCAAAAAT
TCTAATATTTCTTTCACACCAAATTTCCCAAAAAATGGCTATTATTCCACAAAACACGACCTTGCGATTC
GGGCTGAAGCAATTTGGGGACCGAAAGAAGGAAAACACATCCTCCAGATCGTCGATGTTGGGAATCAATC
TGGAAGCATTCTGGAGAAAGGACCATAGAGGCTTCACAGCTTCGCAATGAAGAAAGAGGTGAGTTCCTGA
TTCACTATCCTTCTTGCAAAGAACACACCAGTTGGGTTGAAGGGAAATATTGGGCATTCTTTGTTGAATA
ACCTCCATCGTGTTTATTCTTCTTTGTATCAGGCACCACATAAAAAATTTGATTTTCATTGGAATTGTGG
ACTTCCAAATAATCTCAAGAATTTTTGATTGAGGGTCCTCTGAAGATGGATTCAGCTGCCGAGAGATCAA
GACTTTAGCAGATGCAATGGAAAAGGAATGTTTGCTGTCTGGGATCCAGGTAGGCTTGCTTGAACCTCTA
TTATGTCTCGGAGAAGGAAGAATCTCTAAAATTTTTGCCCATGTACTCCTCTCTCTGTCGTTCAGCTCTC
TTCTAAATCTTATGTTCCATTGGCTGTCGAGCGTGTTCCAAGCAACTTTAACTGTGATTTCTTTATCGAG
TGAAAGAGCAAAAAGTCTCGGATAAGCAGTTGAGAGACAACCTTCTAGAGACCAATTAGAGTACCAGAAG
GAGATCTGATCTCCATTATTTAGATCCCAACTTTGGTTGCTCCTGAACCAGACAATATTGCCTATTATAG
ATCTCCATGGGGCTTTAGGAGTGCTAGAAGAGATGTTTGATGGAATATCTCCTGGGTATTTTCCTTTATA
TTTGCATTGAATCAGATTCCTCCAAAGAGCTGTAGGTTCCGAGAGATAACGCCAGAGCCACTTAGATAAG
AGGGCTTTATTTGTCACATGAAGCCTTGAGATACCCAGACCACCCTCCTCTTTAGATTTCGTGACTTTAG
TCCAGTTGATTAAGTGAGAACCTTCTGATCTGTTATTACCTTTCCATAGGAAATTTCTCCAGAATTTTTC
AATGTTTTTACACGTCAAGGAAGGAGCTTGGAAAACAGATAGTTGGTATATAGGAAGGCTGCTAAGGGTT
GACTTTATTAAAGTAAGTCTTCCTCCCTTGGAAATTTGAGCATATTTCCAATTATTGAGCTTTTTTTGGA
TCTTTCCTTCAACATTGCTCCAAAAAAGTCTGGATTTTGGGTTGCCACCGAGAGGAACTCCCAAGTAGGA
GAGGGGGAGAGAATGGCAGGATATACCCCAAAATGAAGCACATTCCTTAGCTCTAGATTCAGACACATTC
ATTGGCACCAGAGCTGATTTCGATAAGTTTATTTTAAGGCCCGAAGCTCTTTCAAACAGAGATAAAGCCA
TTCGAAGGTTATTCAGGAAAACATCATTATCTTCTATGAAAAGAAGAATGTCGTCAGCAAAGAGGATATG
GGATATGTTACAATTATTACTGAACGAGACCCCTTTAATTGCACCAGAAGATTCCAGATGAGATAAAAGA
CGACTAAGGTAGTCCATGGCAATAACAAACAGGAAAGGAGAAAGAGGATCACCTTGTCTAAGACCTCTGT
TGGCTTTAATACGACCTTGGGGTCTTCCATTGACAATGACTGAGTATGTGACATTGCTTATGCATCCTCT
TATCCATTTTCTCCATAGGATAGGATAATTCTTTTTCTCTAGGACAAAATCGATGAAATCCCAATTTAAA
TTGTCAAAAGCCTTTTCAATGTCGAGCTTCAAAATAAAACCCTTAATCTTCTTCACCTTCCAGAAGTCCA
CAGCTTCATTTGCCATTAGGATAGCATCAGTAATTTGTCGGTTCTTGACAAAAGCCAGCTGGTTTCCTGA
GATGGTATCAGGAAGGGAGGTCTTTAACCTGTTTGAAAGGGTTTTAGCAATGATCTTATAAATGGATGTT
GTTAGGCTGATCGGTCTGAAGTCTTTAGGATTAGAATAATCCTTCTTTTTTGGGATCAGAGCGATGAATG
TGTTGTTCACATTCTTGTTGATAACACATTTGTCATAAAAGTCCTTGAAAATGTCTATGATATCTTCTTT
TAGAAGATGCCAAAAGGATTTGTAGAAGGAGATAGGGAAACCATCTGGACCAGGGGTCTTCTTACCATCT
AAAGAGTTTATGACTCCTTTAATCTCATCTTCCAGAAAAGGGACACAAAGGTTTAACCCTTCAGAATGCT
CAATCGGATTCCAATCTAGATTGTCTATAAAAACAGAATCGCTTTTTGTGGGGCCTTCATAAATCCTTGA
AAATAATTTTATAAAAGCTGATGAAATACCGTTGTTTGTATTCTGAATCAAGCCTTCTTCATCCTGGATT
TCTTGAATAAAATTTCTCTTTTGTCTTGCTGAGCAAATTCTATGAAAGAAGGAGGAGTTTTCATCTCCCT
CCCTAAGCCAAAGCTTTTTGGCCCTTTGGTACCAGAATTGGGACTCCTTAAGAGATAGCTCACTGAGATC
AGCTTTAAGAGCTAGGCGACGATCACTTTCTTCTTGAGACAAAGGGGTATCCAATTCTTTCTTGTCGATA
GAGTCCACTTCCCTGACAATACTATCTTTAGCATAGACAAAGGAGTTTAACTTCTCCTTTTGCCAAGGTT
TGATAAACTTAGCTAAGGATTTTAGCCTTTGTATGAAGGAGAATCCAGGGTGACCATCTTGGATCGAATT

ATCCCACCATCTTCCCAAATTTCTTTTGAACTCTGGATCATTGAGGGCTATGGAGTTTAAGCGGAAAGGG
ACTGGACCCCAACTGAGTTTGGGATTGGAATCTTCACAAACCAGAGGAAAATGGTCTGAAGTTGATCTTG
GGAGGGTCCTTGTTGTGTGTGGACTGAAGAGATTTTTCCAAGAAGAATTGTAAAGGAATCTATCGATTCG
AGAAAAAGTAGGAGGATTCCGAAGATTAGACCAAGTGAATCTATTGTTTATGAGAGGGGGATCAAGCAGA
AGATTATTGTTGATGAAACTGTTCAACATTTTTGAGCTGTAGGTAGAGTTGGAAGTAGATGTTGATTCCT
CTCTCAATCTGATAACATTAAGATCACCTCCTAAAATCCACGGAAATGAATTAAGATGTTGAAGATTATG
AAGATTAGTCCAAAAACGAGCTCTTTCCCTCCTTTTAACTGGACCATAAAGACCTGTTAGCCACCAGGAC
AGATTGTTGTTGGACAAGAAGTTGGCAGATAGGCTAAAAAGCCCTTCCTCGTGACTTAAAATAGAGTGTA
TCTGAGCATCCCAAAGAATTAAAATCCCACCAGAACTATCCAAAGCATTTTTAGCGATCCAATTGATACT
ATTAGAGGGCCATAAGGATTTAATGATTCTCTTGTTTGTGATCTTAAGCCTAGTTTCAGTCAGAATCACA
AAGTCAGGGGAGTATGAAATTATAGTATTTTTTATTATGGCTCTTTTAGAAGGGGAGCCTAAACCTCTTG
CATTCCAAGTAAGCAGTTTCATTTTACAATGGATGTCCCCAAATCCCCTTTCCCCTTTGAAACTATCCCC
GAACTCATATGGTTTATCAAAACATTTGTTGAAGTAGTTGCCCTTGAAGAGTCATTGACCGCAGAGAGTT
TCAGCCCATTATCCTTTAACCAGAAAGCAAGTTGTTTTTTGAAGGCCTCTGAATCCGAATCTTTCTCTTT
TTCTTCTTTTTTCCTGTAGTAGTATTTTCTTTTATGAGCATGTTTTGGTTTTCTGTAGTTGACTTCAGAG
GATGAATTTGAATTCTCGTTAACTTGCATTTTCATTTCAGGTGTCTCGGGGACCACTTCGGTGTTTGTTA
TGTCAATAACTTCTGCATTATCAGAGCTATGATGGTCTTCAAGTGATTTGTTTGGATCCAAGATTGGCAG
ATCTCCCAGGTCAACCGTGAGGAAAAGACCTTTTTTAGAAGCTTCCTCTTCATGTGCCATCATTTGAATT
GGTTGAATATTTAATTCTCCTTTCTTCTGATTGGCTTTTGATTTCGGCTGAGTGGAGGGCAATCTCTTCT
TGATACTTCTCTCTCTACTTACTTTCTGTTTTTTTTCAGGAGAACTTAATGAAGGAGAATGATTGGCTGG
CGCAGAATCCGGATTGAAGATGTTGGTTTTATTACAGGGAGAATAGAAGGAGACTTTCCTTTTAGATTTA
TCCAAATTTAAAGCTGAAATTGGTGGAAGCTGAATGTCAACCTTCTGTTTTCCTTTATCCAACACGCCAT
CATTGGATATCCCAGGTAATATCTCTAATCCGGATTTATTAGCCGTTGCATGCGCATTACTAACTTTAAC
TGCCTCTTCATTTAATAAGCTCGGCGACGTGGCAATTTTGTCAGGTTTAATGATAACCGATTTTAATGCA
GATGGTTGGTCCGGGGTACTACTTTTGCGGCCGTCGGAGCTGGTTGCAAGAAAATCCGGCGATATGGCCT
CCATTCCTTCGAAGAAGAACTGTTCTGATTCAGGATTGAATTCATCAAAAGCAGCAGCAGCTTGTCTCTT
GAAGGTACCGTGAAGTCTTACATTCCTTTCTATTAGCCATTTGCCTTCTGGATGTGTAACTACTTGGATG
GAGAATTTGTTTCCTTCATTATCAAAGATCCTTATATTTGCTGGTAAAAAGCCTGAATAGTTGTATCTGA
TCTTTATCTTTGCTTCTATCAGGTTTTTTGCTGATCTGGTTTCCTCAGCCACTTTAATCAAGCCTCCGCA
AGCTTTTCCAATCTGCTGAAAAGTCTTCATATTCCACAGATGCAGCGGAATTCCTCGGAAAGTCGTCCAT
CCTCCATAGCTCGGGATGAGTTTTGGGGTGGCATGATGTATGGAGCACCATTTTTCAAATCTTACCGAGT
ACTTCCCTACTGTGGTCCATCCTTTGTTTTGACATAGAAGGTTTGCAGGTATGCTTGAACTAAAATGAAC
CAAAGCTTTATCGGCGTGAAAAGCGTTATACGTGAAGGATTCCTCTGTTTGTTTCCTCAGGTTTTGAAGT
ATTTTGTACCAGTCGTCATGAAAGAACCTTCTGATTATCACCACTGTATTTTCAAGAAGATCAAATGAGG
GAGAGACACAAAGACTATTACCTGATGAATGGCTTGAATCACTTGAGTAAGAGTCGCTTGAATCACTTGA
AGCAGAAGATCTTCCTTCAATGACTGCTCTTGCGTATGATCTCTTGTGGTAATCAATGGGAGGGGAAAGG
CGACCATCGGGACTGGACCTTGGCAAAAAAGCTGGCCTTGTTTTTGCTTTCACTTCTACTTTTGGTGTAA
TCATGGACAGAAAGGAGACCCAGCCACTTTTCTCAGGTCCTTCCGGAACTAGAATGCATGATTTTCTGTT
CTTTTGATCAACTCTAAAGATTTCTGCCGTACATCCTTTATTGTTTCTTGTTTTCCTGATCCAGATACAT
TGTTCAGAGTCACGGGTTTCAAGAAAGAATCGGTTTGTACTTGGGGTTGCAATCAAGGATTTCAGGGTAC
TTCTTATCCAGTCCAAGTCTTTTGGGGAAGCTTCAATGGAGAAAGCCTTGTGTGCTCCAGTTTCGGTTAA
CCAATAGTGAGTGTGCTTCGAATATTTATCAAGGTGAAGGACAAATTCCTTCCTTTCGATTTTGCATGAT
CTAGGAAGTGATTTGAAGTAGGCCATTGTTTGGGAAAATGTAGGAAAAAGACAAGGAAGGAGGGTGGAAA
GCAGTGGTAGGGACTGAAGAAGAAAGCGACCTTTTAACAGCAGGAAAAAGGCTGAGCTTGTAGTTAATAC
AGTGGGTGAATTTAGTTGAAATTAGAATGATGGGCGGAGCCGCCATTTTGTGTAGCTGTTGCAAGTGACT
CTGTATCTTCCGGCAAGGGGAATAACTGCTTGTAGGGGATTTGGAGAGAGGAGAGAGGACAGAGCATTTT
TAATTCTCTTCCAAGTTAACATGTTAGTACTAAAGTTTAAAACATCGATATGATAAAAATGTTGATGTCA
ATAAATATTTTTGAAGAATTATAAAAAGGTTCGAAAAAAATGTTCATTTTAAAACATGTTTCTAATCTAT
ATCAAGTTTATGTTTCATTATTTTTAATTTACAAGGTAACTATAAAAATGTTTATTCATTTCTATATTTA
TGCCAAAAAAAAATTGTACAAATATCAATGTAAATATATGGAAAAAAGTATATATATATATATATATATA
TATATATATATATATAGACATGTATGTATATATATTATTTTTTGAATGGAGTATATACAGAATTGGGTTT
AACAATTTAATTGTGTTGCAGTTGACAATTCAAGAATGTAATTGAAATCACTTTCTAAATTTAAGAAATT
AATTAAGAAACATAAGTACTAAAAAAGTTATACCCTAGGTGTACAATAATGTTATATATATTAATTTTTC
TAAAAAAAGATCCTACATTTTTTAGTAAAAAAAATACTAACATTTGGTGATATATTATTCCCAGAATAAT
TTATGTTGCATTTGAAGGACAATGATTGTATATATAAGAAAATCAATAAATAATAGACTCTAGGTCGATT
GACTTCTATTAGCGATATGATCTAAAATCGAAGATTTGAATTTAAATTTGTTACTATATTTACAAATTAT
TGTGTTGTGTTATATTTGACGTCTGTTGTATATATTTATTTGTAGGCTGAATACACATGCAGTAATTGAG
CCATTTGTGATAGCAACAAGCAGACAACTTAGTGTTCTTCATCCAATTCACAAATTACTTATTCCTCATT
TCAAGGACACAATGAAAATCAATGCTATTGCTAGGCAATCTCTCATTAACGTTGATGGCATTATTGAGAG
AACTCATTATCCAACTAAATATTCTATGGAGATGTCTTCTTTTGCTTACAAGAATTGGGTCTTCCCTGAA

CAAGCTCTTCCTACTGATCTAATTAAGAGGTAATTGATCAAATCAAACTACTATGCATCCCAATTTTCTT
TTAATTTAATTATACTTTTTTTGGTAATTGGGTGAGCTAAGGCTCAATTATCATTATGCTTAGGGTTCTT
TTCAATGTGTTCTTTTCAAAAAAACAGAATTTTTTTTTTATCATTATGCTTCTTCCTTCAATTGATGGAA
AGATGCTCACTATTAGGATCTTCAGTTAATAGGAATGTATGGATTTCATAACCAACCATTAATTAGAGTT
TTATAAACTTAAATTTTTAAAATTGATTGAAGATCAATGAGATGTGATGAGAGAGCTAAAGTCACATTAA
CCTAGTTAGTTGAAATAACCAGATGTACTTTTTGATTCGATCTCTCTTAGGACTTGTTAAAGAAAAAAAA
TCGAAGATCAAATGCTTACGCCCTATACAATTCCTTAACAAACTACTTTAATTTTTCTTTCCAAAGCTTA
ACTGGCATTTGATCATGCGAAAATAATCCAATATTTAACACCTTCATTAACCTTGAATTTGATATACGGA
GGTTTCGAATGATCAAATATAAGGATTTATATGAGTGTTCTATATAAACTCGATCTCCAACTCTAAGGTG
CATATGATCTATATTATGGATTTTCTCTTTGTTAATAAAGTCATTCTCTCTTTGCTCGTGGATGTAGCTA
ACATATTGTTAGTGAACTGCATAAATTTGTCTATTGATTGTTCTCGTCAGTGAACCACATAAACATGTGT
CTATTTTCTATTGCTTTATGTTTTATTGTTTTCTTTTTTGTCGATTTCATAACCATTAGGTTGTGGTTAA
TTACTATAATTTGTTTAGAGGAGTTGCAATTGAGGATGCAAATTCTCCGCATGGACTCAAACTACTAATA
GAGGATTATCCCTATGCTAGTGATGGACTTGAGATTTGGAGTGCAATCAAAACATGGGTAGTAGACTATT
GCTCTTTCTACTATAAGGATGACAAAATGGTTTCTAATGACTCAGAGCTCCAATCATGGTGGAAAGAACT
TAGAGAAAAAGGCCATGAAGACAAGAAAAATGAACATTGGTGGCCAAAAATGCAATCACTCCAAGACCTT
ATAGATTCTTGTACAATCATTATATGGATTTCTTCGGCTCTTCATGCTTCAGTTAACTTTGGACAATATC
ATTATGGTGGCTTTTTCCCTAACCGACCCTTAACGAGCACTAGATTCTTACCAGAAGAAGGAAGTATCGA
ATACGAAGAACTCAGATCAGACCCTGAAAAGGCTTTCATGAAAACAATTCAAGTTGATCTTTCAAGTGTT
GCTTTAACTGAAATCTCTTCAAGACATTCTTCTGATGAGGTCTATTTAGGGGAAAGAAACAGCAAGGAAT
GGACTTTGGATGAACAGCCTTTGCAATCATTTGAGAAGTTCAAGAAAAAATTAGTAGAAATCGAGAACAT
GTTTATGAAGAGAAACCAAGATCCTAAGTTGAAAAATAGAGTTGGGCCTGTGAATTTACCTTATACTCTT
ATGTTTCCAACGAGCACTGAAGGTCTTACAGGCAGAGGGATTCCTAATAGTATTTCTATGTGAATATGGC
AAAATATGTTTGAATTATTGCCTAAGGTTGTTGTTTATCTATGTATAATTATTAATGTTTCAATGAATTT
TCTTTTTCAAAATATGTAATTTATTGACTAAGGTTGTTGTTTTTCTTTTCTATGAAAATTCTCTGGTTGT
TTGTTTTTTAATTTATTCAAAATTTAACATTTGTTAAATTAATTCTCAGCCATTTTTTTTCTCTTCAAAA
TTTTCTAGTAATACACTTTTTGTTTACACAAAACATCATAGAATCTCTTACTAAAAACAACAATAATATC
TAGGAAAAAATGTCGTTTTAAATAGTAAAACTGCTGAAAATATTGTCAATCAATTATAGATAGTGATAGA
CTACTCTCATCTATTAGTGTTTACCAGTGTGGCAGAAGATAATAGTCTACCAACATTTATCAATAATAGA
GAAAGTGACATTTTACTACTATATTTATAAATATTTTAGTATATTTTTAATATTTGAAAAGAACCCATAT
AGTTGAGAAAAAGTTCATTACTGCATTATACAGAAATTAAGTTCTATCTCTTCTTCCAGTATCATGTAGA
AAAAAAAACTTTCTATCCTTAGATATCCTTGACCAAAGCCAATGATGGAACTCATTAAACTACTATCATG
AGCTAGCCCAAACACAGAACCTAGCTGTAAGCCAAAAAATCAGACAAGTTCACAGTCCAAAATCATCATA
GAAAGTCACATAGCCCAAGTTCATCATGTTAGAAGAGAAATGGTGTGAATGAAACAAGTGCAGAGAAAGT
GATATTTAGATTCAACTAAACACGCCTTTCTATAGACTTACAACGTGAAACAATAGGATTTTGATGGTTA
AGAAACCAAAAGTAATACTTAATTTCTCATGACTTCTAAAAACTCCCAAATGGAGTACACAATTTGTTTT
AAAAAAACCAAAGGTTACGAATCAATTTAATTCCTTAAAACCTTTAAATTCTATTAATAATATGAAACCT
TTTATATTCTCAACACATTAAAACTCTAATAACACCATTTGACATACGTAAATTAAATCAAGTTTGGACA
ACGAAAATAAGATATCAGAGAAAAAGGGCCATTAAACCTCTTTATTTACTCTTAGTTAACATGTGTGAAA
TGTAATGCACAATTGTCTCCTATCAATTTTGGACATGAAATCATCTTATAATTTCGTATTCAGATTTTTT
TTACCTTATTCAGACTCCATGCTGGTCGTTTATATAATGCTGGATAATAGATTAATAGCATTCCAACGGA
TAAAAAAAAATGTCCATGAGATTAGGTTCTTGGTGGGTGATGTTGACATGACTTCATTTAGGTCGCAAGGC
TTGGTCTTTGACCCTCGAAAAGTGGGTTGAGTTGGGTAGGTAAAAAAATCCACTTATCCTTTGGTTCACT
AATAATTGAAGAGGTTTATTATTTAGCTAACTTGTTCGTGTTAACTTCGTTGTTTTCATATCAACTAGTC
TAATTACCCTCTCATAAAATAATTATCAACTCAACTAAACTCTCCAAATTGGATGAGTTAGATACTATGC
AAAGACCCCACTATATGCCTTGCTTTTTTATTAACATCGATTGGATTTTTATGGAAGAATTGATTGTGAG
AGTTTCAACCAATTTGGGCTTTAGCATATTTTTTCTTGGGATAGGAACACTCCTAAATATGGGGTGATAC
CCTAGTGGAAGAAGATTAAGAAGATGAAAAACATATTAGGAAAGAGAAAGAAGAAGTGGTTTGCCCAATG
GGGAGCATGTGAGAATGTTGAGATTTCAATTTTATTGGAATGGTAGATAGAAATATTGAGAAAATGTCAA
TGATCAACTGATAAAATTTTAGGAAAGGGTTATAAAACACAAAAATTTTGAAAAATATAATCCATAAAAT
AATATTTTAAACTTTTTTGTTATTTTTATTATATTTATGAGTGATATTTTGTTGATTATTTTTTATATTT
CATGAATTTATAAGAAACGACGATCCACGTCATTTATTGTCAACGTTGAATTCATAGTTATATATATTTT
TTTAAAAGGATACGATGGGTCATTTGAGTGCACTCGGACGTCTCCACTAGATGCACACCCCTTTAGCACC
CTCATAACTCCCGCTTCATTAATATTAATAAAATTCAATACAGGATGCAAGGAGAAGGGCTAGAGATAAC
CCCTATTTTACAAAAGCAGAAACATTTAACGCTATGGAACTACAGTTGTAATTTGAAAAAAGTTTAGATC
TACTGCTCCATAAACCAATTAGTGCAAGGATATCTTCTCATAGCTCCGCTGCTTCTTTTCTCAAGTGTTT
AAAAATCCCGCAGTTTCTTTCAAGCCAGATTTTCCATAATATTACAGCAATAGTATTGAAAGTAATGGAT
CCTCTTTGGCTCTTAATGTTAATACAACAAATATCCTTGCAAATGGAGGCCACGTCGGTAAGGGGGTTGT
TCCATTTCAGAAGAGATTTCGCTTTGTTCCAAATAGACTGCGCATAAGAGCAATTTATAAATTTATAAAG
AGATGGTTGATGTCCTCTTGATTTTTGTTGCATAAATAGCACCAGTTTGGGTTAAGATACTAGTTTGGAA

GTCGCTTTTGCAGCTGCTCGGTGATATATTTGGATATCCCAATCATTTGTGATTGGATTCCAAAAATCTT
TGACGCTTCCATTTTTATTTCTGGATAGGGCATACAGGTGAGGGGTGTATAATGATAGGGGAGCTTTGCT
GTTCCAATTATCAAGCCAAAAGGAAATCGAATCCCCATCATTCACCTTCCAGCTGATGTAGTTATTAAAC
CAGTTAGCACAACTTGTTATAGCTTTCCATGGGGTCTTGTTGTTGCTAAATTTTCCTCTGATAGGTATTT
TACCAACAATATCCTGATCATATTTGGCTATAATCAGCCTTTTCTAGAGAGGATCTTTTTCAGATAAGAA
TTTCCAGAGCCATTTGCATAAGAGAGCGAAATTAGTATTGTGGACTGTATTAATTCCTAGGCCTCCCTTT
TCCTTTGGTAACGTAATTTTAGACCATCTGATTAGGCTAATATTATGCCCACTGGATGAGCCTTTCCACA
AGAAATCCCTCCAAATCGATTCAATTTTTTTAGCTATGCCCTTGGGAACTTTGAACACCGATAGTTGATA
AGTGGGGAGACTTTCTAAGGTGGAATTAATCAAAGTGATTCTCCCACCCTTTTGATAGGTGAGAATATTC
CCAGCTGCTTAATTTTTTTTGGATCTTCTGGAGGATATTTTCCCAAAAATTTAGAGATGATGGTTTCCCA
CCCAGAGGCATTCTAAGGTAATTAATCGGCAAAAAATCCTGCCTTAACCCCCAGCTTTTCGCAACCGAGT
TCGCTCGACTAGCAGTCAAATTTATAGGGGATATAGTGGATTTGTTTATATTGATATTCAACTCTGAAGC
GGATTCAAAGAGATAAAGGGCCATCTTGAGGTTTGTAATGTACTCATCGTTATCCTCAACAAAAATGAGA
ATGTCGTCTGCAAAGAGAATGTGGGTAAGGTTAAGGTCGTTTGGGCCTAAGTTGACATCTCCAATTTTCC
CCTTTCTCTCCAGATTAGTGATAAGTCTATTGAGGTAATTCATGACCAAAACAAAAATAAAAGGAGAAAG
GAGATCTCCTTGTTGAATGCAGAGTATTGAACGCTAGATATACAATTAGCAACCGTTTTCCTCCAATCTG
AAGAGTAGTTTTTTTTTTCATCAACATAAAATCTATGAATTGCCAGCTGATTTTGTCAAAGGCTTTTTCA
ATATCCAGCTTGATTACGTAACCTTTGGTTTTCTTTGGCTCTCCAGAAATCAATTGCTTCATTTACTATC
AAGATGGCCTCTGTAATTTGTCTTCCTTTGACAAAAGCCATTTGGAACTCGGATATAGTGTCAGACAATG
TAGGTTTCAGTCTTTCGGCCATTACCTTGGCGATCAATTTATATAAAGCAGTAGTGCGGGTAATTGGTCA
AAAATCTGATGCTATCTCACCCTTTTCTTTTTTAGCTATGAGGGCTATGTGTGTTTCATTAACCGCTTTA
TTGATAATTTTGCTGGAGTGGAAATCCTTGAAAATATCCATAATGTTTTGCTTCATAAATTTCCAAGGTT
TTTTCAAGAATTCCATGGTGAACCCATCCGGTCCAGGGGGCTTTGTTTTTAACAAAGGATTTCAGCGAGT
ACCACACTTCTGTTTCATCAAAAGGTCTGTCAAGCATAAAGCTACTGTGATTTGTATCATATACCAATCA
AGATTGTCAATGAAAATTTGATTTTTCCTCTCATTAGAATATGTCTTTTTGAAATGATGTACAAAAGTCT
CTGCTATATCACTGTCTTTTGTACATATCTACCCAAGAGAGTTGATCACATTTGATATTATACTTCTTCG
TTGTCTGGCTGAACATATTTTATGAAAAAAAGAAGTATTTTCATCCCCTTCATGGTTCCAGATTCTTTTA
CATTTTTGAGCTAATATCTGTGTTTCTGTGAAAGTAGCTTGGGAGAGATAAGCTTTTAGAGACATTCTTT
TGTTGCGATGCAATTCGGTTAAGTGTCCTTCGGCCTCTAATTTATCAATCAGATCGATCTCTTTAATCCA
AACCTTCTTGTTCTCCTCAACCTGTCCCTTTTTAATTTAGCCAATTGCTTGAGTCGACGCATGAAGGAAT
ATCCAGCATACCCCTGTTGGCTTGTGTTGTTCCACCACAATTCCACATTTTTCTTAAAATCCACATCATT
TTGATAAGCATTGGTGAATCTGAAGGGGGAGGGCCCCAGTTGAAAGAGGAAGATTCAAGAGCAATGGGAA
AATGGTCAGATGTAATTATGGATAGAGTTTTTGTATAATGGCACGACAAAGAGTTTTCCCATTCAGGTGA
GTATAGAAATCTGTCTAATATGGATAATGTGGCCTGTGCTCTTAGGTTGGACCAAGTGAATTTTGCATTG
GTGAGGGGAGGGTCAATTAAATTGCAACTGTTTATGAAGAAGTTGAATTTCTTCATACTGAATGTAGCTG
GATTTTTGGCAGAAGTTTCCCATTCCATCTATAACCACATTAAAGTCTCCCCCAAGAATCCAATTAGGAA
GGCATGTGGCTTTGAGGTTGTCAAGTTCCTCCCAAAACATTGACCGATTTTTTCTTTTTACTGGTCCATA
AATGGCGAAAAGCCACAAGCTAGTACCATTTTGATAGCAAACTTTGGCCAAAATGGAAAATTTCCCTTCA
ATAACATTTTGAATTGAGTGTTTAAGGTCATCCCACATTACAATGATTCCCTCTGATCGGCCATTAGATT
TAAGAAAAAATCCATTTAATGCTAATTGAGTTCCAGAGGGATTTTATGATTTTTCTTGAAACCATAGAGAG
TTTGGTTTCAATTAAGATAACAAAGTCGGGGCAGTAGGAAAATATAATTGTTTTTATTTAGGCCGAGCTT
AGCTTTCTAACATTCCAGGATATCATCTTCATGGGTAAACAGTCTGCCCCATGGTTTCTTGCTCTTCACC
ATCTATTACATCATCATTCATTTTTTCAAAGTTTAAATTCGGGGGAACAATTTGTAAAGGGTCTACAACA
TCATCACAAGTAGAAATAAATTCAGAATTAAATTCAGAAGACAATCTAAGATTATTATCTTTCAGGCACT
TGGTCAATTTCCTCTTGAAATTCGCTTCTTCATCATTCTCTTCACCTTGATTCTTCAAGCTCTGTTTTTC
CCATTCTTCTTGGTCACTCATGACCATATTTCCCAAGCTATCCTTCACAATTTCGGACTCAGTTGGTGAT
AAAGATGAAGGTGTGTACGATGATCCCTCAGGGCTAGAAAAAATTTGTATCAGATAGAGGAGATATATGAC
CAAGATCCACGACCAAGTTTATCTCATTAAGTTCTTTCTTTTCCTTCAAATTTTGCCTTTCTGCGTGAGA
AGTTCTCCTTCAGTATCCCAATTCTTCACTCTGTAAACTTTCTTTTTTTTTTTTTTAGAGATTCAGTCCTT
TTAATTTTTACTTCCCGACATTTTTGGCATAACCCATTGGGCTTCCAATCTTTAGAGCTTTTGTAGGGGC
GTTTTGAGTAGAGTAATGAAAAATTTCATTTTTTGGGGAACTAAAAGAGACTTTTCTCTTTGTGTTGATA
GGGGGAAGCTTAAGAGGAAATTCATCATGACAGATTTGTTTCCCCTTTTTTTATCAGTCATGTCAACTGT
CAGCTATTTCTTCACTTTTTCTGATAGGTTACTGTTGTCGCTGTCACCCTCATAATTCAAATTCAAAAAA
CTAACAGCTTTGTTGAATTTTTTATTCATTTTTGTGGGTCCAGTTTTCCGACGAGTGTTCATCTTGTTGT
TTCTGCTTTTTGATGTTTCCGACGAGTCTTTTTGGCTTATAACAGCGAGATTCCTATCAAAGGTATACTG
TTCAGCATATGGATTGTATTCGTTGAATATTTCAGCAGCATTTCTAGTAAAGGAACCATGGATTCCGGGA
TTTCTTTCTCTCAGCCATCTTCCTTCTGGATGTGTTATTGTATGGATAGTGAAATCACTTCCTTCTTCAT
CATAGATTTTGATAAATGCTGGTAAGAACATCGTGTAGTTATCCTTCACCTTAATCAAAGCTTCAGTCAG
CTCTGCTTTGTCTACTGTTTCCGGAGCTGCTTCAATAAACCCACCACATGCTTCCCCAATCTGTACGAAG
GCAGACATATTCCAAATGTGTAACGGTATACCTCTAAAACGAATCCATCCCCCATAGATGGGAATAACTT

TTGGATCGACATGTAAAACTTTCGATCACATCTCAAATTTGACATAGAAGGGGCATACCATGTTCCATCC
TTTATTTTTGCATAGCAACTTTGCTAGATTGTGGTCTTTGATAAAGAGGAGAGCCTTATCTGCATGAAAG
AGCTTTTATCAGAAAGCCGAATCCTTTTGGTTTGTCTGTTCTTTGAGCCTGCCTATGATTTTTTCTCAGT
CGTCGTGGAAACATCGTCTTGACAAAACAACCACTTTATTACAGTCAAAATCATCATTTTTGCCCTCTTT
CTTCACTTCAGATCTTACTGTTGGAGTGAAGTTTTTTAATGAAGTTCTGTTGGTTGCATGAAAATGTTCA
GATTCTAAGCTGGTTGAACTTGAGACGACTTCAGCGTATGATCTTTTGGAGGAATCTGAGTCAGAGGATG
AGGCGTGTTTCTTTGTGCTTTTATCTCGACAAGTGGAGAGTCTCAAAGCTGGCTCTCTTTTTTCTAAGTT
CTTTTTGCAAGTCAACATATCAGAGAAATGGGCTCAGCCAGATTTGTCCATACCTTCCGGGACCAGAATG
CAACATCTTCGACCTCTTTTGTCAACTCTATAAATCTCCGCTATGTACCCTTTTCTATTGTGAATTTTCT
AGACCCATAGACAGAAGTCGACATACCTCTTTTCAATGAAGAATCTTGTGGTTCGTGGGGTGTTCAGTAG
TGCATTAAACATGTTTTTCAGCCATTATTCAGCCATTCAAGAGATTTAGTAATGGCTATGGAAAAGGATT
TGTAGGGGCCACCTCTGTTATCAACAGGTTTGAATCTCTTGATCTTTTATCAGTAGAGAGCACAATTTTT
TTTCTCCACAATACAGTAATGGGGGAGTTGATTCAAGAAAGCCATTTGCCAAGAACAGTAGAGAGAACGA
TAGAGAGAGGGTAGTCTAAAGCAAAAAGAAAATATGTGAAGGGATATGAAGGGTACCTTTGGGCCAAAGA
TTTAATGTGAATCTCCAGTCGCAGCTATTGGTTGTTGGTAATAAATGATTTTGATGTGAAATCATCATAG
TTCAGGTGAAGGGATGAAGCTGTCCTGTGACTTGAAACGACGGTGATGAAGACGAAGGGGCAGTTGGAGG
GAGGACCGCTTGTGGAGAGAGAAGCCATTTTTTTCCCTACCAGAAGAATACAAAGTTATATTTAAATAGC
GATAAAAATGTTTGCATATCGATGAAAATTTAATACATCTTAATCAGATGATATTGACTGTGATAGCATA
GGAAGTATTTTACGTTATTGTTTGGATATTTTATAGGTTATGAGTAATTCATGGATGGAATTTTAGTGTT
GATATCATTTTGGAACTAATTTAACACCAAATTTGGTCCAATACAAAGTCAAACAACATCAACGTTTGAG
GCAAATCGAAGCAAATCGAAGACCAATTGATGCCAATAGACTTTTGCCTTTTAGCGTCGTGGTGCTCCAC
GATGCATGGCACGATGCCAATAGTGCAGTAGGGTGCTTACGTCGATAGTGCAGTAGGGTGCTTTATGTGA
AAATAACTCACTTCTTACACATAATCGTTAAATATAATAACTCACTTCTTACACATGATCGTTAAATATA
ATAACTCACTTCTCGCCCCAAACGCCTTCTAGCAAGTGTTTCATGTTTTTTGGCTAAGTTAATAATAAAG
ATCAACTTATTTATAAAAATGTCACATTATTGTTTTATTTACCCAAATGTCATTAAGCATATTTACTTAA
TTATTAGGTCTTGCTTGGGCTTATCTCTTGCCATTTTTACTTTGTTCTTTCTTTTGCATATTGTATTTCT
TTAATCATTAATGAAGCAGAAATGATGAGGGTGTTAAGGGATGTTCACCTTACCTAATGGAGATCTTTGG
GTACATGCTGACCCACCGTATCTTTTTTTTTTTTTTATTTTAAAATTACCATTAAAAAAACTTTAATATT
TTGTCACAACATCTATGTATATAAAATATGTGAAGTGTATATCATGTATATCAAATGTATATTTGAATTT
TTTTTTGCCACTTGAAAAATGGGAAAAAAAATGTAGAAAAAATTGACAAAAATAACACACTTTTGGTTTTCG
TTTTCAAAAGTAGCACATTTTTTAAAACTCACTGTAATGATGTTTTCTCGATCAATCTCAAGCAAGATCG
ACTACCGGAATTTTAAAAAACTTTTAATATTTTCCAACTTAATGATTATTTTCTCGGTGCATACAATTAG
ACTGAGATTTTGTCATTTTTCAAATATTCCCAAATCTTATTTAATTTTTTTTAACCTTCCCAAATCTTCC
GCAAAATCTTAACTAATATCTACTACTTTTTGCCAATTTTTCCAACTATATTTATTGTAAATTACCATTT
TTTTCTTTTCCCCACTTTTTATCTTTTCAATAGGAACTTGATTCTCTTGTTCAGCACAGCCAAAATCTTC
TCAAGATCTCATGTTTCTTCATAAAGTACTTAATTTAGCCTTAATGTCGCAAGAAAGACATATATATATA
TATATTTACAAATCAAATATGACAATGCATATGAATAATCTTTCCAGTCAGTAAACCAAGTTGATAAATA
GTTGACAATATGAATAACTTTGAGTTGCACATGTATTAATTTGGAACTGTTGAAATTAAATTAATCTAAA
CATAATACATGAATTTACATTATTAAATATGCATGAATAGTTGATATACATGGAATAGTTAATCACTAAA
TACATTGATATATAAAGAGTCACATGTATTGATATTTATTGTTAATGACTTTTAATACACTTTTCTACTA
GTATAAATATGTGTAAGGTTTCTCATTTGTATCTAAGAAAGAAAGTAAGAAATTCAAGTTTAAGAAATAT
TATTCAAGTTCTTTCTTCTCAATTGTGTGAATAAGAGAACAATCTTCTTCTCTTGTTTCTTGATTTGTAT
TGTGAGGGCCTATTACGTTTTCAACAGGAACAAATACCGAAAAAATCAATACAAAATGCTTCTCAGTCAG
TAAACCAAGTTGATAAATGGCTGAAAATATATAAATACTTTGAATCGCATATGTATTTTGGAACAAATAC
CGAAGAAATCAATATAAAATCATTTGTATATATTCCAGCTCTGAATTGATAATCTATTTTTTTGCCGCTA
AACAGAATCACTCCTTTGAGGCTTAGATGGTGTCTTATAATATTGCTCTGTTCTTCTACGCACCGCAAAG
TCTAGAACCTTTTGGGTTGAGGAAGAGAAGGAAAAAATGATAATTTACAATAAATAGTTGGGAAATTGAC
AAACGTAGAGAAAATTAGTTAAGATTTGGTGGAAGATTCGGGAAGATTTGAATATAATTTTTTGAAAAAC
GGTAAAAAAGATTTGGTAAGATTTGAGGAATGGTAGAATTTCGGTCTAATTGGTGAGGTGCATAGGAAAA
ACCCAAAACAATCAATAAGTTGAAAAATATTAAAATTTTAGAAAAATTTCGATGATTGATCTTGGCCGAA
ATTGATCGAGAAAAACAGTTTTACGGAGAGTTTTAAAAAATGTACTATTTTTGAAAACGAAAACAAAAAA
TGTGTTACTTTCGACAATTTTCAAAAAATGTATGACAAGTTTGCAAATCCCCTAAACTCAAATTCCCGCC
ACTTAATTTTTTGTTTTAATTATTTGATTCTCTATGCTTTAGTTCTTTATTATTGTTGTGCACATTACCC
ACATATTGTGAGGTCTTTTTCAATGTGCCCAATTTTAGGTGAACAGTACTTTATTTGGTCGCCTTATTAG
AGTTTCGTGCAGTGCTTATGGTTAGAGTGAAATGATCGTACTTTCAAAAAAGGGTGGAAAGTTTTCTAAT
CACATTTGGGAAGATATTCTTGGTGCTTCAAAAAATTTAGAATTACTTCTATGTCGCTTAAGTGGCATACC
TTTTTGTAATCTATTGGCAACTAAAGAAGTTGAAAGTTAAGAAAGTAAAAGAAAGATAAATAAATAAATA
AAAATTTATTTTAAAATATATAAAATGAACCAAAATATTTATAAAATATAACAAAATATCACCGTTTATT
TACGATAGATCGCGGTAGATTACTATATGTACTTATCTGTATCACCGTAGTTATATGTGGTAATCTAGAT
CTACGTGCACGTTTAATAATCTAAAATAATTTTTCATAAACATAACACTCAATTGTTGTTATAAACAAGA

CTTTAAACTATATTTCAAAGTTCAAAAATTAATAGGAACAATCGAGAGTATTAGAGTCTAACATATTCGT
TTTCTAAAATTCGAAGTCCAAAATGACAATTCTTTCCCAAATTATTATGAGAAGACTTTTAATTTTGATG
TTGTAATTAATGTCATGTAATTCTTTTAAATTAATTAGTAGATATTAGTATTATTGTTGAGCGAGAAATT
TTAGACTAATCACAATTTAGCAATTTATTTATCATATTAACGACGAAAATACAAATAATTGAATGTGAGA
CTAATTAAAAGTTGACATGTGGCCCAAATTGAGAAATTGGAGACATAAATTGATTAATTCCAATGAGGCC
TCGTTTTCATCATGATCGTTGGATCAAGTTAATTATTTTGATCAAATTAAATATTATTGATTTGGGCTAA
AATTCAATTGAGTAAAAAATTTAATTTAATCCAAATATTAATTAAGGCCCTAATCCATATATTGTACTTA
GGGGGCCTGATCTATGGACCAACCAAGTCCAAATCCTTAAAGCCCACTAGAGAGTTTTATAAATAGAGGA
GTTCTTTCATTTGTGGAGAACCCTAGAAAGCCTAGAGATAATATTTTCCCAACAACCAGAAAACTTTCTA
ACTTCTAAAGTTGACCATCAACGAAGATTGAAGTTCAATTAAAGATACAAGTATTCATTCAAGACTCCAA
GAACATCAAGTGGTCGATCCTCTTCCATACATCAACGTACGACATTCAACCAAAAAAGAATTAAAGGATC
AAGTATTAGAGATCGAACCAACATTACATCGAATTAACATAAATACAACATTAACACAAATTTAACTCCA
CAGAACATGTTTCTCCAAAAATCTCGTATGAACAATTATATACTTCGCTAAGTCCAATTTTTCTAATATT
CATGATTAAACTTAAACCAATTCATTTTGGGTGGGTTAGGTTTGATTTGTTCAATTTAATAGTTCACATA
TAGCCAAGTACTTTATTGATAATTTGCTCCTTTAATTTATTAAAATATTAAAACCATATTAAATGATAGT
TCAGCTAAAAGGACTAAACAAGTTTCTAGTTGGTAAAAATTACGCAGACTAGTACAAGTGAAAGACTAGC
ACATATTTTATAATTAGGTGACACAGTAAAAAATTGAGAGTATACTAATAAAAGCATAATTTAATTTAGT
AGGTAAAACATTTATTGTTTTTTTTAATAAAAAAGTATTTCAAATATTAGAAAATATTTACAAAATATAA
CAAAATTTTAAAATTTATAAATGATAGATACATATAGACATTGATAATAGGGAAATGGTAAAAAATAGCA
AATTCGACAAAATATTTACAACACATAGTAAGATTTCAGATTCTATTAATAATAAAGATTGATAGACATC
GATATGCTTCTGGTATTGATAAACAATGATAGAAGTCTATCAATTTCTATTATTGATAGAATCCAAAATT
TTGCTATAGTTTATAAATATTTTAATTTCTTTTGCTATTTTTAAAAATGCTCCTTGATAATATCTATCAT
TGATAGATTTTGAAAATTTACTTTTTTTTTTTTTTAATTTTTTTTTGGATATTTTGGTTCACTTTGTTACAT
TTGAAAAATATTATAAACTAAATTAGTTTATAAAAAATTGATCAAAGTTCAAAAATCACAATTTAAAGGA
AACTGTTTTAAATGACAAAACTACAAAAAATATTTATAAATAAAACAAAATATATATCATAGCTTATCAA
CAATATATTGCGACTATTATCTGTGAGTCTATTGCGACACAGATGTCTATCGCAGTTTTATTACTAATAA
AAAAGTAAATTTTATTATATTGGCAAATATTTTAATTTATTTTGTTATATTTGAAAACTTCACTCCAAAT
TAGAATGGATTAAATTTAGAAAAGAATAATAAAACTAAAAATTCAAATAATTAAAAGGTTAATTTTCATA
AATATAACAAAACACCAAAATATTTACGAATTGTTAACAAAATCAAAAAGCCTATCAAGTCGGCCATCTT
TTTTTAAAATATTTCAGGTTTGCCATCCCTTTTCATCTTCTTTCTTCTCTCCTCCTGCATTTTTTCTTTT
ATTTATTTTTGAAATCACGATCTTTATTTTCAATCAATCATAAATCTTGTAGTATTTTTTTTGTTCAAAA
TTGTGTACCAAATATAAAAGATTAAAGTACATGATCTTGAATAAAAATCGTTGAGATATTGTTATTCAGA
TTTGGCTACTAAATCTAAACGACATTGATATCTTGAAAAACAATTGTTGAGATATCGGTAGTTTAAATTT
AGGTAAACGACCGAGTAGCCAAATCTAAACGATCATGTAGCCAAATCTAAACGATCATGTAGCCAAATAT
AAAAGATTTATTTGTTAAAAAAAATCATTGTGGCACACGATCGTTTAGATTTAGTTACATGATCGTATAC
CAAATTGGTAGTTTAGATTTAGGTACACTATCGCGTAGCCAAATCTAAATAATCACGTAGCTAAATCTAA
ATGATCACGTAGTCAAATCTAAACGAATCTTGGTATAAGATCGTGTACCAAATATATTACGTGCGTAAGT
GACCAATTAATCGCATGTTAAACTATAGCATTTTTGTATTTCTTATTGTAGACATGTGGGCTTTTCCGTT
TTCGAAATTGTTCTATAAAGTGTAAAAATTTTATGAATTTGTTATACGGTTGAAAAAGCCCTAAATATAA
AGATAATGCTGAAGTAGTATTCAAATAAAAACAATAAATAAATAATAATAAAAAAAAATCAAAAAGGCTT
TGGGGGTGTTGTGTCTTCCAACTTGTTCTTTGTCAGCCTTAAATTTATTGGGGCTGTCCGTGAGTCGTTA
AAGCCCCAAACCAAATATGAAAAACATGTTTCTTGTTTGAACAATCATAAAACATGTATGAACTTTCCAC
GGACTTTTTTTTTTCTTTTTTCTCTTTTTCTTTTGTCAATAGTTCTTTCTAAATGCTTTTAGATATGGAA
GTAATTAATCGGATAAATTTCATTGAAATACTTTTGTCTTTAATCATTTTGCAGGACAATATCTATCAAG
ATAAAAAGAGAATAAGAAATCAAACCTTACATCTCTAGGATGTAGTCAATTATATTAATTATTTGCGTGA
GCTATGAAAAACAATTTTGATTCTTGCACTTTTTGGATTTTTTAATTTTAGTTTTTCTACTTTCAAATCT
ATAATTTTAGGTTCAGTATTTTATTATTAATTTACTGGTTTATTTTAACTTTTCTATTGGCTTAATTTTTT
TTTTAAGAAAATGCATATATATATATATATATGGTGAAGTTTTTTTTGCATGATTATTGTCTTTTTTTTT
TGTTATTTAATTAATTTTGGTAAAAATTAACTAAAAAAGAAGGATCGTCTGTCACTAATTATTACAAGGA
CACACATAATTTTACAAGATAAAAGAATGACAAAGAATGAAGCAGACACTAAAGATAATAGAAAGGGAGA
CAAAGTTTGATAAACACAATATCGTACAATTAATATCACCTACGTTTAAAGGACTCTGTTAAAACAAAAA
ATTCTGAATAAAGTTAAGCAATTATGATAATGTCGTACTTGTCTGTTAAATGAAAACATTGTAGAGACAA
TGAAAACTTGACTTTAGCAAAAAAATAACTTGGTGCTGTCCTAACTTTGAGATTTTTTTAAAACTGTAAT
CTTAATCTATTATTGTCTGTAACAAAAACATATACTCTTCTTCGTTGAGAAACTTGCACTCCCTTAGGTA
TGATTATAATCTCTCTCACAAATGATTTAGTGCGAGATAGTCTGGAGGGCATACCTCTTGACTATTAATA
CATAAGGTCTTTCAAAACACTTAAACATCAATGAACAAATGCAACTTCACCAACACACAATATTGATCGA
GAAACCGTCACTTTTTTCTTCTTTGGAAAATAAGTACGAGAGGTATGTAGGATCAATAAACACACTCAGA
TATCTCAAACTAGGTTGAAGACTTACACATATGACATTAGAAATTTTTTTTTTGCGAGAAAAAAGCCACA
ACCCTACAGCAATGAAAAAATTTCACTATATATTCACAATTTTAGGAAGGTAGGAAATATATGAACCAAA
GTTCAACCAACACATGGGGAGTGAAAGAATATCTAGAAAAATAAGAGGCTGCACAGTTAGAACTACATAA

TGCAAATAAAAATAGCATAGGGCTGAGGAAATTTCGGGAAATATCATAGAGAACAAGATATAGAATATGC
TCACAAACATCATGTAGGACCAAAAAAATATAGCCTAACACTAATACACACTAACAAGGACTATTAGAAG
TGAATGAACTAAAATGAGACACTTGCTATTTTTTTTCTCTTTTTTTTAGATAAATAATGTATAAAACTAA
TTAAAATGTACTTTTAAGAATAAAAAAAGAAAAAAAAAACTAGGATTGTTTTCTAACAAAATGAACCAAA
ATTATTATAAATATAGTAAAATTTCGGTCTACAATAGACGTGTCTATCTATAGATATAAACAGTAGTCTA
TCGTGGTTTATGATTGTGAAATTATACTATATTTATAAATATTTTTAGCCGTTTTTATATTTAAAATAAT
TTTTTTGAAAAACAAGAAAAAGGTTATCTTTTTTTAATTTCCTTTCACCAAAAAATTATCGTACATGGAA
AGAGAATATTTATTTCCTTTCTTTTTTAGATGAATAATAATTTGAAGTAAACATTAAAACATAAGAAATT
TAAATTAGATGTATAATGTCTTAAAACTCACGTGCATAGAAAAAGTTGAATCTAAGTTGGCCATTTTTAT
TTATTTTGTTCACATTGCCCAATTTGAGTGTGAAAATAAGAAAAAAAAATGCAGACGTTTGTAAGGAAAC
TTTCCTTACAAAAACCATAAAGTTAGAGTTAGACACATCATCGATGATGACGAGAGCAGTACACTTATAC
TTCTAGAAATGTTCTCTCTTTCCTTCCCAAAACCATAAAGTTTTAGAGTTAGATAATCATCCATAATGAA
GAGAGAGGGAATGAGAGGGAAATTTATTTAATATAGACATGGTGAAGGTAGATCTTAAAATTATTAGAGG
GTAAAATTCCACCGCTCTTATTGCTTAAAAGGTATATATATATATATATAGAGACACGGAGATTGATGAG
CTAGTTCGGTGCAAAATCATTTATATTTGGAGGTAGTGTGCTCGGTGAAACGAAACTTCACTATATAAAG
AGTTTAGGAATTACAACGAAAGTACTTAATTGTACACTCTGTGTTCAGTGACTCATGTATAGTTTCAACA
TAGGCTCCCCTAAGTTGAAGAATTCCTCACTTTCACAATAATATACTTCGCCTTCAAGTTTTGTATAAGA
TCCTCTTAATACAAAATGCTTAGGTGCTCCCTCTAAAAGTGAGATCTACTCATCTTTGCCATATTTAGGC
TTCCCCTAAATACAATATTAAATAAACAATCTTTCTGGAGATCTATAATAGCTATAGAAACACAAAGATA
CTCAATACTAATACACATGAAGGTTGAAGCTTAGACAATTCTTAAGCTCAAAACATTTAATAATGTACAA
AACTTATCTAATGTGGCAACAACCCTAAAAATGAAAAAGATCATCATATAAATATGATTAATGGTGAGAA
TATCTTTTAGGAGAGTCATTCTCTACAAGAAAATAAGAAGGCATATACGAAAAAAATCTACAAATCACAT
TGATATATATGAAAAAGATACTATGCAGAATCTGCACGCGGTCGTCAATTAACATTGTTCTTTAAAAAAT
GACATAGTGATCTTGACTATGTCTTTGACATGCTACAAAACATACTGCTCAAGTTTTGCTTGTAATAAAA
TAAGCCAATCAGTCGTGTAAAAAAATTAATCAACCACGCTATAGTAATAAATACCTAGAAATTTGAGATA
TTTGAATTTCTAACTCCATTAGGTGTGAAACTATGATTAAATAAAAAAAATAGAAATAAATAAATTGAAT
AGTATCATGACAAAATTGAATGCAAAATTTGAGTATGAAAATTTTATGCCTTCACACAATAATACTGTGG
CATTGTCTTTAAAATGGGTAAGTTAAAGTATATTAAACCCAAAAAATGTTTGTAGATTTTTCTATTTCTA
TGGGTTAATTTGAATTTGTTGGAACTTTAAAATAGTTGTTAGTACAACAATGTGTTTCTTTTTTTTTTAA
AAAAGACAGTATATGAAATTTTTACGAAAAGTTGGCGAACCACATAGAAGGATCCGTAACATATCATATG
AGGACTCAATTTTGTTTCTGCGTCTATGTATCAGTGTTTCAATATTCTGTTTTTAATAAAAATTTATTGT
TCCTCTTTTCGAAAAAGTGTCTCTTATCATTGTTTCTTTTTAGTAATGATCTCAATTAACTATAAAGAGT
TGGATCATTACACTTACAATTCTTATAAGATAGATAAAATGTTCTTCTCATTGTCAATTAATTTTAAGAT
GGAACCCCACTCACTACCAAATGATAGATATTGAAACTTTCCTATATATATATATATATTTATAAATATT
TTAAGTCAGTTTTATCCTTTCCTATAATTCTGTATCGAAAAACTTGCAAAAGTTTTTTTTTGGTTAGTTT
AAACTTTAAATAATAATTTTAAAATGTTGTATGCTTTTTATTTTGTAATTTGACTTTAAATGGCCCTACT
ATTAAATTTTGAATAATTGTTTTTTTTTTGAATGAGCACGTTTTTAAAAATTGATTTTAAACGGATTTAT
TAATTACAATCCATTTTTATTAAAGTTTTTTGAAAATGCTCAAAATCTATTTCAAAGGCTAACTAACACT
TAGTCCTTGGTAACTTAAATAATGCTTATTTTGATTTAGATAATTTTAGCAAAACATATTGAGTAACGAT
ATAATTCAATTAAGTAGATACATCTTACGTTTCTAATCCAACCCAAATACCAAAAAGATAAATATACATT
GAGGGTATGGTAGAAACATTATATATGCAAAAAAGAATAGATTTAGTGCTTCATAAAGCCACCAGATAGA
TAATGTCTTCCCAAAGGTGTGAAAAGGCTTTTATTTATTTTTACTTGTACTATTTTAATTTATAGTTTTT
TTTCAATAAGCTCTAAGTATGAATCATTACAATAAGCCTAAATGTAGCACTTCAACTACCGTTCACACAT
AACAAAAAAGACAACAACCACAAAGGGAGGTTCACACATGATCTTCGTGTCTTTATCAATTGCAGTTATC
GTTCTTCATTTGTTAAAAATAAAAGAACTAACCCTAAAAGATATTCAAATCAATAATATCACAATTATAT
TAGAAAAACAAAAGGGAAAATAGAATTACTACCGTTGTAATACTACCCTAAACGTTCCTGATAATAGAAA
CGACATGTTGTAATACTATTTTTCTAAATAAAATTTGGAAAAAAATTGGGTGCATTCTGATGCTCTCATCT
CTTACCAAACAAAAATAAAGTTATCAATTTCTTTTTTTTAAAAAAAAAATCAATTTTGAATGATTGAGAGA
TATGAACATATATGAGATAGGTGCATGGTGCACCTAATCACTGCCACTAAAATTCAGCCAACATATTACC
AATTGTACAATACTTCCAAATAATATAGTTTAATGTGGATATTTGAACTCAAGCTAGTTTAAGGTTCAAA
TTGACCAAGTATATATATATATATATATTTTTTCATATTTTTAAAAATACTGATATAAGATTAAATTTATTG
TAAGCAGTGGAGCAATAATTTTTGAGTTGTATCGTAATTTAATGTAGTATCATTATATTGACAATTATTA
TTAAGTGGACCTTGTGCTAATTTTCAAGCTCTAAGAATAATACAAGTGTGGAAAAACTTTTAAGAATATG
AATAAATATTTAAATTTTTAATAATTAACAATTAAATGCTCTCTCTTCCCAACTAAACCCAAAATCACTT
TAAAATCTACTTTAAACATTTTTCCATCTCTTATGAACAACTTAAAACCAACTGCTTTTCTTTTTAGGCC
AATTTTCGTTTTCATATTTATTGTACTCAAAGTAACTAAATGAAATCGTAAATATATATAACTTTTTGAA
TGCAATGTAAAATCGAACTTAAAAAGAAAGAGCACGATATACTTGAATCAATTATAAAACTAAAAAATGA
AAATAAAGATAAGGTGGTAAAAATGCAAAGTGGCATAAGTATGTGGCAGAGTTTTATTTCAACCGATTAA
AGAATAGTAAATGGCATTAATAATGTCACATGGTATGGGTCCACTCAATTAAGCATTCTGATTCAGTACT
ATAGAAGAGAGAAGAATTATAAATACGTGGCAAAAGAAGTTTAATAAGAAAATTGTTAATTTCTCGTTTGAT

AACTATCTGAGTTCTTTACATCAAAACGTCGAGTATAGTTCAATTAACGTATAACATATGTTATAGTATT
TTAGAGGTATATAGCTAAAATTTTCTCATTTCCATTATACTTGAGAAAATTATGTGAACTCCCATTTGTT
ATCCATTTTGTTTTTCTTTTGTTGTTTTTGAAGATTAAGTTTATTTTCTTTTAATGTCTTACAATGATTT
GCATCTTTCTTAAATACAATAATGGTTGAATTCTTAGCCAAACTTCAAAAACAAAAACAAGTTTTTGAAA
GTTACTTTTTCTTTTAGTTTTCAAATTTTGGTTTCATTTTTTAAACTATTGATAGAATGTGGATAAAAAA
GAAAAAAATTAGCGGTGAAAGTAGTGTATATAGACTCAATTTTCAAAAATCAAAAGCAAAAATGGTTACG
AAATGAATGAAAGAGTTTTGTTTTTTGGTTTTTGATTTTCAAATTTGAAGAGATTACTTTTATCTAAAGT
TCCTACTGTCTATTCTTTATAGATCACATGTTCTCAAAATCTAAGTCAGGCAAATGTACTGATAAAATAG
TAATAATTTAAGAAAACCAAAAAATAAAGAAAATAAATGAACAAAAGTGACAAATCGTAACTATATATAT
ATATATATATATATGTATAAACTCCAAATACATGTGAACTTTAAACTTTATAACTCAATTCAGCCCTCTA
AATAATAACAGCATATTAAAAATAGAGACACAAAGATTTGTTAAGGTAGTTTGATGCAATATGACTTACA
TTTGGAGGGTTATGTGTGAGCTAGAAAGAAATTTTACTATATAAAGAGTTACAACGAATGTGGTTATCCG
ATAAACTCACTCATTCCGTTATGTAATTAATTTAAAAAAGATATCAAATTATTGAATAGTAATATTTTGA
TTATAAGAACTTTAGTAGAAATATCGTGACTGAAAAAAATTCAAATGGATCCATCTCTATCAAAGAGATG
CTGCAAATAAAATCAAATTATTGCTATCATTTGATTATAACAATTCTTTATCTATTTTTGGTCTTTATTT
TTCCTTTTCTAAGTTTGGTACTAATGGATACAATCTGGTATCTCTTCTATGCAATATTCTATCCTCATCA
ATGGTAAAGCCAAAGGTAGAATCAAACCCTCTAGAAGAATTAGACAAGGAGACCCAATATCTACCTTTAT
GTTTGTCATTGCCATGGATTATCTGAGCAGAATTCTCCAACACCATGAGGAAAGACACAAGATCAAAGGA
GTTGTTATAAATGACCTCAATCTGACTCACCTTTTCTTTGCAGCTGATATTTTGCTGTTCGTTGAGAACA
ATGATGAATTTATCAGGAATATGCATATTGCTATGCATCTCTTTGAATGTGCTACTGGTTTAAACCTAAA
ACAGGGCTAAATCTACCATCTCCCCGATTAATATCAATAAAGAAAGAGCTGATGTGGTTCCCAATTGTCG
GGCCGGTTTCAGTACTCAATTTATGCCTATATCCTACCTTGGCATGCCTCTAAGATGAAAACCACTCTCA
CTGGCTTTCTGGAATAACATCACAGAGAGAGTGGAAAAGAAGCCAAACGTGTAAAGTAGCATTTTTAAGA
TTAAATTTCAAACACAAAACACAACAAACTAAATGGTGTTCAAATGGACAGAACTAAATCATTTTCAATA
TCCCCTCATTGAAGAAAATGAACAAGAAAATAGTTTGACATAAAAGGATTGGAAGACTTTCTCTAGCTTG
GACTTTCTTGTTATCAACAAAGCTCGACTGAGAAAAAGGAAAGATGAAGAAAGGATTATTTTGCAAATGA
AACAATTCTAAAAGGAACAAACAATGAAATGCATCTTTATGTGGTATATGACGAATGTGTTAGGGAGACG
GCAACCTAATTGAGATGTTCCAGTATACCAACTAATTCCACTCACCCTATCTTGTTAAAAAAAGAAAAAA
GTGCTAATAAAGAAAGTTCCCTGTAGCAATAACTTGTGTATATATTATTAACGAGAGTGAATCGTGCAAA
GGTGGTTGCCAACTACAAAAAGAGAGCCCACAAGAATTATAGTTCTTGTCGTTTCTCTTCACACAGTAAT
ATCTTTTCAAATTTTCACACACAATAACATTTGTTTTTTTGTTGCATATTTTTTGCTATAAATACTTGAG
GAAATTCACATCTCCAAACCATCCCATTCTTCAGTACTTAAGTAAGAGAAGGAAAAGAGAGAGAAGAAAA
TATGATTGGTAAGATTCTAGGAGATGCTCTTAAAACCACCGGCGACACCCTTAGAACGACCGGGGACATT
GCCGACTCCGTCCTAAATGCTGGAGGGAACTTTCTCGACCGTGCAAGAGATTTCCGTCGGCGCGGGAAGA
AGAAGATTAAAGGGAAGGTGATTCTAATGAGAAGCAATGTGCTAGACTTCACGGAATTTCATTCTACTAT
TCTTGATAACGTCGCTGAGTTGTTGGGCAGTGGAATTGAAATGAACCTCGTGAGTGCAACTGAGGTCGAT
CGCCATTGTAAGTTTCTTTTTAATAATTTACTTTGGTCACTGGATCCACAGAGATGAAATTGTGCCTGCT
TATATGTTGCTTAAATTAAAAAAAAAAAAAAAATCAACACGCATATAATAAGACAAGTGAATTTTGCTTA
AATAAACTATCACAAAAAGCAATCTCGAGTTTTAACGATCAATTTTTAGCGACTATTTTAATATCATCGC
ATGTTGAGAGCGACGAATTTCAATTTTAGTCTCTAACAGTGCAACAAAAGATGGATAAATCGCAATAATA
ATCTTGAATATTGGGGCTGGTTTTGATGTACTATATATATATATATCTTTGAAAGCCACTAAATCCTAAC
GGTATAAGTTTTTGGAGTTATCGATGAATTAACGTCATTGTTTGTATATAGCGAATGACCCGCGAGGTAA
AATTGGAACGAAAGCTGTTGTGGAGAGGTGGTTGACTTCAGCACCACCTGTGTTCGCTGGAGAGTCAGTA
TTTCAAGTGAACTTTGAATGGGAAGATGATTTTGGATATCCAGGAGCTTTCTATATAAGAAATGGACATA
CAAGTGAATTTTTTCTCAAGTCTCTCACTCTTGAAGATGTTCCTGGCTACGGAAGAGTTCATTTTGATTG
CAACTCATGGGTTTACCCAAAAAGAAGATACAGAAAAGATCGAATTTTCTTTGTCAACCAGGTAATTAGT
ACGTTGATTGCTGGTTCTTTCTCTTTTTAGGTTGAAATATTACTTAGGTTTCTCGTTTTTGTTCATTTCG
GTCTCTTTATACTTTCAAAATACTCGTTTTGATTTTTGTAAGCATATTATATATATTTTTTTCATTTTGG
TTATTGATTTTTTTTTAAAAAAAAATTATTTTGTTTCCATAAAATAAGATAAAAATGATATGGTCATTTT
CTCCTTGAGGATTGAAGAGTCTATTCTACATTCCTGCAATTATTCCTGTTAAAAAAAAAAAATGAGAGGA
TAAGATTGTTATTTTCCACAAAAATAAATATAATTTGAAAGTACAAATAATAAATGGAAATTATTTTAAT
AAAAAGTTCTAAAATTTTTAACAAATATAACAAAATATCATAATTTATTTATGATAGACCACGATAAATC
AAAGATAGCTAGCCTACTGTCTATGTCTATCATGATACATATAGACACAGATAATAGTCTATTACGATCT
ATTGTGCATAGACCTTGATATTTTACTATATTTTGTAAATATTCTAATTCATTTTATTATATTTGAAAAC
AGCTAGGCCAATAATAAAGTTATTTTTAGGTTCTTCAAGAATACTTTCCTAACTTTTTAAAGTACAAGGT
GTTAAGAATCTCGTATTGGTTTTGAGATAGAATCTCATACTATTAAATAATACGTATATTTTTTAAACAA
AACACTAACCACACGGGTATTTTTGAATTGTTACTAATAGTTTAACGGTATGCTTCTTTGACACAAACTA
AGTTTAGGAGTATTTTGTATAACTTAACACTAGAACATAGAAATATTTAAAAATGTCATATTCTTGTTTG
CTTTTGCAGTCATGGCTTCCAAGTGAGACACCTGAACCACTTCGAAAGTATAGAGAAGAAGAGCTATTGA
ATCTAAGAGGAGATGGAAGAGGAGAGCGTAAAGAATGGGATAGAATTTATGACTATGATGTTTACAATGA

CATTGCCGATCCTGATGCCAGTGATAAACTTGTTCGTCCTATTCTTGGAGGACCTGAATATCCTTATCCT
CGTAGAGGAAGAACCGGAAGACCGAGAACTAGAAGAGGTTGAAACTAATAGTAATAATAATATTCACATC
TAACTATATAAAAGTATTTATTAATTTGGATCAGGTCATGATGAGTACACACTAAGGAATGTGTAAATCT
AGCTAGTTGAGAGATCCGAATGCACCTAAAATCGACCCCCTGAACTTAATATTTTTTTTAAATAGTTTAC
AAAAATGGTTTTAAAAATATTTTTTAAATAAAGATACTAAAGAAAATGAGAGTCGATTAGTAGGTGCACC
AGACTATCAAAACTAGATCTTGACAAATTATTCCTAGCATCTTAGTTGCATTTGGATTTTGAAAATTAGA
CTTATAATGACTCCCATGCATACTCAACCATCATGAGCTTGACCTAATGAATAACAGGGGGTTGACGTAC
GTTGATAAAGCATTAAGAAGTTATGAGTATAGATCATAGTGACCATCTACCTAAGATTTAATATGTGAGG
TTAATTGAGGTGCCTTGTGTTAACATGCCGGCAAAGTATAGATATTAAAAAAAAATAAAAATAAAACAAT
GGAAAAAGTAAATTAAGTTTTAAAAACTACTTTACTTTAAGAATATGACGTAGAGTCCAAGTGTTTTCTT
AACGAAGGTAAAAATCATAATTCATAAATAAAAAAATGAAATTGGTACCACCTTTGGATTCTGGTTTTTG
AATTTTCAAAATTAAGCTTGAAAACCATATGTACTTCATTGATTTTTGTTATCCACTATCTACTACTAAG
TTTATTTTTATTTGTTTCGGAAACTTCTGTATATTATTTGAATTGAAAGGGTGTCACATTAACTTCTGTC
AGCTACAGTATTAAGAACTGGCAACAAGGCCAAAAATGCTATTGAACTGAAACCTCACGGGTTTAACTGT
CAAGTTGAAAATTTACGAACCAAAATATGCTAACAAAATCAAACCTCTGGGATAAAAAAAGTTATTTTCT
CAGTGTACAAAAAAAAAAAAAGAATTGGCAGGTGGATTGAGACATTTCAACTAGAGTTTACACATACCGA
TCGATAGCACTCTCGTTCTACAATGTTATAATACTTATTTTTTGGGTGAATTTTGCAGATCCAAATTCAG
AGAGGAGATTACAATCAGTGATAGGGCTAAACATTTATGTACCAAGAGATGAAAATTTTGGACATTTGAA
GATGGGAGATTTTCTTGGGTATGCATTGAAAGCACTTTCTGCAAGCATCAAACCTGGGCTTCAAACTGTA
TTTGATACAACTCCAGGAGAATTTGACAATTACAAAGAATTGTATAATCTTTATGAAGGAGGATTTCCTA
TTCCACAAAATCTCTTTACACTCCTTTCTGATTCCCTCACAGCACCATTGTTAAAAGAGGTTTTAAGAAT
TGATGGTGACAGATTCCTCAGATTTGCAGTTCCTGATGTCATCAAAGGTGCGGATAATTCGATTCATAAC
GATTTTCTTTTTCGATATTTATGTTTTCTTTCTTATATATACTTGAATTCTTAGATGAATTTTAAAAACA
AAGAAAACAAAGTTTTTGAAAATGGATTGTTTTTGGAAAGTAAATAGCAAAAATACAAAGGAATTTATTG
GTAAAAATTGATTTTATCATTATAATTTTCAAAAACTAAAAACAAAAAGATCTAGGCTAGAAGCTGATGT
AAGGTGGATTATTATTACTCAATTCATGTTTGTGACTCAAATTATAATAGTTGGGTGTTTCCAAGGTCGA
TCTAACTATAGCCTACAATTTACTACAGTATTATTATTATTATTATTTTCAAAAAGAGATACGATTTTTA
GTGTACAATATTACTATTATTTCAAATCCCTCTTTGCTACAAAAATTTATTATTTTTACTATTCTTATTC
CATCCTCTATTTTTCTCTTTGTCACAGTGTTTACTATTTTCTAACACACGAACATAGTTTTTGTCGAACT
TGGGATATGATGAGAATGCTAATGGGGTATAAAAAAATAATAACCACCTCAAACATAGACTATTATAACC
AATAAATTGTAATTTTTTTAAAATAATTGCAGGTTGGAAGGATCAAAATTAAAAATTATATTTTTTTAGC
TTTCAAAATTTGACATTTTTTAGAAGTTTTCTTATTAATAATTTAAGTGTTTCAGTATAAAAAAAGTGAA
GTCTCGTCGAGCCAAATGATTATCAAACCAACTAAATAATTAAATAATATGTTATCCAAAATAGAAAAAG
ATATTATAAATTAATTTGGTGATCCTTTGATTTGGCCAGATGACAAAAGTGCATGGAGGACTGATGCAGA
ATTTGCAAGAGAAATGATAGCCGGAGTTAACCCTATTCTCATTTGTCGTCTTGAAGTATATTTTTTTCTA
TTTAATTTCTTCGTTTGATCATTAAATTTTGCTTTAGCTTCTTCTTCGTCTCATTTCCAAATATAGCAAA
ATGAAAGAAAATTTTTACAAAATAATATAACAAAACCTCAAAATCCCTCGATGATATACACTCATTGACG
ACACTAATAGACTTTGATTAGTGTCTATTAGTGATAGTCTTCCAACGTCTATCAACATCTGTTATTGATA
CATTTGAAAACTTTGTTATATTTACAATTATTTTTATAAATTTTGTCATTTGAAATATTTTTCATTATTA
TAAGTGTTTATTTTGACTTTTTTTTTTTTTTACTGACAGCATTTTCCACCATTGAGTAAACTTGATCCTAA
ACGTTATGGAAATCAAAATAGCACAATTACCGAAGAACAAATAAAGGATGGCTTGGAAGGGCTCAGTGTT
CATGAGGTGAATTATTCAAATTAACTAATTAACATGGCCTTGATGATGCATTTGTTGAAAAGTTTTCCTA
AAAGAGATTTTCTCTTAAAAAAATTAAGAAAAATCATAGGTTATAAGCTAAGTATGTATTAAGCAAAGAA
AAAAAAATGTTGGAATATTTTCTACTAAATATGGTTAAGAAGGAAAAAAGAAAAAAGAAAAACCATTTAT
ACCTAAACTTTGAGAGCTGTATAAATTAAAACTATAAACTATAAATTTTATTATTTTAAAATATGAACTT
TTTGTCGAAGAAAAAATGTGTTGATGATATACAAAGTCCTATGGATTTTGTTTGACCAATATTGTATTAA
ACTTTGGTCCATTGAACTTCTAATTTTTCATTAGCCAATCACTTTAGACTAGTATTCATAATCTATGAAA
ATAATATCCATGCATCAATTATCAAAATGGGTAGACTTCTTCAAATGCTGATTTACAAAATGGACAATTA
CATAGTAATTGCATTGATTTTTTCAAAAGAAATTTTGTCCAAGTTGTTTCATCTTATAAGTTTCACTGAG
ATTTTTCTAAAAAGAATGTAATGGAAGGTGCAATAACTTGATACAATAACAAAAGTGCAGGGTTTTATAA
ATTGAAACGATCATTAGTTTACTTTTGTCCTTAAGCATATCTCTTTTGTTTTCCTCTTCACCAACTCTTT
TATAATTATTTAAAAAAAACTCTTTTATAATTATTAATATAATTTTTTTTGGCAATAAATCTCCTTCATT
TGGGTTTGAGAGATGTGATGAGAATGATAAAGTTGAGCTAGCCAAGCTGATATATATGTGTGTGTGTGTG
TGGGTGCATCTACACCAACCCCTCCTTCTCCCCTTGGTGAATTGTTAAAAAAAAAAAATATAGTGGAAGGT
TTAAATTGATATAGCCCATAATAGTTAGGGCTATTAATCGATATTTTTCCGAGAAAAACATAGTTAAATT
AAATGATTTTTAGTTTTCTCCTTCTATTTTTCAATGGAAATTGATAATTAGGATATGGTGGATTTGATTG
ATGAAATAGGCAATGAAGGAGAACAAGTTATACATATTGGATCATCACGATGCATTGATGCCATATCTTA
GAAAGATAAACTCAACATCGACAAAGACTTATGCCACAAGAACACTACTCTTTCTGAAAGATGATGGGAC
TTTAAAGCCTTTGGCTATTGAGTTGAGCTTGCCACATCCTCAAGGAGAACAACTTGGTGCCATTAGCAAA
CTATACTTTCCAGTGGAAAAAAGTAATGTTGAAGGCTCAATTTGGCAACTAGCCAAGGCTTATGTGGCTG

TTAATGATGCTGGATACCATCAACTTATTTCCCATTGGTATAATTTCTCATAAAAATCTTCTACAATAAT
GACATTTTGATCTATCTATTTCTGTTTTCTGAGTTTAACATTTTGATTTATGACAATTGAGCTATGCGCA
TCTTGATTATTGAGTTATGTGTAAGATTTGTTTATCTTCTGCCTATCGAAACTGTAGGTTGAATACACAT
GCGGTACTTGAGCCTTTTGTGATTGCAACACATCGGCAATTAAGTGCGCTTCATCCAATTCATAAGTTAT
TGGCTCCACATTATAAAGATACCATGTTCATAAATGCATTTGCAAGGCAAACCCTCATTAATGCTGATGG
ACTTCTTGAAGCAACTCATTTTCAATCAAAATTTGCCATGGAGTTGTCTTCTTACATATACAGAGAATGG
AATTTCCTTGAACAAGCCTTACCCGCAGATCTCCTAAAGAGGTAATCTATATTTTTTTTAAATCATAAAA
AAATGCCTCTATATATAAACTTATGGAGTTAATTTCAATTATACTCTTCAAACTTTGAAAATTATTTGAA
CCATGATGTTTGAAGGTTTTTTTTTCTTTCAAGACAATCCTTTAAAGTTAGGATGAAAAAAATGTGTGCA
AGCTTATATGTGATGACTAAAGTGAACAAATCTAAACACACCTGGGAAGCTAAGGTAAATTTAGAGAATA
GGGTTCGTCAAGTGTCAATATGTTTTTTTCCGAAAATATGAGTTCAGATAAGAAACTAAAAATTCTATTT
CGTCCGTGATTATAGTAGTCAAAGAGTTAATTTATATATTTTCTTTTTTCTTCCTTGATTTATAACAATT
ATAATAATATATAAATGGTATATACCGATAAAATGCCATTGATCATTTTGATATTTTTGTATAGAGGAGT
AGCAATTAAGGACCCAAGTTCTCCACATGGACTCAAGTTATTGATAGAGGATTATCCATATGCTGTTGAT
GGACTTGAAATTTGGTCAACTATCAAAAATTGGGTGACAGATTATTGCAGTATCTACTACAAAGATGACA
CTGCAGTCCAAAATGACATTGAACTCCAATCTTGGTGGAAAGAAGTTGTAGAGAAAGGTCATGCTGACAA
GAAACATGAAGCATGGTGGCCAAAGATGCAAAGTTTAAATGAACTAATAGAATCTTGCACCATTATTATA
TGGATTGGTTCAGCTCTGCATGCCGCTGTTAATTTCGGACAATATCCTTACGGAGGCTTTGTTCCTAATC
GTCCAACGGTAAGTCGAAGATTTATGCCTGAAGTCGGTAGTAAAGAGTATAAAGAACTTGAATCTTGCCC
TGAAAAAGCCTTCTTGAGAACCATAAATTCTCAACTACAATGCCTTCTTGGAATGTCACTTATTGAAATT
TTATCAAGACATGCTTCTGATGAAGTTTATCTTGGGAAAAGAGGTAGCTTAGAATGGACATGTGATAAAG
ATGCATTAGAGGCATTTGATGATTTTGGTCAAGAAGTGAATGAAGTTGAAGAAAGGATTATGGAAAGGAA
TCGCAACATCAAATTCAAGAATAGAACTGGCCAAGCTAATGTTCCTTATACTATGCTTCTTCCTTCTAGC
ACCGAAGGAATCACTGGCAAAGGAATTCCTAATAGTATCTCTATCTAAAATAGCTATTTGGTTTGTGTGT
GTTCTGATTATGTAAGTTTTATTTTTCTTGCATGTTGTTTTTATCTTTTTGGTTTGACCTATTTCTTAAG
CTATATGAAATAAAAAATTAAAAATGCTCAAGGGTAGGTTTGGATTTGAAAGTGTTAGTTGGGTTTTTAA
ATTTTATGCATGTATTCAACTCTGTTATTGTGCTGTCTTACTTTTTGGTGTGTTTGTGAAAGGAATAAAA
GAATTGTGCTTTATGTTTTAATTAATTCCCTTTGTAGTCTCGATTTAGAAACTATGAATAAATTATTATT
GTTCTTTAAATGACCACAAAATAATCTATAAATATGGGATTTCTTGTTTTTGTCTCTTGTAAAGTACACA
AAAATAGAGAAGAACTTTGAAAAGACACATGTTGGTTTAGTAAAAGAGTTCGAATAATATTTAATAGTAA
AGTAAAAAAATGCATCATTTTAAAGGAGCAAGTTAAAAGATTATTCTGATATAGAAAGATGAAATGATCGA
CTAGTTGTATGGATTCGATAAAAATTTGAAATTTGATGTGATCTTAGTACAAGCATACACAGTCAATAAG
TAAGGGCTTTAAAATATTCAAAGTATTGTATCCATGAGACTAATTACAGTAAAAGTTAAAAGGAAAGAAG
GGTAAAATATACGACATCACTTTTTATTCCTTCATAAATAAAATACAAATAAGCTACTTAAGCATATACT
CTATGGCTTTAATTCCAAGAGTAGCTAAGCTAAGCCAAGTAGCTTTTTCTCATTAAACATAAGAGAGAGT
GACAATAATACAGAACAAGGAAATGAAATATTTATAACCCATGCTCAGAGTTTCCTTTCAAATAGAAATG
CTGTTTGGAATTCCTCTGCCTGTGAGCCCTTCAGTACTTGATGGAAGAAGCAAAGTATATGGAACATTAA
CGGGTCCAGTTCTATTTTTGAGATTCACATCTTTATTCCTTTCAATGATTCTACTTTCAACTTCAAACAC
TTGTTTTCCAAAATTCTCAAACACTTCCAGTGCAGCTTTGTCGGAAGTCCATTCAATACTAGCTCTTTGT
CCAAGATAAACTTCATCAGAAGCATGCCTTGACAATATTTCAATTAGTGAAACTCCAAGAAGTGTTTGCA
ACATCGAATTGATTGTGTTCAAGNNNNNNNNNNNNNNNNNNNNNNNNNNNNNNNNNNNNNNNNNNNNNNN
NNNNNNNNNNNNNNNNNNNNNNNNNNNNNNNNNNNNNNNNNNNNNNNNNNNNNNNNNNNNNNNNNNNNNN
NNNNNNNNNNNNNNNNNNNNNNNNNNNNNNNNNNNNNNNNNNNNNNNNNNNNNNNNNNNNNNNNNNNNNN
NNNNNNNNNNNNNNNNNNNNNNNNNNNNNNNNNNNNNNNNNNNNNNNNNNNNNNNNNNNNNNNNNNNNNN
NNNNNNNNNNNNNNNNNNNNNNNNNNNNNNNNNNNNNNNNNNNNNNNNNNNNNNNNNNNNNNNNNNNNNN
NNNNNNNNNNNNNNNNNNNNNNNNNNNNNNNNNNNNNNNNNNNNNNNNNNNNNNNNNNNNNNNNNNNNNN
NNNNNNNNNNNNNNNNNNNNNNNNNNNNNNNNNNNNNNNNNNNNNNNNNNNNNNNNNNNNNNNNNNNNNN
NNNNNNNNNNNNNNNNNNNNNNNNNNNNNNNNNNNNNNNNNNNNNNNNNNNNNNNNNNNNNNNNNNNNNN
NNNNNNNNNNNNNNNNNNNNNNNNNNNNNNNNNNNNNNNNNNNNNNNNNNNNNNNNNNNNNNNNNNNNNN
NNNNNNNNNNNNNNNNNNNNNNNNNNNNNNNNNNNNNNNNNNNNNNNNNNNNNNNNNNNNNNNNNNNNNN
NNNNNNNNNNNNNNNNNNNNNNNNNNNNNNNNNNNNNNNNNNNNNNNNNNNNNNNNNNNNNNNNNNNNNN
NNNNNNNNNNNNNNNNNNNNNNNNNNNNNNNNNNNNNNNNNNNNNNNNNNNNNNNNNNNNNNNNNNNNNN
NNNNNNNNNNNNNNNNNNNNNNNNNNNNNNNNNNNNNNNNNNNNNNNNNNNNNNNNNNNNNNNNNNNNNN
NNNNNNNNNNNNNNNNNNNNNNNNNNNNNNNNNNNNNNNNNNNNNNNNNNNNNNNNNNNNNNNNNNNNNN
NNNNNNNNNNNNNNNNNNNNNNNNNNNNNNNNNNNNNNNNNNNNNNNNNNNNNNNNNNNNNNNNNNNNNN
NNNNNNNNNNNNNNNNNNNNNNNNNNNNNNNNNNNNNNNNNNNNNNNNNNNNNNNNNNNNNNNNNNNNNN
NNNNNNNNNNNNNNNNNNNNNNNNNNNNNNNNNNNNNNNNNNNNNNNNNNNNNNNNNNNNNNNNNNNNNN
NNNNNNNNNNNNNNNNNNNNNNNNNNNNNNNNNNNNNNNNNNNNNNNNNNNNNNNNNNNNNNNNNNNNNN
NNNNNNNNNNNNNNNNNNNNNNNNNNNNNNNNNNNNNNNNNNNNNNNNNNNNNNNNNNNNNNNNNNNNNN
NNNNNNNNNNNNNNNNNNNNNNNNNNNNNNNNNNNNNNNNNNNNNNNNNNNNNNNNNNNNNNNNNNNNNN

NNNNNNNNNNNNNNNNNNNNNNNNNNNNNNNNNNNNNNNNNNNNNNNNNNNNNNNNNNNNNNNNNNNNNN
NNNNNNNNNNNNNNNNNNNNNNNNNNNNNNNNNNNNNNNNNNNNNNNNNNNNNNNNNNNNNNNNNNNNNN
NNNNNNNNNNNNNNNNNNNNNNNNNNNNNNNNNNNNNNNNNNNNNNNNNNNNNNNNNNNNNNNNNNNNNN
NNNNNNNNNNNNNNNNNNNNNNNNNNNNNNNNNNNNNNNNNNNNNNNNNNNNNNNNNNNNNNNNNNNNNN
NNNNNNNNNNNNNNNNNNNNNNNNNNNNNNNNNNNNNNNNNNNNNNNNNNNNNNNNNNNNNNNNNNNNNN
NNNNNNNNNNNNNNNNNNNNNNNNNNNNNNNNNNNNNNNNNNNNNNNNNNNNNNNNNNNNNNNNNNNNNN
NNNNNNNNNNNNNNNNNNNNNNNNNNNNNNNNNNNNNNNNNNNNNNNNNNNNNNNNNNNNNNNNNNNNNN
NNNNNNNNNNNNNNNNNNNNNNNNNNNNNNNNNNNNNNNNNNNNNNNNNNNNNNNNNNNNNNNNNNNNNN
NNNNNNNNNNNNNNNNNNNNNNNNNNNNNNNNNNNNNNNNNNNNNNNNNNNNNNNNNNNNNNNNNNNNNN
NNNNNNNNNNNNNNNNNNNNNNNNNNNNNNNNNNNNNNNNNNNNNNNNNNNNNNNNNNNNNNNNNNNNNN
NNNNNNNNNNNNNNNNNNNNNNNNNNNNNNNNNNNNNNNNNNNNNNNNNNNNNNNNNNNNNNNNNNNNNN
NNNNNNNNNNNNNNNNNNNNNNNNNNNNNNNNNNNNNNNNNNNNNNNNNNNNNNNNNNNNNNNNNNNNNN
NNNNNNNNNNNNNNNNNNNNNNNNNNNNNNNNNNNNNNNNNNNNNNNNNNNNNNNNNNNNNNNNNNNNNN
NNNNNNNNNNNNNNNNNNNNNNNNNNNNNNNNNNNNNNNNNNNNNNNNNNNNNNNNNNNNNNNNNNNNNN
NNNNNNNNNNNNNNNNNNNNNNNNNNNNNNNNNNNNNNNNNNNNNNNNNNNNNNNNNNNNNNNNNNNNNN
NNNNNNNNNNNNNNNNNNNNNNNNNNNNNNNNNNNNNNNNNNNNNNNNNNNNNNNNNNNNNNNNNNNNNN
NNNNNNNNNNNNNNNNNNNNNNNNNNNNNNNNNNNNNNNNNNNNNNNNNNNNNNNNNNNNNNNNNNNNNN
NNNNNNNNNNNNNNNNNNNNNNNNNNNNNNNNNNNNNNNNNNNNNNNNNNNNNNNNNNNNNNNNNNNNNN
NNNNNNNNNNNNNNNNNNNNNNNNNNNNNNNNNNNNNNNNNNNNNNNNNNNNNNNNNNNNNNNNNNNNNN
NNNNNNNNNNNNNNNNNNNNNNNNNNNNNNNNNNNNNNNNNNNNNNNNNNNNNNNNNNNNNNNNNNNNNN
NNNNNNNNNNNNNNNNNNNNNNNNNNNNNNNNNNNNNNNNNNNNNNNNNNNNNNNNNNNNNNNNNNNNNN
NNNNNNNNNNNNNNNNNNNNNNNNNNNNNNNNNNNNNNNNNNNNNNNNNNNNNNNNNNNNNNNNNNNNNN
NNNNNNNNNNNNNNNNNNNNNNNNNNNNNNNNNNNNNNNNNNNNNNNNNNNNNNNNNNNNNNNNNNNNNN
NNNNNNNNNNNNNNNNNNNNNNNNNNNNNNNNNNNNNNNNNNNNNNNNNNNNNNNNNNNNNNNNNNNNNN
NNNNNNNNNNNNNNNNNNNNNNNNNNNNNNNNNNNNNNNNNNNNNNNNNNNNNNNNNNNNNNNNNNNNNN
NNNNNNNNNNNNNNNNNNNNNNNNNNNNNNNNNNNNNNNNNNNNNNNNNNNNNNNNNNNNNNNNNNNNNN
NNNNNNNNNNNNNNNNNNNNNNNNNNNNNNNNNNNNNNNNNNNNNNNNNNNNNNNNNNNNNNNNNNNNNN
NNNNNNNNNNNNNNNNNNNNNNNNNNNNNNNNNNNNNNNNNNNNNNNNNNNNNNNNNNNNNNNNNNNNNN
NNNNNNNNNNNNNNNNNNNNNNNNNNNNNNNNNNNNNNNNNNNNNNNNNNNNNNNNNNNNNNNNNNNNNN
NNNNNNNNNNNNNNNNNNNNNNNNNNNNNNNNNNNNNNNNNNNNNNNNNNNNNNNNNNNNNNNNNNNNNN
NNNNNNNNNNNNNNNNNNNNNNNNNNNNNNNNNNNNNNNNNNNNNNNNNNNNNNNNNNNNNNNNNNNNNN
NNNNNNNNNNNNNNNNNNNNNNNNNNNNNNNNNNNNNNNNNNNNNNNNNNNNNNNNNNNNNNNNNNNNNN
NNNNNNNNNNNNNNNNNNNNNNNNNNNNNNNNNNNNNNNNNNNNNNNNNNNNNNNNNNNNNNNNNNNNNN
NNNNNNNNNNNNNNNNNNNNNNNNNNNNNNNNNNNNNNNNNNNNNNNNNNNNNNNNNNNNNNNNNNNNNN
NNNNNNNNNNNNNNNNNNNNNNNNNNNNNNNNNNNNNNNNNNNNNNNNNNNNNNNNNNNNNNNNNNNNNN
NNNNNNNNNNNNNNNNNNNNNNNNNNNNNNNNNNNNNNNNNNNNNNNNNNNNNNNNNNNNNNNNNNNNNN
NNNNNNNNNNNNNNNNNNNNNNNNNNNNNNNNNNNNNNNNNNNNNNNNNNNNNNNNNNNNNNNNNNNNNN
NNNNNNNNNNNNNNNNNNNNNNNNNNNNNNNNNNNNNNNNNNNNNNNNNNNNNNNNNNNNNNNNNNNNNN
NNNNNNNNNNNNNNNNNNNNNNNNNNNNNNNNNNNNNNNNNNNNNNNNNNNNNNNNNNNNNNNNNNNNNN
NNNNNNNNNNNNNNNNNNNNNNNNNNNNNNNNNNNNNNNNNNNNNNNNNNNNNNNNNNNNNNNNNNNNNN
NNNNNNNNNNNNNNNNNNNNNNNNNNNNNNNNNNNNNNNNNNNNNNNNNNNNNNNNNNNNNNNNNNNNNN
NNNNNNNNNNNNNNNNNNNNNNNNNNNNNNNNNNNNNNNNNNNNNNNNNNNNNNNNNNNNNNNNNNNNNN
NNNNNNNNNNNNNNNNNNNNNNNNNNNNNNNNNNNNNNNNNNNNNNNNNNNNNNNNNNNNNNNNNNNNNN
NNNNNNNNNNNNNNNNNNNNNNNNNNNNNNNNNNNNNNNNNNNNNNNNNNNNNNNNNNNNNNNNNNNNNN
NNNNNNNNNNNNNNNNNNNNNNNNNNNNNNNNNNNNNNNNNNNNNNNNNNNNNNNNNNNNNNNNNNNNNN
NNNNNNNNNNNNNNNNNNNNNNNNNNNNNNNNNNNNNNNNNNNNNNNNNNNNNNNNNNNNNNNNNNNNNN
NNNNNNNNNNNNNNNNNNNNNNNNNNNNNNNNNNNNNNNNNNNNNNNNNNNNNNNNNNNNNNNNNNNNNN
NNNNNNNNNNNNNNNNNNNNNNNNNNNNNNNNNNNNNNNNNNNNNNNNNNNNNNNNNNNNNNNNNNNNNN
NNNNNNNNNNNNNNNNNNNNNNNNNNNNNNNNNNNNNNNNNNNNNNNNNNNNNNNNNNNNNNNNNNNNNN
NNNNNNNNNNNNNNNNNNNNNNNNNNNNNNNNNNNNNNNNNNNNNNNNNNNNNNNNNNNNNNNNNNNNNN
NNNNNNNNNNNNNNNNNNNNNNNNNNNNNNNNNNNNNNNNNNNNNNNNNNNNNNNNNNNNNNNNNNNNNN
NNNNNNNNNNNNNNNNNNNNNNNNNNNNNNNNNNNNNNNNNNNNNNNNNNNNNNNNNNNNNNNNNNNNNN
NNNNNNNNNNNNNNNNNNNNNNNNNNNNNNNNNNNNNNNNNNNNNNNNNNNNNNNNNNNNNNNNNNNNNN
NNNNNNNNNNNNNNNNNNNNNNNNNNNNNNNNNNNNNNNNNNNNNNNNNNNNNNNNNNNNNNNNNNNNNN
NNNNNNNNNNNNNNNNNNNNNNNNNNNNNNNNNNNNNNNNNNNNNNNNNNNNNNNNNNNNNNNNNNNNNN
NNNNNNNNNNNNNNNNNNNNNNNNNNNNNNNNNNNNNNNNNNNNNNNNNNNNNNNNNNNNNNNNNNNNNN
NNNNNNNNNNNNNNNNNNNNNNNNNNNNNNNNNNNNNNNNNNNNNNNNNNNNNNNNNNNNNNNNNNNNNN
NNNNNNNNNNNNNNNNNNNNNNNNNNNNNNNNNNNNNNNNNNNNNNNNNNNNNNNNNNNNNNNNNNNNNN
NNNNNNNNNNNNNNNNNNNNNNNNNNNNNNNNNNNNNNNNNNNNNNNNNNNNNNNNNNNNNNNNNNNNNN
NNNNNNNNNNNNNNNNNNNNNNNNNNNNNNNNNNNNNNNNNNNNNNNNNNNNNNNNNNNNNNNNNNNNNN
NNNNNNNNNNNNNNNNNNNNNNNNNNNNNNNNNNNNNNNNNNNNNNNNNNNNNNNNNNNNNNNNNNNNNN
NNNNNNNNNNNNNNNNNNNNNNNNNNNNNNNNNNNNNNNNNNNNNNNNNNNNNNNNNNNNNNNNNNNNNN

NNNNNNNNNNNNNNNNNNNNNNNNNNNNNNNNNNNNNNNNNNNNNNNNNNNNNNNNNNNNNNNNNNNNNNNNNNNNNNN
NNNNNNNNNNNNNNNNNNNNNNNNNNNNNNNNNNNNNNNNNNNNNNNNNNNNNNNNNNNNNNNNNNNNNNNNNNNNNNN
NNNNNNNNNNNNNNNNNNNNNNNNNNNNNNNNNNNNNNNNNNNNNNNNNNNNNNNNNNNNNNNNNNNNNNNNNNNNNNN
NNNNNNNNNNNNNNNNNNNNNNNNNNNNNNNNNNNNNNNNNNNNNNNNNNNNNNNNNNNNNNNNNNNNNNNNNNNNNNN
NNNNNNNNNNNNNNNNNNNNNNNNNNNNNNNNNNNNNNNNNNNNNNNNNNNNNNNNNNNNNNNNNNNNNNNNNNNNNNN
NNNNNNNNNNNNNNNNNNNNNNNNNNNNNNNNNNNNNNNNNNNNNNNNNNNNNNNNNNNNNNNNNNNNNNNNNNNNNNN
NNNNNNNNNNNNNNNNNNNNNNNNNNNNNNNNNNNNNNNNNNNNNNNNNNNNNNNNNNNNNNNNNNNNNNNNNNNNNNN
NNNNNNNNNNNNNNNNNNNNNNNNNNNNNNNNNNNNNNNNNNNNNNNNNNNNNNNNNNNNNNNNNNNNNNNNNNNNNNN
NNNNNNNNNNNNNNNNNNNNNNNNNNNNNNNNNNNNNNNNNNNNNNNNNNNNNNNNNNNNNNNNNNNNNNNNNNNNNNN
NNNNNNNNNNNNNNNNNNNNNNNNNNNNNNNNNNNNNNNNNNNNNNNNNNNNNNNNNNNNNNNNNNNNNNNNNNNNNNN
NNNNNNNNNNNNNNNNNNNNNNNNNNNNNNNNNNNNNNNNNNNNNNNNNNNNNNNNNNNNNNNNNNNNNNNNNNNNNNN
NNNNNNNNNNNNNNNNNNNNNNNNNNNNNNNNNNNNNNNNNNNNNNNNNNNNNNNNNNNNNNNNNNNNNNNNNNNNNNN
NNNNNNNNNNNNNNNNNNNNNNNNNNNNNNNNNNNNNNNNNNNNNNNNNNNNNNNNNNNNNNNNNNNNNNNNNNNNNNN
NNNNNNNNNNNNNNNNNNNNNNNNNNNNNNNNNNNNNNNNNNNNNNNNNNNNNNNNNNNNNNNNNNNNNNNNNNNNNNN
NNNNNNNNNNNNNNNNNNNNNNNNNNNNNNNNNNNNNNNNNNNNNNNNNNNNNNNNNNNNNNNNNNNNNNNNNNNNNNN
NNNNNNNNNNNNNNNNNNNNNNNNNNNNNNNNNNNNNNNNNNNNNNNNNNNNNNNNNNNNNNNNNNNNNNNNNNNNNNN
NNNNNNNNNNNNNNNNNNNNNNNNNNNNNNNNNNNNNNNNNNNNNNNNNNNNNNNNNNNNNNNNNNNNNNNNNNNNNNN
NNNNNNNNNNNNNNNNNNNNNNNNNNNNNNNNNNNNNNNNNNNNNNNNNNNNNNNNNNNNNNNNNNNNNNNNNNNNNNN
NNNNNNNNNNNNNNNNNNNNNNNNNNNNNNNNNNNNNNNNNNNNNNNNNNNNNNNNNNNNNNNNNNNNNNNNNNNNNNN
NNNNNNNNNNNNNNNNNNNNNNNNNNNNNNNNNNNNNNNNNNNNNNNNNNNNNNNNNNNNNNNNNNNNNNNNNNNNNNN
NNNNNNNNNNNNNNNNNNNNNNNNNNNNNNNNNNNNNNNNNNNNNNNNNNNNNNNNNNNNNNNNNNNNNNNNNNNNNNN
NNNNNNNNNNNNNNNNNNNNNNNNNNNNNNNNNNNNNNNNNNNNNNNNNNNNNNNNNNNNNNNNNNNNNNNNNNNNNNN
NNNNNNNNNNNNNNNNNNNNNNNNNNNNNNNNNNNNNNNNNNNNNNNNNNNNNNNNNNNNNNNNNNNNNNNNNNNNNNN
NNNNNNNNNNNNNNNNNNNNNNNNNNNNNNNNNNNNNNNNNNNNNNNNNNNNNNNNNNNNNNNNNNNNNNNNNNNNNNN
NNNNNNNNNNNNNNNNNNNNNNNNNNNNNNNNNNNNNNNNNNNNNNNNNNNNNNNNNNNNNNNNNNNNNNNNNNNNNNN
NNNNNNNNNNNNNNNNNNNNNNNNNNNNNNNNNNNNNNNNNNNNNNNNNNNNNNNNNNNNNNNNNNNNNNNNNNNNNNN
NNNNNNNNNNNNNNNNNNNNNNNNNNNNNNNNNNNNNNNNNNNNNNNNNNNNNNNNNNNNNNNNNNNNNNNNNNNNNNN
NNNNNNNNNNNNNNNNNNNNNNNNNNNNNNNNNNNNNNNNNNNNNNNNNNNNNNNNNNNNNNNNNNNNNNNNNNNNNNN
NNNNNNNNNNNNNNNNNNNNNNNNNNNNNNNNNNNNNNNNNNNNNNNNNNNNNNNNNNNNNNNNNNNNNNNNNNNNNNN
NNNNNNNNNNNNNNNNNNNNNNNNNNNNNNNNNNNNNNNNNNNNNNNNNNNNNNNNNNNNNNNNNNNNNNNNNNNNNNN
NNNNNNNNNNNNNNNNNNNNNNNNNNNNNNNNNNNNNNNNNNNNNNNNNNNNNNNNNNNNNNNNNNNNNNNNNNNNNNN
NNNNNNNNNNNNNNNNNNNNNNNNNNNNNNNNNNNNNNNNNNNNNNNNNNNNNNNNNNNNNNNNNNNNNNNNNNNNNNN
NNNNNNNNNNNNNNNNNNNNNNNNNNNNNNNNNNNNNNNNNNNNNNNNNNNNNNNNNNNNNNNNNNNNNNNNNNNNNNN
NNNNNNNNNNNNNNNNNNNNNNNNNNNNNNNNNNNNNNNNNNNNNNNNNNNNNNNNNNNNNNNNNNNNNNNNNNNNNNN
NNNNNNNNNNNNNNNNNNNNNNNNNNNNNNNNNNNNNNNNNNNNNNNNNNNNNNNNNNNNNNNNNNNNNNNNNNNNNNN
NNNNNNNNNNNNNNNNNNNNNNNNNNNNNNNNNNNNNNNNNNNNNNNNNNNNNNNNNNNNNNNNNNNNNNNNNNNNNNN
NNNNNNNNNNNNNNNNNNNNNNNNNNNNNNNNNNNNNNNNNNNNNNNNNNNNNNNNNNNNNNNNNNNNNNNNNNNNNNN
NNNNNNNNNNNNNNNNNNNNNNNNNNNNNNNNNNNNNNNNNNNNNNNNNNNNNNNNNNNNNNNNNNNNNNNNNNNNNNN
NNNNNNNNNNNNNNNNNNNNNNNNNNNNNNNNNNNNNNNNNNNNNNNNNNNNNNNNNNNNNNNNNNNNNNNNNNNNNNN
NNNNNNNNNNNNNNNNNNNNNNNNNNNNNNNNNNNNNNNNNNNNNNNNNNNNNNNNNNNNNNNNNNNNNNNNNNNNNNN
NNNNNNNNNNNNNNNNNNNNNNNNNNNNNNNNNNNNNNNNNNNNNNNNNNNNNNNNNNNNNNNNNNNNNNNNNNNNNNN
NNNNNNNNNNNNNNNNNNNNNNNNNNNNNNNNNNNNNNNNNNNNNNNNNNNNNNNNNNNNNNNNNNNNNNNNNNNNNNN
NNNNNNNNNNNNNNNNNNNNNNNNNNNNNNNNNNNNNNNNNNNNNNNNNNNNNNNNNNNNNNNNNNNNNNNNNNNNNNN
NNNNNNNNNNNNNNNNNNNNNNNNNNNNNNNNNNNNNNNNNNNNNNNNNNNNNNNNNNNNNNNNNNNNNNNNNNNNNNN
NNNNNNNNNNNNNNNNNNNNNNNNNNNNNNNNNNNNNNNNNNNNNNNNNNNNNNNNNNNNNNNNNNNNNNNNNNNNNNN
NNNNNNNNNNNNNNNNNNNNNNNNNNNNNNNNNNNNNNNNNNNNNNNNNNNNNNNNNNNNNNNNNNNNNNNNNNNNNNN
NNNNNNNNNNNNNNNNNNNNNNNNNNNNNNNNNNNNNNNNNNNNNNNNNNNNNNNNNNNNNNNNNNNNNNNNNNNNNNN
NNNNNNNNNNNNNNNNNNNNNNNNNNNNNNNNNNNNNNNNNNNNNNNNNNNNNNNNNNNNNNNNNNNNNNNNNNNNNNN
NNNNNNNNNNNNNNNNNNNNNNNNNNNNNNNNNNNNNNNNNNNNNNNNNNNNNNNNNNNNNNNNNNNNNNNNNNNNNNN
NNNNNNNNNNNNNNNNNNNNNNNNNNNNNNNNNNNNNNNNNNNNNNNNNNNNNNNNNNNNNNNNNNNNNNNNNNNNNNN
NNNNNNNNNNNNNNNNNNNNNNNNNNNNNNNNNNNNNNNNNNNNNNNNNNNNNNNNNNNNNNNNNNNNNNNNNNNNNNN
NNNNNNNNNNNNNNNNNNNNNNNNNNNNNNNNNNNNNNNNNNNNNNNNNNNNNNNNNNNNNNNNNNNNNNNNNNNNNNN
NNNNNNNNNNNNNNNNNNNNNNNNNNNNNNNNNNNNNNNNNNNNNNNNNNNNNNNNNNNNNNNNNNNNNNNNNNNNNNN
NNNNNNNNNNNNNNNNNNNNNNNNNNNNNNNNNNNNNNNNNNNNNNNNNNNNNNNNNNNNNNNNNNNNNNNNNNNNNNN
NNNNNNNNNNNNNNNNNNNNNNNNNNNNNNNNNNNNNNNNNNNNNNNNNNNNNNNNNNNNNNNNNNNNNNNNNNNNNNN
NNNNNNNNNNNNNNNNNNNNNNNNNNNNNNNNNNNNNNNNNNNNNNNNNNNNNNNNNNNNNNNNNNNNNNNNNNNNNNN
NNNNNNNNNNNNNNNNNNNNNNNNNNNNNNNNNNNNNNNNNNNNNNNNNNNNNNNNNNNNNNNNNNNNNNNNNNNNNNN
NNNNNNNNNNNNNNNNNNNNNNNNNNNNNNNNNNNNNNNNNNNNNNNNNNNNNNNNNNNNNNNNNNNNNNNNNNNNNNN
NNNNNNNNNNNNNNNNNNNNNNNNNNNNNNNNNNNNNNNNNNNNNNNNNNNNNNNNNNNNNNNNNNNNNNNNNNNNNNN
NNNNNNNNNNNNNNNNNNNNNNNNNNNNNNNNNNNNNNNNNNNNNNNNNNNNNNNNNNNNNNNNNNNNNNNNNNNNNNN
NNNNNNNNNNNNNNNNNNNNNNNNNNNNNNNNNNNNNNNNNNNNNNNNNNNNNNNNNNNNNNNNNNNNNNNNNNNNNNN
NNNNNNNNNNNNNNNNNNNNNNNNNNNNNNNNNNNNNNNNNNNNNNNNNNNNNNNNNNNNNNNNNNNNNNNNNNNNNNN
NNNNNNNNNNNNNNNNNNNNNNNNNNNNNNNNNNNNNNNNNNNNNNNNNNNNNNNNNNNNNNNNNNNNNNNNNNNNNNN
NNNNNNNNNNNNNNNNNNNNNNNNNNNNNNNNNNNNNNNNNNNNNNNNNNNNNNNNNNNNNNNNNNNNNNNNNNNNNNN
NNNNNNNNNNNNNNNNNNNNNNNNNNNNNNNNNNNNNNNNNNNNNNNNNNNNNNNNNNNNNNNNNNNNNNNNNNNNNNN
NNNNNNNNNNNNNNNNNNNNNNNNNNNNNNNNNNNNNNNNNNNNNNNNNNNNNNNNNNNNNNNNNNNNNNNNNNNNNNN

NNNNNNNNNNNNNNNNNNNNNNNNNNNNNNNNNNNNNNNNNNNNNNNNNNNNNNNNNNNNNNNNNNNNNN
NNNNNNNNNNNNNNNNNNNNNNNNNNNNNNNNNNNNNNNNNNNNNNNNNNNNNNNNNNNNNNNNNNNNNN
NNNNNNNNNNNNNNNNNNNNNNNNNNNNNNNNNNNNNNNNNNNNNNNNNNNNNNNNNNNNNNNNNNNNNN
NNNNNNNNNNNNNNNNNNNNNNNNNNNNNNNNNNNNNNNNNNNNNNNNNNNNNNNNNNNNNNNNNNNNNN
NNNNNNNNNNNNNNNNNNNNNNNNNNNNNNNNNNNNNNNNNNNNNNNNNNNNNNNNNNNNNNNNNNNNNN
NNNNNNNNNNNNNNNNNNNNNNNNNNNNNNNNNNNNNNNNNNNNNNNNNNNNNNNNNNNNNNNNNNNNNN
NNNNNNNNNNNNNNNNNNNNNNNNNNNNNNNNNNNNNNNNNNNNNNNNNNNNNNNNNNNNNNNNNNNNNN
NNNNNNNNNNNNNNNNNNNNNNNNNNNNNNNNNNNNNNNNNNNNNNNNNNNNNNNNNNNNNNNNNNNNNN
NNNNNNNNNNNNNNNNNNNNNNNNNNNNNNNNNNNNNNNNNNNNNNNNNNNNNNNNNNNNNNNNNNNNNN
NNNNNNNNNNNNNNNNNNNNNNNNNNNNNNNNNNNNNNNNNNNNNNNNNNNNNNNNNNNNNNNNNNNNNN
NNNNNNNNNNNNNNNNNNNNNNNNNNNNNNNNNNNNNNNNNNNNNNNNNNNNNNNNNNNNNNNNNNNNNN
NNNNNNNNNNNNNNNNNNNNNNNNNNNNNNNNNNNNNNNNNNNNNNNNNNNNNNNNNNNNNNNNNNNNNN
NNNNNNNNNNNNNNNNNNNNNNNNNNNNNNNNNNNNNNNNNNNNNNNNNNNNNNNNNNNNNNNNNNNNNN
NNNNNNNNNNNNNNNNNNNNNNNNNNNNNNNNNNNNNNNNNNNNNNNNNNNNNNNNNNNNNNNNNNNNNN
NNNNNNNNNNNNNNNNNNNNNNNNNNNNNNNNNNNNNNNNNNNNNNNNNNNNNNNNNNNNNNNNNNNNNN
NNNNNNNNNNNNNNNNNNNNNNNNNNNNNNNNNNNNNNNNNNNNNNNNNNNNNNNNNNNNNNNNNNNNNN
NNNNNNNNNNNNNNNNNNNNNNNNNNNNNNNNNNNNNNNNNNNNNNNNNNNNNNNNNNNNNNNNNNNNNN
NNNNNNNNNNNNNNNNNNNNNNNNNNNNNNNNNNNNNNNNNNNNNNNNNNNNNNNNNNNNNNNNNNNNNN
NNNNNNNNNNNNNNNNNNNNNNNNNNNNNNNNNNNNNNNNNNNNNNNNNNNNNNNNNNNNNNNNNNNNNN
NNNNNNNNNNNNNNNNNNNNNNNNNNNNNNNNNNNNNNNNNNNNNNNNNNNNNNNNNNNNNNNNNNNNNN
NNNNNNNNNNNNNNNNNNNNNNNNNNNNNNNNNNNNNNNNNNNNNNNNNNNNNNNNNNNNNNNNNNNNNN
NNNNNNNNNNNNNNNNNNNNNNNNNNNNNNNNNNNNNNNNNNNNNNNNNNNNNNNNNNNNNNNNNNNNNN
NNNNNNNNNNNNNNNNNNNNNNNNNNNNNNNNNNNNNNNNNNNNNNNNNNNNNNNNNNNNNNNNNNNNNN
NNNNNNNNNNNNNNNNNNNNNNNNNNNNNNNNNNNNNNNNNNNNNNNNNNNNNNNNNNNNNNNNNNNNNN
NNNNNNNNNNNNNNNNNNNNNNNNNNNNNNNNNNNNNNNNNNNNNNNNNNNNNNNNNNNNNNNNNNNNNN
NNNNNNNNNNNNNNNNNNNNNNNNNNNNNNNNNNNNNNNNNNNNNNNNNNNNNNNNNNNNNNNNNNNNNN
NNNNNNNNNNNNNNNNNNNNNNNNNNNNNNNNNNNNNNNNNNNNNNNNNNNNNNNNNNNNNNNNNNNNNN
NNNNNNNNNNNNNNNNNNNNNNNNNNNNNNNNNNNNNNNNNNNNNNNNNNNNNNNNNNNNNNNNNNNNNN
NNNNNNNNNNNNNNNNNNNNNNNNNNNNNNNNNNNNNNNNNNNNNNNNNNNNNNNNNNNNNNNNNNNNNN
NNNNNNNNNNNNNNNNNNNNNNNNNNNNNNNNNNNNNNNNNNNNNNNNNNNNNNNNNNNNNNNNNNNNNN
NNNNNNNNNNNNNNNNNNNNNNNNNNNNNNNNNNNNNNNNNNNNNNNNNNNNNNNNNNNNNNNNNNNNNN
NNNNNNNNNNNNNNNNNNNNNNNNNNNNNNNNNNNNNNNNNNNNNNNNNNNNNNNNNNNNNNNNNNNNNN
NNNNNNNNNNNNNNNNNNNNNNNNNNNNNNNNNNNNNNNNNNNNNNNNNNNNNNNNNNNNNNNNNNNNNN
NNNNNNNNNNNNNNNNNNNNNNNNNNNNNNNNNNNNNNNNNNNNNNNNNNNNNNNNNNNNNNNNNNNNNN
NNNNNNNNNNNNNNNNNNNNNNNNNNNNNNNNNNNNNNNNNNNNNNNNNNNNNNNNNNNNNNNNNNNNNN
NNNNNNNNNNNNNNNNNNNNNNNNNNNNNNNNNNNNNNNNNNNNNNNNNNNNNNNNNNNNNNNNNNNNNN
NNNNNNNNNNNNNNNNNNNNNNNNNNNNNNNNNNNNNNNNNNNNNNNNNNNNNNNNNNNNNNNNNNNNNN
NNNNNNNNNNNNNNNNNNNNNNNNNNNNNNNNNNNNNNNNNNNNNNNNNNNNNNNNNNNNNNNNNNNNNN
NNNNNNNNNNNNNNNNNNNNNNNNNNNNNNNNNNNNNNNNNNNNNNNNNNNNNNNNNNNNNNNNNNNNNN
NNNNNNNNNNNNNNNNNNNNNNNNNNNNNNNNNNNNNNNNNNNNNNNNNNNNNNNNNNNNNNNNNNNNNN
NNNNNNNNNNNNNNNNNNNNNNNNNNNNNNNNNNNNNNNNNNNNNNNNNNNNNNNNNNNNNNNNNNNNNN
NNNNNNNNNNNNNNNNNNNNNNNNNNNNNNNNNNNNNNNNNNNNNNNNNNNNNNNNNNNNNNNNNNNNNN
NNNNNNNNNNNNNNNNNNNNNNNNNNNNNNNNNNNNNNNNNNNNNNNNNNNNNNNNNNNNNNNNNNNNNN
NNNNNNNNNNNNNNNNNNNNNNNNNNNNNNNNNNNNNNNNNNNNNNNNNNNNNNNNNNNNNNNNNNNNNN
NNNNNNNNNNNNNNNNNNNNNNNNNNNNNNNNNNNNNNNNNNNNNNNNNNNNNNNNNNNNNNNNNNNNNN
NNNNNNNNNNNNNNNNNNNNNNNNNNNNNNNNNNNNNNNNNNNNNNNNNNNNNNNNNNNNNNNNNNNNNN
NNNNNNNNNNNNNNNNNNNNNNNNNNNNNNNNNNNNNNNNNNNNNNNNNNNNNNNNNNNNNNNNNNNNNN
NNNNNNNNNNNNNNNNNNNNNNNNNNNNNNNNNNNNNNNNNNNNNNNNNNNNNNNNNNNNNNNNNNNNNN
NNNNNNNNNNNNNNNNNNNNNNNNNNNNNNNNNNNNNNNNNNNNNNNNNNNNNNNNNNNNNNNNNNNNNN
NNNNNNNNNNNNNNNNNNNNNNNNNNNNNNNNNNNNNNNNNNNNNNNNNNNNNNNNNNNNNNNNNNNNNN
NNNNNNNNNNNNNNNNNNNNNNNNNNNNNNNNNNNNNNNNNNNNNNNNNNNNNNNNNNNNNNNNNNNNNN
NNNNNNNNNNNNNNNNNNNNNNNNNNNNNNNNNNNNNNNNNNNNNNNNNNNNNNNNNNNNNNNNNNNNNN
NNNNNNNNNNNNNNNNNNNNNNNNNNNNNNNNNNNNNNNNNNNNNNNNNNNNNNNNNNNNNNNNNNNNNN
NNNNNNNNNNNNNNNNNNNNNNNNNNNNNNNNNNNNNNNNNNNNNNNNNNNNNNNNNNNNNNNNNNNNNN
NNNNNNNNNNNNNNNNNNNNNNNNNNNNNNNNNNNNNNNNNNNNNNNNNNNNNNNNNNNNNNNNNNNNNN
NNNNNNNNNNNNNNNNNNNNNNNNNNNNNNNNNNNNNNNNNNNNNNNNNNNNNNNNNNNNNNNNNNNNNN
NNNNNNNNNNNNNNNNNNNNNNNNNNNNNNNNNNNNNNNNNNNNNNNNNNNNNNNNNNNNNNNNNNNNNN
NNNNNNNNNNNNNNNNNNNNNNNNNNNNNNNNNNNNNNNNNNNNNNNNNNNNNNNNNNNNNNNNNNNNNN
NNNNNNNNNNNNNNNNNNNNNNNNNNNNNNNNNNNNNNNNNNNNNNNNNNNNNNNNNNNNNNNNNNNNNN
NNNNNNNNNNNNNNNNNNNNNNNNNNNNNNNNNNNNNNNNNNNNNNNNNNNNNNNNNNNNNNNNNNNNNN

NNNNNNNNNNNNNNNNNNNNNNNNNNNNNNNNNNNNNNNNNNNNNNNNNNNNNNNNNNNNNNNNNNNNNN
NNNNNNNNNNNNNNNNNNNNNNNNNNNNNNNNNNNNNNNNNNNNNNNNNNNNNNNNNNNNNNNNNNNNNN
NNNNNNNNNNNNNNNNNNNNNNNNNNNNNNNNNNNNNNNNNNNNNNNNNNNNNNNNNNNNNNNNNNNNNN
NNNNNNNNNNNNNNNNNNNNNNNNNNNNNNNNNNNNNNNNNNNNNNNNNNNNNNNNNNNNNNNNNNNNNN
NNNNNNNNNNNNNNNNNNNNNNNNNNNNNNNNNNNNNNNNNNNNNNNNNNNNNNNNNNNNNNNNNNNNNN
NNNNNNNNNNNNNNNNNNNNNNNNNNNNNNNNNNNNNNNNNNNNNNNNNNNNNNNNNNNNNNNNNNNNNN
NNNNNNNNNNNNNNNNNNNNNNNNNNNNNNNNNNNNNNNNNNNNNNNNNNNNNNNNNNNNNNNNNNNNNN
NNNNNNNNNNNNNNNNNNNNNNNNNNNNNNNNNNNNNNNNNNNNNNNNNNNNNNNNNNNNNNNNNNNNNN
NNNNNNNNNNNNNNNNNNNNNNNNNNNNNNNNNNNNNNNNNNNNNNNNNNNNNNNNNNNNNNNNNNNNNN
NNNNNNNNNNNNNNNNNNNNNNNNNNNNNNNNNNNNNNNNNNNNNNNNNNNNNNNNNNNNNNNNNNNNNN
NNNNNNNNNNNNNNNNNNNNNNNNNNNNNNNNNNNNNNNNNNNNNNNNNNNNNNNNNNNNNNNNNNNNNN
NNNNNNNNNNNNNNNNNNNNNNNNNNNNNNNNNNNNNNNNNNNNNNNNNNNNNNNNNNNNNNNNNNNNNN
NNNNNNNNNNNNNNNNNNNNNNNNNNNNNNNNNNNNNNNNNNNNNNNNNNNNNNNNNNNNNNNNNNNNNN
NNNNNNNNNNNNNNNNNNNNNNNNNNNNNNNNNNNNNNNNNNNNNNNNNNNNNNNNNNNNNNNNNNNNNN
NNNNNNNNNNNNNNNNNNNNNNNNNNNNNNNNNNNNNNNNNNNNNNNNNNNNNNNNNNNNNNNNNNNNNN
NNNNNNNNNNNNNNNNNNNNNNNNNNNNNNNNNNNNNNNNNNNNNNNNNNNNNNNNNNNNNNNNNNNNNN
NNNNNNNNNNNNNNNNNNNNNNNNNNNNNNNNNNNNNNNNNNNNNNNNNNNNNNNNNNNNNNNNNNNNNN
NNNNNNNNNNNNNNNNNNNNNNNNNNNNNNNNNNNNNNNNNNNNNNNNNNNNNNNNNNNNNNNNNNNNNN
NNNNNNNNNNNNNNNNNNNNNNNNNNNNNNNNNNNNNNNNNNNNNNNNNNNNNNNNNNNNNNNNNNNNNN
NNNNNNNNNNNNNNNNNNNNNNNNNNNNNNNNNNNNNNNNNNNNNNNNNNNNNNNNNNNNNNNNNNNNNN
NNNNNNNNNNNNNNNNNNNNNNNNNNNNNNNNNNNNNNNNNNNNNNNNNNNNNNNNNNNNNNNNNNNNNN
NNNNNNNNNNNNNNNNNNNNNNNNNNNNNNNNNNNNNNNNNNNNNNNNNNNNNNNNNNNNNNNNNNNNNN
NNNNNNNNNNNNNNNNNNNNNNNNNNNNNNNNNNNNNNNNNNNNNNNNNNNNNNNNNNNNNNNNNNNNNN
NNNNNNNNNNNNNNNNNNNNNNNNNNNNNNNNNNNNNATTCATGTTTCGGCTTTCTGCATGTTTACTTTG
GTTATTTTTGTAGATAGGGCCCGCTTAGGGATTTTAAACTTAACTCGTATTTCTACATGTTACTTTAACT
ATCTTTCATAAATACATTTGCTAGATTTCTTTGCATTTATTTACACCGGATTTTATGACTTAAACACTGA
TCCTAGATTCAGTAACCACTTCGATTCAGCATAAAGAGTCGGGTCGTTACAAATTCCATTCTTGTAGTTA
AGAAACATGACTTAATTTCAAAAATAATAAAATTATTAACCAACTAATTTTTTTTGTTTATCTTTTAATT
TACTATTTTTTTTTTTTTGGAAAAGATACATTAGGTCAGTAGGTGCATCCAAACATGAACATCCCCTAGC
ACCATCATCATCCCCACTTCATTAATGATAAGAGAAGGAATACACGAAGCCAAGAGAAAGAAAACAAAAG
GGACTAGAGATAAGCCCAAACAACTCAAAAGTACATATATTAATGGAAAGCATTTAAATGAAGAGCTATG
GTGCTAGCAGTGTACAAAGGCCAGTAAGCGCGCGAATATCTTTCTAAACTGCAGTAGGTGATTTTTCTTT
GTTGTTGAAGATTCTACTATTTCCTGCTTTCCAAATAGACCAAAGAGATACAACGACCAAGTTGAAGCGA
ATAATGTCCTTTGATTTTTGGTTCGAAAGACCAATGCATTTACAAAGAGCAACCGTGCGTTGTAAATCGA
TGTTCCAATTCAAAGTCGATTTAAGAGATCCCCATATGTAATGACTAAAAGAGCAAGAGGTAAACAGATG
GTTTATATATTCTTCATCAGCTTCCTTGCAAGGAATACACCAAGATGGTTTTAGATTCCAATTTGGAAGT
TTTTTTTTTAAGAATCTCAGTTGTATTGATGCATTCATAATGTCGCCACGTACCCGAGACGACGCGGAGC
GGCCGACATCCTTAAAAAGAGTTTTATTTTAAAAACGAAAAAAGAGGAGTCGCCATCAATCTTTTTTACG
GTGTGATTGGACACCGAAATAAAGTGAATCATTTTAAATAAAATAAAAATTGGTCTACGAAAAAATTAGA
GTTGAGTTCGGGAGTCATTTGTGTACGGGGAAGGTGTTAGCACCCCGTAACACCCGTTTTAGAAAACGGT
GGCCAAATTTAATGTCTTGAATAAAATTCATTTGTTTTTAAAGAACCATGTCTTATTTCATTGCTCACCA
CTCCAATATTTGGAGCAATGATCTCCCATAAAATAAGTGGGATTTACCCATTAGTTAGACTATATTGAGG
AAAAAATTCTCACCTTAAGAGAATGAGAAATTATTACAATTTCTCAAATTCTATNNNNNNNNNNNNNNNN
NNNNNNNNNNNNNNNNNNNNNNNNNNNNNNNNNNNNNNNNNNNNNNNNNNNNNNNNNNNNNNNNNNNNNN
NNNNNNNNNNNNNNNNNNNNNNNNNNNNTCTATTTAATTTTGAGTTTTAAAATAAATAATTTTAAAGAAA
GAAAAAAAAAGATTCTAAATGGTTTAAAGAGAAAAATTTAAAAAAAGATTTCAAAATTAAATATAATTAT
GGTTTAAAATATTTTAAAATTTTCAAGAAATTTTTAATGAAAGTCAAATAGAAATAACAAATATACCACA
CAATAAATTAGGCAAATAATTTAAGATATCAATGTATAAATATAACGATAGTAATAAAAAATAAATTAAA
AGGAAAAGAAAAGAAAAGATACATAATAACGATAAATAAAATAAGAGAAAAAATGGAAAAATAACTAATA
AAATAATAAACAAATAAACACGTAAAAAAAGAAATTAAATATATAAGTTAAGAAAAGAGGACATAGTAAG
TTAAAAAGTAAAAATATTTATGAAATGATATAATAATAAAAATAAAAAAAAGTAAAAAAAATCATAAATA
TAAAGAAAAAAAGGAAAAAAAATGATAAATATAAAAAAAGTAATAAATCATAAATATAAAAAAAAAACTA
AATAAAGAAATAAAGAAAAGTTATATATAAAATAAACTAAGATTAAAAAAAACAAACAGAAAGGTAGAAA
ATAATATATATATATATATATAATAACAATAATGATAATAATAATAATAATAATTATTATTATTATTAAA
ATATGATGATAAATAGGTAAATGAATAAATTAGAATAAAAGGAGAAAAAAACATAGGGGTCACCCTAAAA
GAGAAAAAAAAGTAATATATATATAATAAATATGGAGATGATAATAATAATAATATAAGATTCTTTTCTT
ATAACTTTTTCTTTTTCTCTATTTTTTAGAGAGAAGAAAAAAGTTAAGACTTTTTTTTACCATAATGATA
AAAAGGTAGCAAAAAGAAAAAAGAAGTTTTCCTTATATATTTTTTTCTATCTCTCTATGCTTAAAAAGAG
AAGAACAAAAATAAGGATTCAATCCCGAAGAAAATTACTTGTTGTATGAGGTACGGATCATTGGGAGTAA
GCCCTACCTAATACAACCTCCAACTAAGTTTTTCCTTCCTTATGGGATTAGAATTAGGGTAAAAAAAAAG

ACGATATGAAGATAAAAATAAAAATAATAAAGCGGAAAAGCTAATGACCCACCTTTATGCAGAGGAATAC
GTTTCTTCAAATTCTCTATACACACTTTTTTTTTCGTAGAGATTCTCTCTAAAACAATTTTTTCTCAAGA
CTTTCTCTAAAACAATTTCTTCTCCCCCCTTTCCAAATGACAAAGGATCCTATTTATAGAGTCGGGGTGT
GCCACGAATTAGGAAAATTTAATAAGTATAAAATCAAATTAGATAGAGCCGGGGTGTGCCACAAATTAGG
AAAATTTAATAAGTATAAAATCAAATTAGATATTATGTAATTTTATTAATTTTCTTTTTTCTAAAATAGT
AAAAATATGATATTGAAAGATTTTGTCCAAAATAAATTTTTTGCTTTTTTCCTAAAGTGATAAAGATAAG
TCAAATATTGTATTAAAATTTTTTGTATAAAATAAATTATCTTGCTTTTCCTAATATGATAAATCAAAGT
ATATTTTGTAATAAATAAATTAATTTGTTTTCTTTTTCCTAAAGTGATAAGTCAAAAAAAAAAAAACATT
AATGACTTTATTTTCCATTTTAATAAAAAGATAAGAAAAAACATAAGACTTCATATTATTACAAAAAGAT
TTTACTTTTTTCTTTTTTTAACAAAATAAATTAATGAAATATCTTAACCGTTGCTACAATTAATAATTTA
AAGAATAAAAAAAATGTAGGATAAAATTAGGTGGCTACACATAAAAAAGAGACCAGACGAAGAATTTACA
CTTTTTTGATATATTAGCCTTCCAAAGATTGTTGTATATAGTTGGGTCAATATTAATGTTTTCTCTTTGA
ACTCCTTTCATGAATGGCAGCTTTTATAGAGGCAACAGAAAAAAATTCCATTAAAATTTAAATTCCAAAGT
GGAATGTCTTGCCTGCGATTTTGAAGTAGAGTAGGGAGAGATACAATCAATTCATACCATTGTTGTAATT
CAAAATCTTTGGGAGGTCTTCTAGGAAATAAATTTCAATTAGCATCTACTGCATTCCACATGTTTTTAAC
GGAGCTCTCCTTTTCAGAAGTAAGCAAAAAGCCTTGTTGGTAGAGATTTAGAGAATTGTTTTTATGCTAG
TGACTATGCCAAAAGGAAAGGTTTTCACCATTATTTATCTTCCATTTAATTTGGTTCATAAACTAATCGA
CCTCTTTGATGATAGAAAACCATAGAGATCTTTGGCTGCTGTATTTGCCTTTTGAGGGGATATCTCCTAA
AAAAACTTGTTCATATTTTGCTTGTATAATACGCTTCTAGAGAGGATCAATTTCTGTAGTGAATCTCCAT
AGCCATTTGTAGAGTAGAGCAAAGTTTGTGTCTTTAACTCCATTAATACTCAGCCCACCCTTTTCTTTAG
GACAAGAGATTACAACCCAGTTGTTAAGGTGGGTATTTTTCTTATCACTAGAATTTTTCCAAAGGAAGCT
TCTCCATTATTTCTCAATGTTTTTGGAAACTGAATTTAGGGCTTTGAATTTAGAAAGCTGATATGTTGGA
AGGCTAGCAAGGGTGAAATTAATCAATGTAATTTTACCTCCTTTAGACAGATATGAATATTTCCAGTTGC
TCAGCTTTTTTTGAATTTTTTAGAAATGTCTTCCCAAAATTTCTTTGACAAAGGTTTAACCGCCAAGAGG
AACACTCAAGTCGTTTATCGGCAAGATTTGAGAAGAAAAACCCCATTTGGCAGCCACCTCCAAAGTTCTT
GTAGAGTCCACATTAATCGGAGAAATCGTAGATTTTTGGAGGTTTATGTTAAGACCGGATGCCAGCACAA
AGAGCTTTAAAGCAAACTTTAGATTCTCAATAGATTTGTCATGATCTTCAACAAAGAGTAAGATATCATC
CGCAAACAACAAATGAGTGAGATTAAGGATCTCACCTTTTACTATTGTTTTACTGAATCAAAATTTGAGA
AACTACAAAAAATAAATAAATAAATCAATCAATCAAAGGGTTGTTTTCAATTTTAGAATTAGAATTTAAC
TAGAAATTTCAATTTTATTTAACGTTAAAAGTTTGGAAGGGAAATAAGCATTAAATTTAATAAATATTAT
AAAAAATAATTTTAATAAAAAACAGAAAACAAAAAATTAACGAATGAAGACTGAATTTTTTAAGCACAGG
ATAACTTAAAGACTTATAAGATTTTTTTTTTTTTTAAAAAAAATGATTTTCTATGGATGGATAAACCTAC
TTCCTAATATATTAAATTTGTTTATTGGATATACATTAAAAAATTCATAACACTATTATCATAATTCTTC
CCCAAACGAATATTATCATAACACTACCTATCATAATCTTTTCCCCAAACATGTACACCATTATAACAGT
ACCAATAATAATAGATCTTCTCTCAAACACATATGATCATAACACTAGTTTTATCATAATCGTAAGATTA
TCATAACTCTTATCCCTGTAATTCTCTCAATCCTCCGAACGCCCTCGAATGTTTTTGCATCTTGCCTAAT
TATTGCAACAAGATTGTTAATTGAGATGCTAATGATGAAAATTACAAACAAACAATTATTAATTGTAATA
AAAGTGTGTTGGAAATTACAAACAAATAATTATTGATTGTAATAAAAGTGAGTTTGAAATTACAAACAGA
CAATGGAAGCTTTTTTTTAATATATATAAAAAAAGTAGTCTTTCCCTTTGAATCAGTACATAGGGAAAGT
AAATGAAGAACTACAATTGACGTAGACTTTATTTAAAATGTTATTAATTTCCAAAATAGAAATTGAGAAA
TAAAAACTTTTCTAGAAGGCATTTTATTTTATAAAATATGTTCAACTAAAAATAGAAACGTGTTTGGGAG
CATCTCAATTAAATATTCAGCTTATTTTTTTCTTATTTCTTTTTTAAACTTTAGGTTAATAACTTACGTG
TTTGTTTATCCTTTTGATGATTATATGTTTATTTGGTTCTAATAATGTCTAATAAGTCTATAATTTTTTA
TTTACATATGATTAATTATAATCCCCTCTATTTAAGAGGGAGACATGAATGAATTGAAATCACACCTGAA
TGAGAGAGATCATGAAGACATGAAAGTAATATTAAGAAGTCTTAAAAAATTAAAAGTTACTATTCACACC
TTATCTTCCTTTTAGATGACCTGATCGTAGAAACTTTAGAGTTAAGCATACTTAGCTCGAAACAATTCTG
TGTTTGGTGGCCTTCTACAAACTTTCTTAGGATGCATGTGAGTGAAAACGAATTATGCTGAGAAGACCCA
TATTGGTTTGTAGGGCAATTTTCACTCTAATAAGACGAGAATATTGGAGATCTAAGTTCCGAATCTTGGG
TTGGATCGTTACACTAATGATCCTTAAACTTTTAATTTTGTGTGTAACGAGTCCTTGACAAATTCAAAAT
TTTTAAAAATTTGAGCTTTTTGCGAAATAAGATACTAGTGGGTTTCCATACATATTGTATTATGGCAAGG
GTGTTTTTGAAAATTTTTTCATATAATTTGATTACAATAAAAATAAAAACTTTAGAAAGTTGAAAGGGTA
TTTTTTAGAAACAAAATAAAAAGTATATATGTGCATTCTATAATTTAACCTATTTTTAGGTCTTTTTGGA
AAACTGATTCTCTAATTGGTTAAAACAAAAATATTGTCAATTTTAACATAGCCCAAAGTGGATGAAGAAA
CTCATTATTTACAATTTCGTATATAAGAACCTATTTAGATTGACTTAAAGAAAACAATATTTTTAAAGAA
TTTATTTGTAAACTCATTTGATAAAAGATTATTTAAAATAAATTTCAAAAGCTATTTTGAGTAGACGTGG
ACTATTTTTATTTTTTCCCAAAACACTTATTTTTTAAGAATGTATTCTAAACTTTAAAAAAATATAATAA
AATAAAATTAACACGAAAGAAAAATAATAAAAACCGACAAATAATAATTTAATGCCCTAGGTCCAGGTTT
TAGAATTCGAATTCTCGACATTCCCCTGCATCCCCTACGACCTGACAACTAGGGGTGTACATAAGCCGGG
TTGGTCGGGTTACCGGTTTTTTTGAACACCCCTACTGGCAACATCACTGTTACTTGTCTTAAAGGCTTAT
TAGAGTGACATTAGTTCTTTTAGCATGCTTTGTCCTCACTCACATGCATCGGAGGAAAATTCCTTGGAGG

TCACCCAACATAAGATTGCTCCAAGGTAAGCACGCTCAACTTTGGAGTTTTGATGATCGAGCCACCGAAA
AGGAAGGTGCACCTTGTTGATATAGGTAGTAACTTTTAATTTTTTTAAGTTTCTCTTAACATTAATTACT
TTCATGTCCTCAGAATTCCCTCAAATATGATACTAGTTCATTCATGTCTCCCTCCTCAACTTGGGATGTT
ACAATTAATTAGGAAAAAACCATAAACCATAATTTTCAATAACATTTATGTATCCAACAATCGGTGGTTG
GATCCTTTTTTTTAATTGAATACAATTAATTGTTTTTGAAATAATATATATATATATATATATATGTCTG
TGTGAATTTGAAATAATATATATATGTATATATATGATTCTCGATTTGAAATAATATAAATGGTGTGTGG
ATATTATGGATCATATGAGTGCGTGTGTGTAGATATTATCGATCATATGGGTGTACCCAGACATCTCTAC
TAGGTGTATACCCTTAGCACCTATATAATTCTCGCTATATTAATAATAAGAGAAAACAATATAGGAAGCC
AAGAGAAAAGGATATAGGGATAAACCCATGAAAAGTTATACAAAAGTGCTAATATTTAAAGCAATAAAGC
TACCACTGTAATTTTGAAAAAAAATTGGATCACTTTCAACTACTTTAATGTACATATAAAAAGAAAAAAA
AGAACATTTTGATCTTTTTAACAAGTGCAATTAATTATATATTGTAGAGACTTAGATTAAACTTAAACTA
AACATTAAAAAGAAAATAACAAAACAAAACAAAAATAAAGAGGTGGCATGTGCATATATATATGGCTGTT
GACCCACATGGCCAAATATACTCGAATATTTTTGTTATTAATTTATTCATGACGTGCCAATGATCATTTT
GATTAAATAATATAGTTTTATGAAGAGGAAAATAATTAAATGAATAAATGGGATAAGGCAAAAAAAAAAA
AAAAATTTTAATAACATTTTAAGATCATATGCGTTTTAAATTCATTATATTTAATTTATAATAATGTACG
GAGGGGTCCATTGTGTATGGTCTGTATATAATATTAGTTGTAGGCTGTAATCATGGATAAGATGTTCAAG
ATTTTTTTTGTTAATTTTTTAATTATGACAAATATTGATGATACTTAAAACAACGTGAATCACATTAATA
AATTTCAATAAATAATAATAATAAAAAAAAGGGCCACTTTGATAGGATTTTTTGATTTTGATACATATGA
AAGAAAAACTTTTGTAAATGTAACAAACCATCAAACTATTTACGTTTCGTATAACAAAGCTCATAAAAGT
GAGTCATTTTTTTAAATATTTCAGGTTTGGACTTCCATCTTCTTTTTTTTTTTCCTTGCGATTCGTCTTC
TTCCTACTATTTCTTTTATCGTCCTCCTATTACAATTTCGTCTTTCTTCTTTCCTATTTTTATTTCTGCA
ATTTCTTTCTATCATCTTTCTTATTTTTCAATTATTTATTTACGTTGTTTATCTTTCCATCTTTTTTCTT
CTTTTCCTCCTTCTTTTTTTTTTTTTTTTTCACTTTTTCATTATGTTTCTACTTTTTCTCTTCTTTTTTT
TCACTGCGATTTATTTCCATCATCTTTTTTTTTTTTTGACGTCGTGTACAAAATATAGCAAAATCTAAAAG
ATCGTGTATAAAGAATCTTGAAAAAAGATCATTTAGATTGGTGTAGCCAAATGTAAATGATCGTGTGAAA
AAAATAAAAGATTGTGTATAAAGAATCTTGAAAAAAAAAAATCATTTAGATTAGAGTAGTCAAATGTAAAT
GATCTAGTAAAAAAAAAATAAAAGATCATATATAAAGAATTTTGAAAAAAAATCATTTGGATTGGAGTAG
ACAAATGCAAACGATTGTGTAAAAAAAATTAAACGATCCTCTAAAAAAAGTAACGATCTTGTAAAAAAAT
CTAAATGATCATGTAAAAAAATCTAAATCATCGTGTATGAAAAGAATTAAAAAAATTGTGTACCAAATTT
TGAAAAAAAAATCATTTAAATTTGGGTCCTCAAATCTAAACGATCAAATCTAAATGATTATTTAACAAAA
TTTAAACGTAACAAAATTATAAATGATCGTGTAATAAAATTAAAAGATGGAATTGAAAAGATAAATTGTA
GTCATATCTAAACGATCGCGTATAAATTGTAGTCATATCTAAACGATCACGTATAAATTTTAGCCATATC
TAAATGATCGCGTATAAATTGTAGTCATATCTAAACGATCACATATAAATTGTAGCCATATCTAAATGAT
CGCGTATCAAACAATAACCAAATCAAAACGATCAGAAATATATTATGTGTTTGTTGTTGACGGTGTGGTT
AACGGGACATTTTTAGTATTTTACACTGTGGATCTCTGAGCTTTTTAAATTGTTCAACACAGTATAAATA
TTTTGTCATTTTTTTATATTTTTGAAAAGAGAGGCAGAGAAAATTTACAATTTAATTGGTTTTCTAGAAG
TAAAATATATGGTGTCTTCTTTCTAATACTTTTATTGGGTTGTAATTTATTTTCGTTTTTGAATTCTCAC
AAACAAAAGATATGGATGTAAACAATCGATGTATGCTTTTCTTTCCTTTTCTAATTCACGACCAACAAAT
ATTTGGAATTTTAAATTTAAAATTTAAAATAATATATAAACTAAATGTGTTTTCAAATATTGGCGAAAAA
GAAATTTTGTATTTTTCTTCTTTTTTTTTCTGCTTAATTCTTTATTTTCTAAAATAGTTTTTGGATCACA
ATGATTTAAGAGGGTCTAAGTTTTTAAAACATACTTGTGTAGACTCAATTTTTTTATTTAAGTAAAAAAAG
AATAAGGACATTTTGTCCCTATACTTTCAAATTTTACTTTAATTCTCAATATTCTAGGTTCATATGAACT
AGTGTGAAAATTTTGTTTTGTCTAATATCTTACTGATGCTCTTAAAAATTAAATAATCTTCTACAATAAT
GATTTTTCTTTTTCTTTTAGAAAGATATAGAGGGTCGGTAGGTGTACCTAAGTATCCTCATTAGGTGGAC
ATCCTTATAGCACCCTCATCATCCTTGATGTATATTCAATAAAGCGCAAGGAATATAATGTTTATTAAAA
AAAAAGAGCTAGAGATAAGTCCAAAGAAAAAGTCAGCAAAACACATTAATATTTAGAGCAATACTAGAAT
AAGAAAAGTCTTTAAAGAGTTTGTTATTATATGACCATTAACTAGTAAGGTTGCAAATGTTTTTCCAGAT
GTTTGCTACAGTAGAATATTTGTTATCGAAGAGTCTATTGTTTCTCTCCAACCAAACAAGCAAACAGTAA
CCACCATCAGGTTAAAATAAATCACATCTTTTTGGTTCTTAATATTGATGGAGTAAATTAAATATGATGC
AATAATGACATTTTGATTTATCGATTTCTTAGTCTAGAGAATATAATTTATGATAATTGAATTGTGCTCA
TTTTGATTATTGAGTTATGTTTAAGATTTGTGATAATTGAATTATGCTCAATTGAAATGGTAGGTTGAAT
ACACATGCAGTACTCAAGCCTTTTGTGATTGCGACACATCTACAATTAAGTGTGGTTCATCCTATTCATA
AGTTATTGGTTACACATTATAAAGATACCAGGTTCATAAATGCATTTGCAAGGAAAACCCTCATTAATGC
TGATAGACTTCTTGAAGCAACTCATTTTCAATCAAAATTTGCCATGGAGTTGTCTTCTTACCCATACAGA
TCGAGATTAGAATTTCCTTGAACAAACCTTACATGCAGATCGATCTCCTAAAGAGACCGTAATTTATATT
TTTTTTAAGTCATAAAAAATGCCTCTATATATAATATTATTGCCCCAAATTAAACGGAGCTTTTTCTCTC
TCACTTCTCTCGAGAATGTCGACAGAGCTGAAGAGTCGCCTTTAATCCTTCCACAACAGGATTCACCTGA
TGATATTTCATTGGGAGAAAAATGGCGGTGACAGACATTAGCTACCACAACCACCTCCCATGCAACAACA
TTCATCCAGCTTACTCGGTCTTTTTAAAGGTACCTCTCCACCTTTTTCTAACACTTAACTTCAGTTTGTT
TGTACTTCCAAAGAAAATGAGTTTTTTCAACATCTTCCATGCTCATGCAAAATCGAAAAAAAAGATTTTG

TCCTTTCTTTGGACAAACATTCCACCCCTGAGATATTGGATCATTGAGACGCGAGCCCACATGTCCTTTT
TCGTCTTGACAAGCGGTGACTCACTCAACTGGCTTATAAAAACTTTCAAGATCTTCATTGCAACTCCAAA
GACCAACAGATTTTTCTTAGAAAAAAGATATGAAGACTACGTACTTTGGATCAGAATGTTGGAAGCCTTT
GATGAACACCCCAAATAAATAATCACCAAATAGAATATTTGATAGAAAACAAAAACCTTTTTGAAAGACT
TGTTGTGTGTATTCAACTCACACTTGGATGATTTAGATTACAACATTTCAATCCTATTTATAGGATTGTC
ATAAGAATAACTCATATCACAAATAAATACAACAAATTAAATCCTACATTTAACTAAGGTGAAACTCTTA
CTAAATTAACTCTAACATTTTTAACTCACATCCTTCTCTAACTTACATAAATTAACTCACATATAACTTA
TTTATTTAAAACATGTTTAATTTTCAACATCCTCCCTTAAAAATGTTTTATGTTTTGCAACTCCAGCAAA
GTTCTTAAGTTGTTAAACACATTAACTTTGAGTGGCTTCGTAAAAATATCTGCAATTTGATCTTCAGTCT
TCACATATTCAACTTGAACTTCCTTCCTTGAAATGCAATCTCTGATGAAGTGAAATCTTGTATCAATGTG
TTTGCTACGATCATGGAACACAGGATTTTTTGCCAAAGCAATTGTTGACTTATTGTCTATACGAATCACA
GTTGGATCTTCTTGCAAAATTCCAACTGTTTTTAACAAATTTCTTAACCAAACTGCATGACAAACACATG
AAGCTGCAGCAACGTATTCTGCCTCACAAGTGGATAATGTCACAATAGGTTGCTTCTTAGAACTCCATGT
AAATGCAGTATTGCCAATGAAGAAAACATATCCACTAGTGCTCTTTCGATCATTAGTATCTCCAGCCCAG
TCACTATCACAATAGCCTTCAAGCTTGAATTCTTTAGATGAAGAATAAAACAACCCATAGTCAAGCGTAC
CTCTGAGGTAACGAAGAATTCTCTTTACCACTTTCAAATGAGTAGTTGTAGGAGATTCCATAAATAGACT
CACCAATCCAACGCTGAAAAGAATATCTGGTCGTGTGCAAGTCAAATATCTCAAACTTCCAACCAAACTT
TTGAAATATGAAGGATCAACATCATCTCCTTCTTCATGTTTGGACAGTTTGGTCCCAGTTTCAATCGGAG
TTGCGACAGACTTAAAATTCATCATATTGAACTTCTCTAGAATATCTCTAGTATATCGTTCTTGAGAGAT
GAAAATTCCTTCCTCTGACTGCTTCCCTTCAATGCCAAGATAATATGACATTAGCCCTATATCTGTCATT
TCAAATTCTTGGGTCATCGCCTTCTTGAGATCTTCAAACATACTTGCACAATTTCTTGTAAAAATTAAGT
CATCCACGTACAAACAAACTACCAAAATATCTCCATGACCATTAACCTTAATATAAAGAGAATGTTCATA
AGGACACCTCAAATACCCATTATCAAGGAAATATTTGTTGATTCTGCTATTCCACATTCTTGGTGCTTGT
TTCAATCCGTATAATGCCTTCCTCAATTTTAGAACTTTATCCTCTTGGCCTTTCACAGAATAACCAGGAG
GTTGTTCCAAGTAGACTTCTTCTTCTAGATATCCATTCAAAAATGCCGATTTGACATCCATTTGAAAGAT
CTTCCAATTATTTTGTGCAGCAAGAGCAATTAACAACCTTATGGTTTCCAAACCNNNNNNNNNNNNNNNN
NNNNNNNNNNNNNNNNNNNNNNNNNNNNNNNNNNNNNNNNNNNNNNNNNNNNNNNNNNNNNNNNNNNNNN
NNNNNNNNNNNNNNNNNNNNNNNNNNNNNNNNNNNNNNNNNNNNNNNNNNNNNNNNNNNNNNNNNNNNNN
NNNNNNNNNNNNNNNNNNNNNNNNNNNNNNNNNNNNNNNNNNNNNNNNNNNNNNNNNNNNNNNNNNNNNN
NNNNNNNNNNNNNNNNNNNNNNNNNNNNNNNNNNNNNNNNNNNNNNNNNNNNNNNNNNNNNNNNNNNNNN
NNNNNNNNNNNNNNNNNNNNNNNNNNNNNNNNNNNNNNNNNNNNNNNNNNNNNNNNNNNNNNNNNNNNNN
NNNNNNNNNNNNNNNNNNNNNNNNNNNNNNNNNNNNNNNNNNNNNNNNNNNNNNNNNNNNNNNNNNNNNN
NNNNNNNNNNNNNNNNNNNNNNNNNNNNNNNNNNNNNNNNNNNNNNNNNNNNNNNNNNNNNNNNNNNNNN
NNNNNNNNNNNNNNNNNNNNNNNNNNNNNNNNNNNNNNNNNNNNNNNNNNNNNNNNNNNNNNNNNNNNNN
NNNNNNNNNNNNNNNNNNNNNNNNNNNNNNNNNNNNNNNNNNNNNNNNNNNNNNNNNNNNNNNNNNNNNN
NNNNNNNNNNNNNNNNNNNNNNNNNNNNNNNNNNNNNNNNNNNNNNNNNNNNNNNNNNNNNNNNNNNNNN
NNNNNNNNNNNNNNNNNNNNNNNNNNNNNNNNNNNNNNNNNNNNNNNNNNNNNNNNNNNNNNNNNNNNNN
NNNNNNNNNNNNNNNNNNNNNNNNNNNNNNNNNNNNNNNNNNNNNNNNNNNNNNNNNNNNNNNNNNNNNN
NNNNNNNNNNNNNNNNNNNNNNNNNNNNNNNNNNNNNNNNNNNNNNNNNNNNNNNNNNNNNNNNNNNNNN
NNNNNNNNNNNNNNNNNNNNNNNNNNNNNNNNNNNNNNNNNNNNNNNNNNNNNNNNNNNNNNNNNNNNNN
NNNNNNNNNNNNNNNNNNNNNNNNNNNNNNNNNNNNNNNNNNNNNNNNNNNNNNNNNNNNNNNNNNNNNN
NNNNNNNNNNNNNNNNNNNNNNNNNNNNNNNNNNNNNNNNNNNNNNNNNNNNNNNNNNNNNNNNNNNNNN
NNNNNNNNNNNNNNNNNNNNNNNNNNNNNNNNNNNNNNNNNNNNNNNNNNNNNNNNNNNNNNNNNNNNNN
NNNNNNNNNNNNNNNNNNNNNNNNNNNNNNNNNNNNNNNNNNNNNNNNNNNNNNNNNNNNNNNNNNNNNN
NNNNNNNNNNNNNNNNNNNNNNNNNNNNNNNNNNNNNNNNNNNNNNNNNNNNNNNNNNNNNNNNNNNNNN
NNNNNNNNNNNNNNNNNNNNNNNNNNNNNNNNNNNNNNNNNNNNNNNNNNNNNNNNNNNNNNNNNNNNNN
NNNNNNNNNNNNNNNNNNNNNNNNNNNNNNNNNNNNNNNNNNNNNNNNNNNNNNNNNNNNNNNNNNNNNN
NNNNNNNNNNNNNNNNNNNNNNNNNNNNNNNNNNNNNNNNNNNNNNNNNNNNNNNNNNNNNNNNNNNNNN
NNNNNNNNNNNNNNNNNNNNNNNNNNNNNNNNNNNNNNNNNNNNNNNNNNNNNNNNNNNNNNNNNNNNNN
NNNNNNNNNNNNNNNNNNNNNNNNNNNNNNNNNNNNNNNNNNNNNNNNNNNNNNNNNNNNNNNNNNNNNN
NNNNNNNNNNNNNNNNNNNNNNNNNNNNNNNNNNNNNNNNNNNNNNNNNNNNNNNNNNNNNNNNNNNNNN
NNNNNNNNNNNNNNNNNNNNNNNNNNNNNNNNNNNNNNNNNNNNNNNNNNNNNNNNNNNNNNNNNNNNNN
NNNNNNNNNNNNNNNNNNNNNNNNNNNNNNNNNNNNNNNNNNNNNNNNNNNNNNNNNNNNNNNNNNNNNN
NNNNNNNNNNNNNNNNNNNNNNNNNNNNNNNNNNNNNNNNNNNNNNNNNNNNNNNNNNNNNNNNNNNNNN
NNNNNNNNNNNNNNNNNNNNNNNNNNNNNNNNNNNNNNNNNNNNNNNNNNNNNNNNNNNNNNNNNNNNNN
NNNNNNNNNNNNNNNNNNNNNNNGTATTTTAATTTCAACTTTGACTGAAAAAGTTGCTCAGTCATCTACT
TGTTCTTCTTAAGAAGTTTCTCGTCATGGGCCTGTATAGAACCCATTAGTTCATCAATGGACATTGTTGT
CTAAATCCTTTGATTCTTCAATAGCTACAACGATGAAATTAAATTTTTCATCTAATGAGCGAAGTATCTT
TTCTACTACTTGCTCATCGCTTATTGTCTCACCAAATCTTTTCATTTCATTTACTACTGCTAGCAATCTT
GAAGTATAATCTGAAACTGATTCAGACTCCTTCATATGTAGTGATTCATAATCACCTCTCAATTTTTGAA
GGCGAACCTTCTTGACTCGATCGACTCCTTTATACGTATTCTCCAAAATTTGCCATGCTTGATATGCAGT
AGTTGCTCCAGAAATTTTCTCAAAATTGTTATCATCAATGGCTTGATGAATGATGGTGAGAGCCTTTTGA
TCTTTTTTTCTTGTATTTTGTAAAGCTTCTCGTTGAGCTTGATTCAAAGTTGCATCACTTTCTGGTTCTT

CATAACCATTACTAACAATGTCCCACACATCTTGGAACCAAGTAGAGCTTTCATTCGAATACACCAACTA
CTATAATTTTCTTTCGTAAGTCGAGGTACTTGGAAGGGAATTGCAACTCCAAAGACCAACAGATTTTTCT
TAGATTTATTATATGAAGACTCAGTATTTTGGATCAGAATGTTGGAAGCGTTTGATGAACACCCCAAATA
AATAATCACCAAATAGAATATTTGATAGAAAACAAAAACCTTTTTGAAAGACTTGTTGTGTGTATTCAAC
TCACACTTGGATGATTTAGATTACAACATTTCAATCCTATTTATAGGATTGTCATAAGAATAACTCATAT
CACAAATAAATACAACAAATTAAATCCTACATTTAACTAAGGTGAAACTCTTACTAAATTAACTCTAACA
TTTTTAACTCACATCATTCTCTAACTTACATAAATTAACTCACATATAACTTATTTATTTAAAACATGTT
TAATTTTCAACATAGAAAGACAACCAACAGAAATGGTAACACAGTAGAAGTACTTTTCAGGGTGGACACT
CATAGGAGAAAATGTTGTAGGTCCTAAATCAAGTGGGTGGGTGTCTTTCCTCTCCATGATCAGTCCAAAT
GTGGAAATTGAACCAAAGGGAAGACTACCAATAGACTTGAAGATCAAAGAAATTAGAGGAAATGCTTATT
CATCTTCAGATTCAACTTCCTCATGCTCATCTTATAGATCATATGCACAAGTCTTATCTAAAAGCAACAG
GAGCGAATCAAACTCAAGTTCATCAAGGGATGGTAAAAAGCGCATCTTCTCAAAAAACCCTTTCAACTGA
AACAACACTGTGGTTATCACCAGAGAAGATTGTTCCACGATGATTGGGCAAAGATATATAATGTTTAACC
TCAAAAAACAAACTGGAGCTTCCTTCACTTTCAAACCTTTCCATCTAGAAAATGTATTGATCTCCTTTAA
TGATTCAGTACATGTCAAGCTACTGTACAACAACACAGAATAGACTACAGTTGGAACCTTCTATGTCAAA
TTTGAGTCTTGGTCTTCAGAAAAATATTCCTCAATGAAGTTAATTCCCAGCTATGGAGATTGGCTTTCTT
TCAGAGGAATTCCTATACATGTATGGACATACGACACATTTGTGAAGATTAGAAATGTTTGTGGAGGCTT
CACAGCAAAAGAAACAATTGAAATGACTGATCTTCTAGAAGCAAAAATCAAAGTTCACTACAACTACTCA
GGTTTCATTCCAGCTTTCATCAGAATCAACGACCAAAAAGGAAAATTCTTCACAGTACAGACTTATTCTC
CAATATCAGGTAAATGGCTAATGGAGAGAGGGGTAAAGATCCACGGTTCATTCAAGAGGCAGGCAGCAAT
ACAGTTTGATGAGTTTAATTCAAAATGTGAATAGTTTTGCTTCATCGAAAATTAGCTATACCACTGGAGA
AATTTGAATTCAAAAAAATAAGATTAAATGTTGTGATCTCCTCCAAAGAAAATCACACAAAAGCCAAGGA
CTTAAATGTGGAGAATGGCATGAACAAGAAAAATAAGAGAGAGAAGTGGGTAGAGAAAGGAAAAAATTTA
ATGCATGCTAATGATGACTCCATGGAGAAGAGTGAAAATTCATTACATGCTATCATGGATGAATGCAAAA
GAAATAGAAAAGTCAGCTATAGAAGAAAAGGTAAGCTTTATCTCACGAAAAACAAAAACTCATTACTTCA
ACTCGGATACAGCTCCATCAAAGCAAATCACTATGGAGGAGGGATATGGATATTGACAATGACAACATAC
AAGAGATACAAACTCGGATCAGTGGTTGGGTTTCCCAGTGCATGCCGATCAGTTTCTCGTTAAAAAAAAA
AAACCTCTATATATAAACTTATAGAGTATTTTTAGAAGAAAACAAGATGTTTGAAGCTACTAGCGGAACA
GGTCACCAGGACAGACAAACATCGATATATATTAATTATATATCTTCATAGAAATAGAAGGGCTGGAGGT
AAGCCCGAAATTAATCCATCAAAGCATTAAAATTAAGAGCTATTTTGCTAGGTCTGTAGTCTTTGAAAAG
AGGGCAACAACTGCACCAAAGGCCTAGTAAATTACAAGCATCTTTCCATAGGTCTGATATGTTTTTGTGA
ATATTGTTGAATATTCTATTGTTTCTTTCGAACCACCAAGACCAAAGGATAGCCACACCCATATTTAAGA
GGATGATATTTTTCCTACTCCTCTATACAAAAATATCAAAGTCAATGATATGTTGTCAGTACACACCTTT
CATTATTATGATTGTTAGAAATCAAGGAAGAAAAAAGAAAATATCTAAATTACCTCTTTGATTATAATCA
TGGACGAAATAGAATTTTTAGTTTCTTATGTGAACACAAATCTAAACTGATATTTTAAGGAAAAAACATA
TTGACACTTGACGAACATCTGTGAACTATACTTGTTGTCGTGTCTTACTTCTTCCGAAACCTCACATTTT
TTATTTTTACTTTTTCATTTATGATTTTTTCGCTAATCCTATTCTCTAAATTTACCTTAGCTTACAAGGT
GGACCCATGTTCTCCACATGGATTGAAGTTATTAATAGAGGATTATGCATATGATTCTCGAGGTATCTAC
TACAAAGATGACACCGCAATCCAAAATGACATTGAACTCCAATCATGGTGGAAGGAAGTTGTAGAGAAAG
GTCATGGTGATAAGAAACATGAAGCATGGTGGCCCAAGATGCAAAGTTTAAATGAACTAATAGAATCTTG
CACCATTATTATATATATGGATTGGTTCAGCTCTCATGCCGCTATTAACTTCGGACAATATCCTTACGGA
GACTTTGTTCGTGATCTTCCAACGGTAAGTCGAAGATTTATTCCTGAGGTCGGTAGTAAAGAGTATAAAG
AACTTGAATCTTGTCCTGAAAAAGCCTTCTTGAGAACCATAAATTCTCAACTACAATGCCTTTTTGGAAT
GTCAATTTATTGAAAATTTATCAAGGTTTATCTTGGGACAAGAGGTAGCTAAGAATGGACATGTGATAAA
GATGCATTGGACGCATTTGATGATTTTGGTCATGAAGTTGAGGAAAAGATTATGGAAAGGAATCGCAACA
TCAAATTCAAGAATAGAACTGCCAAACTAACGTTTCTTATACTTTGCTTCTTCCGTCTCGCACTGAAGGA
ATCACTGGCAAAGGAATTCCTAATAGTATCTCTGTCTAATTAAAAAAGCAATTTGGTTTGTGTGTGTTAT
GTTATTTTGATTTTTCTTGCATGTTGTTATCTTTTTGGTTTGACGATCAAACCAATCCTATTTCTTAAGC
TATATGAAATAAAAAATAAAAAATGCTTAAAGGTCGGTTTGGATTTGAATTTGTTAGTTGGGTTTTTAAA
TTTTAAAAATTTGTCATTTTATTTTAGTATATAAAATAGTAAAATTTGTATAAATAGTATGATATGAATT
TGTATATTCAAGCATAGTAGAATCTCATATTGTGTGATCTCATATTATCATAACACATATAATTTCTATG
AACATGAAAGTAAGCACACACAAGATAATATTAGCCACCAAACTTTCTCCCATATACATTCTAACTATTA
GATGACTTTATTGTTTAGCTTAATAATTAAAAAAACATATATAACAACCAAACTAATCTAACGTGGTAAA
TTTGGAAATATGTAAATTAAAATCATACACTATATTTCACATCTCAAAGATGAATAATTTCATCATAACA
AACTTTTAGTGACAACCAAATTGAAATTTTGATTTGAATCGCTCATTTCAAAGCTTAATTAAAATTAGAG
TTTTCTTTTTCATTTTTTATTAACTCTTCGTGGCTCACCATCTTTTACAACCTCATAAGACACCTTTCTT
CAAATTTAGTACCATTGAATTGTTCTTAATTTGTCGTAATCGATCATTTCTTGATCTTAAACCCTTCTCC
ACTTTGCTCTAGCTGGAATTGAAAAACATAAATTAACAAAATATCATTTTAATGTTTACCCTCCTCGTGA
TAAAAGAGAGAAAATTTGGGAAATAAGAGAGTATACTTGATAAACTTCCCTTTTTCGTCACTTCCATGCT
TGTATTTAATTTTTCTTTGTAGTTTTCTATCAATTTGAAGGTGGCAAGCTAATAAAAAAGAAGAAATCTT

AAAAAGAACGCTCTAGAGTGAATTCTTCCATAATTCCATACATGTGTATACCTAATTGTTCAATGTTGTT
CTATGTTATTTGATTTTGATTGAATCCAATGATTTATTTATAGATGTGATTTGATCGATGTTGAAATTTG
GTTAGGTTATTGTTTTACTTGTAATAAGAATAAATGATTTTGTAGCTTCATTCTTGAGTAACGTAATTTA
TTCATTAATTTGCTTTACTTAAATGCAATAATGAATGATTATAACATTAATTATCAATTAAATTTAAGAG
TCATTCCAATTACTTTAAAAATCCAGAAAACTACATGAAATTATTTTATTAATTATTTAGAAATAAAATT
ATGGGAGAAACCTTTTCACATATTTATTATTGAATGCAAAATTACCACCAAGTGAGCTATGAGAAAAAAA
AAAGAGTGTTTTTGAATAAAAAGAAATGTTGACTTTACATAATTATGGCCAAAAGTTGTATAGATAAATG
TTATAGCGAAAGAGTTAAAAGATTACATTTTATTACTGGTGTAAAACCATTATATAAAATAACTTGAAAT
TACCTATGAAACTCGAAAGGATTGAAACCTTAGTTTGACAACATTTGTCAAAACATATATACATCTCTTA
TTAATGCAAAAATATTGCGATGCATTCTTGAAAAAATTGAGAGAATGAATTGAGAAAAAAACACACAACA
AACAAATTAAGAAAAATAAATTAAAATTTTCGAAAAAAAAATAGTAGAGGATAACTCTTTATATAGCTAG
GAGCGGGAGGGATTGCATAACTCTTAAAATTCAGTTATTAAATATAGTTAAATGTAAAAAAATTAATTAT
ATAATTAATATCTGGAAATTCAAACCAACAAAACTTTTTGAATTATAGTATTTCATCTTCTAACCTTTCT
ACTTCTTTCTATCTCTTTTATTGATATTAATCATTCATGGAAATGTTATATTATTTAAAAATCTAATTAC
TTATAATTTACTTATTAAACAAATAAATATTATCATTTTCTTAAAGTAAAAATTTTAAGGCATAAATTTG
CACTTTTTTTTTTTTTTTTTACTTTTAAGAATCCATTTATGTAAATTCATGATAAATAAACAATAACAAC
TTAGAATATCATTAGGTTGGGGTTATTTTTATTGTGATTTTCATAGAAATAGTAAAAAAATTGTAAATAA
TTAGTAGTTACTAGTCTTAAATAATGGAAAAGAAATTGTGCTTAGTTTATGAAAAAAACAATTAACTATT
AATTATTTAAAATAAAATTTTTAAAAAAAGATAAAAGCAATTAATTATTAATTATTTGTTGGGTATTGAG
AAAATAAATGTGGAGGTAATATTTAATATTCAATTCTTCAGTTAAAAACAATAATTAATTATAAATATTA
TAAAATGAAATGAAAATGACAATTATAATAAAAGCAAAATTGTAACCGAGTAAATTCAACATTTTAAAAT
AACAATATGAAGAAAGCAATGAGGTACGAAAGTAAAACCTAACAAATTTATCAAATAATAGATTGTTTAT
TAAAATTTATCAAAATAAATGATAAAATAAGATACAAACTAAAAAACTTTGCCATTATTGATGGATTAAT
CTCTAAATTTTATCTACTTCCTTCTATTCTTGTATGTACAAAGAGATAATTGCTTTATACAGGGAAGGAG
GGAGGAAGTTGAAACTAACTTGGAAAAGGAAGAGTCGTGTTTTAATTCATTCAATAATTATCAACATAAA
CTTCTCAAATTTAAAATTTTTAAAAAAATAAAAGACATTATTGTTTTCCGCATAAAAAAAAATTCAATGG
CAAACCCAAAATTAATATTTAATAACCCATTATTAATTAAAAGCCTATTATTTTAATGTAAAGACAAAAT
ACAAAGTTTAAAAAGAGAATTAATAGTAATCTACATAATGTATTAAGAAAATAGATATAATGGAGATAAA
AAGTTTCTCCTTGTATAAGCTTTTATATATATATATAAAAGAAATGCATGTATGCCAACTCTATTATCGT
GCTATCTTACTTTTTGCTGTGTTTGTGAAAGAATAAAAGAATTGTGCTTTAGGTTTTAATTAATTCCCCT
TGTACTCTTGATTTAAAAACTATGAAAAAGTTATATATATTATGTGATTTCTATAAATATGTGATTTCTT
GCTCTTGTCTCATATAAAGTACACAAAAATACAGAGAGAATTGTTTTCCTCCAAATTTTTTTTTTTTTCT
CTTTTCTATTAGTTGCTGACGATCCCACACGTTGGAGAATATGGGGAGACTTTGCAAAACATTTAAAAAG
TCATATGGATTTATAGTATTTTATTTCTTTCTTTGTTTTGGAATGGACTCTAATGTTTATCCGGTAAAAG
AGTCCAAGTAGTATTTAGTGGTGAATCCAAAGGGGTATTCGTTTTAAAGGACCAAGTTAAAGACTCTGAC
GTGGAAAGATGAAATGATCGATTAGTTGTATAGATATGATAAAAGTGTGTAATTTGATGTGATCTAATTA
GTACAAAAATACAATGTTGTCAATAGGTAATGGCTTTAAAGTATTCAAAGTATTGTATGCACGAGACTAA
GTACAGTAAAAGTAAAAGTAAAGGTAAAGAACGGTAAAATATATAAACATCACTTTTTATTCATTCATAA
ATAAAATACATGATAAGCTCCTTAAGCATAGACTCTATGGCTTTAATTCCAAGAGTATAGCTAGGCCAAG
TAGACTTTTTCACATTAAACGTGTAAGAGTGACAATAATAGAGAACAAGGAAATGAAATATTTATAACCC
AATCATATGCTCAAAGAGTCCTTTCAAATAGAGATGCTGTTTGGAATTCCTCTGCCTGTGAGCCCCTCGG
TACTTGATGGAAGAAGCAAAGTATATGGAACATTAACAGGTCCAGTTCTATTCTTGAGATTCACATCTTT
GTTCCTTTCAATGATTCTACTTTCAACTTCAAACACTTGTTTTCCAAAATTCTCAAACACTTCCAGTGCA
GCTTTGTCTGAAGTCCATTCAATGCTAGCTCTTTGTCCAAGATAAACTTCATCAGAAGCATGCCTTGACA
ATATTTCAATTAGTGAAACTCCAAGAAGTGTTTGCAACATCGAATTGATTGTGTTCAAGTAAGCCTTCTC
AGGCTTTGATTCAAGTTCTTTGTACTCAGCACTTCCTACTTCAGGCATATGCCTACGACTTATTGTTGGT
CGATTCGGAATAAAGCCTCCATAAGGATATTGTCCAAAGTTAACTGCAGCATGAAGAGCTGAAGATATCC
ATATGATGGTAGTACATGTTTCAACCAGTTCGTTGAAATTTTGCAACTTTGGCCACCATGTTTCATTTTT
CTTATCAGCATGACCTTTCTCTCTAGCCTCTTTCCACCAAGATTGAAGTTCAACATCATTTTGAATTGCA
CTATCATCTTTGTAGTATAGAGAACAATAGTTTGTAACCCATGTTTTGATTGTTGACCAAATCTCAAGCC
CATCAATAGCAAATGGATAATCCTCTATGAGTAACTTAAGTCCATGTGGTGAACTTGCATCCTCAACCGC
TACACCTCTACATAAATAATGTAAGGATAAGAAAATTTTACAAACCACTACTGAATTATGTGACTAGAAA
TGATTAATTATCAGGCTAGGGAGACTGAGCGCAACAAACTCTAGCATAGCCGGGAGTGAAATCAAGTATA
TAATTAAAAATTCAGGTGTGAAGTGTACCAAACCTTTATAGTTCAAGAGACTAATTGACGTAATTACACA
GACTTATGTAACAGAGTAATTGTTGACATACCTCTTGATGAGATCAGCAAGGGAGTGCTTTCTCATTGAA
ATTCCATTCTTTATATATGTAAGAAGAAGACTCCATGGCATACTTTGATTTAAAATGAGTTCCTTCANNN
NNNNNNNNNNNNNNNNNNNNNNNNNNNNNNNNNNNNNNNNNNNNNNNNNNNNNNNNNNNNNNNNNNNNNN
NNNNNNNNNNNNNNNNNNNNNNNNNNNNNNNNNNNNNNNNNNNNNNNNNNNNNNNNNNNNNNNNNNNNNN
NNNNNNNNNNNNNNNNNNNNNNNNNNNNNNNNNNNNNNNNNNNNNNNNNNNNNNNNNNNNNNNNNNNNNN
NNNNNNNNNNNNNNNNNNNNNNNNNNNNNNNNNNNNNNNNNNNNNNNNNNNNNNNNNNNNNNNNNNNNNN

NNNNNNNNNNNNNNNNNNNNNNNNNNNNNNNNNNNNNNNNNNNNNNNNNNNNNNNNNNNNNNNNNNNNNN
NNNNNNNNNNNNNNNNNNNNNNNNNNNNNNNNNNNNNNNNNNNNNNNNNNNNNNNNNNNNNNNNNNNNNN
NNNNNNNNNNNNNNNNNNNNNNNNNNNNNNNNNNNNNNNNNNNNNNNNNNNNNNNNNNNNNNNNNNNNNN
NNNNNNNNNNNNNNNNNNNNNNNNNNNNNNNNNNNNNNNNNNNNNNNNNNNNNNNNNNNNNNNNNNNNNN
NNNNNNNNNNNNNNNNNNNNNNNNNNNNNNNNNNNNNNNNNNNNNNNNNNNNNNNNNNNNNNNNNNNNNN
NNNNNNNNNNNNNNNNNNNNNNNNNNNNNNNNNNNNNNNNNNNNNNNNNNNNNNNNNNNNNNNNNNNNNN
NNNNNNNNNNNNNNNNNNNNNNNNNNNNNNNNNNNNNNNNNNNNNNNNNNNNNNNNNNNNNNNNNNNNNN
NNNNNNNNNNNNNNNNNNNNNNNNNNNNNNNNNNNNNNNNNNNNNNNNNNNNNNNNNNNNNNNNNNNNNN
NNNNNNNNNNNNNNNNNNNNNNNNNNNNNNNNNNNNNNNNNNNNNNNNNNNNNNNNNNNNNNNNNNNNNN
NNNNNNNNNNNNNNNNNNNNNNNNNNNNNNNNNNNNNNNNNNNNNNNNNNNNNNNNNNNNNNNNNNNNNN
NNNNNNNNNNNNNNNNNNNNNNNNNNNNNNNNNNNNNNNNNNNNNNNNNNNNNNNNNNNNNNNNNNNNNN
NNNNNNNNNNNNNNNNNNNNNNNNNNNNNNNNNNNNNNNNNNNNNNNNNNNNNNNNNNNNNNNNNNNNNN
NNNNNNNNNNNNNNNNNNNNNNNNNNNNNNNNNNNNNNNNNNNNNNNNNNNNNNNNNNNNNNNNNNNNNN
NNNNNNNNNNNNNNNNNNNNNNNNNNNNNNNNNNNNNNNNNNNNNNNNNNNNNNNNNNNNNNNNNNNNNN
NNNNNNNNNNNNNNNNNNNNNNNNNNNNNNNNNNNNNNNNNNNNNNNNNNNNNNNNNNNNNNNNNNNNNN
NNNNNNNNNNNNNNNNNNNNNNNNNNNNNNNNNNNNNNNNNNNNNNNNNNNNNNNNNNNNNNNNNNNNNN
NNNNNNNNNNNNNNNNNNNNNNNNNNNNNNNNNNNNNNNNNNNNNNNNNNNNNNNNNNNNNNNNNNNNNN
NNNNNNNNNNNNNNNNNNNNNNNNNNNNNNNNNNNNNNNNNNNNNNNNNNNNNNNNNNNNNNNNNNNNNN
NNNNNNNNNNNNNNNNNNNNNNNNNNNNNNNNNNNNNNNNNNNNNNNNNNNNNNNNNNNNNNNNNNNNNN
NNNNNNNNNNNNNNNNNNNNNNNNNNNNNNNNNNNNNNNNNNNNNNNNNNNNNNNNNNNNNNNNNNNNNN
NNNNNNNNNNNNNNNNNNNNNNNNNNNNNNNNNNNNNNNNNNNNNNNNNNNNNNNNNNNNNNNNNNNNNN
NNNNNNNNNNNNNNNNNNNNNNNNNNNNNNNNNNNNNNNNNNNNNNNNNNNNNNNNNNNNNNNNNNNNNN
NNNNNNNNNNNNNNNNNNNNNNNNNNNNNNNNNNNNNNNNNNNNNNNNNNNNNNNNNNNNNNNNNNNNNN
NNNNNNNNNNNNNNNNNNNNNNNNNNNNNNNNNNNNNNNNNNNNNNNNNNNNNNNNNNNNNNNNNNNNNN
NNNNNNNNNNNNNNNNNNNNNNNNNNNNNNNNNNNNNNNNNNNNNNNNNNNNNNNNNNNNNNNNNNNNNN
NNNNNNNNNNNNNNNNNNNNNNNNNNNNNNNNNNNNNNNNNNNNNNNNNNNNNNNNNNNNNNNNNNNNNN
NNNNNNNNNNNNNNNNNNNNNNNNNNNNNNNNNNNNNNNNNNNNNNNNNNNNNNNNNNNNNNNNNNNNNN
NNNNNNNNNNNNNNNNNNNNNNNNNNNNNNNNNNNNNNNNNNNNNNNNNNNNNNNNNNNNNNNNNNNNNN
NNNNNNATTCTTTCCAATTCTAAACATTTTTCTCTTTTTTTTAAGAACACTTTTTTTTTTCTCTCTTTGT
TTTAATATAGCTTTAACAAGAAGCTGTTGTGAGGATGAATGAATTTTTCTCTTGTATTTATAGCAAAATT
TGAAGAACTTTGGCCCTCTCTCTCTCTTTCATTTGACTGATAAGATCGCCTTGGGAATTAAGTCAATAAA
GAAATGAGAGCAACTTTTTATTATAATAATATATTAAAAAATGGACAACAAGAAATTTGAATATAAAATG
TAATTATTTTCTCACCTCATGAAAACGTGTTTATTAAAATAATAATAATAACAGGCACTTTCTGATCATC
TTTTTAGAGAGTACAATAAAAATGATAAATAAATGTATTAGATAAATATAAAATTTTATCATAAAATTAA
TCGATAAAGTGAGAAGTAATCATTATCTTATGAAGATGGTGAGCTTTTTCATCTTTTTGGGGTGAGATCT
TCGATATTTTGTTTCAGATTGGATAATGATCTTCACATGACCCCTCATATATTTTGGACTCCCTCACAAC
ATATGGTTAATGAAGTTTTTTTTTCAAAGAAAAAATTCACAAAATTTATCAGCAAGAGGATTTTTAAAAT
TATTTTGAACAAGTCGAGATCAAACACCTGTAGTTAAATATTTTTTTATACAGAAATATATAAAACCTAT
GTTTCAAGAAGGCATAAAAAACTTTCAAAATGCTTCCCATAATAGTATCCTCGAAAATCTCTGAATGTTT
TTGTATCTTGCCTAATTACTGCCACAAGTTTAATAATTGAGATGCTAATGATGAAGATTACAAACAAACA
ATTATTGATTGTAATAAAAGTGAGTTTGAAATTACAAACAAGCAATTATTGATTGTAATAAAAGTGAGTT
GGAAATTACGAACAGACAATTATGGATTATATTTAGGATATTAACGATGGAAGCCTTTTTTGATATAAAA
AAAGTAGTCTTTCCCTTTGAATCTGTACATAGCGAAAGTAAATGAAGAACTACAATCGATGTAGACGTTA
TTTAATAACTTAAATGTTTGTTTATCCTTTTGATGGTTATATGTTTATTTGGTTCTAATAATGTCTAATA
AGTCTACAACTTTTTATTTACGTGGCTAATTGTAATGCTCTTAATTTAAGATCGAGGGAGACATAAGTGA
ATTGATATCGCATCTGAATAAAAGATATCCTAAGGACATGAAAGTTACATTAATAATGACTTTAAAAAAA
ATTAAATGTTACTACTTATATCCAATGAGAGGTGTATCTTTCTTTTAGGTGACCTGATCGTATGAACTCT
AAAGTTAAGCGTACTTCGCTCGGAACAATTATGTTTTTTGTGACCTTATGTAAATTTTCTTAGAATGCAT
GTGAGTGAGGACAAAGTATACTGAAAGGATTCGTGTTGGTTTGTGGAGTAATTTTTACTCTAATAAAACG
GAAATGTTGGAGATCCGAATTTCGAATATTGGGCCTAGGGCGTTACACTAATAACCCTTAAACTTTTAAT
TTTATGTGTAACAAATCTTTGACGAGTTCAAATTCTTAAAAAATTATTTGACTTTTAGTGTTTTTGCGAA
ACGAGATACTAATGGTTTTCTTACATATTGTATTAGGGTAAGGGTGTTTTTGAATTTGTTTTCTTATAAT
TTGATTACAATAATAATAAAAGTTTGAAAGTTGAACGGGTAATTTTTAGAAACAAAATAAAAGAGTATAT
ATGTGCATTCTATAATTTAACCTATTTTTACGTCTTTTTGGAAAATTGATCCTCTAATCGGTTAAAACAA
CAATGTTGCCAATTTTGACATAGCGCCCAAAGCGGAAAAAGAAACTAATTATCTATGATTTCGTATATAA
GAACCTATTTGAATAGACTTGAGAAAAAAATATTTATCGCATTTAAACTCATTTGATAAAAAAGTTATTT
AAAATAAACTCCAAAAAATATTTTGTTTGGTTGTCAACTCCTTTCATATTTTTCAAAAAACTTATTTTTA
AAAAATGTATTCTAAACTATAAAAAGTAATATTAAAAAGAAAAATAATAAAGACAAAAAATAGTAATTAG
GAAAAAACCATAAACCATAAATTTCAATAACATTGATGTATCCAATTATTGGGGGTTGGATCCATTACTC
CTGCTGTTCAACTATTTTTTAAAAAAATTGAATAAAACTAATTATTTTCTTATTTCGTTATCTGTAAGAA

AATTCATTCAAGATTCTCGATCTGAAATCATATATATATATATATATATATATATATATATATATGAATGTG
ATGTTATCATTAATATATAATTGAATGGCTTATTAATTATATACATGCTTATGTGTCCTCCTTTAATTCT
TCTTTTAAAAAATATGTCCTTCGGAATTTATAATATTAATGATATAATTAAACAATTCAAAGTTTAAAAC
ATTGGATAATTGAAGTTGGTGAGATAAAATAATTTGATATCAATAATATATTACTTAATTACCCATTTTG
TTGATTGGTATATAGACCTTTTAATATATTTCCATTATTATTAGAGATCATCTTCTGTTATTGATAGACT
AATTTAATGCTGTTTGGCCACCATTTTCTTGCATGGTAGACTATTTTTATTTTCAATTTTATCATTGAAG
GGCTATCACTTTCATTACGATAGACCTAATAAGCATGTAAACACAATTACTAAAAGATGTAAAAATAGTA
AAAAAAGAAATATAGTGATATGGTTAGAAATGATGCGTTGATAATATTTGTTTAAGTTTGATATATGTTT
TATGGGATGTGTCTAATTTTGTGTTCTTTTGATGAAGGGATGTTTTTAGGAAATGTTGTTTTTTTTTTCT
TTTTTTTTTTTTTTTGCAAAGATTATTTTCTTACATGAAAATTCACTTAAGATTGTTTCATGTTCTAAAA
TATATAGTTTTTAGATTTTACTGGATGCATTACATTTCTTTTGTGTTAGTATTATGAAATTGACAAATAA
AGATAAGTGTTATAAAAAAATTATAATTATCAACACAAGGGATTTACATAGGTCATTAAAAGTGTATTAT
TTGGATAGACAAAAACAATTTATTATAAGAAAGAACAATTCAAATTATAGATTAGAGGTGTGTCTCTAAA
GTTATAGAGTTTATATAGCACACTACCATAAACCCCTAAGTTAAAATCATAAATGATGTAAATATATGAC
AAATAATGATCAAATACAACTTAGCTTACATTTAGAGAGTGTCACCCTAGCTCCTGATTCTTTGATCAGA
CAATAGGACTTCGTATACTTAGTGGTTTAAAGTAGAAGATATTAAATTCGAGGAATTAGATGTTGATTTC
CTTAACCTTTGACAAAGGATAGATAAATTTGATAGAAGAAAAACGTTTCTTTACCTAATATATAATATAA
AATTATCGATAGAGTTTAAAATTCAAATTGGTAGATTAGAGAAATATTTGACAATCTAAGACATCTAAAT
TTATCACAATCTCTTTCGAACAATGGGCTTGAATATTAAAATACAAAAATCGAGACGTAAACTATTAATA
AACTAAAAAAAGAGATGAGAGATTTATTAAACGTACTTAAATCCATTTGTTTTAATTCTTATATGTATTA
AACTATAAGAAACTTCAATCTTTAACTAAAGTATAAGTTGTAGAAACTTCAATCTTTAACTAAAGTATAA
GTCGTAGAAACTTCAATCCTTTACTAAAGTATAAGTTGTAAAAATTTATATGATGTGGTACGATCAAAGT
TTTATACGGTTTGGGTACAAAGGGAAATATTGTTTGTATATTAATTGAGAAAAATATATTTTAATTGGTA
TGAAACATTTTAAGTGAAATCGAAACAAAAGAACCAGAAGAAATATATCACACTAAGGTGGAGAATATGG
GAAAAAGTCATTATCTCTTACAAGTGGTATCAGAGCCATTTTCCTAATTTAGAGTATTAGTAGAATCCTC
AAGTACTTAATAAAGAAGTTGTGAACGTTGAGAACGTGGTAGTTGATGTAATTATGATGAAGATCCATGG
TGGGACTTTATAGGCTTGATGGCGTGCCTCTAAAGAAGAAGAGTTGTCTTGAGTAGGAGCACACTTTGAA
AAGATGAGTTGACTTATGTAAGAACCAGATTGATAGGATAACTATTTGATGGGAGGCTAATAGTATACGT
TGCGTAAAGGAGTGATTGTTGAAAACTGTGGTCAAAAGTTTTTCATTAACATCTAAATTAGAAAAATAAT
TAAAATATATCTTTATCGATATACGAAAACTTAGGTATACGGAAACTTAGGTAATGATGATAATTAATGT
TAAACATATACTCTAATTAAGAAACTAGTAGTTTAATATCCTCACCTCTCATGTGGAAGTGTTAGGTTAC
CAAGAAGATTTAATTTACTGGAGGATTCAATGTTCTTTATGAGTGCAGGTTTATTATGTTGAAACTATAG
AGATCATTAGTCCTAACTTTTCAATCAAGTTTCTCACATGGAGATTGGTAATGGTAAGGATTATTATTAC
TTGATAGATTTGGATGGCAAGAAATAAAAAATGGTGCTTTAGTTAGGCAACCGACAAATGCAGAGTTCAA
TAGAAACTTAATTAACAAGCTTAATGTCCTTGACTTTCAACTTGAGTACCGAGAGGGACAACATAAAAAG
AAATGGGTAGAGACGATCTTCACTCTACTCACTACAAGAAATTTTATCTCTGTCGACGACAAACATACCG
ACACTCCTAAATAATTTCATCGATAGAAGTGGTATACACCAACGAATTGTGTTATCGTTGGCAATAAGTG
GAATTGCCGATGCAAAAATTATAGCGTTGGCGTAGATAGGTTTTTACCGACGACATATTTTTGTACCGTT
GGCATATGTGGACGGTTTATTATGATCGTTGGCTTAGGTGGACCAACGTCGATGGAATTAAATTTACCGT
CGGCGATTCTTTTAAAACAAAAATAGGTTTCTTTCTTTCCAATTCTACTTCTTTCTCCTAATACGCCACC
GCCGAAGAACCCTCATACACGATTTTTCCTTACATTTCCTCCCTCCCACACATGCCGGCCGCCACACCTT
CCCCTTCGGTATTTTCCCTCCCCTCCCCTTCGACATTTTCCCTCTTTTTCTTAGTCATCGGCAAAAGGTG
AATTTCTTATAGGTACTTCTCGGTCTGAGTAGGCAAGACTCCAATGATGTAACTAGAAACTAAATAAAAG
TTGACACTTCTTAATAGTTTGATCACCAAGTTGGGTGGTTTGGGATGGGTACTTTAGAGATCATATGAGT
CATGTACATCGTCTTGCTGGTCTGAAGTTTATATATGAGGTAAAGATTCACGAAATAAAATGAAAGTTTA
TAACGTATGTTTTGGACTTGAAAGGAGTATTTCATCGCTTAATCAACCAAATATCTCCTAGTTAAATTTA
ATTGTTTTTAAAGTAGTAAAGCAGATATTCATACTTAGAGGGAGCCTAATTAGTTGTTTAAATATAAAAA
GGATCTCATATTTAGGGAGAGTCCAAATAAGTTTGGTTTGAAGGATCATTCCAAACAAAGACTTGGAAAG
AAATAACTAATTAAATTAAATAGGGAACATTAAACTTTAAGTAGTTTTTATTGTAACTCATTATTGTTAG
TGAAATTTCTCTCCATCAAGCAGACTACCATATATATGTAGGTGATATTACACCGATTTGAATCACCACT
CTATATCGTCACTTTATATTTTATTGTTTCATTTCCTAGGTCGCCTACTTGTCATCTCCTACAACATGTC
TAATTCGTGTTGACCGGTATAAGATTCGTCTATTCTTTTAATTATTATTTTATACAAATATTAAACAAAG
TAAAATTATAAAAAATGAGAGAGTTGATGTTAAAATAAAAACTTTTTAAAAAATATGTATATGGGATACA
CATGGACACACGTGTCCATGTGCATCTTTTTCTATATATATATATATATATATATATAAAGAACATTTTG
ATCGTGCAATTAACTGCATACTGTACAGACTTAATTATTATAAACAGAATTGTATCAGATGGCAAAAAAC
TTTAGAAAAAGACAGTCTATATCACCTCTTTTTTGTATATTGTAAATATGACAAATAATTAATGATATCA
GACGACTATCAAACGACTAGGAGGACTATCAGATGGTTATCCAGTTTTAGATTTACTACTTTTGTAATTT
AAAAATATAGTGATATAGGTTCTATTATCGTAACCCCAAATTATAATATTAAACTTAAACTAAATATTAA
AAGAAAATAACAAAACAAAAATAAAGGGATGGCATATCCATACATATATATGTGTCGCAGTTGACCACAT
GGCCAAATATGCTCCAATACTTTTGTTATTAATTTATTCATAACGTTCCAATGATCATTTTTATTAAATA

ATATAGTTGTATTGAGGGGACAATAATTAAATAAATGGGATAAGGTAAGAAACTATTTTAATAATATTTT
GAAATCATATGCGTTTTAAATTCATTATATTTAATTTATAATAATGTACGGACGGGTCCATTGTGTATGG
TCTATATGTAATATTACTTGTAGGCTGTAATCATGGATAAGATGTTCAAGATTTTTTTTTTTTTTTTTTT
GGTTAATTTTTTATGACAAATATTGAAGATACTTGAAACAACGTGAATCACATTAATAAATTTCAATAAA
TAATAATAATAAAGCCAATTTGATATGATTTTTGGATTTTGGTAAATAGGTTTTGGAAAACTTATTTTTA
TTTTTGAAATTGGGTTAAGATTATAACTTTCAGCTTAAAAGAAGATCCACCTCCTCTTAAGAAATTCTAA
GGAAAGAATGTTCTTAAAAGTTAAAATCCAAACCAAAATTTGAAATCTTAACATAAAGAAAAAAAAAAAA
AAANNNNNNNNNNNNNNNNNNNNNNNNNNNNNNNNNNNNNNNNNNNNNNNNNNNNNNNNNNNNNNNNNNN
NNNNNNNNNNNNNNNNNNNNNNNNNNNNNNNNNNNNNNNNNNNNNNNNNNNNNNNNNNNNNNNNNNNNNN
NNNNNNNNNNNNNNNNNNNNNNNNNNNNNNNNNNNNNNNNNNNNNNNNNNNNNNNNNNNNNNNNNNNNNN
NNGTTTTAATTTATTAATCATCTTGATAAGTTAAAAACTTAACCACTGTTGAATTATAGATGTACCATTA
TTATTTGTTTTAAATATTTTGTCTATAGTAAAATGGTCCAAACATTACAAATAAAAAAATCGATGAAAAG
TTCCCCTTACCCTAATTCCTTTAAGAAGTAATTAATGCACCTTGTAAAAAAAATTTTCTTTAAAAAAATT
ATTAGGATGTTATAAATAACTTAATAAGAATATTATTGAAAAAAAATTTCAATAGAAAATAATTATTTTT
AAGGGTGTAAAATATTTACACTCTAGAAAACAATTTCAAAAACGGAAAAGTTCAGAGGCCCACCGTGTAA
AATATAAAAAATATTCGAACCACACCGTCAATAACGCATGCTTAATATATTTACGATTGTTTAGATTTCG
TTATTGTTTGATACGCGATCATTTAGATATGATTATACTTGATAGTTTATAGATTAGAATTAGTTCAACA
CAATCGCTTTGGTATAAATACAATTTATACGCGATCGTTTAGATATGACGACAATTTATATGCGATCGTT
TAGATATGACTACAACACGATCGTTTTGATATGACTATAATTTATACGCGATCGTTTAGATATGGTTACA
ATTTATACGCGATCGTTTAGATATGACGACAATTTATACGCGATCGTTTAAATATGGCTACAATTTATCT
TTTCAATTTCATCCTTTAATTTTGTTACACGATCGTTTAGATTTGGGGACGCAAATCTAACTGATTTTTT
TAAAAAAAAATTGATGCACAATCGTTTAGATTTATTTACACGATCGTTTATTGATTGTTTACATTTGGCT
ACTTCAATCTAAATGATTTCTTTTTCAAGATTTTTTATACACGATCTTTTATTTTTTTTTACACGATCGT
TTACTTTTTTAAACGATCGTTTACATTTGGCTACTCCAATCTAAATGATTTTTTTTAAAGATTTTTTATA
CACGATCTTTTAGATTTTGCTATTTTGTTGTACACAGTCTTATACAGTCGTTTAGATTTGATTACTCAAA
TGTAAACGCGTAAAAAGAAATAAAAGAAAAAAACGATGGAAAGAAATCGCAGCGAAAAAAATAAGAGGAA
AAGTAGAAAGACGATGGAAAGAAATTGAAGCAAAAAAAAAAAACGAAGAGGAAAAGAAGAAAAATGATGG
AAAGATTAAAAGGGAGAACATGGGGAAGACGAACGAATCGCGAGGAAGAAAAGAAAGATGGAAGGACAAA
TCTGAAATATTTAAAAAATGGCTAACTTTTTGGGCTTTGTTAGACAGGCCCTAAATAGTTTACGGTTTTG
TTACGTAAGTTTTCCTATTTTTAAATATAGTAAAAGTGGAATATTGCAAAACTTTAAGTCTAACTATTAT
TGCTAGCTATAATCTAAAATTTTATCATATTTTGTAAATATTTTCTAACAATTTTGCTACTATTGGACAA
AATGTTAAACTTATAGAAATAGCAAAAAAAAAAAGGAAAAAAAAAGAAGGAAAACACAGGTAAACATTGA
TACACTTCTATCAACTTCTATTGATGTTAGATATGTATCAGCCTCTATCAATGATAAACATTTATCAATA
TCTACAGCCGATAGACATTAATAGATTTTGATCAGCCTCTATTAATAATAGATTTTGTTTATCAATTTCT
ATTATCAATAGACTCTGATAGACTTATTCTATTATTCTCGTTTGTTTAGGCCTTAAAGACGTAGATATTT
TTACTATGAACTTTTAAACAACTTAGTCCATTTATATATATTTTAAACCTTTTATACTATTGAGGAATTT
TTAAATATAGTGAAATTAACCAAAATATTTATAAAATATAGAAAATTTGTTAATTTATCACTCATAGACA
TTGACAGACTTGTATGAATATCTACTAATATCTATCCTGGATAGATTTTGAATTTTTACTATATTTTATA
AACATTTTCGATAAGTTTACCTTATTTAAAGTAATTTTCCAATATTATTCCCTTGAATCACTATAATCAA
TAGAAAACAAATAAATAAATATTGAATTAAGCATATAATTAACACTCACGTACGTCACATCATTTTTTAT
TCCAAAGGAAAATGAAATATAATTCTCACGTGAAATAAAAATACGATAATCAAAGCTGTAAAATTAGCAA
ATACAAGATATTAAGGTTCTAATAAACTATATTTTTTTCAACATTAACGTCACATTAAACTTTTTGATTC
AACCATACTTTTAATTCAATATTATTCTCGTTTGTATATCCTTTTAAATGTTTTGGTTTAGGATATTTAA
TTATAACTTAGTTGGCTAATCATTTTAATTAATTCTCCTTTCATTTATTTATGTTTGTTGATAAGATTGA
CTTATAGTAAGATGATTTAGTGGTGATTTAACGATATCTTGTCATTTCAAAGATTCTTTTTTAATCTACA
TCGTAGAACCTTGATATATAATATCTAAACTCCTAACTAAGAACTCTCTTTCTCTCTCCCAATATATATA
TTATTTTATTTGGTGATAATCACTTGTATAATTAGTTTTAACTTGTAGTTAATATATATAATAATTTTAA
ACTAATTTAAATTAACTCACATCAACTTGGAAGAAAATAATGGATATGATAAATTTACGATATAACATTT
TATAGATTTCCTTGAAAGTCGTCGTCTGTTGTTAAAGTCTAGACTTTTTTATAACATTTTTCCAAAACTA
TAGTGGATGCTATAACAGTGTATGAATGTAATACGTTGCTATATAAAAACTTTTTATAGTGTTTTTTAAA
GGCTATAATATGTATATATGGCTAAAATAATTTGTTATCTTTAACTTATAATAACGTTTTTCAATGCTAT
AATATAATATTGGGGTCTTTTCAAAAATATAAAAAAGCGGCAAAGTATTTACATTGTATGGAACAATTCC
AAAAATGGAAAAAGCTCAGAGGCTCACCGTATAAAATACAAAAAATGTCCCTTTAACAATGTGCATATTT
GGTAACGCGATTTTGGTACACGATCGTTTAATTAATTGGTTACTTTTCAAAATTTGGTATACAATATCGT
TTAGATTTGGCTATACGACCGTTTAGACTGGTATAGGATCGTTTAGATTTGGCTACAGGATCGTTTAGAT
TTGAGGTTCCAAATCTAAATTTTTTTTTCAAAATTTGGTATAGGATCGTTTAGATTTGGGTACATGATTC
TAAATGATTTTTTTTCTTCAAAATTTGGTATAAACCATTTTTTTCAATATTCTTTATACACCATCTGCAT
AAAAAAAGAAGAAAGACGACAATCTTTATACACCATCTACGTAAAAAAGAAGAAAATAATTTTTTTCAAT
ATTTTTAATACAACGAAAAAAAGAAGATCAGTCAATCTTTATTACTATAATTATTGTAGTAGACTTTTAA
TAACATTACTTTTGCACTATATATAAATTTCCTATATAACTTTTACTCATGAGTCATGACTATCAAAGGT

AGTCTTTTTTGTAAACAATATGTTTTTTCATATAATTTACAAGAGTTCTCTAGACCTCTTGTAAACATCA
AACACTTTTGTCCTCTACTAGCTTGAAAATTAAAGTAAAATAAAATAAAACGAGGAAGTAAACTTTGTAT
GAGTGCATCAGTTCTAATTTCTTTCTTCTTTTTTCTTAACGTAACATACCAAGAAAAGTAACAAAAAGCC
TAAAAAACTCATATAGTTAGAAATAGACACCAAATATATTGGATCCAAATTTAAAGTCTTTTGATATCTT
CCCCTTGTTACTTTATGAGTTGAAGTTATGCTTCAAATAGAGTTGTCCCTTCTTCATTTTCTGGTGCCTC
TCTTATCAAAACCTCCGAAACCCTGAAATGAAGCATACCATCTTTCCATCTAATCAAAAAATTAAAACCA
AATGAGAGTGAAAATATAAGGAAAGGAAAGAGTTCTGGTAGTACCGGATATTATATTATGGCCATAATTG
AGGATTTGAAAACTTCCATGTTCAAAATCAAAGGACAAGAAGCAGCAACTTCAAAACTCAGTTTTATTAA
CTCTTCTAAAGCATCTAAGTTTAGAGAGCAAACTTTTGTCTGAGAAGGAATTGAATGATGAGGATTTCCC
AACTAGAACTTATTTACAGCACTTTGAGAGAAACACTTGAAGAAAGTCGAGAGAAGAACAAGTTTTATAG
AAATTAACCATCACACAAATCCAACAAAACAAAATCCGAACCAAAACTCACGATGATAAAGTGAAGATTA
TTTGATTTCCAACATCTCTACAGCAGTTATCTTTCTGTTTTACAACTATTTTGTAACAGATCTATGCTTT
ATATAAATAGATCTCAGTTCATTTAGATTCATTTGCCGAACCACGTACATCTTTATGTTCATCTTCTTCT
TCTCTTTCATTATAGTTGACTGCATGTGTGTTTTCTTCAAACTTTCTTGCTGAAAATCGCCATACAGAAG
AAAAGATCGTTTTTCTTTGAATTGTTTTCTGTCTTTTCTTACCAAGTGTTTTCCCCAAGGTAGTAGACGT
GGCTCTGTCCTCCAATATTTCACGTACCACAATTAAATTGAAATCTGATTCTCTTTCTCCATTTGGGCCT
AAAAGACAAAATAAAAGCCCAACATAAAGGCATTCACATCCATGGATTTTCAATCAATTGGAAGAGCTAA
AAATATTTCAAGGAAAAAGACAACATCTTCCTCTTCAAACTCATGTTTCTATGTGCAAAAGGGACCAATT
TTGATTTTAAAATATAAATGTGATTATATACAGGACTGCACTTTTAAAATTTGTTCAAAGGGTGGGGACT
ATAATGATGAATATATAGCCAATTTGAAGTTCATTCTTCACCTCTTTGAATCTGCTTCTTGTCTCAACCT
CAATTTAAACAAATCCACCATCTTTCCTGGTGGATTCGAGTAGGGCTGGTTATATTGCCGAAAATTGAGG
AATCAAAAAACAATTTTTGCGGATTATTTATCTTGGGGTTCCCCTGGGAGGCAAACCATTTTTGAACTTC
TGGGACAGCATCACAGAGAAGATCCAAAAGAACCTGGGCAGTTGGAAATACTCTCTCATATCTAAAGGAG
GCAAAATTACCCTCATCAATTCCACCCTTGAAAGCCTTTCCACTTATCAGATGACACTTTTTAAAGCTCC
AAAGGGGTTTATAAAAATATTGAGAAGAGTTGGAGTGATTTCCTTTGGAAAATATACTGACATCTTATCA
GGTGGTCTAAAATTACTATGCCAAAAGACTTGGGAGGGTTGAGCATCACCAAGGAATGCGTTTTTATCCA
AATGGCTTTGGAAATTCATTTCGAACTATGAAGCGCTTTGGAAAAGATTGATTTTTGCCAAATATGACCA
AGCCTTCATCGGGGACTTTCCCGTTAAAGGAAAATACGGCAGTGCCAAAGCTCCTTGGAGATCTATTGTC
AAAATGATTGACTGGTTTCCGAATTAATACTCTTGGAAAATCAATAATGGTAATTCGGTCTCTTTTTGGC
ATGGTAAGTGGAATAATAAATCTCCTCGGACTAGTTTTTCTCCAAGACTTTATGCTCTCTCAAATCTTCA
ACAGGGAAATGTTAGTGATATGTGGAATTCCGATTACTCTGATTGGGATTTTCAGCCACGTAGACCCTTG
CGTGACCATGAAATACAAAGGTGGACTGCTCAAAAAACTGATCTAGCTGCCTGCTCCAAATCAAAATCGT
GGTCCTGATCCCCCGGTATGGAATCTAAACACAAATGGAACTTTTGATATTGCATCGGCTAAAAAGGCTA
TGATTTCCATTTATTCGGGTAATGTTCCTATAGCTCAGGCCAGCACCTTCAAGACTCTCTGTAAATCTGA
TATTCCCAAAAAATGCAAGTTCTTTATCTGAACTCTGATTGATGAATGTATAAATACTGCCGACAAGCTT
CAAAAAAATATTTCTAAGTTGGAGCCTAAATTCTAACCAATGTGTACTATGCAAAAATCATAGGGAGGAC
GTAAATCACTTATTCATAAATATATTGCTCCACTTTCCAAGTTCTCTGGTCGAAAGTTAAATTTGTTAAA
GTGGAATCATTCTCCAGCCAACCTCGGCCTTCTATGTAAGGATCTATGCAGCTTAAATACCAACAATAAG
AAAGGGGCTATCACCTTTAACACTATTGACGTCACGTTATGGATGATTTGGCTTGAAAGGAATAACAGAA
TTCTCAACTGACTCGAGCAAAGGTGCTGTCGAAATTTGGGATGATATTCTTACATTTACGGTTTCTGGAG
CAGTAGATCTAAGCATTTCTTTAATTATAGTGCTAGCTCTAACTTTAAACATTGCGGCATTTGTTTAGGT
TTTTCGTTGAGCTCCTCTCTAGTCCTTTTTTTCCTTGCTTATTGTATTATATTAGTTATATATTAATGAA
GCAAGAGCGATGAAGGTGCTATGAAGATGTTCACTTAGTGGAGATGTCTACGCCCAACTAACCAAAGTAT
CTTTTTTCGAGAAAAAAAAGATGGGGACTATAAACAGAGGTAGAAAATAAAGGAAATAGACTTTTCTCAC
TCGTTGGGGCTGGTCCTTAATTGATGCTCGATCCCCAAACCAAAGCACGGAAAACCAAAATTTATAGATT
TTAGTATGAAAAATCAAACACTTTGTTAATAGGCATGTAAAATTGTTATTTAGTCGAAGAGTTGGCACCG
ATGATATAGAATTATATTCAAAGTTCAGATATGTGTACTAACCCACTAAAAGGACATCATGACACTGTTG
AAGTTATTTAAGACAAAATTTGTAAATTCAACTCAAAAACATAATAAAATACAATACTAAATTCTAAGTT
AAAAAAAAAAGACAACAAACCCAAATGACACAGAAATGCTAGTCAGCATCTGAATTTGTATTCATTGTTC
GGATCTGCCACATTCAAGCTTGGTTGGACTTGGACCTGATCAAAACCTCTGCGATCTACTGTTCACGCTT
GGAGCTGTATCATTAATCTAATGGATTTAGTGATGTGGGTGAGGCTATTTCGTTGAAAGAGATCATTTGC
CAAGTAGTGTAGAAGTAGGCCTTCAACCTCAATAGGAGGAAATTGCAAAAAAAGAAAAAAAAAAGAAAAA
AAAATCATCTGCGATATGCGCAAAAGAATTTCATTTAGACTAAAAATAAAATGTTCTGCCTTCTTTTCCA
ATCTTTTCAATAGCCTATTTTATAAAAAGCTATATTAAGAGGATATAAAATGTGTTAAAGATTAGGCCTA
GAAAAGAAAAATTAACCTTCGATACATATAACCTAGGATCATACCTCAAATTCAAAATTAAGACAACAAT
TATTGTCTCTAAGCGTGAAAATGCAAGATGGGAGTTAGAGAAAAAGCTCACATTTTTGCATTAGATATCA
ATGGAGATATTTTTTTTTTTCAAAACATATATATTCATTGTATTTCCTTTATCAAGTTTGATTATAGTTT
CAATAATCATTTTCTCGAACTAAAAAAACCATTTAGTCTTCATTTTCTTTTCCGTAGAGTGTATCCTTTT
TTTCGCAGATCATCGGAGCTTGAGAAAGGACTGATATCTATCTTATTATAGAAACAAGAAAGAGTTTACT
CTAGCTTCATTCATCAAAGAACCGACTGCTCTTGTTCATTGAGACAGTATAGGTTTCCGTTCTTGCATTA

GATAAATAGGTTTCATTCCCCGTTCCATATTGAATAGTCTACCTAAGCCTAAATAAGAAAACAACCCTTA
AAAATAAACATAACAGATTTTCTTAAATAAGAAAGAATTGTCGGATAACATTTGCAATGTATTAAACAAT
CTGTAAATATCACAAAAGAAAAAAAAAAAAAAAATCATCTCCAAGAAGAAAATGAAGAACAATAATTGCCA
GCAACCTCACTTCAAAAAAACACCAAAGCTAACAAAGAGTACACAATGGATGTCCATATCATAACTATAT
TAGCTACTTTAATATACGTATGTATATATATATATTTTTATTTTTGTTGAAGGGACACATAGGACTTAGG
ACAATACCTAAGACCAAACTAGAATTTTATTGATGAAGGGCTAGAGAAAAGCCCAAACACTAATTACAAA
AGACGCTAAGATTAAGAGCAATTGTAGCTGCTGAATAATTTTTAAAAGTAGGATCTCTACTGCACCAATT
TCCTATGAGTATTTTGCAATCTTCCCACATGTTAGCAATTAATTTTTGATAACTTAAAGTACCAAAAATT
CTATTATTTCTCTAAGTTCAAATACCCCAAAGGATTGCTATCAGCCCACAAAAAATGACCTTGTGCTTTG
GGAGGGATCGGTTTAGAGAGAGGATGAAGGAGAACAACGCTTCAAAGTCGTCGGAATTAAGCGCGAAGTT
GAGAGATCGATGGAGGAAGGACCACAGGGGCTTCACCGCTTCACAATGAAGGAAAAGGTGAGCTCCCGAT
TCACTTTCTTTTTTGCAAAGAACGCACCAATTAGGTTGAATTAAAGAAAATTGAAAGAACTCAACTCAAC
GATAATTTGTAGGATCTTTCATTTATGAGGTGTGAAGTTCGATCCATCATCTTCACAATTGTTTTGTATT
AAATAAATTGAGTCAAAATGATTGAAATTAAAGTGAATTAGGTTGTGTATTTGAAAATAAAATTTGAAGT
GTTGAGTTTGTGATATTAGAAAAATAAATATAGTACTAAAAAACGTTTTTCTTCCCGAAAATCTTTTATT
TACATGTTAACAAGCCAACCGAAAAGTACCAACTCCTCCTAAGATAAATATTTGACTTCTTAATTTCATT
TTTTTAAAAAAATTTAAGTGTATTAATACTATTTTTTTTCATATCTATTTTATTAATATTTTCAAAAATC
AAATCAAAATTTAAAAAAAAAAATTCAAGAAGATAAATTATTTATTTAAATGTTAAATTTTGAAAATATT
TACAAATATAACAAAATATCATAGTTTATATGTGATAAACTATGATAAATCAATATAGAATTCTATCGGT
ATCTATCATAATGCAAATAAACACATATAGATTTGTTTTACTATATTTTGTAATTATTTTAGTTCGTTTT
GCTATAAATACTCTTTTTTTTCATAAACCTTTCAAATTTCTTTTTATTTTGGATAAAAATAAAATATTTA
TAATTAAAAAAATGTAAATCACTACAAGAAATTGAGTAAAAGTGAATTTTTAAAATAAAATGGTTATCAA
ATAGGTTCACTATTTTGTTTCTTTTTTATCTTTTTCAATTATTTGAAAATTGTGTTTATTCCCACAATTT
TTATGTTTATAGCTTTTATACTTTTAGTGTAAACAAAAATAATTAACTATCGTTGGATAACTATTTAGTT
TTCTATGTTTGTTTTTGAAAATTAATTAAGTCTATCTTCTTTTTATGTTTTAATATGGTTTATATTTTTC
TTAAATACAATGGTTGAATTCTTAGTTAAATTTTAAAAATAAAAACAACTTTTTGAAAGTTTTTTTTTTT
TTTTTAATTTTAAAATCTGATCACTTTGTTTTTTAAAGCATCGATAGAATGTAGATAGAAAAAGAAGAAA
GTTGAAGATAGAAGTAGTGTCTATAAATTCTATTTTAAAAAAACAAAATGATTAACAAATGGGATCTAGT
TTTTAATTTATTTTAATTTAATTGATATTCAAAATATAAGTAAGAACAAATCGTGCGAAAAGAACTCGTA
TTAATAAAAACAAATCGTGTGAAAAGAACTCGTATTAATTTGTAGAGATAAATTTCAATCTTTGCAATAT
TCAATGGTTAGGCAGTGAATCCAAATACATTTATATTTCATCATAGATGGTGATAATAATAATAAGTATC
TTGCAAGGACTGCAATTAATAACAATGTCACATTATGATATTCCTTTGGTATAGAAAAAAGTATGTTGCA
GGAACTGCAATCTGATATTCCTTTAATATTTTATTTATTTTAATTATTTAGTAATTCTTGACATTTTTAA
AACGTCGATAAACTTGGAAAATAACTCCCTCCGTCCTTTTATAAAAATTCTTGACATTTTAAAATATCAA
GAATTTAATTGATATAATTTGACGTTTTAAGAAAAAGTACATATTACTTGACGTTTATAAAACGTCAAGA
ATGTTGAATTTGTAAAGATTCCTGACGTTTTAGAAAATGTCAAGAATTTAATAGATAATCCTTGACTTTT
TTAAAACGTCAAGATAAAATACATATTACTTGACGTTTAAAAAACGTCAAGAAATCTGAAAATTGACATT
CCATACAAATTTTTGCAGTTTCTCCTTTCAATCATCCATTTCTTGACGGTTTTTTCAAACAACGTCAAGA
AATAAAAACTAAAACCATCGAGAAAGTTTTTTTTTTGCTAGTAGTAGAAGAAAATCATCTGCATCTCAGC
TAAATATGATCTTCATCAAGTTGGTGATATTCCCTCCAAAAGCAAGTTCAGCAATTTTAAGGTCCCTTGG
AGTTTCATTATCAAGGGCCTGGATTGGCGTCTTTCAAATGTTAGATGGAAACTCAATAAGGGTGAAAGGA
TCTCCTTCTGGAATGGTACTTGGTCTGTTAGACCTCCTCTTTCTCTGTTTAAACCAAAACTTTATGCTCT
CTCAACCCTTAAAAATGGTAATGTCAAAGATCTTTGGAACCCCATCTCCATTAATTGGGACATTAAACCA
AGAAGACCCTCAAGGAGTGTCGATTTACAGCAGCTGTCTGATCTAAAGTCTGTCATTTCTGCTCCAATTG
CAAACGGTATTCATGATGTCCCCATAAGCAAACTTGCCTCTGATGGGTCCTTCAATGTTGCTTCTATCAA
GAAAGCTCTACAAACTGATGAACAGAATCAAATTGATGTCCCTGAAGCCATTTTCATAAATTTATGGAAA
GCCAGCATTCCTAAAAAAATGTAAGTTTTTCATCTGGACTCTTCTTTATGAATGTATCAACGCATCTGAA
AATCTCCAAAAAAAGGTTTCCATACTGGTTCTTAAACCCAAATCGGTGCTACCTAATATGCAAGAAAGAA
TCGGAATATTTAAACCATATCTTTATCTCTTGCGAGTTTGCTTAAATCCTTTGGAACAAATTGTGAGGAA
TTCGATTCAGTGGCAGAATGCATATGATAGTACTGAAGCCAATGCATAAAAATTTATTTGATTTATTATA
TACAAATTGTTGGTGAAATTAGCCAAAAAGAACTACGACTATCAAATATTTGTGAGCTAAAGTAATTTAA
AATAAAGGTCAGGGTCAAATTAATTAATTAGTGGGTATAAGGGTGGTTGGCATCGGAGTTGGGGCGTGGA
AGTGATAGTGGGACTTTGGGCAGAGGTTGCGGCCAAAAAGTTGGCCGGCGGAGGGTACTCTTGTAGCCAC
GACAAAAGATACATAACGTTTTTCTTCACTCTATTTTATCCGTTTGAATTTTCAAACTACAAAATGAAAT
ATATATATATATATATAGATAGATATGTAGTACTACGAAACGTTTTTCTTCCCTCAGATCTAACACTACA
AGCCAACCGACATTATTATCAATCATACGTGTATATTTTTTTTTAGTAAAAAAATGTCAGGATTATTTTTA
AATATCGTAAAATTAAAAATAAGTTTTAAAAATATAACTAAACGAAAAAATATTTATATTGTATAGAAAA
ATTTTGAGAACGAAAAAACTCCACATGGTCAGAATGAAAAATACAGAAAAATGTCTCAGTCAAGAAGCGA
TTAAATAGACAGTTGCGTGTGTAATATACTTGGTACACATTCTTATATCAAGATCATTTAGATTTGACTA
CATTATCGTTTAGAATTGACTACTCATTCCAAATCTCACGATCGTGAATCAATATCCCAACAATTCTTTT

TTTTTTCAAAATCTTTTATATTTGGTACACAATCTTGAACCAAATAACCCTTTGAAAGAAAATAATACAA
GGTTCATGATTAATTGAAAATAAATATCATGATTTGAAAAAAATATAAAAAAAATTGCAGAAGAAGAAGA
GAAAAAGACGATGGAAAGGAAAAGAAAAATTGTAGAAAAAGAAAGAGAAAGAAGATGGAAATGAAGGGCA
AACCTAAAATATTTTTAAAAAAATGGCTGGCTTCATGGCTTTTTGATGTTTACCCACATCTTAAATATTT
TGGTGTTTTGTTATATATATACTAATGAATATAATAAAATATCATAGATCATTTGCGATGGATCGTGATA
GGCTACTATCTGTATTTATTTCTCTATCTGGATTTATCTTCATCTATTATAAATAGATTTTCACATATTG
ATACATTTTTTAAGATTTTTAGAGAATTTTCTTTTAAACATTTAACTAGTATTTGTTCGATAATTATTTG
TTTTTTATTTATTTTAAATTATTAAAAGATTTAGATGTATAAATACTCCTTTCTTTAACCTCTAAATTTT
TAAGGTGGTTTTTTTAAAAAAATTTATTAATATTTTCAAATCAAATCAAAATGTCCAAACAAAGTAAACA
AATTAAAAACTTTTTTTTTTCGGAATTCGGATAAAAATACGACATTTATAATTCAATTATTTGAAAATTG
TGTTTCTTCCCACCAAATCTGGTTCATAGTTTTTATATTTTGAGTGTAAACATGATTTCTAAGGCAAATT
CCAAAAACAAACATCACAAAGTTTTTCAAAACTTTTTTTTTCTTATTTAATATCTAAAATATGATTGAGG
ACAACACATATTGAAAAAGAACTCATATTAGATTTTTGCAAATACTATCGCTCTTCGAAACACTCAAGAC
AAACAAAAACAAAGTAGCCAGATCGTAGAAAATGTTAAAATTTGTTAAAGTTTATCAAAAGTAAAGATGA
AATTTTATATTTAATAATTCAGAAAAAATATTTGTCTTTTCAATGTCTAGAGGTTAGTAGGTGCATATGA
ATATCTCAACTATATTGACGATCTCCATAACGTCCTCGTCACTCCTGGATCCAAAAATGATGCATATTAA
TAAGAAAAAGTGTACAAAAAGAACAAACACATCAAAAGTTTTCAAGAGGGGTGCAACTAAAGAAAAACCT
TTCGATTCAACCGAAGTACAAAATCCTATCATAGTTACGAAAAAGAGATGGTTTAGAGTTCCATAGGGCA
GCAAGATCGAGGATGCCTTCCTAAGAGGTTCTTTAGAGGAATTTGCCATCGTTTTCAAATGCTCCATAGA
TTCAAAAATATTTGTTTGAAATATCATATGCCTCGTCTAGTTTCAAGTTGTAAACTAATTTGTGTTTAAA
ATTAAATGAATATTTAATAGATCCAATAATTTTTAAATTTTCAATTTTGCATATACATGTTGAATTCAAG
TTAAAGGTTTAAGAATTTACATAAAAGTTTAAAGTTGTATCGGATATTTAAAAAAATTTAAAGATTAAAT
AGAAAAAGATTTTACATTCAAAATTCAGAGACTTAACTTATAATTTAATCTATAAGTAAAATAAAATAAA
ATTGAAAATATTTAAATAATTGATGTCGCAAATATCCTTTTTTAACTTAATCAAATTGTACTATAATGTT
TCTTACCATGTTTTCAAATATAGTAAAATTGTTGAAAATATTTACAAATATAAGAAAATTTTACTTTTTT
TTAGGATTGTTCGTATTGACAATAGAGGAAAGTTATAATGTAGGATTGTTTTCGACTCAAAAGATTTACA
AATACACCAAACTAATTTCCTTATAAAAGTATCATTATTATTATTTTTTCCTCTAGAGGTGAGAAAATGT
GATTTCTATCAAGAAATTTCCTTGTCCTCTTCTTATGTGTTCTATGTATGTGTATATATTCTTTTTTAAT
ACAACATGTGGATAGGACCTTCAACCTTTCACACCTGTTGATTAATGTTTATATTGACGAAAATGTCAAT
TATAAAGTTGGGGTTTTTTTCAAGTGTAGTAAAATATAAATCAAAATATTTATCGAATATAGTAAAATAA
TATTCTTATAGTGCAATTTTTTTCTAATGGACACGTTTAAAAGAGGTGAGAAAATGAGTTTTCTATTATC
AAATTTCCTTGTCGTTTTTCCACATACCTATATGGATATATATAAAATAAATGTCCCTGTTCTCTTTCCA
CACACATACAAAAGTACACAATGACGTTGGTAGGAGATTGAACCTTCCTCTCAGTTAAATTATGTCCATG
TGTTAGTGAAATGATAATTACAACAATAACAAAAGTGAACCAAAATATATTTACAAACATGATAAAATGA
TTTTCTTATAAAATATTTTCTTTGACCTTTTTAGTCCTTAGAATATTTGCTTTTCGTCCATAAGATTTTA
AAATCATTTTTAGTACTGAAATTTATAAGAACGAGCTATTTGATCCTAGAGTTATAATATTTGAATGTTA
AAACTATTTTTTTTTTAATTTAAATATGTCATATAACTATTTTTAAAAAACTAAAAAAAAATACTTCAAG
AACCTTTTTAATCTATTTTTAAAAACTCAAAAACTAGGAAAGACATATTTTGAAAACTCAAGAACCAAAT
GAGTTGATTCCTATAAATATGAGGACTGAAAAACGACATTTTCAAAACTTATAGATTAAACACATTTATC
CTTCAAAACTTGGTAACTAAGAAGAAATTTCCATTATTTTTTTTTTCTTCTATAGGACACGTTTAAAAGA
GGTGAGAACTGAGAAGATAAGATTTCATTTAATTAGAAATTTACTTGTCTTTTTTCCTTATATTAATTTT
CCCCCAGTTTCATGTCTGTTAAAATAAAGGCTAGGTGATCTTCTTGTAGACTAATAATTTAATGCATATT
CAGAAATGTGAATGATTGGACATTATGGTGAGCCCCACTTCATATATATATATATATATATATTTTCATT
ATCCTTATCAGTCAAATGAAAGAGAGAGGACCAAAGTTCTTCAGTTTTTGCTATAAATACAAGACAAAAA
TTCATTCATCCTCACAACACAGCTCATTGTTAAAGCTATATTAAAACAAAAGAGAAAGAANNNNNNNNNN
NNNNNNNNNNNNNNNNNNNNNNNNNNNNNNNNNNNNNNNNNNNNNNNNNNNNNNNNNNNNNNNNNNNNNN
NNNNNNNNNNNNNNNNNNNNNNNNNNNNNNNNNNNNNNNNNNNNNNNNNNNNNNNNNNNNNNNNNNNNNN
NNNNNNNNNNNNNNNNNNNNNNNNNNNNNNNNNNNNNNNNNNNNNNNNNNNNNNNNNNNNNNNNNNNNNN
NNNNNNNNNNNNNNNNNNNNNNNNNNNNNNNNNNNNNNNNNNNNNNNNNNNNNNNNNNNNNNNNNNNNNN
NNNNNNNNNNNNNNNNNNNNNNNNNNNNNNNNNNNNNNNNNNNNNNTTTAATTTAATCACTCTCTTCATT
TTTTTTTTTTTACGGTTACATTACGATTTAGTCTCTTAATTTTCTATTTACACTTACAATTATCCCCCTTT
TAACTTCAATAATAATAATTATAATAATAAATTATAATAAATAATATATCTCCAAACTATCAGTTTTGTG
CTAATAGAATTGTGAACATAAAAAATGTCACATCATAGTTTCTAAACAACTTGAATTTTCTTTTATTTTT
TCGAATGGGAGTGAATAAGGGTTAAATCTCGGTAGATCATGATAGAATTATGTTATTTTACAATATCAGT
TGAATTATGTCCGCACAGGCGGTCCCTCGACTCATTCGTCATGAGGGCTTAGGCAATAACATATGTGCTT
TTGTGAGCTAGAAGGTCCCTACTGCTCATCGACAATCATACAATGAGTGCATGCGTTTTGGTCCCATTTG
ACATATTTAAAATTTTAAAAATATATCGCATATAAAATCAATTATTTTCTCGTTGAATTTTAGTTGATGG
ATATTTTGAGAAACTATTAATTATTGATGTAACTAAACTTATATTAAACAATTTTTTTAGTAACAAAATT
TAGAGACTTTTATGACCCAAAATTGAAAACTAAAAGATCGTTTGAATGTAAGTTAGGTCGAGTTTGATTG
AATGATGTTCTTTGATGATTAATTAAAATTTGTAAAATAATGAAATTTTGACAGCTGCACTTGACTCAAG

AGGGAAAGTTGGGAAGAAGGCATTTTTGGAGAAATGGCTAACTTCAATTCCACCATTGTTTGCTGGAGAA
TCAGTGTTTCAAGTGAACTTTACATGGGAAGATGGTTTTGGATTTCCAGGAGCTTTCTTTATTCAAAATG
GACATACAAGTGAATTCTTCCTTAAATCTCTCACTCTTGAGGATGTTCCTGGCTATGGCAGAGTCCATTT
TGATTGTAATTCATGGGTTTATCCTTCTGGAAGATACAAGAAAGATCGTATCTTCTTTGCTAACAACGTA
AGTGTTAAATTACCCACTAATCCCTCAAACAATCATGCTCTGTTCAAAAACAAGAAAATAGAAATAATTA
TTTTTCCCTTTCTGTTTTACCTCACTCTCTTCTCTCTTGCAAGGAATTATTAATAATTTGTATATTTGTT
TCTGTGTATAGACATATCTTCCAAGTGATACACCAAATCCTCTTCGTAAGTATAGGGAGGAGGAATTGTT
GACCCTTAGAGGAGATGGCACGGGAGAGCGTAAGGAATGGGATAGAATATATGACTATGATATCTATAAC
GACCTTTCCGAACCCGGTGATGGGCGTCCCATTTTTGGAGGTAGCCAATTCCCTTATCCTCGTCGTGGAA
GAACCGGTCGACCACGAGAACGGAAAGGTAATTAAGATTTAATATTAATGTTGATCCCATTTGATAACTA
TTTAGTAATTTATGAAATTACTTTACCCAAATTGAAAGCTTAATTTATGAATTTTTTTGTTGTCAGATTC
CAACTATGAGAGTAGATTGCCGGTGGTATCAGGATTAAACATTTATGTACCAAGAGATGAAAATTTTGGT
CACTTGAAGTTATCAGATTTTCTTGGATTTGCATTGAAATCACTTGTATCAACCGTTCAACCAGCACTTG
TAAACATAGTAGATTTTTCACCAGGAGAGTTCGATAAATTTCAAGATGTTCATAATCTTTATGAAGGAGG
ACTTCCTGTTCCATTGGATGTCTTTAGAAATCTCACTAAGGATTTCACACCTCCAATGTTCCAAGAACTT
CTTAGGACCGATAATGACCAACGCTTTCTCAAATTTTCGCCCCCACAAGTTGTTAAAGGTATCCTATATA
TAATGCTTCGATATTAATTTCAATTTCATTATGTTAATTAGCACATACATTCTTATAGTGTGTTGAATTA
GATAGAAAGTTTGGAGTCTATTTTATGTTTTCAAATTTATTGAAATAATACTTACATTTTTTACTCTAAT
AATTACAAACAGAGGACAAGTTTGCATGGCAAACAGACGAAGAATTTGCAAGAGAAATGCTAGCTGGAGT
TAACCCTCTAATCATACGTCGTCTTCAGGTAAAAAGTTTCATCTTTTTTATTCTCTATTCAACTTTACAC
ATTCATTTTCTTTTCAAAGAATTATATATTTTCTTTTGTATATGTTATGTAGGAGTTTCCTCCCAAAAGC
AAACTTGACCCAAATATGTATGGTGATCAACATAGCAAGATTACTAAAGAAGACATAAAGTCAGGCTTAG
AAGGTCTCACAATTGATGAGGTAAATATTAAAATCACCTAAACTTTTGTGTTTTTTTTCAAATTATATTA
TATTATTATAATTTAATTTTTTTTCCTATCTATATATGTAGGCATTGAATCAGAAGAGACTATACATATT
GGATCACCATGATGCATTAATGCCATATCTTAGAAAAATAAATTCAACAAAAACAAAAGCATATGCCACA
AGAATATTGCTATTTTTGAAAAATGATGGAACTTTGAAGCCATTGGTTATCGAGTTGAGTCTGCCACACC
CTCAAGGTGATCAGTTTGGTGCAAATAGCAAACAATATTTTCCAGCTGAAGAAGGAGTTCAAAAGTCAAT
ATGGCAATTGGCTAAGGCTTATGTGGTTGTCAATGATGCTGGTTACCATCAACTTATCAGCCATTGGTAT
GTTTAGAAATAATTAATTACTCATCAAATTAAAATGTTTTATAATACATATGTATGGTTGTAATTATTTG
AAATATATGATGTAATTAACACTTGGAAATTATTATTGTAGGTTGCATACTCATGCAGTACAAGAGCCAT
TTGTGATTGCAACACATAGACAATTGAGTGTACTTCATCCAATTCATAAGTTACTTGTTCCTCATTACAA
AGACACTATGTTTATCAATGCATTTGCAAGACAAGTTCTTGTTAATAGTGATGGTCTTCTTGAACAAACC
CATTTTCAATCAAAGTATGCCATGGAGATATCTTCTCACATATATAAAGAATGGAATTTTCTTGAGCAAG
GACTTCCTACTGATCTCATCAAGAGGTAAATTACAAATTATACACAATTAAGCCTCCGTTACAANTATAT
TTTCTTTTGTATATGTTATGTAGGAGTTTCCTCCCAAAAGCAAACTTGACCCAAATATGTATGGTGATCA
ACATAGCAAGATTACTAAAGAAGACATAAAGTCAGGCTTAGAAGGTCTCACAATTGATGAGGTAAATATT
AAAATCACCTAAACTTTTGTGTTTTTTTTCAAATTATATTATATTATTGTAATTTAATTTTTTTTCCTAT
CTATATATGTAGGCATTGAATCAGAAGAGACTATACATATTGGATCACCATGATGCATTAATGCCATATC
TTAGAAAAATAAATTCAACAAAAACAAAAGCATATGCCACAAGAATATTGCTATTTTTGAAAAATGATGG
AACTTTGAAGCCATTGGTTATCGAGTTGAGTCTGCCACACCCTCAAGGTGATCAGTTTGGTGCAAATAGC
AAACAATATTTTCCAGCTGAAGAAGGAGTTCAAAAGTCAATATGGCAATTGGCTAAGGCTTATGTGGTTG
TCAATGATGCTGGTTACCATCAACTTATCAGCCATTGGTATGTTTAGAAATAATTAATTACTCATCAAAT
TAAAATATTTTATAATACATATGTATGGTTGTAATTATTTGAAATATATGATGTAATTAACACTTGGAAA
TTATTATTGTAGGTTGCATACTCATGCAGTACAAGAGCCATTTGTGATTGCAACACATAGACAATTGAGT
GTACTTCATCCAATTCATAAGTTACTTGTTCCTCATTACAAAGACACTATGTTTATCAATGCATTTGCAA
GACAAGTTCTTGTTAATAGTGATGGTCTTCTTGAACAAACCCATTTTCAATCAAAGTATGCCATGGAGAT
ATCTTCTCACATATATAAAGAATGGAATTTTCTTGAGCAAGGACTTCCTACTGATCTCATCAAGAGGTAA
ATTACAAATTATACACAATTAAGCCTCCGTTACAAATACACATCTTAATTTTTAATTAGTTGATCAGTTA
CCTCCGGTGGAGTTTATTAGTCCCCGTAGAGTGATAATCATTCCTAGCTAGTCCCAAAATTCAAAAGTGG
TTTGTGGAATTTTCCCTTATAACCATTAAGTATATTACAAAAATGAACATTTTCTTATCCTTAGATTATT
TATGTAGAGGTGTAGCAGTTGAAGATGTAAGTTCACCACATGGACTTAAGTTACTGATAGAGGATTATCC
ATTTGCTGTTGATGGGCTTGAGATTTGGTCAACAATCAAAACATGGGTTACAAACTATTGCTCTCTATAC
TACAAAGATGACAATGCAATTCAAAATGATGTTGAGCTTCAATCTTGGTGGAAAGAGGCTAGAGAGAAAG
GTCATGCTGATAAAAAAAATGAAACATGGTGGCCAAAGTTGCAGAGTTTCAACGAATTGGTCGAAACATG
TACTACCATCATATGGATATCATCAGCTCTTCATGCCGCAGTTAACTTTGGACAATATCCTTATGGAGGT
TTTATTCCAAATCGACCAACTATAAGTCGTAGGCATATGCCTGAAGTAGGAAGTGCTGAGTACAAAGAAC
TTGAATCAAAGCCTGAGAAGGCTTACTTGAAAACAATCAATTCAATGTTGCAAACACTTCTTGGAGTTTC
ACTAATTGAAATACTGTCAAGGCATGCTTCTGATGAAGTCTATCTTGGACAAAGAGCTAGCATTGAATGG
ACTTCAGACAGAGCTGCATTGGAAGTGTTTGAGAATTTTGGAAAAGAAGTGTCTGAAGTTGAAAATAGAA
TTATTCAAAGGAACAAAGATGTGAGTCTCAAGAATAGAAGTGGACCTGTTAATGTTCCATATACTTTACT

TCTTCCATCAAGTACCGAAGGACTAACAGGTAGGGGAATTCCAAACAGTATCTCTATTTGAAGGGGCACT
TTGAGCATATGGTTAGATTATAAATATTTCGCGTTTCCTTTGTATTTTATTGTAACTTTCTTATGTTTGA
AAAAATGCTACTTCTTTGGCTACTCATGAAATAAAATCATAAAAGAGTGGGCTTTAGAGTAGCTTTGTAC
TTGATATATTAATGAATAAAAGTTATGCTTTTAAAGTAATGTGTTGTACAATAAGCATACTTCTTAAACT
AGCTAAGTGATTATATATTAAGGAGAAAATTATTGTATTGTTTAGATATTATTTTATTGTATTTCTTAAA
CAAGCCTTAAGAAACTTTTGAGGTCTTGAGGCAAGCCTCAACCCTCAATTTATTTATAACCAAATGTAGT
TACAAAGGCTAGAGGAGAGCCCCACACTACTCTATATAAAAACGTTGATATTTAAAGCAATAGAGCAATG
ATTATAGTCCTTGAAGAAACGATGTCTGCTTGGCACATGCCCACCAAATTACAGATGTTCACCTAACTAT
TGTGTAAGTTGTTGAGGACATTGTTCAAAATGTAATTGTTTCTATCTAATCAAAGAGTCCATAGAGTTAG
CCGCACAGATGTTGAATTTGATGATGTTGGCTGTGTTCTTCTGGTTTATGGAAAATATTTGGTGACAGAG
GTCGGCGCAATTGAGGTTTCTTGCATTGCGAATCCCGTTTCTTCAAGAATTTTCTTCCAAATACTTTTGG
CTTTTGCACAATATGAACGAAAAGGTGATTGATGTTTTCCGCATCTGCCCTGCAAAGTGCACACCAAATC
GGGCTAAGGTAGGAATTTGGATTTTTCTTTTGGATTTCGTCCATTGTGTTTAATCTTTCATGAAATACTG
TCCAAAGGAAGAATTTACATTTCTTTTGTATTTTTGACTTCCAGAGATAGTTGAATGAGATTTGTTGTGG
GTTTGGATTGTGGAAGCAACTTTCTTCGTAGATGGCTCTCTTCCCATGTGTTTATGGTTAGTATTTTGTT
TCTCTCTCTGAAGATTTTGCTGTTTCTTTTGTTCCATATTGACCAAAGAAGAGCAGCACCCCACTAAAAT
TGATGATATTTTCCTCATTTGTTTGTTTGAGTGAGCACATAGCACTTTCAAGGCATTTGATATTGCTTTT
GGTTTGCAAAATCACCCATGTTTGAGTGTAATTTATTCTAGAGAGAATTGGTTACCTTGCACTTGAGAAA
TAGATGGTCCATATCCTCACTATTCTCCTTGCATAAGACACAGATATTTTGATTCACGCAGAAATTGTGA
AACTTCTTTTGTATATATGACATCTGTCGTATTGATTTTTCCTAGAAGAAAGGCCCAGTGGAAAAACTTA
CATTTCATTGGCAGAGAAGATTTCCATAAAATTGAGAAGATCTGCCCCTCAGTGTGTTGTGTGTTATGTA
AAGCCTCTGAAGCTAAACGTGCGTTTGATATATAGAGGCTATTGAGAAGCCATTTTGGCTTGCTGCAACC
TCTGTCAGGGGTAGGAGTTTGGAGCTCACTTTTGATTTGGTTTCAAGTACTTGATTCCCTTTCATTAAGG
GGTCTTCTTGTGCCAGAGATTTGTCCATCTATCTTCGATTGTTGCATTTTGATCCTTGACTGAAGTAAAC
AATCTTGGAAAGGCTTTTGATAGGGGACCTCTGTTTGTCCAATTAGCATGCCAGAATGATAGTAGGTTTT
CGCCATAGTCAACTTCCCATTTGTAACTTCTCTTAAACCAGTTCAGCCCTTTCGCGATAGAAAGCCATGG
AGCTCTAAAGCTGCTATATTTACTTTTAATAGGGATCTCTCCAGCATAGGAAGCTTCATGTTTGGCTTCC
AAAGAAGACGAAACCATGATGCATGCATACCTAATAGCTTTTAGCAACCAACCATAGCTATCTAAACTAG
CCAATTTAGTTGCTTTAACTAAATTAAACTCTCCTTTGAAAAAAAAAAACTAAACTAAATTCAAAAGTTA
ATAAGAAGTTGAATCAAACTTTAAAAAATGAATCATCAAATGAAATTGGGAAATTTATGACAAAATTAGG
AGGAATGATAAAAAATGGCAAAGTGGGCAAAATATTTAGGTCCCATTTGGTTCGTGATCGAAAAATAATT
TTACAAATCAAAGAATTTTGTGAAAATAAAAAACAAATTCTTATTTTTCATTGTATTTGAAAACACGTTT
TGTTATTCCAATTTAAAATTTTGAAATCGAATTCAAAAATCAAAATTTGTGTTTAGTTGTGGACTTGAAA
ATGCAATAGTGTATCTCCTGTTAAAATTTTTTCATTCGCGAGGTTTTTGTTCTCAAGTGGTTTTTTTGCC
ATGGCATGAAAATAGTTTGGTTTTCTATTATATATTCTTACAAAATTCTTAAAATTAGTCTAACAAATAA
ATTGATTTAAATAAAGGGAACAATTAACATCAAATAGTGAGAAGGCTATAATAATACAATAATCGGATAA
CAAAAAGTAATAATACAACAATCGGATAACAAAAATTAGTTTCATTGACAATATTAATATATAATATGAT
AAATCAAATGACAATTAAATTTAATCCCACATTTCGGTCCTTCAACTATTTGATTTGCCTTGAGCTTGTT
AATTTCATCATTCCTGAAACTTTTATGTTTGGACTGATAGCGTATAAAATATGCTATCTATTTTTGCTTA
ATTCAAGTTTTTTTTTTTTTTTTTTTGTGATTTTAGGTTCTTGTTTGGATATTTAATAGGTATGGTTGAG
CAATTTATGGGCGTAATTTAGAGTTGATATCATTTTGGAGTTAATTTAGGCTATTTGGAGTCAAATGACA
TCAGCCATGTGGCAACCTGAGGCAATCAGAAGCAAATCGAGTCGTTTAGATGAGCTAAAGAAGCCAAAAA
GGTTCTACCTTCCACTGCCGAGATGCCCCATGGCAACTCAGGCGACACTGTAAGATAAAAGGGTCCTAAC
TTGCGTAAATGGCCAAGTACTGTGGCCCTGCCACTGCGCTCCAAACGTTTGAGACATAACTTGCATAAGC
TTGAGCGTCGCGGTGGTTGGACATTATTTTAAATACTTTTTTTCTGCTACCAGCTAAACCTAGGTCGAAA
TACGCCGGTCGTTTTACACTTGATTTCTCTCTATTTTCTACTTTCTTTCATGTTGTTTTCATTCCCGAAA
ATTCTCTCATCATCGGGCATGTGGATGAGAGGCTAAGGCGTACTCGTTTCTAATTTAGGTTTCTTAAATT
CTACTTTCATCCTTTAGAATTTGTGAGAACTTAAACTAGTTTTATCCTAATGTTTGGCTCAATTCTAAAT
GTAATTAAATTTTGTTGTATGTTAGACGCATGGGAATATATAACATTTTGCTGCACTAAATTGTTTGACT
TGTAACAATTAGATAAGAATTGGTCAGAAACAATGTAACTCCCCGAGCGGTATCACATCTTAATGAGAGG
GATTTTGAGGACATGAAAGTAAGGTTAAGAAAAACTTAAAAGAATTAAAAGTTACTTCTCATACCAACAA
AATACACCTTCCTTTTCGATGACTAGATCATATAGGAACTCAAAAGTTAAGCATGTTTAGCTGGAGCAAT
TCTATATTTGGTGACCTCGTGGAGAATTTTTTCTAGGATACTTGTGAGGGAGGACAAAGCATGCGAAAGA
ATTTGTGTGTTGGTTTGTGAAAAGAACTTTCACTCTAATACATGTAAGGATAATGTTCAATGAATGAATT
CTTTAGATAAGAAAGAAATCTTAACAACTCAAGCTAGATTATTTTTTTATATATTGAATTTCTTTGAATT
TTCCTTTTTGCAAAGTGTGCTCCCTGTAGTTGACCCGGACTTGCCACTACGACTAATTCTTGATGTGTGA
TTCGGTTTTTTGTTCGGTACGGGTCTGGGTGACGAAATTCGTTCGACCATGGACCAAGTGCTTAATTTTC
ATGTTAATGTTTGCCATTAAGTGCAAGTGGTCCTTATCAAAACATTTTTTTCAAGTTCCAAATGTATTTC
TCTTATCTAGAAAAATTTTATAAAATCATGCGAGCATCAACTTTTCAAGCCATTTTCCTTTTTCAGTTTC
ATTTTTAATCAATTCAACATCTCATAATAATTTGAAGATTTGTTGATTAAACAACTGAACTCGTACCTGG

AATGGAAGAATCTAACTTTCTAATATTTAATTAATCTCAAAAGCAATAATACAAGTCTATATTCTTCTTG
TTGTTTTAATTAACAAGTAACTAGGTGTGGTTGTATCAATAAACACCCATATATCTTAACTAGGTTGACA
CATCTTTATAAAGTTTTATATTAGTTGGGTCGTACTTATGGTGGCAAATAATTAAACACGACCAATTATA
TGCAGTAGTTATGTCCTGTTATATATTCAAATAACACACTTTTTTTTCCTATTCCTCTGCAAATACATCA
ATCATAAATTTGAACAAAAATGATCAAAGTACTCTATGTAATTTACAAGAAAATTAATTAGAAATGAAAC
CGACATTACTATAAATTCAAAGTTCCACCTTTAATTATAAGCAAAGAATGGAAGAAAAAACTGATTATGG
TTAATAAAAAAGAGAGAGAACAAAGGGCTAAAAGTAAAGTTTCAAAGATAAGGTTGATAAGCCTTTGAAA
ATGTAAAAATTAAATATAATGAGTGGTCAAACATTGTATGATTGTGTTCGTATCTTTTTCAAAGAGAGAA
AAAAACACTTTTTTGTGCTATAAATACCATCCTCAAACCCTTCTTTACAAACCATCTTCTGAGTTCCAAA
CATATAGAGAGAGAGAGAAAAAAAGAATTAGAAAAAGAGTGAAAATGTTTGGAATTGGGAAGAACATCAT
TGAAGGAGCCTTGAATACCACTGGAGATCTTGCAGGCTCTGTTATCAATGCTGGTGGTAACATTGTTGAG
AAAGTTTCAAGTATTGGAGGAAAAAAAATAAAAGGGAAAGTGATTCTTATGAGAAGTAATGTTTTGGATT
TCACTCAATTACATTCCTCTGTTCTTGATACCTTCACTGAGATCTTGGGTAGTGGTGTTACCTTTCAACT
CATCAGTGCCACTCAAGCCTGTAACATCTCTTTCCTCTCTAATCTAAATTTAAAGTTAAATTACAAGTTT
AGCATCTCTATTTTGTGTCTTTCTACTATTTTTATAAAAGATACCTAGCGTTAGTTAGTAGGTGCTTCTG
TATGTTTACAAGGTCAGACATATTCTTAACACTTTCATTATATCTCACATTCTTTTCAATATATATCCGT
TTAGAAACTCAAAGAAAACTTAGAAAACAATAGTCCAACAATAGAGAAAAGACCTCAAGTATTTTTTATT
TTTTTTAACAATCAAGTTTAAAGTATTGACAGATATAATACAATATAATGAAAATAAATTTATAGATATA
AAAAATTTGTCTAATGGATCGTATTATTGGTAGGAGTCTATCAACAAATATATAGATTTTGTTATACTTT
CAATTTTTTAAAAATATTAAAATATACTTAATTATTAGGGTATCATTAAATTTGATGCCCAATGCAATTG
GCCTAAAAAGAAAGTATAAAATGAGTGTTGCTAAACATTTAGTATTTTGCTAATAGGAGAATACCGTTTT
TTTCTCACAGCATTTGATTCAAGAGGGAAAGTTGGGAAGAAGGCATTTTTGGAGCAATGGATAACCTCAA
TCCCACCATTATTTGCTGGAGAATCTGTGTTTCAGGTTAACTTTCCATGGGATGATAATGATTTTGGATA
TCCAGGAGCTTTCTACATACAAAATGGACATACAAGTCACTTCTTCCTCAAATCCCTCACTATTGAGGAT
GTTCCTGGCTATGGAAGAGTCCATTTTGATTGTAATTCATGGGTTTACCCTTCTGGTAGATACAAGAAAG
ATCGTATTTTCTTTGCTAATAACGTAAGTATTAAATTCCTTTTATGTGGGCTAAAGCGATGTGTCAAACC
CTAGCTAACATATCCTGATTTATACACTAATCTCGCCGTCTATCTAACTTTGTTCAGAAGAAAGAATAAG
AAAATATTATTTTTCCCTTTTTTGTTTTACCTCTCTCTCTCCTGCAAGGAATTAATAATAAATAGTTTAT
TTGCTTTGTGTATAGACATATCTTCCAAAGGATACACCGAATCCTCTTCGTAAGTATAGAGAGGAAGAAT
TGTTGAATCTTAGAGGAGATGGAACTGGAGAACGTAAGGAATGGGATAGAATTTATGACTACGATCTCTA
CAATGACATTTCCGAGCCTGGTGACGGGCGTCCAATTCTTGGAGGGAGCCAATACCCTTACCCTCGTCGT
GGAAGAACTGGACGACGACGAGAATGGAGAGGTAAGATTTTTAATTATGGCGCATTTCTGAACCATTTAG
TAATTTATGAAATTCTTTAGTCAAACCGAAAGTTTCATTTATGAATTTTTTTGTTGTTAGATTCGAACTA
TGAGAGTAGATTGCCAGTGGTGTCAGGATTAAACATTTACGTACCAAGAGATGAAAATTTTGGACACTTG
AAGTTATCAGATTTTCTTGGATTTGCATTGAAATCAGTTGTATCAACAGTTCAACCAGCACTTCTAAACA
TAATCAATATTATACGGCCAGGTGGAGAGTTTGATAAATTTCAAGATGTTCATGATCTTTACGAAGGAGG
ACTTCCTGTTCCATTGGATGTTATTAGAAATCTCACTAAGGATTTCACACCTCCAATGTTACAAGAACTT
CTTAGGACGGATAATGACCAACGCTTACTCAAATTTTCACCTCCACAAGTTGTTAAAGGTATACTATATA
TAATGCATGTAGATTAATTTCTATTTCATTATGTTACTTAACACGTACATTCTTATATTATGGTCTTATT
CGGTAGCAAATTTGGATTATATTTTATGTTTTCAAATTTATTGAAATAATACTTATATTTTTTTCCCTCT
AATAATCACAAATAGAGGACAAGTTTGCATGGCAAACAGACGAAGAATTTGCAAGAGAAATGCTAGCTGG
AGTTAACCCTCTAATCATTCATCGTCTTGAGGTAAATAAACTCAGCTTTTTTATTTCTCCGTTCAGCTTT
ACACATTAATCTTTTTTCAAAAGATTATATGTTTTCTTTTGTATATATTTTTATAGGTATTTCCTCCCAA
AAGCAAACTTGATCCAAAAATATATGGTGATCAACATAGCAAGATTACTGAAGAAGACATAAAGTCTGGC
TTAGAAGGTCTCACAGTTGATGAGGTAAATATTAAAAACACCTAAACTTTTGTTTTTCTTTTTTTTTTTA
AATTATATTATATTATTGTAATTTAACATTTTTTTTTTTCTTATCTAATATATGTATATAGGCATTAAATC
AGAGGAAACTATTCATATTGGATCACCATGATGCATTAATGCCATATCTTAGAAAAATAAATTCAACAAA
AACAAAAGCATATGCCACAAGAACATTGCTAATTTTGAAAAATGATGGAACTTTGAAGCCATTGGTTATC
GAGTTGAGTCTGCCACACCCTCAAGGTGATCAGTTTGGTGCAAATAGCAAACAATATTTTCCAGCTGAAG
AAGGAGTTCAAAAGTCAATATGGCAATTGGCTAAGGCTTATGTGGTTGTCAATGATACTGGTTACCATCA
ACTTATCAGCCATTGGTATGTTTGATCGAAAATAATTAATTACTCAACAATTAAAATGTTTTATAATATA
TATATGTGTGTGTGTGTGGGGTTGTAATTTATTTGAAATATACGATGTAACACTTGAAAATTCTTATTGT
AGGTTGCATACTCATGCAGTACAAGAGCCATTTGTGATTGCAACACATAGACAATTGAGTGTGCTTCATC
CAATTCATAAGTTACTTGTTCCTCATTACAAAGACACTATGTTTATCAATGCATTTGCAAGACAGGTGCT
TGTTAATAGTGATGGTCTTCTTGAACAAACGCATTTTCAATCAAAATATTGCATGGAGTTATCCTCTCAC
ATATATAAAGAATGGAATTTCTGTGAGCAAGCACTCCCTGCTGATCTCATCAAAAGGTAAATCACCAATT
ACACCATTAATTAAGCCTTTTATTTTCATCAATTAGTCTCTTAAACTATACAGGTTGGTTACAATTATAC
ACCTGAACTTTTAATTAGTTGATGAATCACCTCTAGTGGAGTTTGTTGCAATTAATTATTTATCCCCCTA
AAGAATGATAATCATAACTAGCTCCGTAAATAGAGGTGGTTTGTGCAATTTTTCTTTATAACTATATTAC
AAAAGATGGGCATTTTATTATCCTTTGATTATTTATGCAGAGGTGTAGCGGTTGAGGATGCAAGATCAAC

ACATGGACTTAAGTTACTCATAGAGGATTATCCATTTGCTGTTGATGGGCTTGAGATTTGGTCAACAATC
AAAACATGGGTTACAAACTATTGCTCTCTATACTACAAAGATGATAGTGCAATTCAAAATGATGTTGAAC
TTCAATCTTGGTGGAAAGAGGTTAGAGAGAAAGGTCATGCTGATAAGAAAAATGAAACATGGTGGCCAAA
GTTGCAAAATTTCAACGAACTGATTGAAACATGTACTACCATCATATGGATATCTTCGGCTCTTCATGCT
GCAGTTAACTTTGGACAATATCCTTATGGAGGCTTTTGTCCGAATCGACCAACGATAAGTCGTAGACATA
TGCCTGAAGTAGGAAGTGCTGAGTACAAAGAACTTGAATCAAAACCTGAGAAGGCTTACTTAAAAACAAT
CAATTCAGTGTTGCAAACACTTCTTGGAGTTTCAGTAATTGAAATATTATCAAGGCACGCTTCTGATGAA
GTTTATCTTGGACAAAGGTCTAGCATTGAATGGACTTCTGACAAAGCTGCATTAGAACTGTTTGAGTATT
TTGGAAAAGAAGTGTCTGAAGTTGAAAGTAGAATTATTGAAAAGAACAAAGATGTGGATCTCAAGAATAG
AAGTGGACCTGTTAATGTTCCATATACTTTGCTTCTTCCTTCAAGTACTGAAGGACTAACAGGTAGGGGT
ATTCCAAACAGTATCTCTATTTGAAGGGACACTTTGAGCATATGGTTATGTTGGATTATAAATATTTCGT
CTTTCCTTTGTGTTCTATTGTAACTTTCTTATGTTTGAAAAAATGCTACTTCTTTGGCTACTCATGAAAT
AAAATCATAAAAGAGTGGGCTTTAGAGGTAGCTTTGTATTTGATATATTAATGAATAAAAGTTATGCTTT
TAAAGTAATGTGTTGTACAATAAGCATATTTCTTAAACTAGCTACGTGATTATATATTAAGGAGAAAACG
ATTGTATTGTTTAGATACAAATAGGGGTATGTTTGTAACCATGTCTCTTGTGATCTTTAACTGAGAGCTC
ATTAATTGCTACCATGCACACAATTTAAAATCAAAATTAGCTTATCTTTGAACTCCATATAAAGATTTTC
TAAGTGAATAGACTTTTATTGAAAATAATCTCATTAATGATTTAAAAAAAGAAATAGTTTGGGATTCCTA
GCTATAACGTGGATAAAAGGATCTTTAAATTTAAACTACTAAACTCAATATTCCTTCTCTTTTATTATTT
CTCTCAACCTAGGGATAGTTGTTGAGAAATGGACGGTTCTTAGTTATTAAGAGTAGTTGTAAACACAGTT
ATATCCAATCAGAGAAACTACAGTATTAGGAAGGAAAACATAACACATAAACATTTGTTAACCCAATTCA
ATGATTACACATTTACATCTGGAGACGGTTTGCTGGAGTGTATACAATAATTGTTTAATACATAGTTATG
ACTGGAGTACAACACTCTACATAGTTACTATCGTGCTGTCTTAGAACATCTCAAAACTATGGATGTTGTA
TTCAACTCAATCCCCAAACGACGTGATGTTTCTTCTATCGGTTTTTCGTATTATTTTCCATAAAGAACTA
CTATGAAAAACTCACTATCAAAAGCTTTTATACTTTTTATTCCATGCTTCAAAACAGCATAACTTCTCAC
ACATGATTTTACCAATATATAAGAAATAAATCCTAACCACCTGTGGGACCATCCTTGAAAATAAAATCTT
AACCGATTATTTACTAACTCCATAACCAATCAATTAATTAATTTGCACGTGATAAAATAAAATAGGTGGG
ACCATTTACTAACCGTTATGTTATGGATTTGTGGAATGAAAGAAACAATTGGATTTTCAGAAACTTCGAA
ACAAGTTTAATTAACTCTTGGGAGGATAAGATCAACCTTATAGGCCTTTCGTCCGGCAGAAATTCAACAT
CTAAGGATTACAATCCATCTACTATATCTCTAAACACCAAAGCATTGTTAGACTAAGTTTCTCTTAAGGC
TATTCTCAAGCCTTTGAACCTTTGTAACATTGCTTGATGAATAAAATGGTGACTATAAGTCACTCTGTTT
CTTGATAAAAAAAAAAAATAGTCCTCTTTTAAATATCTAAAACTTCTTAAAATATAACACTGAGCGCTAAC
AAAACTTCTCGTCAAGTGCCCTGGTAATTTCTTACCTTTACCTGAAAAACATTTTAGGAAAATTATGAGT
ATTTAAATTACTTAGCGAGTAACCATACTATTGAGGTTTCAGCTGGTGAAACCTAAAGGTTATCGAATCT
GACCAAAGTTGTATCATGGGACACTTTAGGGCATTTTCTTGTCACATGCCCTTTCCATAAGAATTTTTGT
TTGAGGGCGATTTTCATTTCACATGTCATTTCAAAGCACTTCTATTTGTGTATCATGTTCATTTTTAGGC
TAGCTAATCGTGTCATGTTCCTACAAGGCAGGTTTTTCCATGTCTCTTTTCAAGAAAGTGTGTCATGTGC
CCTTTTTAATTAGGTGAATCCTGTGATGTGCTTGGAAGGCGAGATGGGTCACAATTTGTGATGTTTGAGA
TTGTTAGAGCAAAAAAAAAAAAAAAAAAAAAGCGCTTATGCCTTACCGCTCCTGATAATGGTCGAGGTTCT
GATATACCAACCTGGAAGTTGAATACGAACAGTCAGTTTAGCATAGTGTCTATCAAAAATGCTATACATA
CCATGGACCAACATGATGAATCCAACATCAATCCTATTATTTTCAAAAACCTCTGGAAATCAGGTATCCA
TAAAAAGTGTAAGATCTTCATATGGTCTCTTTTGCATGAAAGCATCAATACACCAGAAAAACTCCAAAAA
CGACTCCCAAACTGGTATCTCAATCCAAATTGGTGCACTCTCTGGAAGAAACACTCTTAACACATCTTCA
TCTCTTGTGTTGTCACTCAAGAAATATGGGGCAAGGCAGAGATTACGATAAACTGACGCAATATAACAAT
CCAAACCTTGCTTCTCGGTGCAAGGAGATTTTTAGAATCAAACAAAAAGAACAAAAAATGGACCATTACA
TTTAACACTATTGCTTTGATCCTACTGATCATTTGGCTGAAAAGAAATAATTATCAGAATCTTAAGAGTA
AGGAAAGAAATACCACATAGATATGGGACGATATTCAAGCTCTCATTGGTTTTTGGATTAGCAGATTGAA
ACTTTTTACAAACTATATAGCGCTAGCTCTATTGCCTTAAATCTTCAAGCTTTTGTATAATTCTCTCATG
TGGACTTTATCTCTAGCCTTTAGACATTCCTAGCTTCCTGCATTATCTTTCTTTATTATTAATGAAGTGG
AAGTGATGAGGGTGTTAAAAGAGTGTCCACCAAGTGGAGATGTCTAAGTGCACCTACTGACCAAACATAT
CTTTTTCAAAAAAAAAAAAAAAAAGAGTTTATGCCTTTGCTTTTCATTTGTCTCTATTAGAAACACTTTAG
TTTTAGTGTGTGAACTCTAATTAAACTCTCTTGATTTTTCTAGTTGGAGAGTGAAAAAACGTGTGCATGG
CCAAACACTTTGAGAGAGTGTTTTTCGTAATCGGGGATGGGAGAGAGAAAATTATTCATTGTGGCTGTAG
CAAATTTTCACTCGGAAAATTTCTTTCTTTTCGGTGATTTTTCTCTTAGTAGGGACTTTTCCACGTAAAT
TTATGTTTCCAGTTTTTATTGTAGACCCCTTAGGTGTTTCTCTAGATCGACTTGAAGCTTTATTCGTTAC
TTGAGAAAAGATTCTCAAACTTAACGAAAAATCTAAGTTCATCAAATAGAGTGGTGATCTTCCTCGATAT
TTGAAATTTGGAAATATCAAAGTTATCCTTCGAAATTGAGTCTCATACTTGAAGGAAGTTTTGTATTTAC
AAGAAAGCCTTGTATTTACAAATAATTACAACGTTATTGTAATTGCTTAACTGTTCAATAGTAAAAATAT
ATTTTACCATGCACACTAACCCTCGAAATGTATGTGATATAACACTGATCAACATAATTGGTTACCAAAA
TCTAGGTTCTATTCTTATTTTGTTTTTTGTGTTTATCTTTGATGTTCTTAAAATTCTATAAAATTGTGTG
TACCTTGTTACGTGGTGTGTCTGATAACTCTATTAATCAAACAAAAATTATATCCATATAGAAGTTAAAC

AACCAGATAGAAATTAGTATCATTAGCTAAGATAGATGTCAACTCCATATGCTCGAGATGCAAGGAGCGT
CTGACACCCTCTAAAAGGCTTAATTTTAAAAGAAAAAAATGAGCAGCAACAAATTAATCTTTTTTTACAT
TGTGGTTGAACATTGAAATAAAGTTTACAATATTATAAGTTCAATAAAAGATTTAACATAAATGATATGT
GAAAACTAAAGTTGTGTTGAAGAGTTGTCTGTGTATGTTCTTAGAAATTGTTACATGATGTATTATTTTT
AAAATTTTAAAAAACATGATAAAATTATAACATAGATAAATATTTAAACTGCATACTGTAAAAGAGTTTA
AATGAAAAATATTTAATAACTTACAGAAATGTGAATTACAAATCGATTCAATATTTTGATTTGTTGGAAA
AATAAATGTTGATTAACATTGTTGTGTCGTGTTGAACCACTTCCTTGAAAACAAAATTCGATGCGATCTA
AGTGCTAAATCGTTTACGTTGGTTGCTTTTCAAAGTTAACACAATTTTTCCTACTCAAGCATACACTCGC
GAGAGAAAGGAGTGGAATCAAAGGAAAATCGTTCAGAGAAGGTAATTAGGGTTTTTCTTTTCAAGAGGAA
GAACACGTCACACAAAATCTTTTTTTAAAAAAAATATATATAAAAGAATAATGATTTTTTAATAATAAAA
TGTATTAAATAATAAAATTATTATTTCCAACTCAAACCTTAATTATAAAACAGTACAATGATAGTCATTT
CTGGGAATGACGACTATCCATTATTCTAGTTAAAATAGTAATAAAACATTTTATCTAAGCAAAGAAAGAA
GTAGTATAAACTTAAACATCAATATCTTTCGATTTGAATCGATACATAGTGAGGTAGGCCAACAATCTAA
TTAAAGAAAGTAAGTTTCGTTTTGAACTTTCAATAACTACGGTTGAGACACCCATAATTCGTTTCACTTA
TTAATTTTCCATCGATTCCGCAATAAAGTCTATCACAATAGACAACTCAAAATCGTGCTATTTAAGACCA
TGCAAATAGAACCTCGGGTCAACTTGAAAGCTCTAGAATAAAATATCCTTAAACTTTTTCATAGAAGGCG
GCCACACCTTCATAATCATGTGTTTATTGCTTCATCAAGTCTATCACAACATATCACTCAAAACTAAGGT
ATGAAGACTTCACAACTAGTCCATATTAAGACCAGCTTCATCAAGCCTGTCAAAGACAAACCACTCAAAA
CTGAGCTATGAAGATTTTACAGTTATCCATCAAGTCCATACCAAGACTACTAAAAGAAGGGCAATGAAAA
TTTCAAAGGTTTTAGTTTCATCTTTCAAATCTATTTAAGGATTACCCACACGTGCATGCCCATGAACCAT
CAAGTCTACTGTAGTAGACCACCCAAAAAAGGGCTATGGACAATCAAGTCTATTGCAATAGACCACCTCG
AAAAGGATTATGAACCATCAAGTCTATCGTAATAGACCACCAAAATAAAGGGCTATGGATCATCAAATGT
TTAAGTCCCATACTTATTAAAAAGACCTAGAATTACTCTCGTTATTAGGCCTTTGGAGAGATGACCTACA
AAATTCCTCTTAGACCTCACATATTTCAAGAAGATAATTATTTCATTATATTTACCTATCATTTCACAAC
TACATGTTTCAGGCAAATATGCCTTCGTTCTTTTTCCATAACACGCTATTCTTGACAATACACCTATGAC
AGTGTGTGAATTCAATAAAGAAATATTGGTATAGAGGCTCCCACAATGGTAAATCCTCATTAGGCTTCTA
TATCGATCTTACTAATGTCCTCATACAATGGTACATTTCTCGTTAAGAACACATATCCACTAGTAAGGCT
TACTTCGTCATTTTAAGTTACCCAATTTGCATACACTAGCTCTCTAGTATGGTAGACAATTTATTAAAAT
ACAAACCAAAATCTATTGTAACCTTACAATATCTTAATAAATGACGAAGAGTGATAAAATGATTGCAATT
AGGATTATGCGTATAACTCAATCTTCTCACAACATATGCAATAACAAATCTTGTACAGTTTATAAAAAAC
ATAATACTTTCTATAATCCTTGAGAAACTCTATCTCATTTATTTTCCTTAAGGCTCATACTAGCATCATA
GGGTGTTCTTACAAGAGCACCATTAAACTATCAAATTCTTTAATAGTATTCAATGCACAATTGACATAAA
TCAAAACATTCTCAATAATACTTAAGCGTATCACATTAGTCTCACATAAAATTTTCACTTTAAAATGTGA
CAACTTTATAGCATTAATAACATTTATGTTTGTATCACATAAATCCTTCACATCAACATACAAACATATA
ATCACACAATCAACTTCAAACAATTTTTAGTAAACACATATATCTACAATTCACTTCTCAAGATATTGTT
TACTAAAGTGTTGTTAAAACTTTTCCTCGTAATACTTTTAGGAACTAGTTAAAGACCATAAAGAAATTTG
ACTTTTCCAAGTTACCATTTGAGGAATATCATCTTTACATACACTTCATGTATTAGAAAACCACATACAG
CTACAAAAGTTTTTAAGACTCTTATAGCGGCTAATTTAGTCATACGAGAACAAGAATCAAAACATTCTAA
ATTTCATGTTTAATTCATAATATTTTTTTTCATAAGTTGCACCTATGTACTTATGATCAGTTTCCCTATC
TAATATGGGATTTAGTGGCATTCCCAACGATCCTCTCCTCGAACAAAGTTCCAATTGAACCTCCCTTAAA
ATAGACATCTATCCTTTTAACTTTTATCTAGGCTAACTCCTCTCTAGAGCATACAGCCTATTCAATAGAG
CTCAACCATGATAATATCACGACTACTTTTACGGTTCCACTACTTCGACCTGAGGGTTATATCAACAAGG
CTAAACCAAGGACGACGTGACTTTGATACCACTTTTAGGGATAACTTAGGGTATAAGTCCTCATGCCTTA
TAAGATATCGCATATTCAATAATTTAGTTACATGCAAATAATGTTGTACGACCAAACTTGACAATTCATG
ATGCAAGTCATGCATAAACTTTGTCAAATATAACATACACGAAATATTATTTCTACCCTTACTAACATGC
TAATAGACACGCTCAAGTAGATCACAATAGTGTCTCAATGTTCACAAACTTATTTATCAATTAAAAATGT
AAATCTATATATAGCACTATTGGGAGCATAATCAATAAACATGGTCAAAAGTTTTTTCAATCAAACCATA
GATTTCTTGAACACAAGATATGCTACTTTAGTAATTAGACACCCCCACAGTTCTAAATATTTAGGTGCAT
AACATATCTAAAGTTCAACAATACCATTTTGTTGACTAACTCAAAGTCATCACAATAGCATTCTATTTAT
GGCATTAACAACAATACAATAACTAAGAGCCAATATGTTTGTCAATGAAATATTCAATATCTACTTATAA
AACATATATTGAATATCATGCTCAAATATAACAATGAAATAAAATTGTCAAATATTTTCAAGTCATATAT
AAAAACTCGAAATATTGTTGAGCCTTAACCTATAGAAAAACCAAACCCATCATTATCCAAAACTTACAAT
AACTTGACTCTTTATCACATGTATCATGAGATAATTTACTACTCGTGCATATCCAAATATATATATTCAT
CAAGTATGTATCAGATATCAAATATTAAATCTAGTAAGAGAAATCTCAACAATGTAAAGTTATGTTTGTG
GAACAAATTATTAGCAAAAATATATCAATAAAACAAACCAACGATCCAATTAAAGCCATACAAATAAATG
CATGGCTTATTAAGCTACATCTTAGAATCTTCAACAGGTTTTTTAAATCGAATTTTTCAGAAATTTTAAT
TGTCATCTTCATCCAAATTGACAAAAGATGAAAATACAACGTGATCAACTATTTTTCCAAAATTTTCAAA
TCATCTTTTCAATTGGATGATTTAGTCTACGAATCACATATGACAGTTTTATTCCTTTAAATTACTTCCT
TTTATTTAGATAAACTTATAGCAATCTAAATATAGAGAATCAAAATATAATCTCTATCATATTTTTGACG
ATCTTCCCAATACCTCCCAGACAACCTCAAATCTATCTCTCAAAGCACCGGCTCTTCACAAGGTGAATCT

TAAAGAAATTCAAGCTAAGAATGGAAATGGCAAGGTTTTCATTCAGACTATTGTCAATATTGTTCTTGGA
GATTGTACCACATGATTCTTACCTCTTCACTCCTTCAATTGCTTGCATGATGTAATGTTAAATATAACTA
AATCTTTTTCTTTTATACTTTTTAACTCTTATGTATAAACTCCTTTAAAAAAAAAAAAGGTCTTGCACAAT
GTATCAAAGAGTTAAATCAATAATAATAGCTCTAAAATTTTGTGTACCATTTTGCAGCCGCAGCTCAGAT
CCACCACCGCCAAAATCAATCCACTCGTTCTTCATCTCTGTTTGTGGTTTTTTTTTTCTTTTCTTTTCAG
ATTGGATCGACCTGAGGCCTTCATTATCCTACCGCCAGCATGTTTCCAAAGGATCATTCTGGCCTCGTCG
ATCGTGAAATTTGGGAATTTCTTCCAGGATAATTTCCTAGATTTGATCGACATTTGAAAATAATTTTGCT
TTCAAATTGTTGGGAAAAAATGTTGATCATCGTTAGAACTACTGTCAGTGCAAAATCACTTATGTCTTTA
TAAGGTTAACACAATTTTCTCTACTCGCAGGAGATAGAGTGCACTGAAAAAAGAGCGAAATCAAAGAAAG
ATCGAGAGAAGGTAATTAGAGTTTTTTTCAAGAGGAAGAACACCTCACACAAAATCTTTTCTTTTATATA
TATATATATATATATATATATATATATATATATATAATATATATATATATATATATATATATATATATAT
ATATAAAAGAATAATGAATTTTGAATAATAAAATATATTAAATAATAGAAATTATTATTTCCAATTCAAA
ACTATACTCTGATAGAACATGTTTATGGGGAAAAATATATAAATTAAGTAAGCAAAAATAGAATATTAAA
AGATTAATAAAAACATATAGAATTTCGATCTCATTTTTTTTTAAGGATACTTCGTTATGGTGTTTTTAGG
CAACCATAACAGGATTTCATTAAAACCAAAATAATAGAAGTTACCAGGCGAAGTTACATGCGCTGGAGAG
TAGCCTGGTAACAAAGTAATTAGTTAGAGAATACATTGAGATTGAGAGCTATCGTGCTTTGAGAATAGTT
TTTGAAAAGGGAGCTTCTTGATGCCCACGTTCCAATCAGAGCGGATATGTTTTCCCATGCATTCCAGTAA
GTGGAAACTTTTTTTTTTTGAAGATTTGGTTGTTTCTGTCCAGCCAAATTGTCCATAAAGTTGCCGCTAT
TGCATTGAAGCGAATGATTTCTTTCATGTTGCTGTTTTTTAGAGCACATAGATATGAGCATAAGTCTTTG
ATGTTTGTTAGAGGGATTGCAAAGTTCATCTCATTTTGCATCTTTATCCATAGTTGTGTAGCATTAGGGC
ACGACAGAAAGAGGTGATTGATGTCTTCGTTTGTCCTTTTACATCTGACACACCAATTTGGGTTGAGATA
GTAAGAAGGATTTTTCTTTTGAATGGCCTCCATGGTACTAAGTTTCTGGTGTAGAATTGTCTAAATAAAG
AACTTACATTTCAGAGGCATGGTGGATCCCCAGAGATTTTTGTAGCCTAGGCTTTGTGTTGATTGAGGGA
GTTGAGAGTGATGAGCTTTTTCCAAGAATGCTTTTTTTGCTGATCTAGTTGTGTAAGTGTCATTGCTCTC
TTTGTTCCAAACTGGTCTGTCATCTGTGGAATTTTGTGTTATGGTGGGGACGAAATTTTTTAGTAATTGC
CAAGCATTGGATTCCCTTTCATTGAGAGGCCTTCTTGGTTTGAAGTTCCAATCGTTTATCCCCGGGTCCC
AAACTTCTTTGACAGTGGCTTCTTTACAGCTTGATAATGCGTAAAGTCTTGGGGCTTTTTGGTTTAGAGA
TATACTGTTTGTCCAGTTGTTGTGCCAAAAAGAATGTGATCCTTCACAGTTTATTTTTCAGGTAATTTTT
TAATTGAACCAGTCTTTTCCTTTTATGATGGAGCGCCAAGAAGCATTTGAGCTTGAGTACTTTCCAAATT
CTGGGATACCTCCAGTGAATGTTTTACTGTATTTTGCATCAATTATCGACTTCCAAAGAGCTTCTGGTTC
CCTGTGGTAGCGCTAAAGCCACTTTGTTAAGAGTGCAAAGTTTGAGGCTTTCACCCTTGTGATACCCAGG
CCACCTTTGTCTTTTGGCAATGTGCAAATACCCCAGTTAATGAGGTGAGGGTTATTTGAATTTGTGGTGT
CTCCCCAAAGGAAATTCCTCCAAATTTTCTCAATATCTTTATAGATCTGATTTGGAGCTTTGAAGATCGA
AAGCTGATAAGTTGGATTATTTTCGAGGAATGATCTGATTAATGTCAGTCTTCCTCCTTTGGAGATATGG
TTGTATTTCCAACTGCTCAGTTTTTTTATGGATGTTTTCAATAATTTGAAACTAGAAAACTCTTGAGTTA
GGTTTGCCTCCTAAGGGAACTCCCAAGTAATTGATGGGGAGGAATTGTTGGGTAAAGCCAAGTATACTTG
CAACTTTGGAGGCTCTATCATTTGAAATGTTTACCGGACTTACAGTAGATTTGAGAAGGTTTATCTTCAG
TCCGAATGCTAATTCAAATAGCATGGGAGCTGTTTGGAGATTTTTGATTGAAACATCATCATCCTCCACG
AATATCAGAATGTCGTCTACAAAGAGGAGGTGTGTTATACTGCAACTGTTGTTTATTTTAGCTCCTTTGA
TAGCTTGCCTTTTCTTTAGGTGATTTAGCAGTCTGCTAAGGTAATCCATCGCAAGTACAAAAATAAAGGG
GGATATGGAGTCGCCTTGCTTGATGCCTTTTTGAGCTTTTATCCTCCCTCTTGGTTTTCCATTGATGATA
ACAGAATAATTCACATTTGATATACATGCTTTAATCCATTTTCTCAATCTTTCTAGGTAATTTTTTTGAT
TTAGCATGTATTCAATGAAGCTCGAACTTATCTTGTCAAAGGCTTTTTCCACATCCAACTTTAGGATAAA
GCCTTTTTCCTTTTTTGATTTCCAATAATCGATCACTTCATTAGCAATGAGGATAGCATCTGTTATCTGT
CTACCTTTGACAAAAGCCATTTGGTTTTTCGCCACAGTGTCAGGTAGAGTTTCTTTGATCTTGTTTGCCA
AAGTTTTTACCATGATCTTGTACAGCGAGGTAGTTAGGCTGATTGGTCTGAAGTCTGTTAGTTTAGAATA
GAGATTCTTCTTGCCAATGAGAGCAATGTAGGTGTTGTTAACACATTTGTTGACACAATTTCCATGGAAC
TCTTTGAAGACTTCAATCAGATCTTTTTTAAGGGTATGCCAGTGTTTTTTATAGAACAGTATGGGGAAGC
CATCCGGACCAGGTGCTTTGTCGTTGCTTAAAGACCAAATGGTTCTTTTTATCTCAGCCTCGTCAAAGGT
CTTGCAGAGATTAGATTGATGAATGTCATTTAAAGGATTCCACTCCAGGTTTGATATGAGTATTTCTATG
CTTTCTTTCTTTTTGAAAAGGCCTTTGAAGTGATTTGTGATGACATTTGAGATTCCATCTGTTGTGTCAT
AAACTTGGCCATTTGAGTCAGTGATTTCTTTGATCAAATTCTTCCTTTGTTTGGAGGAGTAAATCCTGTA
GAAGAAGGCCGAGTTTTCATCACCATACTTGATCAATTTATGTCTAGCTTTTTGTCTCCATATTTGAGCT
TCTTTAAGTTCTATGTCATGGAGATCAGATTTTAATGCTATTCTTTTTTAATGAAGAGGAGCCGTCAGGA
GATTTTGATATTCAAGCTTGTCAATGGCTTCTATTTCCTTTGTAAAAGAGTCTTTTTCTAGTTTGTAGCT
CAAAAATTTTTGCTTTTGCCAGTCTTTAATTCGAGTAGCCAAGATTTTGAGCCTTTGTATGAAGGAGTAT
CCAGGGTGTCCTTCTATTTTGGTGTTCTCCCACCAAGTAGGAAAATTTTTTGTGAAGTCTTTTTCATTGA
GGGATGCATTGTTAAGCCTGAAAGGACATGGTCCCCACGTGATGTGATGTGATTCCAAAGTAATAGGGAA
GTGATCAGAGACTGTTCTCTGCATGGTTTTTGAGTGATGAACTTTGAAAGTACTTTCCCAATCCTTTGTG
TATAAGAATCGGTCAAGTCTAGAATAAATCGGATCAGTTTTGAGATTGGACCAAGTGTAGGAGTTGTTGG

AAAGAGGGGGGGTCGACGAGTCCAAAGTCTTGAATTATGTTGTTTAAGATGGTCATATTCTTTTTGTCTAG
ACTCTTTGCATTGGTTTTGGATGGCCATCGAACAATGTTGAAATCTTCTGCTAGCATCCAAATTGGGGAA
CAAAGGGAGTGAAGAATCTTGAGTTCATTCCAAAACTCATCCTGTCTTTACACTTTATGGGACCATAGAC
AGATGTTAGCCACCAATCTTTTCCATTTGTTTCTCTGATATTGACAATGAATGTGTATCTCTGCTTAATC
ATTTTTTCTACTTTGAAAGATGCACTATTCCACATAAGAAGGATTCCACCTGAACAACCTTGAGCTTCAA
GATATTGCCAATTTGAAGTTGGTTTGTTCCAGAGGGATTTAATGTTGTAATAATTTAGAGTATGAATTTT
GGTTTCTGTTAGGATGATGAAATCAGGATCATAAGACATAATTAAACTTTTTATTTGGGCTACTTTTTAG
AGAGAGCCTAGCCCTCTAATATTCCAACATAGAAACTTCATTTGAAACTATGATCCCCTATTGGCTCATT
GCCTGAAAATTCATTTTGTCCCTTTCCCTCGTTTACATTTGTGTTAGCATTGCTAGGAATAACTAAGGGA
TGTTTAGAGGACAGCTTAAGTTCGTTGTCTTTCAGCCAGACCACAAGTCTTTCCTTAAATGATTTTTCAT
CCTCTTCCTTCGAAGCTAAGTTGCTGTGTCTTCCCTCTTCTTGTATGTTTTGAAGTGCTTCTTAATCTTC
ATTCATTTTTTCCTGTTGTAATTCTATTTCCTCATCATGAGCTGAATTTTTTGTTGATTTGCTTTGTATG
AGCATTTTCAACTTGGGCTAAAATGTCTTCAGTTGCATCCAGATCTCGTAATGGAGGAAGATGTCCCTGC
TCAACTGTCAAACTGAGCTCCTTTTTGTCAAAGGAGAGATTCTGATCTTCACTGCCAGGGCCAGCTTTCC
TTTTTATTCGGTAGTATTTCTTTTGGTAATATTTGTATGGGCTGACTCTTTTAGATGATGAAGCTGCTTT
ATTAGCCGCAAATTGTTCTATCCTTTCATTAATGCTGAGATTTTGTCTCTTTCGAAAATCTGAACTGTGG
GATGAATAACCACCAAGTGATTTAAATGATACTTTTCTCTTTGCTGATCTTTTTGATTTGGTTGAGTAAT
TGCAGATATCTTGATCATAAGTTGACAAGATGGCTTTTCCTTTCTCCTTCCTTCGATCATCAGAGTTTTG
ATCAAAGGGATCATACTCGGATTCAGATGAGCTGTTTTTCTTGGCATAAGTGGGATTTGAGTCAGAGGTT
TTGTCAGGCTTTGATTGTTTACTGTCAGATTGGTTTTTTGAATTTTCTGGTGGAATGGCATGGTTTTCGG
CAAATGTGAACTCCTCTGCGTTGGGGTTCAGATTGTCGAAATCATCAGCTGCTTCAATCTTGAATGTCCC
ATGGAAGTTGACATTTCTTTCTATGAACCATCTCCCCTTTGGAGGGGGAACCGTATATACCACAAAGTTT
TCCCCTTGGTCATCAGTGAGCAGAATTGAAGCTGGTACAAAACCAGTGTAGTTGACTTTACTTTAATTTT
TGCTTCGATGAGGTTCTCCTTTTCCATTGTTTCCTTTGCCACAGCCAGAAAGCCTCCGCAAGCTTTTCCA
ATGTTAACAAATGAATCATAATTCCATAGATGGAGGGGGACTCCTTTGAAACGCATCCAACCTCCGTAGC
TTAGAATAAGAGTTTGGCAAGCATGTTTTTTGGTGTCCCATCGCTCAAATTTGACTTGGGAGTTTCCCAC
TGTTGTCCATCCATTTGTACTCTTATTTGAAAGAGCAAGTTTGCATTATCCTCAGAGAGTGTGAGGATGG
CCTTGTTTGCATGGAAGGATTCATAGGAGAACTCTATTTCAGTTTGTTTCTTCAAGGTAAACATTATACG
GCTCCAATCATCATGAAAGAGTCTTCTTGTAATAATGACTGTTTCACCATATTCTAAAGCTTTTCGAAGG
GGGTCTTTCACACTATGAGCAGCTGTTGAGCTTCGCTTGCATGAACTATCATCATAGTATACATTGTTCT
TCTTATCACTACCATCGTCACTACTTTCCATGAGGGCTCTAGCATATGATTTTCTTGAGGTTTCTGAATC
AGATGATGAATAATTTCTGTTGAAATTTTCTTTTCTTACCTCAGAGCGTAGCCGCTTATGATGGGAGTGA
TGCTTAAAGGTTAGCATGGCTACAAAAGACTTCCAACCAAATTTATCGATGCCTTCAGGAACAAGAACGC
TGCATTTTCTACCTTTGTTGTCAATTCTGAAAATTTCAGTTGTGGAACCAGATTTGCTCTTGTTTTTTGT
TTTTCGTACCCACATGCAGTAATCCTCTTGCCTTCTTTCGTTGAATAAATGCTTGGTGTTTGATGTTTCC
AGTAGGGCTTTGAAGCATTTTCTTATCCAATCAAGAATGTCGGGGGAAATATCCATTGAAAAAGATTTGT
GATGGCATATTTCCGTTAGCCACATGCGTAAAAATCTGGACTTACTATCCAAAGAAAGAATATATTATTT
CTTTTCGATTTGGTAGTGCTTTGGAAGTTGTCTGTAAAAGGACATGTTTGCCAGAAAATGAAGGAAAGCC
AAAGCTGAGAGTTGTTGGAAAGTTACCTTTTTTTGGTGATGCAGCCTCTGTTTATAAGCTTAAAGTTGAC
TTGAATGTTATTTGTGGAAGTTGGTTGAGGTAGGTAGCCTTCAATGTTTCTATTTTGGTAGTTTTTCGGT
GGCTTCACCAGGTGTTGATGTTTCTTCATGCTTTGGAAGTGGAGAGAAAATGGTACTGTAGCTCGAACTC
CATTTCGCCCCAATTTCGATCTCATTCTTTTAAAGAATCAACTTAAAGAAATCAGTTTCTCACGTAAAAA
AATTCTACAAAATTGCACTCTCAAATTTTCCAAATTCTATCCTAATATTTTTAAAAGGAAGATTATCAAT
TTAGAAGATTGAGAACAATATAAATTTATAAGGTAAAGTGCATACATTAAATTGATAACGTGGCAAAATA
TTTTCTTAATACATCTATTAAAATAACAAACCATAAAATTCTAAAGACATGATGTAAATGACATAATCAA
AAGAAAAAAAAAAATATTTTCAACGCTATACCAAAATAAACTAAAATATTTAATAAATGTGAAAAAAATTCA
TAGTCTATCTTAATCTCTGTCTTGGTCTACTATAAATAGATTGTGATATATTTTGCTATATTTTTAAAAT
ATTTTTACAAATTTTTTCATTTAAATAATCTTTTCACTATAATGATATGATTAAAAAGTAAAAATTAAAT
AAAAATTAATATTGAAAATAGTTCTCTACTTGTAAATACTTTCCTTTCTTTTTTTCTTCTATATTTTAAG
ATTCTCCTTGCTCAATGTTGAAAGTTAATTTTCATAAATATAACAAAACACCCAAAATATTTATGCTCGT
AACAAAATCAAAAAGTCAATGAAGTCAACTATTTTTTTAAATATTTCAAGTTTGTCATTTCTTTTTATCT
TCTTTTTTTTTTCTTTTACTTTTGATCGTCTTTCCTCTCTTCTTCTTTTTCAATTTCTTTTATATTTTTT
TCGAAATCATGATCTTTGCATTCAATCAATCATGAATTTTGTATTATTTTATTTTCTCAATGTGCTATTT
GGTTCAAGATGTGTACCAAATATAAAAGATCTTGAAAAAAAATCGTTGGGATATTTGTACACAACTGTCAT
TTAGAATTAGCTAGCCAAATCTAAATAATTGTGTAGCAAAAAAATGTAGCAAATAATTTTGAAAAAAAATTC
ATTGATCGTGTAGCTAAATCTAAATGATTGTGTATCAAAAATTTTGAAAAAAAAAATTGTTTGGATTTATC
TTCGAATATAGTTGCCAAATCTAAATAGTACCAAACAATCTTAAAAATTCATTTAGATTTGGGCGTTCGT
GTAGCCAAATCTACGCGATCTTGTACTAAATATATTAGGGCGCACAAGTGACAAATTAATTGCATGTTGA
TTGAGGCATATATTTTTCATTAATAGGTCTATGGCTTTTTCCATTTTCAAAATTGTTCTATACAGTATAA
ATATTATTCTATTTGTTATATTTTTTAAGAAACCGATAATATTAAATAGTAATAACAATTGACAAGACAT

ATAGTAGGAATAATAAGTAATTCAAAATTGTTTTAAACCTTTAAAAAAGTTTCAAATTGTAACACTTCCT
TCGTAATTCTCTCATCTTCTTCAACTTTTCTCTCGTCTAATTTTTGTTTCTCTTTTCCAACTTCAATCTT
TATCCTCTCTTCTTCGACAAGTTGGCAATATTTTATACTGTAGCAAGAGCACAACTAACTAATTATTCTA
ACATATGTTGCTTTATTTATTTATTTATCTGTTTACTTACTTTCTTCTCTTTTTTCATACATTTTACTTT
CTTTTTTTTCCTTAATTTGTGAATAAAGAAATAAAGTAAAGAAAAAAGCTATGGAAGATGAAACAGAAAT
AAAATTGAAAAATAAAGCAAGCGATAATAGTGTATCCAAAGAAACATATATAGAAGTAGATAAAATATCA
CATAATAATATATGGTAAAAATATGGAGTATCAAATGCAGCATTTAACTAAGTAGAATATTCATTGTACT
ATGAAATATTTGAATTTTTGTTAGGATTTTTCGTTTTCTTATTTGTGGAAATTAAGCTAATTGGTTTTGC
TTTCTATCTCTATGATTTTCCTCATCTATATATTGTAATTCTCCAAGGGTTCTTTAACTGTAAATCTCTA
GGGTTTTTTTTTTTCACTCATCGACACTCTTTTTGAACTGTCTATGGCTAGAGGAACAGTAGCCTTTCTC
TTTGTGTCTATGGCTAAAGGAAGAGTAGGCTTTCACCACAAGAAAAACTTCTTCGAATGGCTTTTTATTT
CTTTGAACAAAATACTAGAAAATGTAATTAGGAGAAAAAACTTCTTCAAATGGCTTTTTATTTTTTTTTT
ATAAAATATAGAAAATATAATTAGAAGGTGCAATACAACATCTCAACATCTCAACTAAGTTGATATCTCT
TTAATATCGTGGTCATACCCTATTAGATAATGTTAAAATTGGCTAAAGTTTTAAAAGCACCCTCAGTTAA
AAAACTTACATCTTTGGCGTTTTTCAACCAACCATAGCTATCTAACTAGCCAATTTAGTTTCTTTAACTA
AATTAAACTCTCCTTTTGAAAAAAAAAACCAAACTAAATTCAAAAGTTAAAAAGAAGTATAAACAAACAT
AAAACAAATGAATCATGAAATGAAATTGGAAAATTTATGATAAAATTAGGGAGAATGATAGAAATGGCAA
CGTGGATAAAATATTTAGGTCCCCTTTGGGTCGTGATCTGAAAATTATTTTAGAAATCTAAAAATTTTGT
GAAAAAACAAAAACAAATTCTTATTTTCTGTTGTACTGAAAAACACATTTTGTTATTCCAATATAAAATT
TTGAAATTTGAATTCAAAAATCAAAATTTGTATTTGGTTGTGGATTTGAAAATGAAATCATGTATCTCAT
GTTCACAATTTTTTAAAACAAAATCTCAAAACAAAGAAAATAATTGTTTTCGAAAATGTGTTGTTTCTAG
TGTTTTTCTTAAATCGTTTTTAGAAACAAGTATACCAAAGACATATTAATTCTTTTTTGTATTTTTCTTT
TCGAAAAGATAAAAACGAAAAATTAATCACGGGCCAAATGGACCTTATACTTCATGGCAAAATCAAACTA
GTATAGTAGACTTTTGTTCTTCGTTAGTTTTTTGTCATGGTATGAAAATAGTTTGATTTTTTATTATATA
TTCTTATAAAACCCTTAAAATTAGTCTAAAAAATAAATTGATTTAAGTAAGGGAACAATTAACATCAAAT
AATAATCAGATTAGCTGTAATAATACAACAGTCAGATGACAAAAACTTAGTTTCATGGATAATCAAATTA
CAAAAAAATTTAATCCCACGTTTTAACTACGAAGGAATTTCGGTCATTCAACTATTTGAATTGCATGCCT
TGAGCTTGTCAATTTTGCCATTTCTAGAAATTTTATGTTGCTGAAATATGCTATCTATTTTCGCTTAATT
TGAGTTCGTTTCGTAATTTGAGTTCGTTTCGTGATTTTACATTCTTGTTTGGATATTTAATAGGTATTAA
ACTATTAAACAATTTGGAGTTGATGTCATTTTGGAGTTAATTTAGATTGTATGATGTTAAATGACGCCAG
CCATGGCAACCTGAGGCAATCAGGAACAAACCGAGGCATTTGGATGAGCTAAAGAAGCCAAAGAGGCTTC
TGCCTTGCACCATCGGCGATGCCCCACGACAAGCCAGGCGGTGCTGTAAGATAGAAGGGTACTACTTGCG
TAATTGGCCAAGTGCTGGGGCCCTGCCGCGATGCTCCAAAAACGTTTGAGACAGAACAGGAAGCGTGCAT
AAGCTTTAGCGCCCTAACGCTTGGACGTTATTTTAAATACTTCTTTTCCGCTACCCAGCCAGACCTAAGC
TCGAAGTAACAGTCTGTCTTACACTTGTTTTCTCTCTATTTTCTGCTTCTTTCGTATTGTTTTCTTTCCC
GAATATTTTCTCATCACCCGGCATGTAGATGAGAGATTAAGATACTCGTTTCTAACTTGGGTTTCTTGGA
CTCTATTTTCGTCCTTTAGAATTTGTGAGAACTTAAACTAGTTTTATTGTTATCTTTGACTCAATTCTGA
AAGTTATTAAATTTTTGTTATATGTTAGACACATAGTATCGTATAACTTTAGCTTGCACTAAATTCTTTG
ATTCGTAACAATTAGATAAGAATTGGTTAAAAGCAAATAGGTTAGATTAGTATGTAAATCTATTTTGATG
CACGTAGATTCTTACCAATCCTAGAATTGAATCTATTGAATGTAAGTTAGGCACGAAGGAATTAGGCACT
CCACCTAAGGGAATTTAATTCTAATTGGGAAATTCAATATTGCATCCGGACTTGCCATTAGGACGAATTG
ACATGAATTTGGGTGACAGAAATCTGTTCGACCATGGACCAAGAGCTTAATTTTCATGTTAATGTTTTGC
CTTTAAGTGCAAGTGGTTCTTGTCGAAACATTTTTTACAAGTTCCAATTTTAATTTTCCTACCTATGAAA
ATTTTATAAAATCATGCAAGCATTAACTTTTCAAGCCATTATCCTTTTTCAGTTTCATTTTTAATCAATA
CAACATCTCATAATAATTTGAAGATTTGTTGATTAAACAACTGAACTATTTTAAAATTAAACCATACATA
CCTGGAACGGAAAAATTAAATTTCTAATATTTAATTAATCTCAAAAGCAATAGTACAAGTTTATATATAT
ATATATATATATATATATATATATATATATATATATATATATATATATATATATATATATATATANNNNN
NNNNNNNNNNNNNNNNNNNNNNNNNNNNNNNNNNNNNNNNNNNNNNNNNNNNNNNNNNNNNNNNNNNNNN
NNNNNNNNNNNNNNNNNNNNNNNNNNNNNNNNNNNNNNNNNNNNNNNNNNNNNNNNNNNNNNNNNNNNNN
NNNNNNNNNNNNNNNNNNNNNNNNNNNNNNNNNNNNNNNNNNNNNNNNNNNNNNNNNNNNNNNNNNNNNN
NNNNNNNNNNNNNNNNNNNNNNNNNNNNNNNNNNNNNNNNNNNNNNNNNNNNNNNNNNNNNNNNNNNNNN
NNNNNNNNNNNNNNNNNNNNNNNNNNNNNNNNNNNNNNNNNNNNNNNNNNNNNNNNNNNNNNNNNNNNNN
NNNNNNNNNNNNNNNNNNNNNNNNNNNNNNNNNNNNNNNNNNNNNNNNNNNNNNNNNNNNNNNNNNNNNN
NNNNNNNNNNNNNNNNNNNNNNNNNNNNNNNNNNNNNNNNNNNNNNNNNNNNNNNNNNNNNNNNNNNNNN
NNNNNNNNNNNNNNNNNNNNNNNNNNNNNNNNNNNNNNNNNNNNNNNNNNNNNNNNNNNNNNNNNNNNNN
NNNNNNNNNNNNNNNNNNNNNNNNNNNNNNNNNNNNNNNNNNNNNNNNNNNNNNNNNNNNNNNNNNNNNN
NNNNNNNNNNNNNNNNNNNNNNNNNNNNNNNNNNNNNNNNNNNNNNNNNNNNNNNNNNNNNNNNNNNNNN
NNNNNNNNNNNNNNNNNNNNNNNNNNNNNNNNNNNNNNNNNNNNNNNNNNNNNNNNNNNNNNNNNNNNNN
NNNNNNNNNNNNNNNNNNNNNNNNNNNNNNNNNNNNNNNNNNNNNNNNNNNNNNNNNNNNNNNNNNNNNN
NNNNNNNNNNNNNNNNNNNNNNNNNNNNNNNNNNNNNNNNNNNNNNNNNNNNNNNNNNNNNNNNNNNNNN
NNNNNNNNNNNNNNNNNNNNNNNNNNNNNNNNNNNNNNNNNNNNNNNNNNNNNNNNNNNNNNNNNNNNNN

NNNNNNNNNNNNNNNNNNNNNNNNNNNNNNNNNNNNNNNNNNNNNNNNNNNNNNNNNNNNNNNNNNNNNN
NNNNNNNNNNNNNNNNNNNNNNNNNNNNNNNNNNNNNNNNNNNNNNNNNNNNNNNNNNNNNNNNNNNNNN
NNNNNNNNNNNNNNNNNNNNNNNNNNNNNNNNNNNNNNNNNNNNNNNNNNNNNNNNNNNNNNNNNNNNNN
NNNNNNNNNNNNNNNNNNNNNNNNNNNNNNNNNNNNNNNNNNNNNNNNNNNNNNNNNNNNNNNNNNNNNN
NNNNNNNNNNNNNNNNNNNNNNNNNNNNNNNNNNNNNNNNNNNNNNNNNNNNNNNNNNNNNNNNNNNNNN
NNNNNNNNNNNNNNNNNNNNNNNNNNNNNNNNNNNNNNNNNNNNNNNNNNNNNNNNNNNNNNNNNNNNNN
NNNNNNNNNNNNNNNNNNNNNNNNNNNNNNNNNNNNNNNNNNNNNNNNNNNNNNNNNNNNNNNNNNNNNN
NNNNNNNNNNNNNNNNNNNNNNNNNNNNNNNNNNNNNNNNNNNNNNNNNNNNNNNNNNNNNNNNNNNNNN
NNNNNNNNNNNNNNNNNNNNNNNNNNNNNNNNNNNNNNNNNNNNNNNNNNNNNNNNNNNNNNNNNNNNNN
NNNNNNNNNNNNNNNNNNNNNNNNNNNNNNNNNNNNNNNNNNNNNNNNNNNNNNNNNNNNNNNNNNNNNN
NNNNNNNNNNNNNNNNNNNNNNNNNNNNNNNNNNNNNNNNNNNNNNNNNNNNNNNNNNNNNNNNNNNNNN
NNNNNNNNNNNNNNNNNNNNNNNNNNNNNNNNNNNNNNNNNNNNNNNNNNNNNNNNNNNNNNNNNNNNNN
NNNNNNNNNNNNNNNNNNNNNNNNNNNNNNNNNNNNNNNNNNNNNNNNNNNNNNNNNNNNNNNNNNNNNN
NNNNNNNNNNNNNNNNNNNNNNNNNNNNNNNNNNNNNNNNNNNNNNNNNNNNNNNNNNNNNNNNNNNNNN
NNNNNNNNNNNNNNNNNNNNNNNNNNNNNNNNNNNNNNNNNNNNNNNNNNNNNNNNNNNNNNNNNNNNNN
NNNNNNNNNNNNNNNNNNNNNNNNNNNNNNNNNNNNNNNNNNNNNNNNNNNNNNNNNNNNNNNNNNNNNN
NNNNNNNNNNNNNNNNNNNNNNNNNNNNNNNNNNNNNNNNNNNNNNNNNNNNNNNNNNNNNNNNNNNNNN
NNNNNNNNNNNNNNNNNNNNNNNNNNNNNNNNNNNNNNNNNNNNNNNNNNNNNNNNNNNNNNNNNNNNNN
NNNNNNNNNNNNNNNNNNNNNNNNNNNNNNNNNNNNNNNNNNNNNNNNNNNNNNNNNNNNNNNNNNNNNN
NNNNNNNNNNNNNNNNNNNNNNNNNNNNNNNNNNNNNNNNNNNNNNNNNNNNNNNNNNNNNNNNNNNNNN
NNNNNNNNNNNNNNNNNNNNNNNNNNNNNNNNNNNNNNNNNNNNNNNNNNNNNNNNNNNNNNNNNNNNNN
NNNNNNNNNNNNNNNNNNNNNNNNNNNNNNNNNNNNNNNNNNNNNNNNNNNNNNNNNNNNNNNNNNNNNN
NNNNNNNNNNNNNNNNNNNNNNNNNNNNNNNNNNNNNNNNNNNNNNNNNNNNNNNNNNNNNNNNNNNNNN
NNNNNNNNNNNNNNNNNNNNNNNNNNNNNNNNNNNNNNNNNNNNNNNNNNNNNNNNNNNNNNNNNNNNNN
NNNNNNNNNNNNNNNNNNNNNNNNNNNNNNNNNNNNNNNNNNNNNNNNNNNNNNNNNNNNNNNNNNNNNN
NNNNNNNNNNNNNNNNNNNNNNNNNNNNNNNNNNNNNNNNNNNNNNNNNNNNNNNNNNNNNNNNNNNNNN
NNNNNNNNNNNNNNNNNNNNNNNNNNNNNNNNNNNNNNNNNNNNNNNNNNNNNNNNNNNNNNNNNNNNNN
NNNNNNNNNNNNNNNNNNNNNNNNNNNNNNNNNNNNNNNNNNNNNNNNNNNNNNNNNNNNNNNNNNNNNN
NNNNNNNNNNNNNNNNNNNNNNNNNNNNNNNNNNNNNNNNNNNNNNNNNNNNNNNNNNNNNNNNNNNNNN
NNNNNNNNNNNNNNNNNNNNNNNNNNNNNNNNNNNNNNNNNNNNNNNNNNNNNNNNNNNNNNNNNNNNNN
NNNNNNNNNNNNNNNNNNNNNNNNNNNNNNNNNNNNNNNNNNNNNNNNNNNNNNNNNNNNNNNNNNNNNN
NNNNNNNNNNNNNNNNNNNNNNNNNNNNNNNNNNNNNNNNNNNNNNNNNNNNNNNNNNNNNNNNNNNNNN
NNNNNNNNNNNNNNNNNNNNNNNNNNNNNNNNNNNNNNNNNNNNNNNNNNNNNNNNNNNNNNNNNNNNNN
NNNNNNNNNNNNNNNNNNNNNNNNNNNNNNNNNNNNNNNNNNNNNNNNNNNNNNNNNNNNNNNNNNNNNN
NNNNNNNNNNNNNNNNNNNNNNNNNNNNNNNNNNNNNNNNNNNNNNNNNNNNNNNNNNNNNNNNNNNNNN
NNNNNNNNNNNNNNNNNNNNNNNNNNNNNNNNNNNNNNNNNNNNNNNNNNNNNNNNNNNNNNNNNNNNNN
NNNNNNNNNNNNNNNNNNNNNNNNNNNNNNNNNNNNNNNNNNNNNNNNNNNNNNNNNNNNNNNNNNNNNN
NNNNNNNNNNNNNNNNNNNNNNNNNNNNNNNNNNNNNNNNNNNNNNNNNNNNNNNNNNNNNNNNNNNNNN
NNNNNNNNNNNNNNNNNNNNNNNNNNNNNNNNNNNNNNNNNNNNNNNNNNNNNNNNNNNNNNNNNNNNNN
NNNNNNNNNNNNNNNNNNNNNNNNNNNNNNNNNNNNNNNNNNNNNNNNNNNNNNNNNNNNNNNNNNNNNN
NNNNNNNNNNNNNNNNNNNNNNNNNNNNNNNNNNNNNNNNNNNNNNNNNNNNNNNNNNNNNNNNNNNNNN
NNNNNNNNNNNNNNNNNNNNNNNNNNNNNNNNNNNNNNNNNNNNNNNNNNNNNNNNNNNNNNNNNNNNNN
NNNNNNNNNNNNNNNNNNNNNNNNNNNNNNNNNNNNNNNNNNNNNNNNNNNNNNNNNNNNNNNNNNNNNN
NNNNNNNNNNNNNNNNNNNNNNNNNNNNNNNNNNNNNNNNNNNNNNNNNNNNNNNNNNNNNNNNNNNNNN
NNNNNNNNNNNNNNNNNNNNNNNNNNNNNNNNNNNNNNNNNNNNNNNNNNNNNNNNNNNNNNNNNNNNNN
NNNNNNNNNNNNNNNNNNNNNNNNNNNNNNNNNNNNNNNNNNNNNNNNNNNNNNNNNNNNNNNNNNNNNN
NNNNNNNNNNNNNNNNNNNNNNNNNNNNNNNNNNNNNNNNNNNNNNNNNNNNNNNNNNNNNNNNNNNNNN
NNNNNNNNNNNNNNNNNNNNNNNNNNNNNNNNNNNNNNNNNNNNNNNNNNNNNNNNNNNNNNNNNNNNNN
NNNNNNNNNNNNNNNNNNNNNNNNNNNNNNNNNNNNNNNNNNNNNNNNNNNNNNNNNNNNNNNNNNNNNN
NNNNNNNNNNNNNNNNNNNNNNNNNNNNNNNNNNNNNNNNNNNNNNNNNNNNNNNNNNNNNNNNNNNNNN
NNNNNNNNNNNNNNNNNNNNNNNNNNNNNNNNNNNNNNNNNNNNNNNNNNNNNNNNNNNNNNNNNNNNNN
NNNNNNNNNNNNNNNNNNNNNNNNNNNNNNNNNNNNNNNNNNNNNNNNNNNNNNNNNNNNNNNNNNNNNN
NNNNNNNNNNNNNNNNNNNNNNNNNNNNNNNNNNNNNNNNNNNNNNNNNNNNNNNNNNNNNNNNNNNNNN
NNNNNNNNNNNNNNNNNNNNNNNNNNNNNNNNNNNNNNNNNNNNNNNNNNNNNNNNNNNNNNNNNNNNNN
NNNNNNNNNNNNNNNNNNNNNNNNNNNNNNNNNNNNNNNNNNNNNNNNNNNNNNNNNNNNNNNNNNNNNN
NNNNNNNNNNNNNNNNNNNNNNNNNNNNNNNNNNNNNNNNNNNNNNNNNNNNNNNNNNNNNNNNNNNNNN
NNNNNNNNNNNNNNNNNNNNNNNNNNNNNNNNNNNNNNNNNNNNNNNNNNNNNNNNNNNNNNNNNNNNNN
NNNNNNNNNNNNNNNNNNNNNNNNNNNNNNNNNNNNNNNNNNNNNNNNNNNNNNNNNNNNNNNNNNNNNN
NNNNNNNNNNNNNNNNNNNNNNNNNNNNNNNNNNNNNNNNNNNNNNNNNNNNNNNNNNNNNNNNNNNNNN
NNNNNNNNNNNNNNNNNNNNNNNNNNNNNNNNNNNNNNNNNNNNNNNNNNNNNNNNNNNNNNNNNNNNNN

NNNNNNNNNNNNNNNNNNNNNNNNNNNNNNNNNNNNNNNNNNNNNNNNNNNNNNNNNNNNNNNNNNNNNN
NNNNNNNNNNNNNNNNNNNNNNNNNNNNNNNNNNNNNNNNNNNNNNNNNNNNNNNNATATATATATATAT
ATATATATATATATATATATATATATATATATATATATATAATTGTAATTATTTGAAATGTATGATGTAA
CACTTAGAAATTTTTATTGTAGGTTGCATACTCATGCAGTACAAGAGCCATTTGTGATTGCAACACATAG
ACAATTGAGTGTGCTTCATCCAATTCATAAGTTACTTGTTCCTCATTACAAAGACACTATGTTTATCAAT
GCATTTGCAAGACAAGTTCTTGTTAATAGTGATGGTCTTCTTGAACAAACTCATTTTCAATCAAAGTACG
CCATGGAATTATCTTCTTACATATATAAAGAATGGAATTTTCTTGAGCAAGCGCTCCCTGCTAATCTCAT
CAAGAGGTAAATCACCAATTACACTATTAATTAAGCTTTTTAATTTCATCGATTAGTCTCTTAAACTATA
AAGGTTAGTTACAATTATATACCTGAACTTTTAATTAGTTGATGAGTCACCTCTAATGGAGTTTGTTGCA
ATTAATTATTTATCCCCATAAAGAATGATAATCATAACTAGTTGCGTAAATAGAGGTGGTTGGGCAATTT
TTCTTTATAACTATATTATGAAAGATGGGCATTTTATTATCCTTTGATTATTTATGCAGAGGTGTAGCGA
TTGAGGATGCAAGATCAACACATGGACTTAAGTTACTCATAGAGGATTATCCATTTGCTGTTGATGGGCT
TGAGATTTGGTCAACAATCAAAACATGGGTTACAAACTATTGCTCTCTATACTACAAAGATGATAGTGCA
ATTCAAAATGATGTTGAACTTCAATCTTGGTGGAAAGAGGTTAGAGAGAAAGGTCATGCTGATAAGAAAA
ATGAAACATGGTGGCCAAAGTTGCAAAATTTCAACGAACTAGTCGAAACATGTACAACTATCATATGGAT
ATCTTCGGCTCTTCATGCTGCAGTTAACTTTGGACAATATCCTTATGGAGGCTTTATTCCGAATCGACCA
ACCATAAGTCGTAGGCATATGCCTGAAGTAGGAAGTGTTGAGTATAAAGAACTTGAATCAAAGCCTGAGA
AGGCTTACTTGAAAATAATCAATTCGATGTTGCAAACACTTCTTGGAGTTTCACTAATTGAAATATTGTC
AAGGCATGCTTCTGATGAAGTTTATCTTGGACAAAGAGCTAGTATTGAATGGACTTCAGATAAAGCTGCA
GTGGAACTGTTTGAGTATTTTGGAAAAGAGGTGTCGGAAGTTGAAAGTAGAATTATCGAAAGGAACAAGG
ATGTGAATCTCAAAAATAGAAGTGGACCCGTTAATGTTCCATATACTTTGCTTCTTCCATCAAGTAGTGA
AGGACTCACAGGTAGAGGTATTCCAAATAGCATCTCTATTTGAAGAGATCAACTCTATGTTTAAACTATC
TATATTATTATTGTGTGGTTACTCTAAATTTATTCCCCAGTAATTTGTGTGTAGCAAAAAGCTACTTGAG
CTACTCGATAAATAAATAAGATGAATAAGAGTGCTTACTAAAGTAGCTATTTACTATGGTGTGTTCCAGT
TTTAACTTTGGTCTACATGTAATGAAGGTGTGTTGTACTTATAGCTTTTTTCTATATGTAACGAAGGTGT
GTTGTACTTCTATAATATTTAGCCTTATGCTTTGCCCCTTTCTTTATTTGCCAATGAATATGAAATAGAA
GTGTGTATTATATATTTCTTTTGAACTATATATATAGTAAATATAAGAATGATCTTGAAATTCACTGAAA
TTATAAATCATAGAAGTATATAATTGTATCGATTTAAGAGTTTTCAAGAGGTTGCCAAGAATCAAACTCT
TTTACACATGAGAATATCCTCATTTTAGTAGCAAATTATTGGTGCAGTGCTAAACCAAAGTTATTATTTT
GTAATTATAATGCTTTCTATATAATCTCTCGATTGGGAGGTTTTTTTCCAGCCTCTCTGAAAAATTAACC
ACAATATAATAATGTCATAATTAAACAACAATATCCATGACGAGAAAGGCAATTAAGAAACAAACAAAAA
TAATAGAAGGATTTAGAGTTTCGTAACTTAGTTTGGTGTAATATCATCTACATTTTGGGAGTTTACATAC
ACAATGGAAAGAAAACTTTATAAAAATTAATGGCTATGGAAATTAAGAGAGATAGAAAGAACAAAACCAA
GTTGATACTATAGCATAAAACTACAACAGAACTTGGATACAATACCTATTTGATAACATGAGATTCTCCA
TCTTAAAACCAATTGACAATATGTGAAAATTGACAAATTATTTAAGAGATTAAATAATGAGATTTTTTTC
TTTATTTAACATTGAGGGCAGATTGGTAATTATGCCTAATGGCATAATTGAAATTATATCAATCTTATTC
TTTTCTTTTAGGTCAACAAGAAAAGAACAAAAGAAGAAGACAAGAAAAAGAGGGGCAGCCGACTTTTTTG
AAATACAAAAAAGAAAGAGAGAGAGTGACGGCGAAAATCAGAAAGGCGCGGCGGAAGATTGATCGGTGCG
GCGCGGCAGTGATTTTTCCGACAACAGGTCGGTTCTTCACAGCGAGAGGCGGCGTCGGTTCCTTCGGTAT
CGTCGAAGGTGTTTTCGGTGGTCTGCGGCGTCGGGATTTGCTTCGGTGTTGTTTCGGCATCTCTTCAGCG
TCGGTTTCTGGGTTTATTCCGACGGGCAGCGTTGGTCTGTAAGCGGCGGTCGACGGTCAGCGTCGTTCTC
TGTGGGGTCAGTTCAGCGGGTCTTAGTTTTTTCAGTTCAGCTAGCATCCATGGGTCTCTCATTCTGATTG
ATGGTATATTTTGGGTTTGCCTCTTAAATTACAGATAGTTGATTTGTAAATTTCTATAATTGTTCCTCAT
ATTTGCTCTAAATAAAATCCTCCCAAACTTATTCTCAAAGTGGATGTAGACCAAATTGGTCGAACCACTA
TATATCGCTGCGTTCTTGCTCCTTTACTGTTCTCTGATTTATTCTCAATTGTGCATTCTGGATTAACATG
TTTAGTTTCACAATCTGAATTGGTAAAGAAAATATAACACAATAAAATGAATAACACATCTATTGTATAA
AGAGTTTGAGTTGTTTTCCACGTACCCCTTAAGATGTTGTCTCTCTTTTAGGTTTAGCAATCTCCATCAA
CCCACAATTTATTTTTATTAGATAGAATACTCCTCATATCCATCATCAATTAGTATTGAAATAGAACCTC
GTACTGGTATCAACTCAATACTAGTACTAAAACTCCAACTTCAATATTTTTTTTTTTTTTTTGTTTTTGA
AAAAGATACTTTGGGTTAGTTGGGTACACCCAGACATCTTCACTAGGTGGACCCTCCTTAGCACCTTCAT
CACTCCTGCTTCATTAATATCAATAAACCAAAAGTACAGGAAGCAAGGAGAGAAAAGGGCTAGAGATAAG
CCCAAATCAAACAAAAGCTAGCATTAATGTTTAAAGCTATAGTACTAGCATTGTAATCTAAGAAAAGCTT
AGATCTGCTGCTCCAAAATCCGGTCAGAGAGACAGTGTCCTCCCAAAGCATGTTTGATTCTTTTTCAATG
CTGTTGAAAGTACGGTTATTTCTTTCGAGCCATATCTTCCATAGGACCACAATAGTCAAAATGAAAGTAA
TGGACCCTTTCCTGTTCTTAGTGTTTAAGCCACACATATCCTTACATAATGAGCTCACGGAGGTCGGGCT
TCGATTCAAGTTCATAATAGACTTTGTTTTATTCCATATTGCCTGTGCATAAGAGCAATGAATAAAGAGA
TTGGTTAATATTCTTAGCATTAGATTTACACATGGAGCACCATTATCTACTACTACAAAAATAACTCCAA
CTTCAATATTGAGTTTGAAAATCTAATTAAAATGCAAATCCATACTTCATTCGCTAAAAATAAAATAAAT
TTTATTGAAGAAACTTATGGGCGGCCGACCCTAATAATAATGCTCCAAACTTCGCCACAATGACATTTAT
TTTCTAACATATCCTTTAGTTTTTATACGATTTAAAGCAACGTCAAGAAGCAGATACCTAACATTACTCA

TTTATTTTTCTTATCTTATTTGCATTTTTAATACCTATGCAAGGGTGGCAAAGAGAGTATGGTGAGAATC
TTTGGCAACCTCACCCCAAAAAGACCTTGGAATGCTAAAAGGATAAAAGGTGGCACCACAAAATTCATCA
AATATATGTATAACCTTTTTTTATATATAATAATGACAACTATAAATGATATGTTACTGCTACAAAAATT
GGGATACGGGATATTATTACAATGCAAGCAATCGAATGGCAAGTTTATAATGTGATCATTTGATTCTAAA
ACTACGTTTAAATTCAAAAATCATTCTTAAAACACAATCATTATTCTTAAAACACACATTACTTTTATTC
ATCAAACACTAGTACAAAGTACATAAATTGGAGATAGTTCCAAATCCAAGAGAGCTGGATACCTAATTTA
AACTAAAAATATGGTCATTTATTTCCTTCATAATAATAATAATGTGGGCAACTTAGAAATGTAATTTGTG
ACCTTCAAATTACACACATCACCCATAAAATGAACCACCTTTTTTTTTTATCAACTTAGATGGAAATACT
ATTAGGAATTCCTCTTGCAGTGAGTCCTTCGTTACTTGATGGAACAAGTAAAGTATAAGGCAGATTAACA
GGTCCAGCTCTATTCTTCAAGTTCACATCTTTATTCCTTTCCATAATCCTATTCTCAACTTCAAATAACT
CTTTCCCAAATTTCTCAAATGCTTCCAATGCAATTTTATCTGAAGTCCAATCGATTGAAGCTCTTTGTCC
AAGATAAACTTCATCAGAAGCATGCTTTGACAAGATTTCAATAATTGAAATACTAACAAGTGCTTGTAAT
TCTGAACTTATTGTCCTCAAGAAAGCTTTTTCTGGATTCGATTCGAGTTCTTTGTACTCAGCCGTACCAA
CTTCAGGCATGAACCTACGACTTGTAGTTGGTCGATTGAGAATATAGCCTCCGTAGGGATATTGTCCAAA
GTTAACTGCAGCATGAAGAGCTGAAGCAATCCATATAATTGTAGTGCACGATTCGATTAATTCACTTAAA
GTTTGCATTTTTGGCCACCATGGTTCATGTTTCTTGTCAGCATGGCCTTTCTCTCTTAGCTCATTCCACC
AAGATTGGAGTTCAAAGTCATTTTGTACTGCATTGTCATCTTTGTAGTAGAGAGAGCAATAATCTTGTAC
CCATGTTTTAATGGCTGACCAAATGTCAAGACCATCAACAGCAAATGGATAATCATTTATTAGCAATCTA
AGTCCATGGGGGGCACTTGAGTCCTCCACAGCTACTCCTCTGCATAAAATTAAACCAAAAAGAGCAAAAG
GAAAGTCAATACATTATGTATATAAGAATAACATTCAACTAAGAAAAATTATGTAGGGATGTTTTTAAAT
ATAGAGCAAAGTTAACCAAAATATTTATAGAATATAATAAAATTTTAAATTCTATAAATGATAGATATAC
TAATAGACACTCATTATTGGTAAAATTGATAGAAACCAATAGAAGTCTATAAGTATCTGTCACTGTCTAT
CATTGATAAAACTTGAAAATTTACTATATTTTTGTAAATATTTTAAGTAGTTTTGTAATTTACAATAGTT
TTCCTTTAAATTTAATCGTATCAAACATTTAAATGGTTACTCTCACTCAATTGTATAAGCTTCAAATTTT
GAAGAGACTTACTAGAAAAAGTCAATATTAATCAACGGAATAAATTAACTTTCAATGATGAGACAAAACA
AAAAGAAGACCATGTTATCACACCAATCACCTAAAAAACTTGTAGGTAGTGATTAAAGTTAATTATATAT
AAAGTAGTTGAACAAAAATGATGATTTACCTCTTGATGAGATTATTAGGGAGTGCTTGATCAGGGAAGGT
CCAATCCTTGTACAAGATGGATGACAACTCCATTGAATATTTTGATGGATAATGGGTTGATTCAATAAGA
CCATTGGCATTGATCAAAACTTGTCTTGCAGATGCATTGATAAACATAGTGTCTTTGTAATGAGGAACAA
GCAACTTATGGATTGGATGAAGCACACTCAATTGTCTATGTGTTGCAATCACAAATGGCTCAAGTACAGC
ATGAGTATGCAACCTGTAGTTTCAAAAGGTTAAATTAGGTAAATATCATGAGCCCATAAAAGAAAAAATG
AATTTGGTAGAAAGTTAAAATTTAGTCCCTATGATTTGAAAAAAATTTGTAATCTAAGGTTTTATTTTAG
AGAGAATATATATACCAATGACTAATAAGTTGATGGTAGCCAACATCATTAACAGTTACATAAGCTTTAG
CCAATTGCCAAATGGATTTTTGAACTCCATTTTCAGCTGGAAAGTATAGTTTGCTAATGGCACCAAGTTG
ATCTCCTTGAGGATGTGGCAAGCTCAACTCAATAACCAATGGCTTCAAAGTCCCGTCATCTTTCAAAAAC
AGCAGCGTTCTTGTGGCATATGTTTTTGTTGTTGATGTTGCATTCATCCTTGTAAGATATGGCATTAATG
CATCATGGAAATCCACTATGTAGAGCTTGTTTTGATTCATTGCCTATTATTCAACCCAATCAACGACTTA
TCAAAAAAAGATAATAATAATGATAAATAAATAAATAAGAGAAATACACATCTAACAATTAGATCCAAAA
TAATAGCATATATAGCAACATTTGAAAAAAATGTATATAAAAATATAGACCATCAGTGATATAAAAGTCG
AACAGCGTATAAATCTGGTTGATAGACGCTGTTATAGATTCATTTTTTAAAAAATTATTATATGTATACT
CGATTATTATTCCTAAAATTGCTATCTATTATAATTACTCAAAAAAAAATCATTTTTGACGTAGGTTCAT
GATAAAAAAAAAAAATGTTTTTTTCAAGTTCTTTTTACTCACCTCATCAACTGTGAGGCCATCTAAACCA
TGCTTGATGTGTTCTTCAGTGATGGTACTGTTTTGATTCCCATAAACATTTGGGTCAAGCTTACTTGTTG
GTGGAAAAACCTATATATAATTAATAAATTGAGATTAATTAGAAATTGTGGTAGAAAAAAAATCATTTGA
GGAAATTGTGGTAGAAAAAAAATCATTTGAGAAAATTGTGACTAATTATTTGTTAAAATAATTACTTCAA
GACGACGAATCAATAGAGGATTGGGTCCTGCTAACATTTCTCTTGCAAATTCTTCATCAGTGCTCCATCC
TATTTTATTATCTGTTCAAAAGTAATCGTAATTAATTAAATATGGTCAACAAAATTGTATAAACAATATT
TAATATTAGTATATTCATTCCTCACAATATTTGAAAGAAAAAGTTCATAATATCTAAAATTTTTGTACCT
TTGACAACTTGGGGAGTAGGAAATTTGAGGAATTTTTCATCATCACTCCTCACAAGTGCTTTGAACAAAG
GTGGTGTGAGGTCCTCAGTGAGGGTCTTAAAAGCATTAAATGGAATGGGAAAGCCTCTTTCAAAGAGATT
ATCAACTTCTTTAAAGTTGTCAAATTCATTTGGAGATACATCAAATATGGATTGAAGTCCTGGTTTGATT
GATATCGAAAGTGCTTTTAATGTATAACCAAGGAAATCTGACATCTTCAAATGCCCAAAGTTTTCATCTT
TTGGTACATAGATGTCTAAGCTCATTAATGGTGACAATCTGCTCTCGTAATTGTGGTCTGCCATAATGAA
ACAATATGATAATGTTAATAATTAGAAAGCAAATAATAAACTTTGTACGAAAAATTGACTAAACTTATAC
GTATAGTTTAAATTTATTAGACGCAAAGGCGACATTTCATAGCCAAAGTTAGCATGACTAACCAGTAAGT
AATTCACATAATCTACTACCTCCCATATCCCCTATTAAAGGTTCAAATTAACACGTATTTGTCATAAAAA
AATCTTACCTCTTCTTGATCGTGGTCGTCCTGTTCTTCCCCTACGAGGGTAAGGATATTCGGTCGTCCCA
CCGAGAATAGGACGATGGTCACCAACATCAGGGTCAGCAATGTCATTATAAACATCATAGTCATAGATTC
TATCCCATTCCTTTCTTTCTCCGGTTCCATCTCCTCTCAAATTCCACAATTCTTCCTCTCTGTACTTACG
AAGAGGGTTTGGTGTTTGATTTGGAAGATAAACCTATACATAGAATACCAATAATTAAACCCAAATTTT

ATTTGAAAACTATGTATTTGTGATGACACATATCGTAGTCCATCATGGTTTATCGCAAAAAGACAATGAC
ATTTTTTTATATTACGTAAATAGTTTTATTTTTTGTCGTTTAAAATAATTTTTCTTACATGATTGGCAAA
GAATATGCGATCTTTCTTGTATCTTCCAGAAGGGTAAACCCAAGAATTGCAATCAAAATGGACTCTGCCA
TAGCCAGGAACATCATCAAGAGTGAGAGATTTGAGAAAGAATTCACTTGTATGTCCATTTCTTATGAAGA
AAGCTCCTGGAAATCCAAAGTTTTCTTCCCATGGAAAGTTGATTTGGAACACTGATTCTCCGGCAAACAG
TGGTGGGATTGAAGTTAGCCACCTCTCTAAATATGCCTTGTTCCCAACTTTCCCTCTTGAGTCATTTGCT
GTCAATTTCATCATTAGGTTACATAATTTAATTCAGTAAACAAAAGATCAGAGGATTTATATATATATAG
GACTGGTTTGGAGTGACAGACTGAGATTTATATGTGTTTTTAAGTGTGCTTAAAATTGATTTTGAAATTG
TCGTGTTTGAAACCACCGTTTAAAAAATGTGCTTTAATGTTCAAAATTAGCATAAGCTACCGTTTTAGTT
ATTGATCATTAATAAATTAAGAAAATGAAAACTTTGAATATATTTATAGAGAGTTTTATAAAAGATTTCT
AATGAGTTCAAGAGATAATTTCTAAATGGTCTAGATGTGAAAATAGACCATTTAGCCTTTGTTAACCATT
TGATCAAAATAACTCCAAAACACAATGTTAGATATAAAGAACAACTGAGAGAGTAGTGTTTTTAAGCACT
CGAAAGAGAGTTTAGGAGAAAAGGGAAAATTACAAGTATGAGTGGCACTAATGAGTTGGATAGAAACACC
ACCACCCAAGAGCTCAGTGAAGTTATCAAGAAGAGATGAATGGAATTCAGTGAAATCCAAAACATTACTT
CTCATAAGAATCACTTTCCCTTTGATTTTCTTTCCTCCAAGATTGGAAACTCTATCTAAAATGTTACCAC
CAGCATTGATAACAGAACCTGCAAGATCTCCAGTTGTATTCAAGGCTCCTTCAATGATGTTCTTCCCAAT
TCCAAACATTTTCACTCTTTTTCTAATTCCTTTTTTTGCTCACTGCTGTGTGTTTGGAACTAAGAAGTTG
GGTTTGAGAAAAAGGATTTTGGGGAAGGTATTTATAGCACAAAAAAGTTGTCTTTCTCTTGGAATTAGCA
GATATGAACATAATCATTCAATGTTGGACTACTCCACATGATTCACATTTTCAAATGCCCTTATCAAGGG
CTATATTTGAAACTTATTTATTAAATAATATTATCTTTGGTTTTTAATTGATTCTTTTAGATATATATAC
TCAATAAAGGTATAACTTAATGTATATTTGTACACAAAAAGTGGAGGCATGAAGGTTACGTTGTGGGCAG
ATTTCGACTGGAACCTAATTTCAAAGGTTCTTGCCTTGATCCTTCTCTAATGCTCTTGTTCTCTCCTTTT
CATAGTTTCATAGGCACATAATTATATTTTGATATGATTCAAAAATGGAAAGCAAAAATTACACTAAAAC
AATTAAAGAAATGTTTTTTAATAAAACATTAATTACAAATTTAGTATTTTTAAAATAGGGACGAAACTAG
TTGAAAATAAAAAGGGAAATTTGTTAAAAATAAACAAATAAATAAAACAAAGAATATATTAAAATTGGGA
AATTTGTTAAAAATAAAAAAATACATTACAGATTTGTTTTAAAGATACGAAAATTAACAAATTATTTATT
TTTCGTAGAATAAAACTACTAAAATACAAAATTTATTACATCTTTTCGTTAAATATAAATATTTTATCAA
ATATTCTATTTTTACGGTTTTCTTCTATCAATAATCATATAAGATAAAAAAAATGCAAATCCCACTTCTT
AAGGGTGTCCATTGAAAAGAGTAATATATATATATATATATATATATATATATATATATATTTATTTTTA
TTTTTTTTATTTTTCAAAAACAACCTTTTCCTATTTCTTATAAACTAATAATTTATATTAAATTTAAACT
CTAAATTTCTAATTATATTAATTTAAATTCTAAACTTGTAATTGTATTAATTTAAATCACGAATTTTTTG
AAGTTGTATCGATTAAACTCCAAATTAATAATCGGACCATGAATTTTTATAGATTTATCCAAATAAAACA
TAATAGAAGGTTTAAAGTGCTACAATTGTATAATTTCAAAGTTTAAATTGGCACCCTCATAAAAATTTAG
AGTTTAAATCGATATAATTATTAGTTCATGATCTCAATTGATATAATGCTTATAGTTCACGGTCTAAATT
GATATTTCTTTCTTTTTCTTTTAAACTACACATGCTATCCAATTAACCCTAATATCCTTCATCAAAACAC
ATTTCTACTAATTTTAACCTTTTAATAAAATCACCGGAATTGATTAAAAATTTAACATGAAGATAAAATT
TTCATAGAAATAAACTCTATACAAGAAATAATAATAAGGTTAAATTCCTATTTTAGTCTTTAAATGTTGT
ATTTTGTTCTATTTTGTTCTTTGGACTTTTAGCAATATCTATTTTGGTCCTTGAACTTTTATGAAAAAAA
AACTTATTTGATCCCTGAACTTTTAAAAGTGTTTGTTTTGGTCCTTGAATTTTTGTAAAAAAACGTATTT
TTGTCCCTGCATTTAGAGTTTCGTTAATTTTTTATGGAATAATGACATGATTTTTCCATATTTTGATGAT
TAGGCTTTAGATTTATCATATTAATTAAATTGATAAATAACAAAAAAAAATCTTGCTAATTTATTCCATA
AATCTTTTATGCCAATATAGAAATGTAATTAATGCACTCTAATTAAATAAACTCAAATTAAACAATTTTA
TTTTATTTTTCTCAAAAATTTTAACCTCTCACATCTACTCCGTGGACTCCCCACCCTTTACTTATTATTT
TTCTCAAATTGTAATATATCTCGAAGCAAATCAGAAGAAGAGTAGAATAAAAAGAATAAATAGAATTGAT
ATATTGGTGTTTAATATCATGACAAGGTAAAATAAGCTAGAAAAATAAAATAAGTACACTTATTTAAAAT
AAAAATAAACTCTACGAAATTGTAAACAAATATTCTCAAAATCAATCATAACCAAAAAAAAAAAGAAATT
ATTGGGACGAGGAGAAAAATCACATCAGTAAATCATAAAATGTCTCAAACTTGAGCCACTTGAAGATTTA
TATGTGTATGCATTGCATCAAACATATGAGTTGAAAAAATAAAGTAGAATGACAATGTTTCAAAATGACA
TCGATCAAAATCGAGAAGATAATGACATTTTGAATTTTTTCTATAAAATATCAATTTATCATCTAATTTT
TAATCGAAAGTTAAAACTCTAGATTATTAAAAATGATTATCTTATTCAGAATCCTAATCATGATGATTAG
GGACGTAGGTAGAAATTAAACCTAATAACAATTATAAAAGAGAGACGATTTATTCATTCTTCAAAAAAAG
ATAATAAAGTTTATATTTTATATATAATTTTATAAGATGGGCATTGAAACTTGCAATTGTTCTTTTTCCA
TATCTTATCGGTAACAGTAAGTGTGGAATTTTCCTCTGTCTTATTCTACAAATTCATAGATTGTCCCATC
CTCCAATTATTCATAACTCAACTCACTTTTTTTAATTATTATTACTATTCATGATGTTCATATATTAAAT
TATTATTTTCGTCTCTATAAAAAGTATAGTGGTGATTGAATTTTAGTTTATATTTTTTAAATGATTACAA
ATAAAATAAAAAAATTTGAAACTATTTTCACCCATAAAGCAAATGTTAAAAAATTGGTTTTGGTTCTTAA
ATTTTCTTTTAGTTTTAGTTTTTCAAATTTAGTTTCTATATTTTACTATGTAATAACTTAATTTTCATTT
TTAAAATTTATTATAATTTACTTCATGTCGTAAATTTATTTGTAATTTTTAGAAAAATAGTAAATCGGTT
AGTCGAAAAATGAATTTTGTTTCTAATATATATATATATATATGATACAAACATTTTATATGTAAAATTT
AATACTCAACAGGTCATAAAAAGTTTAAATGCATGTATTAGAGAAGAAAAGAATAGAAAGAGTTGTAAAA

TTCAAATAAATAAGAAGGAATAATATATGTAAGATTAAGGATTTATGATGATAATATACTTTCCCACCCC
AAGACATTTTCTTTATCAGAAAAATCCATAGCTCATTTTCTTTGATATGTGAACTATTATTACTACGTAG
GGAACACCGCTACATAATTATTGATATCTACATATGTCAGAAGTTTGGAAGCTTTCTATTTTAGGCATGT
GCAATATGATATAAGTTTTTCTTATAAATATATCTGTATCAAGTATGTATGTATCAACAGTGCATCAAGG
ATACTTTGTATTCTTTTTTATAAATTTATAATTACTCGTTTCCGTAGCTAGTTGTTATTTTTGTTCCACA
TTATTTTAATAACAAATTATTTCGTAATTGTAAATATATTGATTATGTTAGATAAAGTATAATACAAAAG
CAAATGTAAGCATAGCTCAACTGATATAATGTTTATATTTTTAACTTTGACTTTAGAAGTTTGATTCTTT
TATCAATACTCTAATTATATTACATTTATGTAAGGAAAAAAACCATAATGCGGCAAGTTTCATAAATATT
TTAAAATTTTAGATTTAGTTCGTGAAATATCTTTTAATTTTGTCATAAGACTCCTGAATTTGGGTTATAC
TTACAATTCATTTAAAATATTGGCATACATTTAATTATTAGTGGTAAGTGTAGCTACTTAATTATTGCAA
TTGCAATAAAATTATTTATGAATGAATTTGCTAATGATTTCATGTATATTTTGGGTTAATTACCATTTTA
ATTTTTATATTTTGAAATTGGTTCAATTTCAGACATTTTACTTTTAGTTGTAAAAACTTTAGTCTGTGTA
CTTTAATAAACTTAAATATATATAGTTGATCCCTCTACCTCCTTGTTAATTAGCATTCTCTTTTTCTATC
TGATCATGATAGTTCTTTGATTCTCTGATTTGATTGATCTACTAGTTTTTCTTAGGTAAATTGTATTGGG
TGACACAATAAAAATCTAATTTAATAATATTAGCATTAATCTTTTAGATTTTGCAATTATAGCAACTTTT
TAATTTTGGTTGATATTTCTTATTTTATTTTTTTCATTATTCCAAAATTGCATTTCTCTACTGTTTTCTC
TTCCTCTTTCGTTTTTCTTCAATTGTTCTCCTTCATTTTTCGTTTTCTTCTTCTCTTCTCTGTCGTTCTT
CTTCACCATTTCTCTTATTCACCTACTTCTCTTCTCCTCGTCTTCTTCTGCCTCTTTTCCACTGTTCTTC
TCCACCGTCCTTCTCTTCTCTCTTCAGTTTCGTTCTTTTAGATTTCTCCCTCTTCGTCGGTTTCTTTTTT
TTATCATCTTCTCCTCGTCTTCTTCTCCTATTCTCCTCATTTTTTTTCAGGAAAAACAAGAATTATGTAT
ATATTTATTCCCCTTTTCTGAAGCCCTAATATTATTTCTGTGAATTGCTAATATATTATTTATTAAAATT
CGGGCTACGATTTGTTCTTCCTCATCTCTTTTATTTGTTCCTAATATCTTTTATCGAAAGGAATTTCGTA
GATCAGTTTGGTTTTATTTAGTTACGTTCGGTTATGTTTTTTTTTTAATTTGTATTAGTTTTTTTATATC
AACAATATGATATACTTATATTATATGTATTAGTTGACTGATATACTTACTTCGTGTTTTTTATTTTCGC
AAAATTGTTGCAGTTTATTGTAGTTATGGTTAAATTGACAGTAATTGTTTCTCATAGTGGTCAATGGGAT
GAGCAACATTATTACGTGGATTATAAAACAAATTGTGTTTTGGTTGATGGAGTGATATCATCATTTGATT
CTTTTGTGAACTTGATTCATACTGTAATTCAGATTGAGTCGTGTATTGAACTTTCAGTTTTAATTTATTA
ACTATAAGCGATAATGATGTTCAACATGTTACTAAGATTTTAAACATACCATAACAACTGTATTGACAAT
ACATAAAGTGTATCATACTTAGTGCATCAGGTGTATTTAATTAATTGAGTGTATCATGTCAACTCGTTGA
CATAGTTGTTGTATAGTTCACATAGTTTGATTATTAATGTATCAATGAAGTTTAGCAAGTGATTAAGTGT
ATTAAATATATCGAGTGTATCAAGAAACAATAATAAAAGTATCAACGATATATAAAGTGTATCATTCTTA
GTGCTTCAAGTGTATTTAATTGATCAAGTGTATCAACAACATATCAAGTGTTTTAAACTAATAATATGTA
TCAGTGAAGTATTATAGAATAAAAAAAAGTGTACGAGGAGTACATCAGCAAAACAAATCAGGTGTATCAA
TGGTATATATTGGTGTATCAACCGTATATATTGGTATCAGTAATGACTGAAGGGTAGCTTCGTAATTTTG
TATTTTTAAAATGCGGGCTGGGCTTCAATTTTGCTATTTTTGCAAATGTAAAAATGATGTGCTATGAGCC
TAATTTATGATACTATAATCGTCATATTTGCAAGAGCTCCTTTTTCTTAACATTAGCATATTTATTTGGC
TTTGCATGATAACTATTATATTTTACCGCTCATTGGAGACCTACTTTTATCTATATTTGTTTTACCGCTC
ATTGTTTTACATTGGAAGGCTTTCTTCTAGCATCTCCTTATTTGTAGGAACTTCTTTTGGTAGAAACGGT
CTAGAAGGTTTAAAATATCGATGTGGATAGAAATATTAAGGTTTCGATTTTGTGAAAATATATATCAATG
GAAGTATTGATATCAAAACTTTATGAAAATCGATGGAAATTAACGAAAATTATTATAATTAGTTAATGAA
ACTTTGATAAATTTGCTTATATTATAATTGGTCATTTTTAATCATCATTTCTATAAAGTAAGACAATACT
TTGATGTCTATTATAAATGTCTTTGTAAATCTTGATATGAGAGCATCAATATTCTTAAAATTAGAAATAC
AATTACAACATGATATAAAATTTAGGAATATTTTGTGAGTAAAATAATGTCAAGATGGTAAAAGAAAAAG
AAACTCGTATCAATTTAAATTTTTAGGTGAAATGATGTGAAAATCTAATAACAAAGTTAAAATTTGGAAA
GTTATCAAAAATTACAAATTTGATAAACCATTTACAAAATATAACAAAATTTCAGATTTTATCAATAATA
GACATCGATAAATACTAATATGTTTCCATCTGTGATAGTAATAAAAGTATATAAGTTTCTATCAAAATTT
TATTATATGTCGTAAATATTTTAGTTTATTTATTAAAAACGTCCCCTGAAATTAAGTATGGCTTTTTTCC
GTGTGATTTTTTCAAATTAAATTTCTCTTGGTAATAAAAGCTTTGAGTTGAAAATAAAGGGAAAGTTGAA
TAAAGTTGGAAATTAGAAAAGAAAAAAGAAGAAGAAGAAGCAATAAAATATTTTAATTTTTATTTGGATA
GAAATTTTACACCAAAGAAATATAATTTTAATTTTGTTTCTATTAATATATTAGTAAATATATCACTGTC
TCATGATGGGGATCACTGCCCAAAACTCATATGATTCACATTTTAATTGTTTCTCATTTTTCATTTTTAA
TCCCATAAATTTTGTTTGCAGCCTCATCTTTTCATAAATCTCATATATCTTGAAATGCTAAATAGGATAA
GATAATTATCTTTATTATATTCTTTTATTTCTAATAGACCCAAAACTTCATTGCTGTTGACAACTCTATC
AATAATAATATACATACATACATACAAAGAAAAAAAATGTATAATTTAAAAAGATTCTATCAAATTATCA
AATTGAAGGTTGAATTTTCATTAATTAGTTCAATCACTTGTAAATTATAAAATTATCAAACATTTTTTAC
CTCTATATTTTTCATAAATTTTCATTCACTGGTTTTGATTCTTTTTTATTAAAAAAAAAGTCAATTAAAA
AAATATTCAAAATTTGATACTTTTTCAACAAAAATAGAGTTCATATTTTCGACAAAAATTAGGGAGATTA
GTAGTTCACAAGATCAAAGATGAAATAAGTCATTCAATATTTAACATTCTGAACAAAAATTCAGTTGGAT
TCATTTTTTACGGAACTTTATTTAATTAACTTTAGTAAAAAATAAAAATAACATATGGTAACGTTTTATC
AGACTATACTTGAGATGCATCAAAGATAGATACATTCAAAAGAGCACCCAAAATTTATATATGTGTGTAT

ATAGATTTTCTATGGAAAACTAGGAGGCGAGGAACATAGATATACTTCCAATAAACCACACTTTCAAGCA
AATAGATATATATGACACTCGTTGAAAATGTGACCAAAGTTTTACATCAATTAAATTAAAAAGAAGATAA
TCATGAATCTATAAAGGAGAACAACTATCTTCAAATAATGAAATGAGATCTCTTTGGATGATACCAAAAG
CAAAAGCAAGAGAGTGTATGGTTCGTAGCAGTATTGATGTTGATCGATTCCAAATCTTATTATCTTATTC
CACTTTTCATCTATACCTAAGAATCGATTTGATTATTAGGACATTCATTTAGCTTTTTTCTAAATGTGAT
TGGCTAAACCATCAAAACTTGATTGACCACTTTCCCTAATCAAAATCTAAAATCTAAAATTTATCATGCA
TTATTCCAATATTAATTATTATTACAACTTTTCACATCTCCATTCCTTCATGAACTTTTTTTATTGATTC
AATTTTTCTTATCCTTTTATCAAATAATTTAAATTAAACAAAGTTAATTACCATTTAAAATATAATTTAT
GGTATTTTAAAATTCTAACATTTTAGATTCTATTAAAGGGCATTCTCAAATAAATAACAAAATATTGAAA
CTATATACATAATATATCAAAATCAATCCATCTCTTTTCTTCATTTAAGTTTTTTTCTATATATTTTTGT
AAAGAGTTTATTGTTATCTATAACAAATTATCCTTCTATTTTAGTGACAAACACTGGGATAGAGCCTAAA
ATTCTGTTATATTCTATAAATATTTTCACCAATTTTGTCATTTACAATCATTTTCCTAATTTTTTTTTTA
GGTACATATATAGTGACAAATTATAACGCAAATAATTATTTGAGATACGCCTAATTTTATTCACATAAGT
AAATCAAACCAATTTCAAAAAAAGTATTATATTTTACCAAACAATATAACTATATTTCTAAAAATTTAAA
ATTCAAATATACGAAACACTATTTTACTTCATTTCATCCAACGGGAACAATATTATTTTAAAAGTTTCCA
TAATCCCAAACAAAGATATAAACAACCAAACAACGTAGCTAATTTATTTCCAACATAAGATGGTTTTATA
AATATCTTTCTTCTCAACAATCTAGTTTTAGGGAGGAAAAAGAAATGTAGTTTTTTAGATAGAGATACTG
TTAGGAATTCCTTTGCCAGTGAGTCCTTCGTTACTAGTTGGATAAAGAGAAGTATAAGGCACATTTATTG
GCCCAACTCTATTTTTCATCTTGGGATCTCTATTCCTCTTCACAATTTCATACTCGATTTCTTCCAATCT
TTTCCCAAATTTTTCAAAAGCTTCCAATGCTGGCTGATCTGATATCCATTCAAGACTCTCTCTTTTTCCA
AGATAAATCTCATCAGAAGAATGCCTTGACAAGATCTCAATCAATGATATTCCAAGAAGTGTTTGGACTT
GTGAATTGATTGTTCTAAGAAAACCCTTTTCCGGATCTGACTCGAGCTCTACATATTGAGGAGTTCCTTT
TTCAGGCATGAATCTTCTACTTATTGTTGGCCTATTGGGAAGGTAGCCTCCATAAGGATACTGTCCAAAG
TTAACTGCTGCATGAAGAGCCGAAGAAATCCATATTATTATAGTGCATGTTTCAATTAACTCTTCTAAGG
TTTCCATTTTTGGCCACCAAGGTTTGTCTTTCAAGTCAACGTGGCCTTTCTCTTTTAGTTCTTTCCACCA
TGATTGGAGTTCAATGTCATCATGAACCATGATATCATTCTTGTAATAGGATGAGCAATAATCTTTTACC
CATGTTTTGATTGCAAACCAAATCTCAAGTCCATCAACAGCAAATGGATAATCCTCTATTAGGAGTTTGA
GTCCATATGGAGAATTTGAGTCTTCAACTGCCATTCCTCTGAGGGAGAAAAAATTGTTTTAATAATTAAT
GATATGTTTGAGAGTCAAGCCAAAACGATAAAAAGGTTAAAAATATTCAAGATCAAAACCTCAAGCATCA
AACTTTAAACTAATTTTGGATGATTAAAGACATTTGTTGTACAAAACTTTCTTGAAACATGCCCTAAATC
ATGAATTATTTTTCATTATCAAATAAGCCTAAAAACTTTCCATTTTCATAATTAATTAATAAAGGTAATG
AATGGTCAAGTATCTATCATGTTCATGTATATGTGTAGTTAGGGTTACTTGCCTTTTGATGAGATCTCTT
GGGAGTGCTTGATCAGTGAAAACCCAGTCTTTATAAATATAAGAAGACAACTCCATGGAGTACTTTGATT
GAAAATGAGTTGATTCAAGAATTCCATCTGCATTAACAAGTGTTTGTCTTGCAAATGCATTTATATTCAT
GGTGTCTCTATAATGAGGAACAAGCAGCTTGTGAATTGGATGAAGCACACTTAATTGTCTATTTGTTGCA
ATCACAAATGGTTCCATTACTGCGTGTGTATTCAACCTACAACAAGAAAAAACACATTGATGACAGTCGT
ATTGTTTGTGAAGAAAAGATAAAACCTCAAAAGTCTTTAGCCAGCATCATTGGGGGAGTAATACCAATGG
CTAATAACTTGATGGTAGCCAGCATCATTGACAGCCACATAAGCTTTAGCTAGTTGCCAAATTGAGCTTT
CAACTCCATCTTCAGCTGGAAAGTACAGTTTGCTAGTGGCACCAAGTTCATCTTTCTGAGATTGTGGCAA
GCTTAGCTCAATAACCAATGGCCTCAAAGTTCCATCACCATTTAAGAAAAGCAATGTTCTTGTGGCATAA
GTCTTTGTAGATGTTGAATTGATCCTATTAAGGTATGGCATTAGGGCATCATGGTGATCCAATATATAAA
GCTTGTTTTGCTTGAGTGCCTAAACAAATAGAGTTAGTGCAAATTAAAGTTACTATTCTCATATATCAAC
TATATTGTAAATGAGAAAATAAAATTATGAAATGCCTAAACAAATAGATAATAGAAATTTCCTCTAATCA
TTTCCTTTGTTAATTATATATGTTTTAATTTTAATTACCTCTTGTACAGTAAGTCCATCCAAACTATTAA
TAATGTGTTCTTCACTAATCTTGCTGTTTTGGTCACCATAAACATTAGGATCAAGCTTGCTTGATGGTGG
AAACTCCTACAATTAAATTAAAGCAAATTAATTAAGATAATTAATTTAATTTAAACTAAATTTGTAAAAG
AAACATAATTAATTATTATATTAATTACTTGGAGACGACGAATGATTATAGGATTAACTCCAGCCAACAT
TTCTCTAGCAAATTCTTCATCAGTCCTCCATCCAGACTTATTATCTGTTTAATTAAAACCATAATTAACA
TAATTGAGTTATTAGTCAAACTTTATATATATATTCCCTCTAATATTAATGTATATATATATTTTTAATTAG
TAATCCATACATACTCAAATAATTAGTCCCCTTCTAAAAAAATTATTTATATAATTTACAAGAACCGAAA
TGATATTTTAACAGATATAATTTTTCCAAACGAATTAAGATAGAGAAAAACTACTGTGAAATGTTAATTA
GATGGTAGAGATTAAAAACACCCTAATTAATTACATACCTTGGATGACTTGAGGGAGTGGAAATTTAAGA
AATTTTTCTCCATCAGTTCTAAAAATTTCCTTAAGCAATGGAGCAGGAATGCTTTGCCTAAACTTCTCGA
GCAAACTTTTGGGAAGAGAAAAGCCTCCTTCATAGAGTTTTAAAACATCTTGAAAAGAGTCAAACTCTCC
TGGATTTCTGCTAAATAATTCATCAAGGGCTGGTTTAATAGATTGAGCTACTGATTTTAATGTATATGCA
AGAAAATCTGATTCTTTCAAGTGCCCAAATCTTTCATCTCTTGGTACATAGATGTTTAAGCTCAACGAGG
ATGACAGTCGACTCTCGTAGTTTTTATCTGTTCATTATTCAAATCGCAAAAATTTAACATAATTAATATT
CATGCATGTTGTTTATTATAACTATATAACGCAATACTCTCGTGGTAAAAAGTCAACAAATTCAAATTAA
AATTGATCATTGACCTTTTTTTGAAGGCGATCTTCCTGTTCTTCCTCGACGAGGATAAGGGTATTGAGTT
GAACCTCCAAGAACAGGCCGAACATATGTTGAGTCACTGCTTGGATCTTCAAGATCGTTGTAAACATCAT

AATCATATATTCTATCCCATTCTTGACGTTCTCCTGTTCCATCTCCTCTCAAGTTCTTGAGCTCATCTTC
TCTATACTTTCGAAGTGGCTCAGGTGTTTCATTTGGAATATATGCCTGTAAAACAATGAACGGACCAAGA
AAACATGTAAGGTAGACTATTTTAATTTCTTCAATTTTTCTACAAACTTAACATAAATTTTGTAATCTAA
TATTTCAAGAATATTACTATCTATTTCTTTCAGATTTTACGATGTATATTTTAACTTTAGCTTTTCAATA
ATGTTTCAGAACTATCTTTACAAAGTAGAATTACTAAAACAAAAAATAGAGACCTAAAATGATGTGACAA
TGTCAATTTCTAATTAAAACTTATTACATCGTACCATTCTTATCCAACATCATTTTAACGAAATATTACT
AGCTAATGAAATCTTACTAGGTCCAAGGTTATTTTTTACGTAGGATAACGTTACGACTTTTAAAGGAATA
TGAGTATATATAATTTATTCAAAATTTAATGAAAACAAATAGAATAATTTAATTAGGATGGACTAAAATA
AAATATTTCAAAGATATGATCAATAAATATAATTAGATCAATAATTTAATTTTGATACAACTTGTGAATA
ATTAAAAGGTTCCTTATGGTTGAATTTTTGTACAATTTTAAATGAATGTGAGAGTCTCTATCACACTATA
TAATAAAACTATATAATCCCCATGACTTTAGATTTAGATTATATATTCTTAAAGAGCTCTTGTCCTTTTG
TAAGAAGAAAAAAAAATATTCCTTTATTTATTGCATAAATCGAGTTTATTCATAATTAAGTTAAATTACA
CTAAAACCTAAAAGTTTTTACCTTTATTTTTTTTTATATAAAATATAAATATTTTATCAAATATTCTATT
TTTTACAATTTCCCATAAATATAAATAAACCTCGAAGAAAGAAGGTTAATTAGACCATAGGCCAGACTAA
TAAAAGATATATTAGAAACAAGTATGCCAATTATAGGCAGTGATGATGGATAGTTATAAACTTCAATCAG
TGTCTATGTCTATCTTTCAATCGGTGATAGATCATGAAAGATTCAGATAAACATTTATCGATCAATGTTT
ATCATTAAAAATGAACATATACCTTATTGATAAAGAAAATACGATCGTTTTTGTACTTTCCGGAAGGGTA
AATCCAAGAGTTGCAATCAAAATGGATTCTTCCAACAAAAGGAACATCTTCAAGAGTGAGAGATTTGAGG
AAGAACTTTGTAAAATGATTATTTCTTATCAAGAAAGCTCCTGGAATTCCAATATCTTCATCCCAATCAA
AACTTATTTTGAACACAGTTTCTCCTGGAGACACAGGAACCATTTTGGTCATCCAATTTCCTAAGTATGT
CTTCTTTCCCACCTTTCCTTGCAATTCCTTTGCTGTATGTGTAAAATCAACATATACAAATTAATATACC
AAATATAAATCATTTATGTTTTCAGAAATAACCCCATTTCTTAAATATTTGAGGAAATTAAACAGCATCA
AAGAATCTCCCAAAAAAAAAAAAAAAAAGTCAACCAAAAACAACTAAAAATTTGGACTACATACAAAATAA
GTTAAAATTTTACGTAAATTTTGTTATATTTATAATTCTTTAAGAATATTATTATATATACACTTAATTA
TTAATCTTAAACAGTATTACTTACTCGATCCAATTATTTAAAATGTATTTATACCAGAACAATAGATGAT
AATTAAGAACATTGTTGTTTTTAAATATATATCCAAATGAACAAAAATTTACAAATATAAAAAAATATTG
CTATTTTGCAACAACTTTAGTCATTTTAAATAATTACCTTAAAGTATTTAAATAATCACTTAAAATGTTA
TATTTTTCTCAAATTAATTGTTAGCTAGTATCAAGAGAAAAAACTTTAATTTTCTTTTTAAATTATAGAA
AATCAATATGGTATAGTTTAAAAACAATAGTTTTTTTTTATTATGAAAATTAAGATGCAAGGAAGCTGAT
ACCAATTATATAATATAGAAATCTTAATTTTGAAAGGTAAAATTGAAAATTTACTAAAAGTAAAAATCTG
ATTTTACAAAAATGTAAAAGCAATTTGTAGGAACTGATTTAAAAAGTTATCCTAAAAGTGATTTTAGTTT
ATTTAATAAAAATTTATTTTACCAAACATAAAATACAATTAATACTACTACTTGTACTATCAACTCCAAA
AGTAGGTCAAAAGTTAAACCCTAATAATTAAAAAGAGCAAAAAAAAAAAACTTACATCTATCACCATTTT
CAGAGCTGATGAGCTGAAAGGAAACTTTTGCTCCCCAAAACTCACTGATATTGTCAAGAACAGAAGACTG
AATTTCATTATAATCCAAAGAATTACTTCTCAATAAAATCACTTGCCCCTTTAATCTTCTGCCCCTTTGCCT
CGACTCTTGATTTTTGATATGGTATGAAGCAACATCTTTCTTCTCTAATATAATTTATCTCTCTCTCTTA
CAATATAAGAAGGAAAAGTAGAGCTATGAGTATTAAAGAAACTAAGAAACCATTCTCAATTTATAACCCA
AACTAAAAGGTGTGAAAACAAAATGTAAGAGTGGGGTTTAAGCACTTTTTTAAAAGAGATTGTTTTCTTC
TAATAACATTTATAATATCAACGAAGTGGGTTGAATTAAAAAATAATTATGTTTTTGTTAATATTTTTCCA
TCAAGTTTATTTTAGAACAATACGTGGAAAAACACGTGTTGGTGTTAAAGTTTTGACAAAATATTAGAAGT
GTAGTTGTCTTACATCGAAAAAGTTTAAAATGATTATAGACTTCATTTAGTACTACAAAAGAAGTCTATG
CGCCCGGTTTCAAATCGTCCTCAATAAAGAATACTTAAGCTTAGGTGCGTATTATCTCGAGTAGTTAAAT
TTTCTTTTGTATTCTTTAGTTTGAGGGTGGGAAAAAGATATGTAGTTAAAAGTTTTGAGATAGTGATTTT
GTAATTGAGGTAAGGGAGAAATTTCTACTTGATTGTAATAATTTTTCCCATAGTGAATTTTTTTTTGTTC
ATAGTTTTATTATAGCAAATTATTTGGAGTTTTTTCACGTTAATTTTTTATGTTTCCAATTTTATTATAT
TATTTTAATTTCTCTCTATTTATTCCGCTGGTAGAAGTGGGAGGTTTAATTTTTCCAAAAAGAAAAGGTG
ACAAAATGTCTTATACAAATAGAAATAATTGTCCTCACTTATATACATACGTCATTGCCTAATTTAACGT
GAGACTTTGCTAGCATTCCATTGATAACACTAAAAATGTGTTATCTAATCCTTCTTATTTGGAATCATTC
CATGATTTTATGTTTTTAACTTGACTATTTTATTGATTTCGAGTAATAATTTGATGGTAGAAATTGATGT
TGATGCTATTTTGGAACCAAAATGAGCGAAAACAAAGTCTTGGTCTCTCTGCATTACAGCGTTGTGGTGC
TATAAGGCAAATAACAACATTGTAAGGCAATAGCCTAATTACTATCCGTGAAACACACACAACGGTGTCG
ACGTTACAAAGATGAAGCGTGCATGGATTTCCATTAGCGTCGCAACGCTGTCCCTAGTGTCACAACACCA
TTACAATTTCTTAAATAAGAAATTTGTCGCCTAGGTTTAATCATCTCTGAATTTCAGCAGCCATATCATG
ATTTCTCTCTAATTTTCTCCTATTTTCAGTTCTGTTTATGCACCTGAACATCTCCCTATTGCTCGACTTT
TTGAAAATGAGCTACGATGCTCTATTATAGCTTAAGTTGTTTAGGATTTTCCATATTTCAATGTATGTGC
TTTTTGAACTAGATTTTGTTTTGGTTGTTAATTGATCAACGAATTTCATCACCGAAATCTAGATCAAATA
ATAAATGAATTCACACTCCAAGAACTAGCTAGTCCTAGTAGTAAGTTCGAGTCGAACTTAGGGAGCACGT
GAGTTTCTCAAGTCGAATTGTTTCCAACTCGAGGTAATCGAGGTGGGGGATTGAGTTGATTTTGTAAAAT
TCATTATTAAAAAATATCTTGGCTTGGGTTGAAACGGATTTTTCCCATCCTAAGTATTTGTACGTTCATT
GTTTCATCATTACTCAAGTCTACCTAATATCTTCACCTAACCAATTTCAATTTAATTAGTGAAGCCAAAT

AAATTGTCCCGAAACTTAAATTATTCTAATTCAACGAGATGAAAAGCTTGCATCAATTGAATTTTCGTAT
ATTTTACTATATATATATAAATAGATTTATTGAAATCTCATCTCACTATTATCAAGTGTAGCAAAAAAAA
AAATTAAAAAGAGAAATATAGAGTTTAAAAGTTATTCTAAACACATTATTGAACTTTACAATTATTAATT
CTAATTTAATCATCAGCTCTATAGTGAGATCACATTTCATTCGTCATCAAATTATTAAACAATTGTGTCA
TAAAAACACACTTATCTTTTCCATTAGGCCATCTAAATTAATATATATATATATATAGCCCCAACAATTT
CCCTATATATATCAATAACAATTAATTTTATATATCAATTCCCCTATATATATATCAAAGAAAAAGAAAA
GAAAATATATTATATTTTTAATAGGGTCTACTCTAAATCTCTTGGTTTGTCTTTTTCTTTATGAAAAGAT
AATGTAAAAGGATAGATTAAAGTTTCAATTTTAATCCATATTTCATCTAATAAGATAGACATAAGTACTC
TTTACAAATTATGACAAGTTGTAGAAAGAGAAAGTATTTAATTAAGAACTTCCACATAGGTTTTCTTTAT
TTATATATATTTAACCAACAATCTCAAGTATGTTGTTGGTTTTAAAACTTATGAAACATTGAAAAAGCAT
CAATTTTAATTTTTCACCAATTGTTTTATGCTAATTTAGATTTTTAATAACTCAATTTTACTACCTACCA
CATCCTTAATTACAAAATATCATTTTCAAATTGGTATCTATATATCAAAATTCCTTTAATTTTTTTCATC
TTCTCTTTTCACATCTTATATAAAGTGTGGATGAAAATTCTATACATAATAAAAAAATAGTAATAAATAA
TCCAACAAATTTATAAACATGTTTTACTTTATTATTCAATTTCACATTAGGCCAAATGTCTTCCTCTAGC
CTAGAGCTCATTAGTGTATGACTTTATTATAAATTTACTAATGCCTAATTTTCCACCATTGACAGCTGTA
TATATATATATTGCAATAGTTCTTTTGAATAATAAAAACATTTTGTTCAAATGTTTTTAGGGAGAAATTA
TTGTATAAAGAAGAAACAAAGAATGAAATCATCCACCTCCAATTCACATTTGAAAAGTGTCATCTCATAG
AGAGAGAATTAAAATAAAGGATTGACATTGACTTGGGAAAATTAGCAATTGAAAATATTATTGTTTATAA
AAATAAAATGGATTATGACAATCTTTCATGGATTATTATATTAAATAAATAATCATTACTTAGCAACATT
ATTTGTTAAGAAAATATTCTTTTTCATTCAAAACATGGTGGTTGAGAGAGTTAAAATATCCATTCTATAT
TAATTTTGTAATGTAAATTGATAAAGAGTTGGATTGTAACACTACGAAGCACAAATACGTCAAAGTGGAC
AGAGAATCGAAATTTTATACATATTTAATATAGATACACATTCAAATACGTGGCATATCAGTGTTAAATA
TGTGAAATGGAGTATCTGACTTACTTTTTTTTAATTTTAAACACTACAAGAAATTCCATTTTCAGTGGCG
CATAGAAAGCGTCACTGAAGATTGACAACGCGTCACTAATAGTTTTAGCGACGAAAAGGCCTTCGTCACT
AGGATTGTCGCAGATTCCGTGTCGGTTAAAGCTTTAGCGACGCAAGTGTTCGCGCCGCAAAAGATGAACT
TTTAGTGACGTTTGTGCGTTACTAAAGGCTTACTTTTAGTAACATGATTTGTGTCACTATTTATTTTCTA
TAGTGACGTTTTGAACATGTCACTAAAGTTGATGTTTTAGTGACAAATTGTACTGTTGCTGAAGGTTACT
TTGGGACCGTTACAAATAGCTTTTGCGACGTATTGAAGTGTGCCACTAAAAAGTCGTACAGTTTTTTTTA
TAAAAATCAATGCTTTTGCGACGTTATTGAAAGCGCCAGTAAAAGAATTTTATTACATAAAAAATAATAT
CTGATTGATTAATTGCAATTTTGATAAAGTTAGCAACACCATCAAAATATTCACTAGAAGCTCGATCTTT
TAAATTTATCCAACTCCGATCCATAATAAATACAACACTTAAAGTACCTAAGTTATAAATCCATGTGTTA
GAATTAAAGCCATATGTAAATTGCTTAATAATAAAGTATACAATCACACATTCGAAAGAACAACAATTAC
ACTTACAAAGAGAAAGAGCAATCAAAACAACAACTCCCAAAACCTAAAATCAAGATCTTCGTATCAAGTC
AATAAAAATTATATTGTTGTTGTTCTATTTATAAACTATAATATAATATACTATAAATATATTATACTAA
ACTCACAACTTCATTCCTAGAAGTAAAAACACAATAACAAAACAAATATTCAACGAGACATTACTTAAAT
CTAATTAACAAAACAAAAATATATTCAACATGACATTACTTAAATCTAAACATTAATCAAGACTATGCAA
ACACTACAAGGATAAAACTCAATCTTAAAACTAATTAAGAGAATAAATATTCAACAAATCAAAATCCAAA
TACACTGCCCTAAAAATTTTAAAAATGAAGAATCTTACCGGAATCTCACTACTTGGCCGTCGAAGTGATT
CGTGTAGTTGGCCATCGGGTGGTTTGTGCAGATTCGACGTCGTGGTTCGCTCGGCCTGGTTCGCTGCATC
GTCGACCGTCCGTTCGGCCTGGTTAGTTCGTCGTGGCTATCGCGTCTGTGGCCGTCGCGTTCGTTATGGT
CGTCGCGTTCGTCGTGGCCGTCGCGTTCGTCGTTGATTCGTTCGGCCTGTGCAGATTCGTGGTCGTCGTG
CGGGTGTAGAAGCGCCGTCATCATCGTGGAGATACGTTGTCGTGCAGATTCGTCGTCGGTCGCTTAGTTC
GTGTGTAGTTCGACCGTGGAGGAGAGGAGTGGAGATGGAAGCTTTTCGGGTTGAGAATAGGGTAGGGGGA
AGGGTTTTTTTAATTAAAATTATTTAATGAAAAAAAAGAGGAGGGAGATGAAGTGGTGAAATTTTTTAGA
CTTTTAGCAACATCTATTTTTGTTGCTAAAGGTATCACACTTTTAGTGACATTATTATCGTGTTGCTAAT
TCTTTTAGTGATGCAAATTTAAAATATGTCGCTAATAATAATCTTTTGTGACATATTTTTTTTGCGTCGC
TATAAATGACTTTTACCGACAAAAATTTGTTACGTGACTAAAAATTTGTCACTAAATAGCAATTTTCTTG
TAGTGAAATACGCGGGGATATGTCAATTAATCTTTTATATATATGTTTGGATTAGTTTTAATCCTAAACT
CTTAGCTCACTTAATTTCATAAAATTAATATTATTATTACTTGCGAACCAAATGAAAAATAAATATTTAA
AACATTAAAAAAAATAAAAAAGAGATATATCCTAAAATCATTTTTTTTCCTTCATCTGTTGTGTATCCCT
CTAAGGTTAATCATCAAATTCTTCCATAAACTCAACCTCTTTCGTTCTTGAAGGATTAAATTAAAGACGA
TATCTTATACATTTGTAAATTGCAATCTTTATTTTATATATATATATATATATATATATATATATATATA
TATATAAGAACTACGTATTCTTAGACATACCTATATCTTAGTTTTTCATAAATTAGCAAATCGTTGTTTC
TGATTGTATCTGATAATATCCGTATGACATATTTATACTCGTGTTTGTGCTTCATGGGTGATAAATATAA
CAAATAGATTCAAAGTATTAACTAACATATTACAATAATGTCAAAAGAATATATAGATATATACCATATC
TCAAAGCTTATTAGTAATATATGATCATATCTCACTTGAAGTCAACAATAGAGTCTATCGATAAAAGAGT
ATCATTAATTATTTTATTATATTTATAAGTTTTTAAATATTTTTATATACTTAATTATTATTTTTAAAAA
TGCAATTCATATTACAATAACACTTTTTGTGTAATCGTATAATCATTGTACACATAATATCAAAACAACT
AATGCAATAATACAATATATATTACTTTGGCCATGTACTTCAATATCTATTCAATTTTAATCTTTTAACT
TTTAAATATCATTTCTTTTATCTTTAATCAATTTTAAATTTAGTCTTTCAAAATTAATTTTACTTTTGAT

GGTTTGAAACTAATAGTGTGAATTTGTAATATTTTACTTTTCCTTGGAATTACATGTGGCACCAACCCTT
CTAGAGGATAATATATAATAGTTTTGTTTACTTTACCTTTTCCAACTTTCATCACACAATAACAATAATT
AATATAATCATTTACCAAAACAAAAGCATATATTTATAGATGGTTTCTTCTTTAGATTTATTTGTTGAGA
GATTTAGGGTTACAATGTCCCCATCAATTAGCAAAATATGAAAAGTGACACTTCATTTTGGATTTTTCTA
ATACATTAATTTATTATATGTGAATACAAGTCTATGCCACATGTGAATAATACAAAAAGGCAGATTGACT
TTTAGATTTTGGCTTTAAGTTACAATAATGTTTTGGAAAATGTGAATTGGATAAAAAATATTATGCTAGT
AGTTTAATCAATTTTATTTTAATTTGGATATATAATAGGGTTAGTTGCATAGATGGTAAAAAAAAAAAAA
AAAGCCTAAAATAATAACATATATAATATAAGATAAAATAATCGTATATATAGCATAAAAATTGGAAAAG
ACTAATGTAAAGTGATATTATGATTGTTCTTTTTTAGCATAAATGACAAAGAAGAGAGATCAGATCAGCA
TATCTTTAAATCACTAATGATATATTATTAATATGATAGCTGCCATCGCTAATGGTACAAATCAAATTAA
TATAGTTGCAACTTTTGTTGAAATAAACTATTGATTTCATGTTATTCTATGAACATTTATATCATTTAGA
AATTCAAAGTTCAATAGTTATTGGTGATAAATTCATGAAAAACATATTGGCATATTAGACACGATGTCAG
TAATAGAGGTCATCATTGATAGCATGAGAGGAAGGAAACTGAAAAAAAAAATTGTTATTTTAAAAACTTT
ACCATATGACCGGTAAAAATGATCATTTTTGCAAATATTGTTCTCTTTTGCTATAGATTTTAAGGGTCTT
TTAAAAAATATAACAAAGCGCGGCAAAGTATTTACACTGTGTAGAACAATTCTGAAAACAGAAAAAACTC
ACAGGTCCACCATGGAAAATACCAAAAATACCTCATTAACCACGCTGTCAACAACGTGTCCGTAATATAT
TAGGTACACGATCGTTTAGATTTTACTATTATTTGGTACATGATCGTTTAGACTTGGTTGTTTAGAGATG
ACTACAATTTATTTTTTCAATTCTAGCTATCGTTTAATTTTGTTACACGATCGTTTAATTTGGTTACGTT
TAAATTTGGTTACACGAATGTTTAGATTTGGATCCAAATCTAAACGATTTCTTTTTTCAAAATTTGGTAC
ACGATCGTTTAGATTTTGCTAAACATTTTTTTTTGCTACACGATCGTTTATCTTTTTTTACACTATTGTT
TAGATTTGTTTACACGATTGTTTCGATTTGGCTAAACGATTTTTTTTTTTTAATTCTTTTGGCACACGAT
CATTTAGATTTGGCTATATGATAATTTTTTTTACACGATCGTTTACAATTGGCCACTCCAATCTAAATGA
TTTTTTTCAAGATTCTTTACATACTATTTTTTTAGATTTTGCTATTTTTTGTACATGGTCGTTTAGATTT
GGTTACCCAATTTTAAACAATGTAAAAAAGAAGAGGAAAAGAAGAAAGACGATGGAAAGAAATCGCAACT
AAAAAAAAAGAAAAGGAAAATAATAAAAACGATGAAAAGAAATTGCAGCGAAAATAAAAAGAAGAAAAAC
AATTGAAATAAAGAAATCACATAGAGGAGAAGAACGATAAAAATGTTCACAGCAAGGAAGAGAAGAAAGA
CCGAAGGACAAAGTTGAAATATTTAAAAAATGACTAACTTCGTGGACTTTGTTACACGGACCGTAAATAG
TTTGGTGTTTTGTTATATGAAAATTACCTTATATTTTATTTATTTTCTAATTTGTTCTTTGTAACGCCCA
ACAATTCGATATTTCTTTTTAGTTTTGTCCATTGTCATTGATATTAAGTGTGGTTATGGAAATTTTAAAT
TTATTGGAAGAACCTTACCATTTTGATTTTCTTGAGTAATTAAGGTTGGGAATCCTTATTTTGCTTAAGA
TAATAAGTATTATTTTTTTTTTTTACCTTTATTTTTGTTGGAAATAATTGGGGGAAGCCCTTATTAAACTA
AAGTTGGGTTTCTTTTTTTATTATTGAAGTTAGGGGTGAAATAATCTAATTAATGGATTAGCAAATTAAT
TATTTGCCTTGGAAAGGGTCAAGGGGTGGTTTAATTGAAGAGAGAGAAAAGAGGGTTTATGGATTTCTTT
TATTATTTTATTATTTTAAAATTCTTTTAAAAAGGAGGCATTGGGAGACGTGAGACTCCTCATCTCCCCA
AAAAAGAAAAACCCTAGCCGCCGCCACTCTCCGCGCCGCCGCCGCACGCCGCCGCCAGCTTCAAACACAA
ACCTCGGAGCCGGCCGTAGTAGAGCCGAACCACCCTCCCAGCCGCGCCGCATCTACAAACCGAAGCCGGA
ACTCGCCGAGACAGCCGAAGCCAATCAGCCGCGCGTCTGCAAGTCGAAACCGAGCCGTCGCCGAGCTTCC
AGCCGCGTCGGAAGAATCGATCAAGCCGATCCGCGCAGCCGAGCGCCGTGTCTGCAGCCGAACTCGCCGC
GCCGCGTCGATCTGAGGAAGAGCAGCCAGCCACGCCGCGCCGCGTAGATCCGACGCAGCGCAGCCCGAAC
GTTCGTCAGCCGTCGCGCCGTCGCATCGACTGAAGCCCAGCCGCCAACGCCGAGCGTGCCTCCAGCCGAG
AACCCGAACCCGGAGCCGCTCCACACCCGCGCGCCCGAACCCGAAGCCGAATCCGCGTGTGAGCCGCGCC
CGCGTGTGAAGCCGCACCGCGCGCCTCCGCGTAGCCGAGCCGCGTCTCCCTCTTGTTGCAAGCCGAGCCG
CACGCGTGAATTCCTGTTGCAAGCCGAGCCGCACGCGTAGATCCCTGTACCGAGCCGAGCCGCGCCTGCG
CCAAGCCGAGCCGGTCCCTGTCCTCTTCCAGCCGAGCCGCCAAGACTAATTTGGCTCCATCCACCTAAAT
TTTGGTAATTTAATTAATTAATGTGGCATTTCTCAGTAAGACTCCGTGCTTCGGACGCTAATTAAATTAA
TTTGGGACTAAATTAAGTTATTTTCCTTAAGGGACGTCTTGGACCAAGTAATTGCTGCAGCGCGGGATTT
CTTCAGTAGGGGCTCGAGCACTGCAACCTCTCTCTAGGGTAAGTTATTTCAAATGAGTCTTGAACCGTTA
GTCGTTGGCGACCTAATTACGAATTTTGGCATATTAAACAGTTAGGACCTCGTCGCTTGGGAAGCGTACT
GCCTCGCGGTTAGGACTCGACAAATAAATCTCCAGGTAAGAGATCCTACTACTAGCTTCATGTTTAGCAG
TATGAGACTATGTATGCTCCAATTTTTCATGTTAAGGATTAGACAGTACGATGCCTGAAATAAATGTTAG
TATGAGGCAAATGGCGATATGGTTATACTATGGCCTGTCATGTGTGGCGAGAGCTGCTATGACTATACGA
CGAATGTCGAGACGGAGAGTGTAGAGATGATTTATATGATATGTTATATGCTGATGCCATGTGTATGTAT
ACTGCGATGAGGGTACCTGTTAGCTTAATCTGTTAAAGTCGTACCTGCATGGGTGTCCTTCGGGATCACC
ACCTATTGAGGACTGTGTGGTCCGACGGGACACCGGTCTAGCATGGATATAGATATGACTCGAGTGACTC
GACGGGGTCCTCGCATCCCGACTGTCCTAGGTGTCCCCAGGGGCACCGAAGACCAGAGTTACGTTCCTAC
GGGAGCGCATGTTGCACGTGTTCGGGCAAGGGCAAGCTGGCGAGCGACCCGAAGTGAGACCGTGGAAGGC
CATAGGGACTACTGCTTCCGCTTATCTTTATTTCAGATTTTCGTATTTGAGTTTGAGTACTTTTCATTAC
TTATCATCTTTTTATGTAGATAGGGCCCGAGTAGGACTTTAGAACGCATTACACCTTTTTGCATAACTAC
CTTGTTTAAATTTTCATAAATAAAATTTCTTAAACCTTATGCGTTTTTAATAAATTTTTGACTTAAACCA
CTTGTTCTATATTTAGTAACGACTTCGATTCAGTATAAGGAGTTGGGTCGTTACAGTTCTTATAGCATTG

GGCTGCAATTCCCCTTTCTTTTTTTTAATGGGCTACATAATTTCCTCTTAATTCTCAGCCCATAGCCCAG
CCCATTGTAGAAGCCCACTTGCCACCTATCAAAAAAAAAAATTAAAAATTAAAAAACCCTAACCATCGGC
GGCCTTCTTCATCTTCTCATCTGCGATTCACGAACGGCCACCACCACCGTCCTCCTTCATCATTCGGCGA
TTCACGAACCACACCACCACCTAACCATTCGGCATTCAAGGTTTGTCTTTTCATTTCTGTTTTAATTTCC
ATAACTTATTTGTTTCAACTTCAATTTGTAAGTTAATATTAAATTTAAGTTAGATTTAGAAATATGTTCA
ACTAAATTAGCATCTTAACAATCAACACGAAGCTTTTTTTTTTCTTTTTAGTAGATTACATTTCTATCCT
AGTTTTTTAGAGATTAATGTATCACTGTCATCGTATCTCTATCTCACATCACACTTTTTAGTTTTCAAAT
CTAGAAACATTTTTTAAAATAAATATTTTCACCAAAACCTTGATTAAGGTCTATGGAGATCATGATTTTT
TTGGTTTTTCCATTAATGCTAACGGAAAATTGTACAGATACATGGGTTTGGATTCATTTCTCACCAAAAC
CTTTACATTTAGATTAGAATCAGGGAAGTCCCTGTTGACTATGTCAAAAGTCATCTCTATGATCAAGTTT
AATTATTCTCTTCTTTTCCTTCAATTTCCGTTCATCAATAGATTTGTTAGCAATGGGGAAGGTCTCTAGT
ATTTGCCTATCGTTATCAAGGTTGATTGAAATTCCATATTAGTGTAAAGGAGGAATGAGGATGGACTTGA
GTACTTTTAAATGCACTCTAGCTGCAATCTTACTTGCAAACTGAATAAGACTGATAAGTGATAACTGATA
GGTTTGTTGTCATTGTCGGATTTGGCATTTCTTTCTTCAATGCAAATTTAAGTTTCATTGAGGCTATCAT
AAAAGATCAATTTTCAAAAAAGTCTTGGAACTCCGTCGGGTTAGGTTATGTTTGATGATGCTCTATGAGT
TTTTTAAAACTTCAACTGTGTAGAAAACCTGTGTAGAAAACTTATTTTTCCTCTAACTGGTGACTGCTAG
GTGTCTTTTCTAGTCAGCTTAGGGCTACTTTTTGCTTAGTGTAAGATCCACTTCTTGAAAGGAAGTAAGA
ACCCCCTGTAGTGTTGAGCTGATTATTAACTTCTATAAGTTCAGTCTTGATTTCCACTGCCATGGAATTT
TTTTCTAAGTCCACTGTCATGGACATAAGGCCCCATTTTGAAAAAAAAAACAAAATGGTTATCAAATGTG
GCCCAAGTACTTTTGATTTTTGAAATGCTTCAATTTTGTTGTTACAGAATAATTAATAGTGTTTTCCATT
GAAAAAAGAAAAGAAAATAAATAGAACTAATAATGCTAGTGAGTATTGTATGATAGAACCCAAACAACTA
GAATATTGTCCAAATATTATTCTTACTCAACAGTCAAGCTTACAGCCACAAAACAATTGTCTATGTTCCT
CTCTTCCCACTCTCTTTATATATAATCAATTCTAATAACTTTTAGAACTCAAGGACACAATTCTACAACC
TATAGAACAACTCCTGTTCAAATAAGTTAGTTACATATTCAATTCAAATTGCATACTCTTTGGATTATTA
CTCTTATGGATTTAATAATTATTTATTCATTACTTGTTTGTTAAGCAATTTTTTTCTTCTTTAATTTTAT
TATATAAATTGTTACGTGCTTTCGCTAGGCATTGGTTGAAGCATTTGAATGGATATCTTTTTGACACTTT
CACGTTGATTTACAATATCCAAGTCTGTTTCTTGAATTTGAGTTGTTAACCATGAAGGATTTAAAAGATT
CAACCTTTTGAAAAAGTTGTTGGAGGTTTGTCACAAAGTTTTTCATAAGATATTAATAGTTGGCCATGGC
CTGTTATTAGAATATGCAGATCGCTTCTGGAACTAGATTATGTAATATAGTAGATCTATATTGGAAGTTG
TGCATTTGATCTTGTTGAACTATGTTTTCATGTGTTAAGTGTCTTGTGACGCATTTTCAGGATAAAGTAC
GTATACAACCATGCTTAATATTGCGACCACATTTTGCAAGCGACTGAATGTCAAGGATTTGGTGACCAAT
GTTCCTGTATATAGCAGTTTTAGTGGTAAGAATGGTTCATCTAGAACATGTTGTACAAACATGTTGTACT
TATCATGCTAATTTGTCTTCATAGTGATTACATTGTTTGTTTAATCTAATCTCAGATGGAGCTACTGCTG
GATTAAACTTATTATTCAGACGTTGGGCTACCAAAAAGACAGCTGGATCGACTAAAAATGGACGAGACTC
AAAACCCAAGAATCTTGGTGTAAAGAAATTTGGTGGGGAGGTGAGTGGCAGGACAAACAAAAATCACATT
GTCATAAACTTGCACTGAAATTTCTATTTATCGAAGCGGTTTTGTTGTGAACTTATTTACGGAGAGTTAT
TCCTTCTGGATACAGAGAGTTATTCCCGGTAACATTATTGTTCGTCAACGGGGCACTCGGTTTCATCCTG
GAGATTATGTGGGAATAGGGAAGGATCACACTCTTTTTGCCCTTAAAGAAGGCAATGTCAAGTTCGAAAA
GCACAAGCTGAGCGGACGTAAGTGGGTGCACGTTATACCAAAGGATGGGCACGTTCTGCATCCTGTTTAT
GCAACTACTTCTACTTCTCCACAATTGAAGACAACAGCATAATCTTTGGTGCTCTGTTGATGAACATGAT
CGTCAATGGTATAAATTTTAAGCAAAGTCGATTCCCTAGGGAGGAGGAGGGGGGGTTGTAGGCAGTCTCT
TTAAGAGACGTTTTTAATGTTTATCATTTCTTTTTCTGGAAAGCCTATTGGATTTCACTTTAACCAATAT
GGATACGATGGATGTCCGCTTCTAGCTCGATGTGCTTAAAAATGGGATTGATGAAGAGAGAAATTTCCCT
TGTAATTAAGTTGCCTCTTTTAAGTAAGTCAAGATGTGCGAAGAATTATAGGTATACTCTCGAGGGAATT
TTAGGTCCTCTTTTAACCTCCTCCCCTTACAAGGAATATTTTCTTTCTTGACATTTCAAATAGTTGGTTT
AATAATATCCAATGTCCATCATAACATTAGTCTTTACAAATCAATCTTCTGATAGCAACCAAATCTTTGT
ATGTACATATATTTATTGTTCTTCTCTCTTGAATAACACGTAATAATATTGGTGTATGGCAATATTAAAG
AAGCAATTAAACCATGGTTTATTGAAACACCAATCATTCCATAAGATTATTCATCAATAAAAATACACAT
AAAACAAAAAATCATAACCCATCTAACCCATTCAAACCATACATACCTTGCTTAGAAAGCACTTGGTTTT
GGGACATCTTAGATTGTACTTGTTTTTGTACAACTCACTCAATTGTCTAACGACAATTTCTCAAGGTTGA
AGTGAGCTGCAAAAAGATGCATATACTAAGATGTATCAAGAAGTATTATTCTACAACTTATAATGTTTGT
CACTCGGATAAATTGAAATAATGAAACCTTACTGAAATAATGAAACCTAAGGTTTCATTTAAATGGAAAT
GCTATTAGGAATTCCTCTGCCTGTGAGGCCTTCAGAACTTGTAGGAAACAACAAAGTATATGGCATATCC
ACAGGCCCAACTCTATTTTTCAATTGAGGATCTTTATTTCTCATAGCAATTTTCCTTTCAATTTCACCCA
ACTTTTCCCCAAACTTCTCAAATGCTTCCAAAGCTTCCTTATCCAAAGTCCACTCGGGGTTACTTCTTTG
CCCTAAATAAACTTCATCTGAAGAATGTCTCGACAAAATCTCAATAACTGACACCCCCACAAGGACTTGC
AATTGTGAAGTGATTGTTCTCAAGAACGCCTTCTCAGGGTTTGACTCGAGTTCTTTGTAGTCAGAAGTAC
CACTTTCAGGTAGGAATCGACGACTGGTCGATGGACGGTTGGGAGCAAAGCCACCATAAGGATATTGTCC
AAAGTTAACTGCAGCATGAAGAGCTGAAGAGATCCATATAATGATTGTACATGTTTCGACTAAGTCTTGA
AGGGAGTCCATTCTCGGCCACCATGGTTCATCTTTCTTGTCTGCATGGCCTTTCTCACGAAGCTCTTTCC

ACCATGATTGGAGCTCGGTGTCATTATGTAATGTTTGATCATCTTTGTAGTAGAAAGAACAATAATCTTG
TACCCATGTTTTGATTGCTGACCAAATCTCAAGACCATCAACAGCATATGGATAATCTTCTATTAGCAGT
TGAAGTCCATGGGGAGAGTTTGAATCCTCTATTGCAACTCCTCTTTAACAAAATTAATCCAAGATTTGAA
TGTCAATAATGAATGAAGTGAGAATTGAACAGAGATTATAGATTGATTTTTATAATCTCTAACTAATTAT
TGATTTTTTAAAAAAAGTGATTACCTTTTGATTAGATCAGCAGGGAGAGCTTGTTGAGGGAAGACCCAAT
TAGTTTTATAAACAAAAGAAGACATCTCCATGGAATACTTAGATGGATAATGTGTTGTTTCAATAATCCC
ATCAGCATTAATTAGAGACTGTCTTGCTAAAGCATTAATGTTCATCGTATCTCTAAAATGAGGAAGAAGC
AGTTTGTGAATTGGATGAACAACACTCAATTGTCTGTTTGTTGCTATCACAAATGGTTCAATTGTTGCAT
GAGTATTCAACCTAATACAAATCATTATTCAACTTTGTTAATTAGAGATATGTAAATGTGTTTGTGTGTG
AGTTTATTAAAAAAATTAGTTGATCAGTAAGAAATGAATTTTACCAATGGCTAATGAGTTGATGGTAGCC
AGTATCATTAACAGTAACATAAGCTTTAGCAAGTTGCCAAATTGAGGCATCAATTCCTGTGTTAGAAGGC
AAAATCACTTTGCTAATCACTCCAAATTCATCTCCTTGGGGATGTGGTAAGCTTAATTCAATTGCCAAAG
GCTTTAAAGTCCCATCATTTTTCAAGAACAAAACTGTTCTTGTAGCATAAGTTTTTGTTGGAGTTGTGTT
TATTCTTCTGAGATATGGTATCAATGAATCATGGTGATTCAATATGTATAGCTTCTTCTTCTCAAGTGCC
TATCAAAAAAATAAAATAAAAATAAACAAAACTTGTTATTAAGAAAATTAATTAATTAAGCTCTAATTTT
TCTTAGTTATTTACCTCATCCACAGTGAGTCCATCTAAGCCATCTTGTATGTGTTCTTCAGTTATTTTGC
TTGTTTGATCACCATAAATGTTAGGGTCCAATGTGCTTTTAGGTGGAAATTCCTGTATATTGTATTTGTT
TAAGGACGTTATTTTAAATATAGCAAAATGAATTAAAATATTTATAAAATATTTTAGTTTATCGATTTAT
AGAGTGTGATAAACTACTATTTATATCTATCTATTTGATCATAATATTTTGTAAATATTTTTATTAACTT
TTGTCCATTTAAAAACAATTTCCTTTTAGTAAATCATATATTTTTATGAAATCGATTAAATAATATTGAT
AATAATTTCAGGAAAGAAAATACAAATATATGAATTTCTTTTCAATTTTTTTAGTGTAAAAGTTGATAGA
TTAAAAAGATTTGATTGTTTCAAATATAAAGAAAAAATGAGTCAACTTATTTATAAATATAACAAAATTT
CACTTACATTAACAAATATATATCAATGTTTAAAATAATGAGTCAACTTATTTATAAATATAACTAAATT
TCACTAACACTAATAAATATATATATCAATGTTTATCGTTGATAAACAATAACATTTTGCTATATTTTAA
CTTAAAAATATTTTAAGCAGTGTTTTTGTTATTTAAAATAATTACCCTAGTTTAAAGGTTAATTAATTTA
GAAAATCATCAACCATAAATTAGTTCTGGGTAGGAACTAAATTTAAGGTTATAATCATCGAAAGTACGTA
GATAGACTAAAATTATATATTAAAAGAAAAATTACTTGAAGACGAGCAATGACTACTGGGTTAACTCCAG
CTAACATTTCTCTTGCAAATTCTTCATCAGTACTCCATGCAGTCTTGTCCTCTGTATATTATTCAAACTC
AAAAAATTCAGCACATAAAAGAAAATAATTAAGAACACAAACAAGAAACAAGAAAAGAAACTAATCTCTA
TTTTGTCGTAAGAATTAAAATCAACGGAAAAAAAAGAATGTACCATGAATAAGTTGAGGAGTAGGGAATC
TAAGAAATCTTTCACCATCTGTTCTAAAGATTTCTTTAAGCAAAGGAGCCGGAATATTTTCAGTGATACC
TTGAAGCAAAGTTCTTGGGACAGGCAAACCTTTCTCATAAAGATCAAACACATCATTAAAGTCATCAAAT
TCCCCTGGTGTGCTATCAAACAGATCTTCCAACTTTGGTTTTATTGATCTTGATATTGATTTTAAACCAT
ATGCAAGAAAATCTGACATCTTCAAATGACCAAATCTTTCATCTCTTGGAACATACACATCTGTTGCTTT
ACTTGAACTTGCTAATCTACTTTCACTTTTCGGATCTGCCACGATAATTAATGTTTGTTCAAACCACAGT
CAACAAATGATGATCTCAATCCTTTCTACTCTTAATGTCTATATATAACTCAACTAGTTGAGATATATAT
GTTATATCATTCGTAACATAAATTAAAAGTTTGAAATTTTTATATTGCTACATATTTAGGTCGAAAATAT
AGACATTTATCCTACCCTCTTGATAAAGAGCATATTTTTTGGTTGTACCTTCGCTTGCATGTGGCCGCAT
TTAAGTTATGTTTACATCTGTAATGTTTGAGTTCTCTACGTCTATGTTTGGTTTTTGAAAATATTTTCCA
AAATATATAGGCAAAATGTTGCCATCTACATCACAGTGATAGTTTACTATGTTTATAAATATTTTGATCC
ATTTTACTAATTAGTATTAATTAAGTCGTGGTTTTTTTAAAAAATAAATAAAAATGAAATTTACCTTTTT
TGGAAGGTGGTCTTCCGGTTCTTCCTCTACGAGGGTAAGGATATTTTGATGAACCTCCAAGTACAGGACG
ACTGAGGTCCAAATTGCTATCTGGATCGCCGATATCATTGTATACATCGTAATCATATATTCTATCCCAT
TCCTTTCTCTCTCCTTTTCCATTTCCCCTAAGGGTCAGTAATTCATCTGCCCTATACTTCACAAGTGGCT
TTGGCATTTCATTGGGAAGGTATGCCTATGTATTAAAAAGCTTTATTAAGGTTTATCCGAACATAGCTTA
ACTAATTAAAACACACATATATAAATCAAAAAGGATAAGATCACACATCAAAAAACAAACAAATAAATAA
ATAGACCGATTTTGAGATTTAATCCTACTTTTAAGCCCCTCATAAAATTACCTAAATGGATTTTCGAATC
TAAAAACAAATTTGATCTATGGTCAACTTATAAAAAGCATCATCTTGTAAATTATCTCGACCTCAAAATC
TTTAAAAGATACGTGACGGTGATGTACTTATCAACTTAGGTTTTTGTTTAAAAATATAAAAATATGAAAG
ATCGAATATTTAATTATTTTTCGTTAGTGTTTGAAGACTTTTTCTTTTTTTTTTTTTTAAGAAAGACTTC
AATTTTTTTCCTCCTTATTTTAAAGATTGATGAGTTAATCTTCCCCTGGCTAATATAGTTTTGAAAGGGA
CTTAATTACATGTTTATCTGTAGTTTAAGCAACTCTTGAGTCTCTTACTCTTTAATTATTTTTTTAATTA
AATAAAGTACTTCAAAACTAATTAAGCACTTAATTTTGCAAATTGCTTTCATCATCTTAATTAATTCCAT
GGTCAAAGGGCAAGGACAAAGTCCTATTTCCAAATAGTAATTATATATCCCAAATAATACAAACCCTAAT
TATTAGCTAGGGATTATTCTTAATTATTTCATATTAATCATACAAACCAATTGACTTAAACCATTTTATG
ATATTTAATCATCATGACAGCAACTTGATAGGTCACTAAAATATAAATTACTACATAGTTGATGAGCACA
CAACCAACTTTCTCTTCTCTTTTTAATTCTTAAATAAAAAATACTAAAAAACTATATACATATATATATA
TATATATAGAATTTCAAAACAATACCTTATATATGTTCTTCTTTTTATCAAAATACAAAAAAATTTACAA
GATTAAACATGCCCAACTTTACGTCGCCTTAACATATAACATCTTACTCGACTTGGAAATAATGAGTTAT
GATGATGCAACGCCTATAGCTAGACAACATAATTTCTATAACGTTTTGATTAATATTACGATGAAATTTA

CCTGATTGGCGAAGAAAATACGAGGGGTTTGGTATTTATCGGCAGGATAAACCCAAGAATTGCAATCAAA
ATGGATTCTTCCATGGCCAGGAACATCGTCAAGAGTGAGAGATTTGAGAAAGAATTCACTGAAGTGTTGA
TTAGTTATGAAGAAAGCACCTGGAATTCCTATTTCTTCATCCCAATCAAATGTAATACTAAAGGCTGTTT
CTCCAGCAAATATTGGAAGTATTGTATTCCCAATCCAATTCTCTAAATATGCTGCTTCTCCCACTTTCCC
TTGAAATTCTTTTGCTGTTTTTTTTTTTTTACATACCAAATCAAAAAAAACAAAACAAATTGGTTCTCTCA
AAGGCTTTGCATAGTTTTTGTTAAGAAACCAACCTAATTATAAATTTTGAAAACTAAAGGAATTTGTTTT
GTTTTTAAATTCACTAAAAATTCAATCATTGTATTTAAGAAAAATGCAAACCACTATAAAATCAAAGAAA
ACATACTTAATTTTCAAAAAACAAAAATGAAATGGTTACCAAATAAGGTCTCAGATAAAGAAAAAAATAG
AGATTTTTTAAAAATATGAAAACAAAAAAGCAAAATAAGTACATGAAAAAGAAATATAGGGTAACTTATC
ATAAAAGAAGATGGTGTACGTTAAAGATATGTCAACCCTAGCTAGTTGAGACATATGATCTGAGTGTGTC
TTTCAATCTTTCATCCTTAATCTGTGATTCATTAGAAAAACAAAAATAGACACTTCTCCAAGAGTTTTCT
CAAAACAAATATTGCAAACCTTGCATGCATAATTCAACATGCATTGGGATCCTTCATCAGTAGTTTTGCA
GTATCTATGTATGTATATATATATATACACAACTTGTTAACTTTTTGTTGTATTTACGAGTGTCCGAATT
AACTTAGTTTCATTCGAAAGATTTTATTGATCACCAGATCTACTACGCATAAATAGTTTTATTTAAAAGT
TCGTCAAAAATCAAAACGAAGAAACATCAAAGACCGAAAAAGTAAGAACTCACAAGGGTCACCATGTTGA
GCGCTAACGAGTTGAAGAGAAACGCCACCACCTAAAAGCTCATGAAGATTATCAAGAACAGTGGAACCGA
AATCATTGAAGTCTAAGACATTGTCTCTCATTAAGACCACAGTTCCATGGATTTTCTTCCCTCCATGTTT
CAATCCAGAGATGGCTCCAACAATGCTTCCAATCAACATGTTGTTATTATCTTTAAGAAGAGAGGATTTT
TTCTTTCAGAACATGTTCTAAAACCCAACAAAGGCTTTGGGATTTTATACCCAAAAATATGGCTGTTTTT
TTCTTCTCATCATTATCGTCATTTTTCTTAATTATTAAAAGCTTTTTCAATGAAGTAGGTTTCGTGTTTC
TCTCTCTCAAATGTCTCATAAATTTAGATATACATACTTAGCTTATCTATAAAAAAATTTCTTTTCATGC
TTGTGTAAAAAGAAAAACTATTTACAAAACATAACATATAGTAAAATCTATCCCATTTTCTATTATTTAT
TTAATTTTTTTTTTGTTATTCATAAAAATATCTTTTATATATATATAACTTAAACCTTATTTAGTAACCA
TTTGGTTTTTCAAAATTAAGTTTGTAAACCTTCCTTTTCTTGAAACGTTGAAAATCCCACGTTGGAAAAA
TTACGAGGATCAACTCACACTTCTTATAAGATTATATGAACTACTTCTCTAATTGTCAATTGATTTTGAA
ATCGATGGAACCTCATTCTATCAAATATCCTTTTATCCCTAATTTTTTTGGTATTGTTATTCGTATTCTA
CCACTTTGTTATAAAAAATTAAGTCAAATTTTGAAAATAAAAGGAAGAGTAAATTTTAAGAAGGAAATTT
GTTTTAAATGAAAAAACTATTTAAAATATTTATAAATATAACAAAATTTTATTATCTAACAATTATAGAC
TCGGACATTTATCAACGTCTATCATTTTACTACATTTGAAAATACTCCTTTAGAAATTATTTTTGTTTTC
AAAACTTGGCTAGTTGAATATTCTACTTACGTGTGTACCGAAGAAAAAACTAATGAGTTTACAAGAAAAT
AAAATGAAACACGTTTAGTTTGTTTTTTTTTTCTTTTAAAAAATGTTTACAGCTATATATACAATGTTGA
AATTTTAAATTTTGATTACATATATATTTAAAGTTTTAAAATAAAGTATTTTATGTGTTAGTTAAATAAC
CACATATATATATATATAGAACTTATTAAATTAACCAATCATATATTTCATTAAATAATAATATTGTTGG
GTGGTGACTGGTGAAGGATATATTATTTGACTATTTTCTGTTCAGAGTACATTTAAGTTGGTTGGGTGAC
GATCCCAAAAATAACAAAATTTATGATAATAGGATTTATGTTAATGTATTTTCTAAATTGTAAAAGTAGT
AAATTTAAGAGTGAATAATCTTTTGATAGTCGTATGATATCATTAATTACTTGTTAGATTTATCATATTC
GCAATATACAAAAAAGATGTGTTATGAACTGTTTTTTTCTAAATTTTTTTTGTCATTCGATACAATTTTC
CTTTTTTTTATTGCTCACACGTAATAATTTTAGAATAAATTACACAAACCACACCTAACGTATGGTACAG
GTGGTAATTACAATTACACTATTTAACTTTCAGTAATTGGGACTCTAAACCTTTAAAAATATTAAAATTG
AACCTACAAATGATAAAAATCGAATCATCAAACTTTTATATATATGTGTATATTAATTAGTACTATTTAT
ACAGTAATATAAGTTTGGAAGTCCAATTTTAAATTTTGTATTAAGTTTGAGGGTTTAATTTTTAATTTTG
AAAGTTTAAAAATATAATATGAATTCTAACTGCAGAGATTATTTTTACAAGTTTTCCAAAATTTTGAAAA
GGTTTAATCAAAATTTGGAAGTGACCAACGGTAATTGACATGGTTTTCTTTATTACACAACCATCAAACT
AAAAAAAAAAAAGACAATAACAAAGGCAAGAACAAATGTTTGTTAATATGGCAAACAATGATTAAATAAT
ATAAAAAATTTCAAAAAAAAATTATTTACACTTATGTAGAAAAAATATTACTAAAAATTAAAATTCATAT
TTTATCAAACTTTTTGTTTTTTCTCTATTCATCATGTTGGATATATAAAACTAATGGAAATAGAGGGAAA
TATTTGGGTCAAAATCTTTAATTTGTTGAGATGTCTAAGAACTAATGTGTTCCCATACTTTTCACATCAC
ATCCATGTATGTATAATGTTCTAATTATGAGAAAAATAAATGGTTTTTCCCCCACACGTTTTCTTTATGA
AAAGAGTAATAATAATAATAATAATAATAATAAAGTTATAGGTTAAAATATTGTTGTTTTTATTATTATT
ATTATTATTATTATTTGGACTTAATTCATTTCGGTTATAATTCTGGAAAATTTAAATAGTATCAATATTT
TGCTATTTGTAAAAATATCTTTTAAATCTTTTCAAAATAAATACCTTTGAAATTTTTCGATTGTTTCAAA
AAATACTATTATTTTTGTAAAAGTTGTAATATTATTTAGAGAAAATTACATTCTTAGTTTCCAAGTTTTA
AAGAACGTGGGTCTCTAAGTTTAAACGATGTAACTTTTTTTAGTTTTTATGTTTATAAAAATAAACCTAC
TTTAGTTTTTGAGTTTTCAAAAGATACCTTTTTAGTCTTCAAGTTTATAAAAATATATTTAAAAAATCAG
TCGAATAACTTCATACTTTTATTTTTGAACAATTATTTAACATTTTAAATTACAAAAATATTTTCTAGAA
ATCAAATTTTCTTTAAAACTATTTTTTTAATTTTAAATATCATATAATTACTTTTTAAAGAAGTTTTTTT
TTTTTTCTTTCTTAAATCAAGGGAACTGTGCTTATATTTATTTTTAATAATTTACTCATTAATTCTTTTC
AATTAATTGAAATAATGTGTTATGTAAATGATTTCTGTACTTCTTAAAATTCACTAAAGAAAATCATCTT
TTTTTTTCATGAAAAAGGTGTATCAATTTTCGGATGAAACAAAAAACAAAGTCGATAGATGCTTAAAAGT
GAATTGTACACAATATCATACTTTATTACAAAGAGATAAAGGGGTATCTTAAAACTCATGTTAAAATAAT

CCTCAACGTGTCAAATAAGATGACTAAGTATCCGTCCTCGAGAGACAAATCTACAACTTCAATATTATTA
AATGGTTGAACTGATGGGAAGAAGAAAAAAAAGGACTGAATTAAATTTTGTTCAATCAATACAAGATAACT
AGAAGAACAATTTTATAATGTGTGGTGTGTTAATTATTGTTAGATAAGATCGAAAGTTTTAAAGAAGTTG
ACCTAAAAATTATTGAGTTTAAATGTTGTGCCAAATTACAATAAATAAAATGATGATGTTGACAAATATT
TTTTTTAAAAAAGTTTCACATGTGAATTTAAATTATTAATAAACAAAGATTTTATTACTTTCAAATAATT
GAATAACTAATATTAGATTAGTACTTTTATTATATTATATGTTAACTTTTGCAAATATTTCTTTTAGTTT
GTCGTTTGCTATTATAATATATATCTCAAGAACAATGTGATATATTAAATATGATTATTATTATTTTTAA
AAGTTGAATATTGTTTATTTGTTTAAGAGTATATTCAATGAGTTTGATGCTGCAGTTAAGTTTATAATTT
AAAATTGCACGAGTATAATGTGTGTATTTATTGAAAAAAAAATAAAAGATATATTAAATTCATCTCGTGT
CTTCACAAATATATATATGGAAACGAATTGAAGTTTATTGGTGTTTGTTAGTGATAGAAATTGATTTAAG
TTTATTATTAATAGAAGTCGATAAAAGCCTATCATTATTTGTTAGTTGTTTTAATTTTAGATAGTTTGAC
ATTTGTTTTATCTGATTAGAGTTTTCACAACTTTGAAAACTAAACTCCAAACAAAGTGGTGTTTTTCTTT
TAGGAATAATATTTCTTTTTGCGCTAATTGAGTTCTTGTACTCTTTATTTTTTTTAATATTTATGGCTAG
ATGTAGGAGTAGTAATTGTCGAGGTTCGAAATATCGAAGTCATGATTTTATGGAATATATCAATATAAAA
ATGGATCTAACACAAGTATTCATCAAAGTTTAGAAACTAAAGAAGTTTTTTCTTTTTTTTCCTTTTTAAA
CAACAATAAATGAAAGTTGTAGTATTTATAAACTTAATTTTTCAAAAACAAAAAATAAGAAACAAAATAG
TTATCCCTTATCAAATACATTATGATAGGAATATAGATGGCTTTGCTTTTGAAAAAAACAATTATTAAAA
TAGTAACCCAAAGTCATGAAATATAGGATATGTGCTTAGAAGCTTAGCCTATGAGGTACTTTAACATAAA
ATAATGGTTAAAAGAGTTGTCCAAATTTGTCATAAAAACATGGTCAAATTTTGATGCAAGGTTGATTTTT
GATCTGAAAATCCTTTGATAAAGAAATAGTACTTAACAAAGCTAATTAAGAAATATTAAAACTCATGCAA
AGAATTTGAAATAGGATTGCACAATGTGATTTAACAAAATAATTATATATAAAAGAAAAAAAAAAGTACA
TACATTATCTTAACCCAATACGTCTATGTAAGTAATATAATTATGTTCCATAAGATAAATCATCAACACT
AATCAAATACATATATGTATATATATAGTAATAGAAATTTAATTATGTGTACCTTATTCAAAGAGGGGTT
ATTTTAAATGGAGACACTGTTAGGAATTCCTTTTCCTGTTAGCCCCTCTTCACTAGATGGATAGAGCAAA
GTGTAAGGCATGAAAACTGGTCCAACTCTATTTCTCAATGTCAAATCTTCATTTCTTTTCGTGATTCCAT
CTTCGATCTCGGCCAACTTTTTCCCGAACTTCTCGAACGCGTCCAACACTTCTTTGTCTGCAGTCCATTT
GGGGGAGTCTCTTTGGCCGAGATACACTTCATCAGATGAATGCCTTGACAATATCTCTATCGACGCGATG
CCAAGAAGAGTTTGCAGTTGTGCAGTGATTGTTCTTAAGAAGACTTTCTCCGGGTTTGTTTCGAGTTCTT
TATACTCTGGAGTGCCTTCCTCCGGCATGAACTTTCGACTTATAGTTGGTCGATTGGGAAGGTAACCTGC
GTAAGGGTATTGTCCGAAGTTTACTGCAGCATGAAGAGCTGAAGCAATCCATATGATGATAGCACATGTG
TCGATTAAGTCTTCAATATTCTGCATCTTTGGCCACCATGGTTCGTCTTTCTTGTCACCATGACCTTCCT
CTCTAAGTTCCTTCCACCAAGACTGAAGTTCAGGGTCATTCCGAACCGCTTCATCGGTCTTGTAATAGAA
AGAACAATAATCTGTAACCCATGTCTTGATTGCCGACCAAATCTCGAGTCCATCGACAGCGTACGGATAG
TCCTCAATTACGAGACGAAGTCCATGTGGAGAATTTGAATCCTCAATTGCCATCCCTCTAAACGAAACAA
TGATTTTAAACACGTTAAATGAATGGTTTGTAAAGTATCCGAACATGTTTTACTTAAAAATTACCCACAA
TCTAAGAATCAACCAGAATGATACATACAGTACGACACAGACATGATAAGACAGTTATTTTTTCAAATTT
TAGGACATGACACAACAAGGACACATTTATTAAAAAATACATTGTTTAAAAAATATATCATTTTTATATC
AAAAGAAAATTCAAGCAAATGGATTGATGTATTTTTATGCCTACAAAATTTAGTTGGATGTATTTCACAC
TCAAATTTTATTATTGTTGCCATATATGCATCTATTTACTCTACTTAACAAGTGTTTTATCTGTGTCTAA
AACATTTTTTCTACTAACAAGTGTATGATATATATCAACAAGTATTCGATATGGACACATTATAACCAAA
TTGAAGTGCTCATGCTTCTTAACTCTCAACAATCTCCTATGTTAATGTTCGAAACAAATTAATCTTTAAA
CAGACTAATGTCCAATACCAACTACAACTATGAATTTTGTACCTTTTGATGAGATCTGTAGGGGGTGCTT
GTTCATGAAACGCCCAGTCCTTATATAAAACAGATGACATCTCCATTGCATATTTGGATGGAAACACTGT
TGCTTCCAAAATGCCACCTGCATTAATGAGTATCTGTCTAGCAAACGCATTTATATTCATGGTATCTCGA
AAGTGAGGATGGAGCAGCTTGAAAACTGGATGAAGAACACTTAGTTGTCTGTTTGTTGCAATCACAAATG
GCTCAATTACAGCATGAGTATTCAACCTACGAGAAACCGAAGTTATATTCAACAATCCATATGATTTAGA
ACTAAACTCAAATTGTCGGTCCACATCACAGAAGAGTTATACCAATGGCTGATGAGTTGATGATAGCCAG
AATCATTTACGGCCACATAAGCTTTCGCGAGTTGCCAAATTGTACTACCAACCCCTTGTTCAGCTGGAAA
GAAAACCCTGCTAACCACTCCAAATTCGTCACCTTGAAGATTTGGCAAGCTCAGTTCGATTGCCAATGGC
TTCAGAGTACCATTTTCTTGAAGGAAAAGTATGGTTCGGCTGGCATAAGTCTTTGTGGAAGTTGTATTTA
CTCGTCTAAGGTATGGCATTAGTGAATCATGGTGATCCAATATGAATAACCTGTTCTTCTTGATCGCCTA
AACCACCAAATTACACAAAAATAATATCAGCTATTTATTTTCTAAGAATCAAAACTTTATTTTTTTTTTA
TTATTATGGACTCGATGATGATAATTCACTCACCTCTTCTACCGTAAGTCCATCCAAGTTATGTATTATC
TGTTCTTCAGTTATCTTACTCTTCTGATCACCATAAACCTTAGGGTCAAGCTTGCTTCTTGGTGGAAAGT
CCTGCAACTCAAAGTATGAATTTTCAGTTCAAGGAAACCAATCTCTCTCTTCCTGTATAATACATTCACC
CCATTTCAGATTTCCTTGATCCCAAATTTTCAAATAAAATAATGAACTCATCCTCACGTCCCACGTTTTA
TATCATTCTCAGAGGTATAATAAATTTGAGGGCAAATGTTTTTTTGTCCCGATGTTCTAGGTTTAGTTTT
CATTTAGTTTATAAGTTTCAAAGTTACCCGTTTAATTCTTAAGTTTTGGGTTTTGTTTCAATTCAAATAT
ATCCTTAATTTCAAAATGTAACAACTTTACCATTGTTTTAGTTTTATTTCAATTTGATCCTTAGGCTTCA
AGATTTGAATTTTTAAACTCGATTTTTCACTAACTACTCACTTTCCGTCCTTAATGTTAAGGTATCTGAT

TAATTTAAGAGAATTATCATCAATCATGTGTTATTTTTGGACTAAACTATAATCAAACTCAATATCAAGA
ACCAAGCAGAAACTAAACCCAAAACTCAAGACTACAAATGTATTTTTTTTTCCCTAAATTTGTAGATTGA
TGAGAGAGTACTTGGAGTCGACGAATGATGACAGGGTTCAATCCAGCCAGCATTTCTCTACCAAATTCTT
CATCAGTCCTCCAAGCAGACTTATCCTCTGTTACAAAAATCATCAAGTTCATCAGAAAATTTAACCCAGC
AAGAACTTACCAAACACTTCAAACTTCCAAATACCTTTAATCACTTGAGGCAAAGGGAATTTGAAGAGCC
TTTCACCATCAGTTCTAAAAATCTCCTTAAGAAAAGGAGCCGCAATGTTTTCTCTGATAACCTCAAACAA
ACCTTCCGGCACTGGAAATCCTCCTTCATACAGATCAAGAACATCCTGTAAACTATCGAATTCACCCGGA
ATCCCTTCAAAGTATTCTTCGAGACCAGGTTTAATAACCTGAGAAACTGCCTTCAACGCATAAGCAAGAA
AATCGGACAACTTCAAGTGACCAAACCTCTCATCTCTCGGAACATAAACATTTAAGCTCTTAACAAGTGG
TATTCTGCTCTCAGTTTCCGGGTCTGAAAATGAAATTGAACCCATGAAACTAATGTTTTCATTCACAATC
AACGAAAAATAGTGGAAGAGATTTTATATTTTCTTGCCTGACTTGGTGGGCGGTCGTCCGGTTCTTCCTC
TTCTTGGGTAAGGATAGGTGGTTGAGCCACCAAGGACAGGACGAGCGTATTGTGGAGCTTTATCGGGATC
AGCGAGATCGTTGTAGAGAGCATAATCATAAACTCTATCCCATTCTTGAAGTTCGCCATTGCCGTCTCCT
CTCAAGCTTTGCAGTTCGTCTTCTCTGAATTTGCGAAGTGGCTCTGGTGTTTCACTTGGAAGATAGCTCT
GTCAACATCCATTATAACCAATAACAAATTTTAAAGTCCAATCGACAACATTTTCCAAATGCTTAACCAA
CTTTTTACGGTACAAAGAAAAAGAAAAAGCGATCACCTGGTTGACAAAGAAGATACGATCTTTGTTGTAA
CGGTGAGACGGGTAAATCCAAGAATTGCAAGGGAAAAAGAGTGTTGTTCCATGAGGAGGAACATGTTGAA
GAGTGAGAGATTTGAGGTAGAATTGTGTATGATGATGATTTGTAATGAAGAAAGCACCGGGAATTCCCAT
ATGTTCTTCATCCCAATCAAATGTAACTTTAAACGTAGCTTCCCCAGTTGTTAATGGCGTTATTGTCGTT
ATCCATTCCTCCAAGTAGGCTTCTTCTCCCAATTTTCCTCTCAACTCATTTGCTATGAACAAAAATCATG
ATATACAAAAGTGCAATTAGTTTTAGTAATCGCTAGATTAAATTCTCAGTTGTCCATGTTGTCATAGACA
CGAGAGGTGAATAGACATTTTCATTATAAAGTAAAATAACAAGATAAATGTGTTTTTATAATTTTTTTTA
GAGATATCTAATTTTGTGAATATATTTTATCAATTCTTTTAACATTTCCATAAAATTTTGACATAGTCAT
TGTTTAGGTTGTTGTCAACATATTCAAATTTTGAACATGGAAGGAAAAAGATGTTTAGATTATCATTAAT
TTGGAAATAGGCTTTATTACAACGAAAAAAGTCATCGTAAACAAAATAGAGGACAATCAATTAAGGTAAG
AAACATCATGAACTTTCTCTTGAGAGAAAAGATGATATGATAGAAAAAATTTTCCAATAACACTCTTTAA
CGGTTACGATAATCAAAAGAAAAATCTCGTCAAAACACTTGACAAGTGCAACGGTAATTAACATCGACCT
TATTCTTTGATTAGGGTATGTTTAGAAAACTTTTTTTCTAAAAAGAATTTTTTTTTTCATTTATTCTCAC
AAATTATCGTTTTTTTTTTTTTTACTAAGAATGTTTGAAGTTTTGTTTTTCTCTTTATAATAAAATAACTA
TGTTATTTTCTTATTAAAAAAGTAACAAATATGAGAGTTTAGTTTTTAAAGAAAAATCTATAAATATGAG
AGACCTTAAGATGATAATTAACAATGACAATATTGAGTATAGGATAAGGTTGAGGGGGGAAATTTTGTTA
AATTAAATTCAACTTTTTTTAGAACTATTAAATTCAATTTAAAAAGGATAATTTTAGGGCCAAATTTTAG
AATTCCTAAAAAAAGAAAATTGTTTTTTTTTTTTTTTTAAACAAAAAGAGTTAATTTGACCAAATAAAAT
TAGCTTATAATATCAACACACATAAATAGGTTTGAGTCAAGAAATGGAAACAATGAAATATTTGAAAAAA
GAAATTAATATTGTTTTGGGTAATTCCAAGTATATATGATATTGAATTTCTTTTAGTGTCCAACATTTTT
CTATTTCCTTTTTCTCCCCTTTTTGTCTTGTTTTTTTTTTTATTTATTTATTTATTTTTCTTCTTTCTTG
CTCCCTAAGTATTTACAAAAAGAGAGAATTTCCAAAGCTTCTTTTGGAAAATTGAAATTTTCTCAAAATT
CTTGGTTTTGTTAGGCTAAAATTAAGGTGGAGTAGAGATCAGGCTTCCAAAACAAAAACAAAAATTGGGT
TTTAATAGAAATAGAAATAATAATAATCTCTAACTTTGAAGTTTAAAAAATAGCAAGTTTTATAATTTTT
ATGTACCAAAATGACAATTATAAACAAATTTCAAAGATTAAATTTGTAATTAATTATTTTAAATCCTGTT
TTTTACCTATTTGTTTGACCAAAAACTTAATCAAATTATATCAATACCCTTTATGCAAATCAACAACATG
ATTCGAATTGTTAAGTCATATTATTCTTAAAAAAAATTACAAAAATAAAGATAAGTCGACTTTTTTCAAG
AGTGATGACTTGAACTATGATTAAATTTTTTTAAACCCTAAACTTTTGCACCATATTTGTTTGTGTTCAT
ATTCTTAATTAAACTTTCCTCTATATATGTATATATATATATATATATATATATATATATATATATATAT
ATATATACATACACATTTGAGACTTGTGAGATAAATTTATTAATGGCAACAAAAAGCCAGCTGGGGTATA
ATTATAATAGTAAATATAATTTTAAAATTACAACATTAGCATTATACTATTTTGTCAAAAATAGGTTTCC
ATATATATTACAAATTTTAATGACTAAATTAAAATATAATTAGTTTAGTAATATAGAGATAGAAAAAGGG
ACTTGATGACACTTTTTTATGAGATAAACAACATAAACAATATTTTAAGCAATAAAAATATTTTAATTAT
TTGAAACGTAAAAAGATATGTAAAATTGTGAGATGGAAAAACGAGCCCTATATCTCCAAAAACGAAAGCG
TTGTACCAATAGTGATTAAGTCGGACTCACTTTATCACATAAAGCAAAGTATTATATGCTTGTTGATAAT
ATTATGCTTGTTGATAGAAAAGAAAAACTTATAAGCCAAGCTTTAATTTCACTAACCTCACATAAAGAAA
ACATTCCATGTCCACTAAAATATTCTTAGATTGAGAATGTTTTATTAAAAGGGTAAAAAGAAAATTGACT
CAAAAGAATGTGATATTTACTAGAAAATGAGAGAGACTTATGCCCTAAGAAAGAGAAAACAAAATAACTT
TTTTTAGGAAGAAAAGAAACAAGAAACTTGAGAATGTCACTTATAAGTCTAGAACATTGTGGTTGTACGT
ATGAAACGCTATGAAACTATTGATCACATTGTTTAGCTAGCTTTCTCTTGAGAAATTTTGTAGAAAGACC
AGACGAGAGAGAGAACGACCAAGTCGAGAAAGAAAAGAGGGAGGGAAGAGAGGAATTACTTACATGGATC
TCCATGGATTGCACTAACAAGTTGAAGAGAAACTCTTTTGCCTACAAACTCATAGAGAGTATCAAGCAGA
GAAGCATTAAAATCATCAAAGTCCAAAACATTGCTTTTCATCAAAACCACTTCCCCTAAAATCTTCTTCT
TCTCTTCTTTTAAAACCATTCTAACTCTCTCTCTATTTTTCACTTTTAGAAAACTCAAGAACTTATGAGG
TATACCAAAACACACACACCTTTGTTTCAAGGCTTTAAATATAATTTGGTTCCCAAGAAGAGAATATATT

CATATCTATTTATATCCACACACACACACACGACATGCAACTGATCAATAATTGACCCGATTACGGTTTA
CCCGACCCGACCCGACATATTATTATTGTTGTTGTTAATATTCACCAAACAACAAATTTAAATGTAGGGA
TTTTAAATGGTTAGGTTCAGGAAGAAGAACCCCTTAGGTTGAGTAAGTTTTTCAAAGGCTACAATATTAT
ATCCATTTATAGCTTCTTCTCTTTTTCCAACTTAAATGTTTCCAATAAAAGAAATATTATCGTTTTAGTT
TTAAGAGTTTTTTAATACTAACTTAATTAATTTATTTACTTTTTAAGAGTGTGAAAGCAACTTGAAAAAG
ATAAATAAAGATTTAATATTCAAATATATGTTTGGCATGGAAAATTAAAAAAAAAATGTTTTAAAAAAGA
AACATTGTTTGATAATACTATTTTTAATCCATACAATTAAACATTAGATTGAATAAAAATTATTAATTAT
TAGTTAACGAAATAGGTTTATGTTATTAAAAATTGTTTATGTACACGGGTCGAGGGATTTCTTGGACCAA
CCAAAAAAATTTGGGTCGATCAGCTTGACTATCTAAATAGTCCAAATAGGGTCTCCAACCCAATTATCTCT
TAAAATTATGTTTTGTTGTAGGGTTATTATTTTTTTCATTTTTGTTACGTTCTTTATTAACTTAGAATAC
TTGAATTTGTGCATACTTAATAACTAAACTTTCATGGAATTCAAACATTAATGTTAAATATTAAAATTCA
AGATAGGAGCGAGATCTATTACAAACATTATAAAAATATATGAGATGTATTATAAGATTTCGATTGAATT
GAATCCATTTGAATTTTTAAAAGTGTATCCCGGACCTAATTACCCAAGATTAATACTTGTTTTTAAATGC
ACTTTACCGTTTTGTTAAAAGAAAAAAAAATGCTCCTATACTTCAAATAAAAAACTATCCTCACTTGAGA
GCATTTATAAACATTTCAAAATTATCTTTGGAATAGAAAGATATGAAATGGTTCAAGTTGAAGGATGTTG
TTATTGTGAAATTTAAAACGTAGGAGAGTACTTTTTTTGGAAGTTGTAGGACAAAGTAGTAGAGAAGTAT
TTGTGGTAATTTAGCCCTAAAGTTCTATATTCATCACTCTTTCCTCCCTCTTTCCTACCTCTTTCCTTTT
GTGCAAATAGAGAAAAAAGAAAGAAAGAAAAGAATACTAAAATTTATAAGGAATAATTTGGTCCCCATTT
GATTTAATTAATTAAGAACAGAATTAACTTTTTGAAAGCAACCACCCAACCTTTCAAATTTGTTTTACAC
ATCATTATCTCTTTAACTAAATAAGACTCGATGTATGGTTGGTTACAATAATTGTATTTCTAAACTTTTA
GTCGTAAAAATTTAATACCTCAAACTTTAAAAGTTGTAAAAAATAAAGACGGTATGCATGTTAAAGAGTC
AAATCAATAGTGGGAATTTAGAAACCAAAACTAGAAACTTATTATACTTTACGTTTTTTGGCACGTTTGG
TACCTAATAATTTTCTTGATTTTTTTTCATTATATATATTCACATATATTTCATTTGATTTTGAAGTCGA
TAGTACAAATATTATATGTCTTAATCTTAAGTTATGCTCATTTTGGCTCGTAATATGTAATACTTTTTGT
CTTCATGTATGGACTATAGAGATCATATTTCTCTTAAATTTCATTTTATAGATTTCTTTTGATATATAAT
TAAAATGTAGCTAGTAAAGTTAATTTCAAAACTAAAATAAATATACTATCATTGTTAGAAATTTTGATAT
AATTAATAAATTTGTTATAATCATAATTTATTATTCTTTATTTTCCAAAAAAAAAAAAATAGTTTATATT
TTTGTAGGAAAAGAAAGTAAAAAAAGAAACAAAAGAAGTAGCAAATTAGAATCAAACATTGGTCACTAAT
TGTCACAAAGAAGAAAGGGGTTTAGGAAAAATCACTTTTCTTGAGCATTACTCCACATTTTGCCCACCCA
ATAACTGCATGCCCCCATGAAGTTATGTCTACTTTTATTTTGGATCTTTTCTTACCATAATCTGTGCTTC
ATTTTTATTTAATAACTAATCCATAAATACTCCAAAAAAGAAAAATCTAATATATATATATATATATATA
TATATATATATATATATATATATATAATACAACAATTCTAGGATAAAAATCGAACCTCAAAAAATCGAATCA
ATCATTACGTTGAGCTAAACTCATTTTGACAATTAATTTATATATGTATATACGGACGTCAAAGACTCGA
CCAAACGTCGAAGCTTCGGCAGATGTTGAGGACGAGGCAGCAGGTTTTCGAATGCGCTAGAAGACTCGAC
AAATTTAGAGAGGCGTGAAGACTTTGAGAAATTAGGCGTGAGATTTAGAAATTAGGGCGTGAGATTTAGA
AAAATTTTGGGATTTTCTATTTTAAAAGATTATTTTATATTCCTAAATCCTTTTATAATAGAAAATAAAT
GTCACGTAAAAATACATTCCGAAAACAACTTTTTTTTCCCGTCAAAATAGCGCTTTTTAACCTTTTATGA
TAATTGTTTCTTCTTTCCTCTCAATTTGAGTACTAATGAAGTGTCATAGAATGTCATTTTTCTTGTGGTG
TATTTAAACTTTGAATTGTATCCACTCAAATTTTATATTAGTAATAATTGTATCAACTTAAACCTTATTC
TCGTTATTTTCGTTTTCGTGTAATCACTAAGGTATGTTTTCACACGATGAAATATCTTTTCTTTATTATA
TATATATATATATATATATATATAGTCACGACTTTGAAAGTTATCCATACATCAAGTCTTACACGAATAT
ATGCATATTTCTTCAAACTCGGATTAACAAAGTAGATAGTTAGAAACTATTATTTGAAAATCTTAATCGG
GAGTCTGAATTCATTTGTACAATAAATTTTAGAGTTATAATTGAAAAATGTAAATAAGTTTCATACTATA
TTTAATCGAACGAAACATACGAAAGAATAAAGATTGATATACATATACAAGTTTAAGTTTGAGTTTTTAT
TAATACAATACAAAATCTAAGTATATTTAATAAAAATTCATAGATGTAATTTTAATCATGTTTATGGATA
AATTTAAAAGGTTCTAATTATGCTCATTAACTTTAAATTATAACATTAACACATTATCATTTAACTAAAC
AATGACGTTTCATATCTTTTATTATTCAATAAACGTGTGAGTAATTGACACAAAAGGATTAGTGTGGAAC
CATTATTAAGCAATAAATAAGGCTCAATAGAAATAAATTAAATATAGACCAACTCACACATTAATTAATA
TAATTGCAATTAATTATTGGAAACTTCAAGAGCTTATTAGTCTAACATTTTCTAATTTCATTTATTTATC
GATCTCGACTGTTGGACACGTAGCGTTTCTTCATATAAATACAAATATTGACGCAAGTGTTTGTGTTATC
GATTTCGAAATAAGATTTTCCATTCATTTCGCATATTAATCGAAAGAGGAAAAGAGCATCAATTTTGAAA
AGAAAGAAAAAATCTTACCTTTTTCGACCTAATAAATCAATGATAAATTATCAAAGACAAGAACAAAAAA
ATTAGAAGTTAAAAGAAGAAGAGCCAAAAAGAAATTAAGTGAGTAAGAACCACGAAAACTAAGAGATAAA
AAGTGAGATAAAAGTAGAACAAGAGTCGAAAAAACCTCTCCGCATGTTGAGCCGACTAAAAAATGAATAA
GAATAATAGAGTTATGATCTAGAAACTAAACTAAAATACAAGCGAGAAAAGAATACGATAATTGAAAAAA
GAGACAAACTTTAGTAACACCTACGAGAAATAAAATAAAAAACTTTAGCTTATTGAGAGTGATTTTGAAA
TGGCAAAATCACTTTTGATATTTTTTAAATTACTATAAAACATGCTTTTTATTTAAAACAAATTTTGATT
CTATTAAATTAATTTTAAGTGATTAAACGATAATTTTAAATAGTCACTTAATAAATAAATTTTAAATCTT
CCATGCAAAAAAACCACTTGAAAATTCAAGGACCAAACACATATATTATTCATCAAAATTTGAGTATTAA
AGTGATCAAATTAATTATCTGTTTTGTATTGAAAAGAAAGATATGAAATGTTTTTGGAATTGACATTCAC

ATCGTATATATGTATATGCTCAATGTATATTTCTAATAATAAGAACTCCTCGAAGAGAGAAAAAGAAAAG
AAAAGGCTTCATTATTTGTTTTAAAAACTTGACGTCATAAACAATGGTGTTTTTGTTTCGCAATTCTATT
AGGTTTGGTTTGTTATTTGATGGGAGAAGTGTTATGAAAGAGTGTACTTTTGTTATTTTGATTTGAAAGA
GCACACCAAAGTTTTTTTTTTCCTTCATTTTTATTGTACTTTTATTTTCAAAAACATAAGAACGAAGACG
GTTCATACCTAAACTTTTGAGAGATTAAGGATGTTAATATCGTTGATGAAAATACAACCCGACCCCATGT
GTAGGACTTATAGAAGATCCGTAACGTAGGCAACCACAAGTGGTATGTGTGTAGTTTTGGAATAAGTGTT
GTTATATTATTAAAACCTAATGTAACCAAACCCCAAGTGATGTGTGTGGTTGTACTTGGAAAGAAGTTAG
TGAAAAATAGATCATGTACCCAACTCAACTCATTTGTGGTTGCACTCAAGTAACACAAATTTTAGTTTAC
ATTTCATATATAGAAAAAGGAATTTAAAAAAAAAAAATTTAACTAATGAAATAATAATTTATGTTGTTTG
GTTAGAGTGACGAAATAGAAATATATGAAATTTAAAAATGTAAAAGATGATCCGAACATGATTGTGTTGT
GTTTACCTTTACGGTCAAATGATACTCTTACATCAAGGTGTGTTTGGAATATATTTTCAAAAGACTAATT
AAAAAGTATAGTGTTTTGTAACGTTAAAATAGTTTTCTTTTAGTATATTTTAAACATTTTTCATCAAAGA
GTGATTTTTTAAAAACATTTTTATTTTTAAGTCAATTCAAACGGACATTAAATCTCTCGTTTAAATGAAA
TGGACCGAAGGGTGTAGTTTCATGACGTATAATAAACTTTTTGTTCTTTTTTTTTCTTTTTTGAGTTCTAA
TAACCTTTCTTCCCAAGAACTTTTCCAATATTATCCTTAAACACAGGTTTTTAGTTAGAAGTGGGAAATC
AAAAATGAGAAAATCAATGAGACTTAAAAATTAAAATGTCTGAAAAACAATAAGGTTGAATTTCATAATA
TATAATAGTTCATTTCTCATATATATATATAAAGGGTTCTTCAACTCTAAAAAGCAATAAAAGATCCTTG
AACTTTAAATTGTCTAAAAGATGCTTGTATTTCAAATTTTTGTATCCAATAATACATTTGAACTCCAAGT
TTGTAACATTCACCTAATCAACTATTTTTAAAATATAAACATATTAGACAAAAATTAAAAGTTTAATATA
GCATTAGATACTTCTTTTTTTTAATAAGACAATATAAAATTTAGTTTTATGTCCAAAAGATTTGCTAATC
TTTAAAGATGTTGAAAAGCCTAGAAACCTTCCTTAATTACGTACCAAATTGAAAGGTAAGTGATTTTTCG
ACACGGATGAAAATACAAAGATTAAAAATTTGTTTAGATTGACTTTAAAAAAAAAAAGCTTTCGAAGAAA
ATCATTTTCATTTGAATTTTTTTTTAAATTGTTTAAAATATACTAAAAAAATTATCTATGTATCCTTCAA
ACATTCTAATTTTTATTAAAATAACTTATTTTTTGAATTAAACATTCGAAAATGTAATCCAAACACTTGA
AAATTTAAATTTGTGTAATTAAATTAAAATGTATAGCTTAAAAATCTTAATAGCAAACAAAAGGTAACAC
AAAGAGACTCACTACACCTCTAATTCAATGTTGATGGGCATAAATTCTAGGGGAAAACAAGTTTGTTTAC
AATGTTTTTGCTTAAATAGACATGTTTTTAACTTCCAAGAACGTTCAATTTACAGCTAAACATGATAGAG
CATCATTATCTTCAGGTAAATATGAAAATACATCATATCATATATACACACAATAATGAAACGAGGCAAA
ATGCCTCACTCAAATGACAGTAAGAATGTAGATGTACATACCAATCAGCCAAGGAGTGAATTGGTGTTGG
ATACAGATTCTTAATCTCATCATTCTTTGTGGATACGGAGAGGAATAACAACGCATTCCAGTAGAAAGCA
CCTGAATAATTTACACACTATCTTTACTCCAAAGCAACATTCTGCCTTTGTGAAAATTAGGCAATGCTCA
TATGCCTCTCCCACCTGATGTCCTCGGCGAATTCTGTAAGAGCAGCTCGAGGATTGCCATTCGAAATGGA
TAAGTAATATTGAGCGGTTTCGTCGTCCACCATCATGCTTCGACTCAATGAATGAAGTAGCCGCTTGTTC
ACTTGAACAGAAGACATACCTTGTTCGGATGTCACCTTACCATACAAATGTTGGGTCATGGGGATTCCAT
CCAAATATAACACTGCAACTTCCCGTTTTGCAATAGCATCCATCTCGATGTAACATATACTGTTAATGAG
TATATTGGGATTGCCAATTGGGATTAACAATCTTTCTCTTGAGTATATGCCATGGTCACTCATCATGTTG
TTCAGACGTTTTATGTCTGTAACCTACAAGAAAGACGTATATATAAACACATAAAATTAATACACCAAAG
TCCATAACCATAGAAAAGCATTCATCTCAACAATGCAACGGTACTACGTCATGTTATTTATATGTTGTAT
GTATATAACCTACACAAACAATATATGTAAACAAGATTAATATACAAAGTCCATAGTCATAAAAAATTGA
ACTTGACCCTTCCTAATTGATTTATTACAAGTGCTTGGGAGTGGGAAAAAAAACTGCTTCACTCTTCATT
TTTGCAGCAAGTTAATGTTGTGAAAGATATACAAGTTTCAACCATTGTTAAGACTATGATGATTTCAACA
ACACACCCGTCCATGGCCACCAAAAAGAGCTGATTTCAACATTTATACTTGATCCTTCCTAATTGATATT
TTACAATTTACAAGGATTAGAGAGCATGTGTAAAAATGCCATTTTTGTAACCCATGTATAGAGGTGAAAG
ATATAGCGGTTTCACACAAGTGATATGATCGAGAAGTTTCTCCTCCATTTGCCATTTCCTCCGAGGGGCA
TTTTTTATGGTTGGCTAAGGTGTGTGCTGTCATCTGGAATCTTAGGGAAAAAATTAACAAAGTGTTTAGA
AAGATGGCAAGGAAACCTAATCATGTGACTTTTGAGCTATAAGATTCATTCTTTTGGGTTCGTATTCGAA
GTTCTTTCTTAGCTATTCTAGAGGCATTATTTTGCTTGATTGGTTCCTTTTATTTAGAGGGGTCCTTCTT
TTGTGCACTCGATTTTTTTAGCTCTTTCATTTTTTTCACCTCAATGAAAGTAGTTTAATTCATTATATATA
TATTAGTTTCAACTGCTGGTGATTTCAACAATGTATCAAAGTTCATAGCTAGCCACAAGAAAGAGTTCAT
CTCAACAGTGGAACACAAATGTCCTTAATTGAACTTTACAAGAACTCAAGAGTTGGAAACACAAACAACA
GAGTACGAAGTACAGATTTTTTCAAAGGTTAACAAATTTCAATTAAAGTTAGAACTTCAAAGCAGCAGTG
TCTTCAAGATCAATGTTAAAGAAATCAAGTATCTATAAGCTACACTGCTGCAATAAACCATATAGCTACA
ACAGGAGTAATGAAAGTTCAAAGTGTTCTCTGTACTCTAGTTCCCTTACTTTCAATTCATATGTTTCCAC
ATCCAACATAAAACAAAAATTCAGTAAGAAAACTTATGAAGTTTGAAGAGCCATAAATCTGTCGCATCGT
CGAGTTTTCAGAGAAAACAAGGAAAACTGATGATCCCAACTTCTTAAAATTAAATTGAGTCTCAGAAATT
CTACTATTTCTTTCCCTAATTTCCAGTCACTTTCTCAATAATAAGACAACAATAATATTCGAAGCCCGTA
CCTGAACAGAATATTTGACCGCTAGACTAGCCAAGCTATCGCCGCGAAGAATTCGATGAGAGATCGCAAA
CTTCCCAAGGCAATTATCTCTCCAGAAGCTCCAGGAGCTCGGCTTCCCGACCACATCCTTCACCTTCCAC
GGCGCTTTGAAAGCTCCGGCGACAATTTCCTGATCGGAAGCAACGGAGTGCCATAGACGGCAAACGCAGC
TGGAACGAGCCAAGTCGGGGATGGAAAGGTGCTCGAAGATAAGGCGGAGGATGTCGCGGCAGGAGAGGGC

GAAAAAGTGGGAGTTCATCGGAGACAATATGGCAGTGGAATGATCCGCCGGGGGAGGTTGAAGCGGTGGA
GAGTGAACGGGCGGCGAGGGATTGAGGTGTCTGAGGATCTCAACGTCGTCGTCTTGATCGCAACAGCATC
CCATTTTAGAGAGAAAGGGAGAAAGAAATGAATGGTTGGTTTTTGTTGGATTTTATTTGAACAAATTACA
ATTAGATTGATAAAGGGTAGGATTGGAAATATCTATAAAGTTAGGCCTATAATCGTCCACTCAACGATAA
ATCTTTTACCTATTTAATTTAATTCTTTCTTTTCCAAGTATTGGATTTAGAAATGAGTAAGACCGAAAAA
ATGAGTCCTACATTCAACCAAAATTAGTTAATTGGAAATCTCAGAGGGTCGATCATGTTGGTTTGAGCAA
ATAAAAAGCAAAATGAACCGATTGATTGTCACTTACTCCTCCATTGTTAAGGTTTGGAAAGATTTACTAA
ACCAATTATGATTGGCTCAGTTTGATCGAAAAATCAACTTCTACCAACAAATGCTTACTTAATTAAATTT
AGAAAAATACTTTGATACTTGTCGGACTTTGATAATTAACTAAAATGTCAAATTGAACAATTGAAATACA
CCAAACCAATGGCTAGAGGTTGAGTCGGGTTTAATTTTCAAAAATCAATAAAAAAATAGGTGATCTAAAT
CGAAACGAACATAACTAATCGGAGACTATGGACTCGGGGATTGAATTTAGTATATTTGCAATTTTGTTAC
TCAATTATTTTAACTTGAAATTGTTATTGATGGCAATCACATGAAAATTGAAATGTATTTTTTTTATCT
AAAATGTAATTAACTAAACAATAATAATAAGAGCATAAGAAGTGGTGGGCGGGACCGGCGGGACGAAGTG
TAGTGTTGAAGGACCTTATCTTCTCTCTCTCCGACGATGCACTCACTACCGTCGCAAGCGATTCGTCCTC
TTTCACTCTCTTCATCATCTTCAACCTCTCTCTACCTCCGTTCCATCTCTTCAACCTTCTCAGTTTCCCC
TTTTTTCAATCTTCAATCCCCTGTTTTTGCCGCCATTTCCAGCCGACTACGACGTTCCACCGTCAGAAGT
TGCTCCTCCATCACCGCCAAGCCATCCTCGGAGATCAGACGGACCCGCCCTAATAACGATGAGCCTGATT
CCAAGCTTCGGGCTCTCCGTGACTTGTTCTCGAAACCCGACATTGGTATTGACGCCTATATAATCCCATC
ACAGGACGCTCACCAGGTCCGTCTCGTAATTTCGTTTTCTCTGGTTTCTTATGTGTTGTTAGAGCATAAA
TTCATTTGGATGTGCTACTCAATCCAACTGGCCGTCGTTTATTTTTATTTTATTTTTTCCTTTACTTCTG
GAAGGAGCAATGTGTGTAATCAAGGCTTCATTTGATAAATTTTGGGTTTTTTGTTTTTGGTTATTGAAAA
TTTGTACTTGTTTCTTCCCAATTTCTTGAACATGGTTTTCATTTGCATTTGAATTCTTAGCCATATTCTA
AGAACAAAAACACGTTTATGATGGGTTCCTTTTTTGTCAAATGGTACTCAATTGGCAATGTTTGTTGTTC
TTGAAGTTTTTTGGAGGGGTGGGAAGAAGAAATCTGGAGTAATTGAGATTTTATGCTGCGAAAGTTTTAT
GGAAGTTCTATTTTGTATCTGTTTTCAGAGTGAATTCATTGCAGAATGTTACATGAGGAGGGCTTATATA
TCCGGATTTACTGGCAGCGCTGGAACTGCTGTTGTCACAAGCGACAAAGCAGCACTTTGGACAGATGGAC
GGTATTTTCTTCAGGTTGAAGACTGAAACTTGCTCATTCTCTTGTTCCTTGACCATTTATAGATTGTGAG
AATAATTTTCCTATTATTGATGATCAGGCTGAGAAACAACTAAACTCTAGTTGGACTCTCATGCGAGCTG
GAAATCATGGAGTGCCCACCCCAAGTGAATGGCTTGCTGATATTCTAGCTCCTGGTGGTGTAGTTGGAAT
TGATCCCGTGAGCAATTGTTCTTTTGTATTCTCTTAAGATGATTTCCCCTGTTCTTTTTTCCAATTCTAA
CTTGGTTTTGATTCCTTTTTAATTTTCTTTTTCAATTCTATAACCATAGAATGTTAACGCTTTGCGTACT
CCTTCCTCTATCTAATGCCACCTATTGATTTCGTGAAGGGAGCAGTTTCTTTTTTCTGCCGATGCTGCAG
AAGATTTGAAAGAGACCGTTTCTAGGAAGAATCACAAGTTGGTTTACCTTTATGATTACAATCTCGTGGA
TGAAATATGGAAAGATTCAAGACCAAAGCCACCTAGGGGCCCTATAAGAGTGCATGACCTTAGATATGCT
GGTTTAGACGTTGCATCAAAGTTGGCTTCTTTGAGGTCTGAGCTCAAAGAAGCTGGTTCATCTGCAATCA
TTATATCTGTGCTCGATGAAATTGCCTGGCTGTTGAACTTGGTAAAGTTCTATCTTATTTGTTTATTTTA
AATTAACTAAAGCTGGTATTTTTATGCCTTTTCAAAATATTAGCCGCAAACTACACTGTTATCCTTATTG
TTTTTGTTGTTTTCCTTCTAACTTCTAATTTTCAAGCTAGGATAACTCAAAGAATGTGATTCCACTTTTT
TTCTGTTTGTATACAATGAATTATATTCGGTAGAGCATTCTATGTATTATTTTTCTATTCCTTTGCAGAG
AGGAAGTGATGTTCCAAACTCACCTGTTATGTATGCATACTTACTAGTTGAACTTGACGGAGCAAAACTG
TTTGTAGATAATTGTAAAGTCACATCAGAGGTGATGGATCACTTGAAAACTGCAGGAGTCGAGTTAAGAC
CATATGATTCCATTATTTCTGCAATTGAAAAGTAAGTAAAGTTAATTGAAACTTTTTTCTCTGAGTAGAT
TAAGGATGGAGATTGTTTTCTATATTGCTTTTAGAAAATTCTAGCGTTCTCATTTTAATTTCTACATGGA
AGATATATCAGCACTATTAAACATGGACAGAACAGAGCTTACATTTGAATGTGAACTATATAGAAACTTT
CATGAGCAAGTTTCACTATCATGGTATGCATAGGGCTAGGAAGATAAAAAAGAAAAAATAATACAAAATG
AGACATGATATTCTTTCCATATTTTGCTTTTTGTGCGAAGAAAACCCTTTGTGCTTCAGTTATCAATTAC
AATTTTAATGTTGCAATGATCACCATGCATGCATATGGTCATATCTTTACTTCAATGATGGCTGGATTTT
TTTTCTTCAGTTTGGCAGAAAAAGGAGCTAATCTTTGGCTGGACACATCATCAATTAATGCTGCAATTGC
AAATGCTTATAGAAGTGCATGTGATAAATACTTTATACGCCTGGGGAATAAAAGAAAAGGCAAGGGTAAG
ACTTCTGAGACCTCAAATAGTCAGGTTGGACCTACTGGAGTCTATAAGTCATCTCCCATTTCAATGGCTA
AGGCCATAAAAAATTATGCTGAGTTAGAGGGGATGCGGAATTCTCATTTGAGGTAACACTGTTCTATCAT
CAACATTTTTCTTCAATCTTTAATCTTGATTTTGTAATACTGTAATAAATGTGCTATGCCACTTGAAATA
TCCGTTGATATAGTAACTGATGACTTGCTACAGCCTACGACTTATCCATTTTGAATTGTATCACTTGCTA
ATCATTTGTGTTTTTGATAGTGTAGATGCTCTTTAAAGTTAGTGAATTTATTTAGTGAATTAGTGGTACA
ATGTTTTCATGTAGAGATGCAGCTGCTCTTGCTCAATTCTGGTTCTGGTTGGAGCAGGAAATTCTTAACG
GTGTTAAACTAACGGAGGTAGAAGTTGCAGACAAGCTTCTAGAATTTCGAAAGAAGCAAGATGGTTTTGT
TGACACAAGTTTCGATACTATTAGTGGTACGTAAATTGCCTTGTGCAATTTTTCTTTTAAATATCTGTTA
TTTAATTAAATGTTCCTGTTCTCAGTTTCTTCAGCTTCACGGCCTATTGTTGGAATGATTTTTCAGCCTC
TGGTGCAAATGGCGCGATCATACACTATAAACCAGAACCTAGTGATTGTTCTGTTGTGGATGCAAATAAA
CTCTTTCTCTTGGACAGTGGAGCGCAATATGTAGATGGAACAACTGATATAACTCGTACAGTTCATTTTG

GTGAACCAACCACTCGTCAAAAAGAGTGCTTTACGAGAGTCCTACAAGTACGAAATCTCTATTTCACTCA
TTTCTATAATTCATACCAAATTGTAGACTGATAATTATCTCTTCTTACTTTTTGAAAGATCTCCATTGGA
GGAACTGAATGTGTCCTCTAAAATATTAATCAATTGCAGGGCCATATAGCTTTAGATCAAGCAGTGTTTC
CTCAGGATACCCCTGGTTTTGTATTAGATGCATTTGCTCGTTCTTCACTCTGGAAGATTGGGCTTGACTA
TCGACATGGTATTCCTTCTCTCTGTTGACTAATGAAGTAATTATCTTGTGTGCGAAACAGTACATATACT
ATGACTTGATTAGAGATGTAATGGCTAATTATTTATTAATCTATTTTCCCTCCCACCTTTCTTTCAGGGA
CTGGGCATGGTGTAGGGGCTGCACTAAATGTTCATGAGGGACCCCAAAGTATAAGCTTCCGATTTGGGAA
TATGACTGGCTTACACAGTGGAATGATCGTTAGCAACGAGCCAGGCTACTATGAGGACCACTCTTTTGGT
ATTAGGATTGAGGTAAAACCAATCAAGTGATAATGTTTGGGTGTGTATGCTTGACTAATGAATTGGAATT
GATCCCTATACTAATTAAACGAGTATGTTTAATTTAAGGGTTTAGCACAAAGTTCTCACACCTTCTCTAT
ATTTTGAATGTGAAATAGCTTAAACTTTTCTGGTTAGGTTTTATCCATTGTTTTACCTATAATTTTGATG
GTCTTCTCGTGCAGAATCTCCTTATCGTGAAGGATGCTGACACTCCAAACCATTTTGGAGGGATTGGATA
TTTAGGATTTGAAAAACTCACGTTTGTGCCCATTCAGGTAAACAGTAGGAAAATTATACGGATGACTAAT
GGAAAAGAAAAATCATTAGAGAATATGACAATGTTTTTTTAGTTTCTTTCTAGAATATATTATAGAAAAA
AAAGATATTGACTGCCTGAGATATCTCATCAGGATTTATGCCTTTATTTTGCCTCTAACTCGATTGTTGA
AGTTTATCTTTAGATATTTCATTCAATCAATGGAATCAAATCTTATCCAAAAAAAGAAAAAAGAAAAAAA
AAAAATACTGGATTGTTGAAGTTAGCATGCATTATGATTACCCATATTCACTCAAGGTTGCTCTTAAGGT
CCATGAAAAGAAGTCTCATTGTTTTCTTAATTTGGAGTCAATTTATCAATTTGTTTTTAACCAAAAAAGA
ACTCTTGCATCAGCGTTGTCTATAACGATCGAGGCCTTTTTTGTGGCCTACCTCCTGTCTACTGTTTAGA
ACTTTTTTACTTCGAAGACTCTTCAGATAATAATCCACATCCTGCAACTATTTTTTCTATGAGGCCCTTT
TTTGAACTCTTTTTTCTGCCGGTTTCCTTATGCAGACTAAACTTGTTGATATCACTTTGCTCTCTGTTGA
GGAGGTCAATTGGCTAAATGATTACCATTCACAAGTCTGGGAAAAGGTATGACATTCATACTCCAATACT
TATCCGTGTTGTAATTTGGAATCGATGTTACATTTGGAGCGTAAGAAAACAGTCATCTGTTTTTCTAAAT
CAAGAATGCTCTTTTAAGAACTAGAATTTTGACTATTTTAGAAGTGATTCCCGGTCAAAGATTAAAAAAC
ACCCATCACTTAGTTCCTTCAAAATTAGTTTGAAATATTTTTATATTTTTCTAAGATCAATTTTGCTTCA
TGCCAAACACCGTCATTTTAAGGAAAAGCAATTTTGGCAAGATAAAAGGCGATTTTGACCCTCTCTAAAT
CACTGCAAAACACACCCTTTAAATTGTTTACAAGAAGAAATAATAATAAATTTGTAGCCAATGACACCAT
TTTTAGTGGTATGAAAGTAGATTATGCTCCAGTTGATAATATGATAAATGATAAGGAGGAGGGACTAGGG
AGTGTTGTTTGTGGATGTCTTCTTTTCTCTGTTTATTGACTTATGGTTGTACAGGTTTCTCCGTTGCTTG
AAGGTTCGGCTCGCCAATGGCTGTGGAACAACACTCGACCACTCATGAAATCCTGATTTTTTTGGTTTGA
TTTTTTTACGTTATTTGACATAATGTTGTGATATGTGTCCGTTGAATCAAATGTAGATGAATAGTGAAAT
GGTATGAATGGGTTTGAAACTGAACTTGGAAATTTACTTCTATAAGAAATGGGTTGTTCTTCTCTTCCTT
TCTTTGATGCCCTTTTTCCCTCATGGATATTATTCTTCCTCTTATTGGGTACTTAGGTTGATTAAAGTCC
ACATCACAAATTTGACTACTAAGACATTACTCTTTGCGTAATTACGGTAGATAGCAATTTTGAAAATAAT
AATTAAGTATATAATAACATTTTTAAAATTGTAAATATAGCAAAATCGATCGATGATGTACTTATGCTAA
TATTTGAAATCTAATTTTACAATCATCCCTACATGCAACTAGCAATTGTAACATTTCTTATAATTTGTAA
ACATGTTAGAAGTAATTGCATTTTATGTTAATATACTATATTGTAAAACTTGAAAGATATCATATCTGGT
TTAAATATATATAAAATTAAAAGTTCAAATTTTGTTAAGCACAAGTATGAAAGAACATCATGAACTCATA
ATGGACACTGTATTAGAAGTTTATGAGTTAAGCTATAACCATCGAGAATTCTTTCTTTTTGTTTGACAAT
GGTCGAACACAAACTCTTCATGTAGCATATCTAGTTATGACTTTATCCCAGAATGAACTTAACTTCAAGT
TTCTCAATCAAGTTTTTCAATCAAGATTTTTTTTTTTTTTTTGTATAACAACTATAAAGTTGGAGAATTCA
AACATCTCATCTTTAGAATAAAAAGCTTATATGAATTATTGTCGACCGGAGTGAAACAGAAAAAAACCAA
TATCAGCACAATATCAACTTGTGTGCAATGAATAAAATGTAGTCTTAAAACTCAATAAAGTTGCAATTTC
CTTCATTCTGAATAAATCAACTTGAGAGGGAGAGATTAAGAATGTGTGAGAGAGAACAAAAACTGGTCAC
CGTCTCCGATTCCAATTTCATGGATGAAACTCAAATGGGTATTTCAAAAATCGAGCTCACACCTTCCCTC
TTTGGTCACTTCTCTAATCTTCCCCTTCAGAAACCAATTCCATCAAAACCCATTTGCAGAAACCTCCTCA
ACATTCGTCCTCAACCATCTAGACGTAAGCTTCCTTCTATCCATTTGTGGAAGAGAAGGGAACCTCCATT
TGGGTTCTTCTCTCCATGCATCCATCATCAAGAGCTTCGAGCCCTCCAACCATTATAACGGGGTCGTTAT
AATGAACTCTCTAATCTCCATGTACGATAGGTGTGGTAAGTTATCCGATGCCGTCAAGGTGTTTGATGAA
ATGCTCACAAGAGATACTATTTCGTGGAACGCATTGATTGGTGGGTTTGTGAGAAATGGGAAGTTTTTTG
CTGGTTTTAGTTATTTCAAGGCTATGTGTTTGGTTGGTGATTGTAAATTTGACAAAGCTACTTTGACTAC
GATTTTATCTGCTTGTGATGGGTTGGAGTTTTGTTGCATTATTAAAATGATGCATGGTTTGGCGTTTTTG
AGTGGGTTTGGACAAGAAATTACTGTGGGGAATGCTCTGGTTAGTTCGTATTTGAAATGTGGTTGTGTTG
GTTTGGGGATGCAAGTTTTTGATGAGATGGGGGAGAGAAATGTGATTACTTGGACGGCTGTGATTTCCGG
TTTGGCTCGAAATGGGCATCATGAGCACAGCCTGAAGCTGTTTAAGGAGATGATGAGTTATGGTTCTGTA
GAGCCAAATTCTTTAACTTATTTGAGCTTACTCACTGCTTGTTCTGGTTTGGAGGCATTAAAGGAAGGAT
GCCAAATTCATGGCCTTATTTTGAAGTTGGGAATTCAATCAGATTTGTGCATTGGAAGTGCTCTGATGGA
TATGTACTCGAAATCTGGAAGAATTGGAGAGGCTTGGAAGATTTTCGAGTCAGCTGAGGAACTTGATATG
GTTTCATTGACTGTTATACTTGCAGGGTTCACACAGAATGGATGTGAGGAAGAAGCCATTCAGATCTTTC
TGAAAATGTTGAAGATGGGGATTGAGATTGACGGAAATGTCGTTTCAGTTGTTCTTGGTGTGTTTGGTGC

CGATACATCTCTGAGGCTGGGTCAACAAGTTCACTCATTTGTTGTCAAGAAAAACTTTATTTGCAACCCT
TTTGTGAGCAATGGGCTTATAAACATGTACTCCAAGTGTGGAGCACTCGATGAATCAATGAAGGTCTTTG
ATAGAATGCGTGAAAGGAACTCGGTTACATGGAACTCCATGATTGCAGCGTTTGCCCGCCATGGAGATGC
CTCGAAAGCTCTACAACTTTATGAGAATATGCAACTCGAAGGTGCAAAGCCAACCGACGTCACATTTCTA
TCATTACTTCATGCTTGTAGCCATGCCGGGTTAGTAAAAAAAGGAATGGAATTCCTCAAATCAATGACAA
AAGATCACGGGATGAATCCAAGGAGCGAACACTACGCTTGTGTTGTTGACATGTTAGGTAGGGCAGGAAT
GCTGTCTGAAGCTAGAAACTTCATTGAGAAACTGCCTGAGCAGCCAGGTTTACTCGTGTGGCAGGCATTG
CTCGGCGCCTGCAGCCTCTATGGTGATTCTGAAATGGGGAAATATGCGGCCGACCATCTGTTTTTGGAAA
CTCCGCATAGTACCGTCCCATATGTTTTGTTAGCCAACATATATTCTTCTGAAGGGAATTGGAAGGAAAG
AGCAAGGACAATTAGGAGGATGAAGGAGGTGGGAACGGCCAAAGAAACTGGTATCAGTTGGATTGAGATT
GACAAGAAAGTACATAGTTTTACTGTTGGAGACAAAATGCATCCGCAAACTGAGATCATTTATGGAGTTT
TGACGGAGCTGTTTGTACTCATGGTAGATGAAGGATATGTGCCAGATAAGAAGTTCATCCTCTACTACTT
GGATGATGACAGGAGGGATCCAATCCATAACGATCAAGCTACCCGTCAAAACGCCATAGAAACTGAAGTC
GTTTGGGAGCTGTTTTAAAAAACAGTTAAAATTTTTATTCTTATTCTTTTAAGCACGACCAATGTGGTAT
AAAAATTTAAATTTCCAACTTAAGAAAACGTATAATTAAAGGAGTACGTGCCCATTAGTTATACTCACTC
CAGCAACACAATTTTGTATTTTATTATTGCTACTTTGTAAAAGTGGCAAAAAGCTTACGATTTAGTTTTT
AAACTTCTGTTAATAACAATTTAGTCTATCTATTATGAAACTCTTAATAATGATTAAGTCTCTAATTTTT
AATAAGTAATAATTTAATCCATATATTTTGACATAGGTAACAATATAGTACCTATCATGAAAGTTATTTT
CAAAATAAAATGACACATGTATTATATAACAACTTATTCTTTATATCATTATAGTTTTTATGTGTGTAAA
AAAGATTGATTAGAATCAATTTCAACCTACGTATGTCAAAATTTTATTAAATTTCAATAACATTTTTACA
AAAGATACGAAAGGGTTAAATTATATATATATACATATATATTTTGCTATATTTTCAAAAAAAAAAAAAG
AAAAATTAAAAGTTTTGTTAGTGTAAAATAATTTGCCATTTTTGGCATTTAAGAACAAAATCAACCTTCG
GCGAAGCCAATCAATAATGGAGGCGCTGTGTTTCTCCACAACCTTTCTTCCATTTCGAAAAACCGTTGGA
TCTTTCAATGACACTTCCAATTTTCCCTCCATTTCAAAATCTTCCATTCTCTTCTCTTCCTCGTCAACCT
CCCGATTCACAACGATTTGTAGGCAAAGAATTGAAGGAGAAGACACGCCTCTCTCCGGTCCTATGGCTGC
GTACAGATTGCTCGGCCTCGACCCAACTTGCTCCGAATCCGAGCTCAAAGCCGCTTTTCGAGCCAAAGTA
CTGTCTCTCTCTTCCCCTTGCCGCTTTGATTTCTTTCGTTGCTCAGCCAATTGAATTGTCCTTGACTACA
TTTCAGTTTTCGGGTTATAGTTGTCGGACTGATTTGGCTACTTGGGTTGGTTTTTTGAATTTTTTTTCAT
GTTTTGATTTTCAATTGGACTTCTGACTTTTGGATTTTTAGGTGAAGCAATTTCATCCGGACGTGACTAG
AAATGGGAATGATTCGGATTCTATGATTCGTCGTGTAATTCAAGCGTATGAGGTAGCTAATAAAGAGATA
ATCATTTATTGAACCAAGAAGAAAGTTGTGCTGTGTTGGTTAGCTCGATGCTTAATGTTGTTTTTTTAGT
GGGATGAGCATTATGGCAATCATAATTCATGTTGAATCTTAATTTGATTGTTGTTGGGAACGACCATCAT
TGTAGTGATAGTCAATTCCCAACAATTATCATCTCCGCGTGCTTAACATTATAGAAATTGAAAACCATTT
TCTGTTGTTTCTGTGCTAGTAGAGTGGATAAGGCTGTTTACCTGCTTACAGACGAGAAGGAGATTGTTGA
TCAACTCTCCTTAAACAACTTCTGTGTTCTTATTGATATTCTTTGAATGATTTAACTGGCAGTCATTAAT
TAGTGAGAAAAATTGTCTAGAAGTTACCGAAAGAACGTGTTTAGAGAGAGAAATTAATGAAAAAAAGGTG
CATAGAGAGTAATCAAGAAAAAAGTGTCATTGAATATTATTTTGCAGGGTAGTTTCGACTCTACTCTAGT
TCATTTTGACTTATATATTTGCAAAAGGTTCATTCTAGTCTCTACTTTCAACTTTTGTTTGTTATTTGTT
GTTTTGAAAGTATAGGGACCAAAATAAACTAAAGCCAAAAGCCAAAAGCCAAAAGTATTGAGATAACAAA
AGTACTTATTTGATTTTTGGTCTCTATTTCTCATAACTTCTAGACTTCGATCATAAGAGACGGTAACAAG
GCTGGAACTGTTTGTGATTTGATAGCCAACTTGTTTTGCCATCTATGTTGTAGTCAATAATGAACTCAAC
AGTGACAATGCTCTGTAAAATATGCAGATGTTATCCAGTTACAGCCGAACAGAGTTCATTGAGAGGTATG
GTAGTGCTTTTGATGTTTGATCTCAGGGTGTATAGAACTTCTTAACTTCTTGTAAAGCTCATCATAGAAT
GAATTGTGTAATGTCCATATGATTGGAAAGCTTTGGGAAATTTTTGTTTATAAATAAACGATGCTATGTA
GTATATAACAATTTAGTACTTTTAACAGAAATTCTACCATATTTTAGGGGCTAAATCTCCATTGTTGATA
ATTTGAGTCTTTGACATTCAATATGGTTTTATGATATCAAGTTTCAGATCCACCTGCAGTCATTCTAATT
GGTATATCTGTTATTTATTTTTATGAAATTCAGGGAATGTTTAGATCCTTTTGAGAACCCAGAATGTGAA
GCATTTGATGTCTTTGTTAATGAGTTTCTTTGTGTTGGAAAAGGTACAATCTTTGCTCCAACTTAAGAAT
GTTAGAATGTTGTAATACAGTAATGATATCCTTCCCACTTATTCCCCTCGCACATGTGTTGTCATAACAA
TATGCAGGCTGCCCATATTCTTGTGTGGATAGAGCTCCCCATGTCTTCACTTTTGCCTCTTCCACCGGGA
CAGCCCGTGCCACGTCTCAAGGTTCGTTCTGAATGATAACACAACTATAATTTCAAATTTGGGTGCATAA
CCAAAAGGCTTATGAGATGTGTGCATCATTCTAACGGTGATTTCTCGATTTATTTCCTGTAATTAGGACA
TAGTGAGGATTATCAGCTTCAGATGGCAGTTGGCCAATGCCCCAGAAGCTGTATACATTATGTAACACCT
TTGCAAAGGATCATTCTGGAAGAGCTGCTTGACAGGTAGAACAATATTCCGACTTAGACATATGTTTTGA
ATATAGAAAATATAGTAGATTATCATTTTTGAAAACAACTCGTTGTATTTATACCAAGAAAGATCGTAGT
TTTTTCCCCTCCTTTCTTTAGCTTTTTGGTAACTGTAGTTTATCTTTGTTTGTGTAGTGCCTTGGATGTA
CCTTATGATAATTCTGCAGAGGCAGATTTACTATATTCTCTAATAGTGAAATCAAAATTCGAGAATAATC
GATACAAAAAGCCAAAGAGGGAGCCAAAGAATTCAACAGAACGAGTGGATTGGTATTGAAATCAGATTTA
GTCTCATGCTGAGAAGAATTAAGAAGAAGCCAACGCAGTTGACGAAATACATCAACAGCATGGTTTGCCT
CTGCAACCGTAGAAGTCAGAAATATCCTCAAAATTTGTGTTGTATACTATCTTGAGCATCGAAGTTAATG

CGAGAATGCTACAATATAGGTGAAAAAGGTGATTTCTATACGTTCAACTATTAAAGTACAGCCTCTAAAT
TATCAATTATTCTAGGGTAAAATCAATTTATATTCCTTAACCTAATAATTGTATTCACAAGTGAATCAGT
TTAGATCACTTCTTAAATTTTCATTTCAAAATTTTTCATTTTAAAATCCTTAATGCATGAGCTTCTACCA
CACAAGTACAAGACTTCTTTAAGTATAGGGATTAATGTAAATTAGAGAAAAAAATCTAGAATCAAATTAG
CATAAGCTTTTCATCTATTTACCTTAATTTTTTTCTATTTTTAGAAGGAATTACGTAATATTTTTTTTTT
GCTACTTGAGCAAGATTTTTGGTGAATAATAATTTTATTTGAGTGATTTTTGAGTTGATTTTAACTAAAA
TCACGTGAAATAGCTATTTACATGCATTGATTTAGTTACGAAAGTTGATTAAACTCTCAAATATTTCAAG
ATATAAATTGATATTTGCTTTAGTATTTAATACAAAGTTAAAGACAAAGTTGAAAATATTTCTCTTCGAA
TTTGAATTGTTAGTCTCATTTGCATATGATTCACTTATCAATATTATAAGGGTTTATGATGGAATTTTGT
TGAGATACTGTAAGGTACCTAGATAGGAATACATTAGTATGATATTAATAAGGGATTTAAGGGTAATTAG
ATAGGAAGTTAGTTACTGAATCTTATTATAAATAGAGGGAGGGGTGAATTAGTGAAGGTAAGCATTAGTT
GAGTGGTTTAGGGCTTGAGTTTGTATTTTCAAGAGGTAGGTTCCAAGTATTTTGAACTTCGTTTATCTTG
TATTCCTTCATCCTTACGCTTTAATATATCCGGTTCTTGTTCTAGTTAGGAAGTATTCTAATTCATGTAG
TAATAAACCGGTAGCTTATGATTGTGGTTTGATTATCTAGTTAATTCAGCTATTTTTTTATTATTTATTA
TAATTGTTGGTGGTGGTGGTGTTAAAAAGAACTCAACTAAAAAAATTCACACATTGGAAGAACAGATATAT
CAAGAAAATGGACCCTGCTTTGGTTTCTGGAACTAGTGATTAGATTTAAGGTAACTCATTAAAGACAGAA
TAGATTGCGTTTGGAGTTCATAATATACAAGGACCTATTATTTTTAAACACATCAAGGAAAATTGCTCTG
CTTATTTTGAAATTGCCTTCATGAATCGGGTTAATGAAGAGAGAACTCCAGCTGCTTTGTCCAAAAATGA
CGGGTCCTGGTCTGAATCGGCTACATTTCCAAATATTATCTGCACAAGATTCTTATACTTACATTAGTGA
TCTAACTAATCTCTTCTCGACTTAAGAACCACTGTAAGATTGAAATTGTGAAAGCTCCACAATTAAGTTA
CTTATACAATCTATATATTTACTTCTGATTATATAATGTGAGGTAAAGTCGATGACACATTAGTAGCTTT
AAGCTCAACTAACATCTTCTAATTTCTCATATGTTATTTTCTCAGGTAACTGACCTCGAGGTCTCCAGCA
TCTGGACGGCTTATTAATTCTTTGGCTAGCAAATATCTGCCAGGCCTATCTTTGTCATCATAGAACACTT
TCCATAACCTGGTCGAAGATTGCAAAGGTGTTAATCACACTTCTTTTTATAAAAATAAATTAAAAAAAAG
GAGGAAGAAGAAAGTGGCTGGAGAAACTTCAACCAAAGGTATTTGTGCATTTAGCGACCTGGGACTTGAG
TTCGATTGTTATTTCACTTTAATGATTCCCTTACTTACATGTAAGATACAGCATAGTATTTGATTATATT
CTTAAATTTGTTCAGGTCATACAACTAAAAAGTAGTCTGGACCATCTGAGTTGTTCCTATATTAACTGGA
ACAGGTAATCATTCTCTAGAGGGTTGAAAAGGCCAAAATAAGCTCAGCAGTCAAATAAAGGAAATAAACA
ACTTCAGCAAAGTGAGTAGATGATTTAATGAATCTTGTAAGATGTTAAAATCTTCTTACCCAGGATAAGC
TCTAAAGACAGCTCCAGATGGCAAAGGCCTCATGGAGTAGACTGTTGTAAACGTGCTGTACAAAAGATAA
CAAAATAGAGGTTGAGTAACACAAATCCTTTCTTACCTGAACTTAATGACCAGTTACAAAGTTTATAGTC
TCTCATGAAACTACTTTTCTCCTTAACAACTGTTATGTATGAGGTTAGATGTTTACTTCATATTAGAAAG
ACGATCATTTTTCGTAAGCAATTATTTCCCAAAGAAAATACCTCTACTTTTATTTTTCATAATGCTGATG
TCTACTCAGACCTTGATTTGTATTCTAGATATGGTAAGATTTTGAAATTTACCTTAAAAAGTATCTTCTC
AATTTTCGAACGTTAAATCCAACCCCTACATCTTCGCTGATGAGACGAGGGTTCCACATGATAAGAGGCC
TTGGCTGCCATCAATGTGGAAAAGTAAGTAGGAAGAAGGAACCAAGTCCATTTGATTCAGTGTACAAGTG
AAAACTGTCAAAGTCCAAATATATGTAAATGATCTGATGATGTACGACACTTTCAGAAAGAATAATCTTC
CTTTTCTTATTATGTTAGTGTATGCTAACTCAATAAAAGATAACCTAATGCATATTAATTTGAACTTATG
TGGCTTCATTATATGATTGCAGATCTCCAATTTTGGCAGTTGAGTATCATGATATGAAAAAAAAATTTGG
GAAGCAACCATTTCAGCACCAAATGGAATTATTTGGACTTTTACCGGATCGTCTGAGAGGGCAGCTGCAA
TTTTCTGTACATTTTCCAACATTTGGTAATCAGGAACAACCATAACAACTATCTCATCTGCAATATCTAC
AGGTTTTCGATCGCTTAAGCTGAGAATGACAACCATATGTCATTAAGTGGTAATAGTACTCAGGCCAAAA
GAAAAAACATATAGAATCAGATTGTCCAAACCCATCTTGGTTTAGCTTTTTAGAGTACACAATTAATCTG
GAACACTAAATGCATTCACTTAAATGGTGGCTAGAACAGACTATGATTGATAAAGATTCTATCCAATGTG
TAACCGATATATTTTGCCTTGGTCTCAAGTTTTCCTTCAATCAAAATCAATGGTATCGGTGTATCGGTGA
CAGTATTTTCAAGGTTTTTTCGAAAAAGGAATTGTATGTTATATGGAACTTACCTGGAAATTTTGAAGGA
GGCATCTTTCCATCGATATTCTAGAAGCGCAGCAGCCCCAGCATCTGGAAACACAGCTTTAACTTTCTAG
CAATTAAATTTGGAGTGTACTTAGATAAATGATTAAATTAACTAATGAATAAACATCCTAAAAACTTTGA
ATCTTTTACAGTTTTCTTTGTTTACTTCTGTTGTGGATCTCACATCATTTATAATTTAAATTCTCACTAA
AAAAGATTAATAGTAATCTGATACACATGGTATATACAATATATTCCAACTGTTCTTCAAATTCAATTCT
TGATTAGTAAATTGTAAATAGGGCCTTAAAGTGATAATTCCATATCCTACTTACCTCACAATTTGTTTCC
TCAATGAGTGACTCCAAGAAAACTCTTGAGAGTTCCCAGAGTTCAGCCTGAGCACCTTCATCGTCCAGAA
ACTGAAGCTGGGGGATAAGGAGCTCAACCTGAAAAGAATATGCAAGAATACAGAGATGAGGGAGATAACA
AGATGAGTTCGGGTTCCAACTTTTTTATCAAAAAACCGAAGGATAAAGAGGAATCATAAGGTGCACCCAA
ACATCTAATATGATTTCAAACTTGGTCGCTAAGAATCAAGGGAGTCATAAAATCTCTCAAAACCAGCCTT
CGAGGGAGTAGGTTTTTGTTAGACCTCCGATCATTTACACCTATAAAAGGAAGAAATCCAGGAAGAGTAT
GGTGTGAATTACATAGAAGACCAAGGATGGAGAGGTTCTTGGGGGGACCCACCCCTGATGACGTATAAGG
AATATTTTGGAAATTAGCAAATCAGGTCATATTTTGGGTAAAGGCTGCTGAGGCTTTTGAGGAGAAGATT
CCAGCCTTCTCGAAGGAGCTGGAGAATAACCATGGCATTTACTTATGTTTTTTCTTTCTTATTTTACTGT
GCTGTCTTTTAGTGTATTCTGTGGATATTTTGTGAACTCATATGAAATTTATCATCAATAGAATCACAGC

AAAATACTGTCTCTGTTTCTCTATTTTCTTGGGATTTTTTACTGGGTTTCAAAGAAGGAATCCTAATAGT
CTTTTCGCAAAATAGTGGCAATGGGGCCCTCGATACCCCTGTTAAAGAAAGAAGAATATAGACTAGGAGA
GAGTGAGCCAATAAAAGCCTTTCAAAATCCAACCAGCTTTTAGACTGGGTGATTTTCTACTACTAGGTAT
AGAGGTGAAATTCGGTACATGAAACATGTAAAATTACAAAGTTTCTTATAGGAAGAATCTAACTTTACAC
CAACATACTATCCTGGCGTACGGCAGGCTGAAATTTGGATGTGTTGAGAATTTCAATTTCTTTTATAATG
TTTGTGCATATTTATAGGGCAGTGACATGAGGTTTCTTAAGGATTAGCATAAAGTAAAAGAGTATCATAC
TATTGCTCTTGCTCCTCCCGAAGACACAAACAGGCCAGCAGCTTCTCCAGATTGCCTAACTGCTCCCTCC
AAGTCTGAAGGCAAACAGCTGAAAATAAGAGTAAAAACATTGAGAAAGCCTTACACTCACCAACAAAATG
GATATTTCAATAGCACAAAAGATAAAAACAAGCAAAAATAAGCCAATACTGTTCCCCTTTTTGTTTCGTA
TCAATTCAGAACGTCCTCTTCTTACCAACGATATTAGGTACCTGGCTGATTTGAACAATTCTAACTCCTA
TCTGTTATCCCGTGGCTTAATCATCTTGTAAAAACATGAGAGCTTTGAATTCGACCAACTAAACAGAGAT
CGTAATAAAGAGAGAAAAACATTGGGAATGATATCATGTTGACCTTGGTGCATATCCTAATTGCAAAAGA
ATTGACCACATTTTCCTTGGCAATAGCCATATCTCTTTTTCTTTTTCCTTCTTATATATGTTACTGTTTG
GTTTAACATCTGTTACTACATTTGGTTGCCAACTAAATTCGTAAGGTAATAAATCTTAGACATGAAACCA
CCATGATTTGAACTCATTTCCTCTAACCCTTTATTATTTACATGGGCTTTAATGATCGCTAGATCAACCC
ACAGTGATTGACAATAACCATATTGATAATCAGACATTGAAACAAATGCAATTAGTTGAAGCCTTATTTC
GATTACTAAACTTAAAGTTAGTTCTTTCTTCTAACCGAGTATCAGAGCGTCAACTTCTTGTATTATGCTC
AGGGTCCTATTCCCCCTTTGTAAACCTACAAAGATTTAAAATCATTCAAATTTCCTATCCAGGAACTTGA
AGTGGCCATTAGAGAATAACTTATTACCATTATCACCTTTCCCTCCAAAAATCTCAATGGATCACTCCTT
ATGGATAACTGAATTACCCACTTCAAGACATCCACCATGATCAACAATAATTTAAAAGTATTTCAAGACT
TTAGGTCACAAATCCTCCATTAGCTTTAACTAACACTATTGGGTTTTGGCAAAATATCGGAAGAGTAGTT
GTTTACATATCAAGAAGCTAAGGTTCAAGGAAGTGATGAACTTCAAACACTATAAATAGAGTCTAATACT
TTAGTTTCTATTCATCCCAATTAGGAGTATTCTAAGCTTTAGTGTACTAATTCTAGTTAAATTCTCTAAT
TTGAAAGTGAAATTAAATAGCGTGAATAGTACACAAGCTTCAAGAGAGAACTCTTGCAATTGGGATAGGA
AAGAGAACTAATTTGAATAATCGTAACAGTTTGTAGCGGATTTCTATCTAGTGGACAGTAGTTTTTCTTC
AGGTTCACAGTTTTCCACGTAAATTTGACAAAATCCATATTTCCAAGCTCCTACTTCCAAGCTCTTCATT
ATCCATCATTTTTTCTCCATAGCTCTTAGTTATACTGAAATATCTTTCTAATTGTATTTAAGCACTTAAA
AGATTCATACCAAAACCGACCTGGAAATTTGGATGCAGAATGAAGTTCATCATTTTGAACCTAGAAGCTA
CTTATATTTCAATCAATTAAGAGCAAAAGAGAATCTTTCCATGATCAACTCTAAGATTCAGTAAACAAAT
ATTGAAGAGGAAGAGAAAAAGAGAAACCTGTCATCTTCTTCAACCTCCTGACTCAGCTGTTGTTGCAACG
ACGATGATGGATTAGCATCTAATACATCGTCTTGGGGAGTCTCGCCTTGAAATTTCTCGAACTTACCATG
AATTTTTGAAACGGACCCATTTTTGGTGGAGGAAATTTGGGGGAAGACAGTGAGGGAAGTTGGAGGATAA
GATAAGGGGAAATGTGTGGATGAGGTTGAATTGGATATTAGGAAGGAACAATGGGGGAAAGAAGGATTTG
GGAATGAGGAAGCCATGAACAAGGTGAGAGCTATATCTGCTCTAAGCTGTCAGCCATAGAATGAATTCAC
TCCAAGTTTATGAGAATATGGTGATGATTGCTAACCGCTAATCTTTCAAATTTAAGTCATCATTTCTTTT
ATTCGTCAAATATTTTGCTTTAAAAATTTAGTAATAATAAATAAATAAATGGAGTATTTTCAAAAATAGA
AAAATATCAAAACTATTTACAAAATAAATCAAAATTCATCCAACTCTTCACAGTCCATTCGAAACTTGCT
ATATCTTGTAAATAGTTTCATTCTTTTGTGCTATTCATAACAATTCCTCTAAATAAATCGTGCATGAGAT
TATGTTTGATTTTTTGAGAAAATTATTTTCAAAATATTTATGAGTGTAGTAAAATTTTTGAATCTATTAA
TGATAGGTATTGATAGACATCGATAGACTTTTATTGGTATCTAATCAATGTCACGTATAAACATTGCTAT
AAATCTCACTAATAGATATTAATACACTACAAGAGAAGGGGGTATTCCTGACGCCAAAAAACGTCGGTGA
AAATGAAAAGTACGTCGGGAAAGGATATCCCGACGTACCAAACGACGTCGGGAAGAATGTCGGGAGAAAT
GCGTCGGTCATCGCTGCGTCGGGAATGAGGTATTCCCGACGCAGCCTATGCCGACACATGTTTAGGCGTC
GGAAAAAGCTCTTTTCCGACGTTTTTTCTGACGTTTCTTTTCGTTGGGAATTCCTTTTTATATATATACT
TTAAATATTTTATTTCTACTTTTTTCCTTATAATATTTTGCTCAAACATTCACAATAATGTTCGGATATC
TCAAAAAAAATTTCCCGACGTTTTGGATTAAATTAAAACAAATTAAATATAAATTAAGAAATATACAAACA
CAAATTAAAAAATAGAATTAAAATAAATAATACAAATAAGTTCACTACAACAAAATATAGTTCTCAAAAT
AGAAAAAACATAAACATTAAGAAAAAGTCTCCCATCGAGGCGCGTACGCGCGTTATTCTACACATTCGGT
TCTAAAAATAGTATAAAAATAACTAAGTTTAGTACATAATCATATATTGCAAAAACTTAAGAATAATAGA
GATATATACGTACCGCAGAGCTAGGGATCATGTGGTGGTCCCTGTTGTGCACGAGTTAGTTCTTCTATCA
TCTTTTTCATACTTTCCACTTGTGAAGCTAATGCTTGGTGATTTCTATCTTGTACTTCAATCCGTTCCGA
AGCTTCATGAAGTTTAGCTTGTAATTCAATCTCTTTTTCTGTGGATTGCAAACAAGATGTCGACGAACTG
CTTGCACTCGCCGTTCTGTGGGCCTTCGGCTTGGGTCCCCAACCAAGGCCTTTTGAGTATCTCATCCTCA
GAGAGTGGCTGACTACCCAACACCTGATCGCATATCTCATCCTCAGAGAGTGGCTGACTACCCTCTGGGA
TAGGCTGGGATTGGAGTTCCAGCATTTGATTCTGCAACAAATTTAAAAACTATGTTAGGTAACGCGCAAA
AATATTTAATGAAAGTGGATAATTAATAAGGGCATAACTTACATGCGCATCCTCGGCGGCCTGCGACACG
AATGTCCCAGCTCGAACGTGTGTTTCTCGGAACAATTCCACACGATCGACCGGCTGCCCTCTTCTTTCAG
CGAGCTCATACTGTCGTTATAGAAACGACTTGGACTCGCTACTATGATTGTAAGGCTGCTTCTGTCTAGC
AGCCTTGTTCGTCCGTGATTGCTCCTGCATTAAAATAATTATATTGTTTATTTCTTATGCATATTAAAAC
TTGTTATCATAATTAATCATGACAAATACCTGGAATGCACGGCTGATATAATGGTCGCAGAGGAAGTGTC

AATCCTCATCACGTCCAACCAATGCATTTGGTGGGTTGGCACGAGCCTCCTACGGGTCGCTGTACTTTTT
GAAATGTCTATGACAGTCGGCCCGAGACTCTTTAAAGGTCGTGAGCATCTGATGCTCAACAAACCCGTTC
ATATTGCTTGATCATTGAAATCAAGCACAAACAATCACTACACATTACAAACATAACACATTAGATTGGT
TAAAGTTAGATATGTTTCAAATGAAATCATATATAAATGTGTAATGCATTTAATTACCTGGAGGTCGCCC
TTGACGACCTCAATGTATTCTCTCCCAACGTCCGCCCACTTAAGACAGCGGACGGGAAATGTCTTTCGCA
CGCACACGTCTATCGCCTGGCTGAAGCGAATGACGTGTGAAGAAATAGGCTTCTCCGCTCCAGGGGCGAT
CGTCATCGGAATGCGCCCATTTATTGCAATGTGGCGCTCTAACTCCAAGAGTCGAGACTGCGCACGTCTC
CTAGGAGTCGGAGTCGTTTGTTGAGAAGAAGACCCTGCTCAATAAATAGACAATAACATGTAAAGTTAGA
AACCCCTACATGAAATATTTGTAAGTTAAAATGAAGAAAATTCTTAGACTCACCCGTATTGTCGCCCACA
GATGACGACCCTCCCGCAATGTTATCTAAATCATCCTCAAACTGGAGGAACATATCGTCCGTCTCCATAA
AATTTGATCGTCGATATGACATAATGACTATGGACATAGAAAATAAACATTCAATAACCAAATACAAACA
CATAGCTAGAATCAAAATATATAAACTAGATATAAATATGTGCGTACGTGGTTTATGTAGCCACCTAATT
TTATCCTACATTTTTTTTTATTATTCTTTAAATTATTAATTGTAGCAATGGTTAAGATATTTCATTAATT
TATTTTGTTAAAAAAAGAAAAAGGTAAAATCTTTTTGTAATAATATGAAATCTTATGTTTTTTTCTTATC
TTTTTATTAAAATGGAAAATAAAGTCATTAATGAGAATATCTATTATTTTAAAAAAATTCAAATCATTTT
TTTGACTTATCACTTTAGGAAAAAAAAAAACAAATTAATTTATTTATACAAAATCTCTTTTGATTTATCAT
ATTAGGAAAAACAAGATAATTTATTTTATACAAAAAATTTTAATACCATATTTGACTTATCTTTATCACT
TTAGGAAAAAAACAAAAAATTTATTTTGGACAAAATCTTTCAATATCATATTTTTACTATTTTAGAAAAA
AGAAAATTAATAAAATTACATAGTATCTAATTTGATTTTATACTTATTAAATTTTCCTAATTTGTGGCAC
ACCCCGGGTCTATAAATAGGATCCTTTGTCATTTGGAAAAAGGGGAGAAGAAATTGTTTAGAGAAATTCT
TGATGAAAAAAATTTGTTTTAGAGAGAATCTCTACGGAAAAAAAAGTATTTAAAGAAAATTTGAAGAAACGC
ATTCCTTTGCAAAAGGTGGGTCATTAGCTTTTTCGCTTTATTGTTTTTATTTTCATATCGTTTTTTTTTA
CCCTAATTCTAATCCAAGAAAAAACTTAGTTGGAGGTTGTATTAGGTAGGGTTTATTCCCAATGATTCGC
ACCTCATACAACAAGTAATTTTCTTCGGGATTGAATCCTTATTTTTGTTCTTCTCTTTTCAAATGTAGAG
AGAGAGAAAAAAAAATGTATAAGAATTTTTTTTTTTCATTTTTACTACCTTTTTACCATCATGCTAAAAGA
AAAGGTCTTTTAAAATTTTTCTTCTCTCTAAACAATAGAGAAAAAGAAAAAGTTATAAACAAAAAAAATT
CTATTATTATATTTTTTTTATTATTATTATTATTATTATTATTATTATTGGATATAATTTTTTTTTCTTTT
AGGGTGGCGGCAAACCTTGCCGTCACCCGTCTGTTTCTTTTTTTTCTTTCTTTTTTTAATTAATTTATTT
ACTTATTTAAATTTAATTTTACTTGTAATTTTATTAATATTATTTTATTATATATATATATATTTAACCT
TCGGTTTGTTTTTTTATTTTTATATATAACTTCATTTATTTAGTTTTTTTTTATATTTATGAATTTTTAA
AACTTTTTGCTATTTTTATTCTTATACCATTATCATTATTTCCTTTCATAATTTTTTTTATCTCTTTTAA
CTTATTATCATCCCTATGTCCTCTTTTCTTCACTTATTTATTTAATTTCTTTTTTACATATTCATTTGTT
TATTATTTCATTTAGTTCTTTTTTTCATTTTTTTTCTTATTTTCTTTATCGTTATTATGTATCTTTTCTT
TTCCTTTTCTTTTAATTTATTTTTTATTATTATTGTTATATTTAGTATATGCATACATTGATATCCTAAA
TTATTTGTCTAATTTATTGTGTGGTATATTTTTTATTTCTATTTGACTTTCATTAAAAAATTTCTTGAAAA
TTTTAAGATACTTTTAATCATAATTGTATTTAATTTTGAAATCTTTTTTTTTTTTAATTTTTTCTCTTTA
AACCATTTAGAATTTTTTTTTTTTGCTTTAAAATTATTTATTTTAAAATTCAAAATTAAATAGATATTTC
ATAAGGTTTCCTAATCTACATTTTTTTATAATAACCACGCCGATTTCGATAAAAATAACCTACGATGGAA
TCTAGAAATTTCTGGGAGTCCGTTATTTCTAGATTCTTGAGGTGAGGGATCAATATCTTAGGGAGTCCGA
GATTTGATCCCAAGAGAAAATATATTTAATTAATGTTTTAAAAAAAAACTTTATTCGAAGACTAGCCTAT
GATAGAATTTGAGAAATTGTAACAATTTCTCATTCTCTTAAGGTGAGGATTTTTCCCCAATATAGTCTAA
TTAATGGGTGTATCCCACTTATTTTATGGGATCATTGCTCCAAATATTGGAGTGGTGAGCAATGAAATAA
GACATAGTTCTTTTTCTAAAAAAATAAATTTTATTCAAGACATTAAATTTGGCCACCGTTTTCTAAAACG
GGTGTTATGGGGTGCTAACACCTTCCCCGTACACAAATGACTCCCGAACTCAACTCTAAATTTTTTCGTA
GACCAGTTTTTATTTTATTTAAAATGATTCACTTTATTTCGGTGTCCAATCACACCGTAAAAAAGATTGG
TGGCGACTCCTCTTTTTTTTTTTAAAAATAAACCTTTTTAAGGATGTCGGGCGCTCCGCGTCGTCTCGGG
TACGTGGCGACAGTTTAATTTGTCACCATAATTCCTCATCGCTTGCATGTGACAAGTGTTCATCCACATC
CTAATTATTAACAACAAATACTTCAAGCTATCCTACTACCACCCCTTGAAATCAACAAATTGAAAACAAA
AAAGCCTACCATAACTGCAGGATAGCCATCCTAAACATAAACAAATTCAAAACAAAAAAGCTACCATAAC
TGCAGGATAGCCATCTCAAATCAACAATAAAAATATTCAACACAAACAGGCTACCATAACTGTAGGATAG
CCAAAACAAATCTTAACACACATATTACATTCCCAATTCAATTTAACTATTAACTCCCCCCTCCCCTCCA
ATTTCAATTTTCAATTCAACTATAAATCCCCCCATTTCAATTTTCAATTTAACTTAAACCCCCCTCCGCC
CAATTCAACTTGCCAGCGCGTTCGTGATGCATCGGAAAAGAGTGTTTTCCCAACGCATCTCTCGACGCGT
TGTTGACGGCGTCGGAAAAACCTTTATTCCTGACGTTCTTTATGCCGACGTCCTTTTCTGCGTCGGGAAT
GCTCCATTTTCTTGTAGTGATATAAGTCTATTAGTATTTATCAAAATCTATTATTGATAAATTTTAAAAA
TTTCTACATTTTGTAAATATTTTGGTTTATTTTATTATAAAATTACTTGCATAAATGACAAGAGAATCTA
AAATAATAGTATACATAGTACAAGGGTAAAATAATTGCATATATATCACAAAAAATAATAAAAGACTAAA
ATACCCACATCCAACTACCATTTTGGACTTTTTTTTTATTGAATTTCTCACTTTAAAAACTATCACTGGTA
ATTGCTATTGGTAATATATGCTATTACTGATAGCTTTCAATTTGAGAAATTAATGATTATCTCACACTTT
TCTTCTTGATTTTTTTATGTTGAAAGTTATCACTAATGACTACAATTAGTGAATATCACTAATAGCTGTT

ATCGGTTATTTTCTCATCATTAGGAATATTTTTTCCTATTTTTCTGAAATTGAAAGCTATCATTGATAGC
AATTAATAGGTGTTGTACGTTGCCATCATTGATAGATATTGTTATCACTATAATAGCTTTTAATTTAAAA
AATATGTTGTAAGTTGATATAATTGATAACGACTATCAACAATAAATTTTACCAATGATAGCCACTAATG
GGTAGAGTAACTTTTAATTTGAGAAATATGGTATCAATTGTTATAGTCAGCTGATGATAACTTCTATCAG
TGGTAGCTAATTTCAATTTAAGAAATGTGATATTAGTTGCTATTACTAATATCTTCTATCATCGATATTG
CTATTAATTGATATTCCTAATAGCTATTATAAGTAATTTTTCTATATTGCTATCCACATAAAATGATATC
ACTAAGATTATCTATCAATGATATATTGCATTAGAAAAATATCACTAATAGCAACTACAAGAGATATCAT
TAGAAGGATATTTTTTTTACAAGTGATATCTCTTTTGGTAAGCATATCAACTTTGAATTGGTAAGGTTTA
ATTAAGCTATCAATTGATATTTTGTATAGCTACTATCAATGGTATTCTTCTATTATTCCTATCACCGATG
ACATCTATGAGTAATATCCCAAATATGCTACTATATATCAATGATATCAATGTATGCTACTCTAAAGTGA
TATTGTGGTTGTTTGTTTTCAGCATAAATGAACAAATTAGTAAAGAGATTGAATTAGCATGCTATCGGTA
ATAGATTTGAATCACTAATAACATACTATTCATATGATAATTTCTATTGTTGATAGTATAAATGAGATTG
ATGTAGCTTCAACTTTTGTTGAAATAAGTTATTGATTTTTAGGTTATGCTATCAACATTTATATCATTTA
GAATTTCAAAGTTTAAGTTATCAGTGATAAATTTATAAAAAAACATATTGACATATTACATACAATGTCA
TTGATAAAAGTCATCACTGATAGTATATGATATATATACTGACATACTATCAGTGATAAACATATAGACA
TACTATCAATGATGACTTCTATCATTGACATCAATTATAGAAGTTATCACTAATAGCATAAGAGCAAGGC
AAGTGGATGGCAAAATTGACATTTTAAAAAAAAATTTACCATATGACTAGTAAAAATTTGCCATTTTTTG
CAAATAATGTTCCCTCGTATGCTATAGATTATATTTATTTTTTAATTTGTGCTATACGGTGCAATTTTTC
CTATTTTTATATATGATATGATTATATGGTGTTTAATGGCACTTAAGGGATGTTCTTGTCCAACGCTTAT
TTCAAGACACTCTGCTGCTTGTCACATTGCAAAGAAACGTTGCACATAACATATAACGATGAAACAGTGT
GATATGTGGAATGGAAGGCATGAGAGACATTGCAATTGCCAACAACTCTCCATGCAACAGATCACTTAAC
TGAATTGTTAAATTTTTAGATGATCCACCCTTAAATTAATTTTCCAAGTTTGTGTTGCAAATGTTTAAAT
TACTTACTAGTGTCTTTAGATCAAATGGTAGAAGGGGGGAAAGTTTTACATTTGTTTAAACCACAGCTAA
ATATTTATTCATCTATACCCCTATGTGTACTATATGTTGCAATCTACAAACAGAGCCATAAAATGAAATT
GAGATCAAGAACTATTTTATTATTCCATGTCTAAGCATATATAAAGAATCCTAAAGATATTTATCTAAAG
TTGACAAGAATTTATTTCAAAATAACAGTTTATCATTAACTTCTTTTCTTTTAACATGAAGATTAAAATG
TAATATGGTGAGAAGTAAAATAATAATAATATCTATGGTGTATTGCACATCTACCAATATCGAACTTTTC
ATCCAACAATTAATATTTTGGTCTTCAGAGGATGCGCCAACCTTGCAAAGCTTCCAAGCGTCCTCAGGTT
ATTAAAGTCTCTTGTAGATTTAAATCTTTCTTGGTGTAGTAAGCTTAAAAGCTTGCCAACAATTGATGAA
AACATGAAATCTTTAAGGTTCTGGATTTGAGTTTTATTCCAGTAAAGGAGCTATCTTCATCAACTGGATA
TCTTACCGAGCTCCATCAGTATATTAGTCCTTGCAGTTTTTTACAAAGAGCATGAACCTGTAGATTGAAG
AACTTGGTTACGTGAACTGATGCATCCAAATTCATCAAGCTTTGATGGGTCAATTTCGCAAAATTACCCA
AGTACCTTTTCCTTCTCGAAGCTCGAGAACTCTTGCTTTGAAATTGAGAAACTGATTCATCTGAATCCCA
AACGACAAAAAGCTAACAAAGTAGCAGTTAGGTTGTTGGAAGAACAGTCGAGCATGTTATTGCATAATTG
TATCCCTTCCGAAAATTTGGAGCGGCAAAGAAATCAAGATACGCATTGAGAAAAGGCATATCCATGGCAT
TCACCGCAGGAAATCAGATGGCAAAGAAGATCGAAAAGCCAAAGCGAGCATAGCTAATCAGCAGTAATAT
ATTGGCATCATCTTAGAAGTGGGATGTTTGAATCATCAACCCTCAATTGTTGTACTAAAAAAAGAATACT
ATTGGAAATGTTTGCATATCTGCCCAAGAAAGAAAAAAGTCTACAAAAATTTCTTCAACCTTTAAATTTG
GAGTTTACTTACTTACTGTTAATTTTTTAAAAAACAAAAGTCAAATTTTAAAAACTAAAATTATTATTAA
AAACTTGCTTTTACTTATAGAATTTGGCTAAAGTTTTGTTTTAAAAAATCTTAAACTAACAAGCAAATGG
TTATTAAAGGAAGTGTTAGTTTTTTAATTTCAGTTTTCTAAAAAACAAAAACCATACATGTGGACCAAAT
ATCAACACTACAAAGAGACTTCCCAACTTCTTGACTTTTATGTTCCCTTCAATTTAATTACGATGTTGAT
GGAAAAATGGGAGTGATAGAGAAAAGTCTTTGGATGAAATTGGAAGCTGTACTGTACCAATTTAATTCAA
AATACGATGCCCCACTTCTTTACTGCGATGCCAACTGAATCAAATTATTGAGAGTTGAAGTCTTTGATTT
TTTCTCTTTATCTAATACACCATTCTTCAATTTTTGCTCTGCATTATTCTTCACAAATATTGCTATCGGT
GTTTTCGATTTCTCTTTTCTTTAGTCTGCGTGTTTCTTTCTGATGGATTCTTCTACCGTTGCAACAGAAT
CACCGACTTTCAAATGGAGTTATGATGTGTTTTTGAGTTTCAGGGGAGAGGATACTCGCACAAATTTCAC
CAGTCATCTTGATATGGCCTTGCGTCAAAAGGGTGTCAACGTCTTCATAGACGACAAGCTCGAAAGGGGT
GAACAAATTTCTGAAACCCTTTTCAAATCTATACAAGAAGCTTTAATTTCTATTGTTACATTCTCTCAAA
ATTATGCATCTTCTTCGTGGTGTTTGGATGAATTGGTGAAAATAATTGAGTGTAAGAAATCCAAGGGCCA
GATTGTTTTGCCAATTTTCTATAAGGTGGATCCATTGGATATACGAAAACAGACTGGTCGTTTCAGAGAA
GCATTGGTCAAACATATGCCAAAGTTCCAAACAAAGACCCAAATTTGGAGGGAAGCTTTAACTACTATGG
CTAACTTGTCTGGTTGGGATCTAGGAACTAGGTATATATTTTTATCGACATTTTGTCTTTTTCTATTCCA
AATCTCATTTTTAAGCCCTGTACGTACGTACCTATGTATTTATGTATGTATGTTAATGAACACATTGACA
AAATTTTAATCTTAGAAAATTCGTTCTCTCTCAAAATGTAATTTTAATTAACTGTGTTCATCGTCTTATT
ATTTTGGTATGATTATGCACTTAACAACAGGAAGGAGGCTGATCTTATTGGAGATGTTAAAAAAGTGTTG
TCTACATTAAATCGCACTTGCATGTCCTTATATGTAGCTAAGTTTCCGGTTGGAATTGATTCTAAATTAG
AATATATGAAGCTTCGTTCAAATAATCTTTTGGAAAAAAGCAACAAATTCCATAATCGGACACAACATGA
GCATGAGTCTGATACTGGTGTTTACATGGTGGGGATATATGGCATATTGGAGGTATTGGTAAGACAACTT
TGGCTAAAGCTTTATACAACAAAATTGCTAGCCAATTTGAAGGTTGTTGCTTTCTATCAAATGTTAGAGA

AGCTTCAAAGCGATTCAATGGCCTTGCTCAACTACAAGAAAGCCTACTCTATGAGATCCTAATGGATGAT
TTGAAGGTTGTCAACCTTGATAGAGGAATTAACATCATAAGGAATAGACTTCATTCAAAGAAAGTTTTTA
TAGTTCTCGATGATGTAGATAAACTTGAGCAATTAGAAGCATTGGTTGGTGGGCGTGATTGGTTTGGCCA
AGGCAGTAGAATCATTGTGACCACAAGGAATAAACATTTACTTAATAGCCATGGCTTTGATGAAATGCAC
AATGTTCGAGGATTGAATCAAGACAAAGCTATTGAGCTTTTTAGTTGGCATGCTTTCAAGAAATGTTGTC
CATCAAGTAATTGTGTGGACCTTTCAAAACGTGCTACGAGTTATTGTAAAGGCCATCCTTTGACTCTCGT
TGTTTTGGGTTCATTCCTTTGTACCAGAGATCAAGCAAAATGGAGTAGTATATTAGATGAATTTGAAAAC
TCATTGAACAAAGATATTAGAGATATTCTTCGGTTAAGTTTTGATGGACTTGAAGACAAAGTAAAGGATA
TCTTTCTTGATATTTCTTGTTTACTTGTGGGAGAGAAAGTTAAGTACGTTAAGAATATGTTGAGTGCATG
CCATGTAAATCTAGATTTTGGAATTATAGTACTCATGGATCTTTCACTTATTACGATTGAAAATAACAAA
GTGCAAATGCATGATTTAATACGACAAATGGGTCATAAAATAATCAATGATGAATCTTCTGAGCTTGGAA
AGCGAAGTAGATTGTGGTTGGTGCAGGACATTTGGGATATATTTTTCCATAATTCAGTGAGTAACTCTTA
GCTAAAGTATCTATAATTTAATAATTATTTCTAAGGCTTCACGTGATCAACAAAGGTTCGCCGAAGGTTT
GTTAAATAACTAATTAAGTATATGACTTTGCAGGGAACAGACACAATCAAAACCATAAAATTGGACTTGC
CTAATCCCACAATGCTAAATGTGGATTCACGAGGTTTTAGAAACTTGAAAAATATGAGACTGCTTATAGT
TCGATATGCAATATTTTCTTCAAAGATTGAGTACCTACCTAATAGCTTGAAGTGGATTAAGTGGCATGGA
TTTGCTCAACCATCTTTGCCTTCACGCTTGATTATGAAAAATCTTGTTGGACTAGATTTGCAGCATAGCT
TCATCAAAAAATTTGGGAAAAGACTTGAGGTAAATTGTATTTCTATAGCATTAATGGGTAGCTTACATTT
TTAAAAATTTCACTAATATGTAGTTTTATAATAATAGTTTTATCATATGATACCTCATTTCTGAAAAAAG
AAAATGAGCAAATCATTGATTAACTTAAACAATAGTAATAACTCATTCCTATTTTTCTTTCCCCTTCTTT
TTTTTCTTCTTCTTTTGGTCTATTGTTTTATTTCAACATGGTATAGTGGATTAAAAATGATGGAAAATAC
TTCATGTGATCTGAAGCCATACTTAGTTGGTGAGCTGTTTTCAGACCGATACAAATTTAAGAATAATCCA
GTGTGTATAGAAATTCACAATTAGCTTAGGATTATGTATCTCATTGCATGGTCCAATCTTATATAATCTC
ATTTCATAGCATGCCCCCCACACGTATGTGTCTACATAAGCATCTCAAGATCACTCCACTTGTACTATAT
ATTCAAAGTAGGTTGCATCCATAGTGTCTCCAGAATAAAGCATCCGACCTTATTCCTATATTATAAATCA
TTTAGACTATTATTCGAACTTGATACATGTTTATATATCTACGTATTAAAATACTTATACTATAACCAAG
GAACCTTGCTACTGAACTCTGTGGGCGAAAACATCAAAGGGGTGAAAATGAATGACAACCTAAATCTGAC
ACACTTACTTTTTGCTGATGATATTCTGCTTTTTGTAGAAGATGATGAGCACTCCCTACAAAATTTAAAG
AATATCATCAATCTCTTCCAGCTAGCATCAGGGTTGAATATCAATCTCAATAAGTCCACCATCTCCCCTA
TAAATATTGATGCTGCAAGAACCGATCAGATAGCTTCTCAATGGGGAATTACTACTAAATTTTTCCAATC
AACTACCTTGGAGTCCCTCTCGGAGGCAAACAAACAACAAAGGCTTTTTGGAAGAACATTGATGAAAAGA
TAAGCAAAAAACTTGCCAGCTGGAAATATTCCATGTTATCCAAAGGTGGAAAAATTACCTTGATTAAATC
TACTTTGGCTAACCTTCCTACTTATCAATTATCAATTTTCAAAGCCCCTGTATCAACCTGCAAAAGCATT
GAGAAATCTTGGAGGAATTTCTTCTGGAAGAACCCATCCGAGGCACACAAACTGCACCTGGTTAGTTGGG
CTAAAATTACTTCTCCAAAAGAGAGAGGGGGGCTGGGCATTAGTCGACTGAAAGATACTAATTTTGCTCT
TCTAACAAAATGGCTTTGGAGATACATCCATGAAGACTCCCCCCTATGGAAGAAAATTATAAATGCAAAA
TACAGAAGCCAATCTAAAGGGGACATCCCATGTGTTTGCAATCATAGCAGCAGCCGCTCCCCTTGGTTTT
CCATCTGCAAAGGATTGGCTTGGTTTCAAAGACATGTTTCCTGGAAAATTAAAAATGGTAGGAACTTCTC
TTTTTGGCATAGCCACTGGCATCAAAATAGTCCTCTTTCATTACACTACCCCAGATTATTTGCTCTCTCT
ACAATCTAGGACAGCTCCATAAAAGATATGTGGAACACTACTTTGATGGATTGGGATCTTAAACCAAGAA
GACAGTTAAGGGATTGGGAACATCCTCTGTGGGCTGAGTTAAAAAACTCTCTAAATGCCAGTTTTTGCGA
AAATGGCAGAGACTCTCCAACTTGGGTCTTAAACTCTGATGGCTTTTACTCTGTTGCTTCGGTTAAGAAA
GCTCTTCAACAACCTGATCAAGGTATTTTAGACCTCCAAAACCAAAACATTTACAAAAATCTTTGGAAGT
CCAGCATCCCAAAGAAATGCAATTTTTTTATCTGGACTCTGCTCTATGATAGTGTTAATACCGCCGACCA
ACTTACAAAGAGAATGCCAAATCTCTGTTCTAGACCAAGCTGGTGTGTGATGTGTAAAAGGAATGACGAG
GACAGAATCCACCTCTTTATCCTTTGCCCCATTGCAAAGTCCATCTGGAAATTAATATCATCCCACTTAA
ACAGCAATGTAAACTGTCTCAGTCCAAAGGATCTATGTATTACCATGTGCAACTGGAAACAGAAAACCAA
AAAGAATATCATTCTCTTCAATACCTATGCCTCTGCCCTCTGGAACATATGGTTGGAGAGGAATGCCCGT
ATCTTCAATGGGAAAGAAAAAACAGTTGCTGATTTATGGGAAGATATAAAAACTCTTGCAGGACTGTGGA
CAAGTAGATCTTCACTGTTTACAAATTATCAAGCTACTTCCATAGCCCTAAACCTTAATGCTTTTACTTA
GCTTTTTACTTTGCTTTCTGTCTGTACTTTGTTATCTCTGGGGCTGTTCTCAAGCCCTTTGTCTTTCGTT
TTGGGCTCTCCTGCTTCCGTGTACTGTTAACGTTATATTATTAATGAAGCGGGTATGATGATGGTGCTAA
GGGGGTGTCCACCTAGTGGAGATGCCCGGGTGCACCTACTGACCCACCGTTTCTTGTTCAAAAAAAAAAA
AAAAACCAAGGAACCTTGTTTATTGAATTATGAGTCTTATCATAAGCATAAATATCTTATTCAATAACAA
TTTATTAAATAATACTTCAACAATTTTGTAATAGAATATTTTCATATTCACAAATTACGAGTATTTGGAT
ATAAAACCCAACATAATCTTGATATGGATCGAGTTTATCACTGATTTACGTTTATGTATGAAGTTTTATT
ATTTTTCCTAAACATTGATTAATGGTTATTATAGATTTGTTACATATATATATATATATATATATATATA
TATATTNNNNNNNNNNNNNNNNNNGTTACTATATATATATATATATATATATATATATATATATTCTATTTT
ATCAAAATTTTGTTTGGTATGCTGGTTACATACCCCTTTTCCTTTTGGTTTCTTTTCCAATGTGTTTTAG
GATTGTGAAAGGTTGAAGCATGTTGATCTTTAGCTACTCTACTTTATTAGAGAAAATTCCTGATTTCTCT

GCCGCATCAAATCTTGAAGAATTGTATCTTATCAATTGCACAAATTTAAAAATGATAGATAAGTCTGTTT
TTTCTCTTCATAAGCTTACTATCCTAAACCTTGATGGTTGTTCTAAACCTAAAGAGCATCCAACAAGCTA
CTACAAGTTAAGGTCTCTTCAATATTTGAATCTCTCGTACTGCAAAAAAACTTGAGAAAATTCCAGACTT
ATCTGCAGCATCAAACCTTAAGAGCTTGTATCTCCAAGAATGCACAAATTTAAGAGGGATTCATGAATCT
ATTGGATCTTTGGATATGCTTGTTACCATGGACCTTAGACGATGCACTAACCTTGCAAAGCTTCCGACCT
ATCTCAAGTTGAAGTCTCTTCGATATTTAGGACTTTCCGAGTGTTGTAAGCTTGAAAGCTTCCCAACAAT
TGCTGAAAACATGAAATCTTTATGGGGATTGGATTTGGATTTTACTGCCATAAAAGAGTTACCTTCATCA
ATTGGATATCTTACTCAGCTCTATAGATTAAACCTTACGGGTTGCACAAACCTCATCTCCCTTCCCAATA
CAATTTATTTGTTAAGGAATCTTGAGAAACTTCTTCTTAGTGGGTGTTCTAGATTTGGAATGTTTCCCCA
TAAATGGGACCCAACCATTCAACCAGTATGGTCTCCTACAAAAATGATGGAAAGAGCTTCATGGAGCTTA
GAATTTCCCCATTTACCAATTCAAAATGAAAGTTTATGTTCCCATTTCACTTTTTTGGATCTTCAATCTT
GCAACTTATCAAATGCAAAATTTTTAGAAATTTTATGTGATCTTGCCCCTTTCTTATCTGATCTACGCTT
GTCAAAAAACAAATTCTCTAGTTTACCTTCATGTGTCCACAAGTTAATATCTTTGGGGAATCTTGAACTA
AGGAATTGCAAGCTTCTGCAAGAAATCCCAAACCTTCCCCAAAATATACAAAATTTGGATGCTGGTGGTT
GCGAATCGTTGGCTAGAAGTCCAGATAACATTGTGGATATAATATCAAAAAAACAGGTTCGGGTCTCTTT
CCATTCAATCTATTCTTTGTCTTGTAAACAATTTTATGCATTATGAGTTCTTATTCTGTAGGACCCTACA
TTGGGTGAGATTTCAAGAGAGTTTTTGCTAATGGGGACTGATGTTCCAAAATGGTTCAGCTATAAGACTA
CAACAAATTGGATGAGTGCTAGCTTTCGTCACTATCCAGACATGGCACGAACTTTGGCTGCTTGTGTTAG
TTTCAAAGTGAATGGAAGTTCATCTAAACGGGGGGCCCTAATTTCATGCAATATATTCATATGCAATAGA
CTTCATTGTTCTTTTTCAAGACCATTTCTTCCATTAAAATCAGAATATATGTGGTTGATAACAACTTCTC
TAGCATGGGGATCAATGGAGGTGCAAGATTGGAATAAAGTTTTGGTCTGGTTTGAGGTTGTTGATGCAGA
TGATGAGGTGAAAGTAAGTATAAGACGCTGTGGCGTTCATGTCACTGAAGAGCTCCATGGGATACAAACG
GATGTCAAGTGGCCAGTGGTAAATTATGCTGATTTTTATCAAGTGGAGAAATTGCAAAATCTGTAAGTGA
GCCTGCATCATTCCCAGTTTTATTGATTGTTTACTTGTTATTTGTTTAGTTTTATAGGGTGAAACTATGA
TAGATCCCAAAGGGAGAGACGTGGGAGAGAGATATACGCTTTTTTGTTTTTTTTTTTTATTTTTTATCTT
TTCTACGCAACTCTCTATGCCAGGGGTTGGTGGATTTCTTGCTGGATCGAGTAATCTATCTGATGTATGT
GTTAAGGAGACTGAAATCTCTACAAATTTATCTCCATAATTTAAGTTTGATATTATCCATTTTGGACATA
AGATTTTATGACTTTGTTTTGTGTCTACCCAAAAGATTTCGTAGCAATGAAGTTGTTGTCCGTCTCTTGT
AGGCTCAGATCTTTTCGTTTCCTAGATAACTTGGAACTCCAATTGCACTTCAATCAATATAAAAATAAAC
GCAAAGTTTTGAAGAAGAATTATAATATAGAAATGAAACATGGAGGGTTTCTCTTGTTGCTGTTTCCCTC
TTGTTGTTGTTTCTCATTCGGCCTTTATAGGTTCTATGAAAATAGCTAGTATCCAAAGGAATTAGGAACG
ACTACAAAAAAGAAGCAAACTGTAGAACACTAATCTTTCATATCGGAAAATTGCTAGGAGGTAATGTTGT
CTAATTAAGCTATATAAAAGGACTTCTATATGACTTTGGTTCTAGACCATCATAATTTAGAAATATTCTT
AGCATGATAAACTAAATTAGAGTTGTGTGAGTGCTATAAAGCTTTAAGATAGTGTTTTCACAATTTAAGT
CAAAGAGAGAATTCTACGAGTGATTGTATTAATTTTCACCTATTGGATTTTTCTTTAGTTTTAGAGATTA
GTAAACAGTCGTTTTTCTACATATTCATCAAGGGAAGCAGTGGAGAAGGAATTTGAAACTATGAGTTTTG
GTTTTGATTGTGTATTATATGATATCTGTTTTTTTGTGGCCCAGGGATATTGAGGATGTTCTTGTCAAAC
GCTTCTTTCAAGAACTCTCCTGCTTGTCAAATTCCCAAGCAATGTTGCATGCAGCAAGTTATGATCCAGA
AGCAGTGGTTGATTCCAACATACAACCTATGGTATTTCCGTTGCACCTAACATATAATGGCGATACAGTG
ATACGTGGAATGGAAGGCATGGGGGAGACTACAGTGGCCAACTGTTTATGCAACAAATTTAATTGGATAA
AGGGCCACCCTTACAGAGAAGCGTTAGATAATTCTACAAGCTGTTTCTTGGTCCGAGGAAGAAAGCTGCA
TTACTTATCCTACTTGTCCCCCCGTAAGCATGGAACCAATATCACAACCCATACAGTATCCTCCAAAAGC
TATTACATAACCTTTGAAAATTTGGATTATAGAGAATTTAGAAATCTACGTGCATGGGTTAAAGCACAGC
GTCGTTGGATTAGATTTGATGTTGGGAGTCATGATTTTGTAGTAAATAAGTATCATTTTCTAAATAAAAG
AGTTGATACATCCTTATGGTAGCAGTCATGGGGGTTGTGGAAGCTCTTGATCGATGCAAAATGTAACAGT
CTGTCGATTCTTTTTTATGTTTTCAGATTCGTAACAGATGCGCGTTCGGTAGACGATCTGAATTCTGTTT
TAAAAAATTATTTTTAGATTATGCGATTCAAATAGAGAAAATTCTAAGAACAATTTTATTGTATTCAGAA
TTTTAGTTTTACATTTTTTAGAAAAGGAGACAAATGGTCTGAAGAGAGCTTTAGGGGGAAACCCAAGATG
GGTTCCTAAAGCTATTCTAAGACACTGATTTATTAATAAGAGGAAAGAGCAATTACAAAGCCGGGCTAGT
ATATAACAATATTAAGTATGATGTTATTTAAAGAAAAATATTTGTGGCATCTAACTCCTTGAATTGATAA
TTTAGGATAGAGAAATTTGTCTTCTGTGACAAGTCGGTTGAATACACAAAAATATCAATTTTAATTTATA
AATAATAAATTTGAAAATTGAAGATTTTTTAGGGAGTCACATCAAAAGAAAAAAAGTACATAAAAAAAAG
AGTGAACATTGACTTCTAAATTTAACCAACGACTTTGATTAATTAGGAAGCACATGTAAGATAATACAGA
AAAGAAAACTTCAAAAGCATTGCTTTGTTACTGTCACAGATTATTATTATTTTCCTTTTCTCTGCGTTTT
GTTTTTGTGGGAGCATGGGTTCTTCTGTTGTTCGAGTTGGATCATCTTTTTCTGATCCTAACTGCAATTA
TGATTATGATGTGTTTTTTAGTTTTAGAGGAGAGGATACTCGCTCCAATTTCATCAGTCATCTTCATATG
GCCTTGCGTCTAAAAGAAGTCAATGTTTTCATAGACGACAAACTCAAAAGGGGTGAGCAAATTTCTGAGT
CTCTTCTTAAATCTATAGAGCGATCTAGACTTTCCCTCGTTATTTTCTCAAAAGATTATGCATCTTCAAC
TTGGTGTTTGGATGAACTGGTGAAAATAATTGAGTGTAAGAAATCCAAAGGACAAGCAGTTTTGCCGGTG
TTCTACAAGGTGGATCCGTCTGAAGTTCGAAAACAAACCGATTGGTTTGGGGAAGCATTGGCCAAACATG

AAGCTAATAAGTTATTGACCAACAAGATTCAACCATGGAAGGAAGCTTTGACTTTTGCTGCTGGTTTGTC
TGGTTGGGATCTAGCAAATAGGTATTTTTTTTTTTAATCTTCCAAGACTCATTGTCCAAGTGAAGTTTAA
ATTTATCAGCTACTATTTTCGATTTTCATTTTTATGATGTTACTTTACAACAGCAAGGATGAGGCTGAAC
TTATCCAAGAAATTGTTAAACGAGTATTGTCTATAGTAAATCCAATGCAATTACTACATGTAGCCAAACA
TCCAGTTGGAGTTAATTCTCGACTAAGGAAAATTGAGGAGTTGGTCTCTCATATTGGGTTCGAGGGTGTT
AACATGGTGGGGATGTATGGCATTGGAGGCATTGGTAAGACCACTTTGGCTAAGGCTTTGTACAATAAAA
TTGCTACCCAATTTGAAGGATCCTGCTTTCTACTAGATGTTAGACGAGAAGCTTCAAAGCATGGGCTCAT
TCAACTACAGAAAACCTTACTCAATGAGATCTTAAAGGAGGATTTGAAGGTTGTCAATTGTGATAAAGGA
ATTAACATCATAAGGAGTAGACTGTGTTCAAAGAAAGTTCTTATAGTTCTTGACGATGTGGATCATCGTG
ATCAATTAGAAGCATTGGTTGGTGAGCGCGATTGGTTTTGTCAAGGTAGTAAAATCATTGTGACGACAAG
GAATAAACATTTACTTTCTAGTCATGGTTTTGATGAAATACACAATATTCTAGGATTGAATGAAGACAAA
GCTATTGAGCTTTTTAGTTGGCATGCTTTCAAGAAAAATCATCCATCAAGTAATTATTTCGACCTTTCAG
AACGTGTTACAAGTTATTGTAAAGGTCATCCTTTGGCTCTCGTTGTTTTGGGTTCTTTCCTTTGTAACAG
AGATCAAGTAGAATGGTGTAGTATTTTAGATGAATTTGAAAACTCTTTGAACAAAGATATCAAAGATATT
CTTCAATTAAGTTTTGATGGGCTCGAAGACAAAGTAAAGGATATCTTTCTTGATATTTCTTGTTTACTTG
TGGGAGAGAAAGTTGAGTACGTTAAGGATACGTTGAGTGCATGCCATGTAAATCTAGATTTTGGAATTAT
AGTACTCATGGATCTTTCACTTATTACGATTGAAAATGACCAAGTGCAAATGCATGATTTAATAAAACAG
ATGGGTCATAAAATAGTTTGTGGTGAATCTCTTGAGCTTGGAAAAAGGAGTAGATTATGGTTGGAGAAAG
ACGTTTTGGAGGTGTTTAGTAGCAATTCAGTGAGTAATTCTTATCTAAATTATCTTTAGTTTAATTATTT
TCAAGACTCAACACCCCGTTAATCACTAAACGTGTAACTTTGTAGGGAACAAGTGCAATTAAAGCCATAA
AATTGGAGTTCCATAATCCCACAAGGCTAATTGTAGATCCACAAGCTTTTAGAAACCTGAAAAATTTGAG
ATTGCTTATCGTTCGAAATGCAAGATTTTGTGCAAAGATAAAGTACCTTCCCGAAAGCTTAAAGTGGATT
GAGTGGCATGGATTTTCTCAACCATCTTTGCCTTCGCACTTCATTGTGAAAAATCTTGTTGGACTAGATT
TGCAACATAGCTTCATCAAAGACTTTGGGAACAGACTTAAGGTAACTTATATTTGTACGACTTGGGTGTA
TTTTTATCAGAAGTTTCTTCCTATTGTCTTTCAGTAAAATTAGAAACGGACAGAATGGACAAAACATTTT
CACTTCATAGCAAAAATATGAAAATTAGAATTTTTTTTTGGCTGTATTTGCAAATTTAGAAAAATATTGT
AGATCTTTTTGCAACTCATTACAATTTCGAAAACTAATGTAAATAGACTATTAATCTTGATCTGGATTTC
CATCACTGATTTGCTTTTATATATGGTGTATTATTAGTTTATCTTATAGACTGATTGATTGATGGTTATT
ATAGGTTGGTGAATGGTTGAAGCATGTTAATCTTAGCTATTCTACGTCATTGAAGAAAATTCCCGATTTC
TCTGCGGCATCGAACCTTGAAAAATTGTACTTAAGGGATTGCACAAATTTAAGAACAATTCATAGGTCCA
TTTTTTGTCTTGTGAAGCTTACTCTCCTGTGCCTTAGTGGTTGCTGTATGATTAAAAAACTTCCGACAAG
TTGTTTCAAGTTATGGTCTCTTAAACATTTGGATCTCTCTGGTTGCACAAAACTTGAGAAAATTCCAGAC
TTTTCCTCTGCATTAAACCTCGAAATTTTGCATCTCAGTCGATGCACAAATTTGAGAACAATACATAACT
CTGTTTTTTCTCTTCATAAGCTCATTTCCCTATATCTTGACTTTTGTTCCACTCTTAAAACGCTTCCAAC
AAGCTGCTTCATGTTAACGTCTCTCAACACTTTGACTCTTTATTCCTGTCAAAAACTTGAGGAAGTTCCA
GACTTGTCCTCCGCATCAAACCTTAACAGTTTGAATGTCGAAAAATGCACAAATTTAAGAGGGATTCATG
AGTCTATTGGATCATTGGATAGGCTTCAAACTTTGGTCTCTAGGAAATGCACTAACCTTGTAAAGCTTCC
AAGCATCCTCCGGTTAAAGTCTCTGAAGCATTTAGATCTTTCCTGGTGTAGTAAGCTTGAAAGTTTCCCA
ATAATTGATGAAAACATGAAATCTTTAAGGTTTCTGGATTTGAGTTTTACTGCAATAAAAGATTTACCTT
CATCAATTGGATATCTTACCGAGCTCCCTCGATTAAACCTTGGCAATTGCACAAGCCTCATCTCCCTTCC
CAAAACAATTTCTTTGTTAATGTCCTTGTTGGATCTTGAATTAAGGAATTGCAGGTCTCTTCAAGAAATT
CCAAACCTTCCTCAAAATATACAGAATTTGGATGCCTATGGCTGTGAATTGTTGACTAAAAGTCCAGATA
ACATTGTGGATATAATATCACAAAAACAGGTTCTGTCTCTTTCAATTCAATTATTTATTCTGATCTGTTG
TAAACAATTTTATGCATATGAATTTTTATTCAGTTTAGGACCTCACATTGGGTGAGATTTCAAGAGAGTT
CTTATTAATGGGCGTTGAGATTCCAAAATGGTTCAGCTATAAGACTACATCAAATTTGGTGAGTGCTAGC
TTTCGTCACTATTCAGACATGGAAAGAACTTTGGCTGCATGTGTTAGTTTCAAAGTGAATGGAGATTCAT
CTAGAAGAATTTCATGCAATATATTCATCTGCAATAGATTCCACTGTTCATTTTCAAGACCATTTCTTCC
ATCAAAATCAGAGTACATGTGGTTAGTAACAACTTCTCTGGCATGGGGCTCCTTGGATGCGCAGGATTGG
AATAAAGTTGTGGTTCTGTTTGAAGTTGATGATGAGGTTAATCTGAGTATAAGAAGCTATGGTGTCCATG
TCACTGAAGAGTTCAATGGGACACAAACAGATGTCAAGTGGCCAGTGGTAAATTATGGTGATTTTTATCA
ACCGGAGAAATTGCAAAATCTGTAAGTTCACTTTTACTTTGTACTTTTTCTTTTTGTTGGTTTTTTTGGA
GTGAATCTATGATAGACTCCAAAAAGGGAGATGTGTGTATGATATGGTTATGCTGTTTTAATGGTACAGG
GATATTGAGGATATTCTTGTCAAACGTTTATTTGATGAACTCTCCTACTTGTCAAATTGCAAAGCAGTGT
TGCATGCAGGAAGTTATGATCCAATAGTAATAACCGATTCCAACATACAACCTATGATTTTCCCATTGCA
TGTAACATATAGTGGTTATACAGTGATAAGCGGAATGGAAGGCATGGGAAAAACTGCACTCGCAAATTCT
CTACGCAACAAATTTAAAAGGAAAGATAACAGCAATTGGGGACAATGTTTAAATGATTCTTCAAGATTTT
ACTTGCTCCAAGGAAGAAAATCCCGTATATTTTCAGGATATGCGAACCCCAGTAAGCGACGAATATCCTC
CAAAAGGTATTATTACATAACCTTTGTAAATTTGGATGTTATAGAAGCTCAAAATGTAAATGCATGGTTT
ACGGCACAACGTTGGATTATATGTTGTTCTCCCCTTGAAAGTTTACGAAGATGTAGCCATTTTGTTATTA
CAAGTGTTGACCCATCCTTATGGCACACCTGTGGGGTTGATGACGTCCTCAGCTCAACGTTTCAAAAAAA

ATTCAGTGAACGGCGGGCTTATATCTTTGGAATACACTTACCAGACAATTTCCTCATGTAATCGATTAGA
AGGGTATATGACCTTCAAACTTGAAATTAAGGATATCATCTTCAAACTTCTTTCTTTCTGTTCATAACGC
ACATTCACATTCACTCGTTGCCTACTCTTTTTACTTTTCATTTTAATTTTCTTCTCTCCCTTCTTCCTCC
ATTTCCCTTTCTTCTCCCCATTCTGTTAAATATTTTCTCCTTTTTCCCCTTGTTCATTTTCAAAGAAAAA
AAAGAAAAGAAAAGAAATGAACAACAATTGTGATAGAGACTAAGAATGTTCACAAAGTTGGAAGCGTGTG
ATGAACACCCCAAATAAATAATCACCAAATAGAATATTTGAGAGGAAACAAAAACTTATTGGAAGAATTT
TTATGTGTATTCAACTCACACTTGAATGATTTAGATTACAATATTTAAATTCTATTTATAGGATTGTCAC
GAGAATAACTCATATCACAATTAAATCCTAAACTTAAATAACTAAATTAACTCTAACATCCTTTAATTTC
TTAACTCTCATAAATTAATGCACATATAACTAATTCATAAAACATGTTTAAGTTTCAACATCCTTCTTTA
AACATTTTTTTTGTTTGAAAAACTCCAAGCAAAGTTCTTAAGTTGTTAAACTCATTAACTTTGAGTGGCT
TCGTGAAAATATTTGCAATTTGATTTTCAGTCTTCACATATTCAACTTGAACCTTGTTACTTGAAATGCA
ATCTCTGATGAAGTGAAATCTTGTATCAATGTGTTTGCTACAATCATGGAACATAGGATTCTTTGCTAAA
GTAATTGTTGACTTATTGTCTACCTGGATCACAGTTGGATCATCTTGCAAAAATTCCAACTATTTTTAAC
AAATTTCTTAACCAAACTGCATGACAAACATATGAAGCTGCACCAACGTATTCTGCCTCACAAGTGGATA
ATGTCACAATAGGTTGCTTCTTAGAACTCCAAGTAAATGCAGTATTACCAATGAAGAAAACATATCCGCT
AGTGCTCTTTCAATCATTAATATCTCCAGCCCAGTCACTATCTCAATAGCCTTCAAGCTTGAATTCTTTA
GATGAAGAATAAAACAACCCATAGTCAAGCATACCTTTAAGGTAACGAAGAATTCGCTTTGCCACTTTCA
AATGAGTAGTTGTAGGAGATTTCATAAATCGACTCACCAATCCAACATTGAAAAGAATATCTGGTCGTGT
GCAAGTCAAGTATCTCAAACTCCCAACCAAACTTTTGAAATATGAAGGATCAACATCATCTTCTTCTTTA
CGTTTGGACAGTTTGGTCCCAGTTTCAATTGGAATTGTGACAGGCTTAGAATTGATCATATTGAACTTCT
TTAGAATTTCTCTAGTATATATATCATTCTTGAGAGACGAAAATCCCTTCCTCTGACTGCTTCACCTCAA
TGCCAAGATAATATGACATCAGCTCTATATCTGTCATTTCAAATTCTTGGGTCATCGCCTTCTTGTGATC
TTCAAACATACTTGTACAATTTCCTATAAAAATTAAGTCATCCACGTACAAAAAAACCACCAAAATATCT
CCATGACCATTAACCTTAATATAAAGAGAATGTTCATAAGGACACCTCAAATACCCATTATCAAGGAAAT
ATTTGTTGATTATGCTATTCCACATTCTTGGTGCTTGTTTCAATCCATATAATGCCTTATTCAATTTTAG
AACTTTATCATCTTGGCCTTTCACAGAATAACCAGGAAATTGTTCTAAGTAGACTTCTTCTTCTAGATAT
TCATTCAAAAATGCCGATTTGACATCCATCTGAAAGATCTTCCAATTATTTTCACTACTACAAATTTGAC
ATTTTTTATATGTGAAGCATGTCAAAAATTGTCTACCTTAATGGTCATTGCCCATCAAGTAGGCAGACAT
CAAGAAAGCTCATCCATAAAGAAAAACTAATTTTCTTGATGGCTATAACATGTCATAAAAAATCATTTCT
TGACTGTTTGGGCCATCAAGAAAGGGGAATCTAATGTTTTTTGCCCATTAAGTATAATGAAAAGCTCATG
AAGAAAACCAGATTTCTTAATGGCCAAACCAAGTCATCAAAAACTCTTTCTTGATGTTTGACCATCAAGA
AAGGAGACTTTGTCATCTTTTGCCCATCAAGAATATGGAAAAGTCCATCAAGATAATTATTTCCATTGAC
ATGTTTTGTCTATTAAGTATTATCCTTATATTAACAGTTTTGCACTGTCAACTAAACTCTTTTGTTGATG
TTTTTTAACCGTCAACTATAACAATTTAAAAACATAAAAAAATATGGGTCATTACGACTCTCTCATATGT
GACCTCATCTAATCAAACGTTAAATTACAGAAATGTCAAAATATGAAAAATCCATATACATACAACTATA
CAACAACTTTTGCATAATACCAACAATCACAAATATTAAAACATTTAACAAAATCAAAATGTATCAACAA
CTTGGTACAAAATTTGTTCAACATCATTAATAAAATGAAAATAATAAACTTGTAATCAAAATTACTTAAA
CATCGACAAGTTTCCAAGTTTGAAATAGGTTCAAGTAACTCATTTGAACTTGCCGTCATATATCTGAATG
ATTGTTTCTTTGCCCCTATTAACAAATGCATAAAACAAAATGTTTTTATTACAATATAAATATAAAAACT
AACAATATCACTTTAATTAAATTTGATAGCCAATTTAATTGAGAAAAGAAAGGTTATACATAATTGTGAT
AGTAAGAAGTCTCAATTTACCTCTAAGGCACACTTACAACTCAAACTAAAAGAGAAAAGGAAGATATTAC
ACTGTTATGTATAATAACAGTATATTATACTTATATGACATTATCAAGTAAAAAAGAAAAAGGAAGATAA
AAGGATTGTAGCATAATACTATAATTCAATTACTTACTCTGCTCTCAAGTCTTTTGTGAGAATAGTTTTG
AAGAAGTGGTAAAAATTCATAAGAAACTAAAAAATTAAAAAAAAAAATCAAGAATCCACTATTCAAATAC
AAAATTCCATTAAAAAAAATATGGTATGAAACCAAATGAGTTACATGAATAAAAGAAATTGTAGGAATGC
GATCAGTTGGCGGAGATCAAAGACGAAAGTAAATTCCAGTAGATCATCACAACCTAAATACATATTGTAT
CAACAATAAAAAAGGAGATTAGAAACTTTAATAAAAAAAACACTAGGAATCAAGATCGCTATTATTAAGA
AACATAAATTGAATTAAGGAAAAATAATTAATAATAGAAAGTTATAAACTAGAATTTGGTAAACCTATAC
GAAAGTATCAAAGAACCTAGAGATTAAAATATTTTGGTGTTGAGAGAAACTAGGTGGGAATGGAGAAAAT
TAAACCCGAGAAGAGGATAGTGGAAAGCAAAAGGCTAGGTGTAAAAATAGGACCGGTGTTGTACTAAAAT
ATAGGTCAAAAAATAAGGAGTAGGGAGTTAACATTTACCGTTTACTTATTTTGTTATATATATATATGAA
AAAAATCAAATTTACCTATTTTGTTAAAATATATGTATGAAAAATTCAAAATTTGAAGATAATCGTTCTT
TTCTAACTAAAATGGTAAACGTTTTTTATGGAGAAAAAAATGGCAGTTAAAATTTTACTTAGTCAATATA
ATATCAATTTTTTTACTAGAAAAATAAAGTTACAAAGTATAAGTTAAAATATACTTAACACATAAAAGAA
ATTTTTGCAACTAATGAGTGGTATATACGATATAACATTTTTTAAAACGCTATATTTCTAAATAGAGGTA
TTTTTTATTTATTTTTTGTGACACTTTTAAATGTTATAAGTAATTATTGCAATCAACTCTATTGCAACCA
TTGTTAAATTGTTGGAACTTAAAATGTCAAACTTCGAAGAGATATCGAGATATCGCCAACAAACTTGAAT
TTTTATGAAAAACCAAAATTGAAGAAACACTAGGGTAAACGTGAGGGATGGCCGCTTACCTCACGATAAA
TCTTTTCTTTATGTTTTTTTAATTATTATTATTATTATTGATTTAGTTATTATCACTACAAGAGATGTGG
GTACTCCCGACGCACAAATACGTCGGCGAAAATGCAAAGTACGTCGGGAAAGGATATCCCGACGTACAAA

ACGGCGTCGGGAAGAACGTCGGGAGAAATGCGTCGCGAGAGGCTTTCCCGACGCCGATTTGGTACGGCGT
CGGGAAAGGCTTTCCCGACGCTAGGAGGTGCGTCGGCATCGACGGCGTCGGGAAAACCTTATTTTTTGAC
TTTTTTTCCCGACGTAATGAACGTCGGGAAATATTTTTATATCGACACTAAAGACAACATCTTTGCCTTG
TTCTGGGCCCATTTTTTTAATTTTATTTTTATTAAAATGTTTCATTATTGTCCAGTTTTTATAAACTGAC
ATTGAGTCCATGTAGATAAATATTTTTTTACCCCAATAAATCAATAAAATTAATATTATTTTAGAAACTA
TAATATTTGTCTTTTACATTTGTATACACAAAATGGAATCTTATATTATGTTTATATATTCTACAACGGT
ACATGAAACAAGATCCTAATACAAAACTTAAATAAGAAATCTTAAACGCCTTCTCAAACCTTCAACGATC
GTCTGACTTTCTACACAATCGCTTAGCTTTCAGTTTGATATGACTTCAATCATTCTACAAGCAATATCAA
GAACACCCAACACGTGGATTTTGAATCTTCAACTGGCGCAACTATGATCAGCCCTGTTGTGGAACTTGGT
TCAGTTTGTAATAATTGCAATTTCCCCGACTACTGTATCAAACACCTACAACACCATTCAAACATTTTCT
TTCATCAAACTGAATTCTCCTACTTAAACCATTAAAATCAGTTTGCATAAGTAAATCTCACCCTCTATCA
AACACTTTTAACTTTACCCAATTGAATTCCACTTTCATTAGAGCCAAACACTTTACCTATGGGTGAAAAT
GTAATGATATTCTCATTTTCTTTAAGAAATTATTCACTGCCTTTCCAACAATCCATACTGAATCATAGCC
AAAAATCAAGGCAAATATTTAAAATAATGCAAGGAAATAACAGGAACCTTAAATTTTGCAACAAACTTAA
TCCAAAAGAAGTAAAACTTGCAGAAACTATAATCGACAATGAAAAGAAAAGAAAGAAAGAAGAAATTGCT
CACCGCTCCAAGAACAAACCCAAGCAAACATAGAACCAGAACCAATATTTCCCGATACTTCATCTTCCTT
TCCTATTTCTGCAACATTAACCACATAAATCTCAACCAGAAACAACATCCATGCAAACAATAAAATCATC
AACAACGAAATAGAAAATCAAGCCAATTTTATAGCAATTGCAACAGAATAAAGAAGAGAAACTGCAGACC
CTACTCACATTTTTTATTTGAACAATGTAAAACAGAGTTAATCCGATCATAGTATTTTTTGGTCAACATG
ATCTAGCACACACCTAAACTAATTTTCATAAAAAAAAAAACCCTAAACTAATTTTTATCAAAAAAAAACCCT
AAAACATAAAGTTAAACTAAATAAATTTACATATTTCACTTACCTAAAGTGGCAGACGGCGACGAGGAAC
AACGGCGAGGGCGGTCGGCGACGGACGGCGGCGGAACAAAAGGATTATGAACGGCGGCGACTTCTTCTTC
TTCTCCTTTCTTTTTCTCCTCCTCTCGGTTTCGGTTCTGTGAATACAGAACTGAAACGAATTTAAAGGGG
ATTTCCCGACGCAGTGACGTGGCGTCGGGAAATCCCTCAAAATGCGTCGGGAAAAGGGGTTTTTCCCGAC
GTATTCTTGCCGACGCCTTCGTGGTGCGTCGGGAAAGATGGTTTTCCCGACGCAGCTCCCGACGCGTTGT
TGAGGGCGTCGGGAAAGCCCTTATTCCCGACGTTCTTTATGCCGACGTGATTTTCGGCGTCGGGAATGCT
CCATTTTCTTGTAGTGTATATTTTATATTCAAATGTGAGGGATTGGTTCGTATCATAACTGTCTTTTCAC
CAAATAATTGAAAAAAAAATAAAGCGGGTCAAAATTTTCCAACTCCATTAAGAAAATGAAATTTCCACAAA
TAAAAACTTTTATAGAAAAATGACATTAATAAAATAACAAAATATATATGACAAGAAGGAAGTGAAACAA
TTAATATAAATATAATGTTAATTTTATATATTCCGTGATGGTTGTTGCCCATAAAGAAAGGTATTCTTGA
TGACCGTTGCCCATTAAGAAAACAATTTAAAAAATAAATTGTTTATTTCCTTAATAGTTCATGCCCATCA
AGAACAATTTTCTTTGATGGGCAACATAAGGAAATCAACGAAATTCATTTTTTACCTATCAAAGTAAATC
AAATTGTTTTTCCACAATTTCTTGATGGGCAATGCCTATCCAATAATATGATATTTTTTACATTAAGCAA
TGCTTTTCTTGATGTTTTTTACCGTCAACGAAACTGCAACCCAAAAACTCATGTATTTTATCATTTTTGC
ATATGTGTCCACAAAATAAGAGATCTTTTCTTGACGGTTAGGACCGTCAAGATAACCATTAAATCAAGAA
AAATATTTTTCTTGATGATCTTTCGCCATGACCATCAAGAAATTAGAATTTATGTGCGTCAAGAAAAGCT
AAATTTGTAGTAGTGTTTGAGTAACAAGAGCAATTAACAACCTTATGGTTTCCAAATGAGCAATGAGAGC
AAATACTTCATCATAATCAATGCCTTTTCTTTGAGAATACCCTTTTGCAACTAATCTTGCTTTGTATCTC
TCCACTTCTCCTTTTTCATTTCTTTTTATCTTAAATACCCATTTCACACCTACTGCTTTTTTTCCATTTG
GAAGAGTAGAAAATTTCTACGTATCATTCTTTTTTATGGCTTTTTTCTCTTCATTCATAGCAATCTTCCA
TTTGTCATTTTGTGAAGCTTCTTTAAAATTCAAAGGTTCACTGTTACCAAATAGACAAAAAAGAGTAAGG
TTATTAAAACTTTGACTTAACTCTTCAGTTTCATCACATATGTCTCGTAAGCTTCTCATGCCACGAGACC
CTTCACTTAAACTTGCAGAAGATAAAGATGTGCTTTGTTATGTAGTGATTGGCGATGTTGGTGGTGTTGA
TGAAGAAGCAATGTCACTAGGCTCATCATGATCGTTGGAAAAAAGAACAAATTTGTAGTCTTCTGGTTCG
TCATTCCAATTCCATGATGCTTCTTCATCAAACACAACATCTCTACTTACAATTGCCTTCTTTGCAACAA
GATTATAAAGCTTGTAGCCTTTTGAGCTTGCATTATAGCTAACAAAAACATATTTCTCACTTTTATCATC
AAGCTTACTACGCTTTTGATTAGGTATATGCGCATAAGCCATGCATCCGAATACTCTCAAATGAGCAATG
GATGGTTTTCTTCCTGTCTATGCTTGTTAAGGAGTTTTATTCCAGAAGCTTCAAGTAGGGGAACGATTTG
ACAAGTACACTGCACACTCAACAACTCGTGTCTAAAATTATTTTGGCAATTTCTTGCTCTTCAACATGCT
TCGAGCCATGTTAAGTATTGTTCGGTTCTTCCTCTCAACTACACCATTTTGTTGAGGAGTAAATGGAACT
GTAATAGGTTGACGGATTCCATTTTCTGCGCAAAAAGTTTTGAATCCATTTAAAGTGAATTCACCTCTCC
TGTCTGATCTCAAAGCTTTAATGTAAAAACCAATTTATTTTTCAACAAGAGCTTTAAATCTCTTGAACAT
GCAAAATACTTCTGATTTCTCCTTGATAAAGTAAACCCAAGTTTTTTTGACTGAAATCATCAATAAATAA
TAAGAAATAATTATTCTTATCGAAAGAAGTTGGTTTGATAGGTCCACAAAGATCCGTATGAACTAACTCC
AGTGGTCTCCTTGCTCTCAAAGATGATTCTTGTGGAAAACTCTTTCTTAATTGTTTGCCATAAAGACAAC
CTTCACAAAATTGATCTGGATGTTTAACATATGGCAACCCTTTCACCATGTCCTTTCTGGCTAATAATCT
CAAGCCATCAAAGTTCAAATGCCCAAATCTCAAGTGCCAAATCCAATTGGGATCTTTCAAACATGACTTT
AAACATTTAACGACATCAGTTTGAATGTTTAATAAAAACATTCTCTTTCGTCATTTGCACTTTAGCAATC
ATATTGTCATGATTACCTCTTATCAAAAGACTATAATCCTTCATCAAAATATTATAGCCTTTCTCTAAGA
GTTGCCCCGAACTCAAAATGTTCTTTATATTAGGCACATAATAAACATTAGAGATAAACTCATGCTTCCC

ATTCTTCAAATTAATCAAAATTTTACCTTTTCCTTTCACTAGAATTTTTGTGGCATCACCAAATACGATA
TCGCCACCAACATATTCATCAAGTTTCACGAACATTGATTTACTTCTACACATGTGATTGCTTCTTGTAC
CACTATCGAGATATCATGCACTGTTTTCACATGTTTCCGCACCTTTGCATGCTAGAAACAATGAGGAATC
ACCACTTTCTTCACTTTCTCAGCATAATTTGCATTTCCTTCAACTATATTTCTGCATTCCCAAGAATAAT
GGTCGAATTTATATATGAAAATTATAACATTCAACCTGTCTTTTGTCATACCTCCTATCATTATTTGATC
TTTCCCCACTTGACCTCGAATAATGTTGTCTTCCTCGACCTCTTGATGAATTTAAGTTTGAATTACTCTC
ATCAAATTTTCTTTGACCGTAACTTCCTCGACCTCGATCTTTAAAATCACCACGTCCACGATTACCACCA
TGTCCTCGACCTCGATTGCCTTTTTTTAGGTTGTTTTCCTTGTCTTTTAATTTCAACTTTGACTGAAAAA
GTTGCTCAGTCATCTGCTTGAAGCTCCCAATTGGATTTGGAATTTGAGATTTGGACATTTGAACTTTGAT
GGCTTGAGACTACTAGCTAGGAAGAACATGGTGAAAGAGTTGCCATATTATGTCAAACATCCAGATCAAT
TTTGTGAAGGTTGTCTTTATGGCAAACAATCAAGAAAGAGTTTTCCACAAGAATCATCTTTGAGATCAAG
GAGTCGTTGGAGTTAGTTCACACTGATCTTTATGAATTAATCAAACCCAAGTTCTTTCGGTAAGAATAAT
TATGTCTTATTATTTATTGATGATTTTAGCCGAAAAACTTGGGTTTACTTTGTCAAGGAGAAATCAGAAG
TATTTGGCATGTTCAAGAGATTTAAAGCTCTTGTTGAAAAAGAAAGTGGTCATTACATTAAAGCTTTGAG
ATCAAACATGGGAGGTGAATTCACTTCAAATGAATTCAAAATTTTTTGCGCAGAAAATTCATCGACCTAT
GATAGTTCCATTTACTCCTCAACAAAATGGTGTTATTAAGAGGAAGAACTAAACAATACTTAACATGGCT
CGAAGCATGTTGAAGTGCAATAAGATGCCAAAAGAATTTTAGGCACAAGTTGTTGAGTGTGCAGTGTACT
TGTCAAATCGTTCCCCTACTTGAAGCTTGTGGAACAAAACTCCTCAACAAGCATGGATAGGAAGAAAACT
ATCAATTGCTCATTTGAAAGTATTTGAATGTATCGCTTATGTGCATATACCTGATCAAAAGTGTAGTAAG
CTTGATGAAAAAAGTGAGAAACATGTTTTTGTTGGCTATAATGCAAGCTCAAAAGGTTACAAGCTTTACA
ATCCTTTACAAAGAAGATGATGATAAGAAGAGATGTTATGTTTGATGAAGAAACACTATAGAATTGGAAT
GACAAACCAGAAGACTACAAATTTTTGCTTTTTTCCAATGATCATCATAAGCCTAGTGATATTCTTCTCC
ATCAACACCACCAACATCGCCAATCACTCCATGACAAAACATACCTTCATCATTTGCAAGTTCAAGTGAA
GGGCCTCATGGCATGAGAAGCTTACGAGACATATATGATGAAACTGAAGAGTTAAGTCAAAGTTTTAATA
ATCTTACTCTCTTTTGTCTATTTGGTGACAGTGAACCTTTGAACTTTGAAAAAGATTTGCAAAATGACAA
ATGGAAGATTGCTATGCATGAAGGGATAAAAGCCAGAAAGAGGAATGATACGCATGAACTTTCTATACTC
TTCCAAATGGAAAGAAAGCAATAGGTGTCAAATGGGTGTTCAAGATAAAAAGAAATGAAAAGGTAGAAGT
GGAGAGATACAAAGCAAATTAGTTGTTGCAAAATGATATATTCTCAAAGAAAAGAAATTGATTACGATGA
AGTATTCGCTCCCGTTGCTCGTTTGAAAACCATAAGGTTATTAATTGTGCTTGCTGCTCAAAATAATTGG
AAGATCTTTCAAATGGACGTGAAATCATCATTTTTGAATGGATATCTAGAAGAAGAAATCTACTTAGAAC
AACATCTTGGTTATTCTGTGAAAGGCCAAGAGGATAAAGTTCTAAAATTGAAGAAGGCATTGTATCGATT
GAAACAAGCACCAAGAACATGTAATAGCATAATCAACAAATATTTCATTGATAATGGGTATATAAGGTGT
CCTTATGAACATTCTCTTTATATTAAGACTGATAGTCATGGAGATATTTTTGTGATTTATTTGTACATGG
ATGACTTAATTTTTACAGGAAATTGTACAAGTATGTTTGAAGATCTCAAGAAGGCAATGACGCAATAATT
TGAAATGAAAGATATTGGGTTGATGTCATATTATCTTGGCATTGTGGTGAAACAATTAGAGGAAGGTATT
TTCATCTCTCAAGAACGATATACTAGAGAAGTTCAATATGATCAATTCTAAGCTTGTCACAACTTCGATT
GAAACCAGAACCAAACTGTCCAAATATGAAGAAGGAGACTATGTTGATCCTTCATATTTCAAAAGTTTGG
TTGGGAGTTTGAGATATTTGACTTGCACATGACCAGATATTCTTTTAGTGTTGGATTGGTGAGTCGATTT
ATAGAATCTCCTACAACTACTCAATTTGAAAGTGGCAAAGAGAATTCTTCGTTACCTCAAAAGTATGCTT
GACTATTGGTTGTTTTATTCTTCATGATCTAAATAATTCAAGCTTGAAGGCTATTGTGATATTGATTGGA
TTGAAGATACTAATGACTGAAAGAGCACTAGTGGATATGTTTTCTTCATTGGTAATACTGCATTTATTTG
GAGTTCTAAGAAGCAACCTATAGTGATATTATCCACTTGTGAGGCAGAATATGTTGTTGCAGCTTTAAGT
GTCTGTCATGTAGTTTGGTTAAGAAATTTGTTAAAGACAGTTGGAATTTTGCAAGATGATCCAACTGTGA
TCCATGTAGACAATAAGACAACAATCGCTTTAGCAAGGAATCCTGTGTTCTATGATCGTAGCAAATACTC
AAGATTTCACTTCATCAGATATTGCATTTCAAGGAAGGAGGTTCAAGTTGAATATGTGAAGACTGAAGAT
CAAATTGTAGATATTTTCACGATGCCACTCAAAGTTAATGTGTTTAACAAGTTAAGAACTTTGCTTGAAG
TTTTTCAGAAAACATGTTTAAGGGAGGATGTTGAAATTAAATATGTAAGTTATATGTGAATTAATTTATG
AGAGTTAAGAAATTAAAGGATGTGAGAGTTAAAATGTTAGAGTTAATTTAGTAAGAGTTTTACATTAGTT
AAATGTAAGACTTAAACTGTTGTATTTAATTGTAGTATGAGTTATTCTCATGACAATCCTATAAGCAAGA
TTGAAAAATATTGTAATCTAAATCATCCAAGCTAGTATGAGTAGAATACACACAAAAGAGTTAGTCTTCC
AATAAGTTTTTTTATTTCCTCTCAAATATTCTATTTGGTGATTATTTATTTGGAGTGTTCATCACACGCT
TCCAACACTTATATGGATGATTTGGACAGAAAGAAACAATCACATCTTTAGAGAAATCAAAACAAGTGTT
GTGAACGGAAGACGTTGGCAATTTGATTGGTCAGGGGTCTAGTAGGCACAAATCTTATATAGACTATGAC
TTGGGTACTATCTCTCTAAAGCTTTGTTAAACTAAAGGATTCTATAAGCTTAACTCTAGCCTCCCTACCT
TGTAATTACTTTGCTCTATATAACTATATTTCGTGTTCAATCACCTAAAATATTCACATTGTGGATACAT
CGTACCTAGATGAGCTGAAACACATCGTTTCCTGATAAAAAATACTTTATTTACAAAAGTTGGATTCATA
TTGTTTTAGAATCCATCCAATTAGAAGAGGACATCAAAGTTATAATTAACTTGTTTAAAACACTTGTAAT
TTTGATTTATCCATTCCCATTATGTACAGTGTTTGTAATCTTGAAATAGGAGCCATAAAATGAATTAAAA
TAATGGATTATTCGATCAAACGTCAAAACATGAATTCATTGTTTATCGACCCCTGGTCAGATCTTAGTTT
GAAATCTCAAATGAATGCTAACATTTAGACAAATATATGTATATGTATATGTATCTATATATGTAGAATA

TATATGTATGTATATGTATATGATATGAATATATGTATATAGGTAGATACATAGATATGTAGTTATGTAT
ATATATCAGTTATCAATTGTGTGATGAATAAACGGGAACTAGAACTTGAGCTTGAGAAGAAAAGGCATTT
TGTGTGAGAAGGGCAGATAAGAATACTTCCCAATGAGAGTATTTGTGATGCACAAACTGATTATTGTAGC
TTCAGTCTAATGAATTCAATCTCGGTGGATGTATTTGTAACCACCTAATTTTATCCTATATTTTTTTAAA
AATATATTAATTGTAATAAAGGTTAAAAATTTTTATTTATTCATTTTGTTAAAAAAGAAAAAAGGTAATT
TTTTTTAATAAGATAAGATCTTAGGATAATTGTTATTATTATTATTGTTATTTTTAAAATCTTTTATTAA
AAGGGAAAATTTTAAAAAAATTAATGAAAATATTTATCTTTAACAAAAAAATAATAATAATAATTTATCA
CTTTTGACTTTTTTTTGTCATTTTAGAAAAAAACAACATATTTTATTTTATACAAAATCTTTTAATATCA
TATTTGACTTATCATTTTAGAAAAAAATAAAATAATTTATTTTATACAAAATTCTTTTAATTTATCTTTA
TCACTTTAGGAAAAAAGAAAATAAATTTATTTTGGACAATATCTTTCAATATCATATTTTTGTTATTTTA
GAAAAAAGAAAATTAATAAAATTACATAATATCTAATTTGATTTTATACTTATTAAATTTTACTAATTTG
TGACATACCTCGAAGTTTATAAAACAATCAATTTCCTTCGGGATTTAATTTTTATTTTTATTCTTCTTTT
ATTTAAATGTAGAGAGTGGAAAAAAATGTATAAGAATTTTTTTTGTACTTTTTTTTATCGTTATGCTAAAA
AAAGTCTTTAAACCTTTTTTCTTCACTCTAAAAGCCTAAGATGGAATCTAGAAATTCATGGAGGTCCGTT
GTTTCTAGATTCTTGAGGTGAGGGATCAATATTTTGGTAGTTCGAGATTTGATTCCAAGAAAAAAATGAAT
TTAATCAATGTTTAAAAATAAATATTTATTCGAAGACCAGCCTAGGATGAAATTTGAGAAATTGACAAAT
TTCTCATTCTCTAAGGTGAGAAGTTTGTCCTCAATATAGTCTAATAAATACAACGTATCCTACTTATTTG
ATGAGATTATTGCTTCAAATATTGGAGTCGTGAGCAATTAAATAGCACATAAAATAAATCTTTTTTAAAA
TGAATTTTATTCAAGACATTAAATTTGACCACCATTTTCTAGGCGGGTGTTACATGGTATTACACATTTT
CCGCACAAAAATGACTCCCGAACTCAACTCTTATCTTTTCGTAGACCATTTTTATTTTATTTAAAATTAT
TTACTTTATTTCGGTGTCCAATCATATCGTAAAAAAAGATTGGTAGCGACTCCATTTTTTTCCGATTTTA
AAATTAATTCTTTTTTAAGGACGTTGGCCGCTATGCAACGTCCCAGGTACGTGGCAATAGTACTCTTAGT
TATCCTCACACCCTTTTACCATGACCAACATCCCATTCAATGTTTTCGTTTGTCCACATACGATTTGATT
GCCGTAGTTAAGAGGTGGACCTCCATAGCCACAGCAAGCCATAATAAGTTGTTGAAACCCATAATACACA
TACCAAAATATAGCAAGTGATCAAAATATTTGACAGGATATTAATTAATAGAAAATGAGCTAAATTTATA
CAAAAGATTAGCAAGATGGCCGACCCACTCATGAAATAATTGAGAAATTGATTACTAAGGTTACCAAAAT
ACCTTTTCCTTCTCGAAGCTGAAGAACTCTAGCTTCGAAATTGAGAAACTGATTTACTTAAACCACAAAC
GAAAATGGGCTAACAAAGGTAGCAGTTGCTGGAAGAACAGTTGAGACTGTTGCTGCATAATTATCCCTTC
TGAAAATTCGGAGCACCAAAGGAATCCACTGCAGAAAATCAGATTGCAAAAAATATTAAAAAGCCAAAGC
GTGCAAAGAATATTAGAAAGCCAAAATAAGTTTAGCTCAACGATAATTTATCTCTTTTCATATCACAATC
AAGCATGAAATTAAAGAAGTCAGTATTTTATAAAAAAAAATTTAAAAAAAAATTAAAAAAAAAAAGAAGA
AGAAGAAAGTTTGAAACTTTCTTATTGTTTGAAGACCTCATTACGCAAACCACTCTCAACTCCTTTCCTA
TAATATTTACGCATTTCTTAGAATCTTCCTTATGCTAATTTTCCAAGAATTAACTAACCCATACACACTT
TGTCGTCCCCCCCCCCCCCCCCCCCACTAACAAAGTATAAATTAAATAATTGCTATAGTTTTGTGAGAAG
TCGGTTAAATATACAAAAAACAATTTTAATTTATGAATAATAAAGTTGAAAATTGAAGATTTGTTAGGGA
GTCAAATCGAAAGAAAAAATAGACAAAAAAAGAAATAGTGAATATTGACTTCTAAATTTAACCATTGACT
TCAAACTTAGATAAAACTTAGTTATATACTTTCTATTGTAATTTTTTATAAAACCTAAGTCAACGTTTGA
AAACTAAAATTAGTATTTGAAAACTTGTTTTTACTTATGGAATTTGAATAAAAGTCCAAATTGTTAATCA
AGAACAATGAAAGAATGGGAAGGAAAATAAGTTTTGTTTTCAAAAATCCTAAACTAACAACCAAATGGTT
ATCAAAAGAAGTGTTAGTTTTAAATTACAGTTTTTTCTGAAAAATAAAAACCATACATGTGGATCAAATA
TGAACTCTACAAAGAGATTTACCAACTTCTTGACTTTTATGTTCCCTTCAATTTAACTATGATGTTGATG
GAAAGATGGGAGAGGTAGAGAAAGGTCTTTGGATGAAATCGGAAGCTGTACTGTATCAATCAAATTCAAA
ATAGGATGCCCCACTTCTTCACTGCGATGCCAACTGAATCAACTTATATTGAGAGTTCAAGAGTTTGATC
TTTTTTCTCTTTATATATTGAAGACATCATTCTTCAATTTTTCCTCTGCATATTATTCTACACAAATATT
GCTAATGGTGTTTTTTTATTTTTTTTCCGTAATCTGCTCATTTCTTTCTGATGGATTCTTCCACGGAATC
ATCGACATTCAAATGGAGTTATGATGTGTTTTTGAGTTTCAGGGGAGAGGATACTCGCACTAATTTCACT
AGTCATCTTGATATGGCTTTGCGTCAAAAGGGTGTCAACGTCTTCATAGACGACAAGCTTGAAAGGGGTG
AGCAAATTTCTGAATCCCTTTTCAAATCTATACAGGAAGCTTTAATTTCTATTGTTATATTCTCTCAAAA
TTATGCATCTTCTTCCTGGTGTCTGGATGAATTGGTGAAAATAATTGAGTGTAAGAAATCCAAGGGCCAG
ATTGTTTTGCCAATTTTCTATAAGGTGGATCCGTCGGATATACGAAAACAATCTGGTACCTTCGGAGAAG
CACTGGCCAAACATCAAGCTAAGTTCCAAACAAAGACCCAAATTTGGAGGGAAGCTTTAACTACTGCTGC
TAACTTGTCTGGTTGGGATCTAGGAACCAGGTATATATTTTTACAGACATTTTGTTTTTTCTATTCCAAA
TCTCATTTTTTATGCTCTGTATGTATGTACGTATCTATGTGTATGTATGTTAATGAGCACACTACCAATTA
ATTTTAATCTTAGAAGATTCATTCTTTCTCACGATGCAATTTACTTAACTGTGTTCATCATCTTTTAATT
TTGGTATATGATTATGCAATGAACAACAGGAAGGAGGCTGATCTTATTGGAGATCTTGTTAAAAATGTTT
TGTCTACATTAAATCGCACTTGCACGCCTTTATATGTAGCTAAGTATCCAGTTGGAATTGATTCAAAATT
AGAATATATGAAGCTTCAGTCACATAATCTTTTTGAGAAGAGCAACAAATTCCATTATCAGACACAACAT
GAGTATGAGTTTGATACGGGTGTTTACTTGGTGGGGATATATGGCATTGGAGGTATTGGTAAGACAACTT
TGGCTAAAGCTTTATACAACAAAATTGCTAGCCAATTTGAAGGTTGTTGCTTTCTATCAAATGTTAGAGA
AGCTTCCAAGCAATTCAATGGCCTTGCTCAATTACAGGAAAGCCTACTCTATGAGATCCTAACGGTTGAT

TTGAAGGTTGTCAACATTGATAGAGGAATTAACATCATAAGGAATAGATTGTGTTCGAAGAAAGTTCTTA
TAGTTCTTGATGATGTAGATAAGCTTGAGCAGTTAGAAACATTGGTTGGTGGGCGTGATTGGTTTGGCCA
AGGCAGTAGAATCATGGTGACAACAAGGAATAAACATTTACTTTCTAGCCATGGCTTTGATGAAATACAC
AATATTTTAGGATTGAATGAAGACAAAGCTATTGAGCTTTTTAGTTGGCATGCTTTCAAGAAAAAATAGTC
CATCAAGTAATTATTAGACCTTTCAAAACGTGCTACAAGTTATTGTAAAGGCCATCCTTTGGCTCTTGTT
GTTTTGGGTTCTTTCCTCTGTACCAGAGATCAAGTAGAATGGTGTAGTATATTAGATGAATTTGAAAACT
CTTTAAACAAAGATATCAAAGATATTCTTCAATTAAGTTTTGATGGGCTCGAAGACAAAATAAAGGATAT
CTTTCTTGATATTTCTTGTTTACTTGTGGGAGAGAAAGTTAAGTACGTTAAGAATATGTTGAGTGCATGC
CATGTAAATCTAGATTTTGGAATTATAGTACTCATGGATCTTTCACTTATGACGATTGAAAATGACAAAG
TGCAAATGTATGATTTAATCAAGCAGATGGGTCACAAAATAGTTTGTGGTGAATCTCTTGAGCTTGGAAA
GAGGAGTAGGTTGTGGTTGGTGCGAGATGTTTGGGAGGTGCTGGTTAATAATTCAGTGAGTAACTCTCTT
TAATTTATTTAATTTCTAAGACTTCACATGATGACGAAGATTGTCAAATGTTGTGAAGTTACTAAATATA
TGACTTTTTCAGGGAACAGACGCAGTTAAAGCCATAAAGTTGGACTTTCCTAATCCTACAAGGTTGGATG
TGGGTCCACAAGCTTTTAGGAAAATGAAAAATTTGAGATTGTTTATCGTTCAAAATGCAAGATTTTCTAC
CAAGATTGAGTACCTACCTCATAGCTTAAAGTGGACTAAGTGGCATGGATTTCCTCAACCAACTTTGCCT
TCGTGCTTCATTACGAAAAATCTTGTTGGACTAGATTTGCAACATAGCTTCATAAAAACATTTGGGAACA
TACTTAAGGTAAATTTTATTTCTATGCCTTGCATTTTATAATAATTCATAAATACCATGGATCTATTATA
TTGCTTTTTGAATATATATATATATATATATATATATATATATATATATATATATATATANNNNNNNNNNNNNNN
NNNNTATATATATATATATATATATATATATATATATATATATATATAAAAGGCTTTTTTTTTTGGCATTAA
GTACGGATTATACATATGTGGAAGTTTATATGTCTGAGGGAGTTTATATGTTTATGTGTGTGGTGGAGAG
TTGTTTTTTTTTAAGTTCATATGTTTATGTTTGTGCTGCAGAGTTGTTCATAAGTTAAAGTTTCGATAAA
CAATTTTTTGAATTATAAGTAATATGCTTTTTTATATGGGAATTGTCAAAAATAACAAATTTGACAAAAT
ATTTACAATATATAGCAAAATTTCATATTATATCATAATAAACATTGATAGACACTGACATGTATCAGTG
ACATTGATAGAAACAGTGATAAAAGTCTCAGTTTCTATCATTGATAAAATCCAAAAATTTTCTATAACTT
GTAAATATTTTAATTTATTTTACTATTTTTAAAAGTATTCCTTTTTTATAAATATTTTTATTTTCAAATT
TCTTTATTCCAAATTCTCTCTTTTTTTTTTTTTTTTGAATAAAACATGAATTGCATTTTTTTTATTTAGT
TTTTTTCTTTCTTTTCGGTAATGAACAACCATTAATCCTAAATATTTTTGGCACCAACATAAATAAATAA
AATGTATAAAACAAAGGGATCTTTTAAAAAATATAAAAAGCGGTAAAATATTTACAATTTATAAAACAAT
TCCGAAAATGAAAAAAGCCCATAGGCCCACCATGTAAAATACCAAAAAGGCCTACCATATAAAATACCAA
AAATACCACGTCAACCACGTCGTTAATAACGCACGCCGTCAACAACGCGCGTGTAATATATTTGTAATCG
TTTAGATTTGGTTATTATTTGGTAAGCAATCGTTTAAATATGGCTACAATTTATCTTTTCAATTCCAGCG
TTTAATTTTGTTACACTATCGTTTAATTTGGTTCCATGATCGTTTAGATTTAAGGACCCAAATCTAAATA
ATTTTTTTTTATAAAAAAAATTGGTACACAATTTTTTTTTAATTCTTTTGGTACAATCTAAACGATGATT
TTTTTTTACACAATCGTTTATTTTTTTACACGATCGCTTACATTTGAGATTTTTTTAAAGATTCTTTATA
TACGATCTTTTATTTTTTTTTCGTTTAATTTGGCTACATTGGTTACATGATCGTTTAGATTTGGGGATCT
AAATCTAAATGTTTTTTTTTTCAAAATTTGGTATACGTCTTTTTTTAATTCTTTTGGTACACGATCGATT
AGATTTCTCCAAACGATGATTTATTTTTTTTACATAATTGTTTGCTTTTTTTACATGATCGTTTACATTT
GGCTACTCAAATCCAAATGATTTCATTTTTTTTTCAAGATTCTTTATACTCGATCTTTTATTTTTTTACA
CGATCGTTTACTTTTCTTAAACAATCGTTTACATTTGGCTATTCCAATCTAAATGATTTTTTCAAGATTA
AACGATCTTTTATTTTTTTTACATGATTGTTTACTTTTTTACATGTAGTTTACATTTGACTACCCCAATC
TAAATGATTTTTTTTCAAGATTTTTTATACTCGATCTTTTAGATTTTGCTATTTTTTTGTACACGGTCAT
TTAGATTTGGTTAACCAAATATAAACGACGTAAAAAAGAGGAAAAGAAAAAAGACGATGGAAAGAAACCG
TAGCAATAAGAAAAAAGAGCAAAAGTAGAAAGACGATGAAAAGAAATCGAAAAAAGAAGAGGAAAATAAG
GAAAACAGATTAAATGACATAAATAAAGAATTGAAAAATAAGAAAGATGATGGAAAGAAATCACAGAAAA
AAAGAGAGAAGAGGAAACAAGAAAGACGATGAAAGAAATCACAGTAGGAAGATGAAAGAAATCACAGGAG
GAAAACGATTTCATCGCGAGGAAGAGAAGAAAATGGAAGGGCAAACCTAGAATATTTAAAAAAAAAAATGG
CTTTATGGGATTTGTTACATCGGTCGTAAATGGTTTGGTATTTTGTTACATTTACGAAAGCTTACCTGAA
ACAAAAGAGAAATCAAAACTTTTGATTCATAACTTTTTCTCGTTGAATTTCATCATTTTTTTTTCTTTTTC
AAATTGGTGCCTATTTTTTGTGTATCATGATTTTTTTTTAATTGAATTTCCCTCACCATCTAGCCACCAAT
GAAATATCACCATATTTCTTCAACAATTTTGCAGTCATTTCCTCTAACTTTGGAGGATCATAAAAAAAACA
TATATCTCCATTTTATTTAACTCTTGCTAGAAAATGATTTAAAAAATAATTCTTTATTTTATGAGTTTTT
TATTTGTTAATTTTGGAGTAATTTTCATAACTGTAACAAAAACCCAAAGTATTTATGGCTCGTATAACAA
AATAATAAAAGGTCATAACATATGATCGGTTTTTCGTAAATTCCATATATGCTTTTGCAATTAATATCTT
CTTTGCGATTTATTAATCTTGAGTCATGTTTGTGCAATTAAGGCCCGAGATCGAATAACTAAAGATAGAT
CAAGTCTAGTTTCAACGTTGGGCAATGTATGCCTGAGATCGAATAAAATAAAGGAAAAATTATATTCTTT
CATTCTGAGCCATATTGTTGCCACTAAGGCCCGAGACAATACAATGACTAAGGGAAGAATTCTTTCATCC
CAGGGCATGGTATGCTTGAGATCATGTATCAAAATCTACAAGATCGTGTATGGCCGCATGTGGCTGATTT
AATGCATGTTCACATACTAAGGTAATTTTGGTATGTTACATTGTGAGTCTGTCAACTTTTTCCATTTTCA
AAATCGTTTTTTTTTTTCGGTTTGTTATATTTTTTAAGAAACCCCTATTTTTGTTTATTTTTGCTTTGCT
TTTTTGTTCGACCATTCAACTACATACATATTCTTTAAAATTCTTAACACTGATTTGATATGTGAGTTCA

TTTTAATAAACAAGTAATAGGATATACTTGTATATAATCATGGTCAACAAGAATCCAATTTCATGCATAC
AAAGTTGTGCCTTTGGATTTGAATTCAATTAAATAAAAAAAGAGAAAAGAGGAAGAAGTGAGAAGCGAGA
GGTAGGAGGGAGAAGTGAGAGAAAGAAGTTAAAAGGAAAAACTCGAAGAAAAAGTTGTTATGCCTAATGA
AGAACAAGAGAAAGTAGCAAGAAAGCGAGAGGAAGAATATGAACAAAGGAAAGAAATTGAAAAAAAAAGC
TGCACTTATGTTGGATGAGGAACAAAAGGAAGAGATCAGAAGTGGGAGGAAGGAACAAGACTTCAAGCTC
TCTTGCATATTTTCACTCGCGGCTGACGAAAGTATAAGACAATTTTTTAACGTGTTTTATTTAACCTTGA
CACAAGATATTTGTTGGATTCCCCTTTTAGGGAAATTGTCAAAATTAAATAGAATCTTTGACAAAATATT
TAAATTTCGTAAAAAACTTTAAGTGTAAAGAGTTTTGAATTTTGAATTTTTATTCTATGAAGCGTAGATA
GTTTGTCAGTTTTTCTCTTATTAAAAAAACTCCTTCATTTTATGCTGCGGCGTAATTACTTAAACAAGAT
CTCTTAATCTCTAGGTGTTGTCCAAAACTGTGTCCTTGAGTTTCAAGGATTCATCTTTGAAGTTGAGAAA
TTTGATTTCATGTTGATTTTTTTCAACTTATATCAAATCAAATAGTTGTGGAATTTTTTATTTTTTTTGA
AAAAGGATACGTCGGGTAAGTAGAGAAAGATGAGGGTGCTAAGCGAGTGTCCACCTTGTGGAGATGTCCA
AGGAGAGATTAGTGACGTAGATTAATGAAAATAAATGATTAATCTTGATATTGATTGAGTTTATCACTTA
TTTACTTTTGTATATGCAGTTTTGCCAAAATATTGATTAATGGTTATTATAGATTTTTTTTTACGTACAT
TCTGTATCATTCTATATTTACAATTATTCTTTTAGCCACTATTTTATCTACATTTTGTAATTTTGTATGC
CAATTACATACATGTTTTCCTTTTGGTTTCTTTTCCAATGTATTTTAGAATTGTGAAAGATTGAAGCATG
TTGATCTTAGCTACTCTACTTTATTGGAGAATATTCCTGATTTCTCTGCAGCATCAAACCTTGAAGAATT
GAATCTCATCAATTGCACAAATTTAAGAATGATAGATAAGTCTGTTTTTTCTCTCAATAAGCTTAATGTC
CTAAACCTTTATGGTTGTTCTAACCTTAAAAAGCTTCCAAGAGGCTACTTCATGTTAAGTTCTCTTAATG
AATTGAATCTCTCTTACTGCAAAAATCTTAAGAAAATTCCAGACTTCTCTGCAGCATTTAAGAGCTTGTA
TCTCCAAAAATGCTCAAATTTAAGAATGATTCATGAATCTGTTGGATCTTTGAAGAAGCTTGAACAATTG
AACCTTAGACAATGCACTAACCTGGTAAAGCTTCCAAGCTATCTCAGGTTAAAGTCTCTTGAATATTTAT
CACTTTCTGGGTGTTGTAAGCTTGAAAGCTTCCCAACAATTGCTGAAAACATGAAATCTTTATACGAATT
GGATTTGGATTTTACTGCCATAAAGGAGTTACCTTCATCAATTGGATATCTTACTAAGCTTTCTATATTA
AAACTTAACGGTTGCACAAACCTCATCTCCCTTCCCAATACAATTTATTTGTTAAGGAATCTTGAGAATC
TTCTTCTTAGTGGCTGTTCTATATTTGGAATGTTTCCCCATACATGGGACCCAACCATCCCAACCATCCA
ACAAGTATGCTCTCCTTCAAAAATGATGGAAACAGCTTCCTGGAGCTTAGAATTTCCCCATTTACTAGTA
CCAAATGAAAGTTTATGTGCACATTTCACTTTGTTGGATCTTGAATCTTGCAACATATCAAATGCAAAAT
TTTTAGAAATTTTATGTGATGTTGCCCCTTTCTTATCTGATCTACGTTTGTCCGAAAATAAATATTCTAG
TTTACCCTCATGTCTTCACAAGTTCATGTCCTTATGGAATCTTGAATTAAGGAATTGTAAGTTTCTTCAA
GAAATTCCAAACCTTCCCCAAAACATACAAAATTTAGATGCCAGTGGTTGCAAATCGTTGGCTCGAAGTC
CAGATAACATTGTGGATATAATATCAATAAAACAGGTTAGATTCTTTCCATTTATTTTGTTCCTATCTAG
CACGTAGACAACTTAATGCATATGAATTCTTATTCTCTATAGGACCTCGCATTGGGTGAGATTTCAAGAG
AATTCTTATTAATGGGCGTTGAGATTCCAGAATGGTTCAGCTATAAGACTGCCTCCAATTTCGTGAGTGC
TAGCTTTCGTCACTATCCAGACATGGAAAGAACTTTGGCTGCCGGCGTTAGTTTCAAAGTGAATGGAGAT
TCATATGCAAGAGGGGCCCGAATTTCATGCAATATATTCATCTGCAATAAACTCCATTTTTCTTTTTCAA
GACATTTCTTCATCAAAATCAGAATATATATGGTTAGTACCACTTCTCTAGCGTTGGGGTTCCGTGGAGG
TGAAATGATTGGNNNNNNNNNNNNNNNNNNNNNNNNNNNNNNTATTTTTTTTTATCTTTTGGAGAAATTGCAA
CGTCTGCAAGTGATTGCTACTTGTTTTTATTAGTCTCTTCTTTTTCTTTTTTTGGAGTGAAAGTATATAA
TAGATCTCAAAGGGGGAGATGAGTATGGTATGATTTTGTTTTTTTAATGGCATAGGGATAGTGAGGATCT
TCTTCTCAAAAGCTTTTTAGAATCAGTCTCCTGCTGGTCAAATTCCAAAGCAATGTTGCATGCAGGAAAT
TATGATCCAGAAGCAATAATTGATTCCAACATACAACCTATGATTTTCCCATTGCACGTAACATATAATG
GTGAAACAGTAATATGTGGAATGGAAGGCATGGGAGATACTACACTCGCCAACTCTTTATGCAATAAATT
TAAATGGATGAAGGATAACCTTTTCGAGATAAAGGAGCACCATTACAGTGAAGCATTAGATAATTCTACA
AGCATTTTCCATATCCGAGGAAGAGAGCTCCAGAGATTTTCCTGGTCATGGGCCGCCCACCATCGTAAGC
GTGGAGATGGTAAAAGAGGAACCAATATCACAACCCATACAATATCCTCCAAATGCTATTTGATGCTCTT
TCATGAAGTCGAGAACTGTAATGATATATTTGACTGGGTTGGTACACAGCGTTGGATTAAAACTTCTGGC
AGTTCTAACGGAAGAGGTGATGTTCAGTTTCTGATTAAAAGGGTTGATATATCATTGCTCTGAAGCTCTC
AATTTTTAGTAAAAATTTACATTTAAACACCATTTTTAATCTTTTTGGGACTAGTATTAACTTAAACACT
ACGATAATTAATATTAAAACGCTCAACAACTTCTGTAAGTTTACATATCAATTGAACACCCTTCTTATTT
AATTTAGATTTTGTTCAAAAATACAGTGTGAAACACATTACTAAAATTTTAAATTGTTATATTAAAGTGT
GAATCGATTTAAACTCTTTATTATGATTACATTTGATCAGGTATTCAATATCAATATCTTCACTTTCTCC
CTTAGCTAGCTAGTGATAGAGATACCAATAACAATTTTTTTTTTTTTTTTTTTTTTGAGATGGAAACAGA
AGTGTTATATAGTGCTAGGCGCACCAAAAACATCAAACCTCATTCGATTAAAATAAAGCATCCATACAAA
GTTGTGTTCAAGAGGCTAGAAACAGGCCTCAAAGTACGAGAGTTAATCTAGCAATGCACTAATATTTAAA
GAGATTGGGCTTGGAATATAGTCTTTTTTAAGAGTTTGTTTCTGTTGGACCATAGACCAATGAGGTTCAC
ACCAACAACGACGTTGAACCGACCGTAACTTTATGAGTTGTATGAATTTTCAAATTTCTATTATTTTATG
TATGATATGATTATATGTTTTTGTCAAAGGCTTGTCAAATTGCAAAGCAATGTTTCACATAACATATAAT
GATAAAACAGTGAAATGTGGAATGGATAAAGCATGGGAGACACTGCAATCGCAAACAACTCTCCATGCAA
CGACGGATGATCACTTAGCTGGATTGTTAAATTTTTAGATGATTCTACAAGCATTTGCTTTTTGCAAGGA

AGAAAGAACCATCTTTAGTGGAGATGGTGAAAGGCATAAAATATCCACCAGAAGGTATATAATTAAGGGA
CATGGGTTAGAGCACAACGTTGGATTAAATCTGATTATTATGAATGTGGAAGTGTAAGAGGAAAGTATCA
TTTTGTTATTAAAAATCCCTATGGCACACCTATATGTGGGGCTCATGATGTGTATCATCAACTCGACGTT
CAAAGAAAAAATTCAATGACCAGCATTGTGTCTTTGGATTTGCTCGTACCAGAAGTAACATTTCCCCATGT
GTGTTCAAATTACAAGGGTTTAGACATGGATTAGTATTCAATTTGCTTCTCATTTTATATGAAGGATATT
AACCTAATTCAAACTTCTTTCCTTGATCTCTTCATGACGCACATTCACATTCACACATTGTCAAACTTTT
TTTTTTTTACTTTTCACTAGAACAAATCTGGCCTTTAATGTCGGTTGAAAAAAATGAAAAAAGAGCTTTA
ATGTCGTTTTTGAAAATGCGGATGTTTAATGTCGGTTTTAAACCGACATTAAACATCCTTGTTTAGAAAA
GCGACATTAAAGCTCCTTTTTAATTTTAATTTTAATTTTTTATTTTATAAAAAAATGACTTTCTCTCTCT
TACTTTACTCTTTCTCAATCACCCACACGATTTCATCCTTCCAATTTCTTCTTCCCTCCTCATTCTCATC
TAACAATCTTCCCTTTCTTCTCTTAACTGTCGCTGCCACCACCACCACCATCGTCCCTCGTGCCCAGCCC
GTGCCCGCCGCCACCGTCGTCACTCCTCACTTTCTTCAAACACTGTTATGCTTGCCACCACCGTCGGGAG
TCCTTTTCTTCAATCATGTTCACGTCCAGCCCTCGCCACCACCGTCGCCGTCACTACAACAAATATACCT
TTTAATAACACCCAACCACAACATATTTTATAATACGCTTAGACAGAGAAGAGAATGGCACGAACGGATC
TTGGTCGGAGAAGAGCGATTTGCGTAACACCCGAATGGTTCGAACGAAATAGACAGACTTCGCGATGGAG
ATTTAAGATACGGACGGAGAAAATGCTGACGTTTCAGCTATACGATGACAGGCAAATAGAGAGACGAAGA
TGACGAGTAGCAGAGGAGGTGGTGCGCGTCGGAGAGGTGTTAACGGTGATTGAAAGATGGCCAGTGACTA
GGGTTTGGTGTGGGAGAAAGTTGGGGATTCGAAGAGCTAATTCCTTCATACTTCTAATAATGATATGAAG
ATGAAAACCTCAGCTGATTCCTTCTACGACCCTGTTTGTATCCGGTAATTATTTTCTCTATTCTCTTCGA
TTTATCGTCTTGAACCATATATATTCCAGAAATCCAAAAGGTAATGTTTTTTTATGTTGTGCAACTTTGG
TTTTGGGCATTTGTTATCATTTCTATGTTCTCATCGTTATATGAATCAGGCTACAGTTCTTGACAACTAC
AACGATATCATGAAATCTTGTAATGCTCTAGACTACCACGACCTAATAAGCTGTTCCTTGAAGCTGCTTA
CTGATTTTTCTGAAGGTTCAATACTAGCTTATCAGTTTTAAATATTATTTGAAGTGTTAGGCATGTTTTG
ACCCATTTGTAGGATTAATGGAAATCAATTATTGCTAATGAGTTTCAAGATACAAGTTCCATGCAGTATA
AGCAGATAACTATAGTTTGTGATGATGATCTGGTTAGACTATTAGTTCTCTTACCTCAATGCAAAATTGT
TCATTTGGCTTCTATGATTTTTTCTTTCTGTGTTTCTTTTTTTTTTTTGTATGCTATGTCTATCTGAAAT
TTATGTCATTCATACAGATCTTATATAATCACCAACTGTGACATGAACATGATCTTCTTTACAGGTTTTT
TCAGGCATTTGTAGTATACGTTGGTTTAATACTCAGAACTTAACTTCTAAAACAGAGTTGAAATATAGGT
TCTTATTTATGAAAGAAGTTAAATTACAAGTTTAGTCTAAAAAATATGATATCGTGGCCATGGGTTCCTT
TGTTGTTTGTTTTGCTTGTTCATGTGGTCCCCAATTATTATTAGTTATTGAGCTCCTTTCAGGAAATCAT
GTTTCTCTTAAGTTCAATTCAGCAGATAAATTTCTATAGGCTGTGAGAAGATTTTGTTTTGGTGCAACTC
TACATTGTCATGTATACTCTCATCTTTCTTTGCATTCAGGTGCTCTCTATTTGCACTTTGCCACATCAGT
TATTGAAGAAATGAAAAATTCTTTGGGAATTCACTGCTTCAGGTGAGAGAAAACAAAGCCACGTGATATA
AATTTCTGCAAGTTACCAAATTGGAAAAGTACTAGGATTCTCTCATTTATTGTCTGGTTCAAACTATACC
GTTTATACACTTTACTTGTACATGTTCATCAATTTACTTCCACTCCACCATGATACAAAGGTTTTATCTT
ATGCTTGGAATAACTGGAGCTTGAATATATCATACCAGATATGCACACTGCTAGCACTACTTGTTTCTCT
TAATCGTTCTTCTATTCTGAAATCTTTGATTGTGGGTTTGAAATATTATCTCTCGACACCTTTTGTCTTT
TTTCTACTTCTTGAATCTCTGATTGTAAAACACATAAAGTTTGTTAAAAGTTAGAGTAAGTAAATGATCT
TAGGAGAGCAATCATGGTGGGGTGGCCAACGATCTGTGTATTTCATCATTCTATTTAAACAAACATTCTC
TTTACATTTTACAGAGTGTGTGAGCAAGGAACATTGAGTGTGCGGTTCTCTTTTCCTTCTTCATTGTCCT
CTGTATTCGTGTGAAAATAAGTAAAGAGTTTTGTGAGGCTTTCTACTATAGGCTTTCTCCTATAACTCTC
CAGTGTTCTTCTCCTTCTTCTATCTTTTATTTACGTTTGCATTATTTGGCTATTGGTACTGTTTTTGATT
GAGAAAAAATCCAGCAACAGTCTACAATTTTTGCGCTAGCTTTTAATTATAACTTGATTAATTCGATCTCA
AAGTTCTATATCTAATTGTACATTGTACATGGCCACTAGTTTGTAATTCTTTTTAATTCTCCTAGCTTTA
AATTTTGTCACTTCCATACTCTGTTATTAGAAGTTGATTCCAAGATTCAAGAACGTATTTTCTTTTTCTT
TTTCTGTTTTTATTTTGATGATTGTTTATTTTAAAGTACTTTTCATAACTTAGTATAACCTTTTTGTTGT
AGTTAAGGTGTTTGAAAAAAATATCTGAGAGATCTTCACTAGATTTTCATAGTTACTAATGTGGCAGCTAT
TGGGTCTCCATGGTATGTCTTTGAGAGAAAAAGGTATCTACAACGTGAATCTAATGACACTCATTGTTTT
TGTTACTTGGCTTTGAGCTCACTATGGTTTAATTGCTTAAAGAGATCATGGAAGTATCAAAGAGATACAT
AGGAGGTTCGTTCTATTTCAGTATACTTTCTATTCATCTATTTAGGCTGTGAAGAATTGCACAAGGTGTT
TTTCTCTTTCAAAATTCATTTCTTTTTGGTGCAGAACAAATGAGATGCAGAAGATGGGTTACAAGCGTTC
AACAAAGAAGTGCAAAGAAAAATGGGAAAATATGAACAAATATTTCAAAAGGACCATTGTAACTGGGAAG
GCTAGTATTTCAAATGGTAAGATATGTCCAAATTTTCAAGAATTAGATATTCTTTATAGAAATGGAGTGG
TAAATACTGGAGCTGTCTCCATTTGTGATAATTAGATATTCTTTATAGATATTCTTTCTAATTTGGTTTT
GTTGGCTATTTTTCTCCATTTGTTGGCACACTTGTTATTGAGTTAATTTTTTTAGAATCATCACCTTGGA
TATGGGCCTTCTTGTAGCTGGCACAAAATATCGTGGGGAGTTTGAAGAAAGATTAAAGAAACTGATGGAG
GAAATAAAACAAAGTGATGAAATGGGGCTGCAAAAGGAGTTATTGATGCAGCAAACATATTAAAACCTGC
CCTTGCAAGGGGTGAACTGCAGGTATTCTCGAGATAAAATAATTCACATAAAAAAGTCCAAAACAACTAA
GAGGTTATGATTGTGGGAAGCTATTGCATAAAGAACTTCCACTCTAGGTTACAGAAAGAAAAAAATAATG
TATGGCAACCTAACAATTTTCTGCGATAAGAAACCGAATACTCCCAACAAAATAAAGCCAAAACAATTTA

GAGTCCTGGCAGTGTAAAATACTGAAATTCTTGTAAGTAAATGGATAGTAGTTGAAGACATCATCTTTCT
TCCTATGGATTTGTGTAGATTTAATGCTTAATTTCTCTGGTTATTGAATTAGTTAGTTTGTAAGAGGAAT
AGATCGATATTCTGAATTCTCCAACAGTTTCTTTAACTGAGGTTAGTTCTTGACGTCAAATGATTTCGTG
ATATGTTTTCTATTGAAAGCTTTGATTTGAAAGTAAGTTACAATATATATGTGTGTGTGATATGTTAGAA
TAAGTCACAACAGTATATGCTGCTGCTGATGATTAGTTCTTAAAAAAAGTTCTAAGAATTGAAGCAAGTG
ATATGAAATGTTTTTTTAGTTTCAAATTATTTTAGTTGCTGCTTGTCTGTTTGCTCCTTTTCTGTTATTT
ATAAAAATAACTTGCTTTTTCTTACTTTGTCTTGTAATGAGTAGCAGACTGAACAAATATCTTAACGTTG
TCTTTCTAAAGCTTGATTGCAACCAAGACAATAAGGTTCATCTCATGATCCCTAATTTCTTGCTGGAAAT
TAGCAATGTATTTACAGAATAGTTCATATAAGAAGTAAGAACACTTAAATTGTCTTTATCTGGCAGCCAT
TGGCAAAGGAACTTGGGATAAAAGTGGTGCCAACTTTTAAAATTCTAAAGGATAAGAAGGTCGTTAAAGA
AGTAACTGGAGCAAAGTTCGATGATTTGGTTCATGCCATTGATACTGTAAGATCCAGTTGAAAACTCTCC
TCTCTTCTCTTCTTTGAAAATTTAAATAATATGATGCAAGGAAGAATGTTCAGGGCAGTTATTTAAATAA
TATGATGTAAGGAATCGACCATGGGAAATGATTCTGTATATAAAAATGTTTGGGAGATAGACATTTGAAT
GTGTACAATGACAGTTTCTACATATTCCAAAAATTATTTTGATATATACTTTTCTTTATGAATGGGTTTG
AATGTTTTTGTAATTGGGTTTGGGTATGTTTATGGTTTTCTTCTAGCTAGTGATAGAACTACTTACTACT
ATGCTAATTGACATGTTTGTTTGTAATAATGGAGATGATAGAAATCAAAAGTTATCAGATTAAATGGTTT
ATTTTGATATATCTTGTAGAATGATTGAAGTCATCAGATTGAAAGCTAAGCGATTGTATAGATAGCCAGG
CGATCGTTGAAGGTTGAAGACTGAGAAGGCGTTTAGAATTTCTTATTTAAGTCTTGTATTAGGATCTTGT
TTCATGTACTATTGTAGAATGTATATATAAACACAATATTAAGTTTTCATTTTGTGTATACAAATTTAAA
AGATAAATATTATAGTTTCTAAAATATTTATCTATATGGATTCAATGTTGGTTTATAAAAAATGGACAAT
AATACAACAATTAAATAAATATAAATTTCTAAAAATGGACAAAAACAAGCCTTTAATGTCGGTTTTAAAA
CTGACATTATTGGCCTCTTTAATGTCGGTTTTAAAACGACATCATAGCCCCGTCGACATTAAAGGGCTTC
AATAACACTATCAAAAATGTCGGTTTAAAACCGACATTAAAGCCCAACCGACATTAAAGGGCTTCAATAA
CACTATCAAAGATGTCGGTTGAAAACTGACATTAAAGGCCTTTAATGTCGTTTTTAAACCGGCATTAAAG
AGGCCTTTAATGTCGTTTTTAAACCGACATTAAAGTCCAACCGACATTAAAGGCCTTTAATAACGCTCGC
AAAGATGTCGGTTGTCAAGTGACATTAAAGCCCTTTAATGTCGGTTTTAAACCGACATTAAAGGTCAGAT
TTCTTGTAGTGTTTTAATTTTCTTCCATCCTGTCTTCTCTCTTGCATGTTTTCTCCTTTTTTTTTTCCCT
TATTCATTTCAAAGGAAAAAAATAGAAAGAACAATAATTGTGTCATTTGTAGGCAAAAGATTTAAAGTAT
CTATGAACATGTCGATATATCTTTAAATCTAAGGTTGAATATTTTCAACATTATCTTTTATAAATGTTCG
TAAAAGAGAACATATCATCTAGTCTAATATGAATAGAAATACTAATAACTATATTTTCATAACTTAGTTT
GGGAGAAAAAAACAACTTAAATATTTATCTAAACAAATTTTAAAATTTTGCTATTTTGAAAATATTTGTC
GATATGTATGTTTCATCGATATATCCATCAATATCATATGTTAATTGTTAATGGTGGGTCATCAAATTGT
TCCCTCTATTGACTACTGTTTGGATCGTCCGTCATTGATGTTGAAGGAAGAAGGTAAGAGAGGCAAAGAA
GCGCAAGCGATGGAAACAATAAAGAAAAGAAGAAAAAAATACATTTTTAATTATATCATTGAAAATATAT
ATTTAGTAATAAAAAAAATCAGCTTTACCAAGAAAATGTCGAAGAAATGGACTAAGAATGTTTAGAAACT
CTTGAAATTTCCAAAATGGAAACGTTAAAAGCTATGGGGACAAATTGCAAGAAACTTGAAAGTTTGGAAA
CAAAATAATATTCAAACACAAATTCTAATTTTATTTAGAAAAGTTGGATTCATATTGTTTGAGAACCCAT
CCAATTAAGAAGGGGAGAGCAAAGTTATAACTTATTTAAAACACCTGTCATTTTGATTTATTGGCTTTTG
TGAGAAGTCGGTTAAATATACAAAAATCAGTTTTAATTTATGAATAATAAAGTTGAAAATTGAATATTTG
TTAGGGAGTCGCATCAAAGAAAAAAATAGATAAAAATAACTGAATATTGATTTCTAAATTTAACCATTGA
CTTCAAACTTGGATAAAACATAGTTATATACTTTCTACTGTTATTTTCTTAGAAAAACAAACTAAAATCA
GTATTTGAAAACTTGTTTTTACTTGTGAAATTTGGATAAAAGTCCATATTGTTACTCAAGAACAATGAAA
ACTAGAGTAAGAATTGGAGAGAAGTTTAGTTTTCAAAAATCCTAAACTAACAACCAAATGGTTACGTTTC
CTTCATCTAAACAAACAATCAAATGGTTATGAAAGGAAGTGTTAGTTTTTTAAGTTCAGTTTTCTAAAAA
TCAAGAAACCATACATGTGGACCAAATATAAACTCTACAAAGAGACTTCCCAACTTCTTGACTTTTATGT
TCCCTTCAATTTAATTACGATGTTGATAAAATAAATGGGAGTGGTTGGAGAATACGATGAAATTGGAAGC
TGCACTGTACCAATCAAATTCAAAAATACAAAGCCCCACTTCTTCACTGCGATGCCAACTCAATCAACTT
ATATTGAGAGCTTACGTTTCCTTCCTCGAAAAAATTTATATTGGGAGTTGAAGCATTTGATTCTTCAATT
TTTGTTCTGCATTATTCTTCACAAATATTGCTATTGGTGTTTTCGATTTCTCTTTTCTTTAGTCTGCACG
TTTCTATCGATGGATTCTTCCACTGTTACAACAGAACCACCGACTTTCAAATGGAATTATGATGTGTTTT
TGAGTTACAGAACCATCGTCTTTTGGTATCACAAAAATGCTTGAAATTAAAGAAGAAGTCAATTTTTTAT
ATTAAAAAAGAAAGTACGATATATACATATATATAGTCTCGATTGTTTGAAGACTATACACAAACCACTC
TGAACTCTTTTCCCATAATATTTAGCCATTTCTTAGAATCTTCCTTTATGCTAATTTTCCAAGAATATAA
TTTACCAACCCATACACACACCTTTGTTCCCCACTAACAAAGTAGAAATTAAATAATTGCTATAGTTTGA
TTTATTATTATTTTTTCTTTTTGCTATCATTCAAACCTACTACATATAATTGATTAAAGGAAATTGTTGT
ATTAAAAAGTCAAATCAAATCATATTTTGTCTTCAAATTTATATTAAAAGAAAAAAAAAAAAAAAGAACCC
TCTCTTCTTTATCGGTGGCTACTTTTGTCCTATTCACTAGAAGAAATATGGTCTTTAATGTCGGGTGGAA
AAAATGAAAAATAAGCTTTAATGTCGGTTTTCAAAAGGCGGATGTTTAATGTCGGTTTAAAACCGACATT
AAAGCTTTATCTTTAATGTCGGTTTAAAACCGACATTAAACATCATGTTTTTTAGAACCGACATTAAAGG
TCTTTTTATTTTATTTTTTATTTTTTTAATTTTGTAAAAAGTTGAATTTTTCTCTCTCTTACTTTACTCT

TTTCTCCTTTCTTCTCTACCAAACCCTACACTTTACTCTTTTCTCCTTTCTTCTCTACCAAACCCTACGA
AGGTTTTCTCTCAACATTTCTTCCCTTTCTTGCTTTCTCAATCTCATCACTTTCTCCCCTCTCTTCTCTG
ATCAGTCGCCAACGCGTGTGAACTCGTCGCCGTGCTATCATCGGAGGCAGCTTCTTCCGTTGTCGCATCG
CGCTCGTGCCTCATTTAGAAAGAAAAAAGGGGATGCATGTGAATACGTCTTCTTTTCGTCAAAGCAAATA
CCCAACAATTCCTCTTTTAACCGCTCGCCATGCTTAGCTTGCTTCTTGCATTTGGCAGCTCAAATCCATG
GAAGAAACTAAGGTCTCTTCTTCCTTTTTCACTCATTCTTCCTTTCATTTACGTCCTCGATTTTGTTTCT
GTTCCATTACTCTTTCTATCTAATGCTTCTTCTCCCGTTTTCTTCACGCAATCTCTTCATTTCATCAAAC
ACCCCGTTTTTCCATTTCAATCTCTGTTCTTCTGTATCTGGGATTTCTTTGTTTTATTGGATCGTTTGCT
TCGATTCTTTGTCGCCTTCTGATTTTTCTGTTTCTACATTTCTTAGAATCCAAGTTTCTAGTTGGGTTCT
CCCTGTTGATAGATTTTAGATAGATTTTTTTGGCTTTTGCGATTGGTAATTGAATTCCGCGTACAATTTG
ACTTTTTGGGTCTATTGGGTACCCTTGTTTTCAATTTCAACTAAACTACGAGGGAATTGTTTGTTGTTTG
AGTTTCTCCCGGCGATTTGAGTATATTTTAGGTTTAACTATTATTATGATTATTATACTTTTGGCTTTGG
TTTATTTTGGTTCATACACTTTCATCTTTTCATAATCTTAGATGTAATCTTGATCCATATGTTCTGGTCC
TTGTATGTGTATTTTGATCTCATTTTTCTCTTCCAAAATTTGATGATAATGACATTTCAATCACTAAATC
ACTTTAAAACTTACCTGTAAATGCATTTAATTATGAGTTCCATTGGGTATAAAGTTTATATTAGATTTTT
GTTACTGGATATCATTTAAAAGTAACCAAAACTAGTGTTTTGAAAGATATTTATAATGAGAATTTTTGTT
TTGCATCGCAAAATGTCGTGACAAACAATTTGTAGTACTATGATCCAGAGGCAAATAATGGATAAAGTAT
ATACATCGAGGCTTATCTTTTTTAGTTAAATTTCGTTTTAGTGGACTTCACAGTTTCATTATGCGTGGTT
TCCTTTCCATGACTCTTTCCTTTTCATATCATTTAATGTTTGGCCATAAAAGTCGAATGTGTTTTTGCAG
TTGGTTGTGAATGTTTTTGCAGTTGGTTGTTTCTTCAATTTACATCGCAAAACCAAGATTAAGAGAGGGG
GAGATTCGGTGAAGGAACTTAATCGAAGTAATAAATCGGATCGTAAACCTACCAATCGAGGGATTGAAGC
ACTGGAAACTTTGCCTACGCCCCGGAGTATACCTGAGTTGTACAAAGAAAAAGAGCATACTCTGAGAGTT
TTCACTTTTGAAGAGCTCAAAATCGCGACTAATGGGTTCATCAGGTTGCTTAGGATTGGAGAAGGTGGAT
TTGGGAGCGTATATAAGGGGCAGATTAGGCTTGAAGGTGATCAGGGAGAGGAAATTATAGTTGCTATCAA
GCGACTTAAGTCAAATAGCTCACAGGTAATTGCTATCATTTTGTAGCCTTAATTGGTGTCCTTCCTTTTG
AGGTTATTGTATATCTGATTTTTTAGAAAATATTTATAATATTTTATGCTTGTGTGCTTCCAGGTCAGGG
AAATTGTTGCTGTGTTAGATTTGAAATTGTCCTTTTCCCATTTCATTTTCATGCCTAGTATCTTTCTATT
TGGAGCAAATCAATTATATTGCTCGAGGAAATGCCAAAAATGAAGGAGGCTAAAATATGACATATCCAGC
ATGAATTTTGTTGGTTTACTGTATTTTTATTTATTTTCCAATGCATCTCAAAATATGATTTTTTTAACTC
GTCACCAAAAGTTTGCATCCCCAATGATTGATGACCTTGGAAGCTCAATTATTCCACTACTTAAAAATTG
AACATTTCCAGTTTCCTCCTTCCCGACAATGTACAGATTGCTATTAGTTTTCTTAATCTCTTGCTTTGGT
TGTGCATGGCTTGGTTGCAACCTGTCAGATTAACCTGCTACGTTTGATCAAGTGTAAAGCTTTGTGTTTG
GTATTTGGTTGCAGGTTGATCCAAACACAGGAGTTTCAATGTATGAATCAGATGACATAATCAAGTATCT
GGTTCAGAACTATGGTGTGGACTCATCACAATTTATATGATCAATTTGATCCTACTTTTATTTTTCTCAT
TATTTCTTATTGTGTGCTTACTCAATCCTACTTGAGTCCTAGTCTTCATCTTGCTTCAGCAGCGGAAGCT
GGAGCTTTGGTGAGTTTAAGACCTTTTTTCCCCCCGATTTTTCCTCTTTCTTTTTGTTGCCTCATTATTG
TTTCTTTATCATTTCTAAGTCATGTTTCAAGTCCTTGGTTTTTTTTGTGCTTGTGAACGTCGAACAATTT
CCTTATTGCACTAACTAGAAGAATATTTCTATGCCCTCATCATTTTGAACTTCTGTTCTTTATTATATTC
ACAACTGTGATTGTACTACTTCTCAGGTTTGTTTTTGCACAAATCCAGTTTGGCTTGTGATGCTGTGAAC
TGTGGTGTGGTTTTTATAGTCATTTTTCTTAGCTGATTCTTGAGTGACTTGGTTTCAGGTTATTCGAGCT
CGGTTGCTACAGTTCAGGAAGAGGAATGTCAAGCCCTTGCTGCATTTGGTGCCTGTTTCCAATATTAATA
ATTGTTTGAAGAAGCATTTTCGAGACCTTTAAAATGTCGTGATCTTTGAATTTATATAATTCTGTTTGGT
TGCAGTTAATTTTACAAGCGTGTTTTCATTGTTGGTGGAATCAGACTTGAATAATATGGGATTTATGTGC
TTTAAGATATGAATGAGATTTATTGTTGCTTTAAGATATGAATGGGATTTATGTGCAAGTGCTAATAGCA
TTTTCATTGTTGGTGGAATCAGACTTGAATAATATGGTTTCCTACGTAATGTGACTTGTAGTTTCAAGTT
TGTAGTTAATTTCACTTGTCGGATTCAATGTTGGTTTATAAAAAATGGACAATAATACAACAATTAAATA
AATATAAATTTCTAAAAATGGACAAAAACAAGCCTTTGATGTCGGTTTTAAACTGACATTATTGGCCTCT
TTAATGTCGGTTTTAAAACGACATCATAGCCCAATCGCCATTAAAGGGCTTCAATAACACTATCAAAGAT
GTCGGTTTTAAACCGACATTAAAGCCAAACCGACATTAAAGGGCTTCAATAACACTATCAAAGATGTCGG
TTGAAAACCGACATTAAAGGCCTTTAATGTCGTTTTTAAACCGACATTAAAGAGGCCTTTAATGTCGTTT
TTAAACCGACATTAAAGCCCAACCGACATTAAAGGCCTTTAATAACGCTCGCAAAGATGTCGGTTGCCAA
GTGACATTAAAGGCCTTTAATGTCGGTTTTAAACCGACATTAAAGGCCAAAATTCTTGTAGTGATTATTG
GCTTTTGTGAGAAGTCGTTTAAATATACGAAAATCAATTTTAATTTATGAATAATAAAGTTGAAAATTGA
ATATTTGTTAGGGAGTCACATCAAAAGAAAAAAAAATAGATTAAAAAAGATTGAATATTGACTTCTAAAT
ATAACCATTGACTTCAGACTTAGATAAAACATAGTTATATACTTTCTACTGTTATTTTTCTAGAAAAACA
AACTCAAATTTTGAAAACTAACATCAGTATTTGAAAACTTGTTTTTACTAATTGTGAAATTTGGAAATCC
AAATTGTTACTCAAGAACAATGAAAACTAGAATAAGAATTGGAGTGATAAAATAAGTTTAGTTTCCAACC
ATCCTAAACTAACAACAACATGGTTTCGTTTCCTTTGTCTAAAAAAACAACCAAATGGTTATCAGAAGAA
GTGTTACTTTTTTAAGTTAAGTTTTCTAAAAATTTAAAACCATACATGTGGACCAAATATAAACTCTACA
AAGAGACTTCCAACTTCAAGACTTTTATGTTCCCTTGAATTTTATTACGATGTTGATAAAAAAAAAAAAA

AAAAATGGGAGTCGTTAGAGAATACGATGAAATTGGAAGCTGTACTGTATCAATCAAATTCAAAATACAA
AGCCCCACTTCTTCACTGTGATGCCAACTGAATCAAATTATATTGAGAGCTTACGTTTCCTTCCACAAAA
AAAAAAAAAAAAAAAGATCAACTCTCCAATTTTTTGTTCTGCACTATTCTTCACAAATATTGCTATTGGTG
TTTTCGATTTCTCTTTTCTTTAGTCTGCGCGTTTCTTTCGATGGATTCTTCCACGGTTATAATAGAACCA
CCGACTTTCAAATGGAATTATGATGTGTTTTTGAGCTATAGAGGAGAGGATACTCGCACCAATTTCACCA
GTCATCTTGATATGGCCTTGCGTCAAAAGGGTGTCAACGTCTTCATAGACGACAAGCTTGAAAGGGGTAA
GCAAATTTCTGAAACCCTATTAAAATCTATACAAGAAGCTTTAATTTCTATTATTATATTCTCTCAAAAT
TATGCATCCTCTTCATGGTGTCTGGATGAATTGGTAAACATAATTGAGTGTAAGAAATCCAAGGACCAGA
TTGTTTTGCCAGTTTTCTATAAGGTGGATCCGTCGGATATACGAAAACAATCTGGTAGCTTCGGAGAAGC
ATTGGCCAAACATCAAGCTAAGTTCAAAACAAAGATCCAAATTTGGAGGGAAGCTTTAACTACTGCTGCT
AACTTGTCTGGTTGGGATCTAGGAACTAGGTATATATTTTTACAGACATTTTGTTTCTTCTATTCCAAAT
CTCATTTTTATGCTTTGTATGTATGTACGTACGTACATATGTATGTACCTATGTATGTATGTTTATGTGT
ATATATGTTAATGAGATCACTACCAAACAATTTTAATCTCATAAGATTCATTCTCTCTCAAGATTCAATT
TAATTAATTATGTTCATCATCGTTTAATTTTGGTATATGATTATGCACTTGACAACAGGAAGGAGGCTGA
TCTTATTGGAGATATTGTTAAAAAAGTGTTATCTACATTAAATCGCACTTGCATGCCCTTATATGTAGCT
AAGTATCCAGTTGGAATTGATTCTAAACTAGAATATATTAAGCTTCGTTCACATAATATGTTTGAGAAGA
ACAACAAATTCCATTATCGGACACAACATGAGTATGAGTTTGATACTGGTATCTACATGGTGGGGATATA
TGGAATTGGAGGTATTGGTAAGACAACTTTGGCTAAAGCTTTATACAACAAAATTGCTAGCCAATTTGAA
GGTTGTTGCTTTCTATCAAATGTTAGAGAAGCTTCAAAGCAATTTAATGGCCTTGCTCAACTACAAGAAA
GCCTACTCTATGAGATCCTAATGGTTGATTTGAAGGTTGTCAACCTTGATAGAGGAATTAACATCATAAG
GAATAGATTGTGTTCGAAGAAAGTTCTTATAGTTCTTGATGATGTAGATAAGCTTGAGCAATTAGAAGCA
TTGGTTGGTGGGTGTGATTGGTTTGGTAAAGGCAGTAGAATCATTGTGACCACAAGAAATAAACATTTAC
TTTTTAGCCATGGCTTTGATGAAATACACAATATTCTAGGATTGAACGAAGACAAAGCTATTGAGCTTTT
TAGTTGGCATGCTTTCAAGAAAAATCGTCCATCAAGTAATTATTTAGACCTTTCAAAACGTGCTACAAGT
TATTGTAAAGGCCATCCTTTAGCTCTCGTTGTTTTGGGTTCTTTCCTCTGCATTAGAGATCAAGCAGAAT
GGTGTAGTATATTAGATGAATTTGAAAACTCTTTGAACAAAGATATCAAAGACATTCTTCAATTAAGTTT
TGATGGTCTCGAAGACAAAATAAAGGATATCTTTCTTGATATTTCTTGTTTACTTGTGGGTGAGAAAGTT
GAGTACGTTAAAGATATGTTGGGTGCATGCCATGTAAATCTAGATTTTGGAGTTATAGTACTCATGGATC
TTTCGCTTATTACGATTGAAAATGACAAAGTGCAAATGCACGATTTAATAAAACAGATGGGTCAGAAAAT
AGTTTGTGGTGAATCTCTTGAGCTTGGAAAGAGGAGTAGGTTGTGGTTGGTGCAGGATGTTTGGGAGGTG
CTTGTTAATAATTCAGTGAGTAACTCTTACCTAAAGTATCTTTAATTTACTTATTTCCAAGAGTTGACAT
GATGACGAAGGTTTGTCAAATGTTGTTAAATTACTAAACATATGACTTTTGCAGGGAACAGACGCAATTA
AAGCCATAAAGTTGGACTTTCCTAATCCTACAAGGTTAGGTGTGAATTCACAAGCTTTTAGAAAAATGAA
AAATTTGAGATTGCTTATCGTTCAAAATGCAAGATTTTCTACCAAGATTGAGTATCTACCTGATAGCTTA
AAGTGGATTAAGTGGCATGGATTTCCTCAACCAACTTTGCCTTCATGCTTCATTACGAAAAATCTTGTTG
GACTAGATTTGCAGTATAGCTTCATGAAAACATTTGGGAAAAGACTTGAGGTAAGTTTTATTTCTATATA
TGCCATAAATGGGTAGCTTACATTTTAAAATAATTCACAAATGCGCAAATCTATAATAATAGTTTTTTTT
AATATAAAGTAAGGTGAGAGATAAGATATCTGAAGTTTATACGTTTGTGGAGTTCATATATGTTTAGGGA
GTTTATATGTGTGGTGGAGAGTTGTTTTTTTTATGTTCATAGGCTTGTTTTAAGTTGTAGAGTTGAGTTC
ATAAGTTAAAGGTATTTTTGAATTATAAATAAGGAAAATTGTAAAAAATGACAAATTTGACAAAATATTT
ATAAAATATAGCAAAATTTTAGATTCTATCACGATAGACATTGATAGACACTAAATGTTTCTATTAGTGA
CATTGATGAACAGTAATAGAAGTCTATATCGATAGAATCCAAATTTTTGATATATTTTGTAAATATTTTA
CTATATTTGAAAATATCCTTATAAATAATATGTTTTTTATAACTAAATATTTTTATTTTTAAATTTATTT
ATCCAATAATTCTACTTATCTTTGTTTGAATAAAACACGAATTACATTTTTTTATTTAGTTTTTTTTAGT
TTTAATATTTTTTCTTTTTGATAATGAACAACTATTAACCCTATATTTTTGGCAGTAACACAGATAAATA
AAATGTATAAAACAAGAGAGAAATCAAAACTTTTGATTGAGTAATTTTTCTTCATGAATTTCATCAATTT
TCTTCTCTTCCAAGTTGGGGCCAACTATATTTTTCCTCTGTCACGATTTTTTTAATTGAATTTCCCTCAT
CATCTTTGCAACCAATCAAATGTCTCACAACATTTCTTCAACAATTTTGTATTCAATTCCTCTAAATTTG
GAGGATAAAAAGAACATATCTCAATTTTATTAACTCTTGCTAGAAAATGTGTTAAAAAACAAATATTCAT
TTTATTAGTTTTTTTTTAGAAGTTTACGTAAATGTAACAAAAACAAAAAATATTTACGGCCCGTATAACA
AAATAAGAAAAGCTCATGACGTGCAACAAACATGTTATCATAAATTTCATATATACCCTTGCGATTTATA
TCTTCTTTTATAATTTATTAATCTCGGGTCATCTTGGTGTAATTAATGCCCAAGATTGAATAACTAAAGG
CAGAAAGTTTTTCAGCTCGGAGCATCTCAAGACATTGTATGCCCCACAAGTAAACAAGTTACATTTTAAA
CATTTCAAAACAAGTTGAGTGCACATAGTATCAAACTAAGAGTGTAGTTCTTTGTTTATATATATAGAAA
TATAACAACTTTCATTTGGACAAAAAATGAAAGAATATAAAGGCATCCAAAAAACCAAGCCACCAAAACA
CACCGACTAAAAGAAGGGGTCCAACGAACTAAAATGTTATCAACGGAATAAATATAAAAGGATGTAGAAT
TATCGAACCTAGCCAAAAGAGAAATGGTATCATATAATTTTTGGAAGAGGATAAATGATAAAGATGCGGA
GATGAAGAACAAAAAGGATTTCCCCAAGACTTCCCCATCCATGAATTTTCACAAAAATACGCATCCTTAC
CCTACCCTAGAAAGCAACGAGTAAACTGAGAAAAAGTCGGAAGCTCAAAAGAGACTCTTTCCAAGAATTA
CGGTAAAAAGTGTGTTTTGTTTTGTTTTGCTTTTTTTTTTTTTTTTTTTTTTTTNNNNNNNNNNNNNNNN

NNNNNNNNNNNNTTTTTTTTTGAAAAAGTTACAATGTGTTTTGTCGTTATTTTATGTAGTATAATAGTTGT
AGATTAGTGATTAAGTGAATTAATGCAAATAGATGATTAATCTTTATATGAATTGAGTTTATTACCGATT
TACTTTTGTATATGGAGTTTTATTAAGTTTGCCTAAATATTGATTAATGGTTATATTATACTATTCTTTT
TACCTATATTCTATATTTTCTAAATTTACAATTATTCTTTTAGCCACTGTTGTGCCTAAATTTTGTTTGG
TATGCCAATTACATACCCCCTTTTCCTTTTGGTTTGTTTTTCAATGTGTTTTAGGATTGTAAAAGGTTGA
AGCATGTTGATCTTAGCCACTCTACTTTTTTAGAGAAAATTCCTAATTTCTCTGCAGCATCAAACCTTGA
AGAGTTGTATCTCATAAATTGCAAAAATTTAGGAATGATAGATAAATCTGTTTTTTCTCTCGATAAGCTT
ACTATCCTAAACCTCGCTGGTTGTTCTAACCTTAAAAAGCTTCCAAGAGGCTACTTCATTTTAAGGTCTC
TTCGATATTTGAATCTCTCTCACTGCAAAAAGCTTGAGAAAATTCCAGATTTTTCTGCAGCATCAAACCT
TGAAGAATTGTATCTTTTCAATTGCACAAATTTAAGAATGATAGATAAGTCTGTTTTTTCTCTTCATAAG
CTTACTATCCTAAACCTTGATGTTTGTTCTAACCTTAAAAAGCTTCCAACGAGCTACTACAAGTTATGGT
CTCTTCAATATTTGAATCTCTCTTATTGCAAAAAACTTGAGAAAATTCCAGACTTATCTGCAGCATCAAA
TCTTCAGAGCTTGTGTCTCCACGAATGTACAAATTTAAGACTGATTCATGAATCTGTTGGATCCTTGTAT
AAGCTTATTGACATGGACCTTAGCGGATGCACTAACCTTGCAAAGCTTCCGACCTATCTTAGGTTAAAGT
CTCTTCGATATTTAGGACTTTCTGAGTGTTGTAAGCTTGAAAGCTTCCCATCAATTGCTGAAAATATGGA
ATCTTTAAGGGAATTGGATATGGATTTTACTGCCATAAAGGAGTTACCTTCATCAATTGGATATCTTACT
CAGCTCTATAGGTTAAACCTCACCGGTTGCACAAACCTCATCTCCCTTCCCAATACAATTTATTTGTTAA
GGAATCTTGACAAACTTCTTCTTAGTGGGTGTTCTAGATTTGAAATGTTTCCCCATAAATGGGACCCAAC
CATTCAACCAGTATGCTCTCCTTCAAAAATGATGGAAGCAACTTCGTGGAGCTTAGAATATCCCCATTTA
CTACCAAATGAAAGTTTGTGTTCCCATTTCACTTTGTTGGATCTTCAATCTTGCAACATATCAAATGCAA
AATTTTTGGAAATTTTATGCGATGTTGCCCCTTTCTTATCTGATCTACGCTTGTCCGAAAACAAGTTCTC
TAGTTTACCCTCATGCCTCCACAAGTTCATGTCCTTATGGAATCTTGAATTAAAGAATTGTAAGTTTCTT
CAAGAAATTCCAAACCTTCCCCAAAACATACAAAATTTGGATGCCAGTGGTTGCAAATCGTTGGCTCGAA
GTCCAGATAACATTATGGATATAATATCAATAAAACAGGTTTGATTCTTTCCATTCATTTTGTTCCTATC
TTTGCACGTAGACAATTTAATGCATATGAATTCTTATTCTCTATAGGACCTCGCAATGGATGAGATTTCA
AGAGAGTTCTTATTAACGGGCATTGAGATTCCAGAATGGTTCAGCTATAAGACTGCATCCAATTTGGCGA
GTGCTAGCTTTCGTCACTATCAGGATATAGAAAGAACTTTGGCTGTCGGAGTTATTTTCAAAGTGAATGG
AGATTCATCTGAAAGAGGGGTCCGAATTTCATGCAATATATTCATCTGCAACAAACTCCATTGTTCTTAT
TCAAGACCATTTCTTCCATCAAAATCAGAATATATGTGGTTACTAACAACTTCTCTAGCGTGGGGTTCCA
TGGAGGTGAATGATTGGAATAAAGTAATGGTATGGTTTGAGGTTCATGAAGTACATGGGGAGGTTAATGC
AACTATAACAAGGTGTGGTGTCCATGTAACTGAAGAGCTCCCTGCGATACAAACAGATGCCAAGTGGCCG
ATGGTAAATTATGCTGATTTTTATCAACTGGAGAAATTGCAAAGTCTGTAAGTTGATTGTTTACTTGTTA
CTTATTTACTTTTTTTTTTGTAGTAAAAGTATGATAGATCTCAAAGGCGGAGATGTGTATGTTATGATTA
TGTGTTTGTCTAATGGCATAGGGATATTGAGCATCTTCTTCTGAAACGGTTTTTTGAAGAAATGTCGTGC
TGGTCAAATTGCAAAGCAATAATGTTCCATGCAGCAAATTATGATCCAGAAACAAACTCTGTATGCCACA
GATTTAATTGGTCAGACGACATTGATTGGAGACAACCTTTAGATGATCCTATAAGCTTTTACTGGGTCCA
AGAAAGACAGTACCGTTTCATGAGTTATTCGGGACTCGACAATCGTGGAGGTGGTGAAAAAGTGACCAAT
ATTATCACAAATAACCACAGTACAATTTTATCCTCCAAAAGGTATTACATATTATACTTTAAAAATTTGG
ATGATAGAGTATATAGATTTTTAACTGCATGGGCTATAGCAAAGCCTCGTTGGATTGAGATTTACAGATA
TGATCATGATGTTGCAAGAAATTGTCATTTTGTCATTAAAAGGGTTGATCCATCCTTATGGCAACCCTGG
GTTTGATCCACCCTCAAATTAATTTTCCAAGTTTGTGTTGCAAATGTTAAAATTACTTACCAGTGTCTTT
AGAAATGGTAGAAGGGGGAATGTTTTACTTTGTGTTAAACCACAGCTAATTTTTGTTTATCTATACCCCT
ATGTATTATATGTTTACAATCTAGAAACAATGTTGTTAGTTAATTTGTGGACCTTCTTCATAAAAGATAT
TTATTGGTTTAGAAAATATTTTATCAAATTAAGAAAATTTTCCTTATGAGTTTCTTTTACAAAAATATTA
TTTTATTTGGCAAAAATAATCTTGTTAAGGAAAGATTTTATATGGCAAAGATTTTATTTTTCCAAATTAT
ATGTGGCGTTTTCTCCTATATATACATCCGGTTTGAAGCACGGACGCTTTGGGTCAAGGACCCCCGGACG
TAGTAGATATTGTGTCATCGAACTGGGTTAACAATCTTGTATGTTTAATTTGATAACTTTTTCCTGATCC
AGTAGACATATCTTCTGTTGGTTAAACTGAAGGTTCATTTGATTTATCTTTGTTGACGCTTTCCTTCTTT
TATTCAAGAGCAATAAAATGAAATTGAGATAAAAAATTGTTTTATTATTCCATGTGTAAGCATAAAGAAT
CCTTAAAGATATTTATCTAAAGTTAGACAAACATTATTATAATATAGAAATGGTAAAATTCTAAGGGTAT
GTTTGGGGTGGGGTTTGGGGGGAAAAGAGTTAGGAAATTAGGCCAAATTACCGTGTTTGGCCCAAGGGTT
ATGATAAATGGAGATAAGGGTTATCCTAATCCCTAAATTCCAAGGGTTTTAAACCGACATTAAAGGTCAG
ATTTCTTGTAGTGTAACTATCAGAAAACTATGCTGAATGTATGGGAAGACTCTTGCAACTCAGTAGGCCC
TCTGGTGCAGTAGAAGTTCTTTGTTTAAGAATTATAATTCAAGCTTGATTGCTCTTAATTTACAACCTCT
ATACAACAACTTCGAGCTTCTCTCCAGCCCCAACTTTATTAATAATATATGTAGGTTGGTGTTTCTATCG
GTCCCTTGGTAGTTTCACCTTCCATTTGTTTCTGTCAATATATATATATTATTGAAAGTGTGATGTAATG
GTGAAAGAAATTTCAATTGTAAAGGTGCATATGTGATAGCTAGATATATTTGTTTTCATTTAGTCGTCAA
GATTATTTCAAAATAATAGTTTGATTAACTACTTTTCTTTTAACATGAAGATTAAAATGTAACATTGTGA
GAAGCAAAATAATAATAATATCTATGGTGTATTACACATCTACAAATATCGATTTTTTCATCCAACAATT
AATATTTTGGTCTTTAGAGGAATCGCCAAAACCTTACAAGCTTGAATTTTTTCTTGTAGATTTAAATTTT

TCTTAGTGTAGTAAGCTTGAAAGCTTGCCAACAATTGATGAAAACATGAAATCTTTAAGGTTCTGGATTT
GAGTTTTACTCCAAATAAAGGAGCTACCTTCATCAATTGGATATATCTAACTGAGCTCCATTAGTATATT
AGCTAGCCCTTACAGTTTTTCACAGAGAGCAAGAAGTTGTAGATTGAAGAGCTTGGTTACGTGAGCTGAT
GCATCCAAATTCATCAAGCTTTGATGGGTCAATTCCGCAAAATTAAAGTACCTCTTCCTTCTCGAAGCTC
GAGAACTCTTGCTTGAGAAACTGATTCACGTGAATCCCAAACGAAAAAGGGCTAACAAAGGTATAGCAGT
TGTTAGGGGTGTAATTGGGTCGGGTCGGGTCGGGTTTTCTTCAAAACCGACCCGACCCGAAATGTACGGT
TGATTCTGAAACCCGACCCGACCCAACCCAACCCACATTCCGGTTGGGCCGAATGGGTCGGGTCGGGTCG
GGTCTTTTTTTTTTTTTTTTTTTTTTTTTTTTTTTTTCCTCCTTCCTTAGCAAGGAGGAGATTTTCACAAG
GGGGAGAAAATGTTCCGGGGAGTGCATCATTTGAACAATACTTGAATAGAAATGTAGATCATCATAACTT
TTTGTAAGTGCATTTTCCCTATGTTAGTACATACTCATCAAGAAGATGTTACCATGAAATCAATTCTATA
TCCATAGTTGTCATTCATTTTGGTCCTTACTTGTCCTCCAAATTATCCAGATCAAAATCAGGAGTCCCTA
AAGAATGGGATGATGGTGATAAACCAGCCCTTGCCATTTGCTCCCTGAAAAATCGTAGCTTCCGAGCCAT
TTCCCCACATCTTTTAATCTGCTTGGTAAAAGTAGCAGGCAAACAAACACCATCAAACCAAGCATAAGAT
ATTTTGTTTACAAAAACTGCACATAACTGCTGTCAATTAAATATTAACCGAAAATAGGAAACAAGAAATG
AGACCAAAAGCACGGATATACCGCACTACACTTGCTACATCAAGGTCGTCTTTACTTATTACAAAGTGAA
GGATTAAAAGTTTCATTTCACAGTATAACTGATTAGGGAAGGGAGCTAATTCAGAAAACTGACGGTATGT
CTGCTAAAATTTTTGAAGTTCTCTAAGCCATTGGAAAGATCTCCTCAACAGATGAACGTAATGGTACTAC
ATAAAATCTTAAATTAAACTGCCTTCACCTTTATGCCACAGATACAGCTGCAACTACAATACATCAGTAA
AGCCCATTATCAACTATGGGATGAAGTCTTGTGTAGCAATTATTCTGTAGGAAAACTTATCTTATAAGCT
ATGACACTAACAGCACAAGTATAGGCAATTCAAGTGCCATCAAGAGGAAAGAGCAGACGGATCCAAGCAG
AATCCAGAGCCACAGTTATGTGCACGAGAAAGACAGGAAAGGCACAAAGAAAAATGAAAAATACTAAAAT
GTTATGTTCACACTCTACTTGTAAGCATGCTGCAGGGAATCTATAGTATTTATTTAATATTCAAGAATGC
TATAACAATAGCTGGAGACCTGCAATAAATAAGTGTAACCTATTAGAGAGGATGGTTAAACAAATCTAAA
CTTCTGTCATATGACCATTTTTTCTTTTGATGCCCAGTCGACTGGGAATACATAATTGCACAGTTCCAGC
TTTATTTAAGCATATGGGAAAGGATAACAAATACAACTAATTCAAACCTATTTTTTCCTGACATGCAGTG
CACCAGAACCCGAGCCTTGTCTCTTTCACATTGCTCTGAAAATCACAAGCAATATCAGGAAAGTTAGACT
TTTGGAATAAGTGAATGATAGAAAGCTAATGACACGTTCTTAACCAAAAGTCATTTGTTCAGTGTCATCT
TATATTGCATTGAAGTAACAACCCAGGGAAAGACTAATCTGCATTGCTTATTAAAGTCACATTTGAATGA
AGAATGTTGCAACACATGGACTAACTTAATAAATGAATTAGCAAATATAGTTCATTTGGGAGAATATAAA
GAAAGGCCACCTAGAAATTCAACTGCGTCATCAAAAGGTAAAGTCTTATCATATTGGAGACAGTGATACG
TGAAGGAGTTCTTGTAGAGATTTTGGCAAGCAGGCACAGTCTGCAAAAATAGTGTGGAGAAAACCAAATG
TCAGTCATTTATTTTCAGATCACCCAATACATTTTAGAGGCAAGTGATGATGCATGACTTCTACAACATC
CGAGAGAGATAACCATGATATGTATTCTACAATGAAACTGCTTTTTAGTATAACCATCAACCCAATAAAA
CTTATTTTTATATCTACGAAAGCCAACAACTTGATATCATTGCATTCAATATAGATTATACATCATAATA
GATTAGAAAAATAGTTATAAGCATAAAATACTCAGAGTCCAAGGTAACCTGAGTCTCCAGGCAATTCATT
CTGGCATTTGCCATATTACCAAAACCTTAATAGGCTAGTAGTACAGTCCATTTACGGCAATTATGTGCTG
GTGCTGTTATTTGTTAGTATCAGAGCTTTCAATTTATAAATAAAATCCATAAGCAGGCTAAAAGCAAGAT
TCCAAGATGTAACGAAGCCGCAAGCATAAATCAGAATAGTAGTCACACCTGGGCAGCATACGTCCGTTGG
AATGGAAATTACATTGGCCTCAAATATTCAAGTAGTGAACTTAAGGACTTTGACAATTACGAATAAAGAA
GCAGAACCTGACAAAATGTGATCCAGGATCTGTAAATATACCTTTGAAATACCATATCCCTATTGCTTTT
GCTTCCTGTCCCCAACTTTGAACCCAATTCATTCTCTTGGGTATTGCTAGATTTAGCAGAAACAACATCA
GCTACTTGATTAGCCACCGGAGCCTTGCCAACCTCGCCCGCATTCGCTAAATCCATGTTTCAGATAGATT
GAAAGTAGAGAGTATAAAAGATAACAAAGGAATTTGGAAAACACTGGGGAACTTTTACAAACGCATGGAA
GACAATAAAAACCAAACCAATAATCAGTAAGTTACCGGTTCAACGAAACCACCATGAAATCAACTAAAAC
AAAAAACAAAACAAACAAACAACCAATAATCTCTCAGCCCTTCTTTTGTTTCTTTTATATAAAGAAGGTT
AACCAAGGAACCTCATGAACAAAGTTTACAATTGGGAAGGTATGAATTAATGAACATCTTCACGAGACCA
CCTTGTTCGTATAACCCAAAGTGCTTCCATCATTGAGCTTCAAGACTCTAAAAAAACAAGATTCGATATT
TAATAAGTTAATAATTTTAAAAAAATGTAAATAAAGATTTGAGAGTAAGGATTTAAACCATAGAGTCATT
ATTCAAATTCTCAAATGCAGCTTCCATCTCCTTTCCTATGTTTATGAATTCTAGATCAAAATGACAAACA
GTTAGCTTATATGGGATAATTGAAAATTTTAAATGCGTACTTCAAAAAGAATATTACCTTCATCAATTTC
TTCAGCCCCGTCAATTTCTTCAGTCATGTCATCCAAAGGTTTAGACTGAATCCAATTCTGAGCACAAATG
AGTGCCTCTGCAGTTTGAGGAGTTAAAGAACTTCGAAAAGAATCTAACACCCGTCCTCCAGTGCTAAAGG
CGGACTCAGAAGGCACAGTTGATATAGGAATACTGTAGATGTCCCTAGCTACTTGGCTAATGATCTTAAA
TCGAGAGGCATTCACCTTCCACCAAGTTAGCAAATCTAAATATTCATCGCCCATACAATCTATACGAGCC
TCATCCAGATAACGAGTCACCTCTGTTTTAGCATCATCTAGACATGTTTTGTTACTTTGTTTAAATCTAT
CATGAACAGTAGCACGTGCCTTGTAAGATCCACTAGATGAGATAGAAGGTATTTCACTTTGACTTTGAAA
GCCAAATCCTTCGATAGGTGTACATGATTGTGTTTGTGAATATTTTTCTTTTGACATTCTCATATAATAA
TCATCACACAATCGACGAAATGCTTCTTCAACCTTATTTGTCCATATTTTTGCACAATCTTCCTCCAAAA
ATTCATTAAAACAATAATTCACATAAGCTAGCTTGTATCTAGGGTCAAGAACTACAGAAACATACAATAA
TAAATTGGTCTTCTCACTTGTAGTTATACCCCAATACTTGTTGAATTTTGTCTGCATGCTTAATGTCATT

TGACTCAATAATGCATTCTCATACGATGAGTATTCACGAATTATTTCTTGGATCAAACAAAGTTCATGAA
AAAATATATTTGAAGTCACAGACATAGATGCAGAAAACTTCATTGTTACCTCTGAAAAAGTCTTTAGGAA
CTTTACAAACACTTTTGCATTATCCCAATCTTCAGTAGTAGGAATATCATCCTTTGGCAAATAACTAGGG
TCATGCTCCTCCAATCTTTCAAAAGTCTTTTGACACTTAATTGCTCCATCCAACATAGTAAAAGTAGAAT
TCCATCGTGTCGGAACATCCATTGTAAGACAATTTTTTGTTGACATCTTATCTTCTTTAGCAAAATCTTT
AAATATTTGCAATCTAGCAGGAGATGACCTAACATACTTCACAGCATTTCTGATTCGAATGATAGACACA
TGCAAATCTTTTAAGGCATCACTAACAATTAAATTAAGAATATGAGCACAACATCACTAACAATATAAGG
TTTTTTTCACCCCTAGCAGTTGTAGAACAGTTGAGCTTGTTGCTGCATAATTGTGACGATCTGAACTTTT
AGATTATGTTAAGGTCATTACTCAAAAAGATGATAACAAATTACACTTTCTTTAAAGAGACAGACGACTA
AAATTCTTATAAAATCAAGATCTTCATAAAATATAAGATTATCAAAATCAACAAAAATAATTTGAAAACG
TGACATGCGGGTTCTAACAATAAAAATATGACAAAATCTTAAGTACAATAGTTAAAATTTAAAATACTGA
AAAAAAAAACATAAAAAAATAAGTGCGGAAGCTGAACTGTGTCCCCATATGGCATGTCACAGATTCCTTT
CTGTCGCTCGCTATCTTCTTAAGTTCCCTACCTTAGCTTGAAATATTAAATATAGAAACGAGTGAATATA
TAAAATATACTTAGTAAAGGACCCCCTACTAGTCTCGCTAGGTGATCTGTTAACTTTTCATTAGAAACAT
AAACGTAACGTCGTGTGTCCAACGGAGTACACCTAAGTGAGTGAAACCATACGAACACCCCTAATCGTGC
GAGTGATCCCATGGGAACACCCCCTAGTCGTGCGAGTGATCGTAGGTACACACTCCATAAACATATATGT
AATACGTAAATACACCATTAATCGTGTGAGTGGTCCTGTCAGAACAACCCTAGTCATGCGAGTGACACCA
TATACATAATATAATTGTCCCCTAACCGTACATGTGATCTGTAGGTACACCCCTAAATCGTGCGAGTGAT
CCCGTAAGAACACCGCTAGTCATGCGAGTGACCCTATAGATAGGATCACAATACAAGTGAGGTAAGAAAC
TTAACATACAAAGTTAATAGACACACCATAAACCAAAGGCATGTGACATCATCATAAACATCATAACATA
ACATGAATATTAATCGTAACGTCTTAAGCATGTGATTAATATATCATGTATCATAGAAATCATATACAAT
ACAGTCATCATTAACAACATAACATCAGTCATCGCCATCAACTTAACATCAATCATTATCTACAGCATTA
CATTATGCGTCTTAGCTACCCTCAATGTATAGTCGTAATACATGTTGTCTCTTAAATTCAGTTCGAAGGG
CTAGTAGTAGAATCTCTTACCTCAAGATTTGTTAAACATTTATTCCCTATCTAGCACAGTCGAAGCTTTC
CAATAGACAAATCCCATAGTTGAGGTTGAAACCTTATTGGGAGAATTCCACATACTATTTTTCCAAAGTG
CTATCCAAATGAACCTTAAAGGGCAAAATCCAAATTCAAATTAAATTAATTGGAGTCCAAAATTTTCATT
AAATGGGTATTACCCAAAACCCATTAAAACTTACCAAAATAGGTCCAAATAGGTGGAAAAAGGACAAAAA
TCTAGTCTAGTGGCTTAACAAAGGGAAAGGTGGCTCACACGTGTGGCTCAACTGAAAGGATGGTTGACAG
CTCACGAAGGGATGGCTCAGGTAGAATTCGACTAAGGCGGAAGACTCGGCTCGGACAACTTGGCTAAAAG
TGGCTCGAATATACGATGGCGAAACAACGACTGAACGAACGACTCGTCGATGGTGCGGCGTCACTTACGA
CGGTGAGACGGAAACGATCGGCTGATGAAGAAGCGGCGACGTCGACGCACGAAACCAAACTGTGACGGAT
GGAGAAACTCGACGAAAACAACATGGTGGCGAGTGATGATGCGGCGGAAGCTTTGACGATGATTGCGAAC
TGAGGAGGACGCTTACGGGCGGCACGTGGTGGAGGGTTCGATGCCCGATCGACGACGACCTTCTTGCGAT
GAAGAGTTTCGGGCAACCACAACAACACCTGTTTCGAGCAGAGCTTCATGGCGGCCTACTACAAAGAAGG
AAAACGCGAGCGGCGGCGGTGGCTGCAGTCGACCGGAGAGGCGCGGCGGCTAAGGTTAGGGTTTTGTGAT
GGTTTCTTAAGAATGAAGAAGATGAATGAATAGTAGATCACGGAAAACGAGGCTTTTATAAATAAGAATA
ATAATATTATATCAAATGCTTGCTTACGGGCGGCGCGTGATGGAGGGTTGTTGCTTTCTATCAAATGTTA
GAGAAGCTTCCAAGCAATTCAATGGCCTTGTTCAATTACAGGAAAGCCTACTCTATGAGATCCTAACGAT
TGATTTAAAGGTTGTCAACCTTGATAGAGGAATTAAGTACCTACCTGATAGCTTAAAGTGGATTAAGTGG
AATGGATTTCCTCAACCAACTTTGCCTTCGTGCTTCATCACAAAAAATCTTGTTGGACTAGATTTGAAAC
ATAGCTTCATAAAAACATTTGGGAACAGACTTAAGGTAAATTTTATTTCTATTCCTTACATTTTATAATA
ATTCATAAATACCGCAGATCTATTATAATGCTTTTTTAATATAAGGCTTTTTTTTCTTTTTCTTCTTTTG
GCAATAAGTAGGGATACGATGTTTGATGTTTACACTACAAGAAATCTGACCTAAAACCGACATTAAAGGG
CTTTAATGTCACTTGACAACCGACATCTTTGCGAGCGTTATTAAAGGCCTTTAATGTCGGTTGGGCTTTA
ATGTCGGTTTAAAAACGACATTAAAGGCCTTTAATGTTAGTTTTCAACCGACATCTTTGATAGTGTTATT
GAAGCCCTTTAATGTCGGTTGGGCTTTAATGTCGGTTTTAAACCGACATTTTTGATAGTGTTATTGAAGC
CCTTTAATGTCAACTGGGCTATGATGTCGTTTTAAAATCGACATTAAAGAGGCCAATAATGTCAGTTTAA
AACCGACATTAAAGGCTTGTTTTTGTCCATTTTTAGAAATTTATATTTATTTAATTGTTGTATTATTGTC
CATTTTTTATAAACCAACATTGAATCCACATAGATAAATATTTTTTTCTCGCTTCAACAAATTAATAAAA
TTAATATTATTTTAGAAACTATAATATTTATCTTTTAAATTTGTATACACAAAATGAAAACTTAATATTG
TGTTTATATATACATTCTACAATAGTACATGAAACAAGATCCTAATACAAGACTTAAATAAGAAATTCTA
AACGCCTTCTCAGTCTTCAACCTTCAACGATCGGCTGGCTATCTACACAATCGCTTAGCTTTCAATCTGA
TGACTTCAATCATTCTACAAGCAATATCAAAATAGACCATCTAATCTGATAACTTTTGATTTCTATCATC
TCCATTATTACAAACAAACATGTCAAACATAGTAGTAAGCAGTTCTATCACTAGAAGAAAACCATAAACA
TACCCAAACCCAATTACAAAAACATTCAAACCCACTCATAAAGAAAAGTATATATCAAAATAATTTTTGG
AATATGTAGAAACTGCCATTGTACACATTCAAATGTCTATCTCCCAAACATTTTTATATACAGAATCATT
TCCCATGGTCGATTCCTTGCATCATATTATTTAAATAACTGCCCTGAACACTCTTCCTTGCATCATATTA
TTTAAATTTTCAAAGAAGAGAAGAGAGGAGAGTTTTCAGCTGGATCTTACAGTATCAATGGCATGAACCA
AATCATCGAACTTTGCTCCAGTTACTTCTTTAACGACCTTATAGATAGTGTAGACCGTTTAGCTGAGCAA
AAGATGTTGGTATCGGACAAGAGAACCGGTTGTTTGAGAGATCTAATGTTTGAAGCAATGGGATAGTTCC

AAGTTCAATGGGAATTGGACCGGAAATGTTGTTATTCTGCAGCAACCTTTAAATTTGGATAAGAAATAAA
ATAGTAAGAAAAACTAAACGTAGTAGATGGGAGAACACACAAGAAACCCAAGAAGGCATAACTCTTAATT
ACGATCTTCGACTAAACAGAGCAAAGAAACCCACACTTCCAACCAAAATAAATTTGAAACTCAAAAAAGA
TTTCATATCACTTGCTTCAATTCTTAGAATTTTTTTTAAGAACTAATCATCAGCAGCAACATATACTGTT
GTGACTTATTCTAACATATCACACACACATATATATTGTAACTTACTTTTCAGATCAAAGCTTTCAATAG
AAAATATATCACAAAATCATTTGACGTCAAGAACTAACCTCAGTTAAAGAAACTGTTAGAGAATTCAGAA
TATCGACCTATTCCTCTTACAAACTAACTAATTCAATAACAAGTGAAATTAAGCATTAAATCTACACAAA
TCCATAGGAAGAAAGATGATGTCTTCAACTACTATCCATTTACTTACGAGAATACCTGCAGTTGACCCCT
TGCAAGGGCAGGTTTTAATATTTTTGCTGCATCAATAGCTCCTTCTGCAGCCCCTGCTCCATCACTTTGT
TTTATTTCCTCCATCAGTTTCTTTAATCTTTCTTCAAACTCCCCACGATATTTTGTGCAAGCTACAAGAA
GGCCCATATCCAAGGTTATAACCTGCCCAACAGATGATTCTAAAAAAATTAACTCAATAACAAGTGTGCC
AACAAATGGAGAAAAATAGCCAACAAAACCAAATTAAAAAAGAAAGAGTGTAGAACGGGTTAAACAAACA
AAATGGTTCCTTGATTTGTATCAAGAGAACAATTACCAAAAATAAATATCATACAGAGTTTCTGTTCCTT
GTCTTGTCTGCTGCTAGCAAAGGGCAGCCTACTTGTATACTTCTGTTTGGTAGAAATAAATACTGTCTGC
TGCTGCTAACAAGCATCTTCATGAAAAGGATCTATACTTCTTTCATCCTTGGAATTATTTTCAATATTTG
TACTATCAAAGACAGCTCCAGTATTTACCACTCCATTTCTATAAAGAATATCTAATTCTTGTAAAATATG
GGACATGTCTTACCATTTGCAATACTAGCCTTCTCAGTTACAATGGTCCTTTTGAAATATTTGTTCATAA
AACTAGCTCATGAAAATTTCTCATCAAAACTTTAAGATAAGAAAGTTATTTACTATAGTAAGAACTAATA
AATATAGCTACAAAACTAAGTTTAAGGTCAAGTCTTATAAACATTACAACAATTAACTCCATCAAATTAA
CAAAACAAATTAAAGTCACCAAGTAAACCAAACAAATTAAAGACTTCACTTACATAACAACCAACATAGT
TATTACTTCATTTACACGGAGAAGTAAAAAGCCTTTAGAAACCTAAAACTTGTTTACATTAGAGAGTTCA
TCAAGATATGCTAGAAAGGAATCAGCCAAAGTAGCCTACAAACCCAAGAAAAGAATCTGAAAATTAACAA
ACAAGATAAATGACAATATCTAAGAAGCATTCAAAACAAGAAGCACCATACCAAGTCGAAAAGCCTAATG
AAAGTACATTTTGTGAATTGCTGGTGAGGCATAACGCACCAGATGACTTAACAATTGTCGCCTCATCAAG
AAACAATCCTTTTCCTTCACGAAATCCATTGTATAAAAATTTAGAGTCTGCAAAAAAATAAGGAGGCATA
ATGAAGAACAACAGTATAAGAAAAGTTAAGGGAGCCATGTTTTCGCTTTGGAAGAAATGAAAGCTGAATT
TTCAGTTTTAGAACAACAGGGTTTCTTGATTTTCTTTGCAATTTGTGTGATTGAATTGGAGGCTAAAACA
AGAATGACCATACTGACTTTGAAATTGAAAAGTTGATAGTGAGCAAATTTCATGGAAATGGGCAAATGGG
AGAGCCGTGTCCGGCAATGAAATGGTTTGATGGGGAAATTATGAAATATGAAAGCCTTTGAACAAATGAA
ATAATCCCACGTTAGAGCAAAAATATCTTTGATGCTATTTTATAGTTTTGCACCCAAATTAAAGCCTCTG
CTTTCTCCTATGTCCTCATGAGTAGACATTTCTCCCCTAGTTAATTCCAATTTTCAAAAATAATTGAAAA
ATTATGAATTCCAACTACCCAAATTAAATCAATTGCCCTAAAATCTCAAAGAACCCAAAAAAATTGAATA
AAAATGGACAAGAAAAATAAGGATATCATCAATGAAAAGAATAGAAATATCGACATACCCAATTTTGAAG
GTCAAAATTAATGTTTTAATTGGCATATCCACCCCACTAACAAAATAGTAATTTGTGTAGATTGTCTCTC
AATAGTTTAAACTACAAAACCTTCGCCCTAACAAATCTGTCTTTCGTTTCAACTCGCCAAAATCAAACTC
AATTTACAGTACTTATCCAAAAATCACCCGAAACAACTTTTACCCTTGCTGGACAGCTCGTAGAAAACCT
CCAAATTCCAAATCTAACAACATAGAGAGGCAGAGATGGAAAATGGCTCACCGTAACGTGGGACGGAGGT
GCAAGCTGTGGCTGGGCGACGCTGAGATATGGAGAATGCTGGGAGAGGAGAGACTTTTTTCTTGATGCAA
AGTGAAAGAATGAAGATATTTTAAATGATACAAGAAATACGACGACGAACGAGCACGAGCTGCTTATGAG
AGATGGCTGTGATGGTGGGGACGGCGACTGATCAGAGAAGAGATGGGAGAAAGTGATGAGATTGAGAAAG
GAAGAAAGGGAAGAAATGTTGAGAGAAAAATGCTTTCGTGCGTGGGGTTTGGTAGACAAGAAAGGAGAAA
AGAGTAAAGTAAGAGAGAGAAAAATTCAACTTTTTACAAAATTAAAAAAATAAAAAATAAAATAAAAAAG
ACCTTTAATGTCGGTTCTAAAAAACATGATGTTTAATGTCGGTTTTAAACCGACATTAAAGATAGAGCTT
TAATGTCGGTTTAAAACTGACATTAAACATCCGCCTTTTGAAAACCGACATTAAAGCTTATTTTTCATTT
TTTCCACCCGACATTAAAGACCATATTTCTTCTAATGTTTATAGGTATGTGGAAGTTTAAATGTCTAAGG
GAGTTTATATGTTTATGTGTGTGTGGTGGAGAGTTGTTTTTTCTAAGTTCATATGTTTGTGTTTAGGGTG
AGTTGTTCATAAGTTAAAGTTTCGATAAATAATGTGCTTTTTTTATATCAGAATTGTCAAAAATAACAAA
TCTGACAAAATATTTACAATATATAGCAAAATTTAGGATTCAATCATAATAAACATCGAAAGACACTAAC
ATGTATCAGCGGCATTGATAGACAGTGATAGAAGTCTATCAATTTCTATCATCGATAAAATCCAAAAATT
TTCTATAGATCGTAAATACTTTAATTTATTTTACTATTTTTAAAAATACTCCTTTTTTATAACTATTTTA
ATTTTCAAATTTCTTTATTCCAAATTCTATCCTTTTTTTTCTTTTGAATAAAACATGAATTGCATTTTTT
TTATTTAGTTTTTTTTCTTTCTTTTCGATAATGAACAACCATTAACCCCAAATATTTTCGGCACTAACAT
AAATAAATAAAATGTATAAAACAAAGGGATCTTTTCAAAAATATAAAAAAGCGGCAAAATATTTACAATT
TATAGAACAATTTTGAAAATGGAAAAAGCTCAGAGGCTCACCGTGTAAAATACCAAAAATGGCCCCGTCA
ACCACGTCGTTAACAACGCACGCCGTCAACAACGCGTGTGTAATATATTTGCGATCGTTTAGATTTAGTA
TTGTTGTCAAGCGATCATTTAGATATGGCTACAATTTATCTTTTTAATTTCATCGTTTAATTTTGTTACA
TGGTTGTTTAATTTGGTTACATGATCGTTTAGATTTGGGGACCCAAATCTAAATGATTTTTTTTCAAAAT
TTGGTACACGATTTTTTAAATTCTTTTGGTACAAACTAAACGATGATTTATTTTTTTTACACAATTGTTT
ACTTTTTTTACACGATCGCTTACATTTAGCTACTCCAATCCAAATAATTTTTTTTCAATATTCTTTATAC
ACGATTTTTTTGTTCGTTTAATTTGGTTATGTTGGTTACACGATCGTTTAGATTTGGGGATCTAAATCTA

AATGATTTTTTTTCAAAATTTGGTACAAGATTTTTTTAATTCTTTTTTTTTAACAAGAAACGATGGGTTA
GTCGGTGCACCCGGGCATCTCCACTAGGTGGACACCCCCTTAGCACCATCATCATTCCCGCTTCATTAAT
AATATATCAAGAAAAGTATAGGGAAGCAGGAAAACCCAAAAGCGAAAAACAAAGGGCTTGAGAACAGCCC
CTAAGAAAACAAAGATAATAACTGAAAGCTAGGTAAATGCATGAAGATTTAGAACTAAAGATGTAGCTTG
ATAATTGGAGAATAAAATAGATTTGCTTGTCCAAAGTCCTGTTAGAGCTTTTTTGTCTTCCCATAAGTCC
GCAACTATTTTTTCTTTGTCATTGAAAATACGGGCATTCCTCTCCAACCATATATTCCAGAGGGCAGAGG
CATAGGTGTTGAAGAGTATGATGTTTTTTTTGCTCTTCTGTTTCCAGCTGCACATAGTAATACAGAGATC
CTTGGGGCTGAGGCAAGTGACATTGCTGTTTAGGAGGGAAGATATTAAGCTCCAGATGGAATATGCTATG
GGACACAAACTAAAGAGATGGATCCTGTCTTCTTCATTTCTTTTACACATCGCACACCAGTTTGGTTTAG
AACAAAGATTTGGAATTATCTTAGTAAGTTGGTCAGCTGTATTAACACTGTCATAGAGCAGGGACCAGAT
GAAGAATTTGCATTTTTTTGGAATGGTGGACTTCCAAAGATTTGTGAAGGTGTTATGATTTGGGATAGCT
AAAACACCTTGGTCAGATTGGTGTATAGCTTTCTTTACCGAAGCGACATAGTAAAAACCATCAGAGCTGA
GATTCCACGTAGGAGTGTCTCTGCCATTTTCACAAAAACTGGCATTTAGAGAGTTTTTTAGCTCGGCCCA
CAGAGGATATTCCCAATCCCTCAACTGCCTTCTAGGTTTTAGATCCCAATCCATCAGAGTTGTGTTCCAC
ATGTCATTAATGGAGCTGTCCTGAATTGTAGAGAGAGCATATAATCTAAGGTAATGTAAAGATAGAGGAC
TATTTTGATGCCATTGGCTGTGCCAAAAAGAGAAGTTTCTTCCATTTTTAATATTCCAGGAAACCTGCCG
TTGAAACCAAGTCAAGCCTTTGCATATAGATCGCCATGGGGAACGGCTGCTACTGTGGTTGCTATCACAA
GGAATATCTCCTTGAGTTTTGCTGCTGTATTTTGCATTGATGATTTTCTTCCACAGAGGGAAGTCCTCAT
GAATGTATCTCCATAACCATTTTATTAGAAGAGCAAAATTTGTATCTTTTATACGGCTAATGCCCAGCCC
CCCTTTCTCTTTTAGCGAGGTAATCTTTGCCCATCTAACCAAGTGTAGTTTGTGGGAATCAGTGGGGCTA
TTCCAAAAGAAATTTCTCCAAGATTTTTCAATGCTTTTGTATATTGATGCAGGGGCTTTGAAAACAGATA
ATTGATATGTAGGAAGGCTGGCCAAAGTAGATTTAATCAAAGTAATTTTCTCACCTTTGGAGAGCATAGA
GTATTTCCAGCTGGCAAGTTTTTTGTTGATTTTCTCATCAATGTTATTCCAGAAAACCTTGGTTATTGGT
TTTCCTCCAAGAGGCACTCCAAGATAATTGATTGGGAGAAAATGTGTAGTGATTCCCCATTGAGAAGCTA
TCTGGTCAGTTCTTGCAGCATCAACATTTATAGGGGATATGGTGGATTTGTTCAGATTGATATTCAATCC
TGAAGCTAGCTGGAAAAGACTGATGATTTTTTTTAAGTTTAGGAGGGAGGTCTCATCATCTTCAACAAAG
AGCAGTATATCATCAGCAAAAAGCAAGTGAGTGAGGTTTAGGTTGTCTTTCATTCTCACCCCTTTTATGT
TTTCACCCACTGAATCCAACAACCTGCTTATGTAGTCCATTACAAGGACAAAAATAAAAGGGGAGATAGG
GTCTCCTTGACGAATACCACGGGATGGTTGTATTTTTCCTCTTGGTCTGCCATTGATGATAATGGAATAC
TGAACACTACTGATGCAAGCTTTTATCCATCTCCTCCATTTTATGGGATAGCCCTTCTTCATAAGCATAA
AGTCTATGAATTTCCAATTAAGCTTATCAAAGGCCTTTTCAATATCCAGCTTGATCACAAAGCCCTTAGT
TTTCTTGACTCTCCAATAATCAATAGCTTCATTTGCAATTAATATGGCATCAATAATTTGTCTACCTTTA
ACAAAGGCCATTTGATTCTCTGCCACCGTAAAAGGAAGTGTTTCTTTTAGTCTTTCAGCAATGACTTTGG
CAATGATCTTATAGATGGAAGTCGTTAGGCTAATAGGTCTGTAGTCCGCTGGCACCGCACATTTATCTTT
TTTGGCAATCAGGACAATATTTATTATATTCACTGCTTTATTGATGATACAGTTGTTGTGGAAATCCTTG
AAAATTTTACAAATATCATCCTTGAGGTCATACCAAGTAGCTTTGAAGAATTCTATTGTAAAGTCGTCTG
GACCCGGGCTTTTATTGTTTGAAAAAGCAGCAAGGGCTGAATGAATTTCCTTTTCTGTGAAAATCGAGCA
TAAGTTTTGAGCTTGGGAGGATGGTATAGGGGACCAATTGAGATTTTCGATAAGCCATTGGCTGTCCTCA
CCATTTCCTTTGTAAATGTCTTCAAAATGGTCTAGGAAGGCTTTGACAATGTTATCATTTGTAGTACAAG
GAATCCCATCAACTGAGTTAATATTTGAAATGATACTTCTTCGTTGTCTAGCATAGCAAATTCTGTGAAA
GAAGGAAGTGTTTTAATCACCATCAGTGTTCCAAAGCCTCTTGCTTTTTTGGTGCCATATTTGAGCTTCT
TTGAAGTCAAATGAGAGGATCTCAGCTTTGATTTTTGTTCTGTGGAGGCTAAGCTCTTCAGAAAAAATTTC
CTGCAGCTTCTATTCTGTCAATGTCGTCGACTTCTTTGACCCAAGCTCTCTTTTCCTCTTCATTATATCG
ATTATTTTTTTTTTTTTGTTCAACCTTTATAATGAAAGATAATTTTTTGAGCTTCTGCATGATAGTAAAGC
CTGGATGACCATCATGTCTCAAATTATACCACCATCTTCTAAAGTTGTTTTTAAACCACGGCTCTTTTAA
GTGCACATTTATAAATCTGAAGAGCATAGGACCCCAACTAATCGTGGAGGTTTCAACAATGATCGGGAAA
TGATCTGATGTAACTCTGGAAAGGGTTTTAGAATAGTGATCAGAGAATAAATTTTCCCATCCCATTGTAT
ATAGAAATCTGTCAATTCTGGAGAGAATCGACTAGGTTCTGAGGTTAGACCATCACTACAAGAAATCTGG
CCTTTAATGTCGGGTGGAAAAAATGAAAAATGGGCTTTAATGTCGGTTTTCAAAATGCGGACGTTTAATG
CCGGTTTTAAACTGACATTAAAGATCAACCGACATTAAAGAAGGATCTTTAATGTCGGTTTAAAACGACA
TTAAACGTCCGCATTTTGAAAACCGACATTAAAGCCCCTTTGTAATTTTTTTTATTTTTTTAATTTGGTA
AAAAATCAAGTTTTCTCTCTCTTACTTTACCTTTTCTTTTTCCTTAAAAGTTTATATATAAATTCTCCTC
CTCTCTTCTTCCTCCTTATAACTTTGAAATCTTCTAACCCTAAATTATGCAGCCACCAATAAAATTTTCT
TGCAGATTCTTTTTTCCATCGACGATTTGAAGATTTACTATGGCCAGTAAAAGAGAAGGTCCGGCGATCG
GAATCGATTTGGAACCACGTACTCTTGCGTCGGTGTGTGGCAGCAGGATCGAGTTGAGATCATTGCCAAC
AATTAGGGCAACAAGATGACGCCGTCGTATGTTGCTTTCATCGACTCTGAGCGATTAATCGGTGATGCAG
CTAAGAATCAGGTCCCCATGAACCCGATTAACATCGTTTTTGGTAAGTTTTCCTTTTCTTTTCTTTTTTT
TTTTATTTTTTTATTTTTTTATTTTTTTTTTTTTTTTTTTTTTTTTTTTTTTTTGCGCTTTGAGAAGTCCTTC
CCAGTTGTTGTATTTCAAGCTCATTCTTTCCTATTTGTTTCTTAGGTTGGCTCTCAGTGCACCATTCTTA
CAGATAACTATTGTGAAGATGCCTATCATTTACTCCAAACCCCATTGTTGAAGAATCTTTTGGTAACTTC

TTAGAAAATTTGTTCTTATTTGCTCCAGTTTAAATTCTACGTTCTCTTGAAAAATATTCTGATAGAAGAT
TCTTTGAGTTTTCACTTCTGACATGTGTCTGTTGTCTTCCAGCTTGCAGGAATCCTCTTGGACACGAAAA
ATCTCGATGCATCGTCTCAATCATCCATGACAAGAGATGCAGAGGCAGTTCAGTTGCTATCTGTTGGCTC
AGCCCTAATTTCTAAGAATGGACTTTATGATCAATGTATGCCTCAACAATTTTGTTTTACCCTCTATTTG
TCTATTAGAAAATATTGACGTTGCAACTATTCCTGGTTTTGTTTTTTATTAGTGATGTGCGTTCAAAAGG
AAAGTTCATTTTTGGATGCGTTAATACAAAATTATGGAAAGCCTCCCAGTGATGGTAAGCTTTCATATTC
TTTCATCTTGAAAGTTTTTTGCATATTAACTTTTTCTTGATCATGTTCTCTCTGTTGCGGCAATCCAAGT
ATTCTGATCATAGTTCTGATCTCTTATATCAAGTTTCTTGTATCAACTTTGTGTTTACATATGATTTTGT
AACCTTTCTATAGGTAGCAACGACGGCGAGGGGCGTTCAGAGCATATCAAGGAGAGGAATCAACCATCCA
GCCCACCGCATGGCGAAGGAATTAATCAACAAAAGAAATCTAGTGATATTGGAACTGCCAAAACTTCCAA
GGTTTCGCCAAAATCCGGTACAAGTGTAAAAAGCATACTATTTCACTATTTCACTATATCCCTTCGAATG
TTAATGCTCATTTTGCATTCTACGAAGATAAGAGATTTTATTAACAAGGAACCTGTCTCTAATTTTTCTG
TTACAGCTAAACGGAAGATAAGAGATTCTGTTTTTGTTTTCAAAACGGCATTTAATATCACGATTCATGA
GTAAGATTAATACAACAGAGATCTCAGGTAAAATGCCAACCTTTAGGTATAACCTAGAGCAGACAATGAG
CCGTATACAATTCATAGTGTTCTTGTATATATATTTAGGTAAAATGCCAACCTTTAGGTATAACCATGTA
TACATTATTCATTTGCTAGTGGAATTTAGATTTCACCTTGTGCTATGTGAAAAGATGAGGAAGATGCATT
CTTGAGTTATTAGTTTATGCTCATAAGATGTTTGATGAAATGTCTCGTAAAAGTCTCTTTTTCTTATACA
TTGAGTGAGCGTTTTGTTTGGTTTTTTAGTATTATTTTTGTGAATATAGGTTGTTCGAAAGATGCAAACT
CCTAAGGAATGTTGGTTTTAACATAGGAAACATATTGTATTATTTAGGAAAAGGCATCGCAAGAGGAGAA
CAGGCAGCTAGCAAATAAGGTATAAAATTAATTATGTCCTATATATTTTCAGTGTTGTATATAAATTAAT
TAAAGTAATTTTATATTGTTGACTAGCCTCTAAGAATGTAATATGTTCATATACTTTGAAAATTTTAGGT
AAAGGAAAATGAGAAGGCTTTGGTTGAACGTGGACAGTGCGATGTTCCAAACTTGGTTCATAATAAACAG
CCCATTTTTGGAATGACACCAAAAATTCCTTCTTTATCTTTCGGGTATATTCCTCTTTCTCTCTTTCAAC
TGAGAAATCTTTTCTCCTCGCGTTCATAGATACGTACTATAGCTTGTATTGATTCAGCTATATCATAATT
TCATCGTTAGAAAAAAGAAGCTGTCATTAGTTATCTTGAAGGATAATTGAGATACTAAGGAGTACTAAGT
TGGTATGATGAATTGTTTCTTTTTTTATATACTGATTTACTTTTATTTCTGTATTTTTTTTCCTTTTTGA
AAAATTGAAAATATTAGAAAAGAAGATGAATATTTTGTGGAGGAAGATGGGTGGACTCAGATTTTGTCCC
AACTTCAAATTAGTTCAAAGGAACAACCCTGCTCTTCTCTTTTCTTCATTCTCTATCACTCGGGCTTCAT
TCTCTGAGGTACCATTTTTCTTTCACTTTCCTCCTTTTCATTTGTTTCTTTTTTACGTGGGCTATGACAT
CACTGCAAGTTTGGTAGGTTGAAGTTGCGAGTTTTGGACTTTTGGAGTTACATATATAGGATCACGTTAT
ATTTCGAGTTCTTTTTTCTACCTAATCTCCTTATTTTGATGTGATTAATAAGTGAATTTAACTACTTATT
AGATGCATATATTCTACCTCAATGTTTAAATACCTCACGTTTAGCTTTGTTTTGGTATTGTTTGTAGAAT
GATTGAAGTTATATCGGGTTGAAAGCTGAGCAATTGTGTAGATAGCCAGGCGATCATTGAAGGTTGAAGA
TTGAAGATTGAGAAGACGTTTAGAATTTTTCTTATTTACATCTTGTATTAGGATCTTTTTTCATATACTG
TTGTAGAATGTATATATAAACACAATATTAAGTTTTCATTTTGTGTATACAAATGTAAAAGATAAATATT
ATAGTTTCTAAAATGATATTAATTTTATTAATTCGTTGGAGCGAGAAAAAATATTTATCTATATGGATTC
AATGTTGGTTTATAAAAAATGGACAATAATACAAGAATTAAATAAATATAAATTTCTAAAAATGGACAAC
AAGTCTTTAATGTCGGTTTTAAACTGACATTATTGGCCTCTTTAATGTCGGTTTTAAAACGACATCATAG
CCCAATCGACATTAAAGGGCTTCAATAACACTATCAAAGATGTCGGTTTTAAAACCGACATTAAAGGGCT
TCAATAACACTATCAAAGATGTCGGTTGAAAACTGACATTAAAGGCCTTTAATGTCGTTTTTAAACTGAT
ATTAAAGCCCAACCGACATTAAAGGCCTTTAATAACGCTCGCAAAGATGTCGGTTGCCAAGTGACATTAA
AGCCCTTTAATGTCGGTTTTAAACCGACATTAAAGACCAAATTTCTTCTAGTGCATGTATATTTTGCATT
TGAGAGAGGCGGGTCAATGAGATTGCTTTCGGAGATGAAGTTGTTAAATTTCCTCATACTGTAATGTCTA
GGATTTTGGGCAGACGTCTCAGAGGAGAATCTCACAACGTTAAAGTCTCCCGCTAAAAGCCAATTTGGAG
AGCAAATATTTTTTAGGTCCAGAAGTTCAGCCCAAAAGGAATTTCTGTTTCTACCACCAGCAGGCCCATA
AATGGCCGACAGCCACCAGGCCGAACAAGGAGGCCCATCAGGAATGTTGATTTTTATGGATAATGAGAAA
ACGCCTTCAATGAAGTCAGACACATTGAATTTGAGATCATCCCACATGATAATGATTCCCCCTGAATTGC
CATTAGCACTTAAAAATCGCCAATTTACACTAATAGAGATCCACAGAGATTTAACAATTTTTTTATAACA
ACAGTCAGTTTAGTCTCAACTAAAATCACAAAGTCAGGAAGATAACGGGAAATAAGATTTTTTATTTGAA
CTCTTTTGGAGGGAGAGTTTAACCCTCTAGTATTCCAGCTTACAATTTTCATTTAAACATAGTATTCCCC
TTTTCAAAATCACCTGCTGGAGAGCTTCCCTTAAGAACATTTGAGGCAGTATTAATGTTGTGATCCTAGT
CCACCGGAGCTAGTTTGAGGTTATTGGTCTTTAACCAATCAATCAATTTTTCCTTGAAATTTGTTTCATC
GTTTTTTTCTTGCCCCTCTTCTTCTTCTGATTTTTCTTCATTCTCAGAAATTAGCAGCTGCTCGATACTG
TCTTTTATGAGGTCAGAATGTGTTGGGGATGTGGGCGATTGTGAGATGGATAATTGATCTGGGCTGGATA
GAAACTCATCAGAGAAAGGAGATAGAGGCCCTAGGTCCACTGTGAGGCTGATGGATTTGGGCCTATTTTC
GTCATCATTTGGCTGAATTTTTAGCCCACTTCCTTTTGTCTTTTCTCCTTTTGTGTAACGGGCTTTGAGT
TTGGGCTCTTTATTTTATAAATCTTTTTGCCTTTTCCTTTGCTCTCTTTTTCAAACTTCTTCAATAATTT
TTTCTTTGGGCTTTCAAGTGGGCTGTTTATCTTATAATGGGTCTGTGGGGCACTCTTTCGGTCATAGAGA
AAAGTCTTGGACTTGGGTGTGTCGAACGACACTTTTCTTTTGCCTCTCTTTTGTCCAGCTGACTTTGAAT
TTTCCCCCTTATAAAAAAGTTTCCCTCCTTTTTGGCTAATCACTGACAGTTCTTCATCTACTATCTCCAT

CTTTCTTTTCTCACTTGAATCACTGTCACCGTCATAATTCAAAAAATCCATCATTGCGCCCAATTTTTTA
TTATTTTCAAGCAATGGACCGTTTCCCTTCTTTTTCCAATCTTCCTTTCCGATCACCATCGACACTGCTT
TTTCCGGCGAGATAGCTAGATTACCTTCGAAGAAGTATTGTTCACTATTCAAGTTAAATTCGTCAAATCT
TCTAGCTGCTTCTCTAGTAAATGTACCATGGATACTAGGATTTCTTCCCAGATGCCATTTCCCTTCTGTT
TTGACTATTACTTGAATAATATAACTGCGTTCTTCTTTGTCAATCAATTTGATGAACGCCGGAATGAACC
CCGAATAGTTATCTTTAACTCTAATAGAAGCTTCAATGAGATCAGTGAGCTCCCTAGTTTCTTTTGCCAC
TTCCATGAAACCACCACAGGCGTCTCCAATTTGAACAAAACTTTCTAAATTCCAAGCATGCAGGGGCACT
GCTCGCACCTTAATCCATCCACCATAACTTGGAACAAATTTCGGTGATGCGTGATCTTTCTGGTTCCATT
CCTCGAACTTAACATAAAAGCGTCCCACTGTAGTCCAACCCTTATTTTTACATATCAAGTTTGCTTGCTC
AAAGTCTTTGAAACAAATCAACGCTTTATTAGCATGAAAAGGCTTGTAACTCACAGTGGTGTCAAGCTGT
TCATTTAACTTTTCCACTATTTTCTCCCAATCGTCATGGAAATATCTTCTAGTCAGGACAACTGTTCTTC
CTCAATCAAAAGATAAGTTTGTGAGGTCTTTACCTGTTTTCTTTGCTTTTATGGACCAGAAATTTGTTTC
CTCGTCAGAAGACCTTCCTTTGATAACAGCTTCAACATAGGATCTCTTTTTTTTTATCGTCCCCTGACTC
AGAGCTGGAGAGTCTGTCTTTCATTTTGGTTCCCAGAAGAGTTTTGTAATTTGCTTTTGTAGAGGCATCC
TTCTTGCCATGTAAAAGACTGACAAATTTTGCCCATCCTGATTTTTTTGACCCTTCAGAGACCAGAATGC
AGCATTTCCTTCCTCTGTCGTCCACTCGATAAATCTCAGCAATATAACCTTTTCTGTTGTATGTTTTTTG
GACCCACAGGCAATATTCCTCAAATCTCTTTTTCAAGAAGAATCTTGTCGTTAGAGGAGTATCCAACAGG
GTTTTGAAAGTGGACTTGAGCCATTCCAAAGATTCCAAAGTGACAGCAATAGAGAAGGATTTGTATGGCC
CCACCTCCGTAATTAGCATCTTTAAGTCTCTTGATCGGTTGTCCAAAGATAGGACAAAATCTTTTTTTTC
AATAGTGCAAGAACGAGGGAGCTGTCGCAGTGAAGACATACTGCAAATGGAAGCAAGCAAAGGAAGGAAG
CAGAAAGTGAGTGTGAAGTCTACTGAAAATGTACCTTAATAAACTCAGATAATCGAGATCTTCCTTGGTT
GGCGGGAGTATCAGCTGGAATAAATATGGTTGTTGAGCAGAGTAAATTTCTTCAAGGGAATCGCAAAAGT
CTTCCATGGCACTTGTTTGAGTTTCTCTGTAGTTTGGGAATCGCAAACGCCGGCGGTTTGAGGAGAAGAG
AGAGAAGCTCTGCCATCTTTCTAAGCTCCATATTGAAAATCTCTCTAAGCTCTGCCATCTCCGATTTTTT
TAATTCTTTTGGTACACAATCGTTTAGATTTCTTTAAACGATGATTTATTTTTTTTACACGATCATTTAT
ATTTGGCTACTTCAATCCAAATGATTTCATTTTTTTAAGATTCTTTATACACGATATTTTATTTTTTTTA
TACGATCATTTACTTTTCTTAAACGATCGTTTACATTTGGTACTCCAATCTAAATGATTTTTTTTCAAGA
TTCTTTATACACAAACTTTTATTTTTTTACACGATCATTTACATTTGGCGACACAAATCTAAATGATTTT
TTTTCAAGATTACAAGATCTTTTATTTTTTTTACAAGATTGTTTACTCTTTTACACGATAGTTTACATTT
GGCTACTCCAATTTAAATAATTTTTTTTTCAAGATTCTTTATACACGATCTTTTAGATTTTGTTATTTTT
TTGTACACAGTCATTTAGATTTGGTTAACCAAATATAAATGACACAAAAAAAAGAGGAAAAGAAGAAAGA
CGATGGAAAGAAATCGCAGCAAAAAAAAAAACAAGAAAAGTAGAAAGATGATGGAAAGAAATCAAAAAAA
GAGGAGAAAAATAAGAAAGACAGATTAAATGACATAAATAAAGAATTGAAAAATAAGAAAGATGATGGAA
AGAAATCGGAGAAAAAAGAAAGAAGAGGAAAGAAGAAAGACGATGAAAGAAATCGCAGTAGGAAGATGAA
AAAAATTGCAGGAAGAAGACGATTTCATCGCGAGGAAGAGAAGAAAATGAAAGGGCACCAATTTAATTCT
TTTGGTACACAATCGCAGTAGGAAGATGATGAAATTTTCTCGTTGAATTTCATCATTTTTTTTTCTTTTT
CAAATTGGTGCCTATTTTTCCTGTATCGTGATTTTTTTTAATTGAATTTTCCTCATCATCTTAGCAACCA
ATGAAATATCACCACATTTCTTCAACAAGCTTGCAGTCATTTCCTCTAACTTTGGAGGATCATAAAAAAA
AACATATATCTCCATTTTATTTAACTCTTGCTAGAAAATGTTTTAAAAAATAATTCTTTATTTTATGAGT
TTTTTTTATTTGTTAATTTTTGAGTAATTTTCATAACTGTAACAAAAACCCAAAGTATTTACGGCTTGTA
TAACAAAATAATAAAAGGTCATGACATATGATCAGTTTTTCATAAATTCCATATATGCCCTTGCAATTTA
TATCTTCTTTGCGATTTATTAATCTCGAGCCATGTTGGTGCAATTAAGGCCCGAGATCGAATAACTAAAG
ACATATCAAGTCTAATTTCAACCTTGGGCAATGTATGCCTGAGATCGAATAAAATAAAGGCAGAATTATA
TTCTTTCATCCCAAGCCATATCGTTGCCACTAAGGCCCGAGACGATACAATAACTAAGGGAAGAATTCTT
TCATCCCAGGGCATGGGAATGCTTAAGATCGTGTATCAAAATCTACACGATCGTGTATGGCACGCATGTG
GCCGGTTTATTGCGTGTTCACATACTAAGGTAATTTTGGTATGTTACATTGTGAGTCTATCAGCTTTTTC
CATTTTCAAAATTGTTTTTTTTTTTCCGGTTTGTTATATTTTTTAAGAAACCCCTATTTTTGTTTATTTT
TGCTTTTCTTGTTTGTTCGACCATTCAACTACATGCATATTTTTAAAATTCGTAACACTGATTTGATATG
TGAATTCATTTAAATAAACAAGTAATAGGATATGCTTGTATATAATCATGGTCAACAAGAATCCAATTTC
ATGCATACAAAGTTGTGCCTTTGGATTTGAATTCAATTAAATAAAAAAAAAGAGAAAAAAAGGAAGAAGT
GAGAAGCCACAGGTAGGAGGGAGAAGTGAGAGAAAGAAGTTAAAAGGAAAAACTCGTAGTAAAAAATTGT
AGTTATGCCTAATGAAGAACAAGAGAAAGTAGGAAGAAAGTGAGAGGAAGAAAAGGAAGAGATCAGAAGT
GGGAGGAAGGAACGAGACTTCAAGCTCTCTTGCATATTTTCACTCGCAGCTGAAGAATGTATAAGACAAT
TTTTTAACGTGTTTTATTTAACCTTGACACAATATTTGTTGGATTCCCTTTTATAGGGAAATTGTCAAAA
TTAAATAGAACCTTTGACCAAATATTGAAAAAATTCCTTCCTTTTATGTCACGGCGTAATTACTTAAAGA
TCTCTTAATTTCTTGGTGTTGTCCAAAACTATGTTCTTGAGTTTCAAGGATTCATCTTCAAGTTAAGAAA
TTTGATTTCGTGTTGATTTTTTTCAACTTATATCAAATCAAATTAAATTTTGAAATTAAATTTCCTCTAT
AAATGGAGGAAATTACACCCTAATTTCTTTAAGGAATTTCTTCAATTCAAAATAATATAGTTAGGTATTA
TTGGTTATTTTACAATGATAAATTAATTAATTAAATACTTATATAGAAACTAAAATGTAATTTATAATTA
TTCTCTTTAATCAGTTTAATTTAACCTCTTATACATAAATTATATTTTTAAACTTTTGTAATTAAGTTAT

ATTTTAAACATTTTAAAAAACGTTGAATGCACATAGTATCAAATTAAACTAAGAAGTGTAGTTTTTTTAA
AATTTATATAGAAATAACTACTTTGATCAAGACAAAATAAAAGAATACAAAGCATCCAAAAAAATCAAGC
CACCAGAACACACCAACTAAAAAAGAAGGGTCCAACTAACTAAAATGTTATCAAAGGAATAATAAAAAAG
GATGCAGAATTATCGGACATAACCAAAAGATCTAATACCATATAATTTTCAGAAGAGGATAAACGATAAA
GATGAGGAAAAGAACAAAGGACCCCCTATCCATTCATCCTCTCGAAAAAACACGATTCCTTACCATCCCC
ACAAAGCAACGAGTAAACCGGGAAAACGTCGAATACTTGAAAAAAATACCTTTCCAAGGATTACAGTAAA
GAGTGTTGTTATTTTTGCAATACAATAGTTGTGGAATTTTTTATTCTTTTTGAAAAAGGATACGTTAGGT
TAGTAAAGAAAGATGAGGGTGTTAAGCGAGTGTCCGCCTTGTGGAGATGTCCAAGAAGACTATATGGAGT
TTTATTAGTTTTTCAAAATATTGATTAATGGTTATTATAGATTTCTTTTACCTATATTCTGTATCATTGT
ATATTTACAATTATTCTTTTAGCCACTATTTTATCTACATTTTGCAATTTGGTATGCCAATTACATACAC
GTTTTCCATTTGGTTTCTTTTCCAATGTATTTTAGGATTGTGAAAGATTAAAGCATGTTGATCTTAGCTA
CTCTACTTTATTAGAGAATATTCCTGATTTCTCTATAGCATCAAACCTTAAAGAATTGAATCTCATCAAT
TGCACAAATTTAAGAATAATAGATAAGTCTGTTTTTTCTCTCAATAAGCTTAATGTCCAAAACCTTTATG
GTTGCTTTAACCTTAAAAAGCTTTCAAAAGGCAATTTCATGTTATGTTCTTTTAAAGAATTGAATCTCTC
TTACTGCAAAAATCTTAAGAAAATTCCAGACATCTCTCAAACATTAAACCTTAAGAGCCTATATCTCCAA
AAATGCACAAATTTAAGAATGATTCATGAATCTGTTGGATCTTTGAACAAGCTTGAACAGTTAAACCTTA
GACAATGCACTAACCTGGTAAAGCTTCCAAGCTATCTCAGGTTAAAGTCTCTTGGCTATTTATCACTTTC
TGGGTGTTGTAAGCTTGAAAGCTTCCCAACAATTGCTGAAAACATGAAATCTTTATACGAATTGGATTTG
GATTTTACTGCCATAAAGGAGTTACCTTCATCAATTGAATATCTTACTAAGCTTTCTATATTAAAACTTA
ACGGTTGCACAAACCTCATCTCCCTTCCCAATACAATTTATTTATTAAGGAATCTTGAGAATCTTCTTCT
TAGTGGCTGTTCTATATTTGGAATGTTTCCCCATAAATGGGACCCAACTATCCAACCAGTATGCTCTCCT
TCAAAAATGATGGAAACAGCTTCGTGGAGCTTAGAATTTCCCCATTTACTAGTTCCAAATGAAAGTTTAT
GTTCCCATCTCACTTTGTTGGATCTTGAATCTTGCAACATATCAAATGCAAAAATTTTAGAAATTTTATG
TGATGTTGCCCCTTTCTTATCTGATCTACACTTGTCCGAAAATAAATTATCTAATTGACCCTCATGTCTT
CACAAGTTCATGTCTTTATGGAATCTTGAATTAAGGAATTATAAGTTTCTTCAAAAAATTCCAAACCTTC
CCCAAAACATACAAAATTTGGACGCCAGGGGTTGCAAATCGTTGGCTCGAAGTCCAGATAACATTGTGGA
TATAATATCAATAAAACAGGTTAGATTCTTTCCATTCATTTTGTTCCTATCTTACACGTAGACAATTTAA
TCATATGGATTCTTATTCTCTATAGGACCTCGCGTTGGGTGAGATTTCAAGAGATTCTTATTAATGGGCG
TTGAGATTCCAGAATGGTTTAGCTATAAGACTGCCTCCAATTTGGTGAGTGCTACCTTTCGTCACTATCC
AGAAATGGAACGAACTTTGGCTGCCGGCGTTAGTTTCAAAGTGAATGGAGATTCATCTACAAGAAAGTCC
GAATTTCATGCAGTATATTCATCTGCAATAAACTCCATTTTTCTTTTTCAAGACCATTTCTTCCATCAAA
ATTAGAATATATGTGGTTAGTAACAACTTCTCTAGCGTGGGGTTCCATGGAGGTGAATGATTGGAATAAA
GTTTTGGTCTGGTTTGAGGTTCATGAAGCACATAGTGAGGTTAATGCAACTATAACAAGGTGTGGTGCCC
ATGTCACTGAAGAGCTCCATGTGATACAAAATGGATGTGAAGTGGCCGATGGTAAATTATGCCGATTTTT
ATCAACTAGAGAAATTGCAAAGTCTGTAAGTTGATTGTTTAATTACTTGTTACTTATTTACTTTCTTCCT
GTTTGTTTTTTTTTTTTTTTTTTTTTTGGAGTGAATGTATGATAGATCTCAAAGGGGGACATGTGTATGTT
ATGATTAAGTTTGTTTAATGGCATAGGGATATTGAGGATCTTCTTCTCAAAAGCATTTTGAAACAATCTC
CTGCTGGTCAAATTCCAAAGCAATGTTGCATGCAGGAAATTATGATCCAGAAGCAATAATTGATTCCAAC
ATACAACCTATGATTTTCCCATTACATGTAACATATAATGGTGAAGCAGTGATATATGGACTGGAAGGCA
TGGGAGATACTACACTCGCCAACTCTTTATGCAATAAATTTAAATGGATGAAGGATAACCTTTTCGGGAT
AAAGGAGCATCATCACAGCGAAGCTTTAGATAATTCTACAGGCTTTTTCCATATCAGAGGAAGAGAGCTC
CAGTTATTTTCTTGGTCGTGGGCCCACCACCGTAAGCGTGGAGATGGTAAAAGAGGAACCAATATCACAA
CCCATACAATATCCTCCAAATGCTATTTGATGCTCTTTCATGAAGCCGAGAACTGTAATGATATATTTGA
CTGGGTTGGTACACAGCGTTGGATTAAAACTTCTGGCAGTTCTAACGGAAGAGGTGATGTTCAGTTTCTG
GTTAAAAGGGTTGATATATCATTCCTCCGAAGCTCTCAATTTTTAGTAAAAATTTATATTTAAACAACCT
TTTACTCATTTTGGGACTTGTATTATATTAATTTAAACACTATGATAATTAATATCAATTTAAACGCTCA
ACAACTTGTGTAAGTTTACATATTAATTGAACACCCTTCTTATCTAATTTAGATTTTGTTCAAAAATATC
ATGTGAAACATATTACTAAAATTTTAAATTGTTATATTAAAGTGAATCGATTTAAACTCTTTATTAGGAT
TACATTTGATCAGTATTCAATATCTTCACTTTCTCCCTTAGCTAGCTAGTGATAGAGATACCAATAACAA
TGTTTTTTTTTTTAAGATGGAAACCGATGTGTTATATACTGCTAGGCACACCAAAAACATCAAACTTCAT
TCGATTAAAATAAATATTGACATAATTAGAGTATTTTTAGGAAATATTATTCTTAGTAAATATTATATTA
TTCTTGTATATTCTTTCCTTTAGTGTGTTTTAATATGGTTATATATAGGGCTGTAATCTGTCATTATTAT
CAATGAATCATCAATGAAACACCGATATTCCTCTAACATGGTATCAGAGCTTTAGGTTTTAGTCGATCTC
TATTTTCGTGTTTGATCTCTACCTCCGTCTATCGTCAATGACTAGCCATAGACGGAGGTAGACCCAGCAG
ACCCAAATGTAAATCTCCCACTTTCACTCTTTGTTTTTACTTTAAAAAAAAAAAAAAAAAAACCATCCGC
CCCCCCAATTCCTTAAAAATCCATCCCCCGCCGGTCGTTTGCAACCCACGTCCGTCCCCCCCCCCAATTT
CAGCCGCCGTTGCCGTTCCCTTGGGATCTGCCTTTCCTCGCCCGTTCCTTTGCAACCCACGTCAGTTGCA
CAAAGTTTTTCGAATCAGCCGCCGTCCCCGCCCCTCCAATTTTAAATCCGTCGCCGCCTTTAAAATCCGT
TCCGCCCCCCCTCCGTTCAAATTTCCGATCCAAGCCGTCAACTCTAAGTGGTCGGGGAACAGCCGGAATA
TCCTCACCCCCCTTTTTCCTTTTCGGGATAAAATCCCATTACTTGTTTATGCTTCATGTTTGCATTTAGG

GTTTGGCTACTCTCCCCTGACGTTCCCTCCTAAAAATAATACCCCTCCGTCTCTATCTCGATCTTTTTCT
TCGTTATCTCGATCTTTGNNNNNNNNNNNNNNNNNNNNNNNNNNNNNNNNNNNNNNNNNNNNNNNNNNNNNNNNNNNNNN
NNNNNNNTATATATATATATATATATATATATATATATATTTATTTATTTATTTATTCTGTTTTCAAATCAG
TAGTTATTTCTCCGACCCAGAAGTTCGGTATGTTCTTCGTAGGTTCTGTTATAGATCGAGGATCTATGTC
TCCATCCCATAAGTTCTTCCTTTTGTTCTTCGTGGGATCTATCTCCATCCCAGAAGTTCTTCGTTCTTTG
TGGGATCTGTCTCCAACCTAGAAGTTCCTTGTGTTTTTCTTCTTCGTGGGTTCTTGATATCCTAGATTTA
CTTATTTGTTTAGTAAGTTTTTCTAACAAACTCAAGTTTGTGATAACCTTGAGTTTGAGGGAGGATATTG
ACATAATTAGAGTATTTTTAGGAAATATTATTTTTAGGAAATATTATATTATTCTTGTATATTTTTTCCT
TTAGTGTGTTTTAATATGGTTATATATAGGGCTGTAATCTGTCATTATTATCAATGAATCATCAATGAAA
CACCGATATTCCTCTAACAGCATTTATACAAAGTTGTGTTCAAGAGGCTAGAGAGAGGCCTCAAAGTACG
AGAGTTAATCTAGCAATCCACTAATATTTAAAGAGATTGGACTTGGAATATAGTCTTTTTAAGAGTTTGT
TTCTGTTGGACCATAGACGAAGGGTTTTTGTGAGAAGTCGTACCAGAAGTAGCATTGTGTCTTTGGATTT
ACTCGTACCAGAAGTAGCATTTCCCCATGTGTGTTCAAATTACAAGGGTTTAGACATGGATTAGTATTCA
CTTTGCTTCTCATTTTATATGAAGGATATTAACCTAATTCAAACTTCTTTCCTTGGATCTCTTTCATGAC
GCACCATTCACATTCACACATTGCCAAACTTTTTTTTTTTTTACTTTTTAATTTTCTTCCATCCTGTCTT
CTCTCTTGCATGTTTTCTCCTTTTTTCCCTTATTCATTTTAAAGATAAAAAATAAAAAGAACAATGATTGT
GTCATTTGTAGGCAAAAGATTTAAAATATGTATGGATATGTTGATATATCTTTAAATCTAAGGTTGGATA
TTTTCAACATTATCTTTTATAAATGTTCGTAAAAGAGAACATGTCATCTAGTCTAATATGAATAGAAATA
CTAATAACTATATTTTCATAACTTAGTTTGGGAGTAAAAATAACTTAATTATTTATCTAAATAAATTTTA
AAAATTTTGCAATTTTGAAAATATTTGTCGATATGTACATATCGATCTATTGTTTATCTAAATAAACATA
CATGTATGTTTCATCGATCTATCCATCAATATCATATGTTAATTGTTAATGGTGGGTCATCAAATTGTTC
GCTCTATTGACTACTGTTTGGATCGGCCGTCATTGATGTTGAAGGAAGAAGATAAGAGAGACAAAGAAGT
GCAAGCGATAGAAACAATAAAGAAAGGAAGAAAAAAAATACATTTTTAATTATATCATTGAAAATATATA
TTTAGTAATAAAAAAAATCAGCTTTACCAAGAAAATGTCGAAGAAATGGACTAAGAATGTTTAGAAACTC
TTGAAATTTCCAAAATGGAAACGTTAAAAGTTATGGGGACAAATTGCAAGAAACTTGAAAGTTTGGAAAC
AAAATAATATTCAAACACAAATTCTAATTTTATTTAGAAAAGTTGGATTCATATTGTTTGAGAACCCATC
CAATTAAGAAGGGGAGAGCAAAGTTATAACTTGTTTAAAACACCTGTCATTTTGATTTATTGGCTTTTGT
GAGAAGTCGGTTAAATATACAAAAATCAATTTTAATTTATGAATAATAAAGTTGAAAATTGAATATTTGT
TAGGGAGTCAAATCTAAATTTAACCATTGACTTCAAACTTAGATAAAACATGGTTATATACTTTCTGTAC
ACTGTTATTTTCTTAGAAAAACAAACTCAAATTTTGAAAACTAAAATGGGAGTGGCTAGAGAATACGATG
AAATTGGAAGCTGTATTGTATCAATCAAATTCAAAATACAAAGCCCCACTTCTTCACTGTCATGCCAACT
CAATCAACTTATATTGAGAGCTTACGTTTCCTTCCTCAAACAAAAAAATTATATTGATAGTTGAAGCGTT
TGATTTTTTCTCTTTATCTAATACATCACTCTTCAATTTTTGCTCTGCATTATTCTTCACAAATATTGCT
ACCGGTGTTTTCAATTTCTCTTTTCTTTAGTCTATGCGTTTCTTTCCTAATGGATTCTTCCACTGTTGCA
ACAAAATCACCGACTTTCGGATGGAGTTATGATGTGTTTTTGAGTTTTAGAGGTGAGGATACTCGCACCA
ATTTCACCAGTCATCTTGATATGGCTTTGCGTCAAAAGGGTGTCAACGTCTTCATAGACGACAAGCTTGA
AAGGGGTGAGCAAATTTCTGAAACTCTTTTCAAATCTATACAGGAAGCTTTAATTTCTATTGTTATATTC
TCTCAAAATTATGCATCCTCTTCATGGTGTCTGGATGAATTGGTCAACATAATTGAGTGTAAGAAGTCCA
AGGGCCAGATTGTTTTGCCAGTTTTCTATAAGGTGGATCCGTCGGATATTCGAACACAGACTGGTAGTTT
CGGAGAAGCATTAGCCAAACATCAGGCTAAGTTCCAAATAAAGACCCAAATTTGGAGGGAAGCTTTAACT
ACTGCTGCTAACTTGTCTGGTTGGGATCTAGGAACTAGGTATATATTTTCTTACCGACATTTTGTTTTTT
CTATTCCAAATCTCATTTTTATGTTATGTTTGAATGTATATATGTATGTATATATGTACGTACGTATGTA
TGTGTATGTATGTATTTTAATGAGCACATTGACCAATTAATTTTAATCTTAGAAGATTCATTCTCTCTCA
AGATCCAATTTAATTAATTGTGTTCATCATCTTTTAATTTTGGTATATGATTATGCACTTACCAACAGGA
AGGAGGCTAATCTTATTGGAGATCTTGTTAAAAAAGTGTTGTCTACATTAAATCGCACTTGCACGCCTTT
ATATGTAGCTAAGTATCCGGTTGCAATTGATTCTATACTAGAATATATGAAGCTTCGTTCACATATTAAT
CTTTTTGAGAAGAGCAACAAATTCCATTATCAGACACAACATGAGTATGAGTTTGATACTGATGTTAACA
TGGTGGGGATATATGGCATTGGAGGTATTGGTAAGACAACTTTGGCTAAAGCTTTATACAACAAAATTGC
TAGCCAATTTGAAGGTTGTTGCTTTCTATCAAATGTTAGAGAAGCTTCAAAGCAATTCAATGGCCTTGCT
CAATTACAGGAAAGCCTACTCTATGAGATCCTAACGATTTATTTGAAAGTTGTCAATTTTGATAGAGGAA
TTAACATCATAAGGAATAGATTGTGTTCGAAGAAAGTTCTTATAGTTCTTGATGATGTAGATAAGCTTGA
GCAGTTAGAAGCATTGGTTGGTGGGCGTGATTGGTTTGGCCAAGGCAGTAGAATCATTGTGACAACAAGG
AATAAACATTTACTTTCTAGCCATGGCTTTGATGAAATACACAATATTCTAGGATTGAATGAAGAGAAAG
CTATTGAGCTTTTTAGTTGGCATGCTTTCAAGAAAAATCATCCATCAAGTAATTATTTAAACCTTTCAAA
ACGTGCTACAAGTTATTGTAGAGGTCATCCTTTGGCTCTCGTTGTTTTAGGTTCTTTCCTTTGTACCAGA
GATCAAGTAGAATGGTGTAGTATATTAGATGAATTTGAAAACTCTTTGAACAAAGATATCAAAGATATTC
TTCAATTAAGTTTTGATGGGCTTGAAGACAAAGTAAAGCATATCTTTCTTGATATTTCTTGTTTACTTGT
CGGAGAGAAAGTTAAGTACGTTAAGAATATGTTGAGTGCATGCCATGTAAATCTAGATTTTGGAATTATA
GTACTCATGGATCTTTCACTTATGACGATTGAAAATGACAAAGTACAAATGCATGATTTAATCAAACAGA
TGGGTCATAAAATAGTTTGTGGTGAATCTCTTGAGCTTGGAAAGAGGAGTAGGTTGTGGTTGGTACAGGA

TGTTTGGGATGTGCTTGTTAATAATTCAGTGAGTAACTCATACCCAAAGTATCTTTAATTTACTTATTTC
TAAGACTTCAAATGATGACAAAGGTTTGTCAAATGTTGTTAATTTACTAAATATATGACTTTTTCAGGGA
ACAGACGCAGTTAAAGCCATAAAGTTGGACTTTCCTAATCCCACAAAGTTGGATGTGGATCTACAAGCTT
TTAGAAAAATGAAAAATTTGAGATTACTTATCGTTCAAAATGCAAGATTTTGTACCAAGATTGAGTACCT
ACCTGATAGCTTAAAGTGGATTAAGTGGCATGGATTTCCTCAATCAACTTTGCCTTCGTGCTTCATTACG
AAAAATCTTGTCGGACTAGATTTGCAACACAGCTTCATAAAAACATTTGAGAAAAGACTTAAGGTAAATT
TTATTTCTATGCCTTAAATGGGTAGTTTACAATTTAAAATAATTCATAAATACACGGATCTCTATTATAA
TACTTTTTCAATATAAGGCTTATTTCCTTTTTCTTTTTCTTTTTCTTTTTTTTTTTTTTTTTTTTTTTTG
CGTTAAGTTTCGATACGATGTCTAAAGTTTATACGTATGTAGAAGTTTATATGTCTGAGGAGTTTATATA
TTTATGTGTGTAGTGGAGAGTTGTTTTTTCTAAATTCATATGTTTGTGTTTAGGGTCCAGACTTGAGTTC
ATAAGTTAAAGGTTTCCATTAGCAATTTAAATTATAAGTAATATGTTTTTGTATAACTATCTATCAATGA
TAGACTCTTAGTAGTCATATAGCCTATCTTTAAAAGAATCTTATTTATGATATGATCCTTAAGGATAAAC
TATAGTATTGATACAATATTTTTAGAGCTTAAGCTAAATTTTTCTACATTTGTAATTTTTTTATTGTAAT
ATATGTGTTAAAACTTTGAATCTAATTGTTACATATTTAAAAAAATATATATATATTGGCTCTATATGTT
AATTAAGATCTTACCTGATTGGATTTTTTTTAAAAAAAAATTACCTTCAGATTTGATTTTAATATAAATT
CAAATTAAAAACTGAAACCAAATTTTAAATAAATTGACTAAATCACATTCTCAATCTCTTCAAGGTATCA
ATGGTTATTTTTACAATCTACTGAATTCAAAACAATATATGTTAGGTATTAATGGTTAATTTACTGTAGT
GATTACTTAATTAAGGAAAATTGCATAAGATGACAAAAAAATTTAGAAAAAAACAGCTCATATCACATCT
TTTTTTGCATGTTGCATACATATGACAAATATTATAGTTATCAGAGGGTTATTAGAGTGCTATTAGAGAG
CTATCAGAGGGTTATCCGTTTTTAAATTTGCTATTTTTATCACTTTGTTATCCACTTTTGAATTTGCTAC
TTTTACAATTTATAAAATGCAGTGACATAGGTTTAATATCTTATGCATAAATTATACATTTTAAAACTTC
TCTAATTTGATGCATTTTAAACTTATAATTACAATACAGTTCAACTGGACACAGTATCAAACTAATAGTA
GTAAAAACTAAAAGTGTGTTGTTGTCAACATGAGCGTTGCTCAATTAATACACTTGTATTATCAATTTCG
AGATTAGAGATACGATGTTATGCCTTCACCCACCTTTAATTACAATACCTTAAAAAAACAAAAAAGGTGT
GTTTCTATTTTATGCAATAGTTGTGGATTAATGCAAATAGATGAATATTCTTGATATGGATTGATTTTAT
CACTGATTTACTTTCATACATAGAGTTTTATTAGTTTGCCTAAATACTGATGAAGGGTTATCATAGATTT
TTTACATATATTCCATTTTTTCTTCTATATTTACAATTATTCTTTTAGCTACTATTTTATCAAAATTTTG
TTTGATATGCCAAGTTACATACCCCTTTCCCTTAATTATATTTTGGTTTCTTTTTCGATATGTTTTAGGA
TTGTGAAAGGTTGAAGCATGTTGATCTTAGCTACTCTACTTTATTAGAGCAAATTCCTGATTTCTCTGCA
GCATCAAATCTTGGAGAGTTGTATCTCATCAATTGCACAAATTTAGGAATGATAGATAAGTCTCTTTTTT
CTCTCAATAATCTTATTGTCCTAAACCTTGATGGTTGTTCTAACCTTAAAAAGTTTCCAAGAGGCTACTT
CATGTTAAGTTCTCTTAAAGAACTGAGGCTCTCTTACTGCAAAAAGCTTGAGAAAATTCCAGACTTATCT
GCGGCATCAAACCTTGAGAGATTGTATCTTCAAGAATGCACAAATTTAAGATTGATTCATGAATCTGTTG
GATCTTTGGATAAGCTTGACCATTTGGACCTTAGACAATGCACTAACCTTTCAAAGCTTCCAAGTCATCT
CAGGTTAAAATCTCTTCAAAATTTAGAACTTTCTAGGTGTTGTAAGCTTGAAAGCTTCCCAACAATCGAT
GAAAACATGAAATCTTTAAGGCATTTGGATTTGGATTTTACTGCCATAAAGGAGTTACCTTCATCAATTG
GATATCTTACTGAGCTCTGTACATTAAATCTTACCAGTTGCACAAACCTCATCTCTCTTCCCAATACAAT
TTATTTGTTAAGGAATCTTGACGAACTTCTTCTTAGTGGGTGTTCTAGATTTAGAATATTTCCCCATAAA
TGGGACCGAAGCATCCAACCAGTATGCTCTCCTACAAAAATGATCGAAACAACTTCATGGAGCTTAGAAT
TTCCCCATTTACTAGTTCCAAATGAAAGTTTATTTTCCCATTTCACTTTATTGGATCTTAAATCTTGCAA
CATATCAAATGCAAAGTTTTTGGAAATTTTATGTGATGTTGCCCCTTTCTTATCTGATCTACGCCTGTCA
GAAAACAAATTCTCTAGCTTACCCTCATGTCTCCATAAGTTCATGTCCTTGTGGAATCTTGAATTAAAAA
ATTGTAAGTTTCTTCAAGAAATTCCAAACCTTCCTAAGAATATACAAAAAATGGATGCCAGTGGTTGTGA
ATCATTGGTTCGAAGTCCAAATAATATTGTGGATATAATATCAAAAAAACAGGTTCGCCTCTAATTTCCC
ATCAATTTATATTCTTATCTTGTAAACAATTTAATGCATTATGAATTCTTATTCTCTATAGGACCTCACA
TTGGGTGAGATTTCAAGAGAGTTCTTATTAACGGGGATAGAGATTCCAGAATGGTTCAGCTATAAGACTG
CCTCCAATTTGGTGAGTGCTAGTTTTTGTCACTATCCAGACATGGAAAGAACTTTGGCTGCCTGTGTTAG
TTTCAAAGTGAAAGGAAATTCATCTGCAAGTGGGGCCCGAATTTCATGCAATATATTCATTTGCAATAAA
CTCCATTTTTCTTTTTCAAGACCATTTCTTCCATCAAAATCAGAATATATATGGTTAGTAACAACTTCTC
TAGCGTGGGGTTCCGTGGAGGTGAATGATTGGAATAAAGTTTTGGTCTGGTTTGAGGTTCATGAAGCACA
TAGTGAGGTTAATGCAACTATAACAAGGTGTGGTGTTCATGTCACTGAAGAGCTCCATGGGATACAAATG
GATGTCAAGTGGCCGATGGTAAATTATGCTGATTTTTATCAACTGGAGAAATTGCAACGTCTGTAAGTTG
ATTGTTTACTTGTTATTTATTTAGTCTCTTTCTTTTTCTTTTTTTTGGAGTGAAAGTATATAATAGATCT
CAAAGGGGGAGATGAGTATGTTATGATTTTGTTTTTTTAATGGCATAGGGATATTGAGGATCTTCTTCTC
AAAAGCTTTTTAGAATCAGTCTCCTGCTGGTCAAATTCCAAAGCAATGTTGCATGCAGGAAATTATGATC
CAGAAGCAATAATTGATTCCAACATACAACCTATGATTTTCCCATTGCACGTAACATATAATGGTGAAAC
AGTAATATGTGGAATGGAAGGCATGGGAGATACTACACTCGCCAACTCTTTATGCAATAAATTTAAATGG
ATGAAGGATAACCTTTTCGAGATAAAGGAGCACCATTACAGTGAAGCATTAGATAATTCTACAAGCATTT
TCCATATCCGAGGAAGAGAGCTCCAGAGATTTTCCTGGTCATGGGCCGCCCACCATCGTAAGCGTGGAGA
TGGTAAAAGAGGAACCAATATCACAACCCATACAATATCCTCCAAATGCTATTTGATGCTCTTTCATGAA

GTCGAGAACTGTAATGATATATTTGACTGGGTTGGTACACAGCGTTGGATTAAAACTTCTGGCAGTTCTA
ACGGAAGAGGTGATGTTCAGTTTCTGATTAAAAGGGTTGATATATCATTGCTCTGAAAAGCTCTCAATTT
TTTAGTAAAAATTTACATTTAAACACCCTTTTTAATCTTTTTGGGACTTGTATTAATTTAAACACTATCA
TAATTAATATCAATTTAAATGCTCAACAACTTTCGTAAGTTTACATATCAATTGAACACCCTTTTTATTT
AATTTAGATTTTGTTCAAAAATATATTTTTTTTTTCTTTTAGATTGGAAAAAGATGTGTTATATACCCTA
GATGCATCAAAAACATACGACGGATAAGTTAGTCTAGCAATGCACTAATTATATCAAATATGAGATTCTA
TCTCAAAACCAATTAACAATGAAAGGAGTAGCCTATCTACCTTATAAGAGATGTAGTTTCCTTGATTTTT
TCAATATGGGACTCAACAACATCTCAATAGGAAACTCACTCTATGCGGTAACGTCAATCTTCTACCTGTT
GAAACTCACTCTTCTCTTTTGTTTGCAGAAAAGTCTCACCCACTCTCTCACTCTTCTTACAGTATTTTTT
TTCTCCTTCGGTTTTGGTTTCTATATTTCAAACATCAAAAGTTGCTTTGCTACTACCACTTTATTATTTT
CTTTTTATTTGCTGTTGTGATCCTATCTTCTGCACAAGCTTCAATCTTCTGCACTTCTAAACAAAACCAA
ATGAAATTAGTTACATTTAGATTTAAACCTCTGTAAATAATTTTTGTTAGAAAAAGGGACAACAAATAAT
CAAATAACAGCTTTAAATCCTAGCTTAAAGCAGGAAACATATAGATTAAGAACAAGAAAAATAATATTGA
TCATCTGTTCATTTGTCGTTTTTTAATCATCTTCTTGAAGAATATGTTAGAATAAAAAGCTCTACCTTCT
TCTAAGAGTGGATGTAAGCAATTTTTTGACGACTATAATATACACTTGTATATTCTTCTCTTCGTCCGCC
GATATGCACATTTGAAAGATGTTTTCACTTGAAACTCTATTTTCTCTTCTCTCTCGGTGAGCATGCAAAA
TAAAAATCAGAAAAGAAGGGAGAAAGTTGAGAGGACAGCTTGCAGAAAGATGAAAAGGTGAGAGGAAAGA
GGTAAAAAAAAAAAATTCTGCTTTGTACACATGTCTCAAGACACTAATTTCAAAATTCAAAAAAGGCCTA
CTTTTTCTTGACATTTGCATTTTTTGTTTTTGTGAGAGAGAATGGTTTCTTCTGTTCTTGGAGATGAATC
ATCTTCTTCTTCTCCCAATTTCAATTACCATTATGATGTGTTTTTTAATTTCAGAGGAGAGGATACTCGC
TCCAATTTTATCAGTCATCTTCATATGGCCTTGCGTCTAAAGGAAATCAACATCTTCCTAGACGACAAAC
TCAATAGGGGTGACCAAATTTCTGTTGAAGTTAAATTAATCTAAACTTAATACATGAATTTGCATTATTA
AATATGCATGAATAGTTGAGATACATGGAATAGTTAATCACTAAATACATTGATATATGAATAGTCACAT
GTATTGATATTTATTGTTAATGACTTTTAATATACTTTTCTACTAGTATAAATATGTGTAAGGTTTCTCA
TTTGTAAAATAAGAAAGAAAGTAAGAAATTCAAGTTTAAGAAATATTATTCAAGTTCTTTCTTCTCAATT
GTGTGAATAAGAGAACAATCTTCTTCTCTTGTTTCTTGATTTGTATTGTAAGGGTCTATTACGTTTCCAA
CAAGTGGTATCAGAGCTCAAGTTAGATATGGCTTCAAATGGTAACATGTTGCAACACCAACTTCCAAGGT
TCAGCGGAAAGAATTTTAATCAATGGAGTATTCAAATGAAAGTGTTATATGGCTCTCAAGAATTGTGGGA
TATTGTTGAAAGAGGATACACTGAAGTTGAGAATCAGAGTGAGCTCACAAATCAACAACTTGTTGAGTTA
AGAGAAAATCGTAATAAAGACAAAAAGGCTTTATTTTTCATTTATCAAGCTGTTGATGAATTTATTTCCG
AGAGAATTTCAACAGCTACTTCTGCAAAGGCTGCTTGGGATATTTTACGATCTACCTATCAAGGAGAAGA
TAAGGTAAAGATGATAAGGTTACAAGCTCTCAGATCCGAATTTGATTGCATTAAAATGAAAGAAACTGAA
ACTATTGAAGAATTTTTCAATCATATTCTTGTAATTGTCAATAGTTTAAGATCAAATGGTGAAGAAGTAG
GCGATCAAAGAGTTGTTGAAAAGATTCTTAGAAGTATGCCAAGAAAATTTGAGCATATCGTCGTTGCAAT
TGAAGAATCGAAAGACTTATCTACGTTGTCTATAAATAGCTTGATGGGTTCTCTTCAATCCCATGAGCTA
AGATTAAAACAATTTGATGTTAACCCCGAGGAAGCTTTTCAAATGCAAACTTCATTTAGAGGCGGTTCAC
GTGGAAGACGTGGTGGTCATGGAAGACGAGGAGGTGGAAGAAACTATGATAATAGAAGCGGAGCAAATTC
TGAAAATTCACAAGAAAGCTCTTCTTTATCTCGAGGAAGAGGAAGCGGAAGAAGAAGAGGCTTTGGCAGA
AACCAAGGAGGTGGTCGTGGTAATTTCTCTCAAATTCAATGCTTTAATTGCAGAAAGTATGGTCATTTTC
AAGCAGATTGTTGGGCACTAAAAAATGGAGTTGGAAATACCACCATGAATATGCATAAAGAACAAAAGAA
AAATGATGAAGGCATTCTATTCCTCGCATGTAGTGTTCAAGACAATGTTGTAAAGCCTACATGGTATCTT
GATAGTGGTTGTAGTAACCACATGACAGGAAATAGAAGTATATTTGTTACTTTTGATGAATCTTTCCAAA
GTGAAGTGAAGACGGTGATAATACCAGACTACAAGTCAAAGGCCAAGGTGATATTCTTGTGAAGACAAAG
AAAAGGACAAAACGAGTTACAAATGTGTTCTATGTTCCAGGTCTAAAGCATAATCTTTTGAGTATTGGCC
AACTGCTTCAACGAGGTTTAAAAGTTTCATTTGAAGGTGACATATGTGCAATCAAAGATCAAGCCGACGT
TCTTATTTCCAAGGTAAAAATGACTGCTAATAAGATGTTTCCTCTTAACTTTACATATGGTCAAATATCT
TGCTTCAGCAGCATATTGAAGGATTCATCCTGGCTTTGGCATTTTCGATATGGTCACTTAAACTTCAAAT
CACTATCTTATTTGTGCAAAAATCATATGGTGAGAGGTATACAAAAATATCAACCATGAGACAAATATTT
GTGAAGTGTGTATTCTTGCAAAACATCATCGAGATTCATTTCCAACTGGAAAAGCTTGGAGAGCCTCTAA
ACCTCTCGAGTTGATTCATACAGATTTGTGTGGTCCTATGCGAACAACAACAAATGGAGGTAATCGATAT
TTCATAACCTTCATCGATGATTTCAGTAGAAAGTTGTGGATTTATTTTTTGAAAGAAAAGAGTGAAGCAC
TTGTATGTTTTAAATCCTTCAAAGCTTTTACTGAAAATCAAAGTGGTTACAAGATAAAAACTTTGAGATC
TGACCGTGGTGGAGAATATATAGTTTTTGGTAATTTCTTCAAGGAGCAAGGAATTCATCATCAAATGACA
GCTCGAATGACTACACAGCAAAATGGAGTTGCAGAGAGAAAAAATAGAACAATCATGGAAATGGCGAGAA
GTATGCTAAAAGCAAAAAATCTGCCAAACGAATTTTGGGGAGATGCTGTTGCATGTACTGTTTACATTCT
AAATCGAGCTCCAACAAAGAGTGTTCCAGGTATGACTCCTTATGAAGCATGGTGTGATGAGAAACCATCT
GTTAGTCATTTGAAAGTGTTTAGGAGTATAGCTTATTCTCATATTCCAAATCAGCTAAGAGGCAAGCTTG
ATGATAAATCTGAAAAATGCATTATGGTAGGTTATAATGAAAATTCTAAAGCTTATCGATTGTATAATCC
TGTGTCAAGAAAAATTATTATCAACAGAGATGTGATTTTCAGTGAAGATGAATCATGGAACTGGAATGAC
GACGTTGATGAAGCTAAAAGTCCATTTCATGTTAATATTAATGAAAATGAAGTTGCTCAAGAATTAGAGC

AAGCGAAAATTCAAGCGGTGGAGTCATCTTCGTCCTCAACGTCATCTTCCACAAGTAATGATGAAATCTC
ACCAAGGAGAATGAGGAGTATTCAAGAAATTTATAATAACACTAACAGGATTAATGTTGATCATTTTGCT
AATTTTGCATTATTTGCTGGTGTTGGTCCTGTAACTTTTGATGAAGCCATCCAAGATGAGAAATGGAAGA
TTGCAATGGATCAAGAGATTGATGCGATAAGAAGAAATGAAACATGGGAGTTGATGGAGCTTCCGACAAA
CAAACAAGCGCTTGGAGTAAAATGGGTGTACAGAACAAAGTTGAAGTCAGATGGTAATGTTGAAATATAC
AAGGCAAGACTTGTTGTAAAAGGCTACAAGCAGGAATATGGTGTGGATTATGAAGAAATATTTGCCCCTG
TGACAAGAATTGAGACCATTCGATTGATTTTGTCATTAGCTGCTCAAAATGGATGGAAAGTTCATCAAAT
GGATATAAAATCCGCTTTTTTGAATGGACACTTGAAGGACGAGATATTTGTTGCACAACCTTTGGGTTAT
GTGCAAAGGGGAGAAGAAGAAAAAGTGTACAAGTTGAAAAAAGCTTTGTATGGATTGAAGCAAGCTCCAC
GAGCTTGGTACAGTCGTATCGACAGCTTTTTTCTAAAGACAGGATTTCGAAGGTGTCCATATGAGCATGC
ACTCTATGTCAAAGAAGACAAGTATGGCAAATTTCTCATCGTTTCTCTTTACGTTGATGATTTACTTTTT
ATTGGAAATGATAAATTTTTGTGTGATGATTTTAAGAATTCCATGAAAAAGGAATTCTAGATGAGTGATA
TGGGTCTCATCCATTACTTTCTCGGAATTGAAGTTAATCAAAATGAAGGAGAAATTGTCATTTCACAGCA
AAAGTATGCTCATGATTTACTAAAAAAATTTCGGATGGAAAATGCTTCACCTTGCAACACTCCCATGGAT
GCAAATTTGAAATTGTGCAAGGATGATATTGGAGAAGCAGTCGATCCAAGTTTATATCGAAGCTTAGTTG
GAAGCTTAATGTATTTGACAGCAACAAGACCTGATATTTTATTTGTTGTCAGTATGTTAAGCAGATTTAT
GACAAACCCGAAAAGAAGTCATTGGGAAGCAGGAAAAAGAGTTCTTCGTTACATTCTTGGCACCATTAAT
TTTGGAATTTATTACAAGAAAGTTTCAGAATCAGTGTTGTTTGGTTTTTGTGATAGTGACTGGGGTGGTA
ATGTGGATGATCATAGAAGTACATCTGGTTATGTTTTTAGTATGGGTTCAGGTGTTTTTTCATGGACTTC
AAAGAAACAATCTGTTGTTACCCTTTCTACAACCGAAGCAGAATATATCTCGTTAGCTGCAGCTGGATGT
CAAGCTTTATGGCTTCGGTGGATGTTAAAAGAATTGAAGTGTACTCAAAAATGTGAAACTGTTTTATTTT
GTGATAATGGATCTGCCATAGCATTATCAAAGAATCCAGTTTTCCATGGAAGAAGCAAGCATATTAGAAT
CAAATATCATTTTATCAAAGACTTGGTTAAAGATGGAGAAGTGATAGTAAAATATTGCAAGACTCAAGAT
CAAGTGGCTGATATTTTTACAAAGGCGCAGAAGTTTGACTTATTTGTTAAATTCAGAGGAAAACTTGGGG
TTGCTCAAGTCTAGATTAAGGGAGGATGTTGAAGTTAAATTAATCTAAACTTAATACATGAATTTTCATT
ATTAAATATGCATGAATAGTTGAGATACATGGAATAGTTAATCACTAAATACATTGATATATGAATAGTC
ACATGTATTGATATTTATTGTTAATGACTTTTAATATACTTTTCTACTAGTATAAATATGTGTAAGGTTT
CTCATTTGTAAAATAAGAAAGAAAGTAAGAAATTCAAGTTTAAGAAATATTATTCAAGTTCTTTCTTCTC
AATTGTGTGAATAAGAGAACAATCTTCTTCTCTTGTTTCTTGATTTGTATTGTAAGGGTCTATTACGTTT
CCAACAATTTCTGAGTCTCTTCTCAAATCTATAGAGCGATCTAGACTTTCCCTCGTTATTTTCTCTAAAA
ATTATGCATCTTCAGCTTGGTGTTTGGATGAACTGGTGAAAATAATTAAGTGTAAGAAATCCAAAGGACA
AGTAGTTTTTGCCAGTGTTCTACAAGGTGGATCCATCCCAGGTTCGAAAACAAATCGGTGGGTTTGGGGA
AGCATTGGCCAAACATGAAGCTAATAAGTTATTAACCAACAAAATACAACCATGGAAGGAAGCTTTGACT
TTTGCTGCTGGTTTGTCTGGTTGGGATCTAGCAAATTGGTAATTCTTTTTTTAATCTGGGAAGACTCATT
GTCCAAGTGAAGTTTAAATTTATCTACTATTTCCGATTTTCATTTTTTTTTTTATGATGTTACTTTACAA
CAGCAAGGATGAGGCTGAACTTATCCAAGAAATTGTTAAACGAGTGTTGTCTATAGTAAATCCAATGCAA
TTACTACATGTAGCCAAGCATTCAGTTGGACTTAATTCTCGACTAAGGAAAATTGAGTTGGTCTCTCATA
TTGGGTCCGAAGGTGTTAACATGGTGGGGATGTGTGGCATTGGAGGCATTGGTAAGACCACTTTGGCTAA
GGTTTTGTACAACAAAATTGCTTACCAATTTGAAGGATGTTGCTTTCTACAAGATGTTAGACGAGAAGCT
TCAAAGCATGGGCTTGTTGAACTACAGAAAACCTTACTCAACGACATCTTAAAGGAGGATTTGAAGGTTG
TCAGCCGTGATAGAAGAATTATCATAAGGAGTAGACTGTTCAAAGAAAGTTCTTATAGTTCTCGATGATG
TGGATCATCTTGAGCAATTAGAAGCACTAGTTAGTCGTCGTGATTGGTTTGGTCAAGGTAGTAAAATCAT
TGTGACGACAAGGAATCAACATTTACTTTCTAGCCATGGATTTGATAAAAAGCATAAAATTCAAGAATTG
AATCAAGACCATGCTCTTGAGCTTTTCAGTTGGCACACTTTTTAAGAAAACTCATCCATCAAGTAATTAT
TTAGGCCTTTCAAAACATGCTACAAATTATTGTAAAGGTCTCTCTTTGGCACTCGTTGTTTTGGGTTCTT
TCCTTTGTGTCAAAGATCAAGCAGAATCGAATGTTATATTAGATGAATTTGAAAACTCTTTGAGAATAGA
TATTAAAAATGTTCTTAAATTAAGTTTTGATGGACTTGAAGACAAAGCAAAGGATATTTTCCTTGATATT
TCTTATTTACTCGTGGGAGAAGAATACAATTGTGCTAAAAAGATGTTAAGTGCATGTCAGATAGTTCTCT
TATGGGTCGAATACACGAAGAGGTCTTGGCTCGCTCAGAATAGAAGGGGGTCCATTTGAACATAGATTTT
GAAATTATGATACTCATGGATCTTTCACTTCTTACGATTGAAATGGGTAAAGTACAAATGCATGAGTTAA
TACAACAAATGGGTCATAGCATAGTTCATAATGAATCATCTAAGCTTGGAAAGAGGAGTAAGCTGTGTCG
CTATGGTTGGTGCAGGACATTTGGGAGGTGTTTGTTAATAATTCAGTGAGTAACTCCTACCTAAAGTATT
AAATAATTTACTTATTTTCAGGACTTCACCTGATAATAATTGTTTGTCAAAGTAAGTTGTCAGTAATGTT
AAATAACTAAATATATGACTTTGTAAGGAACAGATGCAGGTAAAGCTATAAAGTTGGACTTGCCTAATCC
CACAAGGCTAAATGTAGATCCATGAGCATTTAGAAGCATGAAAAATTAGGATTGCTTATCATTTGAAATG
CACGATTTTGTACAAAGATTAAGTACCTACATAATAGCTTAATTAAAGTGGATTGAGTGGCATGGATTTA
CTCATCGATCTTTGTCGTCGTGCTTCATTACCAAAAATCTTGTTGGACTAGATTTGCGACATAGCTTCAT
CAAAAGATTTGGGAAAAGACCCAAGGTAAAATATATTTCTGTCACATGTGAATTTTGACTTGAGATTTCT
TCATTGTTTTTCTTAAAAACTTTTAATGGGTAGCTTAACTACATTTAAGGAAAAGCACAAATATGTGATT
TAGCAAAATTTATGCCGAATTAACTCCTACTAGTTATATATTTTTAAAGAATTTGCAAAATATAGCAAAA

TTTATTAATGATAGGTAGATCTATCACATAACAATAAACTCCTACTAGTCATATAATCTATCATTAATTG
ACTTATATTAGTGATATGATATAATAAACTCTAAGAGTTGAAGCTAAATTTTTCTATATTGATATTTTTT
TGCTTTGTAATATATATGTCATCATTTTGAGTCTGGTTGTTATATCTGCAATTATCATAATGCAAAATAT
TATATGGAAAATTGAATCAGATGATAAAAAAAATAGAAAAATAGCAACCTATTACACCTATTTTTGGCAT
ATTGCAAATATGTCAAATATGACAAGCAATTAGTTATATCAAAGGATATTAGAGAGTGATGAGATGGTTG
TCCACTTTTAAATTTGCTGCTTTTGCACCCTTTTTAGAATGGCTACTCTTCGTCATCTCCCTCAATCCTA
CACCATTGAGAAAAATGATTTTGTCCTCTATGTGGACAACCGCACGAGAGAACTAGAGCTACTTATCACT
GAGGTAGGACCATATAAATCTACCTCTATCGTTGTCACCCTAGAACCATTGGAATGTCTTAAATCCACTT
TCAAAGCTCTCCTGGACACGCCACGAACGACAAGATTCTTTTTGGAAAGGCGTTTTGAAGAATATTGTTG
TGGGTCCAAAAGACTTACAACAGGAAAGGTTATATAGCAGAAATCTACAAAGTGGATAATAGAGGAAGGA
GATGTTGCATTCTTGTTCTTGAAGGGACAGATAAATCGGGTTGGGCTCATTTTGCGGATCTTCTAAATGA
TAAAAAGGAAGCTTCCACAAAAGTCAAACTTACTACAAGGATGGTTGACAAAAGAAAGAAAATAATCTGC
TTTAGCTCAGATACCGATTCAGACAGTCAAAGATCTTATGCGAAAGTGGTTATTAGAGGAAGCTCATCGG
ATGAAGAAATTCACCATATATCTACAAATGCAAATACTAGGGAAAAAAAGAAGAAGGGGGAACAGACTGA
CGAAACTGCTTTCGATTGGAACAAAACTGTTGTACTTATAACGAGATACTTCCACGATGACTTGAAGAGA
ATTATAGAGAAACTGAAAGAACAACTTGACCTACCTATCAACTACAAACCTTTCCATGCCTACAAAGAAT
TAATTTTCTTTGAAGACAAAGAGCAAGCAAAGCTGATTTGCAAAAACAGAGGATAGACAACTGTGGGACG
GTTCTATGTGAAATTTAAAGAATGGAACCACAAATCACATGCCACTCCGAAAGTAGTGCCGAGCTATGGT
GGATGGATCAAAATCCGGGTATTCCATTGCACGTGTGGAACTTAGAAAGCTTCCTTCAAATTGGAGACGC
ATGTGGAGGATACATTGATGTGGCCCGCAAAACCAGAGAACAATCAGATCTCATTGAAGCCTCTATCAGA
ATTAAAGACAATTATTCAGGTTTTATCCTAGCTTTTATTAAGTTGTTTGATAAAAAGGGCAGAGTTACAT
TGTTCAATTTATTGCTCAAGCAGAAGGGAGATGGCATTTGGAAAGAAATCCAAGCATCCACGGTTCTTTT
ACCAGAAAAGCGGTGATTAATTTTGACGAATTCAATTTGAAGTGTGAGTCTTACTATTTTGAAGATAACT
TAGCCATCTCGCCGAAAAAAATCTCGTCGGATATGATTAGAAAGAAAAAAGACGAACAGAAAATCCATTG
CAATGGCTCGCTTCAAGAAATTAATAAAAAAAATGGGAACAATGATGGAAAAATTAAATTATGACGGCAA
CAGTGACTCAAGTGAGAAAAGAAAGATGGAGATAGACAATGATTTGATGTTGACAAGAAAAAAAGGAGGG
AAAAAATTTTTCAGAGGAGAAAATTCAAAACGGTAGGCCCAAAGAAAGGAAAAAGAAAGGTTTCCTTTGA
TTATCCCAAAAGCAAAATTTTCTTCTATGATCCTAAGAGTGCCCCAACGGCGCCCATAAGGATGAAAATC
CCAGAGGGAAGCCCAAAGAGAAATCTCCTGAAAAATAACGAAAAAAGAGACCAGAGGAAAGGAAAGCAGA
TCTACAAGAAAAAAGCCCACCATCACGTGTCGGCCTATTGAAGGAAAGTAGCAAAAAGCAAGGCGGGCTT
AAAATTCATCCTAAAGAAGATGAAAATAGGCCTCCTGCTATCAATCTCACAGTGGATCTTGGGCCTCTGT
CCCTTATATCTGATGAATTCCTTTCCAGCCCAGAACAGTTCTCCTTCTCTTCATCTCCAACTTCCCCTTA
CGCACTCAGATATTATAAAAGACAGTATAGGACAGATGCTCACAAATGAAAATGAAAACAAGGATAAAAA
CAAGAAGAAAAAGGTGAAGGTGAAGGTAGCAATTTCAAGAAGTAGATTGTGGACTGTCTTAAAATAAACA
ATCTCAAATTGGCTTCTATTGATTTAGATATTAATATGAATGTTGTTTCTGATGTAAACAATGGCAGCTC
CATTGCCAGTGTCATTGAAAAGAGGCAAGATAATTATTTATGAAAGTTATCAGTTGGAACATTAATTAGA
GGGTTATGCTCTCCCTCAAAAAGAGCTCAAATAAAAAACCTCATTTCTTGCTACCATCTTGATTTCATGA
TTTTAGTTGAGACTAAATTAAAAGCTGTCACAAAAAATATTGTTAAATCCTTATGGAGCTCGATCAATAT
TAAATGGCATGTTGTAAATGTTGTTGGTAATTCAGGGGACATCCTGATTATGTCGGACGACCTCCATTTC
AATATGACAGATTTCATACAAGGGCTTTATTCCATCTCCATCAAAATAAGTGTTCCAAATGGGTCGATTT
CCTCGGCTTGGTGGCTTTTTGCTCTGTATGGATCTGCTAGCCATAGAAACAGAAACAACTTCTGGTCCGA
ATTAGAAGAGTTCAATATAAAGTCCTCCCTAAATTGGCTATTGGCTGGTGACTTTAACGTGGTGAGATAC
ACCTCTGAGACTTCGGCACAAAATCCGAGCAATTACAGTATGAGAAAGTTCAATGCCTTCATATCAAACA
CAACCTAATCGATCCTCCTCTCACCAATGCCAATTTTACTTGGTCCAATCTTAGAATTTAATCGGTGTTA
TTAAGAATTGACATACTTTTATGTTCTGCAGGTTGGGAAAATATGTCCTTGATGCATTATTCCAAAGTCC
TCTCTAGAATCACATCTGACCATTTCCCGCTTCTTCTTGAGTCATCCAATATCAGTTGGGGCCTTCTCCA
TACAAATTTATTAATCAGCATTTAAAGGAAACTTGGTTTAAAAACAACATTGACATATGGTGGAAAAATT
CGATGTAAAACGGTCATATGGGATACTTTCATAAGGAAGCTCAAATAAATATCTTTCTCAATCAAGACTT
GGAGCAAAAAAAAATAAAAAAGTTAATGAGGATGATAAAAGAGCCTGGATTAAAGAAATCGATGAGATTG
ATAAATCTGAAGCTTCTAACCAAATTTCAAAAGCTCAGAGCATGCGTAGAACTTTCTTAAAGGCCGAGAT
CAACCAATTCGATTATAAAGAAGCACAAATTTGGTCTCAGAAATGTAAGAGACTTTGGACCTTAGATGGA
GACTAAAACTCATCCTTTTTCCACAGAATTTGCTCAAATAGACAAAGAAGAAGTTGCATTTCCAGTATTA
TTTCAAAAAGAGGGGATCTCTACAGTACCAACGAAGAGATCGAACAATCTTTCTTGGAACATTTTGAAGA
CATATATAAGAACAACAATATTGAATCTCCTTGGCCTATAGACAACCTTCATTGGTCTCCTATTTCCTCT
AGGCAACAAGAGGACCTATGCTCTTTCTTCTCTGAAGAGGAAGTTCACATAGCTCTTAAATCCTTCACAA
ACAACAAAAGCCCTGGGCCCGATGGGTTTACTATGGAGTTTTACAAAAGAACTTGGCATCTTATTAAAGG
CGACATCTGTAATATCTTCAATGACTTCCATGCCAACTATATTATTAACAAAGCTATAAATGCTATATAC
ATTGCTCTGATTGCTAAAAAAGATAAATGCTCGGTTGCTTTGGATTAAAGGCCTATCAGCTTAACTACAT
CTCTATACAAGTTATTGCAAAAGTTATAGCAGAAAGGCTAAAAGAAACTCTTCATACTGTTGCTGAAAAC
CAGATGGCGTTCATAAAGGGAAGACAAATTACGGATGCCATTTTGATTGCGAATGAAGCAATAGATTACT

GGAGAGTCAAAAAGGTGAAAGGTTTTTTGATCAAGCTGGATTTAGAAAAGGCTTTTGACAAAATCAGATG
GAGCTTCATAGACTATATGCTTCTTAAAAAGGGTTATCCTCACAGATTGAGAAATTGGATTAAAGCTTAC
ATCAGTAGTGTCCAATACTCCATCATTATCAATGGCAGACCGAGAGGCAAATTCCAACCTTCACGTGGCA
TTCATCAAGGAGATCCCATCTCTCCCTTCATTTTTGTCCTTGCAATGGACTACCTCAGTCGGCTGATTGA
AACGCTGGTTGATAAAATTAAAGGGGTGAGTTTAAATGACAATCTTAATCTTACACATCTCCTTTTTGCA
TATGACATTCTACTATTCGTTGAAGATAATGAAGACTCCCTCAAAAATCTCAGGAATGTCATTCATCTTT
TCCAACTTGCTTTGGGCATAAATGTCAATTTAAACAAATCCACCATCTCTCCCGTTAATTTGGGTGCTTC
AAGAACAAACCAGGTTGCTACATCATGGGGTATTAATACACACTTTCTTCCCACAAATTACCTGGGAGTG
CCTCTCGGTGGGAAGCCCCTTTCCAAAAGCTTCTGGAAAAATATTAACGAGAAAATCAGCAAAAAAAAAC
TGTGCAGCTGGAAGTACTCCTTGCTCTCTAAAGAAAGGAAAATTACATTGATAAACTCCACCCTTTCCAG
CCTTCCCACCTACCAGCTTTCGGTTTTCAAAACTCCTTCCTCCATCTACAAAAGTGTTGAACAAAGTTGG
AGAAACTGTCTTTGGAACAGTCCAAATGAAACCCAAAAATTGCATCTGGTTGGGTGGTCGGTGGTTTCTT
CGCCAAAAGAAAAAGGGGTCTTGGAATCAGCCGCATCAAAGATACAAATTTCGTGCTTCTAAACAAGTGG
TTATGGAGATTTATTTATGAAAATAACCCCTTATGGAAAAGGATATTATAACAGCCAAATATGATAGCCC
AGTTTTGAGAGATCTACCATATAAAAGTAAATATAACATAAGCAGAGCCCCATGGCCTTCTATCATTAAA
GGTCTGAACTGGTTCCGCTCCCAAGTTAGCTGGAAAATTAGAAATGGCAGTAGCTTTTTTTTTTTTTTTG
AACAGCCACTGGCTCTCAAACAGCCCCTTTTCTACTCACTATCTGAGACTTTTTGCCTTATCTACTAAAT
AAAACAGCTCTATAAGAGACATGTGGAATACTGACACGCTTGATTGGGAGCTTAGTCCAAGAAGACTTCT
GAGGGACTGGAAAATTTCTCTATGGGATGAGCTTAAAGCCTCTTTAGATGCTTAAATTATTGTTGCTGGC
AGTGACACCCCGAAGTGGAAACTTAATTCTGATGGCATGTTTTCGGTTGCTTCGGTTAAGAAAGTGATAC
ATTTAAATGATTCGGGGGGATTGCCTGTACCGGATCAGAAAATTTTTACAAACCTGTGGAAATCTAGTCT
GCCCAAAAAGTGAAAATTCTTTATATGGGCTATTCTACATGACAGCATAAATACAGCCGATCAGCTTGTC
AAAAGGCTCCCCAATCTATGTTTGAGGCCAAACTGGTGTGTTCTCTGTAAAAGTAATTAAGAAGATAGAA
GCCATCTATTTATACCTTCCCCCTTTGTTGTGTTCATTTGGAACCACATTGGCTCTCTCTTTAATAGAAG
CTTCTTTTTCCACAGTCCCAAGGAACTTTGTCAACATATTTGTGGCTAGAATGAAAAAAGCAAAAAGAAC
ATCATCTTTTTCAACACTATTGCTTGTGCTCTATGGAATATTTGGTTGGAAAGGAATGATAGAATCTTCA
ATAACAATGAGAAGACAGCTAAGGATACTTGGGAAAAACATTAAAGCTTTATCAAGGCTGTGGACTAGCA
GATCCAGACTTTTTTCAAATTACACAGCTTCTTCTATTGCTTTGAACTTACATGCTTTTGTATAATGTTA
CCTTTGGATTGAGGGTTTATCTCTAGCCCTTTTTTTCTCCCTGCTTCTTTGTACTGATTGCAGATTGATT
AAAGAAGTGGGGATGATGAGGGTGCTAAGGGGGGTGTCCACCTAGTGGAGATGTCTGGGTGCACCTACTGA
CCCAATGTATCTTTTTCAAAAAAAAAATTGCTACTTTTACAATTTAGAAAATGTAGTGATATGGGTTTATT
ATCATAAAAAATTTGTGCTATTTATGCAAACACCCCATAATATATTGGGTAGATTCTTTTTATGTTATGAC
CTAACATGAATAAGATTTGTTATATAGAAGATTGTCCAATTATACTGTGTCCTTTAGTTCCATGATTTCG
TTTTGGTTACGTTTTTTATTTTTTTATTTTTATTTTTTTAATTTGATTAAGATCAAACCAAATTTAAAAT
TGAGACCAATATATCACATTACATGGTTAGTTTACAATCTTCTAAATTCAAAATAATATATTTTAGGTAT
AATGGTTACTTTACAATAATAATTAATTAATACTGTTAGATTGTATGGAATATCAGTGTTTCATTGATGA
TTCATTGATAATAATCACAGATTACAGCCCTATATATAAGCATATTAAAACACACTAAAGGAATATACAA
GAATAATATAATATTTCCTAAGAATAATATTTCCCAAAAATACTTTAATTATGTCAATACCCTCCCTCAA
ACTCAAGGTTATCACAAACTTGAGTTTGTTAGAAAAACTTACTAAACAAATCAGTAAATCTAGGATATCA
AGAACCCACGAAGAAGAAAAACACAAGGAAATTCTAGGTTGGAGACAGATCCCACAAAGAACAAAACGAA
TAACTTCTGGGATGGAGATAGATCCCATGAAGAACAAAAGGAAGAACTTATGGGATGGAGACATAGATCC
TCGATCTATAACAGAACCTACGAAGAACATACCGAACTTCTGGATCGGAGAAATAACTACTGATTTGAAA
ACATAATATATATATATATATATATATATATATATATAAGTAATTTTATGAACTGAATCATGGGCCTGAAAT
GAACACGGAGGACAAAGAGCTTCGTCTTGAAGCAAACGGAGATCGAAGAACGGGGCGATGGAGAGTAGAA
CAGGAGACTAGATTTCATGGAGATCGAAGAGCGACTGAGAAAGGGGGTTTTACTGTGTTGGACAGAGCTG
AAACGTCATCGAAACGAAGTTGGTTGTGGATCTGGAATGGCGGAAGGCAGAGGGACGGATCTAGAACGAT
AACGTCGGTCGTCGGCGATGGAAGATTGGGAGCAAGGAACGTCGTCAAATCTGAGCGTCGTTCGGATCTT
TGTTGGAGTAGAATAGGAGACTAATGGAGCCAATTGGACAGAGGGACGTGCGACGGCCGCGAATTTGGAT
GCGCAAACCTGGATGGAGGAACGCAAATCTTAACGGTTGTTGCTCGAAGAATCTTTGATGTGCGTGGGCA
TGTGTGATGAAGGAGAAAAGAGGGTGGTGAGGATATTCCGGCTGTTCCACGACGACTTGGAGTTGACGGC
TTGGATCGGAGATCTGAACGGAGGTGGGGCGGAGCGGATTTGAAGGGCGGCGACGGATCTAAGATTGGAG
GGGGCGGCAGCGGCGGCTGATGCGAGAAGCTTCGTGCAGCTGACGTGGGTTGCAAAGGAACGGGCGAGGA
ATGGGCGAGGAATGGCAGATCTGAAGGCAGCGGCAGCGGCGGCTGAAGTTGGGGGCGGCGACGGACGTGG
GTCGCAAGCGACTGGCGGCGGATGGATTTCTAGGGAATTGTGGGGGCGGATGTTTTTTTTTTTTTTTTTA
GTAAAAACAAAGAGTGAAAGTGGGAGATTTACATTTGGGTCTGCTGGGTCTACCTCCGTCTATGGCTAGT
CATTGACGATAGACGGAGGTAGAGATCGAACACGAAAATAGAGATCGACTAAAACCTAAAGCTCTGATAC
CATGTTAGATTGTATGGAATATCGGTGTTTCATTGATGATTCATTGATAATAATCACATATTACAGCCCT
ATATATAAGCATACTAAAACACACTAAAGGAATATACAAGAATAATATAATATTTTCTAAGAATAATATT
TCCCAAAAATACTTTAATTATGTCAATAAATACTTTACTTATAAACTAAAATTAATTCAAAACTATGTTT
AAATTAACTAATTCTCATGCATAAATTACATTTTTTTTAACTTTTGTAATGAGTTGCAGTCTAAACTTAT

AAAACACATTCAATACACAAAGTATTAAAATTTTAGTAGTAAAAGGTTTTGTTACTTGTAACTCAAGTGA
TATAATGTTTGTACTTTAATTTCGAGATTATATGATTGATTCTCTCATGCTTTGGTTAGAATACCTTTTA
GAAAGAAAGAAAAAGTGTGTTGTTATTTTATGCCATGGTCGTGGATTAGTTCTGTGGAATAATGCCAAAT
AAATAGATGATTATATCAAAATTTACTTTTGAATATGGAGTTTTATTAGTTTGCCTAAATATTAATTAAT
GGTTATTATAGATTTTCTTTACCTATATTTTATGTATTTCTATATTTACAATTATTCTTTTAGCCACTAT
TTTAGTATTTTATCTAAATTGTGTTTTGATAAGCCAATTACATACACCCTTTTCCTTTTGGTTCTTTTTC
AAATGTGTTTTAGGATTGTGTGAAAGGTTGAAGCATGTTGATCTTAGTTACTCTACTTTATTAGAGCAAA
TTCCTGATTTCTCTGCAACATCAAACCTTGAAGAATTGTATCTCATCAATTGCATCAAGTTAGGCACGAT
AGATAAGTCTGTTTTTCTCTCTCAATAAGCTTATTGTCCTAAACCTTGATGATTGTTCTAAGCTTAAAAA
ACATCCAAGAGGCTACTTCATGTTAAGTTCTCTTAAAGTTTTGAATCTTTCTTACTGCAAAAAACTTGAA
AAAATTTCAAACTTATCTGCAGCATCAAACCTTCGCTTCTCTACCAATGCACAAATTTTAAAAGTAATTC
ATAAATTTGTTGGATCTTTAGATAAGTTTCAAGGTTTGAACCTTAAACAATGCACTAAATGGTGAAGCTT
CCAATCTATCTCAAGTTAAAGTCTCTTCGATGTTTATTACTTTCTGGATGTTGTAAGATTGAAAGCTTCC
CAGCAATTGCGAAAACATGAAATCTTTATACAAATTGGATTTGAATTTTACTGCCATAAAGGAGTTATCT
TCATCAATCGGCTATCTCACTGAGCTAACTCATTTAAACCTTAACAGTTGCACAAGCCTCATCTCCCTGC
CCAATACAATTTATTTGTTAAGGAATCTTAAGGAACTTCTTCTTAGTGGATGTTCTATATTTGGAATGTT
TCCCCATTAAATGGAGCCCAGTACCAAATGAAAGTTTATGTTCCCATTTCACTTCGTTGAATCTTCAATC
TTGCAACATATCACAAATGCAAATTTTTTAGATATATTATGTGATGTTGCCCCTTTCTTATCTGATCTAC
GCTTGTCCAAAAACAAATTCTCTAGTTTACCCTCATGTCTCCACAAGTTCGTGTTCTTATGGAATCTTGA
ATTAAGGAATTGTAAGTTTCTTCAAGAAATTCCAAACCTTCTCCAAAACATACAAAATTTGGATGCCAGT
GGTTGCAAATTGTTGGCTCGAATTCCAGATAACATTGTGGATATAATATCAATAAAACAGGTTCGATTCT
TTTCATTCATTTTGTTCCTATCTTGCACGTAGACGATTTAATGCCTATGAATTCTTATTCTCTACAGGAC
CTCGCATTGGGTGAGATTTCAAGAGAGTTCTTATTAACGGACATTGGGATTCCATAATGGTTTAGCTATA
AGACTGCATCCAATTTGGTGACTTCTAGCTTTCGTCACTATCCAGACATGGAAAGAACTTTGGCTGCCGG
CGTTAGTTTCAAAGTGAATGGAGATTCATCTTAAATAGGGGCCCGAATTTTATGTAGTATATTCATCTGC
AATAAACTCCATTATTCTTTATCAAGACCAGTTCTTCCATCAAAATCAGAATATATGTGGTTAGTAACTA
CTTCTCTAGCTTGGGGATCCATGGAGGGGAATGATTGGAATGAAATTTTGGTCTGGTTTGAGGTTCAACA
AGGTGTGGTGTCCATGTCACAGAAGAGCTCCATAGGATACAAACGGATGTCAAGTGGCCGAAGGTAAATT
ATGCTGATTTTTATCAACTGGAGAAATTGCAAAGTCTGTAAGTTGATTGTTTCCTTGTTATACTTATTTA
ACTTTTTCCTTTTTTTTTTTTTTTTTTTTTTTGTGGAGTTGAAGGATGATAAAATCTCAAAGGGGGAGATG
TGTATGTTATGATTATGTTTGTTTAATGATATGGGGATATTGAGGATCTTCTTCTCAAACGCCTTTATGA
AGAAATGTCGTGCTGGTCAAATTCCCAAGCAATGTTACATGCAGGAAATTATGATCCAGAATCAATTATT
GATTCCAACGTACAACCTATGATATTTCCATTGCACGTAACATATAATGGTTACACATTAATATGTGGAA
TGGGAGGCATGGGAGACACTGCACTCAGCCAACTATTTATGCAATAAATTTAATTGATCAAATAACAATA
ATCGGTTAGATGATTCTACAAGCTTTTTGTATTTTCGAGGAAGAGGGTCTCGGCCTGGAGGTTTCTTGTC
GCGGCTCCCGCACAGTAAGCGTGGAGATGGTGAAAGAGGAACCAATATCACAACCCACACAATATCCTCC
AAACGCTATTTGATGCTCTTTCATGAAGCTGAGAACTGTAATGATATATTTGACTGGGTTGGTACACAGC
GTTGGATTAAAACTTCTGGCAATTCTAACGGAAGAGGTAATATTCAGTTTCTGATTGGAAGTGTTGATCC
ATCCTTGCTGTGAAGCTCTCAATTTTTAGTAAAATTTTACATTTAAACACTCGTTAATTAATTTTTTTGG
ACTTAGTATTAATTTAAACTCAACAATAGTTAATATCAATTTAAATGTTAAACAACTTTGGTATGTGTAC
GTATCAATTGAACACCCTTCTTATTTAATTTAGATTTTGTTCAAAAATATCATGTGAAACATATTAGTAC
AATTCTAAATTGCCTTGAAGTGAATCGATTTAAACTCTTTAATAAGATTATACTTGATTAGGGTATTCAA
TATCTTCACTTTCTCCCACCGCTAGGTAGTGATAGAATTAACACTAATAATGATGTTAAACCAACTATAA
CTTAGGGTTGTATAAATTTTCAAATGTCTGTTTCAAGGGTTACTTGCATAAATGACAAAAAAAAAACGTA
AAATAAAAGTATATGTAGAACAGGGATAAAATAATTACATATATAGCACAAAAATAGTAAAAGACTAAAA
TATCCACATCCAACCATCATTTTGGGCATTTTTTGCCGAATTTCTCAATTTGAAAACTATCACTGATAGA
ATCTATCATTGATAATTGCTATTGGTGATATATGCTATCACTGATGGCTTCAATTTTAAAAATAATTGTT
TCACACACTTCTTCTCAACTTTTTTAGGTTGAAAGCTATCAGTAATAGCTACAATTAGTGAATATCACTA
ATAGTTGTTACTGGATATTATCACTAATAGCTACTACCAGTGATAACTTGTCATTGTTATGAATATTTTT
TCTGTTTTTCTACAATTGAAAGCTATCATTGATAGCTATTATACATTGTTATCACTAATAGATGTATCAT
TATAATAGTTTTTAATTTGAGAGAATATGTTATCAATTGATATAATTGATATCGGCTATCAACGATAACT
TTTATTAATGATAGCCACTGAAGCGTAGAGTTTTCAATTTGAGAAATATGGTTTCAGTTGTTATAGTTAA
CTGCTATTGCTGATAATTGCTATCATGTACTGATAGCTTACAACTTAAGAAATGTGCATATCAGATGTTA
TATCATAAATATCTTCTATCAACGATGTTGCTATTAATTGATATTCCTGATAGCTATTACAGGTAATTTT
TCTAAATCCCAATCTACGTAAAATGATATCACTGACATCATCTATCAATGATTGCATGCATTAGAACGAT
ATCACTAATAGCAACTATATAAGCGATAACATTAGAAAAATATTTTTTATAAGTGATATCTCCTACATAT
CTCCTACACATCAATTTTAAATTGACGAGGTTTAGTTAAGCTTTCAATTGATATTTTTTATGGCTACTAT
CAATGATATTTTTATACGGTTATCGCTTACAATATCTATCAGTGATAACAACGCAGAAGGATATCACTGA
TAGCATATATAAGTAATATCCCTAATATGCTACTATATATCAATGATATCAATGTCATGCTACTCTAAAG
TGATATTGTGGATGTTTGTTTTTAGCATAAATGGGCAAAGAAAAGAGATTGAATTATCATGCTATCGATA

TAATAGATTTGAATCACTAATAACATACTATTCATACGATAGCTTTTATCATTGGTAGTATAAATGAAAT
TGATATAGTTGCAACTTTTGTTTAAATAAGTTATTGATTTTAGGTTATGCTATCAACATTTATATCATTT
AAAAATTCAAAGTTCAAGTTATCAGGAATAAATTTAAGCAAAACATATTGACATATTAGACATGATGTCA
TTAGTAAAAGTCATCAGTGATAGTATGATATACTGATGTACATACTACAAGTGATAAACATATTGACATC
CTATTAATAATGACTTTTATCACTGTCGCCATTGATAGAAGTCGTCACTGGTAGCATAAGAGAAAAGAAA
TTGATCGGCAAAATTGGCATTCTAATTAAAAAAATTTACCATATGTATATTAAATAATTACCATTTTTGC
AAATACTATTCCCTTATGCGATATATTATATTTATTTTATAATTCGTGCTATATGGTTCAATTTTCCTTT
GTTAATATTTGAAAACAACAAAAGGTATCCAAAGTATCTTCTATTTAAAGTAATTTTTTAATATTTTAAA
TAATTATAATACATTTTAGGTAAAAATATCATTTTGGTCCGACTTTTTTTAATTTAGTTAATCTTAAAGT
TATTTTAACCTACATTTTAAACTAGAAGTATTTTGAAAAAAAAAAAAAAAAAAACTTACATCTTCCCTCCA
AATCTATTTTTCTAATTTAAAATGTTGTATAATTAATTAAAATAATAAAAAAGAAATAATTGTGTGGCCT
TTAAAACCTAATCTTTAAAACTTGAGAATTAGAAAATGTTGTCTTCAGATATAGCAAAACGAACAAAAGT
ATTTATAAAATATAGCAAAATTTCAGAATTTATCAATTTGCACTCCATTGATAGATTTTAAAATTTTGCA
ATATTTTATAAACATTTTTAATAATTTCCTCATTTAAAATCATTTTTCAATTAAAAAAAGTTACATTATA
ACCCTATTCTTCAAAATTCAAGGATTAAAAAGATAAACTATACTTTCGTCATGGTCATGGTTATTAATGT
ACAATAGATGGATATGGGGTATATAGATTTAACTCTATTTACAATAATAATAAGAAGAAGAAAAAGAAGA
AGGGAAATTGCATCAAATGACAAAAAAAGTTAGAAAAAAACAACTCATAGCACGTCTTTTTTGCATATTA
CAAATATGAAAAAGTTAATTAAATATATTAACATCTATCAGAAGGCTATCAAAGAGTTATAAGAAAGCTA
TCAAAAGACTATCCTCTTTTAAATTTACTACTTTTGCAATTTAGAAAATGCAGTGACATGGACTCTATTA
TCATAAAAAAAAATTTCTATTTTTTAAAACGTCCCTAATAATAATATTAATATAAATTAATAATAGGAGT
CTTTTCAAAAATATAAAAAAGCGGCAAAGTATTTAGACTCTAAATGATTTTTTAAAATTTTTTTGCTACA
CGATCGTTTATTTTTTTTATACGATCGTTTAGATTTGGCTAAATGATTTTTAAAATTCTTTAGGTACACC
ATCGTTTAGATTTAGTTATCCAAATTCAAACGACTTAAAAAAAGTAGAGGAAAAGAAGAAAGATAATGAA
AAGAAATCACAGCAAAAAAAAAAAGAAAGAAGAAAGACAATTGAAAGATTAAACGACATAAAAAATAATC
AGAAAAGAAGAAAGACAAAGAAAATATTAAACGATGTAAAAAAGAATCAAAAAAGAAGAAAAGACATTGA
AATAAACGCAAAATCAAAAAAGAAAAAGACCATGGAAAGAAATTGCAAAAAAAAAAAGAGAAGAAGAAAG
ATGATGGAAAGATTTAACGACGTAAAGAAAGTATTGAAAAACAAGAAAGATTATGGAAAGAAATTGCAGG
AAAAAAAAAGGAGAAGAAGAAAGACGGTAAAAAAAATCGCAGAAGGAAGAATATGAAAGAAATCGCAAGA
GGAAGAAGATGATTTTATGGCGAAGAAGAGTAAAAGATGGAAGGGCAAACCTGAATATTTAAAAAATGGC
TAACTTTATGGATTTTCCTGTAAATAGTTTGATATTTTTTTAATAATACATAAATAAATAAATAAAATAA
AATGAAATTGGCGGGTCGGGTCGGGGTTCCACGAACCGGATTGGAAAAGTCTGAGCTCCGAAAACGTAAT
TTTGAGATTTATCTTATAGCCGTTTGAAAGAGGTTGGATTCAATAGCCCGCTCGCCATTTCAAATCTTTG
AATTCAATCTCAAACCCTACGCGACCGTTGCTCTCTCTCTTTCTCTCTCTCTCTCTACACTCAGCTCT
GTCATTTCTTCTTCTTCTCCATCAAAATTCTTGGAATTTCTCTCGAAATTCACATCCTCCATCTCTCTTT
CAAGCCATGGAAGGTACTTTCTCTCTCTCTGGGTCGGGATTTTGTGGGGGAAATGAGTGCATCCAGATGG
TTTTGTTGTTAATTGGGTTTATGTTGTTTCAATTTCAGATGTAATGGAAGTCGTCTCACAATTATGCTCC
CCGGCTGTTCCTTCTTGTGATTCAAGGCCTTTGCCTTCCATTTCCGGTTCAGGTTCGTTAGTTCTTTCTT
CTTCTTTTGTTCCTAATGATTCTTTCTTGGTTTCGATGTGCATAGAATGTTTATTTTCTTGGATCTGTTC
CGACCCAGATGATATGGGTCTGTTTTTGCTCTTTTTTATGGTTGTAATGGGAAAGAGCAATCAACTTAGA
GCCCTAGAATTTTCTGTTGTTCACTTGTTTTTGCTCTCCTTTGTATGTTTCTTTCTGTTATTTTTCTACT
TCCACTCCTTTCAGATTTGTCTTGATTTCGGTTTGGCAACTGCGAATTTTGTGGATTTACTTTCTTCATT
ACCAATTCTTATGTTTAGGGTTAACTTTTCCGTTAACTTGTTTGCTCTTTTCTCTGGTTCTATTTCACTA
AATTTCTGATTGGTCCAGATATTGATTTGTGCGAATCTTTTGAATGTGCTGATAAGTTGGAAAACGAATC
GTCCAGTGAAACCGAATTAACATCCTCACATGGACAGCATACTCTTCCAATTCTGCAAAAAGTCATTGAC
TTGGGCAACAAAATTAAGGTATGAAATACTTGAGGTCTGGTTTTTTCTTCAATTGGCTGGCTTATAAATT
CTTGATATGGTGTAAATTTGCAATTACCTTTCATCATTCATGCTCTATATTATTGAGTATATACGACAAT
GTTAAATTTTATTTTCTTCTTTGGCAATTAATACATTGTTTATTTCTGGTTTCAGAATTTGAAGAATGAA
CATATGCTCTTAACTGAACGATTCAAAGTAGAAACTGATGCTTTTCCAGGCCCTGAAGTTGTAGAAACTC
TTCACCTTCTAGGTATTCTTCTGCTAACCAACCATTTTCCTTCATTTTTTTTAAATAACTACTTCTTAGC
AAACTAATCAGTGGCTATTCCTTTTTCCTTTTAGGTACAGAGAATGAACGTTTGAAGAAGAAATACCTTG
AAGAGTCCACTGAGAGAAAAAGACTTTACAATGAAGTGATTGAACTGAAAGGGAATATTAGAGTTTTCTG
CCGATGTAGACCATTGAATCAAAGTGAAATAGAAAATGGATCTACCTCTGTGATTGAATTTGATTCATCT
CAGGAAAACGAGATTCAAGTTCTTTCTTCTGATTCTTCCAAAAAACTGTTTAAATTCGATCATGTGTTCA
AGACTGAGGACAGCCAAGGTGATTATGAGAACTAGTTCCTAATTTAAGTTACAGTGAATAGTTTACTAAT
TCATGTCTTGATGATGGGTTTCCGAATTTGCAGGAACTGTTTTCAGTCAAGCTAAACCTGTTGTAGCGTC
AGTGATGGATGGCTATAATGTCTGCATATTTGCTTATGGACAAACTGGAACAGGGAAAACATTTACAATG
GAAGGAACACCTGAAAACAGAGGAGTCAACTACCGGACTCTGAAAGAGCTGTTTAAGATTTCAGAAGATA
GAGATGGTGTTGTGAAATATGATTTGTACGTCAGTATGTTGGAGGTTTACAATGAAAAGATAAGGGATCT
CTTGGCAGATAACTCCAACCCAAATCTAAAGAAGTGAGTTTTTTCCTCTGGTGACTTCTAATGGATGGTT
CCATATGTCTATATGACTTGCATATTTGTAGACATTGTAATGTCTGTAATGTTGTAGGGTTGAATGCTTT

TAGTTCAAGTACTCTAAAATATTTGTTGCTACATGATAATTTATTGGTAGCTGTCCTCCACTAGATCGAC
TACTGAACACTTGAAACACTTGAATGATTAGTAATGTTCAAAATATTGGAAAGAATTGTTAATATGAGGT
TTGTTTTAAGGTTGGAGATTAAGCAAGCAGCCGAAGGAACACAGGAAGTCCCTGGACTGTTTGAAGCTCA
AGTGTACGGAACTGAAGAAGTGTGGGAACTACTTAAATCTGGAAGCCGGGCAAGATCTGTTGGGTCCACT
AGTGCTAATGAGCAGAGCAGCCGGTCTCATTGGTAATATTCCTGTCTGATCATGTTCTGAATCATCGTGA
AGAACATGTTAAGAGATATCCTCTACTAACATCTTTTACAACAAAATTGATACACTTTTCATATGCCAAC
AACTTCTTAAACAACATATGTTAAGTAAGTTTTGTTCTTACAAGATGATTTGACAGATAGAAATACTTAT
GCACGCCAATAACCATATGTATGCAGTTTGTTGCGAGTGACTGTCAAGGGAGAGAATCTTATAAATGGAC
AGAGGACAAAGAGTCATCTTTGGCTGGTGGACTTGGCTGGTAGCGAGCGCGTGGGGAGGATTGACGTTGA
TGGTGAAAGATTAAAGGAATCTCAATTCATTAATAAGTCACTTTCCGCTCTTGGTGATGTCATCTCTGCC
TTGGCTTCTAAAACAGCTCACATTCCTTACAGGCATTTCTCCATCCAACTATCCCGTTTGCCTCGGAAAT
TTTCTTAGTATATTGGACTGAGTTTTGTTTATTTTTCTTGTAACAGAAACTCAAAGCTCACTCATTTGCT
GCAAAGCTCTCTAGGTATGGGGGCTTTACCTTAATACTTTGCACCCTGATATCTTTACTTTGAAGTCTCA
GTCTGAGCTTCAATTTAGAGGTCTCTGTTATTTGTTTTATTTTTTATTACCGTGGACGCTAATTGAAATT
CTGTGCAGGAGGAGATTGCAAAACGCTAATGTTTGTACAGATCAGTCCAAGTGCAGCCGATGTTGGAGAG
ACACTCTGCTCACTAAATTTTGCAAGCCGTGTTAGGGGAATCGAGAATGCCCCTGCTCGCAAACAGACAG
ATCTCACAGACCTGTTCAAGTTCAAGCAAATGGTATATGAACACACCGGAAATTTCAGATTGTGCTTTTT
ATTTAAAGATTCTTCTTTTTGTGTTTTGGCCTTGTTTTAAACAATTGTTGTTGAAATTTATAGGCAGAGA
AGTCCAAGCATGACGAGAAGGAAATGAAGAAGTTACAAGATAATGTGCAATATTTGCAGTTAAGACTTGC
GGCTAAGGAACATACTTGCAAGAATCTTCAAGAAAAGGTAAATTTGAAGTTCTTTATTATTGTTGTGCCT
TTTTACATCATTGCACAATTTGCTTACATGATCTTCATTACGAAGTGAACACAAAATTATTGTTTGTGTA
CTAACAGGTTCGAGATCTCGAGAGCCAGCTAGCGGAAGAGAGAAAGGCCAGACTAAAACAAGAAAATAGA
GCTCTTGCTACTGTTGCTGGTGCCGCCTCTCAGCCTTCAGCAATGCAACCTCTTCCAAAGCTGGCAGGTC
TTAAAACCATTCCAGAGAAGAAACCACCATTGGGTCCTTCAAAGCTAAGGCTTCCCCTAAGAAAGATAAC
AAATTTCGTGCCACCAACTTCCCCCATACCATCCAAAAAAAGGCGTGTCTCTTCATTCATAAACACTGCT
CCTCCAACCGAAGGCAAAGAAAATGTCCCCAAAATGAACACAACTGCAGCAGCCAACACAAGAAACCTTC
GTCTTGGTAGACGAAGTTCGCTAGCTGTTAGGCCAACTTCAACAATGACTACAACAACCACAACAACTAC
AACACAGGTTTTTCAACCCAAGAGACGTGTCTCAATTGCTACACTTCGTCCAGAGTTGCATTCTCACATG
ACAACCCCATTCCAGACCTCAGCCTCAAAATTCAATAATGGAAATGCTGCATTGGGGGCACAATTATTTG
CAGCAAGGAAAGCAAGATATTCAAAGCTATTCTCTCCATTACCAGAGTTCCAAACAACAACAGTAGAGGC
AACACCTATTGCTGCCATGAGGAGCAGTAGCAAGTTCATGGGAAGTCCTCCAACCCAAGGTGGTGGTTCA
AGAAATGGTAAAGTTATAGCATTACAAAGAAAACCAATTGTGTGGAGTCCTCTCAAGTTAAGAGGACTGA
AAACTTTCAGGAGGCCATCTTTAATACCATCCCGACCTTCCTCGACGACCGAGTTTCAATGACAACATCA
TCTCTTCAACTTTCTGTCCAACGATCGTTTCTTCTCTAACATCTCATTCCCATCAAACAAAGCCATCAGT
GTGGAGTCCTCTCAAGTTGTGTGCTCGAAACTTGAAGGATGTCATTCTTTGATATCACATCGATCGATTT
TCCCACCAAGTTTTCATGACGACCCTTTTTGTACAACAGTGTTTCTTCTCTAACTTCTTCGCTCTGTCTA
CAAATAGTTCTATGTGTATTTGTTTTTTTTTTTTTTTTTATTCATATTATATTTCATTTCTTGGATGTTA
CAGCTAAATTGTATGCTTCTGGCTTACCAAATGAAGTCTCATAAACAAGTTAGTCTTACTTTGATTTTGG
ATTCCTTGAGATAAGATTGTTAGTTTCTTTGTATCTTTTGGTCAATATTGTGAAAAATTGAAAAACTCCT
GAAATAATTCAAATAAATCCATACAATTAGGGAAAGAGTCCTACAAATTACTTCAATTAACCAATTTATT
AATTGCACATACTTAGTTTGAAATACAAACCAATGATATTCTTATGGAGTAAACACTTGGTTATGTACCC
AAAAAACTTGGGGTATAGGTTTTATTTTCCCCTTTTCTTTTATATTCTTATGGGGTAGAACATGAAATAT
TATGTATACATTTTAATAAACAGCACCAGTCGATTTGATGATTATAATATTAGTATGTTGATTTTATTAG
TAGATATCATTTAAATTCAATATTTGGTCTCCCATCCTTACATTTACATTTGTTGTACTTTTAACCACGA
AAAAAAATAAAAACTAAACATAGATACAATTCAAATTTAAAGATTAAATCGAAAAATTCTACCCTTACAA
CTATCATTATAATAATAAATCGTTAAAGTTATCTATTAACTTTCTTCATCTTCAATAAAGATATTTTATC
ATTCTCTCTAAAATTGAAATGTTTATCATAAATTCATAATCGAAAGAAAAATAGAAGAAAAGAAATTTCT
CACAAAGAAAAAATGCATTATTAGACTTTTTATAGACAACTTAAAATAAAAGGAGCAAACCCTAGTTTTA
AGATTTTGTCATTCTAGCTTTTGGTTTTTTTACTTCAATGATAATGGAGAAGAGACCCACACACAGAGAG
AACTAAGAACCAAATTCCAAAACGTAGCTTAGTGGGCCGAATTGGGAATGCATGGAAGGCCCATCAAATA
TTATTTTAACAAAATAAATAAATAAAAGTAGCCGTTGGCCCTTTGGCTTTCATATTGAATAGAGAAATCG
ACCGTTGGAACAACAAAGATACGTTACCAATTATCCAAAAAGCCTTCGCCTTTGACTTCTCCACTTGAGA
GACAAAAAGGAAATTTATTAAGCAACAAGAATTTCAAATAACTCTCTCACAGTTCTGTATAGAGAGAGAA
AATTTTGTACCCTCAAATCCACCCCTCAATTTTTCATGGGTATTTAGAGAGAGAGAGAGAGAGAGAGGTT
TCTTTTTTTGTTTTTGCTTGAAGACCCACTTCGGAGATTCTTCAATTCAATCAAAATGGTTGCTAAAACC
CCACCAAAACAAAAGAAAATGGTAGCATCAGCAGCAGCAGCAGCAGCAGCAGCCACTCTCAATCCAGTTC
AAGTGAGAGAAACACTAAAGAAGGTAATGGGAAGTTCATGGTTTGGTTTCCCATTTGGGGAATTTTTTGT
AAAAAAATTACATTTAGTTCTTTTGTTATGAGTGTGATTTGCATATGAGCTCTTTTTGTTGTGATTTAAG
TTTTTGGAGAAAATTGAAGATCTTTCTTCACTTTCAATTGATTGAAAATTTTACTTTGTTAGGTCTCTTA
ATTTGTTTCTTGAAAGGCATGATGACAACAAAGTTTCTATCTTCCATTTCGTTTTATGTTTCATTACTCT

AATCTGGGTTCTTCATTTTCAGTACAAATTCTCAGCTCAAAGGTTGAAAATTTTAAATTCTGCTTCACTT
TCTATGGAAATGGACACTCTGATCATTGGTTTCTGCAGCTCAATGTTCAGCTTCAATATTTGAAGAATCC
AAAAATTGAGATTGTAAACTCAACTCAATTCAATAAACACACGATATTTGAGCAAATGTTTAGCTTCTTG
TTCATACATCTTTGGACTAACTTCTCTCTCTCTCTCTCTCTCTCTCTCTCTCTCTCTCTCTCTCTCTTCC
TAATAGAAGCCATATGCAAACAAAAAAACATAAAAAATCATTCTTATTCATTTTCTCAGGAAACAAACAG
TCCCCAATGACTTCAGCAAGAACAATATATACTTTCTTTCTATGTAGCCATAAATGAGTGGAGAAACTAC
TCCTATTAAAAATTCAAGATTGAGCAAACACTTTAGCTTCTTCTTGTTCTTACATCTTTTGACTAATTTG
TCTTGTTTTCAATGTGTTTTGAAGGTGGAAATGTGCATGAGTAGGCTACAAGAACTTCAATTCACAGTAA
CAGGAGGAAACAAAGTAATTTCTGGAGTGAGCCTCAGCCCACGTAGTACAAGAACATATCTTAAGACAAG
CCTTAGATGTAAACAAGAATCTATAAGGTAACAAACTCTTTTTCTATTAATTTTTGTTGGTTTTGTGTAA
AGTTGAGATTTTTTCTCTTGTTTTATTTAAAAACTGAAATGGGTGATTCTCTGTGTTTCAACTTTGAAGG
ATTAAAAATGGTGGAGGAAAGAAATCTCCAGTGGGGAAGTTTCCATCAAATGCAGGAGGAGGAGGTCTCT
TTCTTTACTTCTTAAAGTTCTTACCTTTTTTTTCCTTTTTGGTCAGTCGCATGTTTCAATTTTTTTTCTT
TTTGAATAAAGTTGCATGTCACAATTAATAAAGAATAGACTTTTTTAATATCACCTAAAAAGATTTTTAA
GCCCTTCATTGGTCTGAATAACATGATATTTTCAATTAGAAAAAAGAGTTAATGATGAAGTTCTTTAGGA
CATAATGATGAAGTATTTTAAAAGCTTACTAGTTGGTGTATATTGAAATCATAGCGAGTAAAGGTAATAA
TTTTTTTAAGAGCAATATTTAGGTGCTATCATAAACTATGTCATCTTTTTATAAAATCCTCATACCATAT
AAACTAGTTGGGTTGCAAAATGATGGGTGTGACCTATACAAGTATTTTCTTTTTCAACATTTAATAGCCA
TTGATTTTGGTATTTTTGGTATTTTAATTCTTTCGTTCCATATATTGTACAGGGGAGTGGAAGAGGATGT
CTTTACCAGCAATGCTTGTAGGTGAAACCATTGGGGAAATACTAACTGCAAGTAAGTTTGCAAGAGAGAT
GGTTGAAGCAGTTACCAATAATAACACCAACAAAAATACATCCATGGATGACCCGAAAACGCCATTGACA
CGACAAAGAAACCGCAAACCAAACCTAGAAGATTCAGAATTAAGAGCAAGAAGGAAAAGAGAGAAGCAAG
AAAAATTTCAATCAAGACGAATTGAATCATCGCATTCTCCATCACTTCAACGGGTTCGGTCCCGGATCAA
CTTCAAGGTTGTTTCACCTCCAACAAAAAGAGATGTCGTTGATAAAGAGAATGCAGGGTATCTGGCAAAT
CGAGTGTCGCCTAGGAACAGGCCGTGGACGAAAAAGACCATACTTTTTCCAAACCCTTTATTCCTATCAT
CAGATAACTCCTCACAACAGCCAAAGTTTTGTAGAACAAAGTCTCCAGTGATCGCAAAAACAAGACAACA
AACTCCACATAAGTTCTTGATCAAGTCTCCAGTGTCAGCTTCAAAGTTTCAGGTGAAGATTAGCCCACCA
GTGGTGATCAAGCGATCGCCTACAAGACCAGTAGTGATCACGAGATCGCCTACGAGACCGGTAGTGATCA
CGAGATCGCCAACGAGACCAGTAGTGATCACGAGATCGCCTACGAGACCGATGATAAGCTCAAGTAAGCT
GTCTCCAAAGAGATCCACTGCTTCCAAGCTGCGTCCGTCGTTTTCACCATCAAGGCTGGTAGCTTCCAAG
TTGAGACAACCATTTTCACCTGCAAAACTTACAGCTTCCAAGTTGAGACAATCATTCTCACCTTCAAGAT
TGGCAGCTAGGTTGGTGTCTCCCCTGAAAAGCAGAAAAAGTGTACAAAAGTATGATGGGAACATTAATGT
GAGTGGATTGAAACAGAGACCAATCAACAACATACAGATGAGAATTTGAAAGGATGGGTGGAGGAACAGT
TTCTCACAATTATTCTTTGTTTGATATCTTCTCAATGTCTATAGTACTGGTTGATTCTTGTGTGTATGTG
ATTGGTTTCATCACATTATGCATCAGTTTGTCAATAACAGATGAGACTTGATTAAACTATGTAGTAAAAG
TAAAAATCTTTATACTTTTTTCAATGTATTTATTATTCTCTTTGATTGATGAAAGGAAAGTTTGGTCAAT
TAAAAACAGATGACCCACTTACAAAATGTGCAAATCAAACAACAGTTACGTGTTTGATGAATTACAAATG
AAACAGACTTGGAAAGAGAAAACCTTTGCCATTTTTATATCATCAAACCCAGAATATAGAATATTGTACA
CTAATAATACACTTTCTATTCACCACTGGATACAAATTTGAAAAACGTGTATCCTTTAAAAACCTCCCCA
ACCCAACAAAATCAATAAAAAAAAAAAAGAAAAGAAAAAGGAACAAAAACAGATTAGCATATATCGTCAA
TGTCTTCCTCTGTTTTCCCCCCATTTGAGAATCTGATCATGTAAGTCATCAAGGGATCTCTTCACTTCAT
GGACACTCCACAAGGACTGCAGAGGGGAAATGACAAATGAGAAGATAAACTCAGCTGGAGTCGTTTGAAG
ATGCCAATCTTTCAGGAGATGAAGTTTTCCCTGTTGTCCCTCCCAGCACCCGACACATCATCCCTGTCAA
CAGTTCTTCACAATCATGGAGATTGCCATAATAGAGTTTATACTTGCTGTTTAAAGGTAAAAAGAATTTC
AGACATCTACCGGAATGGAGATGCAAACCGTGTCCGCTCTCTTGGCCTGCCCATGGCTTTATCCTTTGGA
GATGAAGTTTTACCTCCCACGCCAGCTTGACGCATCATCTCTGTGAACAATTAAGAAAAATGTAAAATAG
AATACACTATGGTGTGCGTCCGACAACGAACTTCTGGAAACACATTGGGTCATTCAATAGTTATATTCGT
ATTAAAGAGCATTAAATAATAATATCATCTACCTCGTTTTCTCTCGAGCTCTTGATCAAGCTCTTCTTTC
CACTTCCTCTGTATTTCAGAATAATTCTTACTGCTAGATGATAACACACAGCGTCAATAACCCAAACATC
AAAAATACAACACATGTAAACGATGACTGTTAAAAAACATCCAAAAGTCAAACCTTGGGCTACCAGCTTC
TGACTGACCATGAAGTCCTTTCCAATCATTGAGTTGTGACCTTTGATCATTTGCATTACCCCTGCTACCA
CTCCCTGGGCTACCAGTGCCAAAAGCATCATGTGCTGGGGATGGGTTACCAGGACTTCGACTTGGCGACC
GGCTTTTCGGGGGCAATTTCAAGTATTTAGGAGTACTTTCATCTTGTTTGCTCTCAATAAAAACTGGAGA
GCAGGATGCATTCAAACTATTGTAGAACTCCTCATAAAGCGGTGACTGCAGTTTCTTCAATTCAAGAGCC
TAAGCCATTGAAATCAAACTTAACAATTTAGAGCAAACAAGTTACTTTAATAAAACACCAAGAGTAAGGG
AGAACTAGAAAGAAAAAATAAAGGCTAAACACTAACTGAAAATCCAAACCTTTTCATCCAAGAAAGCTTT
AATTTTTGATTCAGTAAGCTCATCCTCATCTTCAGAAAGTGATCGCCCACTAGGAAATGAGAAGTTTTGT
TCCTCGTCGAAAGCTCCAGAACAGCTTCCAGGTGCAAGAGCTTCTTCATGTAAACTGCTTCCTTGGTCTA
CTACTGGACTTCTATCAAATTGGCAATCCTCATCATCAGAGGGCTCACAAATTGGATTATAACTCTACAG
CCAAAAGAATACGAAGAAAAATAAATAACTAAAATAAAATAAAGAGAGTTAACTTCCAGAACTTTGTGAA

CGAACTAAAGTGGCATACAGACATTGGGAAAAATAAATAAATAAAAAGGAGGGGAACAATGAACGAACTG
TTTTACTCACCTTCATATTCTCATGTATAATAGAAGAGCCAATTTTTACTTCATCTAACATAAAATCATC
CTTGTCATCAATCTGACACATTTCATCATCACTGTTTCTTCCCCACATATCACTTTCTAGTTTGTTTGTA
GAGTATACCTTTGAGCAGCTCAAACTATCCAAATTGCAAATATCACTTGATCCAGGATGTGTTGGAGTTT
TACTGTCAAAAGGAAGGAAAAGTCAAAAACCTTAATAATTGTAAGCAGGTAAATGCAAAGTCTAGTCACT
GATTTTGTGGATTCACCTCATTTCAAGTTCTGAAGTACATTGTGGTGAATAGATTTTCAAGGGCTCCTGA
TGATGGAAAATCTACCGTTATAAACATGAAAACAAATAATCATGCATATTTGTTCTGGTTATTGTCTCTG
GATTATAAAGTATTAAATACTATAGGAAAGATTTTTCAACCGTGCATGCATCACGTGACAGAAGTTGAGA
TTGGGCCTCCTCCCCAGTAACAAAAGGATGCTGCATGCAACAAAATAGAAAGGTTAGATGATAATAAAAA
CAATCGCTCCTTTGGCACTATTTCTGTCAGTGTATAACACGTCATTGCTAAAATAAAAGAGGAGAGGCAC
TATTAGAGACTCCGCAGCTCACTATTTGTTTTTATGAAGTAAAGAAAAAAAAAATCAATCAAGTTAAGAAT
TACCTTCAAGAGTTCAGAGGCAGTTGGTCTTAAGTTTGGTTCCCTGCAATTTGACCAACATTGATTTGAC
TTTCCATGAGAAATAGTGGCAAAGAGAAGCAAAAGAAGAACACTTTAAATGAATCTCGAAGTTCAAACAT
TGTAAAGTTATGATTAGCTTCCTGAACATTGGGGTTAAAACTGCGAAGAAATGAAACATACTTCTGCAAA
CATTTCAACAGAAAATCTTTAGCTTCAACCGAAAGCTGCTCAGGGATTGGTGGATGAGACTTCGTTGTCC
CTATATGAAAAAGAGCAGCAACCTAAAAGAAATGAATTGTTAGTTCACTCTAAAAAAACAATTTAATATA
TCCAGCACTAAAGCAACTTCAAGTCATAAGATACCTCTTGATACTGTTGGCTCCAAGGAGGCTTTCCTGT
AGCCATCTCAATAAAGGTGCATCCAACACTCCATATGTCAGCAGAGCTAAAAATGATTATCACATACTTA
TGATCATCCATCTCACCAACTTAATATTTTGACAAAGGCAAAGCTACATAAGATCACCTAATTGTTTATT
AATTATTGGGTTTTCTGAAAGAAGATTAACCTATCAAAAGCAAAATTTGACACGTGCATCTTAATCAAAA
TCCTCTGTGATCAAAGCACATCCACTTATACATAATTCCGTCCTAATTGAGCTCAACCTGATTTATCAAC
TAAATATATATTTAATTCGAATTTAAGTTTGTCATCAGGATTTGAAATCAACATTTTACAGTTGATAATT
GGAATGGTATCCAAAAATTCAGACAAAGATAGTAGATTCTAAATAGAGATTGAAATTAGTCAGTTCTTAC
AAACTATGACCGGTCTGCAGAATTACTTCAGGAGCCATCCAATATGGAGTACCCTTCATAGACTTTGCTC
CTGAAATTGTAGCCTACAACAAAGAGATTTTGTTCAGAAGGAAGACATCAGTCAAACTAAATTAAAGTAA
GGGATCATTGACTTTACCAGCTCGACAACCTGTTTTGATGCCCCAAAATCAGCAAGCTTAATGCATCCCT
TGTTGTCTACAAGGATATTTGCCCCCTGCAGTAGAATACAAGAAACTTGACCTATTTATAACTAAGACGG
GAATAAGAAAAACATCTGCAGATTACAAAGATCATAAAAACCGACCTTAATGTCCCTGTGCATGATACCA
TTCTTGTGTAAATATTCCAATCCCAATAATAACTGTTTTGTATATGTTCTTAAAACCTACATAAAGTTGG
TGTTAAGGATTAGACACTCAGTGCATCTGACATTAACATGTTGAAAGTTGTAAATAAAATAACTAACTTA
CTGCTTCAGGGAAGGCTCCAAATTTCCCCAGAAGTGATGCTATCGATCCACCAGGGACAAATTCCAATAA
TATATTTAAAGAATCGTCCTCTCTGACTGTGCCCAAGTATCTCTAACAAACAACACAATGCATTCAGTGG
GAAAATTAATGGGCTTCTTTATGTGAATGAGAAGCAGAAGGCCATACTCACAACAATATTTGGATGAGAA
AGATCCTTCAGAAGTTTCACTTCTTCCTCAAGCTCCTGAACTTGGGCCTGGTTGCAACAACAAAAAAACA
CTCAAATTTGACAATAAAGAATATATATTTATATATGAAATACACACCAATATGTTCAAATTTTCTTATT
GTAATGAAGGAAAACAAAAAACCAATTACAAAAAATTATGATGAACGAGATTAGAAAACAAAAACAACGA
AGCCTTGACTAAGAAACCATTTCTAGCATTTGCTTTAAATATTAAACAAACAAAAATTGAAGATTATAAC
TTTCATCAAATATCTAAGTCAAATCAATTAAAATACAAAATTAACAAAAACTCGAATTAACTAAGAGAAA
TATGTCCAGAAAATTTTACTGGCGAATACATCAGAAATGTGGTTGGTGGAAAATGGAACCTCTAGACTAC
AGTCAGCCATCTATGATCCGAGAGTATCAATTAAGTAAACACAATCTATTTATAGAGAACAAGTAATCAA
TTTAACCAATTAGAAAAATGGTATTAAATAGCTTCAAAGACGAGAATCAATATTCCAAAATAAAGAAAAA
GGGTCATATAATTAGAATCAGGTCCACAAGTGGAAACAAAAATCCAAACCTGCGCTTTCTCCTTCGAAGC
ACCATTAGCAGCAATCAAAACCTGAAAGAGAAGAATCCAAAAAAAAACGATGAAAAGAAGATCCAACAAA
ACAGCAGACGGAACTCCAAATTGGAAAAGTAGAATTGGAAAACCTGTTTGACAGCAAGAAGCTCTCCAGA
GCCAAGATTCATGCCCATATAAACGCGACCAAAGGCGCCACAGCCAATCAATTCGCCTTTGCGCCATCGA
ATGGGAGGGTCGGTGTCTTTAGGAATGGGAGGGAGAGAGGAAGAGGAAGGGGAGTGTCTAGAGAAAACTC
TGGATTTGCGGATGCTGGAGTTGATCTTATCAACTAGGGCTCCAACTCCAATTGCAGGGGAATGGGAATC
ATCGTTGTCGAGAGGAGGACGAAAGACCAATGATCGGCGAACAGAAGCGAAGATATCTTGCATTTGAGGA
ATCAGTGAAGAAGAAGAAGAAGAAGAAGAAGAAGAAGAAGAAGAAGAAGAGTGAATTGTAAGAGAAAATGGAG
AGTTTTGATTAATGGCGGTCGGCAAGAGAAAATTAGAATCTAGAGAGAGAGAGAGAGAGAGAGAGAGAGA
GGAGGGAAAAGAAAGCGGAGAGTAATTTTCAAAAAAAAGAAAAATAATTAATTTAAAAATATGTGAAAAT
AAAAATAGAAAATAAAAAATAAAAAAAGAAGCCGACAGTGCAATTTTTTTAATAGTGAAGGAATCACTCT
CTAGTGGGGGGAGAGAAGTGTTGAAATAATAATAATAATTTATATTCTCCAGATCCTATGGTATAATAAA
TAGGTCAAACTAGGACGACTATAATAATTGACTTTTACTACTTAATCTTAATGTTCGAGTGTTTAGATTT
GAAGCATGTTTGATGTAATGGTTCCATAAGACTATATAATAATAACCTAACACTATTTCAAACTCTTATT
TGTCATCGTGTTTACTATTTTCTTTTTTAATTTACTTCAAACATATACTATTACCCTTTGAACTAAAATA
ATTAATACCTTAAATCTAAACTATTGCATGTAACCCAATCAAACTATAATAAGTTAGAATTATAATAGTG
AAGCCCTAAATACCATCTTGGTATGTGGGGTGTTGAGTACGTTCTTATTGATTAAATGATATGTGGATTA
GGATACAAACAATTTTAGTATAGTTATAATATCGTGTGATTGGAATGCAAAGTATTTTATTAGTTTAAGT
AGAAAATAATAATCGTTGTAATAAAAAGGAAAATGAATATAAGTAGAAATTTGTGACCATTGAACAAATC

CAAGCTAAGTGTGTTTAGCTTTAAAGTTTCAATGAATGGGCTATCAATTTTTAACTATTGTTGAGTAAGT
AAAAAAACACTTTGTATATATGTAGTAGCTATCGATATTTATAAACCTTTCTTAACTATGTTTTCTTATT
CTCGTGAATTTAAGTATCACGATGAAAGAAGAAAATAATAATAATAATAATATATACAATTCCACACCTT
TTGATGGTGTTTCATACAAGAAGATATAAAAGAATGAGAATATAAAATTTTCTTGTGGCCACTGCTATAA
CTTTGTTTTTTAGAGTGAATGTTGATTGATTGGTTGTTTCTAATTTGTAGTGAAAAAATGGTATATCATA
TTTTCAAAATATTATAAATACAACAAAATTTAATGGTGAATAAACTATATCACTCTATTATTAATTGATC
TACATTTTAACGGAGACTCTTCTAAAGTTAATTAGATCAAAATTTTGTAATACCCATAACTTTTATACTT
TAATATAATTTGCTTTTATTTTAGGCCAACTTTGCTATATTCACAAACTGTTGGACTACTTAAGTATTAT
AAAAAGGCAAGAAAACATAACTTCCAAGAAAATAAAAGAGAAAAAAAAAAGTCAGTTAGTCTATAGTTTA
CATCAATTCCCAAAATTTCTCCATACCACTGTACATTATTCTCATTTCATCAACAAATTGTTTTTTTTTT
TTTTTTATCATCTAAAGTTCAAACCCTGTGTGTGTTTAGTTCAAACCCTGTGTGTGTTTTTTATATATGT
GTAAGAAAAAAACTAAGAGAGAGAGAATTATTAGAACAGTAAGAAGAAGGCAAAATTTGATTCATGAGCT
AGCTTAGGGAGCATGATCATCCATGAGACGGTGTCGTCTAGGGTCAGTGATCTTGATCTTCCAATGAGCA
TCAGCAAAAGTGGAGTTATTTCTTGCAATTCTTCTCCATTTTCTTATTCTTGAATCCATCTTCTTTGTAA
GACCACAAAAATGTTGTAATTCAGTGGGCATGGATTCATAAATTTGCCACCATTTTGCATGGGCTGAATC
ACTTGCAAATATTTGGTGATCTTCCATGTCCCAGTTGCAATCATAATCTTTGTAACACATCCATGGCTTT
AACCCCAGATAATGTATTGCATAAGGATCTGTACCTTACACAAAGAAAGAAAAAACAAACAAACAGTATT
ATTATTCAAAGTCTCACGAAGAAATTCAATTCAAACCTTTTGTGTATCTGTGTTTTAAACTTTGTCTCGT
CTTTGATAACTTATTTGGTCTTCAGATAATGATTTGATTTTTGTTTTTTTTTTATGATCATGTGTTTGAT
AGTCACTTTAGGTTTTGATTATGGTTTTTTTAAAATTATTGGGGTTTTTTTTTATAGAATTTAAGGTTTG
AATACTTTGGATAAATTCAAAAAATTAAAACAAACCTTTTAGACCCTTTCTAATTTTTATGAGAATTTTA
GATTTACAAAATAGATAATAAAGTAAATAAAAAAATAGGTCAATAAGAATGACTTAGAGTTTATAAATTT
AATTTTCATAAACCAATTACTTTGTTTTTTATTTTCTATTTAAAAAAGTAAGCTTAGTTTTCTTGACTTA
TTATATCAAACTCTTAAATATTTTTTTAAAAATAATTGATCTAAAATTTGAAATCTAATAAAAGAAACTG
AAACATCTATGATTGTCTACCAATTAATGTGAAGTGCATAAATTATCTTTTCTTAAGTAAAAGTGAAAAA
ATTGAACAAATATTAATTAGAAACTAATTAAACAAAGACTAAACTCAATTAACTTTTAACCCTCGTACCA
AATAGTAAACAAAACTGATTCCAAAACCTAAAACCTAAAAATTATGAACCAAATCTAAGATATATAAACC
CACAAAGTCAAGATTAAGAAATATTCGTACCTGAATCATTTTCACTGTTTTCCTTCAGAAAGATCTTGAG
ATAGTTGACTCTAGAAGGAAGTCTATGCCACCAAGTAAAAACTTCATTCAAAAACCCTTGATCCCCACCA
TTATAAGACTTCAATTCAAAGCTTTTCTCCATCAATTCCTCAAACAAACAAGCAGATGGCTCAACAATCA
TAACCCCTGAATTGAACCTCATTTTATTATTAGCAGCAGCAGAGAGCTGAGGCAGAGCAAAGAACTGATC
GATGTTTTTAAGAACCAATAGATCAGCATCAATGAACACGATTTTGTCGTACATCGTCAGCTGCCATATT
CGTAGCTTGCTGTAGTTCCATTCGTTGTATGAGCCTTTCTCGGAAAACGGGCTGCGAATGCGATCAATTC
TCATGACATACCAACCTGCGTCCTTGAGTGCTTGAATAGAGTTGGGGGAAATGGAGTTGTCGGCCAAGAG
GATGAGGTCTTTGGATGTGTTGGTTTGGAGAAGGCTTTGAGCTAGAGCAATGGCTCCACAAACATATGCC
TCTGAGGAGTGGAGGATTGTCACATAGGCTTGTTTTGGTTTGTGGTAATGGGTTGTTGTCGAATTGGTTT
TTGGTGGTTTTTGTAACATGAAGTGTCTCCATACTTCTCTCCCTGGAAAAAACAAAAACAATGTATAGCA
TATTATTATGATTCGATTCGAATCGAAAAGTTAGAGATATTTTAACTACACGGAAATCAATACGTTGAAA
TTCCCTTTTAGAGTCTTCTACAGTTTCATTAGTGACACTATATATTCTAAATTCTACTAATATAATATTT
CAGAATGTTTGAATATTAGTCAAATGAGCTACACACTATAGCCTTACCCATGAATGAAGCACACCTTGGA
AAGAATATAAATATATAACACTTAAATTTAAATAAAATCTTCTCCAGACAATAAATATAAAAGTAAATTT
CAAGTTCTTTTACCTAAATTTCTTTGTAGTTCTATCTCATTTTGAACGTCCTTTAATTTAAATAAAAATT
TGTCAAGACTACAAATATAAAACATAAACTTTAGATCAAAATTTTCAAACACTTCAACCATGAGATAGGG
GAAGTTATAATGTTTTCATGTAAGTGTGTGTTGATAACATGATAGATATATATGATCTAAACAAAATCGA
GTCTTGTTCAATATAATTAGGGTTTACCCAAATTATAATAATCTCTATCCCCTCTCTTAATCTTAATGTA
CATTATTAATCGAGATAATGATAGAAACTTTTTAAAATATTAGTTTCGAATTCGTTAAGCACTCAGACAT
ATTTTTTCTTAAGTTGGGATCGGAGATTTAAATTCTCGTATATTATACTAAACCTAATATATTAAATTTC
AAAGAACGAAATTATATATTACATATAAGTTTAGAAGAAGGAGATTCATATTTTGATTTAATGTAATAAG
AATCAGATAGAGCAATAGAAATGAAAAAATGGCTAAAACAGAAAGTAATTTTGTTTGCAAAAATATGTGT
GTGGTTCCCATGAACAATTAATAACTAACCAAACTTAGCAAAAGAAAAAAACAATAAAGCAATAATTGAC
TTCCCAAAATCTCCAGATTTTCAGAAAAGCGTTTCTGTTTACTACAAATTAAACGTCACATACTAAATTA
AACAATTTATGAGCCAAATAATACAATTAAAAATAACTTTAGATCATAGGAAAAGATTAATATAATTATA
AAAAAGAAATTATATAGAAACTAGATAGTGTATAGTTAACTATATGAAATTCATTGTTCATTATCGATTA
AGAATTTTAGAAAAATGGTTCATGATTCTTTTAATTTCTAAATAATATAAGCATATAAAATGGCAGTGAC
TTGAATTAAAAACCTAGAAGAATTAGGAAAAACAATGCAAGAAAAAAAGTAAATAAAATTAAAATTGAAA
GAAAATGGGTTTTTATGGAAAATGAAGAAAAGAAAAAACGAACCTGTCCGAGCATAAACAGGCGCAAGCT
GGCATGATCCAAAAGGCAATAAAATTTTTTGTTTCAATCTCTTAATCTCCGGTTTATAAACCCAAACATT
CTCCACCGTTCGATCATCCTTAATCACAAGATCATCACACCTGAAAATCTCTCTCATCGCTCCACATTCT
CCCAAAAACACAACGTACACCGTCCGGTATATTTCCTCCTCCACCCACCCACTCGCCACCGCCACTCTTG
CCGCAGCAAGATTCGCCTGAAGCCGAAATACATCTCTACTCGTTAAGATTCCTCTCTTACACGGTATCCT

CAAAACGACAATGTTTAAATCAGAGTAATCTTCTGTTTTTGGCATCGGAATGTTCGGACATTTCGGCGAT
GATTTGTATTTCTCATCTTCATCAATCCACTCCGGAAACAATCTCTCCCATTCGAATTCCTTTTCGATTC
GATCAAAATCTACTGTTACCGTCTCGATTCTACTTCTTCGGTTTTCGATTAAGCCGAGGAGATGTAATGT
ATCGTGAATTTCGTCTTTGTCGTCGGTGCTTATGTTGATTAAACCGATTTTGATGGTTTCTTGAAATTCG
CTGTCGATGACGGTGGGGATTTCGCGTAGGCTGTTGGTGATGGTGGTGGAGTTGGAGTTGGAGTTGGAGG
AGAGGAGAGTGAAGGAGATGAGGAATAAAAAACAAAGGAGGAGGATAAAGAAGAGGAAGATGAAGAAAAG
AATTAAGGAGGAGGATCTTGATCGTCTTAACTTGTTGATGAATTTGAAGAGATTTTGCGATGATGACGAT
GATGATGATGATGACGACGATGATGATGATGAAGACGGAGAATGAGATTGAGGAAGGGAATTGAGAAGGG
GTTGCTTTCTAGTGAAGAAGGCCATGGAAAACGGTGAGAAATTAGAGAGAGGAGAGAAAAAGGAAGGGGG
GGTTTTAGAAAAAGAATGGGCGTTGGGGATTTTGCATGGCGGGACGGGCCGTTGAAGTTGTTATTTGGAT
GAACCGAATTTCCGTCCTTTCTTTTTATTTATATATTATTTTTTTTATTTATATTTAATTTATATATAT
ATATATATATATATATTAAATTATAGAGATATATTTTGGGATTTTTGTTGCCATTTAAATATTTACTTTT
TGTACATTTCAGGTTACACTTATCTTTGTATATCTCCTCAAATTGTTTGATCATAATTTGTCACGTGCCA
AATTCTTTTGTTATTTTTATGCATGTCGTTTTATCAGTCATAGATTTTTATCTCTTTTCTCCTAATTGAT
TTGTCATATTGGGTGATTGACTTTAAGGGATAATTTGAAGGGTTAAAATAATGTCATCATGATCACTGAA
AATCTAAAGAGTGTTGAAATGCATCAAGAATTGAGGATGAACTAATAAAAAATGAGTATAGAAATGATAA
AAAAGAGGATTGAGAGTTGAAGATTGTGTGTATCACACCCCCTCTCAAACTACCTACTCTCGACGTAGGA
GGTGGTGTGAAGCTAGCAAATCTTGTCTCTAGATACTTGACGACATCTGTGGACCTGATCCTAACTAAAC
AGCTAAGTTTAATTTATATGCAACTTTAGAATCTAATTAGTGAGTCGTTACTACTCACAACAATACTTTA
TAGATTCAACAAGTCATTAATATAAGTATATATGATATGATGCTAAGATAAACATTGCAACGTTACAAAC
CAACTCTATATGAAATCCAATAACAAATGCAACTTGACAAGTATGTCGCTAAGACATGTTCAAGCATTTT
CACTTGCATGCAGATATATTTACAATACACAAACTCAACTCGTAACAACTAACCACTAGTTGCAAGTGTG
TTAAAAAAACAAGACTGGTACAACAGACTAGCGACAACAAGTCAAGCTCGGGAGTCATGATCTCTTCCAA
GAAAGTGGAATTTATCGAAATCTTTAGCCTTCAAACTATATTATCAATCTTATTTTTAAGTATATATATA
TATATATATATATGAGTGGATCTAGATAACGATCTAGATAGTATTGTTTGAATGTCTTTGATAATATTTA
ACGAATAGATATCATATTTTATTATATATCAACTAATAGAAAAAATATCAACCAATTGCTAATTCAAGTA
AAACACCATTGAAAAAACTATAATCAAAACTTTAGAGGACAGTTAAAATCAATTTCAAACTTAGATAACC
ATACATTGTTAATTACTCAAAATTTGTTAATATAGTATGTTAGAACAAAACTCATTACCTAAAAACAATG
GATATGTATAATTTAGTTAGTGATTCTAAGAGTATCGTTCACTAATATATAATCAAACTAATAATTTTTA
TAATTACAGTGAATTCATATTTATTTATAAAACTTTAAAAACATTATTGCAAAATTATCCACAAACTAAC
CCAAGAAAATTGAAAAATTAAAAATGCTGTGTTTATTTTTCTAAAAGGTTGAAAATATGTATATACCAAA
ACAAAACAAGAAAAGATATACGGAATACATTTTAAGTATAAAAATTTAAAACTATTTTATTAATTATGGT
AAATTCTATTAAATTTTTCATTTAAGTTTATTCGTTAAATTTTATAGATAGTTATATATATATATATATA
TAAAGAGTTTTTGTATCATTTATTTGAAAGGAATATAAACCATTATCTTTGACTATTGTGCTATATAATT
GCATCATTGAATGTAGTTGAATATATAGAAGATTATCCCAACCCAATAATCATTTCAAAAAAAGTACAAC
TTCTGCTTTCATGTTAATTATTATTGTTTTTTTATGAGAAATTAAAATCTATAAACGATGTATTATTTTA
CTGAAATCGTATATTTTAAATAATTATACGGACAATTGCAATGGAAAATACTTTTAACTATATTTCAAAA
AAAAAAAAATTACTACTAACAATATCATCTTTTATAGATAGACTCTTATTAGTAATATAGTCTATAGTTT
AGATTCAAATTAAATGGTAATTTTATAGATTGATCAACATGTTCATACGTTAATCTAGAATAACTCAAAT
CACCAATGCAAACTTATTATTAACTTCAAAATGTATAATTTTTACTTACCATACAATTATATATGCATGT
AAATTTATTTTAAAACGAGGACCATTAATATGGTTAATAATGTTCTTTTAGTATATTACATTTAGGTTTA
TTTTTTAATACATCAAAATAGATACAAAATATAGAAAATTAGAACTATCAATAATAGATATTGATAGACT
TCTATCGTTGACAATGATAGATACATAAATTTATTAATATTTATTATTGATAAATTATAAATTTACTATA
TTTGTTAATATTTTTCAACAAGTTCCCTAATATTTATGTATTATAAAAATAAAATTGAGAAAACGTACCC
AGTTGTGATTTGTTTATATAATAAAATAATCTATAGAAAAGTAATAAATTAAAAGTACGTACTTTTCATT
TTATATTTCAATTGAAATATTGAGCTATATATCAAATAATTTAAATAGTTTGTATAAATATATTTAATTA
CATACAACTTTATAATACTATTTTGACATGTCCATCGAGCTAGTTCAATTTAGTCTCTAACTTTTAGTTT
TAATATTCAATTTTAGTCGCCCACCCTCCACAATTCTTAATATTTCAAGTAACACTCCGTATATATGTCC
CAAAATTTATGGTAATTAAAAGTACTAGTAGAGGATAAAAAGTTTTTCTTTTTTTTTTTTTGTCGAAGAA
AATCAACAATAATATCTAAATTTAAGATTTATTGAAAATACAAGAACTAAAATTAAATATTTGAAAGTAT
AACAACTAACATTAAATAGCTCTTAAAGTATAAAAAGAAAAATAATACTTTAACCATACTTTTATTACTA
ATGAATAAAAGAGGAAAAACGAAAAATGATTAGCAAAATAGTAGTTTATCCATATTTTTATTACCGATGA
ATAGAAAGAAGAAAAACGATGAATGATGAGCATATATATGCGTGAAAGAAAAACTAAGGGCATCCAAAGC
TTTTGAGATGCACCTTGTATATATGGTCATCTTAAGATGCTCGGACATTTCATGATGTCGAAATATATAT
CATATGGTAAACCAAACGTTATGATTTCATAATAATGACGTTGGATTCTGGTCAAACAAACCCCCTCCTA
AAGTGATTTTCAACTATCCATCTTTCAAATTATTCTTTTTTCAAAAGAAGTTTTTAGATTAATCTGATTT
TTATCACGTTTATTTATTAGTAATTCTTTTGGTTCATGTGCTGGAGAATTTTTGCCTAAAAACACTTTTT
GATTAAGAGGTGAAATCAGTTTAGTTTATCGGTTTTGGAAACAATTAATCGATTGGTTTGTTATTATCGG
TTTTGTTTCTCAATTTTTCTTTACTTTCTCTTCTTCCATTTGTTTCCTTTTTCTAATCAAAATAATCTCT
ACCTTATTGATTTGGCCTTCTTTGATTAAAGAAGAATGATGGAAAGAGAAAGACTTCATCTTTATTTTCA

TACAAATCCATGAAATCAATGAAATCCGACAAACACCAAAAGAGTCCGACCCATATCTGATATCTTACGA
ACGTGTGTTGCTTTTTCTCTCAAGCCCCATTTCTTGTGCTACACTCCAAATTCCAATGAAACCCATCAAA
CCCCAAAATTTTCCCATTCCACCCAATTCATATTCATCTCAAAGTTTCCCCCTTTTCCCCCCATTTTCCT
GGGCAAACAAACAAAATTTATCTGTCCAACACCACTTCTACAATCAACCTCACTCACCCGTTTCCATGGA
ACCACCTTTAAATCCTTTCTTTCCATTTCCATTTGATCTGAAAACAGGATTCTCCATCCTTTTGTGCAAA
CCCCTTGTCAAGATTTGATCGGATAGCTCAGAATCCATCAATCTGTAAGCAAGTTCTTAGTTCTTCTTAC
CCAACTTCAGAATCGAGATGGGTCTTTTCTGATTGTGATAATTAATATGTGAAGAGGGAGAAAAAAAGAA
AAAAAAATGAAGCAATGTGATGATGATTTTGTTGCTGTTTTTGTGTCTTCTGGATTTGAACTTTGAACCG
CACTTTCGGTTGTATAATGATTTTCCCCACATTTTGATTTCGAATTTGGGGTCCCCAAAAGGTAAGAGAT
GATGATAATCCTGCAAATTAATACCAACGAAAAGGAAACTTTATAGCTTTATGTGATTTTCTTGGAATTT
TTTGCCTGCCAACAAGTCATTATTGTCGTTGTTTGCTCAGCCTCTGCCCATAGCTTAAAATCTCCATTGA
GTGATCTGTGCTTTTCACTTTAGTATTCTGGTGGAATGGAATCTCTATGTTCTGTTTCTTTCTCTCTCTG
TGCTGGATATACCTCAGTGAGTATATGATACTTTTTTCCCCTATGAGGTTTCTTAGATGAATGCTCCAAA
TGCCTAATACTTTTCCATTGTGATTTGAAAGCTTGAATAATTGTGCCCAAATGGACCCAAATGACATTAA
ATATTTGTCCTTGGTGACTATACATGAAAGGAAAGAATAAATATAGATTAAAGCTACTTTATTCATATTC
CTCTCTAATATTCCCAAGAAATGAACCAAACCAGAATCGTGAGAAATTGCTGTCTACAGGCCAGAGTTAT
GAATAAGCTTGTTTTGAAACCAACTTTCTCCTTCTCTGCTTATGAAGAACTACCTAGCTTTTCTGCCTGA
CAAGTCAAGTATGCTCAAGCAATAAAGATTGAGAAGGTTATTCAAGAAGCTCTAAACTCTAAGCCCCTTT
TACTGGTGCATTTCAGGCATTTTATGTCTTCTTTTCTTCCTATATTTCCCCAAAATTGCCTGCCTCAGGG
TCTGTGAGCCACTTTCATATACCGTTATCGTCTGATTTTCGTTAAGATCATCCCGTTTTTGTTCTTTGAA
GCACATGACCAGTTGCCTCCGGAATTTGATATCGTTCAAATAATTGTAGGTAAGATTTAGCTTCTTTTTT
CCATTTGAGTTTGAGATAATTTCTGATGGAATGAGTTGATATCTCATAAGCTATTACTTAAACTTTAGAG
CTCAGCGATCTAAAAAGATGAGATTTCTCATCAGATATTGTTAGAGTCGTTTAAATTTCTAGTGACATTA
ACTGATCTGTGGGGTCTGCTGCACGTATCAGATTTAGAAACCCAATTAAACCTTTTTCTCCTTCTATTTT
CTAAGAGACCTTTCCCCTTTTCTTCCTTTTTAAGTTATTGACAATGAGAATCAATTGGTGGCACCAAAGC
ATCTAATTTGTTTACCTGCGAGACATCTTGCTTTAACCATGTTTTCCTTTGTCCTTTTTTCTTACCTCTA
TGATTCAAATTTCTGAATAAGTTTTAAGGAATATGTGCAGCTTTAGAAGTTGGCTTTTGAATCTGTTGGG
GGAATAGTCTTTATACCTTGCAATAATGTTGGTTCTAGTATCAGATTAAGAAGGGACCCACTGGTATTAT
TAGATTGCTTCTTTACCATTCACTATGGATTATGTATGAATTAAGCTATTAAAGTAAATAAGATTTGTGT
ATGTGCAGGTCTAGATAAAGATGAGGGAAATGGCAAATAATAGTATGGATTTTCAAGTAGAAACAGATTC
ACTTCTTGGACTGAGTGAATCTGGGAAACTTATTTCGAGTTCTCCACAAGTTTTGTCAATTCTTTCCTCT
GTTTTCGAGAGGTCGATTCAGAAGAATGAAAAGCTATTGAAAAGACTCAAAAAGAAAGACAACGTTACAA
TTTTCCACAGTTCCCGAGCTCCTACCATGGGCATTGGACAATATATCGATCGCATCTTGAAGTACACATG
TTGTGGAACTGCTTGCTTAATTGTTGCCTACATATACATTGAAAGGTATCTTCAAAAAATGGATGTTTAC
CTTACTTCTCTGAATGTCCACCGCCTTCTGATCACCAGCATCATGGTTGCAGCAAAGTTTACCGATGCCG
GGTAAGTTCTACCGTTGCTTAGTTAGCTTGGCTAGGTTGTAACTCAGTTGAGATGAAGAGATTTTGAGCT
TGGTTAATATTATGCAGTTATAGTTTCTTTATAGTTCACATCACACTCACTTTACTTCAGTTGGCTTCCC
CTTCATTCGTTGAACTGAGATCATAGGAGGAGGAATCCTTAAGTTCAAGTTTGGTTCATTTTTCAAACTT
GTCCGCAATCATTTCAAGGTTTATGATTATTTATTTTATTACCAAGCCTTTACTGTCACAAAAATGTTAA
AATTTATTGATGAAAATTTTTATTGTACTCGCGTTCGATCCCTATAGATGCTCACATCACATGCATAAAT
TATCCTTCTACTAAAGTTCAATGATGCTAAGGACTTAATTATAGTCAAGGTATGAAAGATTAAAATGAAA
ATTTTAGATGATTTTTATACAAAATGTAAGAGTGATTCAAACACTGCTTAGAAAGATATCAATAGCTAGT
GAGTTGTTTGCTCGATGCTGCCACACCGATGATATTGTGCATCTCATCTGTCATATTCTGATATCTCTGA
AAACTTCCTTCTAATTTCTTGGATGTTATAACAATACTTTTTATGTATACAATTACACAACTTGATGTAT
TATGATTCTATCAGGTGCTATAACAATACTTTTTATGCCAAAGTAGGAGGAGTGAGTACAAAAGAAATGA
ACAGCTTGGAGATAGAATTTCTGTTCAATTTGGACTTCAGACTTCATGTCACCGCTGACGTTTTCAGTAC
TCATTGTTTGCAGCTCCAAAAGGAAGTTCTTGGGGGAGAGAATCAAGTCGAACGTCGACCCGGTAACAAA
GCTCGAACTAAATGTTTGCCTCAAATTACAGGGTATACTTGCAGTGCCATTTAAACACTCCAAGGAAAAT
CTCTCTTTCTTGAATTTCATGGTTAGTTTAGTGTTTCAGTCTCTGGAGTTGTATGAAATATTTATATTAT
AAAGCTTCAATTCTTAGACTAAATCCAGAAAGTAAAAAGATTGTAAAAGGAAGAGGATGGGATTGTAGAG
ATTCAATGAAAAATGTTTCCAATGCATTCGAAATCTTTTATAATTCAATCATGATTGGTAGATGGATTCT
CCAAAGTTTTACTTTTTTTATATATTTTTTAACCGTCTCCGTCAAGTTAAATTACAACTTAAAAAAAAAA
GGTTGAAACTTTAACCTTCTACGATTTAGTAGAACTTACCAAATTATAGGAACCGAATTTGTAATGGAAT
GAATATTTGGTCGGGGTGAACATGATAGACTAAAAAATCAAACTGTGGATCGAAAAAAGAGGAATAATTT
TAGAAAGTTTCAAATCAAGAATTCTTAACAAGTATTTCAACAAATATTTCAAGTGTTAAACCGATCCGAC
AGAAATCGACCAACTGATTATGGTAAAACCAACTATGCTCAATTCAGTGACTATTTTGTCAAAAAACCAA
CTCCAACCAACCAATAATCACCTCATATTTGTAAAGAATAATTAGTAGTTTAAACTTTATATAGAATCAC
TATTGTTTACTCTTGGAAAATTACATAACCAGCTGCTTTTGACTTTTTTTAAATAAAAAAAATGTTGTCT
AACGTCTCTTTTACACTTGTTTTTTCTTATATATTTTGTTTAAAACACCACCTCATACAAGATTTAAAAC
AATTATTATATCAATCTATATTTAAATATTAAATATCAGTATAGAAATAACTAAAAATATGTCTATTTAT

AACCAAGTCTCACATATTATGATATTACTTCAAAAGCTACAATATTAGGGTTGATTCTGAACTTTTTAAA
AGCATAATTGCCGGCCACTACAACTTCTAAACTATATCCCAACTAAACGAAATGACCACTCAATAAAAAA
ATTCCACATTGGAAATACTAATCTGATTTCAATTTTTTAAAAAAGTACTAATATGACACTAGTTTACAAA
CACTACCATATATATATATATATATATAAAGAAAAAAGAAAAGAAGTCTATGGGGATTTGTGCCAAAAGT
CAGGTTTTAAATGTTAACTTCTAAAATTATAAGAAAAAAACCAATTATTTACATGTTAGAAGATAGAAAG
TGAAGTTCAGAGTATCTACTAAAAGAAATACATTAGTTAGTATGTCTATTGCGCAAACACGCTTGTTTTC
TTACACTGATTACATTGTTCTTTCTTTTCTCCTTTTTAACAAATATTCATTAAATCCGAAAACTAAAAAC
ACAAACCGTCCCCGTTGTTAAGCATAGACAACCAATCAAGAAAGAAGAAAACCCGAAAATACAACCCATC
CTCCAAACTGTAGCGAGGCAAAACTCACAAGATAAATCGCGATAGCAAGAACGCGAGCGACAAAATTAGT
TACTAAAGCATTTGCATAAAGATCCAACACTCACACCCTCTCCCCACTAAAAAAAGTATGTAATAAAAAA
AAAACAAATTAGATCAAGGAACACATAATGGTTTCAAGCAAAAATTGAAGAAACAATTGAATTATTTTGA
TAGCAAAAACTTTTCATTGCCCCTTCTGATTGATTGTACAACCATAAACCCTAGGCATTGGGGAGAACCC
TATAGCAGAATAGGGTTTGACTTAAAATTATTATACATAAAAAGCAGAAAACTAAAACTACAAACATACT
CTTTTGATTGAAAAAATTGCAACTTGTCGTCTATTTATATATCTAATAACCCAATGAAGTCAGACAAATA
TGACAAATGGGCTGACCATTGAACAGCTGAAAGTATAATAAAGATTTAGGATACCAACCATATCTAAAGC
AAGGCGATATTTTCTTCTTCCCTTCTCCTCATTTAGCTGGAAGAATTCTTCCTTGCAAGGTAGACGCGGT
AAGCTGCCAATCCAACGATTGTTACAGCAGCTCCGACTGAAATAGCCACATTACGAAATCAGTTGGAGAG
ACTAAGGGACTTACGAGTTGTGAAATACTTCTTTAGTTGCCAATTCGTTTTGATACCATAGATTAAAATG
AGAGAGAAAGGAGGTTTTTTTTTTTTTTTAACATCTGAAATGTCATATCAATATTAAAAATAATAAATAA
ATAAATGTACTCTGACAGTTACTGACAAAGTAACGCAAGAGACCTTTTAAAGCTATTTCCCAAAACTCAA
TGAGAAGTGCACAGTTTAACACGTTCGAGAACAAAGGAACTGTTCCTGGAAAGTTATGAACCGACAAAGT
AATTTTTCAGGGAAAAAAACCCTCCTAGAAGAATTAGGAGCTCAAAAGTGAACAAAACATCTGACATCTC
ATTATCTATCAAAGGACGAGGGAAAACTATGACGAATTACCCTGAGAAACCAAGAAATAAGCCATATAGT
CCAATTCTCATAAAGGAACAATGATGCAAGCGTGGATAGAGTACACTAGACGACTATTAGTAATCCTCCT
TAGTACTAGTACGGACGTTATGCCCAACAGAGCATTTCATAAACCGTAGACAAAAGGTAAACCTAAAGAA
ATAGCTTTCTAAGGATTTTCTGAATGTGCCTTTCGGCTCACAGAATGAGGTAACACGACCATACTTGTTC
GCAATGGCTAAAAAAATATCACTATTAGCATAATATGCCCTCGTTAACAATGCCTGAACCCACAAGGTCT
AATAGTCAAGAAGACCCTCATAACCTTCTCTAGATCCTTTTTAAGGGAAACAAATATCTTCCACCTGAAG
GATAAAAAAATTGGAATGCCAACACATTGGATGAGAGACAGCCAACTTCTATTAGAGAAGAAGGTCCTCT
CCGAGACAGTCATTTCTTAATCTTCTCCAAGACAAGGTTTCAGAAAGAATCACTCTCCACAATCCCATCT
CGCACTTCTACTCATTAACCACCTCATTAGCGATTAGAATGGAATTTAAGGTTTTCCTTTCAAACAAAAG
TTTAATAAATTGGCACTATGAGTCGAAGATGTCATTCTTGAGAAAATCATTGAATCGTTTACGATATCCT
TTTTGAGGATATCCCAAGAGAATAAAGTTTTTCACTCTCATAAAGAAAAGAAAAAGGAAAAGTTAAGACA
GTTGTCCAAGACATCTGGAAGCGACATCATGGCCAGAAGGTTGCTATCTATTGAGCCAAGGATTAATAAG
ATTATGACTATACAGCAAAGTTAACTCATGGACATCGAATCAAGAGGGAGGTTGTCTAAGCATTTTTCCA
AAATGTTTTCTATTGATGGTATTAACCACAAAAACATCCTTTATAAACCGTGATTAAATAACTTGTAAAA
TTTAAACACGTGATTTTGGAAGAACAATTTTAGTTCTGAAAAACAAGTAATAGGTGGGATGACAGTCTCA
TTTATGCAACCAGCCAATGAAGCAAACTGCCATACACAAAAGTACATGGTAATGTTCATTATCAAGTAGG
TTTTTCATGTTGCTATGAATGGAGGGGAAAACTACAGAAATCAACATACCTGAAACAAACATAAGAGAGC
GGTTTATGAGCTTGTGATAATGCTTGCGGCTTCTGCCCGCCTCAGTTTCAGGGATGCTTAAATGAGGGTG
CTCTGCAGCTGATGTGATTCTTCGGAATACGTTATTAAAATCACCCAATTTCGTGCTGATGGGTATTGGA
GGTTCTATACCCATATCCTGGCTCACCTACCAATTTGCAACGAGACATAATGATAAGGATGGTGATAGTA
AAAGATTCAGGATTTCACTAAAATACAACAATATATTGGATGATGAACAGAGACAATAATTAGAAAAGTG
GACCCGACAAAATAAAGATTACAAATAAAAACCATAAACAGAATAAATACTTGCTAAAGAATTTGTATTT
TATAATTTTATTATCTTATTTACTACGAAAAAAACTTTCTTTCAAACGAAGGAAACTATACGAGTACATT
TTTTGTTCAAATACAGAATTTGAGCAACTGATTCTAATAATTGATTGATAATTGTTTCTATAGGAGATGA
AGTGAATATGAGCACCAAACAATTGTAACACTGATAGGCTATCTTGTAATTATCAACTCATTATTATTCT
TTCGGACTGAGACCTTTATTGAAATTGGGTGGGGGGCTGGTTGTTGAAAGCTTAGTTTCTTATTAACAGA
AAAGAATTGTAACCTAAACATAACATTGCTTTTTCTCATCAAAATAGTAATGTCAGAGAGGTTTATGGTA
AACCCGTAATCTATAACATTCTGATTCAGTGGATGTGAAGATATAAATACATACATATATATGTATTATA
TATGAATTCAGTTCGACTATCAGAACATTAAAAGTGTCCTGTGTGAATGATTGAATATATATGGTACATA
GAGAAAAATAATTACCCTTGTAGAATCTTGTATGGCCAAAGGAAATGAATCGAGGTCATCTTTGGCAGCA
ACAATTAGACAAGGCACTTCATAGCCAGTATCCTCACCATGACTTGCAACTTCAACCAGCAAATCGGTTG
CTTTCTTCCAGGAGGACTCATCGGAGCTGGAAAGAAAACAAAGTCGGTGCTCTACGATAGGAATTCAGTT
CTTCATTGGTATAAACATACAAGGAATTGCTTATGCTAGCAATACATAATTCTTCTTTTTATATTCATAC
AAAAAGGTATGCTATAAAAAGATTGAAGAGAATTCATGACAAAAATATTTCAAGGTATGTTTCATTTGTA
AACCACATTGAGCACCACCATATCCATCATTATCTCAAACATCAATCATCGTATTGAAAGGATTTTAGGG
ATTGCAGTGCAAGAAGAAGCTTGCCACCCTAATAACCAATTAGTCCAAAGAATATTAGATAACCGATCAT
TAAGCAGAAAAAGGAAAAAGAACTTGTAGTCCATGCAACTAGAGGACCAGTCAGTAAAAGGAAGAAAAAA
GTAAAATTTCGAATTAATTTGTTACTGAATCCTTGGGGCAGATCATTCTCAGATTAATGATTTAAGCAAA

CCAAAAAAACCCTACTTAGGATGAAGTACAAATCTAGAGCATCGGAGGTTAAGGCCCTTAGTAAGATACAT
TAGTTGGAGAGGGACTATAGTAGCTTTCTGAAATCCCCCTTAAGAGGTTGGTGAGTGTCCCATTCCTTTC
AAATTTTCATATTAATGAAAAGTACATGCTTAAAAAATAAAATTAAAGTGAAAGAAGAGAAAAAAAGGAG
AAAAAAAGACGAGTGTGGCACAAGCTTATATTTGTCACCAGGCCTTTCTCTTTTTCCAAATACAAATGCC
ATTTATCATCCAGAAATAAAAAATATTATCATCCTAATATAGTGTAACATTGTTAATAAGCAATGGCCGT
TTGCCCGTTTCCATTAGAAAAGAAGTTACCTGTCATGCACAAATAGTGCAATGTCACACGCAGCTAAAGA
CTCTTTGCTAGACAACAGTTTTTTCACTCCATCTTCAGGTATCTCCCTCAAAATAAGGGTTTTCTTTGTT
CCCTGTAAACCAAAAAAAAAAAACTTTACAGACAGAAACTACCAAATAAGAGCAGACATCATAGTTTCTT
AACAATAAAACTAAAGTTGAGTATTTGACTTTATATCCCAAAACGTGCTACCACTTTACTGATTTTTGAG
TATATACATGTAGAGAAAAATAATCAAAAGTGCAACAACTTCAATGGTCAATGAAAATCAAATAGAAGGT
GAATTTTGACAAAGGTGTGAACCATAAATATCCTCAAAAAGCCTCCATTTCTAAGACAACTTTTAGTTAT
TCAATTGATCGGTGAAATGATCTAAACGCAAATGTGTATTATGGTAGTTGGTAGCAACACCATATTTCAC
CAACTTTGAACACGTGCCATCTGAAAAATCATCATAAACTCTCTCCACCAGGAATAATTGCTTCCAATTC
ACAAATTATGGACACGTGATATTATATTAAGACCTTGAAATTTGAAATCTGGATCCATGCTTCACCAATT
AATCAAAACAAGGACATAGCAGAACTTACTTCAGGTTGGTCGACAACGTTTACGGCATAGCGTTCTTCAG
TGGTAGGAGTATAGGTCACAGAAAACGGCCTGTGTATCAAATTTTCTTAACACAAGACGTGCTCTAAAAC
AAGTACTAAAAATTAATGCCTCAAAAAACTAGATAAGTAGAGAATGTTGATAATAAACCTTGCAAGAAAT
GCATCTAATAAAGAAGATTTCCCTGCCTTCTTTGGCCCAAACACAAAGCACTGAAGAACATTTCGGTCTA
ATTGTTGCTTCTTGCGATCCAACCGCCTTTTCCTCGTCACACGAACAGCAGATGCAGGATCACCAGAGTA
ACCAATGTATATCAGATTTTCAATGGTGTAAACTGGGTTTAGGAGTGTCATGAGGGACCACTGCAAAAAT
TCAAGAAGAATACACAACTATAAATAATACACTACTTTTAGAAGAAAAAACTCTCAGAAACACATGGTGG
AAGAGAGGGAGAGGAGGGTTAAAATCAGAAGAATACAGGAAATATGAAATACATCATCTTATCAGGAAAA
CTGGTAGAAAACTAACCAGTGATAAAAAGTCATCAATTGACAATCCTCCCATTGCATTTCGCTCCGCAGA
ATCTTTGTATGGAGCTTCATTCCAAGGACTAGCATTTCAAAAAAAAAATGTCGCATAAGACATCAAAGAA
GCTCATTAACTCGCCAAACAATAAATCATGTTTTTCGAAGCTTTTCTGTAACTGTTCTGTAGGCACTATT
TCGCTTGATTGGCCCCTTTTCTGAAGAGAGTGGTCCCTTTTTATGGAATTATTTTTTGTAGGCCCTTGTA
TTCTTTCATTTTCTTTCTCAATGAAAGTGGTTTTATAAAATGATAAAAACAACAACAACAATAATAATAA
TAATAAATCATGTTGAAAAACATAATGGTTGCCAAATCAGTTTAATAACAAATGCTGGAGAAATTTCAAA
ACCTATTTTGTAAAGAAATTTCAAAACCTATTTTGTAAAGATAAATGTAGGATCTAAACAACCACAAGTA
AAATACGAGAAATCTTCACAACAGAATTGGGTTATAACTAGGCAAAGGGTCAATGTTATATCGCAGTTGC
TTTAATATATTTGAAACATCATGAGATCTGGAGTATTAATTTGACACATGGAAGAATGAAAACATGGAGA
AAAAAAGTATTACAGTATCAACAAAGTTCAGATTTCTTTGGGAACATAATATAACCCAAGCAACTAGAAC
AATATTGATATAACAACTTTCATACAGTAGAAATGATTCTAAGTACACTGCATACAAAAAAAACAAACAA
GCCCACAAAAAGGGAACCCAATTATAAATAGAGGCCCAGCTATACTAAATAAAATCTATATGACAAATAT
ATCCTATCTGTCGTAGACTCATAAATACTCAAAACTCCAATTTTAAATCTCGACAAGATTTCTACAAAGG
CATATGGATAAAAATAAAGCCACAAAACATAGATGCAAACTAAAAGAATTCAGGGGCATTCAAAAATCTT
GATAGACTTTTCATGAGAATGTTGCAGAACTAAGAGTAAAAACCAGAGGGGGTGTGAAGAGGCCGTTTAG
AAAGAGAAGAAATAGCACTCTCACCAGTAAGATTCTAAAAGGACAACCCACAGTGCATAGAATTCTGAAC
TTGCAATTTAGTGTTTAGTAGGTCTAAGAATATTAAGTCCAGGACTAATCGACACAAAATTGAAATTTCA
TGGATCTATTTGACACTAAATTGAAAGTTTAAGGACCTTTTCAACATTTCTTAACATTTAGGGACCAAAT
GACATTAATATGAAGCTCAGAACCTATCAAACACTTTTAGACACATACCTGAAAGTTCATGAACAACTTT
ATAAATTTTAAAAAATAAAAAATAGCATATTTCAAAAAACAAATAAATCAGAGAGAAAATATGACAGATT
ATTTTTTCGCTCAATGCAAGAGGATGTTATCTTATGTATGTTATCTTATTTACACAAGTTTGAACCATAA
TCCCCCAAATGACAGGAAACTACCAAATACTGTAAAGCCAATACCTCTCGGGTGCCGTAGAAAATAGTTC
TTCAAGATCACGAGGACGCAAGGCACCATCCTGGAGTGTAAAGAAAAGTCATGGATTACTTGGCTACGAA
AACAGATCTATAAGCTCGATTATACTAAACAATTTTAAAATACAAAAAGGAAAAAAAACAAACAAACTAT
GGCTGAGAAACCAACAAAGAAACTAAGAATTTATAATTTGGGAAGTTGTAAACTATGACATACACCATCG
CCATCATAGAGCTCAAATATTCCCCTTAGAAACTCGAGGGCTTCATTTGTCAGCTCCACACTCTACAGAA
AGAACAGCCATTGGATAAGAAATTTTGAACTAGAATTAGAATAATATGGAACACTTTAGGAAAAATTACA
AAATGCAGTATATTGTCTTACAAAGTCATTAACACCTGATGAGAGAAAAAGAACCAAATCCTGATATTCT
TTATAATATTATATCATACCTCAAAATGATAGATATAAAACAACAAGAGAACAAGAAATCCTCCCTAAGT
CTAGTTAAAGAAAATCACTCCACTTGAAAATGAAAGTTTTCACATCCCAACCATTCATACAGGAAAAGTT
AATGGTTTCCACTCTAAGTATTTACCACATCTACATTATTAATTTAGAGCCTGCTCCATGAAAGTGAAAG
AAAGAACTATTTCGAGAAAAATGTTGGAGATAACAAATATCCAACAGTACACAAAATGTAAAAAGTGCAT
ATATATTATGTTAAGAATAATTTTGAAAGTCATCATCAAAGTGTGTGATGCAGTTGGCGTTTTCTACAGA
AATTGAGAGGACTGCTGCAATATTATTTTCATTACCTGATCTGGAGCTCTCTTTCCAAGAGTTGGGATAA
GTTCATCCGAAAGCTTGATATCATTATCGTATCCAAACTTCCTGAGTACAGTCCATGTTGTCTCCAAACG
CCCCTTCTCTATAAATAAAGCATGAAGAAAGAGGAACCCTGTCAAAGTGAGTCCACGATCATTCACTCCT
TCAGGAAGTTTTTCTTGCACAACCCTCTTAACCCCAACAATTTCAGAAGGTTGTAAAGGAGCATTGAAAC
ATTTGACCTGAAAGAAAGGGGAAAAAAATAAAAAAAATAATAAAAATAATGATAATAAGAAAGCTCCATA

GAGTCAAGTCAGTTATTTTGGACACAGATATTACAGATGTCGAAAAATGCAACTAATCTATCAATAAAAA
TTTTATGAGGTGTTCACCAAAAAAATTGTAGATTATGCTTAAAGGAAGACCTGAAAATCATTCAGCTCCG
CATCGCTCAAAGCACCGTCTTTATCGTGATCACAAAGAATAAATATCCGTTTCAATGCCCGTACACACCT
AGGCTTTAGAGTCTGAGTTTCTTGATCAAAAAGTGGACCTGTTGGGTGAAGGACAGCTTTCTGGGCATAG
TAAAAGACCTCAGGAATCTGCAAATGAATAATAAAATGTTGAGAACATGTGGTACTGTTGCCTTAAGAGT
GAACTAAGCAAAAATGAGAAGTGAAAATGTACATCTATGAGATATAGATGGCAACATTTTCATATAAGCA
TGAAAAACTACTCCGCAATGATTTTAAGTGAACCGATAAATGAAATTGGATGTGAAACCAGAAAACAATG
CAGAACGGTTTGAGGGTTCCTCTTCTTCATATAGTCATGAATGAGGGAGCAATAAGTAAATCTGTTTCCT
TTTTTCTCTGAGAATGCAAATAATTTCATAGATAGATGAATACCTGGATATGTTTAAAAGCTGAACACTC
GATGCATGTTTCAATTTCTCGAAACTGCTGCATTATTGGGGACATAACCTGCTCCAAGCTCACCTGCTGA
TTCTCATCTCTCAAATCCAGCTTACAGCCCACTACTATGACGGGGACCCTCACCTGCTCGTTTAAACCAT
TATAATCAACATCAATAAAATAACCAACATAACTTATAAAATACTGAGCTTATCTAGGAAAAGGAACCCT
TTGCTTTTCAAATTGAAGAAACTTATCAAGATAAACTTATTAGGTTAGCACCAAGATCAGTGATTTTATT
ACCGTGTCATTTTCCATCCACCAAGGTAAAAGGAGGGAGTAAAAGTTAACTAACCTCCAGTTGACGAAGT
TTTGGAAGCCAAAAAGTACTCAGCCGATCAAGGGTGTTAGGCTGATCACAAGCATAAGTTAACACCACTG
CATCAGCTCGCTTAAGTTCCTCAGCAACTTTTGCACTATCCTCAGTGCTGGGACAAAAAAAACAAGCAAA
CAAAAAGAGGAACCTTAGAGACTTCAAAAATGCCCTTGAATACCTTGTGGAAAATTACATTAGCGAGAAA
TACTGAGCTTCATTTTCCAGTTGGAATTTGGGGCGGGGGGTGTAATTTGCATTAAAGAAGCAAATAAAAC
ACATTAGTGGGACGGTAGACTAGCAGTCTAGAAAGCTAAGACTTAGAAAATGGTGCTCCAACCACCAACA
AGAAAATGGGCAGAAGCAGTAGTCGTGCTTATATATAGAAGCAGGCAGCAAAGCAGAAAGTAGAGTGAAC
AAGAGCCAATTCTCTGGACTCCCTTTTCTATTTAATTTTCACCCACTAATAACAAGTAGCCATGCTTAAA
TATAGAAGCAGCATGGGAAGAAAGGGATATACAGATATATCGCCGTGGGTAGCAGAATTCTCCTATTATT
ATTTATTAAAGTTGACACTAGTTATCTAAAGACCATAACCCTAAAAGGCGGGTATTAATGGCTCCAATAG
CACATTCTCTCAGTCTATTGTCTCAGTATCAGGACAGAATGAAACAAACAAAACAAACATAGCAACCAAT
ACCGTGATGAAGTATCGATGATTGTGGTGGGAACACGATCTGGGTAAAAGTCTTCAGGCAGCCTCGTCGG
CGGCAATACCGGGGGGACATTCACTGGAAAATTGTCCGCCGCAGCAGTAACGATCAAGCTCGACTTTCCA
GTACCACGGTCCCCAGCGATAACTATACGAACCTCGGTCCTGCCACCTGAGTGTACATTACTAGCTGGCG
CTCTTGCCATTGGAAGTCTAAAACCTGCAAAAGCGAAATCCCAAAACAACCTTCAGTGACTTGAGAATTT
CTTCAACAAAAAGATAAACACCATAAAAACAAAAAAAATTAAAAAATCGAGCTACCTCCATCAACAACCG
GATTCTTCAACAGCTCAAAAATGACATAAAAATAAAAATAAAAATAAAAAATACAATGAAATTTTCCATA
GACTGTGTGTGTGTGTGAGAGAGAGAGAGAGAGACGCAAAATACAATCAAAGCAAAGCGTTTATCGGAGT
GTGAAAAACAAGTAGTAACGAATGAAAGAATGAGATAACAAAAGAGGAACGAAGAGTGATATCGTGGTAT
TGAAAAGTAATCGACATGGAATCCTAAGGGTGAAGAAACTAGCCTTTAGAGATTATATTGTATTTGAAGG
TGGAAAGTTGTAAAGCGCCACCAGACGCGTAAGCGTCAGTAGCAGCGACGACGGCGATGTGGCGGAGCTG
CTGGCGGAAGTGGCAGCGGCGCGGCTGCTATGGATGCGTCGGGAAGAAGATGGAGAGAGAGAGAGAGAGA
GAGAGAGAGAGAGAAATGGGCTTTCCTCTTCCTTTTCTTCGTGGCTATTTTTCAAAATACGAAAATAGAA
AAAAAGAAAGGAAAATTATTGCCCTCTTCCGCCTATCGTTACCCACAAGTGCACGGAGTTTCGGCGTTTT
GGATCAACGTCGGGCTTTTAGTCCGGCCCAACTTCTTCTTCTTTTTTCTTCATTACTTCGCCTTTACTTA
CTTAATAAGTTTTACAATTTCTCTCCTTTTTTTTTTTTTGGTTTAAAAATAGGTTTAGGGTTCTTCTACTA
ACTTTATATTTTATTTCAAAGTTTAATATCGTGGATTTTAGTTATACGTTTAGAAATTGCATTAGGGTAA
TTCGTAATCTAACTAACCACGTTGTTCTTAGCTAACTTTTTCTCATCCACGTAATTGAATGTTTTTTCCT
CCCGTTCTCTAGTCTTGTTGAGTCGGATGTAGCTACTTTAAGTTTTAATTTGTTGATTTAATATATTGTT
TTAATTTGTTGATTTTTTTATTTTAAAGGATTATATATTATTTTGTATTAGAAGAATGGTTTCTAGAAAT
CATATCCTTTTATAAAACTGCTTTTTCTAATTTTAAATATCATGTAGTTATTTTGAAAAAATGAAACAAA
AAATACTTCAATGATCTTCCCAACATATTTTTTAAAATTTTGAAAATTTGGAGACTAAATGGGTTTATTC
TCGTGAAATTAGGAACTAAAAAGGTACATTGTTAAATCTCATGAACTAAACATACGTATTCACTTTTAAT
ATTATTTAAAAGTGTAGTTTTTCTTATGAAAAATTATAATATTATTTAAAGTGGTCATTAAAGTATAAAT
TCTTTAGAACCATATATAAAAGATAAATTCTTATGGATAGCAAAAAATTTGAAATGTCAAAGCAGCATGT
ACATCTAAGATTCTTCTATTTTAGTAACCTATTAAGTTGTTTATTAAACTACTAGAAAATATGTAATATA
ATTTGACCATTTGAAAAAAAAAAAACTTGAAAACAAATCAATTTCTTTATACTCTGATCGATAATCATTTG
GTTTTTTGTTTTTTGCTTTTGAAAATTAAATCTATAGACACTATTTTTACTTTCAAATTTATTCATTTAG
TTTCTACTTTTTTATTCATTGTTAAAAAAAAAAAAAACAAGTTAGGTTTTGAAAACTAAAAAGTTATAGCT
TTTAAAATATTGTTTTTGTATTTGAAATTTGCCTAAGAATTCAACCGTTGAACTTGATGCAAGTCATCGT
AAGAAATTAAAAAAATAAAATAAGTTTTGAAAAAAAAAATACTTGATTTTCAAAAACCCAAAAAAACAACG
AATAAAATAGTTATCATACCGTGTACAACTTTGATATTTTATCAATCATTTTTCACCTTCACCCTTTAAA
TATGTTAACATACAAGAATACTCAATCAAAAAAATTTAAAGCAATAAAAAAAAACTAAAATTGAATGAAT
GGTTATAGAGATATCTTAGATAATTTGACATTTAAATCATTAAATAAAGAAATTCGTTATTATTTGATAT
TCACATTCTAAGGAAAAATAAAAAAATAAAAAGTCATATTTTATCTGTAATTTGGGTTAATAAAAATATA
TATATATATATACAAATCACGTTTTCTAAAAAAAAAAAAAATTATATTGCTTATAAAAGCTTAGCTCAAC
TTACCGGTCCATCTACGTAAGAATCGTTAGGAACATATGTTTTCCTCAAATCATCGATTTTTGTATTTTT

AATATTAATTTTGAAGTATCATTTTACAATATATATTGTATAATGTCTTTTAACGGTTAGGTTGGTATAG
AATATAAAAAATAATTAATAAATGCATTACAATCAGTCAAATGGTTGTGTGGTTCATTGATACGAGGGCA
CACTTAGGAACATGGCATATAAGACTTGTTTTTGCCTAATTTTAGTTCTACCTAACTTAGGTTTTATGTT
GTAATTGTAAATTAAAACAGTGCAATTTCTCCGTCTTTTCTAGAGATTTTTCCCTAAAAATTTTAAAAAG
TTATCAAACTCGATTCGCAACAACATTTTATTGAATAGATAACGTTTTTTTAACGTAATTTTCATATCAT
TGTGATAATGTTAGTTTAATACCAATAAAAAGTACTAAAAAACTTATTTACAATTATGTGAATTTTTTAA
ATTTAATTAATTGATGTTAAATCAAAGAACTAAAAGTGTATATGTAGTGAAAAAATAAGCTTAAAAACGT
AAAATTTTGAAACTAATGTAGAAGTGTAACAATTTGTAACCTAGAGACAAATCTTTTTTACCTTATATAT
AATTGTGTCCCTAAGTTGGATTTCTAGATTCGCCAGTGGCTCGATTGTATCACAAGACAGTAGCTTCCTT
GTTCAAACTCGCCCGGAAGACAAATCATGCAGTTTCTTTTTTCCTTCTTCCTCTCACACAAAACTCTCTC
CCATATTGTGGTTTCTAATGTGATTGACATTATGTGATTGTTTTGAATTAACAACCTTCACAAGTTTTAT
GTCTCCTTCACGACTCACCAGTTCTACAGTAGAATGGGTTAACAATACATGAATGTCAATATAGTGCACC
TGACCAAGTACCGATTACATATTGGTCGATATATTTATACTCAACCTTTTCTATAATACATTTTTAAGGT
CAGGATAAGGACAAGACGGCTAATGACAAAAATGATCCATAAAAGAGTTAGCTTCTGCATATAGAGTCTT
GATTTTAGATAAACATTTAAGTTTTTATTTAATTATTTTATTTCGTTTTATTTAAAATTACACAGGAGTA
CTCCAAACTTTTTTTAACCTTTTATATTAATTGATTTATTATTTTATTATATTATTCTTTCAAAGTTTTG
TTAATAAAGAAGCTTTACATTTTCCTACTTGAGTTTATTGAAAAATTAAGTATTTTAGTTGTTGCATCAC
GTAGTAATGTAAGTGAAAATACATAGAATTTTCGAGTTGTTACATTTATATACATTTACAATGAAATATT
CCAAATAAATATATTGTGTATGAAAATGTCAACAATCACTTTTCTTAAAGTCTGACATAAAAAAAAACCA
TCAATACAATCTTTGAGTCTAACGGTCGACTTTTTTTTTTTTTTTTTTTTTTTTTTTTTATTATTGTCAAC
GAAATCGTAAAAATTGATGCTAGCAATATTGATGTTAAAAGAGTGTAAACTATAACATAGGCCAACTTTG
TAATAATTTCCAATCCATAGACATCTTATTTTTCTTCATACATTAAATTAGATAGAATGCATGGTTACAT
TTGAGAAGTTTCCAAATTTAAATGTGAGTAATGACTGGTTTAAATACTAGCTACAAATATATCTATTTGG
CCCAACCAATTTCCCACCCTAGATTGACATCCAATCTCATATTCAAATACCAACCAATAACTTTTTTTTT
TTTTTGTTTTTTCTTTTTCATAAATTTAATTAAGAATTTTTAAAAATTATGAAAGAAGAAACGATAATCC
ATAAATGAGAATAGGTATCATACAAACCAAATCATATATGATAGTGTTTTGAGAGGAAATATTATGTCAG
AAAAACGAATCTCTAACAAGCAAGCCAACACGAGAACTACGTTCTCAGGGCAACTGTCCACATTGGCATC
AATAGAAGAATAAAGATCCAACCAAGTACTAACTTTTGTTTATAACATTTAGTGAAGCCATATCTTATGA
TGTGCAATCAGTTGGAGGAGATGAGAGACTGAAGAGGATAGTAAATAGTACAAGAGACTGAAATACGAGA
ATATTTTGATACTCTGAAAGGTGTCCTAACCAACACATGAAAATTGGGAACAAAGAGTATTTTTCAAACA
AATTAATGCTTTTGGTAAAAGTTTAAATGTTTTTCTTTTGCAAAAAATTTAAACACGTGCAAGATGTAGA
GACCACAACTAGTGTCCAAAAAAAGTAGCTGAGTTTCTCTTCAAATCGAAAACACTAACCCCAAAATGGG
ACCTAAGAAGTGGATCGATCTTCTCCGTCCATCCAGTTGCCGTGAACCTAATCCGACGACCAATACCCTC
AACCGAACGGTGGCGCCACTTCTTCAAAAGACGCCACCGGCATCACGCGCTCTCAAACCTCTTACTCGGC
AATTCCCCGTCTTCTTCCTGTGTTTTGTTCCCATTTAAAAAGCCTTACCTACCCAAAATAAGACAATCCC
ATTTTAAATTCCTTAGCAATTCATTCGTTCGTTCATTCATTCATAGGAAGAGAGGATCATGAGTTTTGAT
CTTGAATCGCTCTCGGAGGCTACTTCCGGTGCGATCGGTTCGCTTGTCAGCACCACTATCTTGTACCCGC
TCGATACCTGCAAGTCAAAGTACCAAGCCGAGGTTCGAGCTCATGGCCAACAGAAATACAGGTTGCCTAA
AGTTTCGTTTTTGTCTTTTTTGATCTGGGTTTGTTTGTTTCCCCTGTTTTTCTGATGGGTTGGGGTAACT
TTCTTGGTTTGCTTTCAAATCGTGATCCGGGTGTCGTTGATGCAATCGTTACCCTGTTTTTGAAGGAATC
TCAATAGTAAATTGAGTTAGTTTCTGTTGAATGCTTCTACCGTATAATTGGAGTTCTGTTTTCCCATCTT
ATATTCGATCTCATGATCTTTGAAAATTTATCTCGTTATTGATCTCACGACTTCTCTTCATCGAATTTTA
TGTGGTAAGGGTTGTTCTTTGTTTAATTGAATGATCTATAGAATTTGAGCGCTTCTGAAGTGGTTGGTTG
GTGAGCTGTGAGAATTTTGTGATAGTTTGAAAGCATTTGCGGCTGTTTCAGGTTCCTTGGTATCTGGAAA
ATCTCTTGTTATGTGTTCAATTTTAGCTCCTGTTGTAGGCTTGTTCAATCTTGTGAGTTGGATGAGATGA
TTTAGTTGAAGCGTGAATGCAAAATGGGCTTGTTTGGGTTCATGGCGTCTAGATCATTAATAAATTGATG
CTTGTGGAAGAACAAAGCAATACAAAAAACTTGTGGAAGAACAAAGCAATCTGGTTCACACTTTTGAGTT
TTGACACACACAACACAGTAATGGCTCTGCTTTTCTGCTAGAAGACTGATTCGATGCCTAAAAAATGTTG
GATGCTGTTGTCGTTTTTGCATCTCTATTGATTCAACTGGTTTTGGCTTTTGTAGGTATCTTTCAGATGT
GTTGTGGGAAGCAATAAAAAACAATCAAGTTCTTTCGCTGTACCAGGGACTGGGAACCAAGAATCTACAA
TCTTTTGTTTCTCAGTTTTTGTACTTCTATGGGTATAGCTACTTCAAAAGACTATATCTAGAAAAAACTG
GGGCTAAATCAATTGGAACAAAGGCAAACCTGTTACTTGCTGCTGCTGCTGGAGCTTGCAATGCCATCTT
AACTCAGGTAGAAATATCTCTGCTGTCAACCTTGTAATGCATAAAGCATAATCTTTCCATCTTTGCGATC
CCTGAATTTGAGTTCTTAGAGCAGAGGAAAGGATGATTCTCATTTTTCATTTGGAATTACAGTATTGCTA
TTATGTTAATTGCCAACTTTACAACATAGTCAATTCTTGTTTGGGATCTGTGAAACATGTTATTTTGATG
TCTTGACTTCTAAAAAAACGGATCTCTACCTTTTGCGTTGAAGCTGACACATCATTCTTTTGATTTTGGC
TACCAACATGGAATCTTGTAGGTTTTGGCTTCATATTCGTTCATATGCTGTAATTTCTCGATTCAGTGTG
TGAAACTTTTTTAGAATGAATGAATGAAGAAATAATTCAAGAAAGCAAAGCATATTTAACTTTTTGTCCA
TTCACTTTTAGTTTCTGCCTGTTCTGCTTGTCAAGATAGGTTTGCTTTTATTACATTATGAAATGCAACA
TTGAGCTGCACAGTTTGATGAACGTCATTGTCATATAAAGTTCCAAGTCTCTTTATTTATGAAGTCTTGT

ATGGACAAGAACACGTCTTGAATATTATTGAAGTTTAATGGTTTAACTGAACGTGTCTTCGGAAATTGTT
TCTTGTATATAAAAAATGATTTAACTGAAGAAACTCTCTTGGTTTTCCATTGTGTAGCCCTTGGATACAG
CTTCATCAAGAATGCAGACAAGTGCCTTTGGAAAATCGAAAGGGCTTTTCAAAACACTGACAGAGGGCAG
TTGGAACGACGCATTTGATGGTCTTGGAATTTCTTTGCTGCTCACTGCAAACCCTGCCATTCAGGTATTT
GTCCCATGCATTATTTTCAGTCATCCATTTGTCGGTAGCCGGTGCGACAGAAATCACTAAGTTTCAACGT
ACTCTGCACCCAATTTATCTTCTTCTCAGTCATCCATTTGTCGGTAGCCGGTGCGACAGAAATCACTAAG
TTTCAACGTACTCTACACCCAATTTATCTTCTTCTAAGTATTCTCTAGATTTGACACATGAACTATGATT
TATTGCAGTATACAGTATTCGATCAGCTGAAGCAGAACATCCTCAAAGGAAAACAGAATAAATCAGAACA
AGGTTCATCTCCAGTAGTTCTTTCTGCTTTTACAGCTTTTGTAATTGGTGCAATTTCAAAAAGCATTGCC
ACCGTTCTGACATATCCCGCAATCAGGTGCATAATTTTTTCTTTGTTCACCAGAACTAGCATATCCTTAT
CCTTCAAACACTGGCATATTGAAAGAATGAAACTTTCCCTCGGGGTACAGGTGTAAGGTGATGATCCAAG
CTGCACATGACGACGAAACAAAAGAAAATCGTCAAAAAGTCCAGCGAACAGTTCCGGGCGTTGTCCATTC
TATATGGAGAAAAGAAGGGATACTTGGATTTTTCAAGGGATTGCAAGCACAAATCTTGAAGACTGTACTC
AGTTCAGCTCTTCTTTTGATGATAAAAGAGAAGATCACTTCAACTACTTGGGTTCTAATACTTGCAATCA
GAAGGTATCTATTGCTTACTAGACCTAAACTAAAAAGTAGCTGAAGGTATTCATTAAAGGCCAAAAACAT
ATGAAGAAGAAGAAGCAATAACACGTGGGAATAAGCACCATTCCCATGGAAATAATGCAAGCTTGTTGAT
CTTTACCCATTTTACCACAGTAATAATGGATAGATTGTCGTTGCGATCCTTATGGTTTGGAAAAAGTTAG
AATTTAGTTTTTGTTTGGAAAAAGTTAGAATTTAGTTTTTGTTTGGAAAAAGTTAGAATTTAGTTTTTGT
TTTAGTTTTTGTGGTTTAGAACTAGAATTTAATGCATATGTTTTGATAAAAGCTTTTGTAAATAGTCTCT
ACAATAGGGTCTATTAACGAGAATTTTATCAAATCATAGAGATGGTGTATCAAACTACAAGGATTAAACT
TTAACTTTCTCTAAACTATGGGGATTAACATCGCAACTTGAATGATAATAATTTATCTTATGTTGAGTTT
AGTATTTAACGTGGAAATGGAGGATTTTTTTTGTTAGAGGGAGATTGTGTTTAGTTAGAACATAACTGTA
GCCAATCCCATAAGTATTGTATTCAATTAGTAGTCCATGTAACTTAGTTTGTAATTGTAACGAGAAAAAT
TCCATTTCGTTCCCAGATTTTCAAATGTTTAGTTTTGAGTAGTGTTTGTTGTTTTCATTTGGTTATAAAT
TTAAAAGGATTGAATTCATTTTATTTTAGTTAGTTTGATTACTTTTAAATTTTTATTTTTAAAAGTTATT
AAAAACATGGGTTATTCTCAAAGAGTAGTAAAAGAGTTTATTATTATAAAGAAAAATCTTCTGATAGCTC
TTTGATAATCGTATAAAAAACAAAGTATTATGATCTGTTTTTTATTAATTTTTGTCATGCAATTTCTTTA
TAAGTGTCCTACTTTAATTAACTGAAAGTAAAAGATTAAATGAACTGGATTAAGTTTAAAACCGTTTGTA
GTTTAGAGATTAAATGAACTGGATTTAAGTGGTTTTAGTAATTTAAAAGTTTTTTTATTATTTTTTATTA
ATCTTATGATTAAAGAATAAAAATGATTTAATAATTAATACTAACAATACTATTTTCATTATTATTTACT
ATATGTGAATAGTTTTTTTATTATTATTTACGATAACTTTTAAAAAATATATTTGGATTTGTTGTACTTT
ACTTTTTTAAAATAAAGGAATGGTGTTATGAATACCAATCCAATTGGGACCTTGACTTTGGACCAATGTG
GAAAATATGTTGATTTTGTTGATTTTTTTCCTTTTTTCTAAAAAATAATTTCTACATCTAGGTTATATAA
TTCTTTAAATTGTACTTCACTAAGTTTCCCTTTTTAGACAAACTGTTCTTTGTATTTCATCTATGTTTGT
ACATATCTTTTTAATCTTAATTTCATGATAATTATTTTTTCTCCATGTAACAAACTAGTTACCCAACTAC
GACATGTCATGTAGAAAAAACATTATAGCTTTAAAATTAACTACAAAATATAGTAAGATATTATAATCTA
TTTACGATGAATCATAATAAACAAATTACTGTCATGGTAATCTATCTTGATCTAGTTTATCATAATAGAC
CAACTATTATCGCAGTAGTCTATCTTAGCTTATCACAAGTAGATTGTGACATTTTCCTCTATTTGTAAAT
ATTTTCAGAAGTTTTGTCATTTAAGATAAAGTTATACTTATTTATTCACTAGAACAGTTTAGTTTTCCTT
TTTTTTTGGCTAAATGTTCCAAGAACTACAAGTGAAATCCATATTTGATTGGTTTGACTATATTCCAATT
CAATTTTAAGCTTCTTGAAACTATATGAAAATATGGGTTTATATTATTAGACAATATATATGGTCGATAG
TATACATTTTATGTCAATTGAACCACGATCATTTTAGAGTATTCTCCTAAGAGGGTAAAAATTAGAAAAG
AATAATCAAGAATAACCTAAAGAACCCATAAAATCAAAGTTCTTGCTAATAAACTTGATCATGTTCATGA
GTTCAAGATTTGTTGATGGCTAATAAAGAAATGCAACACTTCGCATCATCACACGAAATATTTTCAGATA
ATACCAATTCAACTCAGACATTGTATGTAGTACTAAGAAAACACGTTCACGCACATTGTAAAAACAATGG
TAAAAATGAAATAAATTACATTCATATTATAAGTACAACAATGATTCTGGAAAAGGACAACCTATATATA
CATTTTACAAAGTTCATCTCTGATAAACAGATCTTGTTCTGATTCTGGGATTAGAAACTAGAGATACAAC
ATTTAAACAAGAACCAAAAAGATTAAAAAGAAAAAATATATATGTGGGTAACAAAAATGATCTCCAACAT
GACGAAATTCTCTCTATGTATGTGGTGTTCTGAATCCATCCAGTTCTGGACTGCATAAATCATACTATGA
TACAGATATGAAACATCATCTAGTTGCTACACAGTAGTTCTTCTAAAGAATGGCCGAGTATGGGTAGGAA
TGTGGATCCCACGAGACTTAGATTGGATTTATTTGGTCTTCTTGAAAACCTGGAAAGTGTTCTCCTTTCT
TAATTTCATCTCAAGGAGTGTAATCATCTAGCATTTCCTATTACAACATCAACACGAATGAGCAGCGGGG
TCGACGAAGGTGATGAAACCTCATTTGGAGAAAATAGCAACAGCCACATCATCCATAGGCTATTATACAA
AACAACATGGCTGGATCCTCTTATCACCTATTAATCAAGTACTGAATGATTGGAGGATCACGACCTATTA
GGTTTGCCCATAAGTTCGCGAGCTTCAAAAAGTAGTTCTCCCCATTCCAGACCACCAAATCCATACCTAA
CGCCAAACCAAAAACTCAGGAAGATGGAACCGTAAAAATGAATTTCACCAAGAAATGTACAAAGGAGGCA
TAAAGATAGGAACGAGAGTAGTATTTCATTGCTAGAATTTCAAATATTTATCTTTCCAATTTGGAAGAGG
CAGGGCTTAATTTAGTGTACCGATTGCAATAATCTGATAGGTAGATATCGTTGGTGCACAAAACTGGACG
ATTCAAAGTTTTTGGTGTCATCGAACCCAAACTTAAGCCATAATCTGGTTACTCAGATTACAATGAAATC
CCAAGAAATTTGAAGTAACGTTTGAAATTTTGCACAACATAACATGGAACTCAAAACCTCTTTACTAGAA

TGTTCCACGTCATGTAATTATGACTGCTATTTCAGAGCAAAGGCGATCTTTCCCATGCTTTGCTTGGAAT
TACCTAGTTGCTAATGGCGCAAGAATTTTTACATCAACAGACTGCCAAATCCATGTGGCAATCATCACGG
CATTCTTTTATCTTAGATTTTCTGAACTCATTCTCCATTGGGTTTGTTAGCTAGTACTTTCTTTTCTTTT
TACCGAAAGACTTACTATCATTGCGAAGGAAATTCGCAAGTGAACCTCATCATATGCTTCTTTTAGAAAG
TCAAGCTTCTCTTCTTTATTCTTTGCTCATCCACAACTTTCCGGTGATTTCAACTTAGATCACTCTTCCA
GGAAAGCTAACTGCTATGTGGAGATTGTTTGGGAATTGACAGAGCCTTTTGTGATTGTACTAATGGGGGC
TATCTTGGGAATAGTAATAGGACAGTAAGGGTATAGTGGTAATTAGGTAAGAGTAACCTGGTTATAAATC
TAGGGTATTAAAGTTTTTAGAAGAGGATCATTTTGGTGAGATGGACCATTGTGGCACATGGGAGACACAT
AACCCTCTCAGAACTGAAGCCAATTTGGGTAATTTTTCTGGACATTACAATATAATGTACGGTGGAAGTC
ATTCAAAAGAGAGCAGGACCGAAAAAACCACCATTTTGAGCTAGTGGGACTATTAAGTTCTTTTAAAATT
AGGTTATTACGTGGTGACTGGTCATTTTTTTAAAAGAAGAGCAATGATGATATGAATGAGGATGAACACT
GTTAAAGCTATTCTTTGGGAGTTGTGAAAGGAAAGAAATCCTAGATCTCCGATGATCATTGTAACATGAA
TTTGGAAAATTGGAATCATGTTAGCTTTATGAGGTTCTTTTGGTATACTCTTGAAGTTAATTTTTAATTG
TCCTTTTTTCTTTTCATTTCCTATTTTACTTCTATATATATGTGTGTATTCAAGCACATGGGAGTAGTTT
CTCTGTAAGCTCTTTCATCTGTGGGTGGGTTGATGAATCACCCCCTGATCTTTTGTAATTTTCGGTTCTA
ATGAAATATAAGTTCCTAGCTTGGAAAAAGAAAGTACAATAGATTCAGTGCATACCTGGAATTGATTCAG
CACTCAAGAGCGATAGATTACAAGTAGGTTGCTGTCCCAAGAGAACATGCTTTCTCAAACCCCATTCATC
AAGATTATCAATAGCATTGATCGAATCCAATAATGCTTTGCAATCAATAGGACCATCTATCTTGCAGAGA
GCTACAACAAGTCCTGAACTCAACCAAATATTGGAGCATTCATTCGCAAGAGAAATCAAGTTATTGGGAT
CAACCTGACCTAATAGTATCCATGGCTTGTAAAGTACAAATGAAGCTCTAAGCACTTGAACTACCCTATA
AATCTCTCCGAGGGCGCAGATATATTGTTTTCCTGAAAGAATGGCAGATATGAACAGGTCAGCTAATTTG
AAACTTGCAATATTATCCACAGGCTAAGTAATGCAGTACCTTGAGGTTCAGATAATATGTAACTTTCAAC
ATTTCTCTGTATAGATTCCTTCCAAATCAAAGCACCGTGTTTCAATTCTTGAAAGCAAATAAACATCATT
TCGTTCCATATGGAAACATAATTAGATTGCTCCTCCACTGATACAAGTTTAAGAATCGTCAAAGTCGACA
CGACGTGTTTCAAGAGCTCAACAGCCGATCTCAAATCATTTTCTGCCTATCAATTGAACAAAGGAAGTAC
TATCAAGTAATATTCATAACCAAGGGCAAGGATAAAAAGTGTTCAAATAATTATAGTTTCTGGAGCCATA
AAATTGATGCAGCGCACAAGACTTGCGAGTGTAATTCAAATCGCTATGTATTCCAGATGTATACTTAAAA
AATACAAAAAAAATATACCATAAAATTATAAATCCTATAAAATATACCAGAGTAGAACACTCTTACACTC
GGCGGCAATTATAACCTTCCTAATAACTTGGAATTAAAAAATACATTCATGCTTGCAACAAAAGGAACTC
AACAAAAATTTGGGTTTGTGCACTCAACTAGGCAATGGAAGGTGGTTATGGAACTTGAGCACTAGGAAGT
TCAAGTTTCTAAAAGTTACTAATGAATATTTGTTTCCTGTCAAAAGAAATATATATCATCCTAATACAAG
AGAGTCTTACCAATAGTAACCTTTCAGATAACTGGAATTCTTCATCAAGAATTTGAAACCTTGGATCCCG
CAGCATCTCAAGAAGCTCTCTGAAAGTATTATTTTCTGGAAGGAAAAGATCGCTATCAGCTGCAATGTTC
TCTTGTGACAATTCGGCACTGAAAATCTGAACAAAGAAATAAGAAAATTTTGTTCAGGAGTTCACAAGAA
AATAGCATTCCATAAGAAAGACACATTATCATCTCATAGCAATTATCATAATGGTTACGAAAATAGATGC
TAATGCAAAAGGAAAAAGAACAACTTTTAACAATAAGCATATAGTGTTTACTCAAACTATCAAAGAAAGT
ATACATCTCGAGAAAGTTTAATTAAAGGGATCTATCATGGCTTAAGATTGAAGAGAAAATAATAAAAGAA
AAGCCAAAAAGCCAGTAATAAGCAACCATGGTTGCTAACCTGAATTTCTTCACAAATGGTTCTGACGTCT
GCTTCTTCACCAGAAAGAAATTCATTACTTCGGGCTTTCTGTCGAGGAATAGAATTTTAAAATTAAACAA
TGTTAAGAACACATCATATAGCAACAACCATTTCATCTAACACACACATATCTATAAGATTACCAACCTT
TAAATTTTCAAGAAGGTCGTGGACAACATGGTTCAAAACAAGATTTAATTTGTGATAAAAATCCATGTAA
CAATCAAACTGAAGTTTAGTAGATGACAGCTGGGGTACACCTCCAAGAGTAGTAGTGTATGTTTGATCTA
GGATGTTAACATCAGGTGACGCATCTTTGAACTCCCAGGAATCATCATCATCATCATTGCCAAAATCGGA
GTGTGACATTCTTGGTGATGGAATTATTCCATTTTCATTTTCTTCTGGGAAAGACTTGATGGAACCATTG
TTCTCGGCTTGACTGTACAGACTTGATATCAAGTCATTAATAGATACAGTAGAACCAGGATTCTTGCTAT
CAAGTCCTTCATGGGCGACAGATACAAAGGTAGAAGCACCTTGTTTCATTGAGAAATCATCAGTAGTTTC
CAGCTCCTCATCTCCAAAAATTGACAAGGGCAGGGCTTTCTGGTGACTTGACAATAATTCTGAATTCCTC
TGGAAGAAAAATTTGAAAGAAAGAATCATTAAGAACCAAACCAGAATATGTCTACAGAAACAAAAGAAAC
ACAGAAAGATGAAGGTTGAGAAGATATAAACATGAAAGGTAGTATATGCAGTAAGATTGTGGATCTGGAG
ATACATATTATATATGCAATACAAATAGAAGGTGAAGTGCACAAAATTGAAACAAGCATATTTTGAACTC
GACGTGAATTGAACGCGCAATAGAACAAGAATATTGAAATCATCCATGTTCGATCTATTTACAGTTACAA
GGTTTATAGGAACCACCCCAACAATAATATAAGAGGAAAGCTATAAATTCAAGTTTATTTTAATACCAAT
ATGTATGTATATATATATATATATATAATAAAATAATAGGAGGACATCCTCAAAATTCAAAATAAGAACC
ACAAAAAACTTTCCCATCCAGAATACAGATGGAAAACTAGTCTAAGACTAAGGTTCAAAGATTTAATATA
TGCAGTCATGGTGTAAAGATTTAATATATGCAGTCATACTTTTAGACAAGAACAAAATACATACAAATTA
CACATCCACACTCCGAGAGTGACCACGATAGCATCAGTAATTGCCTTTTAAGAAACAGAGCAAGAGTAGA
AGAACTTTTTTTTCCTTCTTTTCAAAAGAGTACCCACCCTAAGGCCCAAGAAAGAGCAGAGAAATCTTTA
TAAAAGAACTATTGCTGACACAATAACAGCTTCTCTGCACTAATCTTAGTGTATTATGAAGTAAACATCA
ATTCCTATGAAAAACAGGTTTAGACTTTACAGGAATTGAAAGGACTCCATAGAAACTATTATACAGATGG
AGAAATGAGCAACCACCTGAATACCACTACTGAAACCAAATGCAGGTGCTTCAAAACCAGTAGCAAATTC

AACTGGCTCTGCCTACAGCCAAATAAGACATGGAACATCAATTTCAAATAAAGGTAAAACGGAACTTAAA
TATCACAAATATGATGGGCTAGAAAAAAAGACCTTATTGTTTGATCCAGAATCTGAAAGTGCAGACTTGA
AATCCCAAATATTCTCATTGAAATCATCATTCTCATTAACTAAAGAAGTGTTTAAATCTTTCCCAGTAGC
ATGGAAGTTGTCGTCCAGGTTTCCATTCAAAAAGTTCCTGTCTTCTTTCCCCCAATTCAGATTAAAACTT
GAGTCAAAATTTTGTTGTTCACCAGATTTGTGAGAAATCCCATCAGAAGCAAACAAAGAAACATCGACGC
TGCCATTCAGAACAAGCACCTCTATACCATTGGGAGGAACGATGGCAGGCTTTGACTCTTCCTACAATGA
AAATAAAAGGTATAGCATGATCAAACTTATTAAATATCTACTTCTCAATACAATGCTAAAAATATTCATA
TCAACTCACCAACTTATAGTCTGGTGCATCAGAAAAAGCATCCTTGAAATCCCACACATGCCCAACACCA
TTGGCATCCCGTTCAATAGGAGAAGGGCTTAAAACATTATCTAGATCATTTTGACTATTCTTATTGTGTG
AGTTTGGTAAGATGCCATTTTGGGCCACAGGACTTGCATCAAGACTAAAACCAAAGTCCCATTCATCTAT
GTTGTTAACAGCTCCATTTGATTGAACAACCGAATCTCCATGGCTATGTCCATTGATTGTTGATTCAAAT
GCCTGTGCCACTCCATCAAAACCGTCTTGGATTGGTCTGACAACCTTCATATTATGGAATGTCAATCTTA
AGTATAATCTCTTACACGCATAGCAGTTCATAGACATTGTTTTAAAAGGCACTGAGAACCAATCAAACAC
AACAATAGGAGTCTCTGGTATAAGTTGAAAGTAAAGCACATACAAGCGAATAACTAATAGCAAATAAAAC
TCTACAAATATTTAACCATGTTCCTGTAACCTTAAACAATAAGAATAGGTAACCGTAAAGTAAATAAAAT
TAATAATACGGGCCTGAAATGCACCTTCAATTCGCATTATAGGCTCCTCTTCAGACGAAATGGTTCAAAA
ACCAATTCTTCCTGGTATAGTTATGAAAATGAGTAGCTCCTACTGTTACTTAGACATTAATAAAATGAAC
AACAATAAAACCTGAACTCTGAATTCCTATCTAATTTTTAAACTATGTTTAACTATCTTCTACTTTTAAT
ACATTGAAACTCTAAGACACCTACATTTCCATTCCAATGATTCGGAGCTCCAGCAAATTTGAAAACGAAA
CAAATTCATGGAGTTTGATTTCAACTTCCTTAAAAAGCCTCGAACATCGTTAGTAATTTCTGTTTTCAAT
TTACCTTTGAGTTCCGATCATCACCCGTAGGCGTAACCGACTCTGCAGCCTTAAATTCCCAGCCATCAAA
ATCGTCCACCTCCTCGCCATCATCCTCAATACTCTCACCGACGTTTGTCAACTCAGAATGAAACCCATTA
GACATCACTCCATTAGCTTCAAAATTTAAAGCATCGCCATCAAAATTAGTCTGGCTCCCATAAGAATGAA
CACCATTGATATTCAAATTGGAGCCAACGGATTTCAAATCCAACGAATTATTGAAGTTCAAAGGATCGAA
TTCCATGTTCGATTTCAACGGACTTCCAGCTTTTATCTGATGATTCGGACCATACAAATTAGAAATCAAA
TCATCAATCCCAACCCCAACGCCCAAACTCCCTCCCTTCTTAGCCGAACCACTCTCTTTCCCAACAAACG
AAATTTCCCCAAACCCAACACTAGAACCAACTACACCAGATCCCATTTCCTCCTTCTCTTCCTCCTCGCC
AAAAATGGAAAGCGGAATAGCCCCCTGAGGCTTCGCCCACTGAATCGCCGGGGACGTGTCGGAGAGGTTT
GAATTGGGAGAAGGCTGAGGACGGGAGAGATCAAAAGGGTCACCGATCTGAGAAGAATGGTCGACGAAAT
CGCCCCAATCGTCGTCATCGATGGTGGTGGAGGAAGTTCGGTTGTTGATCGGATCGGGATGGTTTGCAAC
AAAATTGAAGTGGCCGAAATTGTCATCGTCGTCGTCGTCCACCATAGCGAAGAAACTTCAATCTGATAGA
AACCCCGTGTGTGGGCGAATCTTCACCGTCGGCGTGGGATGGATTGAGAATCAGGGGTTGGGTGCTTTGG
AAATGCGAACGGCGCGCATTTTAGCCGGAATGTACAGAGTGTATGTAAGTGGAGGCTGAGGTTTGGACCC
ATTTTATTAAATTATTGTTGTTTTCTTTCTCCTACGTTACATTCACACATTAATCTCTCACCTTATTAAA
CTTTTTATGCCTTAAGGTTATTCTTAGACCAAAAATAATAAGTAAATTTGCCATAAATTTTCATAAATAG
AACAATTTTTTTTTTCTTTCAAACCGAACAAATATATAATTACGTGTTGATTAGAGATATTTTTTATATA
TAATTTTATTTTATATGTATTTGCAAGCTTTTTCATTTTATTTTCTATTATGTATTTGTGAGTTTTTTTC
TCTTTTAATTGTTTTGTTTATTTGATATGTCACTGAACTGGAAAAGACGTATATTAATAAATTAGCCATA
AATCGTAATTTTATTAGGAGACTTCCCCTCCATGGAAAGTGAAGCAAACCGTGGATCAAATGAGTTGGAA
ACAAAGACGTTATCGAATTTGAGTTGGAAACACAACGAAATTGAGTTGGAAACAAATACCGACAAAGAAG
AATTAAAACACAAAAAGCTTCCAAATACGTATAGGTACATATAGATTAAAACATAAAAGACCTACATAAC
ATAACTACATAAAGTCTACCCATGAGCTAAACACACAAATTTGCACAAAAATTTTGAACACATAACTCAT
GAATAACTAATCGCACGAAGAACTTCTGAATTGGATGAAGAGCTATACTTATGAATTAGAGAAGAAAGCT
TTACATAAAATGTTTTTGTTTACATGTATTATTTAAAAATTAATTACATCTCTATTCTCTATGCTTCAAG
GGAAATACTGATTTAGTTTGGTCTATAAACATCCAAATTTGTACTCATTTACTCTTTAATTTTTTTTTTT
AAATTAATAAAAGACTTCATGTTTCTCTCTAATTTAGTTTTTGCTACAGGCTTTCTACTTTTATAGCCAT
TTATAATGAAGTTCAAAATGAAGCATCGAACCACTAAAACTGTCCATTAAAATGATACAATACAAAGCCA
CATTTCAACCTATAAATACAACAAACGGAAAACCCGAAAAGAAGAAGGAAAAAGAAAAGGAACGTTGGCG
GCAAAATCTCAAATTTCCCTTTTTAGAATCTCACAATATCCCCAATCAAAAGCCCTACCAAAACCAACAC
AGCCCATCCCCAAATCGAACCCACCAACGACCCGGCCCCCGATCCTTCGAAGCTCCCCGCGTTCGTTCCG
GTGGTCAGAACCACCACCATCCAGTCGTCCTCCTTCCCTAGCCCCGCACCGACGAATCTTGAGTCGTTCA
AATACTTCGCGTACTGGGAGTGAGTGTAGTTCGTAAGCAGCAGAGTTTGGACTAGTTTTGGTACGCACAC
CGGCATCACGACGGCGTCGGCGGTGGTGTTGATATCTACCTTGCATTTCTCGGCGTAGTCCGGGAACTTG
GTGATTTGTGACGGTCGGCTTGCAACGACGTTGGATTTGGTGGTGGTGACAGCACAGGGTTGGTCTTCAG
CATCGTCGGCCATCTCGTTTGCAATGCAGTCGGCTTTTGCGTTCTTCACGAGCGGCGGGAGGTTTTGTGC
TTGTCGGTAGCTGTTTAGGCCTTGGAGAAGATTGTCTTCTTCATCTGCAATCAGGTGAATGGTAACAAAA
TTTACTCAAAATTCTCATACAGACTTATAATAATCAATATTGCTCCACGGAGAACAAAAACTAGATGGAG
ATTCAAATCCACTTTCTAGGTATGGTGCAATTTACAAGAATCTTTTTAATTAAGTTTATGTGGGAGCGTA
TTGAACGATCAATTATGAGATTTGTGATTCCACTGTTTTGGGGTCGGAAATGTGACTAGGATTTATGAGA
CTTAGAGAAAAAAATCTAAGAGGTGTTGGTTAGGCGATCAAGAACTCACAAGGAAAATGGGATCGTGAAG

TTTGAAGAAGATTCTCAGAGAACCTAAGAGGTGTTAGGGATGGGAGAAGTGTTGGGATGAGTGAGTAATT
GAGGGTGGAACGAACTTGAGATCGCCCAAAACTCTAAAACACTAAAATGCTTTGGGGGATCGTTGAAAAG
CATGAGTGACCGAGGATGAAAAGAAAATGTGATGGCAAAACGTGAAAATGGTTGAAAAAGAGGCTAGATG
GAAAACGGTATAATAGGAGGCTGGATTACCTCATCTAAAAAACTCCATCCGAACATGAGAGTTTTGTATT
ACATTATATCATAAACATATTATGCTATTATAATAACTCATCTGTCTCGGATGACCAAACACCCCTCAAT
AGCTCTACATTAAGAGCCCATTACATTCCGACCCAAACAGCCCCAAGTGGTTTCCCATCAAGGAAAAGAA
CAAGGAACAGAGGACTGGGAACCAAATATGTATATGAGGGCTACAAACAACCTATTATGGGAGCTAACAA
GTAGCTAGAAGCACCATTTTGGTCCTGTTTTCAAATTTTATTTATTTATTATTATATATATTTTTTTCATGT
AAGTTAACTTTAAGAAGTTATTTGTTTCCTGGTAATCTAAGATAGATTTTCAGCATTTGGATTCTTCCAT
TTGATTTGTAGGGATGTAAGACACCAAAACATATCAAGATACCGACATCTTAAGTTGCTCATACAGTAGA
ATCTAACCGATTTTGGGAACACTTCAAGATATCGACATCTTAGGCTCATTTTTAGACCCCCTTTTAAATT
ATAGACCAAAAAGAGTAAAAGTTGTAGAGTAAAAGTCGTTAAAAGAAACAGAGAACCAAATAGAAAGAGG
GAGACAGGGTGTTTACCTTTAGAGAGAACAGGGGAGAAGAAGAGGAGAGCAATGACGAAAACCCTAGAGA
CAGAAAAGAAAGAAGAAGAAGGCATTTTTGTTGTTATTATAAAAAGGGTGGATGAATGGATGAACAGATG
AGAGTGTGTGGTAAATTAGTATTATTTATAAAGAAAAGAGAAGAGGGAAGCAGTTGTGTTTTTGTGTCCG
GGTTTTGTTGTAGCAGTTACAAGTGGCCTCTTATGTAAGAGGAATTTTGACACCCAAAATAGACACGTTT
CTTCTTCCAAACTCTTCCGTCTGCATGCCTTTTCCCTCTTTCCATGCAATTCCAATCCAATCTCTCCGCT
TTCTCTATGTGTTAAAATACCATTTCCGTCTTCTACTTTCAATTCTTTTTAATAATAATTTTAGTTGATG
TGTTTCCATAGGTCTAATTTTAGTTTGTGTAATTTCAATAATTATCTTAAATTTAGTGTTTAATTGAATT
GTACTGAGATGGCTAAATAATTAAAAAAAAAAAAAAAAAATGCTTTGGTGTAGTTTGAAAAGCTTTTTTTT
TTTTAATTGTGTATGACATTAGCATGTTCGTAAGCCGGGATTTATTCTTATTGACGTCGAGTTTATTAAT
ATTTATGAAAATATTTTCTTATGTATAAAAGTATTAAGTGTTCAATTATAAGATGTTATGTGGTAACATT
TTTTAAAGTTTAATAATAACAATGTATGAAAATATAATATATGGATCTATCACTATAATTAATTATAGTG
AAAAATGTGTAACCTAAAGTATTGATTTCAAATATAATAAATTGAGCAAAACTATTTATAAATATAGCAA
AGCTTAAAGTTAATTAGTAAAATTAGCTTTGATCAAAGTACAATATCCAAAATATCCTTCTAAATTGACC
CAAAATCCAAAAATTCACTCGACACATGAGACCAACTTGTAATCTTTGGTCTAACAATTAAGGTGACTTC
GTTTGTGCTTGATCCATCTCTTATTAAATTGTACAAATGACATAAAATTTAATAGGGTTAAATATCAATA
AGCAACATAACCCAATTAATGTAATGTTTGTACTGTCGAGGTTAAAAGTTAGATTTTTTTTAATTTATTA
ATTTAACAAATTTGAAAAAGTAAAAATTAATTAATAAATAAATATAAATGGTATTAAACTTGCAAATACC
TCTTTAAAAAGTTATAAATTTTTTATTATGTGCCTAAATAGGCTTATAACCATTTCAATATTTTTTAAAA
TCCACGTACTTACCAGACATGAAGTAAAAATTAGTATGCATAGTTTTGTTTGTATTCGCAAAATTTAAAA
TGTTAGCATTTTTATTTCTAAAATTTAAACTTAACTTGTGTTTAACTTTTTAATTTAAATATTACATTTT
TATGCTTAAATTTTGAATTTAGTTTCCATTTCATCTATAGATTTGATAATTTTATTTTAAGTAAATAATG
AATTGTTTTGATATAAAATCAAGGGACAAAAACATATTTGCAGTTTTTGCATGGATGATACTCATTTAGA
GGAGAAAATGAGATTTAGTATACTTTTAAAAATAAAGGGTTTTTTAGCCCTTTACTAGACATCAATACTA
GGTGATAATGTGACATTATCAAAATGAAACCCTCTTGTGCACATTGAATGGCTAATGTCATTTTTGATCC
ATATGTTTAAAGTTTGTTTAATTTTATTCTTTATATTTTTAAATATCTAATCTTTTTTCATATCGCTAGC
TTATTATCAATCTTTTGGTTATATTTTTTTATTAGCATTTTCACTGTAAAGTGCGAAAGGAAAGTGAAAA
TTATTATGATTAAGTATTAAATTTAGACAAAAGAATTAATTTCGAGGACTAATTTTAAAATTTTATTAAT
TTAAAAGAGCTGAAATCGAACAAATTAAGAGAGAGAGAGAGAGAGAGAGAAAAAAAAAAAAAAAACAACGGAG
ACCAAAATGTATTTATTTTTAAGAAAGAGACGAGAAAGGCAATATTGTCCCACCATGCCTTGAAAGTTAA
AATTATCGACATGTCGTTTCGGATGGAGAATATTGTCCCACCATGTTTGGGTTGAATCTTCAGCCTGAGA
GAAGAAACAACCCAAACAACAAGGATGACACCAAGTAGAAATGGTGAAGACCCAACTTAGACAATGTCAC
GGCAGAATTAAAGCTTCCGGTGGGAGAATCGGTGGCGAAAGCGAACACTGCCCATTGTTCATAACACCCA
ATTCCGATCGATGTGAATTCAGGTTTGCTTAAGTAATTGGCCATTTGGGAATGTGTACATCCATGAAGAA
CAAGATTCGACACCAACGTTGGGATGTGAAGGGGCAAAATCAGGCCATTTTTTGTGTTGTTGAGCTCGAT
GTTGCACCTGGTTAGGTAGTCCGGGAGATTGGGGAGTTGGGTTAAATTGCTTGGAGTAATGTTGGAGCCG
CTGGTGAAGATGCTGAGGGGCAACTGATCGTCGAAGTCCCAAGCAATTTGCTTGACGAAGCAGTCTGCTT
TTGAGTTCCTATTCATGGGGGTAAGTTCTTTGGAACTTCTGTAAATGTTCATGGCTTTGAAAAGTGTGTC
TTCATTCACTGCAAATGATGGAGGATTGAGTGTGAGGTTTTATGTGGCAGCAATAAGGGAAATAACGTCT
ATGGATAAGGGAGAACTGTAATTGTGAGATTGCAAAAGAACCCAGCAAAGATATGGGACTACGGAACTAT
GTGTGTGAGAGTGTTTAGAAGCATAGAAATAGATAGAGAGAGCCATCGCAATGCACAGAAGAACCCATCA
AAGACTAGGAACTACGGAACTCTTGTGTTTCGTGTGTGTGTGTATATTGAGAAAGAGGGAGAGTGAACCA
TCACAGTGCACTAAGCTCGCCAATAGCAACAAGCCATGGGCAAGACCGCAGAGGTAGAGGCCAAGTTTGG
GGGTGGTGAAGGCCATTGTGCTGCTGTAGGGTAAAGGAGATGGCATCAGATCTGCATGGACAGGAAGAGT
CGAATAGAAATAATTAGTATTTGAGGTTCTTGCTTTCTTTAGTCTTTTCCTTTTTGTTTCTTCTGCCATT
GGGGCATCTTAGAATTTGCTTGTTCTGTTAATATTGCTTTGCTTTATTCATGAAGGGTATGGGGTTCCCA
TGTCCAACTTTATGTATTCATAAATGGTAACTAATGGAGTAAATGGAATTAAAGGTGCTCAAGGGACAAC
TATTAGTTGCTCAATCATTTTGCTTGTAAAGGCGTATACGTATAAAAGCCCTCTACTTCAGGTCTAAAAA
TCCCATCTACGTCAGGTTAATTCTTTTTTCTTCAGATTTGGAACTCCAAATACCTTTCTGAACTTTATCA

CTACTGGTGTGTTTGAACTGTACAAATGTGAACGGCTGGAATACATTATTTTTCATTTAATTAAGGCAAA
GTGTATTCTTTTTCTCAACGCATTTCAGTTTTCTATGTTATGCATAATGTTCCAAACAGTTGAAGTGATG
GACAATTCACAAGCAAACTAAAGGCGTTGTAGTTTCTATTACTTAATATGTGAATAGTTTAGCTTTGATC
TTTGTTATCATCGTATCTTGATGTGAGAGTAGAGGAGGGTACATGAATAAACTCACAACTCATTCAAATG
AGAGAGACATGAGGCTAGTAAGGATGTTTCATATAAGCTTCAAAAGATTGGTAGATACTCAGGTCGAAGG
AGAATCATCTAGGGTCCTATTCCTAGTTTTAGGACTTACATTGCTCTTCGTGGTTGTTTCATTGGGATTG
ATGATCTATTATCTCTGTTTTGAGGGCTTCCCGTTGTATTTTGAACTCCATTATAGTGAGGCATTGCGAT
TTGTCAGTTGCTTTCGTTTTAAAAGTTGTTTCTTACCAAATTTTTTTTAAAGAGGTATAGATTAATCATT
TAAAGGAATTTTAGGTAACAGCTTCAGAGGTTTTTTCCCCTCTTTGGACAAAAGTTGCTTCTTTTAGAGG
ATTCTTCAGGTGTATAGTCTCTCTCTCTGTCCATATATATATAATATATATACACTCCTTTGGCTGCTCC
TGCATATTCTTTTCTGAAGTTGGGGTTTCTTTTTTTGTTTCCTTTTCCTATTTCTTAGTTCACATGTTCA
CTGGTCCAGCTTTCTACGTGCTTTATATGATAAGATGTTTCTCTTGGGATTTGATACGAGTTCCATTTGC
CGTCATATTGGTTTCGATGATGAAATTATTTTCAACATCATGATGGTCTTCTAACCCAAATATAAATTAT
TTGAAAATATCATATTAGGTGGTAGTTTCCTATTCATGTTAGTGTAGAGCTCATAAGTTGACCTAAAGAT
GCACAGTTACAGAATACAAAGAAAAGAGAACACTTGTTTGCCCCCTTTAAATATCCTTTATATTAATATG
CTGATGTTCAGTGTAAAGCATTACATAACTCCTTGGGACAATCATGGACTATACATCGTAGATAACAATT
TTAAAGAATGAAAGAAAACAAAAGCTTTTCACCTTCACCAAAACAAAGAATTGACAAAAACAAAAATCCC
AGCCAATAAACCCAACAAGTATGTGGAACCAAAGATAGACCAGCGGAATAGCTTCCACTAATTGCATCCG
TACCCAAAGCAATCACTAAACAGTCTTCATTTGTAGCAAGACCTACACTTGTGTAAGTTGCATTATCCAA
ATACTTCGGAGCCTGAGAATGAGTGCAATTAGTAAGCATAAGTTTATCTGCTGAGTTAGGGACGTGTACT
GGTAAAATAACCCCATCTACTGTATGATTGAGGTTAACTTCGCACTTTCTTGAGTACTCTGTGAACAAAG
GAAGTTGGCTCTGGTTGGATGGCAAAAGGACGTCGCCCTCAATGGCAGCACTGCAAGGCAATTGACTATA
CCAGTTCTTTGCAATTTGCTCTGCTATACACTTTGCTTTCGAGTTCTGGGCAAGAGCCGACCTGTCTTTT
GAGTTTCTGTAGATGTTAAAGCCTTTAAAAAGCTCTTCTAAATCAACTGCAGGTAGTCAGGTTTGAAGTT
TAATTCATCAGTGTACGAAGATATGACAAGAAGGCCAGCCATTCGTCTTTCACTAAAGAGCTGTAAGTTC
AACTGACTTTATCATACCGTCTATTTCTTTGATGAATGTGCCAGGAAAGGTGACGGAAAAATCTTAGCTT
GAAAATGTGGAGAAAAGGATTTGTAAAGAAAAATATTTTTGGGCAACTCCATAAAACTAGTTGGAGAATT
TCAAGTTCTGTTTGCATGAATCATCTAGGGTAAGAATGTATTGTTTTGTGTGCAGTTAATGAGCTTTATA
AACTTAGAGAGAGAGAAAGAGAGAGAGAGCTAACCATCACAGTGCACCAAATGTAACAGAGGAAGCAAAC
AATGTAGAAGAGCACAAAGACTGAGGAAACATTTCAGGAAAGCCATGGCAGTTTCAAATCAGCGCTGGGA
GCAGAAATATGTGTCTGTCATGAAAAAGCAAACACAAAAAACAATGAGAATCAATCAGTTTGTTTCTCTT
ATTTCATAACCTTCCTTTTCTTTTTCGTTTCTTTTTTGGCTTGTTTGTTAAATTATATCTTCGTTTGTGT
GTTTTTGCCTTGGAAATGAAGGTAACAAAATTCCACTCCCTACTGCAAACATGTGAAACATGTTGCTGTC
CTTGGAACCAGGCAAAAAAGTTAAAAGGAAAAGAAAAAGAACAAGAACACGGCGAAGAAGAAATGGCTTA
TCCTCTTACGATATTCAAATGTGGGAAGGCGATATTTGAATTTTGGATCAACTTGTTCATTCAATTAGAT
TTAGTCATTGGATTAGCTCGAATTGGTTGTTTCTGTCCTTGAGGTCCCATCTGATCAATATTTTGTTCTT
TTTTAAATTTCGATTGTTTTCTCACCACTTTTCATGTTTTTCATAATCTACCAAATATACATTTAAATTC
TTAGTTAAATTATATTTCTAATATGCTTTTAGTTTTCATGTAGATGGAAGGGAAGAATTTTCTTTATGCC
TCTTCAACATATTGGCCAAGCAGCATCATTGGCTAAATGTGTGACATCTCCATGAAAATCTTATTCTTTG
ATCTGAAGCAAGAATGTAAAATGAGAAGGAATAGGCTTTCGTAAAGCTAAGCTCTGAGCATATCTTGGGA
AGCACATCAAGAAAAAGAGAAAAAAAAAAGAGCTGTACTTGCCTGTTGAAGCATATGTTCTTGTTATTCA
TTTATTTATTTTCTTTTTAAAATTCCAACTTTGCTTGAAGGCTCACGTTTGCGAATTAAAAGAAAAATGG
TATATGTCTCTACATGTTTGAATTATTCTGATGTATACAAATTTAGAAACTTCAACTTGAAAACCAATGT
TGAAGGAGATGAGAGACTGAATTAGTTTGAGTTATAGCTAAGAAATTTATTGTTTAGTATTAGTTGACAA
CTCAACTTTGTTTATCGATTTAGTCTGTGTAACTTATTTACAATGTTGGTACTTAACAAACTTGTCAATC
ATTGTATGTATCGTAGTAGAGTCCAAATCGTTCTAAATTTTGAACTACGACACTAATTTCATTTAATTTT
CACTTTATATTAAATTATTGCCTATTACTGCACAAGTGTCTTTAATGTAACTTGGGGAGATAATGAACTT
TTTCCATATGTGATGATCTATTAATTGTTACCTACCATGCTTCATTGGTTAAGACGAGATTTTGGCTTGT
AATTTTAATAAATGCATCATGACATATAATATAGGTGTAGATGATTAAATTGAACAATTCGTATCTAAAT
AAGCTTATGTTCTAAAAGATTTGATGTCTTTTGACTTACACAAATTGTAAATCAACCATTAAAAAAAGAA
AAGCATAAAAGATTTTTTTACAGGGACGACCCATTTTAAGAATCTAAAAGAAAAAAAAACCTACCTACAT
TTTAAAAACCAAGTTTCGTTCATCCTGGTATCACCGTCGTACTTGCTGACTCAACACTTGCACTTCTATT
CTCACTCTCACAACTCATTTTCATATATGGTTCCGTATATTTTAAATTTGGACAAATATGGAATGTTGTC
TTCAGAACAAGAATAGAAAATGAGTGGTGAGGGCAAGAGCAAAGAATGTGTCGAGCGATGGTGTCATAAC
AAGAATGAGTGTCCAAAATAGAGTTTTAAGACCTAGCATGTGTGCTGTATGAGTTATTATGGTAATTTTA
CCATGTGATAAAGCAAGTAGAAGGCCAGAAAGTAGAGTGTCATGAGCAGATAGAGCTTTCTACTCTGATC
TTGATTAACAATTTACCCTATAGACCCTATCCTGAATCTTGCCTGGTTGATGAACAAATAAACTGAAAGA
GAATTTTTTTTATACCTCTTATTAGGGACCGGTGTGAAGCATTATATCAAGAAAATTTCAAGTGATAGTG
CGAGTATTATACTTCATGAAAGGAAGGGACAACAGAAAAAGAGTTCGTTTCATTCAAAGAACACCTTGAA
GATTCAAGACAAATCACTGAACGAGATCTCTACTCCTTTTCCTCCAATCCCAGCAATGAAGCATTCAAAT

CCACATGACTCAAGCTCTGCGATTACTTCATCAACAACTTTCCCTGAAAGTACTGTTTCACATGGTTAAG
AAAATACAACAATATGTTTCTTCTGACGACAATAAGATGAATCCAAGCAGCAGTTTTCTTTTCTTATGGA
CTAGAATAAATGAATGCTGATATAAATATTATAAAATTATCCTCATTGGAGGGGGCAATGCATAAAATAT
GGAATAATGACACCATCCATGTTGAGTGCCTGAACTTCTCATATTGCTGCTAAGTTCAGGTTAAGTTATC
ACTTGAGTTCCCTAGTTTGTTTTTGTTTCAAGTTAGTCCCTATGAAAATGATAGAAAACAGATATTTTAT
TGACAGCATAGAATATAATAAACAAGGGAGAGTTGGGATCCCAAGTTAACAAGAGGACACAAAAAAGGTC
TTCAGTTTTCCTTGAAATCAAAAGAAGAGGAGTGATGACATTTACACTAGCCCCTATTATTTTATTTGAT
CATATGTTAAAGTCAAATATCTTAAAAGGACCTAATTGTTACTAGATACTAACAGTATTTAATGTTGCAC
GTTTTCTTAAGGTACCAGCTCAAGAAAATGTATCACATGAGAAACCTATCCACATTTGAAATCAACAACA
AAATTTAAGGAGATTTGACCAAATCATGGAGATCAACCCTTAAATAAATAGCATACTGAGAATCCTACCA
ACGAACTCCAAAAACATCAATACTCATAAGAAGGAAATAAAAAGGTATAAGTGAGGGCAGATGAAGAATC
ATACTATAAGATTCCATATCAGAAAAAGGATACAGTTAGGCAACAGTGTAAGAACACAGCCTCCACCTCC
AGCTCCTGTCAATTTGGAAATTAGCTTGTATTTCAAGCTAGTCCGGAGAACGGTTTCGATAGATGCATGG
CTTACACCCATACATTGAAGTAAACCTTGATTCATTTCCATCAGTTCTGCCAACTTCTCTTCATTCTCAG
TTAGGGACACCTCGTCGTGAATTGGTGACTGAATAAGGATTGATAACTCATTGCTGATAGAATTGACAGC
ATTGAACACTGATTTCATTGCATCAGGATGTCTAATGGCCCTTTCTGAAACACCAGCAACTAAAGCCTTT
GTGTTTCTTCCAACTTTTGTGTTTGTAATAAGCATTTTCAATGCCATATTGGATTTAATGAGAGTCAAAT
TTCCTGACCTGAACTTGATCATGCCCCCTGATACATAAGATTTTCTTGTATGAGAGAGACAAAACAAAGA
CTTCTGTTACTTTAGAACTCTAACAAACAAACAATTAATTTGATGTGAATTGTAATTTGAAACCGAATTC
AAGTAATGAAATTACACACACACAAACTAATAGAACGCCAGTATGGTGACTCTACTTTATTGATATTCAA
ACTTAATTACAATAAGAAATGAAAAGAAAATCAAAGAACACTGCTAGGACAGCAATCCTTCCCTAACCAG
AACACAAGACTTCCAACAAAGCAATGAAAACAAGATCAAAACAGAAACGTAATTACAGAAAAATAAACCA
GAGCGATGGAACCTGCTACTACTACTCCTCCTATCTCTCAAGGAATGAGATAGTCCTGATTTTCCTCCAC
AAAATTATGCACCCCTTTCTCTCTGAATCTTCCCTATTAAAACTCCCCCCAATCCTAGCTAACTGCTGGG
CCCACCATTCAGTTTCCCTAATCCCGCATTTCCCTCCATCTCACAGGCCAACTTCCCCTTATTTCTATAT
TTATATCATATGTGAAAAGTACCGGTGGCTTAACACTACTTTCCTCCACTAAATCCACCTTGTCCTCAAG
GTTAGTCAAGGAACTGTTGCTTGAAGTCATCACAAACTTCCCAAGTGGCTTTGTGTGGGGTAAACCTTTC
CAACTGATGAGCACCTCCCAATCCAGTGTACTAGGATTCTTCCTGTAGCCATACACTTCTTCTGGTTGTG
TCATCCATTCATGGGTTTCAGTCAAGTAAGGTTCAATCGGCTGGACTTGAGTATGATCCCCTAAAGCCTA
CTTCAATTGAGACACGTGAAAAACTAGGTGAATGGCAGCTGTGGTCGGCAACTCCAGCTTGTATGCTACT
GGCCCGTACTCCTCCAATATCTTGTAAGGCCCAAAATATTTCGGTGATAGCTTCTTATTGCATTTTCTTC
TTAAAGATACCTGCCGGTAGGGTCTAAGTTTTAGAAAAACCATGTCACCAACTTGAAAATCATTAGCCCT
CCTCTTCAAATTTGCATTCTTCTTCATTTTCTCCTTAGCCATGCATACGTGTTCTTTTAGGGCTCACAAA
GCTATGTCTTTGTCTTTCAGCTGCTGGTCTAATGCCAAATTGAGTGTTTTCATGTCCCCAATAATAAAAC
AAGGGAGTGGTAACCTTCCATATACCGCTTGGAAAGGGTGATGCCAATAGATCTGTTGAAAGTGGTGTTA
TACCAATATTTAGCATAGTGCAGCCAATTCATCCACTCTCTCGGCCTTTCTCCAGAAAAACATCTGAGAT
AAACCTCAACCCCTCTATTCACCACCTCGGTTTGTCCATCGGTTTGTGGATGGTATGCCAAACTTCTATG
GAGTTTCGTTCCAGCCAACCTGAATAGCTCATTCCAGAAGTGACTAACAAATACCCTATCTCGATTTGAT
ACAATTGATCTCAGGTATCCATGTCATCGAACAATTTCCTTTACAAACACTTCAGCTACTGATTTAGCCG
TGTAGGGATGTTTTAGGGCTATGAAATGGGCATACTTACTCATCCTATCCACTACTACAAATATTACTTC
ATATCTAGCCAACTTGGGTAAACCATCTATGAAATCCATAGATATGTCAGTCCATATAGTATCCGGTATC
TCTAGGGGGTGAGCAATCTTGCTGGTGATAAAGCGAATATATTATTCCTTTGGCAAATAGAACACTCCTC
ACAGTACTTCTTACTGTCTTTCTTCATTTTGTCCCAATACAAATCTCCAGTAATCCTCTTATAAGTTCTA
AAAAATCCCGAATATCTACCAAACACCGAGTCATGATATGTATGTAAAATAGTTGGTAATAAAGAGGAAG
TCTTGGACATAACTAACCTGCCCTTGAATTGTAACACCCCTTTATGTATGGTGAAGTTGGGTATGTCATC
TGGATGTTGCTCCACTATGCTTTTGATCTCCTTTAGTCTAGGATCATTCTCCACTTCTTGTATAATAGCC
AAGTCTATGAGGGCAGGACCAGTAATCTAATTTAGATGTACAGTAGGTGACACCCTTGACAATGCATCGG
TTGCCTTGTTCTCTAACCCGGGCCTATAAACCTCTTCAAAAGAATAACCCAACAATTTGACTATCCATTT
CTGGTATTGTGGCTGAATTACACGTTGTTCCAGCGAGAACTTAAGAGACCTCTAATCAGTTTTCACGATG
AACTTCCTTCCCAATAAATATGGTCTCCATCTCTGCATGGAAAACACCACAACGATCAATTCCCCCTCAT
ATTCTGGTCTGGCCCTATCTCTTGTGCTCAAAGTTCAGCTAAAGTATACAATCGGTCACTTAGCTTGAGT
CAGAACAGCCCCAATTCCATATCCAGATGCATCTGTTTCGATTTCAAATGGTAAGTTAAAATCAGGCATG
GCCAACACGGGTAAAGTCATCATGGCCGTTTTCAACTTCTCAAAAGATTCATTTGCTTCTTCATTCCACT
TGAAAGCTCCATTCTTCAATAGTTGGGTAAGCGGGCCTGCGGTACTGCCATAATTTTGCATAAACCGGCG
ATAATACCTAGTCAAACCCAAGAAACCTCTTACCTCTCGCATGTTGTTGGAGTTGGCCATTCTTTAATGG
CTCTAATCTTCTCGGGATCCACCTCCACTCCTTTTTCAGATATGATATGTCCCAAGTAACCGACCCTCTC
TTTAGCAAAACTACATTTGGCTAGGTTCGCATACAATCCATTTGCTCTCAAAATTTCCAGCACCAATTCC
AGATGTTGCATATGTTCTTCCAATTCCTTGCTATGACTAAGATGTCGTCAAAGAAAACTAATACAAACCT
TCTCATATATGGTCTAAAAACCGCATTCATTAAAGCTCGGAAGGTAGAGGGGGCATTAGTTAAACCGAAA
GGCATGACTAAGAACTCGTAATGCCCTTCATGAGTGCGAAAGACCATCTTTTCCACATCCTCTTGGTTCA

TTCTGATTTGATGATAGCCTGCCTTCAAGTTGATCTTGGAAAACACATTGGCACCGTTCCATTCATCAAA
CACTCTTCAATGATTGGTATAGGAAACTTGTCCAATATAGCGACATTGCTAAGTGTTCTGTAATCGACAC
AAAACCGCCAACTTCCGTCTTTCTTCCTTACCAATAGTACAAGACTTGAGTATGGGCTGGTACTGGGTCG
AATAATTCCCGAAGCCAAATCTCATCAACTAACCTTTCCATCTCTTCCTTTTGTTGATAGGCATAATGAT
AGGGTCTCACGTCGACTGGATTGGTCCCTTGTTTTAAATGTATATGGTGTTCAATTCCTCTTTTGGGAGG
CAAAGTTTCAAGCCATTCAAATACATCTTCAAACTTTCTCAACAAAGGTGAAATAGCATTGTCAACTGTA
AGCTCACTCTCTTCCTTGTACAACTCTTCCATTGCTACATTCCCTTTAATTGCTTGGCATTCCACCAAAA
ATCCTTGGTCTTCACCCACCCATGATTTTACAATGCTCTTCAAACTCACCCCTTTCTTGGTGAGGCTAGG
ATCCCCTCTAATTATCACATTTCTTCCCTCACACTAGAATGTGATAGACCCCTATCATCCAAACATAGGG
TAGAAGAAAGAAAAGCAATTAAGTTTACTTGTAAGTCTGATAACAGAAACTATTACAATCAATTAGCTAA
GTCAGTTAGGATTTCTGTTAGAAAAGTAGTTGGGTCTGCATATGATAAATAGCCATAGAAGACGAGTAAA
TAGGATGGAAAATCTTATGAAAAAGTCTTTGGTTAATTCTTGAGAGATAGAATAGCCAAGAGAGAGAGTA
TTACTTGTAATTGAGCATCACTATCAATAAAAAGAGATTGTGATTGAATACCAGAAAGAGTTCCATCAGA
ATGTCATTACTAAATGTTTCCAATCCACCTCTGTTACCCCAAGGGAACATAATCAATGCATTTCGAGAAT
CACATCAACTCCCCCAAGCTCCAATGGTAAGAGGCTATCCACGACCTTCCAGTCTCCCAACAAGACTTCT
ACCTTTCCACAAATTCCCTTTCCTTTGATAGCTACCCCTAACCCCAGAATAACTTTATAACTAGATGTAG
ATTTCAATGGCAGATTTAGGTCAGAAACTAATTTCTCAGATATAAAATTGTGGGTGGCCCAACAGTCTAT
CAATACTACCACATCTTCATTCTTCACTTTACCCTTCACCTTCATAGTACCTGGGTTTGTCAGCCCCACT
ATTGAATGAATGGATTAAATTCTCTACAATACCTACCTCAATGGTGTTTTCTTTAGCTACTTCGCCTTCC
ACTTCTTCCTCAATTATTTCAAACTCTTCTCCATCTTCTCAAACTACCAGCATCTTCAATTCCTTGTGTT
CTTTAGCCTTACAACGATGCCTTGCATAATATTTCTCGTCGCATTTAAAGCACAGTCCCTTTTCTCTCCT
AGCCTGAAATTCAGCATTCGACAATCGTTTTGAAGGACCCTCTCTCCGGTTATCCGCCGTTGCTACTCCC
CTAAGGGTGATCGTCCTCATTGGAGTGGTTCACCAATTGTGGCTGTTGCTGATTTGGTTTCTGTATTGTC
CTTGGCTTTGGGCAGATTATATTGGAATTTACTTCCATACTCACTAATAAGCCCACATTCCTTCCTTACT
CTCTCCCTATTCTCAATCTTTAAGGCTAGTATCATCATTTGGGCTAGCCCACGAGGTTCCAACACTTCTA
CTTCAGTCTTTAACCATGGCTAAGCTCATTCATAAACGCTCTTCTAGCACCACTGTCTGCAGAAAAGCCA
TCAGAGCTAAGTACTTGTCGAACCAATTCCGATATTCCCACAGTGGTTTCCTGTTTGATCGTTAGGAACC
TTCCCACCAACGTGCCATCCCTAATCATTTGAAACCTTGTCAACATCTTCTCCTTCAAGTCATCCCAACT
CTTAAACGATTCCTGCTCATCCTGTGATCGATATCAGTTAAGGGCCGGACCATCGAAGCTTATAATGGCT
ACTGACATTTTCTCGGATTCAGTGAGGTTATGGATTTTGAAATAACAGTTTGCTCTGAATAACCAAGAAT
CGGGATCCGTTCCGTTGAAGATAGGCATCTCGACCTTCTTAAATTTGCTCCGATCAAACGTCTTATTGTC
ATCATTCCTCCGATCAGTTTTCGGCTCCCCTGTCATCATCTCAATCGCTAAATCAGTCCCCGTCGCCACG
CTCGGAGAACCTTCTGTTTTGATCGCTGTAGCTGCCTTGTCCTGGATGATTCCCTCAATATATTTCAGAA
TAGTTGTTGTAATTGTTGTTTCTCCATCTGCATGTTAATATTCTCAATGCTCTTTGAGATCAAGGACATA
TTCTCTTCCATTGCAGGTAACTTCTTCATCTCTGTCCTCATCTCCAGAATCTCTTGCTCAACAAAGTCTA
GTCTTTCTTCGCTCTTTTTAGCCATCTCCTTGCGTTTGCCCAGATTCAGTTTCTCTGATACCAATTTGAT
AGAACACCAATATGGTGACTCTACTTTATTGATATTCAAACTTAATTACAATAAGAAATGAAAAGAAAAT
CAAATAACACTACTAAGATAGCAATCCTTCCCTAACCAGAACACAAGACTTCTAAATGAAAACAAAATCA
AAACAGAAACGTAATTACAGAAAAATAAACCAGAGCGATGGAAACTGCTACTACTACTCCTCCTATCTCT
CAAGAAGTGAGATAGTCCTGATTTTCCTCCACAAAATTCTGCGCCCTTTCTCTCTGAATCTTCCCTGTTA
AAACTCCGCCCAATCCTAACTAACTGTTGGGTCCACCATTCAGTTTCCCTAATCCCACATTTCCCTCCTT
CACCTCACATGCCAACTTTCCCTTATTTCTATGGTTATATGTGACAAGTATTGGCAGCTTAACACAAACA
CATAAAAAAATTGGCGCAATTTGATTAAATGGTCCAAGTAACCCAGTGATTGAAACATATTATTCAGAAC
ATCATAAACTTATCGTACTTGATATTTCCATTCGGATAGCATGCTATCTCTGTACTCTGAAAAAGTTCTA
TGATACAATTGGATTACTTTTTGATATTCCTAAATCTTAGCCCTTGACACATAAGACTTAATCAGCTTTC
ATAGTGGTATAGAGTAAAGTCAGTTCTTGAGCAAGAACGCCTAGCAGATGCAAAGTTAGTTCTTAAACAG
ACCACTTTGCTTCAGAGACAAATAAAATCCAATAAAGTACTGCAGTAGCCAACAAAAAACTACAACCTTC
AAACAAGCAGTTGAATAAAAGTGGAAGATTGAAGAAAAATGGATTTTGCATACCATATGTGCTCACTGTG
TTGTCTATTCCAGAGGGTTTTCCATGGATTATCTTTTCACCCTCAAAGGCCCACTTGTTCAACAAATCTA
GTTCATCTTCTTTATATACCATCCATCCATGATGCTCCCGATCCACGTTCACAGAACCTGATAAAGCAAG
CAGAGCAGCTGATAGGGCAACGCAAAACGCTGCTGATGATCCCAAGCCGGATCCAAGAGGAAGCTCAGAA
GTGATGGCCACCTCAACAGGCACAAATCTGAAACACAAGGGAGCAGACACTGTGAAATGGAGACTGCACC
GATTAAAATCTGTCAAACCTTTCATGAGTGAAATACATACCCTAGGATGGAAGAACAGAGCCAAAGAAAT
GCTGCCACTCCAGAAGCAAGTCCAATTTTCGCCTCCGGAATGTTTTGATCCTCGACCAGAGATGCAATTG
ATTTCAAGCACTCTGCAGGGCATGTTGTGGGTGACGAGATGGCACCAACAAATACACCCAAAGCTTCCCT
GACTCTACTAACTGGCCATGAAAACTCAAGTTCCAGGTCCTTCAGATGGAGTTTCACAATATCATTCTCG
TCTGATAAGAAAGCATGTAAATTGATGAGGAAAAGGGAGTACAAAATACTAGAGGGATGAGATGTTCATA
AATTTTTCAATCATGAGAAGCAATCTGTGACAACCTATTAAACCATGAGAAGCAATAAGCTACTAAACAA
AGGCATATAGCTGTTCTGACACCTTCTACAGCAACAATTGTGCATCTAAATTCATTTCTATCTTCACTTT
TTCTTGAATGCTAAGAACAAGAGTTTCAAGCAAATTCACACATGCAAATCAAGTAACACCAGAATGTGAA

TATGGTTGTAACATGAACAAGAAAAATACCCAGAAAACTGAAAATTAGAACATAACTACAAAAGGCTACA
ATGCTTTCCTACCCAGATTAATAACCCATAAAATGAAATGGGTTCATTGAATCAAAAACCCATAGAAATA
TCATCAATCATCAATGGGTATCCAACAATAAGATCACCCAAATGAAAGACACTGACAATCCACAAATCCA
CCTTCCAAATCATACGTTCAGTACCAAACACCCATATAAAAGTCAACCCAAATAAGAGATAGAGAAAGAG
AGAGCACCTGAAGAACTTGGCAATCTAACTGAAGCTGTAGTGTAGAGATCAACAGAAGCGGCAACGGCGG
TGGATCCATGGACAACGGCATGTTCACCGGCGAGTATGATTTTTCCGGGAGCTCTGGCTTTGACTTCCAT
TATTTTCTAAAGAATTTCAATAGAGTTTTCTAGCTTATTCTGGGTGTCTCTACTCTGTGTAGCACAGCAT
TCGAAGCAAATCGTTGAATTGACAAACTGCCGCGTCGATCATTGAAACCAATATGCTTTCAATAATATTT
TTTTCCCCATTTCTTTTATTCTCCTCATGATACATGTGACTTCCTTGCCCAACAAAAGCTTCAATAAATG
TCAAAACACTGCTTCGTTGTTTTGTTTGTTTCCCCGAGAGATCGTTGGTTTTCACGATGTGGGTATGAAT
CTACTTCAGATGCACTCATTTCTAAAATGCTTTTAAGAAGGAAAAAAATAAACTTTGTTGGTTTAACTTT
TGGGTCTAATTTAATTGTTCTTTTAAACGATCTGATTTATAGTATATATAAAATTTGGAGAAACTTTTTT
CTATGCATTATATTTTTGAATTAATTCTATATTCTAAATCTAAGTTTTATGATAAATTTATTTTTAATTT
TTCTAATATATATAATTATAAAAGGTACATCATTCTTGAATTAATTATATCTTATTGGAATTAAAATATA
CACAATGTGAATGCAAATGGTTTTTCATTTACCCACTGTATAATTAAAGCACGGATGTGAAGACTTGAAC
TATCTTATTAATTCTAATTAACAATATACATTTAAAGATTTGAAACTTGAAAAATTTTTGTAAAATTTTT
TCTACTATATAAAAGATTCCATTGATTTTTTCTACCAAAGTTGTAATTGAAAACTTTGTATCTATCAAAT
AGATTGTGTAATGGGACAAGATTGATATGTCGATCATTTAACAATAATGTTGAAATAACAATTGGCAATT
ATGTAGTAAAACAGATTTTTCTTCCACGAATACCACTAAACCGCCAAACAATAATAGATATCCATTCAAG
TTCAAGAGAAAATAATTTCTCTTTCATTATGCTTTGTAAGGGCAATTAACAAAGCACAAGGACAAACAAT
TTCTAATGTTGGAATATACCTTCCTAAAAAATGTGTTTTCTCATTAACAACTTTTTGTTGCACTATCTAA
AGAAATTTCAATGAAGACAACAAAAGTTTGGATCAAGTCCATAGACAACAGTGATCAAATTGTACAAAAA
ATATTGTTTAAAAAAAATCATTTTTTTTATACATGAAATCTTACATTTAGATTCTTATATTTTAATCTAA
TTATTGTAACTTCTTCCATATACATCTAATTTTATTACGTTTTGAATTTTGTATCTTTAAATTTTACACA
ACCTAAACATACACACGCAATGTTTACTATATTGCTACACATTATATGTGTCATGCACATGTCTTTCACT
AGTATTATATAAAAGTCTAAAAGCTAAGAACTACGACTACCTATAAAATTGTTCCGACACTTTTATTTTA
ATAATATCAAAATTTAGATGAAAATTAGTGTAGAGTTAATCCAGAACAATGCGGCGACTCGGCCAGAAAG
GCTTTGATGACTCAACAATTTTCATTTTGAAGTTAAAAAAAGTCTAATCGAGTTTTGAACGTTTATTAAA
GTTCATTAGTTGTAATGTAACTTTCAAAATAACGCACATATGCTCTAAACAAAGGATAATGCGGACATGA
TGGAGCGGAAGGATGCCTCCTGGTGTGCGAAAGAGTTTTGGACAGTAAAGTGCTCTATGACATCACCAGA
AGGCTTTGTTTAGGACTGGGTCATTCCATATTAACTCGTCATCTGTACACAAATTCCATCCATTCAATGT
CTATTTAGTTTCTCTTCTATACAATTAACTCAAATCGATTTTGGCTAAAATCAAACCTGAACCGACCGTT
TTTCCATCTGTGTTGATGGCTGATGGCTCTAGTAAGCAGCCAATCTCAATAAGAGAAGTATTAATATGTA
GTTACAAGATTCTCAAAATTCAAATTCAACAACGAAAAGTTAAAGCAGACAGCTTAACAGATGCCAACTT
TTAATTATATTCGTACTGAAAGGTGGGGAGATAACACAAGACTTACTTTCACAAAAATAATTAAAATGCG
TAAGAGCTTGTTTAATCATCATCAGGGTCATTGTCAAAGATATACAAGTATGGAGTTACAGATTTGAGTC
CAAGATCTTCTTCGTTAGCCACGCTCAGCACTATAGGCTCGTTCCAACCCAACCGTTGCAAGTAAACTGA
ATCATAGGCTTCAATGTTCAAACCTGAAGCAAGAAATAGCTCCAACTCGAGCATGAATCGATCAGCTCTT
GCCGAAAGGAATGGCTTTGCAGCATCAAGAATCGTTTGGCTAAACCTCTTCCTCTTCAGTTCAGGAGTTT
CTGTTTGATACTTAGGTTCATTTCTTTGAGGAGAAAAAAACAAAGAACAAGAAGCAGATTTCAGAGAGTA
ATCGGTTATCATATCTATGCATACTTACATTCATGTGGATAGGGGATAGGTCATAGTAAGAGAGATGAAT
GAAAATGAGTTTTGAACAATTCTACATGTCAAGCCAAAATGAATAGCATTGAAGAAAAAGAAAAAAATAT
CAGCCCCCGAACTCTCTAGTTTGTAACAATTTTGTCTATCTACTTTCAAATGTGTAACAACTTAGTATTT
TAACCTTTAGTATGTAACAATTTAGTCTCCGTACTTATCATAATGATTTAGTCAGAGTGTCATTTTTTAT
TAGGTAACATCTTAATCTTTGTAGTTTATAAGCACATGTGATCCCTAACTAGTTTATCAAGTTGCAAATA
GAACAATCTGATCTAAAACATTACAAGATACAGCACAAAATGCAAAAGAACTTGTAGATATAGCAAAATT
TAGATAAGCTCTCAAAGCCTGTTAGTGATAGATTATATCGTTGATAAGAGTCTAGCATTGATAAATTTTA
TAATTCTTTAAAAATGTTATTACACACTTAATTATTAACCCCAAAAGTGCTACTGATTGCATATACTTGG
GTAAGAAATTCCATTAATTTTTTACGGTACTTATATGGTATGGACACTATATTATAAAAACTGCTCACAG
GGACTTATTACAAACTAAAACTTAGGGACTAGCCACGAAATTGGTGTTTTTAACCAAATTTTTATTTGAC
CAAAGTATAGAATGTAATGATGGATGATTATTAGTATTACCTACGAAAAAATGAATTAATTAAACCAACA
AAGTGATGCATAATTATGTCAACATCTTCTTCCTGTAAAAGTGAATAAAACTCTATCAGTGATCATCAAC
AATGAACTGTACTACAGAGAAACAGAATAGATAAATCAAGAAACTATGCAATAGAAACGGTAACATAAAT
CTACCACCGCTATCTCATTTCGTCTACAGTAATCCCTTTTCTACTAAAACAGAGTTCTCAATTCCTCACA
GATAAAGGAAGTTGCACATCTTTACCTAATCTTGTCTACTTCCACAATACACATAGGTTTCCTCACCATT
GAAATAAAATAAAGTTCTTAAATCCTCAATCAACAGACAAACCCCCTCATCCTTAGAAGGACAACCCATC
CCAAAGCCCACTATGCCACAAATGGGAGAAACTCCATCGCTGTCCTACCTTTTTACGTGCCTATGTGGAG
GGGAGATTCGATCTTCGTCCAGGGTGAATTTCATTCATTATGAGCTCAAAAGTGTAAGCCTGATTAGTGG
GGTCTTAAAAAACTCACTAAAAAACTCATGAACTCAATGAATGATCGGCCATTCCATATATGCTTGAAGC
AAGTAGGCGACACTAGTCAATGATTTCTATGTTTCAATAGGATACCAATTACTTGGATAATTGGATGGCT

TTGTTAGAAACTATTGGGATGATTTGCAGAAAAAATACTTTCTAGGTTTGTCTTGTGTCTCATTAACAAA
TGGTCCATTATTGATGGATCAACAGGGACATTTTGTAATTAAGAATTTCATTAACTACTAGGAGCAATGA
TAGAATTCATTTCCCCAAGAATCCGGCTGTAGGAATGAAAAGCTCCCTCCCTTTACCATCCACCTATTTG
GTCTTGACAATTGAGAATGTTGTTTGATATTGGAGGCAAACAATTCTCCAAGGAAAAAGGGGGCCAAAGG
AAGCAATATCCCATAGAAGTATAAATGTGATGTAAGAAAATAAGACACTAACCTGAATCAAAGCTTGAAG
TTCCCTCTTCAACCAAACTTCAAGCCACTGGTTTGCCTGGAGATACTTCTGTAACTTCCAATACCTCTGT
ACATCAAATATGTCATTCAAGAAACCTACATGCAGACAATTAGGGAAATTCACAAATCCAAGTAACCAGA
TGTAAATGTTGGAGCATCATCACATAATAAGTTGAACGATAAACAAAAAACGACCTGGTTCAGTATAGTA
GCACTGTAATCTATACTTATGAGCTTTTGATAAGATAAAGCTGCAAATAGCATTAATAATAGCCGTAGTC
AATTAAGGTAGAGAAAATAAAAATAGAAGAACTAGGGGAAATGTCAACACGAAGATACCTATCCTGGAAA
TCTGGATTAACATAATGTCTTTGGAAATGGTGTCCATCTAACCCATGTATTATAGATGAACTTTCTGTCT
GGTGATTAATTATAAGAAAATAAGAAATGCAATACTAGAAGTATGGAATGCTAAAGTAAAACAAGATTAA
CGGGGGAAGATAAATTGAACCTTGCATAAAGGACATTTAATGGAGGAAAGTCTGCAGGAGCGCTTCCCAG
AAACCACTTTGGTCCATTGTACAATGCAATTGTAACAAAAGTTATCTGCAAGACAGATAAATAGAGTTAA
AAGATAAAACCATTCAAACTTCTCGACAAACTTTATCGACATTCCTCTTTTTCTAAATGGTACGTTGTAC
TCAACATGAATGAACAAATAGACTCTTTAGAGTTAACTAAATGATCAAATTCACCAAAACTTTAAGGTTT
AAACTTTAGAAATTACTGGTAATCAACATGATACCAGACTAATATTCATGCCTAGACATAAGCAAGTATA
TTAAATTTAAATTAAATGAACGTATTCATCTCAAAGATAGTAGAAGAGTTCATCAATGATTACATTCAAC
ATATCCAAGAAAACTAACGCATTCTCGCTGCTGTTTCTGTTTTCCATTTCATCAAGAAGATGATGCCATA
ATGACACTCAAAATTCCTTACTTAAAGTATACAGCCACTAAAGAACTGTGTATATTAAATCAAATTAAGC
ATCTCCAGAAGAACTTTTTTTTCCGCTCAAAGCTAAGGAATTAGCTAGTTCATCTCTATAACAAATGAAT
TCAGTTAATCATCATACAAGACACTATAATCATCAAACAAGTTCGTTCATCAAATACGAATAACAGTGGC
AGAAACCTTGATAATTGGGGGAAAAGCTAAAAGCTAAAATCTCCGAATAAAAGGAGAACAGAGTGATTCT
CAGTTCATCTGTTGATACAGTAACATGTAAAGGATTATTTCCAAATGGGTCACTACAGGAATCGGAAAAA
AGAAACCCCGATTCATAAAAATGAATAAATTTTCTAAATCAATTTCTATTTAGATGACAGGAAAATTCAA
GAAAAACGAAAGAAAGATCAGATTAAGAGAAATAAAGACTTACGAAAGCACTTGTCGAGATAGGAAGATT
GATTTATGGGTCCAAGGCAGATCGGGCAAGGGTCCGTATAGTCCGAAGTGGAAGTGCTTGTCTTAATCTC
CATTGACGCAGCGCTCTGCAATTCCATGGACTTCTCCGACATGGGTTTACAGTGAACGACAGCTCGATTA
AACGACAAGTCGACTTTGAATATATACACACATAAATTATATTAAATTATAAATTTGCCCCAAAATTTCC
GACTTTTAACGGAAGGAAAAAACTTCTAAATTTTGTTCTAAATTATAGTTAGATTAAACAGGTTTTTTTT
TTAAAAAAATAGAACAAAATCCAAAAAATATCTATTATCTCTCTTCCTATTTGGGATAGACAACATCTTT
TAACTATTGATACACGATCATTTAGATCCTATACGACTATCATTTAAATACTAATATCATTTAAATCTTG
TACCAAGAAAAAAAAAACAAGATAGAAATGAAGAAGAAGGAAGGAATTATGGAAAAAGAAATGAAGAAAT
TAATAAATATTACATAAATAGTCGTAATATTATAAACGAAAGAAGCCGTAATAATATGAAAAATTAATAA
TGTAGAGAGAAGATGAATAAATCTTAAAGAAAAATAAAGAAAATGAAGTGAAAAATGATCCGCGTTATTC
AAAGATATATTTAAAATTTATAAAAATAGTACGAACTTTCTTATTTTGTTATCTAAAACGTAAATATTTG
GGTTTATTATTATATTTATAAAAATGAACTTAGATTAAATTATAATTTTGTCTCTAGAATTCCAAAGTGT
GTGATATTTGGTTTATTTTTTTGCTATATTTCAGTAACCCTAAATTTCAACCTATTTTTTTGAAGAAAAA
ATTGCCTAAAAAATTTCAATTTTTTTGCGAACTCCAAACGCCAAAGTATGTGGATCTCAACGGTCATTTA
TACGTCATCTTCTCCTCTATCTCTCCCTCCCAAAAGCAACGCATCAAACTAAAATGCACACCACAGCCAT
AGCTTCTTCTTCAAGTTGCTCCATTTTCTTCAACCCTCTTCTCAATTTCACCCTTTCAAATCCCATAACT
CCACCCAAATCCCCATTCATTTCGACCCTTGTAGCAGTTTCCGATGGATTGCAGCCTCAAACATTGACCA
TTTGCGCTAGAAAGAAGAAGAGAAGTCCTGGATTCCAGAGATCAACCAAATTAGTGCTTGAATTGGCTTC
TCTCTTAGCCTCCAATATCAAGATCTTACCCCCACCTCTGGATTTAGTCGTCGCGGAATTGAGTGGAGGA
GATGGAAACGGAGGTGGTTCGAGGCTGTGGAGGGGTTTTGGTGGAGGTGACTATGATGGATGGAGAGGTA
AAAGGAAGAAGACGCCATTGTTTATTGGGTTTTTGATGGTTTGTGGTTTGGTTTTGTTGTTTGTGACTGA
TTTTGAGATCAATGAGGTTTGTGGGATTCTTGGATTTGCTGTATTCAGTGTTGCATTGATTCAATTGTGG
CAGAAAATCGGGATTTCGAAACATTTTGTTTTGGGGTTTGGCCTTTTTGGCATTTTGATTGCCTTGGGAT
TGAGAAGATCTGAAGTGCAAAAATGGGTTAGGAAATTAGGGTTTTACAGTCCAAAGATGAAGAGTTTGAG
AAGAAAACTTAAAGGTAGGAGAATCTTCTGAGCGAGCTATTCGATAAAGAATATGTTATCTAATAAGCAT
TTTTTTTCTTCGAATGAGATTATAGCGAGGGATTTAATAGATATATCCCCTTCATATCTTTAACATAATT
CTTACTCGATGGTTCACTTCCATAATCTTCAATCTCGCATTTCTGATTGAGAACGAAGATAATGAACCAG
AGTTATTACACTATTTTGAGTTTCTTGGAAAAATGCAAAACCATGAAGGAGTTGAAACAGATTCACAGTC
TGATGATCAAAACTTCAGTCGTCAAGAACATAATCCCCTGCAGCAGACTTATTGATTTTTGCGCAAATTC
CGAACTGGGTGACATTGAATACGCCAGAACAGTATTCGATCAAACTGATCAGCTAACTGTCTACGTCTGG
AACTCGATGATTAAAGGGTACTGCAATGGCGGTGACAAGTTTGGAGCTCTGTTTATGTATGAAGAAATGC
AGCGTAAAGGCTTCTACCCGGACCATTTCACCTTCCCTTTTGTGTTGAAAGTTTGCTCTGCAATCGATCT
TCTTGTGTATGGACAAAGTGTTCATAATCGAATCGTGAAAACTGGGTTTGAATTAGATGTGTACACTTCT
TCTTGTTTGCTCAATATGTATGTTTCTTGTGGAGATTTGAATTCTGGGCTTAAGGTGTTTGAGTTTATTC
CCAAATGGAATGTGGTCGCTTGGACTTCCTTAATTGCAGGGTTTGTGAACAATGATCAGCCCAAAGAAGC

TTTGAGATTGTTCAAAGATATGGAGAATGAAGGTGTGGAGCCTAATGAAATCACCATGACCACTGCCTTG
GCTGCAGCTGCTCGTTGTAGGGATATCCATACTGGGAAACTGGTCCGCTATCGTCTTCGCCAACTTGGTT
TGGATCCATTTCACACAAACTCTCGCTTCAATGTAATACTTGCAACTGCAATCATGGATATGTATGCAAA
ATGTGGCAAGTTGGTGACCGCACGGAATCTGTTTGACAAGATGCCTCAAAGAAACTTGGTTGTTTGGAAT
TCAATGATCAGTGCTTTTAGTCAATATGGTCGAGGAGCAGAGGCTTTGCGTCTTTTCGTTGACATGGAAC
TGGCTGGGTTTGTTCCTAATAAAGCAACGTTTTTGAGTGTGATAAGAGCTTGCACCCACCTGCGGTTTCG
ATCAACAGGACAAAGTTTACATGCTCGTGTGTTGAAAGCAAATTTCCATGAGTTTGTTGCCATTGGAACT
GCTCTTATGGACATGTATGCCAAATCTGGAGATGCAGATACTGCATTGAAGATCTTCAGCAAGTTGAGGA
AGAAAGATGTAATGGCGTGGACAACGATGATATCAGGCTTAGCAATCCAAGGCAAAGGCAAAGAAGCGCT
GAATGTGTTCAGAAGAATGGAAGAAGAAGCTGAAGTTGCTCCTGACCAAATCACCTACATAGCAGTTCTA
TGGGCATGCAGCCATCTCGGGCTGGTCGAAGAGGGTCAGAAACATTTTACTTCCATGATGGAGGTTTACG
GTATTGAGCCTACCATGGAGCATTATGGCTGCATGATTGATCTTTTGAGTCGAGCAGGCCACTCCGAAGA
GGCCGAGGAGCTCCTGGTGAAAATGCCAACGCAGCCTAATGCAACTATCTTGAGTTCTATTCTGAATGGT
TGTGAAATGTATGGAAATGTAGGTCTTGCCAACAGAGTGAAAAGTCACATAGTAGAGTTGAAGAATTCTA
GTAGTGGTGTTTATGTTCTTTTGTCAAATATTCATGCCAAAGCTTGTAATTGGGAGGAAATGAAGCTGGC
TAGAGATATTTTAAAGCATAAAAAAATTGGGAAAACACTTGGGAACAGTTTTGTTGAAATGAACTTACTG
CCTTGGTAGCTGATGCATGTATTGGATTACGAAAATTCCTTCTTGTTTAAATGAAAAATCAAATATCTGA
TACGTTATTAGATGATTTTATTATTTAATTCTATTGGTCCGTGCAATTATTGTGCAGTATTGGCAATGTC
TACATGGAAGATGGTTTATGATAGAATACAAGCAGTATTGTGCAGCCACCTCCAACAATTTATTCTCACA
AACATAATCTCTTTGAAAAAAAAATCTAAGAGTAAATAATTTTCTCTCAAACGGCAGCCAAAATGCCACA
GTACTGACATCAGAAAACACGGATAAATGCCTGGATGTAGCAACCTCCAGGGACAGGCACAAATGAGTGC
ACCACATGTATAAAAAAAAGGCACGAGTTTAATGTTGATTGATGTGCTTGGACTATCAAAATCTGTGAGG
CCTGGTCTGCTTTCAAATGGATATTACGTGATTTTGGACATCTGGGAGTATACATGAGGAAGACAGAGAA
CAAAACAGCAAAAGAAAAAAAAAAATCCTTAGAATAGAGGAGGGTGTGACAAGAGGCCACTAAGGCGTCC
AAGAGAAATTAGATACCGTGGGTTGGAAGCCAAAACAAAAAACAAAATTGATCAAATTTAGTGTTAATTA
TAACTCTGACAGATAATTTGATTTCCAAATCGCTGACATTCCCTCGTATATTTATGGGATTTCCTCAGAA
AACAGATAACCAGAACAACACAAGTACCGTTATGAAATATGAGGTACGCATATTTGCCATTTCCTTGTAT
CATGGCCAACTTCATAGAAACAATAACTCATTTATTTATGTTTTAATTCAGTATATATGGTTTTGAAAGG
GAAATAAAAGGATAATATCGGCTATTTAGCTTGAGAGTTAAAACAAAAATCAATAAGGAGAAGCAACTAC
AAACATTGACATGGACCAAGGGAATTTGAATGCGTCTTAAAAAATATAAAAATTCCCTAAACATTTTCTA
GAAGTAGGACAAATTTCAGGTCAATACTTCTATACATCTAAGCAAAACGTCATCATGGTTCAACACTACT
TTGACAGATTGCTAAGGCAAAAGAAAAGGGAAGTCTGAAAATATCTACTTTCCAAATAGGAAATGACCGT
TTTCCCCCTCCAAATCTTTCTAAAATAGGCATATTAAAGAACTCATGAACTCATGAACTGAATCTTTGGT
GAAAGCTTTCAACAAGACTCTATGGAGGGAAAGAAACAACTGCCTTTTGGAAAGCTAAGCCTTATTATGA
CTGCTTAGACTTGCTGCTCTTCTCTGCTGTTGGTGCAAATGTACTAATTTTTTAAACGACCATAGTCTGA
GAATCTCCCTGATTTTATATCCAAGTAGAAGACTTTTGTAATCCACTCTACATGGTTGGAAGATTCTCCT
TCCCTTTTTCAAGGAAGTGTAATGAACATGGTCATCTCTGTCTTACCAACTATTTGATTTCATGCCAACC
AAAGAACATATCTCGTCCCAAAATATAAACAGAGCCATAAGAGAAAACACCAATGGTTTATTTCAAGTGA
AGTGTGAGTATTATACTTCATGAAAGGAAATTTTATTCAAAGTAACAACTTGAAAATTCAAGACAAATCA
CTGAAAGAGATCTCTACTCCTTTTCCTCCAATCCCAGCAATGAAGCATTCAAATCCACATGACTCGAGCT
CTGCGATTACTTCATCAACAACTTTCCCTGAAAGTACGGTTTCACATGGTTAAGAAAATACAACAATATG
TTTCTTCTAACGACAATAAGATAAATCCAGGCAACAGTTTTCTTTTCTTATGGACTAGAATAAATGAATG
CTGATATAAGTATTCTAAAATCACCCTCATGGAGGGAGCAATGCATAAAATATGGAATAATGACACCATC
CATGTTGAGTGCCTGAACTTCTCATATTGCTGCTAAATTCAGGTTAAATTATCACTTGAGTTCCCTAGTT
TGTTTTTGTTTCAAGTTAGTCCCTATGAAAATGATAGGAAACAGGATATTTTATAGCATAGAATATAATA
AACAAGGGAGAGTAGGGATCCCAAGTTAACTGCAGGACACAAAAAAGGTCTTCAGTTTTCCTTGAAATCA
AAGAAAGAGGAGTCATGATATTTGCACTAGCCCCTATTATTTTATTTGATCATATTTTAAAAGATCTAAT
TGTTACTAGATACTAACAATATTTAATGTTGCACGTTTTCTTAAGATACTAGCTCAAGAAAATGTATCAC
ATGAGAAACCTATTCACATTTGAAATCAACAACAGAATTTTAGGAGATTTGACCAAATCATGGAGATCAC
CAAATTAGATGGAGATAATTAAGGGATTGGAATGACTATTCAACCCTTAAATAAATAACATACTGAAGAG
TCCTACCAATGAACTCCAAAAACATCAATACTCACAAGAAGGAAATAAAAAGATATAAGTGAGAGCAGAT
GAAGAATTATGCCATAAGATTCCTTATCTGGAAAAGGATACAGTTGGGCAACAGTGTAAGAACACAGCCT
CCACCTCCAGCTTCTGTCAATTTGCAAATTAGCTTGTATTTCAAGCTAGTCCGGAGAACAGTTTCGATAG
ACGCATGGCTTACACCCATACACTGAAGTAAACCTTGATTCATTTCCATCAGTTCTGCCAACTTTTCTTC
ATTCTCAGTTAGGGACACATCGTCGTGAATTGGTGACTGAATAAGGGTTGATAACTCATTGCCTGATAGA
ATTGACAGCATTGAACACTGATTTCATTGCATCGGGATGTCTAATGGCCCTTTCTGAAACACCAGCAACT
AAAGCCTTTGTGTTTCTTCCAACTTTTGTGTTTGTAATAAGCATTTTTATTGGCATATTGGATTTAGTGA
GAGTCAAATTTCCTGACCGGAACTTGATCATGCTCCCTGATACATAAGATTTTCTTGTATGAGAGTGACA
AAACAAAGACTTCTGTTACTTTATAACTCTAACAAACAAACAAGTAATTTGATGTGAATTGTGATTTGAA
ATCGAACTAAAGTAATGAAATTACACACACGCAAACACATAAAAAAACTGGTGCAATTTGATTAAATGGT

CCAAATAACCCAGTGATCGAAACATATTATTCAGAACATCATAAACTTATCGTACTTGAAATTTCCATTA
GGATAACATGCTATCTCTGTACTCTGAAAAAATTCTATAATACAATTGGATTACTTTTTGATATTCCTAA
ACCTTGGCTCCTGACACATAAATCACTAGGTCTATAAGAATTAATCAACTTTCATAGTGACATAGAGTAA
AGTTAGTTCTTGAGCAAGAACGCCTAGCAGATGCAAAGTTAGTTCTTAAACAGTCCACTTTGCTTCAGAG
ACAAATAAAATCCTATGAAGTATTGCAGTAGACAACAAAAAACTACAACCTTCAAACAAACAGTTGAACA
AAAGTGGGGAGATTGAAGAAAAATGGATTTTGCATACCATATGTGCTCACTGTGTTGTCTATTCCAGAGG
GTTTTCCATGGATTATCTTTTCACCCTCAAAGGCCCACTTGTTCAACAAATCTAGTTCATCTTCTTTATA
TATCATCCATCTATGATGCTCCCGATCCACGTTCACAAAACCTGATAAAGCAAGCAGAGCAGCTGATAGG
GCAACACAAAATGCTGCTGATGATCCCAAGCCAGATCCAAGAGGAAGCTCAGAAGTGATGGCCACCTCAA
CAGGCACAAATCTAAATACTTGTGAAACCTATTCCGATATTCCTCCACCACGAGTTCCTGTGTGATCGTC
AGGAACCTTGCCACCAAGGTGCCATCCCTAATCGTTTGGAACCTTGTGAACATGTTCTGCTTCAAGTCAT
CCCAACTCTTAAACGATTCCCACTCATCCTATGATCAATACTAGTCATGGGCCGGACCGTCGAAGCTTAT
AATGGCAACTGACATTTTCTCGGATTCGGTGAGGTTATGGATTTTGAAATAACAGTCTGCTCTGAATAAC
CAAGAATTGGGATCCGTTCCGTTGAAGATCGGCATCTCGACCTACTTAAATTTGCTCAGAACAAACATCT
TATGGTCATCATTCCTCCAATCAGTTTTCGACTCCCCCATCTTCATCTCAGTCGCTGAATCAATCCCAGT
TGATTCCATCAATATATTACAGAATAGTTTGCTGTTGTAATTGCTGTTTCTCCATCTGCACGTTAATATT
CTCAATGCTCTTTGAGATCAAGGACATATTCTCTTCCATTGCAGGTAACTTCTTCATCTCTGTCCTCGTC
TCCAGAATCTCTTGCTCAACGAAGTTCAGTCTTTCTTCGCTCTTCTTAGCCATCTCTTTGCATTTGCCCA
GATTCAGTTGCTCTGATATCAATCTGATAAAACACCAGTATGGTGACTCTACTTTATTGATAGAAATGAA
AAGAAAATCAAAGAACACTACTAGGATAGCAATCCTTCCCTAACCAGAACACAAGACTTCCAACGAAGCA
ATGAAAACAAGATCAAAACAGAAAACGTAATTACAGAAAAATAAACCAGAGCGATGGAAACTGCTACTAC
TTCTCCTCCTATCTCTCAAGAAGTGAGATAGTCCTGATTTTCCTCCACAAAATTCTGCGCCCCTTTCTCT
CTGAGTCTTCCCTGTTAAAACTCCCCCCAATCCTAACTAACCGTTGATCCCACCATTCAGTTTCCCTAAT
CCCGCATTTCCCTCCTTCATCTCACATGCCAACTTCCCCTTATTTCTACGGTTATATGTGACAGTATTGG
TGGCTTAACACAAACACATAAAAAAATTGGCGCAATTTGATTAAATGGTCCAAGTAACCCAGTGATTGAA
ACATATTATTCAGAACATCATGAACTTATCGTACTTGAAATTTCCATTAGGATAGCATGCTATCTCTGTA
CTCTGAAAAATTTCTATGATACAATTGGATTACTTTTTGATATTCCTAAACCTTGGCTCTTGACACATAA
GACTTAATCAGCTTTCATAGTGATATAGAGTAAAGTCAGATCTTGAGCAAGAATGCCTAGCAGATGCAAA
GTTAGTTCTTAAACAGTCCACTTTGCTTCAGAGACAAATAAAATCCTATAAAGTATTGCAGTAGCCAACA
AAAAACTACAACCTTCAAACAAGCAGTTGAACAAAAGTGGAAGATTGAAGAAAAATGGATTTTGCATACC
ATATGTGCTCCCTGTGTTGTCTATTCCAGAGGGTTTTCCATGGATTATCTTTTCACCCTCAAAGGCCCAC
TTGTTCAACAAATCTAGTTCATCTTCTTTATATACCATCCATCCATGATGCTCCCGATCCACGTTCACAG
AACCTGATAAAGCAAGCAGAGCAGCTGATAGGGCAACGCAAAACGCTGCTGATGATCCCAAGCCAGATCC
AAGAGGAAGCTCAGAAGTGATGGCCACCTCAACAGGCACAAATTTAAAACACAAGGGAGCAGACACAGTG
AAATGCAGACTGCACAGGTTAAAATCTGCCAAACCTTTCATGAGTGAAATGCATACCCTAGGATGGAAGA
ACAGAGCCAAAGAAATGCTGCCACTCCAGAGGCAAGTCCGATGTTCACCTCCGGAATGTTTTGATCCTCG
ACCAGAGATGCAATTGATTTCAAGCACTCTGCAAGGCATGTTGTGGGTGACGAGATGGCACCGACAAATA
CACCCAAAGCTTCCCTGATTCTACTAAGTGGCCATGAAAACTCAAGTTTCAGGTCCTTCAATTGGAGTTT
CACAATATCATTCTCGTCTGGTAAGAAAGCATGTAAATATATGAGGAAAAGGGAGTACAAAAAACTTGAG
GGATGAGATGTTGATAAGTTTTTCAATAATGAGAAGCAATCTGTGAAAAGTAACAATTAAACCAAGATGT
AGAGGTCACAAAGGCATATAGCTGTTCTGACACCTTCTACAGCAACAATTGTGCATCTAAAATTCATTTC
TATCTTCACTTTTTCTTGAATGCTAAGAACACGAGTTTCATTCAAATTCACACATGAAAATCAAGTAACA
CCAGAATGTGAATATGAATGCAACATGAAGAAGAAAAATACCCAGAAAACTGAAAATTAGAACATAACTA
CAAAAGGCTACAATGCTTTCCTACCCGGATTAATAACCCATAAAATAAAATGGGTTCATTGAATCAAAAA
CCCATAGAAATATCATCAATAATCAAATGGGTATCAACGAATTAGATCTCCCAAATGAAAGAAACTGACA
ATCCACAAATCCACCTTCCAAATCATATGTTCAGTACCAAACACCTATATAAGAGTCAACCCAAATAAGA
GATAGAGATAGAGAGAGCACCAGAAGAACTTGGCAATCTAACTGAAGCTGTAGTGTAGAGATCAACGGAA
GCGGCAACGGCTGTGGATCCATGGACGACGGCATGTTCACCGGCGAGTATGATTTTCCCGGGAGCTCTGG
CTTTGACTTCCATTATTTTCTAATGAATTTCAATAGAATATTCTAGCTTATTCTGGGTGTCTCTTCTCTG
TGTAGAACAGCAATCGAAGCAAATCGTTGAATTGACAAACTGCCGCAGATTCGTCGATCATTGAAACCAA
TATGCTTTCAATAATGTTTTCTCTCTCTTCATTTTCTTCATTTTCATCACCTACAGAAAAATCTTCCATA
AAAAAGGAGAAATCAACACGCACAGAAGAACAAAGCAAGAAGAAGGAAGCTTGAGCTTTCTCTGTAAAAC
CCAATCACGAAAGGAAGAAATGAAAAGGGCATAAAGAAAAAAAGTAACCCTTTTTACATGCAAAAGTGTG
GCCATTTTGTACGTGGCAGCTGCAACAGAATATCTTATCTAAAAAGCATTTTTTTTTGTTGGGTTGGGTT
GGCAAAATTGTTTCGAATCTAATCCAATCCGTAAACTATAGATTAGATTGGATTGAATTGTCCGATTGCA
TTATTTTTTTTTCAAATTCAAAATCATTTTTGGTTCTACAAGATTCTACAAAATTAATTACAAAATGTAA
ATGTAATTACAGTATTTTCTACAAAATTGTAATTACAGCATCATAAAATCAAGGTTCTACGATCAGCTAA
ATTAAAAGGTAGAAGCAAAGGCGTTTCTTGTTCTTTTGTTTTTTTTATTTTCTTTTCTTTAAAAATAAGA
TACTAACAATGATGTAAAAAGAGGTTTGTAAAGTATTCAAATAGGATCTTTATGATATAAAATCAATTTG
TACTAACCTCAAGATGGAAGGCGAGGGCCGAACTGACGGTGAGGAGGAGACAGACGGCGTCACGGCGAGG

ATGAGGAGACCGGCGGCGAGAATGAGAAGACGAACTTGAAGGCCGATGAGATATAGGATTCGGATCGACG
TGGGTCTGTGGGCGAAAATGAGATGGAAATCGATCGGACAGAGAGAGACGAAGGAAAGCAGAAGACGAAT
CGGCGTGGACTGGGCGCGTGTTTTTACTTTTCAGTTGAACTTTATATTTTATTAAAAATATAATCTAATA
TATAATATTTATTTATATATTATATGTTATATTAATTTAGATTCGGTATATATCGGTTGAATAGTCCGAA
TTTTGGAATTGATCGGGATTTGATACCTATATCCCCTTCGTATCTTCAACATAATTCTTACTCGATAGTT
CACTTCCATAATCTTCAATCTCGCATTTCTGGTTAAGAACGAAGATAATGAACCAGCGTTATTACACTAT
TATGAGTTTCTTGGAAAAATGCAAAACCATGAAGGAGTTGAAACAAATTCAAAGTCTGATGATCAAAACT
TCAGTCGTCAACAACATAATCCCTTGCAGCAGACTTATTGATTTTTGCGCAAATTCCGAACTGGGTGACA
ATGAATATGCAAGAACAGTATTCGATCAAATTGATCAGCCAACTGTCTACGTCTGGAACTCGATGATTAA
AGGGTACTGCAATGGCGGTGACAAGTTTGGAGCTCTGTTTATGTATGAAGAAATGCAGCGTAAAGGCTTC
TCCCCGGACCATTTCACATTCCCTTTTGTGTTGAAAGTTTGCTTTGTAATCGATTGAGTTTATTCCCAAA
TGGAATGTGGTCGCTTGGACTTCCTTAATTGCAGGGTTTGTGAACAACGATCAGCCCAAAGAAGCTTTAA
GATTGTTCAAGATATGGAGAATGAAGGTGTGGAGCCTAATGAAATCACCATGACCACTGCCTTGGCTGCA
GCTGCTCATTGTAGGGATATCTGTACTGGGAAGCTGGTCCGCAATCGTCTTCGCCAACTTGGTTTTGATC
CATTTGACACAAACTCTCGCTTCAATGTAATACTTGCAACTGCAATCATGGACATGTATGCAAAATGTGG
CAAGTTGGTGACTGCACGGAATATGTTTGACAAGATGCCTCAAAGAAACTTGGTTGTTTGGAATTCAATG
GTTAGTGCTTACAGTCAATATGGTCGAGGAGCAGAGGCTTTGCGTCTTTTCGTTGACATGGAACTGGCTG
GGTTTGTTCCTAATAAAGCAACGTTTTTTAGTGTGATAAGAGCTTACACCCATCTGGGGTTTTGATCAAC
AGGACGAAGTTTACATGCTCGTGTGTTGAAAGCAGATTTCCATGAGCCATTGGAACTGCTCTTGTGGACA
TGTATGCCAAAGGTGAACGTGAGTTGGAAGAGGGGCCGACAGTCAGTAACTATTAAGCTAACTGTAAATA
TTGAAAATTAAATAGTTTTTTTTATATATATATTATATAAAGATAATAAAGTTGAAGAGAGCTTGAGCTC
TCGGACCTATACTAAATTCTCCGGTAGCTGGAGATGCAAATTCTGAGTTGAAGATCTTCAGCAAGTCGAA
GAAGAAAGATGTAATGGCGTGGACGATGATGATATCAGGCTTAACATTTCAATGCTAAGGCGAAGGAGCG
TTGAATGTGTTCAGACGAATGGAAGAAGAAGCCGAAGTTGCTTCTGACCAAATCACCTACATAGCAGTTC
TATGGGCATGCAGCCATCTCGGGCTGGTCGAAGGTCAGAAACATTTTACCTCCATGACCAAGGTTTACGG
TATTGAGCCTACCATGGAGCATTATGGTTGCATGATTGACCTTTTGAGTCGAGCAGGCCACTCCGAAGAG
GCCGAGGAGCTCCCAATGAAAATGCCAACGCAGCCTAATGCAACAATCTTGAGTTCTATTCTGAATGATT
GTGAAATGTATGGAAACGTAGGCCTTGCCAACAGAGTGAAAAGTCACATGGTAGAGTTGAAGAATTCTAG
TAGTGGTGTTTATGTACTTTTGTCAAATATTCATGCCAAAGCCTGTAATTGGGAGGAAATGAAGCTGGCT
AGAGATTCGTTAAAGCATAAAAAAATTGGGAAAACACTTGGGAACAATTTTGTTGAAATAAACTTATTGC
CTTAGCTGTTGCATGCATTGAATTACGAATATTTCATTTCTGTTCAAACGAAAAATCCAACATCTGTTAT
GTTATTAGATGATTTTATTATTTAATTTTATTGGTCCATGCAATTATTAAATACTTCTTTTTTAGCAAAA
AGTGCAAACGTAACCATGGTCCAAACTAAGAATTGAAAACGCAATCAAACCATATTTTATTTATGGGTTC
ATACAACTATTAAATACTTTCTTTTTTGGCAAATAGTGAAGGGAAGTAACTGTGTTACTAACAACTTTCC
TTGTCCAACTCCAAACTAAGAAAACAGAAAAAAGATCAAATCGAGCTGAGTCAACCAAGCTGGTAGCAAA
CATGGTTCTGATAATGTCAATTTTCAATTTCAGTTGGGGGGGGGGGACTGTTAGAGATTTTAAGGAGAGA
GGAATCAAGAATGATAGACATACAGATATCATGAACTTGAAAGTAAAATGGACTAGTCACTTTGTTTATC
TATACAACAAATCTTTTGACAAAATTACAAATTTCATAGCATTGCGATGATTTTCTGGGACCTTGGTTGA
GAAAATAAAAGCTGATCCTTTCAAAGCATCTCCTTCAGAATGAGAGACAAAAAAATGAGTTGTCTTTCCT
AATAGCCCATGTTTCCTAAAAAATAGTTTTCTTTCCACAGAGAGGCCTTTTCCTAAACCTATCATACCAA
AATGAGCCAACAATCATCCTGTATTCTAACCCATTAACACCCCAAAACAAATCATTTAAGCCTGTTAAAT
CACCTTTTACCTACACTCCCTTCTAAAATCCCATCTGCACCAAAAGCACCTGAAAATTGTAGGTTATTGA
CAGGACCAGGTAGCTGGAAGGTGATAGAGAAGTGACCTGGCGGGCATAGATTTTTCGATTGCATCCGAAA
TATTTGTGAATTCTTACAAACTATGTTTTCCCCTGTTGTTGTTACTCCTCTTATTTTCACTTTCCCATCT
GGTTCCATGTCGCAGCTGAAACTGTCTGCTGCCATTAAAGAATAGTCTGGTCAGATAAGAGAACTAATCT
TGTGCCTGTATTGTTAGGATAACATTAACGCTGCACATTATGGTAGCAAAATGAGAGAAAATCGATCAGT
TTCAACAAACACTCACTTTGATCTCTAGCAACCCCTGGAAGAGATACGCGGAAGAAGTATGAATTCTCGT
TCTCAGCAATGTCTACACGTCCAATGACTGGTCCAACTTTTCCCATGGCTGCAGTCCCAGTGAGGGAAAC
TCCAGCTTTGGTGGCAGATACTATATCATCCCACTCTTGACTGGTTGTGAGAGGGCAATATACCATAGCT
GGCCCAACCTTCACCATAGCCTCTGGATCTTCAACTCGTGGAGAAGAAGGAATGTAGCTGTGTGGTAGAG
GTGGACCAATATATGGCATGCTATTGATGGGAGCCACATTGAGAATACGTTGACGAGGATTTGAAGAATC
TGCCTCATTTTTTCTTGTTTCCTCAACCCTGCAAGACAAGGAGTAATATGATCACAACATAGTCTGATGG
CACTCTGTCATACCATTCATTGAACATTGAAGAGATATTATGAATGAGTCTTTAAACTTCCATTCTTTTT
CTAACAAAAAATTCCAAAGGATCTCCCTCACATTATGCTCAGTGCACACTCCACTGATCTCAAGGAGTTT
CATCCATCAAAAGGAGAACTCCAGGAGAACAAAGATTTTGAACTATAAAGGGCCTGTTTGGTCAAACTAT
TCGAATGCACACATACTTCGTATTTGAACTATATGCTTGGGTCTACACTTTCCATTTGATCAAATCTATT
CCAACGCACACATACTTTCAAGAGAAAATTAGTGGGAAAAAGAAAGAAATTCTAGTCTTGGAGCATTTGG
AGGTTAGGTTGGGTTGGGTGGGTAAAAAGAACTCACCCACTGTTTGGTTTGCTAATAATTAAAGAGGGTA
GTTATTGAACCCACCCATTTATCACCCTGTTATTCATGTCAATTCTCCCAATTATTCCACTCCTATAATA
ATTACCAACTCAACTTTACACGCCAAAGTCCCCCAATGATTTTCTTTCTTCACCAAGGTGAACCTGGTG

TGGTTGAAACAACAGATGGAAGATGGGCTAGGATAGAACACAAGCAGATTGTGCAGCCACCTCCAACAAT
ATCTCCTTACAAACATTATCTCTGTAAATAATTTTCTCTCAAACAGCAGCCAAAATGCCACAGAGAGGAT
TCACTTGATGGTCTAGTTTCTTTTTCAAAGGCAAAGCATGTATTCACGTTACTGACATCAGAAGACACAG
ATGAATGGCTGGGATTTCCTCAGAAAACAGATAACCAGAACAACACAAGTACCATTATGAGGTACGCATA
TTTGTCATTTCCTTGTAGCCAACTTCAACGAAACAATAACTCATTTATTTTTGTTTTAATTCAGTATATA
TGGTTTTGAAAGGGAAATAAAAGGATAATATCGGCTATTTAGCTTGAGAGTTAAGAAAAAAATCAATAAG
AACAAGCAACTACAAACATTGACATGGACCAAGGGAATTTGAATGCGTCTCAAAAAATATAAAAATTCCC
TAAACATTTTTTAGAAGTAGTGCAAATTTCAGATATACTTCTATACATCTAAGCAAAACGTCATGGTTCA
ACACAACTTTGACAGATTGCTAAAGCAAAAGTTAAGGGAAGTCTGAAAATATCTACTTTCCAAATAAGAA
ATTACTGTTTTGCCCCCTCCAAATCTTTCTAAAATAGGCATATTAAAGAACTCATGAACCCATGAACTGA
ATCTTTGGTGAAAGCTTTCAACAAGACTCTGTGGAGGGAAAGGAACAACTGCCTTTTGGAAAGCTAAGCT
TTATTATGACTGCTTAGACTTGTTGCTCTTCTCTGCTGTTAGTGGGTGGAAATGTACAAATTCTTTAAAC
GACCATAGTCTCAAAATCTCACTAATTTTATATCCAAGTAGAAGACTTTTTTGTAATCCACTCTACATGG
TTGGAAGATTCTCCTTCCATTTTTCAAAGAAGTGTAATGAACAAGGTGATCTCTGTCTTACCAACTATTT
GATTTCTTGCCAACCAAAGAACATATCTCATCCCAAAATATATAACAGAGCCATAAGAGAAAACACTAAT
GGTTCATCCACGGACTCCCATTACAAAAGAACCTCAGCATGTTTAAAATCGCAAAACAAAGTTTCCCCTG
ATTTCCATCTCACCAAGTTCCCTACTCAATCAACCCAAAACCAAAAGCTCCCACTCAACATTAAGCAAAA
AATAAAAACATAAACACAGCAAAATGATCACTACATATGCCCACATTCAATTCCCAACAATCAAAACACA
AAATTTACCTTTAACTCTTCTTAAAATCCGAAACTAGAGCTCATTTCTCAATATCATAAAAACCCTTTAC
CCTAATACCCACCTCTTCATACATACAACGTTCAAATACCAATAAAAAGGAGATAGTAGTTAATAAGAAA
AGAGAAAACCCCTCCTCTGCTGCATTAACCGATGATGAAGAACAGAAGAGATAATATAGAGAGAGACAAA
GATTGCCCAGTCCAATCGAACAATAAAAGATGGAGATACCTAGCCGGAGAAGTCATCGGAGAGTGGCCGG
AGACTTTGAAAAGGTTCGGATTTCGTCAACGTATTCAGTAACACTGACAAGGAAATGTTAGTGAAGATGT
TGAATTTATAAGGAAGCATTTTGCATAGGGCTGAGATGAATATGGGTTCCCTCCCTCCATATCTTACTCA
CGTGATGTTTTTCTTTTCTTTTTTAGTCTCTTTTAGAAAATGCAAATAATTTCCAAATTTGTATTTAAAT
CTAACTATCCATTCAAATAAATCAATAAATTGTTCCAAAAAGTGTTTCCATTGTTATGAAATTGGTTAGA
AAAACTATCAAAGATATCACTTATATCAACATTTAATCTATTATTTTTTACAAACAAATGTTTGAGTTTT
TAATTTCGTGTCAAATATATCTGGACGTACTTAAATTTCTTAGACATATTTATAATGTTACTAATTTGAG
TTTAATCCTTATTTGCTAATTATTTGAAATAGTTATCTGGTCTTTTATTCTTTAAGAAGACTATCTTTCA
AAATTTAAATAAATAAATTTTTTTTTTTTTTAAATAAATAAAAACTTAGACTAAAAATTTAAATGTTTAG
AAAGAAAAAATTTAAAATTATGGTTGTTTTTATATATAAAAAAACATTTGAGAATTTACAACTAAATTTCC
AAAACTAAATAAGTTTTTATAAAATAAAAAATTTTTCTTAAATTAAATAAATAAAAAGATATAAAAGCAA
TAAAAAGGAAAAGAAATACTCAAATTATCTCAAAAACTAAAATCTTTATGAAAAAAACAGTGGAGAAAAA
AACTTCCTTTTTAAGTAATTTTCTTTGTTGAATAGTGAGGCCGTTAAATGGAAAGATTCGACATTGGTGG
ATTCTTCAACATTTCATTTCTTGATCTTCTTAGATTGTGGGGCTGTTGTACTATTGTTTGTTTGATGCAA
GCTCAACCAATTTTCTCCTCCATGTTTGATTCGAATGCTCTGCTCTTGAGTTTTTTTGTGGCAATTTCGG
GTTTTTCTTTTTGTGGGATTTCTTTTATTTGGTACTGATTGCGTTTGTTCTTCGATTCGGCTGATCTCTC
TGTGCCATTCATATTTGATTCCTTCCAATTAGGTAAAATTATGGTGGAACTTGAGTGGAACTCAACCCTC
CTTTTTCTCTTTGACCTTATCATGATTTATGACTGTAATTGTTGAATTCAAATGGCACTGGCTTGATAAT
TGGATATAGAACGTTTTGATATTTGGGCCAGTTTGTATCATCTTCTTCACACTTTCAATTAAGAAGTTTT
TAGCTGGTTTGGCTTTATTACATTAGTTGTTTGTATCTGGCAAATATTGTTTTGGAATTTCTTGGTTAAC
TTCATTACTCTTTTCTTTGTAAACCAGCTCAAGCTGACCCTAGATTCTTTTTTCCCTTCATTTCTGGAAG
CAAGGTTTTTTCTCTGTCCCAAAAAAGGGAAGAAAACTGCTAGTATGGATGAATGCTGATGCTAATTATA
CTTTTAGCTTGGTGTAGGTTGAAATTTTGGAATTTGTTAGTGATTGTGGAGATTGTGAGAGAAAAAAAAT
TGGTTTCAGTTTGTAAATTAGCTTCAGGCAAATGTGGGGAGGGGATGTCAACAAATGTGAGGAAGGATTT
AATACCAATCTGTAATTTTAAAATGGCAGTTTGATGGTTGTTTCCTCTAACATTTCTTGTTTCTGGCAAA
TTATTTGATAACTGCTTACTTCTGCTGGTGACATCCATGTATTCCATTCAAGCTTCTGCCTGGCTCATCA
ATTATAATTTAGGATATTCACTATCGTAAAAAGGTTTTATCTTTCTAATAATTTTGTATTAACGTAATAA
AGCGAGTGTGTATTAATTTTATTGTAGTTGTCTTTCACATTGCCTTTTGGTTTTCTGTTGATATTAGAAG
TAAATTTACGTTCAAAGTTTAGAAAGAAGCGGTGGTTAAATTTTTGGGTAGCCTTTACTGTATGTAATTA
TGAATCGATGCATGTGAGCATGTGGGAAGGGAGAGACATGAAAGGGGTATTCTTTCCAGATGATTGTTAT
TTATCTTTCCAGCATTTATTTAATCTATACTTTTTCTCCGTCACATCAATTAGCAACTTTTTCTTGCTTG
TAGATTTCTATTTGGCATCTGCTTCTGCAATGGATGGAAGCTCTCCCCAGAACACAAAATCTCCTATTCC
TGTTGTGCCAACTAAAGCACCAGATAATGACCAACATTTTCTCTTGTACTTCATTGCCGGTATCTATTTT
GGGCCTAACCTCAAAGGCAAACCAGCACCGAAGTCTGCTCTACAGAGACTTGCTGAGGAATTGCCTCCCT
ATACTTCTGATCAACTTGCTGGCTCGCTCATGAAGATGGTGGAAGTAGAGCGCATATTTTATTATGTTTT
GAGGAAGGCTGATGAATCTCTTATTATGAAGATGTCTTTATTGCATCAATTCTTCCAAGGAAAATTTCCT
GCACAAGGACGAGATATTAGTTCTCCTCAGTTTCCTGATCTTTTTCCACTTGAATTACATCCTCATACTC
GGTCCAAGAATTGGTATAGATATATCGAGAACCTTTTGTTTATCAACAACCCAGAAGTCTACTACCTGAA
TCCGGAAGATGTTGAAAGGTTTAAGAGGCTAACAGGACTGGATGACTTCTTTTTGGATAGAGATGCAGCC

ACATCACACAATTCGTCAGCTCGTAAAGCTTCACTCAATGTTGAAGCTACAGAGAATAGATCGAATAAAG
AGTTCTCTCCTCTTAAGGATGATCAGCAACATGACTTAGTTACTTCACCTGTTCGTAGTGTTCCATATAA
TGGTAATTTGACACCTCCACATACCAATTCCGACTCTAATCTTTTGGAGAAGAAATTTGGTCCAGCAATG
CTATTTCTTCCAAGACAACCATCTGAAGAAGATTGGGCAAATCTAGTGGCTGCTACAAATTCAGGATTTG
CATTGACTGGAACTGCAGCAATGGGACACGTTGGACCAATAATTGGATCAATGGACATTGGGGAATGTGA
AGACTCGTACTTGTTTCGTGTGTCTCTTCCAGGCGTTAAAAGAGATCCATGTAAGTTGCTTTTGTTTAAT
ATATTTCAATGATCTGAATATTACGTATTAGTTTTTCTTTTTATAAAAAAGGGAAACAAAACTAATGCTC
ATAGATACAAAAAATAGGCTAGCAACTGAAACTTAAAAACAAGGAATCATAAAATAACAAAGCAAAAGTT
CCGAAACTAGAAGCTGAATCTATTACCGCAACCACTCAGATTATCCCAGAGTATTCTGAGTTATAGTTAT
ATATTTGTATACACTAAAAATAAAGGATATTAGTAAGAACAATATAGTTTAGCCGCGATGATATTGGTTA
CATACATCACTCATTGCAGATAAGGTTTTGTAATCATAAATATATGTTTCTTTAATTTCTAAACTTCAGA
AAGGTTCAATTACATTCCTTTAACTTTGAATTCAATTCATTTTACTATGGTAATGAAATGTTGATATGAC
ATACTACGAGTTAAATACAGGGAGGATTCGACATGATTTTGATAAACAAACGATGGAAACAATTGTTTCT
ATCAGGTTGATAGATGGAATGAATGTCACTTTAGTTTGTCTAAGCTATCCGTGTGTTCAATATCTACCAG
ATTATGACCATTTAGGCAACATATACCTATTGGAGTTAGAGACATAATTGAGATCTTTTAAAATCGAAAG
ACTAAATAGACACAACCGTCGGAGTTAACTAATATATACATAATATTAGTCAGTTTTTCAGTGGGTTTTT
TTTCTTTTTTTTTGTCATAAATTGATGAGAGCTTTGATTCATTGATCAGGTGGATTTAACTGTGAAGTTG
AAAAGGATGGGAGAGTGGTGATACAGGGAGTTACAACAACAGGTGAGAGAACAGTGAAAAAACATTCTCA
AGTGTTTGAAATGGTAACTCACAACCTGTGTCCACCAGGAGAGTTTTCACTTTCATTTCAACTACCTGGC
CCTGTTGATCCTCAACATTTCTTAGCTAACTTTGACATTGCTGGGATTCTTGAAGGTGTTGTGATGAAAG
ATTTACAATCATGAATATTAGTAATTTAATTTCATGCCTTGATTTAATGGATAATAAATGATATCCAATT
TTCAAGCTCTGTAGAAGCTGATTTATTAGTCACTTCTGCACCGTTTCATATAGAAGCTGATTTATTAGTT
TGAATGTAGGTTTTGTAAAGGGTTTTTATTCAAAAGCTTAAAAGCATATTTTATACAACGGGATGAGATC
AAACCTCCGATCCAAATCATGACACTTATATTGTTGAGATGTAAGCATATTTATTTTATAAATTATCACA
GATACAAATTATTAACTTATCGAAGATAAGACCTATCGTTCAATATTCCAGAACATCTCGTTTTCTTAGT
AATGAAATACATCATTCTTGTCGAGGTTGCTGGACACGCTAAATATTACATTCCAATTTAAAACAAAATA
ATCTTGAAAAATCTGGAATGATAAATCAATGGAAAAGTTAGTTAAGAGGTATTATAACTTCGATTTATAT
TCGATTAGATAGTCAAAATTACTTCCGAAATAGAATCAAGTAGAATCATAAAACTAAAGATTCTAACCAT
TATTATTCATAAAACTTACTTTTAGAAATTATTGAATAAAAGTTTATTGGGAAAGATCAATTAGTGTGAA
AGCCACATGCTAAGGCTTAACACTCGTACATTTGCAATCAATAACAAGGTCATGAAAAGAATATAGTAGG
TTTAATCTTTTGATATTGAGGAATGAGGTTTAAGAACAATAAAAAAGATCAATCAATTCAACCATCTTAA
TCATTAAATGGTGAGACATCCATTTACCATTTCAAGAGTTCTGAAAATTTTTATTTCCATAGATATTAAA
CTCAGGAAGAAAACAAAAGCTTTGTTATTTTTCCAGATTTCTAACATTTTAAAGAGTAATACTTGGGTCC
TGTACGAGTGTATATCTTTTATTATATAGCGAAAACAAAAGTTTGATGGTATTCTTTCTTTTCAAAAAAA
AAAAAAAAAAAAAAGAACAAGTTTGTTGTGTGACAAACATTGACTGGATAACTAAGAAGGATGAGCCAAA
GTATTATCTTATTTGAAGTACTTTAATATTAGAAAGTGATGATTCATATTGAAGTTCATGTAGATCAATA
ACTAAAATTAGTCATTAGGGTCATTTGCCATCACAGAGACTGTGCAATTGAGAGGTGGGAAAAAAATAAAA
ACCAAACTGTATCATATCTCAATTCATTTAAAACCCTGACGAATTCATGAAAAAATGGAATACACAAAAT
TCCTCCTCTTCCTATTCCATTCATGAAATGTTCAGTGTTTGTAATATTGTTGATTAAGCTGCTGATGCTA
AAGCCTATTGTAAGGTTTAGTTCAGAAATTACAATCAATGGAGAAATTTTCTCATCTATATTCTTGTGGC
AAACGATATCCAGACATCACCAATTTATCTGCGAACATCTTGCCTGTCTTTGGCATCACATGCCAATGCA
ACGTCAAGTTAAACTCTTTACCTCTCAGATTGCTTCCCTGAACATGGAGAAATACAAGTTAGTCACTTTT
CGGTTTAAATCTTACTAATCATCATAAACAGGTTGGAGAAGGCTTTGCAAAAGGACTGGAGATGACCAAC
CTGATCAACAAAGCGATACTTGTTTGATGTATGGATTTGAAACTTTGCAATCTCTTTGGAAGGTACGATA
CCGTCCCAAAGTGAGATCTAGAAACCGCATTTTGGAAAGGGATTAAATTGAAGTAAATGTATGAAGAAGC
TCCGTATAATGAAATATGTGAACTTTTGTGATTTCAGTCATGAGAAAGGCAAAAACCTAAGGCGAGTCAA
GTTGACAAAAGAGATGAGCAGAAACTCACCTGATTCAAGGAATTCGATGGGGTTTCATATTCAGCAGCAA
GAAACACAAAAACCTGTAGCACATATTAAAATGAAGATTATGATAACACTGCCGAATCTCAAGAAAGAAT
CTCAAGAAATATGTATATAAACAGATGAAACCCTTGTGATCCCCAACGCCATTATTAACTTGAATGACGT
AGTTCACAGAATATACAAATAAAGGGAAGAAGCGACAATGACCGCCCTGTGCCAAATGGACATCCAGCTA
TTTGAAACCTATTAACTTCAGGGATTATGACAATTTGTATGACGTTTATAATAGACCCTCCACGGCAGAT
AGTTAAGAGTATATAGAAGTAAGAAAGTAAGGGGAGAATGACTTAGGATTTAGTATGAACATAAGGAGTC
GGCTGGCGCTTAATGTAACTAGTTCAGTTAGATTGGTGGGAGAGAACGAGTCAAGAGGGTATAGCTTGAA
TTCTTCAACTATGGAACACTCAGTGTGAATAAACAAACGAATTTTATCAACTAATTATTTATAACCACCA
TAATTAACCAACCAGACTGTAGAAAGGATAATGAGAGAAAGCAAAAGAAGAAAATAGTTTGCCATTGCAC
CTGCTTTGTATTCCACGTAAATAATGACTGCAAGTCCGCTGATATGTTTAAAGTCATGCTAACCTGCAAA
AAGAAGCATTCTTTCAAATAATCAAGAAAGAGTAATTGCATTATTTTATGAAAATAAACTCCCATCAATC
CCCTTCTAAAAAATGTGCTTGTGCCTATCAACGCCAAAAAATTGATTTTTTTTCAGTGCCTCGTCATTGA
CCAAAGTGCTTCTGAAGAACCAAAGTGTCCTTCAAGAAGAAAGGAGTACATTGTTTCAGGATAATAAGCA
TGTCAACCGCTCACACCAAGTCCACCTTTTCACGAAGGGAATAATCACAAAAAAATCAAATATAATTTCC

CTAGAGATCTGATCATAAATAGAGAATAATTACTCAATATAACTATAAGGTAGGAGATGATATTACGTAC
ATATTCATCAAAAGCCTTGACAAATGACTCGTTCATGAAGATGATGGAGTCACGGAAGTCTAGCTTAAGG
GAAAATGTTAGCTCCAAGAAACTTATTCACACTCAATCTCACACTTACACAATCAAACAAAAGAAACCTA
TTGTTTCAAAGAACTCAACAGAAAGAAGTCCTCTTTTAACTTTTACTTTCCTCGATCACTTTCTTACCTA
GAGTGAGAAAAGGACCTCTTTATTATACTATCCTGTGGGATCTTTACTTTCTCAATATATTCACGCATAT
ACTTGACATATTTAATAAATTGCTCCTCATAATTCATATATTTGTGTATCTAAAAATCCATCTAGTAACC
AATTTTGCTAAGATTTTTTTTTGAAACGGAGGCAAGTTTCTTTATTAATGTAAGACTCAAAGTTCAAGAG
ATTTATAAAATGAGAATAATAGAGAAGCCTAAAAAAAGGGGAGGAGAGAGAGGATCTTGAGGCACACCCA
AACATCTCAACCAGGTTGAGAGAGTTAAAAATGATACAGTTAAAAATACTCTTAATGAAATTACAGAACC
CCTAATCAAGATTACAGCCAACCCACTATCATTTATCTAAGAAAAACACGGCTGTCCATCAGATAAGAAG
AAACAGCATTCGACTGGATTAAAGACCAGCTAAATATTGTTTCCTATACATACAAAGAAAAAACAACAAA
ATATGGTTTAAGTCTGTTCGGTCTAAGCTGGTGCTTCCCTAGGAGGAGTGAAGAGGCTTCATTTCAATTC
ATTTTTGGTACTCTCATCAGGAGTAAGGTCATAGTTATTTGGTCTGATGGTATCCTGGCAATGCTGTGGA
AGTTATAGCTCGAGAAGAACAACAAGATCTTCAACAAGGAGGTGATTAGCCTTGACGAGCTTTGGAACTC
CATTAAACTTTTTGCATCCGTACGTTCTTTTGCCTCTTATAATTAACAGCAAAATCTAAGTAAAACATGG
AACTTATCCCAATCCTAAGAAGACGAAACCTCAGATAAGTTACCCAACCATGATTTTCATACACACAAGG
GAAAGTCTTACCTTTTTGACTGGCTGCGGGCGAGACAGTAGTCGAACAAGGATCAACCAACATCAACATT
CACAGGTACGTGCCCTATTTTTCTAGACTACACGTTCTCCGATAACCAACTTGGGCCTTTTCCCCAACAA
CTTTCCATAATTTCAAATACACAAATGCCCCATTTCATCACATCAAATAACAAATCATGCAGTACAAAAG
GAAAAAGAGGGAGAAAAAGGACCCCTAAAGCTCAACACGTTAGGAAACTGAAATGCCCTTCATTCCTTCT
TCTCAAACAAATTTTCACAAACCCAATAGAACCCCATTTCATCAAATGCTAAAGAAACAAGAAAGTCCAT
GAGATCATACCTCATCATTTCCATTGGGTTGATTTTGAAACCAATTGATGCTCAGAACCTAAGACACAAA
AACAAGAAAACCCAAATCAAATCACCAAATGGGTGTCGAAATCCAAGAAAAATGAATCCACAGATCTAAA
ATGGAAAGGAAAAAGAACCCAGAAAGAGGGGGAAAGAAGAAGAAGGGAGACCTGAACTTGTGCAGTGGGT
GAAGGGGAATTGAGGGTATCGGAAAAGGAAGCCAAAGCACAAATGACGGCGAGAATGGTGGCGGCGAAGG
TCAGCAAAGCGTTGGCTCTGAACCCAAATGAATGCATTTTTGAAATGGGTTCTTTAATTGAGTTTCACCC
AAATTGTTCGGCAGCCTGGAATTGCTTCAATGAATTTAGAGGGGAGAGGGAAAAGGGGGAAAAGGAAAAG
AAACAATGCGAGAGTTGGAAAGGAAAATAAAGTCTGGCTGGAACAGGGGAGAGAGAGAGATTTAGAGAGA
AGGGTCGCATGATTTGGCAATTTTGAGGTCTTTCATTGGAGGGGACACGTTGAATGAACACTGGTACCCA
CTCCATGGACACAAATTTTGATCGTTATCCTTAAAATTCCATTCCACAAATTCATCACTCTTTCATGCAT
TTCTTTTTTCAAAATTTATAAATATAGTAAAATATTACGCTAATCCAAAATATATTATTTTTGTCTATCG
TATTTTGTAGTTAATTCGATTCATTTTACGAAAATGGAAGGATTGATAATTAATTTATTCCTCACTCCCT
CCTCGATTCATCAACTAAATGGAAAATTTTCGCTCTATTATAGATCAAGTCAAGGGTATAAAAGTATTAT
TTAGGCTAAGGCCAAAAACATTGTTTGGGACTATGATTGGATCAAAGTCCTAAGACATTGTTTGGGTTTG
AATTAGGTGGACTCAACTTTCAATAGCAAACCTAATAGTCCAACACTTTGGTTTTGATCTTTGGATCAAG
TTTAAGTTGGGTTCAACTTGCACATTGGATTAGCTTATCAAATGATATAATTATTTGAAAATATATGTAT
TTTTGGATTTTTTTTTTAATGAAATGCTATTAAAGTTTATTAAATTTGTGTTTTTATTAAATTCATTTTA
TTATTTTTATTACTTTTCTAGATAAAATTATTGATATTATAAATTGTCTTAAAATTTTTGTTAGTTTTAA
TACTGAAAAAAAAAACAATTTTAAATATTTTAGTTTTTAATACATAAAATATAATATATGAAAATTATAT
ATATATATATATATATATATATATATATATATATATAAGCATATATATTTGAGTTTAGTGGTCGTCCCAATG
GACCTATCCAAAAAATGTTCTAAACGGATTGGTCCTAACTATAAAAATCATAATTCATATCCAATCAAAA
GAATAAATGAGTTTGATTAGATTAGTTCGACTAAATATTGACGTCGGACCAAATTTGAACAAATTTAGTT
TAATTTTTGACTAGTTATTGGACTATAGACCCGTGGCCAATTTTGAGATGCTAAGGGTCCCACATTAAAA
AAATCATAGAGACTCGCACTCCTTATAAGATAGATGAGTTACTTCTCTCGTTACCAACTAGATTTTAAGA
CGAAACCTCATACTATCAAATATATAAAGCTAAAAAAGCATACATGTTTTAAATTTGTCACTTGTGTTTT
CGCTGAACCTTCATTAATCGTCTTTATCATTAATATGTTTATGCATTATATTTAACATGCAAAGATAGAG
TAAACACAAGAAAAACATATGAGATGTGGTTTGAGAAGTTCAAAACTATTGATACCAAACTAAAACTAAG
CATACCTATAAAATTCTCAAGTGAAACATATAAAATATATAATTGTTCAACATATAAAGATCAAAATTTT
AATTTAACAACAGTTTAAACCGATTATTTGATTAAATTAGTCATTTCGTAAATTCTCTAAATTTAATTAT
GTTCAAAACTATAAAACTTTATATTCCACTAATCTACTAGTCCATACCCCATAACCAATTAGAATGTCAC
ATCTACTTTCTTCCATCAATATATACACACACAACCACTATCGCAAGTTGTGTTCGCTTTAGTCAAAGAT
AAAAAGAAGAATAGAACTTAACTTTAACTAAAACGTTAAAAAAATAAAATGACAATCAATACAATTAAAG
GCTAAGATTAGATTAGGTAGAAAAATAATTATATTAAATAATTTAGGATAAGAATCAAATTTTCCTTTTC
AAATATATATATATACACTTACACCAAAGAAAGAAGTTTTCCTCTTAAAAAGAAAGGAAAAAAAAGAGAG
AAAAGAAGGGATTCGTGTAGCTTTAAAATTACTTTAATATAAAGCTTATGCAATTTATTAAAATGATTGT
TATATATGTAAGATTGATGGTACTTATCATTAACTACATTATATTTCTCACATAACAAGAGAAAGTTTTA
TTAGTTCATCAAAAGTTTGATAAAAGTCATTTTATAGTATAATTTTTTAAAGGTAGGGGGAGAGAGAGAG
AGAGAGAATAAATTCAAAATGGGAATAATGGAAATTTTAGTTAATTCTTTTGTTTTTTCTTTTATTTTTA
AAATGTGGTTTAAACTAGAAAGTGTTTTAAACACACCTATAATAATGTTTAGAGAAATGATTTAAGCATC
CCATTATTACATAAATATTGGCAAACTTCATTTTGGAAGAATAATAATATAGAAAAGAAAAAAAAAATTT

ATATAGACACATACACCCAAACAAAATTATCTAAAACTCGATTCAACGGTTCATTTTTTATTTTTTAGTA
TAATAATATGGGGTGAGAGTTTAAACATTACATAAAGTTTCACTGATTAAGAACGAAGAGCATGACAATT
ATATATACTACTAAAGCAAACTCACTTTGACCTAATTTTTCGAATTTAATGTTTTTTACATTTTTAATTA
TACTTTTTTTTAGTAACATGATCCATTGATTTAAAAATCCTAACGTCTTAAAGCTGTTTTTCCTATTAAA
TTAGTGTAGTTTAGTACTTATACTATCAATCCCTTGATGTTAGAGGTTTGATTCAACTATTCTATACAAT
AATTCAACATATTTGCAAGGTTAAATAGTCGCTTAACAAATCAACCATTACGCTATAGTTTCATTTCAAA
ATCAAAGAGAAAAATGTTAGGGTTGAATTAGGAAAAAAATTAAGTTAACTTACGAGAATATACTACTGTT
TTAATACGTGTAAATATTTATTTGTTTTAATAATTTAATCAAAGGATTAATTGGGTTCATTTTAGATGTT
TTTATAATATATTAATACCTCATTGTTAAATAATATTAGGGTTGATGTGAATATATAATTTTAATCTAAT
TAATTTAATAACTAAAATTGAAAAGTTACCTTATCATAATGATTTTTTTTTCTTTTCAAAAGGTATGGG
AAAAACCCACTACAAATTAGGTGGAAAACATATTTGCCATTAACTATAATTTGAGAATCATAAGCACTTT
CTTTTAGAAAAAATGACAAATAAAAAGAAAAGAATAAAAGAACCATTTTTGTATAACTTTAAGATTGCTT
TTAAGATAAAAAGCTTATACTTTTTTTTTTAAAAAAAATAAATATTTAAAGGCATTTACATATTGCTTTTT
ACCATTTACAAATAAGAGAGGTTTTTTTTCCCAATGATTTTCATTATTCTCAACATGAAACATATATATA
TATATATATATATATATATATATATTTTGTTGTTTTTATCATACCTACGTATGACAAATATGTAGTATGA
AAAATTTAATACGTTAGAAATAAATGTTGCTGACAAAATGATTGTGTTTTCAATATGCTACCAGAGTTTC
GAAACATTAATTTTGAAATCAAAATTTTAACTATACTTGACGTTATTTTCACGTCAAGCAAATGTCAACT
GTTCTTGACGCTAAAACACCGTCAAGTAAAATCGGCACTATACTTAACATTAAAACACCGTCAAGTAAAA
ATTAACGTAAAATAATTCGAAAAACTCCGTGAAAATTCCGTTTTTTTTAGACTATACTTGACGTTTTTCC
TTTAAAATGGTCAATACTTGACGTTTTTGTAAAAAAAACATCAAATATGTAAAAATACCTAATGTCGAAT
AGAGTATATTTTGTAGTAGTATTCAACGTAAACGAGAATGTCTATAAAATAAATGTCGATTTGAATATCA
CATCACTCAAGGTGAAAAGGTTAATCCAAAGTATGTAGTCTCTTGCGTAAAGATTTTTTATTTTAGTATA
ATATGGGTTGTTGACGCATCAATTTTGCTACAAATTAAAATTAGCTCATAGACTATTTGACTTTCTACAT
GCATATAATTCGAATTGTCCTCATTTGTGATGAGAGAGTTAGTTTCCTATCTAGATTTATCGTATTTCTG
AAGTGATGTGTATCTTTCTAGTTATTGTCATTTTTCTCAACGTTTGTTAAAAAAATAATATAAAAGAAAA
ATTGATCAAGAGTGTATAAATTACTTCACTATCTCAAATCATTATTTTGTTAAAAAAAAAAATTAACTAA
TTGATTATCATTTGTCAATTTAAGGTTGTGCTTTTTTTAAAAAAATGATATTTATGGTATACAATTTTTG
ATTCCAATGGATCAATTTTAATTTTTCAAAAATATCTTTGCTGCCAATACTTATCAACAAAATTCTTTAA
CATAATATATGTTTCTCTTTAAATATGGCAAAGATATACTATGAAATATTATTACATACACATTTGCAAT
ATAATTACCAAATAAAGAATCAAAGGAATAAATTTCTTTTGAAAATCTACCATTTAATCAAATTTAGCAC
TTGGGTCATTTCACTTTTCTAAAGTTTTATTTTTAAAAAGAATAATCATTATCTCTTACTTTGTATAATC
ATTTCTAAAAAAGTGTTCAATTATTCCTCTACGATCAAATTATACATTCAAATTTTCTTTATACTATTTT
ATATCAAAAGATCTTCACTTTTTTATAATCTATATTCGTTTTAAGTTAGATGATCAAATCTCATGATTAA
ATAAACTATATATATATATATATATATTAGTCCATGATTTTTATCTCCTTATTATCTATATTGTCCATTTAT
ACAAACGATAATTGAAGATTTTTAACATTAATCTTTAGATACATATTATTAGTAATATTTTTATCCGTCT
TAGTTAATGTTACATCAACCTTGATATGAAAGAGTAAACATAAATGGTTGAGTATAATATGAATGGAAAA
GAATTTAAATTATAGACTTCTTAGTCACTTAATTATATTATATATAAAATGATTATTAGAAAAAATTCTT
TACAATAACATCAAACAAAAGCGTTATTTATTATTTTTTTTCTAAGATCTCTATAGCTAAAGACTAATTA
ATTAGATCCATACCAAATATTAGTAAATATAGTACCATTTAAAAAATTACAAATCAGTAGAAGTCTATCA
ATAATATAACTCTACTGATATATATATCTATACTAAAGCTATTAATAAAATGGTTTTGTTAATAGGAATA
TAAAAATATGATGATCCAAAGAGATTTTGAGAATATATTCTCATAAGAAAACAATTAAAAAAGTGACAGC
TGGGTTTTCTTTGTATTCTCAATTATTTCAAACTAAAAAATGTTGTCTCTTTGTGAGAAGAAAAATTATG
ACTTTGTTTGTATCGTTTAAATATAATCTTTAATAGTAATGACACCTTCACTTAGGTATCAAATTTAATG
GGAAATAAAGTGTGACATTGTCAGATGTGACAAACAAACTATGGTCCTTAATCAAGCAATTAAGTTCGTA
TCATAGATTTTGATCTTTCGTATACTCGAGTTTTGAAAAGTAGATTTCATTTAATTTAGGATGCAAAAAA
TTTCCAAAAAAATTGACCAAATGAAGATAATTAATCGATCTAATCAATTGATCGAAATATTTCACAATTGT
TTTCTCGGTTGGTCTTGTAGATATAAAAGATAATGGACGAGAAAGTCATCTCCCCATTCTAGCCCCCGAA
CATTCCTAACACTTTCGTCCCGAGGGAAGTTTACTAATCTAAACTTCTACTATTGTTACTATTACCAAAA
CACCCTAAAGAAACCCCCTATTAAAATGATAGCCAAGTTGATGTTTGGAGAAAGGTTGAATTTCTTTATG
TTATATTTAATGTCCAATTGTTACGCTAACACACACCTACATTTCTTTAACTAACCTATGCTCAATATCT
TCAAATCAATCGTATGATTATAATAAAATAAAATAAAAATTTAATGTGTTAGCCCATATCTACTTCTACT
ACAAAATAAATTCAACCAAATTCATTTGAAAACATATATTTTATTCTATCATTATTTGTAAAAGAATAAT
TGAAAGACGAAATTGATTTAATTAGAATAGAATTGAGACTATTTAGGACACTTTTAGACTATTTATAAAG
TTCTATTTATTAGATTAAATTCTAACTTCTTTATTTTCTTTCAAACTATAGATGAAAATTTGTAATTTAC
TCTAAATAATCATTAATTTTTTTTTAAAAAAAAAACTTCAATTTGAAGAAATTGTTTTTTAAAACAAAAA
AAAAATTGTTACATAGTTTATAATAAAAATTTCAACACGTCTAAGAACCAACATCTATAGTTATAGAAAG
AATAATAAACAAGAATTTATGTACACATTACTTTTGATATATATAAAATTTTATGAAACAAAAAGAAAAT
TTCCCCTTGGGAAATTCACGATTAATTTAAATTTTTATATAATGTGGCCAAAAAATCATATAAGAATAAA
ATAAAATAAAACTACTTCAGACCGGAAGCCTACTTGAATCGACCGCGGTATTCCCCTGTCCAAGTTCTCG
GCCCGAACCGGGTTCAGCACGGTTACTCCCTCCACGCGGAGTCTTCATCGACGACATACGCGTAAAAAAC

GGCGGTGTCATTGACAACACCCACCGGTCCGACTTCGATGTTCTGTCCAACCGCTCAAGCCTCCGGTAAT
ACTTCCCTCGACTACTCCCTTCACCTGAACCAAATCGGTATCCCCTCATCGAACTGCTCTGCCTCGCCAG
AACCACCATACTAGTCGCCCGGTGCAGCTTCCGGTTCACCACTTGTTTCCTTATCTCCACTGGCAACCGG
AGAGTAAACCGCTCAGTGTCTTCCCCAGGCTGAACCAAGGAGTGGCCGGTCGAGTGAGACCGGGGAAATA
GCCACCGCAATCTATTGGATCGGGATCCCCGTGTACGTGTCCGATTCAACGCCTTGTTACTTGTCGTCGT
ACTGAGCTCTGATTCCGTTTGTATCTGAACCGTAGGTGGAGCCGTCGTCTCTGAAACAACGTCGTTGGAC
TGAGCTTCAATATCACCTCCGTCGGTATCGGAGTTAACGACAGCGTTGGAATCGTCAGCTCGGTGGACGG
AGTCAGTGGGTTGGGGAGACAAATTAGCTCGACAAACAGGGCAAGTAGAGTGAGAAGCCAACCAAGCATC
AATACATTCAGGGTGAAACACATGATCACATTTAGGGATTAAACGCAGCGTTTCATCATCTTCAAATTCA
TTCAAACAAACAGCGCATTCTAGGGCACTTTTACCAATTTTATGCTCTTTCACATCGGAGTAAATCAGAG
TCGGAAACGTTTCAATTACCGCCGGATCGAGACCGCGGGTTGCGCGACGGGAGCGGCCGGCGGCGGCGGT
GATAGGGCGGACGGTGTTGGATGGTGAATCGTTGCAGTGACGGATGTAGACGGAGAAGAAAGCCATGAAG
AAGAGAGCGGCGATGAGGATGACGATGATGACGGCCATGGAACCGCTGAGGCGGTACTGGTAAGGGTCTG
ATCTGGGGTCGGGGGCGGGCTGGAACTGGGCGGAGACGGAGGAGAAGAGGAAGAGGAGGAGGAAGAGGAA
GAGGGGAAGGCCATGGGGGGCGGTGAGAGAGGGCATGATTTTGGATGATGGGGTGAAGAGGAAAAGAGAG
GGGGGGGGGGGTGTTATTATGGGAGAAAGTGGAGGTTGGTGGAAGAGGGAGCCATGGCGGAGGAAAGTCA
AGGGAAGGTTTAAAGGAAAAGAGAGAAGGGGGAATCAGTAGAAGAGGAGGGAGGAAGTAGGAAGACTCTG
AGAAACGGTGTCGTGTTGGGCAAGGAACGTGATTGAGGATTGTTTGGACTTTCAGTGTGGGCCCGCGTAT
GTGGGAGAAATTACCCATTTTGGGTATTAATTTCACTTTACAAACATAACATTAGCATATATATATATAT
AACACAATCTATCCAATATAATAACATTAGTAGAAATTTTTTCAACAAAATGGTAAAAATTAGTATTCGT
AAGTCTTTAAAAGGGTTTTGGTGATGAGTATCGATCGTTAGAGCTAATTTTCCAGTCAAATCACTGTTGA
GTTAACTTTAGTAAACTTTTACATTAATACCAACGGTAAAGGAATACAAATAAGTTGATTAGCTGATTAA
GTCTTTGAAAAAAATATTTAAATATTTTTAGTTTGGATTTTTTTTCAAACCAACACCGATCAAACCCTTT
TTATCTTTGACTGACTTATTCGATCGACTTAACTTTAAATGTTAGGTACTAAGAAAATCTCACTTTATGA
AAATATCATAAAAAATATTGACATCAATAATTCATTCAAAAAAAAATTTAACGATATTTTAGTTTTCCAT
AATGAGTTTAAACTTTGTTTTAATATTTATATTGTGGGCAAATTAGTGATATATTTTTTCATTTTTCTAT
GATACAATATCAAAGTATTGTATTGTATTGTATTGTATTGTATTGTATACTTTCAAACTATTGCATGCTA
AATTTTAAATATTAAATTGTTGTTTTCAAAATGAATATGTATATATAAATTCAGATATGTATATATTGAA
AGAAGAAGATACCAAAAAAGTAGTTGTTAAAGCATTTGTTTTTTATACAATTTAGAAGGGGAAAGTTTTG
TAATATTGGGAGAGTGGGCGGTTGATGGTGAAATTAAAGAGTGACAATTTGGAAGAATATTAGTCTTAGA
TGGCGTTCCATGTGGTGGAATTTATGAACACGATTTTCATTTTTTTTTCCTTTCCTTTTCCATTTTCTGA
TTTATTTAGATATTTCTTTCTATACCATTACAAATATCTAAAATTATTCAAACACTCTTCCTAACAAAAT
ATAATGAATTTTTGTTGATTGCCAAAATTCAACTCATAAATTGTTTTAGAGGAGGATGGAACATGACTTT
GCAAAACAAGAAAAAAATTTGTATATTAGTTTGGTGTTTACCGAACACAATCTAATTGATGCAAGTCAAAG
TGTGAAAGAGTAGAAAGATTTAGAGGTCATTTAGAACAACGATTCTTATTTAAAATTTCTAGTTAACTAA
TGTAAACATTATTTTCGAAGTCCAAATTTGCTATAGTACTTTTTTTTTACTATTTTTATATTCACCATAA
CGCTCATAGTTTGTAACTCTATATCATATTATATTCAATGAAGTTATTATTGAGAAATTATTAGTGAAAT
TGAATACTATAAATCTTAAATCCAATAAACTAAAGTCCTGAAGGTTTTTTTTGAGTAAACTTAAACTTTC
TATAGTATATGATTAAATGTTGGACACTTCATTCTAGGAACACTATGAAAGCGACTCACTTTGTAGTTAT
TACAAATAATGCGATTCTAAATCGTGTTCATGTGGAGACATAAAAGTGAGGGACCCCCCTATATAAAAAG
TTTATATAATACTATAATCATGAAATAGTAACTTTTACATTATAATAACGTTGACGAACAAATTGACTAT
TTCGATTATTGATGACCTAAATAACTTAATCTTAATCTTGAGTTAACAACGACTCGTATTCACATGAGAT
TATATCCTTAGATCCTGAGCTAATTAACTTAATCCTAATTTCGAGCTAATTAGGGTAAAATTTTGATTTG
ATTTCCGTTATGCATGTTTAAAATCCATCATTTTTATTATTTCGGATAATTAAAATATAATGGATAGCAA
TTTTAGAGATAATAATTAAATATATAAAAATATTTAATCAAATATAACAAAGTCTATCAACGATATACTT
TTATTGATATACCAAAATTAATTCTGCTTTATTATTTTATCGATTTTGTATAGTATAACAAAATGGTTGA
TTCATCTCTTCTCTCAATATCTAACCCATTGATATAATTTATTAATACTACAAAGTCCATAAAGAATACA
TCTATTATCTTATAATGAGAGGGAAAAAGTTTGTATATATCTCTCACAATACACTATTTCTCACCCTAAA
GTGTTCACTTTTTATGTCATTTTTTTAATGCATTCCTCTTTTTAGGTAGGTTACCAACTAAATAATTATG
AAGACACATACTACACTAGTCCAATTAAATGAGGTCACCTAAAGTTATGGGTTGAGATTAATATCACACA
TCACACTTTGGGATTAGTTATGAATAGGGGTATGTTTGCTTTTAGTTCAAAAGTGGTGCACTTTTTAAAA
CCTAATCGAACCAACTTTCTCTAGCTAGCTTCTTGGCCTCTTTTTTTGAGAAAAAAGGTGTCACATTTGA
TTAATTATTGATGTTAAGGTCTATATTGAATTATATATTCGTTACTATTTGGTATATGCAAGTATCATAG
TCTCTAACCAACTTAACCATTAAAGTGTTGTTTGGTTGACATTAATCATTACGTGTAACCCTAGTTCCAT
AAAACATACATTATATAGCTATATAAAGAATAGATTTTTTTTAATAATTTATAAATATATATTTATAATA
TTAAATTCTCATCAACATGTTGAGATTTTCATGTTTCTATGGATTTGATATCGACATATGGAGTGAATCA
ATATTTTTTTATATTGTATTGTAGAAAAATCCACAAAACATATAAAAATAAGGATCAAAACGCTACTAGC
TAGTATAAACATAATACACACACTCGTTATGTTTACTCATTTGAAAATAATGAAATGTTTGTTTATTAAT
TTTAAACATAGTTTTTGAATGGTTTGTTTCTATAAATTTTCAAAAGAATAATCCACTAAAATTGATAATT
TCATCGAAATTTTCGTAGAATTATGATTTCAACATTTCGGTTGACATCGATATCTTAATCTTGCATATAC

TACAAAGATATTAGCATATCTAAAATGAGGTCTAGGAAACCACCAAATGTTATGGAAAGAAATGATCATA
ATATTTAGAGTCATTTGGCAAGAAATTTGAAATTAACCATATGCAAAAGATTCGATAAAAACAAAATTAC
ATTTCATGTTTTTAAATATGTGTTTAGTAGTACATCCAAAGAATTTGATTCTAATTATTAAATCCATCGT
TAATTAATTTATGAACATGTTTTATAATACATATTTAAATTAAAAAAATAAAAATAGAGTTTATAATAGA
AAATATTGTATGGGTAATGTTTTTAATTATCTTTATATAATATAACTCAACATCTCAAAATTTAAATATA
GTAATGTATAAAAAATTTAGATTTGAATAGAGAATTAAAAAAAAAAATAAGGATCTGAATTGGAAAGAAGG
TTTTGATATATTTAAAGGGATTATTGTTGATGATTATATATATATATATAAAAATGTGATTGGTGATGAA
ATGAGGAAATGAAATGGGAATTAAAGAGAGATGGAAAGGAGAGGTTGGAATTGCAAGTAATAAGGAAGGA
AAGCATAGGATAAGATTACGTTATACTTCTAGAAGCCATACATTATATTAAATTTAAAAGTAAACGCTAT
GGGAAATGGGAAATGGAAATAACAAGAAATTCTTCTCAACCCAAGAATGGCGGAATCTGCAAAGAATCCT
TCCTTCCTTCCAATTATTCTTTTATTTATTTTCTCTTTCCCCCTTTTTTTTTTCTTTCCAAGTCACATTG
TCTCGGATGCGACAAGGAGTTAGTTGTTATCGTTGAGTTAGGCCTATACTTATTTATAGATTTAAATTTA
AGAGTTCCCTTCAACTAGACGATCACTAACATATTTTTAGGGTTAAATGGATTTTTTAGTTCTTCGATTC
TTATTTTTAATATGAAAATTTAAAATATATCTTCGTATCGATTTGAAAAATTATTATAATTATTTGATCA
ATTTTAATAAAATTAACTTTTAAGAGATTAAATACAAAATTTGTTGAGTCAAAATTGAACAAACACGAGG
ACTAAGTGACATTTTATCCTATATTTATCAACGACTGATATTTTTATTCATATTTTTATATTTTAATTAG
CTTGATACTAATAGGTATGAAGGATGAATTAAAATAATGTTATTAATAGAAATATAAAATATAAAATATA
AATAATTTAACTTATTTAGAAAAGCATAATTTTGATTTTTTTTGTTTTATTCTCTTTTTACAAATATCTA
TTTTAACATCGACATCTTACCGATTTTTTTATTAATATTTTTGTAAAATTTAAACTTCTACATTTTCTGT
TGACATCAATATTTTAAACCTTGACCAAAGGTATATGGACGTTTTATTTTTTTCTCCCATAATTTCATAT
GAAATATGTTTGTCTTTAGACATATGATTTATGAATAATCTTTTAGATTTTGACTAAAATGAGTAGCTCA
ACTAACATAAAAGCATATACTATCATCTTCAAATATTCTTCGGTTATTAGGGTTACATTTTATAACTATG
GATGTCAAGGACTCGACTCTCAAACCCTAAAAAAATGCTTATAAGATTTACATATATTTGTATATTTTAC
CCTTGTGTTATAATGTTGAGCAGAAAATTGATATAAGTGGAAATTCGAAATTAGATTTCTTCTAAAATGG
GGTAAAATTAATTGGAATGAGTAGATTCTTATTTTGCTAATAATATATACAAATACTATTTGCTCACTTT
ATTAATTTACTTACTAATTAATAATAATAATAATAATCATTTTTCTCTGTCACTAAATCTTTAACAAGCA
AATAAATGAATAGTACATGTGATCACATATATATGTAGAATAAAATTATGGTATATGAATCTCATTCAAA
TTTGGTCAGAAAAAATCCCATTAAAAATAATATTGTAAAGTTTGATAGGAATAAAATGTTTATTTAACTT
CCTAATCACATGCAAAATAGTAAAGCTATTTTTTTAATTACTTAATTCTTTTATATTATAAGTGTGTTAG
ATTTCATTGGAAAATAATTTTGATCATAGAAAAAGCATTAATTTAATCATAGTCAATATAATGTAAAATA
ATAGAGAGTAGTTTGACTAATTATAAAATGTTTAATGATAAAACGAGTATAACTTATAATTAACACATAA
TATATATTAGTAATTAAAAAAATATAAAAAATTAAACTTATAAGAGATCGATAATTGCTTTCCAATTTTA
GAGTATATGTATATATATATATATATTTTTTATTACTCTCATAGTTTTTTTTTTCCTTTAGCAATGATTGAC
TAATTTTATTTATTTTTTAAAAAAAGAAAAAAAAAAAAAAAAGGCGACCACACAAACGTTTTAATTGAATT
AAAGAAAATAATAATGTTTGACTCAATAGAGACATTAAAATAAGTGCTTTGTGTGGGTGTAAGCATTACA
ATCCCAAAGTTGGGAGAAAGTGTTTTGAATTATGCCATTGACAAAGCAACCAAAATAGACCAAACCAAAC
AAAACTAATTTATTTCTTTATATATATATAGAAACATTATGTTTGGTATCTTAATTTTAAATAAAATGGT
GTAGGTTCTTTCCAAGAAATGGAGGTCTCATATGATAACAAATTAAGCAAAGGTCAAAAGTGGAGCATAT
GACTAAAATTAAAAATTAAAAAAAAAAAAGAAAGAAAAAGAAGAAGGAATAAATAAAAGGGAAAATTAAA
TTGTTAAAATTGCTTTCAAAGGCAACATCACACCTTGGATGATATATAATTATATTCATGAATTCAAACA
TATGAAATGAAGTTTCTTTGTTTGAAAATAAAGACAGATTCTTAGAAAAATAAAATATTTACATTTTCAT
AAACTTTTTTTTTATTTTTTTTTTTTAATTTTTAAATTTAGATAAGATTCTTTAATTTTATATGGTCCTC
ACAAGATTAGATTATTATTCTTTGACAAAGAACTTTGGAACTCTCTTCCTCCATATAAAATTTCTTTTCA
TTTTAACATTAACTTTATGTTTTCTTTTATTCCCTTTATAATGTAGAAAGAGACATAGTTTTAATTAAAT
AAGCAAACAATAGGGTGGGTAGAAATTTATTTTTAAGAAAAATTGGCCTAAAGTAGTAGCCCTAGTATCA
ACCCTAGAATGTTCGAGGAAATTTTGCATGGTCGAATCCATTGTTGATGTTTTTTTAATTTAGTATATAA
TTTAGCAAAGTCCAACCGTAACGTAGTATAATAGTACAATATCATAGATATTCATCTTTTTTTTTTTAAA
CATATTCTCATACTTTTCTTTTTTGTTTAATAAATTTTTAAATTTACTGTTGAAGTAAAATAATACATTA
AAACGTGAGATAGAGATTGTGATATAAAATTGTATGTAAGATCTAAAAATTCGTCACAAAGTTCCGAAGT
CATCGTACTTATCAATTGTTCCAAATTAGAATTAATTTCCATGCAAAATTTTACTTTTGTGATAATTTTA
GAACGTTTATGAAATTCTAAAAGATAGTCCTAATGTTGAGCTAAAAATGTCATTTTGTGAAATTATCTAA
GGTTGAACGAGTAAATTTCCTCTTTCTCTTAAAACTAATATTACAATCTATATATATATATATATATATA
TATATATTGAATTTGGAACATGGATTGGATGTTGACCTACCCAATAAATGGTGAACTTGCAATATGACTT
TTGACTTTTTCAACATTTTTAATATATTGGAGAATTAACATGCTTTGACTTCAATAACCAATAAGCTAGA
AAAAAATAAAATAAAATAGGTGAACGTTATTATATAAATATATGTCCATAATCACATGTCATTTCTTTCT
TCATCAAACTCTCATGTCATGTGTCTAGATTGTGTTCATCCTTAGAGTTCATGTAATCCACTTCATGCTT
GTCAAATTATCGATAAATAGTGACATTTGATGGATTTTAATAAACACAATCGTATCGGAGGAATTCAAAA
AAGTTTGGAGAAAGTGAAGAAATGCGATATTTTTTATTTTATTTATTTTTCTTATTTTCTTATCTATATT
ATTTTATTTATTTATTTTGTAATATCATAGTTTTGATCTATATCCATGTTCCAGTTGTGCCTCGTTTTTA
CAACAAGAGTTATTTATTTTAAGATGCTTTTATCAGCCGTAAACTAAAAAATTGAAACAATTTACAAAAT

AGAGTAAAATCTATCGAATTATGTATATCCCATTTAAGTTTTGAATTTTTCCTTTTATCAATTTGTACAT
TTAACTTTTTGTAACTTTATATTTATTAATATTGATATTTTATAGATATTAAATCAACATTTTTGCGAAA
TTGAAAGTAAACATTTTCGACAACTCATCGATATTTTAAACCTTAGTAGATGAAGTGAATATGAAAATTT
GAATGACAAAAGGCTAATTTCCCATCCTAACCCCAACCCCACCCCTCTAATTTCTAAATTAGAGAAGACA
TACATTTCAATGGCCCATAATCTACTTTGATCAAAGCTGGGCCTTAACCATTGCAACTTGTTGGGCTGCC
TTTTAATCAATACAAAAATGACTAATTAATTTAGATTGATTTTTTTTTTAAAAAAAATCATTTACATTAA
AATCTACAATTTTATTTCATTTTTGTTCATAAAGTTTTATAATATTTAATATAATTTCTATATAACTTTT
AAGTAAGTTAATTTTAGTATTTTGAACCTCAAACCTTCAAAAAAAAAAAAAAAAAAAAAGGCCTAATATAT
TTTACAAAATTAGAAATAATACTAATTTCGATTCTTGATTTTTAAAAATCTTTACAAGGTACTAATTACC
ATTGATTTTTTAACGTTTAGAGATTAGAATATCATTCTAAAAATATTGTTACAAGATATAACAAAATTTC
AGATCTACGATGTGATGGTATTGTCTATTAGTATCTATCGTTGATATAATTTGAGGTTTTAATATCATAT
ATTATAAGTATTTTCAACAGTTTTGTCATTTATAATAATTTATCTTTTGTACATTTATATATACTAACAA
AAAGGTTGGCTTATTAATTTCTTTTCAATTTTAAAAATATATAAAAAAAGGTGTACACATAGATAGCTTA
ATTAAACTTCTCCATGGGATAATATAACCTATACACTCTCAAATTAACTTAAAAGACAGCAATACATTTT
TTAAATGCACTAGAGAGTGAATATAATATTGAAATTCATAATACAAAAACTATTCAATTTGTCGGTCTAA
AAAGACATTATATTATATATATATATCAATTTGCGATGCATGGAAACTTTGAACCTTATAATAGATACAA
GAATGGCTTAAATCACCTAAATTTTGTCAATTTAATTATAATTGTTTGACATGGACTACTTTTGTTTATA
TATGCAGAATTGTGAATTTAATCAATAAAAATGTTCATTTGTTTATTTATTTATTATTATTATTATTATT
TTTTGGTATGTCATCATATTCGACAAAAGTAATTATGAAAATATTAGAAATATTTAATTAAGGGAGTTTG
AAGCAATTTATTTTAGAGAGAGTTGGAAAGCAATTTTGAGGTGTATTATAACCTACAAATGGTAACATAC
TTATTTTGATAACAAACTCAACTGACATCTAAATGAATTAGTGACTATAAGGTTTGTAGCTCGAATGATC
CAATTCTTAATAGTACTAAAAAAATAATTTTTAAATAGTAAAAAAAATACTTTTAAGCACTTAAAAAATC
ACTTAAGATGTAAAGAGAGGAACAATACAAGAACAAAACAATCAAATTAAACAGCAAAGTAAAAACGTAA
GAAGAGGAACATGAATAAACTGATATCATATTTGAATGAGAGGGATCCTAAAGACATGAAAGTAAAATCA
ATTAAGAAATATTAAAAAAAAATTGAAAATTACTACTTATACCAACAAAACGTATTTTTATTTTCAGAGG
CTTAATTATAGGAACTCGAAAGTTAAGTGTGTCTAGCTTTTAGCAAATCTATATTAAGTGGTCTCATTTT
TCTAGAACATGTGTGAGTGAGGACAAAACATGTTGAAAGAATCAATGTTGGTTTGTGAGAACGTTGAGAA
GTGCTGAGATCATCAATTACTCAATCTGAATTTCAAATCTTAATTCGAATCCTGGGTTTGGGGCGTTATA
TAAAAAAGAAAAAGAAAATAGGGAAGAAACAAAATTCAATTACATAGGATGTGAGAATAGGTGTATTTAC
AAATTGAAATTTAATAAGAAGTTTATGAAATTTGGAAACCTATTTGAATGTATCTACCAATAATTATTAC
ATTTAATAATTTTTGTTAATTTTTCCCTTTCTAAAATGATTAAAGAAACAACATGACCTCACTTGAAAAC
ATTTCTCTCAAATAAAGTCTTCACAATGAGTGCTTTTCTAAAAAAAAAGAAAAAAAAATATTTATGTCTT
TTAAAAAAAAAAGAAAAGAAAAAAAGAACATTATATTTTATTTTATTATTAAAAAAAAAATCCATTGTTG
AAAGCTTTTTGGAAACACATAGTTTAGGTTAGGGCATATCATCAATGATTAAAATTTAAAGAGTTTGTCA
ATGTAAATTATGAATGGTTGATAATCCATCCTTCAAAGAAAAAAGAAAGACTATATAGCTACTAACTAAT
GCAACTTTTTGAAACAACATTACAAAAAAATGGCAAATCAAACCAAATTACCTTTTGGCTTTTTACATGA
TTATAGTTATTAGTGAATATTTGAGAGAAAAAGAATACCTTTTCCTATTTTTATTTTTATTTTTTATTTT
TTTTATCTTTCATTGGTATAATTATCATATATTTTCTCATTGGGATCCAAAACCCTAATCATAGATTTGT
TTGAAAAATATGTCACATGGGGTCTCATCTCTCCCTCTAGGTATTATTTCTTCTTACTCACTCACTCACT
CACTCTAGGATATATTGTCTTCATTTTGTATGTTTGTTTAAGTTCAAAGCAACCCATTATGAAAAATATA
AATATATACAATATATTTCAATCATAACTAATAATTAAATGGAAAAAAAAAGTTGAATTATTATTGGATC
TATTTATATATAATAAACACACAAAAAGAGAGACTTAATTGGCTCCTCCCTTATTCATTTTTGAACTATA
AAAGGGAATATATATTGAATTGATTTTTTTTTTTTTTTTGAATGAAGGAGAAAAAGTGGGATAAAAAAAGG
AATTTTGATTTCTTAACTTTAGATTTCCAAAGCATAATTATTATTTCTTTTTTCTTACTAACTTTTTCCT
ATCCAAATCATTGCAAAGTTTTGTGACAACATTATATCCTCCCTTGCATGTGAATTATTTTGATGTGTAG
TTGTCAAAATTCCAAAATTTCAATTCTCTCATTTTTTAACTTTTTACCCATATTATATTCTTAGTTCAAA
CAATTTCTTCTTCTTCTTCTCTTTTACCATTTTACTTCTTCTTCTTCTTTTTGGATAGTGTTGGAGGACA
TTGAGTTCATATAGGCTTCATCAATTTTTTTTTTAAATGTAGTTTTATTGAATATTTTGTGATAAATTTGT
TAGGATGTTTCATATTAGAAGAGTGCTTTTATTTTTTAAGATGTGATTTTATAACGTCATATAGGTAGAT
AAAAGAGATCAACATCATAAATTTTACTTAAAACCAAATAGAACAGAAAAGAAAAAAAAAGTCAATTTGT
AGACATAATCTAGAAGGTATGACATATTTTAGGAATGTACGAGGAAGAAATGTTATCGAAATAACATTAT
AATATGCATCATTCGTGTAATTTAGTCTAGAGTTCAATTTGATAAAATTAGCATAATTTTTTTTTAAATA
TAATAAATTGTTAAAATAATTTTTCTTGACATTTAGTAAAAATCCATTAATTTTAGAGAAGTTAAAATGA
ATTACAACATAACGTTTGAGTTTTTATTTTTTAAAAATATCTACTTTTTTTTATTATTATTTTATTGTTT
GAAAGAGTTTTATGTATCATTTAATAATATTCAATTCCAAGTTACATAGACAATCATATCATCCAAATAT
ACATTTTTCAAAAAGAAAATTGTATAGCATCAATTTATCTAATAAAATTCATCCATTCCTTTATTTAAAA
TTTTACTACTCCATGTTTTATAAAAAGAAAAAAAGAGAATAGGAAAACAAATTATTGTTAGATTAATATA
TTTAAACAAAAAGTTTGATTTCCTAAATACAAATACAATTTCATTAAATACAATTTCATTAACCCATGCA
TATATTACTTAAGTTAAATGTTATATATAAACACTAATTATACATATATGTATATACACTAAATCGTTTG
GTTCGTTTCATATATGCAAATTTAATTTTTTTGAATTGGTTGGATAACGTAACCGTTTCTACCATTATCT

CTATAATGGATTAGTGTATTTAATTTTTAATTTATGATGTTGCTAGTTCTATGTTGTATTCAACCATTTT
TATTTGTTGATCAACATAGTTCAAATAAAAACAAAGACAACACATCGTCTTTAAACATATTTTACATATT
CACTATTTGATCTTTTATGAAGTCATTGGATTCTTTCATCAAGTTTATACTATTACTCATGTGGGTGTAC
TTCTCTTTTAATTAAAGAGACAACATATATGTTAATAATAGATATTAGAAACCGTTGCTTAAAATCAAGT
ATTTGAACGTTGCATAAATTCGTGTGTTATGTATAAAAAAGTACATATTAAATGATCTAATTAAATAAAT
GTGACCTTTTGAGATTAATAATTAATATATACTAAGGTTAAATTCTTATTCAAACCTTACCTAACAAATG
CCTGCATTTATAAACAGTGGCAACAGAAGATTAAATTGCAAACATATGAAAAATGAGGTTTTGGGATTAT
ATTTATTTTAAAATATATCATAGTCTCTAATTTCTTTAGCATAGCTGTCTTCACCCAATTAACTTTGGAT
ATTTTGTTTTATATGATAGAGTTATTTACAAATGCCATTACACCAATCTGATCACATCCACAAATGATCA
ATTTTTTCTAACTTAGTATGAAAAGAAATAAATAAAAATAATTATTGTACAACTTCTTTTGTCAATAGAA
TGAAAATAAAATTCTATTAATTAAAAAAAAAGAATTAAATTTAGAAAAAGGTGTGACAAGTGAACGAACT
ATTCAACTCCCTATCACTATTAGTTAGAATTATTTTTGAGTTCATATAAAATCATGTACTTTTTCTAGCG
ATTATAGTGTGACAATACATTACAATAATCGCTTTCAAGAATGTTTCATCGAGAATCCTTTAAGGACTTT
ATTGAAAAGCACTTATACAGAGACTACGAAATTAACAATTAAAGTAGTGTTGGCGTCCAACCGATTTACA
TATATATATACGATTCTAAAACAATATTAGAGAATTGTTATTGAAAATATCATTAAAATGTTGATGTATG
AAGACTATAAATATCAAAAATTCACAAAGTAAAAATACTTAAATTACTTTCACCACTTTTAATTAAATAG
GTTGCAGTAGTAATACTTCTTTTTCTATTTTATTAATTTTTTTATAATAGAAGTTTCGAACACATAGATA
TGAGAATCTCGATAGAAATGACAAGTCAATGAAAATTTTGTAACGACCCAACTTTTCCGGACTAAGCTGA
GGTCACTACCAAATACCAAAACTCGACCATTCAACGTAAAATTTAAAACGGACCAGATACGATTCATTAA
AACAGTATAAACCTTACAAAAGACAGTTTCGGGCCCTATTTTAAATAATTCAAAAAGAAAATCACAAAAT
AAAATGTCAAGTCACCAGTCCAAAATCACAGTCAAAATGTTCTGACAAAATACATAGCGGAAGCGAAAAG
AAAACCAGACGCGTCCATATGGCCTTCACGCATCCTTCCTGCCTCTCGTCGGTCTGCCCCTCGCTGTACC
CCTACCTGAAAAGTTAAAGAAAGGAAAGGGTGAGTATAAACATACCCAGTAAGGGACCCACTACTGGGCC
CGTTAGGGAACAACAGTTAACTTCCTATTCGGGGGTACCCTACATAACAGTCTAGTGGTTCCGTAGAACG
CACATATCAGTCTAGTGCTCCCGAAGGATGCACATATCAGTCTAGTGCTCCCGAAGGATGCACATATCAG
TCTAGTGCTCCCGAAGGATGCACATATCAGTCTAGTGCTCCCGAAGGATGCACATATCAGTCTAGTGCTC
CCGAAGGATGCACATATCAGTCTAGTGCTCCCGAAGGATGCACATATCCGTAAGGTACACTACCCCATAG
ATGAAGCTAACCGTTACCCCTCAGCCCTTACCAAACTGTCTACATCAGTCACATCTCAACGGCATTCATA
TCACAGTCCCGCCATAGGCTTTGTCAGTCAGATAGTATAGGATTAACATCTACACCCTCAGTTGCTTTAC
GCATTACCGATTCGCACACCAATAGGGGAACCCTAGGTCCAATCGACTAAGCAACCAAAACCGGACTCAC
TGTCCTTCCCCGTCCAAACTTCATGAACCACATCCAGATCAGTGTTTAATACATACTAAACCGTAACTTT
AAGGTTCATCAACATATAATTTCAATCCACAAACAGCAGTCACAGTATATTTTCATCAGACAGAATATAA
TATCAGTACTTAACAGTCAACACGCATACAGATATTCAGTACAGTCACTAACGTGTAATCCCCTGTGGAT
TACTACGGTTTTAGCCTGGACTCGGGGTCCAGTAGTAGGAAAACCCTTACCTGATACTCGGTTATGCCCC
TAGATCGAATCCACGCTCAACAGATCCACCTAAACGAAACACGAAAGTTTTAGTAGCTGACTTTAGTAGA
AGCCGTTTTAAAAGGTCAGAAGCGATATCCGTTGACTTACCCCAGGAAAAGAAGCTATCTCCAACCAGGC
CTGCCGCGGAACCCGAGAACTTAGGCGTCAACTCTGCACTTCCTTCAAGGGAGAAAAGTAGGGCCATAAC
TTAATTCAACATTCAAACTCGAATCGGACGAGGTAACATTAGGGAATTCATCAGAACAAACCTTACCGAA
AACTCACCGTGAACCGAAATGAAGGAAGGAAGGCTTAGGTGGCTCGGCTCGGCTCGGCTCGGCTCGGCTC
GGCTTGGTTCGGCTCGCGGCTCGGCTCACTCGGCTCGCGGCTCGGCTTGGTTCGGCTCGCGGCTCGGCTC
GCGGCTCGGCTCACTCGGCTCGCGGCTCGGCTCACTCGGCTCGCGGCTCGGCTCGCGGCTCACTCGGCTC
GGCTTGGACAAGGATGGCGGCTCGGCTCGAACAAGTATCGCGGCTCGGCTTGGCAGCGATTTCGGCTCGG
CTTGGACCGCGATCTCGGCTCGGCTTGGACAGCCGGCTTGGAGCCGATTTAATCTTCACACTCCCGGCGG
CGGAACGCGGCGGCGATTTGCGGACGACACACAAGCTGATGGCGGAGATGATGCCGGCGGTGGTAGAACG
AAGAGAGAAAGCTGGAGAAGCGTCGGCTGTAGATGGATGGTGGCAGAGAGGCGCGACGGCGGCTGTGAGG
TGTTGAAGGCGGTGAAGGAAAAAGAAGAAGAAGATGCGGCGGGCTGGGTGTTTGCTGTCGTCGGTGGGGG
CTGTAAGGAAGAAGAAGGAGAAGAGAAGGAGAGAACGAGGGAGAAGGAGAAAACGAAGGGAGAGATGGCT
GGCGGCGACGGGCGAAGAAGGGAGAAAAAGAAGAAGAAAATGGATGGGCGGCGGCGCTGCGGTGGAGAAG
AGACCGAAAAAACGAAAAAATAAAAGGGAGAGGGGGCGCGCGTTAGGGTTACAGTTTTTAAAAAAAAATT
TATTAATATATATATATATATATATAACTTTAATAATAATAATAAATAGGATATTATTTTATATATATATAT
CTATTAATTTTTAATTATATTAATTAATAATAAAAATCTATAAAGTATTATTATATACATATATATATTA
ACTTAAAAAATTTTATTTAATAACAATAATACTAATAATAATAATGATAATAGAAAACATTAATATTATA
ATATATATAAGGATAATGATAACTACTAATAATAATAATAATATAATTACTATTATATTATTATTATATG
AGTTTACAAATATCAAAATTTCAGAAATTCAGAAATAAATCCAAAAATTAAAATTTTCGAAAAATTACAT
AAAACGGTAAGAGTTTACCTCGAAAATTCGGGGCGTTACATTCTTCCCTCCTTAGGGAACTTTCGTCCTC
GAAAGTTTTATTCCCCGAACAGTTCGGGATAACGGGATCTCATGTCGTCTTCACGCTCCCATGTAGCCTC
TTCTACCCGGTGATTCCGCCATAAGACTTTAACTAGGGGAATTTGTTTATTTCTCAACGTCTTCACCCCT
CTAGCCAGCACCTCAACAGGTTGTTCAACATAGCTCAAGTTTTCATCAATCTCTAGTGGCTCGTAATCCA
CTACATGGGATGGGTCTGGCACGTACTTCCTCAACATAGAAACGTGAAACACATCATGGACTGTCGAGAG
TGATGGAGGCAACGCCAAGCGATAAGCTACAGGGCCAATCCGCTCCAGAATCTCAAATGGCCCAACAAAA

CGGGGACTCAACTTTCCCCTCCTTTCAAAACGCAAGACACCTTTCATAGGTGCTACCTTTAAGAACACCT
TATCCCCTACCTCAAACTCAAGGTCCTTCCGCCTCACATCTGCATAACTCTTCTGCCTACTCTGAGCGGT
ATGCATGCGTGATCTAATCTTCTGTATTGCTTCGTTAGTAGACTGAACTAACTCAGGACCCATCAATCTC
TGCTCACCTACCTCACCCCAGCAAACCGGGGATCTACAACATCTGCCGTACAGGGCCTCAAACGGTGCCA
TGCCAATAGTAGCCTGATAACTGTTATTATAAGCAAATTCCATCAAATGTAAGTGGGAGTCCCAGCTACC
TGGAAATTCCAATGCACACGCCCGTAACATATCCTCTAAAACCTGGTTCAGACGCTCAGTCTGACCGTCA
GTCTGTGGATGGAAAGCCGTACTAAAGTCCAACCTCGTGCCCATAGCAGTCTGCAAACCCTTCCAAAATT
TGGAAGTGAAACGGGCATCTCTATCAGAAACAATCGACACTGGCACTCCATGTAATCTCACTATCTCAGA
CATGTACAACTGTGCCCACTTACTAGCAGTATAGGTGGATTTACCCGGAACGAAGTGCGCTGATTTAGTA
AGTCTGTCCACCACAACCCAGATCACTGTAAAACCCCTCAGAGTTCTCGGTAGCCCTGTAATGAAATCCA
TGGACACGTTCTCCCACTTCCATTCCGGTATGCTCAAGGGTTGTAATAAACCCGCTGGTTTCTGCCTTGG
TGCCTTAACCTGCTGGCACACCAAGCATTTACTAACAAATTCTGCTACTTCCCTCTTCATGTTACGCCAC
CAATAAACCCGCTTCAGGTCCTGATACATCTTCGTGCTACCTGGGTGCATGGAAAATGGGGAACTGTGCG
CCTCAGATAATAATTCTGTCTTAACCGCACTATCTGACGGAACACAGAGGCGTCTCTCAAACAACAGTCC
ACCATCAGAGGATAACGAGAACTCAGCCGTTTGCCCTGCCTCTGCTAGGCCACGCTTCTCCACCAGATAA
GGATCGTTACTCTGAGCATCAATGATCCTCTGCCTCAAAGTCGGCTGTACCGTCAACTGGGCTAACTGCA
TAGTAACTGCCCCCACTAACACTGCAATCTCAGCCCGCTCGAGATCCCGATGCAATGGGGCCTGCCGGGT
AATGAGTGCTGCTGAATGTGACACTTTCCTACTGAGAGCATCGGCTACCACATTTGCCTTGCCTGGATGA
TACAGTATCTCACAATCGTAATCCTTCACTAACTCAAGCCACCTTCGCTGTCTCATATTCAATTCTTTCT
GAGTAAAGAAGTATTTCAAGCTCTTATGATCTGTGAATATCTGTATCTTTTCACCATATAAATAATGCCT
CCATATCTTCAAAGCAAAAACCACTGCTGCCAACTCTAGATCATGAGTAGGGTAGTTCTGCTCATGACTC
TTCAACTGACGAGACGCATAAGCGACCACCTTACCCTGCTGCATCAAAACACAACCCAGACCCTTCTTGG
AAGCATCACTATAAATCACGAAATTGCCAGAACCATCAGGTACCGTAAGTACCGGTGCGGTAACTAACTT
CTGTTTAAGGGTCTGAAAACTGTCCTCACATGCCTTGCTCCAAACAAAAGGAGCTCCCTTTCTGGTCAAC
TGAGTAAGAGGAGTAGCTATACGAGAAAAGTTCTCCACAAACCGTCGATAATAGCCTGCTAAACCCAGAA
AACTACGAACCTCACTGACTGTGGAAGGTCGGGTCCAACCGGTAACTGCCTCTATCTTAGCTGGATCTAC
AGAGACTCCAGCCTTAGAAACCACGTGGCCCAGAAAGGACACCTGCTTCAGCCAAAACTCGCACTTCGAG
AACTTTGCGTACAACTTATTATCCCGAAGTGTTTGCAAAACCATACGCAAGTGCTCCTCGTGTTCGGCCT
CCGTCTTAGAGTATATCAAGATGTCGTCGATAAACACAATCACAAAAGTATCTAGGAATTCCCTAAACAC
TCTGTTCATCAAGTCCATAAACACTGCCGGAGCATTCGTTAAACCAAAAGACATCACAATAAACTCGTAG
TGTCCATATCTGGAACGAAATGCTGTCTTCGGTACATCCTCATCCTTAATTCTCAGCTGATGGTACCCCG
ACCGAAGATCAATCTTAGAGAACACTGTGGCTCCCTGTAACTGGTCAAATAGATCGTCTATCCTGGGTAA
GGGATATCTGTTCTTTACTGTTACTTTGTTCAACTCCCTATAGTCAATGCACAGACGCATCGATCCGTCC
TTCTTCTTAACGAATAAGACTGGCGCACCCCAAGGTGACACGCTCGGTCGAATGAATCCCTTATCAAGCA
ATTCCTGTAACTGTACCTTCAGTTCTTTCAATTCTGCGGGGGCCATTCTGTAAGGGGCTCTGGATATAGG
AACCGTGCCCGGCTCTAACTCTATGGCAAACTCAACCTCCCTGTGCGGAGGTAACCCTGGAAGTTCCTCA
GGAAAAACGTCCGGATAGTCCCTCACCACTGGTTCTGATGACAGGGATACATCGGCCTCTCTAGTATCCA
CCACGCTCGCTAAGATACCCCAAGTACCCTGACTGAGCAGTTTACTGGCCCTGATGGCTGAGATTACCTG
AGGCAACGACTTTGACCCTCCTCCCTTAAATTTAAAACTGGCCAACGAGGGAGGGTTAAACGTTACCTCC
TTACGTGAACAATCTATGCTGGCGTGGTTAGCGGCAGCCAATCCATACCCAGGAATACATCAAAAGTCCA
GCATATTCAGAACTAATCAGCGTACCTCAATCACATGGCCTGCTATCTCAATCTGACATGCCTTCACCTT
TTCCTTCGACAACATACATTCCCCGGAAGGAGTAGATACTGACAGAACATGGTGTAAGGGCTCTACCTCT
AAGCGGGCATGCGACACAAATGCGGAAGAGATAAAAGAATGTGACGATCCCGAATCAAACAAAACTAAGG
CGTAATGCCCCAACACTGGGAGCGTACCTGTCACTACTGTGCCTGCCTTCTCAGCCTCAGTCCTGTTGGT
AGCAAAGGCTCTACCCTGATGTGGAGCACCTGCTCCCTGATTCTGCGCGATCCCCGTGACTCTCAACGGG
CATCTATCAGCTGTATGACCCTCTTGCCTGCACTTAAAGCAGGTCCTGGTCCCGAATAAGCAACGGCCCA
AATGGTGCTTCCCACAAGTGGTACACAACGGCTTCCCTCTGGCAGCCTCCCCTGCCTCAAAAGGTTTCTG
CTGGAAGCTGCGAAACTCACCACCTGGTCTGAAGTTCCGCTGTGGCACTGGAACAGGCTGCTGCTCAGCC
TTCCTCTTCTGTCCCGACGTCGAACCTCTACCAGCGGTCTTAGACGAGTTGGCCCTCTCCTGTAAACTGA
GATCCACTGCCAGGCGCAGTGCATCGGCATGAGTAGCGGGTCTGAAAGCTCGGACCAAACCCTGAATGTC
CAGTCTGAGGCCTCTAACAAACTTATCAGCTCTGGCCGCCTCGGTCGCTATCATCTCGGGAGCGAAGCGG
GATAACATGTCAAACTCCGCATCATACTGCTCCACTGTCATGTCACCCTGCTCTAAGTTCAGAAACTCCT
GCCGCTTGGCATCTCTCAAACTGGCAGAGAAGAATTTCGCATAGAAACTCTCCTTGAACTGCTGCCACGT
GATCTGACTCACATCACCACCTAGCATCCTCTCAGTAGTCTCCCACCATGCAGTACCTCTGTCAGTCAAC
ATAAAGACAGCACACTGAACCTTCTGATCCTCAGGGCATTTCATGTAACGGAATATGGTCTCCAAGGACG
ATAACCACATCTGAGCTCTGGTGGGGTCCTCCAAAGACCCATCAAACGTCGTGGGATTATATTTCCTGAA
ATCCCTCAGGTGCTTAGCCTCTGCTGACAACTGATCCGGCACAAACTGGGGTGCAACTGGTACCGGAGCC
GGAGCTGGAGCAGGAACTGGTGCTGGAGCTGGCGCTGGAGCTGGCGCCGGAGTTGGTGAGGCAGGCTTCT
GCTGCTCCGGCATCTGCATAATCATATCTCTAAACCTCTGCTCCATGGCGGCTAGGTCCGCATGAGTAAC
TGGCGCAGCCGGGTCAGGGGCTTGGGCTACAGGCTGCACCTCAGGCTGAACGCGTCCTGCTCCCCTTCCT

CCGGCTCTCGGGCACCCCTTCGTGCAACTCTCCTTGGTGGCATTTCCCTAACAACCACCAACGATTCCTT
TAGTCACAAATGGTAATTGAATTTGCAGTTTAACTTAGGTAAGGTAATGCATGTAGAGTGATACATATAC
TTTCATGAGAGCGTCCTTGGCGAGCGGCAAGGATCGTTTCAGCCATAAGGACACAAAACACAGACTCACA
TTATAAGTCAGTCTACAGAACCTAAAACTTAGGCTCTGATACCAACTGTAACGACCCAACTTTTCCGGAC
TAAGCTGAGGTCACTACCAAATACCAAAACTCGACCATTCAACGTAAAATTTAAAACGGACCAGATACGA
TTCATTAAAACAGTATAAACCTTACAAAAGACAGTTTCGGGCCCTATTTTAAATAATTCAAAAAGAAAAT
CACAAAATAAAATGTCAAGTCACCAGTCCAAAATCACAGTCAAAATGTTCTGACAAAATACATAGCGGAA
GCGAAAAGAAAACCAGACGCGTCCATATGGCCTTCACGCATCCTTCCTGCCTCTCGTCGGTCTGCCCCTC
GCTGTACCCCTACCTGAAAAGTTAAAGAAAGGAAAGGGTGAGTATAAACATACCCAGTAAGGGACCCACT
ACTGGGCCCGTTAGGGAACAACAGTTAACTTCCTATTCGGGGGTACCCTACATAACAGTCTAGTGGTTCC
GTAGAACGCACATATCAGTCTAGTGCTCCCGAAGGATGCACATATCAGTCTAGTGCTCCCGAAGGATGCA
CATATCAGTCTAGTGCTCCCGAAGGATGCACATATCAGTCTAGTGCTCCCGAAGGATGCACATATCAGTC
TAGTGCTCCCGAAGGATGCACATATCAGTCTAGTGCTCCCGAAGGATGCACATATCCGTAAGGTACACTA
CCCCATAGATGAAGCTAACCGTTACCCCTCAGCCCTTACCAAACTGTCTACATCAGTCACATCTCAACGG
CATTCATATCACAGTCCCGCCATAGGCTTTGTCAGTCAGATAGTATAGGATTAACATCTACACCCTCAGT
TGCTTTACGCATTACCGATTCGCACACCAATAGGGGAACCCTAGGTCCAATCGACTAAGCAACCAAAACC
GGACTCACTGTCCTTCCCCGTCCAAACTTCATGAACCACATCCAGATCAGTGTTTAATACATACTAAACC
GTAACTTTAAGGTTCATCAACATATAATTTCAATCCACAAACAGCAGTCACAGTATATTTTCATCAGACA
GAATATAATATCAGTACTTAACAGTCAACACGCATACAGATATTCAGTACAGTCACTAACGTGTAATCCC
CTGTGGATTACTACGGTTTTAGCCTGGACTCGGGGTCCAGTAGTAGGAAAACCCTTACCTGATACTCGGT
TATGCCCCTAGATCGAAACCACGCTCAACAGAACCACCTAAACGAAACACGAAAGTTTTAGTAGCTGACT
TTAGTAGAAGCCGTTTTAAAAGGTCAGAAGCGATATCCGTTGACTTACCCCAGGAAAAGAAGCTATCTCC
AACCAGGCCTGCCGCGGAACCCGAGAACTTAGGCGTCAACTCTGCACTTCCTTCAAGGGAGAAAAGTAGG
GCCATAACTTAATTCAACATTCAAACTCGAATCGGACGAGGTAACATTAGGGAATTCATCAGAACAAACC
TTACCGAAAACTCACCGTGAACCGAAATGAAGGAAGGAAGGCTTAGGTGGCTCGGCTCGGCTCGGCTCGG
CTCGGCTCGGCTTGGTTCGGCTCGCGGCTCGGCTCACTCGGCTCGCGGCTCGGCTTGGTTCGGCTCGCGG
CTCGGCTCGCGGCTCGGCTCACTCGGCTCGCGGCTCGGCTCACTCGGCTCGCGGCTCGGCTCGCGGCTCA
CTCGGCTCGGCTTGGACAAGGATGGCGGCTCGGCTCGAACAAGTATCGCGGCTCGGCTTGGCAGCGATTT
CGGCTCGGCTTGGACCGCGATCTCGGCTCGGCTTGGACAGCCGGCTTGGAGCCGATTTAATCTTCACACT
CCCGGCGGCGGAACGCGGCGGCGATTTGCGGACGACACACAAGCTGATGGCGGAGATGATGCCGGCGGTG
GTAGAACGAAGAGAGAAAGCTGGAGAAGCGTCGGCTGTAGATGGATGGTGGCAGAGAGGCGCGACGGCGG
CTGTGAGGTGTTGAAGGCGGTGAAGGAAAAAGAAGAAGAAGATGCGGCGGGCTGGGTGTTTGCTGTCGTC
GGTGGGGGCTGTAAGGAAGAAGAAGGAGAAGAGAAGGAGAGAACGAGGGAGAAGGAGAAAACGAAGGGAG
AGATGGCTGGCGGCGACGGGCGAAGAAGGGAGAAAAAGAAGAAGAAAATGGATGGGCGGCGGCGCTGCGG
TGGAGAAGAGACCGAAAAAACGAAAAAATAAAAGGGAGAGGGGGCGCGCGTTAGGGTTACAGTTTTTAAA
AAAAAATTTATTAATATATATATATATATATATATATATATATATATATATATATATATATATATATATA
TATATATATATATATAACTTTAATAATAATAATAAATAGGATATTATTTTATATATATATATCTATTAAT
TTTTAATTATATTAATTAATAATAAAAATCTATAAAGTATTATTATATACATATATATATTAACTTAAAA
AATTTTATTTAATAACAATAATACTAATAATAATAATGATAATAGAAAACATTAATATTATAATATATAT
AAGGATAATGATAACTACTAATAATAATAATAATATAATTACTATTATATTATTATTATATGAGTTTACA
AATATCAAAATTTCAGAAATTCAGAAATAAATCCAAAAATTAAAATTTTCGAAAAATTACATAAAACGGT
AAGAGTTTACCTCGAAAATTCGGGGCGTTACAAATTTAGTGTCCAACATCTTGTCACACATTAACTTAAA
ACAAAACCTCAAAACTAAAAAAAACGACTTCTCTAAGATTTGTATGCTAGCTAGACCAAATTGATATTGA
GACTAAAAAATGTAAGAATCAATTTTACCTCTAAACTTCGTAAAACGTAAGCTTTTATGAAAGAAGCTTT
TCTATTTTCCGACGAATTATATAAAGAGGACAAGATAGGAAGATAGTGCGTACACCTTTGTTTGCTTCTT
CTATATCAAACATGGAAGTTTAGGATAATTGTAAGTTAGTGGTTACTTGAGAAAACTCCAATTTTGAACC
ACTCCCGTTCTCGACTGGAAGGCATCTACCTCATCCCGATCACAAAGAGGGGTTCCCTATTACTACGACC
CGAAGTCATGATGGATTGAAGGTACTTTTTCCTTTTTTTTGAAACAAAATGAAGTGCATAGGGGACCATC
CTTTTGTTGCAACAAGCTGATCCTGAAGTACGAACTTGCAAGGGGCCTTAGGCCCTACTGAGTTTCATTG
ACAACGAGAAAGAATTACGAAACTAATTAGCACCTTTTGAATTGAATCTTAAGTAGTATATAGTGATGCA
GAAGTTGTATTGCTCCTTTTCAATTAGAGAGTTGAAAGGTTAGACTTTTAGAAAGTTAACGAAAAAAATA
GTTTGTTTATATATAGGTATCCATTCTCTTTGAATTGACTCTCCTAATATCTAAACTAAGTGATATCTTG
TAACCTTCGTATAACATAGCAAATCAAAGGCTACACACTGATGCTTTCTTATATTAGCCTAAGTGTCAAA
ATCTATTGTTTTTATTATCATTATTATAATTAAAAAGACCACGTGTTAACAACTCAAAATTGATTGAGAT
TTCAAAATTACCCTTGAATTATCCAGAAGTTTAAATCTCTATAAAAAATGTAAGACATACGAATCTTTAA
TTTAAAGGTATAGGGAATGAATTTTAAGAAGATATCACTTTTAAGAATAGAAAAAAAAAATCAAAATATA
TTTCTCCCATGTATCAATACCAAATGAGTTAAAAAACATTTTTGATTCCTAAACTTTTATTCTAGTAACA
ATTTTGTTCCTAAACTTTAGTTGGTAACTCCATTTAGTCCGATCCATGTACTTTTCTATTTATACCAATT
TAGTTTTTCAAGTTTAGTAAACAAAAACAACAACAACCTATATTTTGAAAATTGTAACAACATAGTCTAT
ATCATTAAAGAAAAAAAAAATCTTTTTAAATCAAGTTAACCGCTAAACTTTTATTATATAACAACTTAAT

TTGTATAGTTTATAAATAAAAGTAAGAATAACTTTCACAATAAAGACTTATTCATTATAAATGGTAAAAG
TATAAATTTTATAACTTAAGGGGCGTTTGGCCCTAGGACTATAGGGAAGTGAAGTGAGTTATGTTAACCC
ACTCCAAGTCTGGGGGAAGGATGGATTAGGTTTTCCATAATCTCTCTTTTTCACTATTCCACTCCTCCCC
CAATCCTCCTCCAATTAACACATACTATTATAACACTACTATATATTGTAATCATTCCTTCAAACAGATA
CCATCATAACATTACCTATCATAATATTTTTCCTAAATGCATACTATCATAATGCTAGAATTATTATGAT
ATTAGAATTATCATAATACTATACACTCACATAAATCACTCAATTCCACGAACGTGCCCGATATTGTTGG
ACAAAATAGTTTCATATTTGAAAGTAAACGGAAATGGTATAAAATAAGATTTTGATTGGTTGAAGAAGCT
GAGAGTTGGGTCCCATGTGAGGTGGCAGAAATGTGAAAGAGAGGAGAGGAGTGTGTGAGTGTGAGTGTGT
GAAGGACGTAAGAGGAGGGAGGAGGTAAGAAGTAGAAGAGTGGTTTTTAGGAAGAAGCCGCAGTTTTGTT
AGAAGAAGGTAGGGAGGGAGGGAGGGAGGGAGGGAGGGAGGGAGGGAGGGAGGGTAAAAGGAAGAGAGAC
GTGTACGGGGGAGAGAAGAGGAAATTATATATATAGAATTGTAGACTTCCAATAACAAAATGACAAATCA
CTTTATGTCCAAATCAAACAGTAACAGTATGGTGAACCTTAATCAGGCAAGTAGGTGGTGGTGGTGGTGA
TGCTGGTGGTGCAGCACCATGTCCTACAAAACACAAAACAATCAAAAAGCCCCACACTATATATATATAT
ATATATATATATATCTCTTTTACCACCATTTTCTTGCATGCATGCAACTCTAAGCCCACACTAAAACTAA
ACCTCTCTCTCTCTCTCTCTCTCTTTTTCTTTTCTTTTTTCTTTTAAAAAAATTTAGAATCTATATAGAT
TATTTCAAATGTTAGTAATTACGTGGAGAAATTATTTCAAACAATAAAACTAGTAAAGATATTTATAAAT
ATAGCAAAATTTTAGAATTTCTATAGATGCCGATAATCAAATTTGTAATTAATAATAATGAATTCATAGA
TTTTGTTGACAGAGGCTTTATATTTCAACAAAAATAATATAGAGAATATAAAAAGTGGGGCTTCAACTCG
AGTGTAAAGGAGATTGAAGGTTAGCTAATAATATTGATATACTATCAATTATATATACATATATATATTA
TATAACTTAGCATAAAGTTGAGGGGTGTTCAAATCCCTTGGATTTATATTAAATTTGTCTATGTTTTTAT
CGGTATCTATTATTAATAGAATATGAAATTTTGTTAATTTTAAACATTTCAGTTCATCTTACTATATTTG
AAAACAAAAATCCTAAAATCAGCAAAAACAAATAATCTTAAACATCTGTTTAGAATAATATGATTTTGAC
TTTTTATATGTAATTAAACTTTCCCCTTGTAATTTGGGTTTATACGCTAACTCCAAAAAGGATATGCCAA
ATTTACATTTGGCTTTTGAAATTAAGCCATCTATATCTAAATTTTACCATTATGAACACAACTTTCTTTT
TCAATTATTTGTTGTTTTTTTTTTTGTTATAAATATTAACACAAAGTTGATTACACAAATTAAGGCTAAT
CCCTATATTCATTTTTACAATCCAAATATCATAGTCTATGCAAGAAAAAAGACTCTCGAGATCCTATTAA
ATTTTAACAATTAAAATAGCATATTAATTATACACGAAAATGACTAATCTTGGTTTAGCTTCAATTGTTA
ATAATAGTAATCCATTATTGCTTCACTTTGTACAATTATGGATGATTTATATAATCGATATTTGGACTAA
TTATGGTTAAATATTTAATTAGTCAATGGTTAGAAACATATCCAAACTTTTTTCACATTATGTGTAAACA
TTTATAGTTTTTATTTGGACTATTTGGTCCTAATCATCCTAGGTGTCTAACCCTAAAAACCCAATATAAT
CACTTAATTTCTACAAGAATTTGGATATATAAACTTAATTTTGGATTCTCAACTTTTAAATTGGATTCCA
TTTGTTGTTGTTTTTGTAGTGTTAAAATATCATATATGAGTTTCATCTAAAAACTTATTGATTGTGAAAA
GAAATAATTATATTTTTTTTTGAAAAAGATACGATAAATCAGTAAGTATTCCCAAACATCTCCACTAGGT
GGACACCTTCTTAGAACCTTCATCCTCCAAACCTCATTAATAATATAAGTAGGGCAAACCAAATAGATGG
GAAAACTTCAGAGCTAGAAATATAATATATACTTTCTATTAAAATTTATGAAGTCTACCTTGAGATTTTT
CCAATATGAGATATCACATCCGAGCATGAGTTTCAATCTCAGACTAAATACTTAAATTTTTCAATGTAAA
ATTCTTAACAAAGAAATAGATAAACAGAAAATTAATAAAAGAGAAACTAAATGTGTGTTAAGAGTGATCA
ATAAATTGCAAAAAAATTTCTTTTATATGCATGTGAGTTTGATATTGACATAAGAAGTGAATCTATTATT
CCACTATTTCATATAAAATTATGAAATATTCGAAATATAACAAAGACGTTTAACAAAATATATATTTAAT
GTCAAGTCTATAACAAAGAAAATTTAACTTAAAAGGTGAAATGCTTAAAATATTTGCTAATGCAAGTTTT
TACGTTAATTTTTTACGAAATATATATCCATCTGCATCAATATTTTATCGATATTACTATCAAAATGTTC
ATATAATTGATAATATTAATTAAAGAAAAAGTAAAGAAGGGGACAATAATTCTTGGAAGTGTGATAGTGA
GAAGTAAAAATTTGAGAGATAGAATTCGATTTTCCGACATGTTTTGTGTTTTTCTTTGTCCTCTTCTTTT
TGTATCTCTTTGTGAACTTTTTGTTATCCTACGTAATAAATGTGCACAATAACAACCACACATATTATAA
GAAAACTATTTTTAAATTATAATTTCACATTAGACTATCATTTTGAAATCAACATTGATAAATCAAACAT
ACACAACATGTAAAAAAATTTCATTCTACACTATAATAACAACGTTTGACTATATATCAATATAATCATC
TTGATCTCTATTAATAGTAAGTTTTTTTTATGTAGTTTTTAGACAATACTTAATACAAATAAGTCTTGAA
TACGATTTGATTCGAATATAGACCAAAGTGTTTGGTATGGTAGCTCTCGAACTTTCATATTAATTACACA
TAACATTAGGTCAAGGGTTTGTTGTTTTTGTTGATTTGAAGGTGGATAAGATGGGATGATTGGCTTTTTG
AATTTACTTGTAGCATTAGCCTTCTTTTAAAAGATAAATGATAAGGACAAAAATAAAGGTTAATCTCTTG
CTCTAAGAAAATAAAATGGTTGTCTTTTTGCAAAATTTAACTTTTTCCCCTTCCCAAATTAGACCTAACT
TGGTTCGGTTCAATGTAGGTATCATTCAGCCACCATCTATTTATCTCTCTTTCATACACAACTAATTTGC
GATTATAACTATTAGGGATATTATAGGACCCCACATGCCAATATTTACTCTCGAAACAACTTTTAATAAT
AATGGGAGTAAAGTAAAAAGTTCCGTTGTAAAGTTTATTCAACGAATAAATTGGTTTGATTTTTCATTTT
ATGAATGGAATGAAAAAAGTATATTTTCTCAAAGAATTGGGAGACAACAAAACCTAAAAGGTAGACTAAA
ATGTTTGATTTAACGTGTGTAAAAGATATAAAGTGCTTTCTCTATATATTTAAAGTTGGATAGTTAATTA
ATATAAAATACATTATATTAATTAACCATCTACACATTATAATCTAAATGTTGATTATTTTCAAATTTAC
GATTGTGGGAGTCTCCTGTTGCATATTGGTACGATATTATCTTTGGGTATGTTCATTTATTATATATATT
TATAAGAATGATGGATGACCAATTTATAGTTATTAATTTAACGAGACTAATCATGTTTGATTTCAATGAA
AATATTTGATCTTAAAAAAAAATTGTAGGAAATTTAAATGATCTTAATAAGTGAGTGACCCTAACTATAT

ATATTATTGAGCGAAAATTTTCTATAAATCCTTCGAGTTATATTTAAGACTCTTTTTGACCATGGGAGCA
ATTCGAGATGGTTCGTAAATGGAATATTATGTTTCCTATTTGGCAAACAAACTATGAATATGTGTAAGTG
TTATTTTTGTTATTATTATTTTTGGCATTCTTGTTTGTCTAAAAGCATAGGGTACATTCCTTCACTTATT
TGTATAATTATTTAAAAATATAAAATTATAATTTTTTTTTGTACTTGAGACTCAAGTAGATATAACAACT
ACTAGTAATTAGTAAATCCATCACTCACAAAGTAATTATAAATGGATATTAAGAGTTTTAATTTTAGCAA
AATTTACATAATCTTAGAAAAAAACGAAAGAAGAAACATATATGGATCATCCTTTTTTTTTTTTTAATAT
TCTTATAATTATCAGTTTTATTGAAGAAATATTTCAATTCATAATCAAATTTTTTAAAATCGTATGTATC
AAATTTTAGTTTTGGATATTTAACTTTTTAATTTGGTGTAAAAATTAAACAAAGAAGTTATTTATCTACT
TAAATTTTTAAAAAATTAAATGATTATTAAACCGATCATAAAACAAATTATGCTATTAGTTTTTTTTTT
TAAGTAGTTAATGACATTCAAAATTATTCCTTTTGTTTGTCATAATTTTGTATAAAAAAAAGACAATCAT
TGTCTCTCTGATTTCCCATTAAATATTTCTTGGATATATATAGCTATGTGCATTAAATCAATCATAGATC
AAATAACTAAATTTAGACAAAAAATTGACATAGAACAAATGTTTTGCATTTAATAAGAAAGAGAGAACTA
GAACCACAAATCACATTGGAATAATGAAAAAGAATTATATATATATATATATATATATATATATATATAT
ATATATATATATATATATATATATATATATATATATATATATATATATATATATATATATAATGTAGGGTTGAT
TATATGTTTTGAATTATTAAGTAAAAAAAGGACAGACAAATAGTCTCACATTATGTGTGTCTTCTACGTT
AAATTGCAAAAAGAAAGAGACAAAGGCTAAAAATATAGAGTAGAAAAGGTGAGGTTGGTGTTTTGATTTA
CACCTCACTTCCCAACAACAAAATCTCAATAATTTTGTTTCCATTTGATTCATTCATTTCATCTCTTTCA
ACAAAAGAAAAAGAGAAACACGAATTCTCATAATTATTACCTCATCATTTAAACCCCTTTCAAACTTAGA
CCCTTTTCGTTTTTTCTTTTTCTTTCTTTCGTTTTCATGACAAAGAAAAACTAATCATCTTTGATTTGTA
CTAAAAATTATAAATGAAGAGTTTAGTGAAACTAAGATTTGAATATTTTGATAAATGTGTCTGTTGTGGT
AAAATTGGATTTTGGATTAGATTTATTGAAATAAATTTTTCTTAACACTTTCGGGTTTTTTTTCTAAAAT
TTTTTTTACTACACTTTTTCTCTCATCTTACATAACAACTTTGACTCTTTTTTAATTCTTTCTTGTGTCT
CGCCACTCTCATGTGTTTAGTCGTTGTTGTCCCCTATCTCCTTTCCCTTACTATGGTGTTTTTCACTTTG
TGTCCAATGAGTAGTACCAACTTCATGTGTAGTGCAACATCAAGTGTGGTGTTGTTTCTCTCTCAATGAC
CAACGGATGCACAAACATTCATCTTGTCAACAATGAATGCCATATCCAAAATTTTGGTTGATAGAAGCTT
TAACAATAAAAATAATCTAGAAAAAAGTAATATCAAGTAAGTCTTAAACATGAGTTTAGAAGATTGAATT
ACCACTAAGCTACCTAATGATATAAATACTATCTTAGGTTATATATATACATATATATATATATATATTA
CATAATAAACATAAAGTTGAAAGGGGTTAAGAGCTATACTAAATTTGTTGGTACATACATGTGGACCATG
TCGGTGTTAAAGAAGTAGTGATGACAAGCTATGCATGACATGTGGCATCTTTCAAGTCTTGGAATTTCGA
TGTGCAATTCTCATCGTTAATCCACATGTAGAGGGAAACGAAAAGTGAGATGAAAAAGAGAGAAAAAGAA
GAAAAGAATACAAACGAAAAGTTAGATGACCTTAGCCAAAGGGTTGTTTAATAAGAAAAAAAAAGAAAAG
AACTTAGTTTGAAAAAGTCTATCAGTCAAATTATTATATCATTCTTGTACAATATTATAGGTCATGCAAG
AACTCCCCCACAACTTTTGAGCTTCTGTCATATATCCTTAACCTTTAAAAAATATTCAATATTCCTAAAT
TTTTTAGTAAATATAAAATTAAAAGTTTAGATTCAACACATATACAAATATATGTTTATCATTAAAGTAA
TATATGTCTATTATCTAGGTCTTATTGAAATCTATCACACACCCTGAAAGTAAAATTTTCACTATATTTG
TAAAAAGAGTTTGTACATATTTGATTCTTAGGTATAAATCAAAATTGATTTTGAATTAATAGAAAAACCC
ATCTGAAAACATTTTCTAAGAAAAATTAGATAGTCCATTGCATGAACTGTAAAGAAACTTCTGATTAGCA
ATTCAACCCTTTTTTTTTTCTTTTTTTTTTTTTTTTGTTGTCATTTGATTTGATGATGGAGTTGAAAAAA
TTGAAATGAATAATAATAATAATATTGATAGGATTTGTGGTAGCTGAAAAGATGACACAAGAGGGGGTTC
TGACAAATGGTAGGCTAAAAGCTATGAAACTTTTGTGGGTGTCTGATTTCATAGAAACTCAAAAAGCCTT
CTTCTCTTAAACCTCTTTCCTTTCTTCTCTGTTTGTGTGTAGAAATGCAAAAGTAAGAGCTTTGTTTTAA
GCAAATGGGCAGTCTATCTTTCAGAAACTGTGTAAGCAAAATGTCAGTCTTTTACCATTCTTACATCTCA
CTCTCTCTCTCCTACCCCATTGTATTATTTACATATCACTATCATCCCATCACCCATTCTTCTCTTTCTC
TCTCTCATTCTTATTCAAACCTTTCTTATGACTTTTATTGGGATTGTTTGGTTCACGGTTTTCTAAACGA
TTCTAAATCCTAAATTTTGTTATGGTTAGCAGTGGAGTTGGGAGGCCAATCTCAAGAGTTTGTGAACTGA
TCCTGTGGTCCATTATAATTATGAATTTAATTATATATATGTGTCGGTAAATGTCGAAAATGCTTGGTTC
ACTGTCTTTAAAGTGAACATAAACCCTAAACTACAATCGAGTTAATAGTGTTTGTAGCAAGAAGTGAAAT
TGTGAAGTTTGAGAGTAGTGTACTGCATGAATACCTTGAATTTTATTATTTATCTGTCGTTTCAAATGAT
TACTAAATACGTTTTTTACCCTACAAGGTAGAAAAATGTAATATATAAGTTCTCTAATATTAGAGGGGTC
TTTAATTTGTAATTTGAATTATTCATTCTAATAATAAAATTCTCTCTCACACTCTCTACCTTTGGACGTA
AATAACATATTATTAGTGAGTGAACTATGTAAATGTTTGTATCAATTTTTTACCATTAATGATTTTTATT
TGTTGATTTCAATGCTTGACCGGTTGAAGTTTATAGCGTAAGAGTTAATAAAGTATAAAACCAGTGTTTT
TTAGTCGTACGAAGTCTAATCTACGAACTTTTTGAGCCAAACAAACTTCTCCTCCAATTCCTATTTCCAA
TTATTCCTTATGCTATGGACATTGTCCCTTTAGGACTTTGAAAGGTTGATTAATTACTGTATGTATGTCT
GTGTGTGTATTTTATGAGGGGTAGAAAGGTGAGTGTATTTTGGTTTATGTTTATTTATAGTAGCATGTTG
CGAAATTTTGTAATTATCGAAGTGTGTTTGGTTTTATATATATATGCCTTTGAAGAATAGGGATTGTTTA
GCTAGGCTTTGTTTCTTACTACGACGTTTTGTTTATTTTCGACGATCGATAACATTTTGTTCTATTAGAA
AAAAAAAGTTTTCTCGACGAGGTTCTACGGACACTTGTGTCGACCGCAAAGAAATCGCCTATAATCGAAT
CTCTCGACCGCTTTTATGTTTTTACCCGATAATTTTCTGCGTCGGAAAAAGAGAACGAAATTTCTTGTAG
TGTTTGTTTGATAATTATTTTATCTTTTTGTTTTTAATCATTTTGCTTTTTCCTAAAATTTTCTATTAAA

TTTTTTACATTTCTTAATAAAAAAAAAACAAATACTTGAATTATCTCACACAAGTTTTCTAATTTATAAA
AAAAGTCTTTTTTTTTTACTTTTTAAAATTTAACTTGATTTTTGAAAATATTCGTAGAAAGTTGAATAAC
AAAACAAAAAAATCTTAAAGGTGGATTTATAATTTTTTAACAAAATACTAGAGATAAGAGATATTAATAG
GTGCATATCGAGACATTTCAACCTAATCATACCCCAATATGATTCGTCTAACTTTTAACAACCAAATGTT
TACTGAAAATGAGTTTACATCACGAGTTTAAAGAATTATAATCAGTGGTTTGAAAGTTTGAATTTATGTT
TACATTTAAACCATCGATCTATGTAAAAAAAATTCTAATGACTAAGTAACTTAGTCAAGAGTTTAGGTTT
GCATCTTAAACATATATAGTTTTAAAGCTTAAGAAAATATGAACGATGTGTTTTTTTATCCCTCGACTCC
AAATTTTTTAGTAAAAGAATATTAATCATAAAAATAATTATTTTCTTTTATCTGTATAACTATAAATAAA
AATCAAGAAACACATTATTTTTAATTAGTATATAATTTGTTTCTATATCAACGATTTTCTTTTGTCTATA
TTGATAATATTATTACTAAAAAATGATAAGCTAAAATTTTACCTTTTATTTTCTTTGTCTACATTGAATT
TTTTTTTTATTTAATGTCAATTATATAAAATATTCAACAACAAATATGTGACAACAATATATTGGCTTCT
TCTTTTCCATCTATAGTGAAGCATATATCTACATGAAATTTTGTAAGTAAACTAATATCACAAATATAGA
CACACTTCTTAAATAATAACTATCATTACTATTATTTAAATAGTTGGCAAAGATAATTACAATATTTACT
CATTTATTTCATTTATGTTTTATCGTAATCGAACTTTCTAACTTTAGAGATGTCATTATTATGTATTTTG
TAATATTTTATTTTTTCTTTTTCATACACCTTGCTAACAAACTACTAGTGAACAATGTAAATATAAACGT
CAATTTCCTTATTAATTTTAGGTTTTATGTACTATCGATTTTATAACAAAATCCAACATGGCAAAGTGTT
TGATCTACTGACGTATCAAATGAGAGACATTTTACTATTTATTACAACCATTCAAGATCAATATTTATTT
TGGATCAATGTTATAAATAAGACAATATTTTCTTTTTCTATTCGACGATGAATGTGAAGATAGAGATTTG
AATATTTAATTTTTTAAGTTAGATTTTAAGTTTAGAATATTATAAAATATAGAGGACCATTTATCGAGAA
ATATATATAATCCATGAATCAATTATTACTTTTCTTTTTGTCCAAAGGGAAAACAATTTGATAACTCATC
CAATATACTCAATTCTCTTTTCTTGTTGACAAGAAATTAATTACTTTTATTTTGGGAGAGATATAGCTTT
TCTCATTTATCAATTGAATGACACTTCACTTTACTAATTTAAAAAACATATTGTCACAAAAGAAATGTCA
TCAATTAATTATTATGTTGGGTACCTAACTTTCCCACCTCTTTCACTATCCAAAATATTCTCCATCCCAC
TATTATAACTCCCAAAAGTTTCCATTTTTATTCCATTTAATTCATTCATTCAATTGTGAGATCTCTACTC
TTCTCCTTCTTCTAATTATTATCATTATTATGTATGAAAAAGTCCAATGGATCCAACATTCTTGCATTGT
ATGATAGATATAGATAACGAGTTTTAGTACAATATCGGTTAACTTCGTTAACATGTGAGTAAGCTAAGTT
TGATTACTTTTAGTGAATAAAATCCTTGTTTAGTAAAAATAACGTATGATCATAACATCCACGTAGACTA
AGATAATGATACTATATCACTTGCTTAATTTTTCTATTTTTGTTAATAAGTTAACAAATTTTGTTCGAGA
AGATCCATTTTCTCCTTTTTTATTAACATTAATTAGAATATAACTCTTTCCATTTTTATTACATTAAGAA
AAAGTTCAAAACTTTGCATCATTTTTTGAAATAAAATTACGTTAGAGTAATTTCAATTACTAGCTAGTTT
CGATACTTTCATTTCAAAGAAAAGAAAAAAAAAAAAGGCTACACCGTTTTTTAGTTACTTTTAAAGAATTG
TTTGAGAACAAATAAAAAAATTTATTTATTTATTTATCAAATCACATGTGAGTGTGAATATGAATTTGTT
TGGATTGACTAGAGAAAAAAATATTTTTAAAATAATCAAATTTGTACTCTTCTGATAAATACTTTTTTAG
AATATATTTTGAAAATATTTTACAAGCTCCTATGATAAAAAAAAAAAAAAACACTTAAAATATACATCAAC
GTCCATGTGTGTACCACAACTCTTAAAAACTAGATTTGAAAAAAAAAAAACATTAACCTACGATTCTTCT
ACCATCTACAAAAGTAAAATATTAAATAGCTAACATAAAGATTTATGTTGTTCACTAACAACATACAAGT
TATGTTCGCAAATAGACAGAAGAAATCAATTTATTACTAAAGAAAATATCAAATCGCAAATCTAGAGGTG
AGAGTAGAGTTAAAATGTCTATATTATGCACTTGTTAATGCCTAGAGCCAAGATTCGAAATCTTGATTCG
GTAACTAATGGCTTTGACATTTTCCTACGTCATATTCGCTTGTCTTAAGGTTTATTAGAGTGAAAAAAAA
TTGTATCTACAAACCAACAAGGGTCATTTCAACGTGCTTTGTCCTCATTAATTGCCTACATCTTAGAAAA
ACTTCCAAAAGATAACCTAACATAGAATTGCTTCAAGCTAAGCATGCTTAACTTTGAAATTTCTATAACT
GAGTTACCGAAAAGAAACGTGCATTTTGATTATATAAACAGTAATATTGAGTTCTTTTTAAATCTTTTTT
AACTTTACTTTCATGCCCACATAATCTCTTTTACTCAAATGTGATCTTAGTTAATCTATGTCTCATCCTA
AACTAGGAACGTTCAACATTTCTTTAAATCTCAACGTCAAAATCGTAAATAATATAAAAACAAATACAAT
TTAGGTACAAGGAGTACTGATCCTAAATTCCACTAGAGCTTGACAATTTTGTACACTCACTCGTTAAAAG
ATAATAGATTCAAACCATTAAACATAAAATTAACAATGACACGTGACATAACGATAATCAAATTACAACT
AAAACCAGGGAAACACATTTAACAAATCCAACAAATACAAACACTATTCCATCCTTAAAAATTACAATAG
TTGAAATTTAAAACTCATATACATATAATTTTACTTTATCAAACTCAAAGGTCTTTATTTATTTATTTAT
TTAATAAATTAACTATTTATAATTTTAGCATCATTTTCAAAAAAATTCCAATGAAATGACTTGTGGGTCT
CAACAATAAGATATGTATGTTTCTTTGTATTGAAAACCACATGTGAAAGAAAAACATATTGATATGAAAT
ATTGTTCTGTTTATGTCTTATTTTGTTCAATTTTTTTTATAATATATATATATATATATTTATTTATTTA
TTTATTTATTTATTTTTCCTTTTTCCTTTTTCCTTCTGTTTTTTTTTAAATTTTCTTTTGGCTCCTTCAC
GTGCAATATTCTGTAGATTTGCTTCCTTCCCCCATCGCCCATCACAAAGCCCTTTAAAAACCCCATTTTT
TCTGCTCCCAATCCCAAAACCCACCATTTTTCTTCCTTTTTCTTCTCTTTTTTTTTTTTTTTTCAAATGGG
TGTCCTTAGAAAATAATCATTGATTTAGAATCATCACTTTATTTTTAATGGCATTAGAGGCTGTGGTTTT
TTCTCAAGATCCATTATGTTACAATGGAAGCAAAGATCTTTACTCTTTACTTGGAGGAGGAATTTGGGCT
AATGGAAGCTTTGAATATCCAGAAATCCCTCATGATTTTCCCGAGAACCAAACAGAAAATTTCCCATTTG
AAGATTGGAACTCATCTTCTTCTGTTTTTGTTCCTAACCCTTCCCCTGAAGCTGCTGATTCCAGAAATGG
GTTGCTGAAACCGCCATTAGAGGCTGAATCAATTACTCCACACCCAATTCGTCCAAGGAAACGAAGACCC
AAATCACGCAAGAACAAAGAGGAAATCGAAAATCAAAGAATGACTCACATCGCTGTTGAACGAAATCGCA

GAAAACAAATGAATGAATATCTCTCTGTTCTTCGTTCCTTAATGCCAGAGTCTTATGTTCAAAGGGTACC
TCTCTCATTTTCTCCCATTTTTCTTTTTCATTTTCCTTTCTCCCCAACAAAAAAAATTAAATGGGCTTCT
CGAGTTTCGTATCACTATTGCAACTCATTAGTTTGAGTTTTTGAGTTAAACGATAATTTAACATTGTATT
AGAATTGTTGGTTCTTTTTTGTTATAACTAAATTTACCGTATCCCATCAGCTTAAGTTAAGTCATTGGTG
ATGTGTTGGCGTCGCTACCGTAATCCATATGCTTAAATATATTTCTGGGTTAATGGGTTTAGGTTGAGTT
ATTGTTCATCTCCCTCGAATGAAGTAAAATTTATGTTGATCCATCAACTTAAACTTTTGACTTAAACTTT
TGGCATTGTCTTTGTTGTTAGGGCCTGCAACGTCATTTTCTTTTGTTTGAGCTTAAAGTTTTTGAATTGA
TCGATGATTTAACGTTATTTCGGCAGGGGGATCAAGCTTCAATTATTGGGGGAGCAATTAATTTTGTGAA
GGAGTTAGAACAACAAGTTCAAGTTCTATCCACAATAGAAACAAAAGGGAAAATTAATAATTCTGCTGAA
GGGTGTTGTAATTCGAATTCAAATTCAAATTCAAAGATTCCTTTCGCAGAGTTCTTCAGTTTCCCTCAAT
TCAAAGCAATGGAAGGTTGTTCTTTAGTGAGTGAAAATGAGACTCAATGTTCTTCCACCGTTGCTGATAT
TGAAGTAACAATGGTAGAAAATCATGCAAATTTGAAAATAAGATCAAAGAGAAGACCAAAACAAATCTTG
AAAATTGTTGCTGGCTTGCATTCTCTTTCCCTTTCTGTTCTTCATCTCAATATTTCAACTATCAACCAAA
TTGTTCTTTATTGTCTCAGTGTCAAGGTTTGCTTCTCTTCATTTTTTGAAATGGGTATTTGTGTTTAGAT
GTTTTTCATTTGAAAACTTCTTTTTTTTTTTTTTGGTTTGGAATTTTGTTGTAGGTCGAAGATGATTGCA
AGCTGAGTTCTGTTGATGAAATTGCTTCTGCCCTGCATCAGTTGCTTAGTAGAATAGAAGAAGATTCACT
TATGAACTGAAATTTTTTTCTTTTAAATTTATGGGGTTAACATTGTTTTCTTTGCCTTGTAGGTTGTAAT
ATCATCTTGTTTCTAACTAATGTAATCTCTATGTTGAATTTTTGTACAATTTATCTCTTTTTTGTTTTCC
CTTTCTTTTTCTGATTTCTCTTTTCATTTTCTGCACTAGGAATTTGTTTGTTGTTCTTATTTTGACAGAT
AAACAAAAGGAGATATTTTTTTTCTTTAGAGAAATCCAATCTTTTGCTTTTCTGATCTATCATTCAAAAG
GGGAAAAAAGAAAAATGTTAAAAGGGAACAGAAAAAACATACAAACATAATCAAAACATAGATTTACGTG
GTTCACTATCTTAACGTTAGCTATGTCTATGACAAAAAGGAAAAGTAGTTATCATATTATAAAGTTCTAG
AGAGTTTTATAACATACTTCTTCTATATGTTTACTGTTTTGAAGCAACGGATAAGAGATTTCATAATATA
AGAAAAAAAGTGAGAAAAAGGGAGCACCAACTACAGATTTGTGTGTTCATCAATGACTTTAGGAAGATCT
CACAAAACCAGAGGTTTGAAAGATGATTTTGGCAGCAGAAAGTTGTCCTTGTATGGATGGGCAGCTTTAG
AAGTGATAAACTCTCTCTCTCTCTCTCTCTCTTTTTCCCTCTTGAAGTCTCCATACTTGAGAAGAGAAAA
AAAAAAAAAGGACAATTGAATTGAAATTTGAAGCTCTGTTTTCTTTATTATTGAAGATTCAAAAGGACAG
GTCTAAAAAGCACTTTTTCTTATGTAGGGATTCTGATTTCTTAGAATCATTGGCATTGATAAAAGGATGG
GTAATGAAAAAAGTTGGCAAATTTGTAAGATTTGAACTTTTGTTGAATTGATCTTTCCATGAAGTTTTAG
AGTCTTGTTATTGAGATGTGATTGAATAACTTTGAAAGGGTTAATTCCAAAAATAAATAAATAAATAAAT
AAAAACTTGGAAGGGTCCAAATGCAATTATTTGGATAAAGCTATTTTTGATCGATTTTGAGTTGTATTCA
TCTTTATATAATAAACTTTAATCATACTAATCAAAACAAGAAAAAAAAAAACATAAAAATTTGTGATTTTT
GCGATGATTATACATACGGTTTTTACGATGCTAGACTTGGATTATCAAATATGTATTATAATACATTTTT
TTCTCTTCAAGATATCGATTTATCAATGTTTTTCAATTTTAAATAATATTTTCAAATTTAACATTTTAAT
AAAAAGAAATTCTACTTAAACAAAGCTCTAAAATATATATTTCTTAGTTCCATTTTATGTATTGAAAAAC
AAAATAACAAAATATAACTTCCATTGAGATAATTAGCAAAAGAGTTTGGGTGGTTGAAGTAATTTAAAAG
AAAAGCAATTAAGTTGAACAAAAGTAGAAAGAAAAAAGAGGATAAGAAGAAGGAGAAAGAGAGAGTTGGT
AAATATTGTGTCCATTTATGAGGAGAGATTCAATGGGACTAAAGACAATGTTATAAAGGGTCAAGTGAAC
AATAAACTTTTCACAATGTGTTTGAAGAAATGACTCAAAACATTGGCCCTTTTTCATTTTCAACCTCTTT
TGGGTTAAACGAGTACTTTGCTTTTAAATCGGTTAATATTTTCGGTTACGTTTTTAGGCTTTAAAAGTTA
AATTACTTATAAACTTTGAAATATGTTATAAGAATGATGAATTCTTGAAATTTGATTTTATTTATAAATT
TTTGATGACACGTTTGTTGTATACATACTAGATGTCCGGGTGCATCTATCGACTCAACGTATCTTATTAA
AAAAAAAAATTCGTTTTGTTTAAAATATCTATGTTTTTCAAGTTTTACGTATTATATTTACTATAGTTTT
TGGATCTATAAATGTGAAAATGTTTTTCTTCTTTTGAATCTCCATTGATTTTTCTTGAATTTTCTTAGAA
CTTTTGGTAAATTATATTTAGCAAAAATTTTGTTGTAATTGAAACTCTGATTGATGCAAAATTATACTTT
ATACCAATGACAACTTATTCTTATATTAAAAAAAGAGACCCCTTAGAAACTAAATAAAAACATCTAAAAT
TTTATAAGGAAGATTAAATAATTTTAGAAGTCAAGAGAATCAATGTTGATGTAAGAAAAAAAAAACATAAA
ATTTTAATTTTTTTTTATAACAACCTAGTTGATATTTTTTTAAAAAATAAATCAGTGTAGGTTGTCAACT
TTCTAATATTCTTTAGTATAATTAGTTGGCAAAATGTGGAAGACCGAATCGATGACTTTAAAGGAGAAAT
TTTATGTCAATTAATACCACTGTATTAAGCCCTCTTAAGTTAACCGTGATTTATTTATTGTATTTTTCAA
AACATCTAATAAGAATCATTGTCCAATTTAATTAAAAAGACATGCAACGTGGAAACTCTAGCTTTAAGAA
TTTAGGTAGGATTTCATGCTTTACTTCAAATCATCATGTTAAACAAGATTATAGTTTATGAAAAAACATA
AAACTAAAAGCAAAATCATAATCAAAACAAAAGTTGAGAAATGATCGATTGTTATGAAAGTTATTCGACA
TGTAAGCCTACATAATTAATTTTTTTTTTGTTACTTATTGTCTAACTTTTGTTGTATGTGCCATGTGATA
AATGGCAAAGAGGAAAATGATGAATTTTTTCATTTAAACAATTTTCATGGTGGGACATTTCACCATGAAA
TTTTGAAAAAAATGGTTATTAGTCTTTCATAGAGGAAGAAGACAGCCTCAATTCTCAAAAAGACCTATTA
GGGTTTATATATATATTATTAGTGAAGATTCCACTTTTTACCTCTTCTTCTATTTGTCTTCATCTTATTG
TTTTATAGTGAAGAAGAAATATAGGTTGAAAGATTCCCAAATCTCTATCTCTCTCATATCATTTGAAAAC
CCTATAGTTTTTTTTTCTTGATATAAAATTTACATTATTTTAAGAGAATATATATATAAAAATACACAAA
ATGATGAGACTTTTATGTTTCTTAATACAAAGAAGAATACAAAAGAGAGTTTCATAGATTATGTTTTGCT

CTTTACAAACATGCCAAAAATTTCATTAAAAAAAAGGACATATAAATGAAGATATATAATGTCTTTTTCC
TGTAAAAAATAAAATAAAATAAAATGAAATAAAATAAAATAAAATGAAAGAAAGAAAGAAAGAAAGAAAG
AAAAAAGAAAAGTGAAAAGTGAAAGGAGAGAGATAAAGTCATAAAACCATGCATGGAATGACAAATGCCC
CATAACACCTCAAAAAAAGAACCATTAATATATTGAAGGGGAAGAAAGAGATTGATTACTATATTATTTT
GATCTTAATAATGTTTGATGGAAGAGTGTGGAAGTTGATAATTGGGTTTTTGAGAATGTCTCTTTATCTA
CAAAAACATTTTGGATTGTTTTCTTTATCATCAATATTAAATAGATACTACTGATTATATATATATACAC
ATATAAAAAGTCATTTTGTGACTCTATATATATATATATATATATATATATATATATATATATATATATA
TATATATATATATATATATATATATATAAAGTCATTATGTTAATAGTAAAAAAACTCTTTTCCAAACTAT
TTAGAAAGTGATTTTAAAATGTGTTAACAATATTTTTATTTTATTTGACAAATTATTTACATTCCATAGA
AAAGTCAAGTGTAAAATTTTTTATCTTTTATTAGTAATTTTGTTATCTACGGTGTAAATAGTTTGTCAAA
TTTCTTTTTTCATTTAAAAAAACCCTATTTAGAACTTGTTTTTATATATTTGTGTGGTATGAAATTCAGA
AACTGATTTCTAATTTAAATAATTATTTAAAATGTGTTTGGTATAGCAAAACTAGTTTCTTCTAACTATT
GCTAAATATTAATCGATAATAAACTATTTGTTTATTTATTTATTTATTTATTAACACGTTATTATAAAAA
AAATTATATTATAGTTTATTATTTTGTAATGTTATATAACTTACACTATATTATATTATTTAATACATAT
TTTATTTTTTAGAAAAATGAATTGAGTTATAACCTAACGTAATATATTTTTAAATATTTTCAACTTTAAT
TAATTTATTGTTGTTGTTTAATTGATAGCATCTAAATGAATTTATGTAGCAATTGTTAGTTAAATATTCA
AAGAATGAACCAAATAAGGAAACAACTAAGTTAGACTAAGATTTTTCTAATTTGCAATTCAAATAATATG
TATATGATAGCATGCCCACACCCTCAAAACTCAAATGATAAAATAGAAATCATAAACTAATCGATAGCAT
GCCCACACCCTCAAAACTCAAATGATAAAATAGAAACCAACGTGAGTTTATGATGTAATCGAAAGTGAAG
AGTGCAATGCCTTGTAAAGACGATATTAGCGGATTGTTCGGTAGTAGAGACATATTGTAAGAGGACTTTA
ATGTTGCTTAGATACGATGACAAACAAAGTAATAGTTATTCTCAATGTGGTTGTTGAATTCATGAAATAC
ATTATTGTGTGCGAAATATGGATATATATAACACAACAATTGTAACAACAAAGTAAGACTAGCATAGAAA
CACCCATATTAAGTTAGAAGCCAACAAAACCATACTAATTTAGAAGTAGCATTAACAAGTGTACAATATT
CAAAATCTATACTCGAACAGGAAGCAACAAATTGTTTCTTATTTCACTGAGAAATAAGGGGTTGGTCCAA
GTACAAATAATAATTAACCTGGATTGGAATGATGTAGATAATTATCTATTTAATATTCATGATAAAATGA
AAAACAATCCAAGAAAAGAACATGAGTAAAGCGTATGGTTCAAGAAGAAATCATGAATCAATAACTAATA
ATATGAAAACCTAAGCAATATTTAGGTGAGTTACAATCAAATAAATAAGACTACCAATAAGTTCTTGGTG
TCATGAACAATAAAAGAAAAAGAGAATAACAATATCTTGTGAAGTATAACAGACAAACAACGTTGTATCG
TGTTGATCTTACGAAAACCCAAGTTGAGTTATAATGGAGGATTGGATGTGACAAGTTAAACCAAGCTGGT
GCAATTTGTTTCTGACCATAATAAGAAAACAATTACATTAGCCTAAGATTTTGATGCTAATTTACAATTC
AAATAATACATATATATGGTAACTAAACTAATTCTGCATGTTAAAAATTAAAGTTAAAAATTTTGATATA
ATCAAATCATTGAACGGTTTGGATCATATAAACTAGAAAAGAAATTTTAGATCCAAAATATGGGTTGTTT
GTCGAACTTATATTTGTTGAAATCAATGAACATAAAAACATAAAATATAATTTATATTATCAACCAAACA
AATTTTTAATTTTTAATAGAATAATTTATTTTGGAAAAAGAATTTATAAAATTTCAATCGGTTGGGTTTC
TTTTTTTAATATTGCTATGAAGTGTTTGCATTAGATTTTCGGAGAAACGCGTGTTCATGAAGTTGAACTT
AAATTTATGTAGCTGGTTTATGAAAATACAATTTGATTTCTAATACTTGATTTGAGGAATGTAGAATGTT
AATGTTGTTCGGTCTTTGGGAGTTTGAGAGGGTTTTTTGATAGTTGGAAGAATTTTATAAGGTTTTTTTG
AATTGTAAAATTTTTTATCTCTCAGAAAAAAGAGAAACTCCTTTATTTATAATAAAGTTTTTGTAGAGGC
CATGGTGGGCGTGAGCTTAATTTGCTCATAGACTTGGCCTTAATTAAATGTTGGGTTTGGTTGACTTTCG
ACAAAAATAAGCATATATTTCGTCTCAATTGATCCTTGAGCCTAAATACTTATAATAATTTTGATAAAAT
TTAATTTATTTGATCCAACGATCATGACAAAGACCACAACATATTGTTGAGATCATCTTCAACTTTAATT
TTTAGCACAAGTTAAATTTTTTGATTAGTTCTAAATTGGATACATTGTAATTTCATCGTTAATTTCAAAT
ATGAAAATTTGTAATTAATCCAAAATTTATCATTTAACCATAAGGACTTTTAGTACTAATGACCATTTTA
TATATATATATACATATATATTTAGAAAAACATGATTATGTAAATTAAATGATGGTTTTTTGCGAGTAAA
ATCTAAATGGTTTGTTTATGTTATAGATTTTGAGGCCATGAGGTGTTCAACTTTTCAAGTATAAAAGTAT
GCTATTAAATTATTGAAGGAAAAAAAAAAAAAGAAAAGAGAGAAGAAATTAATTACTAGATATATATATAT
ATATATATATATATATATATATATATATGTATATGTTACCTAAATGAGGGACAGTTGATTGGAAGGGATT
GGCTTTTTTATTTAGAATTAATGCTTTTGATTACAATAATGCTTCTAACTAAATCAGCCCATTGGTTTTT
TATTTTGTGCAATAATTTAATTATGTGCCTATACATTTTAATTTAATTTAGGCACCTACCTACTTGATAA
TGTTTCTTTTTACTTTTTCTTTTTCAATTTCTATTTTTGGATAATATATACTTTTTAATCGTTCCTTTTT
GGAAGATTTGACAATTCTTTATGTGATTTTATTTGTTTACTCTAGGGCTGTTTTAAAAATAAAAAAAAAT
ATATATTTATCAGAGTCGTCTATCAAAATCTATCGACAATTTTATCATTTAAACACAAATTTTTGTAAGT
ACGACAGAAATTAGTAGTTTACTTATGAAATACATGACATGCATACAAATACCATTAAAATTATATATAT
ACAAAGAAATAAACATTTTCAATAAAGAAATTACATCAGATCACAAAACAATTTAGAAAAAAAAAAGAAG
CTCATAACACATTTTCTTTTGCATATTAGAAATATGAAAAATATAACAAATAATTAATGATATTAGACGA
CTATCAAAGGGTTATTAAAAGACTATCAGACGAATAAAATAAATATATGACAAATTGATATACTAATAAT
CCATGTGATATTTTAAGTTATTTGAAAAACATTTTGTTTCATCTATTTTCTCTAAACTACAATATTCATT
AGTTTCGAAAAATTTAACTTCTAAATCATTTTAATCGAAGTTGTAATAATATTTTATCGAATTTGATCTA
CGTCATCTTTAGCTCGTTTTGTTTACCATTGTCATTTTACCATAAGTAAAATGAAAAAAAAGAAAAATTC
AAATATATGATTTAAAACAGATGGTACAAAATTTATGATTAGGTATTCAATATAATTTTTACAAGACCTT

TAGCTGACTTTTATTTTAAGGCATGCATGTCCATGTCCATGTCCATGTCCCTCTTTATCTTTTTCTCTAG
TGATTTAATTAAGAGGTGGGCTTGATCGATCCACCATAATGGGCCATCGTTTCTTCGGTTGATGGCTTTA
TATAATATAATGGTCTTTTCATGATTTGTGTAAAGACATATGTTGATAAAGATAACAATGTGTGAACGAG
GACAATTATTTGTTAATTTAACGTTTAGTTCAACTAACACTTATATGTATTTGATTATCAAAAGATTTCA
AGTTTACATTTGTATATTATCAAGAGATTCCAGGTTCGTATTTGTCGTACGAACTACTCTCGTAAATTTT
CTTTGATCTCTTTGAGGTTGGAACATTTTTTCTTTTTAATCTTCGTACATATTGTGCTAGCTCATACATG
TTTAAATGAATTTTTTATATGTGTAAAAGTTATAGATTAACAGTTTAAAAAAAAACTATTAACGAAGTTA
AATTGATTCACTTGAATTATAAAATTTAAGTAGGGAGTTTTAAATGAAACAATATTTTTTTTGGTTGTTT
CCCACGATATGTTATTTTTTTTATATATGTAAAAGGATCGGATCAACTAAGAGTATTACGTCGTAAATAA
GAGGAAAAATAAATCAAGTAACCTGCGTTATCCTGAGTGTACGTACGTGTGTTTCTGTGTAGAATCAGTT
CAAACAACAACATTTGGGAGATCGAAACATGATCTTTTGAGATCGATGGCGCAGAGTAATTAAAAAAATT
TATATAGAGGACACTATATATATGAACTAGGATTGGCAACAGGGTTAGGATCTATTAACCTCATCTCGTC
CCCTTGGGGTATTTAACCTCATCCCTGCCCTAGCCCCATCATTTGAAGGTGAGGACGGGGGCATGGATTC
CCTATTGAGGGGGAACGAGTCCTTGTGGAGACCCATTCCTAGATTTCTAAGTTAATTCATGCACTATCTA
TTACTTACTAATTTTAAAATTTAAGGTTTAGTTAGAAACAAAATTAAAATTTTGTAGGATAAGTTAGATA
TTAAAATGCCCCTAAATTAAAATTTTGATATATATTAAAATATTTTAAATTAAAACTAAGTTAACATAGA
AAAAAGAAAGACCAAGACTTTGAGGGAACCTTTTTTGAAAGTCAATAAGAAACAGACAAGGAAGGTTGTT
GGAAACAAGAAGTCACCAAAAATATATATATGAGAGAAACCGAGAGAGATACCAAGAAGTATATTAATAA
GGTTTTGACTTATCTACCTCTACTTTTCCATTCCTCAATATGAATCAAGCAGGTAGATCATCTTCTTCCT
CATGTTTTAGATGGAGTTTCGATGTATTTTTAAGTTTTCGAGGGGAAGATACTCGATCCAACTTCACCAG
TCATCTTAATATGACTTTACGTCAAAGAGGAATCAATGTCTTTATAGATAAAAAGCTTTCAAGAGGTGAA
GAAATCTCTTCATCTCTTTTGGAAGCTATTGAGGAATCCAAAGTCTCAATCATTGTAATTTCCGAAAGTT
ATGCATCTTCGAGTTGGTGTTTGAATGAATTGGTGAAAATCATTATGTGTAACAAACTGAGAGGACAAGT
TGTTTTACCAATTTTCTACAAAGTGGATCCATCTGAAGTAGGAAACCAAAGTGGAAGATTTGGAGAAGAA
TTTGCCAAACTTGAAGTTAGATTCTCGTCGGACAAGATGGAAGCATGGAAGGAGGCTCTCATTACTGTTT
CTCATATGTCTGGATGGCCGGTTCTTCAAAGAGAGTATTCTCTTTCTTCATATATATATACACTTCTACT
TCTTCATATATATATATATACTTCTACTCTCTTGCTCTTCATATTCTATTGTTACTATTCTTAGCTTGTT
GGTCAATGATAAAAAAGTAGTAGAAGAAGAGTGATATTATTTATTTTAGGTTCAGTTTGATAACATTTTT
CGTTTTGTGCATCACACATTGTTCGAACAAAATTTGTTTTCAAAGTGGTACAACTACGTCTTTGAGTGAA
AACAAAAACAAAACAAAATTGTTTTGCGTAATTTTCATTTTTAAAATTTATATGTCTTTTATCTTCTAAT
TTTTCAATTATGGTTTAAAAGAAAAATTCTAAAAACAAACAAAATAATGATTTTTAAAACTTAGTGTAAA
TTTTTTAAAATAGTAAACAAAATGGACAACAAATTATAGAAACTTATGGTGGGAAGGTAAGGTTTGAATA
CTTATTTTAAAATATTAAAAACCAAAAACCAAATAGTTATCAGGTAAGTCTTAGAGATTTTATTTTTCAT
TATCTTATTTATCATGTCCTTTTAATCAAAATTTTGTTAGAAATTTACACGAGATATAAATAGATGCAGA
ACAAACTAAATTTTATCTCAAACTCCCACTTCATAAGTTGAGAGCCAAATAAGCAGTTGATCATAATTTT
TAATAATAAAACAAAGATAAAAATGAGAATGACACCGAAAATTTTATTGGTTCATTTCAAACTCAAAACT
ATATCCAAGTTTTGTGCAAGGTTCTACTATCATGGAGATTGAAAGAAAACCTTGAACACGTGTTTACAAA
TTTCTCTTTCCAAATAGACCAACAAAAGCTTATTTGATACCATTGACTCGATAACTTGAAATAGGGTAGT
CCTTGACTCAAAATAGAAAAATACACACTGTAAGGATGTGCTTGTAGCCACTTAATTTTGTCTGACATTA
TTTAAAATCAATTTAATTGTTAGATTAATTTTTGCCATAATTTGGTTTATTTTTAATTTTGTGATTAACT
ACCTTTTTCTAAAAAAAAAAAATGTTATTTAACTACTTTTTAAGTTTTGTAGGTAGTTTGGTTAAAGTAAC
TAAGCCATTTAGTTAATTAAGGTAACTGTAGGTAGACAAATACCAAAGATGATAACTTTTTTTAATTGAT
TTTGGTTATTAAAGGCTCTATGCCATTTTGATATGAACGCTAAAGTTGGTATTATTTTCAAATGGAAAAA
ATCCCACCATTTTGAATTTTTAGCCATTTGAAGAAGTGATTTTGGCTATAAATAATATCTTCTTCTTCAT
ACACACAACATCACATAATGAAAAGTTCACAAAAAAAAAAAAAAAAAAAAAACCTAGAAATTTTTACAACA
TTCACATCCTCTCTTCATCTAAATCTCTAACCTTCATAGCCCCTACTTGTAACCTTTATATCTTCTTACA
AAAACCAAAAGATCTACAACATCCATTATTTTATCTTCTCTAAATCTTCATCTATCTTCCTACGATGCCA
ATAATTCAACACACAAAAAAATTCTAACTTAATTGGAGGTGTATGGTAGGGCTTTGGTTTCAATGATCCA
CACATCAAACAACAAGTGAATTTAGTGTGTTGGGATTTCAATCTCTCTCTTTTCTTTTTTTCTTATTCTT
CTTATTTTTTTTTAATTTTTCTTTAAAGTTCTTTAATTTATTTTTTTCTTAGTCTTATTATTCTCATTAT
TTTACTTATCTTTCTTCAAGCTACCATGAAAAATGCACAAAAAAAAAAAAAAAAAAGAAAAAGAAAGAAA
GAGAGAGAGAGCAATGATTAAATATTTGCTTATTATTTTTTGTTCTTATTTCTTTAGTCTCTTATAATTA
TTTATTTATTATCTTGTGATAGTTTTATTTATTTATTCATATGTTGTTATCTTTTATTTATTTATTATCA
TGTTTTTCCTTTATTAATTTATTCTTAATTTTTATTCTTTATATATATATATATATATATAATTAGCATTTT
TATTTATTTAATATTTTTCTCATCTTTATTATTGCTTCCTTAATATGTTCTTGACTTTTATATATTATTT
AATATTGTATTTTCTTTCCTTCTTTTGTGATCAATTTAATATGTTATTGCTAATTTGTTTTTTAAAAAAA
TTGTTAATATTTTAAATTATTTTTCTTTAAAAACATCCAACGATGGAATATAGGAAATTTAGGAGTCCGA
TTTCTAAGAATTCTTGAGGTGAGAAAATCGATTCTTTGGAAGTTCGAGATTGATTTTCAATATGTTTTTA
TTAAAATCTCAATATGTTATTTTGTGTTTGCATAATTTGTCATGATGCTTCTTTACTCTTCTTTCTACTT
ATCTTATTTTCATATTAAGTCTTATTTTATTTCCTATTTAAAATTTTCAAGTTTTAAAATCCTTTCTAAT

TTAAAATTTTCCAAGTTTTAGAACCTTTTACAAAACACTTTTTTTTTTTAAATCATTTAGAGTTTTTTTC
TTTTAAAGTTATAATTTTTCACGTTTTTCAAATGTTCAAAATTTAATCTATGATTTCATAAAATTTTTTA
ATCAATTTTTTAAAATAATTTATACTGTTTTTTTAAACAAAATCCTACGACGGAATTTTAGAAATTTGGG
AGTTCGATTTTCTAGAATTCTTGAGGTGAGGGATCACATCTTTGGGAGTCCAAGATTTGTTAAAAAAAAT
AAATTTTTAGTTAATGTTTTAAAAAAATCTATTTATTAGAAGGTTATTCCTACGATGGATTTTGAGAAAT
TGTACTAATTCCTCACTTTTTTGAGGTGAGATGATTTTCTTCAAACATTGTCTAATTAATGAAATGTTTT
CCACTAGATCATTGTTCAAATATTGGAGTAACGAGGGGTAAAATAAGACATTAGTTTTTAAAGAAATTAA
AGAATATTTTCAATTCAAAATTTGGAATTTGTTCACTGTTTTCTAAACGGATGTTGTGGGGTGCTAACAC
ATTCCTCATACACAAATGACTCTCGAACTCAACTCTAGTATTTGCAGACCATTTTTTAATGATTTTATTT
AAAAGGTTTTAATATATTTCGATGTCAAATCACACCATAAAAAAGATTGGTGATTACTCTTATTTTATTT
TAAAAAACAAACCCATTTTGAGGACGTCGACCGTTGTGCGTCGTCTCGGGCACGTGGCGACAGTGCTTAC
TATCACTCTAATCACGACCCACAAATTTTTCTTTCATCAACAAAGAAATTGAGCTCGTCATACACCCACG
CAAAATTTTCTTGCAAAATAAAGCTCACGGCGATGTCTTCCAATTTTGCTTCAAATCTTTGCTTTTTATA
AAGAAATCCACCATATATCCACAATAACCAATTAACTTATTTCATTATAATCCACCTTCATGCAAACCAT
TCAAATGGTTCCTCATGCTTTAAATATTCAAATGGGGCTTGGTGTTTTGGGTTCCAAATTTCCTAACATA
TTTTTTGGTTGGATTTTGTTTGAGTTTATTATATTGTTGCTTTGATTTTTTGGCTTCTAATTTTGAGAAC
TATTTGAGGAAACAAATGTGTATTTCAAACAAAAATTTAGTGTAGTTTTTTTTCTTAAATGACAGTGACG
AGGCTAATTTGATTCAAAACATTGTTCAAGAAGTTTGGAAGGAATTAGATCGTGCAACGATGCAGTTGGA
TGTAGCTAAATATCCAGTTGGAATTGACATACAAGTTAGGAATTTACTCCCACATGTGATGTCTAATGGA
ACTACTATGGTTGGATTATATGGAATTGGAGGTATGGGCAAGACAACTTTGGCCAAAGCTTTATATAATA
AAATTGCTGATGACTTTGAAGGTTGTTGCTTTCTGCCAAATATTAGAGAAGCTTCGAATCAATATGGGGG
CCTTGTTCAACTTCAAAGGGAGCTACTTCGTGAGATTCTAGTGGATGATTCGATCAAAGTTAGCAATCTT
CCTAGAGGAGTTACCATCATAAGAAATCGACTATACTCAAAAAAGATTCTTTTGATTCTTGATGATGTTG
ATACACGTGAACAACTACAGGCATTGGTGGGAGGACATGATTGGTTTGGACATGGAAGTAAGGTGATTGC
GACAACAAGAAACAAGCAATTACTTGTTACTCATGGATTTGATAAAATGCAAAGTGTTGTTGGATTAGAT
TATGACGAAGCTCTTGAGCTTTTTAGCTGGCATTGTTTTAGGAATAGTCATCCCTTAAATGATTATTTGG
AACTTTCAAAACGTGCAGTCGATTATTGTAAAGGTCTTCCCTTAGCTCTTGAAGTTTTAGGTTCCTTCCT
TCATTCTATTGATGATCCCTTCAATTTCAAACGTATTTTGGATGAGTATGAAAAATATTACCTTGACAAA
GAGATTCAAGACTCTCTTCGAATAAGTTACGATGGACTCGAAGATGAAGTAAAAGAAATATTTTGTTATA
TTTCTTGTTGCTTTGTACGAGAAGATATCAACAAAGTTAAAATGATGTTAGAAGCATGTGGTTGTATATG
TTTGGAAAAGGGAATAACTAAACTTATGAATCTATCACTTCTTACCATTGGTAGATTCAACAGAGTTGAA
ATGCATGACATAATACAACAAATGGGTCGAACAATTCATCTTTCAGAAACTTCTAAATCTCATAAAAGAA
AAAGATTGTTGATTAAAGATGATGCTATGAATGTCTTAAAGGGGAATAAGGTAAGAATTATCGGTCAAAC
ATCATCATTTTTATTATTTTGAAAATTAATGACTTGAAATGTTTGTTTTGCAACCTTTTGTAGGAAGCAA
GAGCAGTCAAAGTCATAAAATTTAATTTTCCTAAACCTACGGAGTTGGATATTGATTCAAGAGCTTTTGA
AAAAGTGAAAAATTTGGTAGTACTCGAAGTTGGCAATGCCACATCTTCAAAAAGCACTACTCTTGAGTAT
CTACCTAGTAGTTTAAGGTGGATGAATTGGCCTCAATTCCCTTTTTCATCTTTGCCTCCAACCTACACAA
TGGAGAACCTTGTTGAATTGAAATTACCATATAGCTCCATCAAACATTTTGGGCAAGGATATATGGTATT
TATAATATAAATTATTGCATATGTTTTTAATTTCTATGTTGAAATTTGATTGTTTACTTTCTTCTTATAT
ATTCATTGCAGAGTTGTGAAAGGTTGAAGGAAATTAATCTTACCGACTCCAATTTTTTGGTGGAAATTCC
AGATTTATCTACCGCAATAAACCTCAAATACTTGGACCTCGTAGGATGTGAAAATTTAGTAAAGGTTCAT
GAATCAATTGGATCTCTCAATAAACTTGTCGCACTTCATCTTTCTAGCAGTGTTAAGGGCTTTGAACAGT
TTCCATCGCACCTCAAGTTGAAATCTCTTAAGTTTTTGTCAATGAAAAATTGTAGAATAGATGAATGGTG
TCCTCAATTCAGTGAAGAAATGAAGTCTATAGAATATTTGTCCATTGGGTATAGTATTGTAACACATCAG
CTATCTCCAACAATTGGATATCTTACTAGCCTAAAACATTTGACCCTCTATTATTGCAAAGAGCTTACAA
CCCTTCCAAGTACAATTTATCGTTTAAGCAATCTTACTTCTCTAATTGTGTTGGATTCTGATCTTTCAAC
ATTTCCTTCCTTAAATCATCCGTCTTTACCTTCCTCACTTTTTTACCTAACCAAGTTACGTCTTGTAGGT
TGCAAGATAACAAATTTGGATTTCTTAGAAACAATTGTTTATGTTGCCCCTTCATTGAAAGAGTTGGACT
TGTCCGAAAACAACTTTTGTAGACTACCCTCGTGTATTATTAATTTTAAATCCCTGAAATATCTTTATAC
AATGGATTGTGAGTTGCTCGAAGAAATTTCAAAGGTTCCAGAAGGTGTAATTTGTACGAGTGCCGCAGGG
TGCAAATCATTGGCTAGATTTCCCGACAACTTAGCTGATTTCATATCTTGTGGTAATTCTGCGGTGCGTA
CCATATCTCTTTCTCATGACTTCACCATTATCTCTAGCTCATGTATATTTAATTTCATTCATATAATATA
TATTACTTATAACTATTTACTGATCTCATGGTGCAGGAATGTTGTAAAGGTGGAGAATTGAAACAACTGG
TATTAATGAATTGTGATATTCCAGACTGGTATAGGTACAAGAGTATGAACGATTCATTAACATTCTTTTT
GCCAGCTGATTATCCAAGTTGGAAATGGAAGGCTTTGTTTGCTCCTTGCGTCAAATTTGAAGTTACTAAT
GATGATTGGTTCCAGAAGCTTGAATGTAAAGTGTTTATCAACGACATTCAAGTATGGAGTTCTGAAGAGG
TGTATCCCAATCAGAAGGAACGAAGCGGGATGTTTGGAAAAGTATCACCAGGTGAGTACATGTGGCTGAT
AGTACTTGATCCTCATACACATTTCCAATCATATTCCGATGATATTATGGACAGGAGGTCACCGAAGATT
ATTGATCTAAATCAACCAAGTTTTGGGATTAATTCCTCACAAAGTATTTTGGGTAAAATTACGGTGTCCT
TTCAGGTTACTCCATGGTATAAAGACGTTGTAAGTATAAAAATGTGTGGTGTTCATGTCATCATGTGGGA

ATGAAGATTATACACCCCTTGATCTTTGTATTTACATATTGTATGTAAAAGACATTAAAATTTTCCTTGT
GTATATAAGATTTAAATTTTCACACGATTGATTTGAATCATTTAATTGTGGTACTTTTAAAAACTTTTCA
TTTTAATAATCTCTTAGCATTTTGGTTATGTTTAACATCTTGGCTATTTAATTTTGATATATTTTGGTGG
AGGACAAGTTTGAAATAAAGGAATTTGAAAGAGAAAGAAAAACATATTGGGAAAGTTTAATTTGTGTTAA
AGGATTAGTAATTGGAACTTTAAAAAAAAAATGGAAAATGAGTGTTTGGGAGATTATAGGGGAGAGAAAAA
AGAAAAGGAGCAACATATATCTCATCTTTCTTTTTAGATAGTATTAGGATGTAATGCCCCCAAAATTCAA
CATTCAAATAGGAATTATTACCAACAACGTAAGAGTACTGAGGAAGAAGAAGACGAAAAACGAGAATCTA
TCTTTTATAAATTATTCTTTTGCTACAGATAGACAGCAAGGGATCATATAAATCGACCTCCAAACTAGAT
ATGAAGAATTAAATGGACCAACTTGATGGCAAAACAACCTCTTTCCTACAAGGATTCCTAAATGGAGTCG
TATATATACAAGAAGCAAGCACTCTGGTTACGTAAGGAAATGAGAAAGAAAGAATGGTTTGTGCCAAACA
TTTTGAATATTCAATTGAACTTTAAATGGACCTTAACAATAATGTGAAATATATATGCTAATATATTTGT
GGTATGTGTGTGTTTTGAAAGGTTTTGAACTATATTCAATTATATATGGCACACAAGATAATAGGTACAA
AATGCATAATGAATATATGTATAGATAGTAGACTACCATTGTATACCAGACGTTAGGAATATCGTATGTG
TTTTCAATACCTATTCGTTTTCATTGATAATAACAACAACAATAATAAAACATTCTTAACATGGCTAAGG
CATCGATGTCTTTATAGATAACAGTCTTTAGAGGGGTGGATTAATTTCATCATTTCTTATGGAAGTTAAT
GAAAGGATCTAGATCTCAACTGTTATAATTTCAGACAGCTATGCATCATCAAGTTTGTGTTCGAAAGAAT
CAGAGAAAATCAAAATTTAGAGGGGAAGCTGTCTTAGCTTTTCTTCAAATTGCATATTAAACTTTGTTTA
TGTTTATTTTATTGGTTGAGCACCTAACTAATTATTCTATTGATTTGATTTAAGGTTGGTTAGATCAATA
CTCTTGTACTTGAAGAATTGTATGTTAAGTCAACCATTTTCTTTAAGAAAAATACACAATCTAGTGTCAC
AATATTGAAATCAAACAAGAAAACGAAGAAAACAACAGGTCATATGCCCAAAATGGCCGCACGGATCAAG
AGATATGTTAGAAGAATGTTTGGGCTCGTGGTTGTTATATTTGTTTAGAATTATCTCCTTGTTGGTTGAC
GTGGCAAAAAACTTTTTATGTGAAGTTGTGAAGAATAATCTTGTATAATTTTTTTTCAAAGTGTTGTTTG
ATTCAAAATCGTGTATAAAATATAAAACATCTTGAAAAAAAAATTGTTGGAGTTCAAAACACGTTCGGGTA
TTAAAGAATTTTGAAAAAAAAATCACGTACCAGAAGTTTTGAGAAAAATCGTTTTGGGGATCATGTACCA
AAAATCTTGAAAAAAGAATAATTTAGATTTCGACGATCGTGTACCACAAATCTAAACAATCGTGTAGACA
AATCTAAACAATCGAAAGACTTTTGAAAAAAAAATCGTTTAGATTTGATATTAAATCTAAAATATAAACT
GACAATTTGTAAACAACCACCGGCATCCGCTCATTTTTAAACAATGTGGAATCTTCACATAATGTTTCAA
AGAAACTTACAATTCCTTAGTTTAAGAAGCCATAATGATGTAAAATTTTGGAGAGAAGGTAGACAACATA
ATTTGTTTCCGGACAAATTTAGCTCCTTCAATGTAGTGTAGAAATTAGATAAATTTTCCAAGAAATCTGC
ATTTGATATGTTGCAATTTTGTAGATCCAATATAGTCAAGTTTGAACATAAGCTTTGTGTTGCGGAAAAT
TTAATCTCGATCCCGAGGGAAACATGTCGAGTCTAGAACACTCATATTGAAGATCAAGTTCCTTATCTCT
TTAACAAATGAATTTCACTTGAAAGAGAAATCAATAACTAAGGTTCAAATTCTCAATCCCAATAAGATAT
CTAACTGATGAGGGTAACTTCCTCATGGCTGGACCTTTCAAATTCATCTCCCTCAAACCCTTCATATTTT
CATCAAATTCTGGAAGTTGTTCAAGCTTATAACAGTTAGTGAGACTCAAAGAATCAAGAGACTTCAAGTT
GATGGCGAGAATCAAGTTTTAAGGCAATAAGTTCATCAAGAGATGAAACAGACTCATAAATCGTTCTTAA
AGAGAAGCAGCTCCAAGATCAAATATCTCAAGTTAGAAATTCCCTTAAGATTTTGACAATAACTTAAGAT
TCGAAACTTAAAGAGATTCGAACTTTAGGTAGCTTGAAAACTTTTCAAAATAGTTGTGAATTTTTTTTCG
ATTTTTTGGCATGAATCAAGATTCAAAACATCAATAGACTCTGACTTGCTGATGTAACTTGAAAGTCTTT
CAAGGTTTTTGCATCCTTCAAGGTCTAGGATAACAAGTTTATCTCAAAGTGCCCAACAGAGTCATGAATT
ATTCTCATATGATAGCATTCTCTAAGTTGTAGTTCCTTAAGGTTTGACAATGCCGGTGCATCCACTTAGA
TTCAAATCTTCAAGAGACTTTAACATGAGAAAGCTACTTGGAAGCTTTTTTAGATTTTCATAACCTTCAA
AGTTCAAGGTAACAAGCTTACTGAGAGAAGCACAGACTCATAAATCATTTTCAAACATTTGCAACTTCTA
AGATATAATTTCTCAAGGTTTAATGTTGCAGAGAAGTCAAGAGTTTCCTTTAATAACCGCCAATAACTCA
TATCAACATGCTTCAACATTTTGCGACCCTAAAACACATGGAAAAAATCATTAAAAACACTTGGAGTGAT
ATCTAGCTAGCACACAAGTAGAAGTTGTATATAATTTAATTACCTTAAATATAATCCCAGAATATTTGTT
GGATACACCATTTATGACTTGTCCAACTAGCCCACCATTCAAAACAAAGCTTATAGGGAAATACCATTGA
ACACTAGATGATGAGTACTCAATCCACTTAATGTTAGATAAATACTTGAATATATTCGTAGGAAATTTTG
CTCTATTTTGAAGGATAAGCAATCTATGATTTCCCATGTTTATAAATGCTTCTGCTTCAACAATTAGTGG
TTGGTTGCTGCGCAAGTCTAGCTATTAGCAGTCGATCAAAGATGCTTTCCTACCATTTCTCTTGATAAAG
GCCCAAGTAGACCACTTTGGAGCTTGAATGATAGCAAGAAAAAAAAAACCCTAAAACCACTCAATCCGTC
GCTGCTGCCACGTTCCTTTGCGTTCGACAGCCACCATAAGTCGTCTGCCCTCCATCGAGTTGCGTCTGCC
GTAGATCTCTTCGTCCGTCTTCGTTCGCCGTCGGTTGTCTCGTACGAAGATGCATCCTGGCAGTACAAAG
ATGTACTAGTATTTGAAGCGAGTTTACTGGAGGCGAAATATGAAGAGAGAAGTGGCAGACTTTGTTAGTA
GATGCTTGGTGTGCCAGCTGGTGAAGGCACCAAGATAGAGGCCAGCAGGTTTATGTTGCAACCCTTGAGT
GTGCCAGGGTGGAATTCAGAGAGTGTGTCTATGGACTTCAATACAGAACTTCCGAGAACTTTGAAGGGTT
ATATATTGATTTGGGTAGTTGTTGACAGACTCACAAAGTCAGCTCACTTCATTCCAGGGAAATCAACTTA
CATTGCTAGTAAGTGGGGACAATTATATATGACTCAGATAGTAAGACTACATGGAGTGCCTGTATCTATC
GTTTCTGATAGAGATGCTCATTTCACTTCAAAGTTCTGGAAAGGACTTTAGCTTGCCTTGGGCACGAGGT
TAGATTTCAGCACAACCTTTCATCCTTAGACGGATGGTCAAATAGAGCGTTTGAACCAGATTTTGGAAGA
TATGCCGCGAGCTTGTGTGCTGTAGTTTTCAGGGAGTTGGAATTCTCATTTGCACTTGATGGAGTTTGCC

TATAATAACAGCTATCAGGCTACCATTGACATGGCACCGTTTGAGGCCCTGTATAGTAGGTGTTGTAGAT
CTCCTATATGTGTGGGGTGAGGTTGGTGAGCAGAGAATGTTAGGCCCTGAGTTAGTTCAGACCACCAATG
CAACCATACAAAAGATTAGGGCTCGTATGTTGACAGTGCAGAGCAGACAAAAGAGATGCTGATGAACGAT
ATAAGGATCTCGAGTTTGATGTGGGAGACATGGTTTTTCTGAAGGTAGCACCGATGAAGAGTGTTTTGAG
GTTCGAGAAGAAGGGGAAGCTAACTCCACATTTTGTAGGGCCATTTGAGATACTTATGCAAATTCTTCTT
GGAATGCCCAAAATACAGGGTTTCTCAGGAGAATCTGCCGAAACCTATTCCCCTGCCCACTCGGTATCGA
ATTCGGTAAAGCCGGTCTGTGACGCTTGCCTCTTCCAACACCCAGCAGGCTTCGCACGCACCTCTGAGAA
GTCTATTTCATACCTCTATACGTCCTCTCCTCTTGGTCTTTTCTTTGTTACCGGGAAAGAGCTGCTGAAG
AACCCGATCCTCTTCTACATTGGGGAGCTACTTGAACCTAGCCTGATTTCGAGATAGGAGATGAGAACTC
TGATTCGGTATCCTACCCCCTGCCTATTGATGTCTCGCTTCGTCCCGAACCCATGATAAGAATACCATCA
TAACGAATACCCCGACCGACTTCTGAGTGAGTTAGGCGCGTCCCACTTAAATGAGTTAGTCGATTCCCCT
TTGCTTCGGAAACATCTCATCCTTCCTATAAACTCATGAATTGAGAATGAGACGGATCTTTCTAATACAT
ATTAGGGAGTTAAGTAACTAGAACCAGAATTGCCTAGATAGGCTAGCCCCTCTTTCTTCTTGTTCTCTAT
ATGTTTCTACTCTGTCGAACTCTTTATTCAAAGTAAGCAAGCTAGCGGAGAATAGCTCTGAGAAGTAGTC
GTATTCACACCTATGGGTTGGTTATCAGCTGATAACCGCGGAGTGGGAGTGTCTCGACTAATAACCTATG
CCTTTGGTGTGCTCGAAGCAGGCAGACAACTTCCTATCATTCTCAGAGTTAGAAAAAGAATTCCAGGATA
GACAGAGAATGAAACTGAGACAGCTGACTCTCTTATTCATCTCCGATCCTCTTCTCTGAACATAAAATAA
GTACGAAAGCCTATCCTAGAAGTCTATTCCATGATCAGATTTGAGATATTATCTCTTTATGGGGGTTCGG
AATCGGATCTCTTTATCAAAGCAGAAGTCAGAGGAAAAATAGTGGCTCGACTTTGACGGGGGGAAGCTCC
CGACCGAGTAGAGCGCTCCTTCGGTAGTGGCTGTCGCTGTCTAACTATTCTCATCGTATTCTCTCTTAGC
CCGAGAAGCCTCAGAGGGAGGAGGCGATACCTGATTGAGTCGGGTCAGATACCAGAGTTGGGGAGGAGAG
ATAGGGTCTTTCTTGAGTATGAGAAAAAGTCTTCTTTGGACGCCCTAGCCGAAATCAAAGACAAGAAGAA
ATTCAAGCCTAGGAAATTCCCACTCAGCGGACTCAAATTCTTCGATCATTCCCCGCCACAAAAACAAAGG
TCCAGGTGACCAGTCCGAAGGACCTATCAATCCATTATTTTGAGAGCCTTGCCTCCTATCCTTATATTAT
GCCTCTCCTTTACCGGTCCGTGGTAGTTGAATTTCTCTTAAGTTTATCTTCTTCTTCCTTTTCTACATTG
ATACGTGGCTTCGCTTCGCTAGGCTTGCTCAACTCAATGAACAAGGGCCTGATCTTTTTCTTCTAAACCT
ACTAAAAGATAATTCGCTAGGCTCGATCTTTATTTGATTTTAGAAGGTTAACACTCGTCCGAAGTAGTTT
AATTAGATTTGCACGGAGCAGACTGATTATCGTAAAAAGGCAGTCGCTTCGCGCCTTGCCTCCGGTGATG
CCGTAGGTTTAGAAGGCATCAAAATAGAAGTTGCACTTTCCCCGTAGCTTATCGTTTGGCGTTGCCTCCA
TCGTTTTCTGCTGTTCATGATATGTTTAATGTCTCTATGCTGAGAAAATATGTAGCAGGTCCGACGCATG
TAGTTGACTTTGAACCATTGTAAATTAATGAGAACTTGAGTTATGAGGAGCAACCTGTTGAAATTTTGAC
AAGAGAGGTCAAGGTGCTTCGTAATAGAGGAATTGCACTAGTCAAAGTTCTTTAGCAAAACCACGGAGTT
GAAGAGGCCACATGGGAGAGAGAGGACGACATGAGAGTCCACTATCCCGAGCTGTTCGAGATTTAGAACT
TTCGTGGACAAAAGTTTCTCAAAGGAGGGAAGATTGTAACGCCCCAAATTAAGGTCATTAGTCTTAATTA
TCTTAAGTTTAATTTTGACAATTGTTGAAATTATTTTGGGTGTTATTTGATTTAATTGTTGGAAATCCTT
GATTTGTAGAAATTAAAATTTATTACATATTTTGAAAAAATATCTGATTAATTGAATTTGAAGTTAAAAT
AATTATATTATGAAGATAATGTAATTATTGAAGGTTATATTTTATTATTTTGGAAAGAGAGGGAGATTTG
AGTTTAAAAAAAAAGATTTGATGAGTTTCAAATGAAGAGAGTGAACATGAGATTTTATTTTGAGATAAAA
TAAGGAGAAATAATAATAGTTACAATATTATTATTATTTCTTTAAATAGGACTTCGGGATGCGTGGGTTA
CTAATTACCAAATTCCTCAAGAAACCCTAGAAAAAAAAGACCCTAAAACCACTCAATCTGTCGTCGCCGC
CACACGTTCCTTTGTTCGACAGCCATCGCAAGTCGTCTGCCCTCTTTGTTCAAAAATTACAATCCTGCTG
TAATTTCCTTAAACTTAGCAACTCTTCTGGACTGATGGCTTTCTAATCTGGGCTTGGCTTAAGCCCTTTC
TATTAGGTTACTTTATATTATATTAATAAAATTGGGGCATAGCATATAGCTGATACTTCTTAACGGAGAA
GCTATGCTTGCTAGGTCCTGCTGTTTCCTATTTCAAAAAAAAAAAAAAAAAAAAATTGCATTATTGTATAA
TAGTTGATATTGCATTGCATATTCTCTATTCCAAGGACTTCTATATAGGAAAATAAGTGTATTCGGTCAT
CATCGTTTTTGAACTCCACATCCCATTTTAAATATAAAAATCATGAACATGTCTTCTATGGGTTTAGACA
ACAAGAGTATAAAATTCTCTCTTACTTCATCTTCAAATGAGATTTATCTTTGAATGTCAACGACGTTATG
AAGAAAAGAAGTGAACATACATCACACTGTAACAAAAAAATTGCTCTTCCATAAATCTGAATATCTTATT
GAAAGGTGAAAATAATCATTGGATGGTAAGTGAATTCATTCATGATCTATGATGCACGGTATTTGGATGT
CCTTCCATTAAGCCTCTTAGCGCTTCTCTACTATCTTCTTCTCTTATATTGACCCTCCACCCTTACAATA
TGACTCCGACCTCTAAATCGATGCTTTAGTTCATAGGGTGCTGTATCATTGCCAAAACTTCTTGAATCTT
CTTGAATGATGGATTTAGTATAGGTTCAGGAGGGCTCGAGCCCTCCTCAATGTTATCGTCTTTATATAGT
ATGAATAAACAAAAATATATGTCCATTAAACATAAAAAGTAAAATTATCAAAAGTTTATTCATTTAATAC
AAAAAAGTACTAGTGAACAAAAAAAAATATAACATAAAATTGTATCTATCCTTTATATGTTGCCCAAATTT
GATTGGCAATATCATTTATCACTTAGGTCGGACCATTTCTCTTAGATGATATCGACTCACTTCTTAATCC
ATCAATTTCAACAACCTCATTGATATTTGTGAGAATGTAATATTAATTTTAAAGTAAGTTATTATGTTCT
TTATATTTAGAATTGAAAGAAACAAAACAAAAGTTAACCTTTAACTCTACTACCTACGGTACTTTAGAGA
AAAAACCCACAACGAAAACCCAAATCCGCGCAAAAAGGAATACGAAAACTATGGATCCGGGCCATCTAGC
GAAGAGGACAGATGAAAACTTTGGATTCGGCAAAGAGGATAAGCGAAAACTTTGGATCCGGCGAAGAGGA
AGAGTCCGACTTGTAGAGGAAGACGATGAGAAGCGAAGAGGAAGAGGATGAGAGAAAGAGGAAGATGATG

GTTATTTGGAGAAGATGATGGAGATGAGGAATAAAAAGAAATTCATGTGGAGAGGAAAATGGAAAAGGAA
AAGGAAACCAATAGAATTTTTTTTTTGAAAAATGAAAGGAAATCAATAGAAAAACACTACAAGAAACTTG
TTATTTAGTGACAAATTTTTAGTGACGTAACAAATTTTTGTCCGTAAAAGGTCATTTATAGCGACGCAAA
AAAAAAAAAAAAAAAGTCACAAAAGATTATGATTAGCGACACATTTTAAATTTGCGTCTCTAAAAGAATTA
GCAACACAATAATAATGTCACTAAAAATGTAATAGTTTTAGCAATAAAAATAGATGTTGCTAAGGGGGCA
GTTTGGTGCGTAGGTTCGGAATCAAATTTCAAGGAATTGAATGTCTGTGGGCTTATTTGATAGGGTTATG
ATGCCTGCAATTATAAATGCATGTGTTTGGGGTGCAGGATTTAGGAATTTGAATTTATGATGCCTATACT
TTGAGTTCTGGTTTTGAATCCTGTGTTTAACTAACCTGTTTTGTTTAATTTAGTTTTTTTTTTTGTGAAT
TGTACATCCTAAATTTATCACCTACCAAAATTTTAATCATTATTTTTTCATATTTAGTACTTCTATTTCA
ATTTCTTAACTCTATTGATTTTGACAATAATGTAAAAATGAAAATTTTTAATATTTAATAACAATAATTT
TGATTACACTTTGCATTAATAGGAATAATCTAATTATGAATATTACAAACCATTCATAAAATATAATAAG
TTGAATGATGTTTACGAAAGTTTCAGTTAAACAACATTAAAATGGTTTAATAGGTTTCAAAATAAGCATA
ACCAAAAGGATTATGCAAAAGCGAAAGTCAAGTAGAGTTACCTTTAGTCATAGTTTGAAAAATGTTATTA
AACTAAAAAATAAATCATGGTACATGTGATATATATTTAAAATTTATATTATATTACCAATATATTAATA
TTTGATTTATATATACTTGAGATCACAATAGATAGTTCATCTATTTTCACCTAGAAAAAAATATAACAAA
ATAAACAAAATCTCCTAAAATGAAGAAATATTAGTTTTTAACGAGAAAGAGAAAATAGATAATTAAAAAA
GATAATTTAATAATAATAATAATAAATAATTAAAAAGAAACTCAAGAAGAAAAGAAAATTGAAGTATGAA
AAATATGTATTTTAAAAAATGTTATTGTGATTAAATCAAATACGTATTTGGAAAATGACTCAAAGAAAAA
AATTTCAAAATTTTATTTTATTAATTTTTCAAAGTTTGCGGCGGTTGCCGGAGTTGATGGTCGGAAGTTC
GCCGATGGAGTTGTCAAAAACGACACCCAATAGTTAGGCGACAATGGTCAACGAGGGTGGTGTTTGGAGT
TAGCTGACGAATTTTTTAGATTGAATTAGAAAAAAAAAAAAGGTAATCCATGGGCTTGAATATTGTTTACT
ATTCAAACCCATGGGGAAATAGTGTAAGAAGCCTTAGTGAAGTGGTCGTTGATCAATGGTGAAGACGGTC
AATGATTGCCGATGTTTAATTAAGACAATTGGTTGGTCGTCAATCATCATGACAATCGATTGCCAAGGGT
CATGACTATTGGTCATTGACCATCATGACAAGAGGGACGACATCAATAATTAACGATAACGATTGATCGT
CGTTGATCAAGATGTTCACTTGACGAAAAAAACATCAAAAGTTGTAGGTCACCAATGATTAAAATGATTG
GTCATTGACGGTCGATAATCGACAGTTAATACGATTCAAAGGCGATAATCAACATGATCCGGAGTTGATA
GTTAAGATGATGGATCATCGTGACCGTTGATCGGTCATGATCAATCTTCATGAATGTCGATCGGTCACGA
TCGGTCATGATTGATTTTCGTGACCGTCGATCAGTCATGATTGATTGTCGTGACCGTCAATCGGTCATGA
TGGTGGATCGTCAAGTTGGCTAACATTTGTTGTCAACGATTTTTAATGATTAATACTAGGACATTGAAGT
GAAATTTTTTCTAAAACATATGACATTCAATTAAAAAAAACATATTTTAAACTCATGGGTTTGATTTCTT
AATCCCAAGAATTTTATTGCAATGTTTCAAACATGGATTTGGCTTCAAATGTCAAACCCCCCTAATTCCA
AATCCATGGGCCAAACATCCTCTAAAAGTCTAAAAAATTCCCCCTATTTCCCTCCTCTTTTATTCATTAA
ACAATTTTAATTAAAAAAAACCCTTTCCCCTACCCATTACCCAACCTGAAAAACTTTCCATTTCCACCTC
CTCTCCTCCATTGCCGAACTATCCGTCTGTACGAACTGAGGCGATGGACGGTAAAGCACGTGGAACAAAT
CTGCACGACGACGCTTCTACGGCCACGCTAGACGAACCACGGAGAACGAATTACACCGAACCATGCGAAG
AACGTCTACGGACAACTGCACGAACCACACCGATGGCCAGCTGCACGAAGCATGCAGACGACCAACTTCA
CGAACCATCCGATGGCCACCAACCTCGAACGGTCTCGAAAGCTGCTTCTTGCGACGATTTTGCCCGAACT
TCCTTGAACATTTATGCTCCACACCCATAACAACCCAAGACATTCCGGTGAGATTCTTAATTTTTAAAAT
TTTTAGGGTAGTATATTTGGATTTTGATTTGTTGAATATTTATTCTCTTAATTAGTTTTAAGATTGGGTT
TTATCCTTGCAGTGTTTGCATAGTCTTGATTAATGTTTAGATTTAAGTAATGTCTTGTTGAATATTTGTT
TTGTTAATTAGATTTAAGTAATGTCTCGTTGAATATTTGTTTTGTTATTGTGTTTTCACTTCTAGGAATG
AAGTTGTGAGTTTAGATTAACAACAATATAATTTTTATTGTGTGTTTAGATTCTTCTTCGATCAATAACA
TAATTAGCTTCAATGAAATTCTTATTATTGTTGTTGTTGTTGTTATTATTATTTTTTTGTTATTGGATTT
TCACTTCTAGGAATGAAGTTGTGAGTTTAGATTGACTTCAATTATGTTTTTGGATTTCTTTCTCATTGCT
TTCTGTTGCAACCATTTTCCCTTTTCTATTATTGACTTGATATGAAGATTTTGATTTTAGATTTTGGCAA
TTGTTGTTTTGATTGCTCTTTCTCTTTGTAAATGTAATTGTTGTTCTTTCAAATGTGTGATTGAATACTT
TATTATTAAGCAATTTAGATATGGCTTTAATTCTAACACCTAAATTTCCAACTTAGGTACTTTAAGTGTT
GTACTTATTATGGATCGAAGTTGGATAAATTTAACAGATCGAGCTTCTAGTGAATATTCTGATGGTGTTG
CTAACTTTATCGACAAGAAGCTTTAAGAAGATTATTATGATGGTGTTGCTAACTTTAAGAAGATTATCAT
GTCAAGAAACTACCTCCAACATGGAACGAAGTTTAGAACAATCAAATATAGTCAACAAGTAATTTTTTCA
ACTTAATTAGTTTGATATGAAGTGTCAGATTATAGTAGTATATAGAAACATGGTATATTGTGAATTGATA
TGACTATCCTGCAGTCATGGTAGTCATAGATTCGTTCTTCTGATGATTTTGGTCATGAATTTGTTGCTTT
CGACTTAATTAGTTTGATCCGTTTGATTAGTATCATAATGCTTTGAAGCCGTTTATTGAGTTTAATTGTA
ATGAATTGGTTGCTTTAGACATGTCCCCCTCCCTTCTCATTCTTTGTTAGAAAGTTTGAGTTTTATCCAA
TTGAGTATGATATATTTAAAGTACCTAAGTTTCATTTTTTGTTTTAGACATGCCTCCCCCATTCTTTCAA
CTTAATTAGTTTGATATAAAGTGTTGGATTAGAGTAGCATATACGAATATGATATATTCGTTCTTGTGAT
GATTTTGGTCATTTGATGTAGACATGCCCCCCCCCCCCCCCCCACACACACACCAATTGTTTTAGAATGT
TTGGGTTTCATAGTGTCATAATGCTTTGAAGTCGTTTATTGAGTTTGATGGTAATGAATATGTTGCTTTA
GACATGTCCCCCTCCATTCTCATTCTTTTTTTTATAAAGTTTGAGTTTTATCCATTTGAGTACGTTATAT
TTAACGTGCCTAAGTTTCATTTTTTGCTTACATGTTTTACTTATTTATGCTGTTATATTGCTTTTCAGAT

TTGACGCACAAATGGACTTCTGATGAATATGAAAAGAGTTTGGTTCGTATCTTATAATTTTTTTGTTATG
CACATACTTTTGGTCCAAGACTAGGTGGAATTTTTTGTTGTGCACAATACTTTTGGTTCGTAGGTGTAAC
TTCTTGGTTATAATTTGGAACTAACTAGATGTAACTTAGTGCTTATAACTTATGTTGACATTATATAACT
ATTTATGAAGATATTGAAGTTTCTTGATATATATTTCTTGTGATGAAGTTGGTGTTATTGCATGTTGCTT
AGTGATAAGTTTTTAAAATTATTATGTTTGACATCTTATAACAGGTTTATATAATTAATCAATTAGATAT
TATTTTTTAATGTAATAAAATTCTTTTACTGACACTTTCAATAACGTCGCAAAATATCATTGATTTTAAT
TAAAAAAAACTGTAGGACTTTTTAGCGGCACAGTTCAATACGTCGCAAAAGCTATTTATAACGGTCTCAA
CGTAACCTTCAGCAACAATACAATTTGTCACTAAAACATCGCCTTTAGTGACATGTTCAAAACGTCACTA
TAGAAAATAAATAATGACACAAATTCTGTTACTAAAAGTAAGCTTTTAGTAAGACACAAACGTCACTAAA
AGTTTCTCTTTTGCGGTGCAAACACTTGCGTCGCTAAAACTTTAACCGACACGGAATCTGTGATAGTCCT
AGTGACGAAGGGCTTTTCGTCGCTTAAACTATTAGCGTCGCATTGTTAACCTTTAGTGATGTTTTCTATG
CGCCACTGAAAATGAAATTTCTTGAAGTGAAATAATTAGAAAAATAAAAAAGAAACCAATAGAAAAATAA
TTGAGAAAAAGGAAAAGGAAACCAGTAAAAATGATTAAAAAAATGAAAAAGGAAACCAACAGAAATAATA
GAGAAATAGGACGGAAGAAACTATTTTTTTTCCCAATAATTGCTTATAAAATGGAAAAGTTTCTACTTTT
TTTTAGAATGTTATCAAACATTTCTATTTCTTAAAAAGTAAAAACAGAAACAAGAAACAAGAAACGATAA
TAGTTTCTAAAGATCTGCCGCTGATCTTCTCGGACGAGTCGTCTATCGAGTGTGACCCGATAATATTTAC
CTCCATTTATATATCCACGAACCGCACTAGAATCATTAATAGCTAGTAATAAAAAGGAAATATTGTGATT
TTTTACAAATTTGTCCTTTTTAGATTTGGAGAGGGTTTTCTCTCCCAGGAGGTCTTCCATTTGACGATTA
CTAAATGGATATGAGTGTCCTTGCATTTCGGAATTAGTCCCAATTATAAAATTGGTTGCATGCAACAGAC
CATATTTGTGATCAGCTGATTGGGCATAGAGTTCTTGAGCAAAGGATTTGACCACATCCCTGTACCTTTA
TGAACATCATAACACAAGAAAAACAAATATGAAAACGAGCATAATGTACTCTAAAAATAATATTCAACTT
ATTAAAACCAAAATAATGGCAACAAAACTTAACACACAAATAACAATATCAACAACAGCAACAAATTAAA
ACCAAATCAAATAATAGGAATTGATTTTGAAAATAAACAAATGCTGTGTTTGAATGGTTTATGGGTAAGA
TAATTAAGAACCTTACATTTTTCGAAGAAACAGTTATGGATCGGATGTACTATTGAGAATGATTTTAGAT
AAACCTTCTTTTAGAGAAAGATGTGTTTAGATGAATCATATTTTTCAAATAGACAAAATAGTGGTGTTTC
AAAACTTTCTAGAACAGGACAACTAATCAAGCCTTTGTTAAAACAATTAGTAATCTCACTCACCATCTCG
GGCGCAATTTTTCCGGTTGATTAAAATATGTGTAAATTGCCCTTGGCTCCTTGACATCATTTTGCATCCC
AGAGACCTTTTCAGTGAAATAGACACCACACCTTCTTATAGTTACGACCTCAGCACATTTCACAGCCTCA
AACCAGACTATAACATCATTCCAATCATTCATTTCCAAGGAGCTCCTACGATATGTTGCAGAAGTTTCTA
CTAACCATGTATATTCTGATGCTGATTTTGGAAATTTTCTCATATAACGACGGTCGAGTCTACAGCCGAT
GAGTATGTTACATGAAACTAAGGCCATTCCTTGATCTGAATCTCCATCCACTCGGAAAGTAACAGATGTA
GCCAAAGTTATTTTTGTATCGCGATTGTGTTGAAAAGTAACCATTATTGCATTTGATGCAATCTGATAGC
TAAACCATTCTGGAATCCCATTATTCATTAGAACGAACTCCCTTATGCCACGAGGACGATTCCTTAACCA
TGCAAAATTGATCTATGGCATAAGAATTATTATGCATGGAATGTTAGTGTAAATGACAAGAATTTGATAT
GAACAAGGAAAATGAATAGGTAGGAGATGAACCTGCTGGCTTGATATTATGTCCAGAATGTTGTTTGGAC
TTCTACTCAACGATACGCAACCAGTGGCATCTACTCTTTGTATACACAGAGGGAGGTTTGGAATTTCTTG
AAGAAACTTGCAATTCCTTAATTCGAGATTCCGCAAGGACATAAACTTATGGAGACATGAAGGTAGACTA
GAGAAGTTGTTTTCCGACAAGAGTATACTAGATAAGGAAGATGCTACATTACATAAAATTTCCAAAAAAT
CAACATTTGATATATTGCAACCTTCAAGATCCAACAATGTGAAGTGTTTGAAACATAAGCTTTCTTTTGG
AACTCGTGAATGGAAAAACTCGGAAGTCAATGAAGTTTCCATAATTTTTGAAGAAGAGCATACTGGGTTG
ATGGTTGGGTCCCATATGTAGGAAAACATTTCAAACCTAGAAGACCCAGAAAGATGAAGCTCGCCAAGAC
TCTTTAACAAATGAGTTGTACAAGGAAGGGAGATGAGGTTTGTGCAACCTTTAAGATCAAACATATAAAG
ATGAGTAAGGTATCCAATTGACGGAGGTAGCTCCCTTATGGCAGTAGAATCCAAGCGCAATATGTATAAG
GATTTCATGTTTTCATCAATTTCTGGAAACGTTTCGAGCTTACAGCAACCAGAGAGTGTTAAATTTTGAA
GAGACTTTAACTTGAGGTAGCTTGGAAGCTTTTCAAGGTTGGAGCATTTTTCAAGGTTCAAGGAAACAAG
CTTACTCAAAGATCCAATAGAATCGTGAACCACTCTTAAACTCGTGCATTGTTCGAGAGACAAGTGTTTA
AGGTTTGATGTAGAAGAGAAGTCAGGAATTTCCTCAAGCTTTTTGCACCAAGAAAGGTTCAAATCTTGAA
GAAAGTTCCATGAAATGTACCTTGGAAGCTTTTTAAGGTTACTGCAGTTTTGAAGTTTCAAGGTAACAAG
CTTAGTCAGAGATCCAATAGAATCATGAATCATTACTAAATTTGTGCATTGTTCGAAGGACAAGCTTCTA
AGGTTTGATGCGGAAGAGATGTCAGGAATTTTCTCAAGCTTTTTGCAGTGAGAAAGATCTAAATCTTCAA
GAGCCTCCCATGAAATGTAGCTTCTTGGAATCTTTTTAAGGTTTACACAATGATGGAGGTCCAGGGTAAC
AAGCTTACGAAGAGAAAGAAATGACTTGGGAATCGTTTTTAAATTTGAGCAGTTGCTAAGATACAATTCT
TCAAGGTTTGGTGCTGCAGAGGACTCAGAAATTTTCTTTAAAATAACTGAGTGTCTAAGATCAAGCAACT
TCAACCTCATACAATTCTAAATACACATGGAAAGAAAAACAAAAAAGAAAAGGAGTAAATACTGATACAA
ATATAATCAAAATAAAATATTATAAAAAAAACAAATACAAATTAGGATATGCAGAATAATTAGGATAAAT
AATGTCTGATAGTTAAATGAATATGTTTAAATTTACCGATTTGTATATATAAACAAAAACTAAATAATTA
TTCAATGAAATGGAAGAAAACAGGCAGTACAAATGTAGCCCAACTAAATTTAATTATACGAGTTGTTTTC
AAATATAGTCAAATATGAACAAAAATATTTACGAAATATGACAAAATTTAGAATGGATACGGATAAACTT
CTATTAATGTTTTGACAACATCCATAATTGATAATATAAAATTTTACTATATTTGAGAAGATTCTTTAGA
TAAAATACTATTATTCAATTTAAAAAGGATTTAGTAACTTCTAAAAAGTTATAAATTTTTTTACGCAAAG

TTATATTTGAAAAAAAAAAAAAAAAAAAAAGAAAGAAAGAAAGAGCACAAAGAATTTAATTTCTTAAAAAA
GTATGCTACCCATTTTAATGTTCTAAGAAATGGAAGGCAAAGGCTTCAAATCAAAATGTAGATGAGGTAG
AGCAATATATTACCTGAAGTCCTTTTCCAAAATTTGTGATGAAGCTATGTTGCAAATCCAGTCCAACAAG
ATCTTTTGTAATAAAGCATGAGGGTAAAGATGGATGAGCAAATCTATGCCACTTTATCCACTTTAACCCA
TTTGGTAGATACTTAATCTTTTTACAAAACCTTACATTTCCATCGACCATAAGTATTCTCAAATTTTTCA
TGCTTCTAAATGCTTCTGGATCCAAATCTATGACCCTTTTGGGATCAGTTAATACTAACTTTATGGCTTT
CACTGCATCGCTTCCCTACAAAGTCATAGATTTAGTAATTTGAACATTACTTTGACAAACATTTTTCATC
ACGTGAAAGTCTAGCAATTAATTAAATTAAAGATAAATTAATTTAGGTAAGAGTAACTTACTGAATTGTT
ACTAAACACCTCCAAAATGTCCTTCTCCAACCATAATCTACTCCTTTTTCCAGGCTGATCATGAGATTCG
TCATGAACTATTTTATGACCCATTTGTTTTATTAAATCATGCATTTGCACCCTATCATCTTCAAATCTAA
TAAGTGAAAGATCCTTGAGTTTTGTAATTCCGAAATCCAGAATCGAATGGCATTCACTCAACATTTTCTT
AACGTAGCTAACTCTCTTGCCCACAAGTAAACAAGAAATATCAAGAAAAATCTCCTTTACTTCGTCTTCG
AGCCCATCAAAACTTAATTGAAGAATATCTTTAATATCTTTTCTCAAAGAGTTTTCAAATCCATCTAATA
TACCACTCCATTCTGCTAGATCTGATCTGTCACAAAGGAAAGAACCCAAAACAATGAGAGCCAAAGGATG
GCCTGTACAATACCTTGTAGCACGCTCTGAAAGGTCTAAATAATTACTCGATGGTTGAAGATTTTTAAAA
GCGTGCCGACGAAAAAGCTCAATAGCTTCATGTTTACTCAATCCTTGAACTTCGTACATTTTATCAAATC
CATGGCTAACAAGTAATTGTTTATTTCTAGTCGTCACAATGATTTTAGTACCCTGACCAAACCAATCATG
CCCACCAACCAATGCTTGTAATTGCTCGAGCTTATCCACATCATCAAGAAGTATAAGAACTTTCTTTGAA
CGCAGTCTATTCTTTATGATATTAATTCCCCAGTCAAGATCGACAACCTCCAAATCATACTTTAAGATCT
GAAAAAGTAGCTTTTTCTGTAGTTGAGTAAGGCCATCGAATAACTTTGAAGCTTCTCTAACATCTCGTAG
ATAGCAGCAACCTTCAAATTGACTAGCCATTTTGTCGTATAAAGCTTTAGCCAAAGTTGTTTTACCAATG
CCTCCAATGCCATATATCCCCAACATGTTAACACATTCAGACTTGTACATTTCTTCTTTCCGATAAAGGA
ATTCTATTTTGGAATCAATTCCAACTAGATGCTCGTTTGCGTTTAAGGGCTTGGTGTGATTTAATATAGA
CAACACTTCTTTAACAATGTCTTGAATAAGATGAGCCTCCTTTCTGTTGTTAATTAGTACACAATCATTG
CAAAATTAATGGATGATGAGAGAACATAGCTAATTAAATTGCATCTTGAGAAAGTAAATCTTCTGAGATT
AAATTATACAATGTGATAATTTCTAATAAATGAAATAAATGGCAACAGATAGCGTCCTTGAATAAAGTGA
AAGTATAAACAAATGATAAAAATATAGGAAAGCAAAAAATATTGACAAAAATAGTTGTGAAAAATAGTGA
ACGAGGTCCACAGTTTCCATAATTCAAATCTCTATTCCTTGTGAGCATCGATGCGTTCAACAACAAAAAT
AACAGAACTCATGGTCCTAGTCCATGACTTTTACTTGTCTATGAGTCATACAGTATTCCTGAGTTCTCTC
TCTTTTCAAAAAACTGTTTAGATTTATAAAACACTAAGAAACAAACATACGACAAAGAGAGGACTATTAA
TACTACGTATATATTTTAAAAATAAAGCAAAAAAAAAAAAAAAAAAAAAAAAAGAAAAAATCTTGCATACC
TCAATATAACCATTCCCTTATTAAAAAATGATCATTAAATCCTCAAAATGCATAGACGACATATTTAATC
AACTTGTTTACAATGGTACAAAATGATAGAAAAACAGAAGTAGAAGCCCAAATCCAACATCCCTCCACAT
ATGCAAAGTAGAAACCCCAATTGAAATAATACTATTTTGTTTTTTTTAAAAAAAAAACAGAATATATTTA
AAATGGGATTTGTAGAAGAAAAGAACAAATAACAGTAAAAACATGTACCTTGCTCCCAGATGCCAACCAG
ATAAGTTGGCAGCAGTAGTTAAAGCATCCCTCCATATTGGAATCTTCTCCATGAAATTAGCCTCATGTTT
GGCCAATCCTTCTCTAAACCAACCATTTTGTTTTCGTACATCCGAAGGATCTACCTTGTAGAAAATTGGC
AAAACCTTTTGGCCCTTTGATTTCTTACACTCCATTATTTCCACCAATTCATCCAAACACCACGTAGAAG
ATGCATAATTTTCAGAGAATATAACAATAGAAATCAAAGAATTCTGTATAGTTTTGAAAAGGGTTTCAGA
AATTTGCTCACCCCTTTCGAGCCCGTCATCTATGAAAACATTGACACCCTTTTGACGCAAGAACATGTAA
AGATGACCGGTGAAATTGGAGCGAGTATCTTCTCCTCTAAAACTCAAAAACACATCATAACTCCAATTGA
AAATGGGAGAAGAAGACGACGATTCGGCTCCAGCAGCGGTAGAACCCATCGGAAATCTACCCAAAGACTT
CCAAATTTCTTTGTCTATGGTCTTCATTCGTCGTTCAATAATAAACTGAAGACTTTATAGAGAGCAAATG
TTGGGTGAATTAGAAGTGGGGACATTATATATTATGATTGATCAGTAGGACTTTTCATTCCCAATTCCTG
CACTTCGGCCTACTTAACCCATTTTTTACGCACTTTCTTTAATTTAGTCAAATAAGCGTATAACCATAAC
TTTTTCTTTGGTTCACCCACAAAATACACTAAACATAATTTTATTACATTTCATATTAACACTTAATAGA
AACTATTATTATAAGAAGACTCGAGGGAAAACGCATTGTTTCAAAGTCAATAAGGAAAAGACAGAGGAAG
GTTCTTGGAAAGAAGAAAAGCACCAAAGATATGATACTAAAAAGCAAGAAATAGAAAAACAGAGAGAGGG
ACCAAAGTATTGATAAGTTATTCAGTTTTCTTCTACTTTTCCATTCCTAGATATGAATCGAGCAAGTGGA
TCATCTTCTTCCTCACGTTTTAGATGCAGTTTTGATGTATTTTTAAGTTTTTGTGGGTATAATTAAATAA
TGTAATTTTTTGAAAAAATTTAAATTTCAAGTAAAAAAAATGATGGGGTGAAAGTAGTGAACGAATTTCT
AAGTTTAATTTGGGCTCCTCTACTCCTAGCAGTGATAGTAATGACAACAATTCTAGCTAATTGAATTGAG
GAGTAAAAGAGTTAGAATTGTTAAAGGTTTGGTTCTGATTTTTATATTTACAATGTTCAGGAATCACCAA
TTTCTTTGCAAGAAAGCTTTAACTTCTCCATATCCATGCAATCTTCTGAAAATTGGCTATGAATGATGAG
TATGTGTTTCTTCGTTAAAAAGTTGAAAAAATATGCAATTTCAAGAAATGTTAAACAACTTTACATTTGT
TACTGAGCTTTTAATTAAATATTCAATATCCAAAGGTTAGTACTATAAATACATCACATATACCATTCTC
TCAATTTACACTAACAAAAATTGAAATACATCTTCGTCCTTGGGCCCAACTCAAAGGTAATTATTTCACT
ATCAACCTTAAATATTAGGGGTGGTTTGCGCCCATTAAGTTGACTTGTCATTTGTCCTACTTGTGATTAA
TATCTGTGGGTCTCTGAATTCACCAATTTCTCATTCTCTGTCTCTTTGTGTTCCTGTAGTTCATACCTAC
ATGTGCTCCTTCCATTCCATGTTCTTATCCCTTATCGTGATATCATTCTTGATATCACTGTGACCAATAT

TACTGAAAACGGTTGAAACTTGTTGTATCAAACAAAAATCGGTCTCTCGGTTTGAAAGCCAATAACGCTT
CTTAATTAATCATTACCTTCTACTTAATTAATTCATCTCATCTTTTAAATTTAAATTGGGTCATTTTCTA
ATCAAAAGTAGTAAAAGAAATTAAAATATTTACAAGCTATAACAAAACTTTAGATTCTATCAAATTGGAG
GTGTCACCATCTACCCGAGACGATGCGGAGCGTCCCACGTCCTTAGAAAGGGGTTAATTTTAAAACAAAA
AAAAAAGTCACCATCAATCTTTTTTACGTGTGATTAGATACCGAAATAATATAAATAATTTTTAATAAAA
TAAAAAAAATGATCTACAAAAAAAAAAACCTAAAATTGAGTTCATGAGTCATTTGTGTACGGGGAAAGTGT
TAGCACCCCATAACACTTGTTTAGGAAACGGTGGCCAAATTTTATGTCTTGAATAAAATTATTTGAATTT
TTTTTATTATATATATCTTATTTTGTTGCTCATTACTTCAAATATTGAAGTAACGATCTCATAAATAAGT
GGAATATATTCCATTAATTAGACTATATTGAGGAAAAATTCTACCACTTTAAGAAAATGAAAAATTATTA
CAATTTCTCAAATTCTATCATAGGTTAGTATTCGAATAAAGTTTTTTTTTTTAAATAGTGATTAAATTCA
TTTTTTCTTGGGATCAAATCTCGGACTCCCAAATATTGATCCTTCACCTCAAGAATATAAATAACAGACT
CCCAAAAATTTTTAGATTCCATCGTATCATTTTAAGAAATCTTATAAAACCAAATCAAATATACCACACA
ATAAATTAGGTAAAAAATTTAGAATATTAATGTATGCATATACTAAATATAACCGATAGTAATGAAAATA
AGTTAAAAGAAAAGGAAAAGAAAGATACATAATAACGATAAATAAAATAAGAAAAAAAAGAATAAAAAAA
GAAAAATAAATAAATAAAATAATAAACAAATAAACAAATAAATATATAGAAAAAAATTAAATAAATAAGT
AAATAAAAGAGAAGGAGATAGAGGAATGACGGTAAGCTAAAAATAAAAAATTATAAAATAAAATAATAAT
AACGATAATAAAAATTAAAAAAAATATGAGTATATAAAAAACCTAAAAAAATAAATGAGTAAAATGTTAT
ATAAAAAAGAAAACAAGAACTTTTTTAAAAGTTTATAAAATAAAATAAAATAATAATATTGATAATAATA
AAAGTAATAAGAAGTTAAAAATAATTATAAATAAAAAAAACTAAGGAAAGTTTTTCTAAATATAACAAAC
TACTTAAATATTTACGTAACAAAATCCATAAATTTAGCAATTTTTTAAATATTCTAAATTTGTCATATTT
GCGTAACTAAATTTATTTCATTTTATTTTGTTTCTTTTTTTTTTTCATTCACCGTATGTATTGTATTGTC
TTTATTCTTTTATTTTTTAAACGATCTTTTCTTTTTTAAACAGATGTATAAAGAATATTGAAAAAATTGG
TTAGACATCTACCAAAACTAAAAGATTGTATATAAAGAATATTGAAAAAAATAGTCTTTCTTTTTTTCTT
TTTTACGCATTGAAAAAATTGGTGTACAAAGAATATTGAAAAAAATTGTTCATACCAAATTTTGAAAAAA
AAAACGTTTAGATTTGACTGATATAGCAAATTTTGAAAAAAAAAATTAGATTTGGAAACCCAAATGTAAA
CCATCGTGTAGCCAAAATCTAAACGATCGTATACCAAATTTTAATTTTGTTTTTTAAAATTTGGAACCCC
AAATCTAAAAAATCGTGTAGCCAAATCTAAACGATCCTCAAGCCAAATCTAAATCATCATATACCAAATT
TTAAAAAAAAAAATGGTTTAGCGAATGTGTACCCAATTAATTTATACCAAATAGCATTAGCAAGTATATT
ACGCGCATTATTGACAGTATATTTTTTGTATTTTACATTGTGGGCCTCTAGGCTTTTTCCATTTGCGGAA
TTGTTCTACACAATATAAATACTTTGTCACTTTTTATATTTTTGAAAAGACCCTAAAAACTAAATAAATA
GAGTGAATAATTTTTTCTTAATTAAAAGTGAATAAATTTCAACTTGTAGAGACCCACCTAAAATAAAATT
TAAACTAAAAAATAAATAGAAAAGGTAAAAAGACTTGGGGGAAGTAAACGTTGGTTGTTGCAAAGTCAAA
AAGAAAAACAGAGGAAAGTTCTTGGAAAGAAACAACCGATATGAGGAGGGAAAAAAAAGGAACAATTTAG
ATCCACACATGTATCGGATGTTGTTTTATAAGCATATATATATTATTGTTTCAGAAACTCACCCACCAGA
TTAATTTGAGTCATCCTTCTTCTCCTTTTCCTTTCCTTTCTGGTTCTTCTTTGATTTATCCTTCAAATTA
CTTAATTAGTATGAATCGAGCAACTGGATCATCTTCTTCCTCACGTTTTAGATCCAGTTTTGATGTATTT
TTAAGTTTTCGAGGGGAAGATACTCGTCCCAACTTCACCAGTCATCTTTGTATGGCTTTGCGTCAAAGAG
GAATCAATGTTTTTATAGATAATAAGCTTTCAAGGGGTGAAGAAATTTCTACATCTCTTTTGAAAGCTAT
TGAAGAATCCAAGATCTCCATTGTTATAATCTCTGAAAATTATGCATCTTCAAGTTGGTGTTTGAATGAA
TTGGTGAAAATCATTACGTGTAACAAATTGAGAGGACAAGTCGTTTTACCAATTTTCTACAAAGTGGATC
CATCTCAGGTAGGAAAACAAAGTGGAAGATTTGGAGAAGAATTTGGGAAACTTGAAGTTAGATTCTCGTG
GGACAAGATGGAAGCATGGAGGGAGGCCATGATTTCTGTTTCTCATATATCTGGATGGACGGTTCTTCAA
AAAGAGTATTTTTCTTTTTCATATATATCTTTCTAACTCTTTAGCTCTTCATATTCTATATATACTTTGA
CTATCTCAAGGTGAGAGCATGGTGGACTAAGAGCAAATGCAAAGAAAATGTGACATTTTAAGTTCCCATT
CGATGACAATTTTATTTCCGAGTTTTTCATTTTTTAAATTTATATACTCTTGTTGTTTATCAAGGAAACA
TTTGAATTTTTAACTTTATATAGTTTTACAGAAAAAACAGAATTTGAAAATTATATAATATAATTGTTTG
AAAATATTTACATTATAACAAAATTTTGTTGCGGTAGACTCGAGTTTATCTTTTTATAAAAAGTAAAATT
TTACTACATTTGTAGATAACTTAGCTCATTATTTTATATTAATTAAGAACACATTACAGCTTCCGAGACT
TATAAGTTGACTTAATTTTTAAAAATATTAGAAGAAGGCAGTAATTAACAAATCATAAAATCCCATGGGT
GAAAATAATGGCATATGTACTTAATTAATTTTAAAAAACAAAAATTCAAATACCAAAAGTTATTTATACA
AAGGCATTAGAATTGATTTTTGTAAAATTGGAATTATTTCATAATATTATTTAATTTGTTATTATTGTTC
TTTCTCCAAATAGATATTACTTTAAAAGTTGGATTTAGTTTGACTTTATAATGCTGCTTTCACTTTCCCT
GGCTTCTAATATTATAGGAATTATAAGTGAATTTCAAAACAACTTTTAGTGTATGTTTCTTTCTTAAATG
GCAGAGACGAGGCGAATTTGATTCAAAAAATTGTTCAAGAAGTCTCGAAGAGATTAAATCGTGGAGCAAT
ACAATTGCGTATAGCTAAATATCCAATTGGAATAGACAGACAAATTAATAATATACTCTTTCAAGTTACG
TCTGATGAAAAAATTACTATGGTTGGATTTTATGGAATTGGAGGTATTGGGAAGACAACTTTGGCCAAAG
CATTATACAATAAAATTGCTAATGACTTTGAAGGATGTTGCTTTTTGGCAAATGTTAGAGAAGCTTCAAA
TCAATACCGGGGTCTTGTTGAACTCCAAAAAGAGCTACTTCGTGAGATTCTAATGGATGATTTAATCAAA
TTTAGTAATCTTGATGTAGGAATTAGCATCATAAGAGATCGACTATGCTCAAAAAAGATTCTTTTGATTC
TTGATGATGTTGATACAAGTGAACAACTAGAAGCATTAGTGGGAGAACATGATTCATTTGGACCAGGAAG

TATGGTCATTGTGACAACAAGAAACAAACATGTACTTGTTATTCATGAATTTGATATATTGCAAAGTGTT
CAGGGATTGAAGGATGATGAAGCCCTCAAGCTTTTTAGCTGGCATGCTTTTAAGCAGAGTTGTCCATCAA
GTGATTATTTAGACCTTTCAAAACGTGCCGTACGTTATTGTGATGGTCTTCCCTTGGCTCTTGAAGTTGT
AGGTTCATTCCTTCACTCCATCGAACAATCCAAATTTAAACTTATATTGGATGAATATGAAAACCAATAC
CTTGACAAGGGCATACAAGATCTTCTTCGAATAAGTTACGATGGACTTGAAGATGAAGTAAAAGAAATTT
TTCTTTATATATCTTGTTGCTTTGTAGGAGAAGATATCAACGAAGTTAAAACGAAGTTANGATATTGAAT
TTACTTTTATTCTTTTCTTATTTAAACATAGAAAGAAAGAAAAAAATGTATAAGGAAAATTTCATTTTTT
TTTTTTGCTACCTTTTTATTGTTATTCTTTTTAAAAAAAAAAAAAAAGCATTTTAACATTTTTCTTCTCTC
TGTTAAAAAGAGAGAAAAAGAAAAAAGTTATAAGCAAAAAGTCTTATATTATTATTATTTTTATATTTAT
TATATATATAGTTTTTTCTTTTTCTTTGCCGTCATCCCTCTGTTTTCTTTTTTTTTTTTAATTTATTTATT
CACTTATTTAAGTTTAATTTTGTTTATAATTTATTTATTTATTTATTTACTATTATTATTATTATTGTTT
TTTTTTATTTTATATATATAACTTTTATTTATTTAGTTTTTATATTTATGATTTTTTTAACTTATTATTA
TTATTATTATTATTATTATTATTATTATTTCATTTCATAATTTTTTTTAACTTACTATCATCCCTATGTT
CTCTTTTCTTCACTTATTTATTTAATTTTTTTAACTTATTTATTTGTTTATTATTTCATTTAGTTTTTTT
TCTCTTATTTTATTTACCGTTATTATATATCTTTTCTTTTAACTTATTTTTCATTACTATCGTTATATTT
AGTATATGCATGCATTAATATAATAAATTATTTGCCTAATTTATTGTGTGGTATATTTGATTTGGTTTAT
GATGTGTTATTTCTATTTGACCTTTATTAAAAATTTCTTGAAAACTTTAAAATACTTTTAACCATAATTG
CACGTAATTTTGAAATCTTTTTTTTTTTTTTTAATTTTTTCTCTTTAAACCATTTAGAAATTTTTTTGTGC
TTTAAAATTATTTCTTTTAAACTCACAATTAAATATATATTTCATCAGGTTTCCTATTCTACTTTTTTTT
TTATAATGACCCATGCCGATTTTGCTAAATAAAATCTTACGATAGAATCTAGAATTTTCTTGTTATTTTT
AGATTTTTGAATTGAGGGATCAATATCTTTGGGAGTTCGAGGTTTGATCCCAAGAAAAAATAAATTTAAT
CAATGTTTTAAAAAAAAAACTTTATTCGAAGACTAGCCTATGATAGAAATTTGAGAGATTATTAGTTCTC
TTAAGGTGAGGAATTTTTCTCAATATAGTCTAATTAATGAGTGTGTTCCACTTATTTTATGGGATCATTT
CTTCAAATATTGAAGTGGTGAGCAATGAAATAAGATAGTTTTTTTAAAAAACGAATTTTACTCGAGACAT
AAAATTTGACCACTGTTTTCTAAACGGGTGTTACGGGGTGCTAACACCTTCCCCGTACACAAATGACTCC
CGAACTCAATTCTCATTTTTTCATAAACCATTTTCTTTATTTTATTTAAAATGATTTACTTCATTTTGGT
GTACAATCACACCGTACAAAATATTGGTGACGACTCCCATTTTTTTTTAAAAAAATTAAACTCAAAATCT
TTGGCGTCTCTGGAGCTCAAAGACGAGAATCCCAATGATCAGCACAAACACAAAAGATCTTTGCCTCAAG
CCTATGCAAAATTAAAACAAGAAATGTAAGAAATATCATTAGCTTCAACGCTGCCGTAGCTACTCTCTGG
ATAATTTGGTTGGGAATAAACAAACTAATTTTCTCCGAAAAAGAAACAAATATTCGAAACTCTTAGGAAG
ACATCTGCACCATTACCGGTTTATGGTCCACAAAAAGCAAATTGTTCAAACTCTACAGCCAGACCACAAT
CTCCCTTAACATCAAAGCGTTTTGTTGCCCTTCTTTGTAAACAGCTTTAATTGGGATATCATTTTGGAAT
AAAATTAGTGATAGGATGGCTATTCTCTAGCTCCCTTCACTCGTATCCTTTAAAAAAAAAAAAAAGTTTAA
CTTAAAACAAAATATTGGGATATAGACATTTGATTATCTTTTAATTGTCATCTGATTATCTTATATCTAT
GATAAGATATATATTTATCATTTTAATATTATGATTGTCACCTTCTACTTTTAAAAATTTACAATTTTAG
TTAGCCTTTTAAGATAATCATTTAATATGATTACCTTCGGATTGTTATGATTATATTTTACCTTTTTTTT
TTGTTTTTTCAATTTAGTTAGGCATGTAAGATAGTCATTTGATCATCTTCCGATTGTCATCTAATTAACT
TATACTATTTTTTTTAATTATCTTCCAATTACTTTTTGATTATCATATGATTACCTTCTATTTTTTTCAA
TTTTAATTACATAATCATATTTACCTTCTTTTTACTTTTTTTTCATAGTAATTTTTTTTTTTAAATTGTTT
GATTGTTTTCTGATTGTCATCTAATTAGCTTTCGTTCTTTGACACGTCTAATTGCTATTTGATATTGATT
GTGTTATCTACTTATCGCATATATTTATTTGTGAAGAATTCCACATCTCAAACCATATATATATCTTGTA
TGTAACTGATCAATAAATAAGGTCATCAATGAACTCCATCAAAATTTGATCAATCTCAATTTGATTGACA
TCTTATTATCTTAAGGCTCCCATGTTGTATAAAGCTTTTGCTAAAGTTGTCTTACCTTTGCCTCCAATTC
CATATAATCCAACCATGGTAGTAATTTCATCACACAATCTAACATGTGAAGGTATTTTTCTAAATGGCTT
ATCAATTCCAATTACTGAGTATTTAGCTACATGTAACAGCATCATTGCACCATTTATTTTCTACCAAACT
TAGCCTCATCACTGCCATTTAAGAAATAAGAAACAGCTTCAAACTTTGTGTGAAACACACATTTTCTTTC
CAACAACCCTTCCTTCTTCCTTCTTTTTAATTCTTCCTTTTTCTCTCATATTTACTAAAGTGGCTTCTTC
TTTCCAAGAACCTTCCCCTGTCTTTTTCTTATTGACTTTGAAATAACGTTTTCCCCCAAGTCTTCTTCCT
TTTTTAAAAAATTATCCTTTGGTTAAAATATTGTTTTATCAGTGGGTCTTTATAGAAAGAACAAGGTGGG
TGGTTATCATGTATTATTGAACTCATGACTCCTTATTAGTTCTTTGGGGTATTCTTTCTTCTTTTATGTT
CTCAACCATTAGTAGGCCAACTTATGATGGTTATCAAATTTCGTTCTTACACCTAGTTTATTGCTGATTC
TTTTTTTTGTATAAAAAAACATTATTATCTATTAACATTTTAGATTTTACTATAAATTTTGGGCTCCTAT
ATTATATTTTCTTGCATTAAAATTATTAATATTAGGAACATTTTTAAAAATAGCAAAATAAACTAAAACA
TCTGCAACATATATGAAAATTTTGGATTCTATCAAATCTAGACATCGATAGACTTTTACCACTGTCTATC
AATATGGCCGATAGAATCATATTGATAGAATATGAAATTTTGCTATATTTTGTAGATATTTTGTCAAATT
TTTCATTTTTTATAATTCCACTTTATATTATTTAATCAATTCCTATATTAAAATAATTAACTTCTTGGGA
TTAAATTTAATATCATTGATATATCTAATTACTTGTCATATTTGCAATATATAAAAAAGAAGTGCTATGA
CCTGCTTTTATCTAAATATTTTTGTCCTCCGATGTAATTTCTAGTTTAAGCATTTTAAATTACATATATT
AAAATTCAACAAAAAGCAATATTTTAATTGAAAAGATATTTTTTTTTTTTCGCAAAATAAGAATAAGAAG
ATATCACGGAAACTGCAAGGACGACTTGGAAACGACGATTTTTAAAGTTGAAAAAGGAAAAAGGAGTATG

GTTCTTCGAGAGAAGCCAATGGACGCGATTCATTAAATAATTGTTATTTAACTATATATTCCTCAACTAT
ATATTAAATATATAGTTCTCTATCAACCATAGAAGGTACAAAGTGTTATGAAACACACATTTTTAACTCC
TAAAATTATATATGAGCTAGCAAGAAAAGAAACTTAAACTAGATCCAAAACCAAAAGCAAGCAATATTAT
CTATCAGAAGGAATCAGTGAAGACATGATAACAAATGGCTGTAATTAAACATACAAAAATTAAAAATCTA
GATGATGGTTTGAAAATTGAACTTAGAAGAGTAGTTTTCAAAATCCAAATGGTTTTATAAAAGGGAGACA
AATTAGATATGTCATCCTTCTTACCAACGAAGCTTTTGACTTCTGGGGTGCAATGAAGGTCAAAGTCTTT
GTCATCAAGTTTGACGTTGAAAAATCTTTTGACAAGATAAACTGAAATTTCATTGATTACATGCTTCTAA
AGAAAAATTTGGCCAAACAAAGGTATTCGCCAGCGTGACCCCATCTCAACTTTCATTTTTGTCCTTGCTA
TATGAACTATCTCAGACGGATCCTCAGTACTCACCTTGAACAGAATAATCAAGTCAGAGGTATTGCTATA
AACAATGTCAATCTTACACACCTCCATCTTTGCGGATGATTTCTTAGTTTTTGTAGAGGACAATGATGAT
TACCTCAGAAATTTTGCAATATGCGATCCATTAATTATTTTGAAGAGGCGGCTTAGCTAAACATAAATTA
AACCTCTCTAAATTAAATCTGCCCTTCTGTCCTATTAATGTCACCAAGGGCAGAACTGATTATTTTGCGA
ACAATTGGGGTATATCCACACACTTCCTCCCTTCCAGCTACCTTGGGAAAACCTCGATCCCTTACCTTTT
GGGAAAATATATTTGATAAAGTCCACAAAATGTTCCAATAAAAGGGTGGGAGACACACTCATCAACTCTT
CTCTTCTTAGTCTTTCGTTTATTATTTATTTATTTATTTATTTTTGAAAAGATACAAGTGAAGTGAAGGG
AGCTAGAGAGTAGCCACCTTGTTACAAAGATGTTACAAAGTCTTGTTTCATTTACATAGATGAAAAGCAA
AAAGCATTGATATTTAATGAAACTGTGGCCTAGCTGTAGTGCTTGAACAATTTGCTTTTTGAGGATCAAC
TACCGATTAGAGAGCAAATATCTTCCTAAGAGTTTCGATGATTGGTTTTTTTCTCTGTGAAGATTTGTTT
GTTTCTTCCTAACCAAATTGTCCATAAGATAACAACGACGGTGTTGAAGTTGATGACATTTTTTGCAATT
ACTCCCTTTAATTTTGCACAAACTCAAACATATTTCTATAGAGATACCAGTATTTGTACTCCAAAGTTGC
CATAGCTTTTGTGCTTTAGGATAGTGGATGAAGAGATGATCGAGGTCTTCATTTGAAGACTTGCAAGAGA
TACACCAGTTTGGATCAAGGCACATATTCAAATTCTTTTTTTGGATGAGGTCCATTGTGTATCCGTAAGA
AAATTGAAAAACACTTATCAACTCTCTATCTTAAAAGCCCCATGTCTATCTGTAAGAAAATTGAAAAACA
CACTAGAGGGAGTTCTCTAGAAAGGAAATCATGAAGACCACAACTTCAACATTGATTAAAAAAAATAAAT
TGCTATATGGAGAACTTAATGCTTTTTGGGATATTCAATATTACATACTAATGAATTAGAGCAATTAGAA
CTCAAGCTAGATTCTTTTTTCGTTGAATTTTCTCAAGTTTTACTTTCTTTTTATGCAATTTACTGTCTTG
CATCAATTTCAACTCAAACCCCTTGGTTACATGGTTGGAAATAATTTATTCGTTTGAGGAATCAATGTGC
TCCTTGAAATCGACCCTAACTTACCAAACTAAAACTAGTTTTTGGTGTGTGATTTGGTTAATTATTATAT
TTGTCATGGGTTTGGTCGACGAAACTCGTTAGACCACACATCCAAATCGTCTTTAAACAAAATATGTAAT
CTGATTTACAAGCGGAGGGTGATAAATCTAATTGCGCCTGAAAACTACGTCTTACCGTTACTATTTAGGT
TTGGGTTATGGTTACCATTATTGTTACAGATAGACTAATATGATGTATATTTGCTATGTTTGTAAAAAAA
TTTAGAAGTTTTGTCATTTAACCAATGTTTATTAATTTGTAATAAAAAAATTTGTGTAATTGTAATTAAA
TATCATTGATAGAACAATCGTAATTGATTGCAATCTCAAATGTAATTGAATTGACAATTTGATTGCATTA
CCTAAAATACAATAATGGTAATATTTAAGTCTATCACGATATAGATAGTAGTCTTATTGCAATCTATTGC
TGATACACGTACAATATAGGATGCAAATTATATAAGATTATAGCCAAAGCACAAGCTCATTGAATCTTAA
TGCTTTTTGCAATCGACTATGATTCCTCTTCTTATAGGCTTACCTCCAGCCTCGCTATGCAACTTCTTTG
CTCTATGTTTGTAATAAAATTCATGTTTTATCCAATAACTTAAGATATATATATAAAAAAAATTATCAAT
AAACCAAATGAATTTGTTTGGTTGGTTCCTTTTAAATAATAAGATTCAAATAATTACTTGTTTGGTTTCA
TTGAGATTCAAATTAAAAAGATTTTAAATTTTTTGAAAACTTTTGGGGATTGTTTATTAGTTGTGAGCTA
GTAGAGAAACCTAATAAGAATCCTAACAAGAGTCCAGTACAAATGGTAAGCTCCACTATTCCAATCTTAT
TTATATATACTATAGTCATTAAGTTCCTACTTGAACATGATCTATTTGTATGTCAACTACATACTGTTTA
TAAGTCTTGTAAATTAAAATAACCTTAAAAGAATGTTGCACGTAGTATTCCTATTAGGAATTTTAGGAAG
AGATCTCAATATTTGGGAGTAGAGTAATTGATGGTAAAAAAAAGAACCAACGTTGATTTTTTTTTTTTTT
TTTACATGAAATTGATGAGGATATTAGATGAATCATTTGAAACTATACTCTCAATTTTGATTACAAGTAA
TAGTACAACACCTATAGATTTCTTGATATTGAGCTAGCGAATATAATGTGATTTTTTAAATACATCACAT
ACCATTCTCTCAATTTACACTAACAAAAATTGAAATACATCTTCGTCCTTGGGCCCAACTCAAAGGTAAT
TATTTCACTATCAACCTTAAATATTAGGGGTGGTTTGGCCCCATTAGTTGACTTGTCATTTGTCCTACTT
GTGATTAATATCTGTGGGTCTCTGAATTGATCACCAATTTCTCATTCTCTGTCTCTTTGTGTTCCTGTAG
TTCATACCTACATGTGCTCCTTCCATTCCATGTTCTTATCCTTATCGTTATATCATTCTTGATATCACTG
TGACCAATATTACTGAAAACGGTTGAAACTTGTTGTATCAAACAAAAATCGGTCTCTCGGTTTGAAAGCC
AATAACGCTGCTTAATTAATCATTACCTTCTACTTAATTAATTCATGACATCTTTTAAATTGGGACATTT
TCCAATTAAAAGAAATTAAAATGTTCAAAAAAAAAAAAAAAAAAAAAAAAAAAAAAAGAAGAAGAAAAAGGA
TATCAACGAATAAAAAAAATGCAAAAATATGCTACTAATATCGTGTAAAAACTCCATAATATATCAAAAG
TAACGTCGTAGGATTACACATGAATACTTGGATTCCAGAATGGTTCAACTATCCGTCAATATCAAATTCA
ATAAGGGTTAGCTTTCGACACGATCTCAATATGGAACGAACTTTGGCTACATATGCTACTTTCCAAGTGG
TTGGAGATTCATATCGAGGAATGGCCTTAGTTTCATGTAAAATATTTCATTGGCTACAGACTCCAAAGTT
GTTTTATGAGAAAATTTCCATCATCAACATCAGAATATACATGGTTAGTAACAACTTCTTCTCCAACATT
TAGCACTTCCTTGGAGATGAATGAGTGGAATCATGTCGCAGTCTGGTTTAAGGTTGTGAAATGTTCTGAG
GTCACCGTCACTATGAAATGCTGTGGTGTCCATCTCACTGAAGGGGTCCATGGAATACAAAACGATGACA
AGGGGCAAGGGGTAATTTATACAGGTTTTGATCAACCAGACAAAATGCCGAGCGGGTGACTGAGATTAAT

TGATTGTTTTAAGAAGGTTTGTGTTAGTTTGTATAGAATATTGGTATTTTCTTGAATATTGGTATTTCAT
CGATAATAATCAAAGTTATTTTAAAATACTCTAATTATGTCAATAGGTTGATTAGTTGTCCTGTTTTAGA
AAATTTTGGCAACATTATTATTTTGTCTATTTGTTATCTATCAGATGGGAGCGAGACAGGACCTAGCGAA
TAGCGGGAATAATTCCATATTCAAACAAGAAATCTAAGGCTCACTTAAAATCGACATTTGTTTATTAGTT
TTTGAAAATTATTTCTACAATTTGTTATCTACTTTTATTTCAATTATTTTAAAAAACTAGGTGATGGTTT
GATAAAATTGAACTTAGAAAAGTAGTTTTCAAAATCCTTTTCTGTTTTTGCCTTCGAGTGCTTCTATATC
AAACTTCAAAACCATAATTGAAAAAAATAACAGGAGAAAACAAATATAAATTAAAAAAAATAAACAAATT
ATATAAACTCAAAAATCAATTCCTGTTTTCACTTCATTAACAATGCTTTCTTCAGGTAACATATATTTAT
AAACATAAAAGTTTATAATTAGAATTGCAAAAGAGTAATGAGAGTCTCTCTCCAGGTTTGCATATTCTAA
CTTCAACTTAATTGGCAAATTCTTCTCCAAATTATTCCTCCACGATGACATGAATACCACATATTTTTAT
ACTTACTGCGTCTTTCGAGTTTGGAGTAAACTCAAATAACATTGTAATTTTATCCAAAGTACCTGTTGAG
GAATTAATCCTAAAACATGGTTGAAGATTAATCGGAGAGCTGTCCATATCTTCAACATATGGGTCGAGAA
GTATACAATGATCAAGCACTGCCATCCATGGATACTCACCTCGTGATGTTTCAGATTTAGTCCCATGGAT
CCAAAAGCGTTTCCCGGTTACTTCAAAATCGTTGATAAACAGTCTACATAAGAGCTCAAAGAACTCGTCA
ATATCAAAGGTACCTTCAAATTATTTGACACAAGGAGCAATAAAAGCATTTCTTTCCCAACTTAGATAAT
CAGTTGGAATAAGAAACGTTATTGAATTGTTCATACTCTTGTACTTGCACCAATCTGGAATATTACAATT
CATTAATACGAGTTCTTTCAATATTCCGCCTTATACATAGCAACTGGTGCAATGAGCCCCCTCTATCACA
CCAAGCCCCTAGACTATACACTCTCTTGAACATACAAAGTGTAACAGTTAAGGAAGCTTGAGACCAAACT
ATTGGAGAATGGAATATCTATCCTAGAAGACCATTAAATGAAAGAGAGGTCCAACTTTGGCGGACTTTGA
AAAACAACATTGCATCAAAACTTATAATTTAATTTCAAATATAACTGTATGTGTCAAAAATAGATAAAGT
CACTGTTAAAGAAGAAGAGAGTGCACGTTGAGATAAGCTCACTGTAATAATGAGTTAGATTTATGAAGAA
TTTTCATATTATTTCCCATTCATACTGTTAAAAACGTTGTCTTTTTTAACATGTCTTTACACCTTAACTT
ATCAAAGGATGTATATATAAAATCCTCATTTGAAGAACAAATAGCCGATTGGTAGACCAATACAGCTCCT
AAGAAGAAAAAAAGAGAGACTTGCCTTAAAATAACGGAAAATTATAGTAAATATCACCGTTTAAAAATTA
AATTAAAACTACAGCATACAGATAAAATATTTACAAAATTAGCAAGTGTTCAGACCAGTCAACTAGGAAT
TTTTTTAAAAAATGTTAAACTTACCCTTCACAACTCGTTAAAATTGTGCTATCAATGATAACAATGGGTA
GCACATTTTTTCAAATTGAAATTAAAGTTATAATTAATTATTGACAACTTTTAGTGACGTTAGCTATCAG
TGATGACAACTATCAGTGATGATAGCCATCAATGATGATGGGTATCACTAATAGGTTCCAATATTGAAAA
ATTAAGAAGAAACTTTTTAAATGGTAGCGTGAGATTTTTAGATTTTACCCTGAATTTTGTGTTATATTTG
CAATTATCTTAACCTTTTGCAATGTATTTTATCATTTAGTTTAAATGTCATCTAAGGAACTTGTCCCAAA
ATAAATGAAAAATGAAAAAAGAAAAGGAAAAAGGAAAAAGGAAAAACCAAAATAAAACTCAACAAACAAT
TCATATGTCAAAATGGTTAAAGCATAGTTCTGTTCGATGATTTTCAAATAAATTTAATTCGTGGAACTTG
AGCTTTATATTTGTATTAATTTTAATATAGTATACGGTCTTTAGTACATAATAATTTGATACTCTAAAAA
AATAAACTTAAAAACGTTTAAGCTTTTTATTCTTTCCAAATTATTGACGTTTGGGTTTGATGATCTTCCT
GGATATGATTAGCCTTTAAAATTGTTGATCTTCATTAAATGATCGACCCTTGAATTTATTGATCTTGCCT
AGAGGGTCAACCTTTAGACCCTTGGATTTAAGGATCTTCCTAGAGGACCAACCCTTTGATTTATTGATCT
TGTTGATCTTCTTGGATCGACTTTAAAACTTTAGAAGAGTTGTGTTCTTCATATTTTCAAAGCTTAGCTT
TAAAGCTTCGATCTTCAATATTATTTCAGGGCTTTAGAGTTTGAGGGGAGATATATTCTTGAGATTTTTA
GAGCTTCTATCTCTAGAACCTTAGATCTCTAGAGAGTTTTAGCTCGTAGAAGTTTTTCCATGAGTGGGTT
TTATATCGATTAGGGTCCAGTTGTTTGTTGGGTTGGACTTGGCCCAAATTTTCATCATAGATTTGAATTG
GCTTGGACTTGAACAATTTTAGTTGTTCAACACCTAACCATTTATATAATTTTCATAGTGACCTTTTTAA
TTTTTATTATTATGACATGGTGAAATTTAATTAAATTAGCCAAAAATTTATTATTTAACATATCCAAATG
ATATAAAATTTGTATATATAGTCAACTTTAAGGAGGAGGGTTGGTCAACAAATACTCAAGGTTGTGAGAT
GATNNNNNNNNNNNNNNNNNNNNNNNNNNNNNNNNNNNNNNNNNNNNNNNNNNNNNNNNNNNNNNNNNNN
NNNNNNNNNNNNNNNNNNNNNNNNNNNNNNNNNNNNNNNNNNNNNNNNNNNNNNNNNNNNNNNNNNNNNN
NNNNNNNNNNNNNNNNNNNNNNNNNNNNNNNNNNNNNNNNNNNNNNNNNNNNNNNNNNNNNNNNNNNNNN
NNNNNNNNNNNNNNNNNNNNNNNNNNNNNNNNNNNNNNNNNNNNNNNNNNNNNNNNNNNNNNNNNNNNNN
NNNNNNNNNNNNNNNNNNNNNNNNNNNNNNNNNNNNNNNNNNNNNNNNNNNNNNNNNNNNNNNNNNNNNN
NNNNNNNNNNNNNNNNNNNNNNNNNNNNNNNNNNNNNNNNNNNNNNNNNNNNNNNNNNNNNNNNNNNNNN
NNNNNNNNNNNNNNNNNNNNNNNNNNNNNNNNNNNNNNNNNNNNNNNNNNNNNNNNNNNNNNNNNNNNNN
NNNNNNNNNNNNNNNNNNNNNNNNNNNNNNNNNNNNNNNNNNNNNNNNNNNNNNNNNNNNNNNNNNNNNN
NNNNNNNNNNNNNNNNNNNNNNNNNNNNNNNNNNNNNNNNNNNNNNNNNNNNNNNNNNNNNNNNNNNNNN
NNNNNNNNNNNNNNNNNNNNNNNNNNNNNNNNNNNNNNNNNNNNNNNNNNNNNNNNNNNNNNNNNNNNNN
NNNNNNNNNNNNNNNNNNNNNNNNNNNNNNNNNNNNNNNNNNNNNNNNNNNNNNNNNNNNNNNNNNNNNN
NNNNNNNNNNNNNNNNNNNNNNNNNNNNNNNNNNNNNNNNNNNNNNNNNNNNNNNNNNNNNNNNNNNNNN
NNNNNNNNNNNNNNNNNNNNNNNNNNNNNNNNNNNNNNNNNNNNNNNNNNNNNNNNNNNNNNNNNNNNNN
NNNNNNNNNNNNNNNNNNNNNNNNNNNNNNNNNNNNNNNNNNNNNNNNNNNNNNNNNNNNNNNNNNNNNN
NNNNNNNNNNNNNNNNNNNNNNNNNNNNNNNNNNNNNNNNNNNNNNNNNNNNNNNNNNNNNNNNNNNNNN
NNNNNNNNNNNNNNNNNNNNNNNNNNNNNNNNNNNNNNNNNNNNNNNNNNNNNNNNNNNNNNNNNNNNNN
NNNNNNNNNNNNNNNNNNNNNNNNNNNNNNNNNNNNNNNNNNNNNNNNNNNNNNNNNNNNNNNNNNNNNN

NNNNNNNNNNNNNNNNNNNNNNNNNNNNNNNNNNNNNNNNNNNNNNNNNNNNNNNNNNNNNNNNNNNNNNNNNNNNNNNN
NNNNNNNNNNNNNNNNNNNNNNNNNNNNNNNNNNNNNNNNNNNNNNNNNNNNNNNNNNNNNNNNNNNNNNNNNNNNNNNN
NNNNNNNNNNNNNNNNNNNNNNNNNNNNNNNNNNNNNNNNNNNNNNNNNNNNNNNNNNNNNNNNNNNNNNNNNNNNNNNN
NNNNNNNNNNNNNNNNNNNNNNNNNNNNNNNNNNNNNNNNNNNNNNNNNNNNNNNNNNNNNNNNNNNNNNNNNNNNNNNN
NNNNNNNNNNNNNNNNNNNNNNNNNNNNNNNNNNNNNNNNNNNNNNNNNNNNNNNNNNNNNNNNNNNNNNNNNNNNNNNN
NNNNNNNNNNNNNNNNNNNNNNNNNNNNNNNNNNNNNNNNNNNNNNNNNNNNNNNNNNNNNNNNNNNNNNNNNNNNNNNN
NNNNNNNNNNNNNNNNNNNNNNNNNNNNNNNNNNNNNNNNNNNNNNNNNNNNNNNNNNNNNNNNNNNNNNNNNNNNNNNN
NNNNNNNNNNNNNNNNNNNNNNNNNNNNNNNNNNNNNNNNNNNNNNNNNNNNNNNNNNNNNNNNNNNNNNNNNNNNNNNN
NNNNNNNNNNNNNNNNNNNNNNNNNNNNNNNNNNNNNNNNNNNNNNNNNNNNNNNNNNNNNNNNNNNNNNNNNNNNNNNN
NNNNNNNNNNNNNNNNNNNNNNNNNNNNNNNNNNNNNNNNNNNNNNNNNNNNNNNNNNNNNNNNNNNNNNNNNNNNNNNN
NNNNNNNNNNNNNNNNNNNNNNNNNNNNNNNNNNNNNNNNNNNNNNNNNNNNNNNNNNNNNNNNNNNNNNNNNNNNNNNN
NNNNNNNNNNNNNNNNNNNNNNNNNNNNNNNNNNNNNNNNNNNNNNNNNNNNNNNNNNNNNNNNNNNNNNNNNNNNNNNN
NNNNNNNNNNNNNNNNNNNNNNNNNNNNNNNNNNNNNNNNNNNNNNNNNNNNNNNNNNNNNNNNNNNNNNNNNNNNNNNN
NNNNNNNNNNNNNNNNNNNNNNNNNNNNNNNNNNNNNNNNNNNNNNNNNNNNNNNNNNNNNNNNNNNNNNNNNNNNNNNN
NNNNNNNNNNNNNNNNNNNNNNNNNNNNNNNNNNNNNNNNNNNNNNNNNNNNNNNNNNNNNNNNNNNNNNNNNNNNNNNN
NNNNNNNNNNNNNNNNNNNNNNNNNNNNNNNNNNNNNNNNNNNNNNNNNNNNNNNNNNNNNNNNNNNNNNNNNNNNNNNN
NNNNNNNNNNNNNNNNNNNNNNNNNNNNNNNNNNNNNNNNNNNNNNNNNNNNNNNNNNNNNNNNNNNNNNNNNNNNNNNN
NNNNNNNNNNNNNNNNNNNNNNNNNNNNNNNNNNNNNNNNNNNNNNNNNNNNNNNNNNNNNNNNNNNNNNNNNNNNNNNN
NNNNNNNNNNNNNNNNNNNNNNNNNNNNNNNNNNNNNNNNNNNNNNNNNNNNNNNNNNNNNNNNNNNNNNNNNNNNNNNN
NNNNNNNNNNNNNNNNNNNNNNNNNNNNNNNNNNNNNNNNNNNNNNNNNNNNNNNNNNNNNNNNNNNNNNNNNNNNNNNN
NNNNNNNNNNNNNNNNNNNNNNNNNNNNNNNNNNNNNNNNNNNNNNNNNNNNNNNNNNNNNNNNNNNNNNNNNNNNNNNN
NNNNNNNNNNNNNNNNNNNNNNNNNNNNNNNNNNNNNNNNNNNNNNNNNNNNNNNNNNNNNNNNNNNNNNNNNNNNNNNN
NNNNNNNNNNNNNNNNNNNNNNNNNNNNNNNNNNNNNNNNNNNNNNNNNNNNNNNNNNNNNNNNNNNNNNNNNNNNNNNN
NNNNNNNNNNNNNNNNNNNNNNNNNNNNNNNNNNNNNNNNNNNNNNNNNNNNNNNNNNNNNNNNNNNNNNNNNNNNNNNN
NNNNNNNNNNNNNNNNNNNNNNNNNNNNNNNNNNNNNNNNNNNNNNNNNNNNNNNNNNNNNNNNNNNNNNNNNNNNNNNN
NNNNNNNNNNNNNNNNNNNNNNNNNNNNNNNNNNNNNNNNNNNNNNNNNNNNNNNNNNNNNNNNNNNNNNNNNNNNNNNN
NNNNNNNNNNNNNNNNNNNNNNNNNNNNNNNNNNNNNNNNNNNNNNNNNNNNNNNNNNNNNNNNNNNNNNNNNNNNNNNN
NNNNNNNNNNNNNNNNNNNNNNNNNNNNNNNNNNNNNNNNNNNNNNNNNNNNNNNNNNNNNNNNNNNNNNNNNNNNNNNN
NNNNNNNNNNNNNNNNNNNNNNNNNNNNNNNNNNNNNNNNNNNNNNNNNNNNNNNNNNNNNNNNNNNNNNNNNNNNNNNN
NNNNNNNNNNNNNNNNNNNNNNNNNNNNNNNNNNNNNNNNNNNNNNNNNNNNNNNNNNNNNNNNNNNNNNNNNNNNNNNN
NNNNNNNNNNNNNNNNNNNNNNNNNNNNNNNNNNNNNNNNNNNNNNNNNNNNNNNNNNNNNNNNNNNNNNNNNNNNNNNN
NNNNNNNNNNNNNNNNNNNNNNNNNNNNNNNNNNNNNNNNNNNNNNNNNNNNNNNNNNNNNNNNNNNNNNNNNNNNNNNN
NNNNNNNNNNNNNNNNNNNNNNNNNNNNNNNNNNNNNNNNNNNNNNNNNNNNNNNNNNNNNNNNNNNNNNNNNNNNNNNN
NNNNNNNNNNNNNNNNNNNNNNNNNNNNNNNNNNNNNNNNNNNNNNNNNNNNNNNNNNNNNNNNNNNNNNNNNNNNNNNN
NNNNNNNNNNNNNNNNNNNNNNNNNNNNNNNNNNNNNNNNNNNNNNNNNNNNNNNNNNNNNNNNNNNNNNNNNNNNNNNN
NNNNNNNNNNNNNNNNNNNNNNNNNNNNNNNNNNNNNNNNNNNNNNNNNNNNNNNNNNNNNNNNNNNNNNNNNNNNNNNN
NNNNNNNNNNNNNNNNNNNNNNNNNNNNNNNNNNNNNNNNNNNNNNNNNNNNNNNNNNNNNNNNNNNNNNNNNNNNNNNN
NNNNNNNNNNNNNNNNNNNNNNNNNNNNNNNNNNNNNNNNNNNNNNNNNNNNNNNNNNNNNNNNNNNNNNNNNNNNNNNN
NNNNNNNNNNNNNNNNNNNNNNNNNNNNNNNNNNNNNNNNNNNNNNNNNNNNNNNNNNNNNNNNNNNNNNNNNNNNNNNN
NNNNNNNNNNNNNNNNNNNNNNNNNNNNNNNNNNNNNNNNNNNNNNNNNNNNNNNNNNNNNNNNNNNNNNNNNNNNNNNN
NNNNNNNNNNNNNNNNNNNNNNNNNNNNNNNNNNNNNNNNNNNNNNNNNNNNNNNNNNNNNNNNNNNNNNNNNNNNNNNN
NNNNNNNNNNNNNNNNNNNNNNNNNNNNNNNNNNNNNNNNNNNNNNNNNNNNNNNNNNNNNNNNNNNNNNNNNNNNNNNN
NNNNNNNNNNNNNNNNNNNNNNNNNNNNNNNNNNNNNNNNNNNNNNNNNNNNNNNNNNNNNNNNNNNNNNNNNNNNNNNN
NNNNNNNNNNNNNNNNNNNNNNNNNNNNNNNNNNNNNNNNNNNNNNNNNNNNNNNNNNNNNNNNNNNNNNNNNNNNNNNN
NNNNNNNNNNNNNNNNNNNNNNNNNNNNNNNNNNNNNNNNNNNNNNNNNNNNNNNNNNNNNNNNNNNNNNNNNNNNNNNN
NNNNNNNNNNNNNNNNNNNNNNNNNNNNNNNNNNNNNNNNNNNNNNNNNNNNNNNNNNNNNNNNNNNNNNNNNNNNNNNN
NNNNNNNNNNNNNNNNNNNNNNNNNNNNNNNNNNNNNNNNNNNNNNNNNNNNNNNNNNNNNNNNNNNNNNNNNNNNNNNN
NNNNNNNNNNNNNNNNNNNNNNNNNNNNNNNNNNNNNNNNNNNNNNNNNNNNNNNNNNNNNNNNNNNNNNNNNNNNNNNN
NNNNNNNNNNNNNNNNNNNNNNNNNNNNNNNNNNNNNNNNNNNNNNNNNNNNNNNNNNNNNNNNNNNNNNNNNNNNNNNN
NNNNNNNNNNNNNNNNNNNNNNNNNNNNNNNNNNNNNNNNNNNNNNNNNNNNNNNNNNNNNNNNNNNNNNNNNNNNNNNN
NNNNNNNNNNNNNNNNNNNNNNNNNNNNNNNNNNNNNNNNNNNNNNNNNNNNNNNNNNNNNNNNNNNNNNNNNNNNNNNN
NNNNNNNNNNNNNNNNNNNNNNNNNNNNNNNNNNNNNNNNNNNNNNNNNNNNNNNNNNNNNNNNNNNNNNNNNNNNNNNN
NNNNNNNNNNNNNNNNNNNNNNNNNNNNNNNNNNNNNNNNNNNNNNNNNNNNNNNNNNNNNNNNNNNNNNNNNNNNNNNN
NNNNNNNNNNNNNNNNNNNNNNNNNNNNNNNNNNNNNNNNNNNNNNNNNNNNNNNNNNNNNNNNNNNNNNNNNNNNNNNN
NNNNNNNNNNNNNNNNNNNNNNNNNNNNNNNNNNNNNNNNNNNNNNNNNNNNNNNNNNNNNNNNNNNNNNNNNNNNNNNN
NNNNNNNNNNNNNNNNNNNNNNNNNNNNNNNNNNNNNNNNNNNNNNNNNNNNNNNNNNNNNNNNNNNNNNNNNNNNNNNN
NNNNNNNNNNNNNNNNNNNNNNNNNNNNNNNNNNNNNNNNNNNNNNNNNNNNNNNNNNNNNNNNNNNNNNNNNNNNNNNN
NNNNNNNNNNNNNNNNNNNNNNNNNNNNNNNNNNNNNNNNNNNNNNNNNNNNNNNNNNNNNNNNNNNNNNNNNNNNNNNN
NNNNNNNNNNNNNNNNNNNNNNNNNNNNNNNNNNNNNNNNNNNNNNNNNNNNNNNNNNNNNNNNNNNNNNNNNNNNNNNN

NNNNNNNNNNNNNNNNNNNNNNNNNNNNNNNNNNNNNNNNNNNNNNNNNNNNNNNNNNNNNNNNNNNNNNNNNNNNNN
NNNNNNNNNNNNNNNNNNNNNNNNNNNNNNNNNNNNNNNNNNNNNNNNNNNNNNNNNNNNNNNNNNNNNNNNNNNNNN
NNNNNNNNNNNNNNNNNNNNNNNNNNNNNNNNNNNNNNNNNNNNNNNNNNNNNNNNNNNNNNNNNNNNNNNNNNNNNN
NNNNNNNNNNNNNNNNNNNNNNNNNNNNNNNNNNNNNNNNNNNNNNNNNNNNNNNNNNNNNNNNNNNNNNNNNNNNNN
NNNNNNNNNNNNNNNNNNNNNNNNNNNNNNNNNNNNNNNNNNNNNNNNNNNNNNNNNNNNNNNNNNNNNNNNNNNNNN
NNNNNNNNNNNNNNNNNNNNNNNNNNNNNNNNNNNNNNNNNNNNNNNNNNNNNNNNNNNNNNNNNNNNNNNNNNNNNN
NNNNNNNNNNNNNNNNNNNNNNNNNNNNNNNNNNNNNNNNNNNNNNNNNNNNNNNNNNNNNNNNNNNNNNNNNNNNNN
NNNNNNNNNNNNNNNNNNNNNNNNNNNNNNNNNNNNNNNNNNNNNNNNNNNNNNNNNNNNNNNNNNNNNNNNNNNNNN
NNNNNNNNNNNNNNNNNNNNNNNNNNNNNNNNNNNNNNNNNNNNNNNNNNNNNNNNNNNNNNNNNNNNNNNNNNNNNN
NNNNNNNNNNNNNNNNNNNNNNNNNNNNNNNNNNNNNNNNNNNNNNNNNNNNNNNNNNNNNNNNNNNNNNNNNNNNNN
NNNNNNNNNNNNNNNNNNNNNNNNNNNNNNNNNNNNNNNNNNNNNNNNNNNNNNNNNNNNNNNNNNNNNNNNNNNNNN
NNNNNNNNNNNNNNNNNNNNNNNNNNNNNNNNNNNNNNNNNNNNNNNNNNNNNNNNNNNNNNNNNNNNNNNNNNNNNN
NNNNNNNNNNNNNNNNNNNNNNNNNNNNNNNNNNNNNNNNNNNNNNNNNNNNNNNNNNNNNNNNNNNNNNNNNNNNNN
NNNNNNNNNNNNNNNNNNNNNNNNNNNNNNNNNNNNNNNNNNNNNNNNNNNNNNNNNNNNNNNNNNNNNNNNNNNNNN
NNNNNNNNNNNNNNNNNNNNNNNNNNNNNNNNNNNNNNNNNNNNNNNNNNNNNNNNNNNNNNNNNNNNNNNNNNNNNN
NNNNNNNNNNNNNNNNNNNNNNNNNNNNNNNNNNNNNNNNNNNNNNNNNNNNNNNNNNNNNNNNNNNNNNNNNNNNNN
NNNNNNNNNNNNNNNNNNNNNNNNNNNNNNNNNNNNNNNNNNNNNNNNNNNNNNNNNNNNNNNNNNNNNNNNNNNNNN
NNNNNNNNNNNNNNNNNNNNNNNNNNNNNNNNNNNNNNNNNNNNNNNNNNNNNNNNNNNNNNNNNNNNNNNNNNNNNN
NNNNNNNNNNNNNNNNNNNNNNNNNNNNNNNNNNNNNNNNNNNNNNNNNNNNNNNNNNNNNNNNNNNNNNNNNNNNNN
NNNNNNNNNNNNNNNNNNNNNNNNNNNNNNNNNNNNNNNNNNNNNNNNNNNNNNNNNNNNNNNNNNNNNNNNNNNNNN
NNNNNNNNNNNNNNNNNNNNNNNNNNNNNNNNNNNNNNNNNNNNNNNNNNNNNNNNNNNNNNNNNNNNNNNNNNNNNN
NNNNNNNNNNNNNNNNNNNNNNNNNNNNNNNNNNNNNNNNNNNNNNNNNNNNNNNNNNNNNNNNNNNNNNNNNNNNNN
NNNNNNNNNNNNNNNNNNNNNNNNNNNNNNNNNNNNNNNNNNNNNNNNNNNNNNNNNNNNNNNNNNNNNNNNNNNNNN
NNNNNNNNNNNNNNNNNNNNNNNNNNNNNNNNNNNNNNNNNNNNNNNNNNNNNNNNNNNNNNNNNNNNNNNNNNNNNN
NNNNNNNNNNNNNNNNNNNNNNNNNNNNNNNNNNNNNNNNNNNNNNNNNNNNNNNNNNNNNNNNNNNNNNNNNNNNNN
NNNNNNNNNNNNNNNNNNNNNNNNNNNNNNNNNNNNNNNNNNNNNNNNNNNNNNNNNNNNNNNNNNNNNNNNNNNNNN
NNNNNNNNNNNNNNNNNNNNNNNNNNNNNNNNNNNNNNNNNNNNNNNNNNNNNNNNNNNNNNNNNNNNNNNNNNNNNN
NNNNNNNNNNNNNNNNNNNNNNNNNNNNNNNNNNNNNNNNNNNNNNNNNNNNNNNNNNNNNNNNNNNNNNNNNNNNNN
NNNNNNNNNNNNNNNNNNNNNNNNNNNNNNNNNNNNNNNNNNNNNNNNNNNNNNNNNNNNNNNNNNNNNNNNNNNNNN
NNNNNNNNNNNNNNNNNNNNNNNNNNNNNNNNNNNNNNNNNNNNNNNNNNNNNNNNNNNNNNNNNNNNNNNNNNNNNN
NNNNNNNNNNNNNNNNNNNNNNNNNNNNNNNNNNNNNNNNNNNNNNNNNNNNNNNNNNNNNNNNNNNNNNNNNNNNNN
NNNNNNNNNNNNNNNNNNNNNNNNNNNNNNNNNNNNNNNNNNNNNNNNNNNNNNNNNNNNNNNNNNNNNNNNNNNNNN
NNNNNNNNNNNNNNNNNNNNNNNNNNNNNNNNNNNNNNNNNNNNNNNNNNNNNNNNNNNNNNNNNNNNNNNNNNNNNN
NNNNNNNNNNNNNNNNNNNNNNNNNNNNNNNNNNNNNNNNNNNNNNNNNNNNNNNNNNNNNNNNNNNNNNNNNNNNNN
NNNNNNNNNNNNNNNNNNNNNNNNNNNNNNNNNNNNNNNNNNNNNNNNNNNNNNNNNNNNNNNNNNNNNNNNNNNNNN
NNNNNNNNNNNNNNNNNNNNNNNNNNNNNNNNNNNNNNNNNNNNNNNNNNNNNNNNNNNNNNNNNNNNNNNNNNNNNN
NNNNNNNNNNNNNNNNNNNNNNNNNNNNNNNNNNNNNNNNNNNNNNNNNNNNNNNNNNNNNNNNNNNNNNNNNNNNNN
NNNNNNNNNNNNNNNNNNNNNNNNNNNNNNNNNNNNNNNNNNNNNNNNNNNNNNNNNNNNNNNNNNNNNNNNNNNNNN
NNNNNNNNNNNNNNNNNNNNNNNNNNNNNNNNNNNNNNNNNNNNNNNNNNNNNNNNNNNNNNNNNNNNNNNNNNNNNN
NNNNNNNNNNNNNNNNNNNNNNNNNNNNNNNNNNNNNNNNNNNNNNNNNNNNNNNNNNNNNNNNNNNNNNNNNNNNNN
NNNNNNNNNNNNNNNNNNNNNNNNNNNNNNNNNNNNNNNNNNNNNNNNNNNNNNNNNNNNNNNNNNNNNNNNNNNNNN
NNNNNNNNNNNNNNNNNNNNNNNNNNNNNNNNNNNNNNNNNNNNNNNNNNNNNNNNNNNNNNNNNNNNNNNNNNNNNN
NNNNNNNNNNNNNNNNNNNNNNNNNNNNNNNNNNNNNNNNNNNNNNNNNNNNNNNNNNNNNNNNNNNNNNNNNNNNNN
NNNNNNNNNNNNNNNNNNNNNNNNNNNNNNNNNNNNNNNNNNNNNNNNNNNNNNNNNNNNNNNNNNNNNNNNNNNNNN
NNNNNNNNNNNNNNNNNNNNNNNNNNNNNNNNNNNNNNNNNNNNNNNNNNNNNNNNNNNNNNNNNNNNNNNNNNNNNN
NNNNNNNNNNNNNNNNNNNNNNNNNNNNNNNNNNNNNNNNNNNNNNNNNNNNNNNNNNNNNNNNNNNNNNNNNNNNNN
NNNNNNNNNNNNNNNNNNNNNNNNNNNNNNNNNNNNNNNNNNNNNNNNNNNNNNNNNNNNNNNNNNNNNNNNNNNNNN
NNNNNNNNNNNNNNNNNNNNNNNNNNNNNNNNNNNNNNNNNNNNNNNNNNNNNNNNNNNNNNNNNNNNNNNNNNNNNN
NNNNNNNNNNNNNNNNNNNNNNNNNNNNNNNNNNNNNNNNNNNNNNNNNNNNNNNNNNNNNNNNNNNNNNNNNNNNNN
NNNNNNNNNNNNNNNNNNNNNNNNNNNNNNNNNNNNNNNNNNNNNNNNNNNNNNNNNNNNNNNNNNNNNNNNNNNNNN
NNNNNNNNNNNNNNNNNNNNNNNNNNNNNNNNNNNNNNNNNNNNNNNNNNNNNNNNNNNNNNNNNNNNNNNNNNNNNN
NNNNNNNNNNNNNNNNNNNNNNNNNNNNNNNNNNNNNNNNNNNNNNNNNNNNNNNNNNNNNNNNNNNNNNNNNNNNNN
NNNNNNNNNNNNNNNNNNNNNNNNNNNNNNNNNNNNNNNNNNNNNNNNNNNNNNNNNNNNNNNNNNNNNNNNNNNNNN
NNNNNNNNNNNNNNNNNNNNNNNNNNNNNNNNNNNNNNNNNNNNNNNNNNNNNNNNNNNNNNNNNNNNNNNNNNNNNN
NNNNNNNNNNNNNNNNNNNNNNNNNNNNNNNNNNNNNNNNNNNNNNNNNNNNNNNNNNNNNNNNNNNNNNNNNNNNNN
NNNNNNNNNNNNNNNNNNNNNNNNNNNNNNNNNNNNNNNNNNNNNNNNNNNNNNNNNNNNNNNNNNNNNNNNNNNNNN
NNNNNNNNNNNNNNNNNNNNNNNNNNNNNNNNNNNNNNNNNNNNNNNNNNNNNNNNNNNNNNNNNNNNNNNNNNNNNN
NNNNNNNNNNNNNNNNNNNNNNNNNNNNNNNNNNNNNNNNNNNNNNNNNNNNNNNNNNNNNNNNNNNNNNNNNNNNNN
NNNNNNNNNNNNNNNNNNNNNNNNNNNNNNNNNNNNNNNNNNNNNNNNNNNNNNNNNNNNNNNNNNNNNNNNNNNNNN
NNNNNNNNNNNNNNNNNNNNNNNNNNNNNNNNNNNNNNNNNNNNNNNNNNNNNNNNNNNNNNNNNNNNNNNNNNNNNN
NNNNNNNNNNNNNNNNNNNNNNNNNNNNNNNNNNNNNNNNNNNNNNNNNNNNNNNNNNNNNNNNNNNNNNNNNNNNNN
NNNNNNNNNNNNNNNNNNNNNNNNNNNNNNNNNNNNNNNNNNNNNNNNNNNNNNNNNNNNNNNNNNNNNNNNNNNNNN

NNNNNNNNNNNNNNNNNNNNNNNNNNNNNNNNNNNNNNNNNNNNNNNNNNNNNNNNNNNNNNNNNNNNNN
NNNNNNNNNNNNNNNNNNNNNNNNNNNNNNNNNNNNNNNNNNNNNNNNNNNNNNNNNNNNNNNNNNNNNN
NNNNNNNNNNNNNNNNNNNNNNNNNNNNNNNNNNNNNNNNNNNNNNNNNNNNNNNNNNNNNNNNNNNNNN
NNNNNNNNNNNNNNNNNNNNNNNNNNNNNNNNNNNNNNNNNNNNNNNNNNNNNNNNNNNNNNNNNNNNNN
NNNNNNNNNNNNNNNNNNNNNNNNNNNNNNNNNNNNNNNNNNNNNNNNNNNNNNNNNNNNNNNNNNNNNN
NNNNNNNNNNNNNNNNNNNNNNNNNNNNNNNNNNNNNNNNNNNNNNNNNNNNNNNNNNNNNNNNNNNNNN
NNNNNNNNNNNNNNNNNNNNNNNNNNNNNNNNNNNNNNNNNNNNNNNNNNNNNNNNNNNNNNNNNNNNNN
NNNNNNNNNNNNNNNNNNNNNNNNNNNNNNNNNNNNNNNNNNNNNNNNNNNNNNNNNNNNNNNNNNNNNN
NNNNNNNNNNNNNNNNNNNNNNNNNNNNNNNNNNNNNNNNNNNNNNNNNNNNNNNNNNNNNNNNNNNNNN
NNNNNNNNNNNNNNNNNNNNNNNNNNNNNNNNNNNNNNNNNNNNNNNNNNNNNNNNNNNNNNNNNNNNNN
NNNNNNNNNNNNNNNNNNNNNNNNNNNNNNNNNNNNNNNNNNNNNNNNNNNNNNNNNNNNNNNNNNNNNN
NNNNNNNNNNNNNNNNNNNNNNNNNNNNNNNNNNNNNNNNNNNNNNNNNNNNNNNNNNNNNNNNNNNNNN
NNNNNNNNNNNNNNNNNNNNNNNNNNNNNNNNNNNNNNNNNNNNNNNNNNNNNNNNNNNNNNNNNNNNNN
NNNNNNNNNNNNNNNNNNNNNNNNNNNNNNNNNNNNNNNNNNNNNNNNNNNNNNNNNNNNNNNNNNNNNN
NNNNNNNNNNNNNNNNNNNNNNNNNNNNNNNNNNNNNNNNNNNNNNNNNNNNNNNNNNNNNNNNNNNNNN
NNNNNNNNNNNNNNNNNNNNNNNNNNNNNNNNNNNNNNNNNNNNNNNNNNNNNNNNNNNNNNNNNNNNNN
NNNNNNNNNNNNNNNNNNNNNNNNNNNNNNNNNNNNNNNNNNNNNNNNNNNNNNNNNNNNNNNNNNNNNN
NNNNNNNNNNNNNNNNNNNNNNNNNNNNNNNNNNNNNNNNNNNNNNNNNNNNNNNNNNNNNNNNNNNNNN
NNNNNNNNNNNNNNNNNNNNNNNNNNNNNNNNNNNNNNNNNNNNNNNNNNNNNNNNNNNNNNNNNNNNNN
NNNNNNNNNNNNNNNNNNNNNNNNNNNNNNNNNNNNNNNNNNNNNNNNNNNNNNNNNNNNNNNNNNNNNN
NNNNNNNNNNNNNNNNNNNNNNNNNNNNNNNNNNNNNNNNNNNNNNNNNNNNNNNNNNNNNNNNNNNNNN
NNNNNNNNNNNNNNNNNNNNNNNNNNNNNNNNNNNNNNNNNNNNNNNNNNNNNNNNNNNNNNNNNNNNNN
NNNNNNNNNNNNNNNNNNNNNNNNNNNNNNNNNNNNNNNNNNNNNNNNNNNNNNNNNNNNNNNNNNNNNN
NNNNNNNNNNNNNNNNNNNNNNNNNNNNNNNNNNNNNNNNNNNNNNNNNNNNNNNNNNNNNNNNNNNNNN
NNNNNNNNNNNNNNNNNNNNNNNNNNNNNNNNNNNNNNNNNNNNNNNNNNNNNNNNNNNNNNNNNNNNNN
NNNNNNNNNNNNNNNNNNNNNNNNNNNNNNNNNNNNNNNNNNNNNNNNNNNNNNNNNNNNNNNNNNNNNN
NNNNNNNNNNNNNNNNNNNNNNNNNNNNNNNNNNNNNNNNNNNNNNNNNNNNNNNNNNNNNNNNNNNNNN
NNNNNNNNNNNNNNNNNNNNNNNNNNNNNNNNNNNNNNNNNNNNNNNNNNNNNNNNNNNNNNNNNNNNNN
NNNNNNNNNNNNNNNNNNNNNNNNNNNNNNNNNNNNNNNNNNNNNNNNNNNNNNNNNNNNNNNNNNNNNN
NNNNNNNNNNNNNNNNNNNNNNNNNNNNNNNNNNNNNNNNNNNNNNNNNNNNNNNNNNNNNNNNNNNNNN
NNNNNNNNNNNNNNNNNNNNNNNNNNNNNNNNNNNNNNNNNNNNNNNNNNNNNNNNNNNNNNNNNNNNNN
NNNNNNNNNNNNNNNNNNNNNNNNNNNNNNNNNNNNNNNNNNNNNNNNNNNNNNNNNNNNNNNNNNNNNN
NNNNNNNNNNNNNNNNNNNNNNNNNNNNNNNNNNNNNNNNNNNNNNNNNNNNNNNNNNNNNNNNNNNNNN
NNNNNNNNNNNNNNNNNNNNNNNNNNNNNNNNNNNNNNNNNNNNNNNNNNNNNNNNNNNNNNNNNNNNNN
NNNNNNNNNNNNNNNNNNNNNNNNNNNNNNNNNNNNNNNNNNNNNNNNNNNNNNNNNNNNNNNNNNNNNN
NNNNNNNNNNNNNNNNNNNNNNNNNNNNNNNNNNNNNNNNNNNNNNNNNNNNNNNNNNNNNNNNNNNNNN
NNNNNNNNNNNNNNNNNNNNNNNNNNNNNNNNNNNNNNNNNNNNNNNNNNNNNNNNNNNNNNNNNNNNNN
NNNNNNNNNNNNNNNNNNNNNNNNNNNNNNNNNNNNNNNNNNNNNNNNNNNNNNNNNNNNNNNNNNNNNN
NNNNNNNNNNNNNNNNNNNNNNNNNNNNNNNNNNNNNNNNNNNNNNNNNNNNNNNNNNNNNNNNNNNNNT
TCTTTATGGTAGCTTGAAGAAAGATAAACAAAATAATGAAAATAGTAAAAATAATGAAAAATAAAGAACT
TTGAAGAAAAATAAAAAAAAAAATAATAAGAAGAAGAAGAGGAAAAAAGAAAAGAAAGATAGATTAAAAT
CATATGGCTCAAAGGAGGGACCATAAAGTTCAACATCATTGCTACAACTCTTTGGATCATATGGCTCGAA
AGAAACAATAGGCTCTTCAAAAGCCTTGACAGAAATGATGTTGAGATTTGGGATGATATTCGCGCTCTCA
CGGGTTTATGGATTAGTAGATCGAAACTTTTTTCAAATTATAGTGCTAGCTCTATTGCTTTAAATATTAG
CGGTTTTGTATAACTACTCATGGGCTTATCTCTAGCCCTTCCTCTCTTGGCTTCCTCTATTATTGTCTCT
TATTATCAATGAAGTGGGAGTGATGCGACTACTGAGGGGTGCACCCATCTGACTCATCATATCTTTTTTT
TAAAAAAACAAAATATAAGGAAATGATTGTATATATTTACTTTCTTGATATCGACAAAAAGACCTAACAT
GAAGCAGAATTGCAAGAGAACATGCTTCCTAAGATAATACCTACGGTACAAAAAACCATCTTCGTCCAGC
ATGGTAGCATCACCAGCATTTGGATTGTTTCCTTCAATCTATACATTTCAATAGACTCAATCAATCACAA
GAGATAATCAGAATGGATTGGACACAACCCATTTGATATAGTTTCAAGTCAACAATAGATGGTTGATTCA
ATGAAATACAATTCAAACATATGATTTTACTGTGTTTTCAAATTACATTCTTTGAAGAAATTTGTGCGTA
AAGGTCACACCGTTGGAAATTCAATACCTTTGCAAGAATGTTGTTGAGAAGATTATTGTGATGAATCTTT
GAATCCAAATTCTAGAGTGTTGTTGCACTTCAGTCTTCAAACACAAAACTCGTCCAGATCCGGCCACCAG
TAATTAAGAAAGAAGGTAACAAATGAACGGCAGCAGAATAGATGGATAAATGAACAGAAACTGCAAAAGA
AAAACTATATTAGTAGATGGTTGAGTAGAGAAACTTGCTCTCTTTAAGACGTTTTGCAGCTCTGCTCTGA
ATTGTGCAAACAAATTGTTGTCCCGTCGTCCCTGGGATAAAACAACCTCAGTACTCGTCAGCTGATTTTG
CACATAAATCAAACTAGTAATCTATAAATCACTCAAAGCTCGATAGGCACACACACAGCTCGGGAGACTT
GAACAGGAAATGAAATGAGGAGAAGATCGTTTTTCTATATTGTATCTAGAAATGAAAACTAAGGGATATA
TATATATATACTAGCGGAGAATCAGCAACAGCCAAAAGAATTTAATTGTGAAACGTCACAAATTTATTAT
AATAAAATGTTTAACGGTCGAATTAATAAACGTTTAACAATAAAAAAATTAATTACGTTTAATTATAATT

CTTAAAAACATTAGAAAATTAAAATATATATATTATTTCTCTACGCTCTGTCTCATAGACGGCGGTAGCG
TGTGGAGATATAGGCATATCCTCATTTGCCTATATGCTCCCTTCTTCCAAAATTTGGGTGTCCTAAGGGT
CCAAACCCATTGGCCAACCAATAGGTATATGAAGTAGCCTCTCCACATTCTAAATTTATCAACGTGTGAA
TGCTTTGGAGAGTGAAATAATATATATATTTCTCTTTACAATTTTTTCATCCAATATGGAGGCAAGATAA
ATAAATGAGTCCCGATTCTAACTGTTAAAATAAGAACAGTGGAGTTGAATTATCAGCATTTATTTTAATC
AAACTTATGAGAGCAAACAAAATTACAGAGAGAGATAAAAGAAATAGTCTTACGTTTGGTGAGTTCCTAA
GTGTTGTGGCAGTCACAAGAGTTATTTCACTCGAATTTTCGTAGAGAGGAAGCTTCCATTCAGAGACCGC
TGAATATATTTATAACATGCTCAAGTTGGCTTGAAGCAAAATGAAAGAGAAACTACTGAAGTAGTCATAG
TTAAAGCAGCTCATCTAGAAGCATAAATGATCCTATAAGAAGAATTCATGAAAAGGAGGCTACAAAGCTT
CAGAAGTTCCTTTTTCTTTCTTTCTATTCGTTGCTCATGAATTATTCCACCGAAAATCATGGGTCATCAA
ACAAAGAGCTGTCTAGGATAACACCTACGCCAGTTCACCTCAATCGAAAAAATTGATCAGAAGCTTGAAT
AAGAGCTTGGGTGTTTCAGCAGCATTCTTGTATAATATGCATCAGCAAATAGGGGTTAGAGAAAAATTTT
GAATTCTTCTATTGGATTTCATTTAGAATGTTGACTTCTTAATCCAAGCTTAATCATGTTCTTTTAAGAT
TTTCAGGCGCACCGTTGGAACATACGAAAAAAGGTAATGCCACTAATAAAGTTTTATGCATAATTATTCC
ATCTATTATATTTTCAACTTGAAAGAACATAAAAACCAGGCTGGCTGCGTTGATACTAGAGCAAAATTTG
CATTACCAATTAAGGTACATCCGTAAAGATTGAACTGGGGGCTCATTCCGGAAGTGACCTACAAAGATAA
AAATAAAAACAACTTCTTTTCCTCAAACTAAATAATTTATCTAGGGTGTGTTTGGATTGGTTTTTGAAGT
GTTTAATTTTTAAAATAATTCATTTTAAGATAAATTGGGGTGCTTGGCATTTCGTTTGAAATAATTTTTG
GAAAAATGAGATTATGAAATAAAAATCATTTTAGACAAAACACATACAACCAAATTTTAGAATAAATTTT
TATTTTATGTTTAATAAGAAATCCCTTCCAAATATCTATTTTTCTTCCTTTATTTTTTCTATTCCTTTTT
ACTATCTTTTTTTTACTCCGTCGTTCTTTTTTTTTCTATTTTTAAATACTTTGAATATTTTTTTCAATGT
ATATGAACACATATTTAAATATAGAAATTAGCAAATGTTTCTCTCTCAGTCAAATCTATTTCGACTTAAC
ATCTGTGAAAAAAATTATGAGGACGTCAATGAAAAACATGAACGTTCGGTTCGTGAAATAAAGATGAATG
AAGAACATAATCGAATTGCACATCTTTTTGTCTAGTTGATATTATATTGTCTTGAAAAAAATTATTTATC
TTATTTATATTTTGTTGATGGAAATCAAATTTTTTTTTATGGTTATATATTTACATTTTAGATATTTGTG
CTTTTAAAATTAAATTTTTAAAAAATATTAATACGAAAAATGAAATTAATGTTTAGTTTCAAGTAACTAA
AAAATTAATAATTTCAAGATAACGTACAATTAATTTTAAAATATATTTATGAAAAAAAAATATTCAAAAT
TTGTGTATTTTTTTATGAAATTTGTTCATAAATATAAACCAAACAAACCAAACCCAAACCAAACCAAAAA
CCAAACCAAAACCAAACCCACAATTTTGTATATAAACATTTATAAAGAGGTCAAACCAAACATGTGAGTA
TTTAGATTTTAAACTCATTTATAAAAAGTACTTTTTAAAAAATGTATTCTTAAAAAAATATTTTTTTTCA
TAGGCAATCCAAATAGACCGTAAGTTCCTTCTATAATTTTTAATTGTAATTAGTGTAGCCAATGGTTGTT
TTATTAGATTTCATATTAACACTTGATAGAAACTATTATTATAAGGAAGAAGACTTGGGGGGAAGACGCG
TTGTTCCAAAGTCAATATGAAAAACACAGAGGAAGGTTCTTGGAAAGAAGAAAGGCACCAAAAATATGAT
ATAAAAAGGAAGAAATAGAGAAACAGAGAGAGGCACCAAGTATCGTTAAGTTATTCAGTATTCTTCTACT
TTTCGATTCCTAGATATGAATCGCGCAAGTGGATCATCTTCTTCCTCACGTTTTAGATGTAGTTTTGATG
TATTTTTAAGTTTTCGAGGGGAAGATACTCGTTCCAACTTTACCAGTCATCTTAATATGGCTTTGCGTCA
AAGAGGAATCAATGTTTTTATAGATGATAAGCTTTCAAGGGGTGAAGAAATTTCTGCATCTCTTTTGGAA
GCTATTGAAGAATCCAAGATCTCGATCGTTATAATCTCTGAAAATTATGCATCTTCGAGTTGGTGTTTGA
ATGAACTAGAGAAAATCATTATGTGTAACAAATTGAGATCAGGAGAACAACTTGTTTTACCAATTTTCTA
CAGAGTGGATCCATCTCAAGTAAGAAAACAAAGTGGAAGATTTGGAGAAGAATTTGGGAAACTCGAAGTG
AGATTCTCATCGGACAAGATGGAAGCATGGAGGGAGGCAATGATTTCTGTTTCTCATATGTCTGGATGGC
CGATCCTTCAAAAAGAGTATTTTTCTTTTTCATATATATCTTTCCAACTCTTTTGCTCTTTCATATTCTA
TTGTTATTATATATACTTTGACTATCTCAAGGTGAGAGCATGCTCATGCAAAGAAAATGTGACATTTTAA
GTTCCATTTGATAACAATTTTATTTTCAAGTTTTTCATTTTTGAAATTCATATACACTTGTTATATAATA
CCAATTTTTTTTTTTAATTATAATGAGTTTTAACTTTATTAAGGAAACATTTGAATTTTTAACATTAACTT
TAAAAAAAAAAACAAGATTTGAAAATTATATATATATTATGTATGTGTGAAAATTTTCAAATTGTTTTAA
ATGATATAATTGTTGAAAATTTTATATGTAGATTGAGATAGACTCTTATCTATTTTTAATAAAATGTAAA
CTTTTGCTATATTTGTAAATAACTTAGCTCATTTTTCTATATTTAAAAACACCATCTAACTTTCGACTTA
GTTGACTTAATTTTTAAAAATAGTAGAAGAAGGCATTAACAAATCATAAAATCTTATGACTGAAAATAAT
GGCACATGTCCTCGATTAATTTTAGAAAAGAAAATTAAAAATTCAAATACCAAAAGTTATTTATACAAAG
CCATTAAAATTGATTTTTGTAAAATTGGAATTATTTCATAATATTATTTAATTTGTTATTATTGTCCTTT
CTCTTAATAGATATTAACTTTTAAAGTTGGATTTAGTTTGAGTACTTTATAACGTTGCTTTCATTTTTTA
CGGCCTTCTAATATTATAGGAATTATAAGTGAATTTCAAATCAACTTTTTAGTGTGTATGTCTCTTTCTT
AAAATGGCAGTGACGAGGCAAATTTGATTCAAGAAATTGTACAAGAAGTCTTGAAGAAATTAAACCATGG
AACAATGCAATTGCGTTTACCTAAATATCCAGTTGGAATAGACAAACAAGTTAATAATATACACTTCCAA
GTTATGTCAACAGATGAAAAAACTACTATGGTTGGATTATATGGAATTGGAGGTATCGGCAAGACAACTT
TGGCCAAAGCATTGTACAATAGGATTGCAGATGACTTTGAAGGTTGTTGCTTTTTGCCAAAAATTAGAGA
AGCTTCAAATCAATATGATGGCCTTGTTCAACTCCAAAAGAAGCTACTTTGTGAGATTCTAATGGATAAT
TCGATCAATATTAACAATCTTGATATAGGGATTAACATCATAAGGAATCGACTATGCTCAAAAAAGATTC
TTTTGATTCTTGATGATGTTGATACGAGAGAACAACTAGAAGCATTAGCGGGAGGACATGATTGGTTTGG

ACACGGAAGTAAGGTCATTGCGACAACAAGAAACAAACAATTACTTGCTAGTCATGGATTTAATAAATTG
GAAAAAGTTAACGAATTGAATGTCATTGAAGGCCTTGAACTTTTTAGTTGGCATGCATTTAGAAATAGTC
ATCCCTCAAGTGATTATTTAGACCTTTCAAAACGTGTCGTACGTTATTGTGATGGTCTTCCCTTGGCTCT
TGAAGTTGTAGGTTCCTTCCTTTACTCTATCGAACAATCCAAGTTTAAACTTATATTGGATGAATATGAA
ACTCAATATCTTGACAAGGGCATACAAGATCCTCTTCAAATAAGCTATGATGGACTTGAAGACGAAGTGA
AAGAAATTTTTCTTTATATATCTTGTTGCTTTGTAGGAGAAGATATCAACGAAGTTAAAAAGAAGTTAAA
AGCATGTGGTTGTTTATGTCTGGAAAAGGGAACTACAAAACTCATGAATCTATCACTTCTTACCCTCGAT
GATTTCAACCAGGTTGAAATGCATGATTTAATACAACAAATGGGTCGCACAATTCATCTCTTGGAGACTT
CTACATCTCATAAAAGAAAAAGATTGTTGATTAATGATGATGCTATGGATGTGTTAAATGGCAATAAGGT
AAGAACCATGGAGCAAAACTAATTTTTATATATGCTTATATTTATTTATTATGAGAACAACTTTTATTAT
TTTTCAATGTTTGTTTTGTAACCTGTAATTTTGTAGGAAGCAAGAGCAGTTAAAGTCATAAAATTAAATT
TTCCTAAACCTACGGAGTTGGACATTGATTCAAGAGCTTTTGAAAAAGTGAAAAATTTGGTAGTACTGAG
AATGCTACATCTTCAAAAAGTATTGATGTGGAGTATGTACCTAATAGCTTAAGGTGGATTAATTGGCCTC
ATTTTCCTTTTTCATCTTTGCCTTCAACCTACACAATGGATAACCTTATGGAATTGAAATTGCCATATAG
CTCCATCAAACATTTTGGGAAAGCATTCATGGTATTACTTTTTCTCAATAATATTTGAGTTATATTAATT
TAATTACAATGTCGGCTTATTTATTTGAAATTTAATTATATTTTCCTTTTATATTAATGGCAGTGTGGTG
AATGGTTGAAGGAAATTGATCTTAGTTTTTCTGAGTTTTTGGTGGAAATTCCTAATTTAACTGCTGCAAT
AAACCTTGAAATGTTGGATCTTCAAGGGTGTATAAATTTAGTAAAAATTCACGAATCTGTTGGATCTCTC
AGTAAGCTTGTCGAGTTTTATCTTTCTAGTAATATTAAGGGCTTTGAGCAGTTTCCATCGTGCCTCAAGT
TGGAATCCCTTTACACTTTGACATTGTACAGTTGTAGAATAGATGAACGATGTCCTCAATTTAGTGAAGA
AATGAATAGCCTAGAACTATTATGGATTAAAGATAGTGTTGTAATTAATCAGCTATCTCCAACAATTGAA
TATCTTACTAGCCTACAACAATTGTGGATCATAAACTGCATGGGGCTCAAAAGTCTTCCAAGTACAATTC
ATCATTTACGTAATCTTACACTTTTATATGTCGATAGATCTGATCTTTCAACATTTCCTTCCTTAAATAA
TCCTTCTTCACCTTCCTTATTTCGCTGCCTACTTAACTTAACATCAATAACCCTTTGCCATTGTAAAATA
AAAAATTTGGATTTCTTAGAAACAATGGTTCATGTTGTCCCTTTTTTGAGACAGTTGAATTTATCTGAAA
ACAACTTTTGTAGACTACCCTCATGTATTACTAAATTTAAATCCTTGAGATATCTTTATACAAGGGATTG
TAAGTTGCTTGAAGAAATTCCAAAGGTTCCAAAAGGAGCAGTTACTATGAATGCTTCAGGGTGCATATTA
TTGGCCAGATTTCCTGACAACATACTTGATTTCATATCTTGTTATGATAATTATATGGTAGGTATCATTT
CTTTCTCATGACCTCATCTCTCATATATTTTGTTCATATAAGAATTAAGTTTATGTCCTATTTTAATTAA
TATAACTTTTAGCTATTTACTCTCATCGTGCAGGAAGAGAAACATGAATATAAAGTAATCAAAGAACTCA
TATTAATGAATTGTGATATTCCAGATTGGTGCCAATACAAGAGTACAAACAATTCAATAACCTTTCTTTT
TCCGGCTGATTATCCAACTTGGGAAAGGAAGGCTTTTATTGCTTTTTGTGTCAAATTTCAAGTTATTGAT
GAGGAATTTAAGGTAGACTCTAGAGTGTTTATCAACGATTTTGAAGTATATAACGGCCACTTTTGGACGA
ATGAGATTGTAGGTCGAAAAAGACCACGAGGTGAGTATCTATGGATAGAAGTAATTGATCCTGATATACT
TCTCGACCCATATGATGATTGTGAACAAAATCAACCAATTATTTTTGATAGAGTTACAGTGTTATTTGAG
GTCATTACTCCAAACGCAGTAAATATAAAAAAGTGTGGTGTTCACGTGATCATGGAGGAATAATTTGGAG
AAGAAATTGCCAAAGTTGAAGTTAGAATATGCAAACCAGAAGAGAGTCTCTAATTACTCTTTTGCACTTC
CTAATTTTTATGTTTATAAATATAATGTTATCTGAAGATAGCATGTGTTAATCAAGAGAAAACAGAAATT
GATATTTGATGAGTCCCCTAAATATTCCTTAAGAATTTCATTTTCTGCTCTTTTTTTTTCAATTTATATTT
GTTTTCTCCTATCTTTTTTAATTACGGTTTTCAAATTCAATATAGAAACACTTGAACAAATAATCCAATG
CTATGAAAAAAGGAATTTGAAAACTACTTTCTTAGTTCAATTTTCAAACCATCACCTGCATTTTTAAAAT
AATTAAAATAAAAGTAGATATAACAAATTATAGAAATTTACTTAACACTCGAGTTAATAACTAATATAAT
AACTCGGATAAAACAATAAGCAAGAATAACGATCAGAACACCAAACATTGGTAACCTAATTCGATGATAC
AACACCTACAATCCTTGACCCATAATAAGAGATCGTATATTGCATTGTGATATATTACATGATTACTGAG
ATATGAATTATACAGTATATACATCATCACACATTCTCAACATGTTGTACATGGTGATAATATTACACTG
ATAAATGAATTAGAAGAGTAATCGATCTTTATAGTAGCAATGAAGGAAACACTCTCCCTCGATCAAAATT
CACCATGCAATAGTTCAATCTAGCTTTACTTGATATTCTATGATCTCTATTAACTAAGAACTTTTCTTCG
AGTAGCGAACTTAGTTGATACTTTTTTTAGATTACTCACCACATAACACACTTTCTATCTTTTTCAGAAC
AGCACGGAATTTTTCTCAAAATCTTTCAATCTTAATTCACAATCTGCACACTTCGGGAACAACCTTGAAC
AGAATAAAAGTACTGGAAACAACTAGCTCAACAGAAGCTGAGAAAGAAGGCCAAATCTCAACGACCAACC
AAAACCGAAACATCCCAAACCAGGAAAGATAGATAATTATATAAATAAATAAACAAAGGGAGTGGGACCA
TAATTCTCAACAACTACGAAGTTAAAGCAACTATCTACACGTTGTAAATCATGGAACCTTAGAGTCAAAA
AAGATATTGAAAATGCTGCTGATATTTCGAAACTTAACTTTGATATTGATAGCAAAGAGGAATAGGCTAT
TATGACAGAAGAGGCCTTAAACTGGCGTTATGCATTAAAAATTGACCTACAAAATATTATGCTCCTCGAA
GCAAGGAAATGGAGACAGTTTTGTAAATACACATGGCTCAACGAAGGTAACGAAAATGCTGCTTTTTTTT
CCATAAAATGTGTTTGACAAGAAGACGGATCAATTTTATATCAGAAATCCAAAACACTCAAGGAGTTAAT
TTCTGTACTGACCCTCTGATTGCAAAAGATTTAATAAATTATTGCAATGACATATACATTGGTGAGAGAA
ATGTCAAATGGATGATTGGTGACTTGGATTGGAATGCTATTTCTCAAACACACATGCTGCAAATCTTACT
AAGCCTTTTGTTCATTACAGTTCTATGAAATCTTTGGGCAGCAACAAAACTCCTGGGCCTGATGGGTTCA
CCCCATCGAACTCTTTAAAAAATACTGGTACATTTTCAATACCGATATTTTGAAGTTGTTCCATGATTTT

TTTTTTTTTTTTTGAGAAAAGGATTATTAACAAATTTGTCAACACCACATTTCCATCGCTTTGATTCCAA
AGAGAACGACTGCATTGAAGGTGTGCGATTATAAGTCCATCAGCTTGACTACGAGCATTTACAAAATACT
TGCCAAAGTTCTTGCCGAAAGACTTAAGAATGTTCTTCCAGACACAATTGCTCCCCAACCAATCTGCTTT
TGTATCTGGTAGACAGATAACTGAGCTCATTCTCATTGCTAACGGATGTAGCCTCCTATTTTTATTCTAC
ATTTTTTAAAAAAGAATTATTAATTGTAGAAATGGTTAAGATATTTTACTCATTTATTTTGTTAAAAAAA
GAAAAAGGTAAAATCTTGTTGTAATAATATGAAGTCTTATGTTTTTAATCTTTTTATTAAAATGGAAAAT
AAAGTCATTAATGAGAATATCTATTATTTACAAAAAAATTCATATTATTTTGACTTATCACTTTAGGAAA
AAGGCAAATTAATTTATGAATACAAAATCTATTTTGATTAGGAAAAGCAAAATAATTTATTTTATACATA
ATTCTTTAATACCATATTTGATTTATCTTATCACTTTAGAAAAAAAAAAAGCAAATTAATTTATGTATACA
AAATCTATTTTGATTTATCATATTAGGAAAAGCAAATAATTTATTTTATACAAAATTCTTTTAATACCAT
ATTTGACTTATCTTTATCACTTTAGGAAAAAAACAAAAAATTTATTTTGGACAAAAATCTTTCAATATCA
TATTTTTACTATTTTAGAAAAAAGAAAATTAATAAAATTACATAATATCTAATTTGATTTTTTTACTTAT
TAAATTTTCCTAATTTGCAGCACACCCCGGGTCTATAAATAGGATCCTTTGTCATTTGGAAAAAGGGGGA
GAAGAAATTGTTTTAGAGAAAGTCTTGAGAAAAAAATTGTTTTAGAAAGAATCTCTACGAAAAAAAAAGTGT
TTATAGAGAATTTGAAGAAACGTATTTCTCTGCATAAAGGTGGGTCATTAGCTTTTTCGCTTTATTGTTT
TTATTTTTATCTTCATATCGTCTTTTTTTTTAATCCTAATTCTAATCTCATATGGAAGAAAAAACTTAGT
TGGAGGTTGCATTAGGTAGGGTTTACTCCCAATGATCCGCACCTCATACAACAAGTAATTTTCTTCGGGA
TTGAATTCTTATCTTTGTTCTTCTCTTTTCAAATGTAAAGAGAAAGAAAAAAAATATATAAGGAAAATTT
CTTTTTTTATTTTTACTACCTTTTTATCATCATGCTAAAAGAAAAAAAAAAGATCTTTTAACATTTTTCT
TCTCTCTAAAACAATAGAGAAAAAGAAAAGTTATAAACAAAAGAAAATTTCTATTATTATCATTTTTCTT
TTTATTATTATTATTATTATTATTATTATTATTATATTTTTTTTCTCTTTTAGGGGGGCGGCAAACCTTG
CCGTCACCCTCTGTTTCCTTTTTTTTTTCTTTCTTTTTTTAATTTATTTATTTACTTATTTAAATTTAAT
TTTATTTGTAATTTTATTAATATTATTTTATTATTATTATTATTATAAATATATATTTAACCCTCTGGGG
TTTTTTTTTATGTTTTTTATATATAACTTTATTTATTTATTTAGTGTTTTTATATTTATGATTTTTTTAA
AAAAACTTTTTGCTATTTTTATTCTTATACCATTATTGTTATTTCCTTTCATAATTTTTTTTTTTATCTC
TTTTAACTTACTATCATCCCTATGTCCTCTTTTCTTCACTTATTTATTTAATTTCTTCTTTACATATTCA
TTTCTTTATTATTTCATTTAGTTCTTCTTTTTTTTTCTTATTTTCTTTATCGTTATTATGTTTCTTTTCT
TTTCCTTTTCTTTTCCTTTTCTTTTAATTTATTTTTTATTATTATCGTTATATTTAGTATATGCATACAT
TGAAATCCTAAATTATTTGTCTAATTTATTGTGTGGTATATTTTTTGCTTCTATTTGACTTTCATTAAAA
ATTTCTTGAAAATTTTAAGATACTTTTAATCATAATTGTATTTAATTTCGAAATCTTTTTTTTAAAATTT
TTTCTCTTTAAACCATTTATAATCTTTTTTTTTTTTTGCTTTAAAATTATTTATTTTAAAACTCAAAATTA
AATAGATATTTCATAAGGTTTCCTAATCTACATTTCTTTTATAATAACCACGCCAATTTCGATAAAAAAA
CCTATGAGGGAATCTAGAAATTTCTGGGAGTCCGTTATTTCTAGATTCTTGAGGTGAGGGATCAATATCT
TAGGGAGTCTGAGATTTGATCCCAAGAGAAAATATATTTAATCAATGTTTTAAAAAAAAAAACTTTATTC
GAAGACTAGCCTATGATAGAATTTGAGAAATTGTAACAATTTCTCATTCTCTTAAGGTGAGGAATTTTCC
CCAATATAGTCTAATTAATGGGTGTATTCCACTTATTTTATGGGATCATTGCTCCAAATATTGGAGTGGT
GAGCAATGAAATAAGATAGAGTTCTTTCTTTTAAAAATAAATTTTACTCAAGACATTAAAATTGGCCATC
GTTTTCTAAAACGGGTGTTATGGGGTGCTAACATCTTTCCTGTACACAAATGACTCCCGAACTCAACTTT
AATTTTTTCGTAGACCAGTTTTTATTTTATTTAAAATGATTCACTTTATTTCGGTGTCCAATCACACCGT
AAGAAAGATTGGTGGCGACTATTTTTTTTTTTTTAAAAAAATAAACCTTTTTAAGGATGTCGGCCGCTCCG
CGTCGTCTCGGGTACGTGGCGACAATGGAAAATGAGAGTTTGAGAGATTATAGGTCGAGAAAAAAGAACA
AAGGTGCATTATCTGATCATTCTTTTTAAATGCGCCCCCAAAATTCAACATTCCAGTAGGAATTATTACC
AACAACGTAAGAGTACTGAGGAAGGAGAAGATGAAAATGAGAATTTATCTTTATAATTATTCTTTTGCTA
CAGACGATAGACAGCAAGGGATCATATATCAATCGACATCCAAACTAGATATGAAGAATTAAATGGACCG
ACTTGATGTCAAAGCGACCTCTTTTCTACGTACAAGGATTGCTAAATGGAATCATATACATGAAGCAACC
ACTATTTGTGTGGTAGAAGGAGTCATATTCACTGATGCTGGGAACGTATGAAGTAATTAAATAAACACAA
TAAAATATAGGAACAACTTCAAAACTAAGCAGAGTTAAATAACACATTTTGTTCGGTGCTATCATATATC
CTTGGTGCAACGACTTGACTCCATTGTCCTTTAGTAAAAAAAAACTGTTTCTTGTTTATCCTTTAATGGT
TGATTTTTTTAACACATATTTTAAAGTCAACTATTAAAAGTCTTCCCTTTTCTAGAACATTTCGTAACCG
GTCGGCCATCACATAATTCGTATATAGATATTCCACTATTATAATTAGATTTCACCTTTTCGATAAGTAA
TCTAAATATAATCTTATAAAATATTCAATGATCATCTCTGCAACATTAATAATTTATTTCATTAGATTTG
AAAAAGTCGTAAATATTTGATGATGACAAGGCCCATTAGCAATGAAATAATTTAACAAAACCTAAACTTG
AAGAAGAAGTTAAATATGTTTAAACAATTGCACTTCGTGTTAAAGGAAATATCGGACGAAATTTATTTTG
GTCTTTCAAGCATATAATAGGCCTAATTTGAGAATGTCTCCATCAACGAGAAAGCAAATATCAATTGAAA
TTTCCTTGGCTAGTTAAAATAGATAGTAATTAAAAGAAAAATACACATATTGGCAATGTCTGTCACCGAT
AGAACCTCCATGGATTTTATTAGGAAAAAATATTGTCGTTCATGGTCTATCATTGATTGTCCATACAAGT
TTATTACCAGCAAAAGTTTATTATTGATAGATCATGATGATTTATCAGCATAGAAGTCTAACGATGGTAG
ACTTTGTAGCAAAAGCCCTATTCGAAGAATCGATGGGGCTAACTGGATTTCTCACAAGGAATCACCCTAT
TTTGTTTTTTGGAAAGATACGAGTGAAGGGAGCTAGAGAGCAGCCATCCATTCACTAATTTTATTCCAGA
AAGATGTAGCGATTATAGATTGTTTACAAGGATTGGGCAACAAAAAGCTTTGATGTTAAGAGAGATTGTG

GCGTGGCTGTAGACCTTGAGCAATTTGCTTTTTGAGGACCAGTAACTAATAAGGGTGCAAATGTCTTGCC
AAGAGTTTCGAAGATTTGTTTCTTTTTCGGAGAAGATTTGTTTGTTTCTTCCCAACCAAATTTTCCAAAG
GGTAGCAACGGCTGCGTTGAAGCTGATGATATTTCTTGCATTGCTTGTTTTGATTTTGCACAGACTAAGG
CATAGACCTTTTTGTGTTTGTGCAGGTAATGGGAATACCCGTATTTGTGCTTTAGAGACGCCATAGTTTT
TGTGCTTTGGGGCAGAGGATGAATAGGTGATCCAGGTTTTCATTTGAGAATTTGCACGAGATGCACCAGC
TTGGACTAAGGTAATGTGCAGATTCTTCTTTTGTATTTGGTCCATCGTGTTGATTTTCTTTCTGGTGTAT
GATAGACCATATGAAGAACTTAAATTTAAAGGGGATGTAAGATTTCCAAAGCGTTTTGAATTTGCTTTTT
GATGTATCCCCCTGTGGTTAGAGCTCTCTTTGAAAATCATGTGTTTTGCTGATGCAATTGTGACATAGCC
ATTGTCATAAGAACCCCAAATAGGTTTGCTTTGGCCTCTGTCACTTCGTAAAGGGGGCATTAAGTTTTTT
AGGTTTTGCCAAAATTGAGCCTCCCTTTCATTAAACGGTCTTCTAGGATGGATATTCCAATTTTCTATGC
TTTGATCCCATACCTCTTTAACTGTTGCGTTTTGTAAGTTTGAGAGGGCAAAGATTCTTGGAGCTAGATG
GGATTGAGGAAGGTTATTGCACCATCTGCTATGCCAGAAAGAAAGACTTGAACCGTCATTGAGATCCCAT
TGGATTTTTGAATCATACCAATTCTTCCACTTTTTTATATGATGCCATATTGGGGATTTTGCACTGCAGT
ACTTACCCTCTATAGGGATATCTCCTTGATGGTCTTTCGAATACTTTGGATCAATACATTTTTTTCCACA
GTGAATTAGGTTCAGTATGGTATCTCCACATACTGGACATGTGCATGTGTTCCAGTTAATAAGGTTAAAG
CTCTGTCTATCTTGGTTGCCTTTCCCGAGGAAGTCTCTCTAGTGTTTCTCTATTTGTTTATATACTGAGG
CATGAGCTCTAAGGTGGATAGTTGGTAAGTGGGTAGACTTGTTAGTGAGGATTTGATGAGGGTAAGTCTA
CCTCCTTTTGAAATTTGGCTGTACCTCCAACTGTTTAGTTTTTTGTGAACATTCTCAATAGATTGGTTGC
AAAAAGACTTCGAGATAGGATTGCCACCTAAAGGGTCTCCTAGAAAGCTACTGGGAGGAATTGGGTAGTG
AAACCAAGTTTGTTGGATATGTAGGTTGTTCTATCTGCAGGGATGTTTACTGGATTAATTATAGACTTAG
AGTGGTTGAATTTTAGTCCAGATGCTCTTTCAAAGAGAGAGAGGGCCATTTATGAAGGTTGTCTATGTAG
GTGTCATCGTCCTCCACAAAAATGAGAATGTCATCTGTAAATAGAAGGTGGGAGATATGGTAGCTGTTAT
TGAGAGTAACTCCTTTTATTGCTCCTCTAGCCTCAAGGTGCAGTAGTAGTCTACTTAGGTAATCCATGGC
CAAAACAAAAATGAATGGGGATAGAGGGGTCACCTTGTCTAATGCCTCTGTGAGCTTTAATTCTGCCTTT
CGGATTGCCATTGATGAGGATTGAATAATGCACATTGCTTATGCAAGCTTTTATCCAGCTTCTCCACTTG
TCGGGGAAGTTCTTTTATTCAAGGATGAAATCTATGAATCTCGCTAATTTTATCGAAAGCTTTCTCTAAG
TCTAATTTGAGAACAAAGCTTTTCGTTTTCTTATTCTTCCACTAGTCAATTGCTTCATTTGCAATGAGGA
TTGCATCAGTGATTTGTCTTCCCTTGATAAAAGCCATTTTCTTTTCAACAATAGTTTCAGTGAGGGTAAG
TTTGAGTCTATTTGCAAGAGATTTTGCCATAATCTTGTAAAGGAAAGTGGTTAAGCTGATAGGCTTATAG
TCGGATGGGAGAGCACACTTTTCCTTTTTGCTGATAAGAGCAATATAGGTGTTGTTGACAATACCTTTAT
TGTAGAAGTTCTGAAAGACTCTTGATAAGTCTCCCTTGAGGTGCGCCTAAATTTTTTTTGTAGAAAAGCA
TTGTAACACCGTCAAGACCAGGAGCCTTTTCGTTGTTAATAGACATGATAGTTTTCTTAATTTCTTGCTC
CTCAAAAGGATTGCATAATTGTGAGTGATGCAAGGATGAGATTGGTTTCCAATCCAAGTTATCTATGAAG
AACTCTTCCTTGTGCTTTTTGGAGAAAATGTCTTGGTAGTAGAATTGGAGAACTTCTGAGATGGCCTCTT
TTGTGTCTACCATGTTTCTGTTTGTGTCACAAATAAAGTTGATCATGTTTTTCTTTGATTGATAGTACAC
ACTCTATGAAAATATGCACCATTTTCATCCCCTAGAAGGTTCCACTTCTGTCTTGATTTTGGTACCAATT
CTGCGCTTGTTTAGTTTCAATGTTCAGCAGGTCGGATTTGAGGGAGATTCTTTTCATGTGATGAGCTTCA
GAGAGCAGACCTTGGAATTCCAACTTGTCAATCGATTCAATTTCTACCATGAGGAGTTTTTTGTTTGCCT
CAAGTTGATATGTTCTTTTACCTTGCCATTCTTTGATGTGTTTAGACAAAACTTTGAGGCTTTGTATAAA
AGCATAACCAGGGTAACCTTCTTGTTTAGATTTGATCCACTAGTCTTGTAGGTTTTTCTTAAAATCTTTG
TGTTGGGATTTTGTCAAAAAGATTTTATGAGATTATCTTTTATTTAATTCTAAAAAGATAAAAGATTCTC
CTTTATCTTATTTTATAAGATTATCTTTTATTAATGAGTTATTTAGTTAAAAAAAGATAAAAGATCCTCC
TTTATTTTAGGAATCTTGCTAATTATCTTTGAAGAAATAAGGTTTGTACATATAAATTTTATATGTATAA
ATAAGACTTTCGGATACAGGGTTCGTGGGTGGACATATACTAAAATGATTATTCACGGCTGAAGGTTAAT
GAAGAACTGATTGCAAAAGCTGTAGGAACGAAAATGCAGTATGATCTTATGGCTATAAGATCAACATGTT
GATCTAAGTTGATATTGTTGATGAGTAGTGAAAAGGACGAGACAGTGTGGTTTGCGTGATCACAATAATC
TTTAGAAGTATATAAAGATATCGTCGAAGACATTCACTCATGCATTCAGGGGGGAGCCTGAAGTCTGAAGC
TAATATGCATGTTATATAGTCATATAGTTGAACAGAGTACATATGCTGTATCGATGTATATTGACTAAAA
TGAATCTTAATAAAATTACTCATCAGGGTTGTGCGCAGCTCCCCAGGCGTAGACAACATTGGCCGAACTG
GATTACCAAAGTTCCTTGTGTAATTATTCTCTTCTGTTGTCCCTCTAGTTATTACAACTGTTGTTAGCTT
TAATATTAACTGAATGTTTAATTGATTATCTCACTGTTTTTTCACTTGGTCTAGCAAGGAGGTTTCGTAT
AAAGTCTGAAAGGGCAAGGTCCCCATTTGATATGTGGAGTTTCCAGAACAATAGGAAAGTGATCCAAAAC
AACTCTTTGAAGTGTTCTTGAAGAGTGGATATTGAAAAGGTTTTCCCATCCTTTAGTGTAAAGAAAGCGA
TCCAAATGGGAAAAGGTAGGGCTGATTCTGAGGTTAGACCGAGTGAAATTGTTGTTGATAAGGGTGGATC
AATGAATTCATTTGCTTGAATAAAGTCATTGAAGAAGGCCATATTCCTGGTGTCCAAACAAAGTCACAAG
GAATCACACTATTAACTACAAAAACCTACGGGGATCCAACATTCAAAAGAAATGCAAGTTCTTTCTATGG
ATGTTAAATCATGGCATACTTAACACTATGGATGTCATTTAGAAAAGAAACCCAAATACATCCTTAAGTC
CAAATTGGTGTATTGCTTGCAAAATTCATGTAGAGGATATAAACCAGTTATTCATACATTGCAAGAAGGT
TAAAAATTTATAGGACAAATTGGAAGGGATCCTACCAACAAAGCTCAATCGATGTAACCTAGTTCCCCTA
TGCGTCGAGCTCTGCAATCTCCACCAAAAAAGTCCCAGTGCCATTAATCAAATTCAACCTAATTGCAGCC

ATTCTATGGACTATTTAGATCAAAAGAAACAATATAATCTTTAGGGAAATGCATACAAGTTCGACGGCCT
CTTGGGAAAACATCTACAACCTCGTCGGCACTTGGTCCTCACGACACCATTAGTTGAAGGATTACAGTAC
TAGTACCATTGCCATGAATTTAAAAGCACTTTTTTCCTAACTACTTTTGACGTTTTTGATGGTTTTGGCT
TACCTCTAACCTCTACTGCAACTCCATTTCTAATAAAGGTAGGATAAGAGGTCTTCCCCTCTTATCCGGT
TTGTTTCTTCTAAAAAAAAAAAAAACGATGGTAGACTTTGTCATATTTATAAACACAATATGTCATGCTAT
AATTAAATGTAATTCATTTTTTAAAATATAATTAATATATAGCTAACGATAGATAGTATAGTAGTCTATA
AAAGTAAAATATAGTTTTTTTTATTGGATCAGGAATACTCATTTGAGGTTTATGAACAATAACTATGATA
CTTGTAAGCCCCAAGCCCAGTGTTGGAACAAGGATTTAGAATTCGAATCTCCGACATTCCCCTTGACCTA
GCAACAACACCTTTACTTGTCTTAGAGGCTAATATTCATTTCCCTTTCCTAAATTCATGGCATTGATATA
GTATGAGGTTCTATCTCAAAACCCAAATTGGCAACGAGAAGAGAGTAACACATCTATTTTATAAAGAGAA
TTTAAGTTCTCTTGATTTTTAAATGAGAATCCAATATTTAAATCTACTACCAAAGACACATATACATGAA
CATGAAATACCATTATACTCTTATTGCAATCTATTTTTCATTGAATCTCTTGCTTTTCAAAATTTTGTTT
TTAAATTCTCTACCAAACATGCCTTTAGTCTCTATAGTTGAATAAAAGTTTTCAATACACGTATGAAAAA
TATGATATATAATATTGCAATAGTCATACATTAGCTATATGTCTAATCTATTGGGCGGTGCAAACTGTCA
ACAAATTGCATACTTTTCATTGATTATGATATTTAAAAATTGCAGTAATGATGAATATTAGTGGATAGTT
TTCATTGAATATGATATTTAAAAGAAGTCAAAGGAAGGATATGGGATATTTCCAAACTTTCAAATGATCA
TATAATCCCAACGCAGAGAATTTAAGATATTTAAAGTAAACCGGCGGCAGAAGAAACTTTCCATAGTTGG
CTTTATATCTGATATACCTTCTCATTGCTCTGTACCTTCAATTTTCTTTCATTATTCTTGTTTAAGATTC
TTTGATATGTATCGAGCAAGTGGATCGTCTTCCTCGCATGTTAGGTTGCCTTTTGATGTATTCTTAAATT
TCAGAGGAGAAGATACTCGTTCCAGCTTCACGAGTCATCTTCATATGGCTTTGTGTCAAAAAGGTGTCAA
AGTTTTTATAGATGATGACAAGCTTCCAAGGGGTGAAGAAATTTGTACATCTCTTTTGAAAGCCATTGAA
GAATCAAAAATCTCCATTGTTATAATTTCAGAAAATTATGCATCTTCCCATTGGTGTTTGGATGAACTAA
TAAAAATCATCATGTGTAACAAATCCAATAATCGGCAAGTCGTTTTTCCTGTTTTTTACAAAGTGGATCC
ATCTCAAGTACGACAACAAAGTGGAAGATTTGGAGAAGAATTTGGCAAACTTCAAGTTAGGTTCTCCAAC
AAGATGCAAGCATGGAGTGAGGCTCTAACTTTCATCTCCAGTATGTCTGGATGGGATCTAAAAAATTAGT
ATCTTTCTATATCCTTTTCTATTTCGTTTTCTTTTTTCGCTCTCCTGATCCATCTGTATTCTACCTATTT
TGTTATTCGTAGAAGAGCATGTAGGCACGTGAAAAATAGAAGGAAATTGTTTTAATGGATAAAATTGCTC
AAAAAATATATAAATTATATAGCAAAATTTCATAGTTTTTCGCAGTGATATACATATAATAGTGATTAAT
TATAGTAGAGACTATTACTGTCTATTAGCATCTATTAGACATATTTTGTTATTTTTGTAAATATTTTGGT
TCGTTCTACTACATTTAACAACAATCCTAAATTTAGGTGCTCGATTTCATTTTTTAGCTTTTTTGTTAAA
TAATTTACATAATTTTAACAAAATACAAAAATATTTATGATCTGTATAACTAAAAATAAAAGCCCATGAC
TCCACCATTGTATTTTCATAAATTTCAGACTTGCCCTTGAATATTGCGAATGATTCGTCACTTCTCTTTC
TTCTTCTTCTTTGCGATTTATTCATTGTCATTTGTGATTTTTTTCATCTTCTCTTCTAGATTTTTTTCTT
CTATTTTTAAACTATTTACTCTTCTTTTTAAATCTCCTATTTCAATTTCATCATTTTCGGTAAGATTCTT
TGAAGCTATTTCACGATTGTGTCAATGTTCTTCTTCATCTTCCTCATCATCGAGAATATCAAGATCGTTT
AAATATCTTTTTAGATGATCAATTCTATCGTTTACTATGGTCAACACAATCGTTTACCTTGGTTCAACAC
GATCGTTTAATAATTGAAATCTTTTCTCATCATTTAAAAGGAAATACTGATTGTGTATATATAATGATCT
AAACGATCGCTTATTACAATCATCACAATCGTTTAGAATGGTCAACACAATCGTTTACTATGGTCAACAC
GATTGTTAGATTTGAAGTTTTTAACAAACATTTACACTAGACTACACGATCGTTTGCCATGGTGAACATG
ATCGTTTAAATTTGAAATCTTTTTTTCCATTGTAAAAAAACTACACAATCATGTATCATGATCTAAATGA
TCGTAAATCATTCATTTAGACTGTAATACACGATTGTTAGGAAGAAAGATTACTCACGCACGCATGTGGT
CGATTAATTGCATGTTGACTGTGGTATTTTTGGTATTTTCCATTATGAGCTTGTGGACTTTTTCTATTTT
TCAAATTGTTCTATACGGTGTAAAATATCTTACTGGTTTGTTATATTTTTGAAAAAACCTTTTTTTGTTT
TTAAATTTATATTTATTATTTTTTAAAAAATTGTAAATATAATTTCTACCTTATTTAAAGAAAACTTGAA
TTTCTAGTTGAATTCAAAAAATGAAAACAAGTTCTTCAAAATTATTATTATATTATTTTTTAAGAAAACT
TTGTCTTAATATATAAAAATATTAGAATAAAATGAATAACAAATCATAAAAATTATTTATGGATGGAGGT
AACAATATTTAAGGTGAGTTGCATAAATGACATTTAAGCAAAAAAAAAAAAAAAAAAATTGTAGAATACAT
AACACAAGGATAAGATAATGGAAAATTGCATTAGATGATAAAAAAATCTATTAAAAAAAAAAAAAAAACAG
TCCATAGAACTTTTGTTTTTCCATATCTCCATCAGTGATAGACCATAAATGATAATCTATTACATATTGT
TAATATGAGTCTATCAATGATACTACTTGTAAGAGTATATTAATGATAGACTATATTGTTGGTAGGAGTC
GAGCATTGGTAATTTTGTAAAAATAATGTTACAGTTACCCGTTAATCGTAGGAGTCTATCATTGGTTAAT
TACCGTTGTTATACAATTTAAATTCTAATCCCCCATGAAACGTTGCAATTATTGTCTTTCAATTTATTTC
ATTTGTCTTTTCACCACACCTTTTAATGGATAGAAGTAGCGTTGGATTTAATTTGATTTAGTTTATTTGG
TCGTTTTCCTTATCTCTTCTGCTTCTCAGTTTAAGAAATGTTGTTGAGAAAGAAAAGTCAATTTCATTCC
TCTGTCACGTATGTTTAAAATTTTTCTTTAAAATGTCAGTGAAAGTGAAGCAAGTTTGATTCAAATAATT
GTTCAAGAAGTCAGGAAGAAATTAAAGAATAGCGGAACGACACAGTTAGATGTAGCTAAATATCCAGTTG
GAATTAACATACAAGTTAATAATTTACTCCTACATGTTATGCCCAATGGAGTTACTATGGTTGGATTGTA
TGGAATTGGAGGTATGGGCAAGACAACTTTGGCCAAAGCTTTATACAATAGAATTTCTGATGACTTTGAA
GGTTGTTGCTTTTTGGCAAATGTTAGAGAAGCTTCAAATCAACATTGGGGTCTTGTTGAACTCCAAAAGG
CGCTACTTCGTAAGATTCTAATGGATGATTCAATCAAAATTAGCAATATTGGTATAGGAATTAGCACCAT

AAGGGATCTATTATGCTCAAAAAAGATTCTTTTGGTTCTTGATGATGTTGATACGCATGAACAACTACAG
GCATTGGCTGGAGGACATCATTGGTTTGGACATGGAAGTAAGGTCATTGCGACAACAAGAAACAAGCAAT
TACTTGCTAGTCATGGATTTAATATATTGAGAAGAGTTAACGGATTAAATGCCATTGAAGGTCTTGAGCT
TTTTAGTTGGCATGCATTTAAAAATAGTCATCCCTCAAGTGATTATTTACACCTTTCAAAACATGCTGTA
CATTATTGTAAAGGTCTTCCTTTAGCTCTTGAAGTGTTAGGTTCCTTCCTTAATTCTATTGATGATCAAT
CCAAGTTCAAACATATATTGGACGAATATGAGAACTCCTACCTGGACAAAGACATCCAAGATATTCTTAG
AATAAGTTATGATGAACTTGAACAAGATGTAAAAGAAATTTTCCTTTACATTTCTTGTTGCTTCGTAAAT
GAAGACAAAAACAAGGTTCAAATGATGTTACAAGCATGTGACTGTCATTTCAGATTAGAAATGGGAATTA
AGAAACTCACGGATCTGTCACTTATTAACATTGATATGTTCAATTGCGTTGAAATGCATGACTTGATACA
ACAAATGGGTCACACAATTCATCTTTTGGAGCCTTCTAATTCTCACAAAAGAAAAAGATTTTTGTTTGAA
AAAGACGTCATGGATGTCTTAAATGGAGATACGGTGAGAAGGTTTACACGAAATTTGTTTTGTATATATT
GATTTTATTATTTCATAGATGACTTTAATTGAAAGTTTTATTTTGTGTCTTTGCAGGAAGCAAGGGCTGT
GAAAGCCATAAAGCTAAATTTTCCTCAGCCCACTGAGCTAGACATTGATTCAAGAGCTTTTGAAAAAGTG
AAAAACTTGGTAGTGCTCAAAGTTCACAACGTCACATCTTCAAAAAGTCTTGAGTATCTACCGAGTAGCT
TAAGGTGGATCATTTGGCCTAAATTTCCTTTTTCATCTTTGCCTTCAAGCTATTCAATGGAGAAACTTAT
TGAACTCACAATGCCAAGTAGCTTCATCAAACATTTTGGAAATGGATTTATGGTACTAATTGATCATGTC
TATATATACTTATCAATGATGCTTTAGTTTAATTAATTGATTTAAAATGTCAGCTAATTATTTGAAATTT
CATTCTATTTTCTTTTGTACGTAATTCATTGCAGAATTGCGAATGGTTGAAGCGTATAGATCTTAGCCGC
TCTGAGTTTTTAGAAGAAATTTCTGATTTATCCAGTGCAATAAACCTCGAAGAGTTGGATCTTTCTTGGT
GTAACAATCTTGTAAGAGTTCATGAATCAGTTGGATCACTAGGTAAACTTGCTACATTGGACCTTTCTAG
TCATTCTAATGGCTTTACGCAATTTCCATCCAACCTCAAGTTGAAGTCCCTAAAAGAATTGGTAATGAAG
GAGTGCAGGATTGTTAAACGGTATCCTCATTTCAGTGAAGAAATGAAGTCTAGTTTAGAAGAATTACGGA
TTGAGTATAGTTGTGTGACAGACCTATCCCCAACGATTGGACATCTTACTGGTCTCACACATTTGACGAT
CGTTGAATGCAAAGAGTTCACAACTCTTCCAAGTACCATTTGTCATTTAAGCAATCTTATTGCTTTAACT
GTTATCAATTCTGAACTTTCAACTTTTCCGTTCTTATATTCTCGTTCCCTTGCCTTATTTCCCCACCTAA
TATGTTTAGATCTTAGTAACTGCAATATAACAAATTTGAGTTTCCTAGAATCCATCACACATGTCGCCCC
TTCATTGACAGAGTTGTACTTGACTGGAAACGACTTTCGTAGCCTACCCTCGTGTATTGTTAATTTTAAA
TATTTAAGACATTTTGATATAAGGAATTGTCGGTTCCTTGAAGAAATTTTAAAGGTTCCTGAAGGCGTAA
TTTTTATGAATGCTCAAGGCTGCAAATCATTGGCTAGATTTCCAGACAATATAGCTGGATTCATATCTTG
TGATTTGGTACATATCATCTCTTTTCTCCTGGCCTTCATCTCTCTTGTACTTTTTTCATATTATATATAT
TTTAACGCATTCACTCTTTATATGGTGCAGGAATTTGTAGATAGAAAATACAGACAACTCATATTAATGA
ATTGTGATATTCCAGAATGGTTCGATTACAAGAGTAGGAACAATTCGATAACGTTTCCTACGACATTTAA
TTATCCGGGTTGGAGATTGAAAGTGCTTGCTGCATGTGTTAAAGTTCAAGTTCATGATTGTGTTACTCAG
TATCATAATACGGCGGAGCTTGAATGTCAAGTGTTCTTCAATGACATTCCAGTGTGGAGTTCTGAAGACG
AGGAAAAAATGTCTTGTAGAAGAGTCAAGATGGTTGAGCCTAGAAGCATCACCAAATGACTATACGTGGTT
TATTGTACTCAATCCTCATAGAGATTTCTACCTAGATTTGGATGATATGATGGAGGGATCACCAGAGACT
GATGTAAGTCAGCTATGTTTTGGAATTAATTCCATGGAAATGGACCATAATATTATACCAGATGATAATT
GGAATTCTATTGGGGGAAGTATTTGGAAGAACTTTACGGTGTTGTTTACGCCTCGTCCCGAGCTTTCAGA
CGCTAAAGTAAGTATAAAAAGTTGTGGTGTTCATGTCATCATGGAGGAATGATGTGGGAATTACATATTC
ATGGTGATGAGCTCATGTGAAAGTCGGAAAATATAATTTTTTTAACAAGGTCTGTATTGATAATTATATA
TTGTTTTTTGAGTGGTTGCCTAGTGTGTAATTGTAAAGAATATTTTAAAGAGCCATTTGTAACCATTAGA
TAAAACAATATGTGTGCAAAATTTTAGCCATACATCTATAGGAGCGATGTCCTATCACTTTTATCAAGCC
AAAAAATTAAAAGTGTCTAATAACACAAGTAGCGTATTTTTTCTCAATTCTCTCTTTCAAATTTTCCACC
ATCTGAAATTTCTTTCGAACAGTTTACTCTTTTTATGCATTAATATATATTTGACCAACTTGCATGTGGC
ATGTGTGTTTGTAATAATGTTAAATAAGGATAAGGGTAATTGTTTGGAAATACAAAACAATTTGAGGATT
TGGGGCCATCACTACTATTATAAGGAAGTCACCAAATATAGGATTGTCCCAACCTAAACACATTTTAACT
TAAAAGTTTCTATAAAATTGAGCAGCCAAAAGTAAGATAGAGCATAATGGTATAGGTAGTAACTTTAAAT
TCTTTTAAGATTTTTTTAAATACCGTACTTAATTCGTGTTCTTAGAATTTCTCTCATTTAAATGTGACAT
CACTTCATTCATGTCTTTTTTTTAAACTCATAGATTTACAGATACTTCTAAAAATTTTTGAAATACGTAA
TTGACATGTAACTTGGTGCATACTTGTTTTTTAATGTAGGATATGTGTGTTCCCTCCCTAAACTAAAAAG
CCTATTTAAAAACGGGCAACCACACCAAACCAAAACTAAAAAGAAAAAATAAATAATTTACATAATTGTA
ACAAAACCTAAAAATATTTACGATTCATGTAGCAAAATTAAAAGTTCATGAAGTCACCATTGTATTTTTC
ATAAATTCCAGATTTGTCTTTGAATATTCTTCTTCTTTGCTATTTATTCGCATCTTCTTGTGATTTCTTC
ATCTCCTCTTCCAGATTTTATTTTTTCTATATTCTTAAATGATTTATTCTTTTGTTTAAATCTCTTATTT
CATTTCCATCATTTTCGACAAGATTCTTTGAAGCTATTTTACGATTGTTTCAATATTCTTCTTCATTTTT
CTCATATTTGAAAATATCAAGATCGCTTAAATATCATTTTAAATTATTAACCAATTATTTAGCATGGTTA
ACAGGATCTTTTAAATTTGAAGCTTTTTTTCCATTGTTTAAAAAGAACTACAATATCGTGTATCATGATC
TAAACGATCTCTATTTACCATAGTCAATATCACAATGTGATTGCAACGCGAAACGGCCAGCGTCATCGTT
ATTTAATCTTAATAAATAAAGAGGTTTGGAGTCGCCACCAACCACGTTAAGCTATGATTGGTCATCCAAA
AGAACAATATTGGTCTACATAAAATATTCAGATACAAAACAAAGTTTTGATTTGTTTTTTTTTTTTTTTT

AAATGCCCCATGATTATCCATTCTCATCTAAGAAAAAGAATGAATTGGGAATTATGGGTGACAAAAGAAT
AATTAAAAAGAATGAAGTTTATTTTGGTTTCAAAGATGATTTCATTCACCTCTAAGATTATTCAAATATC
AAACTTTGATATTTTAATCTCAATCATAGGACATTTAATAAGAAATTTTATGTATCTAAAGAATTAATAA
ATTACAAATAAAACATTACTCAGTCTCATCTTAAAATATATAATTGTTGGTTTTTTAAAGATAATTTATT
AATTAATTTTTAAAAGGAAAAATTTTATGTATTTATGAAATTTAAATCTTCGAACACATAAAGAAATATT
AATCCTTCTCAACTAAAATTTTTATATTCATATAAAAATAGCAAAAAAAACAATTATTTTTTTGTTATGC
ATTTTGTACTTATGGAAATGGGAAATAAATAAATAATAATCATAACAATAATAAAAAATTTAACAGCATC
CTAAACTAAATATTTACTTTTACCCTATAGAAAAATAAAAGAAATCAAATGTTTTTTTTTTTTTCTTTTG
ACATCTTAAACTACAGATATAATTGAGAATCATAATTCAAGTTATATGAAATGTAGTTAAATATATAAAT
GATAACTTGAAATTTGAGATATTAATTCATTATGTTATTAATCAAATTGCACTCCATGAAGAAAAAGAAA
AGGAAAAAGAAAAATTAGCAATTTTTGGTGAAATATGACCTATTAAGTCTAATTTAAGAAATCAATTAAT
TGACATCCCAATTAATATCGAATCCAACAAACTCTCAATTAATATACCATACAATAAACCATAAATCTAA
TAAATTTAAAGGAATATTAAACTCAATTCAACAAAATTCGAATTCTAACAACCCAAATCTAATGGGAAAA
ATGACTGCGGGCAACAGTGACATTTTCTCTCTTTTCCTGAACTCTGAAGTTACTACCATTACTAGAAAAA
TAGTCTTTCTTGACGGTTGTATTTTTAATTTCTTGACGATTTTTGAAAAAAATGTCAATAAAGGGGTGAT
TTAAAAGAAAAACCATCAAGAATTTTTATGAATAGGCGGAGGGTAGTAAATTTTCAGCTTTCTTGACGGT
TTTGAATCATCAAGTAATATGCATTTTTTCTTGACGTTTTAAAACCGTCAAGTATTATTTATTAAATTCT
TGACATTTTTAAAACGTCAAGAATTTTTAAAAATTCGACATTTTTGACGTTTTTTAAACGTCAAATGATA
TATACTTATCCTTGACGTTTTAAAAGCGTCAAGTAATATCGATTAAATTTTTGACGTTTTAAAACGTCAA
GAATCTTTATGAAAAGACGGAGGGGATTCATTTTCAAATTTCTTGACATTTCTTGACGTTTTTAGAACGT
CAAGAATTACTAAATAATTAAAAAAATATATAAAACCTAATTTTTTAAAAAATAAACCTATTCCCCCTCC
CCCCCTAACTTTTTCCCTTCCCCTCCATACAACCCCAATTTCCCTCTTCCTTTCTCACAACCCACACAAC
GCCATATCTTCTTCTCCAAACTCACCCATCCCAAACACCGTCGAAACGCTGTCAAACGCCACCGAACCTA
CTACCATCCGAAGCTGAATGCCTGCTCGCCGAACGTCGCTATTGCTACCTGCTAAGTACCATCCGAACGT
ACACCTGCTTGTCGAATGCTGCTCCCTGCTCCCTTGCTTGCCAAATGCAGAACAACAAATGTCGCTCCCC
TGCTAAGTACCATTTGAATGCCGCCGCTCCCCTTTGTAGTTGGTTTTACCAAGCCAAAGCTCCATAGAAG
AAATCAAAAGACCAAAATGAAATTAAAGAAATAGAGAGAGAAATTAAAGATGGCTGGTGGTGGATTTGGA
GATGGTGGAGCTCCATTGAAGAGAGCTCATCTTTATGAGTATCGGATTACTTCATATTTTGTTACCGCTT
GCATTGTTGCTGCTCTTGGTGGATCATTGTTTCTATGATCTTGGAGTTTCTGGTGTATATTTTTCATCTT
CTTTTCTTTTTACAGAATTATTTTTTTATTCTAATTCTTGGATAGTTCTTTTCTGGGGTTGTTTGAAAAA
ATATACAAAGGGTCAGTCGAGTGTACCCGAGCATCTCCACTAAGTGGACACCCCCTTAGCACCCTCATCA
TTCCCACTTCATTTATAATCAATAAGGATCGAGTCAATACAAGAAGCAAGGAGAGAAAGGGCTAGAGAAA
AGCCCGATCAAACAAAAGCATTTATGTTTAAAGCTATAGTGCTAGCATTGTAATCTAAAAAAGTTTTGAT
CTACTACTCCAGAAGCCGGTCAGCGCCACAATGTCATCCCAAAGCAAATTTGAGTCTTTTTTGAAGCTTG
TGAAGATGCGATTATTTCTTTCTAATCAAATCTTCCAAAGAGTCACAGCTATCAAATTGAAAGTGATGGA
CCTTTTCCTGTTTGCCGTGTTAAAACCACAAAAATCCTTACATAAGGATCTAACTGATGTCGGTTGCTGG
TTCCAATTTAGAATATGCCTTGCTTTGTTCCAAATTGTCTGAGCATAAGGGCATTGAATAAAGAGATGGT
TTATATCTTCTGCATTAGATTTGCACATGGAGCACCATTGTGGACAGAGATTGTAATTGGGGAGCCTCTT
TTGTAGTTTTTCAGCCGTGTTGATACACTCGTGGATAAGAGTCCAAATAAAAAATTTGCATCTTTTTGGA
ATTTCAGACTTCCAGAGAGAATTGAAGGTGCTAGCCTGTATTGCAATGTTGTCGTCAATTTCAATCGAAT
TTAAAGCTTTTTTGACAGATGCAATACTGAACCCCCCATTTGAATTTAATTTCCAAAAAGATTTTGGGGC
TCCTTTGTTTGGGTGAGGGACTGCAAGGGGGGGCTTTTATATCATTCCACGTTTGCAACTCATAATCACGA
AGAGGGCGGTGAATATGTATGTCCCAATCTATAACATTGGAGTTCCACATATCTTTCACAGATCCGGTTT
TTAAATTTTAGAGCGCGTAAAGTCTAGGGATAGTATTAGCAAGGGGGCTTTGCTGTTCCACAAATCATGC
TAGAAAGATATGGACTCACCATTGTTAACATTCCAACCATAATGATTAGAGAACCAATCAGCACATTGAA
TGATGGAGTTCCAAGGGGCTTTTTTGCTACTGAACTTGCTTTTGGAGGGAAAATAGCCAGTACAACCTTG
ATCGTATTTTGCCATGATAAGGTTTTTCCAAAGAGAATTTCCATCATTGATAAAACACCAAAGCTTTTTG
CTCAACAAAGCAAAATTTGCAGTATTAACTTTGGTGATTTCCAGTCCTCCCATTTCTTTAGGTAGCGTGA
TTTTTGACCACCTCAGTAGGCTAGCGTTTTCTCCATCCCCTGTTCCTTTCCATAAAAAATTTCTCCAACT
TTTTGCAATATTTTTTGCGACTCCTTTTGGAGCCTTGAAAACAGACATCTGATATATGGGGAGGCTTTCC
CAGGAGAAATTGATTAGGGTGATTCTGCCTCCCTTTGATATATAAGAGAATTTCCAACTGCTAAATTTTT
TATGAATTTTTAGCTGGAAACTCAATGGAAATGGTTTCTTCTGGGTGGCTTCTGTGAAAAGAGCTATTTT
CTATGAATCAAACATCATTGTAGGAAATTCACATTCGTAAGCTTTCTAGAACTTATGAAATTAAATCCTC
TACTTCTTCAAAGTGAAAATTTTTCATTTGCACTTTGGTCCATCGTCAAAGAATCAACACTATGGATGCT
TTTTAGAGGAGAAACCCAAGCATCTGTTTATTGCCCCAATGGTGTATCTCATGCAAATCTTCTCTCGAAG
ACATTGATCATTTATTCATACACTGCACCAAGGCCCAATACCTATGGAAAAAGACCCTTTCAGAAATTGA
TGTTACTCTGTCTATGTCTAATACAAAAGATCTTTGTCTTATGCTTTGCGAGCTCGAAAGCAAAAGCATA
AAAAATGTCATAAGGTTCAATTCCATTGCAGCCACTCTCTGTACAATAAGGACGAACAGAAACAACCAAA
TCTTCAATGGTAGAATTTAAAGCTTTTGGATCAAGTGGGAAAACATATGCAACTTTATAGGCATGTGGTC
TACCAAAAGCAGCCTCCTCAAGAACTATAACCAAGTATCAATTTCTTTGAACTATAACCAAGCTCTTTGT

ACTGATACTACATTTCCTATTCGGGCTCATCTCCAGCCTATTGTAACTTCTCTTTGATAAATAAAATTGG
TGTTATGGTTGCTTGAGGCGGCCATAACATATAAGTATCTTTCTAAAAAAAACAACGCAAATCTAACAAA
TAATTAGTAACCACCAAATTTAATGAAATTAATCAGAATCACCTTAAATCGGGTAAAAAAAAAAAAAAAG
AAATGCAATTTATAAAAGAGGAAAAAAGAAATACCTTAGTTTGGGAGAAAAGTTTCTACTTCTTCCAACC
CTCCCAATTGAAAGCAAATTGAGCAAATGAGAATATTTTTTTACAGTGAAAAAGAAAATCTCAACATAGC
TCCTCCTTTAATTACTCTTGGGATGGTTGTCAGTGTGGAGAAAGTGAGCGAAGGGTGAAGAGCGAAAGAA
AGAAAGAAAAATTGTGGAGAGATATGGAGAAAGTAGTTGAGGGAAGCTGAGGGGAATCCTAAGATTAGGA
TAAGTTAGGTTTTTTCCTACTTTGTTTTCTTTGCTTATTATTAATTTAAAAAAAAAAAAGTAAAATTCAA
ATCCAATTTTCTTTTTATAAAAACAAAACTAAACAAAATAAAGTAAATAAATAAATAAAAGTAAACAAAA
ATAAATAAAAATAAAAAATGACAAAATATGTTATAGCATTGTGAACGGTGAGTATATGTGGCTAATAGTA
CTTGATCCTAAAATACATTTCCACCCATATTGGGATCATGTTCTTATGGACAGCTAGCTCATCAAAGATT
ATTGATCTAAATCAACCAAGTTTTGGGATTAATTCCTCACAGCGTATTTGGAAGAATATTAGTCTCGTTT
GAATTCCCTTCCTGGTCAAAAGACTTTGGAGTAAGCATAAAAATATGTGGTGTTCATGACTCATCATGGA
GGAATGAAGAATAGCTTTAAGAAATAGAAGTCAACTGATAGTTGAAAGGGAATTTTTGTTGCTACATTTG
AAGAACACAAGTTTTGGGATCTCGTTGAACTGTTAATTGCGAGTGGTTATATTCCCAACTATTTTTAATA
TATAAAGAATATTGAAGTGCTTATTTTTTGCTAAATGTTGTAAAGTTTTGTAGAAAGTACGTTTGAAGTG
TTGCCTTGTAAGATATATGAAGTTTACTATTATCATAAATGTTTGAAAGATTTATGTGTTTATATATATA
TATATATGGTTTGATAAAATGCCTTAAGATTCTTGAGAAATACTTCATGGTTTAGCTTAGTATATGATAT
ACATTACAAAATGAAAATCTAACAAAAAAATATTTTACGAAATAGAGTTGTATCACTCATTTTATTAATA
TGACAAATATGACAAAATAGCAAATAAAATTTATTCAGTTGCTAATTTTGTCATATGATCACATTTAATG
TTTTCTTTTCTATTTTCAAATTTAACCTTAAATCTGGAAAAAAAATTCTTTCTTCTTGCTTGTGTTCATT
GCCCCAAACCTCCCCCCTCATCTTCCAATTTTGTCCACCAACTCTAAGCTAAAGTGCCGCCGCCGTCTTC
CATTCAGTCGTTGTCACTTTCTTCTCGCTTCCTAGCCATCGACGCACTGCTCCTCTATGTTTCACGTCCA
ACCAGTCAGTCGTCGCGTAGTTTCCATTATATGATGATTGACGTCGCTCATACCAAGCGACATCCACCGG
TAATAATGGCATCACTCAGCTCTTGCGGTTTCTCTTGCCATGGATTTGATATGTTTGAGCTTTTTCAGTG
AAATCTCTAGCAAAATTAACACCGAGATCCTATATATATCCCAAAATCTTAGCCATATTTTTTTTGCAAA
ATTTAATCTTCTCTAAAATTTTAGTTTACATTCTAATTGAGTAGTATTTTTAATTTGTCAAAAATAATAT
ATAATGTTAGCAAAAAAAAAAAAAAAAAATCTTCTTTCTAATTGTCTATGACAACATATCAATTTCCTTT
CCTCCACAATGGAGTGCCATGGATGAATTTATTTGCAAAATGCCATTGGTTTTAGTTTAATTAAATGCCT
TTGATGTTAAAAACTCAATCATATAGAAATCACATGATAATAATAAAATATCAGATTGATTGAATTTTGG
TAGAAGAATATTGATGACCTTATTTATTGACTTAAAGTTTTATATATATGATTTGAGACGTGGAATTCAT
CCCAAATAAATATACAATAGCAATTAGTTAACACAATCAATATCAAATAGCAATTAGACGTGTCAAAAAA
TGAAACTAATCAGATGACAATTAGAAAACAATCAAACAATATAAAAATAACGATAAAAAAAATAAAAAGA
AGATAATCAAATGATTATATAATTAAAATTAAATTAAAAAAATAGAAGGTAATCAAAAGGTAATTAAAAA
AATAGTATAAGTTAATTAGTTAACAATCAGAAGATAATCAAAAGACTATCTCACAAATGCTAACTAAAAT
TGAGAAAAAACAAATAGAAGGTAATCATACGACAATAAGATGGTAATCAAATTAAATTATTATATTAGAA
GGCTAACTAAAATTGAAAGTTTTTAAAAGTAGAAGGTGACAATTAGAATGTAATCAAATAACTATCGTTT
CCAATATTTTTTTTTTGGGATAAACTTTTTATATGATCACTTTCCATGGCAACAATGGGATTATTTAAAA
AATGGATGGTAATCAAAAAATATTCAGTAATTCATGTAAAAAAAGATGTAAGGTCATTAGATAACAATCG
GCATCATGAATTTGCAATTAAATAACAATCAATATTAAATTAAATAATAATGAGATGGTAAATCAATATG
ATCATTAAAAAGATAACAACAAACATCAAATAGCAATTAGACAACAATCAGATGGTAATTGATATCATCA
ATAATTAGTGGTAATCAAATAAACAATCCAATAACAATTTAACGGTAATCAAAAAAAGTTCCACAATTTT
AACTAAGAGGGAATTTTGGACATCCGACATTTTCAATTGGGTCGAGCTTGTTAATTTGTCATTCCTGCAA
ATCTTAAAATGTTGGGTTTTGGACTCAATTTTTCATTTTATATAATTTACATGAGTGGAAGTGTCTTTTG
TAAAAATGATTTATTTATAACAATGAGATTTATGATACTTTTATAAAACGTACATGAGATTTATGATTTT
CCATGTGTTACGTGAATTTGTTTCCATAATACACTGCACCCAAATTGTTAGGGATCGTCGGAGCCAAGGC
CAAATCCAGAATCTCCAAATCTTTGTTTGTGGTACGGAGAGTATTGGTCTTTGGGACTCATTGTAGCACA
CATATAGCCTTTGAAAGGTGTGTGTTAGGGTGCTTTACCCCTTTGAGGGCAGCTATGTGTATGCGTATGA
CAAGTGATATTAATGCGCTATTATGTATTTGCGATATTGGATAGAAAATGAATGGTTCTAACAGTAAACA
TAATGTCAAGCATGTTGTCACAACAATATATCCCACCCGAGGGTTGTTTACAAAAGCAAGCAAAAAAAGT
AATGAGATAAAGATTGATATTTCAGTTTGGTGCAAAATCATCTACATCCAGAGAATAGTGTACTGCCGAT
TAACAAATATTTCATTAATTGTGTTTTTCCTATTTTGTCAATTTTAGGATGCTTAGTTGTTGACAAATGG
ATTTGGAGTTGTCTCTGATGAGGGTTCCACCCATGTTGGATTGGAATTTGTCCCACATAGTTTTTGCAGC
TTAGCAGTTAACGAAAAGATGATCGATACTTTCACTATCAGCTTTGCAAAGAACGCACCTGTTTGGATTT
AAGCAAGTGTTTGGTAGCTTGTTTTGTGAGACATCCATCCTGTTAACTTTCTGATGAAGGATGGACCACA
CAAAGAACTTTGCATTTTTTTTTTAATATTTTATTTCTAATGATTCTCAAAGCCACGAGTTTCCATTTGT
TGCAATGTCACCTGTTCATTTCCCTGTATGGCATTTTGTACCGAGGCCACTGTGCGTTGGAAGCGTTTGA
TGAACACCCCAAATAAATAATCACCAAATAGAATATTTGATAGAAAACAAAAACCTTTTTGGAAGACTTG
TTGTGTGTATTCAACTCACACTTGGATGATTTAGATTACAACATTTCAATCCTATTTATAGAATTGTCAT
AAGAATAACTCATATCACAATTAAATACAACAAATTAAATCCTACATTTAACTAAGGTGAAACTCTTACT

AAATTAACTCTAACATTTTTAACTCACATCCTTCTCTAACTTACATAAATTAACTCACATATAACTTATT
TATTTAAAACATGTTTAATTTTCAACATTGTGAATGAAACATTTGTACTAAGCTGCCAGATTAANNNNNN
NNNNNNNNNNNNNNNNNNNNNNNNNNNNNNNNNNNNNNNNNNNNNNNNNNNNNNNNNNNNNNNNNNNNNN
NNNNNNNNNNNNNNNNNNNNNNNNNNNNNNNNNNNNNNNNNNNNNNNNNNNNNNNNNNNNNNNNNNNNNN
NNNNNNNNNNNNNNNNNNNNNNNNNNNNNNNNNNNNNNNNNNNNNNNNNNNNNNNNNNNNNNNNNNNNNN
NNNNNNNNNNNNNNNNNNNNNNNNNNNNNNNNNNNNNNNNNNNNNNNNNNNNNNNNNNNNNNNNNNNNNN
NNNNNNNNNNNNNNNNNNNNNNNNNNNNNNNNNNNNNNNNNNNNNNNNNNNNNNNNNNNNNNNNNNNNNN
NNNNNNNNNNNNNNNNNNNNNNNNNNNNNNNNNNNNNNNNNNNNNNNNNNNNNNNNNNNNNNNNNNNNNN
NNNNNNNNNNNNNNNNNNNNNNNNNNNNNNNNNNNNNNNNNNNNNNNNNNNNNNNNNNNNNNNNNNNNNN
NNNNNNNNNNNNNNNNNNNNNNNNNNNNNNNNNNNNNNNNNNNNNNNNNNNNNNNNNNNNNNNNNNNNNN
NNNNNNNNNNNNNNNNNNNNNNNNNNNNNNNNNNNNNNNNNNNNNNNNNNNNNNNNNNNNNNNNNNNNNN
NNNNNNNNNNNNNNNNNNNNNNNNNNNNNNNNNNNNNNNNNNNNNNNNNNNNNNNNNNNNNNNNNNNNNN
NNNNNNNNNNNNNNNNNNNNNNNNNNNNNNNNNNNNNNNNNNNNNNNNNNNNNNNNNNNNNNNNNNNNNN
NNNNNNNNNNNNNNNNNNNNNNNNNNNNNNNNNNNNNNNNNNNNNNNNNNNNNNNNNNNNNNNNNNNNNN
NNNNNNNNNNNNNNNNNNNNNNNNNNNNNNNNNNNNNNNNNNNNNNNNNNNNNNNNNNNNNNNNNNNNNN
NNNNNNNNNNNNNNNNNNNNNNNNNNNNNNNNNNNNNNNNNNNNNNNNNNNNNNNNNNNNNNNNNNNNNN
NNNNNNNNNNNNNNNNNNNNNNNNNNNNNNNNNNNNNNNNNNNNNNNNNNNNNNNNNNNNNNNNNNNNNN
NNNNNNNNNNNNNNNNNNNNNNNNNNNNNNNNNNNNNNNNNNNNNNNNNNNNNNNNNNNNNNNNNNNNNN
NNNNNNNNNNNNNNNNNNNNNNNNNNNNNNNNNNNNNNNNNNNNNNNNNNNNNNNNNNNNNNNNNNNNNN
NNNNNNNNNNNNNNNNNNNNNNNNNNNNNNNNNNNNNNNNNNNNNNNNNNNNNNNNNNNNNNNNNNNNNN
NNNNNNNNNNNNNNNNNNNNNNNNNNNNNNNNNNNNNNNNNNNNNNNNNNNNNNNNNNNNNNNNNNNNNN
NNNNNNNNNNNNNNNNNNNNNNNNNNNNNNNNNNNNNNNNNNNNNNNNNNNNNNNNNNNNNNNNNNNNNN
NNNNNNNNNNNNNNNNNNNNNNNNNNNNNNNNNNNNNNNNNNNNNNNNNNNNNNNNNNNNNNNNNNNNNN
NNNNNNNNNNNNNNNNNNNNNNNNNNNNNNNNNNNNNNNNNNNNNNNNNNNNNNNNNNNNNNNNNNNNNN
NNNNNNNNNNNNNNNNNNNNNNNNNNNNNNNNNNNNNNNNNNNNNNNNNNNNNNNNNNNNNNNNNNNNNN
NNNNNNNNNNNNNNNNNNNNNNNNNNNNNNNNNNNNNNNNNNNNNNNNNNNNNNNNNNNNNNNNNNNNNN
NNNNNNNNNNNNNNNNNNNNNNNNNNNNNNNNNNNNNNNNNNNNNNNNNNNNNNNNNNNNNNNNNNNNNN
NNNNNNNNNNNNNNNNNNNNNNNNNNNNNNNNNNNNNNNNNNNNNNNNNNNNNNNNNNNNNNNNNNNNNN
NNNNNNNNNNNNNNNNNNNNNNNNNNNNNNNNNNNNNNNNNNNNNNNNNNNNNNNNNNNNNNNNNNNNNN
NNNNNNNNNNNNNNNNNNNNNNNNNNNNNNNNNNNNNNNNNNNNNNNNNNNNNNNNNNNNNNNNNNNNN
NNNNNNNNNNNNNNNNNNNNNNNNNNNNNNNNNNNNNNNNNNNNNNNNNNNNNNNNNNNNNNNNNNNNNN
NNNNNNNNNNNNNNNNNNNNNNNNNNNNNNNNNNNNNNNNNNNNNNNNNNNNNNNNNNNNNNNNNNNNNN
NNNNNNNNNNNNNNNNNNNNNNNNNNNNNNNNNNNNNNNNNNNNNNNNNNNNNNNNNNNNNNNNNNNNNN
NNNNNNNNNNNNNNNNNNNNNNNNNNNNNNNNNNNNNNNNNNNNNNNNNNNNNNNNNNNNNNNNNNNNNN
NNNNNNNNNNNNNNNNNNNNNNNNNNNNNNNNNNNNNNNNNNNNNNNNNNNNNNNNNNNNNNNNNNNNNN
NNNNNNNNNNNNNNNNNNNNNNNNNNNNNNNNNNNNNNNNNNNNNNNNNNNNNNNNNNNNNNNNNNNNNN
NNNNNNNNNNNNNNNNNNNNNNNNNNNNNNNNNNNNNNNNNNNNNNNNNNNNNNNNNNNNNNNNNNNNNN
NNNNNNNNNNNNNNNNNNNNNNNNNNNNNNNNNNNNNNNNNNNNNNNNNNNNNNNNNNNNNNNNNNNNNN
NNNNNNNNNNNNNNNNNNNNNNNNNNNNNNNNNNNNNNNNNNNNNNNNNNNNNNNNNNNNNNNNNNNNNN
NNNNNNNNNNNNNNNNNNNNNNNNNNNNNNNNNNNNNNNNNNNNNNNNNNNNNNNNNNNNNNNNNNNNNN
NNNNNNNNNNNNNNNNNNNNNNNNNNNNNNNNNNNNNNNNNNNNNNNNNNNNNNNNNNNNNNNNNNNNNN
NNNNNNNNNNNNNNNNNNNNNNNNNNNNNNNNNNNNNNNNNNNNNNNNNNNNNNNNNNNNNNNNNNNNNN
NNNNNNNNNNNNNNNNNNNNNNNNNNNNNNNNNNNNNNNNNNNNNNNNNNNNNNNNNNNNNNNNNNNNNN
NNNNNNNNNNNNNNNNNNNNNNNNNNNNNNNNNNNNNNNNNNNNNNNNNNNNNNNNNNNNNNNNNNNNNN
NNNNNNNNNNNNNNNNNNNNNNNNNNNNNNNNNNNNNNNNNNNNNNNNNNNNNNNNNNNNNNNNNNNNNN
NNNNNNNNNNNNNNNNNNNNNNNNNNNNNNNNNNNNNNNNNNNNNNNNNNNNNNNNNNNNNNNNNNNNNN
NNNNNNNNNNNNNNNNNNNNNNNNNNNNNNNNNNNNNNNNNNNNNNNNNNNNNNNNNNNNNNNNNNNNNN
NNNNNNNNNNNNNNNNNNNNNNNNNNNNNNNNNNNNNNNNNNNNNNNNNNNNNNNNNNNNNNNNNNNNNN
NNNNNNNNNNNNNNNNNNNNNNNNNNNNNNNNNNNNNNNNNNNNNNNNNNNNNNNNNNNNNNNNNNNNNN
NNNNNNNNNNNNNNNNNNNNNNNNNNNNNNNNNNNNNNNNNNNNNNNNNNNNNNNNNNNNNNNNNNNNNN
NNNNNNNNNNNNNNNNNNNNNNNNNNNNNNNNNNNNNNNNNNNNNNNNNNNNNNNNNNNNNNNNNNNNNN
NNNNNNNNNNNNNNNNNNNNNNNNNNNNNNNNNNNNNNNNNNNNNNNNNNNNNNNNNNNNNNNNNNNNNN
NNNNNNNNNNNNNNNNNNNNNNNNNNNNNNNNNNNNNNNNNNNNNNNNNNNNNNNNNNNNNNNNNNNNNN
NNNNNNNNNNNNNNNNNNNNNNNNNNNNNNNNNNNNNNNNNNNNNNNNNNNNNNNNNNNNNNNNNNNNNN
NNNNNNNNNNNNNNNNNNNNNNNNNNNNNNNNNNNNNNNNNNNNNNNNNNNNNNNNNNNNNNNNNNNNNN
NNNNNNNNNNNNNNNNNNNNNNNNNNNNNNNNNNNNNNNNNNNNNNNNNNNNNNNNNNNNNNNNNNNNNN
NNNNNNNNNNNNNNNNNNNNNNNNNNNNNNNNNNNNNNNNNNNNNNNNNNNNNNNNNNNNNNNNNNNNNN
NNNNNNNNNNNNNNNNNNNNNNNNNNNNNNNNNNNNNNNNNNNNNNNNNNNNNNNNNNNNNNNNNNNNNN
NNNNNNNNNNNNNNNNNNNNNNNNNNNNNNNNNNNNNNNNNNNNNNNNNNNNNNNNNNNNNNNNNNNNNN
NNNNNNNNNNNNNNNNNNNNNNNNNNNNNNNNNNNNNNNNNNNNNNNNNNNNNNNNNNNNNNNNNNNNNN

NNNNNNNNNNNNNNNNNNNNNNNNNNNNNNNNNNNNNNNNNNNNNNNNNNNNNNNNNNNNNNNNNNNNNN
NNNNNNNNNNNNNNNNNNNNNNNNNNNNNNNNNNNNNNNNNNNNNNNNNNNNNNNNNNNNNNNNNNNNNN
NNNNNNNNNNNNNNNNNNNNNNNNNNNNNNNNNNNNNNNNNNNNNNNNNNNNNNNNNNNNNNNNNNNNNN
NNNNNNNNNNNNNNNNNNNNNNNNNNNNNNNNNNNNNNNNNNNNNNNNNNNNNNNNNNNNNNNNNNNNNN
NNNNNNNNNNNNNNNNNNNNNNNNNNNNNNNNNNNNNNNNNNNNNNNNNNNNNNNNNNNNNNNNNNNNNN
NNNNNNNNNNNNNNNNNNNNNNNNNNNNNNNNNNNNNNNNNNNNNNNNNNNNNNNNNNNNNNNNNNNNNN
NNNNNNNNNNNNNNNNNNNNNNNNNNNNNNNNNNNNNNNNNNNNNNNNNNNNNNNNNNNNNNNNNNNNNN
NNNNNNNNNNNNNNNNNNNNNNNNNNNNNNNNNNNNNNNNNNNNNNNNNNNNNNNNNNNNNNNNNNNNNN
NNNNNNNNNNNNNNNNNNNNNNNNNNNNNNNNNNNNNNNNNNNNNNNNNNNNNNNNNNNNNNNNNNNNNN
NNNNNNNNNNNNNNNNNNNNNNNNNNNNNNNNNNNNNNNNNNNNNNNNNNNNNNNNNNNNNNNNNNNNNN
NNNNNNNNNNNNNNNNNNNNNNTTTTTTTTTTTTTTTTTTTTTTTTTTTTTTTTTTTTTTTCAATTTTGTAT
CTCCACAACCCATTTGTTTGGGAGAGCAAAGTTTGTATCTTTCATCCTTGGACTCCCTCCCTAAGAGTTA
TATCACCTCACATTAGTATCTAAATTGTTGTATCTAATTATCTTCTATTTTTTTTCTTCTAATTTTAGTT
AGCCCTAAGAGATAGTCATTAGATTAATTTTTGATTGTTATCTAATTAACTTTTATTCTTTGACACATGT
CAAATTGCTATTTGATATTGATTGGTATATAATTGGTTGCATGACAAACACATGTCAAATGCTTAAATTT
GAGGAAAATTACATCCGAGGAAAAGTAGATAAAAGCAACCTATAGCACTTCCTTTTTCTACATTGCAAAT
ATGACAAATAATTAGATATATGAACGATATTAAATTTAATATGAGGAAGTTAATTTTTTTAATATAGGAA
TTGATTAAATAATATAAATTAGAATTATAAAAAATGACAAATTTTACAAAATATCTACAAAATATAACAA
AATTTCAAATTCTATCAATATGCTTCTATCAATCCTAGAAATATAAAGTGACAGAAGTTTATCAGTGTCT
ATAATTGATAGAATCCAAAATTGTTATATGTTGTAAATATTTTAGTTTATTTTGCTATTTTAAAAAATGT
TTTTAATATTAATAATTTTAACGAAGAAAAATATAATATAAGAAATATTTTCTCAAAATTTATAATAAAA
TCTAAAATGTTAATAAATAATAATGCTTTTTTTATACAAACAAAAAAATCAGCAATAAACTAAGTATAAG
AACTAAATTTGATAAATATCGTAAGTTGGCCTACTAATAGTTGGGAACATAAAGGAAAAAATATCCAAAA
GAACTAATAAGGAGTCATGAGTTCAATACATGATAACCACCCACTTTATTCTTTCTATAAACAACCACCG
ATAAAATAATATTTTATTCTAAAGTCAATAAGAAAAATGTAGATTACAAAAATAGATGGTAATTAGAAAA
TATTTACTAATTGACAAAAAAAAAATGTAAGGTCACTAGATAAGTACAATCGGTATCATTTGCAATTAAA
TAACAATCAATATTAAATTAAATAACGATGAGATGGTAAATCAATATCATTAACAAAAATATAACAACAA
ACATCAAATAACAATCAGACAGTAATCAGACAACAATCAAATAAAAATCGATATCATTAATAATTAATGG
TAATTAATCAGATGACAATTCGATAACAATCAGATGATAATCAAATACAGTTCCACACTTTTAACTAAGA
GGGTATTTTGGTCATGCAACATTTTCATAGTTGAGCCAAGCTTGTTTTTTTTTGAAAAAGGATACGGTGG
GTCAGCAGGTGCACCCGGGCATCTCCACTAGGTGGACACCCCCTTAGCACCATCATCATTCCCGCTTCAT
TAAGATATAACAAAAGCAATACACGGAAGCAGGGAGGCCCAAAAAAAGGGCTTGAGAACAGCCCCGAAAG
GACAAAGTGCATACAGAGAGAGAGAAAACTAAGGAAGTGCAAACTAACTAAAAGCATTAAGTTAATTTGT
CATTCCTGTAAATTTTAAAATGTTAGGTCATGTACTCAATTTTCATTTTATATTTTACGATGATTGCAAG
TGTCCCTTGTAAAATGATTTATTAACAATGAGATTTATGATATTTTTATAAAACTTAGGTGAGATCTATG
ATTTTCCATGTGTTACATGAATTTGTTCCATAGTACACACTCCACCCTAATTGTCAGGGATCGTCGGGGC
CAAGGCCAAATCTTTGAGTCTATGGTAGGGAGAGTATGTGTCTTTGGGATTCATTGTAGCACACATGTAG
GCTTTGAATGGTGTGTGTTGGGGTGCTTAGCCATTTTAGGGTAGTTTTATATGTATGCGTACATGACAAG
TGATATTTATGCACTATGCGTATTGAGATTATTGAAACTAAAAGAAAAAAAAACGAGTGGATCTAACAAC
GTTGTCGCAACAATCTATTCGACAATAGGGTTGTTTACAAAAGCAAGCATAAAAAGTAATGACATAAAGA
TTGATATTTTAGTTTGGTGTAAAATCACTTATGTATAGGGAGTAGAGTGAAGGTGGAATGAAACTTTAGT
ATATAAAGAGAGTTTAGCAATTAAAACGAAAGTTACTTATATATCTGACAGACTCCCCTCTTCAATAACT
TCGATATAGATTGCTCTTCAAGTACACATTTCAACTTAGGCTCCCCTTAAGTCGTTTGAGTTCCCCTCAC
TTCTACAATATATACGTAAGTTTTGTATGAGATCCCCAATACTATATGTTTAGGTTCTTCCGAAGAGTTA
TATCCTCTTACATTATATCCAATTGTTATTTAATTATCTTCTACTTTTTTTTTTCAATTTTAGTTAGCCT
TGCGAGATAGTCATTGGATTAGCTTCTGATTGTCCTCTAATCAACTACTATTCATTGGCACATTTAATTG
CTATTTGATATTGATTGTTATATAAACAATTGCTAGGGGCAAGTGCATGACAAACATATTTGAATGAGGC
TCATTGCCAAAAGGCTTAATTTTTGCAAAATTGGTCGTCTAACCTAATTTAAAAGAGGAATTGAAACTAA
AAAGTCGATAATATCTTATTGATTCACGAATATGCATGGACATGATATTTGATTGTTAGTGATTGTTAAT
CTTATTGTTATTTGATTATCTTCTATTTTTCTCAATATAGTTAGGCTTCTAATAGTCATTTGATTATTCT
ATTCGCCATCCGATTACTTTTTTTTTATATAGTCATTTTTGATATTGTTTGATTGCCTTCTAACAATTAT
GATTAAATTAGGTTATATTCTTTGACGCGTCCTAGTTGCTATTTGATAATAATTGTTACCTAATTTCTAT
CGTATATTTATTTATAATCTCATAGTTGTTTGATCGCTATTTGATAAAAATTGTTCTCACATTCTCAATA
ATGCCAATTTTTATCTAATTACTATCTAATTATCATTCTTAATGACAATGAATGTTACTTGATTTGAATT
GTCATCTCATCTAACAACAAAATGAAATAATGAAATTTTAATTATTTGTAGGATGAAATTACATTCCTTG
CTATCGTGGCCATAACATATGTTTTTTAGGGCTTGAAGATACAACAGCAGCGAAGTTTCCTACGTTGAAG
TCTGAAATAAAATAAAACAAAATAAGCAAAATAAAATATAAGAGAGGTAAAATTGGAATAGATTAAAAAT
TAAAATTAGAAATGGAATACTTCGCTATTTTTACATAATCAAAAAGTTGAAAGTCATTCAAGCAACCAAA
TTCTTAATTTCACCTACTAAATGTTCTACTCTAATACATAATTCTAAGTCTACAAATTTGTTTAATCAAA
TAATATATGAGTTGGATCTTTGAAAGTCAAATGAAGAAAGTCTCCCCTTTTAACATTTCGTGATCCATCT

CATATTAATATAATCTATGCATTTTCTTGTCATCATTCAACAACTTCATTATCAATTGGAAACTTTTCAT
GGATGATCGCAAATGGTATTTCAAAAAGTCAAAAGAAAGCACATGGGAGTTTAAATTTCCAATGGAATAT
ATCACAAACGCTAAAGCCTGCCGACTGATCAGGAAAAAGAAAAATAAATATAACTCAATTATCTTCTGCC
TTTTCATTACCTCATTGTTCTTTGATATAATTTCTTTCATTTTCTCCTTATGGATCGTTCAAGTGGATCA
TTTTCTTCGCATTTTAGATGGAGCTTCGATGTATTCTTAAGTTTTCGGGGGGAAGATACTCGTTTCAACT
TCACGAGTCATCTTTATACGGCTTTACGTCAAAGAGGAATCAATGTTTTCATAGATAACAAGCTTACAAG
AGGTGATAAATTTCCTCCATCTCTTTTGAAAGCTATCGAAGAATCGAAGATCTCGATTGTTATAATCTCT
GAAAATTATGCATCTTCGAGCTGGTGTTTGAATGAACTGGTGCACATCAATATGTGTAACGAATTGAGAG
GACAAGTTGTTTTACCAGCCTGGAGAGAGGCTTTGACTGCTGTTTCTTATATGTCTGGATGGGTGGTTCT
TCAAAACGAGTATTTTTTTTTCTTGTCCCTTCTACTCTTTTTTCCCCTTCAAATTCTATTTTTACTATTT
TTTTGTAAGGAGATTCATGCTTTGGTCTAGAGGAAAAAGGAAATTAGAAAGCTTGATATTTACATCTGAT
TTGATAACAGTTTCAGATTGTATGCTTTTCTGCTTTATAAATTTTTATGTGGTTGTTTTGTCATCCCAGT
GTTTCAATTTTGGTGAAAACAATTTCCCCCTTATCAATACTTGGTTTGATTTTCAAGACAATAAAAGGAT
AACAAATCATAGAAATTTAGGATCAGCGCTTAATTTTCAAAAACTAAAAATAAAAAATCAAATGTGGTTA
TCAAAACTAGCATCGCGATTTTCGTTTCAAACCGTAATAATTCACTTTATATTATTTGTTATCTTTATTA
AATTACTTTGAGTTTATTACGTTGCTTTAATTTCTTGGCTACCCGATGCCTGTTAGGAATTATTGTGAGG
AAAAAATTGCTTGCTACATGACACTTTTTAGTGAATGCTTTTTTCTTATATGGCACTGACGAGGCTAATT
TGATACAAAAAATTGTTCAACAAGTTTGGAAGAAATTAACTTGTTCAACAATGCAGTTGCCTGTAACTAA
ATATCCAGTTGGAATTGACAGACAAGTTGAGAATTTGCTCTCTCATGTTATGATTGATGGAACTAGAATG
GTTGGAGTGTATGGAATTGGAGGTATGGGCAAGACAACTTTAGCCAAAACATTATACAATCGGATTGCTG
ATGACTTTGAAGGCTATTGCTTTTTAGCAAATGTTAGAGAAGCTTCAAAGCAACACGAGGGCCTTGTTAA
ACTCCAAGAGAAACTACTTTATGAGATTTTAATGGATGACTTTATTAGAGTTGGTGATCTTTATAGAGGA
ATCAACATCATAAGGAATCGACTATGCTCCAAAAAGATTCTCTCAATTTTTGATAATATAGTTACAAGTG
TTTAATGGCTGGCATTAGCCAGCGGATACGATTGGTTTGGATATGGACTCTCTCCCCTGCCCNNNNNNNN
NNNNNNNNNNNNNNNNNNNNNNNNNNNNNNNNNNNNNNNNNNNNNNNNNNNNNNNNNNNNNNNNNNNNNN
NNNNNNNNNNNNNNNNNNNNNNNNNNNNNNNNNNNNNNNNNNNNNNNNNNNNNNNNNNNNNNNNNNNNNN
NNNNNNNNNNNNNNNNNNNNNNNNNNNNNNNNNNNNNNNNNNNNNNNNNNNNNNNNNNNNNNNNNNNNNN
NNNNNNNNNNNNNNNNNNNNNNNNNNNNNNNNNNNNNNNNNNNNNNNNNNNNNNNNNNNNNNNNNNNNNN
NNNNNNNNNNNNNNNNNNNNNNNNNNNNNNNNNNNNNNNNNNNNNNNNNNNNNNNNNNNNNNNNNNNNNN
NNNNNNNNNNNNNNNNNNNNNNNNNNNNNNNNNNNNNNNNNNNNNNNNNNNNNNNNNNNNNNNATTGTTAA
TTTTAAATCCTTGAGATTTCTTGAAACATTTGATTGTAAGTTGCTAGAAAAAAATTCCAAAAATTCCAGAA
GGCTTAATTTCTTTGGGTGCTGAAGGGTGCATATCATTGGCCAAATTTCCTAACAATTTAGCTGATTTCA
TATCTTCTGATTCGGTGCATATCTCATCTCTTTCTCTTGTCTTGATCTCTCTTTTGTAATTTTTATTCAT
CAGTAGTTAAGTTGATGTGTGGTTTTGAAATTAATAATATTATTACTCTCCATTCACGTTGGGCAGGAAC
ATGTAGATGGACAGTTCAAACAACTCATATTAATGAATTGTGATATTCCAGATTGGTTCAGTTACAAGAG
TAGAAACAATCCAATAACGATTTTTATGCCATCCAATGATCCATGTTGGGAATTGAAGGTTTTTGCTCCT
TGTGTCAAATTTCAAGTTAATCATGTTGACAGGAATCGTTATATGGATCTTGAATGTAAAATGTTCATAA
ACGACATTCAAGTATGGAGTTCTGAAGAAGTACCCTTTCTTGAAGAATCGAGAAGGATATTGATAGAAGC
ATCCCCATACGAGTACATGTGGTTATTAGTACTATATCCTCATATAGATTTCCCACTAAATTCGGATGAT
ATTATCAAGAGATCACAGGAGATTAATCTACATCAGCCAAGTTTTGGGATCAGTAATTCCATGGGAAGGG
ACAATAATAATTGTAATGTAGATGACGATCGGAGGAATCATTTTGGGGAGAGTACTTGGAGTAAATTTAC
GGTGTCGTTTGGTGTTAATTCCAAATATAAAGACTCCGAAGTAAGTATAAAAAGGTGTGGCGTTCATGTC
ATCATGGAGGAATGTCGTGACTGATGTAGCCTTTTTACTATCACGTGTAAAGGACCAATCAAATGAAATT
TATACTATAATGCTCAATACTCTACCTATAGTACTACTACATAGTACAAGTGACAAAATCGGGTCGATCC
GTAAGAAGCACGAAGATTTGTTTGTCAAAGTTAATTCTTTAGTTAATGTGATAAATAGGGGGGTTTGGTG
TGGTTTGGGATTCAAGTGAATAAAGTGCAAGAAAAAATGAAGATAGATTTGCAATTAAAACATTCTAGTTG
TCGCCACGTGCCCGAGACGATGGGGAGCGACCGATGTCGTCAAAATGGTTAGTTTTAAAATAAAATAGAA
GTCACCGCCAATCTTTTTTACAATTTGATTGGATACCGAAATATAGTAAACACTTTTTAAATAAAATCAT
TAAAAAATGGGGGGCGAAAAATAAAGTTGGGGTCATGAATCCTTTTTGTATGAGAAATGGTTTAGCATCT
CCCAACCCCTTATTAGAAAACGAGGGCCAAATTTCAAACCTTAATTGAAAAAATTTTTTAATTTATTTTA
AAAACAATGTCTTATTTTACCCCTCATTACTCCAAATATTTTGAAGTAATGATCTCATAAAGTGAACATT
TCATTAATTAGATTATATTAAAAAAAAAAAATTCTCACCTCAACAAAGTGAGAAACTTGTAAAATTGCTC
AAAATTTATTATAGGAATAGTCTTCTAATAAAGAGATTTTTTAAAAACTTTTTCTTTCTTTTTTTTTTTT
TTTTTTGAGGAAGATACTTTTATGCTATGGCTGACTTAGGCAACCATAACATCATTTTATTTATTAAAAC
ATTAATTAAAATCATTTTACATGGGATTAAATCTTGGACTCCCAAAGATGTGATTCCTCACCTCAAGAAT
TTTAGAAAAGCGGACTCCCAAATTTCCTAGATTCCGTCGTAGGTATTTATTTAGGGAAAACTGTATAAGT
TATTATAAAAGATTTATTAGGAAACCTAGTAAATTATTGATTAAATTTTGAATGTTTTAAAAACATAAAA
AATTATAACTTTAGAAGAAAAAAAATTCTAAATGATTTAAAAAAAAAAGTTTTGCAAAAGATTTTATAAC
TTCAGAATTTTAAATTAGAAGGATTTTAAAACATGAGAATTTTAATTAGAAAGAGATGAAAATTTTAATT
AAGAAATAAAATGAGGCTTAATGGGAAAATTAGATAAGTAGAAACATGAGTAAAGATACATCATAATAAA

TTATGCAAACATAAAATAACATATTGAGATTTCAATAAAAACATATATTGGAGATCGATCTCGAACTTTC
AAAGAATCGATTTCCTCACCTCAAGAGTTCTAAGAAATCGGACTTCCAAATTTTTTAGATTCTGTGCTCT
GATGTTTTTAAAGAAAAATAATTTAAAATGTTAACAAATTTAAACAATTATTAGCAATAACATATTAAAT
TGGCCACAAAAGAAGGGAAAAAGTAAAATGTTAAATAACATATAAAAGATGAGAACATATTTAAGAAGCA
ATAATCAAAATGGAAAAAAATATTAAAGAATAAATAATCAAGATGACAAAATACTAAAGAAGCAATAATCA
ATGCTATTTCAAGAAAAAAAAATAATTAAAAAAATAAAAATGCTAATTAAAAAAAATAAAGATACAATATA
TAGAAAATAAAGGTTAAGAATAAATTAAGAAATGAAAGACATAATAATAAATAAAAAAAAGATAACAACA
GGAAGCTGGTCCTCAACACGAGGACTTCCCAAGTGGTCATCCAACTTAGTACTACTTTGGCCCAAGCATG
CTTAACTGCAGAGTTCTAATGGGATCCGATGCATTAGAGTTGGTATGATCGCAACCTCAAAAAGCAAATT
TTTCAAACACTACAACGGTCACAATCTCCTTAAACATCAATGCTTTTTGCAGTTTACCTATGTAACTGGA
TCAATCCTTTGTAACTTCTTTCTGACAATAAATTTGTGACAGGGTGGCTTATCTCTAGCTCCCTTCACTA
GTATATCTTTTTTTAAAAAAAAAAAAAGTATGAAGAAATATGTAATAAATAAAAATATATATATAAAAGAG
AAGCTCATAAGACAATAAATAAATAACTAGACAGCTGTCCAAGTTCATGAAGCTCATGAATCAATGAGGA
ATTGAAATAGAAAATTCAAAAAATTCAAGTATATCGTCATTGATTCATGAACTTAGACAGCTGTGTGATT
GTTATCTTATTATCATCCGATTACCTTCTATTTATTTGTAAGCTGATAGCTGTTTGATTGTTATTTGATA
AAGATAGTTCTCTCATTCTCAACGATAGCAATTGTTATCCAATTATGGTCTACTGTTAATTAAGAAAAAA
TGTTATTTAATATATTAATTGATCGTCATCTCATCTAATAATATATGAGTTGGATCATTAGAAGTCAACT
AAAAAGAGTCTCCCGTTTTAACGACTCGTGATCCATCGCGCATTCATATAATATATGCATTTTCTTGTCT
TAAATTGACAACTTGAATAATATCAATTGGATAATTTTCATGGATGATCATGATGGTATTTCAGAAAGTC
AAAAGAAAGCGTATAGGAGTTTTAAATTCCAATGGAATATAAAACAAACGCTAAAGCCTGCCTACTGAGG
AAAAAGAAAAAAAAAAAGTGCTTCCAATTTCACGCACCTGCTTCAGTGTTCTTTGATATAATTTCTTTCAT
TTTTCTCGTTATGGATCGAGCAAGTGGATCATTTTCCTCACATAGATGGAGGTTTGATGTATTCTTAAGT
TTTCGAGGGGAAGATACTCGTTTCAACTTCACAAGTCATCTTTATACGGCTTTACGTCAAAGAGGAATCA
ATGTTTTCATAGACGATAGCGAGCTCACGAGAGGTGAAAATTTCCCCTCATCTCTTTTGAGAGCTATTGA
AGAATCAAAGATCTCGGTTGTTATAATATCTGAAAATTATGCAACTTCGAGTTGGTGTTTGAATGAACTG
GTGTACCTTATTATGTGTAAGAAATTGAGAGGACAAGTTGTTTTACCGATTTTTTACAAAGTGAATCCAT
CTCAAGTACGGACACAAAATGGAGCATTTGGAGAAGCATTTGCTAAACTTGAAGTTAGATTCTTTGACAA
GATGCAAGCATGGAGAGAGGCTTTGACTACTGTTTCCTTTATGTCTGGATGGGTGCTTCTTCAAAACGAG
TATTTCTTTCTTCGTCCTTCTACTCTTTTTTCCCTTCAAATTAATTTTTACTATTTTTTTGTAAGGAGAT
TCATGCTTTGGTCTAGAGGAAAAAGGAAATTAGAAAACTTGATATGTACATCTGATTTGATAACATTTAA
TTTCAGTTTGTATGCTTTTATGCTTTATACATTTTTATATGGTTGTTATCATCCAATTTTTCAATTTTTT
GGTCAAAAAGAAACCCTTGAAATTTTAATCACTTCTAAAAACAAAAGCAATTTTTTAGAACCTAATTCTT
TTATGTATCAAGAGGACTACTCTAAATATAGCAAAATAAACAAAAATATTTACAAATTTAGCAAAATATT
ATCGATAGATGTTAAGCGACATTAAAAGGCTATCATCTATCATTGATTCATGTTGATAAATCCGGGACTA
TGAAAATTCGTTATGTCTGAAAATATTTTTAGCAGTTTTGCCATTTAAAACAGTTTCCTTATCACGAGGC
ATGATGAAAGCGTTAAAGATGCATGTATCAATTTAGTTGAGATATCCGGGTGTGTACCTACTTATTCCTT
GATTTCAAGTATTTTTTGCCTTTAAAAAAAATATCCCTCTTATCAGTACTTGATTTAATTTTCAAGAAAA
TTGAAAGAAAATAGATAACAAATCATAGAAACTTATGACTGTGGAATTAGAGTTTAATTGCTTAATTTTC
AAAAGCTAAAAGTAAAAAATCAAATGGTTATCAAAACTACCATTGCCTTTTTGTTTCAAACTGTAATAAT
TCAGTTATATTATTTGTTTTGTCCTGTCTTATCTCCCTAATCATAGACATATTACTGGTTGGATTTAGTT
TGAGTTTGTTATGTTGCTTTTATTGCATGGCTACTAATGCATGTTAGGAATTATTGTGAGGAAAAAATTG
TGTGCTTCGGAACACTTTTCAGTGTATGCTTTTTTATTATATGGCAGTGATGAGGCTAGATTGATACAAA
TAATTGTTCGACATGTCTGGAAGAAATTAACTTGTTCAACGGTGCAGTTGTCTGTAACTAAATATCCCGT
TGGAATTGATAGACAAGTTAAGGATTTGCTCTCGCATGTCATAATTGATGAAACTAGAATGGTTGGATTG
TATGGAATTGGAGGTATGGGCAAGACAACTTTGGCCAAAGCATTATACAATCGGGTTGCTGATAAATTTG
AAGGCTGTTGCTTTTTAGCAAATATTAGAGAAGCTTCAAAGCAACACGATGGCCTTGTTCGACTCCAAGA
GAAACTACTTTATGATATTTTAATGTATGACTTTGTTAGAGTTGGTGATGTTTATAAAGGAATCAACATC
ATAAGGAATCGACTATACTCCATAAGGATTCTCTTGATTCTTGATGATATAGATACAAGTGAACAACTAC
AGGTATTAGCTGGAGGATACGATTGGTTTGGACATGGAAGTAAGGTCATTGTGACAACAAGAAATGAACA
GTTACTTGATATTCATGGATTTTATAAATTGAAAGAAGTTCCTCAATTGCATTTTGGTGAAGCTCTTGAG
CTTTTTAGCTGGCATGCGTTTCACAATAGTTGTCCACCAAGCGAATATTCAACACTTCCAGAGGATGCTG
TAAATTATTGTAAAAATCTTCCCTTGGCGCTTGAAGTTTTAGGTTCATTCCTTTATTCTACTGATCAATC
CAAATTTAAAGGTATATTGGAGGAATTTGCAAACTCCAACCTTAACAAAGACATCCAAAAGCTTCTTCAA
GTAAGTTACGATGAGCTTGAAGGTGATGTACAAGAAATGTTCTTGTTTATTTCTTGTTTCTTTGTGGGAG
AAGATAAAACCATGGTTGAAATGATGTTGAAGAGTTGCGGTTGTTTATGTTGGGAAAATGGAATTAAGAA
ACTCATGAATCTATCCCTTCTTACTATTAACAGAATAAATAAGGTACAAATGCATGACTTGATAAAACAA
ATGGGTCACACAATTGCTCGTTCAAGGACTTTTATATCTCATTCAGAAAAAAAAAATAATGGTTGAAGATG
AAGCTATGCATGTCTTAGATGGCATTAACGTAAGAAGTTATTGCTCAATCTTTGTTTATGCTTATTTATT
ATGAGAGCAACTTTTATAATTTTGAAAAAACTTGAAATCTTTGTGTTTGTTTCATTTGCAGGAAGCAAGA
GCAGTTAAAGCCATAAAAATGGAATTTCCTAACCCGACAGAGTTGGACATTATTGATTCAAATGCTTTTA

GTAAAGTAAAGAACCTCGCAGTACTCAAAGTTAAGAATGTCACATTTTCAAAAATTAGTACTCTTGATTC
TCTACCTAATAGTTTAAGGTGGATGAGTTGGTCTGGATTTCCTTTTTCATCGTTTCCTTCAAGCTACTCA
ATGGAGAATATTATTCAACTCAAATTGCCACATAGCTCCATTAAACGTTTTGAAAAAGAAGCATTTACGG
TATTTCTATCAATAATTGAGTTTTATTAATTTCAATGTTTGTTTATTTACAATTTCATTATACTTTCTTT
TTATATTCATTACAGCATTGCAAATGGTTGAAGGAACTTGATCTTAGCAACTCCATCTTTTTGGAGGAAA
TTCCTGATTTATCTGCGGCGACAAATCTCGAAAAATTGTCTCTTTCTGGGTGTGAGAATTTAGTAAAGGT
TCATAAATCAGTTGGATCTCTCGGTAAACTTGTTGATTTGTGTATTTCAAGCCATGTTTATGGTTTTGAG
CAGTTTCCATCACCGCTAAAGTTGAAATCCCTTAAAAGATTTTCAACTTATCATTGTACAATAGTTCGAG
GGTATCCACAATTCAGTAAAGAAATGGAGTCTAGTCTAGAACATTTGTGGTTTTATAGAAGTTCTATAAC
AGAGCTATCTTCAACAATTAGATATCTTACCAGCCTCAAAATTTTGTCCATCACAGATTGCAAAGAGCTT
ACAACTCTTCCAAGTACAATTTATGACTTAAGCAAACTTACATCCATAGAAGTCTCACAATCCGATCTTT
CAACATTTCCTTTCTCATATTCTTGCCCTTCCTCACTTCTCCACCTAACAAGATTAGACCTTTATGAGAA
CAAGATAACCAATTTAGATTTTTTAGAAACGATCGCTCATGCTGCTCCATCACTGAGAGAGTTGAACTTG
TCTAACAACAACTTTTCTATACTACCCTCATGCATTGTTAATTTTAAATCCTTGAGATTTCTTGAAACAA
TTGATTGTAAGTTGCTGAAAAAAATTCCAAAGATTCCAGAAGGCTTAATTTATTTGGATGCTCAAGGGTG
CATATCATTGGCCAAATTTCCTGACAACTTAGCTGATTTCATATCATGTGATTCGGTGCACATCTCATCT
CTTTCTCTTGTCTTGATCTCTCTTGTAATTTTCTTTTCATACAGTAGTTAAGTTCATATGTGATTGTGAA
TTAATAATATTATTACTCTCACATTCACGTTGGTGCAGGAACATGTAGATGGACAATTCAAACAACTTAT
ATTAATGAATTGTGATATTCCAGATTGGTTCAGTTACAAGAGTAGGAACAATCCAATAACGCTTTTGGTG
CCATCCAATGATCCAAGTTCGGAATTGAAGGTTTTTGCTGCTTGTGTCAAATTTCAAGTTAATCATGTTG
ACCAGGATCAATATATGGATCTTGAATGTAAAGTGTTCATAAACGACATTCAAGTATGGAGTTATGAAGA
AGTACCCTTTCACGACGAATCGAGAAGTATATTGATAAAAGCATCACCACATGAGTATATGTGGTTACTA
GTACTATATCCACATATAAATTTCCGACTAAATTCGGATGATATTATCAATAGATCACAGGAGATCAATC
TACATCAGCCAAGTTTTGGGATCAATTCCATTGGAAGGGACAATAATAACTGTAATGTAGATGATGATTA
TCGGAGGAATCATATTGGGGAGAGTATTTGGAGGAAATTTACGGTGTCGTTTGGTGTTACTTCCAAATTT
AAAGACTCCGAATTAAGTATAAAAACATGTGGTGTTCATGTCATCATGGAGGAATGGTGTGACTGAGGTA
GCCTTTTTACTGTTGCCTGTAAAGGACCAATCAAATGAAATTTATACTATGATGCTCATTACTCTACCTA
TAGTATTATTAAGTGGTACAAGTGACAAAACCGAGTCGATCCGTAGTGAGCACGAAGATTTGTTTGTCAA
AGTTAATTCTTTAGTTAATGAGATAAATACGCGAAGATGGTAAAAAAGAACGAAAACAGGGACCCACATC
CTTTTGACTTGATTGCAGAGAGAAGATGAAAGAAATTTTGATTAATTTACCTTAATAGTTACTAACCTTA
ATTAATTTACGAAATTTTGATCAAGAAAATACTTACCATTTACCAAGTTAAAGTTCACCCAAAACAAGTT
TTGAAGCAACTTTAAGTCCTTCAATTTTTAACCAAATAACATCAAATCTTCACTCATAGCAATAGTAGCA
GCCTTTTAACTAATATATTTGAGAAATTGTATTGAGTGGCACAATAAAAATCTAATTTAGCAATATTAAC
ACTAACCTTTTCAATTTTGCAAATATAGCAACTTTTTAATTTTGGTTGATATTTCTTATTTTATTTTTTT
CATTATTCCAAAATTGCACTTCTTTGCTGTTTTCTCTTCCTCTTTTGTTTTTTTTTTTTATTGTTCTCCTT
CATTTTTCGTTTTCTTCTTCTCTCTCTGTCGTTCTTCTACAACATTTCTCCTATTCACCTACTTCTCTTC
TCCTCGTCTTCTTCTGTCTCTTTTCTACCATTCTTCTCCACCGTCCTTCTCTTCACTCTTCAGTTTCGTT
CTTTTAGATTTCTCCCTCTTCGCCAGTTTTTTTTATCATCTTCTCCTCGTCTTCTTCTTCTATTCTCCTC
ATTTTTTCTTCAGGGAAAACAAGAATTATATATGTATTTCTTCCCCTTTTTTGAAACCCTAATATTATTT
CTGTGAATTGCTCATATATTATTTATTAAAATTCGGGCTACGATTTGTTCTTCCTCATCTCTTTTTATTT
GTTCTTTTATTTGTTCCTAATATCTTTTATCAAAAGGAACTTCGTAGATCATTTTGGTTTTATTTAGTTA
CGTTCGTTTCTGTTTTTTTTAATTTGTATCAGTTTTTTATATCAGCGGTTTGATATACTTATATTATATG
TATTAGTTGATTGATATACTTACTATGTGTTTTTTATTTTTTCAAAATTGTTGCAGTTCATTGTAGTTAT
GGTTAAATTGGCAGTAATTGTTTTTCAGAGTGGTCAATGGAATGAGCAACATTACTACATGGATTACAAA
ACAAATTGTGTTTTGGTTGATGAAGTGATATCATCATTTGATTCTTTTGTGGACTTGATTCGAATTGAAA
TTCAGGTTGAGTCATGTATTGGACTTTCAGTTTTAATTTGTTGACCATAGGCGATAATGATGTTCAACAT
GTTATTAAGATTGTAAACATACCATAACAATTGTATCGACAATACGTAAAGTGTATCATGCTTAGTGCAT
CAGGTGTATTTAATTAATTGAGTGTATCAACAGTTCCACAAGTGTATCAACAACATATCAAGTGTTTCAA
ACTAATAATATGTATCAATGAAGTTTAACAAGTGATTAAGTGTATTACATCTATCAAACGTATTAGTAAA
CAAACAAGTGTATCAACGATATATAAAGTGTATCATTCTTAGTGAATCAAATGTATTTAATTGATTGAGT
ATATCAATAGTTGAGCAAGTGTATCAACAGCATATCAAGTGTTTGAAACTAATAATATGTATCAACGAAG
TTTAGCAAGTGATTAAGTGTATTAAATCTATCAAGTGTATCAACAAACATTAACAAGTGCTTCAACGATA
TATAACGTGTATCAACGATATATAAAGTGTATCATTCTTAGTACATCAAAGTGTATTTAATTGATTGAGT
ATATCAACAGTTGAGCAACTGTATCAACAACATATCAAGTGTATCAAACTAATAATATACATCAATGAAG
TTTAGTAAGTGATTAAGTGTATTAAATCTATCGAGTGTATCAAAAAATAATAACAAGTGTATCGAGTGTA
TTTAATTGATTGAGTGTATCAACAACACATCAAGTGTTTTAAACTAATAATATGTATCAGTGAAGTATTA
GAGAGTAAAAAAAAAGTGTACGAGGAGTACATCAGCAAAACAAATCAGGTGTATCAACGATATATATTGG
TGTATCAACCGTATATATTGGTATTAGTAATGACTGAAGGGTAGCTTCGTAATTTCGTATATTTAAAATG
TGGCCTAGGCTTCAATTTTGCTATTTTTGCAAATGTAAAAATGATGTGCTATAGGCCTAATTTATGATAC
TATAATTGTCATATTTGCAAGAGCCCCTGTATATTTTTGTCTTCCTTCCATATAAAAGAAATAATAATAA

TGGCATAATTCTCTGAAAAACAAAAGAAAGTAAAATAACACAACATAGATGAATATAAATTTATTATAAT
AATTGTTATTTTAGTGCCACATCATTTTTTTTTATTAGTCAACTAATGGTCAACTTTAACGTAAGGACCA
TTTAGAACTATTTTCCAAACTTTGAGGGCTCAAGTATCACTTTTGAAACATCATGGACTAAATAGACAAT
AACACTAAATCTAAGATACCAAAACTGCATTTGCCCTTTTATTTATTGTTTTGCTTTTTTTCTTTTCCTG
GTTTTGTAGTGGGTGAAATGTTATTAAGCATCCACAAATTCTTTTCTTTCATGTTGTACTAATCTTGATT
TTTCAATGGTACATTAATTGCATTATGAAATACAATAGGGCACTTGCAAAAATAGCAAAATAATTCATGA
TAAAAAGGTTCATGTTACTACATTTTCTAATTGCAAAAATAGCAAATTTTAAAACTGATAGCCCTCCAAT
AGCCCTCTGATAATCGTCTGATAGTCGTCTGATATAATTAATTATTTGTCATATTTGACATATTCGCAAT
ATACAAAAAAGTGGTGTTATGGACTTTTTTCTAAAATTTATTGTCATCTGATGCACCTTTCCAATATAAT
ATATGAACATGTTCCTAAAATTTAGAGTAAAATGTTAGTAGATAATAAAATGTTTTTTTTAAATCAACAA
TACACTAGGTGTAAGACAAAATTTAAGAATCGTTTAAATTACAAAAATTGAAATTGGATAATCATCATAA
GTTGACGTAGTAATAGTTGGAAACAATAAAAAAAAATAAAAAAATAAAAAATAAAATAAAAAAACCAAAGAG
CTAATAAGGAGTCACGAGTTCAATACATAGTAACCACTTACCTTGTTCTTCCTATAGAATCTCACAAATA
AATTAATATTTTAAACAAAAAATAAATATTTTAAAAAAGGAAGAAGATGTTTCAAAGTCAATAAAAAAAA
TGGACAGAGGAAGGTCGTTGTTGGAAAGAAGAAACCAAGTATTAATATGACAGATAAAAAGGAAAAGAAA
TAGTGAAACAGAGAGAGGCACCAGTCATCTCAATATGAGTAGAGATATGAGTAGAGCAAGTGGATCATCT
TCTACCTTTCAATTCCTCAATATGAGTAGAGCAAGTGGATCATCTTCTTCCTCACATTCTAGATGAAGTT
TTGATGTATTTTTAAGTTTTCGAGGGGAAGATACTCGATCCAACTTCACCAGTCATCTTAATATGGCTTT
GCGTCAAAGAGGAATCAATGTCTTTATAGATAACAAGATTTCGAGGGGTGAAGAAATTTCTGCATCTCTT
TTGGAAGCTATTGAAGAACCCAAGATCTTCATTGTTATAATCTCCGAAAACTATGCATCTTCCCGTTGGT
GTTTGAATGAATTGGTAAAATCATTATGTGTAACAAATTGAGAGGACAAATTGTTTTACCAATTTTTTAG
AAAGTGGATCCATCTCAAGTACGGATACAAAGTGGAAGATTTGGAGAAGAATTTGCAAAACTTGAAGTTA
GATTCTTCAACGAGATGCAAGCATGGAGGGAGGCCTTGATTACTGTTTCTCATATGTCTTGATGGTCGGT
TCTTCAAAAAGAGTATTTTTTTTCCATATATCCTTCTACTCTTTTGCTCTTCATATTCTCTTCTTACTAT
ACTTTAATCACTAGATCATGAGCGTGCTGGTCTAATGAAAAATAAAAAGAAAAAGTGATATTTATTAGGT
CCTGTTTAAACTTTGTTAATGAAACATTTGTGTTCCCTTATTTCTCTACCTACTCAACTTAACATCAATA
ACCCTTTGCCATTGTAAGATGACAAATTCGGATTTCTTAGAAACAATGATTCACGTTGCCCCTTTATTGA
AAAAATTGGACTTATCTAGAAACAACTTTTGTAGACTACCCTCATGTATTACTAATTTTAAATCCTTGGA
ACGTCTTTCTACAAGGGATTGTAAGTTGCTTGAAAAAAATTCCAAAGGTTCCAGAAGGAGTAGTTTGTATG
AATGCTAGAGGGTGCATATCATTGGTCAGATTTCCTAACAACATACCTGATTTTATATCTTGCAATGATA
ATGTGGTGCGTATCATTTCTTTCTCATAACCTCGTCTCTTATGTATTTCGTTCATATAAGAATTAAGTTT
ATTTGATTTGTTAATAATTTAACTTTTAATTATTTATCTTCATTGTGATATACCAGATTGGTTTTTGTTT
AGATGGAGTAATCAAAGAACTCATATTAATGAATTGTGATATACCAGATTGGTACAGGTACACGAGTATG
AACAATTCAATAACGTTTCTTTTGCCAGCTGATCATCTAAGTTGGAAACGGGGGGCTTTTTCTTCTCCTT
GTGTCAAATTTGAAGTTACTAATGATGCACTACAAGAGACGTGGGTACTCCCGACGCACAAATACGTCGG
CGAAAATGCAAAGTACGTCGGGAAAGGATATCCCGACGTACAAAACGGCGTCGGGAAGAACGTCAAGAGA
AATGCGTCGCGAGAGGCTTTCCCGACGCCGTACCAAATAGGCGTCGGGAAAGCCTTTCCGACGTCGGGAG
ATGCGTCGCCATCGACGGCGTCGGGAAAACCTTATTCCCAACGCCGTCTATGCCGACGCATCTCCCGGCG
TCGGGAAAACCTGTTTTCCAACGTTTTTTTCCCGACGTAATGAACGTCGGGAAATATTTTTTATATATTT
TTTTAAATATTTTATTTTTACTTTTTTCCTTATAATATTTTGCTGAAACATTCACAATAATGTTCGGATT
GCTCAAAAAAAATTTCGCGACGTTTTGGATTAAATTAAAGTATGTGATCAGAGCTTTGTTAGAATGTAAAG
TGTTTATCAACGATATTCAAGTATGTGATCATTCCTTAAACCCCTTTTTAACTGATGGGATAAGAAGTTT
GTATGGAGATCATGAATCAACAGGTGAGTACATGTGGATGATAGTACTTGATCCTCGTATACAGTTCCAC
CCTTGTGGGGATGATAGTATGGATAACTCGCCGGAGATTGATCTAAATCAACCATGTTTTGGGACTAATT
CCTCAGGAAGTAATTTGGATGAATTTAAGTTTGAGGTTAAACCATGGGATATACAGAAAATAATTATAAA
AATGTGTGGTGTTCATATCATCGTGGAGAATGAACATTATACACCTTTCAAGTTGTATTTATATATATAT
ATATATATATATATATATATATATATATATTATTGTTATATAAAAGACATTAAAATTCCCTCCTTGTATA
TGTAAGATTTCAGTTTTCACATGATTGATTATTTGAACCATTTGATTGCGCTACTTTCGAAAACTTGTTA
TTTTAATATGGAATAGCTGCAAATATATAGCAATTAGATTTAAAATATTTGCATATACAGCAACATTTTA
AAAAATTACAAAAATTAAGAATATGACAAAATATTTACACATCAAAGTTTGTTAATGATCATAAAAGTCT
ATCATTTATAGACAATGTTGAAAATATTTGGTCTATTACTGATAGATCATAAGGGTGTGTTTGGGGGAAG
GAAAGGAATAAGGGGGGAAAAGAATAAGGAAAATGAGGGATTATGAGAAGGAAAGGATTATAATAATTCT
TGTTTGGGGAAAGGAATAAGTGAAAGGATTATTTATAATCCTTGTTTGGAATAAGGAATAAGTGGAAGGA
TTACGAGAGATTATTTATAATCCTTGTTTGGGGGAAAGAATGAGTGAGAAAGAATATGACAATTTTTTTT
ATTATTTCTTTTTTTAATTAGTACAAATATAATTTTTTAAAGATTCATATCGTAAATCATGTACACAAAT
ATTCGTATTTTATTTACCAAAATTTGCAATTTTTATCAGAAAACATGTCTCATTAGTTTTCGTAAATACG
ATGTAAATTTTGAACTTTTTGTATGTAAGTTTTCCGTGTCAAAAAAATTAATTGATGTGTTACATGTGAA
AGAAAGTACATGTATTCTCTTTAAATTGATGTTGTAATTGCAATATCGAAAAATTGCATGTCGTTAACAA
TCGATTTAAAAAATTTTATTATTATTTTTTAATTCGTACAAATGTATTTTTTTAAATTCGTATTATAAAT
CCAATATACAAATATTTATATTTTATTTACCAAAATTTGGAATTTTTATTGGAAAATATGTTTAATTAGC

TTTTGTAAATATGATTGTTAATTTTGAATGTTTCGTTCGCAAGCTTTTCGTGTCAAAAAATTAATTGATG
TATTACTTGCGAAAGAAATTACATGTATTTTCTTTAAATTGATGTTGTAAGTACAACGAAAAATTACGTA
TCGTTGACAATCGATTTAAAAAATGACGACCAACGTAATAGCATTGAAACGAAATGAAATATGGGAAACA
AATGCTTTTTTTCCGAGTGCAGTTAAACGATGAACATCAGTTGTATCATATAACTCTATTTTGAAATATA
ATATTAAAATAATAATAATAATAATAATAAATTTCTTAATTTAAAATAATAAAAATAATAACTTCACACC
TTTTAGGAAAGAAATAGAAGAAATAAGAGGGGATTGAGAGGAAGTAAGAGAAGGATTATGAAGGATATTA
TTAAAATACAGCTGTTTTTAAAACTTATTACGATAAATAATGCAATCCTAAAACAATTTACCAAAATACT
GCTACTTTTCTTTTTTTTCATTTAAATAATGCAATAAATAATGCAATGTTTTTAATTGGGCGAGTTCCAA
AACTCACCCAAGTTAGTTTATTTAAAAAAAAAAAAAGCAAAGGGATGGGCGGACTCGCCCTCCTCAAACTT
TTTTATTTAAATATTTCTATTTTCTATAATTTAAAAAACTTATTTAAATATTTTTATTTTCTATCATTCA
TTTAATTATTTACTCTTAAAAAATGTAAAATTTAATTTTTAATTATTATATTATATTATATTATATTATA
TTATATTATATATATATATATATATATATATATATATATATATATANNNNNNNNNNNNNNNNNNNNNNNNNN
NNNNNNNNNNNNNNNNNNNNNNNNNNNNNNNNNNNNNNNNNNNNNNNNNNNNNNNNNNNNNNNNNNNNNNNN
NNNNNNNNNNNNNNNNNNNNNNNNNNNNNNNNNNNNNNNNNNNNNNNNNNNNNNNNNNNNNNNNNNNNNNNN
NNNNNNNNNNNNNNNNNNNNNNNNNNNNNNNNNNNNNNNNNNNNNNNNNNNNNNNNNNNNNNNNNNNNNNNN
NNNNNNNNNNNNNNNNNNNNNNNNNNNNNNNNNNNNNNNNNNNNNNNNNNNNNNNNNNNNNNNNNNNNNNNN
NNNNNNNNNNNNNNNNNNNNNNNNNNNNNNNNNNNNNNNNNNNNNNNNNNNNNNNNNNNNNNNNNNNNNNNN
NNNNNNNNNNNNNNNNNNNNNNNNNNNNNNNNNNNNNNNNNNNNNNNNNNNNNNNNNNNNNNNNNNNNNNNN
NNNNNNNNNNNNNNNNNNNNNNNNNNNNNNNNNNNNNNNNNNNNNNNNNNNNNNNNNNNNNNNNNNNNNNNN
NNNNNNNNNNNNNNNNNNNNNNNNNNNNNNNNNNNNNNNNNNNNNNNNNNNNNNNNNNNNNNNNNNNNNNNN
NNNNNNNNNNNNNNNNNNNNNNNNNNNNNNNNNNNNNNNNNNNNNNNNNNNNNNNNNNNNNNNNNNNNNNNN
NNNNNNNNNNNNNNNNNNNNNNNNNNNNNNNNNNNNNNNNNNNNNNNNNNNNNNNNNNNNNNNNNNNNNNNN
NNNNNNNNNNNNNNNNNNNNNNNNNNNNNNNNNNNNNNNNNNNNNNNNNNNNNNNNNNNNNNNNNNNNNNNN
NNNNNNNNNNNNNNNNNNNNNNNNNNNNNNNNNNNNNNNNNNNNNNNNNNNNNNNNNNNNNNNNNNNNNNNN
NNNNNNNNNNNNNNNNNNNNNNNNNNNNNNNNNNNNNNNNNNNNNNNNNNNNNNNNNNNNNNNNNNNNNNNN
NNNNNNNNNNNNNNNNNNNNNNNNNNNNNNNNNNNNNNNNNNNNNNNNNNNNNNNNNNNNNNNNNNNNNNNN
NNNNNNNNNNNNNNNNNNNNNNNNNNNNNNNNNNNNNNNNNNNNNNNNNNNNNNNNNNNNNNNNNNNNNNNN
NNNNNNNNNNNNNNNNNNNNNNNNNNNNNNNNNNNNNNNNNNNNNNNNNNNNNNNNNNNNNNNNNNNNNNNN
NNNNNNNNNNNNNNNNNNNNNNNNNNNNNNNNNNNNNNNNNNNNNNNNNNNNNNNNNNNNNNNNNNNNNNNN
NNNNNNNNNNNNNNNNNNNNNNNNNNNNNNNNNNNNNNNNNNNNNNNNNNNNNNNNNNNNNNNNNNNNNNNN
NNNNNNNNNNNNNNNNNNNNNNNNNNNNNNNNNNNNNNNNNNNNNNNNNNNNNNNNNNNNNNNNNNNNNNNN
NNNNNNNNNNNNNNNNNNNNNNNNNNNNNNNNNNNNNNNNNNNNNNNNNNNNNNNNNNNNNNNNNNNNNNNN
NNNNNNNNNNNNNNNNNNNNNNNNNNNNNNNNNNNNNNNNNNNNNNNNNNNNNNNNNNNNNNNNNNNNNNNN
NNNNNNNNNNNNNNNNTTTAATATTAATAATGATTTTAAAATAGTTAAAGTTAAGCTTTAAAAGAATTAT
TTAAAAAATTTATATATATATAATATAATATAATAATTTAAAATTAAATTTTACATTTTTTTTAAGAGTA
AATAATTAAATGAATGATAGAAAATAAAAATATTTAAATAAGTTTTTTAAATAATAGAAAATAGAAATAT
TTAAATAAAAAAGTTTAAATAAGAGAAAAGAAAGGAAATGATGAGGAGGGCGAGTTTTGAGAACTCGCCT
AATTAAAAAAAAAAAAAAGAAAAGTAGCAGTATTTTAGTAAATTGTTTTAGGATTGCATTATTTTCGTAA
TAAGTTTTAAAAACAGCTGTATTTTCATAAATTATCCGATTATGAAGGAATAAGAGGGGATTATATATAT
TCCTTTCCCTCCCCTTATTCCTTGCCTCAAACATGGAAAGGATTATATAGTCATCTCCTTTCCTTTTCCT
TTCCTTCCTCCAAACATACCCTAAGAGTTTATCAGCGATACAAATCTATTATTACTAGACTTTGTTATGT
TTGCATTTTTTTAAAATGTTGTTATACACTTAATTATTTTTCCTAAAATTGCTAGCTATATTACAATTAC
TCTAATATTAAAGGTTAATTATAATAGATACCAATTTCGTAAATAATAATTAAATATATAGCAATGTTGT
GTTAAAAAAAAGTACAAATATAGCAAAGTAATTTCAAATATCAATATAGCAGAAAAAACACTCTTTTTTC
AAAACTAAAGGACGATTCGCTACAACCCCAAGATTGATCTTATTGAGTTATATTAGTTCATCATCTCACA
ACCTTGAGTATTTGTTGACCAACCCTCCTCCTTAAAGTTGACTATATATACAAATTTTATATCATTTGGA
TATGTTAAATAATAAACTTTTGGCTAATTTAATTAAATTTCACCATGTCATAATAATAAAAATTAAAAAG
GTCACTATGAAAATTATATAAATGGTCAGGGGTTGAACAACTAAAATTGTTCGAGTCCAAGCCAATTTAA
GTATATGATGAAAATTGGGATAGTTACAAATGTAACAATTATATTCAAAATAATTAAGTATATAACAACA
TTTTAAAAAAATTACAAATATAGCAAAATTTGTCAAAATCTATCAATGATAGAAGTCTATCACCGATAGA
CCATGTAGCAAATGTTGGTCTATCACTGATAAACCATAAGAGTCTATCAACGATAGAAGTCTATCATCGA
TAGACTTTGCTATATTTGCAATTTTTTTAAAATATTGCTACATACTTAATAATTATTCAAAAAATTACTA
CCTATACTGATTACCCATAAATTTGTCATGTTGTCAAAAATCTATCAATGATAGAAGTCTATCACCGATA
AACCATGTAGCAAATGTTGGTCTATCACTGATAAACCATAAGAGTCTATCAACGATAGAAGTCTATCATT
GATAGACTTTGCTATATTTGCAATTTTTTTAAAATATTGTTACATACTTAATAATTATTCAAAAAATTAC
TACCTATTCTAATTACCCATGGAAATTTGGGCCAAGTCCAAACCCAACAAACAACTGGGCCCAAATCCAT
ATAAAACCCACTAATGGAAAAACCTCTACGAGCTAACTCTAGAGATCCAAGGTTCTAGAGATTGAAGCTC
TAAAGATCTCAAGAATATATCTCCCCTCAAACTCTAAAGCTCTCAAATAATAATGAAAATCGAAGCTTTA
AGGGCTAAGCTTTGAAAATATGAAGAACACAACTCTTCTAAAGTTTTAAAGTCGATCTAAGAAGATCAAC
AAGATCAATAAATCAAAGGGTTGATCTTCTAGGAAGATCCTTAAATCCAAGGGTCTAAAGGTTGACCCTC

TAGGCAAGATCAATAAATTCAAGGGTCGATCTTTTAATGAAGATCAGCAATCTTAAAGGCTAATCATATC
CAGGAAGATCATCGAACCCAAATGTCAATCGTTTGGAAAGAATAACAAGCTTAAACATTTTTATTTTTTC
AGAGAGTATCAAAGTATTATGTACTAGAGACCGTATTCACTATATATACTAAAACTAATACAAATATAAA
GCTCAAGTTCTACGAATTAAATTTATTTCAAAATCATCGAACAAAACTATGCTTTAACCATTTTGACATA
TGAATTGTTTGTTGAGTTTTATTTTGGTTTTTCTTTTTTCCTTTTTCCTTTTCTTTTTCTTTTTTCATTT
TTCATTTATTTTGGGACAAGTTGCTTATATGACATTTAAACTAAAAAATGATAAAATACATAGCAAAAGG
ATAAGATAATTGCAAATATAGCACAAAATTTAGGATAAAATCTAAAAATCCCACGTATAGCTACCATTTA
AAAAGTTTCTTCTCAATTTTTCAATATTGGAAGCTATTAGTGATACCAATCATCATTGATAGTTATCATT
ATTGATAGTTGTCATCACTGATAGCTAATGTCACTCAAAGCTGTCAATAATTAATTATAACTTTAATTTC
AATTTGAAAAATGTGCTATAAATTGTTGTCATTGATAGCATAATTTTAACGAGTGAAGGGTAATTTAACA
TTTTTAAAAAAAATTGCTAGTTGACTTTGATAATTTTGTAAATATTTTATCCGTGTCCTAGTTTTAATTT
AATTTTTAAACCGCGATCTTTACTATAATTTTCCTTTTTTTTAAGACACGTCTCTCTTTTTTCTTCTTAG
AAGTTGAATTGGTCTACCAATCGGCTATTTATTCTTCAAATGAGGATTTTATAAACACGTCCTTTGATAA
GTTAATGTGTAAAAAACATGTTGAAAAAGACGACGTTTTTAACAATATAAATGGGGAATAATATGAAAAT
TCTTCATTAATCTAACTCATTGTTAAAGTGAGTTTATCTCAATGTGCACTCTCTTCTTTTTTAACATTGA
CTTTATCTGTTTTTGATACATACAGTTATATTTGAAATTAAATTATAAGTTTAGTCTCTAAATTTTTAAG
CATTTAAAAATTTATAAGATATATGAACTTAAATTGATATCTATTTATTTTTTACCATCTATGAATTCGA
AAATTCAAATACTAAATTTCTAAATTTTGGATTAATTAATCTTTTTTTTTTCCTTTTCTTTTGAAACTTG
ATATAATAATAAATCATTTATGCTTTGAAAGATGAGACAATGAATTATTGAGGTCAAAAACTTAGATATC
ACGTAATTAATATCAAATCCTTGACTACAATAATTTTGTTTTTAAAAAATCAAAATCTTCTCTTTATATA
TATACTCATCCTTTTGGGTGTTTACAAAAATCAATAATGGCAACCAAGCAGAACTAAAAATCAAATCAAA
TACTATTTCTATACTTTGATCCTCTTTTTTCTTTTTTCCAAAATTTTTTAAAGAAAACAATAATAGTTTC
TAGGAAATTACAAGAAAAAAAATACAATGTGAGTGTTTAAGGTAATAAATCTTATAACATACAATAGTTAA
TATTATTTAGTGTTTACTATTATTTAATTAATAAAAAATCTTCTAAACTTGGAAAATCATTTTTAAATTA
ATTTGTCCAAACATTTATTCTTAATTTCAAATATATCCCTCATTTAATTTTTACTTTGTCATTATTTTAA
AGAAAATTAAACCTATTTCGAATTCCACGACTACATATTCAATGTTATATTCATTGATTTATTACTCTTT
AAGTTGGTTTTAAATTTTTTTATTCCTAATTTCATAATAACAAAAAAGGTGCTTTAAATTTTGATTGAAT
TTAGAAGGTCTTTTTTCAAAAGAGATATATAAAATGTAATTATGGTTTATATTATTTTGATATATCTTTA
TTAATCTTTTCAAACTCCATAAAAAATAGGATAATTAGAATGGATAGCAATTGTTAGAATAATTATTAAC
TATGTAGCAACATTTTAAAAAAATTACAAATATAGCAAAATCTATCCGTGATAGACTTCTATCGTTGATA
GACTCCTATAATTTATGAGTGATAGACTAACATTTGCTACATGGTCTATTGATAGACTCCTATCATTGAT
AGATTTTGACAAATTTTGCTATATTTGCAATTTTTTTTTAAAATGTTGCTATATACTTAATTATTTTGAAT
ATAATTGCTACATTTGCAACTATCCCTAAAAAATATAGAAATATTCAAAGTTAATCTTCCCTATAAATTA
AATAGTTTCATGAATTGTTCATGGATTTATAAAGAAAAAATGATTCTTTTTCTTTTGTGTCAAGATGACT
GATTCAAGTTAAACCTCAATCTCTTAATTTTTAACTTTTACCTTATTTTTTTCTTTACATTTTATTTTGA
GGAACTGTATTTTGAATTTTTTTTAGACTACAAGTTTAGATTTAGGGATAGTTGCAAATGTAGTAATTAT
ATTCAAAATAATTAAGTATATAGCAACATTTTAAAAAAAAATTGCAAATATATCAAAATTTGTCAAAATCT
ATCAACGATAGAAGTCTATTACCGATAGACCATGTAGTAAATGTTGGTCTATCACTGATAAACCATAGGA
GTCTATCAACAATAGAAGTCTATCACCGATAGATTTTGCTATATTTACAATTTTTTTCAAAATGTTGCTAC
ATAGTTAATAATTATTCTAACAATTGCTATCCATTCTAATTACCCTTTAGATTTATAATTATTTGTTTAA
GTTTCATTTTAAACTACTCAAGCTCTTGGCAATACATTAATACAAAATCTCAAACATATTAGAAATATGC
TCATACATGTTTTGGAACTTTCTTAAAATAATTAATTAGAGTTTTCCAAATATAAGAACTCTTTGAAGTA
CGAAGAAAAGCAATCTTTTAAACAATAATAATTATAATTTATTATCTGTTTGATGTTTTACCTCTCAAAA
ATGATTTTTTTCTAGCAAGCACATAGATCAATAATATCAAGTATGTATGATATGATCTCTAAAGAGTTCC
ATCAACGAGCCCAAGATTATCCTCAATGGTATTATTGCAATTTTATGGAGTCTTTGGATTGAGAGAAATA
ATAGAATTTTTGGTAGCTCCCCTATCATAAATCCCAATTAAGCATGTGGGAGGATTGTAGAAGTTTCATA
GGTTGTCGGTGCAGTAGAGACCTTTCTTTCAAAAACTATTCGGCTACAACCATAGCCTTAAATCTAAACG
CCATTTGTAATTAATTTCTCAAACTTCACTCTAGCCCTTGTTCTTATATCAATAAATTTGTGGCGGTTTG
TTCGGTCTTAGGCTTCCTTGTGAAGTCCTAGATTTTCGGCTCTCGTTGTTTCTTTTCAAAAAAAAAATGAT
ATGATCTTGAGCAAGAGGTTTAATCCACACTATCCCAAATATTGTCAAAAAAAAAAGAAAAAAAACAGAA
TGAATTTTCAATTTGTTTGATGAATTCAAAATTTGTAGTTGAAGATACAATAAATTACATGATTAATATA
TTTGATAATTTAAAATATTTTTCTTTTTACAAAATATACTATATTTGAAATACTTGGAATCTCTTTGATA
ATTTTGTTGTGTTTAGATTAAAAGAATAGAACCAATTTTCTTCCTAAAAGTCAAAGGTTTGATTCTCATC
TTCGAAATTATTATACTAAGAAACTTATGACAACACTATCATACGTACATTATATCTCGAGAAAAAGTCC
ATTCATTAGCCTTAATTAAGTTCATACTGCATTCATGAAGTTAGAGCTGAATTATCAGGGTATCCTTTGG
TGATTTCTCACCATTTTTGTATAATAGTTCTATTTCATCAAGAAACTTATAATTTTATTACTCGAATTAT
GAAAAATTAAAATAGGTTTTTAAAACTATTTATTTTAGTTTTCTAGACTAGGCTTAAATCATAGAAATGT
TTTTTAATAGTAGATAACAAAAAAATATAACAAATTAAGGTTGTATGCATAGGCAAGATTGGGATGAGTT
GTCCGATGGCTAATAACTAAAATCTCATACAATCCAAACATTAAATATCAAATATACATGATACATATAT
ACACACATATATATTACAAACTTTAAGTAAAAACAAACATTCAAAAAAAGAAAAATATTAAAAAATAAGC

```
CTAAACGAATTTAAACTAAGCAATAATTGACATGATAATCTTCTATATATCTTAGAGATTTGAATTCAAT
CTCCAACCCAGTAATTGTTGTACTAAAATAACATTAAAAATTCACAAGTTGATCAATCCTTAAAAAAATT
TTAAATTATTAATATATATATAATTCTTAAAAGCAAAACAACTCTAACCAACTCTTACGGTTTGAATTTG
GTAATTCGATTACTGGATCATTTGAGCCCTCTAAGTAGAATAGTAATTACAAACTTGATTAAATTTTCAT
AAATTATATAGTTATCCAACAGACCCTCAAACTAAGCCCAAACTAATTGACTTTTTTTTTATTTCCATTG
CTTATATTCTTTCAATTATGTATCATTAAAGCTAGCTTCCTTGTGGATTGACAAATTAGAAAAAAAAATGT
ACGAAAAGCAAATTATTTTACGTAATAACAATGGCTTAAGTTACAAGTAATAATGCCAAAAACTAGATAG
GCTCCATTGGACTTTGTCCGATTTTCATTTCCTCCAAGCTATATCTATAAATTGTGCAACAATACACATT
CATAATCACAAAACACAATCCTTTTCCTAGACATATTAAAATATCAAGTACTTGAAGAATAACAAGATGA
TGATGTTACATAAATCACTGGCCATCTTTGGCTATGCATGCCTCTTCATGGCCATAACTTCCACAGCTCA
ATCGCCTCCAGTGCTTGACACCAATGGTCAACCTCTGCGACGCGGTGTAGAGTACTACATCTCGCCTGCA
ATTACTGATGTTGGGGGCAATCTCACCCTAAAAAGTCGTAGTAACGCTCCATGCCCGCTCTTCGTCGGCC
AGGAACCCGTTACTTCGACAAATATCGGTCTCCCGGTCACCTTCAGGCCCACTGAGGCAGGCAAAGATAT
AATTGACGAAGGTACCAGTTTGAACATTGTGTTTGAAGCGTTGTCAACATGTGCAACATCGACACAATGG
AGAGTGGATGCAACTGAGTCTGACACCGGAAGAAGGTTCGTGGGGATCGGGGATGAGGACGGTCCTGCCG
GGATCTTTGGGATCAGTAGGGACAATGGAGCTTACAATATAGTATGGTGTCCTGCGATGATGGGGAGGCC
AAGGTGTGGAAGGGCTGGGATTTTGGTTGAGAATGGAGTGAGGCTGGTGGCTTTGGATGGTGATGCTTTT
CCTTTTGAGTTTATCAAAGCTTGAGATCTAAAAGGGAAGAATTAATATAGTTTAATTAATCTTCTTGCAA
GGGAAAAAAAATGTGTCTAATTGGTTGAATTATTGAACTAGTAGTATAATAATAAGTTGAAATAAAAGCTC
TTGTAAGAATATATATACTTATGTTATTTCTTGTTGTTGTTAACTAATGTGTTTTGGTTTAGAATTTGGA
TTTGTTTCAGATTGAAATATATGTGTGTTTAAACAGTTATTGTGAATCTAATGACTTGTCCCTCAATAAT
TTTTTTATTATTATTATATAATTAAAATCTTATTTTTAAAGTCAAAATATGTATCTAGGTCAACTGATAT
AAAGTTTTGTTTTGAATTCAAAGATAATAGGGAGGACCAAGTTTATGACTAAGATACTAAAAAAATCAAC
AAAAAAAAAAAAAAAAAANNNNNNNNNNNNNNNNNNNNNNNNNNNNNNNNNNNNNNNNNNNNNNNNNNNN
NNNNNNNNNNNNNNNNNNNNNNNNNNNNNNNNNNNNNNNNNNNNNNNNNNNNNNNNNNNNNNNNNNNNNN
NNNNNNNNNNNNNNNNNNNNNNNNNNNNNNNNNNNNNNNNNNNNNNNNNNNNNNNNNNNNNNNNNNNNNN
NNNNNNNNNNNNNNNNNNNNNNNNNNNNNNNNNNNNNNNNNNNNNNNNNNNNNNNNNNNNNNNNNNNNNN
NNNNNNNNNNNNNNNNNNNNNNNNNNNNNNNNNNNNNNNNNNNNNNNNNNNNNNNNNNNNNNNNNNNNNN
NNNNNNNNNNNNNNNNNNNNNNNNNNNNNNNNNNNNNNNNNNNNNNNNNNNNNNNNNNNNNNNNNNNNNN
NNNNNNNNNNNNNNNNNNNNNNNNNNNNNNNNNNNNNNNNNNNNNNNNNNNNNNNNNNNNNNNNNNNNNN
NNNNNNNNNNNNNNNNNNNNNNNNNNNNNNNNNNNNNNNNNNNNNNNNNNNNNNNNNNNNNNNTTTTTTT
TTTTTTTTTTTTTTTTTTTGAAAGGAGATTAATCACTATACCAAAATTCATTAATATATGGCAAAGTTAC
AGATGTTTATATTGGTGAGGGACCATACTACAAACATTACTTTAACAATGCCCTGTAGTTTAACGATATT
ACGGTTTGGTTGTAGTCTTTGAACATTGAGTTTTTGCTAGACCACTTACCAATAAGAGAGCCTATATCTT
CCCATAAGTTGACCAAGCTTTTTTCTACTTCTCTGAAGATCCTCCCATTTCTGTCCAACCATATTGTCCA
CAAGAGAGCCACCCCTGCATTTAGGCTGATTACATTCTTTCTGCTTGTTTGTTTCAATTCTCCCAACGAG
ATACAGAGGGAATTTATGTTGCTTGTGTCAAAATTTACTCCAATGTATAATTCGAGCCATTTATCCAACA
ATGAAAGGGTGTGTTTGATATGGCTTTGAATAAAATTATGAAATGGCTGCAAATATAGCAATAGATTTAA
AATATTGCAAATATGGTCTATCAGCGATAATAGAAGTCTATTGCGAATAAATTTTACTATATTTACGATT
TTTAAAAATGTTTGCTATATACTTAATTATTATTCCTAAAATTGTCTCATATATATATTATTGGCTAAAG
AGATTGGAGTGATAGTTAATTTATTGACTAAATTATTACAATTAATTGAGATAGTTTTTACTCGTTGAGA
TTGAAAAAAAAAAAGTACAAAATAAAGATAAATTAGTCTTTTTTTTCTAGTTCCTACAATCCAAATGAAA
TGCATGGCATTATTTATTACTATCGTCTAAGCACTCTTAACTACAAAGTTCTGATGAAATCCAATGCATT
AGTGTCGATATGATTGTACCGGCAATAAAAGCTCAAGAGACTTCTCACCATCAAACTATAATTATTGAAC
TGTTACAATTTTATATAGTTTTCCTTTCTTTAATTCTACTAGTAAAAGACATGTACAATGTGTAGCTTTA
GATTATAATAAACATTGCATGTATATGTTTAGGTTGTGCTAAATTTAAAAGTACAAAATTCAAAATGTAA
TAGAATTAGATATACATGGGAAAAAGTTATAATAATTAGATTAAAATATAAGAATTTAAATATAAGACTT
GCATATATCAAAAAAAAAATCTTTATATACAATATTTTTTTGTACAATTTGAAGATAATTTATTGTCACC
CATGGGCTTGATCCAAACTTTTGTTGTCTTCACTAAAATTCCTCTAGATAGTGCAACATAAAGTTGTCCA
TGAGAAAACACATGAAGATATATTCCAACATTAGGAATTGTTTGTTCTTGTGATTTGTTAATTGTCATTG
CAAAGCATAAATGAATAGAAAATTGTTTTCTCTTGAACTTGAATAGATATCCGTTGCCATTTTGTGGGCT
TAATGGTATTCATGGAAGAAAAACTTGTTTTCCTACATAATCACCCATTGCTATTTCAGCATGTATAATA
TTTTTGCTAAATGATTGACATAACAATCTTGTCCCATACACAATCCATTGCATTGATCCAAATTTCTCAA
TAACAATGTTGGTAAAAAAAAAATCAAGAAAGCCTTTCATACAGTAGAAAAAAATGTTACAAAAACTTTT
CAACTTTCAAGTCTTTAAATTTGTATTGTTAATTAGAATTAATAAGATATTTAATTTGTAACTAATTTCT
AAAATGTAAATTAAAAACCATTTGCATTCCAACTTTTCACATCCTATATTTTAATTCCACTAAACTTAAG
TATAATTAATTAAAAAGTGTAATGGGTAAATGAAAACCCATTTTGTGGGCCGGAAAGAAATGGCAGGGTG
TTCAGAGATGTAGAAAAAAGTTTGGCCAACATGTGGGAAGATATAGGAACTCTAATTGGTATGTGGTCTA
GCAAAAACCCAATGTTCAAAGACTACAACCAAACAAATATATCTCTAAACTATAAGGCTTTGTTAAAGTA
ATGTTTGTAATATGGTTCCTCACCAATATAAACATCTGTAACTTTATTAATGAATTGTGGTATAGTGTAT
```

GCTCACTATGCCTCTCCGTTTCCTTTTCAAAAAAAAATGAAAACCCATTTGCATTTAACTTCTCACATTG
TGCATACTTTAATTCCACTAAACTTGAGTTGAATTAATTCAAAAAGTAGTGGGGAAGATGTCCCTTTTAT
AATTATATATAGAAAAAATTAGAGAAAAGACCTAGTTTGACAAAATGTCCTAAAATAAAATTTTTTTCTT
GATTAGGATTTTCTATATTTTAGGACAATTTTAACCTTATCATTAACATTAATTATATAGGTATTTCGAA
ATTAAATAATATATATTTCCTTATTTACATTATATTGGTCATTATTATCTAATTTATAACTTTCTTAATA
TTATTATATTAATTAATGTAAAATGTTAATTATTAAGGAAAAAACGTTACCTTATATAATTTATTTTCAT
TTAACATCAAATAATATATCTTTTAATATGGAAATAATGTTCAAAATAGATCTTGATTAATTCCGGTACT
AAATATTTCATCTAAATTGTTCATCTGTTGCCTAAATCATCTAAATTGTTCATCTGTCGCCGTCAACTTC
TTCAATTTTCTCACCGGAAGTTTCTGCAGTCATCCAGTTTCGTTCTTCACCAATTTCGGTACATCCAGTT
TTGTTCTTCACCGATTTCTATTTCGTCCATCACCAATTCTGATTTCGTTCTTCATCGATTCTTAATTACT
TTTGGTTAGTTGTCTTTATTATCAATCATGATATCATTGTCATGATATTATACTTCCGGTTCGAGAAACA
AGAGCATAAATTTGTATGACGAGAATTTATTGTCAATCAAATTTATGTATGCAAAACAGAAGCATGGAAA
GCAAGATCTTGCTATGCACAAAACAAAACTTAGGTTTTGGCCACAGAAACGGGAAGTATCGTATAATATC
ATGAAAACAAGATCTTGCTATGTACAAATCAAATTTATTGACAATAATGATATTATACTTCCGATTTTCA
ATAAATTTTCCTCATACTTTCGATTAGTTGTCTTCCTCAATTTCTCATATTTTCGGTACTCCATTACATC
GTAGTTTTCCTAGAACGTTTAACCTTATATTTCGGAATATTTGTTTTAGATATATACTACTTCATATATA
TAATAATGCATATATTTTTACATTCAGTAACCGAAGAATATGATATGTTTATTCTGTGATTTAGTGTATA
TATACTTCTTCATATATACAATAATACATATAAATTGATATACAGTAATCTATAAATATGATACATGTGT
TATGTGATTTAGAATATATATACCCCTTCTTATTTACCATAATACATATATATTGACATCCAGTAACCGA
ACAATATTATATGTTTGTTACGTGATTTATTATATATATACCTCTCCTTAAATACAACATAAATTATTAG
TGACATATATTTCTAATATTTTTTGGCAGGATGACCAGAATTGCAGTCTTCTTCGATGGTTTTTTCAATG
AATCGAACAATTACGTTAGTTTTAGTGTGTTCAAAATAATAGTTAATGAATATATGTGCTATAAAGAATT
GTACGCTAGCCTTAGATCCCAATGAGACAGTTTTATTGATATAAACAATATTGATATATACCTTTACCGA
GGTCTTATAGAATGTGTTTAGAAGGATTTGTTATTTACTGTGTTTTTTACTTTCAATTCTATTGTTTAAT
ATTTGTTATATACATAATATTATTACATATATACGATAATTTTCCATTATAATTTCAAATTCAATTCTTT
TAATTATCCTCTAATCTCCTGAATATTTCATATCTACCAATGATATTTACATTATTTTCCTTAGTTTTAT
AGTTGAAAATTTGCATTGCATTATTATATACATAATATTATTGCAAATATACTTAACACAAACACTTTAT
CACATTTTTCATTTTAATATATATCCAATTCTAATGTATAACATTCATCATAATATACATAATATTATTA
CATATATACGATATTAAATCCAATGTAATGTATTCATCCCAATCAACGAAAAAGTGGTTGAATGGAGAGG
ATATGTTAAATTGATTTATGAATATATGAAATAATACATTTGTATATTTATGAATGAAATGTTGTGTTTT
CAATTTGTTAAATTAGTTTATGCATTTTATTTCAAATATTCATATTATATGTAAAAAATAATAAGTTATT
TTTGAAACAATAGTGATTATGTATGAAGGATAGTATATTAAATATAATAAATTGAGGAACTTTAATTAGT
ACGCTGGAAATTTATGGAAAAATATGAAAATAAAAAAATTGAACTAAAAACTGGAATATAGATATTTCAA
ATAAAATTATGAAACAACATACTAAAAGTCTAATAATAAAAAGGAAAAACTAATTCAATAAGGAAAACTT
ACATCTCTTTCGATTATGGCCAACACGTCTACAACAGTCTCATTTAACGCATCTTTTGAACTCTAGTTTA
GACAAAATTATAATCTTCTTTGGTCGACCAAATGAACGTTTTACATTCGGTGGGAGGATGACAACTTCAA
TTTTTGTATTATTAAATATATGCAGTAGTATATTTGTAACAAATCATTGAACATGAACCTATTATACAAT
AGTATATATGTAAGAAATCAAAGAAAATACTAATCGAATCGAGAAAAAAATAAAAAAAAAATGATACAAA
ACATGAAGGAAAAAAATGAACTATAGGTGTCAGTAAATACCTGATATTGTATATATACAGATGTATACAT
TCATAACATCATTTGTGTATGTAGTGATATATAATGAAATTCAACTACAACTTATGTATTATTGAATATA
TGCAGTAGTATGAATGTAATAAATCACAGAACATAAATATGAATAAGATGTATATATCTGATAAATCACA
TAATAAAAGTAGATTAAACAGTGGTATATAAATGTATATATTGTCCAGGAAAACTTTACTAAACAAAAAT
ATGAATAAGATGATCGGAACATGGTGTAATTATTGAATATACGCAGTAGTATATAAATTGAGTAAGAAAA
AGTTACAGAGTATGACAAAAAATATGTATATGTATTATTGAATATATATAGTAGTATATATATAACAAAT
GACAAAACATAAATGTATTATTCAGTAGCATATGTGTAAAAAATCAAAATAGAAGGAATGTTACCGAAAT
ATATGCTACGAAACGGAAGAAGAAATAATAAATTCTTGAAGGAAAAAAATATATGTTACGAAGCGGAAGA
AGAAAGAACAAATTTTTGAAGGAAAAAAAATATCTGTTACGAAACGGAAGAATAAATAATATACGAAACA
GAAAAAAAAAAAAAAAAAAAAAAAAAAAAAAAAAAAAAAAGACTAACCATATCACAAGAAGCAAATCAGAA
GGGAAGAAGAAAGAACAAATTTTTTTTTGTAATAGAAATCGGAGAAGAATGTCGGCACCGGAATTGATGAT
CATAAGAAAATAACTTTTGATTGGGCATGAAATTGAACATATTTATTTTCTAGTATAATTAAAAATATTA
TTACCATATAAGATAATAATTTATACCATATTTAAATCATTCAAAAGGTAATAAATATTACATTAATAAA
TACACATATATATGTAATACTTAAGGAAGTTACAATTGATATAATAAATATGGTTGTTGATAATTAAGGA
AAGTATTATTCAACATTAATGAAATTTGGATAATAAGGTAAATTGACTCGTTCTTTTGTGAAAAATGTTA
GCCAATTTAGGACTTTTATGAAATATATTAGCAAGAAAGGTTCATTGTCTAATATCATATAGCAATTTAG
GTTATTCTAGTTAAAAGCCCTTATATATATTACAAGAAAAATAAAATTAAAAATGAATTTACCATAAAAC
CAAAGAGTGATTTGGAACAATTCTAAACTCAAGGGTATAATTGAAAGAATAAAGTTTCTCCACATTTATG
CTATTATATATACTATAGATAAGTTTAATAAATGTAGCATCAACCTCTTTGTCCATAGGTATATGTGAAC
TGATTTTAAGACGACGGCTTAATTATATATAAATATTTTTAATGCTCTGTTGATGCTTAACAAATGAATA
CTTTTTAATCAAAAGTATTTTATTGAAACGTTTTCAAAAGTAAATTAGAATCATAACTATTTAGGAGGCG
TTAACGGAAAAGGCAAAGGGGCAAGATATAAAAAGATGGCTTCTATCTTCTTCATTACATTTACAAAGAA

CACACCAGTTCGGCCTCGAACACAGATTGAGGAGTCTTTTGACAAACTGATTGGTTGTATTTATGTTGTC
ATATAGAATAACCCATATAAAGAATTTGCATTTTTTGGGCAAACTGGATTTCCATAGGTTTGTGAAAATT
TTCTGATCAGTTACAGGCAATCCCCCCAGATCATCTAAAGGTATCGCTTTCTTTACGAAGCAACCGAGAA
CATGCCATCAGAATTAAGTTTCCATGTTGGAGTGTCACTGCCAGCATCAACAATTTGAGCATCTATAGAG
GTTTTAAGTTCATTTCACAGAGAAATTTCTCAGTCTCTCAGAAGTCTTCTAGGACTAAGCTCCTAATCAA
GCGAGTCAGTATTCCACATACCTCTTATAGAATTGTTTCGTTTATTAGATAATGCAAAAAGTCTCGGGTA
GTGAGTAGAAAGGGGGTTGTTGGTGTGCCAGTGACGGTGTCAAAAAGAAAAGTTGCTGCCATTTCTAATT
TGGGAGCGGAACTGCCATTATGATCTATGTCATTATAAGATGTGTTTGGAGTGAAGACTATTATAATCTG
GGTTTTCCACTACTTTTTTTTTTTTTTTTGTCTATATACTATACATAAAATACACAATTTTCTTCTATTG
ATTTTATTAAATCTTGTTAATTATAATGTTCATTTAATAAATTGAGTTTCATACTTTGTTTTTCAGTACT
TTTTTTTTTTTGTCATGTATATATATGGTACATGAAATACACATTTGTTTTCAACTAGTTTTATTAAATC
TTGATGTTCTTTTCTTCAATTACAATTTTTAGAAGTCATTAATTACATATCATAAAAATAATTTTAAGAG
AAAGTTTAATTACTAATTATTCATTTTCATTACTTAATTACTAGTGTTATTTTTCATTACCTTTGTGTTT
TTTTTTCAAAAATGATTTTTATAACTATTACCTTTGTGTTTTTTTTTCAAAAATGATTTTTAAAGGAAGT
TATAAGTTTGTTAATTTTGTAAAAAAAATTAAAAAAAAAATTATATAAGTTTTTTCATGATTTTAACTAC
ATACCGAATGCATTTGCCAAACTATCATTGATTTTTTATTTTTCCTAAGTATGATACAAATTTATTCTTT
TTATGATAGAAATTTATTATTTTTTCTCTTTTATCATGATACCATTCTTTTTATAACTAAATTGTCTTCT
TAAGAAAATGCATTATGATGCATATCTTTTTTTTCTTTAGATTTATGATACAAATTTTGTTATTAAAATT
TTGTTCTATGCTAAATCTTCTCATAAAATAGATTTTTAATGAAAAAAGTAGAATCGTATTTTTTCTGAAA
ATTATTCCTTTGATTTATTTAGAAATAAAAGCTAAAAAAAAAAACAAATTTTGATTAAATGGGAAAGACA
AGTATAGTTTCATTTTACCCCACCTAATTACCAATATTTATTTTTATTATGTTTATTTATTTCTATAACA
AATATTGTCAAAAGTTGTTGATTATCTAGTTTGTTTGTTTTTTTTCCTTTTTGAAAACTTGAAATAAATT
GATAAAAACTTTGAATTACTATTTTTCCTAATTTTAACACATTTTATTTTTCATATTATGTTTAGGCATT
AACCGTTTAACACAATTTTTTTATGATATTTTTCTAAATGAATTTTTCCTAAGTAATTTTTACTACGTTA
TTTTTGCAAATATATTTTTCGTAATTTATTTTTCCTAACAAGCCATATTCCTAAATTATTTTTGCAAAGT
AGATTTTTTCTAATTTATTTCTTTGAACATAATTCATTAGATGAATTTTTCTGTGGCATAATTAAAATTT
GTGAAATATATAATAACCCTTGGAGGAGTGAGGACTAGTGATAAAAGGAGAGATGTGGGAGTGAGAAATA
ATGTAAGAGAAGAAAATGAAGGAGTGAAGAATAGTGACATAAGAGAAATAGGAGAATAAGTATAATAATC
GTAATCCTAATCCTCGAATTATAATAACCCTTGGGCTAAATTACATCCACTCCACTCCTCAATAATCTAA
GGGCCAAACAACCCCTTAAACAGATCATGTTACGTTTGAGAAGAAAAAAAGAGTTAAGAGAGTGGTGAAA
ATTAACATGAACTCTCTTTGACCCTCGAGATAAGAGTCCTCTTTCTTTGATAATATATTCATTTGATTTG
ATTTAATTTTTATTTGAAAGTATAACATAATTTACAAAAGGAAGTTCTTCTTTTTCTTTTTCTTCATTTA
TCTATTTAATATCATCTCATTTTTGTTAAACTTTCTTGATCCCTCTATATTCTACTATTTTTGCAAAATT
AATTAGGTTGTTGAGTACATATTTAAAATGAAGTTGGGTTGAATATGTCCCGAAACTACTCAATAATTTT
TTAAAAAAAAAATCAACCAGCTAAAAACACTTCTATTATTGGAGGAGAATTATTGTAAATTACGAAATTG
TTCATAATATTGCAAAATTTTAGATTTTATCATTGATATGTGTTTATCAATATCATTCATAAAAATTACG
ATGATAAAAGTCTATCAATATCTATCAATAATATATCTAATACATATGAAGTTTAGGCTATGTTTGAAAA
TACCCTCTATCGAATATGCATATGTGAAATCATAATGAGAGAAAAAAAAAAAAAAAGGTCAAACTCCCCAA
AAGCTTTCAAATTTGGTGATAATTACCGAACTTCATCCATAACTATTTGACTTTCTTGTCCAACTTTTTC
CAACATTAGCATTAAAGTTTCAATAAAATCTTCTCAAGAAAAATCCATTCTTAGCCTCAAGTTCACACAA
CAAATTTTTTTTTTTAGAAAGATACTGAAGGTGAATAGGTGTACCTAAGCAATCACCAATAAGTGGAAACC
CTTTTAACACTCTTTTAGTGACCTTTTAGCAACCCTAACTAAGTTCACACAATATTAACAGGGTTAGTTA
GAGATAATTATATATATCAACGTACCCTTTTTGTATTTTCTCATATAGTTATATAGTCTTTCATATCGAA
AGATTTTGAGTTTAATAGTTAAATTTCAAAACAAAGATAAGCTTTTAAAACTATTTATTTTAGTTTTAAA
TAACGGACAAAGAAAAACTCATGGGTAGAATTAGGCTGTGGAGTTTTATTACACTAACATAAAAATTTGG
GTTAGTTGGGTCGGCAACATAAAAGCTCTGAATAGGGTTCAACTCAACCCTTAAATTTTGATTTGGACAG
AAGAGTAGAATTAGGTTGTTAAATTTTTATTATTGTTGTTATTAATTTAAAATACTTAAGTTCATTTGCA
ATACATGTTGAATAACTAAAAACTCATAAAACTGCAATGCTTATAAATACCAAATATACATGATATCTAT
TGAATATACTGTATTTTGTTCGACTTATTTTTCATTTTTTATTTTATTTATGAATATTCTTAATTTTCAT
GTAATTTTACTATAAATTTTACATAACAAAATATATAAAAAAAACGAAAAATGAATTTGAATTTAAGATA
TTTGAGAAATTATTTTATCTAAGAAAAGTAAACAAAATTCAATTAATCTAATTTTCTTTCTAAGTTTTTT
CGAAAACAAAAAAATTGACAAACTATTTATACTTTATACAATAACAAAGCCACTAGAACATAAAATTCAT
TATACTTATTTTTCTATAGTATAAATATTTTGTCAAATATTCTATTTTTACATTTTTTTTTTTTTTCCTTT
TGATCTGTCCACATTCTCAATCTCATGTTTTTCCACTTCCCTTTTTTCTCTTCACACCTTCTCTCCTCAA
TTTTTTCTCAAAAAAATCTTTTCTCTTGGGATAAATAATTATGCGTTTGGATCTTGGAAAAAAAAATATTT
GTCAAAAATACATTTTCTTTTAAACACTTTCTTTAAAATGAGTTTAAAAGTTAAACACTTACATATTTAT
TTGGATCTTTTTATAACTGTACAAATCTTTTTTCTATTTATTTGAACACCCTACACCTCAAACTGAGCCC
AAGCTATTTGACTTTTATTTCCATTGCTTATAATCTTTCAAATTTGTATCATTAAAGCTAGCTTCCTTGT
GGATTGATAAATTAGAAAAAAGGAACAAAAAGCAAAGTATTTTACGTAATAAAAATGGCTCAAGTTACAA
GTAATAATGCCAAAAACTAGATGGGCTCCGTTGGACTTTGTCCAATTTTCATTTCCTCCAAGCTACATCT

ATAAATAGTGCAACAGCACACACATTCATAATCACAAAACACAATCTTTTTTCTAGACATATTAAAATAT
CAAGTACTTGAAGAATAAGATGATGATGTTACATAAATCACTAGCCATCTTTGGCTATGCATGCCTCTTC
ATGGCCATAACTTCCACAGCTCAATCGCCTCCAGTGCTTGACACCAATGGTCAACCTCTGCGACGCGGTG
TAGAGTACTACATCTCGCCTGCAATTACTGATGTCGGGGGCAATCTCACCCTAAAAAGTCGTAGTAACGC
TCCATGCCCGCTCTTCGTCGGCCAGGAACCCGTTACTTCGACAAATATCGGTCTCCCGGTCACCTTCAGG
CCCACTGAGGCAGGTAAAGATATAATTGACGAAGGTACCAGTTTGAACATTGTGTTTGAAGCGTTGTCAA
CATGTGCAACATCGACACAATGGAGAGTGGATGCAACTGAGTCTGACACCGGAAGAAGGTTCGTGGGGAT
CGGGGATGAGGACGGTCCTGCCGGGATCTTTGGGATCAGTAGGGACAATGGAGCTTACAATATAGTATGG
TGTCCTGCGATGATGGGGAGGCCAAGGTGTGGAAGGGCTGGGATTTTGGTTGAGAATGGAGTGAGGCTGG
TGGCTTTGGATGGTGATGCTTTTCCTTTTGAGTTTATCAAAGCTTGAGATCTAAAAGGGAAGAATTAATA
TAGTTTAATTAATCTTCTTGCAAGGGAAAAAAATGTGTCTAATTGGTTGAATTATTGAACTAGTAGTATA
ATAATAAGTTGAAATAAAAGCTCTTATAAGAATATATATACTTATGTTATTCCTTGTTTCGCATCATTGA
ATATATGTGTGTTTTAAACAGTTGTGAATCTAATGACTTATGCCCTTCTATAATTCTTTTTATTATTATA
TTGTTCAATGTCTCTGTAGAGAGTACAAATAAGTATATAATTCGGTTGACATAAAATTTTGTATTGAGTT
TGAAGATAAAGGTTCATGATCAAGCTCATGACTCGAAAGAAAAGAAAGAAAGAAAGAAAATAAAAGAAAA
GAAGAAAATCTCACTTTTCAGAAAAGTTTAAAGTCACTTCTTTCAAGATGATTTATTGGTTTAGGTTTTC
TAGTGTAATCCAAAATAAAAAGGTCAAAAAGAGAATCAATTTTGTACCATAAATGCATAATTTGATCCAT
TTTTTCAAGATGAAAAGGACAATTGTGTTTGGGATATATGGCTTTGTATAAACGTATCATATATACATCG
TTCTATACACAATATATAAAAGCTTTATAGTGGCAAATAACAATTTATACATATAAACTTTGGAGGTTGT
ATCAATTTAAACCTTAAATTAATAACTGTATCTCAATTTAAATGTAGATATTTGGGGTTGTATCAATTTA
AACCCTAAACTAATATATGTATCAATTTAAATCTTAAACTCTTATAAATGTATCAATTTAAACTCTTTAA
CTTTCATATATGTATCAATTTAAATCATCAACTATCACGAGTGTGTCCAAGTAAAACATATATATTGAGG
TTCTAAATTGGTACATGCATATATAAGTTCAGGTCTAAATTAATACAATTATTAGTTCAAGAATTATCAC
TTCAGAACAGCTTGTCAGACGTCTCCCAAACCTTTATTCGAAGCCTAACTGGTTTGTTATGTGTAGGGGC
GAAGAAGAAGACAAAAACCACCTATTTATTATCTGCACTTTTGCAATCTCCATTTGGAAGAAGATAAACA
TTCTCTTTAATTGGAGCTTTCAATTACAAAACACCACATACCTTTGCAAGCACATATGTGAAGTGGAGCC
AAAAATATAACAAAAACATTATCATCTTCAACACTGTAGCTCTCTCCCTTTGGAATATTTGGTTTGAGCG
GAACAATAGGATCTTCAATGACAAAAGTCAAATCCATTCAGGTTTTTTGGGACGATGTTAAAGCTTTGAC
TAGCCTCTGGACTACTAGATCAAAGAATTTTTCAAGTTACTCGGCCACGAATATTGCTTTAAATTTGAAT
GCTTTTGTGTAATTCTTTTTTTGGGTTGTTTGGGCTATCTCTATCCCTCTTCTCTCTCTTTGCTTCCTTG
TACTATCTTGTTTTATCATTAATAAAACGGGGATGATGAGGGTGCTAAGAAGGTGTCCATTTTTTTATTT
AAAGAATGATAGATCTGCACTTTTTTTTGTTCCTTCATCTACACTCTTTCTCCCGACAAAGTTTTCTTTT
TTTGTATGATCGGCAGACAAAGGCAAAAAAGTTATTAAAGATTGTCATCACACCCCAAAAATTTTTAATA
GTTATGCATGCTCTTTCGAATACTAGCACGAATACTAGCAAATGTTTGTGGACTAAATTTAGCTTGAAAA
ACTATCTTTGGGTCTTTGAGACCATTAATAGATTTTTTCTTTGTTACGATATTTTTATTATTGACTTGAT
TTTTTTGGTACTAAAAAAAAGACAAGAAGCACACTATGTGGTTTTAGGCTTAATTCTTTGGTGAAAAATT
GCAAATATTTTTTTATGGTTTTAAGTTTTTCATTTTCTTTTTGAGTTGATACTCTATTATTTTTGCTAAA
ATAATAGGAAATAATGTGAAGTAGTCCTTGATATATTTTAAAATCTAACTATGTTTTGATTTTTTTTTTT
TTCTTTCTGAAGTTTAAGTGTATACTTCTTTCCTCTAATTCAATGATATAGAAAGAATTTTTTATCAATC
TACCTTTCTTACTTATAACATTCTAATATTTTAGCAATTTTTTTACTTCTTCCATTATCTCTACTTAAAT
TTTCAAAGATGAATATGACAGATCAGCATAGTATAGAAAAAAGGTAAAAATATCACGTGCATTATATTGC
TTGTTTAAGGACTAATATATAGTCAATAAAACTCACTCTCTTATAGCCATTATATATATTAGTCCTTAAA
CAAGCAATATAATGCACGTGATATTTTTACAATAATTATTAAGCCTTTGAATGCATAAAAAGTGTACAAA
ATTAAAATTACCAAAAATTTCAACAATTATCACCATCAAACCTTTGTTACAATTTAAAATAATTTTGTTT
CAATTCTCTTAAAGTTTTTAATAGATATAGTATTTAGCCCATAAATTTTTTTGGTTAATCGATCAATGTT
GAGTTGCCATCTCATATTAATTCTATACCCTTTAATTTCGTTCGTCTAGGAAAAACCATAATAATCAACT
TCTTTGTCCATAGCTATATCATTTCAATATCTGATTTAAGATTTAGTGCTCTTCATTAAAAGTATTTTAT
TAAATGACTAATATATGTTTTCAGAACTAAATTAGAATTATACATTTGGTTATATTTAAAAATGTCTCAA
AAAAATTATTTTAAATAACAAGTTGTTGTTGAAAATACTTACAAAATTTAACAAAAATTTATTTGTCGTC
TATCAATAATCAACAATTATAGGTTTCATCAATGTTTATTAATGATGGATGATAAATTTATATTAATGAT
GGATGATATAGTAGTATACAGTGGTGTTTTATCAGATCAAGACATTAATTAACATTTTGCTTTATTTGTA
AATATTTTGATTCATTTGTTATAATCGGAAAAAAAAAAACACTTATTTTAAAAACAAATTTTCCCTTTCA
ATCACTTGACAAAATTGATTTTCACAAATTTGGGTGATATCTAAGTTCATCCACATCTATTGACTTTCAT
GTCCCAACTAATCCAAATCTCTTAATATTTTGTCAAGAGCTCTTCTCTTCTAAAACTACCAAATAATAAA
TAATATATTTTTTTAAAGTAATAAAAGCCTTAAGGTACAAGTAAATGAAAGAAATAACAATTGTGATAAA
GCTAGACTTTGGTCATTTTCATCTCCTCCATGTTGGCTATAAAATGGCACTACACATTTGCATCCCATAA
CCATATCATTATTTTTCAAACAAAAACTAAATCACTCTTGTTGAAGAATCAAAAGATGATGAGGTTCCAT
AAGTCACTATTTTCCATCTTTAGCTTGCTATGCTTCTTCATGGCCATACCCTCTACTGCCGGCCAATTCG
ATGGTCCTCCGGTGCTCGACACCGAAGGCCGACCTCTGCAGCGCGATGCCGAGTATTTCATCAAGCCTGC
CATCACCGATGTTGCTGGCAATCTTACCTTAATTACCCGAAAAGGTGATCAGTGCCCATTTTATGTCGGG

CAAGTACCACTTCTTTCTCAAGAAACAGGTATGTATTAAATCTATTACTAAACTAAATTGATTCAGTAAG
CTATATGATATTTGTTAGAGTGTTGATAGACAATATTAAATGTGAACCTACACTCGCTCACTATTATTTA
AGCCGATAGATTACGATATATTGAATTCTTAACAATAGGTTTCGCGGTCTCCCTCGCACCTTATCGAGAG
GGTGAAGATACGATCAGAGAAGGCAGAGATTTGAAGTTTGTGTTTCAAGTGTTCACCATTTGCATAACGG
GGACTCAATGGAAAGTGGGGGAGGCAGATCCAAAGACAGGAAGGAGGTTTGTGAAGCTCGGATACGACAA
CACTGCCACCGGATACTTTAGGATAGACAAGAGCGACCTCGGAGTTTATAATATAGGATGGTGTCCCTCG
GATGTGCCCATTAAAGGGAGGCCAAGGTGTGGAAGTGCTGGAATTTTGATTGAGAAAGGAGTGAGGTTTT
TGGCTTTGGATGGACCTGCATTTCCTTTTGAGTTTGTGAGAGTTGATCCTGTAGAAGAGCTTGGTTCTAT
CCTGCAAGAGAAATAATAATGTGTAAATTTGTTGTAATTGTATACACAAACTCAATTAAAATTAAATAAA
ACTCTTTGTAAGGACTTAGTATTATGTGTGTTTATTGGGTGTTGTATATGATTAATATATATAATGTGAT
TTTGGTAGGTGGATATTGGTGTTCAGTAAAAAAGAAAAATTGAGAAATAACAAGTAGAGAAGAAATGAAA
TTGTCAACAAATGCCCCTAACATTAAAATTGGATAATTTGTCAACATGCAGAGAAGAGATTATTAGGTGA
TTTTTCTAAATTTTCTTACCAATTATTCTAGCAAATTAGTTTTCATTTATTTCTTTTAGGTGTGCCAATA
TTATTTTCCCCAAGTTTTGGTGTAAATTGTTATGTATCAGAAATGGCAAAACTTTTAAAATAAAATCATG
CTATAGTTTCATAAAATATTTTAGTTCATTTTATTATATTTAAAAATGTCATTTTAAATTATTTTGTCTT
AATGTAAATTTATAATCAACAAACTTTTACGTTCTTACGTTGATCGATTAAAATAATATAATTGATAAAT
ATAAAAACCACAATCTTGTTGCCAGGTACTTGCTTCAAGATTTGGGTTTGAATATTCTCTGATGTCAAGA
TTTGGTGTCCCAACCCCCTTTGCTTATTGAGTTGCACTCGAGCGAGGCAATTGCTTCTTTAATTAACAGA
TCTTACTGAAATTAAAAGCTTGTCTTTCGTGCGTTTTGAAGTTGATAGGGAGACTCCAAGGTGTTTCTAT
CTTGTACTATTCTAGGAGTTGTAATTCGACAACACACTCGCTGGCTTGATATTGACTTGGTCTCAGATTT
TGTATTCTTTTTTCAGTCTTGGGAGATCATTATCTTCATCTTCCTTGAATGACAATGGTTTCTGTGGTGA
TGATATCTGCTTCTCCCCTTTATTTATGGGGAGTCTGTTGGTGTTGTCTGAATTTTTTCTTCTAAATGCA
TTTTACCTTTGAAGAAAACCACGATGGTTGAACTAAAAAGACAGGTAAAAAGTTATAATTTCACTTTTAA
AACGATTCTCAAATGCATGTGTTCTATCTATACAACTTAGTATCCATTACCCATTCAAAAATTGGTGATT
CGTAACAATAATGGTATGTTGTATGAACTCCAAGCTACAAGTAATATCCCATGTCTTCCTCTTTTGTTAA
ATATTAAAAAAATTAATAATAGAAACAATTCAATTCTGTTTCTAAAAAAAGATTGTCACAATTCAAATTT
TTAACAACTATCTCAAATGCAGAAAAGTAAAGTGCACCCAACACATTTTCATAAAGTCTCATTCTCAAAA
TAATAATAATAATAATGTTGCAAAATCAACAATTAAAAAAATACGAAGACGGTAAATATCGACATACAGA
TATATGTAATTCATTAATAATGTATTATCTACATCCACGAGCAGAGGTAGAACAAAAATTATTAGAGGGA
ATGTTTTAGAATAAAATACGCACTTCTCGAAGAATTATAGAATCAAAATTGTACTTAATTGTGAAAACTA
AAGAGTTTCAGAGGCCACTGTAACGTTCCAAAAATTAAGATAATTTCAGAGTTAATTATCTTAATTTTGT
TTAATTAGATTTAATTCAATGGGCATTATTTTGAATCCATTTAATAAGAATTATTTGATTATATGGGAAT
TGAAATTAATTAAATATTTGTTAGATCAAAAATCCACAACTTTACTTAATTCTCTTAACCTTCCATTTAA
TCAAAATCTTACCAAACACCATGCTAAAACTTCTAATTAATTAAATATTCATCCAACAAGTATTTAATTA
ATTTCAATTCTTATATAATCAAATAATTTTTATTAAATGAATTCAAAATAACGTCCAATAAATTAAATCT
AATTAAACAAAATTAAAATAAATTATCTTAATTTTTGGGGCAGCTAAACCCTAACTTGATGACCAGTTAA
CAAGAGTTTTGTCATATGTTGTTCGTAATGCTACATAACGAATTCAAGGTATTTTAACAAATAATAAGAA
AAGTAGATGAAAGGTAGAATAAATTATTTTATAAAGTCAAAAGAAATACAAAATGTGTATTCAACAAAAC
AAATTAATTTGTAGTGAACAAACAAAAGAAGTTAATTAAAGATAGGGAAAGGCATTAATGAAAACTACTG
CAGCCACCCCTAATGACTTTTAGAAAATTAGGCCGATGATATGTATTATATGTTTCTTTTTCCATCATTT
TCTTTCCAATGATCTTTCTTTTTCTTTTTCTTTTCCCTTTTAGCCGACTTGAGGATAAGTTTAGACATTT
GCAGCCTTTTTTTAAGACAAAGTTCGAAATAAAAAAACAAAATGGTCGTGTTTGGATTAATTTTTTTTAG
TGCTATAGTTATAGGGATACGGGATTGAACTTCCACTTTTAAGATAAATGGTCATATCAATTACCATTTA
ACTAAGCTCACGTTAATAGAGAATTTGAACCTTCAAGTATGTTGTTTAGACTTTTCACCGATTATTATTT
GTATAAATTAATAGTAATAATCACATATCCACTAATGCCAGTGAAACAAAAATGGCATTTTTATTCTTAA
TATCATCATCGTTCATCGTCACTTATTGTAGGAAAAAGGAGCTAGTGGGTGTTTATAGCCCCAAAGGTAT
ATGTGTAATTTATTGTACCCTAGTTTTATTTCCTTTTTTGTCGGGCTTGTCATAGCCTATATATATTCTT
CCCTCTTGCACTCTTTCGATTATTAGAAAATAATAAAGGCTTCTATCGTGATTTTTTCTCCCTATGCTAT
AATTTTCCACATATATGTTGTGTCTGTCTTTTTCTCTTCTATCGTGGTATTAGAGCATGGTGATAAAACC
TAGCTGCCATTGGACGAAAACCTGGTGCAAGATAACCATAGTTCTTGCACCAACCCCACCTCAATCATAA
CTGTTACCACTACCAACAAAGAATTCGCCATAACCGCCATTTTTCCGCTGCCGATATTGTCACCGCTGTC
CAAGCTACTGTTGATCATTATCTTCAACCCTAACATATCCATCCAACTATAGTGTCACCACAACCATTTT
GTACAACATGGTGAGTCGTACTAGCCGAATTTACACCGTGAAGACAAGATTCATGAGTCATCCGCCTACC
ATAGAGGTAAATTCCTCATCATGTGTCGCCAAGTTAAGAGCATCAAGGTTTGGTTGGTTTATTATCGATA
ATGGTTCAACATCAGTTAGACTAATCTTCAGACAAAGTTTTAGCAACAAATCGCTAATCTTGAGGCAGCT
TTAGGTGCTTCCACAAGCTTCACAAATTCTGTCACTCCTTTTATCTTACCAATGTATACAGATAACCCGA
TAACCTATTTCTCTTCCTTAACTGCTACAAATTATTTATTTCACCCCATAGGAAATTCCATAGGAGTAAT
TTTCTTGTTTATTTTCTAACCTTTTGACAAACATGTTTTATTGAAGATTCAGTCAGCAATGGTCTAATTT
ACCACAACCCATTGATCTTAATTACAAGCGTAAGATGTTTGTATTTTGAAGAACAGATAGCCAATTGGGT
TGGGTTGATATTATTCTTCTAACCATCTTAATAGACATACAGGTTACATGCCTAATCGTGCTTTATACAA

ACTCAACTCGAAAAGACAGTAAGCCTCGAGACTTAAAATTTGTGCCTTGCATCCACGCAATGCAAAGCCT
TCTAGAACATTCATCTACCAGTGAAATTAGAAAGTCCCAACTCATTGTCATCAAGTGGACAAAGATGATT
TCAATTATTTGTAGCAAGGAAAATGGGACCTTGTTGAGAGATGTTCACTTCGTAGTGTAGGTTGGAATAG
GCACTCCAGATTCTTCCGTCAGTTTCTTGTATGCATTTTGTAACATTCTTGTCACAGGTCCGACTTGTCC
ATCGCCAATTACACGCCCATCGATCTTCACAACCTGAAAGTTCAAAGGAAGCTCCTCTTGTTAGGTGTTT
GAGTATTAGTTGGGGTTAGTTCAGTCCTTTGCCGAGTTGAAAAATGAAAATCAAAATAAAACTTTCAATA
TTACAAACATAAAGGAACTGATTAAGTTGGTTTGTGGCATTTTCCAACTTACATGAAAATAAATCTAACT
AATTTCAATGGCACTTAATAGTCTAATCTTATCCAAATTCCAGACAGTGCCATATAAGCTACAGTTACGG
AAAATTGAAAAAACATGAACTATCAAGGATACAATGAAGTGTTCATAATGTCCCAAGCTATATTTAATGA
CCAATATTAACAATAACACTACGAATTAAGACAGACAAATCCAGAGTTCAGAGGTTTCACGTCAATATTT
CCCCTTAACACGAGAGAAACCGAAGGGCTTTGGAGGATCTCAAGTCTTCCTTTAGAAGAGACCTTCGGGA
ACCTAGTTAAATTTTTGCCTCTTGTTGGTGTACAATTTATCTCATTCATATTGCAGTTGCGTCACAACTG
CAATTTTTTTTAAGATCATTTTTGGTAAACGCCTTTGGCTTCGAGGAATTGTTCATTCCCACCATTTGTA
GTTCTCTTATACTTTTATAAGCATATTTCTCAGTTTTCCAAAAAAATAAAATAAAATAAAATAAAAATAA
AAAAATTGTAGCCATAAGGTACTTACTGGGGTAAGCTCTCCCATCGTTCCAGTAGTCCATACCTGCAGAA
AATCCTCTTTAGTTGTACACAAACAGTTACAAGTGATGGAAGTCGGTTAGTCGTTATCTTAGTTGTCGCC
ATTTTTTAGCTTCAGCTATAATCACAGATTCACATCTATAATTCGTTTCAGTGGATCTTTTCAAGCTATC
TGTTACACTTCAAGTGCTTTGGATGATGTAGGAAATCAATTTAACAATGTCGACATAATGCAATTAACTT
TGAACACTTACACAAAAACTTAAGCAGATAATTCATGATGAACTAAAAATAAAGTCGTGCATTCATGATT
CATCATGAATTATGTGCTTAAGTTTGTCATGGTAGCCAAAATTTGAAAACTAAAAAGATTTGAATTTCAT
GAGCATTTTCTTTCTAACTCAATGTTATGCACAACATTCAACTTCAAATTTTAAAATGTAAATACGAAAA
CCTCATCTGCAGTATGAAATTCTGATAGGCTGATTCTTCGCTCCTCCAATACCAACTTCTCCTTCACCAC
AAGATCCATAACCTGCAACCATAAGATCAATATTGGTTAAATCAAGGTAAGGAAAAATGTCTATGCAATC
AAACTATCACAAGTCATGGTCCATATGGGTGGAATTCTTGAAGGATGATTCATCTGTGTGGCACCAAACA
GTGAGGCCACAGCATTTATAGCTCGGCCTAGTAATGTATTACTAGTATATGTAACTTATAGTGTAAAGTA
GAATAAATATACCTCAAATGTAGGTATAAGGGAGAGAGCTCATCCTAGTCAACCATCTTCGAGTAAAAGG
GTGACCTCTTTTATAAGGTGAACAGTAAGGCAGTTTGTTCCTAGACGAGGCTCTTAGGCTAATGGATGAC
AGAATGTCATACCTTACATTTTCTAGGAATGAAATGACAGAACTTAAGCCCTAATTGTGACATTTATAAT
GATGAATTCATATTGGCAACATATAAGGTGAAGTATCTTACAGTTGCACGAGTTATTCCAGGGAGACAGT
AATCAGCATGAGGCGTCAGAACATTGCCTTTCTTCACTAGAAACTGCACAAGTAAATGTATAAAATATTA
TATCATTATCTCAATTCCAAGTACAGAATATAAACAAATGAATGTATATATTTACTTTCTTGAGATCGAC
AAGACCCAACAAGAAGCAGAATCACAAGAGGACATGCTTCCTAAGATAATACCATAAGGTACAAAAGTTA
TGAAATTTGATACTTACAATGTTCGTAGCATTTGTTTCGGACACAAAACCATCTTTGTCCAGCATGATAG
CATCACCAGCATTTGCATTGTTCCCTTCAATCTATACGTTTCAATAGACTCGATCAATCACAAGAGATAA
TCAAAATGGACTGGACACAACCCATTTGATATAGTTTCAAGTCAACAATAGATGGTCAATTCAATCAAAT
ACAATTCAGTCAGTTTTACTGTGTTCAAAGTTACATTCTTTGAAGAAATATGTGCGTAAAGGTCACACTG
TTGGAAATTCAATACCTTTGCAAGAATGTTGTTGAGAAGATTATTGTGATGAATCTTTGAATCCAAATTC
TGGAGGCAAGATAAATAAATGAGTTCCGATTATAACTGACTGAATGAGGGTTAAGAGAATGAGAACAGCG
GAGTTGAAGCATTTACTTTAATCAAACTCATGAGAGCAAACCAAATTACTGAGAGAGATCAAATACTAGG
ATAAAGGAATAGTCTTACATTTGGTGAGTTCCTACGTGTTGTGGCAGTCACTAGAGTTATTCCACTCGAA
TTGTCGTAGACAGGAGGCTTCCATTCAGCAAGCACTGAATATAGTTACAACATACTCAGAAAGTTAAATC
CTAGAACGCACCAACACAAATAGGAAAACCTATATTATACAAGAGCCATTTACTTCTTGCATGATGCAAC
TAGGAAGTTTTAACATTTTTTTTATGAAAGTTCTTCTGTTAAAATATAAATACAAGCACAAGAGTACAAG
ACAGGCCAAGGAGATGAAGACATCCAGTTGGCTAGAAGCTAAACAAAAGAAAAACTACTGTAGTCGTCAT
GGTTAAAGCAGCTCATCTAGAAGCATAACTGATCCTATGAGAGGAATTCATGGAAAGGAGACCATAAATC
TTCACAAGTTCCTCTGTTTCTTTCTATTTGTTGCTTCTGATTTATTCCACCGAAAATCATGCAAAGAGCT
GTCTAGGATAACAACTATGCTAGTTCACCTCGTTCGAAAAATTGATCAGAAGCTCGAAGCAGAGAAGATT
TCGTAAAAATAAGAGTTCAGCAGCATTCTTGTATAATATATGCATCAGCAAATAGGGGATAGAGAAGAAT
TTTGAATTCTTCTTTTGGATTTCATTTAGAATGTTGACTTCTAAATCCAAGCTTACCATGTTCTTTTAAA
AAAAAAGGCTAATTATTCAATCTGTATTTTTCAACTTGAAAGAACATAAAAGGCAGGTTTGGCTGCATTG
ATACTAGAGCAAGAATTTTCATTACCAATTAAGGTACATCCATAAAGATTGAACTGGGGGCTCATTCCGG
AAGTGACCTACAGAGAGTAAAAACATGTAAATAAAGATATGAATGCTCCAAATTCGACCAATAGAGAAAA
GGAAAAGAATTCAAGTCCTTGACATGAAGTAAATATTAAACAGATGCATTATGGATGTTAAAATCCTTAA
TGTCAACAATCAGTCTACTTATTATAGCCCCCTTACAAAATTCATTAATAAGCCACTTTAGTTTAAACAT
AAAGAGCAATTGAAACTTTCGAGAAAATAAGATTTTGTTCCTTATCTTGAAAATAAAAAATAGCCAAATC
AACAATCCAAATAATAATAATAATAATAGTAAATAACAAAGGTCCAAAAGTGCTTAAGAATTTATTTGTC
ATGACGGTCGACATCAACTCTGCTAATGAAAAATTTTGTCTTTTACGAAAGCGGTGAACCATTTTTCTTA
TTTTAAGTTCTAAACTAGGTAGTGCGATTAAAATGTAAAAACAAATTTCTACAGAAATAGTTCACAGAAA
AGGTTAGAAGAGTTAGGGAGCAAGAGGAACTTTAGAATTACAGAACCTTTTTCCCACGTGTTAAAGATAA
TCGAATGTGTGCATTATCAAACATTCCATTCCGAATGAGCGTTCTGAAAATGGCATCTTTCACCTATGGT

TGCATTCAAAAAAAATATTACAAGAATAAACAATGCATCCTCTTTTAAGACTTCAGGAAAGTGTCTAAGA
CTCCATTTCCTACCTGGATTAGACAACGATAGAGCCAAAACCAATAGAGGTTCCAAGAAGATGGCATACT
ATATATACATATTGATGTTCAGTTTAGTGGATAGAAACAAAGTCATCCTTTGATGCTTACCTCTTCCCTA
CTTGGAACATTCTGGAAAGCTAAGGCTTTTGATGAATCAAATAACCTACAGAAGAAAAAAAAGATCTAAC
TATCAAGGTTCAGAAAATGTTAAAAAAGACAATTGGCCATCTAGCTTGAGCTCCTTTTTCCACTAACCTA
TCTAAATGTTCATCAAGCTTAAATATTTTTCCTCGATAAACTCTAAGTCCCTCCCAAACTGAGTCTCCAC
CTTGGACAACAGAGTCGAACACAGAAACCTTACGACAATTGAAGGTAAGAAATGAGAAACCAAGGAAATA
CAAATTCAAAAAGAGGACTTATTTTAACTGCATGTGTAATTTATTCCATGTAGCAGTACCTTTGCACTAT
CACGAGGTAAGATCTCGTCCCCCACCCATGCCAGTAGCTTCTCATTTGCAGGAACTGGAAGATCTGGAGT
AGGCAAGGGAGACATGAGGAGAAGTGATTTCTGCTTTACATGGCGCCTAAGAAAGTGGTAGAGCGGTATC
GTTTGCTCAAGCAAATCATATAGATTAAATGGGAAAGGCTGATATAGATTTGAAAAAAAGGAAAAAAATA
TAGTGAATCTCATTAGCTAAAACAGAAGAGATTTTCAAGATATTTGTCAAAGGTAAACATGTAGGCAAGG
AGACAATTGGTTGGTACTAAATACAAATCAAAAAAGTAAAGAATCACTTCATAGATCGTTATCACCATAT
CATGTTATGTTGTAAAAATAGTGAACTCGTTTTACTATTAGCAACATATCATTAATCTTTATATTCAAAG
TATCAAAGAGTTAAAAGTGCAAGAACAAAACTAAAACCAAAACTCACACGAAAGTAGAAAAGATATACAA
GAGCTTCAACCAGAATGTAACTAAGACCAAAATTCTCACTCAAAGAAAAAAATACAGAGTAAAGACATAC
CAACGGATATTTTCTTGGTGCCTCAAAAGCTGTTGATTTATGTACAGTTTTATACCACCATGGAGCCCAT
ACACCATCTATAGCTTTGGGACCAGCTTCCCATCTGAAAAATGATTGCAAGTCATCATGATAAGCTTATC
AATACCTGCAATAAACGGGGATGTAACATACCAGCCATGCTAAAATACAAAAGAAGAAACTTTGACTAAA
TATTTTGAATATCAAAAGCCAACTAGTAGCCAAGAGGTAGAGAGATGAGAGGGAGACAGATGCACCATAC
GTTGGCTATTATGATTAAATAGATTATATTCCTATAGTTGTAGCCAAAAGTGGAACTGCCTCCAAAAGAC
AACTAAGGAGTTACTAGTTAAGACTGAAGATGGTACAATAGGCTCTGAAACAATTATAGTTCACAACTTG
AAAGGAAATTACAATTTCAATTTTCATCAGTTTTGATCACGAATGGAAAGGTAGTATGTGAATCTATTCA
ACTTTCTAAAGGAACAAGAACTCCATACTTCAGCATTTTGTCTTGAAAAGGAATGCCGAGTTCTTCACAA
AGACCACGTAAAGTTGCCTGTGGATCAATAAGACAGAGAAAGTCAAATGTAGCACGTCAATATGATGAAT
CAAGTTAATCTAGACTAAAAGAATAAGCACTGAACGTAGGGGTAATGCAATATAGAGATCTTATTAATAA
GTTCTCATGCATTGTCATTTTACAAGGATTCAAGAACTACAATGAATGTGCTATATCATTAACAATATCA
ATTTGGCACATGCAAATTGTAATTACCACTTCCTACATGAACCTTTTGGATGTGATTATACTTCATGGTT
TTGAAGAAAGTGGTGATACATAAAGTATAACATGAAAATCCAATCATTACATCATAACCATCTCTCTAGT
GCAAAAAAAAAATGTGGTAACTCAGAAAGAAGTAGAACCTCGGGATTCTTTTGAAGTTCAGCTGCATCAA
TGATAGGAGGGAGTCTTCCCAACTCTTTGAGCTCATTGTAAACAGATACCAATTCTGAGAAGCCCAATTC
AGAAAAGGATGCAGGAATAACCTTTTCATAGGATGGCTGAGACAAGTTCATTAATTGTCAAGAAAGGTAA
ATAAAGAGTTTGGCGACCATCAATGGAACTTATAATGTACACAGAACAACCAATCCAAAGAATACAACAA
ACAATATGGTTCTTACGCTTCACATCACAACTACAAACAAAAATAAATTAAAATTTTAAATATATTGGTT
TTTCATAAGAAACTTCACCCTTGATTAGTAACAAGGAAGAATAGAAAATCCTTCAAAATTCGTTGTACAA
AAGCAAAAAAGAACACTTTTCGTCAAGATCTCTCCTGAGCAGGTATTTGTACAAAAGTGAGTTACTTTTC
AAATAATAGCGTAATCCATATGGGAGGTAAAATATTATGAGGTTAATACAAGTATATAGCTATTCATTCT
TTAGAAACCTTGTTGAATACCAACATAAAGGTAGTGAGAAATATCTTACCAAAATATCAAGTGGATTTCT
TATCAATATGAAGTGCCTTCCCTTCTTCATCAAATCACTTGCCAAACCAGGTACTTTTTGTTTTGCTATA
TGCTGTATGAATATCAAGTAGAAAAAATAAACATGAAACAACAAAATGATGTACACAATTGGTTAACAAG
TATCAGTTTCCTTTTCCTTTCTTTCTTTTTGGTTTATCCTTGTTTGCATTCAGATGAAGTTTATTTTTTT
GAAGGAGTAGAGATGATATACACAACGATTAAGCCATAGCATTCTAGTTGCATACTGATGTCTTTATTCC
CTATGCATTTTTGATTAACCGCTCATCTGCTTTGAGTTAAAGACCTCAAAACAAATAAGAGAAAGAGCAG
GAAAATGCAGAATTGTCCATTTACTCTCTTTCTACATAGATCTCTCTCATCCATACATCATGCACGGATT
ACATATTTTGATTTTTATGTTATATTCTAAACATCTAGACAAACAATTCCTCATCCTGGCAATGGTCCTT
TCAAAAGTTTTCAATGTCTTATTACTTATTAGTCAAAAGCATACAGTCAGTCCATCCAAAATTCATGTAA
TGTACATCAAGGAACACAATTCAATAGGGTATAGTTCACATTACCTTGCAAAATCGGAACTTCTTTTCTC
CTGGTGCAAAAATGATTTCCTTTATGACCTTATTTACGTCAGGTTCCTACAGCAAGCAGACCAAAAGGCC
AATGAGAATGATATAATAACCACCAATATATATGGTCACTTTTCCTTTCATCTTTTCAAGCAACACAATA
CTCTAGGATGAATATCAGTTGATATTGCCTACTTTTGGACAAGATTTGTGCATCCAATACAAAAGTTGTC
TTTCACTGTTTCTTCTTTCACTCTGTTTTCATTTTTTCAGTTTCTGTTGGCCTCCCCAACCAATTGTAAT
ACAACCGCCTACTCCTACAGGCTACAACGTCAATTCCAACAAACAAGGTAGGCATCATAATGTTAACCAA
CAGCCTTAAAAGGCATATAGACATAAAGCTTACTATCTTTCCTGCCAAACCATGGTGGTTAACGTTGGCA
CTTTTGCACTCAAAAAGGATTAACAAAGTTTTAGTGTTGGTTGTGGAACCCTGTAACTGATTGTAGGTAC
ACCACTCCCTACCTTCAAAGATCCAATTTTTTTATTTAAAAACAAAAAGAAGAAGAAGAATAAGAAGAAG
AACGTCCATCCTTCAAACAACTGGCACGGACTGGAAGTTAAAACAAATTAAAAATACTATGCTTGATATA
CAAACCATTTTGGATAGTAGCTCCTCACGGTATGGCCTATCATAACCCGTCACCCGAAGGAAATTTGCAT
AGAGTGGTTCATCTAGCACTTCCACATCATCTCTCTGAGGACAAAAACGGTGCATAATATATATGGAATG
AGCAAGAAACAAGTTTTTGTTGAAAAATTATTCAAATAAGCATTCAATGTTATACTAGTCATATCTGAAC
TTCTCCATATTCAATAATATAATAACATCTCCTAACTTTTCCTCTGCATTACAATGTTTCAGAGTTTTAC

AAATTCGTTAGTCCAATAAATGTACTTTCAATTAGTTAAAAGTGATTAAACTAGATGCATAGCTCGCCTG
TCCAAGAATTTTTTTTTTTTTTTTCCTTTTCGACAACAACTTTCATTGTAAAAAAATAAAAAAGTAGAAG
GATAAACAAAAAACCAAATCCACAAAAAAGGAGCTCAATTAGAGGGGGCTGCAGCTGCACAAAACAGTAC
CTGTAGAATAATTACAATCAAAATTAAAGCCTAGAGGGAAACATGAATACAACAATGGACCAAACATCAT
TTGGATCCCTCTCCAACCCTTGAAACACTCGGCTATTCCGTTCATCTCAAAGAACCCAAAATATAGTACA
CCTCAGCAAGCCACAAGAAAAAACCCTTCTCACTACAGGGGAAATGGAGGAGCACTCCCCGTTCTTATCA
AATAGCATTACATTAAGGAACAGTTAATATAATAATATTTGCTGCTGCCCAAAATTGCAGAAGAAGATAG
TTATAACATATTACTTCAACAATGCAAACAGGATTCAATGAGAAAAGAGAAACGGCAGAGGTTTTTGTCA
GACTGCCCAAAAAAAGTCATTTTTTCTTCCAAATCCTATCCCAGGTAGTAAAAGCAATATTAATCCTCAA
AATCTTTAAAAAAAAAAAAAAAAAAAAAAAAAAAAAAAAAAAGATGACTTTTGACTCGACAATGAAATTTC
CAATACAGTGCTAAATTTGTCCCAAGCAGAAAGCAACTATTACACAAAAGCAGAGAAACCCCATCAAATC
ATCACCTAGATAGACAATTGACCGCCATTGATGTTTTCATCCCCTTTTCCTTTCCATTTTTCAGCAACCC
AACAGAGTAAGCAAGCAATGGACTATCAGATTGAGAATTACTGAGTAAATCAAAAGCTAAATCATTGAGT
AACAGACAATTAATCACAATGGGTAAAGAGAAAAGCTATAATTCAGGAAAACCCATTAAAGAAAAATCAT
TACTCATACAAACAAAACAATGCAACAGCCACCAACACATAATTTTCACTACACTATAATCTCCATCTCT
TTTCCTCCTCATTTACTCAGCAACCGAACAGAGTAAACAGGAATGCTAGCTAGAGAAAGACTGAGAAAAC
TCAAATAGCACCTGGGCAAAAGAGTACATAAGGCTGGTACTAAGCGATCTCGGAGTGGACCATAAATGAA
TCACCTCCACCTCCCCCATAGCAAGACACAGAGCTCGAGAATCAAACTGCGTTGAGGCTGAGCTTAAAGA
CCCCAAAGTGGATAAAAAGGAAGACTGAAAGTTTGAAAAAGGAAAAAAAAAAAAAAGGAGAGAGAGAGATT
GAATGCGTATACGAAATGGGTATTATCTCTGTACAGTGCTTATTCTCGATGAATCCCCGTGTCCTCCACT
CAATTAATCCTCAACATTTCACAGAATCAGTGGTAAAGTAGAGAAAAAAAACCCATCAAAACTCAACTGA
AAAAGGAAGCAAATCAACACGAATGGCACGTAACACAAGGCAACAAATAGATGAAATGAAACCCACTACT
CAAAATCCCTTTTTTAATTTGTTCAGTTCATTTTATTCTCAAAGAAACCATGTTCTTGATTTTTTCTTTT
TTTTCTTTTTCCCTTCAATTCTTCATTTGTCTGTACATGTTGTCGCGAAGTTAATGAATAAAGATAAATT
TAAGATTTGAAGGTTACTCAACTCTCTTCACTAAATAGCTTAAAACACCTAGACAATAAAACCCTTATTT
AATTCATGTGTGAAGATAAATTTAATATAACAAATTATAGTCGTCATCTTGTTATCTGTTTATTTTATAT
TCACAATTTATATGATTTAACATGTAGGGCATACTTAACTCACAATTATTTAAAGTCATATTAAGTTGTT
TCCATTGAAAAAGGTTAATATAGTATGCTATTATTCGACGAAGTTAAAAAAGTTGTCATTTTTAAATACA
TGTATATGATCTTCTATATAGTAAAGCACTGAGTGATGGCAATGGCTCTTGAAAAAAGAGATAATATATA
AACATCCCGTTGAAACTTTTGTAAAGATTGTGGACCAAATTAAAAATAGAAAAAGTATATATATTAAAAC
TTTAGAAATTAAAATATAGATTTGTCACTTTGTGAACTGAAACTTTGTGACAGATAATGTGTGTTGAATG
AGATTGGAATAATAGATTGGCTTTGCATAATATATACCTTTTTCATTTGTAGTGATGGCAAATCAAACAC
AAACTTCCTTTTCAACCTATGCAATTCAATGTGCCACTCCTCTTTTTCATTGTCACATCCCATAAAAAGT
TTAGCAAAGTTACGCTAAGAATAATAGATTACATTTTTTTTTTTAATATCATTTGTCTTCGAGAATTATTA
CAAACTAACAGAGTGAAAAGTTTTTGTTTTTCTATTTTTAAAAATTTATTATATTTTCAATGTTTCGTTA
ATTTAGTATCTATTACTAGTTTATTGTTGATTTTTTCTTCAAAAATTTAACATTTTTACTATAATTTTTT
AAAACGTATTCATGTATTATAATTTAAAATAATTTTTCAAAAATTAATATCAATATCATTTAACAAAATT
TTAATAAATGGCAATTTATTAAAAATTAAAAATTAGACATTTAGAAATGCATAACTAAAATTAAACCAAC
TTAAAAGTATAAGAATCGAATTATATTTTAATACTTTTTTTTAAGAAATGCTAATTATATTATTTGATGG
ATAAGAAGCCTACAAAACATTTTTATTTTATCTTAAACATTCTAAATTGAATAACCGAACGACACCCTTT
TTTTCAACCATAATAGAAAAGAGGATTCGAATCACAAAGGTATTTGTCACTCACATATATTATATATTTG
TTGAGTTATTTTTCTTTTAAACAAATAAATACTAATATTTTAAATTGTCTTATGCACAATAGAAAATTTA
TATGTAAACACTTCCAACTATCTATAAAATTTAACAATGGAACCAAATCAATCTTCAAAGTTCAAACTAT
AACTCCCAGTATTGTTTCTTCTACAGTAGTCATTAAGAATGTAACTAAAAGAAAAAGCTACTTGAATTTC
AGAGTAAACAATCTGATACTTGATCTATTGTAAGTGTACATATAAGGTAGCAAGTTCATGGGTTTTAATT
CATCCGTTACATCACAGATACTAAGATTAGCCTGTTCTACTAGACTTAGAAAACGATTAAACGTGTCGAT
ATTCCGAATTACAGAAGCGATATATGCAATCGAGTGATCCCTTGAAGCTTTGAATCCATGGCTTCCATCC
TTTCCTTGATCATCGATATGTTCACTCACTGGAAAAGAGTGCGTGGAACTACCAATTTGCTTTGGGCGTA
AAAGAATGGAAAGAACACGTACAAGGTCCGGGAGACATATTGGATCATATATTACATCTGCTCCTAACCT
ACACAAGATGGATAGTTTTCAAGAGCATTTCATTTTTGTTAGCAGAAAATTGTTCTCTATTTGCTACGTT
AAGGAGAGTTACTTTACTTAAATCCAATTATACTTTATTTTGTATTAAAAAAATGGTATATCAAATCATA
AAGTAGAAACTCACACAATATGCGGTGCAAAAGCTTGGAGCTCTGTTTCTGATGTTGATTCCCATGGAAG
ATGTATGCATTCCACCTGAGATTCATAGTTCTCTCATTAAAAATTGCCACGAGAAAGCCAAAAGTCGGAG
ATGTACTTCTTTCAACACCTTGAATCTATTATTTGACAAACACGGAGACTCGCTAGATGATCAACAAGTC
GGAGTCCAAGAGTAATGTGCCCTAGTGGAGGTTTGGGGTAGCGTCCAATCTATGTTATTTGCGTTATTTA
CGACTATGAGTCATGACTCTAGGATTTAGAGAAGTAGAGGACTAGAGGTGCACTATGTAGACAACAGATA
TATGGTCCAACGTATTTATTTAACTAATTAATTTATTCAATATATATATATATACACATATTATTGTTCT
TTTCTTATTTTTATATTTACCAATTCTTCAGGCAGAGCGCCTAGGCGCTGGTTCGCATTGTGCGCCGTGC
TAAAAACAGTAGCTAGCTATTTTTTAGTGCAATTATAAAATCAGCACTTCGGCTTTTAGGTCACCAATTT
TTTAACAAACCCTCTTCTTCTTCTTCCGCCATTCTCTCCTTCCAATCCTCCAACCTCTTCCTAAAAGCTG

AACCTCAACCTTGGGTACCCCCTATAAAGTATGGAGGAAAGAAGACAAAATAAACACTTGCTAACATTTG
GGAGGCTCGCCAGACTGTTTTTGGTTAATGGAAATTGCTTGTGAAACTTTGAAAACTTACTTTAAAAAAA
TGATTTTCCCTCCTTTCCTTCATCCTTTCCCTGTTTCTATCTTATGCTCGCTATAGATTTATAGGAGAGG
CCCAAAGTTGAAGTTGAGCTTTTAGAAGGTTGGGAGAAAAGGAAGGAGAGAAATAGGAAGAAAAAGAACA
AAGGAAAGGGGTTGGTTACAGAATTGGTAAAAATAATAAAATAATGATAATAATAAATATACAAATAAAT
GAAAGTATAATTATAGATAATAAAATTATAAACCAAAATCACTAAAATAAAATAAAACTATATTGAATAA
ATTAATTGATTAAATAAATAAATATGTCGGACCAAAAACGGTGTATGCACACTATATAAACTCTTTAACC
CGAGAAGCACCTCTTTCAATATATAATTTGATATCTCTCTAATAACTAAACGGCTCTCTCGGCAGAGATG
TAACTGGTACACTATTAGTAAACCATGGGTAGATTTGCTCGTTTACTGTTTTCTTTATTTCAAAAACTTT
CAAACATTCTGTTCCCATTTCAAAATATAAGCATGTAAAGAAAGTAAGGGCTTCAGAGAACTAAAAGCAT
GTATGGAACTCCTAATAATAAGCACTTACAGTTTGAGTACCCTCATTAGTTCTTTCTGATGTAGCAGTTG
GAGAGCCGAGGCAGCACAACCCATTCAACCCTAAATTAACCTTCATATTTGCTAATGTTGATGGGTCACC
ATCACTTAGCACTATCTGTTTAAAGACAGCGCTGTCAACTTTGTGGTAAAGTTGAATGGGAAAATTTAAT
GATGCTACCCAATACGGTGCACGCTTAGAAGGTTTTGTCAAAATAAAAAGCAAAATTTATTGTACTAATC
AATCAATTTTTTCCAATATACCTTTGATGCTTTGACATGAGCAAGGCAGATTCCAACCAAACCTACACCT
GAACCTACCTGTTTTCAAGTAAAACATAGCAAGAAAAGTTTATGGAAATCTTCTAAGGTGTTTGAATCAA
AGGTTGGGTTTGTGTAACATAAGTTAGAAAATCGAACTCATGTTTGATATATTGGTTTACTTATCTAAGA
ATATGAATGTATTAACTGTATTGGATGCATATTCCAAATTATGAAACTCCTCATTTATTATCGTACTAAC
TTTAGCAAATCATAATCTCACAAACCCAAATTCTCAGAATAGCCATTGTAAACACAGAAAAATTCAACTG
ATGTTTGTCTCTTGTTTCTTTTTGTATATGAACCTTTGCCCTGTTGGTAGTCAGGTGGTCATGCAATCTG
TTTTCCTACTCATGGGTTTTGATCCCAGCTCTTATTTCTCTTACTTATAAATAGAAAAATATATATATGT
TTTTCCTAATAATGCATGATCTTTCGGAAAAAATATATATAATATATAATGTCTATATGAATAACAAGAA
AAATATTTGAGTATGATGACCAACCATCAAGGCATAAAAATTCCAAGGTTCAGACTGCAAAAGTAAGAAA
GACTACATTAAAGGCAGGAATAGATGTACTACTTTACCTCAAAGCATTCTTTTGTTGAGAACATATCTGG
AAATGAGAGTATCAATTCTGAAAGATATAGACTTGAAGGCCAGATTGAACACCTGTACCACACAAAGAAT
TACAGACTTGAAACAACTTTGCAAGATTATATTAACTAATATAACAGTTAAATAATTGTCATAGAGTTGT
AAACATTGCATTAGTTGAAAGCATGAAGAATTTTGCAGATACTCTTTATCAATATATGTGAAAAGCCTTT
GACTTACATTTAGACCAATCTTTTCTTCTTTTGGAAAAGCAAGCAATAGGCTTATATTTAGCTTCATATT
CAAAGCCCAAGATCCCATATTTTGATTAGAGAAAATTAGTCAATAGTTTATGTCCAGATGCTTAAATCAA
AGTTGAATTCATAATTTGCGTGTAGGATTAACTAGACATACATGTCATACAACGAACAATAAACAAATTA
CTGTTCTTTGATTAAGAGAATGTTTAAAATGAAAGCCTCGTCAGCAATCAAAAAAGGGGAATCTGGATAT
CAGCTCTAACAGAGGGGAAATGCGTGAAATCTGTTATCTTTAGCTGTTCCAACTATAAGCTATAAAACCA
AGTGCACATCAATCCAGATGATAAAGCAATGTTCCAAGTACATCAATGTATGTAGCACACACAGCCATTG
AAGAGAAAAATGCAAATCATATGAATTATGACATGTAATTGTAAAGATAAATCCTCCACAAATTATTACG
CTAATACATGATGAAAATAAGAAGACTTAAACAAAGTTGGTGAGTTGAAGCATTCCCTGTATCACCTTCA
AGCATGTTGAGAGAGCATTGCATTGGGACTACAAATTTCCTGAACTTTTGGCAATTCATAGAACCTACAA
GAACCAATTCAATGCAATATTTGTAAGCATGGAGGCAAAACCATACTATTGACTACTTCCGTCAAGCGAC
AATACAGAAATGAATAAAATAGTTACCATCTGGAAAAAGGAAAGAAACAAATTTGGAGATTCTTGCACTT
TCTTTCCTCTGATTCTCATCCTATCAGACAAGAATTTTTATCATTCAAGAGCAAAGAAAAAAAACAGCCA
AGGGACTATCATGTGCAAATGAATCGATGTAAAAGTTTTTCCTTCTTCAGCAGCTTACCTTCCATGAAAC
CATATAATGAGCATACAATTCATATAACTCGTCCAACACTTCAGCCTGACTTAACTCAACTTCAGATATT
AGTTTCTTCAGAAAATTCTTCAGATATGGTACATGAAAATGGTTCATTTCTTGCTGAACAAAGTATGTAG
GAAACATAGAAATAAGTAAACTCAAATACCAAATGTTGTTCCAATAGCAGAAAGCACTAGAATCCAACTC
AAAAATTGCCAAGAACATCTATCTACAATCTATCACTTGAGAACCAACAAATATTGAAATCCCAAGTGAA
CACTAAGCAAGCAGAGACTTTAAATTGAATTGAACTAAAACAATGAAACGGAAGTAAAAAGGTTTAGAAT
CACTACAGCTTTGGTAATGCAATGATCCCAAATGAACTTCTGCACTGTCTCGGTCACCAACCCGTGACCC
AAATCCCTAAAAGAGCCAAAAAGAAACATTATCCACCTCGATTTGAGAAACCCAGATAAATAAATCGAGA
AGAAATGGGTCATCCATTTCAAGTAATAGTAAGCGCGGTAGTATTGAAGTGGAACCTGGCAATGGAAAGT
AAGGAATCAGCTGGCTCCATGGAGAGGAAAGCAGAGAGTAAATGGAGACTAGGAGGAACAGAGCCGTCAA
TCCCGAGCTCCTCCGCCATGGTTGATGGTCGTGTTGCGCAAATAGTTTCAGCAGAAGTGATCAGTTGCAC
TGAAAACTGGAAAAGCGGCGTAGAAAACAAATCAACGGACCTTGGTTTGGATTAAGATAGAAAAAAAAAA
TTCTAACAACTCTCATTTTTGTTTAATCATATTCATGTGAAAATTTTTAAAATAATTCCAAACACTCATT
TTTTTTAGAAATAACTTACTTTTTAAATTAAATATTTATAAATGTAATCCAAACCTACTCCAAATTCAAA
CAAATCTAACTACGATTACAATACAAAACTTATTTGTCTAGACATTTTTCATTCAAATATTCTTTATCAG
ATTCTTTGATATCACGAACTAGGGTGTAAACAAGTTGGGTTCCCAACCTAACCCATATTTTTGGATGGGT
CGAGTTGCCAACCCGAATTAAGTTTCCAACTCAATCCATCAAATATGGATTGGGTTTGGTTGTCGGGTTA
TATATATTTTTTTTTCGTTTCTAAATTTTAATATTTGTACATGAAAATCATCAAGTATAAATTAAATATG
TTTCATTAAGTAAAATAAAACTCCTATATATTATAAATATTAATTCGAGTTGAGTTGGGTTGACTCGAAT
TTTTTAACCCTCTAGTCAAAGACCCAACTAAAAAAATCAAAATTTTCAACTCAACCCAACCCACATCTTA
AACTAACCCAACTAACCCATATAATATAGATTGATTAGTCTAAGTTGTCGGGTTACTTGGTTTATTCTTA

TACTCCTTATATCACAAACTTATTTTCTTCTTATATTCGACTACTCATGTTTTTCAATACTCTCTCATCT
CATCTCTTCAAGTAATTCCTATTTTAGATTTAATCATTCAAAACTAATTTTTATTCAATGAATAATTTTG
TTAGAAAATTTAATAAAAACTTATTTTTTTTAGAAAATATTATTAGTAATTTAATATGAACAAAAGAAAC
ATCACCCAACTCTTATCCATAATAATTGGCCTTAGATGAAAATGCGACTTCTATAAGATCCTTAAGAACG
ATTGAACAAAAGGTTAACAAACTAGTCTGATACCACAAACAACCGGCCAACATTGAGAGAGGTAATAATG
TCGAAGAGTCCTTCATAAATAGCCAATACTTCAAGGACCTTGTATTGTTCCATATTTTGATAAACTTCAG
AATAACTAGACCAACTCCACCTAAATGGTCTCAGAGCATCCAATCAAGATCATTATCTTAATGGCCAGGG
CTCCAAAACAACTTCGATGTTCAACTTCCACTGGGAGGCGGATACCAACCAACTGGAAAAAGAAGAGGGG
GGCTAACTAGACCCTCAAACAACTCTATAAGCATATGTCACATCACAAATTTAATTTAATGTTCATGGAT
ACACCTCTCAATAGATACATATATAAATTCAACATGAGTAGAAATTCTATAAAACATCGAATTGTTTTGG
ACCCCAAATCTTTCACTCAATAATCACCGCTAAGTCGAACTCTTAAAGCAAGATATGATTAATGATCCAA
CCCCATCCAGTCATCACAAATAGAGCCCACATTAGTACTTAGAAATTAGAGCCAAATATCTCTTGTCATC
CAACATCTCCACCTAAGATAAGAGGAAAATTGTGTCTGGTCCTAGGAAACACACAAAACATTAGAATGAA
TTTTTTTTTTTAACCAAATTAGACTTAGTTGGAACAATATCATTAATGATCTTCCATTGTAGTTCTTGAC
AAAACAATATCATGAATGTCAAGAAATTCATCGACAAAATTTGAATTTCGTGTCTAAGAATTATAGGCAA
ATGAAATTTAGATCAATAACATATTAAATATAAAATTAAAAACTTAAAGACCAACAAGACATTTTCTTTT
AGACTGAAAATAGAGGAATTAAAAATTTCATATAACGAACGTGAGCATAATTCAACTATTATTATTTTTT
TTTAACTCAGCTACTACAGAAACACGAACTATAGATCAAAATGATAAAGGGTCCAAATTCTCTCATTCTC
CAACTATCGTACTAGAAAGTTTGCTTGTTATTAAATCAAGCAGTGACAATATTATGCTACAATAAATGGA
ATTTCGTAATCCCTCAAATTGGATGATTAAGGTAAAATAGAGGCCTATCATGTGATCGAATCTTTGGACT
TGAACATATCTTACAAAGAGAGGTATTGTGTGGTAAAGTGATACTAAAATAGTAGTACTTTTGCAACCAC
AAGGCAATAATGCCCATAGATTGGGGAAAAATGACCTAAATGATTCAATCATTGGACCTAATTTTCTTCC
AATGTAGAACTAAATTTTAAAAAATTGTCTTTAATTACTAAGATTTTCTTTCCACCTGTGAAGTTCAAAA
AAAAAAAAGTTTACCCAAAAAATATGGTGGTAGATCACCAATTAAATTAGGTGTTTATTATTTTTAGTAA
CACCACACAATAATATATAATATATAGCATACCACTTTAAATTTGTCATATGAAATCCTCTTATTTTAGA
AAATTTGTAATATTTTTTTTTTATTTTAGAGATTTTTTTTTTCTTTTTCTTTTTCTTCCATTTTAAGACT
TCTTAAAAATTGAAACAAAAATATTGTCCTATATTTATGAAAATGGAAGAGAAAGAAATTGGAACTTGGA
ATGGAAGGAAATTTTTGAACAACTATTTTGCTTGCAAAAATGTCATTTTTCTTTTCTTTGGCAAAAGTTT
GATATCAAACTTTAACTTTAATTGGACACCCCTTGAACGTAAGATTTTAGCTACATTTTCTTTTTTCTTG
TCATACTCAACTTTGATTTACTACCTATATTTTTGTAAGGACTTCATCACTTATATACCCGTTGCTTAGC
TAGGTTGGTAAAACAATCTTAGATCGTATTATTGAGAGTAACTAAGGTGAACACGATCGATCAAAAAGCT
TAGCCAATCGATCAAAACTAATCGAATTGAAGGTGGTTGGTTGGAGATAAGGAGAGGTTCACTTGGTGTC
AGTTTAGGAAAAATCAAAACTAACAACATTTTTTTTTTTAATTTCAAAGGCTTAAACCAATAGATCAACTG
AGCAAAGGTCAGTTTGCACTTGTTCGTGGCTGAGGATGATATGGTCGAAAAACCAACTTCAATCAACCGA
TGCTCATTCCTAAGAGTAACAAAAATTTAACATACAAAAAATTAGTTGTCCTTCTCTAGTTGAAATTGAA
ATATAAGACATTTTCAAATATATAGCAAAATGAACCAAAATAATTACGAAATATAGGATTCTATCAATAA
TAGACGCAGATAAACTTTTATTTTTTTTATCAAAGTTTTTATTCGCGATATTAATAAAAGCCGACAAAAT
GTCTATTTCATTTATAGAGTCTAAAATTTTGTTATATTTTACTCTACTTTTAGCAGTTTTATCATTTACA
ATGATTTACTCCCTTAAAACGTTACTTCAAAGAATATGGGCATTAGTGTTAATTAATACCCAAAAGTAAC
ATCAACTTTTAAACTTTCTTTTTGTAAAGGTTTTGGATTAATACATAGAGTGGATAAATAAAACCTCTGA
GTTTGGTGTTATGTTACATTTTTGAAAGTTTCCCATAAATAATGAAGGCCGGCTTTTTCAAAGATAGAAA
AATTTGACAAACTATTTATATTACATAAAACAAAACTACTAAAAGACAACAAAGTTTACACTTTTTCTAT
GAAATGTAAATATTTTGTCAAGTAAATATTTTGTCAAATGTTTTATTTTTTTTTTTATAATTTTTTGAAAA
GGAAAAATATTTACAAAGATTTAGGAAAAAACTTAAAAGAATATAGGAAGATAGATAAATTTAGCTACAT
TCAATAAATAGTTTCAATATTTTGCTATTTTTTAAAATATCCCTTTAAAGTATATAATAAGTTGTGATCT
TTGAAATTTTAAATGATTAAATAATATAAATTTTTAATTAAGTGTTTAATATAATATTCGCGTGGTGGTA
AGCTAGCCGATCTGTTAAATATAAATTTAAATTTTTGTATACATTAATATCCAACACATGTATTTCTGTA
CTGATAACAACTATTAGTGATAATAACTCATAGCAATTATCGATGGTAATGATTAATAGCACACATTTCT
TAAATTAGAAGTTATCACTGATAAAAGTTACAGATGAATATTCGTTAATAGCAGCTATCAATAATAAGAA
CTGATATTTTTTTTAATTAAATGTTACCAACAACAACATCACTGGTAGCGGTTATCACTTGTAGCAATTA
ACAACACATTTTTCAAATTTAAAGTTATAACAACTTTGGGTGATATTCATTGATATCAATAAATAATAAT
TATCATTTTTTGTGCTAAACATGATATTTTTATGTTATACTAGTAACAACACGTGCACGTCAAATGCAAG
GTTTTATTAAAAATTTAAAATATACCCTTAAAAAAAAGTTATAATATTTATTATATATATATATATATAT
AACCTACTTTTATTTTTATAAACTTGTTTAAAGTTAAAAAAAAATATCATAGAAATTGTGTTAATAAATT
ATTTGTTTAATACACATATTAAGTATTGTAATCTACACACAATATATCAAATATTTTTAATGATATTTTC
AAATATATATATTATATTTTTCGTTATGTTCTTATTAAAGTATATTTTTTCATAAAATATTTTTTACATA
GTACAAATACAAAGTAAAGTAAATCATTAGAACAATAAATTTTTAAATGGAAAAAAGTTTGTTGGAAGTA
TGTGTGTTTTTTACATATTGCTAATAATCTTTTTTTTTACTCATAATCATAAGTTAATAATTATCTTTTTT
TTTTTAAGACAACCCATGATTATGAAGTACAATAAAAATAAATCTTAGAATCTCCTCTTCATTATTCATG
TGATAAAACAAGTGTAAAGACAATTTTCCAATCAAACCTCCGCTATATATGTAGTCAAAATGCAATTACT

GTGAGTAGCAATTAATTAGCATCACTATGACATAAATCTTGTGCTCGGCGCTCGCTATTTGTTGAATAGA
AATTTTAAATAAGCTATTCTTGAATTGCTCAAATAATTGTTGTGTATACTTACCTTAATCAACAATACTC
CAATACGCAAATCTCTATTAATTTTGTTAGCTAGTGATTTTTGGTACACCATTCCTCTATTTTGGTTTTT
GTCTCAATCGATTGGTTTCTACGTGACTTGATGCGCCAATGGTGTTGCCTCAAACATTCTCATATTTCTA
TATCGTTGTCCATCATCTCAAGCTTGAGAGAATTATATTTGTCAATTAGCATAATGAGCTTTATATAGTG
AATTAAATGTGTAGAATGAGAATAGATCGTAGATGCAAAAATTTATTATTGGTGTAAATTGCCTAAACTA
ATGTAGTTAGTAGATGGTTTTGTAGTATTTGGTCTATAAGGTCATTTTTTTCGGGATTATTGCCACCCTT
TGGAGTATTTGGTATGAGAGAAACAATAGAATTTTTGGTACTTTTAGTTATCAAAAATATATCGTAAATT
TGTGGGAAGAGTGTAGAATTCTCATAAGCAACTGGTGCAGCAGAGATCCTTTTTTTAAAAGCTATTCGGC
TGCAACAATTGCTTTAAATCTGAACGCTTTTTGTAATTAGTTGTTGGGCTTTTCTCTAGTCCTTCTTAAT
AAATTTGTTGTGCTAGGCTTGGTCTTAGGCTTCTTCCGAAAGTTCTAAGTCTCATGCTTATGTCGTTTCT
CTTTAAAAAGAAGTAGATGGTTTTGTAACAATAAAAAATAATGAAAAGTCATTTTCTAAAAGAATGAATG
AAAATTTTAAAAGCTAAAAAGGTTTTTGAAATTATAAAAAAAAGTGTAACATCTCATTAATGTATTTAGT
GTTAATTTGTTTTAAATGTAATTAATATTTAGATATTAATTGTTAATTAATTTAATAAGTGTCTTCACAA
TTTTTTTTAATAAATTTTAATGTTATAGTTAGAATTACTGAGTAACGTTGAAAGAAAAAGTTTCTCTATA
GATTCTTTTATATATAGTATAGATATGGATATGTAATTATTGTATCATTGTACTATATATTTTATTGTTT
TTTTTAAATTATCATTTATGCATCCGATCGTTAGAGGATTTAAAAAAAATAGAACAATTGACAAAATAAT
AGAACAAAACTAGTAAAAATAAAATTAATTACGCTTTATTTTTCTATAAAATATAAATATTTCATGAAAT
GTTATATTTTTTACTAACTCCCTCAAAACAAATTATAACAACAACTAATTTTTGCTCTGCTTTAAAACCG
CCATTCAAATCTTGGCGATGGATGAAAGTTAGTGTGTGTGTGTGTGTGTGAATTTAGATTTAGTGGAAGA
GAAAGCTTTGCTTCAACCTTTCATTTTACCTTCCCTTAAATTTAACCCAATAATCCTCCTTCTCCGCCGA
TAAAATCCAGCCGGAATTATACTTTTAGACTCATCATATCATATTCTGACTCTGTTTCTCAATTTAGGAT
CGGCCATGGCGATTTCTTTAACCAAATTCTTCTTCGCCATTCCATTGATAATGCTCACAATGTGTTCGAC
GATTTTCCGGTCTGGAAGTTCGGCTGGATTCGATTATCCGGCGGTGTTCAACTTCGGCGATTCAAATTCC
GATACCGGCGAACTCCTCGCCGGCAAAGGGTTCAGTCTCCTGTTGCCTTATGGAGAAACGTACTTCCAAA
GTCCTTCTTCTGGAAGGTTCTGCAATGGCCGCCTCATCATTGATTTTCTCAGTAAAGCTTCCTTCTTCCT
TTTCCTCCATTTTCCGATACCAATTTTAGAACATTTTTAAATTGTTTAGAGACCTATAAGATTATGTTTA
TTTATAAAAATTTATTTTAAATGATAAAACTTCTGCATTACATATAGAAACACTAATAATAATATATTTG
TAAACATTGCCGTAAATTACGAAGTTTTATTACATTTTATAAATTTTTAGATTTATTATATTATATTCGA
AAACAATCATTTGTTTATAAATTTTTTATTTTTGTGATTTTTTCAATCTATTCACCGATATCGTACCTTT
TAAATATTTGGGTTTAGTCGATTTAAGCATGAAAAATATTGAAGTATTAATTTTACAAAAATGTTCGTCG
TCATCCATACAAATATTTTTAAAAGAATTATTTAGAAAAGGTTTAAAATATATAAATAAATGTGCTATAT
TTTTTAAACAATGTTAATATGTTTATTATGGTAGAATTATTGTAAATAGAAAAAAAAGTAAAACAACAAA
ATTTATCCGATGAGAAATTTATTGATTTTATTGTTGTTCGTAAATAATTTCAATTTATTTATTATATTTA
AAAATATCCCTCTCTTATTTATACTATTATATTTACATTAATTTATTATATATTAAGTACAATAATAAAA
TATTTATTTAATTTTATGAGGTCGAACTTTGGAAGATACGAAAATCTTAGTATTAATGAAAATAATGACA
TATCCTCCTAAATTTAATATCATTGGATTAACCAAACTTTTTGTGATTTTACTTTGATCTTTCCTTTAAA
AAACCATTTAATTTGATCATTCTTTCTATTAAATTACTAGCTAAAATAAATCATTTACAAACGTTTCAAA
GCTACGATTAAACTAAAATTTTGTTAAAATGTTTGGATTGGATGAAAACTTAGGCATGTTGAAATAGATG
TAACGCAATATGCATACATACCAATGTTGTTATTCAATTTTCATCTAACGTTATATCAGAATAGTTCAAA
TATAGATTAAATTATGTCACTAAATACTATATTTCAACGGATAATTATGTAGTTCAAATCTCTTACATTT
GCTCTATATGAAAAAAAAGTAAAGTAATAATCCAACATTAATTTTGAATAGGGTAAATTAGATGATACAT
GTACTTTTTTTTTTCAACAATAAATGACACTTCTCGAAGAAGAAACAAAACTTGAGAGTGAGAGTTATTA
TTATTGTTATCATCATCATTATGATTACAATTATAATAAGAGTAATTATTATTATCATCATCATTATTAT
TGTTTTGTATAATTTAGGTTAAATGGAATGAAATATATGCTGAAATACAGTAGTTTACTTGGCCATTATT
ATGTTAATTTTGATGATTGAAAATGGGTGTAGTGGAAGCAACGGGGCTTCCATATTTGAGGGCGTATTTG
GACTCAGTGGGGAGGCCGAGTTTTAGAAAAGGATGCAATTACGCAGCTGGTGGTTCCACTATTCTTCCCG
GAACGGCTGCATCTATTAGCCCTTTTTCCTTTGGGGTTCAGATCAACCAGTTCATCCATTTCAAATCTAG
GGTTCTTCAACTTCGAGCCCAAGGTATATAAATTGAATCTTCCTTTGTTTGGTAATTATTTCGTTCTTTT
AGCAGCTGTTAGAAAATTATTAAGCCCGAAAACGTACGTTCCATTTCTAAGTTTATAATTTTATTATCTA
ATTTCTATTATTATGCTTTAAAAAAATTTTAAAAATTTAACCAAGTTTTTTTTGTTTTGGAATTTGACTA
AAAAGTTTAATTCATTTATTTAAATTGAGAAGAAATGGGTTTATTTTTTCAAATGTCAAAAATAAAATAT
CATATACTTACCAAACCATCCCATCGTCGTTTATTTATTTTTTATTTTAGTTTCTCAAAATATAATGAAG
CTATCTTATATAAAATTAAAATTTTGTTTTCTATAAAATCTACAACTCAACCTTGAACTTTGTGTGTAAG
TTTGAAGGAAAAAAAATGTCAAATATGGACATATTGACTATTTATGTTATGTGTTCTATTCGAGAGTTAT
TCAATTTTTAGTCTTTAGTTTTTTAAGTTAAGTTTATAAATAATATCTTTACTATTTTTTTGTTTTCTAG
TCTAATTTTTTAAGGGTTTTCAAAGTTCAAATGTATGTCTTGAAATAATGAATAGTTTTTTAAAAACTTG
AAATTGGCTATAAATATTTCAAATGTTTTCAGAGAGAAAAAAAAAATGATGAAAACCATTGAACAAAAGT
TCAGAAATATAACAAAAATTTTGAAGGGTAAACTATTCAAAATGACAAAACTTATTATCAACATAGATCG
ACAAAACACATTTTTCAGCCTTTGATTAAAGAACAAAAGGGAGAATTATAACAAAACAATTATACCCAAA

CACAAATAATCTTTTCTTATTTTCAATTTTTTTATATCAAAACAAAAAGAAAAGAAACAAAAAGTTCAAA
CATATACAAAATCTTATAATATAATGTTTGTATTTCATGTCAGGTGATAAGAAAATTGGGAAGTTTCTAC
CAGTAGAAAAATACTTTAAGGATGGAGTTTACATGTTTGATATTGGCCAAAATGACCTAACAGCAGCATT
TTACTCTAAAGCTTCTATGGATCAAGCAATTCCTACCATTTTAACTGAGTTTGAGATTGGTCTTCAGGTA
AGTACTTCACTATTTTCTCTTTTTAATTCGACAACAAGCTAATTTGTAAACCATATTCTTACCTATATTT
TTTAGAAACTGTATGAGCAAGGAGCAAGAAATTTTTGGATCCACAACACTGGCCCTCTAGGCTGCTTGGC
TCAAAATATTGCTACATTTGGATCTGACCCATCAAAGCTCGATGAATTTGGCTGCCTCACTTCACACAAC
CAAGCTGCCAAACTCTTCAATTCCCAACTTCACGATCTCTCTAAAAAACTACAATCCCAATATGTTGATG
CCACCGTCACGTATGTCGATATCTACACGATAAAATTCAACCTCATTGCCAATTATTCTCAATTAGGTCA
GTCGGACTAATTTAAGATCTGTTAGCTAGGAATGATTTTTAAGAAATATATGTCGACGTACGTTATTATG
ATCCTATCTTGGTCGTTGTGATTTGAATTACGTTATTGTGGTAGGTTTTGAACAACCTATTATGACTTGT
TGTGGCTTTGGAGGTCCTCCGCTAAACTACGACAGTCGACTCAGTTGCGGGCTAACGAAGACGTTGAACG
GGACGGTGGTGACGGCGAATGGGTGCGACGATAGCTCCAAGTATGTGAATTGGGATGGAGTTCATTACAC
AGAAGCTGCAAATGAGTATGTTTCATCACAAATACTCACTGGAAAATATTGTGATCCACCCTTCTCATCA
GTTCATCAAAAAATGCCTTTCTTTTTCAAGTACTTCAAGTTCTAGTAATTTTGGTATGCAATTTGGATTA
GATGGATACAACTTCATTTCTATCATACATCGTTATCATAACCTTGTTTGTATTATATATAAATATATAT
GGTAAGAATAATTCAATTGAGGATTTGAAAAACTATGATCTCTCACTATATTGTGGTGGTGTATGATCAA
TTTTATTAGTGACATGTCCAATATATACATGTTTTGGGAGTGATGATATTTTATGTACTTAAGACCAAGG
TCTTAAATGTGGATAATAATTTATTTAATTTTAACACATAGTAATTACACTCAAATAAAATATTTTTTGG
TTTTGAGAAGGAGAAGTTAATGTTTAATATCTTAAAAGTTATTTAGTAAGTATCATTGAAGTTCTTAATT
TTAATTTTTTTTTTTTATTTGCTTGAAGTAATGATGTTTAATGGGTCAAAAGTAAGTTTAGTCAAACGAAA
ATTGATATATGATCTTACATTTTAGAACCTTCATCCCTATAAACAAAGAAAAACTATATTTGATATGTTT
GATAAAAGATTAGGTCAAAATGAGACTTTGATGATATTGCCCCTATGCACTAACTGGTATTCAACCACGA
AAAAGGTTAAACAACCTGTAAATATTAGGTAAACAATACATACGTAAATCTATATGGGTTGTTTTCAAAT
ATGAAAAAATAAACCAAAATATTTACAAATAAATATAACAAAATTTTACTGATGATCGTATGTTGTATTT
GTAAATATTTTCAACAATTTTGTTATTTAATATAATTACCGTTAAAATTTTTAAAATTTTATTTATTGAA
TATTTTCTTTTAAATTTTTAGTTTTGTTAATTTTTTTAAAAATATTCATCGAGAACATTTTCGTATGACT
ACAAAATACATTCCAATTTTTTCCTTTCAAACTCAATTTCTGGTATATCAAATTTTTTTAGTTCGTTATG
AAATCTCTCTTAAATTAAGGAAAAAAATGATTTTTTCAAAAATAGAAAAAAAAAATTAACAAACTATTAA
TGCTCCACTAACCAAAATCCCTAAAAAATAAAATTCATTACATTTAATTTTTATATAAAGTTTAAATATT
TAAAAAGCATATTATATACCATATAGTGTGTTTTCATGTTAACACACATTTTTCTTTCCCTTTCTTTCTA
CGGTCCTTAAATTATAGCAAAATTTCACAACTTTCCCATTAAGATTCCCAATTAAGAATGTGTAAATTAC
AATTAAAATTTTGTATTTTAATGGAAAATGAAAAAAGAAAAGAAAAGAAAATTAATTTTCGAACAACCCA
CAATTTTATTAATTGCAAAATCATTTTTTTCTCTCCTTAGCAAAACTTTCTTCTATTTTTGAAAATTTTG
TTTAAAATTTCAACTTCAATATTGTCCTTATATATATATATATATATATATATATTGTAATTGCAAAAAGGA
CTCTCCCATCAAATTTAGTTTCATTTTAATTATTTGTCTTTTTTTTTTTTTTTTTATATAAATTTTTACTA
CCTAGAAACTTTTGCAATAAATTTACATTTTAGCCCCTTATAACTTTATTGGCAAGGATTAATTAAAACT
CAATGTAACAAAATTAGATATGTTTATGCACTAGAAATGACAATATAAATCTAATTATTGACCAAAGTAA
TGGCTTAAAGTTAAAATATCTAATCCAAATATTTGTTTACCAACTTTTGCCACATTATTTTACCTAATTG
TTTCAACTTATTAATGTGTCTAACTACCCTTAGCCTTGCTATTTTAACTTGAATGATTAGAGATGTAAAT
GTAGCTTAATCTCTAAATTGATACTAATTATATTATTCCATCTTTTGAAAGGAATCTTAAACATAAGTGA
AAGATAACAAAAAAAAAATTGGGTGATAAAAAAGTAATTAATACCATATGCCAAATCCAACAACATTCTTT
TTAAAGTTAAGTTACACCGGAATAACATAAATCCAATGTTTCGTTTGTTAACAATATTGTTTTAATTAAT
TTTATGTATTTAAAATTAATATTTATTCTCTTTAATTCAGTTTTATTTTGGAATTTTTTTTTCATAAATA
TAATAAAACATTAAAATATTTACTGTCCATGTGTAATAAAAATCAAAAAGCCTATAGATATCTTTCCCTT
TCGCCTTTTTTCTTCTGCAATTGTTCTTTTCCTTTCCATCGTTTTTTTTGTGTTCTTCTTCTACAATTTT
TCGTTTACTCTTTTATATGGTTCAAGAAGATCCACTTTTCTTTTATTTATTTTTTAAATGGTTATTTAGT
TGTTCAAGATTGTGTACCAAATATAAAAGATTTTCGCATTAACGAGGCATTTTGGTATTTTCTATTGTGG
GTTTACGGACATTTTACTTTTAGTAAATTGTTAAGTGTAAATATTTTGTTGCTTTTATTATATTTTTCAA
AAAAACTTTTTTTTGTTATGATTTTCACCTATGTAAGAGAAAATGCTTGAATTTCTTATCAAATTTTAAA
AATAAAAAAATAAGTTTTTGAATATTAAGACTGCGTTTGGTACTATTTTCTTTTTTATTTTTGAGATTTAT
GTTTTGTTCCACTCAATTACTTTCTTATGATTTTCATATTTTCTACATGAACATTTGAATTCGGTCGTAG
TCAATTATCCAAACACAAACGAAAAATTCTTTCGTTTCATTTCTTTTTTTTTTTCTTTACTTTGAAAAGA
CTCGTAAAATGTAAACAACAAAACAGAAGGACATGATGTTATGAATTTAAATTTTAAAAACTAAATACCA
AATACTTAACAAAACGCGATAATGCATTATGGGATAAGGATTTGGTTGAGGGTTTGTATGGATAGAATTA
GATGATAAAGTAAAAACGTATTAGGGAATGAATATTGTTAAAAAAAGTAAACTTAATTTATAATAACAAT
AAACTAAGAATTAAATTAAATAGTTATGTATACAAAAGAATAAAAAGGTTAAAAAATCAACATTGAAATC
CAAGTTAGTTAGTTTTTGCTGGCTTAATTAAACAAACAATTTCCATATTTCTTCATGCAAAAATGTTATT
TGAAGTAAATTAAGTGTATAGATTGAAGAGAAAGAGAAATCTTAGCTTCAATCCTTCACATTGCCTTCCC
TTCAATTAAACCCCTAACCCCCTCTTTCTCCCCCGATAAATTCCCCCCCGCCGGAAAACCATTTTCTCAT

CGTCGGAAAATCATCGAAATCCTCCGGTACTCTTTTTCTCAAGAATCGGCTTTTCTTCCAATATATATAT
ATGGCCATTTCTTTCACCAAATCCTTCACGATTCCGCTGATGCTCACAATCTGTTCGACGATTCTCCGGT
TCGGACGTTCGTACGATTATCCTGCGGCCTTCAACTTCGGGGATTCAAATTCCGATACCGGGGAACTCGT
CGCCGCCCAGGGGTTTAGCCTGAGCTTGCCGTATGGGCAAAGTTACTTCAATACTCCTTCGTCTGGAAGA
TTCTCCAATGGCCGCCTCATCGTTGATTTTATAAGTAAAATTTCCAACACTCCATATTCCCTCTTCGCTT
TTCATGTTTCTAATAATTTTTGGTGTCCATAATTAATCACAAACCTCTATAAACTAATATCAGCTAACAT
TCGAATGTACTAATAACATTTTTTCTTAACTCTTGGTTGTTGTATTAAAATTAAAAAATAAATCGATTTA
GGATTTTTAATCTCTGTTTTTTTGAATCGATACATTTGGTTTTCTAGTTTTCAATTTGTACATTTTTAGT
CTATAGGGGATTTTGAGAATAGTTTGGTTTATAAAGTTTGATGAAGTAATTTTCTTTGGCTAATTTAAAT
AATTATATGAAACTTTGGATTTAAAAACCTTCTAAAAATTAGATCTCGAAATATTTATAATTTTACTTTT
TAATAATTTAAAATAATAAAAAGTTACCCAATGAATCTTTTAGACTTGTTTCTAAAAATCGGGTACTAAA
AATGTACGTTTGGAAATTTGAGGACCAAATAACACACGTATTATTTGAAAACTATGGACTAATTTTTTTT
TTTTTTTTTTTAAAATTAGTTTTCAAATTTAATCTTAAAGAAAAAAAAAAAAAAGAACAATTAACCAAAAA
GTATTAGGTTAATTATTTGTGTTAAGATAAACATAATTCAATTTCAAAATTGTTTAAGAAATTTCCATGC
ATCACCTAAGATATGGTAATTAAAATTCTTCTCTATTATACTCAATGTTGCATGAAATTTCAATCTTTGT
GTAAACAACATGATTTCACACCTTAATTTAAAATCACATTTATTATAATCCAAAAGTTCCCTCTAAAATA
TATATTTAAAAATTATGCATATTAAATTAACTTTTAGGGGAAAAAAATAAAATTTGAAATTGTTCATTGT
TTCTCTAATAAAAAATGTTTCCAAATATATTTTTAGAAAACAATTTAAAAACAAATGTGGAATTGAAATG
TTCTTATCTTCAAAATTAAATTGATTTCTTAATTTAATTAAATTCTATATGTTTTATAGATGACATTTTA
AGATTTGTTTCATCTTAGTTTCTAAGCTTTACGAATATATATATACACACACACACACAAATATACAAAT
TTTGTGGACTACAATAAAATAAATAAATAATTTAAACATAAAATTTTAAAATTAAAGGTAATTAGAAAAC
AATTTTTTATACTAATTTTGTTATTTTGAACAATTTAGCTCATGAGGGAAAAATAATATTGTGTGTTTTT
ATTTAATTGTAGCAACTTAAGTTTGGTTATTCATAAAGACATTTTTTTGTTTCTAATTAATTTACTCATC
TATACAAGTGAGAAAAAGTTCATTAAATTTAATTAATGAACCATTATTTTCATTGTAGGAATTAAAATAG
TTACAAGTTAAAAAATTTATATAGAAACTAAAATTTAAAGACCAAATGTCCCATGAAAGAGAACTAAATT
GTTACGATACTAAAATAAAAACAAAGTACATTTTTGGTATTTAAAGTTTGAGTTTGATGTATTTTTGATA
TTTTTAAGGATATAAAAGTAACGCGATCTTTTTATTAGTTAACTAATAGTTTAATTAAAGTTTAGGCTAA
GTCATTTTGAAAATTTGGGGATCAAATAGATATCAATCTCAAACCTCAACAACCAAAAGTGTAATTTATC
CATATAAATTTAATTATATGACTAAATCATTACATCTACAAAAGTTTAGATCTTGATTAGTAACTATTTA
GTTTTTTTTAGTTTTAGAAATTAAATCTATTTTCTCTCCTTTTTTTTTTTTTGCAATAGTTTTTTATATT
TTAAAAGTAAAACCATTTTTTACAATTTTTTTAAAAAGAGTTGCATTCTCGGTCAAATTTAAAAAATAAA
AACAAGTTTTTAAAAGGGATCTTTTTCAAAAATAAAAAAAAATTGACAAAATTCGTTACACCTGATTTTT
ATATAAAGTGTAAATATTTTATCAAATGTTCTATTTTTGATAAATTCTATTTTTAAAAACTAATTTTCTT
TTAACTTTTGAAGCTTAACTAATGGGATGCTTGGAAAAGTAGTAAAAAATTTTATAATAATAGGACCTAC
GTCACTACATTTTTTAAATTGCTAAAATAGTAAATATAAAAGCAGATAGTTTTATGATAGCCTTTTGATA
ATATAATCATATGATATATTTAATTACTTAGTCATATTTACTATATAAAAAAAAGTAATGGTATGAGTTG
CTTTTAATCAAAAAATTTGAAAACTTTAGAGTATTTGGCGATGAAAATAAACTTTGTTAAAACATGATTT
TTTTCTAAGTAAAAAATCAAACAAACCGAGGAGTAGAGATAAATTTATTCCTTTCCACATCTCTACTTTG
TTGAGTTGAAAAACAAGAATGAATGCTCCAACACATACACAAAAATCCTCCTATGTTAGTGCGGGTCCCG
TGTTTATTGAATCGCTCTATCCATCCAAAATCACAAGTCACAGCAAATTCCTACGCTTCAAAAGCCTACC
CCTTTGCCTTTGCTTTGTCCCTTCCTTTTCCTTCCTTCGTCTTTTTGCTTACTGCTCACTGAACTGACCC
ACCATCACCAACCATTCCTTCAACGCCATCACTTTTCTTCTCCCATTTCTGTTTTCTCATCACTTCCCTC
GTATAAATCCAACCCCCACATCAATGCCGACCCCAAATCCTCCTCCCTTACACTGCCGGCCATGTCCACA
CTCACCCACATCCTCACCTTCTTCCTCCTCTGCTTTTCCTTTGCCAGATCCAACCCTTTTTCTCGTCCGG
CGGTTTTCAACTTCGGAGACTCCAACTCCGACACTGGCTGCCTTGTGGGTGCCGCCATTGAGAGCATCAA
CCCCCCTTATGGCCACCGTTTCTTCGGACACCCCTCTGGGAGATACTGCGATGGCCGTCTCGTTGTTGAT
TTTCTCTGTGATTTTTTGTTTCTCTAACACTCTCTTCAGTTTTCGAATCTGCGATGGCATATTTTCTCCC
TTTTTTGTTTTCCTGCTTTTGGTTCCTGAGTTTCTCTGGAATTTCTGTTTAGTGGTTGATTTTCTATCAA
ATTTTGTATTTTTGCGAGTTGGGTTTTCGGTTTTTGAATCTGCAATCTCGTATTTCCATTGCTTTAGATT
CTCGTTGATTTTCTCTGTTGTTTTCACTTCTTAACTCTTCCATCTGCCATTTCCCCTGTTTTTCCCCTCC
TCTGTTTTATGTTTGAGAACGTTGTTGTTTTCATCAGTAATTTTCTGTCTTTTTAAGTTTTCCACCACTG
TTTTCAATGCTCGAGGTTCTTGTGCTTTTTCTACAATCGCGTGTTAACATTTCTATTTTTTGCGCTCTTT
TTTTCTGTAGTGGATGCCATGGATATGCCTTTTTTGAATGCTTATCTTGATTCCATTGGGGCGCCAAATT
TTCGGAAGGGATGCAATTACGCAGCAGCAGGCTCGACTGTTCTTCCAGCAACTGCCACCTCTGTTAGCCC
TTTTTCCTTTGGGGTTCAGGTGAATCAGTTTCTTCATTTCAAAGCTAGAGTTCTTGAGCTTCGCGAAGGA
AAAGGTAATCTTCGTGAACATGAAGCTTGAAATCCCGGAGAGAACATTTTTTTTTTTTTTTAATATTTTTT
CTGTTGCTTTCTTTAGGTGGTAAGAAACTTGATAAGTATCTACCAGCTGAAGATTACTTCCAAAAGGGGC
TTTACATGTTTGATATTGGCCAGAATGACCTTGCTGGTGCATTTTACTCCAAAACTCTTGACCAAATTCT
TGCCTCAATACCTACCATTTTGGCTGAGTTTGAGAGTGGAGTTCAGGTTAGACTCGATGGTTTCTTTCTA
AGTTATCTTTGCACAATGGTAATTTAGTTTAGATGTGTTTTTACCTACTTGATTTTATGTTGGGTTCAGA

TATCTTTTTTGAATTTGGACATTTTTGTAGCTTGGTTTTGAGTGTTTTGGAGGTTTTTTTGTATAATTTC
TTGGTGGTTGATGATGGCTTTAGTTATAAATAGGCATCCTTTATAGAAATATTAAGCCATTAATTCTTAA
CCAGCCTCAGGGTAGCTAAAGTTTGTCTTTGTTCTATGAGATTAGTGGGGTTGGACACTAACATAAATTA
CCAACGTGAAATCGAACCTGTTAGGCTGAAGCTTAATTACTGCCAAGTAAAATGTCAGAAAACAGAACAA
AGAGAGGGTGAAAGTTCAAGTTCATTTTTTCTGTCATATGATAAGACTTTATAACTGTAAAATGTTACAT
TTATGGTTATTATATCATCTATTATAATTAACTAGTCTCTCCTCTGCTTGTTGACATAGCTAACACACTT
TTAGTGAAACACACGAACTTGTGTGTCGATTTTCTATAGTTTAAACATAAGTATTGCCCCAAGTAAAATG
TGGGAAACAGAATAAGTAGAGGCTGAAAGTTCGAGTTCAAGTTCATTGTCTATATGCCATATGATAAAAC
TTTTACTGCCATTACTTGACTATTCTCGTTGGAATGCCTTAAGTTTATTATTTGACATTTCGAATGACCT
AGCACAAGAGCTTCTAAGTCTGTAGTTCGACATTTTCTCTTTAATAAATAGCTAATACACAGTTAGTGAA
CCACATAAGCTATGTGTCGATTTTCTATCGTTTTTGTATTTTCAGTTTTTTTGTTTGTCGATTCCATAAT
AATCCCTTTTGCCTACTTTTTAGAGACTGTACGACCAAGGAGCTAGGAACTTTTGGATACACAACACAGG
TCCTCTAGGATGTTTGGCTCAGAATGTTGCTAAATTTGGAACTGACCCATCAAAGCTTGATGAATTTGGA
TGCGTTAGTTCACACAACCAAGCAGCCAAGCTCTTCAATCTACAACTTCATGCTCTCTGTAAAAAACTGC
AGGGCCAATATACTGATGGCAACATTACGTACATTGATATCTACTCGATCAAATCGAATCTTATTGCCAA
TTATTCACGACTGGGTCAGCCATCTTGCTAATATTCTGTATAGATTTAATCTTGTTTTCTATTAATCCTG
ACAAATGTGATGACTTGCTACATTTTGTGTGTGAATGGCAGGGTTTCAACAACCTATTATGGTTTGCTGT
GGCTATGGAGGTCCACCTCTTAACTACGACAGTCGAATCGTATGTGGACAAACGAAGATGTTGAATGGGA
CATTGGTCACGGCGAAAGGGTGTGATGATAGCTCAGAGTACATCAATTGGGATGGAATTCATTACACTGA
AGCTGCAAATCAGTATGTGTCATCACAAATACTTACTGGGAAGTATTCTGATCCACCCTTCTCAGACAAA
ATGCCTTTCCTTCTCAAGCTCAAGTTCTAGTTCCCTTTTATATTCATTACCCAATTGATAACTGATTTAT
ATGTCTAAATGTTAGCATTCATATATTTGGGATTTCAAACTAAGATCTGACCAGGGGTACTCGATGAACA
ATGGATTTATGTTTCGATGTTGAATCAAATAATCCATTATCTCAATTCATTTTATGGCTCCTATTTCAAG
ATTACAAACACATTACATAATGGGTATGGATAAATCAAAACTACAAGTGTTTTAAACAAGTTATAGCTTC
GCTCTCCCCTTCTTCCTTCAAATAAATCAAAATTTAAAATGTATTTTGTTCTTTTCTCTATTATTTCCAT
GGCTTGCTCTTCTTTGTCTCTCTTATCTTTTTCCTTCCACATCAAACAATAGTCATTAGGGTGAACAACT
TGGGGAAATGCTACTTCTGGTACGATAATCCAAAGGACACAATGCTGTTCATTGAATTCTTCTTTGAAAC
GTTGAGTTGAGGATATACATCATGAGCCCCACATGCAGGTGTTGCCATAAGGATTGATCAATTCTTAATT
TTAATAACAAAATGATACTTTCCTCTTACACTTCCACATTCTGAATAATCAAATTTAATCCAACCCTGTG
TTCTGACCCATGTCCCTTAATTATATCTTCTCTAACATTCAAATTTTCAAACGTATTGTAATATATACCT
TTTGGTGGATATTTTATGCCTTTCACCATCTCTACACTCACGATGGGTCTTTCTTCCTTGCAAAAAGCAA
AAGCTTAATTTGTAGAATCATCTAAAAATTTAACAATCTAGTTAAGTGATCTGTTGCATGGAGAGTTGTT
GGCGATTGCAGTGTATCCCATGCCTTCCATTCCACATTTCACTGTTTTATCATTATATGTTGTGCACATT
GCTCTGCAATTTGACAAGCGTTTGACAAACAACATATAATTATATCATACATAAAAAAAAATAGACATTTG
CAATTTGACTAGCCTCTTAAACACAACTTTGTATCAATGCTTTATTTTAATCGAATGAAGTTTGATGTTT
CTGGTGCGTCTAGTGTCACGGGCATACTTGTCCAAGGCGTTATTTGCCTATGGCCGTATGCCCAAATACC
CCAAAATTGCTTCGTCTTTATGTTTTCTTTATAGTTTAGTCTATTGCTTTCATTTTCGTGTCATCGAGTT
ACAGTGTGACATTTCGGCAGTTTAACGTTCAAGTCTGCCAAAGCTAAAAAAGTAAAAAAATGTACTATAG
GGGAGGCATAGTAGTTGAATCCCCGACAAAGCCTTGACGCACTCTTTGCAAACTAAGTTTTTTTTTTTTT
TGCAATTGACAATCTTTAATGTTTTCATTTATGATGTTTTCAATGATTTTTTTTTCTCTCTCACGTTTTA
TAAAATAATTTTCTCGACAACGTGAACGGAGACTACATCATATCGCGAGTTTGTCCGCGGATTTAAAATT
TTGAATAGCTGACTTGTTGACCGAGCAACACAATCGTGTTTGAAGGGTCACGATTGTGACACCCAACACT
AAATAACACATCTGTTTCCATCTAAAAAAAAAAAAAAAAGACGCATTGTTATTGGTATCTCTATCACTAG
CTAGCTAAGGGAGAAAGTGAAGATATTGAATACCTAATCAAATGTAATCATAATAAAGAATCTAAATTGA
TTCACTTTAATATAACAATTTAAAATTTTAGTAATATGCTTTGGACGATATTTTTGAACAAAATCTAAAT
TAAATAAGAATAGAGTGTTCAATTGATACGTACACTTACAAAAGTTATTGAGCGTTTAAATTGATGTTAA
TTATCATAGAGTTTAAATTAATACAAGTACCAAACAGACTAAAAGGACATTTAAATGTAAATTTTTACTA
AAAATTGAGAGCTTCAGAGCAAGGATGTATCAACCCCTTCAATCAGAATCACACCATCACCTCTTCCGTC
ATAACTGCAATATGAAGCTTTTATCCAACAATGTGTATAAAACCAGTCATGTACATCCTGATAGCTCTTG
GCTTCACGAAGGAGTATCAAATAGCGTTTGGAGGATATTGTGTGGGTTGTGATATTCGTTCCTCTTTCAC
CATCTCCACACTTACGATGGTGGACCGCCGGCGACCAGGAATAGCTCAGGAGTTGTCTTCTTTTGATATG
GAAAAAGCTTGTAGAATTATCTAAAGCTTCACCGCATTGGCCCTCCATCCCTTTAAATTTATTGCATAAA
GAGTTGGCAAGTGCAGTGCCTCCCATGCCTCCACATATATATGTGCCATCATTATTTGTTACGTGCAATG
GAAATATCATAGGTTGTATGTTGGAATCAATTATTGCTTCTGGATCATAATTTCCTGCATGTAACATTGC
TGCTTTGGAATTTGACAGGCAAGAGACTGTTTCTAAAAAGCTTTTGAGAAGAAGATCCTCAATATCCCTA
TGCCATTAAACAAACAAAATCATAACATACTCATCTCCCCCTTTGAGATCTATTATATACTCTCACTCCA
AAAAAAAAAAAAAAAAAAAAAAAAAAAAAGAGAATAAATAAATAACAAGTTAACAATCAACTTACAGACTTT
GCAATTTCTCCAGTTGATAAAAATCAGCATAATTTACCATCGGCCACTTGACATCCATTTGTATCCCATG
GAGCTCTTCAGTGACATGAACACCACACCTTGTTATAGTTGCATTAACCTCACTATGTGCTTCATGAACC
TCAAACCAGACCAAAACTTTATTCCAATCATTCACCTCCATGGAACCCCACGCTAGAGAAGTTGTTACTA

ACCACATATATTCTGATTTTGATGGAAGAAATGGTCTTGAAAATGAAGAATGGAGTTTACTGCAGATGAA
TATACTACATGAAATTTGGGCTCCTCTTTTAGATGAATCTCCATTCACTTTGAAACTAACACAGGCAGCC
AAAGTTCTTTCCATGTCTGGATAGTGACGAAAGCTAGCTGTCACTAAATTGGATGTAGTCTTATAGCTGA
ACCATTCTGGAATCTCAATCCCCGTTAATAAAAACTCTCTTGAAATCTCACCCAATGTGAGGTCCTATAG
AGAACAAGAATTCATAATGCATTAAATTGTTTACAAGATAAGAATATAAATTGAATGGAAATTAGAGGCG
AACCTGTTTTTTTGATATTATATCCACAATGTTATCTGGATTTCGAGCCAACGATTCACAACCACTGGCA
TCCATTTTTTGTATATTCTCAGGAAGGTTTGGAATTTCTTGAAGAAACTTGCAATTCCTTAATTCAAGAT
TCCACAAGGACATGAACTTGTGGAGACATGAGGGTAAACTAGAGAATTTGTTTTCAGACAAGCGTAGATC
AGATAAGAAAGGGGCAACATCACATAATAATTCTAAAAATTTTGCATTTGATATGTTGCAAGATTCAAGA
TCCAACAAAGTGAAATGGGCACATAAACTTTCATTTGGTACTAGTAAATGGGGAAATTCTAAGCTCCAGG
AAGCTGTTTCCATCATTTTTGAAGGAGAGCATACTTGTTGGATGGTTGGGATGGTTGGGTCCCATGTATG
GGGAAACATTCCAAATATAGAACAGCCACTAAGAAGAAGATTCTCAAGATTCCTTAACAAATAAATTGTA
TTGGGAAGGGAGATGAGGTTTGTGCAACCGTTAAGTTTTAATATAGAAAGGTTAGTAAGATATCCAATTG
ATGAAGGTAACTCCTTTATGGCAGTAAAATCCATATCCAATTCCCTTAAAGATTTCATGTTTTCAGCAAT
TGTTGGGAAGCTTTCAAGCTTACAACACCCAGAAAGTAATAAATTGGAAAGAGACTTTAACCTGAGATAG
CTTGGAAGCTTTACCAGTTTAGTGCATTGTCTAAGGTCCAGATGATTAAGCTTATCCAAAGATCCAACAG
ATTCATGAATCACTCTTAAATTTGTGCATTCTTGGAGATACAATCTCTTAAGGTTTGATGCTGCAGATAA
GTCTGGAATTTTTTCAAGCTTTTTGCAGTAACAGAGATTCAATTCTTTAAGAGAACTTAACATGAAATAG
CCTCTTGGAAGCTTTTGAAGGTTACAACAACCATCAAGGTTTAGGACACTAAGCTTATAGAGAGAAAAAA
CAGACTTATCTATCATTCCTAAATTTGTGCAATTGATGAGATGCAATTCTTCAAGATTTGATGCCGGAGA
GAAATCATTAATTTGCTCTAATAAAGTAGAGTAGCTAAGATCAACATGCTTCAACCTTTCACAACCCTAA
AACACATTGAAAAAAAAAAAAAACCAAAAGGAAAAGGAGGTATGTAATTGCCATACAAAACAAAATTTAGA
TAAAATAGTGGCTAAAACAACAATCGTAAATATAGAAAAATATAGAATATAGGTAAAAGAAATTGTATAA
TAACCATTAATCAATATTATGTCAGTTCAGCTGTACTCATGTTAGCAACAATGCACTTTTTACAACTATT
AGTTTGATACATATCGATAGTAAGTGTGTTTTATAAGTTTAAAATGCAACTTAATACAAAAGTTTCAAAA
AATATAATTTAGGCACGAGAGAATTAGTTAAATTAAACATAGTTTTGAATTAAATTTTAGTTTATAAGTA
ATTAATTAATTAATATTATTGTAAAGTAACCATTATACCTAAAATATATTATCTTGAATTTAGTAGATTG
TAAACTAACCATTTAATTTGATACCTTAAACAGAATGAGAATGTAGTTTAGTCTTTTTAATTATAGGGTT
ATCATAACCTTAGTTTTATCCCCTCAACCTTTCTTCAACCCTTCCTCAATTCATTTTTTTCTCATTTATA
ATTTTTATATTTATGAGTTTTGTTCATTTTAATCTTTTTATTTTAAAAACACAGCTTGTTTTTTAATTAT
GAATTTGGTATAATTACATTATTAAAATTTAAATTTTTTCCTTTACTCTTGAATTAGATAATTTATAGTG
ATTTCACAATTAAATTTTTAATTTAGTTACATTCAATCAACTCTATGTTTGAATTCATATCAATATATTT
ATGAAATTAAAATTTTAATATAAATCTCAATCTCATTGTTTTTTTTTAGTTAGCTAGGATAAATATCTAA
GGAATATCAATTAAATAAAAAAAATGGTAAAATTTTAACAAACTACCATAAATTGATTAAAACAATTAGA
AAATCATAAGGACTATTTGAATAATTTAAAAATTGGTGGGGCTATAATTTAACAAATCTATTATTTAGAT
GCAGTTTGTCTATATTTTCCCACTTTCATAATGTTAATTTGGTTATTAAATTAAGCTTGGTTAAATTAAT
ATAACAAATTTTTTTGTTATAGAGGCGGTAGATTCATGAATTTTTTACCCAATAATTGTTGCAAAATGAC
TAAAATAAATATTTTGAAAGTATAAATCACCGTTAAAAAGAATAAAAAATAAAGTTAATATTTTAAAAGT
AGACTAAAATAAATTGAAGAGAAACCTAAAACTAGATTCATCGTAATTTTTCACTAGAAAACAAAACTAG
AGTTTGTAAAAGCGTTGATTATTATTTTCTGTTTTCGGTAATTCGCATTTTCAATTGTTATTTACCCTTA
TTTACATATTTACAGGTTGTGTGTCATATTCATGAAAACCAAATTATTGAAACGTAACGTATACTTAAAA
AATGTTGAAAATAAACAATGTGTATGTTGAAATTGAATAACGTAGTTAAATAAATTAAGGCAAATTATTT
TATGAAAACTAGGACAATTTAGTAAATTAAATATACGAAATTTGTGTATTTGATTTATAATANNNNNNNN
NNNNNNNNNNNNNNNNNNNTATATATATATATATATATATATATATATATATATATATATATATATATATA
TATATATATTTATCATAACCCTACCCACATAACCCTTCTCCCAAACATATTTTATCATAATACTTCCTCC
ATAACCCTTCCACTAAATACATCTTTATCGTAATCCTAGATTTGTCATAACACTAGTTTTATCATAACCT
TAACTCCCATAATCCAACCCTTTCCCCAAAAGACCCCTTGGAATTAATCCATGTAGATCATATCACTAAT
AGGAGTATATTAATGATAGATCATATGACTAGCATAAGTCTATGGTTGTGCGATAGATCACTATATCATT
AATAAACTTTGATATAATTTGCAAATTCTTTAAAAGTATGTGATTAGCACGGGTTAATCCATGTTAAATT
TTGCTAAATCACCTATTTGTGATTTTTTTTTTTCCGGTGCAGTTAAGCTACCCATTAAAACTTTTTAAGAA
AATCAATGAAGAAATCTCCAGTCAAATACAGATGCAAAAAGAAATATATTTTACCTCGAGCCTTTTCCCA
AATCTTTTGATGGAGCTATGTCGCAAATCAAGTCCAACAAGATTTTTGGTAATGAAGCATGACGGCAAAG
ATCGATGAGCAAATCCACGCCACTCAATCCACTTTAAGCTATTAGGTAGGTACTTAATCTTTCTACAAAA
TTGTGCATTTCGAATGATAAGCAATCTCAAATTTTTCATGCTTCTAAATGCTTGTGGATCTACATTTAGC
TTTGTGGGATTAGGCAAGTCCAACTTTATGGCTTTAACTGCATCTGTTCCCTACAAAGTCATATATTTAG
TAATTTAACATTACTGACAATTTAATTACTTTGACAAACATTTATATATTATCAGGTGAAGTCCAAATTA
TTAAATACTTTAGGTAGGAGTTACTCACTGAATTATTAACAAACACCTCCCAAATGTCCTGCACCAACCA
CAACCTACTCCTCTTTCCAGGCTCAGATGATTCATTATGAACTATGCTACGACCCATTTGTTGTATTAAC
TCATGCATTTGCACTCTATCCGTTTCAATAGTAACAAGTGAAAGATCCACGAGTATCATAATTCCAAAAT
CTATGTTCAAATGGCATGCACTCAACATTTTTTTAGCACAATTGTATTCTTCTCCCACAAGTAAACAAGA

AATATCAAGGAAAATATCCTTTGCTTTGTCTTCAAGTCCATCAAAACTTAATTGAAGAACATCTTTAATA
TCTTTTCTCAAAGAGGTTTCAAATTCATCTAATATACAGTTCCATTCTGCTTGATCTCTGCCACGAAGGA
AAGAACCCAAAACAACGAGTGCCAAAGATAGACCTTTACAATAATTTGTAGCACGTTCTGAAAGGCCTAG
ATAATTACTTGATGGATGGCTTTTCTTAAAAGCGTGCCAACTAAAAAGTTCAAGAGCATGATCTTGATTC
AATTCTTGAATTTTATGCTTTTGATCATCAAATCCATGGCTAAAAAGTAAATGCTCATTCCTTGTCGTCA
CAATGATTTTGCTACCTCGACCAAACCAATCACGACCACCAACCAGTGCTTCTAATTGCTCACGATCATC
CACATCATCAAGAACTATAAGAACTTTCTTTGAACACAGTCTACTCCTTATGATGTTAATTCCTCTATCA
CGGCTGACAACCTTCAAATCCTCCTTTAAGATGTCATTGAGTAAGGTTTCTTGTAGTTTAACGAGCCCAT
GCTTTGAAGCTTCTCGTCTAACATCTTGTAGAAAGCAACATCCTTCAAATTGGTTAGCAATTTTGTTGTA
CAAAGCCTTAGCTAAAGTGGTCTTACCAATGCCTCCAATGCCATACATCCCCACCATGTTAACACCCTCG
GACCCAATATGAGAGACCAACTCCTCAATTTTCCTTAATCGAAAATCAATTCCAACTGGATGCTTGGCTA
CATGTAGTAATTGCATTGGATTTAATACAGACAACACTCGTTTAACAATTTCTTGGATAAGTTCAGCCTC
ATCCTTGCTGTTGTAAAGTAACATCATACAAAAAAAAAAAATGAAAATAGGAAATAGTAGATAAATTTAAA
CTTCACTTAGACAATGAGTCTTGGGAAATTAAAAAAAGAAATACCAATTTGCTAGATCCCAACCAGACAA
ACCAGCAGCAAAAGTCAAAGCTTCCTTCCATGGTTGAATCTTGTTGGTCAATAACTTATTAGCTTCATGT
TTGGCTAATGCTTCCCCAAACCCACCGGTTTGTTTTCGAACCTCGGACGGATCCACCTTGTAGAACACCG
GCAAAACCGCTTGACTTTTGGATTTTTTATACTCAATTATTTTCACCAGTTCATCCAAACACCAAGTTGA
AGATGCATAATTTTTAGAGAAAATAACGAGTGAAAGTCTAGATCGCTCTATAGATTTGAGAAGAGACTCA
GAGATTTGGTCACCCCTTTTGAGTTTGTCGTCTATGAAGACGTTGACTCCCTTTAGACGCAAGGCCATAT
GAAGATGACTGATGAAACTGGAGCGAGTGTCTTCTCCTCTGAAACTAAAAAACACATCATAATCGTAGTA
ATAGAGAAAGTTGGGAGAAGAAGAAGAAGAAGAAGATTCAACTGGAAGAGCAGAAGAACCCATTCTCTCA
CAAAAACAAAACGCAATGAAAAAGAAAATGGTAAAGTGATTGCGTTCTACAAAAAAAGAAGAGTCCGCGT
GGGGATTCCACATTTCCAATCATTGATTGATACAGTTTCCTTTCCATCCAAAGACTTTTCTTCACCACTT
CCATTTTCCCGCCCTCTTTCCTTTATTAATTACAATTATAAATTAGTTTCAATTATTACAAATATCTCTC
AAAGATAATTTTATCGTAAATTTTAGATGAAGATCGTTAAAAGTTTTGTTTCAAAAGTTCCTCTCAATTA
TCGAGTACATTCGAATTATAAAAAGTTAAAGTCTACTCTCATAATTAGTACATGAACTCAACCCATTAAT
ATCGCAAAAATAACTCTAAATTAAAAATCTCTATATAAATTTAAAATATTTCGATTTATTTGAAAATTAT
TTGAAGGTTACTTGAGCTCGGAAAATATCAATTTACAAACAAATTATATTTGGGCGTCTAGTGATCTTAA
TTTTTTAGTTAAGTCTAAGCAATAGAGACTCCTTCTTTAAAGAACTTGAGCTCGCCCCAAGTCTCTGAGA
CTCCTTTCACGTTATTATACCTTTATGTTGAACAAAATCTTCTTTGTTCAAATGCATGCCTTAGGCTTTT
CCTAATTTTATTGGATCTCTTCTCAATGAATCACATAAATATAGCAAAGTCTACGAGAAACTCTTTGCAA
TATTGGTGTATTATTAACAACCGTTTAAATAAATTGGACATATTTTGTTGTATTTGCAACTTTTTTTAAA
TTATTAATTTGATTGGTGTATTATTAACAAAGGTTTAAATAAATTAGACATATTTTGTTGTATTTGTGAT
TTTTTTAAACTATTAATTTGAATCTAACTGTTACATTTGTAACTGTTCTTTTATTTTTAAGCTTTTAAGT
TTGTGCTATTTTATCGTGGAACTTTTGGTGTGTCAATTTTTTATATGAAACTTTTAGATTTGCTTGATTT
TTTATTCTAAATTTCAAATGTGTTTTAACCATGAACTTCAAACGTTAAACTAACCGTTATATTTCATTTA
GATAAAGATAGTGTAACTTACAATTTTATCAATTAAACTTATAAACTTCATTTAGTACATCAATTTAGAC
TTTCAATTAAGATTTATTTTGAAAAATAGTCCATACATCAATTCTTGTCGTAAATTTTATTAATAATTCA
ACATCCAAAGAAATCACAAACTTCATTTTCTCTTCGTCGACTCAGACTGCAAAAACACTCACTTGAAGGT
TTATACCATTTCTATTACTACAGTTGGTTTATCATGCTTGAAATGTCTCTAAACTAAGAGATTTGAAACC
AAAGCCATAGAGTCCTTTTATAGAGCTTAAGATCCCATTACTTTCTAAAGTAGATAATATCACACTAATG
GGAAGATTTGTAGAGGTTTTGTTCGTGTTAACACATAGATCAGATAAATTGCTCAATTTATCGATGAATC
TACTAAGCCCTAGCTCAGAGAGTCAAAGAAAATCAAGCAAAAATATGTTCTCACTATCAACCGTATATCG
CTCTCACACAGCTACCTGTTTGGGATGTATCATAGTTTCATTCCAAAGAAGTAAATAAATAACAAGCAAA
CAATCAATAGCGACACTGGGATTGATGTAGGCTCACTTACAGATCTGGCAATTTCTCCGGTTGATAAAAA
TCAGCAAAAGTTACCACCGGCCAGTTGAGATCCGTTTGTAACCCATGGAGCTCTTCTGTGACATGGACAC
CACAGCTTCTCATAGTTACATTCACCTCATCTTGTGCTTCAAACCAGATCAAAACTTTATTCCAATCCTG
CACCTCCATGGATTCCCGAGCTAGAGAAGTTGTTACTAACCACATATACTCTGATTTTGATGGAAGAAAT
GATCTTGAAAAAGAACAATAGAGTTGATTGCAGATGAATATATTGCATGAAATTAGGGCTCCCCTTGTAG
ACGAATCTCCATTCACTGTGAAACTAACACAGGCAGCCAAAGTCCTTTCCATATCTGGATAGTGACGAAT
GCTAGCAGTCACAGAATTTGTAGTAGACTTATAACTGAACCATTCTGGAATCTCAATGCCCGTTAGAAAG
AACTCTCTTGAAATCTCACCCAATGTAAGGTCCTGTAGAATAAGAATGCATATCCATAAAATTGTTTACA
AGATAGAAACCAATTGAATGGAAAGAGATGAACCTGTTTTCCTGATATTACATCTACCAAGTTCTCAGGA
CTTCTAGCCAACGATTCGCAACCAGTGGCATCCATTTTTTGTATAATATCAGGAAGGTTTGGAATTTCTT
GAACAATTCCTTAATTCAAGATTCCATAAGAACATTAACTTATGGAGAGATGAGGGTAAACTACAGAATT
TGTTTTCCGACAAGCGTAGATCAGATAAGAGAGGGGCTTCATTAAATAAAGTTTCCAAAAAGTCAGCATT
TGATATGTTGCAAGATTGAAGATCCAAAAATGTGAGTTTGGAACAAAAAATTTCTTTTGGAATTAGTCAA
TGGGGAAATTCTGAGCTCCATGAAGTTGTTTCCATCATCTTTGAGAAAGAGCATGCTGGTTGGATGATTT
GGCCCCATTCATGGGGAACAATTTCAAATCTAGAACACCCTCTAAGAAAAATATTTTCAAGACTCCTTAA
CAAATACAATCTTGACAAACAAATTGTATTGGGATGGGAGATGGGGTTTGTGCAACCGTTGATATTTAAT

CGAGAGAGCTGAGTAAGATATCCAGTTGATGGAGGTACCTCCTTTATGGCAGTAAAATCCAAATCCAACA
ATTTTATAGATTTCAAGTTCTCAGCAATTGTTGGGAAGTTTTCAAGCTTCCGACACCCAGAAAGTCCTAA
ATATTGTAGGGACTTTAAGTGAAGATGGCTAGGAAGTTTTGCAAGATTAAAGCAACGTCTAAGGACTAAG
GTAACAAGCTTATCCAAAGATCCAATAGACTCATGAATCATTCTTAAATTTGTGCAATCTTCAAGATATA
AACTCTCAAGGTTTGATGCTAAGGATAAATCTGGAATTCTCTCAAGTTTTTTACAGTAAGAGAGCTTCAA
ATGTTTAAGAGACCTTACCATGAAGTAGCTTGTCGGAAGCTTTTTAAGGTTAGAACAGCCATCAAGGTTT
AGGATAGTAAGCTTATCAAGAGAAAAAACAGACTTATCAATTTTTCTTAAATTCGTGCATTCGCTTAGAT
GCAATTCTTCAAGGTTTGATGCCGCAGGGAAATGAGGAATTTGCTCTAATAAAGAAGAGTAGCTAAGATT
AACATGCTTCAACCTTTCACAATCTTAAAACACATGGAAAAGAAACCAAAAGGAAAAACGGTCGTAACTG
ACATACGAAGCATAATTTAGATAAAATATTACAGAAATAGCGACTAAAAGAATGCTTGTAAATATAGAAA
AATAGAATATATGTAAATCAATAATAACCAATAATCAATATTCAAGTAAACTAATAATACCCATATACAA
AAGTAAATCAATGATATATATAAGTAGATCAGTGATAAACTCAATCCATATCAAGATTAGTAATCTATAT
ACATTAATCCACAGTACTAATCCGTAACTATTGCATAAAATAGCAACATACTTCTTACTACTATTTTAGT
TAAGGAATATTGTAATGAGTTGAAAAAAGAATCTGATTTTCGTATTTTCTATGATTTCAATTTTACTCTC
ATTTATGTATCTTAAAGTATATTTGACTTGTCTTTGTAAAGTTGATTGATTTACCAAATACAAAGTTGAT
TGATATACCAAATATAAAGTAATCATATAGCATGACTTTAATTACGTTATGGCAAATGCTTGTTCGAGTA
TGGTGAATATTGTTTTTGTTTTTTGTTTCCTTTTGATATGAAATTGATTTCATTTGATCAGTGTATCAAC
AAAATAACATTGTTTGTTTAAAATACAATGCTACCCGTTTACTAATTTTTAAAAATTATCATATTATCAT
TTTTTTTATTACAAAACTACATATTTTGAACTTTTCTAAAATGAAAGCTACCCATTTAATTTCTAATAAA
AACAATGAAGAAACCTCCAATCAAGTAAACATCCCGTAGAAGAATAATTTACCTCAAGTCTTTTCCCGAA
TTTTTTGATGAAGCTATGTTGCATATCTAGCCCAACAAGACTTTTTGTAATGAAGCATGACGGCAAAGAT
CGATGAGCAAATCCATGCCACTTAATCCACTTTAAGCTATCTGATAAGTGTTTAATCTTTGTAGAGAATC
GTGCATTTTGAACAATAAGTACTCTCAAATTTTTCATGCTTCTAAATGCTTGTGGATCCACGTGTAACCT
TGTAGGATTAGCCAAGTCCAACTTTATGGCTTTAATTGTATCTGTTCCCTACAAAGTTATAGATTTAGTA
ATTTAACATTACTGACAACTTACTTTGACAAACATTTGTTGTCGTGTAAAGTCCTGGAAATAAGCAAATT
TTCGATACTTTGCGTAAGAGTTACTCACTGAATTATTAACAAACACCTCCCAAATGTCTTGCGCCAACCA
CAACCTACTCCTCTTTCCAGGCTCTGGAGATTCACCATAAACTATTTTATGGCCCATCTGCTGTATTAGA
TCATGCATTTGCACCGTACCATTTTCAACCGTAATAAGCGAAAGATCCATGAGTATTATAATTCCGAAAT
CTACATTGAGATGGCATGCACTCAATATATTCTTAACGTAGTTAACTTCCTCTCCCACGAGTAAACAAGA
AATATCAAGAAAAATATCCTTTACTCTGTCTTCGAGCCCATCAAAACTTAATTGAAGAATATCTTTAATA
TTATTGCTCAAAGAGTTTTCAAATTCATCCAATATACTAATCCATTCTGTTTGATCTCTAGTACAAAGGA
AAGAACCCAAAACAACAAGAGCTAAAGGATGACCTTTACAATAACTTGTAGCACGTTCGACAAGGCCTAA
ATAATTACTTGATGGATGACTTTCCTTAAAAGCATGCCAACTAAAAAGCTTAATAGCTTTGTCTTGATAC
AATCCTCGAATATTGTGCATTTCATCAAATCCATGGCTAGAAAGTAAATGTCTATTCCTCGTTGTCACAA
TGATTTTACTACCTTGACCAAACCAATCACGTCCACCAACCAATGCTTCTAATTGCTCGAGCTTATCCAC
ATCATCAAGAACTATGAGAACTTTCTTTGAATGCAGTCTACTCCTTATGATGGTAATTCCTCTGTCAAGA
TTGACAAACTTCAAATCATCCTTTAAGATTTCATAGAGTAGGTTTTCCTGTAGTTGAACAAGGCCATTGA
ATTGCTTTGAAGCTTGTCGAACATTTGATAGAAAGCAGCACCCTTCAAATTGGCTAGATATTTTATTGTA
CAAAGCTTTAGCCAAAGTTGTCTTACCCAGGCCTCCAATGCCATATATCCCCACCATGTAAACACCAATA
TCGACTCATACTCATTTTGTGTCCAATAATGGAATTTGTTTCCCTTCTTGAAAAGATAATGTGAGCGAAA
CTTCATGTATTCTAGTTGAGAATCAATTCCAACTGGATATTTGGCGACATATAAGGGTGTGCGAGTGCGA
TTTATTGTAGATAACACTTCTTTAACAAGATCTCAAATAAGATCAGCCTCCCTCCTGTTGTCAAGTACAT
AATCATATCAAAATTAAAGAATGATGTACACACAATTAAATGGCATCTTGAGAGAGAATGGATCTTAAAA
ATTGTACAGTGTGCTCGTTGATAAATGAGTAAAAAAAACATGGCATTGAAAATGAGATCTGGAAAAGATA
GCACAAGCAAGATTTTTGTAAAAATATATACCTATAAGCTCCTAGATTCCAACCAGACAAGTTAGCAGCA
GAAGTTAAAGCTTCCCTCCAAATTTGGGTCTTTGTTTGGAACTTAGCCTGATGTTTGGCCAATGCTTCTC
CGAAGTTACTTCGTATATCCGACGGATCCACCTTATAGAAAACTGGCAAAACAATTTGGCCCTTGAATTT
CTTACACTCAATTATGTTCACCAATTCATCCAAACACCGGCATAATTTTCAGAGAATAAAACAATAGAAA
TTAAAGCTTTCTGTATAGATTTGAAAAGGATTTCAGAAATTTGCTCACCCCTTTGGAGCTTGTCGTCTAT
AAAGACGTTAACACCCTTTTGACGCAAGACCATATCAAGATGACTGGTGAAATTGGTGCGAGTATCCTCT
CCTCTAAAACTAAAAAAAACATCATAACTCCACTCGAAAGCGATGGATTCTGTTACAGCAGTGGAAGAAC
CCATCAGAAGAAACGCAGAGATTGAAGCAAAGAAAAACCGGGAACGCCAACGGCAATGTTTGTGAAGAAA
ACAGAGCAAAAATGAAGAAGAAGAAGAAGAATGGAATAAATAAAGTGGAAAAGTCAAAAGATTGGCCTCT
CATTTCTCAATAAGCCGATGTCAGTTCTTCCACGGAGCAGAACAGTTCACATTGAAGAAATGGGGCATCG
CTTGACGATTGATTCAACTTCCATGGAAAGACTTTTCTCCAGAGCTTGCACTATTCCCGCGCTCTTTAGC
TTATCTATTAATTATGGTTAAAATAAAAATATCATTCAAAGTCTTAGAATTTTATATATATAGTCCTCAA
AGCTAAACTCAAAAACACAAACCTAACTACTTGATATTTTGAAAGTTATGGATTAAATTAAAAATATTTC
GAAAAATCTAGTCAATATCATAAATATTATCAAAATAGATGCTTGATTATACTTTAGTGTCTGTTGACAT
GATTGTTGTAGGTGTCGGAGAAGATATTAAACTCGTGTAACTTCCTTTTCATGCTCGTTAAAGTATACAT
TTTGTAGGTTTTATAAGATACATATAGTTAAAATTTTCAAATAAATGGGAGAAATATTCCTTTTTAGATT

TTCTTAAATTATCTGCATATTTTATTTTTCATTTTAATATTATTATATTTTATTGATTGTGCCTCATTTT
TACCACAATTAAAAAGTATTATGTCAATATGTTTACCCATTTATAGACATACAATTTTTATTTTTATTAT
TCTTTTTGAAGCACCAACGAGAAATACAAAAAATATTTGGAGCATCAACAAGAAATACAAAAAATATATA
GACTAAATAGTACCTAAATCAAATTGTAGCAAAAATTAAACAATATTGGTACAAGATCTTCTACCAAATA
TAAAAGATCTTGGTACACGATCATGATAACAAAAATTAGTGAGGCATTGGTATTTCCCATTGTAGGTCAG
GGACATTTTTGTTTATTATATATTTTTAAAGAACCCTTATTTTGTATATATTGAATTAAGATTCACAGCTGT
AATTTACATTATGAAAATTATTTTTGGGTTCAATTTTATTAATAATTTTAGGTTAGTTTAATTTATTTAA
ATACGATGTACTTAAGATCAAGTTGAACTAAGAGGTAAGAGTCAACACAATCCAATCCAATCCATAAATT
TTGAGTTGGGTCATCGGGTTTTTTTTCTTCGTAATTAAAATTATTTTTCTAAGTTAAAATTTTTGAAGGA
AAAGTGTAGTAAATAACCTAATATATTATATCAATTTTAAAAAATTATAATCAAACAAACAATTAATATT
ACATTACATCAATAATAACAATAACAAAAGAAAAATTGGGTTGAATAGTATTGACCGGATTAATCAACTC
GGATTGGTTAGCGGTTTTTTTAACACCCCTATATATATACGCCTGAACCGCTCCAAAACTTCTTTCCCCT
CTTCGGTTCAATACCCAGTTCGAGCAGATCTGCGTCGTCGTCGGCTCTCCTCCCACCGCAAATCGCCCCC
CTTCGTTCTTTCTCTTCTCCGGCTTGTATCAACTCTCATCGAATCCCGAACAGTGACTCCTTCTTCTTTT
CTTCAGAGCCACGTTTGTTTCAGCTGTCTTTTTTTCTCTCCCGTCGAGTTTCGGTAAAGACCACTCTTAT
TATTTGATTCCAGTTTAACGCTACTTATTTCTAACCCCTTCTAGGCCAGTTTTAGGTCTTAAATCTCTGT
TTAGATGTTTCTGAATTCATTTTAAGTGCTTTAATCTAGTTTATGACCCTTTGGTTCTATCTATTAGATT
ACTCATTCCAAATGCATTATGAGATTTTGAATAAGGTTTCGAACCAAGTTTTTTTTTTTTTCGTTTTAATT
ATTAATTAGGAGTCTGCAGTAAAGTAAACTTCTAAAGTTAGATTATGTGACCTATTAAATCAGTTTGTAA
GCCATAGTCATATTCAAGATTATTTTGAGGGTTCTCTTATTTCTTCTATTCTGGAATCTATCTCTTGTAC
CTTTTGTTGTGCGAATTTGATGAAACCCAGACATATGGAAATATACCCGATAATTGAATTACAAGATAAA
CCAATTTTTGGAGGTACTTGGACCCTCCCATTCTTGAGATCACTCTCAAGCCCTAATTCATTCTCCAAAA
TATTTTCTATCTTCTTCATTTCCCTCCTTGTTTATAACCAACGAGTGTAACGAACCCCCTAATTAGTTAT
TAGTGTACCCTTAATAGTCTAATAGCACCCTAATATACCATTTCTATCAGAATTTTCTTGGAAGATTAAT
TTTCTGTATGTTTGCCTAATTCTGTTGCCAATTGCCATAAGTATAACTATATTACTTACCTATGTCTTCT
TTTCTATTTTGGCTAGGGTTCTCAATAACATTGGCCTTACTCTCTAATAACCCTTCGGTTTCTTCTGAAG
ATGGCTACTGCATCAGGTACATTTCAAATGAAATAATTTTATCTATAACAAGAAGTCTCGGTAGAATTAT
GAACATAGTTTCACCTTTTCCGTGTCTTGCCCGATTTTTTTTTTTTTTTTTTTTTGGTGAGTTGTAGATTG
TTGAACTAAAGGTGGTTTTGGTTCTCTCCTCTCTCTTTTGAAAATGATACGTGATTTTTCATTTAGCAGA
TGAAAGGAAACTATTGCTCAAAGTACATTTGCTTCTTCTTTAATATTGTTGAATCTAAGTTCAGACTGAA
AACTAAAAGGGCAATAGGAGATGGGATTAATCTTAGGCGACAGTGGTTACCCTATTAAGTTAGAATAGAA
TAAAGCTACCATCCTATTATAAAACATTGTTTTGATTGTTCTCTCCTTGGCATTGCAGTTGCTTTCAAAT
CTAGGGAGGATCATCGCAAACAACTGGAATTAGAGGAAGCACGTAAAGCAGGGCTTGCACCAGCTGAGGT
CGATGAAGATGGGAAAGAAATCAACCCTCATATTCCTCAATATATGTCCTCTGCACCTTGGTATCTTAAT
GCTGAGAGACCAGTAAGTTTTTCTGTTTCTCACTCTTTTCCTGGATTTTATGTTTGTTGTTCATGATGAT
TGAACTTCTTTGGATCTTATTCTAGAGTTTAAAACATCAAAGGAAATGGAAATCAGATCCGAATTATACA
AAATCCTGGTACGACCGAGGTGCAAAAATACATCAGGCAGACAAGTATAGGAAGGGTGCATGTGAAAAGT
AAGTAAGATGAATGTAGGGGACATTGAATTAGATCTCACTTTTTATTAATTGTTTTTCTGTGAAAAAAAT
GTCCATATTATTGGCTTAGAACTGGGAGATTCTTTTGAATGTGGATGGCTAAGTTTCCCATCAGGAAAAC
AAAACTATTGCCTTTTCTCTTGCCTGTGTATTTAGTACATTTACGTACTAGTGGCACTTCACAATGCTTG
TATATGGTAATCTCTATTAGAAGAAGGAGCCTTTAAAATCATGGGCTATGACTCTATGGCTGCGTAACTT
TTGCTGGATATGCATAGTTCTTCATTCATAATCATGAGGTTATGGATGCGTTAAAAACTCTTTCATTTTG
AAGGTTTGAAAATATGGTTTTATTCATTTAGAATTTTTGATTGCTCTATTTCTGCAGCGGTCTAAGAAAG
AAAATTTTAACTAAAGATTGAATGCACGATCCTTGTAGTCTACTTAGGAGGTTTCTTATGTTGGCCACCT
TGCATCCCTAAATTCAATTATTTTGTAGATGAGTTTGCTCTTTAATTTTTTTTCGGTGTAAAAGGATGCAA
TTTAAATTTGTCACCCTGGCTCAACTGCAAATACATGATCTAACAATGACATTAAATTTTCATATGTTAC
TGTACAGATAGGTATAGTCTAAAATATTATGGGTTTGGAATCATGATCTTAAACTTTGTGATATATGGAA
GTTTCCTCTTTATCTTAATTTTCCCAATCTATAGTTTTTTCTAATTTTCCAATGTTTATTTTTATTTTCT
TATGACTTAACTGATATATTTGCTTGTCCAGTTGTGGAGCAATGACACATGATTCAAAGTCATGCATGGA
AAGGCCCCGAAAGGTAGGAGCAAAATGGACAAACATGCACATAGCACCTGATGAAAAGATAGAGACGTTT
GAACTTGATTATGATGGAAAAAGGGATCGTTGGAATGGCTATGATCCAGCAACCTATGCTCGTGTCATTG
AGAGGTATGAGGCTAGAGATGAAGCAAGGAGAAAATTTTTGAAAGAGCAACAACTTAAGAAATTGGAAGA
GAAAAATACCAAGCAGAATGAAGATGATGAAGTTAGTGCTGAAGATGAGGATGAAGATGATTTGAAAGTT
GATGAGGCAAAGGTAGATGAAAGCAAACAAATGGACTTTGCCAAAGTTGAGAAGCGAGTACGTACAACAG
GTGGTGGAAGCACAGGAACTGTAAGGTACAGTATGTAACCATTTACGTTTGTTTATCCTTTTATTCCTCC
CTAGCTTAGCTCTTTTCTAGAGCATATGGATTTTTTACTAATTACTTGTTCACATCTTAGGAACTTGCGA
ATTCGGGAGGATACTGCTAAATATCTTTTAAATCTTGATGTTAATTCTGCTTATTATGATCCCAAAACTC
GGTCCATGCGTGAAGACCCTCTTCCTGATGTTGATCCAAATGAGAAATTTTATGGAGTAAGAATCTGAAA
CAAAATAATGAAGTTTCTGTTTGGTCTCAATTATAACAACGTAATACTTATCGGTTCGCATTGATCTAAT
CTTTTTTTATCATCTCTGTTAAAATAGGGTGATAACCAATATAGAATCAGTGGGCAAGCTTTGGATTTCA

AGCAACTCAATGTGCATGCCTGGGAAGCATTTGACAAAGGTCAAGATATCCATATGCAAGCTGCTCCATC
ACAAGCTGAACTTCTCTATAAGAACTATAAGGTTATTAAAGAGAAGTTGAAATCACATACGAAGGATGCC
ATCATGGAGAAGTATGGGAATGCTGCTGCCGAGGAAAAGCTGCCTCGGGAACTTTTACTGGGACAGAGTG
AGAGGCAAGTTGAATATGATCGTGCTGGCAGAATTGTTAAAGGCCTGGTATTAAATCTAATCCGAACAGT
CCTTTTTTCCCCTCACAGAGGACTACTTCATTGTCATGTAGCATATTTGACCGACTATGAGTTAATATAT
GATTTTGTTTTAGAATAATGTCCTTCCTGTCACATTTTTACTGTTACTTCTGATGAGATTAATACCTAAC
TATATTTTATTTTCTGTATCATGCTTCCACACTTTATCAGACTGATTATCTGGAAGCATGGAAAGGAGTC
AAAATTTCTTATCCTGTTTCTGTTTGTTATCCCATGCGTGCTTGTGGATGATTAGTTGAATTCTAATAAT
GAAGGCATCATGGCAACTGTTTATATACATTCTCTCATTTACAATGTTTTCTGCTTGGTCATTTGAGTAT
ATTAAGTTTTAGTCATTATGTTGGGATCTATTGATCCACTTTTAAGAATAGTATCTTCCTTGATATTCAA
GTTCTTGAAAATAAATGTTGTGTTGTCTATCCAAAAATTTATAAAATAAATATTATGTATAAAATTTATC
ATTCATTATATCTATCCATTGACAATTAGTACTTGCTAATTATGATTTAACAATGTCATATTTTAGTGTC
TTTTCACTTGTTTTCTGAAACAGCGAAACTAATAGATTCTGATTACAATGCTCAACCACAAGGTGGTTTG
CGTTGTAGTTAATGGAGCTATATAGCCAAAATTTGGTCTAGCCCTTCAATGAATTTTATCTACTCCAGTA
CACTTGAACTTGATATTTTTTTTTATAAAAGCTATTAAAGCCAATAAAAGTTATAATATACTAAGGGTTA
TAATGCTGTTCATTCAATCTCAAATTAGAAAGTTTGAGATTAGAACTGTTGCTGTTTTTGTATTTTTTGC
ATTTCATTTTCTCATTTTAATCTTCTTATCTCTGTTGTCTCTGTCATGTTTTAGATGTGTGAGAAAAGCT
TAAGGTTTGAAATTACTGATTGATTGTGATTTGCCTTCTAATTCCTTTTTCCCTCTTGAAGGAGATGGCT
CTTCCTAAAAGTAAATATGAGGAAGATGTTCTCATTAATAATCACACGAGTGTTTGGGGATCATGGTGGA
AGGATCATCAATGGGGCTATAAATGTTGTAAGCAAACCATACGGAACAGCTACTGCACTGGAGCTGCTGG
AATTGAGGCCGCTGAGGCATCAGCAGACTTGATGAAGTCTAATATAGCTCGTAAAGCAACTTCTGAAGGT
TAGTGTACCTAGAAATTCATAATACCTTCCTAAATGTGGTTAGTCCTGTTATTTTTACAAAAATTGGCTT
TCATTTTAACTACATGCATAAATGGTTGGAACATTCTTGTTTTGGAATTTTTTTTTTATGCTTCCTTAGTT
TGGCTGATGATTGTATCCGTTACACTAAATCAGATTTCCGTGTCCATAACACTAAATATATATATATATA
TATATATACATACATACATACATACATACATATATGTATTGAAAAATGCACTTTTTTTCTTCACGAATGT
AACCGTTGATTAGCTATTATATGCTTGCTACTTTTATGTATGAAGTTTATAATGAAATCCTGAATGGTTT
TTTTGACAACAAAACAGATACACCAGCCCCAACGGAAGAGAAAAGGCTGGCTACATGGGGAAGTGAAGTA
CCCGATGACCTCGTCCTTGACCAGAAGAAGCTTACCGAATCTCTCAAGAAGGTATGACATTTAATTGCCC
ATCCCTGCAGTAATAACTCATGTTATAATCAAGTTTAAAATCTTGGTTTTCTCCTAGTCACTTGTTTCTT
ACACGAGATCTTTTGTTTCACTAGGAGGATGAAAGAAGAAAAGAGGAGAAAGATGAAAGAAAACGTAAAT
ATAATGTGAGATGGAATGATGAGGTACGTAATTTTCTCTCGTTTTGATCAAGTGTAGGGGGTTCCGTCAC
TTAGTTTGAGTTATGTGTTAGATTTGTTCTTTTTTGGGCTCAAAATAATGCAACCTGGCCTTGTGCAGGT
AACTGCGGAGGACATGGAGGCGTATAGGATGAAGAAGGTACATCATGATGATCCCATGAAGGATTTTCTC
AACTAATCTAGCAAGATCGATGTATTGTAATATACGTTTTTATTTTAACAAGAAACAACAAGTGAATGAT
CCAATGAGATACAAAAGGGGACTCGATTTTAGTGTATACAAAAAGTTGTTCCTATTTGTAAGAAAGTGAG
CCAAGTTTAGTTGTAATAAATGTTTCAATGAAAATAGTCGTCTCCAAAACTTAGCTTTGAGTATCTAAAA
TTGCCTTAAATTATTGATAACAAAGTCAAATATATATACATCGAACATTAATCAAGATGTATATAGATAA
ATTTTATGATCACCATTGTTTTTTTTAATGCTATTGAACTCTCAACCCAATTGAATATTGCAAGTTTTAT
CTTGTTTAGAGGTTAATTCATCCACTCATTTAGTGTTAAAATAAGTTTGATTAATGAACAAATTAATCCT
TACATACGCTTGGTAATAAATTTGATTGAAAAGAAAACTCAACATCATTTTTTCTATAAAGTTTTATTAA
TTTATCCTTTTGAACAATATATATATATATATATATATATATATATATATTAAAAAAGTATTGAATTAAATG
TCGGAATAAGAATTTGAATCAAGAATTACATTTGAAGTGAAAACTTAATGCACGCATTGAAGATGTAATC
TAATTAAAAATTTAAGAATTAAAGTAAATTTTCTCGTTTATTTTTTCATGTATAATAATTTTCTTTGTTT
AGTCCATTTTATTTTTAAATTTAGTTTTTTTTCGAACTCCTTTTTTTCGACGTATTTTAAAAATGTTCAT
TTTGGTTATTGTACTTTAATTTTTATTTGATCACTGCCTCTAAAGTCTAAAGAGACAATAATGTTAAAAA
TGCCAAAATCGACCCTATAATAAGGTGGTTTTGTACACTTACTTATTAACCAAACAATTAATAAACAATA
AAAACTAAAATAAACAAACGAAGTTTAATTAATATAATACCTTTACTATCTATCTTGAAATAAAAAAAAT
GATTCCTTTTATCTTATGTTAGAAAACTACGTAGTAGTTTACGCTCTCTAACTTAGCTTATGCTATAATT
CAAAGTATAATATTTAAAGTTGTAACCATAAATTACGGTTGATTGCAAGAAAAAACTTCCAATCTTGGGA
TTCAACCAACAAAATAAGAATCTACATGAGTCTCTTTCACAAAACCCTTTTCTACTTCTCTCTAAACCTC
TTTTGCCTCCTTTCTTCTTCTTCTTCTTCTTCTTCTTCTTCTTCTTCTTCTTCCTCCCTCTTTCTGCTTACTG
AACTGACTGACCCACCACCATCACCAACCCTCTCTTCTCCCCCTCACATTCCCATATATGCTTACATTCC
CTATGCCACTGGCATTTCCCCATCCTCATCTGTTCTACCCTCCATGGCCTCCGCCGTTTTCCTCTTCTTC
CTCCTCTTTTTCGGTCTTCCGATGGCCAGATCCAGCCCTTTCAACCGCCCCGCCGTTTTCAACTTCGGAG
ACTCCAATTCCGATACTGGCTGTCTTGTTAGTTCCGGGATTGAGACCATCGGCCCTCCTTATGGTCACCT
CTTCTTCGGAAACCCTTCTGGAAGATACTGCGACGGCCGACTCATCCTTGATTTCCTCTGTAATCTTCTT
CAATTCCGTTTTTCTTTGTTTGATATTAACTGTCATCAAATTATTACTTTTTTTTATTTGATAATTAATAT
ATATATATTTTTTCTTTCAATTTTATTAGAGGACACATTACTAAATCCTTTTTGCATAGATTGAAGGAGT
GGCATATATATATATATTTATTTCTTCCATAAATAAATCAATAAATTATTTAGACTCAATAACCTTTTAA
AATACAAAGTCTACATCATAAAATAATAAAAATTGATATCAAATATGATGATTTTTTTCTTTTTACATTA

TAGACTAAATTGTTATAAAATTTGAAATTACAATACCAAAATTCTTACTTATCAGAGTTCATGAACTAAG
TTGATACAAAATAAGAAGGGTGTATTTTTTTACTTAATTATTTTTTATTTTTCATGTTTTCAAAGCGTTG
AGCAATGAAAATATACGGTGAGTGTATAATAGAATTGATCGAATCATGTAAGGAACGAGGTTCGAGTCTA
TTGGTCTGTTAGGATACCTCCATTGCATAAGTTTAGTGTCCTCGAATTTACAATTCCTGCATTGGGCTGT
ATTTTAAGAAAATTCAAATGGGACTGATCATAAAAAAATGTTCGTATCTTAAGTCAAAAAATTCTTAACA
AAGAGCTTTTGATCTTAATTCAATTTGTGTCTTTCAAAATGATTTAAGGATTTCAGTGATTAAGATTCCT
GATTTATTTTTGGTAGATGCATAGTAACTAAGATTTTCTGCAGTGGATGCAATGGATATGCCTTATTTGA
ATCCATATCTTGATTCCATTGGCGCGCCAAATTTCCGCAAGGGGTGTAATTATGCGGCTGCGGCATCGAC
CATTCTTCCGGCGACTCCTACATCTTTTAGCCCTTTTTCATTTGGGGTTCAGGTGAATCAGTTCATTCAT
TTCAAAGCTAGAGTTCTTGAGCTTCGATCCAAAGGTATGATTATCAATTATCACGAGCTTTAAGATTTTG
AATGATCAAGGAAGGAAGAATGACATTAACCATTCTTCTTTTAGATAAGAAACTTGATAAGTACCTACCA
GACGAAGATTACTTTGAGAAGGGGCTTTACATGTTTGATATTGGCCAGAATGATCTTGCTATCGCATTTT
ACTCCAAAACTTTGGATCAAATTCTTGCTTCAATACCCACCATTTTAGCTGTGTTTGAGACTGGACTTCA
GGTTTGATTCAATGCTTTCTTTCCGAATTTTGGCACAATGATAGTTTAGTTTGGATTCATGATAGTTTAG
CTTGAGATGGGATGTTTAGTGATTAGAGCTTTAAAAGTTTTGGAAATACATCCCTAAACTCGAAGGGATG
TATTTTAATTAAAAGTAATAGCTGTTTCAATTTAAAATTTGAATTTTTGAGACCGTATCTATTTACATCC
TTCATTATATTCCATTTGAAAAAACCTAAATTTATGATTAATCGTCTAGACATTTACTGGAATCATCCAT
TTTGTAAAACTGACATTTGAAGAAATCTACATACGTTTGAGAATTATTACATGAATGATTTTCAAATATA
ATCTTAAAGAAGAGTCCAAATTGTTTGACTTGTGAAACTTTAAGAATTTAATTGACTTAAATTATAATGT
GATCGAATGAAATTGGGAAGGAGTAAATGATACTTATAAGAATTGAAGGTTTAAAATGATGCAATTATTA
GTTCAAGAGTGTAGAGGTATAAATTGATTTTGGCTAAAAATTTAAAATTAAAAAATTCAAGTCCTTTCAA
CTATAGATCTCTTTGTTTTGCTATCAATTTGGTACCTTTTATTATTATTATTATTATTATTATTATTTTC
TTTTAGATCAAGTAAAGTTGGTTTCTAAAAAAACAATGTGATAACCATTTATCATAGCAAGTCAATTTTT
TTTTCTTTTTGAAAAAACAGAACTCAGAAATGAAATGAACTATGTGTTTTTCCTTAAATTTTAAGGATGC
ATTTGACAGAAATATTATAACTTGAGCGAATGGGGTGAGGAAGAAAGTCCAAGTTTAGGCCATTTGAAAT
GGTTTTATGCTTATGAAGCCATTGTTGCCTAAATTTCAGAAATTGCACAAACAAGGGGCTAGGAATTTTT
GGATTCACAACACTGGTCCTCTTGGATGTTTGGCTCAAAATGTAGCTAGATTTGGAACTGACCCATCAAA
CCTTGATGAACTTGGATGCGTCAGTTCGCACAACCAAGCTGCCAAGCTCTTCAATCTACAGCTCCATGCT
CTCTGCAAAAAACTACAAAAACAATATACAGATGCGAATGTCACATATGTCGATATCTATACAATAAAAT
CCAATCTCATTGCCAATTATTCACGATACGGTTAGCATCTTACAGTGTTTTTGTGTACATATTTTGGCCT
CATTTGTAATCATGTAAGGAAACAACATCTTGCATTGGTTGTTGGCAGGTTTTGAACAACCTATTATGGC
TTGCTGTGGCTATGGAGGTCCACCACTCAATTACGACAGCAGAATCGTCTGTGGGCAAACTAAGATATTG
AACGGGACAGTGGTCACAGCAAAAGGATGCGATGATAGCTCGGAGTACATCAATTGGGATGGAATTCATT
ATACAGAGGTTGCAAATCAGTATGTGTCATCACAAATACTTAGTGGAAAATATTGTGATCCACCCTTCTC
TGACAAAATGCCTTTCCTTCTCAAGCTCAAGTTCTAGGCTTGATTTAATAATTTTCATTTCCATGATATC
TTTGCCATCCTAATATGTCTAAATGTAGGGAATTGAAATAAAGATACGACCAACAATGCTCTTACGTTTA
GACACTGTATATTGTGTAGCTAAATAAAAAAATTACAATGATTTTAAACAATTATTGGAACATCATAGAG
AGGTACACTCTCTCTTTCTCTCTTAGTCTTGAAAATGTGAAAAGTTGACTTTAGGGTGGATAAAATCTTC
TTGAAATTAAAGGTGATAGGGAATGGACATGATACTGAGCTATAGAAATAACTCAAACTCTTGTTTTATA
ACAGGTTTCTTGCAAATCAGCTTGTAGTGATCCTCTTTGTACAACATCAATGTTATACATTTGAATGAGA
AAAAAGTAGATGGGAAGATCTGCCTCTTACACATTTCTGTTACTTCTTGGATACATAAGCATAGTATTGA
GCCATAGGAACAACAAAGGCTATGACTAACAAACCTCCAACGACAAGACTAAACTGTCCTCGTTTCTCCT
CGGTTTCTTCCCTTGTTTTGAAGTTCGATTCACGCTTATTGTCTTTAAATGAAGGACCACCTGGATCTGG
AAGGCCATCAATGGCAGCCACTAACCGCTTTGCACTGCTGTATATGGCTTCGTTATATTTTTCATCAGTA
GCTAAGACTGCAGTTACACAAGTAACAATCTTAGTCAGTTACAGTTTGAAGAACAATAAAGTAATAACTA
CTAGCATGATTCACAAGTACTAGTTGGGAAGGATATTGACATATGATAGGATACAAGATCGTCACATCAA
ACACATACACTCAATTTGAAGTTCAACGATAAGATACAAGTGGACTTAAGGCATCTACTAGATGTGAATG
AGTTACAATTTAAGTTTAGAAAGAGAGGTAGTAGAAATTGTTGGCTGAAAAGGCTGAGGAAATTGCAATA
ACTGCTTTAGGTGGGGAGTTTTGTTCAATGTTGATGAGAGTGAAGATTGAAATAAGTGGTAGTTAGTTTA
CCTGGGAGATTCTCTGTTACTGTGGCATCAAGAATATTTTCTCCAACAGCTTGGATAAAGGCAGGGCCAC
CAGTAATGGCTCCTTCTTTCTGACTGGTGACAAGAACAACTATGCCTTTGTTGTTTCCATCTTCCACTGT
GGGATACCAACGCTCCAAAACTTGATCAGCATACTCAAATGCATCAGCTTTGCTCTACAGCCACCAGGTT
ATTAATTCATATCAGAGTTCGTACATCTCCCTTTTCATTTGATATCTTAGTGTGTGTACTTAATACCATA
GGTTCACTATTCCGAGAAGCTTTTCCCACCCAAGAGTTTCAGGGCCTTATTTGATAATCGTCTGGTTTTT
ATTACTAAGCTCATAAAACACATTCTACATTCCACCTATAAGTTTCTATCTTTGGTTACCCAGTTTCTAG
TTTCTATAGTCAGGTTTTGAAAACTAAAAGAAATAGCTTTTTACTTTTTGGACTTTGACTAAGTATTCGA
GTGTTTCTTTCAGAGCTCAAACTAAGACCTTCTGATGGCCTTCATTTTGAACAAGCCATCACAAAGTGAT
GCCTACTTTCTGATAATGTATATTATAAACATTTTCAAAATTTCATAAACATCCACCAAGTTCATCAACA
AATTCCTCTAAATTAGAATTATTACGCTGTAAAGGATACTCTTCAAGAATAGTTCTACAAAGTGGATCAC
AGCAGAGTAAAATGAACATTCAAAACCCAACAGCTTTTCCAAACCACAAGAATTCAGTTTCTCTCTCTAG

AATTTGAAGGGAGATAAAAGAAAACAACTTACAGTGAGCTTTCTAACAGTGACAAAATCAATATGAAAAT
TCTTCCTCGTCTCCAAATCCGTCAACAACCTCTTCAAATCTGACTTCGTAACCCGACTAAGAACTCCAGC
ATCGTCCACCACATGTGTTTCCTTCGGCGGCCCATCATTGAGAACATCAAATTCAGACGCTAGAGCATTA
TGCCCAGCCAACAACGGGGAAAAGTTAAGCGCCAAAGAAATCGCTACAGCCGCCAACCCATGTTGGAGAT
GGGAGAACCAAGTCGAAGGAACTGGGAGAGATGATCGGAGAGACTTGGGAGGAGAAGGTGTTTTGAGAGA
AAGTGAAATGGGTTTTGATAAAGAAACAAGATTTGATCTGGGTTGGGTGGAATAGAGGGAAAGATTCTTG
GAAGAGGAAGGTTTAGGATTGAGAAGAGGAGAGAGAGAGTGAGGAGAGAGAATGGTTTCCATAGGAAGAT
GAAGAAGAGATTCAGATATTGTGAGACATGGAATGGAGAGGGAAAATGGGAGTGAAGATCTGTTGGTTCC
ATGAGAAATTGAGTGGTTTGGATTTTGTTTGGTAGGGACAAAGTACACTTGTGAGTATGGATAGTGTTTA
TATGAGAGCCATGAAAATGTTTTGAGGTCATTTTGGTCCTCAAGAGCATTTTACATGATGGATAATTGTT
TTTCATTCCTTTTCCCCCTAATATATGTATACTATATTTTTTTATATAGAAGACTAAATTATAATGAATT
AATACCCAAAATTATTCTTATAATTCAAAAGTTTGAACATTATTCCTCAATTTTCACAGGTATTTGGAAA
ATTCATTTGGAATATTAATAAAGTATAATTTCAAGGAGAAATCATGACTTACAAATTGTGTCGTAATCAT
ACCAAACTTCATTTGAATTTGTTTTCTAAGTCACTACCATTCATGTCATGTCGTATAAAATCATTAAAAA
ATTTCTACTCCAATAATATTTTTGAAAAGATTATGAAAGTTCAAGATAATATTGTAACTTTTAAAAATAT
AGGGATACTTTTAAGGAAAACAGTCAAAAATCAAAAAAATTGACAAAATATTTATATATAATGGCTAAAT
TGTATACACGTTATTTTTGGACTTTAGTGATATTTTGTAATTCAGGTAAATATATTGTGATATTTTTTTA
TATATTTGAAATTACCCTATTTTACAACCAATAATAAAGATAAAAACAATTTCTTATTGTCAAACCAGAA
TTTAGCTAACTGACATTTCTACATATCTTTGTACTTAAAAAATAAACTTTCTTACTCTTTAACTTTTTTA
AAAATAATGTATAAAAATATATGAGTTTTGAAAACAAAATTTTGCGGTCTAGGTGAAAAAGAAAATTGAC
TCATTAAGTTGTGGTTAGATTCGTTGAAATTAAAATTGTGTTACAGTACCATATTTCTTTTGTTCACACT
AGAAAGATCCGTTATATTTTTAGAAACACATGGATGTAACACAATTAGTAAAGACTATATAGGAAACTGT
ATATACTATATAGTATAAGAAAATTTAATCAGCGAACATATGTTACCGGTAAAACTATACGAATATTACA
ATTATTTTTCAAATATGTGAATGAAAAAAAATTAAAGAAGAAAATACATTTTCATATACAGAAATTTAAA
TCCATTCTTGCTTTTGAATAATTTGAATAAAACAATCCTAAGTAAAGTTTCCTTTTTTTCTTTCATTGTG
GATATGAAAGCTATCCTCATTTCCCTATGGCCATTCCTCTTTCTTTATGCCTCCACCACCCTCCAAACCC
TCACCAAAACCCCACGTCCGCCGTCCCTCCGCCCACCACCGTCTGCTTTCCGGCGTCTTCTACTTTGAAT
TCCCAATCCAAGAGGCATTTCATCCTCAAAACAGCTTCACTCTGTCTAATTTCTTTCTTTCCAAAATGCC
CAGTTGTTCAATCTTCTGAAAATTCTCCAACTTCGAAACCTGGCCTTCCTGCTATAGCAAACACCAAATC
TTGGTTTCAGTTCTATGGCGATGGGTTTTCCATTCGGGTGCCTCCTCAGTTTGAAGACCTCACGGAGCCT
GAGGTATAGACAAAAGTACCCCTGATGTTTGATCTGTATGAAGTGAAACACTGTTTATCTTAGCCTTTTC
TACTTTCTTGGTCAACTTATTTCTGTTTGTTTAATCCTAGTGTTTATGTTATGTTTACTTATGTTATGTT
TACTTACACCAATATGAATAGTTCAAGGTAATTCATATGCTTAGTTGCTGTCAGTGTTTTTTAGAGCTCT
AGAGCAGTAAAGCATAATAGTTTGTTGAGGCTGAAGTGCAAGGTCCAAAACTTTTCAAAGAGTAATATTT
ACTATCAGTGTTTTTTCAAGGTGCAAAAGGTGTGCGCCTAATGCGGGGCTTACTTGTGAGCAAACCTTTT
CAGGGTGAGGCACAATACTTGTGCCTGATGCCTAGGCGTTTGCCCGGTAGGGTTTTTAGAACACTGGTTG
CTATTGGTGTCAATGATATCAAGTAGAAGTTTGTAGCTTTAGGGCTTTGATAGTCTCGTAATGTGTGTGT
GCACTCACTTTTTTGTTTCTCTTTCGTGGAACATTTAGCTTGAAGGAAATAAGTAGGTTCAACACCACCC
TTTCGTTTATGTATTCTTGTAGCTATTCTTTATCTTTTATTCTCTGGAGCCCGCCCTTTCTTGTACTTGT
AAAAAGTTTATCATTTGCATCTTGCTAAGTTATGTGCTTGGAGTTTGTAGGACTATAGTGCTGGACTCTC
TCTTTATGGAGATAAGGCCAAAACAAAAACCTTTGCAGCACGGTTCGGGTCTCCTGATGGGTAATGATAT
TCCCTTTTGTTCATATTGTTTGTTCTAACTTGTATTTTGCTTCTAGGTAGGATGAAGAATTTGAGTTAGG
TCAATTGACCTAGGTATACTAAAGTTGCTTTGTAAAAAGTTCAATTTATTTATTTATTTATTTTTGAACT
TTCTCATATCCTCATTTTCTTCTATTTTTTCTTCTTTGTTGGTCAACGCAGCTCTGAAGTTCTAAGTGTT
GTCACTCGTCCGACGAATCAACTTAAGATCACCTTTCTAGAGGTAGCACAATGATGGTTGTGAAGTTTTC
AAGTCTTATGACCAAAATGCCCAACATTCATTTTGTAATGCATCTAACTGGCTCTCTGATACCTGTCTTC
TTTTTCCTAATCTTTTGGTAATTCAAAAGGGGATTGGTGTCTTATTTTTCCTAATCTTACTAAACATTTT
ATATCTTGCAACTCTTTCTAAGCTTGGTGGCTCTTTCATATCAAGAAGCTCTACCTAGAGAAAATCCCAA
AATGCAGTCTTAAAATGTTTAGCAATGTTTTGGAAAGGTCCGAGAGAATTCCTTCAATCTGAGCATCTCA
AAAGAGTTTTTTGAAGTTGTTCATGACAAACTTTTATGAGAAGATTTCGCTTAGTTCCAACTTATGTACA
TGCTTGTTTAGTTATGCTTATTTTTTATGGACAACTGTTTTAGGAGCTTCCATTCTTACTTGCAAAAAAC
AGGCGAAAGACATAACTGATATAGGTTCCTTGAGGGAGGCTGCAAAAATCTTTGTTCCAGGTATAGATTT
CCTTTCTATTTCATATTTTGGGTTCAATATGCAAAGTAACTTTTTGTGATTTTTTCTAGACATTTTGGTT
TTTAATTATTTGGTTCTTTTGCTATTTTCTCATCTTCCCTTAGATTCACATCTCATCTAGCTGATTTTAG
TGTTCTGACAATGATAGGCGGTTCAACTTTATTTTCTGCCCGAACGTTTAAAATTAAGGAAGATGAAGGC
TTCAGGTAACTATGCTACGAGGGTGCTGTTTGTATATACAAAAATGTTCGATTGAGATCATAATATTCTT
AGTTAGTTCATTTAGATATTACATGTCATGTGTTCACCAAGTATATGTTGCATTTTAATACGTAACTCCA
AGATATGTGAACGACTATGATAGAATATATCTTTTATTGCAGAAACTTCTCAAATTTATTATGAAATAAA
TTCTGTAATTTGATGATGACGTTGTGGTCTGTAAGAGCCAATGCACATGATAAAAAGCTTGATTGAATGA
ATGCAAGAGACGGTAGCCGTCTACCTAAGAGTTAGTATTCTATAAGTTTTCTTGGCATCGAGTTATGTTA

GGAGGTTAGACAAATACTATGTGAGAATAGTCTAGTTGAAGTATGTGTGAACTGCCCTGTCCTAGACACT
TGAGAATTTAAAAAATGTTATGAAATAATAATATTGATCAAAGTAGGCAGCTGTGAATACTTTGTTCTAG
CCTTCTTTGGCTATATCAACTTTCATATTTTCATTCCATTGCCTCTGTTTCTTTAACTGCAGAACTTATT
ACTTCTACGAGTTTGGGAAGAACGAACAACATGTTGCATTAGTAGCAACTGTTAATAGTGGACAGGTTTG
TGATAAACAATTTACAGTACACAAATACAAGCTAAACTTCTTACTCTGATTAAGACCTGAGTTATAACAT
GGTAGAATTTGTTTAAACATTAATTGATTCAAAGCTAAACTGTTCATGTTAGCAAAGTCTTATGTTGAAT
ATGCCAAACATAACTTTAGAATTCTGAGTTCTGCTCGACGATTATGTTGGTCGAAATTTCTCCTTCAACA
CTGTCGTCCAAGCTTTCATTTTGACATTGTGCTTCTTTGTGATAGGTGTTTGTTGCTGGAGCAACTGCAC
CATTGTCCAAATGGGATGAAGATGGCATAAAACTCCGTTCTGCTGCCATATCTTTAACAGTGTTATAATC
AATTGTTGATAAATTTCATTTCATTAGCTTGTGAGTACTCCCTAAGACTTAACTCTCATGTATTCGATAG
CATATAAAATCTAGGACTATAATCTAGGCTAAAATCTTTGTAATCTAGACTAAATTTTTTGTAATTGTGA
GCCTGAAATGAAATTCCATTTAATATAACTAGGTCTTTTTGTAACTATGATCTTAATTTGATTTTGTTGC
ATTGTCTTGGAGTTCCTTCTTGTAGTTTGTCGAGGTCTTTTTTTTCTTGTTTGGATAGTTTTTGTTGTAG
GTTCTTACATTTTTCTCAATGAAAGCTCGGTTTTTTCATAAATAAAGAAAGAAAAAATTTATTTAAGATC
CTATGGATGTTTCTGTTGACACGAGTTAAAGTTTGATTTTTCCTATGCAAATACTGAAATGATCTTTGAA
TGTTCAGATTGGTCTTCAGATTGTTTCAACTTAATTTGTGAGAGTTATTTGATTGTGTTTGATCCTTCAA
TCTTTTTGATGGACAGGAGGAAGCTTGGTGAGTTTTCTATTTAGCAACGAGACAACTCTAGCTGAAGATA
AAATTTAGATTTTGTATAGAATTTCCCACACTTTTTTTTTTTTTGGTGGTGGTTGTAAGAATTGTCTTTT
GTAGTGGCTCTGCTGAGTGGTACTTGGTTCAAGTGGTTTTTCATTTCTTAATTCCCTTTGTTTTTATATG
GTTTTATCAAACTAAAAACTTGAGATTATTGGAAGTGGCAGCTGGTCTCAATGGGACTACCACACATTGA
ATGCATGGTTCAATTTATCTTCCTTTTGTCTTTTGATTAGCACTAGTTAAAATTTCATGGAACACATCAC
TTTACAATCACATGGATACTGGGCCTTCCTCTCTGTAATTTAGTTAATGGATCAATTTTATCTTTTTGAA
AAAGGGTATTCATGGTTTGGTAGATGAGCTGAACCATTTTTTTTTCCTAAAAAGATCAAATCGAACTACA
AATATTTGGTTCAGTTCGGTTCGATTTGACTCTTGTTCTCTTGCTCCTCTCTTGTCCTCTTGCCTTGCTT
TTTCCACTTCCATTTTCCCACGGTCTTGCTATCCTCTCTCCCGTTTTCTCATGATCTTGCTCTCCCTCTC
CTTTCTCACTCAATCTTGCTTTTTGGTCTTGCTCGATCTCATTCTCCAATACTTTGTCTCCTTCTACAAC
ACAAACTGAAACGAGGGAGGGAGTTGGGTCACCTATGCAGAATCTGATCTGCTGGAAAAGAAAACCAGAA
GTTGAGAGGGAGAGAAGAAAAAGAAGAAGAAAAGAAAAGAAAAGAAAAGAAAATGTTGAGGAAGAAAGAA
AAAACGTGCGGTAATTTTGTAAAATTACTTGAAGATGGTATAAGCCGGAGGTAAAAAGCTTAAGTTTAAA
AAGTAGGCCAAGACTCATTTTGAACTATGGAAAGTCTTTTAATCAATTATCTCTTTCTAACTTGGCACAC
ATTTATCCTAGATTTCCTAACTCAACTTTGAACTCCAAATAACTCCAAAAACATTTATTTAATGTAATCT
TGAAAAATATCTATTAGCATGGTCTAAAAGTAGTTCACATTAATGTTGATGTTCGCAAATATTCACGAAA
AGAAATTATTTTTTAGTTTATTCAGTATGATCCAAAGAAAATATCGTACACGTGTTTAAAAATTGGGTTT
AGTAGGCCAAAAAAGAGTGCGGCAGTTGGCAACTGGTCAAAATGTCACTTTTCTTCTCCCAACTGCTCTA
TAAAATGGTCAGATTCTTGAATGTTGGAGCCATAGACAAAATTTCGAAGTCTTAGTGTAAATCTCTGATG
GGTTCTTCCATTGTTGGAGTGGAATCATCAACTTCTTTGAGTTTCAAGTGGAGTTATGATGTGTTTTTGA
GTTTCAAGGGAGATGATACTCGTTCTAATTTCACTAGTCATCTTGACATGGCCTTGCGTCAAAAGGGTGT
CAATGTCTTCATAGACGACAAGCTCAAAAGGGGTGAGCAAATTTCTGAAACCCTTTTCAAAGCTATACAG
GAAACTTTGATTTCTATTGTTATATTCTCTCAAAATTATGCATCTTCTTCATGGTGTTTGGATGAATTGG
TGAAAATAATTGAGTGTAAGAAATCCAAGGGCCAGCTTGTTTTGCCAATTTTCTACAAGGTGGATCCTTC
CGATGTACGAAAACAAACGGGTTGCTTTGGAGAAGCATTGGCCAAACATCAAGCTAATTTCATGGAGAAG
ACTCAAATATGGAGGGATGCTTTAACTACTGTTGCCAACTTCTCTGGTTGGGATCTAGGAACTAGGTATA
TTTTTATTGACATTTTGTCTTTTCTTTTCCATATTTCATTTCCATTCCTCTAATTTTGTTGACAAAGCAC
ACTAACAAATTCCTACTTGAAATAATTTCAACAATACAAGTGACTGATGTTAATCATAGAAGATTCACTC
TCAAGATGAAGTTTAATTTTATCTACTATTTTTTCTCATGTTCATCAAGTTTTATTTGTTTACATATGAT
GTACTTAACAACAGGAAGGAGGCTGATTTTATTCAAGATCTTGTTAAAGAAGTATTGTCTAGATTAAATT
GTGCCAATGGGCAGTTATACGTAGCTAAGTATCCGGTAGGAATTGATTCTCAACTAGAAGATATGAAGTT
ACTCTCACATCAGATACGAGATGTGTTTGATGGCGTTTACATGATGGGGATATATGGCATTGGAGGCATT
GGTAAGACAACTTTGGCTAAAGCTTTGTACAATAAAATTGCTAACCAATTTGAAGGTTTCTGTTTTCTAT
CAAATGTTAGAGAAGCTTCAAAACAATTCAATGGCCTCGTTCAACTACAGGAAAAACTACTCTATGAGAT
TTTAAAGGTTGATTTGAAGGTTGACAATCTTGATGAAGGAATTAACATCATAAGGAGTAGATTGCGTTCA
AAGAAAGTCCTTATAGTTCTTGATGATGTGGATAAGCTCAAGCAATTGGAAGCATTGGTTGGTGGACGTG
ATTGGTTTGGCCGTGGTAGTAAAATCATTGTGACAACAAGAAATAGTCATTTACTTTCTAGCCATGAATT
TGATGAAAAGTATGGTATTCGGGAATTGAGTCATGGCCATGCTCTTGAACTTTTTAGTTGGCATGCTTTT
AAGAAAAGTCATCCATCAAGTAATTATTTAGACCTTTCAGAACGTGCGACAAGTTATTGTAAAGGTCATC
CTTTGGCTCTTGCTGTTTTGGGTTCTTTCCTTTGTACCCGAGACCAAACAAAATGGAAAACTATATTAGA
TGAATTTGAGAACTCTTTGAGCGAAGACATTGAACATATTATTCAAATTAGTTTCGATGGGCTTGAAGAA
AAAATAAAGGAGATCTTCCTTGATATTTCTTGTTTGTTTGTGGGAGAGAAAGTTAATTATGTTAAAAGTG
TGTTAAACACGTGCCATTTCAGCCTAGATTTTGGAATTATAGTTCTCATAGATCTTTCGCTTATTACGGT
TGAAAATGAAGAGGTTCAAATGCATGATTTAATTCGACAAATGGGCCAGAAAATAGTTAATGGTGAATCT

TTTGAGCCCGGGAAAAGGAGTAGGTTGTGGTTGGTACACGATGTTTTGAAGGTGTTTGCTGATAATTCAG
TGAGTAACCCTTACCCAAAGTATCTTTAGTTTTCACTTATTTCCTAGACTTCATGATGGAAGATGTTTGT
CAAATTAAGTTGTCAATTATGTTAAATTACTAAATTGATGTCTTTGTAGGGAACGATTGCAGTTAAAGCC
ATAAAGTTAGACTTGTCTAATCCCACGAGGCTAGACGTGGATTCACAAGCTTTTAGGAACATGAAGAATC
TGAGATTGCTTATCGTTCGAAATGCAAAATTTTCGACAAATGTTGAGTATCTACCTGATAGCTTGAAGTG
GATTAAGTGGCATGGTTTTTCTCATCGTTCTTTGCCATTGTCCTTCCTTAAGAAAAATCTTGTAGGACTA
GATTTAAGTCATAGCTTCATCAAAAATTTGGGCAAAGGATTTAAGGTAATTATATATCTACCTGTATTTA
GTTGGAAGCTTCTTCATTGATTTTCTTAGAAATTTTCATGGGTAGCTCTAGCTTAGTTTCTAAAGATTTG
CAAATATTTTATGTTAAAAACAACAGCTTGCAAACATAAAGAGAGTTATGTTCATGTGAAAAATATTTGT
TAATGCGATATATATGCTTTAATCACTAACTCGTTTACTTATGTAAATAACAGTGGTTTATTCTAAACCA
TAACTTTGATATTTTTTCTCTCATTTTGAAATAATGTCTAATTATGTCCGATCAATATTTACTCCTTTTT
TTTTTTTTTTGTTTCTTTTCCATGTATTTTAGGATTGTAAAAGGTTGAAGCATGGTGATCTTAGTTACT
CTTCTTTATTAGAGAAGATTCCCGATTTCCCAGCAACGTCAAATCTTGAAGAATTATATCTTAACAACTG
CACAAATTTAAGAATAATTCCCAAGTCAGTTGTTTCTCTTGGTAAGCTTCTTACTTTAGACCTTGATCAT
TGTTCAAACCTTATAAAGCTTCCAAGCTACCTCATGCTGAAGTCTCTTAAAGTTTTGAAGCTTTCTTACT
GCAAAAAACTTGAGAAACTTCCAGACTTCTCTACAGCTTCAAACCTTGAAAAGTTGTATCTCAAAGAATG
CACAAATTTAAAAATGATTCATGATTCTATTGGATGTCTGAGTAAGCTTGTTACCTTGGACCTTGGAAAA
TGCTCTAACCTTGAAAAGCTTCCAAGTTACCTTACATTAAAGTCTCTTGAATATTTGAATCTTGCTCACT
GCAAAAAGCTTGAAGAAGTTCCCGACTTCTCTTCTGCATTAAACCTTAAAAGCTTGTATCTTGAACAATG
CACAAATTTAAGAGTGATTCATGAGTCTATTGGATCTTTGAATAGTCTTGTTACCTTGGACCTTAGACAA
TGCACTAACCTTGAAAAGCTTCCAAGCTACCTCAAGTTGAAGTCTCTTACACATTTCGAACTCTCTGGCT
GCTGCAAACTCGAAATGTTTCCAAAAATTGCTGAAAACATGAAATCCTTAATGTCATTGCATTTGGATTC
TACTGCCATAAGGGAGCTACCTTCATCAATTGGATTTCTTACTGCGCTTTTGCTATTAAACCTTAACGGT
TGCACAAATCTCATCTCCCTTCCTAGTACAATTTATTTGTTAAAGAGCCTTAAGCATCTTTACCTTGGTG
GGTGTTCTAGATTTCAAATGTTTTCCCATAGATGGGACCCAACCACCCATCCAGTATGCTCTTTTTCAAA
AATTATGGAAACTTCATCGAGTTCAGAATTTCCCCATTTACTAGTCCCAAAAGAAAGCTTATGTTCCAAG
TTCACCTTGTTGGATCTTCGATGTTGCAATATATCAAATGTAGATTTTTTGTAAATTTTATGTAAGGTTG
CCCCTTTCTTATCTAGTATACTTTTGTCGGAAAACAAATTCTCTAGTCTACCCTCATGTCTTCATAAGTT
TATGTCCTTGTGGAATCTCCAATTAAGGAATTGCAAGTTCCTTCAAGAAATTCCAAACCTCCCTCATTGT
ATACAAAAATTGGATGCCACTGGTTGCTCATTGTTGGGTAGAAGTCCAGACAACATCATGGACATAATAT
CGAGCAAGCAGGTTCATCACTTCCCTTTCCATTTTCTTTGTTCATATCATGTTATTGTCCCTCATTAAGA
TTCTGTCCATATTAATTTGTATGCCATAGGACGTTGCACTCGGTGACTTTACAAGGGAGTTTGTTCTAAT
GAATACTGGGATTCCAGAATGGTTCAAGTATCAGTCAATATCAACTTCAGTAAGGGTTAGCTTTCGACAC
GATCTCAATATGGAACGAACTCTGGCTACATATGCTACTTTCCAAGTGGTTGGAGATTCACATCGAGGAA
TGGCCTTAGTTTCATGTAAAATATTCATTGGCTACAGACTCCAAAGTTGTTTTATGAGAAAATTTCCATC
ATCAACATCTGAATATACATGGTTAGTAACAACTTCTTCTCCAACATTTAGCACTTCCTTGGAGATGAAT
GAGTGGAATCATGTCACAGTCTGGTTTGAGGTTGTGAAATGTTCTGAGGTCACCGTCACTATAAAATGCT
GTGGTGTCCATCTCACTGAAGAGGTCCATGGAATTCAAAACGATGTCAAGGGGCCAGGGGTAATTTATAC
AGTTTTTGATCAACCGGACAAATTACCGAGCCGGTGAGTGAGATTATTGATAACAAGGCTTGATGAGTTG
TCCTGTTTTAGAAAATTTTTGCAAGAACATTATTTTGTCTATTTGAAGATATGATTCATCTAAACTTTTC
TTTAACACAGTTTATTTAAAAATCTATTTCGATTAGCATATCCATAATTGTTTCTTGAAAAAGGTTTAAG
GTTCTTACTCTTAACCAATTCAAACAGAGCAGTTGTTAGTTTACAAAATTTCTTGATATTATTTGTTTTA
ATTTGGTTTTGGTTTTGTTGTTATTATTATCTGAAACTTATTGCACATTATTTTGGTTGTAATCAGTTGA
ATATTGTTTTTAGTAAATTGTGGTGTTTCTTGTGTATGATAATGTTCACTATGGCACAGGGATGTTGTAA
AATCCTTTGCTCAAGAAGTATCTGCCAAATCAGATTGCAATGCAATTTTGCATGCAGAAAATTTTCCAGT
TTGGAACGATTCCAAAATGCAACGACATATGAATTTTCCCTTACATGTAACTTCTCAAGGTGTTACAAGG
ATACGTGGTATGGAAGGCATGGCAGAGACAATACTTGCAAACTCTATATGTAACAAATACGAAAGAAGTC
AGAATCTTTTCTCTGCAAAAAAAGTTTTGAATCATTCTACTGCCTTTCTTCGTGGAGATGGAAATGGACT
TTCTTGGGAAATGGTAGACAGTCCGATCTCAAGTGATAGATTGTCTTCCCAAAAATATCTTAGAATTTTT
GACGATCGCGATCGATATGGAGACCTAAATGATGTGGCTTGTGGGACTGGTAATAGGTTCCGTTCAAGAT
TTTTGAGGATGGATGATATAAAAGAAGATGATATCAGAGAAGAGCCTCGTTGGAAGTACATGGAAAGGTC
ATTCCAAACAGATCCTATATTATGACAAGGTTGTAAAGTTTTTAGTATGCATGGTTCTTTTGAGAAGAAC
ATCTACGTCAAGTGATTATTTTCTGCTTTCAAGATGTTCACTTCACATTATGCTTTGTCGAATTTGAATA
GGGATATTTTCCGTTTTTCTGCTGTGCTATTTAAGGGCCAAAAGTGATCTTGCTAGGGTTGCCGCATGTG
TGTTGTAAGAGAAGTATTATTGTATTTTGTGATAGAAGAACAAACCTCTCAGTGATGCAAAAGTTAATGA
AGCATACTCAGTTCTCAACATATAAGTTCATGAGGTTGTTGAATTACTCTTGAGAAAAGTTGTCCTTATG
GGGAGTATAGGACACTGTCGTTGAGAAGAGATTTTCATTCCTATGGATTTCTAAGGTAACATTGATGAGA
AATTATTCTAAAACTCAGTGGGAGCCTAAGTGTATCAAGTGAAAAGGTTTTACTTGAAGCAGGAAAGATA
ATGCCGTGGTGAAATGGGTATCATGCTTGGGGGAGCCTATGTAATTCTTCAGTAATATCCACATACTCAA
GGGTAGCCTTTGTTCTATTGAGAAGGAGTCCCACATCTAAGAGGAGTCTGGTTGATCGAATAAGTTAGAC

TTTATGTGTGTCACTACAATTGTCATTACTTTAAGTACAATATTGTAAACGCTTACCTTTTTCATATTAG
AGAATTTATATTTTTTAGACACACTGCCACAGAGATGACATATCACCGTATTAGGTCACCAAATTCTATT
GGTTCTTCTCTTAGTTTATTGTTTACATGATTGCCATTAACTCTTCATTTAACATTTTTATTTTTTTGTT
ACAGAAACCTCAAAATATAATTGAAACATAATCCCAAAGAAGGCCAAAATATGAAAATGAGAGTCCATCG
ACTTTAGACCAATGGAGTTCATTATTATTAATATTAATATTGGCATAGTCCCAATTTACAAAAGTCATTG
TTGACTTTCACTTGTGAATCATCTTTTTACCCTTTTCTTCCCTTCTCTTTCTCTGTTCCTTCCCTTTTAA
ACCAAAGGTTGATTTCATTGGTACGTCCCCAAATATGCAATATATTAAAATTTCATCAATACATGGGCAT
ATTTCATTGACATTTATAGACTTTGATATTGATCTTTGGAGTGAACTGACATCTTCATTATATTATAAAG
AAGTCTACAAAATATGAACTCTTTGGTAACCATTTCGTTTTTTTTTTTTGTTTTTTTTTTATTTTTAAAAA
TTAAGTCTAATTCCTTCCTATTTTATAACATGACCGTTTACATTATTCTTCAATACAATTCTTGAATTCT
TTGATAAATTTTAAAGATAATTAAAATAAATTTTCAAAAACTATTTTTTTAGTTTTGTGGCTTGTTTTAA
AATGCATTACAGAATTGAATATCATTGAAACAAATTTGTCTTCGTGGCTCCAAGTTTTTGACAGAAATTC
CTGAATTTTCTATTGCGCTAAGTCTTGAAAGGATGGATCTCGATAAGCTTTTTTATTTGAATCTTAGGGG
ATGTCGAGCAACTTCCAAGCCACTTCACTTTGAAGTCCCTTGAAAAATTAGAACTGTCTCGTTGTGTAAG
ACTTGAAGAGTTTAATTTCCTCATATTGGTGAAGAAATGAAATCGTGTGTACATCTCACGACCATTTGTT
TAGTAGAGACCAACATAAAGGAGCTACCTTTATCCATTGTGAGTCTTACTGGTCTTCGGAAGTTGATCAT
AATTTTAGGGAGAAATCTCACAAGTCTTCTGCTTCAGTGCTTACCTAATCTTGAGTGTTGCAGCATAATG
CAGCGCTCTAGTTTTACTAGAATTTCATGTCCACGTCCATGTTTCGTTGTCGTTTTCATTATTTTCTTCG
CAGGTATTACTTCCCAATTTACATAATATCCAACTCGGTTAATGCAATACATTTGTCTGGAAACAAGTTT
TCAAGTCTACCTTGCTGTATGCTTAATTTTGAGTTCTTAGTTAGTCTTGACATATTCGATTGCGAGTTAC
TTAAATGGAAAATTTTATATATTTAATATATTAATAAACAATATACATTATCTTAAATACATGTCAAGTA
TACTATCATACTAGACGGATAAATAGTGTCAACTATATAATTTTACTTGATGTATAAATAGTATCAACTG
TATTATCTTACTTGACGGATAAATAGTGTCAATTATATTATCTTACTTGACGTGTAAATAGTGTCAAGTA
TATGATCTTACTTGACGTGGAAAAAGTGTCAAATACAATGACATCTTACTTGACACATAAAAAGGCATCA
AGTATACGACCTTACTTGATCTGTAAAAAGCATTAAGTGTGATATCTTACTTGACACGGAAATGATGTTA
AGAAATAGAATACTAGACGGGTTTTTAGTGTCAAGTAATGTAACTATACTTAATAGTTGATTACTTGACC
ATTTTTAAAACGTCAAGTATAGGCTTACTTGATCACACTGTATTAATGTCAAATAATCTAATTTATGTAG
TAGTGAATTCAAGTTTTGAAAATTTAAAAGGAAAAGTAGTTTTGAAAAACTTGTTAGAAGTTTTTAAAAT
TTGTCTATGAATTCCATTGTGTTAATTTCTTTACTAAAAGAATTAGGACACATATATCCCTTCTGGAAAA
CAACAAATTGGAAATGAAATCCTTATAAAATGGGGATGAAGTTATACATTGTTGATAAATATTTTTCTCA
CGTACTAAATCTATTCTTGTGTATTAGGATTACGAGGATGAAGTTCAGTTTGGCTACAGGGAGTTCGTAT
TAGCAAATTGGGATATTCCAGAATGGTACAGTTACAAGAAAGCAAACATGAACAACTCAATAACATTTTG
TATGTCCGATGATGAAATTAGGAAACTGAAGGCCTTAGCTCCTTTTATTTTCATTGAAGGGAATGGACAT
CAAACAACATCCTACGATGATCACTTTCCAGTTAAGATTAGAGTGAGCCTCAGCGCCAATGGTTCGACGC
GGAGTCATTGTGAGAGGTTCTTTCATTATGCATCATTATGTGCACATATGTGGTTGGTTGTATTTGGTGT
TGAAACTTTCACCCAAATGGATGAAAAATGTAATCACACTGATGAAAGGGAGATTGATGTTGGGATTGGT
TTTAAAGTATTGTTTGAGATTATTAGCAGAAAAGCTGTGGTATTTATAATATACTGTGGTGTTCATCTCA
TTTATGAGGAAAATGATGATCAATCCTGATGTAGGGAATTGTAGACAAACTTTTGTGATTTTCTATGGAT
TTAATTTACAAAGCATGTATCTTTGACTCTATTAAGTATGTGAAGGAAAAAGGCAAAGCTGTGGTTGCAT
TAATAATAGCCAGTATTATTGTTCACCCAATTTATTTTACTTTCCTTGTCCTCTGCATGTTTTGAGTAGA
CTGTATATATAGAGTTACGTATTGACGGGAATTGATCTCTTAAGTTTGAAGTGGACATTCTTAGTTGGTT
CATATCAATGTTATATGTCGTCATGTCATTTGTTCTGTTCTCTGGCTGTTATAAGCTTGATAAACATCTT
CCAGAATTTTATGAAAACATGAAATCTTTTAAGGGTGATAGAATCTGAATGTGGTATGGTTGCCTTCATC
AATTGGATATCTTATTGAGCTTGAAAATCTTAACCTATGTAATTGCTAAAGCCTGATTGCTCTTTCAATC
AAGTAAGTTTCATTTGTCAAAAGTTTTTTTTTTTTTTTTTTCTTTTGGAAAAGATACTAGGGTCAGTTGG
GTGCACCAACTATTAGGTTTATTTGACGCTTTTTACCTGTCAAGTATTGGTTTATTTGACGCTTTTCAAT
GTGTCAAGTAATCTAGTGTCAAGAATAAGTGATGTTTACTTGACAGTTTTTAAGTGTCAAGTATAATTAT
ATTTGACAGGTAAAAAACGTTAAGTATATAATTATACTTGATAGTTTTTACGCGTCAACTAATCCAGTGT
CAAGAATAAGAGGCTTTATTTTGACAGTATTTACGCGTCAAGTTAAATTATACTTGACAGTTCATAAGTA
TCAAGTATATAATGTTTGTTGACATTTTTTACACGTCATGTCAAGTTTTATCATGCACCTGTTTTGAAGT
TCAATAACATTAAAATACATATTAATTGAACAATTAAAATTAACTCAAACTTTACATCAAAGAAATTCCA
TATATATAACAATGAACCACATATATAAGTAGGTGATTACATACTTAATTACAACATATATATGTGCAGT
CCAAACTTCTAACAATAGCCTAAACTTTTCATCAATACGATATAAACAAAATATATTTGTAGCCTAAAGG
TGTACCATATCAAAACTAGTTCACCTATATCCACTATCAACAAAACTAGTTTACCTATACTCAGTATCAA
CAAAATAACAACAAACTAGTTCCCCTATATCCAGTATCAACAAAATTTGTCTATCTAACACAAAAAAAAC
CTTAGCTACCACCATAATTAATCACATGTGGCCACCCTAAAATTCAGCAAGCTCCATTTGTACTTCATCC
AACTCGAGTTGGCTGAGGTCCTTGTATCAATTTGATATGAAACCCAAAATATTGAAGTTAATACTTTGAT
TACTTGAATTATATAAAATAATTCAAACAATCATTTAAACATAACATACCGAATCTGTGACTACTATGCT
CCCATTTGTGATTATATCTCTCATGTATCTTATCACATAGTACCCACATTCAACCACTCCAACTTGCAAA
GGACACTGTAAATACATAGTACCCAATATAATGCATAATGTCAAGAAACAACTACATAGTATTCAATCTA

GTGCATATTGCCAAGAAACAATCACATAGTATCAATATAGTGCATATTGTCAAGAAACCAATCACATACT
ATCCAATATAATGCATGAATATATGAAAAAGAAATACAGAAAGTAGGAGAAAAAGGGCTAGAGATAAGTC
GAATAGAGAGTTATACAAAAGCATTTATGTTCAAAGCAATAGAGCTAGGACTATAATTTGAAAAGTGCTT
TTGATCTACCTAGTCCAAAATCCTGTAAGAGCTTGTATGTCATCCCATAAGACAATAGCATTGCGCTCCA
AGCCCTTAAAAATCCTATTGTTGCATTCCAACCAAATGACCCATAAAGTAACAGTCATGGTATTAAAAGT
AATGATCTTTTTTCTGCTGTCCGATTGCAAAGATCTTTGCAAAGAGAAGCAACATTTGTATATAGCACAT
AAGACCCAATTCAAAATATCAGAAACCTTCTTTCAAAGATCATGAGAGAATATTTGTACACAGCACATAA
GACCCAAACATGTTCCTCAACCCAAACTGGTGCATCTCCTGCCGATCCTCAAATGAAGACATGAATCATT
TATTCATACACTGTCCTAAAGCCCACAAGCTTTGGCAACTATGGAGTTCTGAAACGGGCATTCCTGTGAC
TAATACAAATGTAAAAGACCTCTGTCTGAGCTTATGCAGGTTAACAGGCAGTAGTGCAAAGAACATCATC
AGTTTCAATGCGGCCATAGTTACCTTATGGACAATATGGGTAGGGAGAAACAAACTTATCTTCACAGAGA
AAGATTCAACCTACCAGAACACATGGGAAGAATTGCATTAAACATAAATGCTTTCTGTAATCTACCTATG
TAAAAGGAAAACACTTTGTACTTCCTTTTTTGGTAATAAAATTTATGATAGGGTAGCTCTTCTCTAACCC
CCTCACTAGTATCGTTTCAAAAAAAAAAAAAAGATCATGAGAGAATATGGAGAGGTGGTCTAGATATTCCG
AGTGTCTTTTGCACAGAGAGCACCAACTTGGGTTGAGATTCCAGCATGGAAGCCTTTTTTTGAAGATTAT
CAGTAGTGTGAATACATTCATGAACTTACATTTTTTAGGAATACTAGATTTCCAAAGGTTCTTGAAATTG
ATAGAATTGATGCCAGAAATATCACTTTTGTCAATCAGAAGAACCTCTTTGATGGATGCAATATCAAACT
GACCATTTGAATTAAGTTTCGAAATAGGCATATCAAGTCCTTGGCTTTGCTTTGGTGCAGAGAGCTCCAG
TTTAATCGTATTCCACATGTATTCTTCGAAGCTTCTAAGGGGCCTTTTGGGTTGGAAATCCCAATCAGAT
TGTGTCTGATTCCATATGTCATTTACATTGTCCAGTTGGAGATTAGAAATAGCAAAAAGCCTATGGGCAT
ATGCAGAGAGGGGAGATTTTTCGTTCCAAGCCCCATTCCAAAAAGAGATAGATTCCCCATTATTGATTTT
CCAAGAATAATGAGGCAAGAACCGATCAACACATTTAATAATGGATCTCCAAGGAGCTTTTGAGCTACTA
TATTTGCTTAATAGAAACTTACCAAGAAACTCTTGATTGTATTTAACAAAGATAAGTATTCTCCATAGGG
ATTGCTTCTCAATGATGTACCTCCAAAGCCATTTACAAAGTAAATGCAAAATTATTGTTCATGGGACTTG
ACATGCACCTCCTAAGCTATTTGTCAAACGTTTTAGACTTGACATGTTCCCCAAAGGTCAAAGTTTATGT
TCCAAGTGCAGCCATTCGAATTTTATTCGACATATTATCTAAAATTAATTTAATTGTTAGTTAATTTTTT
GTCATAATTTTGTTTATTTTTAATTTTGTGATTAACTCCCTTTTTTATTTAACTATTTTTTAACTTTTTA
AGTTTTGTAGGTAGTTTGTTGATGGTAACTTTTTTGAATTTGATTTTGGTTATTAAAGACTCTATGCCAT
TTTGATATGAATGTGAAAGTTGGTATTATTTTCAAATTAAAAAAAACTAGTCATTTTGAATTTTTAGCCA
TTTGAAAAAACATTTTTGGCTATAAATAATCCTTCTTTTTCATCAATGAGACACAACACAACAAAAAGAA
AAGGCCACACAAAAAACCTAGAAATTTTTACAACCTTCATATGCTCTCTTTGTCATCTAAATCTCTAACT
TCCATACCCCTACTTCTTTTAACCTTCATACCTTCTTTAAAAAAAAAGAAGAAAAAATATACAATATCCA
TTTTATCTTCTATTCTAGCAACCCCTAAATCTTCATCATTCTCTCTACAATGCCAAGAATCCAACACACA
AAAAACTTTAATTTAGTTGGAGGTGTATGATAGAGTTTTGGTCCAAATGATCCACATCTCAATAAAAAGT
GAGTTTAGTGTGTTGAGATTTCAACATTTCTTTCTTTTCTTTTTCATATTCTTCTTTTTATTATTATGTT
TATTTTCCTTCAAAGTTCTTTATTTTTTATTATTCTTACTATCGTAACTATATTATTTATCTTTTCTCAA
TCTTCCATAAAAAATGTAAAAAAAAAGAAAAAAAGAAAAAGATAAAAAGAAAAAGAAAAAGATAAAAAAA
AAAAGACTAAATGATTAAATAGTTGTTTATTATTATTGTTCTTATTTCTCATATAATTGTTTATTTATTA
TCTTGTGAGCTTTTCTTTTATATTTTATTTATTTATTTATTACATATTTCTTTGTACCTTGTTGTTATCT
ATTATTTATTTATTTACAATATTTTATATATAATTAGCATTTTTATTATTTAATTCTTTTTTCTTTTTAT
AGCATTTATTATTGCTTCATTAGTATTTTCTCATCTTGATGATTGCTTCTTTAATATTTTTCTCATTATT
GATTATTGCTTCTTTAATATGTTCTTATCTTTTATATGTTATTTAATATTTTTCCCTCTTTTGTGGTCAA
TTTAATATGTTATTGCTAGTAAATTTTAAGTTTGTTAACATTTTAAATTATTTTTCTTTAAAAAAATCCG
ACAATTGAATCTAGGAAATTTGAGAGTCCAATTTCTTAGAATTTTTAAGGTGAGGAAATCGATTATTTGG
AAGTTCGAGATCGATCTTCAATATATTTTTATTAAAACCGCAATATGTTATTTTGTGTTTGCATAATTTA
TTATGATGCTTCTTTACTGTTATTTCTACTTATCTAATTTTCATATTAAGTCTTATTTCATTTCATATTT
AAAATTTTCAACTCTTTAGAATTAAATTTTTCAAGTTTTAAAATGCTTCTAATTTAAAATTCTCCAAGTT
TTAAAATCTTTCACAAAACACTTTTTTTTTTTAAAATTATTTAGATTTTTTTTTCTTTTAAAGTTATATT
TTTTCATGTTTTTCAAACGTTCAAAATTTAATCTATAATTTCATAAGGTTTCTTAGTCAATCTTTTTTTA
ATTACTTATACCATTTTTCTTAAACAAAATCATATGACGAAATCTATGAAATTTGGGAGTCCGATTTCCT
AAAATTCTTGAGGTAAGGATCATATCTCTAGGAGTCCAAGATTTGATAAAAAAAATATTTTAATTAATGT
TTTTTAAAAAATCTCTTTATTAGAAGGCTATTCCTATGACGGATTTTGAGAAATTGTATTAATTCCTCAC
TTTCTTGAGGTGAGAAGGTATTCTTCAACATAGTCTAATTGATGGAACGTTTTCTACTTATTTTATGAGA
TAATTGCTTCAAATATTGGAGCAATGAGGGTAAAATGAGATATTAGTTTTTTAAAAAATTAAAGAATATT
TTTAATTCAAGATTTGGAATTCGGCCACTGTTTTCTAAACGGGTATTGTGGGGTGCTAACACCTTCCTTA
TTCACAAATAACTCTCAAACTCAACTCTAGTTTTCGCATTTGCACACCATTCTTTTTATGATTTTATTTT
CAAATTGTTTACTTTATTTCGGGGTCCAATCACACAATAAAAAAGATTAAAAAGATTGGTGGCAACTCCT
ATTTGTTTTGAAACTAACCCATTTTGAGGACGTGGGCCGCTCCGCCTTGTCTCGGGCCAAGGTGATTACA
TTGAATCTTGAAATATGCAACATATCAGATGCAATTTTTGTAGAAACTTTGTCTCGTGTTTCCACTTCTC
CTTTATACCCTCTCTCCAAAAATTTACATTCTTACGGTCTCTTAAATTAAGGAATTTGCAAGTTTCTTTG

AAACAAAACGCAAAGCGTTTCTGTTGTCTAGAACAGTTGAATTCTATTGGCTGCGAGTTGTTGGTTATAA
GTCCTGACTATATGATGCTTCGGAAAGCAGGTTCCCTCTCCTTTTCATCTCTAATAACTATTTGTTCGTG
TCATATATTATGCATTTCGAATCTCTTGTTACTTTGATTATATGCTTTTTAAATTTAATTCTATAGGACC
TTAACTTCAGCGACTTACCGATCATTTAAAAAGTTGTAGATAAAACATGATTTCTGCATTTGTTTTGTCG
TCGACCTTTGTAGGATGATGGAGCTGGACTTTGTCTTGATGTAAATGGTAGTTGGTTGGCGTTTGTACTT
TGTAGGATTACGGAGCTGAACTTTCTTTTGATGATCAAAGCCTTTGCAGTACGGTTTTGTCCTCTGATGG
GTAATGATATTTCCACTTCTTCATTGTATATTTAACTTCTTGTAGTTAATTAAAGAGTTTATAAGGCTCA
TCCCAACAACAGAGTTCTCTATTGACAAGGAAATACCCTGCTTAAACAAAGAACATTCTAAAGAAATACA
AGAAATGTCTCATGGTGGAGGCATTATTCAAGACCAAGGAAATTTCCTCTATCATGGGAGAGCATTTGGT
TGAAACATTCAAGGTTTGAAATTAGGTTATGGTGGTAGTTGAAAAACTACTTGAAGACCTAAGAAAGTTT
CGGTAGATATTCCTGTTAGTTGAAAAACTAATCAATTTGTCACTTCTTATTTATTAATTTTAAGGTGGTC
TAGTTACACAAAATATTATTTTATATTATTTGTAGATAGACTTTTTTTTTCCCAATTATTATTATATTT
GTATCTCCGTAGAAGAAGGCAGTATTAAACATAAAGTGGAAATGTTAACTTTACAGTGATGCTTCTGAGT
TTTTTAATTTGAAAAATAGAAATTATAAATGCAAAGATACATAGCAAGATTAGTTGCAAAAGGTTATTCT
CAAAGAAAAAGACATTGATTATGACAAAGTATTTGTTCCCGTTGCTTGTTTTGAAACCATAAGGTTGTCC
AATTACGCTTGTTGCTCAAAATAATTGAAAGATCTTTTAAATGGACGTCAAATCAACATTTTAGTGGATA
TCGAAAAGAAGTCTACTTAGAACAACAAGTGCAATTGGACAACCTCTCTTGGTTACTCTGTGAAAGGCCA
AGATAATAAAGTTCTAAAATTGAAGAAGACATTATACGGACAAAAATGTAAAATAGCAGAATCAACAAAT
ATTTCCTTCGTAATTGATATTTGAAGTGCCCTTATGAACATTCTTTTTATACTAAGACTAATGGTGATGA
AGATATTTTGGTGGTTTGTTTGTACATGGATTGCTTAATTTTATAGAAAATTGTGCAAGTATGTTTGAAG
TCTCAAGAAGACGATGACCTAAGAATTTGAAATGACAGATATAGGGCTGATTTTAAATTATCTTGGCATT
GAGGTGAAGTAGTTAGAGAAAGATATTTTCATCTCTCAATAACGAAATACTAAAAAAGTTCAATATGACC
AATTCTAAGTCTGACACAACTCTGATTGAAACTAGGACTAAACTATCCAAATATAAGGAAGGAAATGTTA
ATCCTTCATATAATTTCAAAAGTTTGGTTGGGAGTTTGAGATATTTGACTTGCACATCCGTAAAGTAATG
TCTATATAATTCTAAGTCTTCAGATAACATATATAAACATAAAAATTAAAAGTGCAAGAAAGTAATGTTT
GTATTGTAATCAATCCATCCAAATGTGAAGGAGAATACACAAGTCTTCCAATAAGGTTTTATTTTTATTT
TTATTTTTTATTTCCACTCAAATATTTTATTTGGGGATTGAACTTACAGAAGTAGTTTTCAAAATCCTTT
TTACTTAGCATTGGATTATTTGTTCAAGTGTTTCTATATCAAAGTTGAAAACCATAATTGAAGAGAAAAT
TAGGAGAAAACAAATATATATAATTACTCAAATATCAATTCCTGTTTTCACTTCATTAACAATGCTGTCT
TGAGATAACATTTTATTTATATAAATAAAAATTAGGAAGTGCAAAAGAGTAATCCAATGCTAACTCTAGA
GCCTCTCTCCAGGTTTGTGTATTCTAACTTCAACTTTGGCAAATTCTTCTCCAAATTATTCCTCCATGAT
CACGTGAACACCACACTTCTTTATACTTACTGCGTTTGGAGTAATTACCTCAAATAACACCGTAATTCTA
TCAAAAATAATTCTTGAGGACTTAATCCTAAAACATGGTTGATTTTGTTCATAATGATCATATAAGTAGA
GAAATCTGTACGGATCAAGTACTTCTATCCATAGATACTCACCTCTAGGTCCTTTTCCACCTTCAAACTC
GTGCTTCCAAAATTGGCCGAGATGTACATCAAAATCGTTGATAAACACTCTAGAATTGACCTTAAATGCC
TTGTCAATACCTTGTAATTTGACACAAGAAGCAATAGAAACCTTCCTTTCCCAAGTTGGATGATTAGCTG
GAAGAAGAAACGTTATTGAATTGTTTGTACTCTTGTACTGGCACCAATCTGGAATATCACAATTCATTAA
TATGAGTTCTTTGATCACGTTAGGGTTATATCTCCTTTTCTGCACGAAGAGAGTAAATAGCTAAAAGTTA
TATTAATTAACAAAGTACATAAACTTAATTCTTATATAAACGAAATACATGAGAGATGAGGTCATGAGAA
AGAAATGATACGTACCGCATAATTATCATAACAAGATATGAAATCAAGTATGTTGTCAGGAAATCTGGCC
AATGATGCGCTCTCTGTAGCATTCATATAAAGTACTCCTTTTGGAACCTTTGGAATTTCTTCAAGCAACT
TACAAAAACTTGTAGAAAGATATATCAAGGACTTAAAACTCGTAATACATGAGGGTAGTTTACAAAAGTT
GTTTCTAGATAAGTCCAACTCTTTCAAAAAAGGGGCAACATGAACCATTGTTTCTAAGAAATCCAAATTT
GTTATCTTACAATCAAAAAGCTTTATTGATGTTAGGTAGGGAAATAAGAAAGGTGAAGAAGGATCATTTA
AAGAAGGAAAGGTTGAAAAATTATATCCTAAGACCCTCAAACAAGTAAGATTACTTAAACGATAAATTGT
ACTTGGAAGAGTTTTGAGCCCCTTGCACTTTATGATCCACAATTTTTTTAGGCTAGTGATATATCCAATT
GTTGGAGATAGCCGATTAATTACAGTACTATTGTAAATCAACAATACTTCTAGGCTATCCATTTCTTCAC
TAAATTGAGGACACCATTCATCTATTCTACATCTGTACAAAAACAAAGTTTTAAGGGATTTCAACTTGAG
GTACGATGGAAACTGCTCAAAGCCCTTAATATTGCTAGAAAGATAAAACTCGACGAGCTTACTAAGAGAT
CCAACTGATTCATGAATCTTTACTAAATTTACACATCCTAAAAGATCCAACTCTTCGAGGTTTATTGCAA
TAGATAAATCTGGAATTTCCACTAAAAACTTGGAGCCTCTAAAATTAATTTTTTTCAAGCATCCACCGCA
CTGCAAGTAATATAAAAAGAAAACATAATCAAATTTCAAATAAATAAGATGACATTGAAATTAAAATAAT
AGAACTCAAATATTATTGACAAAATAATTAATACCATGAATGCTTTCCCAAAATGTTTGATGGAGCTATA
TGGCAATTTCAATTCCATAAGGTTATCCATTGTGTAGGTTGAAGGCAAAGATGAAAAAGGAAAATGAGGC
CAATTCATCCACCTCAAGCTACTTGGTACATATTCCAGATCACTACTTCTTGAAGATGTGGCATTGCGGA
TATCCAGTACTACCAACTTTTTCACTTTTTCAAAAGCTCTTGAATCAATTTCCAACTCTGTAGGTTTAGG
AAAATTTAATTTTATAACTTTAACTGCTCTTGCTTCCTACAAATTATCAAAACAAACAATTCAAAATAAT
AAAAGTTGTTCTCATAATAAATAAATATAAGCATATATAAAATATTAGTTTTATCTAATAGTTCTTACCT
TATTGCCATTTAAGACATCCATAACATCATCTTTAATCAACAATCTTTTTCTTTTATGAGATTTAGAAGT
CTCCGAAAGATGAATTGTGCGACCCATTTGTTGTATTAAGTCATGCATTTCAATCCGGTTGGAATGTTCA

TCAATGGTAAGAAGTGATAGATTCATAAGTTTTGTTGTTCCCTTTTCCAAACATAAACAACCACATGCTT
CTAACTTCGTTTTAACTTCGTAGATATCTTCTCTTACAAAGCAACAAGAAATATAAAGAAAAATTTCTTT
TACTTCGTTTTCAAGTCCATCATAACTTATTCGAAGGGGATCTTGGATGCCCTTGTCAAGGTATTGGTTT
TCATATTCATCCAATATAAGTTTAAACTTGGATTGTTCGGTAGAGAAAAGGAAGGAACCTACAACTTCAA
GAGCCAAGGGAAGACCTAAACAATAACGTACGGCACGTTTTGAAAGGTCTAAATAATCACTTGACGGACA
ACTCCTCTTAAAAGCATGCCAACTAAAAAGCTCAAGGGCTTCATAACCATCGTTCAATCCCTGGACACTT
TGCAATATATTAAATTGATGAATAGCAAGTAAATGTTTGTCTCTTGTTGTCGCAATGACCATACTTCCAG
GTCCAAACCAATCATGTCCTCCCGCTAATACTTCTAGTTGTTCTCTCGTATCAACATCATCAAGAATTAA
AAGAATCTTTTTTGAGCATAGTCGATTCCTTATGATGTTAATCCCTATATCAAGATTGCTAACATTGATC
GAATTATCCATTAGAATCTCACAAAGTAGCTTCTTTTGGAGTTGAACAAGGCCATCATATTGATTCGAAG
CTTCTCTAATTTTTGCCAAAAAGCAACAACCTTCAAAGTCATCAGCAATTCTATTATACAATGCTTTGGC
CAAAGTTGTCTTGCCGATACCTCCAATTCCATATATTCCAATCATAGTAATTTTTTCATCTGCTGACATA
ACTTGGAAGAGTATATTATTAACTTGTCTATCTATTCCAACAGGATATTTAGGTAAACGCAACAACATTG
TTCCACGATTTAATTTCTTCAAGACTTCTTGAACAATTTCTTGAATCAAATTGGCCTCGTCACTGTCATT
TTAGGAAAGAAACATACACTAAAAGTTGATTTCAAATATCTAATATTATAATTAATTTCTATAATATGAG
AAGCCAGAGAAAATGAAAGCAACATTATAAAGTCAAACTAAATCCAACCAAGTTATAATATCTATTATAG
AAAGGAGGAGACAGCACAATATAATAACAAATTAAATAATATAAGAATTTATGATTTGTTATTGCCTTCT
TCTACTATTTTTAAAAATTAAGGCAAATAAGTTTTGAAAACTATAAGGTGTTTTCCCTTAGTCCAGGATG
CTCACTGTGAGATACATAATATAATAACACAATAGAATAAGAAGAGCAGAAGAGTAGAATTAAAGATATA
TATATAAAAAAGAAAAATACTCATTTTGAAGAATCGGCCATCCAGACATATGAGAAACAGAAATCATTGC
CTCCCTCCACGCTTCCATCTTGTCCGATGAGAATCTCACTTCGAGTTTTCCAAATTCTTCTCCAAATCTT
CCACTTTGTTTTCTTACTTGAGATGGATCCACTTTGTAGAAAATTGGTAAAACAAGTTGTACTCCCCATC
TCAATTTGTTACACATAATGATTTTCTCTAGTTCATTCAAACACCAACTTGAAGATGCATAATTTTCAGA
GATTATAACGATCGAGATCTTGGATTCTTCAATAGCTTCCAAAAGAGATGCAGAAATTTCTTCACCCCTT
GAAAGCTTATCATCTATAAAAACATTGATTCCTCTTTGACGCAAAGCCATATTAAGATGACTGGTGAAGT
TGGAACGAGTATCTTCCCCTCGAAAACTTAAAAATACATCAAAACTGCATCTAAAACGTGAGGAAGAAGA
TGATCCACTTGCTAGATTCATATTAATTAAGTAATTTGAAGGACAAATCAAAGAACCAGAAAGGAATGGA
AAAGTAGAAGGATAATCAAAGGTGGTGCCTGAGTTTTTGAAACGATAAATATTCTTATAAAACATCCATA
AGTGTGTGGATCCCATCTAAATTGTTCCTTTTTCTCTCTTATCATGTCGGTTGTTTCTTCCCAAGAACTT
TCCTCTGTTTTTTTTATTTTTTATTTTTTTGACTTCGAAACAACGTCTTTTCCCCCAAGTCTCCTTTCAT
TTTCTATTTATTTTTTAGTTTAAATTTTATTCTATTGGTCGGTCTCTACAGTTTAAATTTATTCAATTTT
AATCAAGACAAATTTATATTTTAGTATAGTTGAATGAAACAATGATCACTTAAAAAGTGGGTTCCATGTA
ATAACATTTGGTGTTATTTACTAGTGTACGATATGAGACGTTGATACCATATGAAACATGCTATCTATTT
TTGTTTAATTCGATCTTGTTCCGTAATTTTACAATCTTATTAATATGAAGTGGGACTTGATGTAGCGCTA
GCCTCGACATTCCAGTGCCTAATGGCAGAATGCTTCTGCCTTTTTGTGTAAATGACCAAGTGCTGCACGA
CACTGGCCTTTAATTTGGTGTCGCGTTGCTCTGAACATTCGACGGAAAGAGAATGAGAGCAGCGATGTTG
TTGTTGTTGCACTTCGGTCTTCAATGCAAAAAATTGTCCAAGTCCGACAAATAGTGGTGAAAGAAACGAG
GCAACAACTGATTTCTAGGGATAGCATTCACCTCTTCTTTTTTATCATGCTTCTTTGCTTCCTCCTCTAT
CCTTAGCCTCGTGATTAGGCTTTCCAGTGAGAACTCTTTGGTTTTGTGCCTTAGAGTGTTCTTAAAATCC
TTCCACAGTTGAGGTAATTTATCAATAATAACAGCAACTTGAAATTGATCATCGAGTGGCATACCTTCAC
TAATAATCTCGTGAGCTATTTTCTAGATTTCATATGACTGCGCCTCCACGGATCTGTCATCAGTCATTTG
ATATCATAGGTATCGGCTGACATCGTACTTCTTCGATCCCGCTTCCTCAGTATCGTACTTCTTTTGTAGC
GCGTCCCACACTTCTTTTGCATAGTCATGGTACTGTAAGAATCATATAGTTCATCAGTATGACCATTAAG
AATTAGGTTCTTACAGATGAAGTCAGTTTCTGTCCAGGTGGCGAGGTTCTTTAGTTGTTCTTCTGTAGGA
TCTTTTTCTGAAACATTTGGCTTTTCAGGAGTACAAGCAGTGGCCACCTTCTTCAGCGTGAGAAAAAATA
ACATATTTTATTTCTACCTTTTGAAGTGTGCTCCTTCAAAACGGAACGAACGATTGAGATCAGAAGACAT
CAAGTTGGATTGGATTTGTCCGATCATCACAGCAAAAAAGAAGCGTCTTAAAATTGTTGTTGCACTTCAG
TCTTCAATGCAAAAAATCGTCCAAGTTAGGCAAATAGTGGTGAAAGAAACGAGGCAGCAACTGAACGGAT
TGTTAAAAGGAACAGAGAATGCAAAACAGAAACTGGATTAGTGAAAGGTTGAGTCGAGGAACACGCTCTC
TTTAAGACGTTTCGCAACTCTGATTTAAATCGTGCAAATAGAATTATGCCTCGTCGTCCCTAGGATAAAA
CAACCCTTTTTCGTCAATTAGTTTTGCATATAAACTAATTAGAATTGAAATACTAAAAAAAGAACACAAC
TCAAAAAACTTTGTAAGAAATGAGAAGAATGTTTTGTTGTGTGTTTTGAGAATGAGAGTGAGGGATCTAT
TTATAGTGTGGGTGGATTACCAATGGTCGTATTCACAACGGTCACAAATTAATTTTGTAAACGGTCAATT
TAATTCTTAACGGTCAAACGGTCAAAAAATTAATTTGGTAACGTTTAACAGTCAAAAAACTAATTTTGTA
ACTTTTAACGGTCAAAAAATTAAGTTTTAATAAACGTTAAAACGTTTCATAATTCCTTACAAATTTTTCA
TCCAATAGACTTTGATAAGATATTATAAATATTTCTTTTTCGTCGCTAGCTTAGACCTATTTCATTTCCG
GTTGTTTTTTTACTCTTGTTTTCTCCTATTTTCTGTACTATATTTAATGTTGTTTTCCATCTTGAACGATT
CCTCATCATACAACATGTGGATAAGAGGCTAATATACTCGTTCTTAGCTTGGGTTTCTTAGATTCCTCTC
CCTTTGTCCTTTAGAATGTATGAGAACTTGAATTTGCACATTGTTATATATGTTGGGCGCATGAGAATGC
ATAACTTTAGCTTTGCACTAATTCTTTAACTTGTGACCATTGGATGAGAATTGGTTAGAAGCAATAGGTT

AGGTTAGCATGTAAATCTATTGTGATGTGTAGTGATTCTAACCAACTTAGAATTGAACCTTTAAATGTCA
TTTTTTTTAAGAGGAAACAGCAAGCTGGGACTTAGGACAACTCCTAAGACCGAGCTAAGCATAAAAATTT
ATTAATAAGGGGCTAGAGGAAAGCCCAAAAGCTAATTACAAAAGACATTCAGATTTAAAGCAATTGTAGC
AGCTGAATAATTTTTTAAAAAAGGACCTCTGCTGCACCAATTGCCTATGAGTATTTTGCACTCTTCCCAC
ATGTTTACAATAGATTTTTGATAGCTGAAAGTACCAAAAATTCTATTGTTTCTCTCAGACCAAATACCCC
AAAGGATAGCTATGAGCCCGCAAAAAAGAACCTTATGATTCGGGAGGGAGGAATTTAGAGAGAAGATGAA
GGAGAACAGAGCATTGAAGTCGTCGGGAGCAAAAGAGAAGTTGAGGGACCAATGGAGCGAGGACCACAAG
GGCTTCACTGCTTCACAGCGAAGAAAAAGGTGAACTCCCGTTTCACTGTCTTTATTGCAAAAAACACACC
AATTTGGTTGAAGAAGAGAATTTGGCATTCTTCGCTGGATAACCTCCATAGTATTTACCCCTTTCTGTAT
CAGACACCACATGAAGAACTTGATCTTCATAGGAATGTTGGATTTCCAGATATTTTCTAAAAGCTTTGCT
TGAGGGTCCCTAGGGGTTTGGTCGTATTGACGCGAAATGATTTTTTTAGCTGAGGCGATGGAGAAAGAAT
TGTTATTTTCAGATATCTAGGTAGGCTTACTTGACCCTCTGTTAGTCCTCGGAATAGGAAGGATATCCAT
AATTTTTTCCCAGGTGCATCTTTCTCTGTCATTTAGCTCTCTTCTAAAGTTGATTTAGATGTCAATTAGA
CACATGAAAATTATGTATTCCACATAAAGGACAAACTTAATGCTTTTTGGGATATTTAATATTACTGCAA
ATGAATTAGAACAATTAGAACTCAAGCTAGATTCTTTTATCCTTGAATTTTCACAAATTTCACTCTCTTT
TTATGGAAATTACTGTCTTGCCTCGATTTCAACTCAATCCCCCTTCGGTTACGTGGTTGAAAATTAATTT
GTTTGAGGAATTAGTGTACTGCTCTTTAAGATCGACCCGAACTTGCCCCTAAAACTAGTTTTGATGTGTG
ATTTGGTTAATTATTTGGCATGGGTTTGGTCGACAAAACTCGTCAAACCAACATGTCTAAATTTGTAAAC
AAAAGTGGCGAGTGGAGGGTAATCAATCTTATTGCACTCAAAAACTATTTATTATTAGTTTGTGCGATAG
AAATCTATCCTTTTTAGACACAATGACTCTAGACATAGGTGGCATATTGCGAATTAGGTTACCAAACTTT
ATCTATTATTCTCTTAGTTTATTATTTACATGATTAACTTTTTATTTAACATTTGTATTTTGTGCGATAG
AAATCTTAAAATATAATTGAAACATAATCTCAAAGAAGGCCAAAATATGAAAATGAGTGTTCACCGACTT
TAGACCAATGGAGTTCATTATCATTATTATTAATATTAATATTGGCTTAGTCCTTTACTAAAGTCATTGT
TGACTTTGACTTTTGAATCATCTTTTTAACATTTTCTTCCCTTCTCTTTCTCCGTTCCTTCCCTTTAAAA
CCGATGGTTGATTTAATTGGTACTTCCCCAAATATGCAATATATTAAAATTTCATCAATACATGTGGATC
TTTTGATGTAGACTTCAATATTGATTTAACGGGTGAATTGACATCTTTACTATTTGTAAAAAGGTCTACA
AAATATGAATATGAACTATTTGATAACCATCTCGTTTATTTCTTTTTTTATTTGTTTTTAAAAATTAAGT
CTACTTCTTTCTTATTTTATAACATGGTCGTTTGCATATGCAATCTTACTAGTATTTGATCATCCAAAAA
TAGTCTTCTCAAGAATTATAGTCAGGCCACAATATCTTTAGTCTTAACGCTCTTTGTTCTAAGCTTTTGT
AATCTAGATGGGCTTATCTCCAGCCTGTGTAACCTATTATTCTCAATAAAAATTGGTGATATGGCTTCCA
TATCACAAGTATCTTTTTTGAAAAAAAAAATATTGGTCATTTGCATCATACAATGGTTGAATTCTTTGAT
AAATTTTAAAGATAATTAAAATAAATTTTCAAAAACTATTGTTTTAAGTTTGTGGCTTGTTTTAAAATGG
ATTACAGAATTGGGAATCATTGAAACAAATTTGTCTTCGTGGCTCCAAGTTTTTGACAGATTCTTGATTT
TTCGACGACGCTGAATCTTGAAAGGGTGGATCTTAAAGAATGCACAAGTTTGGTGAAGGTTCATGAGTTA
GTTGGATCTCTTTATTTGAATCTTAGGCACTGTCGTAACCTCAAGCAACTTCCAAGCCACTTCAATAATT
TGAAGTCCTTTGAAAAATTAGAACTGTCTCGTTATGTAAGACTTGAAGAGTTTCCTCATATTGGAGAAGA
AATGAAATCATATTGGTGAAGAAATGAAATCGTGTGTACGTCTCACGACCATTTGTTTAGTAGAGACCAA
CATAAAGGAGCTACCTTTATCCATTGTGTGAATCTTGTCTTCGGGAGTTGATCATAATTTTAGGGAGAAA
TCTCACAAGTCTTCTGAATCATTGCTTACCTAATCTTGAGTATTGCAGAATAATGCACTGCTCTAGTAAT
TTACAGGACTTTCATGTCCATGACCATGTCCCTACGTTGTCTTTTTCATATTTTTCTTCGCCGGTATTAC
TTCCCAATTTACATACTTTGCAACTCAATTATTGCAATATATTTGTCTAAAAACAAGTTTTCCAGTCTAC
CCTGCTGTATGCTTAATTTTGAGTACTTACTTAGTCTTAACACTTGAAATGGAGAATTATGTATATTTAA
TATATTAATAAACGATATACATTTCGTTAAATATATGTCAAGTATATATACTATTTTATTTGAATCATAT
ATACTATTTTACTTGACGGATAAATAGTGTCAAATATACAATTTTACTTAACGTGTAAATAGTGTTAACT
AGGTATACCATCTTACTTAATGGGTAAATGGTGTCAAGTATATTATCTTACTTGACATGTAAATATATAC
TTGACGTGTAAATAGGGCAAATATACGATCTTCCTTGACGTGCAAAAACTGTCAAACACAACAACATATA
TTTAACACATAAAAAGGCATCAAGTATACGACCTTACTTGACACGTAAAAATTAAAGCATTAAGTATGAT
ATTTTTTACTTGATACGAAAATGACGTCAAGAAATAGAATACTTGATGGATTTTTAGTGTCAAGTAATGT
GACTATACTTGATAGTTGATTACTTGACGCTTTTTAAAACGTTAAATATAGGTTTACTTGACACTCTATT
AATGTCAAGTAATCCAATTTCTGGAGTAGTAAATTCAAGTTTTGAAAACTTAAAAGGAAAGTAGTTTTTA
AAAACATGTTTGAAGTCTTTAAAATTTGTCTAAGAATTCCATTGAAAGAATTAGGACACATATATGGCTT
CTGAAAAACAACAAATTGAAAATGAAATTATTATCAAATGGGGATGAAGATATATATTGTTGATAAATAT
TTTTCTCACGCACAAAATCTATTCTTGTGTTTTAGGACTACGAGGATGAAGTTCCATTTGGCTACAGGGA
GTTCGTATTAGGGAATTGGGATATTCCAGAATGGTACAGTAACAAGAAAGCAAACAGCAACAACTCAATA
ATTACATTTTGGATGTCAGATGATGAAAGTAGGAAACTGAAGGGCCTCAGCTCCTTTTATTGTCATTGAA
GAGAATGGACATCAAACAACATCCTACGATAACTTTCCAATTCAGATCAGAGTGAGCCTCAGTGCCAATG
GTTAGACGCAGAGTCATTGAGAGAGATTCTTTCATTATGCATCACTATGTGTGCACATATGTGGTCACTT
GTATTTGGTGTTGAAACTTTCACCCAAATGGATGAAAAATGTAATCACACTAATGAAAGGGAGATTGATT
TTGGGATTTGTTTTTAAAGTATTGTTTGAGATTATTATTAGCAGAAAAGAGATGGTATTTATAAAAAAAT
TGTGTGATTTTCTATTGACTTAGATTACAAAGGATGTTTGTTTGAGTATGTGAAGGATACAAAGCAAAGC

TGTGGTTGCATTAATAATAGCCAGTATTATTGTTCACCCCAATTTATTTTACTTTCCTTGTCCTCTGCAT
GTTTTGAGTAGACTGTATATATAGAGTTACATATTGACGGGAATTGATCTCTTAAGTTTGAAGTGGACAT
TCTTAGTTGGTTCATATCAATGTTATATGTCGTCATGTCATTTGTTCTGTTCACTGGCTGTTATAAGCTT
GAACATCTTCTACAATTTTATGAAAACATGAAATCTTTAAGGGTGATAGAATCTGAATGTGGTATGGCCA
TAAGGGGGTTGCCTTCATCAATTGTGGAAATCGATCTTATTGGGCTTGAAAATTTTAACCTATGTAATTG
CTAAAGCCTGATTGCTCTTTCAACCAAGTAAGATTCATTTGTCAAAAGGGTCACGAGGAACTTCATCAAA
ATGTGGGTGTTTCAGACTTGACATGTTTTCCCTGAGGGCAAAGTTTATGTCCCTGAGGGCAACATATCAG
ATACAAATTTCATAGAAACTTTATCTCGTGTTTCCACTTTTCTTGTCTACCCTCTCTTCACAAATTTACA
TTCTTACGGTCTCTTAAATTAAAGAACTGCAAGTTTCTTTAAAACAGCGGAAAGCATCCTTGATGTCTAA
AATAGAGGTGATGATGATACTCGAAACAACTTCACTAGTCAGTAGGTGTCAAATGAGTGTTCATGGATAA
AAGGAAATGAAAAGGGAGAAGTGGAGAGATACATAGCAAGATTAGTTGCAAATGTTTATTCTCAAAGAAA
AGGCATTGATTACGATAAAGTATCTATTCCCGTTGCTCGTTTGAAAACCATAAGGTTGCCAATTGCACTT
GTTGCTCAAAATAATTGGAAGATCTTTCCGATGGACGTGATATCAGCATTTTTGAATGAATATTATCTAG
AAGAAGTAGTCTACTTAAAACAACCTCTTGGTTGTTCTGTGCAAGCCCAAGAGGATAAAGTTCTAAAATT
GAAGAAGACATTATACGTACAAACAAGAACGTGGAATAGAAGAATTAACAAATATTTTCTTAATAATGGG
TATTTGAGGTGCCCTTATGAACATTCTCTTTATATTAAGACTAATGATCATGGAGATATTTTAGGAGTTT
GTTTGTACACGGACTACTTAATTTTTACAGAAAATTATGCAAGAATGTTTGAAGGTCTCAAGGAGACGAT
GACCCAAGAATTTGAAATGACAGATATATGATTGATGTCATATGTTATCTTCGCATTGAGCTGAAGCAGT
CAGAGGAAGGTATCTTCACCTCTCAAGAACAATTATACTAAAGAAATTCTAGGGAAGTTCAGTATGATCA
ATTCAACTTCGATTAAAACTGAGATCAAACGGTCCAAATATGAAGAAGCTAAGGAGATGTTAATCCTTCA
TATTTCAAAAGTTTGGTTGGGAGTTTGAGATATTTGACTTGCACAGGACCATATATTCTTTTCACCGTTG
GATTGGTGAGTCGATTTATGGAATAATCTCTTACAACTACTCATTTGAAAGTGGTAAAGAAAAATTCATCG
TTACCTTAAAGGTATATATGCTTGACTATGGGTTGTTTTATTTTTCATCTAAAGAAAAGATAGAAGGATA
TTGTGATAATGACTGGACTGGAGATACTAATGATCAAAAGAGCACTAGCGGATATGTTTTCTTCGTTGGT
AATACTGCAATATATTTACTTGGAGTTTTCTAATAAGCAAACCTATTGTAACATTATTGACTTGTGAGTC
AGAATATGTTGCTGCAGCTTCATGTGCAGTTTGGTTAAGAAACTTGTTAAAGACAGTTAGAATTTTGTAA
GATGATCCAATTGCGATCCATATAGACAATAAATCAACACAATTGCTTTAGCAAAAAATCCCATGTTCCA
TGATCGTAACAAACACATTGATATAAGATTTCACTTTATCAGAGATTGTATTTCAAGGAAGGAGGTTCAA
GTTGAATATATGTGAAGATTGAAGATCAAATTGCACATATTTTCATGAAGCCACTCAAAATTGATGTCTT
AAAAAATTAAGAACTTTACTTGGTGTTGTTAATTAGAAAACGTGCTTAAGAGAGGATGTTGAAAAGTGAA
AGCATGTTTTAGTTAATTGAAATAAGTTACATACAAAATTAAGACATGTGAAAGTTATATATAATTAATT
CATAAATATATAATTGGCAAGATTGTTTAAATTAAATATGTTGATAATTAAGTTAAATAGAAAATTTAAT
TGTTATTTGAAGTCATGGATCTAGACAATCCTATAAATAGGATTGGTATGTTGTATATTGTAATCAATCC
AAGTGTGAAGCAGAATACACAAAAAAATAGTAAGTCTTCTGATTAAGTTTTTTTTTTTTTTTTTTAATTTC
CTCTTAAATATTCTATTTGGTGATGTCAGAAGGGAGTAGAGACAAGAGATAACGAATAGTGGGAATTATT
TCGGATTGAACCAAAAAAATCTAAGGGTCACTTAAAATCGATATTTGTTTATTTGTTTTTGAAAATTAAG
CATACAAAAACATACTGCTTCCACCCATAAATTTCTACAATTTGTTATCTACTTTTATTTCAATTATTTT
AAAAATTAGGTGATTGCTTGATAAAGAACTTAGACCAAAAGTAGTTTTCAAAATCATTTTTTTCTTAGCA
TTGGATTATTTGATCAAGTGTTTCTATATCAAAGTTGAAAACCATAATTGAAAAAAAATTTAGGATAAAG
CAAATATAAATTAAAAAAAAAACAAATATATATAAACTCAAATATCAATTTCTGTTTTCTCTTGATTAAC
AATGCTGTCTTGAGATAACATTATATTTATAAACATAAAAATTAGGAAGTGCAAAAGAGTAATTAGAGCC
TCTCTTCAGGTTTGCATATTCTAACTTCAACTTCGGCAATTTCTTCTACAAATTATTCCTCCACAATGAC
GTGAACACCACACATTTTTATACTTATTGCGTCTTTGAACTCTTGAGGAACCTCAAATAACACTTCAACT
TTATCTAAAATACCTCTTGAGGATTTCATCCTACAACATAGTTGATTTAGATCAACCTTCCCTGAGCTGT
CCATAATATCACCATATGGGTCGCGATCAACATCATATGGGTAGAAAACAACACCATATCGGTAGGGATA
TATGTAAGAACCAAGTACTACCATCGATAGATACTCACCTGTTGGTGTTTGTGTGGCGTCATATTTAGTC
GTAATTTTGCCAATGGAACTTAACATTTCACAGTGCAGTATTTCAGTATCGTTGATAAACACTGAACATA
AAAAGTTAGGTTCCCCGTCACCAATATTAACTTCAAATTTGGCACAAGCAGCAATAAAAACCCTACTTTT
CGAACTTAGACAATCAGCTGGAAAAATCTCTATTGAATTGTTCATACTCTTGTACTTGCACCAATCTGGA
ATATCACAATTCATTAATATGAGTCTTCTAAATTCTTCGTGTACTGCATGTTCCTGCACGATGAGAGTAA
ATAGCTAAAAGTTGTATTAATTAACAAAGCACATAAACTTAATTTTTATATGAACGAAATACATGAGAGA
TGAGGTCATGAAAAAGAAATGATACGCACCAAATTATCATCACAAGATATGAAATCAAGTATGTTGTCAG
GAAATTTGACCAATGATACGCACCCTGAAGCATTCATAGTAACTACTCCTTTTGGAACCTTTGGAATTTC
TTCAAACAACTTACAATCAAATGTATACAAATAATTCAAGGATTTAAAATTAATAATACATGAGGGTAGT
CTACAAAAGTTGTTTTCAGATAAATCCAACGATTTCAATGAAGGGGTAACGTGAACCATTGTTTCTAAGA
AATCCAAATTTGTTATCTTGCAACGGACAAGCTCTATTGATGTTAGGTAGGGAAATAAGGAAGGTGAAGA
AGGAGTATTTAAGGAAGGAAAGGTTGAAAGATCAGAATTAAAGATATGTAAATCATTAAGATTACTTAAA
CGATAAATTGTCCTTGGAAGAGTTTTGAGCTCCATGCAGTCTGTGATCGACAGGAATTTTAGGCTAGTAA
GATATCCAATTGTTGGAGATAGCTGATTAGTTACAATACTATAATGAATGACCAATACTTCTAGGCTATC
CATTTCTTTACTAAATTGAGGACACCATTCATCTATTCTACATTTGTACAAAATAAAAATTTTAAGGGAT

TTCAACTTGAGGTACGATGGAAACTGCTCAAAGCCCTTAATATTGCTAGAAAGATAAAACTTGACGAGCT
TACTAAGAGATCCAACCGATTCATGAATCTTTACTAAATTCACACATCCTAAAAGATCCAACTTTTCGAG
GTTTATTGCAATAGATAAATCTGGAATTTCCACCAAAAACTTGGAGTCTCTAAAATTAATTTTTTTCAAG
CATCCACCACACTGCAAGTAATATAAAAAGAAAACATAATCAAATTTCAAATAAATAAGATGACATTGAA
ATTAAAATAATAGAACTCAAATATTATTGACAAAATAATTAATACCATGAATGCTTTCCCAAAATGTTTG
ATGGAGTTATATGGCAATTTCAATTGGATAAGGTTATCCATTGTGTAGGTTGAAGGCAAAGATGAAAAAG
GAAAATGAGGCCAATTAATCCATCTTAAGCTATTAGGTAGATATTGAAGATCAGTACTTTTTGAAGATGT
GGCATTGCCAACTTCCAGTACTACCAAATTTCTCACTTTTTCAAAAGCCCTCGAATCAATGTCAAGTTGG
GTAGGTCGAGGAAAATCTAATTTTATAACTTTAACTCCTCTTGCTTCCTACAAATTATCAAAACAAACAA
TTCAAAATAATAAAAAAATTTCTCATAATAAATAAATATAAGCATATATAAAATATTAGTTTCATCTAAT
AGTTCTTACCTTATTGCCACTTAAGACATCCATAACATCATCTTTAATCAACAATCTTTTTCTTTTATGA
GATGTAGAAGTCTCCAAAAGATGAATTGTGCGACCCATTTGTTGTATTAAGTCATGCATTTCTATCCGGT
TGGAATGTTCATCAATGGTAAGAAGTGATAGATTCATGAGTTTTGTTGTTCCCTTTTCCAAACATAAACA
ACCACATGCTTCTAACTTCGTTTTAACTTCGTTGATATCTTCTCCTACAAAGCAACAAGNCACTTAAGAC
ATCCATAACATCATCTTTAATCAACAATCTTTTTCTTTTATGAGATGTAGAAGTCTCCAAAAGATGAATT
GTGCGACCCATTTGTTGTATTAAGTCATGCATTTCTATCCGGTTGGAATGTTCATCAATGGTAAGAAGTG
ATAGATTCATGAGTTTTGTTGTTCCCTTTTCCAAACATAAACAACCACATGCTTCTAACTTCGTTTTAAC
TTCGTTGATATCTTCTCCTACAAAGCAACAAGAAATGTAAAGGAAAATTTCTTTTACATCTTGTTCAAGT
TCGTCATAACTTATTCGAAGAATATCTTGGATGCCCTTGTCTAGGTAGAAGTTCTCATATTCGTCCAATA
TACGTTCAAACTTAGATTGATCATCAATAGAATTAAGGAAGGAACCTAACACTTCAAGAGCTAAGGAAAG
ACCTTTACAATAATGTACAGCACGTTTTGAAAGGTCTAAATAATCACTTGAGGGATCACTATTTCTAAAT
GCGTGCCAACTAAAAAGCTCAAGACCTTCAATGGCATTCAATCCGTTAACTTCTTTGAATTTATTAAATA
TTCCATGACTAGAAAGTAATGGCATGTTTCTTGTTGTCGCAATGACCATACTTCCAGGTCCAAACCAATC
ATGTCCTCCTGCTAATGCTTCTAGTTGTTCTCTCGTATCAACATCATCAAGAATCAAAAGAATCTTTTTT
GAACATAGTCGATCCCTTATAATGCTAATTCCTATATCAACATTGCTAACTTTGATTGAATCATCCATTA
GAACCTCACGAAGTAGCTCCTTTTGGAGTTCAACAAGACCCCGATATCGATTTGAAGCTTCTCTAACATT
TGCCAAAAAGCAACAACCTTCAAAGTCATTAGCAATTTTATTGTATAATGCTTTGGCCAAAGTTGTCTTG
CCCATACCTCCAATTCCATATAATCCAACCATCGTAATTTTTTCATCTGCAGACATAACGTGGAAGAGTA
TATCATTAACTTGTCTGTCTATTCCAACTGGATATTTAGCTACACGCAACTGTATTGCTCCACGATTTAA
TTTCTTCAAGACTTCTTGAACAATTTTTTGAATCAAATTTGCCTCGTCTCTGCCATTTAAGAAAGAAATA
TACAGTAACAGTTGTTTTGAAATTCACTTATAATTCCTATAATATTAGAAGCCAAAGAAAATGAAAGCAA
CATTATAAGTCAAACTAAATCAAACCAAATAATATCCATTATATAGAAAGGAGGAGACAGGACAATATAA
TAACAAATTAAATATAATATAATAATGGAATAATTCCAATTTCACAAAATAATTCCAATGGCTTGGTATA
AATAGTTTTTAGTATTTGAATTTTTGTTTTTGAAAATTAATTAAGTACATGTGCCATTATTTTCACCCAT
AAGAATTTATGATTTGTTAATGCCTTCTTCTACTATTTTTAAAATTAAGAAAACTAAATAGAGGAAAATA
AGGTAATTAAGTTATTTATAAATATAGCAAAATTTCATTTTTTATAAAAATGTAAATCATAGTTGAGTCT
ATCGCAACCTATCGCAACCTATCACATGTAAAAATTAAAATTTTGTTATAATAAAGGTAAATATTTTCAA
CAATTATATATCTGTTAAAACAATTTTAAATTTCTCATATATATGTATGTATGTATATATATCTCTCATA
AAGTTAAAGTTAAAAATTCAAATGTTTCCTTAATAAAGTTCAACTCATGATAACTAAAAAATTGGTAGTT
ATATAACAAATGTATACAAATTTAAAAATGAAAAACTTGAAAATAAAATTGTTATGGAAGGGAACTTAAA
ATCACTTTTTGTTTGGATTTTCCCTTAACTCAGCATGCTCTCACCTTGAGATAGTCAAAGTATATATAAT
AACAATAGAATATGAAGAGCAAAAGAGTTAGAAAGATATATATGAAAAAGAAAAATACTCTTTTTGAAGA
ACCGTCCATCCAGATATATGACAAATAGAAATCATGGCCTCCCTCCATGCTTCCATCTTGTCTGACGAGA
ATCTAACTTCAAGTTTGGCAAATTCTTCTCCAAATCTTCCACTTTGTTTTCCTACCTGAGATGGATCCAC
TTTGTAGAAAATTGGTAAAACTACTTGTCCTCTCAATTCATTACACATAAGGATTTTCACCAATTCATTC
AAACACCAACTGGAAGATGCATAATTTTCAGAGATTACAACGATGGAGATCTTGGATTCTTCAATAGCTT
CCAAAAGAGATGCAGAAATTTCTTCACCCCTTGAAAGCTTATTATCTATAAAAACATTGATTCCCCTTTG
ACGCAAAGCCATATTAAGATGACTGGTGAAGTTGGAACGAGTATCTTCCCCTCGAAAACTTAAAAACACA
TCAAAACTGCAACTAAAACTTGAGGAAGAAGATGATCCACTTGATCGATTCATATTAATTAAGTAATCTG
AAAGATAAATCAATTAGAACCAGAAAGGAAAGGAACAGTAGAAGAAGGATAACTCAAATTAAGCTGGTGC
ATGAGTTTTTGAAACAATAAAATATGCTTATAAAACTACATCCAATAAACGCGTGGATCTAAATTGTTCC
TTTTTTTCTCTCATCATGTCGGTTCTTTCTTTTCAAGAACTTTCCTCTATTTTTCTTTTTTACCCCAAGT
TTTCTTCCTTTTTCTATTTATCTTTTAGTTAAATTTTTATTTTATTGGTCGGTCTCTACAAGTTGAGAGT
TGTTCGATGTTAATTAAGAAAAATTGTTTTAAGGCCATTTGGCAACATCATCGTTCTCTTTACAAACCCA
CGCGACTTCTTTCATATATCCTAAGAAAATGCTCAGTAGGTCATTCAACATAGAATTGTTGTTCTACAGC
TAAGCACACTTAAATTTGAAATTCTTATATTCGAACCAACGAAAAGGAAAGTGCACCTTGTTGAAATATG
TATAGCTTTTAATTTGTTTAAGTCTTTCTTAACCTTACTTCCATGTTGTAATATCTGTTCATTATTTATG
TGTTCCCTTCCTAAACTTGGGGGTTACAATTTATCTATGTACTGACACATGGGATTCATGCCCAAATTTG
TAAACAAAAGTTGTAATCTAATTGACGAGCGGAGGACTATCAATCTAATTGCACATAGAAATCACATCCG
AATCTGAGTTATGGTCACTATTATTTTTACACTTTTAGATAAAAATAAATTCTATTTTAACACCGTGACC

GTTAACATTATGGTTTGTGAAAAAAAAAATGTTTTGGCCCACCATGAAATAAATAAATGGTGAACGAAAA
AACAAGTTTAAGGATAATAAAGTAATAAAAGACAAAAGCAAAAATGAAGTTAGAAAAAGGAAAGCAAAAG
AGGTAAATTGGGCAGAAAATTATGTTACAATTAATTGGAATGGCAATGAACAAAAAGAAAAATGAGTTGG
CAAATTTAATTTAATTCTCTCACTACATATTATATAGGAATTTAATTTAAACTTAATCTATCACATTCAA
TTAATTAATTAATTTCAGTTCTTCCATATAATCAAATTACTAATTTAATCTTTAGTTTTCTTTTTCTTTA
TATTTAGAATAAACGGTCACTATGACTCTTTATTTATTTATTTTCTTTTTTGAAAAGACGACATAGTGAA
AAATAGAACTATAGGGAATGCTCAAAAGGGATCCCAAGAGCTCTAACAAGACACCGAAATTTATTGATTT
AGAAGTGATACATTGTATAAGCAAAGCGGAAGGAAAGCCCAAAGGAAGCTCTTAGTCGTTGAGAAAGCAT
TAAAATTTAGAGCTATAGTACTAAATTTGTAATCTTGGAAGAAATGGTGTTTCCTACACCATAAACCTAG
ATAGTTACACGAGTCTTCCCACAAATTTAATATATTGTTACAAGAGTCTTCCTACAAATTTAGTATACTA
TTTTGTTTATTTAAGAAGATAAAGTGCCTCTAAAATTGGAACCAAACGTCTCCATTCTTCATGAACTTGA
TCGTCTCCATTAATTCTTCATGGACTCGATGGATATAATCTAGAAGTTTGTTATGTAATTAATATGATGT
TAAATTCTATATTTAATTTGCTAATCCTATTTAATCTCCCTTTATCTAATACAATTTAATTTATATATTT
CCCAATGACATCTGTTTCATGTTAGACAATAAATTAAAATGTGTGTTCATTTAGAAATATAATTGAATAG
TACAATCATATAGAAATGAGATGCCTATCGAATGTAAATTAGAGGTCGTCAGTCATGAGAAAAAGAATTA
AGCTTTCTTCTATGTCTATATATCCTTACTTCCATTGGAATCACTTGCGTTGAGGAGGGAGAAAAAATTG
CATAGAGAGAGAGAGAACTCTCACTGGGATAGAAAACAACTTTATTTTTAACTTTTTCCTTTTCTTTTTA
AAAAGTTGTGTCTTCCAAATTCTAAATTTATTTCTTTGAGTTTTCTTGTCTAATAAACATGAAAATGAGA
CGTAGTGGAGGCAAAACTTTATTCCTTACATCTTCGTTTTCACATTTCCAACGACTAAATTCTTTCCACA
CATGACATGAAATTAAACCTATGAATTATTTTCTTATTTAACTGAGGTAGGATTTTAATCGCATTATTTT
CAACATAACTTTATTTAAACATATGAAACTAAACATGAAGTTAACATGTCACAACTTTCTGCAACTTTAG
AGTTCTTTCTGATGTCTTTAATTCAAACTTCATATAAATATCTTCTAGACATCAGTGTCTATTGGCCAAA
ATATAGACTTTTTCATCAAACAAATAAATGTATCTACGGTAATATGTAACATCTCATTGAATATCAATCT
CTAAATTTTTAAGATTGAAAAATACAGATTCGTTCTTTCACTAAAAGACTGTTGCTAACGAATCATGTGT
GAAAGAAATCTTGTAAAGAGTACGAAAAAGTGTGTGTGTAAGAGAGAAATTTGGTGTCAATTTGGAGTAC
CCAATCTGTAATTCTTTTAAGATAATAAAGTGAAAGAATACTGTTTGGGATGTACTATGATCATTATTAA
CCACTACTTTTCTCCCATCCTCTTTCCCACCTATTGCTCTTTGCTTTTGGTTTTTTCAAAAAAGGAATCA
TCATCCATCGTTATTGCTCTCTCACAATGGCAAATATTGACACGAAAGTTCGATATCTTGAGTTTTGATC
AGCACTTATCTTCAAACTGTGACAATGTCTTCCCGTGGATGTAAGCATCTTCTTGGCCAAACCACGTAAA
ATTTCTTGTGTTTCTTCTTCTTCGTCTCTTTATCATCCAATGCAAGATCAAGATTCTCCATTAAAAGCCT
CATAGTGCTTAAATGAATTTGGGTTGGATTAGGAAGATTTTTTTGGATCAACCGAATGTATGAGTTTGTC
AAATTGACAACTCAAATAATACCTAATAAAATCTTCAACCCAACCCTCAAAATTCGGGTTGGGTTTGGTT
AGTAGGTTATTTATTTTCATTTGTTTAAAGTATATTATTTATTAACTTAAAATGCTTAAATTTGTCTATA
ACACATTTTAATAACTAAAATTTTATTAAATTCAAATGAAGCTCAAATGTTAAATATTAAAATTCTATTA
CAAACTTAAAAAATTCTTCATAATGATAGGTATGAATTATAATATTTACAAGTTTTAATATATATTTTAA
TAATATTAGAAATTCAAGTTGGGTTTGGTCAATTTTTTTTTTTTTTTGTGTTGGATAAAAAAAAAAAAAA
CTCATCAATTTCTCTCTCTCTTCTTATGCTTTGACATCAGTTCTTGGAAAAAAATAATTCTCTTTCTTTG
TAAAAGTTATTTTGAGACTTGTGAGAAGGTAAGAAAATTACATTTTTACTCTTTAGTTTTCTTTCTCCTT
TAGACTCCTTTGCAAGTTGTCAAATTTCTCTTTATTCATTGTATTTTCTTTTGTTTTTGTGCATGTTCAT
CCATAGAGAATGGGAGAAAGAAAATAGGGTTTCAAAGGGGTTTCTTGATGGAAGATGAAGGGATGAGAAA
AGTGAACAAGATGGTGTTTATAGTGGTTCAGCTCATAGCCTACATCCACTCTTCAAAGGTAAGAATCCTT
TTATTCAACATTCTTTTTTACAAGTAGACTTGATGCCATGAAATTGTTTTTGACAATTGAATTTTATGTA
CTACATTGAATACTGTATTTATGGAAATAGTAATAGTTTATTTGTAGATGGAATTACAAAGCCATAGTTA
TTGAGGATTAGAAACCAAATTGTAGTATCTTGTTACCTTTTTCTATTTAAAAAATTTGTTTTATGAACAA
TCAGCTTTCATTGAGACAAGTATAATAGGTGGCCACATAAAACTATGGCATAAAAATTACCAAAGAGCCT
AAAAAATTATGATAGTTTTACAGTAGATTTTTTCATTTTGTCCATTTTGTTACTGAGAAAATAATATACA
ATAAAATGAGTATGGATAGTTTTGAATGAGTAAAGATTTATTGATGTTATTATCATCAGGAAGCTGAATA
TAAAGGAAATACTGATTATCCATTAGTTTCTTTCCACCAATAAATCTTCATTAACCATTAATCTATTTCA
CCAATAATGAAAGCCTAACATATTAATTAACCATTAGTTTCATTCTACCAATGAAATTAATGGTCAATTA
ATATTTCCTTTCTATCCTTCCAAATTTTACAATGAATTCCTTTCTTATGAAAAAAACATAATGGTATATG
AGAGTGTATAAAAAAGATATTGTTAGTTTCTAAAAAAAGAGTAAAAAAATTACCAACACTAACTTCTGGC
TGAACAAAAAATTTAGAAGGATATCGTAATAATTCAAATTGTTACAACTCAAACCTTTATATTTCTTATG
AAGAATGAGGTAAGTGAATACTAGTTTCCTAGTTATTATAAGAGGGGTAGATGCTTTAAGCCTCATTGTA
TTTTGGCCCAAAGGAAGCCTATGCGTTGAGGCTAAAACTTAGAATTCTCGTCAATCTTTTTTACTCGATG
TATAGTTAACATTCTTTAATATTATAATTTTTCTTATTTCTAGTGAGTTTAAGGAAACTTGATGAACAGC
CTTAGTGGTTGAAGGGAAGCAAGCTTCGAGACTATCAGCTTGAGGGTTTGAATTTTCTTGTTAAAAGGTA
TATTTGTTATTTATTTATTATTTCTAGTGAAATTTGAGGAAACTTGATGAACAGTCTGGGTTTGTTTACA
TTCCATATTTTTTGCTTCTTTAATTTGTCATTTAAGTGATGTTGTTAATACAAAGTAGTCAATCAGAAGA
AACAACATGAAGGGCATGTGTGTAGTATATGTGGGGTAAACGAAAGTTAATGATTTTGGTTGTTAATTCC
TCCCTTTTCAGGAGACCTTTTCTTTCTTCTATGAGTCCTCACAGGCTATAGAACTGCTTACTACTATGCT

TGACATGTTTGTTTGCTACTGCTTAAATGGTTTATTTTGATATTGCTTGTAGAATGATTGAAGTCATCAG
ATTGAAAGCTAAGCGATTGTGTCAGGTGATCGTTGAAGGTTGAAGACTGAGAAGGCATTTAGGATTTCTT
ATTTAAGTCTTATATTAGGATATTGTTTCATGTACTATTGTAGAATGTATATATAAACACAATATTAAAT
TTTCATTTTGTGTATACAAATCTAAAAGATAAATATTATAGTTTCTAAAATAATATTAATTTTATTAATT
TGTTGGAGCGAGAAAAAATTATTTATCTATATTGATTCAATGTTGGTTTATAAAAAATGGACAATAATAC
AACAATTAAATAAATATAAATTTATAAAAATGGATAAAAACAAGCCTTTAATGTCGGTTTTAAACTGACA
TTATTGGCCTCTTTAATGTCGGTTTTAAAACGACATCATAGCCCAATCGCCATTAAAGGGCTTCAATAAC
ACTATCAAAGATGTCGGTTTAAAACTGACATTAAAGCCCAACCGACATTAAAGAGCTTCAATAACACTAT
CAAAGATGTCGGTTGAAAACTGACATTAAAGGTCTTTAATGTCGTTTTTAAACCGACATTAAAGCCCAAC
CGACATTAAAGGCCTTTAATAACGCTTGCAAAGATGTCGGTTTCCAAGTGACATTAAAGCCTTTTAATGT
CGGTTTTAAACCGACATAAAAGGTCAGATTTTTTGTAGTGATAGATTCAAGATAAAAATGAACTTATTTT
CATACTACAACTCCTTGTATTTATTCTGTAAATTTTCTTCTCAAGGTTACTAGACTTTTAATTTGTTATT
TTGTTGAAGATAACTTCCTGTTCTAACAAAATTAGTTTGTAATTGATTTGATTCTTTTTTTTAACAGCTT
AGAAAAGAAGTTGAAGCCCTCTTGCAGAAGATAGTTCAACAAATCTCAACACTATTGAATCTCTGAGTAC
GCTTGTTACCTTGAACCTTCAAAAATGTTCTAACCTTGAAAAGCTTCCAAGCTACATTTTATGGGAGGCT
CTTGAAGATTTAGATCTTTCTCACTGCAAAAAGCTTGAGAAAATTCCTGATATCTCTTCCGCATTAAACC
TTAGAAGCTTGTCTGTTTAACAATGCACAAATTTAAGAATGATTCATGATTCTATTGGATCTCTGAGTAA
GCTTTATGATTTTAACGCTGCAGAATGCTCTAATCTTGAAATGCTTCCAAGCTACCTCAAGTTAAAGTCC
CTTGAATATTTAATACTCTCTGGTTGCTGTAAGCTCGAAACGTTTCCAGAAATTGATGAAAACATGAAAT
CCTTACACATATTGCGTTTGGATTCTACTGCCATAAGGGAGCTACCTCCATCAATTGGATACCTTACTCA
TCTTTATGAATTAGATCTTAAAGGTTGCACAAACCTCATCTCCCTTCCTTGTACAACTCATTTGTTAAAG
AGTCTTGGCGAGCTTCATCTTTCTGGGTCTTCTAGGTTTTAAATGTTTTCCTACATATGGGACCCAACCA
TCAACCCAGTATGCTCTTCTTCAAAAATTATGGAAACTTCATCAACTTCCAAGTTTTTCCATTCACGAGT
TCCAAAAGAAAGCTTATGTTTCAAACAATTCACGTTGTTGGATCTTGAAGGTTGCAATATATCAAATGTT
GATTTTTTAGAAATTTTATGTAATGTAGCATCTTCCTTATCTAGTATACGCTTGTCGGAAAACAACTTCT
CTAGTCTACCTTCATGTCTCCATAAGTTTATGTCCTTGCGGAATCTCGAATTAAGGTATTGTAAGTTTCT
TCAAGAAATTCCAAACCTCCCTTTGCGTATACAAAGAGTAGATGCCACTGGTTGCGTATCTTTGAGTAGA
AGTCCAGACAACATTGTGGACATATATCAATCGAGCAGGTTCATCTCCTACCAATTCATTTGCCTTGTTC
ATATCAAGTTCTTGTCATTTATACTAACATTCCATGCATAATAATTCTTATGCCATAGAAGAAATTTCAA
TGGATTAGGAATCCTATAGATGGCATAAGGGAGTTCGTTCTAATGAATAATGAGATTCCATAATGGTTGA
GCTATCAGACTGCATCAAATTCAATAAGGGTTAGCTTTCAACACAATCGCAATACAAAAATAACTTTGGC
TACATCTGTTACTTTCCGAGTGGATGGAGATTCAGATCAAGGAATGGCCTTAGTTTCATGTAACATACTC
ATCGGCTGTAGACTCGACCGTCGTTATATGAGAAAATTTCCAAAATCAGCATCAGAATATACATGGTTAG
TAGAAACTTCTGAAACATATCGTGGGAGCTCCTTGGAAATGAATGATTGGAATGATGTTATAGTCTGGTT
TGAGGCTGTGAAATGTGCTGAGGTCGTAACTATAAGAAGGTGTGGTGTCTATTTCACTGAAAAGGTCTCT
GGGATGCAAAATGATGTCAAGGAGCCAAGGCAATTTACACATATTTTAATCAACCGGAAAAATTGCGCCC
GAGATGGTGAGTGAGATTACTAATTGTTTTAACAAAGGCTTGATTAGTTGTCCTGTTCTAGAAAGTTTTG
AAACACCACTATTTTGTCTATTTGAAAAATATGATTCATCTAAACACATCTTTCTCTAAAAGAAGGTTTA
TCTAAAATCATTCTCAATAGTACATCCGATCCATAACTGTTTCTTCGAAAAATGTAAGGTTCTTAATTAT
CTTACCCATAAACCATTCAAACACAGCATTTGTTTATTTTCAAAATCAATTCCTATTATTTGATTTGGTT
TTAATTTGTTGCTGTTGTTGATATTGTTATTTGTGTGTTAAGTTTTGTTGCCATTATTTTGGTTTTAATA
AGTTGAATATTATTTTTAGAGTACATTATGCTCGTTTTCATATTTGTGTTTCTTGTGTTATGATGTTCAT
AAAGGTACAGGGATGTGGTCAAATCCTTAGCTCAAGAATTCCCTGCCAAATCATATTGCAAATATGGTCT
GTTGCGTGCAATCAATTTTTTAATTCATACTAATTCCAAAATGCAAGTATACTCACATGCATATAGTAAT
CGTTTTACACCGAAACGTGGCATGGAAGGCCTGGAAGAGGTAACACTCTCCAAATCTAAATGGGACAAAT
TTGTAAAAGATCATAATTTCTAGTGGATTGGTTCGCGGATATATAGATGGAGATCAACTTTCTCTGGTCA
CACACGATAGACAATTCTTCCAAGAATATTTTATAAGTTTTGGCCCACCAGCGCTACTATCATTTTTTTT
TAAAAGGAAACAGCTAGATAGGACTTAAGAGTGAACCTAAGACCGCTCTAGACACACAAATTTATTAATG
AGGGGCTAGAGAAAAGCCCAAGAGCTAATTACAGAAGTTGCTAAGGTTTAAAGCAATTGTAGCAGCCGAA
TAATTTTTAAAATAGGAGTCCCTACTGCACCAATTACCTATAAGAATTTTACAATCCTCCCACATATTGG
CAGTTGTTTTATAAGAGCTAGAGATTCCAAAAATTCTAAGATTCCTTTCACACCAAATTTCCCAAAATAA
TGCTATAATTCCACAAAAAACGACCTTGTGTTTCGGGATGGAATCCTCCTGAAGTTTTGGCACACCAGCG
CTACTATCATTGAAACCCATATTTTTTGGCGTGGGTATAGCCTGTGAGTTAGAGCATCCATTTAGAGGTC
GGGTCATATTATCAGGGTTGATGATCGATAAAAAAAGAAGAAAAAGATAGTAGAGAAGCTAAGAGGCTTA
ATGGAAGGACATATCCAAATTCGTCCATCATAGATCATGAATGAATTCATCTACTATCCATCGATTATTT
TCATCTTTTTTCAATTACATATTCAGATTTATGGAAGAGCAATTGTTTTGTTAGTGTGATGTATGCTCAC
TTCTTTTCTTCATAATGCCACTGACATTCAAAGAAAGATAAATCTCATTCGAAGATGAAGTAAAGAGAGA
ATTTTATATATACTTCTTGCCTGTAGTACATATTCATGATTTTTATATTTAAAATGGGATGTGGAATTCA
AAAATGCTGATGATCGAAAGCACTTATTTTCCTATATAGAAGTTCTTTGAGTAGAGAATATGTGCAATGC
AATATCAACTGTTATACAATAATTTAAATTAACCATAACAATGTTTTCAAAAAAAAAAAAAAAAAACAAGC

AAATTAAATTTTGAAAGGAAAAAAATGTGTCGTTTTTCATAAATCTCTTTTTTTTTTTTTTTAAGAATTT
GACTAAGAATATTTCAACTTTGTTTCTTTAGTAAAATAATTGGGGGACACAAGCCATAGACTGAAAATAA
GATTGTTATGAAATGGGGCTAAAATTATATTGATGAAATTTTTTGTCACACAAGTTATTCTTGTATTCTA
GGATGATGAGGAAGGAAGTTGATTTTTGTTACATGGAGTTCGTACTAGTCATGAATTGAGATATTCCAAA
AGAGTAGTCACAATGAAAGTAGGGAAACAGAAAGCTTAAGCTCCTAGAAGAAGGTAACGGACATCCAACA
ATATCCTAAAATGATTTTCCTGCTCTTGTTGACATCAAAGTGAGTTTCAATGCCCAATACGCCAAAGTGT
TTTTAATGACTTTTTCCATGTGTGTTAGGATTGCAAAATGTTGAAGATGTTCATCTGAGTTATTGGCGGT
TATTGGAGGAAACTCTTGACTTCTCTGCAGCATAAAACCTTGAGAAATTATATCTTAGAAGTTGCAAATG
TTTGAAAATGATTCATGGGTCTGTTGCTTTCTATCTCAATAAGCTTGTTACCTTGGACTTCGAAGGTTGT
AAAAGTCTAGAAAAGCTTCCAAGTAGCTTTCTCATTTTAAAAGTGGATGCATGAAGCATAAAGAAACTTG
AGACTTATTGACATTAAAAAACCTTAAGGAACTACATCTTAGAGAATGCTATCATATAAGAATAATTCAT
GGCTCTGCATGTTGAGAATGCTATCATATAAGAATAATTCATGACTCTGTTGGGCGATTTCTTGATCAAT
TTGTTATCCTAGACCTTGAAAGGCTTCCAAGTCACATCAGCAAGTCAGAGTTTATTGAAGTTTTGCATCA
TGATTCATGCCAAAAGATCATGGAAAAAAAAAAATTCACAACTATTTTGAAAAATTTTCTAGCTACCTCAA
GTTCGACTCTCTTAAAGTTTTGAATTAAGGGAATTAGTGACTTTTCAATTGCATTAAACCTTGAGATATT
TGATCTTAGGGGCTGCTTCTCTTTCCGAACAATTCACAAGTTGTTGGCTGTTATTAGAGGAAACTCCTAA
CTTCTCTACAGCATTAAACCTTGAAAAATTATATCTTAGAAGCTGCAAACAACATTTGAAAATGATTTAT
GGATCTGTTGTTTCTCTCAGTAAGCTTGTTACCTTGGATCTCGAAGGTTGTGAAAATCTATCAAATATAT
AGCAAAGTTTATCAGCGATAGATTTTTATCGTTGATAAACTCTTATGGTTTATCGGATAGACGAATATTT
GCAAGATAGTCTATTAGTAATAATAGATTTGGACATATTTTGCTATATTTGTAATTTTTTTAAGATGTTG
TTGTATATTTAATTATTTTTAATCTAATTGCTATATTTGCAACTATCCTTTTTGAAAAATTAAGCATAGA
TAAACATTATGTTTCTTTCCTCATCAATTTCTACCATTTGTTATCTACTTTTATTTCAATTATTTTTTAA
AAAAATTTAGGTGATGGTTTGAAAATTGAACTTAGAAAAATAGTTTTCAAAATCTTTTTTTCTTAGCATT
CGATTATTTGTTCAAAAGTGCTTCTATATCAAACTTCAAAACCATAATGGGAAAAAAAAAATTAAGAGAA
AACAAATATAAATTGAAAAAAAACAAATATATATAAACTCAAATATCAAATCCTGTTTTCATTTCATTAA
CAATGCTGTCTTGAGATAACATTATAATTATAAAAACAAAAACTAGAATTGCAAAAGAGTAATTAGAGCC
TCTCTTCATGTTTGTATATTCTAACTTCAACTTTGGCAATTTCTTCTCCAAATTATTCTTCCATGATCAC
GTGAACACCACACATTTTTATGCTGCTTACTTCGTAGTAATATTTCCATTTTTCTGGAGTAATTACCTCA
AATAACACTGTAATTTTATCCAAAATACCTCTTGAGGAATTAATCCTAAAACGTGGTTGATTTAGATCAT
CACCATATGGGTTGAAATGTACACAGGGATCAAGCACTTGCATCCATAGATACTCACCTCGTGATCTATC
ATCCATCCAAAAGCTTTTCCTTACTTCAAAATCGTTGATAAACACTCTATAGGCAAGGTGAAAGGGGTAG
GCAGTAACTTGAATTTTGACACAAGGAGCAATAAAAGCCTTCCGTTTCCAACTTAGATAATCAATTACCG
GAAAAAGAAACGTTATTGAATTGTTCGTACTCTTATATTTGCACCAATCTGGAATATCACAATTCATTAA
TACGAGGTTTTTGAATACTCCATGTTTTGTATATTCCACTTCCTGCACGATGAGAGTAAATAGCTAAAAG
TTATATATTAATTAACAAAGCATGTAAACTTAATCCTTATATAAAGGAAATACATGAGAGATGAGGTCAT
GAAAAAGAAATGATACGCACCTCATAAAACTTACAAGATATGAAATCAGGTATGTTGTCAGGAAATCTGG
CCAATGATACGCACCCTGAAGCATTCATAGTAACTGCTCCTTCTGGAATCTTTGGAATTTCTTCAAGCAA
CTTACAATCACTTGTATAAAGATATTTCAAGCATTTAAAATTAATAATACATGAGGGTAGTCTACAAAAG
TTGTTTTTAGATAAGTCCAACTCTTTCAATAAAGGGGCAACATGAACCATTGTTTCCAAGAAATCCAAAT
TTGTTATCTTACAATCGAAAAGCTTTATTGATGTTAGGTAGGGAAATAAGGAAGGTGAAGAATGATCATT
TAAGGAAGGAAAGATTGAAAGATCAGAACTAAAGATACCTAAATAAGTAAGATTACTTAAACGATAAATT
GTACTTGGAAGAGTTTTGAGCTCCATGCAATTTTTGATCAACAATTCTCTTAGGCTAGTAAGATATCCAA
TTGTTGGAGATAGCTGATTAATCACAGTACTATCATCAATCAACAATACTTCTAGGCTATCCATTTCTTC
ACTAAATTGAGGACACCATTCATCTATTCTACATCTGTACGAAAACAAAGTTTTAAGGGATTTCAACTTG
AGGTACGTTGGAAATTGCTCAAAGCCCTTAATATTGCTAGAAAGATAAAACTCGACAAGCTTACTAAGAG
ATCCAACTGATTCATGAACCTTTACTAAATTTGCACACCCTCCAATATCCAATTTTTTTAGGTTTATTGC
AGCAGTTAAATCAGGAATTTCCTCCAAAAACGTGGAAAAACTAAGATCAATTTCCTTCAACCATTCAGCA
CACTGCAATTAATATAAAAAGAAAATATAATTAAATTTCAAACAAATAAGCCGACATTGAAATTAAATTA
ATATAACTCAAATATTATTGACTGAAAATAATACCATGAATGCTTTCCCGAAATGTTTGATGGAGCTATA
CGGCAATTTGAATTGGATAAGGTTATCCATTGTGTAGGTTGAAGGCAAAGATGAAAAAGGAAAATGAGGC
CAATTAATCCACCTTAAGCTATTAGGTACATACTCCAGATCAATACTTTTTGAAGATGTAGCATTCTCAA
CTTCCAGTACTACCAAATTTTTCACTTTTTCAAAAGCTCTTGAATCAATGTCCAACTCTGTAGGTTGAGG
AAAATCTATTTTTATGACTTTAACTGCTCTTGCTTCCTACAATAGGTTACAAAGCAAACATTTCAAGTCA
TTTTAATTTCAAAATAATAAAAGTTGTTCTCATAATAAATAATTAAGCATATGCGAAGTTTGCATGATAG
TTCTTACCTTATTGCCATTTAAGACATCCATAGCATCATCTTTAATCAACAATCTTTTTCTTTTGTGAGA
TTTAGAAGTCTTTGAAAGATGAATTGTGCGACCCATTTGTTGTATTAAATCATGCATTTCAATCCAGTTG
GATCCAACAATGGTAAGAAGTGATAGATTGATAAGTTTTGTTGTTCCCTTTTCCAAACATAAACAACCAC
ATGCTTTTAACTTTATTTTAACTTCATTGATATCTTTGCCTACAAAGCAACAAGAAATATAAAGAAAAAT
TTCTTTCACTTCGTCTTCAAGTCCATCATAACTTATTCGAAGAGGATCTTGGATGCCCTTGTCAAGATAA
TGATTTTCATATTCATCCAATATAAGTTTAAACTTGGATTGTTCGATAGAGTAAAGGAAGGAACCTACAA

CTTCAAGAGCCAAGGGAAGACCATCACAATAACGTACGGCACGTTTTGAAAGGTATAAATAATCACTTGA
TGGACAACTCATCTTAAAAGCATGCCAGCTAAAAAGCTCGAGGGCTTCATCATCCTTCAATCCCTGAACA
CTTTGAAATATATTAAATTCATGAATAGCAAGTAAATGTTTGTCTCTTGATGTCGCAATGACCATACTTC
CAGGTCCAAACCAATCATGTCTTCCCGCTAATGCTTCTAGTTGTTCTCTCGTATCAACATCATCAAGAAT
TAAAAGAATCTTTTTTGAGCATAGTCGATTCCTTATGATGTTAATACCTATATCAAGATTGCTAACATTG
ATCGAATTATCCATTAGAATCTCACAAAGTAGCTTCTTTTGGAGTTGAACAAGGCCCTCATATTGATTCG
AAGCTTCTCTAATTTTTGCCAAAAAGCAACAACCTTCAAAGTCATCAACGATCCTATTGTACAATGCTTT
GGCCAAAGTTGTCTTGCCGATACCTCCAATTCCATATAATCCAACCATAGTAATTTTTTCATCTGCAGAC
ATAACTTGGAAAAGTATATTATTAACTTGTCTATCTATTCCAACCGGATATTTAGGTAAACGCAACTGCA
GTATTCCACGATTTAATTTCTTCGAGACTTCTTGAACAATTTGTTGAATCAAATTGGCCTCATCACTGCC
ATTTTAAGAAAGAAACATACACTAAAAGTTGTTTCCAAACTCACTTATAATTCCTATATTATTAGAAGCG
TGATAAAATGAGCAACATAATAAAGTAAAACTAAATCTTAACCAAGTAATATCCATTATAGAAAGGAGGA
GATAGGACAATATAATAACAAATTAGATAATATAATGGAATAATTCCAATTTTTACAAAAAACTAATTCT
AATGGCTTGGTATAAATAATTTTTGTTTTTGAGAATTAAGAACATGTGCCATTATTTTCGCCCATAAGAT
TTTATGATTTGTTAATGCCTTCTTCTACTATTTTTAAAAATTAAGTCAACTAAATCATGAAACCTATATA
TATAATTGTAAAAAGTAAAATTTTGCTATAATGTAAAGATCTTTAACAATTATATCATTTAAAACAATTT
GAAATTTCATACATATATATATAATTTTCAAATCTTGTTTTTTTTTGTAAAGTTAAAGTTTTTCTTAATA
AAGTTCAAACTCATTATTAAAAATTGGCAGTGATATAAATTTTAAAAAATGTAAAACTAAAAAATAAAAT
TGTTATCCAACGGAACTTAAAATGTCACATTTTCTTTGCATGAGCATGCTCTCACCTTGAGATAGTCAAA
GTATAAATAATAACAATAGAATATGAAAGAGCAAAAGAGTTTAGAAAGATATATATGAAAAAGAAAAATA
CTCATTTTGAAGAACCGGCCATCCAGACATATGAGAAACAGAAATCATTGCCTCCCTCCATGCTTCCATC
TTGTCCGATGAGAATCTCACTTCGAGTTTCCCAAATTCTTCTCCAAATCTTCCACTTTGTTTTCTTACTT
GAGATGGATCCACTCTGTAGAAAATTGGTAAAACAAGTTGTTCTCCTGATCTCAATTTGTTACACATAAT
GATTTTCTCTAGTTCATTCAAACACCAACTTGAAGATGCATAATTTTCAGAGATTATAACGATCGAGATC
TTGGATTCTTCAATAGCTTCCAAAAGAGATGCAGAAATTTCTTCACCCCTTGAAAGCTTATCATCTATAA
AAACATTGATTCCTCTTTGACGCAAAGCCATATTAAGATGACTGGTGAAGTTGGAACGAGTATCTTCCCC
TCGAAAACTTAAAAATACATCAAAACTACATCTAAAACGTGAGGAAGAAGATGATCCACTTGATCGATTC
ATATTATTTAAGTAATTTGAAAGATGAATCAAAGAACCAGAAAGGAAAGGGAAAGTAGAAAGGATAAGTC
AAAGCTGGTGCTTGAGTTTTCGAAACAATAAAATATGCTTATAAGACTACATCCGATAAATGTGTGGATT
ATTCGTTTTTTTCTCTCATCATATCGGTTGTTTCTTTCCAAAAACTTTCCTCTGTTTTTCTTTTTTACTT
TTGTTAAAAGGAAAATTAGGATTTATTCTTCATTATTATTTTTCTTTTTAATATTTTTCTGTTTATATAA
ATTTTACTTTAGCGGCTTTTTAGAGTTTTATATATTTACTGTGTAAAACATTATGTTTTATCTTATTGAA
ATTTAGTTGATGTTGCCACCACTAGCTGACGTCTGCCTCCGTCAATGACTAATCATAGTTCAAAATCCTT
CATAGGAATAGCCTTCTAATAAAGAGATTTTTTTAAAAAAATATTAAATAAAACCATTTTTTCTTGTAAT
CAAATTTTAGACTCCCAAATATGTGATCTCTCACCTCAAAAATTCTAGAAAATCGGACTCCTAAATTTCC
TAGATTTCGTCGAATTTCGTTTAGGGAAAACGGTATAAGTTATTAGGAAAGATCAATTATGAAATCATAG
ATTAAATTTTGAACGTTTGAAAAATATAAAAAAAAAAATGTAACTTCATAGATTAAATTTTGAACGTTTG
AAAAATATAAAAGAAAAAAATTCTAAATGATTAAAAAGAATAGTTTTGTAAAATATTTTAAAATTTGGAT
AATTTTAAGTTAGAAAGATTTTAAAACTGAGATGAAAATTTTAAATAGGAAACAAAATGAGGCTTAGAAA
GTATAAATAAACAAGAGTAAAGAAGCGTCATAATAAATTATGCAAACATAAGATAACATATTGAGATTTT
AATAAAAACATATTGAAGATCGATCTCGGACTTCCAAAGAATCGATTTTCTCACCTGAAGAATTTTAAGA
AATTGGACTCCCAAATTTCATAGATTCCATCGTTGGATTTTTTTAAAAGAAAACATAATTTAAAATGTTA
ACAAACTTAAAAATGATTAGTAATGACATACTCAATTGACCACTAAAGAGGAGAAAAACAAAATATTAAA
TAACATGTAAAATACAAGAACATATTAAAGAAGCAATAATCAAGATGAGAAAATACTAATGAAGCAATAA
TAAATGCTATAAAAAGAGAAAAATAATGAAATAAATAAAAAATGCTAATTATATATAAAAAATTGTAAAG
AAACAATATATAAAAAATAAAGTTTAAGAATAAATTAATAAAGGAAAGACATAATAATAAATAAAAAATA
GATAACAACAAGGTATGAAGAAATATTTAATAAATATAAAAAAACTATAAAAGATAAGCTCACATGATAA
TAAATAAATAATTATAAGAGAAATAAAAGTAGAGAGATAAGAACAATNNNNNNNNNNNNNNNTGTCTTTTT
CTTTTTGATTTTTTTTATGAAAAATTGAAGATAGATAAATAAAATAATTAAAATAGTAAGAATAGTTAAA
AATAAAGAAATTGGAAGGAAAATAAACAAAATAATAAGAAAAAGAAGAGGAAAAAGAAAAGAAAGAAAAG
TTGAAATCTCAACACACTAAATTCATTTGTTATTGAGATGTGGATCATTGGGACCAAAACCCTACCATAC
AATTCGGACTAAGTTAAAGTTTTTTTTTGTGTGTGTTGGATTCTTGGCATTGTAGGGAGAAAGATGAAGA
TTTAGGGGTTGTTAGAAGAGAAGATAAAATGGATGTTTTATATATTTTTTTTTTCTTTAAGAAGGTATGA
AGGTTAAAAGAAGTAAGAGTATGGAAGTTAGAGATTTAGATGAAGAATAGAGGATGTGAAGGGAGTAAAA
ATTTCTAGTTTTTTTTTTTTTTTTGTGGCGTTTTCTCTATATTGTGTTTTGGATGAAGAAGAGGAATATT
TATAGCCAAAATCATTTCTTCAAATGGCTAAAAATTCAAAATGGTGGGATTTTTTTGAATTTGAAATAAT
GTCATCTTTGGCTTACATCTCAAAATGGAGCCTTTCTTAACAAAACCAATTTAAAAAAGATATCATATTT
GGTATTTCTCCACTTACTTTAGTTACCCTAACTAAATGGTTTAGTTACTTTAACCAAATTTCCTACAAAA
CTTAAAAAGTAGTTAAATAACATTTTTTTTAGAAAAAGGTAGTTAATCACAAAATTAAAAATAAACCAAA
TTATGGCAAAAATTAATCTAACAATTAAATTGATTTTAAATAATATCGGACAAATTTAGGTGGCTACATT

AACTATTTAGAAAATTTGTCTCTAATGTTATAATTTTTTTTTATTTTTTTCAAACTATCAAAATTTAATC
TATGATTTCATAAGAAGGAAATCAATCTTTTCTAATGACTTATACAATTTTCCTTAAACAAAATCCTACG
ATGGAATCTATGAAATTTGGGAGGAATTTGTGAAATTGAGGAGTCTGATTTTCAAGAATTCTTTAGGTGA
AAAATCACATCTTTGGGTGTCCAAGATTTGATTCCGATAAAAAATGATTTTAATTAATATTTTAAAAAAT
ATTTCTTTATTAGAAACAATTCATATTACGGATTTTGAGAAATTGTACTAATTTCTCACTTTCTTAAGGT
GAGAAATTTTCCTTCAATATAGTCTAATTCATGGAATATAATGTTCTCCACTTATTTTATGAGATCATTG
CTCTAGATATTGGAGTAATAAAAGGTAAAATAAGACAATAATTTTAAAATAAATTAAATACTATTTTCAA
TTCAAGATTTGAAATTTGGCCACCGTTTTCTAAACGAGTGTTATAGAATGCTAACACGTTCCTCATACAC
AAATGACTTATGGACTCGTCCTTAGTTTCACAGACCATTTTTTTAATGATTTTATTTAAAAACGGTTTAC
TTTATTTTGATATTCAATCACACCGTAAAAAAAGATTGGTGACGATTCTTTTTTTATTTTGTTTCAAAAT
TAACCCATTTTGATCGGGTTGCCGGTCGCTCCGCATCATCTCAGACACGTGACGACAATAGTTCTTAGTT
ATTGTATTTGGATTATAATAGTTTGTTGTAGATTATTTTAGTTTGGGTTAAATTAGTATACTTGTGGTTT
ATATTTTTACGATAAATATTTTTTCAATCTTTTGACAAAAGTTTAAAATCTTAAATCCAAATATTTGGTA
TTTCAAGTGTGCATATCAAGTGCCCAAGTGTGTACGTATCAAATGTATATCACAGGAATCAAGTGTGTCT
ATCAAGTATATCTATTAGAAGAATATAAGTGTCCTAAAATAAAACAAAACCTAAAGGACAGGAAAACCAA
CGTAGTTAAAAAGTGGGACAGTTGCAAATATAACAATTAGATTCAAAGTATTAGCATTTATAACAATATT
CTGAAAAGTCACAAATATAGCAAAATTTATCAAAGTCTATCAAATCATATTGCATTGATAGACTATTCGT
CTATATCACAATAGAGTTTATCAACTTTGTTATATTTACAACATTTTTAAAATGTTATTATACACTTCAT
TGTATACTATAATTTTCCAAAAATTATTTATATTAAAGTACAAACCTTTTCTTTTTAGAATCAATGAGCA
AATTATTGTAATATTTTATGGGGTTAAGATACTTGATAGAATTGACAAAATTTTAGTTAATTAATTAATT
AAATTAAATTAAATTAAATTTCTTGATTAAAAGATAATTTACATAAATCTAATAAAATCTAAAAATGTTT
AAGGCTCATGTAACAAAAAAAAAATAAAAAATAATAATAAAAGAGCTCATAAGTCATCAATGTATTTCTAT
AAATTTCATATTTGCCACCATTGATCGTCACTTTTCATTCTTTTTCTATTTTGCGATTTATTCATCTTCT
TTGATCTTCTCTTCCAAATTTTCTTTCTTCTATTGTTTACATCTCATATTTTATCTTTATCATTTTCGAC
AAGATTCTTTGAAGTTATTTCACGATTGTTCAGTATTCTTTCTCATCTTCCTCATATTTGAAAATATCAA
AATCATCTGAATATTGTTTTTAATGATCAATACGATTGTTTGCCATAGTCAATACCCGATCGTTTAAATT
TAAAGTTATTTTCTCATCACTAAAAAAGAACTACACTATCGTGTACCATGATCTAAACAATCGTAGATCA
TTCGTTTAGACTGTAGTACACGATCGTTTAAAAGAAAATTACTCACACGAACGTGTGGCTGATTAATCAC
GTGTTGATGGGATATTTTTTATATTTTCCATTATAGGCATGTGAACTTTTTCTGTAACGACCCAACTCTT
TATACTAAGCTGAGGTCGTTACTAAAACGGAACAATGACAAGAGACACTTTTTTGAAACGAGGGAAGAAT
AAATTTTCATTAAAAATGGAATATTAAAACACTGAAACATAAACGCGGAAGCAAAACTGAGTCCCCATAT
GGCATGTCACGGATCCTTCTCTGTCGCTCGCCAGCTTTCCTCTACCTTTACCTTCGCCTGAAATGTTAAA
CATAGAAAGAGTGAGTATAAACATATACTCAGTAAGGGACCTACTACTAGTCCCGCTAGGTGTCTGTTAA
CTTCCCATTAGAGTCCTGAAAATGGTACCCAATCTCTGGCACGTTCCCGAACACGTGCAACATGCGCTCC
CGTAGGAACGAAAATCTGGTCTTCGGTGTCCCGGGGGAGCACCTAGGACATGCTGGTCTGTAGTGAACCC
GGGGGTAACACTAAGACAATCGGGATGCGAGGACCTCGTCGAATCACTCGAATCATATCTATATACATGC
TAGACTGGCGTCCCGTCGGACCACACAGTCCTAAATAGGTGGTGATCCCGAAGGACACCCATGCAGGTAC
GACTCTAATAGACAAAGTTAACAGAACACCCTATCCATAGCATGTAGCATAACATAACATCATAACATGG
CATGAGTATTAATCTTAACGTCCTTAATCATGTGATTAATATATCATGCATTAACAATCATCAACATCAA
TCTATCAGTCCTTAACATAACATCGGTCATCATCATCAATCATCAACATAATAATCTCAATCATCATCAT
CAACACCAACTATCATCATAATCTCAGTATAATCATTATCATCAAATTACGCATCTTAGCTACCATCAAT
GCATAATCATAATTACATGCGGTCTCTTGAATTCAGTTCGAAGGTCTAGTAGGAGAATCTCTTACCTGGA
GATTTTAGCCAAACAAGGTACTCCCTAGTTGACAGTAAAAATTCTCCAATTAACTTGATCCTAATCATAA
AAGGAAAACTTAGTATCTTAATTAAATGAAATTAGCAATTGGCTAACATCCAAAAATCCTCCCAAATTAA
TTAACTTTCTAAAAATTGGGTTGAAACCAATTCAACCTTGATTGGGAAAAATCCAAGATTTAGATCTTAA
AAAGTTTAGCCAATTGAACCTTTACAGAAACCCCAAATAGATCCAAAATTAAATTAATAAAATATTAATT
TAATTTCATTGACTTACCAAGGTTACTCAAATGAAGGTTGGAAAATCCTCTTATCCTTGATGAAAAAATTC
ATCACTTTAAATCTCAAGCTTCCAAAGAGACCAGTCTTAACTTCGACTGATGAGGCAGCGACAGCAGAGG
GTTATCTTAGAGAAGAAGATGAAGAACTTTTTCTTTTTCCTTTATTTTCCATCTTAAGCATTCCAATGCT
ATTTATAGACTCACATAATAACAATAATTATTATTATTATTATTATTTCCTTTTCCTTTTCCTTTTAGGA
TATATATATATATATATAATACCAAAAAAATATACATACATCTTTATTTCCATTATCTTTCCTAAATAGA
TGCCTTAATCTTAGGCATTTATAACCATTAATAATAATAATAATACTTCTTTATTATTATTATTTTTCTC
TCACCAAAATCTACAATAAATATATATTTATTTAAACCATTATTCTCTCTTATAAATAAATATATATCTT
TTTTGTGCAATCAAATCAACCATCTCTCTCCTTATGGATTGTCTTCTTTTCCAAACAAATATAATTATAT
TCTATCATATAATTAACTTCACTTTTCCATATTATTAATTAAATATATATATCCATATATATATACTCAA
TTAATTACAATTCCACCAAAACTAACTTTTCCCTCCAAAATCTCAAATTAACTTAAGTCCTCAATTAATT
TAATCAATCAAATCTTTTCCAATAAATCACTTATAACTTCCAACATGAATTATCTTAACCCAACCATAAT
AACTTCACTCCAGAATCAATATTTATCTTTCTGCAGAATAACTAATTATCATCCACAATAATTAATTATT
ATTTACCTTTCTTCCAAAATAATTAATTATCTTCCACAATAATTAATTATTATTTATCTTTCTCCAAAAA
TAATTAATTATCTTCCACAATAATTAATTATTATTTATCTTTCTCCAAAAATAATTATCTTTTCCTTAAA

TAATTATACTTCAACAAATATAATTATCTTTTCCTTTAACATAATAATTATATATATATATATATATTTCCA
CATATACATATAATTATTAAATCTCCAACAAACTTTCACCCCACAATTTAATTAAATAACGTCCATAATT
ATTTAATTAAATTCAACTTCAACAACACTAAAAATCCACAACTTTACTTAATTCTCTTAACCTACCATTT
AATAAAAACTCAACAAACACCACGTGCCAAAACCTCTAATTAATTAAATATTCATCCAAGAAATATTTAA
TTAATTTCAATTCCCACCTAAATCAAATAATTCTCATTAAATGAATTCTAAATAACGCCCAATAAATTAA
ATCTAATTAACTCCAAAATTATCTTAATTTTTGGGCGTTACATTTTCCGTCTTCAAAACTGTTCTTTTGT
TATATTTTTCTTTAAATCCCTTCATAAAATATAGAATCATAATTTTAAGTTTTCTTATTTATGTAGACCT
TTTTATTGTGAAAAATTATCTCTAATTAAGACTATTTTAACACTTGAATTAAATTTTGTTTGCAAAACTT
GAATCTTCGTATCATTGTTAAAATTATACCCTTAAATTTATTAATATATATCAGGTCGAAATTGGATTCT
CACTTGATTTTATTCAACATTTGAGATTTCAATTCTATAAATATTGTAATTTTGTAGAGTCTCACTTTCG
CAATAATGAAAGTTTGATAATATACATAATTACAACTTTAAAAACTTAACATGTGAAGTCAAATTTAATT
GATAGCTAAACAACACTCTCTAGAGAAAATAATATTGCAAGGGATTGAATAACGAAAATTTTTAGCCACA
GGCTCTTTGGACTCTCAATCTCTCGTACCATTTTAACAATGTACTTAGACGACTCTCATGCTGCTTTTGG
GGATTACATTTACAATCATGAAAAGTGATTTATATGATGAACCTTTATAACTACGTGCATTGTTTTTTGG
TAAAATGAATGGTTTATTAATCTATCAAAAATCACTTTTAAATTTTAACTAACTTGAACCCAATGATACA
TATTAGCTTTTAGATTTGGCTTTCCTACAAATTTACCAACTTCTTAACAATTCAAATAAATATACTATAC
TAATATCTTTTCTTTATTAATATATTTTTCTAAATGTTTTTTTTTTTTCAATTTTAGTTGATTCATTTTC
ATGAGAATTATTTTTTTTTAACTACTCTCAAATAATTTAGATTTAACAAAATGAGTTGAAATTTAAATCA
TATGTAATTTTGTAAAAAAAAATAACAAATTAATTATTCATGAAAAAGTAATTAAAATAATAAATCTTAC
ATTGGTTCATAAAACTAATTTACTCCGATTTAATATTAATTTATCAAATAGATTGTAACTAGTTTTTTCT
TAGGTTAATCTTAATAAATGTAATAAAACTTATCGTACCGTTCACTTGGTCGATAAAATATAGTATAAAT
TTTATATTTGTCTTTGATATTACGGATGATTCGTCATTTCACTTCCTTCATCCTTTGTGATTTATTCTTC
TCTTCTAAATTTCTTCGGCTCCTCTTCTAGAATTTTTCTATTTTTATTCATTTCTTTTTCTTTCTTTCTT
CATCTTCTCTTTCTTTCTTCCGGTTCTTCTTCCACGATATTAAGATTGTTTAAATATCTCTTTAATATGA
TCTAAACAATCACTTATCTAGTCAACACAATCGTTTAGATTTGAAGCATTTTACATCGATAGAAAAAACT
AGATGATTGTGCATTGATACTACCTACACGATAATGTATCATTTCTTATACGATCACGTATCATGATATT
TTAGAAAAAAATGTTACACGCGCGTATGATCGATTAATCCCATGTTGACTAAGACATTTTTTATATTTCT
TATTGTAGACCTGTATACTTGTGGACAGTTTTTTTTCGTTTTCAAAATTATTCGTATTTAACCGATTTAT
TATATTTAAAAAACATTTTTTTTAATTTAAGATTTAATTATATTTATCTATTTTTTCAAAAGTAAAACTT
CTAAATAAAACTTAAAACATTAATTGATCCGAAACTTAGATAGGTGAATAAAAATAAATTTAATATAATA
TCTAACGCGTAATACACGAAAACTTGAATAAATGATATAATTTGAGAATAAAAAAAAGATAATAAAGATA
AATTTATAATAGGGAAGAGGTTGACAATACCAACAAGTTCAATCATTTATATTCTCCTCATCTCCATCTC
TCTCCCTCACTCCAAGGCTTCTGGATTATCACCATTGACCCATTTCTCACTTCCCAACAAACCCACTAAA
TCATAGGGATAATTATTCTTTTCTATATAACGTCATTACTATTCATAGAAATAAAAACTTGAATGCAAGA
GATAAGGAGAGACAATAAAGAATAATTAATAAAACCCTAAATTATCCCTAAGAAGTTTTGGTAGCATATC
AATTTAAGGTTATTATTATCCTACCATCAAACCATAATGAACTATTAGATCTTAGAAAATTTGAATAATA
GTGGAATGTGATTTAAAACCTAAACAAGAGTCAACTTAATTTATCTTTATCTTTTTTTATAAGTAATCAA
TTACAACCATTTTTAAATGTAAAAGATTGAACTTTAACGTCAATAGATTATTTTAATTGACCTTTTCTAC
TATACTATTCATTAACACATACTTATCATACTCTTAAAAATTCATCAAAGTCGTTAATCTAAATAATTAA
AAACTCAATTGGTCTAATAAGCATTTTGACAAAGATCATCTCTAGAGTATATATTATTGTTTATTCCAAA
TCTTTTGGGCATCTCAGAAGGGACCCGCCACCCTTTCCTCATCAGTCCAGCTCAATCCTCTTCACCATAG
CCCATAACTTCTAGTACAATTTATTTTCTCAAAATAAACTAAATAGTTTCTTTGTCATGTGCCAAATTTT
AATTGGATAACTTTGATATGACCTAAGTATTTCTTTTTTCTAGATATATATATATATATATATATATAGA
CAACTTCTTCTCAAATTTATCCATTTATTTCTCCAAATTACAAACAAAAATTTACAAGGAATGACAATGA
TGATGAGTTTTTACAAGAAGAGTCTCCACTCATTCTTATCCTTCACCGACAACCTTTCTTCCAAACCTTC
ACCACCTTTCAATAATTCTAATCCCCACCTTGAATTACACCATGCCTCCTCAGGCTTAGGATTGATTAAC
TCCGATCATAATAATGCCATGCAATCCAATAAATCTCCAAATATTCTTGAATCTTCCTCCCTCATCAAAC
CCCCTCTTTCCTCGTCCAGAAAAGACCCCGGTGGGGTCGGTTTCATGGACGACGTTGGCGGTGGCGTCGA
CGGGTTGATGTCGTGCACTGAGAGCCTTGGATTTGAGAGCTCCGATGAGAGATTGGTGAATGATGAATTG
ACGACCATGGAGGATAATTGCGGCGGGTGGTGTATGTCGAGGGTGCGGCGTAGGAAGGTGGTGACGGAAG
AGAGGAAGTTTCCACCACCGTTGACGTCGTTGAATCAACATGGACATCCGAATTTTTATCTCCGGTCAGT
AAGGAAAGATGGGAGATTGGAGCTGACGGAGGTTAGGATCGAACGGACTGAGATTCTTCGAGCGTGTCGT
GGAGATGGACGGTTGAGATTGCATCTTATCAAAGATGAAGAACAAGGAGAAAAAGATGGCCGCGACGGGG
ACAACGAAGGTGGGATTGAAGAGGAGAAGGAGGAGAAGAAGGAGGAGGAGGAAAAGGAAGAAGAAGAAGA
AGAAAAAGAAAAAGAAGAAGAAGAAGTAGAGGGCTTAGTGAAGGAAGGAAAAATGGAATTTAGGCGATGT
GTTGAGATGATAAACCAGCAGCGTCGCCATGAGCACCACCGTAGCCACCACCAACACCACCACCAGCATT
TGGATGTGTGGAGACAGCACTGCGTTACAACAAGTTAAGAATTTTTTTTCAAAAAAAAATATATATATAT
TTTTATTAATTATGTCGGTAGAATTAATTAATTCATGAAAGCGGTGGGTGTGCATAAAAATGAGAAATTA
TCATTATTATTATTATTAATTTGTGTGAAATGTCAATTTACCATTATCTTTACATTCTTTTTCTTCTTTT
AAAAAATGCATCTACCAATTGGTTCTATCTCTACTGAAATAATATCATCACCGATAATTGGAATATTATG

TAATGCAAATTCCAAGATGAAAATACTCCACTTGCATTTTTTTTTTTACTTTTTTTAAGTTAAAATGATAA
AGATTGGCAAATTAGTTAGATAATTACGATTACTTTTATCCAAAAAGTTAAATAAAATGGAAAAAAAAGT
GGGTTTTTATTGGTTGAATTCAATTTTGACTTTGTGTTACTAGAAGGGATTTGGATTTGGATTGACTTTA
GCTAAAATGAGAATGAGAATATAAATAATCAAAGCAATGGAGAAGAGTAGAGTACAGCACACACATATGT
ATAAAAAAGTATGCAATTGTTTGTGTAATCTACTAATAATAGTTAAAATGAGTCGAAGCAAAGTTACATT
TAAAAGCAAATTAAGTAGATTAAATTAATTTATGTAAGCTTAGAATAATATCCCCTCCAAGAAAGTAGGA
GGGTGGAGAATTTTGTTGATCTTTAGAAATATTCGTAGATTTAGTCATCTATGGCTTATAGCTTTATTCC
ATGCAATTAGAAAATTAAACTTTTTGTAAATATTGTTTTTATTAGGATGACATTTATAAGTTTGATTGAA
AATATCTACTTAAATATGGATAAGATAGCATAAACTAAAAAGAAATGGAAAAATAAGAAAAATAATTTTA
AGATAATATAAAATTTAGGAATATTTAAAATTGTATAAAATTTAGGAGTTTATAAAAAAAATAGTTATAA
GGATAATATAGAATCTAGGAATATTTAAATCATATAGAATTTAGAAAATTGAAAGTTTTGGATAAAACTT
ACATCAAATCAATTTTGGGGTGAGATGAAGCAATATTTTAATAAAATATTACAATGAGAGTAAAAATAAA
CGTAGATATTAGTTTTTTCAAATTTCAATTTCTAATTTATATATACATATAATTTGTAATTTTGTGTGTA
AATTGAATCATAGAATTATAGACAATTAATTAGGAGCCATAGAATTATAGACAATTAATTAGGAGCCACA
TTATTGGGTATATCTCTGACCTACAAAGAAAATGGTATCCACACACCATATGAAGCTTTTTTAATTAAAG
CTTAATTAATTCTTCCATTTTGCTTGGTAGTATGTATGATTTGATTGTTTTTGCTTTTAAATGTGATAGG
AGAAAGGTGAAGAATGTAACTTAACTTGGGTAAATAAAATTCTGGTCTCTTTAAATAATTGACATAAATA
TACGACTCGTCAAAGGACCCCAAACTAATCGGAGAGTTAATAACAAGAATGTGTTTTTAACTAGTGATGT
ATTCGAACATCTAACTTATAGAATTACTAATTGTTCTTTAGATGATAAAAAATAAAAACATAGAATATTA
AACTATGTGTATTGAAAATTAGTCACAACGTAAATCACCTATATCTTATGAGTAGTTATTTTAGATACTT
TTAAAAAAATTACACTTTGATGTGTAAATATCATCATTTATACATTCTTACCTGCTACCTATTTTCGTTT
ATTATTCTCTTAGTTAGTAAGTTTGTAACTTTTAAAAACGAGAAAGTTTCGAGAAAAAGATATCGTAAGA
TCATATTTAAATTAGTTTAAAAGTGCGTTGAGAGTTATATTTAAGTAACGATCGATAAGATATTTGCACT
TTTTTAACAAAAACACACAAAAAAAAAAAATTAAATACATATGAGATTGTTGTTGAAAAAATAGTATAGTG
TAAACTCATGTGGTTGGATGTTAGCTAAGTTTTTGTTATGTTGCAATAAATGAGAATGAGGAAAAGAAGG
AATTTAAAACTTCCAAGTTTTTTCACCTCCATCAATTGTTTCAATTCCCAAAGGTTTAAGCTTCGTTGGC
TAAACTTTGTAGCTTGGCCTTTTCAACCTTAATTTTGGGTACCAAAGATGTATGAATATATATATATATA
TACATACTTATATCAACGTCATTTTAAAATTGACTTAAAAAATGTTGATTGATATAAGTAAAACCTAAAC
ATTTGATGGTATAAATACTAAGCCTTTTTTTTAACCATATTGTTGTGGATCAAGACTCATGAGGGTAGAG
ATTATCACTCATTTTTATATCATAATGTTGGATTATTCAAATAGAAGTTTTTAGTTTTATTAATACTATT
ATTATTTTGTGTAAATATATTTTGGTTTTTTTTTTTTTTTTTAATTTAGACGATTCACATTACAACATAAA
AATTGAAAATGTTTTCGAACTACTCTTATCCTCTAACAAAAAAATGTGTTATTAAAAAAATATAAATAAG
AAAGTTAAATAAAAAGCATTATTCAATTTCCTTTACAACAAATGAGACATTGAGAGATTTGAACCTCTAA
CTTTAGAAGAATAAGATATAAGTAACTTCTGTGTTCATGTAATTTCAAGCTAAGAATATTATAATTTCCT
CCTATTCTATGAACCATTCTATTTAATTTCTTTGAGTAAAATAATATTGAAAAATTTCACATTACAAAAA
AATCAACGTATACTTTTGGATTTTTTTTTCTTTTTTTGAGAGTGAAAAACCAAACATATAATAATGTATT
CATAAAATACATTATTGGTTTTCTATTTTATTTTTAACCAACTTGCCTAAGTTATTATCAAATAAAAATA
AATACTTAACTTTATATGAAATTTTTAACTGTAGAAATTAAATCGTTATAAATTTTAAGATAAAAAACTA
AATTAAAATAGGTACATGTATTAAAAAACATGAATAATTAAATTGTGGTAAAAAAAAAAATACATGAATAA
TTAAATTGTGGTAAACAAAAAAAGTTAAGGAAAAATTTATTACCTTCCAAAGTAAAACACCAAATTATTT
ATAGCGTATAATAAAACCCATAAAGTTAGTCATTTTTTAAATATTATAGGTTTGCCCTTCAATATTTCTT
TCATCATCTTTCTGCGATTTCGTCTTTCTTCTTTCCTATCTTTTTGATACAATTTCTTTCTGCTTTTCCA
TCATCTTTATACTTTTTCTCTTCCTTTTTTTTTCGCTACAATTTCTTTCCATCGTCTTTCTTTTTTTTTT
TTTAGATTGTGTACAAAAAATAGCAAAATCTAAAAGATCGTGTATAAAGAATCTTGAAGAAAATCATTTA
AATTAGAGTAGCCAAATGTAAACAATCAAAAAATTAAAAAATTTTGTATAAAAAATCTTGAAAAAAATAT
CATTTAGATTGGAGTAGCCGAATGTAAACGATCGTTTAAAAAAAAAAAGGTAAACGATTGTGTAAAAAAA
ATAAAAGATCGTGTATAAAGAATTTTGAAAAAAATCATATGGATTGGAGTAGCCAAATGTAAACGATCGT
GTAAAACAAGTAAACGATCGTGTAAGAAATCAACGATCGTGTACCAAAAGAATTAAAAAGATCGTGTACC
AAATTTTGAACAAAAAAAATCATTTAGATTTGGATAAATGATCAAATCTAAACGATCGTGTAACCAAATT
TAAACATAACCAAATAAAACGATTGTGTAACAAAATTAAACAATGGAATTGAAAAGATAAATTGTACCAT
ATATAAATCATCGCATATAAATTGTAACCATATCTAAACGATCGAGTATAAATTATAGTCATATCTAAAC
GATCGCATATCAAACAATAATCAAATCTAAATGATCGCAAATATATTACGCGCGCGTTATTGATGGGGCA
TTTTTGGTATTTTATATGGTGGGTCTCTGAGCTTTTTTCGTTTTTAGAATTATTATATAGAATATAAAAA
AAACGACAAAATATTTACATTTTGAAAAAGACCCCAATTTATTCTCATCCCTACTTAGTTGTTATTTAGC
CACAAATTATAGACTTATTCTCGTAAAATTATAATTACATAAAATAATAAGATTTTGTGCTTTTTTTTAA
GAATAATAAAAGAATGGTTAATTTTCAAAAATATAATAAACTAATAAAATATTTACGACTCGTATAACAA
AGTTTATTAAGTTAGCAATTTTTTAAATATTTCAAGTATGTCTTTCTATCTTTCTTCTCCTCCTCGCTAC
AAATTCTTTCCATCGTCTTTCTTCATTTTCTCATTCTCTTTTCTTTCCATCATCTTTCTTGTTTCCTCTT
CTTTTTGTTTTATGTCATTTAGATTTGGGTAACCAAATCTAATTTCTTTTTATCATCTGTCTTCTTTTTC
TATTTTTTTTCCACTACTATTTCTTTCCATCTCTTTTTTCTCTTCTTTTTTTACGTCGTAACCAAATCTA

AAAGATCATGTATAAATAATCTTGAAAAAAATCGTTTAGATTTCTCCAAATCTAAACAATCGTGTAAAAA
AAAGTAAACGATCGTGTAAAAAAAAAAATAAACAATCGTGTAAAAAAATAAATGATCATGTACTAAAAAA
CTGTTAGAAAATCGTTTAGCCAAATATAAATTTGTACCAAAGAATTTTGAAAAAATTCGTTTAGTCAAAT
TTAAACAATCGTGTACCAAATTTTAAAAAAAAAATCAATTAGCTAAATTAAACGATCGTCTAACCAAATT
AAACAATAGAATCTTAACTATCCAAATCTAAACGATCATGTACCAAATATATTAAACGCATTGTTGATGG
CGTGATTGATGGGACATTTTTTGTATTTTCTAACGTCGACCTATGAGCTTTTTCCATTTTCAAAATTGTT
ATATGTCGTGTAAATATTTTACTGCTTTGTTATATTTTTTAAAAAACCCCTAAAAGAATCAAAATATTTA
CACTTCACACAAAAAGGCGCCAAAAAAAAGATCACTCCACTTAATTTTGTCGTGTATAAATATTTTATTC
ATTATATTATTTTTGATATATCACTAAATACTTTTTGGGGAAGGAATCAATTTTTGACTTTATAGTGCCT
AGAATAACTTTATTTTATTATGGAAAAAAAAAAAAAAAGAAGTTTAAACTTTAAATAATGTGACGAAATAT
ACATTGTAATGTTTTGATAAATATTATTTATTAAGGACATATGTAAGTAAACAAAAATGACGGAAAAGAA
AGATTATGACGTCGACAGCAGGGTTCGAACCTGCGCGGGCGAAGCCCAACAGATTTCAAGTCTGTCTCCT
TAACCACTCGGACATATCGACGTTGATGCCCCACAACTTATAATTTGCATTCAAAACATTAAACATAACC
TCAAGCTCACAAATAGTGACTTGCAGCTTAAAGGCATGCTTGGCAAGGTGATCACTACCAGGAAAGTGAG
AAAACATTTGAAAGAATGAATCGAGAAATTCAGTGAGTGATGGTTTTACGAAACATTATTTGCTAATTAG
TTTATGCATAAATCTTTGGCTTTTAGAAACACCAAAGCAGTTGAATCATATTTCATAAGCATATATCTTG
AAACTTATAAACGTTTGAAATATACATATCATTAACTTTACTTGATTATTAATTTATAACTCATTCATAA
CATGAAACGTGGGTGGTGTGTTAGTATACCTCACTGTCAATTATGTAATTCTTTCAACATAGGCACATTT
CTAACTATAATTAACCTTCTCCACATAGGCACAAACGAATTGTACCTTTACCGATGTACAAGGAATATAG
GTTTGTTAGCCTTCACATCAAAAACGAAAAACAACCATGCAAATATGCATAAACTGGTCATAGATATATA
GAACATATGGACATGGTTGATTCATGAAAACATACTTAAAATCTTAGCTTTGAACACATGCTTACTTTAA
ATCTCTTTCAAATCTAAGACATGCTTGGAAACAATTTAAAAATCATGCTTTGCAAGTCGTTTTTAAAGTC
ATGAAAAAAATATCATTTAGCACCTCAAAGCATGTAAAGAAACTTTTAGCTTTAAATTCATGTTCATGAT
AAACATAGTAATTCATTTGTCACTCACAAACTAGTAGCTCAACTCCTTAGCTCTTGGAATTTCCTCTTTC
CTCTTTTTGGTATGAAGTAAATGAATTAAAATTTCACATGAACCTTCTCATACTTAGAAAATATCAAGAA
ATCAACCAAAAATCTCAAAAACTTCAAAAATTCACTTTTGACCCAAGGCTAAATGCACACAAGTAGCCCA
TGACATGGCCCTGGGCAGCATTGACTAACCTCACAGTCGCGCCATAGGTGCATGTCTTGTGCTCAACGCC
TACCTGCTTTGGAACTTCTTTCCTTCTCCTTTGGCCGCCCACTTTTGAAACTCATTCAATTAAACATTTG
AACAAATTTTTTTTAACTTCTTTCTTTGAAAACTCAATCTTAACTTATCCACAACTTTACTTGGATATCC
ACAAAGTAACTAAAGTTTACAAGCGAATAAATACGATAATAAATAAGAAACGAAAAAACGAGTAGAAAAT
AATAAACCTCATGCTCATAACCATTTTCTATGCATTAAATGTACCTCAACTTCTCATAAAGCTTTGAGGG
TAGTAAATATTATAACCAACAAAAACAAAAAAGTTTGCATTATCCCTATATTAAATACCTCACTGAAAAG
ATATATATATATATATATGTATGTATATATTTTTCTTTCTGAGCCTGAAAAAAGTAAGTTCTAAAGAGTA
TTAATATTCACATGAAATTTGGCCCTATTTTGTGTGAATGGAGACACGTTTAGTAACTGATATATATGCA
GCACTTTGACTACTAAATGCTCTCAATATTTAATATACACACTTACACTATCAAACTCAAAACTCTTTTG
AAACGTGTGCCCCTTTTTCCTCATTATCAATTTTTTCATTGTCTCATTCAAATGTCATCATTTTGAGTTT
TATATAACAATCTCTCTAAAATATAAAAAGTAAATCAAACATATTCATGTATCATATGGATTTACTTTTT
AGTTCATACCAATATCAACGTAGGTGTTGATATCGAATTTTGAATTTTAAAACCATGTTGATAAAATATT
GATATGCGTAAAAGTTACAAAATTTATTTAGATTAATTAGTTAATTTATTTTTATTCTAAAATTAAGTTA
CGATAGTTATTGATATTTCTATTCATATTAGACTAAATGGATGACGTATTGTCTTTTACAAGCATATTTA
TAAAAGATGTTAGATATATTTATGGATATTTTATGACTACATCGATATATCCATGGATATTTTAATCGAG
TGTTGCAACCTTCACTTTAAATTAATTTAGAAAAGAAAATGTTGTTACAAACTCAGCCTAATCAATGTTT
ATTATTAATAGATCTAAAATTTAACTATATTTTATAAATATTTTAGTTTATTTTTTTATGTTAAAAAATT
CTCTTATAATAACTAAAATAGATTTGCAATCAATTCTTTTCGATTTTTTATAATTTAGAATTTAAAATTA
TTGTTTAGAATTATAAATTTTTTTTTGTTATAAATTAATCTTTAAACATTAAGGAAATAGCTTTATTCTT
TAAAAAGTTCAATATGATATATATTTTTTTTTTTGTTAAGATACATTCATTCCATCATAATAGAAAATAT
ATTTGATTAGGTGAGAGAAAGATAAGTATATAAAGTAAAGAAAAATTATAGCAAACAACAAAACTGCTAA
AAATATTTATAAAATGTAACAAAATTTTTAAATTTATCGATAATAAATATTGGTAGACTTTTATCAACTA
GCTTGACTTTAAGAATTGGTAAGAAAAATCTTCTAAAATAAGTATGAAAATAAACATGTTGTTTCTAAAA
GAAATTTTTTTATAAATATAACGAACTACTAAAATATTTACAACTCATGTAACAAAACCCATAAAGTTAG
ATATCTTTAAATATTCCAAGTTTGTCATTATCCTTATTCCCCATTCGTCTATCCAAATTTAAACGACGTT
TTTTTCCAGTCTTTTATATTTAGTACACGATCTTGAACAACCAAATAAAAGTTTGAAAAAAAAATATATC
TTTTATATTTGATATACGATCTTGAACAAAAAAAAAAATAAGGAAAAGAATGAAACATGATGGAACAGAC
GAAAACGGGAAGAAAGAACAAAAAGGAAGCGCGTTGTTGACGAAACATTTTTGGTATTTTTCATTGTAGG
CCTATGAGCTTTTTCCATTTTAAAAAATGTTCTATACAAGTTGATATTTTGCCACTTTATTATATTTTTT
AAAATACTCCATTTTAAAAAAAGTGATTTTAATAAATTTAAATTCAATATGTTTTACCAAACATATAAAA
AAAAAAATTGAGATTTGTCCCTAACCACTCTAACCCGCAAAAGCAATAATCCAAATTGATGCCTAGGAAA
AGGATAGGACTCGAGCTCCATGACCCAAATGGGCGGAGGAAGATAGAAGTTTAGGCCAAATTTGTTGGGA
AAAACCTCAAATTACTAACCCAAAAAAAAAAAAAAAAATCAAATATTTAAAGAAAGAAAAGAATTTGCAT
TGAAAAATTTGGAAGAAAAATTTAGAAAAAACAGTTGATATCACATCTTTTTTACATATTGCGAATATGA

GAAATATGATGGCTATCAAACGGTTATCAAAGGATTATCGACTTTTAAATGTGTTACTTTTGCAATTTAG
AAAATGTAATTACATGAACTCTATTTTTATAATTCTTTTTGCTATTTTTGCAAAAGCTCCAATTTTATAC
TCGACAAAAAATGGTGATTCATAAAATAGAAATTCATTTTGTAAAAAATACATCGTTATCATCAAGTGAA
ATAATTATCTCTTTAATACTCATTACAAGAGCACTCTTTGAAGTTTTGGTATTACTCACTCATTTTTTCT
TCTCTTGAATTAAAGGAATTTTAACTCACATTCGAAGCCCTAGATCAAACTCTTTGTAAGTACCATCATC
ATCACTAAAGAGAAGTATGAATGTATATTCTCAATTTGAAATTTTTGGTTCGATACAATTATTTACTTTT
TTACTTACATAGAGTATGTGCATACGATTATATTAATGGCTAGATCTTCCATTTTTACATGTCTTCTTCT
CAAACAAATTAACTATTGTTATCTGTACTAAAAAAAAATTTAATTATTATTATCATGTGAAATACCAAAT
GCCATGTTCTAAATTTCAGCATAAAAACATTCAATGAACTATAAAATATATATGTCTATGGTACAAAAAT
GATGGGATTTTAAAGGAAAATGACACAAAAACATAATATATCTACGTATTGTAAATATAGCAAAATTTGT
TAAAGTTATTTAATGATAGAATATGTTGCAAATATTGGTCTATCATTTATGAACCATAAGAGTCTATAAG
CGATAGAATCTATATTTGCAATTTTTTTATATGTTGCTATATAGTTAATTATTATTCCTAAAATTGCTAC
CCGTTATAAATACCCAAAGAAACAATTAGGCTTTGGACTCTAGAATCCCGATTGACCTCTGGCTCGATCG
AGATGAGGCCGAATTTGACCAACTACGTTTGATGAACAAAATGTACCATGACCAATTTGGGTATAAAGAA
GAGGAAAGTTCAAGTCAAAAGCCCAAACCAACATCTTTTGCCTCAATAGAGATCGAGCTTCAAAACTTGA
AGAATGAGTCATGCCAAATGCATATCTTGATCCCTTTAACAATAGAGATATATAATTTCTATTTACAAAT
GACCTACTAATGATAACTCAATCGATAAGATAAAATAATCAGTTCTATAGTTTCTATAATAAATTTCAAG
TCTAATTGATATATTATGATCCAATCATTAATGATGACTCAACCGACAAGATCTAATCAATTTTACCGTT
TCCATAAAACAGGTTCAAAATTTCGTTACTCCATTTGCTCAACTTTAAAAGGGGAAACTAACGGACTTAC
ATTATTGCACTCTACACGTCAATGATGATTCGATCTAACAAAATCTAATTAGTCAGTAGTTTCCATAAAA
GGCTCACATCCAATTACTCAACTCGCAGAACTTTTATCATTCATACGTTTGTTAAAAAAGAACTAAAGTC
GTTGTCTGACACGAAATCCGTTAACAATGATTCGACTGACAAGATCTAATCAATTATACAGTTTCCATAA
AAAGTTCAAGTTTCATGACTCCACTTGTCCAATTGTACGTAACAGTGGAAAAAAACAACCTTATACATTA
CACCAGGGCGTTAACGATGATTCAACAAACAAAATCTAATTGATTAACAGTTTCCATAAAAAAAGAAAAA
AATTCAAATCCAATTACTCAAATTATTTGCACACATTTTCAAAATAAACTAAAGTTGTCATCTGACACAC
AATCCATCAACAATATTCAACCGACAAGATCTAATCAATTTATAATTTCTATAAAAGTCGATCAAAACTC
AATCCTTTACGTCCACACTACTCATCATGATTAAAAAAAAATGCAAATGAAAACAACCTAATTATACAAT
ACTTATATCCACAAACACTAACTCAACCAATGAAAGAGAAAACAAGAAAACATTTTAAAACTTCATCCAA
TAAAATTAAAATCTTTAATCGTAACTTGTTATACTTTTTGTGAGAATCAAAGTACATTGCATATTCTTCA
TTTTAGTACACACGAAAATATATGATTGTAATACTTCTCGCAAATGGAATTAGCCTAAAATTGGGCAACA
AAATCATGCCCCTTTTTTGGATGCATATAATGAAACCAAAACTATATATTGGAATTATCCAAAACGGCCT
GCATGCATGCCTTTGTTAGCTCCATAATGAAATATGTAACGCCAGCAACCGGCAAACTTTGGTCCATCCA
ATTTATTCACATTCATTATTTTGATTTTACTCTTCATGGATTAATTTGCTGTTTCTTTTTCCATATCCTT
ATCTACATCTCTCTCTCTCTCTCTCTCTCTCTCTCTCTCTCTCTTCCCACGACGAGACAACTCACTAACT
ATTTTTCATAATCCAATTTTAACGTCGCCTTGGAATGACTTTTTCAAAAGCCAATCAAAACGTATATATC
CTTACTAGTCCTTAATATTTCTAAAGAAAATGAATCGGTAATGAAAACCCTTTAATTATAAAGCTTTGGA
AACATATTGACATGGTGTCATTTGTTGCATGAAAATAAGAATGCAACTATGTAGTGTCGTCTTTTATGTA
ATCATTACTTATTAAAGTTTGTATGTAGATAAAATTGTTATAAATTTAATGAAATATGGATCAAATGATT
TCATGAAAACATTTAGTAACAAAAATAAAATTATTTTTAACTAATTTAAAAGAGGATAACGAACTCGTAT
GATATCATTAATTACTCGTCGTATTTGCCATATTCGCAATATACAAAATAATAATAAATGCTATGAACTG
TTTTTTTTTAAAAAAATTTGTTTATCATCTGATGCAATTTTTCTTTATTGTTTAAATATAATAATATGTT
CGTATAGAGTCAAAAGAGAGTAAGCTTACTTAGCTTAAGAAAAATATGTTAACAACCAAGAATTACTACT
TAAAAAGTTTCAACAATTTATTGAGAAAAATTTATTTAAAATACTTTATCAAATCGTATAAAAAAAAAAT
TGTCACTTTGCGTAAATCTCAATCCTTAATACGAAAAGAAATAGAATATAACTTTCGATTATCATTGAAA
TAAGCTCGAGTTATTTTTGTACTATTTTCTCACTTGAAATCTATTGTGTTTTGAAAGAAAGAAAAAAGAA
AACATGTTGTTGATAATATGATTATTCTATCATCATATGGTATGGTCTCTATTTTGGAGACAAAATAACA
TTCTGCACCCCATTTGCTCTCTTCACTAATTGGGAATAGTCTTCTAAACTTTTTTTTTTCTTCCAAGTCC
TTCAACTTCATTGAATTTACCAACATTGGACCCTACACCATTCCCAATTTCACTGCCCAAAACACATTTG
AATCTTTAAACAAATTTTGGATATACAACAAGATATCTTAATTAATTAATTTCTTAAAATTAAAATACAC
TACAAAAAGGGGTTTTGATTCATAAACTTTAATTTTGTAGATTATAAAATCATCCAACATTCTTTATTTT
TTCCAATGTGTATATATAACCAATTATATTATTACAATTAACCACCTATTAAAATTGTGTTTATTTTCCA
AAACAAACAAAAATTTGTTTATTTAAGTTTAGTTTTTATATTCATTATTTGAATCTCTAAAACAAACCTA
ACTTTATTTATTTATTGTTATTAGGGATAAATTGAAAGAAATAAATTAATGACCTCATGAGGCATAACTA
ATGAATCCAAGTGGCAAATAAAATTGGTAGTACTTTGCAATCAATAACAAATTTAATGTTTTTCTTTTCT
TTGGGTATGTCAGCATCATCAGTTACTTTGGGCATATTTTGAAATATAATGTTTGAAGAGTTAGCATGGA
AACATTATTCATTCAATAATATTGCTACACAATTGGGTACAATTTCTAAATTCCAAATTATTAATTAATT
CTTTCTACCTATATATATTTTATTACATATTGATGTATGGTTTAATATATAAAGAGTAAACTTTCTATGT
CATAGAGTCTAAGTTTAAATCTATCCGAGAATGATTAATGATAGATATTGATAGATTGTGATATGTTTCT
ATCAGTCATATTGATAGACAGTAATAAAAATCTATCGATATTTATCATTGATAGAATCCAAAATTTTGTT
ATATGTAGTAAATATTTTAATTTATTTTACTATATTTAAAAATGAGAAATTGCATTCGATGACAAAAAAA

AATTAGAAAAAACAACCCGTAGCACCTCTTTTTTGCATATTACAAATATAACAAGTAATTAAATATATCA
GTCAACTATCAAATGACTATTAAAAGACTATCAAAAGACTATTCGCTTCTAATTTTGCAACTTTTGCAAT
GACATGAACTCTATTACCAGTATTTTTTTTCTACTTTTGCAAACGTCCCTTAAAAATGTCCCTTTGATCA
AACTATTAGTTTAACTAAATTGTTGGGTAATCCAAAATAAAAATTCCATCGCTATATATATATTATTTAA
TTAAAAAAAGAAGAAAAAGTTGCTTCTTGAACAATCCTTCCAACCAATAACTTATCAATTACACAAAATC
TCCACATGAAAACAAGCAAAATCTAGTAAGTATTTGTATTCTCATTTGTTGTACTAAGAATAGTTAATAT
CTACCTGAACTTAGGTTAACCAATTTAGGCATTAGTTATTTGGCAAAATAATTGAATGTATATTCAACTA
ATTTTTTAGTATAATAAATTACAAAAATAGAGAATTAAACCTCCTTCTTAATTTAACATGTGCATATGCT
CTATATTAATTTGTTATGTAGACCACATTTTCTTAATTGTTGCTATCATAATAAATAATGAATATAAAGT
AACATGGAAATAAATTTCATGCTATATAGTGATATTTTTTAAGAAAAAGACCTTGGAAGAGAGTACAATT
CATTTATATCGTTTTTACGAAAACTATAATTTGAGTAATATTCGAATCTAGTCTCTCAAATTACAAAGGA
AAAAAACATATATTCTTTACTAAGAAAAGTTTATTCGATGATTAATTACTTGAATGTGTTACATGGAGAT
AGATGTAGTCGAGGTTGAATCGATATTGTTATTATTATTATTATTATTGGGAGGGGGAAAAGAAAGG
AAAAAGGAAAATCCTTCCATGCAATACTCAATTGGACATGGTGAATCCGAAAATAAAAAGAAAGTGGATT
AAAGACCTGATTTTGATATTATCTTTCTCTCTCTCTCTGTTCTGTCATTCCTTCTCTTTCCTCTGCTG
CTTTTTGAAGGTTTGAGAGGAGAAGTCGCTTTAATACGCCATTTCACCATTTCTGCCTCTGCCTAAAACC
TCCATGGCCGAAACTTTCGCACCCCATTTCCTCAAACTTCCCTCTCCTCAACATAGTGAACAACCCATTA
TTCCATCAAATCTCTTTCTAATCATATTTCTCCATTTTTAGCCCTTCTCTTCTCTTCTCTTAACCCACCA
TGAAAACCCATTCCCAAACATTCTTTTCTTTTCCCATTTCAAATCTCTATTATTGCTTTCTTACTCCTCA
CACAAAATACCCTTCTCTAATGCCACCGTATTGGAAGGGTATCCGCTAGCTGGACTTTCCTTTTTTGTAT
TTTGGACCTTTTCTCTCTGTTTCTCTCTGTTTCTTTTTCCTCTGTTTTTGAGTTTTCGCCATGGAGAATG
ATACTACTCGCAAAGCTTCTATTCCATGGTATGTCTTTTTCATTTTTAGCTTTTCTTTTGCTCTGTTGAA
GATTTTGGGGGTTTTGGTTTTTTGAATTTTGCATGTTCTATTCGGTGGTAGAGCTTAGAAGGTGGATTTT
GGTTTCTGGGTGTATGTTGTTTGGTCATTTCTAGTGGAAAATTACATTCCCTTTTGGATTCATGTGCTGT
CTGTGAATATCTTGTTTCTTTTTGTCCTCTTTGTTGTTGGGCTCTTTTGGCTTGCTTGAGACTTTTGGCT
CCTTTGTTGGGAAGATGGGGATTCCAAATTCCCCCATGTTTGTGTTTCTTCAACTTTTTCCTTTTCGGTA
AAGTGGGACAAGAGAGTTGGCTGAAGTGAGAAATGTTGTAATCTGTTTTTGCTTTTTGATATGTTTGATA
ACTTGAGATTTGAGAATGATTGTGTTGAGGTATATTAACTTTATACTTTTATATGTGAAAAGTGAAGAAT
AGTTAATTAAATTGAAAACAATTATAACCAGGAAACATTTAAAATTTGTTGTGGGGAATGGGGACGGCCT
TCCAATTTTAAAATACAAAAGCAATACTAAACTGAGTCATTGCATTTGTTGTGGTCACAAGCATAATCAC
CCTTTTTCGGAATGTTTGGAGGGGAAGTCTTGAAGGTTCTGGTTTTGAGCCCTCATTGAAAATTCTTGAT
GACTCTTCTAGTTGATGTCGGGGTAGTTAAAGTTGGTATAAGTAAAAGAATTACTCTTTCAATATCTGTG
GTGTCTCCATTCGGATTTTGGAGACTCATTGCAACATTTACACTATTTGTTGGGATTTTGACTGTTCTGA
ATTCTTTGTTGCTTCATAGGGAGAATGATAGGGGTATAGTGGCCAAAAGACCTCCACTTTACATGGCTAC
TTATGGGAAACATAAAATGCGGAGCAATATGTTTTCATCTGCTTCGGCTCGAGGTGAATATCTGTTTGTT
TTTTCGGTTTTTGTGTGCTCCTATAGTGCATCAGTTAGGCTTATGAATTGTGGGATTTGTGTGCTGTGTT
TTGTAGACTTGAGATGTTTTGACCTGGAAAGTGAGCAACGTGATGGATTGTCCCCAAGAGGAGTTTTGGA
AGCAAGCATGCAGAATTCAGAGTTCGATGCGGATTCTTTGAATGATAGTACCCCAGAACCTGAAAATCAA
CCTTCACAATCAAGGGCTCTTTTTCACTGGAAAAATTTCTTCAAATTATGGAAAAAAAGATCGTTCAGGC
GCCTGGCCTCGTTTCCTCCTCTTGGAGTGCTGAAGATTTCAAGAAGAGGGAACAGAAGCGGGAGAGAGAA
TCCGGGACTAAGTGACTTGTACAAGTTCAAGTCCTCACTGGGAAACTTCACTTTCTCCGAGCTCCAAACT
GCGACCAACAAATTTAGCCCTGGTTGGTTGCAATTTGTATTTTATATACTTTTTAGTCTAACTTTGGTGT
CAACTTTGTTTATTGTGTTTTTTTCTTTTGCGTAGAAAATTTGATCGGAAAAGGTGGTTATGCTGAGGTA
TACAAGGGCCGTTTACATGATGGGCAGCTGATAGCAGTGAAGAGGCTAACTAAAGGAGCCCCAGATGAGA
GGACTGCTTGTTTCTTGTCCGAGATTGGCATCATTGCTCATATTGATCATCCTAACACAGCCAAACTGAT
CGGTTGCAGCATTGATGGAGGCATGCACCTTGTTTTCAAGCTATCTCCAAATGGAAGTTTAGGATCTTTT
CTCCATGGTGATTCCTTTAACCTCGTTGAACTAACTGCTCTTTGGTTTTCAGAATTCTTATTAACCTTTC
TATTCACCATTTTTCAGGTCCAAATGCGAACAAACTCGATTGGAGTAAAAGATACAAGATTGCTCTTGGT
ACAGCTGATGGTCTGCTGTATCTCCACGATCATTGTCAGAGGCGTATTATTCACCGAGATATCAAGGCCG
ATAACATTCTGCTTACAGAAGATTTTGTACCTCAGGTAACTTGCATATTCTAAATATCAAATTCATTATT
GAAGTCTTTTCTTGCTCACACCCCAAAAAAAGATAGTGGCCGTTGAATGAAAAATTTATTTTTCATGCTT
CTATTAAAGTTGAAATGACAGAGAAGACCAGTAAAGTCTACTTTATTCCTCTAGCCTTCTATCTTATTTC
ATGATTGAGATCATTTTATACTCAAATGGATGCAGATTTGTGACTTTGGCCTTGCAAAGTGGCTACCCAA
ACAGTGGACTCACTACAGCGTGTCAAAATTCGAAGGCACATTCGGGTCAGTAGTATGCCTACATCCTCCT
CCTCGATGTAACCGTTTTTTCTCTTGTTAAATTTCTCATCTCCTCAATGCAGATACTTCGCTCCTGAATA
TTTCATGCACGGGATAGTTGATGAGAAAACTGATGTTTATTCTTTTGGAGTTCTACTGTTGGAGCTCATA
ACTGGTCGTCGAGCTTTGGACGAGTTGTGTCAAAGCCTTGTGCTGTGGGTAATAATAACATTAGAACGAA
CTTTCATTAGTACCCATTAGTTCATTATATTCCTTTAAACATTGTTAAATTTTCTTTATTATAGGCAAAA
CCTCTCTTAGATAACAACAATCACGAGGAGGTCATTGATCCCGCACTCAAGGAAAGTTACGATCTCGAAG
AGGTTGAGCGCATGATTTTAACTGCATCTTTATGCATTGAGCAGTCTCCTATTCTCCGACCTCGAATGAG

TCAGGCAAGTAGGAACTACAGCACGGTATCCCCTCATCTCTCTTCAAGAAACATGTAATCAAAACACATT
TTGGTTTATTGCAGGTTGTTGTCCTGCTAAGAGGCGACAAATACGTAAAGGAGTGTGAAAAAGGTACAAG
GGTACCACTGCAACGAACGTACTCGGAAGAGCTCTTAGATGCACAAGAATACAACAAGACGAGATACCTA
AGCGATCTGAAAAAACACAGGCAGCTTGCATTTGGATCTTGAAAGTGATTTTGAAACCATTCACCTTAGA
GAGGAAGAAGCTATTGCAGTGTTATAAGGGGCTTGTTTCTGGAGGTCAATGAAATGAAGAAAAAAAAGAA
AAAGAAAAAGAAAGTATTTGTGTATGTAAAGTTTATAATTAAAAAGAAAAGTGTTAATAGTTAAGTTAAA
AGTATTTTTAACTTTGTAATCTAGTGGATCAAACAGCCAAACAGTTACTTAATTAACCCTTTGTATTGGT
ACTGCCATTGTTTCAACACAAATATGAAGCTGTTTGAAATGAAAAGGTGAACTTTCAACTGTTTGTATCT
AAAAGTTTCTTTTGTTATTTCTAAGTTCTTTGTATGGTCAATGGCAAGGTAACTAATTCTTGGTAAATTT
AAAATATCAACCTAATGACTTCTTCTTTCTTAAAATTAAAAAACGCGTTTCTTTTATTTGCTAGCTGATA
CATTAAGATAGAACTTTATCGACATCTATAATTAATAAAATTTTAAATTTTTGTTCGATTTGTTTTTGTT
TTTTCTACGAAGTCATTGTATTTTTATGATTTAGATATAACATAGATTTTGGTCATGATATTGACACTTA
TTCAATTATTGGTTTTAGTATTTATTATATTTATAACTATGTTTATGATTGTATTATCTTACATTTATTA
TACCAATAAATGTAGATATAATTACTGTGTCAAAGAAATAAATTTAGATTTAAGTATGGTGCCATATAAT
CATGAAAGTTTCTCATGTTATTTATTTATTTATTTTTTAATTTTGAAATATACCATTTTATAAAGTAAGT
GTAAGACATATGTGAGTTTTTATTTTTTATTTGGTAAGTTTTTTAGAACAAAGATTTAGGGGATGAAATT
GTAATTTTCTTGCAAGGCTATAAAAAAGAAAAAAATAATAATTATAGTAGAAAAAATGTATGTAAAAAAA
AATACTAAATCTTACATTGATATTAAAATGGAATTTATTTAAATAGTTTAACTTTGGTTGTTAAATGTTT
GGTAATTATTTGATTTTTGAAAAGTGTTTGTGTAATTTCTTACTCATAATTTTTACATTTAAAAGTTTAA
ATTTTTAGTTAATTTCAAAAAATAAAAATCATATTTTTAATTTAAAAAATCTGAATTTAATTTTGAAAAC
AATCTTAAAATATAGACAACAAAACAAAGAAAGAAATAGATTGAGGTTATATTTATATACTTAATTTTTT
GAAAAGGAAAAAAATGAATGGTTACTAAATGTAACTTTAGTTTTTTAAAATTAAACACTTAAACACGGTT
TTCGGTTTTCATTCAGAACATTAGTTCTTTTGCTATTCTAAACAAAAAAAAAAAAAAAATTACAAATGTTA
GCTTCCAATTTTGTTAAAAAATTAAGTATATTTTTTAACAAAATGAAAATCATAATAAAAAGGTGAAAAA
GAAATAAATAAAACTAAAAACTAAACCCACACCCATAGCTATCTTATATACTTATTAATAATTTTTATAA
TTAATCAATATTTCCTATCCAAATTATCATGTATAAAATTTTTATTTTTGATTTAAATTTAAATTAACGA
TTGTATAAATTTAAACTCTAAATTTTTATAAAGGTATCAATTTCAATCATAAACTTAGGTAAATGTATCA
ATTTAAACCTTCAATTATGATTTACCTAAATACATTAATAAAAGTTGAGAGTTAAATTAATAAATGCGTA
AATTTATGAAAATTTAAGGTTTGAATTGGTAAACTATTAATTTAGGACATAATACAATTATTAATTTAGA
GTTATAAAATCTCATATATTTGCTATATATTATATATATACCTATTCTCACATTTTTAATAAAGAAAAAG
TACAAAATAAATAAAATAAGAGTGTGGTACAATTACCAATTTTTCATTACAATTAATACTCTTTAATTTT
CCAATTTGCAATAATAATAATAATAATCAATAAACCTTAACCCCCAAGAAGAAGAACAACCCTACACGTG
GGTATCTTTCAACACGTGTAGTAAAGCTATGTATCGATTGATTCAAACTAAAACGGAATTGTTATTTCTC
AGCAAAATCCAAAGCGATTTTGAAATTCCCAAAAACCTAAAAAAACCCTCCATTTTCCCTCTCTAACTCA
CAACTTGCCGTTTCCGATATTCTTTCTTCTCCACCTTAAAAACCCCATCAAAAACCCAATCTCCCATAGC
CGCAATCCTTTCAAGAAACCCCCACAAAAATGGCGAAAAAGAAACCCACTAGATCGGCCAAAGAGCCAAA
GCAAATACCCAATAACCAGGAGGAAACTAGCGATTCAGAGCAACCCAGGTCTGCCATGGATGATGACTCG
AAGTTGCAGAGCTTGAAATCGTTGAACGAACGGCTTCTTAAGGAGATGGTCGAGAAGAGAGTGGTGGTCG
GCGATCTTGTTCAGACTAAAGAAGCGTTGGAACTCGACTTGAAGCGGAATGTGAACGAGAAAGAACAGGT
AATGGGTGAGTTGAGTGAGGCTCGTGATGGGGTTTATGGGTTGGAATTAGAGAGGAATGTTGTTTGTGTT
TATCTGCAGAGTCGAATAGAAGAAATGAGTGGTGGGATTTTTGGGTTACTTGAGAGTGAGAGAGTTAAGG
GTTTGGAGATTAGGAATCTAAAGGCTGAGATTAATGGTCTTGTTTTGGAGGTTGAGGAAGAGAGGGAGAA
ATGGAGGGGAGTGTGTTGTGAGAGGGATGAGATTAAGGTTGAGTTTGATGGGTTGTTGAAGGAAACAGGG
GATTTGAGAGGTAAAGTGGTTGAAATGGAGAGAAATGAGAGAAGAACATTGGAAGAGATTGATGATTTGA
AGGGAAAATGCAAGAAGTTGCTGAGTGAAAAAAAAGAGCGCGAGATTTTGAATGGGAATCTGACGAAAGA
CAATGAATTGATTAAGAAGTTGTTGGAGGAATCAGGCAGGGTAATTGAAGATTTAGAGAGGAAAGTGGAT
GTGAAAATGAAGGAGAAAGGTGAGATTGAAAAGGAAAAAAATGGGCTGAAAATGGAGGTTGAGAAGTTAG
AGAAGGAAGTTGCTCAATTAAAAGAGAGTACATTCTGTTTCAAACAGGAAAAGGAAGAGAACGGGAAGAG
AATTTCTGAGCTTCAAATGAGAATTGAAGAAGCTTTGGTGAAAGAAAGTGGGATGCTGATGGAGTTTGAT
GTTCTTGTCAAAGAGTTACAGAAGAAGGAGAATGCTATGGAGATGCTAACTCAACAAAGAGATTCACTTG
ATGTGAATTTGAATCTAATCCAAGAGGAGGCAAAAAGTTTACAACGTACGCTTGAGATACTCACCCATGA
TAAAGCTGAAATGGAGGAAGCGAAAACTGAAGCACAGAATATTATTGGGGACTTGCAAAAGGAATCAAGT
AAACTAAAAGAAGCTATAGCTTCTTTGACCAAGATGAGTGACGTCGGGAAAGCAAGAAATGAGGAATTGA
TAATCCAAATAGGTCGTCTTCGTGATGCTTTGGATGAAGTTTCATTTGAGAGGGATGATGCTAGAAAGAG
ATTTGGTGATGAGAAGGAAAATGCCGAAAAGCTGCGTCTGTTACTCAAGGACAAGGAGAGGAGAATTGAA
GAAGCCGTGAAAGAACTAGATAAAGCAAAGATTGCACAAGAAGAGGATTCACTGAATGTGAAGAAGGAGA
TGGAGAGGCGGCTCGTTGCCTTGATCGGGGAAAGGGATTTGATGGAGAAAAACTTGTTGGCGGCAAAGAT
TAGAATTGATGAACTAAAAGCAAAGGTAAATTCAGCAGTTTGTAATTCAGAGAAAGCATTGGCATTGTTG
AAGAAAACACGTTTGACTGTTTGTGATGGTTATGGGAAGGGAGAAGTGGAAGAAGCTTCTTCTGACGAGC
ATAAGATTGGTGAAGAGATGCAGCCATTTGTTGAACATTTGGATGCAATAAAAACATCCTTCACAAACAA

GGAGAAAGCAGTGGAAGAAATGACACGAGTTCTCGAAACCGAACGAGTCGAACAACAGAAGAAGAAGAGC
TTCTTCACCATAGTGACTGCAGCAACAACAATATTGGCTGCTGTTTCCGCTCTTTATGTTAGCAAAGGGC
GCTGAAGCTGATGAGGTCATACTTCTTTTTTCTTCTTTTTTAGTTGGTAATGGCACCCAGTAGCTCTTAA
TTAAATCAACAAACTGACATGTATTTGGAGAAGCAATGTTGAATGACTGATTTAGTAGTTTTAATGAGAA
AACACATTATTGACATCTAATGACCATTACGATGTTTCCTTTTATTGAATAATTTCGATTATATTGGAAT
GAAATATCATTGAATTCTCAAGACAATTGGGAAAACGTGAGATGGGCCATATTGTAATATAACATGATAC
AAAATTTTAAAGTTGTTTATTACTGTATTTAGTTTTGACAATTTCTACAAGGACAATGTATCAACTTACT
TTTATTGGGTTTGAAACATGATTTCTTAAAAAGAATAAGGTTTATAGTGATTCAAAAACATAAAGTTGAA
CAGTATTCAAGTGGGTGGACTTCTTAAATATTATTAATTTTTAAGGGGCATTTTAAACTAAAATATTTGC
AAAAAAAAAAAAGAGCACAACATCAAATACTACATATCTCTCTCTTGTATAATCTTGTTTGAATGAAGTA
TAATAAATGTTATTTTCTTAGTGGTTTGAAATGAAGTATAATAAAGCAAGTATAATGAAGAATAACAAGT
CATTCTCTTTTGGATGTCTATACCTAAAAATATCATTCGTTAAATAAAACATATACTATCTTTTTTTGTA
TGGTGCAGCATATTGTCTCTCTATTTTGTTTTCTCATTCTTTACCCTCTAATTTTTTTCCATTCCTTTTC
CTCCATTTTCACTTCTGGATTTTCCTTCTTCTCCCCTTTATTTCTCTCCGTCAGTTGATCTCCTACTCCC
CAATTTTATTTGATAAGTTTACTCAAATGTAATAATAAACACAAAAGATAACCTCTATTATTTTAGAGTG
GCAAAGGGAAGATGGGTACACATGAATATGACCAAAATCCATACTAAAACTATAACGTCAACCACATCTC
CAATTTCAGAATCTATCTTGCAAAGGCGATCGGTCGTCCAAATCTGCTGATCATTGGTCGCCGGAAGAAG
AGGGTGGTTGGTAGAATCTGCAATTCAGCGGAGTGTACATAATAAGGAGACACCATCACAAGTCTTGAAG
CTTTGGCGCTCTTGAAATTCAGCTTTTAAATTAATTAGTCATGTTTTTCAAATATATAACATAGGAGAAT
ATGAGTTTTGCGTCAAATCCCATCTTGGCAGAGTGTATTTTGATGAAATTCCTTATTGGAAGGAACTGCC
ATATTTTTAGAATTTGGAATCTGTAAAGTTATTCTCATGTGGTTTTTTGGGCAGTGGAGGGAGTTGGAGA
AGTTGATTGGTGTGCAGATCTATGTATAGGACAGATCATTGAAGAGGAAAAAAGAATTGGAATATTAGGG
ATTTGGTTAATTAAGCAATATGGGAAGTTGGATTTCAGAATTGCAATTCATGATTGGGTTTCGATTTAGG
AAGAATGGACTTGCAAATTCAATTTAACATAAAATTTCTTTTTTTTTCCTAAATTAATTTTTATTTGATC
TTCGGACTCATTACAGGTCACTACAAACAATATCCATGTCAGCACACATAACGTGTCTAGTAGACATTAC
GTTAACTTAATGTAGGAAACAAAAAGAAAAACGAAAAAGGCAAGAAATGAAAAAGGAAACGTTGTGTATG
ATGATTAAAATGTACGTTGATAGCTAAATTCAACTTTGTTCTTCATTAGCAACAACAAGGGACAACAAAA
TGAAAATTTCAGCACAGTTATGGAATGTTGAAGAAGAAGGTGACGAGAGAGATGCCACATCATAAAAAAT
TAATATTATAATCATTTTTGTCACATCAATTTTCTGATTACTTATATTTAACCTACACATCATTTTGCCG
TTACTCAACTAACGATCGATATGACCTTTAAAGCTCGATGATTAAAATGTTTATTTTGAACTTTCAGGGA
CTATATAAATGTATATATATCAACTTCAAATCTTAGCGGTCAAAAGTGCATTTTGTTTTAGTTTAAAACA
CCGAGATGTGACGATTAATAATGCCTACACACTCACGTACTCACACATGCAGTGAGTTTTTTATGTTTTT
TTTACACACATTAGACGCAAATTGAATTCCTAATTCCATTCCCTAAGACAAGTTTCTAATGACTTATACA
ATTCACATAGGCATGCATGCAGAACAAGCGAAAAACGACGAATTTACCTTAAGAAAATGGCCCAACCAAC
CACTTACACAATCTCAGGCATACCATTCCCCGGGATGAAAGAATTTTTCCTGTAATTCATTCGATCTCAG
GCATACAATGCATCGAGAAGACGCAAAATGAAAGACAAAAGTATAGAACAACACAATCCTGTACTTCATT
TTAAACGATAGTTTTCAAATTTAACGATCGTGTAGATCATGATACACGATCTCGAGCCTTAGTAGCACCG
AGATGGCCCGAGATGAAAGAATTATGTCTTTATTTTATTCGATATTAAGCATACATTACTTCGAGATGAA
AGACCTCTACCTTTAGTTATTCGATCTCGAGCCTTAATTGAATCGAGATGACCCAAGATTAATAGATCGG
AAAGAAGATATATATCGTAAGGGCATATATGAAATTTATGAAAATACGATCATGTGTCATAAACTTTTCT
TATTTTGTTATATAGACCGTAAATATTATAGATTTTGGTTACATTTGTGTAAATTACTCTATTTTTTTTA
GTAAAACAAATGAATTAAATTTTTTAAAAAAAAATTATTGAGAGTTGAAGTAATTGGGTGTTTTCTAAAA
GATTGAAATAAATACCTTTATTAACTTTGCAAATATATTTTCAAAGAGGCTAACAAATCAAATATATTTT
CTAAAAAAATCACATTGCGTTGGGGATAAGGTAAGAGTAATGGAAAAAATGAAAGGTTGTGACAATTCGT
ACTCTTACTTCATGAGATTTGACAGAACAAATATTTTTCAAATACTCTTTTTACGTTTTCTTTTTTAAAA
TTAAATAACAAATACTTTTCCACTGCTTTTCTTTTCTTCCATTGACTCCTCAACTAATAATTTTGGTCTT
CCCATTATTATCTTCTTCTTCTTCATTTTTCTCTGCACTGTCTCTTCTCCATTTCAACATCTTCTTATAT
CTTCATTCAGGTTAAATATTTCATCCCTTACCATTTTTATTTACATTTTCCATCTGATCATTTCTATACT
TATAAACTCGATAATTTGTTTAGACTTTCTTTAGATAGTTTTTACTTGGTGATCTGTATGTGTTCACTCC
TACTTTTCTGTTAGGCAAAATCTTCAAAATTATTATGGTTCATTCCTCGTGGTTTTTTAGCTCTTCAAGT
ATTTTTGTAGGTTTTGATCCTTTTCAAGCTCAAGAACAGAGGATAATAATGGACATCCTTATTTCAGTCA
TTGCAAAAATTGCTGAATACACTGTTGAGCCTGTTGGACGCCAACTTGGTTATGTATTTTTCATTCGTTC
CAACTTTCAAAAACTTAAGACTCAAGTAGAAAAGCTGAAGATTACAAGAGAGTCTGTGCAACACAAGATC
CATAGTGCAAGAAGAAATGCTGAAGACATAAAACCTGCCGTTGAGGAATGGTTGAAAAAGGTCGATGACT
TTGTTCGAGAATCTGACGAGATATTAGCCAATGAAGGTGGACATGGTGGACTCTGTTCCACCTATTTCGT
CCAACGACACAAGTTAAGTAGAAAAGCAAGCAAAATGGTAGATGAGGTTCTTGAGATGAAAAATGAGGGG
GAAAGTTTTGATATGGTATCCTATAAAAGTGTTATCCCATCAGTTGATTGTTCACTTCCAAAAGTGCCTG
ACTTTCTTGACTTTGAGTCAAGAAAGTCGATTATGGAACAAATCATGGATGCACTATCTGATGGTAATGT
CCATAGGATTGGAGTATATGGGATGGGGGGTGTTGGCAAAACAATGCTAGTGAAGGATATTTTAAGAAAA
ATTGTGGAGAGTAAGAAGCCTTTTGATGAAGTGGTAACATCCACGATCAGCCAAACACCAGATTTTAGAA

GTATCCAAGGACAACTAGCTGACACGCTAGGTTTGAAATTAGAACAAGAAACAATAGAAGGAAGGGCTCC
TATTTTACGAAAGAGGTTGAAGATGGAGAGAAGTATCCTAGTCGTGTTGGATGATGTCTGGGAGAATATT
GATTTGGAAACAATAGGAATTCCAAGTGTTGAAGATCATACGGGATGCAAGATCTTGTTTACCACTAGGA
ATAAACATTTGATCTCAAATCAAATGTGCGCCAATAAAATTTTTGAGATAAAAGTTTTAGGAGAAGATGA
GTCATGGAATTTATTTAAGACAATGGCAGGTGAAACTGTGGAAGCAAGTGATTTGAAGCCTATAGCCATT
CAAATTGCGAGAGAATGTGCAGGTTTGCCTATTGCTATTACTACTGTTGCTAAGGCATTACGAAATAAAC
CATCCGACATTTGGAATGATGCCTTAAATCAGCTTAAAAGTGTTGATGTGGGTATGGCAAACATTGGAGA
AATGGAAAGGAAAGTGTATTTGCCACTAAAACTGAGTTATGATTGCTTGGGATATGAAGAGGTGAAGTTA
TTATTCTTGTTATGCAGCATGTTTCCAGAAGACTTTCCCATTGACGTGGAAGAGTTGCATGTATATGCCA
TGGGCATGGGATTCTTACATGGTGTTGATACTGTGGAAAAAGGACGATGTAGGATTAAAAAATTGGTTGA
TGATCTTATATCTTCTTCTTTGCTTCAACAATATTCTGAGTATGGGTGCAATTATGTGAAAATGCATGAT
ATGGTTCGTGATGTAGCCCTATTAATTGCATCTCAGAACGATCACATACGTATATTGAGCTATGTGAAAA
GTTTAAATGAAGAATGGAAAGAAGATAGACTATCGGGTAATCATACAACAGTGTCTATTGATGGTTTACA
TTATCCTCTCCCGAAGTTAACGTTTCCCAAAGTTCAACTATTAAGGTTAGTTGCACAATCTTGGTGGGAA
CATAATGAGAGTGTGTCGGTGGTAGAAACTTTTTTTGAAGAAATGAAAGAGCTCAAAGGTTTAGTATTAG
AAAACGTAAATATATCATTGATGCAACGAACATCTGATCTTTACTCCTTAGCAAACATCAGAGTATTACG
TTTGCAAAGATGTCAATTATTAGGGAGCATAGATTGGATTGGTGAATTAAAAAAGCTTGAAATTCTTGAT
TTTAGAGGATCTAACATCTCACAAATTCCTACAACCATGAGCCAATTGACACAGCTGAAAGTTTTGAATT
TATCTTTTTGTGAACAACTCGAGGTAATTCCACCAAATATTCTTTCAAAGTTGACAAAATTGGAAGAATT
AAATCTGGAAACTTTTGATGGATGGGAAGGAGAAGAATGGTATGAAGGAAGGAAAAATGCTAGCCTTTCT
GAACTCAAGTGCTTGCGACACCTTTATGCTTTAAACTTAACCATTCAAGATGAAGAAATTATGCCAGAAA
ACTTGTTCTTAGTTGGGAAGTTGAAGCTTCAAAAATTCAACATTCGTATTGGTTGCCAAAGCAAATTAAA
GTATACTTTTGCATACCAGAACAAGAACAGAATCAAAAACTTCATTGGAATCAAGATGGAATCAGGAAGG
TGCTTGGATGATTGGATAAAAAATTTGTTAAAGAGGTCGGACAATGTGCTTTTGGAAGGATCGGTTTGTT
CAAAGGTTCTCCACTCAGAATTGGTAGGTGCCAATAACTTCGTAAGTTTGCAGTATCTCTACCTTTATGA
TAATTCAAAATTTCAACATTTTATCAACGTTAGCAATACCATCAACATTGAAGAATCATTTTTTAGTGAA
ATGGTAAATTTCATCAACACATTTTTCTATGTTTCTATATACACATCAAAATTTATAAATCACCTGTTTG
TTCATTTTTATTGTTCCTTCCACAAGTTCCTTCTAACATTTGTGGTTAGTAATGATATTAGGGATAAGAT
TCTATTCTTCTTTGTTTTAGAAGGCTCTTCTCTTGGCGTAGAGAGACTCTTTCTTGTTTCTTTCTTGTTT
CTATATAAATGCATGTCAGGTGATATTCCAAGAAAATTTAAAGAAGTTTATTAGATATTTCCTATCTAAG
GAAGCAGATTTGAAGTAATCATTTTACACAATTTGTTATGTTAATTTTAAAACACTATATAAATTATAAG
TTGTTAGTTAGCATCAGTTGTGATGATATGGTTAATTACGTTGTCACAAACTGAACTTTTAAGGACGTAA
ATTTGTAGGTATCGCTTCCTAATTTGGAGAAGTTGGAAATTGTGAATGCAGAGAGTTTGGAGATGATATG
GAGCAATAACGTGCCAATTCTTAATTCCTTTTCCAAACTCGAGAAAATAAAAAATTTGTTCATGCAACAAT
CTTCAAAAAGTATTATTTCCTCCAAATATGATGGGCATTCTTACATGCCTTAAAGTCTTGGAGATTGGAG
ATTGTAAATTGTTAGAAGGGATATTTGAAGTGCAAGAGCCAATTAGTGTTGTTGAGACCAATAATGTACC
CATTCTTAATTCCTTTTCCAAACTCGAGAAAATAAGAATTTGTTTCTGCAACAATCTTCAAAAAGTATTA
TTTCCTCCAAATATGATGGGCATTCTTACATGCCTTAAAGTCTTAGAGATTATAGATTGTAATTTGTTGG
AAGGGATATTTGAAGTGCAAGAGCCAATTAGTGTTGTTGAGAGCAATAATTTACCCATTCTTAATTCCTT
TTCCAAACTCGAGGAAATAAGCATTTGGTCATGCAACAATCTTCAAAAAGTATTATTTCCTCCAAATATG
ATGGGCATTCTTACATGCCTTAAAGTCTTAGAGATTATAGGTTGTAATTTGTTGGAAGGGATATTTGAAG
TGCAAGAGCCAATTAGTGTTGTTGAAGCGAGTCCTATCGTGCTCCAAAATTTAAGTAGGTTGAAATTATA
TAATCTTCCAAACCTTGAGTACCTGTGGAGCAAAAATCCTTGTGAGCTTCTAAGTTTGGAAAATATAAAA
AATTTGACCATTGAGGAATGTCCAAGACTTAGAAGAGAATACTCAGTCAAAATTCTCAAGCCACTTGAAG
ATGTAAGCATAGATATCAAACAATTGATGAAGGTTATTGAGAAGGAAAAGTCAGCAGATCATAATATGTT
GGAATCAAAGCAATGGGAGACTTCATCTTCTTCTAAGGTACGTATATATTCTACAACAAAACATGTTTGT
TCAATTTAATTTTCAGAAAATAAATTGTTTTCAAGACCTTAGTTGAACAGAATTGGTTCTGTTAGTTGAA
CAGAGTTTTAAAGAAAACAAATTATTATGTGTGCAAATATAAGTTATTATTGCATTTATTTAATAGATTT
TGATCATCAGGATGGGGTTCTACGGCTGGGAGATGGTTCTAAGTTGTTTCCAAATCTTAAAAGTTTGAAG
CTATATGGTTTTGTTGATTATAACTCAACCCATTTACCAATGGAAATGTTGCAAATCTTATTCCAACTTG
AAGTCTTTGAATTGGAAGGAGCATTTATTGAAGAAATTTTCCCCAGCAATATACTGATTTCAAGCTCTAT
GGATTTACAGAGTTTGATTCTATCTAAACTACCCAAGCTTAAGCATTTGTGGAGTGAAGAATGCTCACAA
AACAATATCACCTCAGTTCTTCAACATTTGTGTTCTCTAGGAATTTCAGATTGTGGAAGATTGAGTAGTT
TAGTGTCATCATTAGTGTGTTTTACAAACTTGCAACATCTTCATGTGAATAAATGTCATAGACTAACCCA
TTTGCTGAATCCTTCGGTGGCTACAACGCTTGTGCAACTTGAAGGTTTGACAGTAGAAGAATGCAAAAGG
ATGAGTAGTGTAATTGAGGAAGGATCAACCGAAGAAGATGGAAATGATGAAATGGTTGTATTCAACAACC
TACAAAATTTATACATTTTTAATTGTTCCAACCTAACAAGCTTTTATTGTGGGAGATGCATCATTAAATT
TCCATGTTTGGAAAGAGTATTCATTCAAAAGTGTCCTGAAATGAAGGTCTTTTCACTTGGAATTGTAAGC
ACACCTCGATTGAAATATGAAAATTTTTATTTAAAGAAAGATTACGATGATGAACGGTGTCATCCTAAAT
ATCCCAAAGAGATGTTGGTGGAAGATATGAATGTCATGACCAGAGAATATTGGGAGGATAATGTTGATAC

CGGAATTCCAAATTTATTTGCCGAACAGGTTTGTATATTTAATTACCTTTTCATATTTGGTAATAATTAA
TTTTTATTATTTGTGTGTTAGAGTATGAACTTTAATGAATTTATTTAATTAATGCAGAGTTTGGAGGAAA
ACCGATCTGAAAATTCTTCTTCTTCAAAGAATAATGTTGAGAAAGAATAAGGAATTATATGTGTGCATTT
ATTTGCAAAGGAAGCTGCCAAATAACCTACTCAATCCTAGCAGATGCGTTCTTTGCAAGTCGGCTGTCGA
AGACTTGAACCATATTTTCACAACCTGTGCCAATTCGCAAATAGCCTCTGCTGGGTGAAACTGCAGTACC
AAATTGGTGGAAACTTTGATACAAACAATATCAAAGCCCTCTGTTGGTCTCTTAGCTTGCTGAAACAATC
AAACAGAAAGAACATCATTCTCTCGATGTAGGTGTGGATCTTCTTTTGTCCATTTGGGTGGAAAGAAACA
ATAGGATTTTCATAGACACAGAAAGAAGCTTAGTCACATTTGGGAAGATATCGAAACTTTGATTGGACCA
TGGTCGAGTAGAAACAAAATGTTCAAAGACTACAATCCAACATCAATCTTTAAACTTTAGAGCTTTGTTA
GATTAATGTTTGTTGTATATGGCTTCCCTGTAGCCAAAGAAATATACATTGTAACAATGGTTTGATGAAA
TGATAATGAAGTGGTATGGTGTGTTTCAATTGCAAAAAATATGATTCTTCAAGAAAGGCCGAGAGGATAA
GATTGTGATCGTGACATGCGCTTGGGTTGTGATTTAATTACAAATATATTCATATAGTATTTGGAGCAAA
ACAGTCCTAATTAATTAAATATAATGCGTTATTTTATTTTTCTGAATAAGTTAAATTTAACCACTAGAAA
TTTTTTACTAAATGAACATTTTCATAGCACTTTCATAATCCCCACTTCATTAAAATGAATCAATAAAGTT
CAAGGAAAGTGTAGAATGATGAATCTAAAAGAAACAAAAAAACAAGGTATATAAGTTTAAAGCAACGGTG
GTAGCACTGTAATGATCCGAGGGAGCACACATTTTTTCATAAATTATTTTATTATCATCAATAGAAGAAT
TTGAATTTCTTTATCTTTTAATTGATAATATAATTTCATAATTAGCTATGCTATTATACACTTTGAGTAC
CCTCTCGGCCTCAACAAGATTCCCAAGTTCAATAATTTAAACTTTGAAAAATGTGTGCAATCACATGTAT
TCAATTTCTATGTCGGTATCACATATTTTCATGTTTCCATACGTTTAATTTCCATATAGAAGTGAATCCA
TAATTTTATTATATTGCGAAAAAATCTACCAAACATGGAAATTAGCTATGAAAATATTAGTAATATAAAT
AATAGATATGTTAGACTTATTTGAAAAAAATTAAAAAAATATTCATTTATGGGTTTAAATTTATTTTTAA
ATTTTTTGTTTTTATATATTATTTTAAAAATATATATTGAAATTAATATTTTATTGATATTATATCATCA
AAATTTTTGTAAAATTAAGATCTTTAATATAACGCCCCAGACCCAAGATTTGGAATTCGGATCCCTGACA
TTCTTTTGCATCCACTGTGATCTGATAACATCATCTTTACTTGTCTTAAATTATTAGACTGAAAGTTCTC
TCCACAAAACAATAGGAGTCATTTCAACATACTTTGTCCTCACTCACAGCATCCCTATCACTCATAATTA
ATGTAAAATTTTATTATATTTGTAAATATGTTTTGGTGTACTTTACCATATATTAAAACCTACTACTAAA
CAAAATGAAATGATTAAAAAGGAAGGGAAGGTATTAAAAATTATAAATTTTAAGAAAGGAAAAGAAGAAG
AAGGAAAAAAAAAAAAGAGAATGATGAGTGAGAGGCACCAAGTGAGGACATATACTACTCTTTGAGTACA
TAACCTAATGGTTAAGAAAAAAAAAAAATCTCATATCAAATTCAAAGTGCCATGCTATTATTACTTAATAT
TTTATATGGAAGTTAAATAAATTGTTAGAGAGAAGTCTTGTTTTCTGTCTGTTTGTTAACTCATTTTTGT
AATTAATGTTTAATTTGATCATTGTCATTCCAATTAATTGTAACATAATTTTCTGCCCAATTTATCTCTT
TTGCTTTCGTTTTTGTTTAGATACCCTACTCGGCTACTCCTCAACTTTTCCTCATTTCATTTTCAGTTGG
CCATATGAATGATTTTGTTTTAACCTTATCTTCATTCTCCCACTTCTTTTTTTTCTTTTATTACTTTATA
TATTCCTTTTCTTTTCGTTTATTATCTCAGGGTGGGCCAAACATATTTTTAGTTTTACGACATAATCATT
TGGTTAATGTTTTTTTGTTATAGTTGTTTATTTTACTGAATTCGATCGATTATTAAAATAGATATATAGC
AAGCATAAGAAATTAAATAAAAATCTTAAAGTACTTAATATCTAATGAACAACCACATAAACAATGAAAC
TAAAATATGAACTAAGAAAAGTCAAATCATGTGCATATTGATGAATCTACACTCCTCGATCTCTTCAAAA
TCTAGCAAAATCATCTCAATAGAATTGAACATCAACAATCTAAGTATACTTGTGAATATACCATTTTTCG
GAAAAATAAAAATTAGGTAAGAGAATAAGAACTTATGGGTAAATTAAATCATTGAAATGGATTGAAATCT
AAATGTTGTGTGATTGCTACTATCTGATTTTCATTTGATATTGACTGTTTTCTATTGCAATATGATATAC
TAATTTTCAAGGATATTGATTGCATGTTGTCTAATTAATATCTGATTTGTATATTTGATATTTGATTGTT
ATTTTATACTATATTTGTTATTTAATAGTTGTTTGATGGCTATTGATTATTGATTGTTCTAATAATATCA
ATTGCTATCTAATTGTTGTTTGATTAAGATCTAATAATATTGGTTGTCAATTATAGAAATTGTTATATGA
TAATGATTGTAAAATTGTTGATGATACTAATTGTTATTTGAATATAACAATCGATGATATTGATTGTTGT
TTGATTGCTCTCAAATTACCATCTACTACTTTAAACTAATATTAATTGATACTTGAAAAAGCACAAGAAT
GAAATCCAAAAACTATCTCACTTTCAATAAAATAAAAAACACTTTTTTAGTTTTAAATCAAATACAAAAT
GACAGTTTGTTCAATTTTAATTTAAAAATTAAAAAATAAGAAAAACAAACAAAACATATCTAAAAAAACA
AACAAACAAGACATATCTAAAGAGATAAACACTTTTCTCAAAATTTGAGAAATTGTTCTAAAATTCCTTT
GTTTTTTTCTTCTTCTTCTAATTCTTCTCTCTATTTTTTGTTTCTTCTTCTAACTCTTTCCTCTTCCTCG
CAAATCCAATAAAAAGAACCATAGAATCGTTTCAAGATTTATTCAGGGGAAGGGGGGTTGGATTTTTTCA
AAGTGAGTTTTGTTCTATTTTTGTTTTTGTTGATGATTATTAAATGACGAAGTTGATTGGGCAACCTAAA
TTCGAAGTTTCAAAGCTGTAAGTGTAACGACCCAACTTCTTATACTAAACCAAAGTCATTACTAATTTAA
AAGATAAATAAAATTTGTAAAATTTAGATAAAAATAAAACAAAACCTCAAGATTTTTATTTAAAAGCTTA
AACAAATGTGATTAGAGATAAATATAAAATAAAATCCTAACTCGCACCCTATCTAATTTTAAAGAAATAA
AATAACAAATAAAGAATAAAATAAAATGCAAACATTAAAATCTGAACTATGACATAAAGCGGAAGCAACG
TATCCCTATGACTCGTCATGGTCATTTCTGGTCATTCGCTAGCTTGCCTTTGCTCTTACCTCTGTCTTTG
CCTGAAAAAACGAAAATGGAAAGAGTGAGTATAAAATACTCAGTAAGGGACCCACTACTAGTCCCGCTAG
GCGCCTGTTAACTTCCAATTAGAGTCTTGAAAAGTGGTACCCAAGAACTGGCACGTTCCCGAACACGTGC
AACATGTGATCCCGGAGGAACATATAATTGGTCTTTGGTGATCCCAAAAGGACACCTAGGACAAACTTGT
CTGTAGTGTACTCGGAGGAAACACTAAGACAATCGGGCTACGAGCGATCTCGTCGAATCACTCGAATCAT

GTCTATGTCAATCCTGTCGGACTACGCAATCCTAAAAAGGTGGTGATCCCGAAGGACACCCATGCAGGTA
CGACTCTAATAGGATAAGCTAACATACACCCTAACCATAGCATGCACGTAACATATCATCATCACGTCAT
ATCATATGATTCTTCAAGAAAACAGTTATTTTTCATTAACAATACTTAACGTTTCTAAGTAAAACATAGT
CTAAAGGAGGTTTTTCTGTTGTTTGCAAGAAAGTAAAAGTGCATCTACAAATATTAAAGCAAGAGATTGA
AATTAAATATGGATAATGCAATGAGAGAAATATTGAGAATTAGTGTTATATGATTGAGAGGAGTCTAGCC
ATGATCGTATATGGAGCACTCATAACACCCTTTATTATATCTGATCCCATCAAATTAATTAGAAGTAATT
ACAAATTTAACTTTTGTAATATCAATACATTTTGATGTCTCAATCTAATTGTTAATCTGTTTCTTTTTTC
TTGGACGTAAAACCAACCAATTAAATTAGATATAACAATGAATTTAATTCACACACACAAATATATATAT
ATATATATATATATATATATATATTAAATTCAACCAATATAACGTTATTTCACTTTTTTCTCTCTTTCAC
AAAACTATAATAATATATACAACAAAAGGTAAACCAATATGACTCTTCAAAAAAGGCCGAGTGGATGAGA
TTGTGATCGTGACATGTGTTTGGGTTGTGATTTAATTACAAATCTATTCATATAGAATTTGGAGCAAAGC
CATCCTAATTAATTAAATATAATACGTTATTTTATTTTACTGAATAAATTAAATTTGATCATTAGAAAAT
TTTCACTAAGCGAACATTTTCATAGCACTTTCATAATCTCCACTTCATTAAGATGAAATAAAGTTCAAGA
AAGTGTAGAATGATGAATCCAAAAGAAATAAAAAACAAAGTCGATAAATTTAAAACAATGATAATAGCAC
TGTAATAATCACAACAAACATTTTTCCATAAGTTATTTTGTTATCATCCTCGTAAGAATTCGAATTTCAT
TTCTTTTTATTGATGATATAATTTCATAATTAATTATAAGTGGATTTTTCAAATGATTGGATCCATATAT
CAACTTCAATCAAATTTTTAAAGGTAAAATTTTAAATTTTAAAATGCTTTTAATTAGTACAAAATTGAGA
AATCACCGAATACAATTTCTCTTTTATGTATGAGATTTGAACCTATCTTTTTGTCTGGAGACTAAATTAC
TTATTACGTAGCACTTGAACGTCATATTGCTCAAATTGTTACTATTTGCCTTAATTTGAAAGAAACATTT
TAAACCGAATTGAAGAGATGTAGAAAGGACAAAAAGAAAACATATGCATAAACAGGCTACAAATCATTCA
AGGGATCCAAGTACTGTACATACGAAAAAAAAGTTTGCTAAAATATGAAAACAAAGAACAGTCAACAACA
TAGTCCAACTGAAGTAAGAAAAAAACAAGCAAGTCACCGAATGAAGGACATAAACTATCTGGACAAGTCT
CGTAGGACTTTGCAAAAGTTCTGACTCTCATCATCAGTCATCTGTACGAAACCCTACATGTCGTCCATAC
GAGACAACAGGTTCCTTTGGCACAACGCTCTATCCAAATTGGTTAGTTTCGGCATCTTACGCAATAGACG
TAAGAATTCTAAGCACATTCGGCAATCGTCCTGAGTTGGTATTTCGTACACTCGAGGGCCATATGTATGA
CTTCAATCTCACCTTCCCGTTGGGTTCCCCTATTTCTCTTTGATCTGCTCAATCCAATCCTACCTTCCCG
TTGGGTTCCCCTATAATTCACTAAATATTTATTTCTAGTCATCATCGAAATTGAAACTTGTAAAAACCCT
TCTTGAAAACAATTTACTTTTTTTTTTTCTTACAAGTTATTTTTAACATTATATGAAAAGTTATAGTAAA
GTGTGTTTCCAATTAAATTACCTTTTTCTAACGACAAAAAAATTACAGATGAGATTTTTGAAAAAGTATT
TTACAAAAGTTTCCTAATCAATGGAAGCTTTTTTAATACAACTTTGGACGATGAGCTTAACACATTTTAC
ATTGTTGTCAATTTTAAAGTTATGAACGTATGGTGTCAATGTACTTTTTAAAACACGTAAAAGTGGGGAA
CCTATTTCTTAACTTTTTAAAATCAGAACACCAATTTGAATCCGATGTTCTAATTCTTATAGCGTTCCAA
TTAATCACATTGACGCCTATGAGCTACGTATGAAACTGTATTAATACAAAATATTGCCAATTAATTTCAT
ACTCAATTTTTTTTTACACTAGTGTGACTGGATGAAGTGCATTTAAGAACATTGGGATGCTTATTTGGAA
AATAAAACCTAATTTCGTGCATGTAAAAAAAAGCCAATAAATTGAAAGTTTGAAATCATAAGATATAAAA
TAATAATACAAAATTTATTGAATAAGTGAAATGTCCATAGAAATGTCCAAAAATTCAAACATAAGAAAAT
CAAAGTTTGTCCATTTCAAATATAATTAAAGTAGACATTTGAATCTAGATACGGGTAATCCGTATTACCT
CTTAAAAGGGTGGATGACCGCAGATATTCTACGAAAAAAATGAAGACGTTACGAAACGACAAATGGCGAA
AAGCATACACTATTTTAAAAAAAATCCCAAAATGAAGGAAAAAAAAATAAGAAGAAAACATTGAGTACAT
ACCTTTTGCATGGACAAATGCCTTGCATTTGAGGATTCCAAAATGTAATACGTAAAAAAACAAACAGTGA
AATGTAGAAAATATATTTTGGAAAAAGTTTGTTACGAGCCTCAGAGTCATGGAATCAGTTAAAAAATGTG
AACTAGATTTTATAGTAGTGGAAACATCAACAACATAGAACAAAGCAAATTAAGAGAGAAAATATAAGAT
ATAGGCAGCATGCAAGAAACCACAGAAAATGCATCGCTTGAAGTAAACACATGTAACGACATTAAGACAA
GATTCATGGTGGTTGAAACATCAGTGAACATACTTGAAGGTAGGTAACAGTACGTATATTTGCATCCAAC
ATATCATGAAAAAAGAAGACAAGTATTCATGGAAGGTTCCTAATAATATAGAAAGAACAATAGGAACAAA
GAATGTGCATGGAAAAGTCCTACAATGATAGGGCAGAAAGTGATGGATAATGTAGCAAAAAATGGAAACA
AACAAATTACATACAAAAAATAAAGTAGCAAACGACAGAAGAAGTGAGCAATAGAAGTAAACAGAAGAGG
AAAGGTGAATATATAGAGGTGGATAAGATAAGTTATTATGGATTTATTGAACACAAACAAAAAGAAATCA
GGGAATCTTTGCAAATGAGAATGTGAAAGCATAGAAAAGCCACACATGTGGTTAAACCCTATCCATTGCA
ACCTAGCATTTTTAGCACAGAAGGGGTAGCTTGTCAAAAATATTGAAGAGACTAAACAATCTACAACGAA
AGGGATTGGGCGACCAAAATTTATCACAAAAAGTACTGACTACAAAAATAAACAAAAGAATGAAGAAAGT
ACATAATCACAGAGAAGGGAATTTACAATGGAGTTTGATCATAATCAGACAACCCAAACAAGAACAAATG
AAGGTAAAAATAATACAGTGTGAACCATTGCACAACCAATCAAAGTAGGGGACCAAAAACATATTTCATA
GTTTTTAAACAGACAAATAGGTAACAAGGAAATAAATTATGACCTATAAAGTATAAAAAATAAACAAAAT
TACATAATTCAAACATCATGAAATGTGAGGATACAAATTCAGAAATCGTGAAATAGATGTACTCATCATC
GACTTTCATTAAAAAAAAAATTACAAAAGAGAAAATATACCTCCCTACGGTGCGCCAAGCTTACCGAATG
CCATAAAAAAAAGGGTGTGGCATGTAGCATCGGTTCGAAATAAGTGAAGGCAAAGAGAAATCGGTAACGAA
AACACCCACGGACAAATCGACAAGTGCAGCGGCAAGGCGAGTTCCAATTTTGTAAACCCCAACATGCAAG
TCGACGGAGAACACCGATCGAAGATGTGATTGAGGATTTCAGAGGCAAAGAATACTCCCACCGTTTAACA
AAACCTACAACCGAAAAGCCAAACGAATTTGGGGGCAAAACCTTTCGGCCAAGCCTTCAATAAATTCGTG

AAGGGTTTTGGAGAGTGAGAAAGAAGGTGAATAGAGATGTAGATACGGTAACAACATCGTGAAAAGGGAA
AAATGCAAATACGGAGAAGAAGAAGAAGAAGAAGAAGAAGAAAAACGTCAGAAATCGAAGGGGGTTTAAG
TCGCCTGACGCTATTCACAACGATTTCAACTCCGAATCGAAGAGAAACATGGAGAAGAAGAAGAAACAAG
AAAAAAGAAGAAGAAAGAAGAAGAAACGCAAGTAGTGTTCGATGATGGAAGAGAAATCGAAAAACCAACA
CGGGTTGAAGGGATGGGTGAAGACGAGTTGAATGGGGAAGTGGAGAGACAGAGTTTTTGACAGAATGAAA
GAGAAAGAGGGAAGGATTTGAGGGCAAAATCGGGTTTAGGGAAATCCTAAAGCTAGGGTTTTCATAACCC
TAGCACCAAACAGGGGTTATGGTTAAGTAAACATAACCCCACAGTGTTAAATAGCCCCGTAAACAGCCCC
TAAAAGTCTATATAACAATGGTAGAAATCTATTGCTAATAGACTTGTTATATTTATAATTTTCTTAAAAG
ATTATTATATACTTAATTATTTTTACTACAATTATTACCGATTATAATTACTTAGATATTACATTTGTAT
TTAAGGTGTATAGTGGTGCATTGATACAATCGGTCAAAAGGACTGATGGTGGGCCGTTATGCTATTTTTC
AATTTCGTCGTTTCCACCCTCTCCTCGACAAGATTCCCAATTTGAATAATTGAAACTTTGAAAAATGTGT
CCATCATAATTATTAAATTTCTATTTCGGTATTTTCATGTTTCCATATGTTTAATATCCATATATAAGTG
AATCCTTTAGTTTTATTATAAGGTGAAAAATCTACCAAACATGAAAAATAGCTATGAAAATCTCAGTAAT
ATAAATAATACACATATTGACTTATTTGAAAAAATTTTAAAAAATATTCATTTACTGATTTAAAATTAAA
CTTTTTTTTTTTGTTTTTATATATTATTTTAAATATATGTATCGAAATTGACATTTTATTGATATTATAT
CATCAAAATTTTTGTAAAATTAAGATTTTTAATGTAACACCCCAGGTCCAGGATTTGGAATTCAGATTCC
CGATATTCGCTGCATCATATGAGACCTAACAACATCATCTTTATTTGTCTTAAATGCTTATTAGAGTGAA
AGTACTTTTCCTATTTTTAGAATTTGGAATTTGTAAAGTTATTCTCATGTGGTTTTTTGGGCAGTGGAGG
AGTTGGAGAAGTTGATTGGTGTGCAGATCTATGTATAGGACAGATCTTTGAAGAAGAAAAAAGAATTGGA
AAGATTAGGGATTTGGTTAATTAAGCAATACGGTAAGTTGGATTTCAGAATTGCACTTCATGATTGGGTT
TCGATTTTAGGAAGAATGGAATTGCAAATTCAATTTAACATAAAATTTCTTTTTTTTCCTAAATTAATTT
TTTATTTGATCTTCGGACTCATCCAATGCAATTTACATGTCACTACAAATAATATCCATGTCAGTACACA
TAACGTGTCTAGTAGATATTACGTTAGCTTAATGTTAATTCAGGGAACATTTAGAACTATTTTGCAAATG
CGAGGGATTAAAATGTATGCTTTTTAAACTTGAGGGGACCAAAAGTATACTTTACATTATTTTTTTTCTC
ATTTATTTAACAATTTAATAAACAATAATAATTAATTAGTGAAAAGTTACGAATGACAATGGATATTAGT
TTTAAGAACAAAATGTATTTTTGATCCATAAGGTTTGAAGTTGATATCTGTTTAGTTTCTTAAGTTTCAA
AATAAACATTTTAGTACTCAAGATTTGATGTACGTGAAGTCACATTGAATGTTAGTTTCAAAATGATGTG
GCAGTTAAATATAATTAATCAGCAAACTGAATTGGCAAAAATGATTATAATATTAACTTTTTGTATGATG
GGGGCAATATCCATCTCCTCACCCTCTTCTTCAACCTCCCAATTACCATAAGTGCAAAATTCTTTTGTCG
TCCATATTATTGTTAATGAAGAACAAAGATGAATTTAACCAATCACCATACATTTTATTTAGATCGTATG
CAACATTTTCTTTTTCATTTCTAGCCTAAATCCCTCATATTTTCCCCTAAAATTCCTCTGTTTTTCTCTT
CTTCTTCTGATTCTTGATCTCTCTCTCTCTCTCTCTCTCTCTTTTTGGTTTCTTCTTCTAACTCTTCTCT
CTTCCTCGCAAATCCAAGAAAAAGAACCATAGAATAGTTTCAAGATTTTTTTCAGGGGAAGGGGGGTTGG
ATTTTAATTTTCAAAGTGGGTTTTGTTCTATTTTTGTTTTTGTTAATGATTATTAGATGACGAAGTTGTT
TTGGGCTACCTAAAGTCGAATCTTCAAAGCTGTAAGTTTTGTTATTAAGAGTGGATGAGAAAGAAACTTC
CAGGCAAATTTCCAATGATTTTTACGCTTACACATACTGATATTACCTTCTTAACTTACTCTTGCCATTT
CTCACTCTATTAACAACAAAATGAGTGTATGTAAGTGATAATATTGGTTGGACGAAAAAGAAAAAAAAAA
AAAAGGTTCTAGGAAACAAAAAGAAAAACGAAAAAGGCAAGAAATGGAAAAGGAAACGTTGTGTATGATG
ATTAAAATGTACGTTGATAGCTAAATTCAACTTTGTTCTTCATTAGCAACAACAAGGGACAACAAAATGA
AAATTTCAGCACAGTTATGGAATGTTGAAGAAGAAGGTGACGAGAGAGATGCCACATCATAAAAAATTAA
TATTATAATCATTTTTGTCACATCAGTTTTCTGATTACTTATATTTAACCTACACATCATTTTGCCGTTA
CTCAACTAACGATCAATATAACCTTTAAAGCTCGATGATTAAAATGTTTATTTTGAACTTTCTGGGACTA
TATATAAATGTATATATATCAACTTCAAATCTTAGCGGTCAAAAGTGCATTTTGTTTTAGTTTAAAACAC
CGAGATGTGACGATTAATAATGAGATGAAAGAATTATGTCTTTATTTTATTCGATATTAAGCATACATTA
CTTCGAGATGAAAGACCTCTACCTTTAGTTATTCGATCTCGAGCCTTAATTGAATCGAGATGACCCAAGA
TTAATAGATCGGAAAGAAGATATATATCGTAAGGGCATATATGAAATTTATGAAAATACGATCATGTGTC
ATAAACTTTTCTTATTTTGTTATATAGACCGTAAATATTATAGATTTTGGTTACATTTGTGTAAATTACT
CTATTTTTTTTTAGTAAAACAAATGAATTAAATTTAAAAAAAAAAATTATTAAGAGTTGAAGTAATTGGG
TGTTTTCTAAAAGATTGAAATAAATACCTTTATTAACTTTGCAAATATATTTTCAAAGAGGCTAACAAAT
CAAATATATTTTCTAAAAAAATCACATTGCGTTGGGGATAAGGTAAGAGTAATGGAAAAATGAAAGGTTG
TGAACAATTCGTACTCTTACTTATGAGATTTGACAGAACAAATATTTTTCAAATACTCTTTTTACGTTTT
CTTTTTTAAAATTAAATAACAAATACTTTTCCACTGCTTTTCTTTTCTTCCATTGACTCCTCAACTAATA
ATTTTGGTCTTCCCATTATTATCTTCTTCTTCTTCATTTTTCTCTGCACTGTCTCTTCTCCATTTCAACA
TCTTCTTATATCTTCATTCAGGTTAAATATTTCATCCCTTACCATTTTTATTTACATTTTCCATCTGATC
ATTTCTATACTTATAAACTCGATAATTTGTTTAGACTTTCTTTAGATAGTTTTTACTTGGTGATCTGTAT
GTGTTCACTCCTACTTTTTCTGTTAGGCAAAATCTTCAAAATTATTATGGTTCATTCCTCGTGGTTTTTT
AGCTCTTCAAGTATTTTTGTAGGTTTTGATCCTTTTCAAGCTCAAGAACAGAGGATGATAATGGACATCC
TTATTTCAGTCATTGCAAAAATTGCTGAATACACTGTTGAGCCTGTTGGACGCCAACTTGGTTATGTATT
TTTCATTCGTTCCAACTTTCAAAAACTTAAGACTCAAGTAGAAAAGCTGAAGATTACAAGAGAGTCTGTG
CAACACAAGATCCATAGTGCAAGAAGAAATGCTGAAGACATAAAACCTGCCGTTGAGGAATGGTTGAAAA

AGGTCGATGACTTTGTTCGAGAATCTGACGAGATATTAGCCAATGAAGGTGGACATGGTGGACTCTGTTC
CACCTATTTGGTCCAACGACACAAGTTAAGTAGAAAAGCAAGCAAAATGGTAGATGAGGTTCTTGAGATG
AAAAATGAGGGGGAAAGTTTTGATATGGTATCCTATAAAAGTGTTATCCCATCAGTTGATTGTTCACTTC
CAAAAGAGCCTGACTTTCTTGACTTTGAGTCAACAAAGTCGATTATGGAACAAATAATGGATGCACTATC
TGATGGTAATGTCCATAGGATTGGAGTATATGGGATGGGGGGTGTTGGCAAAACAATGCTAGTGAAGGAT
ATTTTAAGAAAAATTGTGGAGAGTAAGAAGCCTTTTGATGAAGTGGTAACATCCACGATCAGCCAAACAC
CAGATTTTAGAAGTATCCAAGGACAACTAGCTGACACGCTAGGTTTGAAATTAGAACAAGAAACAATAGA
AGGAAGGGCTCCTATTTTACGAAAGAGGTTGAAGATGGAGAGAAGTATCCTAGTCGTGTTGGATGATGTC
TGGGAGAATATTGATTTGGAAACAATAGGAATTCCAAGTGTTGAAGATCATACGGGGTGCAAGATCTTGT
TTACCACTAGGAATAAACATTTGATCTCAAATCAAATGTGCGCCAATAAAATTTTTGAGATAAAAGTTTT
AGGAGAAGATGAGTCATGGAATTTATTTAAGACAATGGCAGGTGAAACTGTGGAAGCAAGTGATTTGAAG
CCTATAGCCATTCAAATTGCGAGAGAATGTGCAGGTTTGCCTATTGCTATTACTACTGTTGCTAAGGCAT
TACGAAATAAACCTTCCGACATTTGGAATGATGCCTTAGATCAGCTTAAAAGTGTTGATGTGGGTATTGC
AAACATTGGAGAAATGGAAAGGAGAGTGTATTTGCCACTAAAACTGAGTTATGATTACTTGGGATATGAA
GAGGTGAAGTTATTATTCTTGTTATGCAGCATGTTTCCAGAAGACTTTACCATTGACGAGGAAGAGTTGC
ATGTATATGCCATAGGCATGGGATTCTTACATGGTGTTAATACTGTGGAAAAAGTACGATGTAGGATTAA
AAAATTGATTGAGGATCTTATCTCTTTGACTTGGCTTTAACAATATTCTGAGTATGTGTGCAATTTTGTG
GTGGCAAAATGAAATGAGGGATAANNNNNNNNNNNNNNNNNNNNNNNNNNNNNNNNNNNNNNNNNNNNNN
NNNNNNNNNNNNNNNNNNNNNNNNNNNNNNNNNNNNNNNNNNNNNNNNNNNNNNNNNNNNNNNNNNNNNN
NNNNNNNNNNNNNNNNNNNNNNNNNNNNNNNNNNNNNNNNNNNNNNNNNNNNNNNNNNNNNNNNNNNNNN
NNNNNNNNNNNNNNNNNNNNNNNNNNNNNNNNNNNNNNNNNNNNNNNNNNNNNNNNNNNNNNNNNNNNNN
NNNNNNNNNNNNNNNNNNNNNNNNNNNNNNNNNNNNNNNNNNNNNNNNNNNNNNNNNNNNNNNNNNNNNN
NNNNNNNNNNNNNNNNNNNNNNNNNNNNNNNNNNNNNNNNNNNNNNNNNNNNNNNNNNNNNNNNNNNNNN
NNNNNNNNNNNNNNNNNNNNNNNNNNNNNNNNNNNNNNNNNNNNNNNNNNNNNNNNNNNNNNNNNNNNNN
NNNNNNNNNNNNNNNNNNNNNNNNNNNNNNNNNNNNNNNNNNNNNNNNNNNNNNNNNNNNNNNNNNNNNN
NNNNNNNNNNNNNNNNNNNNNNNNNNNNNNNNNNNNNNNNNNNNNNNNNNNNNNNNNNNNNNNNNNNNNN
NNNNNNNNNNNNNNNNNNNNNNNNNNNNNNNNNNNNNNNNNNNNNNNNNNNNNNNNNNNNNNNNNNNNNN
NNNNNNNNNNNNNNNNNNNNNNNNNNNNNNNNNNNNNNNNNNNNNNNNNNNNNNNNNNNNNNNNNNNNNN
NNNNNNNNNNNNNNNNNNNNNNNNNNNNNNNNNNNNNNNNNNNNNNNNNNNNNNNNNNNNNNNNNNNNNN
NNNNNNNNNNNNNNNNNNNNNNNNNNNNNNNNNNNNNNNNNNNNNNNNNNNNNNNNNNNNNNNNNNNNNN
NNNNNNNNNNNNNNNNNNNNNNNNNNNNNNNNNNNNNNNNNNNNNNNNNNNNNNNNNNNNNNNNNNNNNN
NNNNNNNNNNNNNNNNNNNNNNNNNNNNNNNNNNNNNNNNNNNNNNNNNNNNNNNNNNNNNNNNNNNNNN
NNNNNNNNNNNNNNNNNNNNNNNNNNNNNNNNNNNNNNNNNNNNNNNNNNNNNNNNNNNNNNNNNNNNNN
NNNNNNNNNNNNNNNNNNNNNNNNNNNNNNNNNNNNNNNNNNNNNNNNNNNNNNNNNNNNNNNNNNNNNN
NNGAAGAAGATTTTAAGTAATCATTTTCGCAATTTGTTATGTATTTTAAAACACGATAAAATTATATTTT
TTTAGTTAGCATCATTTGTGATGATATGTTTAACCCGATGTCACAAGCTTAATTTTTAAGGACATAAATT
TGTAGGTAACACTTCCTAATTTGGAGAAGTTGGAAATTGTGAATGCAGAGAGTTGAAGATGATATGGAGC
AATAACGTGCCAATTCTTAATTCCTTTTCCAAACTCGAGGAAATAAAAATTTGTTCATGCAACAATCTTC
AAAAAGTATTATTTCCTCCAAATATGATGGGCATTCTTACATGCCTTAAAGTCTTAGACATTAGAGATTG
TAAATTGTTGGAAGGGATATTTGAAGTGCAAGAGCCAATTAGTGTTGTTGAGAGCAATAATGTACCCATT
CTTAATTCCTTTTCCAAACTCGAGGAAATAAGAATTGGGTCATGCAACAATCTTCAAAAAGTATTATTTC
CTCCAAATATGATGGGCATTCTTACATGCCTTAAAGTCTTAGAGATTAGACATTGTAATTTGTTGGAAGG
GATATTTGAAGTGCAAGAGCCAATTAGTGTTGTTGAAGCGAGTCCTATCGTGCTCCAAAATTTAAGTGGG
TTGGAATTATATAATCTTCCAAACCTTGAGTACGTGTGGAGCAAAAATCCTTGTGAGCTTCTGAGTTTGG
AAAATATAAAAAATTTGACCATTGAGGAATGTCCAAGACTTAGAAGAGAATACTCAGTCAAAATTCTCAA
GCCACTTGAATATGTAAGCATAGATATCAAACAATTGATGAAGGTTATTGAGAAGGAAAAGTCAGCAGAT
CATAATATGTTGGAATCAAAGCAATGGGAGACTTCATCTTCTTCTAAGGTACGTATATATTCTACAACAA
AACATGTTTGTTCAATTTAATTTTCAGAAAATAAATTGTTTTCAAGACCTTAGTTGAACAGAATTGGTTC
TGTTAGTTGAACAGAGTTTTAAAGAAAACAAATTATTATGTGTGCAAATATAAGTTATTATTGCATTTAT
TTAATAGATTTTGATCATCAGGATGGGGTTCTACGGCTGGGAGATGGTTCTAAGTTGTTTCCAAATCTTA
AAAGTTTGAAGCTATATGGTTTTGTTGATTATAACTCAACCCATTTACCAATGGAAATGTTGCAAATCTT
ATTCCAACTTGAAGTCTTTGAATTGGAAGGAGCATTTATTGAAGAAATTTTCCCCAGCAATATACTGATT
TCAAGCTCTATGGATTTACAGAGTTTGACTCTATCTAAACTACCCAAGCTTAAGCATTTGTGGAGTGAAG
AATGCTCACAAAATAATATTACCTCAGTTCTTCAACATTTGACTGATGTATCCATTTCTGAATGTGGTGG
ATTGAGTAGTTTAGTATCATCATTGGTGTGTTTTACAAACTTGAAAGATCTTCATGTGATTAAATGTCAT
AGACTAACCCATTTGCTGAATCCTTCGATGGCTACAACGCTTGTGCAACTTGAGTATTTGACAATAGAAG
AATGCAAAAGGATGAGTAGTGTAATTGAGGGAGGATCAACCGAAGAAGATGGAAATGATGAAATGGTTGT
ATTCAACAACCTACAACATTTATACATTTTTAATTGTTCCAACCTAACAAGCTTTTATTGTGGGAGATGC
ATCGTTAAATTCCCATGTTTGGAAAGAGTATTCATTCAAAATTGTCCTGAAATGAAGGTCTTTTCACTTG
GAATTGTAAGCACGCCTCGTTTGAAATATGAAAAGTTTACTTTAATGAATGATTACGATGATAAATGGTG

TCATCCGAAATATCCCAAAGATATGTTGGTGGAAGATATGAATGTCATCACCAGAGAATATTGGGAGGAT
AATGTTGATACCGGAATTCCAAATTTATTTGCCGAACAGGTTTGTATATTTAATTACCTTTTCATATTTG
GTAATAATTAATTTTTATTATTTGTGTGTTAGAGTATGAACTTTAATGAATTTATTTAATTAATGCAGAG
TTTGGAGGAAAACCGATCTGAAAATTCTTCTTCTTCAAAGAATAATGTTGAGAAAGAATAAGGAATTATA
TGGATATTGTTGTACACTACTTAATATATCATTTCATCCACAAGGAAAAGGTCAGACTCTTGAAATCCTC
CATTCTTTTTTATGAGAGAATATCATCCAATGTCAAATTGAAAAGTCTCGATAGATTTGTTAAATTAATT
TTTGATACAAGTCATAAAATGTTAATTAGTATAATAATAATATATCTGATCCCATCAAATTAATTAGAAG
TAACGACAAATTTAACTTCTGTAATATCAATTCAATTTGATGTCTGCATAAAATTTGATGTCACAAATTT
AACTTCTGTAATATGAATCTTTTTTTTTTTCCTTTGGACATAAAACCAGCAAGTTAAAATAGATATAACA
ATGAATTTAATTCACATATAATAAATTCATCCAATATAATGTTCTTTCACCTTTTTCTCTCTTTCACAAA
ACTGTAATAATAATATCTACCACAAAAGGTAAACAATTAATATGATTCTTCAAGAAGGTTGTTTATTTGG
TCAAATTTTCATGAAAGTATTAATACAGTGTATGTTTTGCAAAGGAAGCTGCCAAATAACCTACTCAATC
CTAGCAGATGCGTTCTTTGCAAGCCGGCTGTCGAAGACTTGAACCATATTTTCACAACCTGTGCCAATTC
GCAAATAGCCTCTGGGTCAAACTGCACGACAAAATTGGTGGAAACTTTGATACAAACAGTATCAAAGCCC
TCTGTTGGTCTCTTGGCTCGCTGAAACAATCAAACAGAAAGAACATCATTCTCTCGATGTAGGTGTGGAT
CTTCTTTTGTCCATTTGGGTGGAAAGAAACAATAGGATTTTCATAGACATAGAAAGAAGCTTAGTCACAT
TTGGGAAGATATCGAAACTTTGATTGGACCATGGTCGAGTAGAAACAAAATGTTCAAAGACTACAATCCA
ACATCAATCTTTAAACTTTAGAGCTTTGTTAGATTAATGTTTGTTGTATATGGCTTCCCTGTGGCCAAAG
AAATATACATTGTAACAATGGTTTGATGAAATGATAATGAAGTGGTATGGTGTTAAAATCACCTTTATCA
CTCTGTGTTTCAATTTCAAAAAATATGATTTTGAACATTTTGATAGTACTTTCATAATCCCCACTTCATT
GATATGAATCAATAAAGTCTAAGGAAAGTGGAGAATGATGAATCAATAAAGTTCATGAAAAGTGCAGAAT
GATGAATCCAAAATGAAAAAAAAACAAGGTCGATAAGTTTAAAGTAACGGTGGTAGCATTCTAATGATCA
TAAGGGAGCACACATTTCCCCACAAATTATTTTATTATCATTCACATAACAATTCAAATTTCTTATCTTT
TAATTGATATATAATTTCATAATCAGTTATAATTGAATTTTTCAAATGATTGGATCCATATATCAACGTC
AATCAAATTTTTAAAGGTAAAGTTTTAAATTTTAAAATGTTTAATTAGTATAAAATTGAGAAATCACAAA
GAACAATTTCTCTTCGATATATGAGATTTGAACCAACCTTTTTGTTTGGAGACTAAATGTGCAATATAAT
TTTATATAATTTTATAAACATGCAAATATTGAAATTAGATTCAAAAGTATGTATAATAACAATTTTTTTT
TTTTAAATCGTAAATGTAGCAAAATTTATTAGAGTATTTATTAATAATTGAAATGTGAACCATATTGCAA
AAATTGGTTCTATCACTAATAGATCATAAGAGTCTTTAAAAAAAATGATTAAAAATAGTTTGTTGCTAAC
AAACTTTGTTATTTTTATAATTTTTTTTTAAATATTATTATATACTTAATTAAATGTCTAAAATTATTAC
CATTATAATTACTGAGATATTACACGTGGATTGATGGTGGGCCCTTATATGCTATTATACACTTTGACTA
CCCTCTTCCCAACAAGATTCCCAAGTTAAATAATTGAAACTTTTAACATCTTTCATTACAGTTTGATAAA
TGAACGCAAACATGTGTTGTTTATTTTACTGAATTCGCGATCGATTATTAAAATAGACATGCAGTTGATA
GCAAGCACACGACATTAAATAAAAATCATGAAGTATTTAATATCTAATGAATAACCACATAAACAATGAA
ACGAAAATATAAACTAAGAAAAGTCAAATCATGTGCATATTGATGAATCTATACTTCTCGATCTCTTCAA
AACCTAGCAAAATCAATAGAATTGAACATCAACAATCTAAGCATACTTGTGAATATAACAATTTTTCGAA
AAAATAAAAATTAGGCAAGAGAATAAGAACTTGTGGGTAAATTAAATTATTGAAATGGATTGAAATCTAA
ATGTTGTGTGATTGCTACTATCTGATTTTCATTTGATACTGATTGTTTTCTATTACAATATGATATACTA
ATTTTCATGGATATTGATTGCATGTCGTCTAATGAATATCTGATTTGTATATTTGATATTTGATTGTTAT
TTTATACTATATTTGTTATTTAATAGTTGTTTGATTGCTATTTATTATTGATTGTTCTAATGATATCAAT
TGTTGTTTGATTAAGATCTAATAATATTGGTTGTCAATTATAGAAATTGTTATATGATAATGATTGTAAA
ATTGTTGATGATACTGATTGTTGTTTGAATATAACAATCAAAGATATTGATTGTTGTTTGATTGCTCTCA
GATTACTATCTACTGCTTTAAACTAATATTAATTGATATTTGAAAAGGTACAAGAACGAGAACGAAACCC
AAAAACTATCTCATTTTCAATAAAATAAAAAACACTTTGTTAGTCGTAGATCAAATACAAAAGAATAGTT
TGTTAAATTTTTTTTTTTAAAAAAAAAAACAAACAAACAAACAAACAAACAAAACATATCTAAAAAAATA
AAAACAAAAAACAAATTTTTATTATGCAAGCTGCCATCTCCTACCAATGAGATATTTTTCCATATAAAAA
ACTACCAAAAGGAAAGAGAAGAGAAGGAAAGGTTAAACTTGAAAGTTTGAAAGAATGATTGAAGTGATTT
AGATCTTTTTTATTTATTTATTTATTATTATTATTATTATTATTTAGTTGTCAGTGAAACTGAAACTGTA
GCCATCTAATTTTATGCCACTGAAGCCAACTAATTTTATGCTACAATGGTAAGATATTTTATTTATTTAT
TTTGTTAAAAAAAGAAAAAGGTAAAATCTTTTTGTAATAATATGAAATCTTATGTTTTTTAATCTTTTTA
TTAAAATAGAAAATAAAGTCATTAATGAGAATATTTATTATTTAAAAAAATTCAAATCATTTTTGCCTTA
TCACTTTAGGAAAAAAGCAAATTAATGTATTTATACAAAATCTCTTTTGATTTATCATATTAGAAAAAGC
AAAATAATTTATTTTATACAAAATTCTTATAATACCATATTTGATTTATCTTTATCACTTTAGGAAAAAA
GTAAAAAATTTATTGTGGACAAAATCTTTCAATATCATATTTTTACTATTTTAGAAAAAAGAAAATTAAT
AAAATTACATAATATCTAGTTAAATTATTTATTTATTTATTTAAATTTAATTTTCTTTAATTTTATTATT
ATTATTATAAATAGTTTTTTTATACATATTTCTTTTTAAGCTTCTGTTTTTCTTTATTATATATATATAT
ATATATATATATATATATATATATATATATATAACTTTTTATTTATTTAGTTAGTTTTTAATATTTATGA
TTTTTTTATTTTTTGTTATTTTTATTCTTATTATCATTATTATTATTTCATTTTATAATTTTTTTTTAAT
TTTTAAATCTACTATCATCCATATGTCCTTTTCTCTTTACTTATTCATTTAATCTCTTTTTTTATATATT
TATTTCTTTATTATTTCATTTAGTTCTTTTTTCATTATCTTTTTATTACTATCGTTATATTTAGTATATG

CATGCATTAATATAATAAATTTTTTGCCTAATTTATTGTGTGGTGTAGTTGTTATTTCTATTTGACTTTC
ATTAATAATTTCTTTAAATTTTAAAATACTTTTAACAATAATTGTATGTAATTTTGAATCTTTAAATTTT
TTTTCTTTAAACCATTTAGAAATTGTTTTTTGTTTTAAAATTATTTTTTTTAAACTCAAAATTAAATATA
TATTTAATATGGTTTCCTAATTTACTTTTTTAAAATAATCCACACCAATTTCACTAAAAAAAATCCTACG
ATGGAATCTAGAAATTTCTGGGAGTCCATTATTTCTTGATTCTTGAGGTGAGGGATCAATATCTTTGGGA
GTCCGAGATTTGATCCCAAGAGAAAATATATTTAATCAAAGTTAAAAAAAAAAAAACTTTATTCGAAGAC
TAGCCTATGATAGAAGTTGAGAAATTGTAATAATTTCTCATTCTCTTAAGGTGAAGAATTTTTCCTCAAT
ATAGTCTAATTAATGGGTGTATCCCACTTATTTTATGAGATCATTGCTCCAAATATTGGAGTATTGAGCA
ATGAAATAAGACATAGTTCTTTAAAAAAATTAATTTTATTATAGACATTAAATTTGACCACCGTTTTCTA
AAACGAGTGTTACGGGGTGCTAACATCTTCCCCATACACAAATGACTCCCGAACTCAACTCTATTTTTTT
CGTAGACCAGTTTTTATTTTGTTTAAAATGATTTACTTTATTTCGGTGTCCAATCACACCGTATATAAAC
ACATAGGCTACAACTAACAATCACATCACCTTTAATCAGTCAAGAGTGCATATTAGTGATGTTTTCTAGG
CAAACATGCATCAATCGATTTAGTACAATTGCTATCGTATAATTCCCATGTGGTTTACTACATTTCCAGT
TTCGGTCCAAGGTCCAGTAGTAAGAAATCCCTTACTTGAACTGTAGCTAAAATCCTTGTTCAATTCGACG
TCCAAACGAATCACCTAAACACAAACATAAGGGTTTAATAATGACATCACCAAAAATCCACATTACAAAA
CGTTCCAAAGCAACATCTACCGGCTTACCCAAATACGGGAGAGTTGGCTCCAACTATACTTGCCATAACA
CTCCTGAACTCGAGCGTCACCTCTCTAACACCTTCAGGGGATCAACAGTTAACCCAACCAATCAGTTCAA
CGTTTTATTTGAATATCGTCTTGAATCGGCTAAAGCAACGTTAGGAAACTCATTAAGTAATCATTACCAA
AGATTCACCGTGACCCGATGTGAAAGGAAAGTGGCTTATCTCAGCTCGGGAAACAAGGAGGTCGGCTTGG
CTTTGGAGGCGGCTCGACTTGGATTGGTTCAGCTCGTGGCTCGGCTTGGCTCGACTCAGCTCGCGGGTCA
GCTTGGCTTAGCTCGACTTGGTTCAGCCGTGGCTCGACTCAACTTGCTCAACTCGGCTCGGCTTGATCGT
TTGTGTAACGACCCAAATCTTTGTACTAAGCTAAGGTCATTACTAAAAAGAAACAATAATAATAGACACT
TTCTTGAAAAGAGGGAAGACTAAATTTTCATTAAAAACAGAATGTTCAAACACTGATACATAAACGCGGA
AGCAAAACTGAGTCTCCATATTGCATGTCACGGATCCTTCTCTGTCGCTCGCCAGCTTTCCTCTACCTTT
ACCTTCGCCTGAAATATTAAACATAGAAAGAGTGAGTATAAACAAATACTCACTAAGGGACCTAGTACTA
GTCCTGCTAGGTGTCTGTTAACTTCCCATTAGAGTCCTGAAAAGAAGTATCCCTAAACTGGCACATTCCT
GAACACGTGTAATCTGTGATCTAGTAGGAACAAATCTGGTCTTCGGTGAACCCTAAGGAACATCTAGGAC
AATCTGGTCTTTAGTATACCCGAAGGAAACACTAAGACAATCGAGCTGCGAGTGATCCTGTCGAATCACT
CGAATCATGTCTATGTTAATGTCACTGTCAGACTGGCGGTCCCGTCGGACTACGCAGTCATAAGTGTGAG
CGATCCCGTCGGATCTCTCAAATCATGTCTGTGTCAATGTCATAATGGACTAGTAATCCCGTCGGACTAC
ACAGTCATAAAAAAAGGTGGTGATCCCGAGGGACACCTATGTGGGTACAACTCTACTAGACAAAGTTAAC
AGAACACTCTATCCATAGCATGTAGCATAACATAACACCATAACATGACATGAGTATTAATCTTAACGTC
CTTAATCATGTGATTAACACATCATGCATCAACATAAACATCAACAGTCGTTAACAACATACTATCAGTC
ATTAACATAACATCAGTCGTCATCATCAATCAATATAATGAATTATGCATGTTAGCTACCATCAATGCAT
AATCATAATTACATGCGGTCTTTTAAATTCATTTCGAAAGTCTAGTAGAATAATATCTTACTTGGAGATT
TTAGCCAAACAAAGTACTCCTTAGCTGACAGTAAAAAAATCTCCAATTAAGTAGATCCTAATCATAAAAT
GAAAACTCAATATCTTAATTAATGAAATTAGGTTGAAACCAATTCAACCTTTTGGGAAAAATCAAAGATT
TAAATCTAAAAAATTAGCCAATTAAATCTTTAAAGAAACCCCAAAAAGATCCAAAATTAAATTAATAAAA
TATTAATTTAATTTCATTGGCTTACCAAGGTTACTCATATGGAGGTTGAAAAATCCTCTTATCCTTGATG
GAAAATAATTCATCACTTTAAATCCTCAACAACTAAAACTGTTGACGCGACAACAGCAGAGGGTTATCTT
AGAGAAAAAGATGAAGAACCTTTTATATATATATATATATATATATATAATACCAAATATATACATATATCT
TTATTTCCATTATATTTCCTCAATAAATGCCTTAAATGCACAAAATCTTAGGCATTTATAACCATTAATA
ATAGTAATAATAATAATACTTTATTATTGTTATTTTTCTCTCACCAAAATCTACAATAAACATATATATT
TATTTAAATCATTATTCTCTTCTAGATAAATATATATCTTTTCTCGTCCAATCAAATCAATCATCTCTCT
TCTCATGATTATCTTATTTTCCAAACAAATATAATTATATTCCATAATATAATTAACTACACTTTTCCAT
ATTATTAATTATATATATATATATATATATATATATATATATATATATACTACTAANNNNNNNNNNNNNN
NNNNNNNNNNNNNNNNNNNNNNNNNNNNNNNNNNNNNNNNNNNNNNNNNNNNNNNNNNNNNNNNNNNNNN
NNNNNNNNNNNNNNNNNNNNNNNNNNNNNNNNNNNNNNNNNNNNNNNNNNNNNNNNNNNNNNNNNNNNNN
NNNNNNNNNNNNNNNNNNNNNNNNNNNNNNNNNNNNNNNNNNNNNNNNNNNNNNNNNNNNNNNNNNNNNN
NNNNNNNNNNNNNNNNNNNNNNNNNNNNNNNNNNNNNNNNNNNNNNNNNNNNNNNNNNNNNNNNNNNNNN
NNNNNNNNNNNNNNNNNNNNNNNNNNNNNNNNNNNNNNNNNNNNNNNNNNNNNNNNNNNNNNNNNNNNNN
NNNNNNNNNNNNNNNNNNNNNNNNNNNNNNNNNNNNNNNNNNNNNNNNNNNNNNNNNNNNNNNNNNNNNN
NNNNNNNNNNNNNNNNNNNNNNNNNNNNNNNNNNNNNNNNNNNNNNNNNNNNNNNNNNNNNNNNNNNNNN
NNNNNNNNNNNNNNNNNNNNNNNNNNNNNNNNNNNNNNNNNNNNNNNNNNNNNNNNNNNNNNNNNNNNNN
NNNNNNNNNNNNNNNNNNNNNNNNNNNNNNNNNNNNNNNNNNNNNNNNNNNNNNNNNNNNNNNNNNNNNN
NNNNNNNNNNNNNNNNNNNNNNNNNNNNNNNNNNNNNNNNNNNNNNNNNNNNNNNNNNNNNNNNNNNNNN
NNNNNNNNNNNNNNNNNNNNNNNNNNNNNNNNNNNNNNNNNNNNNNNNNNNNNNNNNNNNNNNNNNNNNN
NNNNNNNNNNNNNNNNNNNNNNNNNNNNNNNNNNNNNNNNNNNNNNNNNNNNNNNNNNNNNNNNNNNNNN
NNNNNNNNNNNNNNNNNNNNNNNNNNNNNNNNNNNNNNNNNNNNNNNNNNNNNNNNNNNNNNNNNNNNNN
NNNNNNNNNNNNNNNNNNNNNNNNNNNNNNNNNNNNNNNNNNNNNNNNNNNNNNNNNNNNNNNNNNNNNN

NNNNNNNNNNNNNNNNNNNNNNNNNNNNNNNNNNNNNNNNNNNNNNNNNNNNNNNNNNNNNNNNNNNNNN
NNNNNNNNNNNNNNNNNNNNNNNNNNNNNNNNNNNNNNNNNNNNNNNNNNNNNNNNNNNNNNNNNNNNNN
NNNNNNNNNNNNNNNNNNNNNNNNNNNNNNNNNNNNNNNNNNNNNNNNNNNNNNNNNNNNNNNNNNNNNN
NNNNNNNNNNNNNNNNNNNNNNNNNNNNNNNNNNNNNNNNNNNNNNNNNNNNNNNNNNNNNNNNNNNNNN
NNNNNNNNNNNNNNNNNNNNNNNNNNNNNNNNNNNNNNNNNNNNNNNNNNNNNNNNNNNNNNNNNNNNNN
NNNNNNNNNNNNNNNNNNNNNNNNNNNNNNNNNNNNNNNNNNNNNNNNNNNNNNNNNNNNNNNNNNNNNN
NNNNNNNNNNNNNNNNNNNNNNNNNNNNNNNNNNNNNNNNNNNNNNNNNNNNNNNNNNNNNNNNNNNNNN
NNNNNNNNNNNNNNNNNNNNNNNNNNNNNNNNNNNNNNNTGAATAAAACAATATATGTTAATAAGACAACAT
TGTAATGGAAATCTATCTGTTTTTTTTTTTTTTTTTTCCTCTTTTACTAAAGAACTTCAAGTTTAGTCCTA
AAATGTCATTAATTACTTATTCTTAATTAATGCTGGAATCTCAAGGAATCAATTCATATTATGATCCTAC
TCCATGTTTTTGCAATTCACTCAACAAAATCAAGCTAGCACTTGCACCAAAAACATGGCAAACGTTTCTT
CTTCTCGTGTCGCCATCATTATAGTTATCTTTGTAGGTTCTCTTCTACATATGAATTGAATTATATATTT
TTATACGAGTTATTATTAAGAGATATTCATCTATATGCATAATGCTTAATTATTTATTTTAATCTTGTGG
CGGCTATGTAGTTTCAATGATTTTGGTACCGCAAGAAACCTCAGCTACAAAATGTCAAGACAGATTGTAT
GTTGGCGGGTGCACACAAAGTGATTGTGCGCATAAATGTAGTCAGCTACACCATGGTGGCACAGCTAAAT
GCATATCTACTGATCCTACGTTCACCAAGTATGCTTGTTATTGTTTCTATAATTGTCCTGCAAATTGATT
TTTCAGATGTCTCTCTCTTTTATTCATCGTTTTGGACACATTCCAAATAATGTTATCAAATAAAAATGGT
CTATCCAAAGTTATCTACTCCATATGATTGCGTATTTCTCTTAATTAATGTTATCTATACACAGCACATA
GAATGTTTTGTTGTGATCATGACTTCACAGATATTTATGTTGCTACAAAACTTTGACGTTGTGAACACTC
CAAATTTCTAATAAATGACTTATTTTCTGAAGTTAAACATTTGAAAATTTATTTCAAACCAACCCTAAAC
TTTATTGAATATGCTAAAAGAATAGTCCTTAATTGAAACTATATCTAAATAAAATGAAATATAAAGTTTC
TTTATTGATCCAAAGTAAAAAAAATAACAGTAACAAAATGGTGACAAAGTACATAGCGTTGATTTTGCTA
ACTATATCGATCAGTGTGATTGGCGTGATAAATGTCGTTAATTCTAAGACCACCGATCAAAAGATATTTA
TATAAACAATTATTTCATAAAAAAAGGTAGTGAAAATAATAAGATCGGAATCTATGAAAAGTTTCTTAGA
ATTTAAAATGCAAAAGTAAAGAAAGAATTGTTTTTACTTTTTGATATTAGAACACAAATTGTTAAAGATT
CTCTTAGTCAAAAGAATTTAGAATAAGAATAGTTAAATTCTAGTTTTATCATTGAATTCAAACAAGGCTT
AAGAATTTCATAAAATAATCCACAACCGATTAGACATTAATCATCCAATTGCTCTCTTTTAATGAACAAG
GGTTGGCTCTTATAATTAATCCATATACTAAATTCTTCGACAACATTTGTATCCATAGTGGTTATCTATA
TTATGATTGAAATGAAATGATGAGCAAGTTCAATTGGAAAAGTATTTGTGATTTAAAATAATCGCTTTGT
TTAATTAGAGTTATAATCCACGCCTAATTTTATTTGAGATAATAAATTATTAAGAAACTAAGGCATTATT
AAACAAGAGAAAGAATTAATTTATAGATAAAATCAATTCTACAGTTTGAACCTTATCACACATATTAGAA
AATGTGAGTGTGTAGTATTTGAGCATCTTGAAGTTGCTTGCCTTAAATAAATGTTGAGACATAATAATGT
CATATACATTTACTTAGATAAAAATTATGTATTCTAACCTCAACATTTATATCACGAAAGTGAATATACG
TACAACAAAGGCCTTGAGAGGTAACTCGTCTTAAATTAAAGTTAAAATATGAGATATTTATCGATTCAAA
TAAACTGTGAACCTTTTAATTCCAGTTACTCTATAGATAAACTTTTATGTTGCTTATTCTTGATATGCTT
GTACTAAGTTAAAGTTAGTTCAACATCATCCACATTCATCTATTTCCATGTCATTCATTGCATTCCATTC
CGAATCGATAGATTTTATCACCTAAATTTAGATCAAACATTTAATCGAATTATACACCAAGAGTTAATAC
TAGTGGCCAGTCTGAGTCGAATACAAGGAGCGTGTAAATTTCTCTAGCAAATTATTTTCAACCCAAGGTA
ACAAAACGAGGGGGTTGATTGAATTAAAAATAAATTGTGAAAAATAAATTAAAAGTGTAAATCCTTTTTA
GAAAACTTAGCTTGGGTTTTTTAGATCTCCCAAAACATTATATTTGAACGTTAAGTTCTCATTATTGAAT
TTAACAACCGATGCTCCTAAGCAATTTGAATAGAATAGCTAACCCAAATTGTCCTATGCATCAAATTAAT
GCAAATTAAACCGATGAAAGGTATACAATGTCCAATTTACGCTAATTGCATTACAAATAAGGGTTAAATT
AGGTTGAAAGCCTAATTTTCATGCACCTTATTAAACATTCATCAAAGTTCAATTGTAAGTCGATTGGAAA
AATACAAGATCTAATGGATTTTTACAAGCTAATCTAACATATTTCTTCTAATTGAATTACATACAACTTA
CTAAGAGAATTGTACATGATAAAAAAGCATTTGCCATCAAGTTCCCTTGCATCTAACTATATGAACTCAT
TAAAGACAAAATTTGACTATCAATGTATTACAGTCAATTCAAATCTCACGAATGCATGAATTAAAGGGAG
ATACTACAAACTGCAGCTAAGAAAAGAGTACCTTAACCTCTCAACAACATGTCGATTGATGCAGAGATGT
TAGGGAACTAAAATTAATAAAGACATCAGGAAATAGAGAGAAAACTAGTGGAATAAAGCAGGAATCATAA
ATGAAAAGACCTAGGCGACAAAAAATATTTTTGTAAAAACCACACTTGTTCTCCTTTTTGAACACAAATG
GTAAAGATAAATCAAGCAATATACATCTACGCTTCCTTACTTATAAGCATCATATCGACTCTATTTAAGG
CGAGTAACCTCTCAACGCCTAACAACCACATTTGTTTTCCTGTCGGGACACAAATGATTAGAGTTAAAAT
ACTAGAATGGAGTTTGGATAAACTCCATTTTGAGCAATTTTAGCTATGTCCTTGTAACTTATTCTCTAGA
ACAGTTGGTGGCTAACGCTGGTGAAGCGATTGAGTTTAACTTAAGCTAAACACCTGACACTTATTAACAA
AAGAATCCTTATCAAGTACAAAACATAAATTTAAACTACTTAAATCACATTAACAAGATGAATTGTAAAG
AAATGGAATAGAGAAATAGGATAAAAGTACATTATATTGATAATGTTTCCCTTTCGACCATGGAATGAAG
AACATTCATACAATACATCATAGAGTATACAAAAATGGAAAGAATAAAAATGTTTTTAGCCAAGGAATGC
AAGCTGCAGAATGCATTGGTTTACATTCCTTGGCTCTTTTACTATTGAAATTTATGTCCTAAAGCTCGTA
ATTTGTAGCTTAAATTATATTCTATTCAATAAAGTGGTTATTAGGGTCTTATTAGTGAAAATTGAATAAT
GTAATCTTGAATCCAATAAACTAAGATCTCGAGGCTATCTAGTATAAACTTAAACTTTATGTAGAGCCAT
AAATATGAATCAACTTCGAGTATATATCCTAAATGGTCTATCATATATGAATAAGGTTGGGTGCCTTATT

CTGGAGACACTATAGATATGATCCGCTTTGCAATTAGTACAAACGATATAATTCTGAATCATTCATATAC
AGATATGAAAGTGGAGACATTCTATGTAAAGAGTTTACATAAGACTAGAACCATGAAATAACCACATTTA
AGTTATAATGTTGTTGACTGTATAAAACTAACTATTTCAATTATGATTACTTAGGCAACTTACACTACAA
GAAATTATGTATTTTCCGAAGCATGTCAAGCACGTCGGGTTAAGGGACGTCAAGAGAAAAGACTTCTCCT
GACATCGTAGAACGCACTGTTGAATAAGAAATTTTGGAACTAATTACAAATTGCCATGTCATTTACTAAA
ATAATTGTATTTGGGATTAAGTAAAAATTGACATGTGTCCCAAATTAAATTTAAAGACAAATTGGACAAT
TCTAATGATGTCACATGTCTTTATTATGATAATTGGATCAAATTAATTATCGTGGTTTAATTAAATATTA
TTTATTTGGGCTAAAATTCAATTAAGCCAAAAAATAAGCTTAATTTAGCCCAAAATTAAATATGACTCAA
ATCCATGTGATTGAGCTCATGGGTATGGTCCATGGACCAGACCAGGCCCAAGCCCATAAAAGTCCATCAG
GGAACTCTATAAATAGAGGAGTTCTCTTCATTTGTGGGGTTGGAAATTGTTTACTCTAGAAGGTTTAGAG
ATAATTCTCTCAAGAGCTAGAAGACTTCCCAACTCCTGAAGTCAACCACCCTCGAAGATTGAAGCTCCTT
TGAAGATACAAGCTTTCTTCCAAGACTCAAACTTCAAGAATATCATGTGCTTCGCTTCCTGAAATCAAGC
GTCAGCATCCAATTGAGAGAGAATCAAACGATCAAATTCTAGAGATCGAACCACATCACATCAAATCAAT
ATAAATACAACATCAACACAAGTTCAACTCCACGAACCAAATTTCTCCGAAAATCGCGTGTAAACAAATT
GGCATGTCCAGTAGGACAACTCTACCCCTCATCTCTCTCTCGTCATCCAAATCTACAGATAAGCTAATGG
CATCAAGAAAAGTTGCATCTAAAGCTACTATTGCAAACGACTCTTACACTGGACTTGTCACCCAAAATCA
CTTAAAGAGAAGCATGCAAGAACAAGAACAAGGTTCTGTCCTCAAGAAGAAAAGTTTGGAACAACTAATA
GAATCTCCTGAAGGCGGGATCATCATTAGAGAGAATTCTTTGTTCAATAACCTACGTCTGCTTCTAATCT
ATCAGATAAAGAATCACACCTTGAAGTAGTGTCTGTCATGATTGTAGATGTGACAGCTGAGACAACGATG
GTAGAGATGGAGAGGAAAATAAATTTCCTAATGAAGATTGTTGAGAAGCAAGATCATGAAATCGCCGCCT
TAAAGATCAAATGAAGGCTTGTGAAACTTCTGAGTCGAGCAAAACTCCTACTGTCAAAGCTGATGATAAA
GGAAAAATTGTATTGCAGGAAAACTAGACACGGTCTATCTCCGTTGCCTCCCTGTCAGTCCAACAGCTAT
AGGATATGAACAAAAATTCCATTAGAGCTCAGTATGGAGGACCACTGCAAACTTCTTTTATGTACTCTAA
GCCATACACCAAGAGAATTGACAACTTGAGAAGCCGTTTGGGTACCAACCTCCAAAATTCCAGCAGTTCG
ATGGAAAGGGCAACCCAAAACAGCACATTTCCCACTTCGTCAAAACATGCGAAAATGCAGGATCAAGAGA
AGACCAACTAGTCAGGCAATTCTTTCAAAGCTTAAAAGAAAATGCTTTTAAGTGGTATACTGATCTAGTG
CAAGAAGTAATTAACAACTGGGAACAGCTGGAAACAGAGTTTCTCAATTGCTTCTATAGCACTAGGTGTG
TCATCAACATAATGGAGTTGACAAACAGCAAACCATGAAAAGGAGAGCTAGTCATCGACTACATAAACCG
GTGGAGAGCTCTAAGCCTTGTTTGCAAAGACAAGCTTACAGAACTGTCAGCAGTGGAGATGCGCATCCAA
GGTATGCACTGGGAACTCTATATTTTACAGGGAATAAAACCTCGCACGTTTGAAGAATTGGCAACTCGCG
CCCATGATATGGAGCGTATATCGCCAACAGAGGAGCAAAAGATTTCTTGCTTCAAAGAACGAGGAGTGAC
AAGAATGAAATCAATGATACTAAAAAGATTACAAATAGTGTCCTAAACGAGTCTATGGTTGTTCAAGAGA
CTCCATTGAAATCTTTCTCCAAAAGAAAACAAACAAAACATGAAATAAATCATGATGACAATGAAAAGCG
ACGCCCAACTCTTAGAGAAAGACAGAAAAAAGTTTTATCCCTTTTCTGACTCTGATGTTGCAGACATGTT
GGAGAAATTGATAAAGAAGCAACTTATTCTATTGCCATAATGTAAACAACCAAAGCAAGCAGAAAAAGTA
GATGATCCTAACTACTACAAATATCATCGGTTAATTAGTCACCCTATTGAAAAGTGCTTCGTGTTGAAGG
AAATAATTCTAAAGTTGGCTCGTGAAAATAAGATTGAGTTGGATATTGATGAAGTAGCTCAAATGAATCA
TGTTGCAGTCAAGATGACTTCAAGTGTTCTGCCATCAATGTTGCTTTATGATCAAAAGAAAAGTTCAATT
TGGGACTTTCGAACCTATACTTGTTCGATTCCAATAATAGATCATGACAACAAACTCTCAAAACAAAGAA
GAGTCTATTGAGGATGATGGCAAAGAATGGATAGTCGTGCCTCGTCAAAAAGGGAGACAAACAAATTCCA
TTTAAACAAAGTCGCACTTTCACCAAAAGTATGCAAAAGGAATCATCTCCCATATAAATAAAGGAAAAAG
GAGCAAGATCAAGATGTGGAAGCCTAAGCCTATCAAAGGAAAAGACGTGGACTTCCTCCAACTTCGATAG
TCAATAACTTTGGCTGAATTCCTCCCAAAAAACTTCCTTGAGGATCATCCTAAAGAAATATTAGAAGTCA
CTGCATGTCATACTGTCAGCATAGTAGAAGTCTATGTCCTTTTCCTCTTATCCTTTAAAAAAAAAAAAAA
AACAAAACAAAACACTTAAATATATAAAAATAATATATAATTAATTTTCATTTCCTTTTTGCTTTATTTT
CTTTTTCTTTTTCTTTTTCTTTCTTTTATAAAAAAAAAATCATCCAAAGAGAATCTTGGGTTTACATAGAA
AGTGCCAAGTACACTTTATCTTGACGCTTAAAAACTGTCAAGAAAAAAACTCTTTTATTATTAACATTGG
ATTACTTGACGCATCTAAAAGCGTTAAGTAAACAAATACTTGACAGGTAAAAAGCATCAAGGCCAATCTT
GTAGTAGTGATTTAAAGTTTGGAGGTTGCAAGATGTTTGCAAGATGCATAAAACAGTAGCTAAAAACTAC
CTAAACATGTACAAAAGCACCCTAAATCACATAAGCGCATGTATTTAATTCATATGATACTAAGCATTTG
AACTTAAGAAGCTACAAGATGCCTACGAGATGTTTGCGAGATGGAAAAAAGATAACAAAAAACTTGCTAA
TAAGTTTGAAACAACTCTAAATCAACTTGAAACAATTAAAAACTAGATTGGGTTTATAGATTTAAGATTT
AAATATTCCACCAAGAGAGAAGGACAAAAAAAAAAAAAAAAAGGGTTTTTTTCAAATTTTTTTAGGGATTT
GAAATTTGGGGGCTTAAAATTGTAGAGAGGGGCAGCCCCTTTGAGGGTTTTTTTTTTTTTTTTTATTCTT
CTTAAGTTATATTAAGGGATCTTTTTAAAAATATAAAAAAGCGACAAAGTATTTACACTGTATAGAACAC
TTCCGAAAATAGAAAAAGCCCAGAGGCCCACCTTGTAAAATACCAAAAATATCCTGTCAACTACACCGTC
AACAACGCACGCTTAATATATTTGTGATCGTTTAGATATTTGCGATCGTTTTGATTTGTCTATTGTTTGG
TACACGATCGTTTAGATATGACTACAATTTATTTTTCAATTCTATCGTTTAATTTTGTTACACGATCGTT
TAATTTGGTTACGTTTAAATTTGGTTATACGATCGTTTAGATTTAATCGTTTAGATTTGGATTTGGCTAT
TGTTTGGTACACGATCATTTAGATATGTGACTACAATTTATTTTTTCAATTCTATCCTTTAATTTTGTTA

CACGATTGTTTAATTTGGTTACATTTAAATTTGGTTACACGATCGTTTAAATTTGGGGACCCAAATCTAA
ACGATTTTTTTTCAAAACTTGGAACACGATCGTTTAGATTTGGCTAAACAATTTTTTAAAATTCTTTTAC
TACACGATCATTTATTTTTTTATACGATCGTTTAGATTTGTTTACACGATCATTTATATTTGTCTATACG
ATCGTTTAGATTTGGCTAAACGATTTTTTTAAATTCTTTTGCTACACGATCGTTTATTTTTTTATACAAT
CGTTTAGATTTGTCTACACGATCGTTTAGATTTGGCTAAATGATTTTTTTAATTCTTTTGGTATACGATT
GTTTTATTTGTCTGACGATGATTTTTTTTTACACGATCGTTTACATTTGGCTACTCCAATATGAATGATT
TTTTTTTCAAAAATTTTTATACACGATTTTTTAGATTTTTATTTTTTTGTACACGGTCGTTTAGATTTGG
TTATTCAAATTCAAAGAAACCACAACGAAAAAAAAAAAGAAAGAAAGACGATGGAAATATTGAACGATGT
AAAAAAGAATCAGAAAAGAAGAAAAACGAAGAAAATATTAAACAATGTAAAAAAGAATCAAAAAAGAAAA
AAAGGAATTGAAAGAAATCACAAAATCAGGAAAAAAAAAAGATGATGGAAAGAAATCGTAGCAAAGAAAA
TGAAGAAAAGAAGAAAGATGATGGAAAGATTTAACGACGTAAAAAAAGAATTGAAAAATGAGAAAGATTA
TGGAAAGAAATCACAAAAAAGAAATCAACAAAAAAAAAAAAAGAGCGAAAAGAAGAAAGACGATCAAAGA
AATCGCAGGAGGAAGAATATGAAAGAAATCGCAAGAGGAAGAAAACGATTTCATCTCGAGGAAGAGAAAA
AGATGGAAGGACAAACCTGAAATATTTAAAAAATGGTTAACTTTATGGACTTTATTACACGGGCTGTACA
TAGTTTGGTATTTTGTTACATATATGTAAGTTTCTCTTATATTAATTTAAGGCTGCTGGATAAAAAAAAA
AAAATTCTTATACTTGACATGAAAAGAAATATCAGTTAAAAGTGAATATTACTCTGGATATTTTTAACGT
GTCAAGTAAATATTAACTACACTTGATATTTATGGAACATCATGTAATAACTAACATAAAAAAGTTTACA
AATGTCCATGGAATCTTTGATATTTTAACAATTTGTTATACATTTTTTCTTGGAGCGTTAGTAAAAATAA
CAAAAAAATTTATGATAATAAGACCCATGTCACTACATTTCTAAATTGCAAAAATAAAAAATTTAAAAGA
GGATAACCTTTTGGTAATGCTTTGATAGCTATTTAATATATCTAATTACTTGTCATCTTTGTTATATTTG
CAATATACAAAAATTAGGTGTTATGGATTACATATTACTAAGATTGATAGAGTAAGAAGAAAAGAAGACT
ATGTCATATCAAAAAACTTTGAAGGTTATCAATTTGGGAACACCAGAAGAAGTGAAAGAAGTGTGAATTG
ACACTTTAGCTCCAGAGTAGATCAATCAGATCTGGTGATCCTGCTACATGAGTTCCAAGATATTTTGCAT
GGTCCCATTGAAATATGCCTGGTTTTGATATAGAGATTTGTGACATATCGATCACCATTTAAACTAGAAT
GTAAACCTCTACGACAAAAGCTTCGCAAGTTGAGCTAAAGGTATCAAACTACTATCGATTCATTTTGGTA
GCCCTAGACTATTTCAAGAAGTGGGTGGAGGTTGCTTCATACAAGAGTGTCACCAAGAAGGCCACTGTCA
ATGGACAAAACCTGAATAACAAATTAATGGAGGAGTTGTGTAGTTAGTTCAAGCTTAAACATTCTAATTC
CACTCCTTATTACCCTAAGATGAATGGGGTAGTAGAAGCATCAAACAAAAAATATAAAAAAAATTCTCGA
GAAAATATCTATCACTTATAAAGATTCACATGAAATGCTGCCTTTCACATTGCATGGGCATAGAATATCA
GTTAACACATAACAAGGGCAATCCCTTTTTCACTGGTGTATGGTTTTGAAGTAGTTTTACTTATTGAAGT
TGAGGTGACATCCCTTAGGGTAATCCAAGAGGTAGAACTAGATGAAGCAAAGTGGGCTCAAGTAAGAAAT
GAGCAATTGAATTTCATAGAATAAAAGAGACTGACTGCTCTATGTAAAGGACAGAGTTGTACAAAAATAA
AATTGCACGAGTATATGACAAGAAAGTTTGACACTGTCATTTTCAAAAAGGGAGTTTTAGTGTTTAAAAG
GATCCTACCTTTTCAAAAGGATCATAGAGGAAAATAAACTTCTAATTAAGAGGGATCGTATGTAGTAAAA
AGAGCTTTCTCAGGAGGAGCTTTGATATGGATGAAGATGACTTGCCTAACTCGTTAAGCGTGGAGTGACT
ATGTGAAGAAATACTATGCATAGAGTGACGATTAGCTCTTGATCCTAGCATTGTCTCGTCAAGTCACTAA
TATTTTAAAAATTTGAGCTTGTACTTGAGGTTTTGGAACTCATGAACGTTATTGGGCTATTACGCATGTT
TTATTTTTTTTTCTTTCTCGAGAAAAGTAGACAATGATTTAATTTTTTTATACATAATTAACATGTTCAT
GCATACATGGCTTTTCACATTGTCTGCATTGTCTGCATTTCTATAGGTGACATGATATATATTATTGATA
TGATAATTTAATATATGACATCTAGCTTGATTATTTATTTCAATTGTCATGAGGCACTTGTTTTATCCCT
GGCTTTAACACCATGTTGCTGCAATGACACTTGTTTTATCCCTGGCCTCCAGCTTATTTATTTCGTTCTC
ATGAGGTGCTTGTTTTATCCATAGCCCTTGACACCATGTTGCTGAGATGACGCTTGTTTTATCCTTGGCC
TCCAGCTTATTTATTCTTTTGTCATGAGGCGCTTGTTTTATCCCTGGCCCTTGACACCATGTCGCTACGA
TGACGCTTGTTTTATCACTGGCCCTTGACACCATGTTGCTACGAGGATGCTTGTTTTATCCCTGGCCTCC
ACCTTATTTATTTCGTTTGTCATGAGGCGCTTGTTTTATCCTTGGCCCTTGACACCATGTCGCTGTGATG
GCGCTTCTTTTATCCCTGTCCTCCAGCTTATTCATTCTTTTGTCATGAGGCGCTTGTTTTATCATTGGCG
CCTGAAACAGTTAATTTATTTAAAATATCTTAGTTTGATGAGTTAGTGGCTACACCTTCGTCCACCTTCT
AATTCTATAAGGATGCACGAAGGGGGACATGTTGTAGCCACCTAATTTTATCCTACAGTTTTTTATTCTT
TAAAATATTAATTGTAGCAATGATAAGATATTTTATTCATTTATTTTGTTCAAAAAAGAAAAAAGTAAAA
TCTTTTTGTAATAATATGAAATCTTATGTTTTTTTAATCTTTTTATTAAAATGGAAAATAAAGTCATTAA
TGAGAATATCTATTATTTAAAAAAATTTCAAATCATTTTTGACTTATCACTTTAGGAAAAAAGCAAATTA
ATTTATTTATACAAAATCTCTTTTGATTTATCATATTAGAAAAAGCAAAATAATTTATTTTATACAAAAT
TCTTTTAATACCATATTTGATTTGTCTTTATCACTTTAGGAAAAAACAAAAAATTTATTTTGGACAAAAT
CTTTCAATATCATATTTTTGCTATTTTAGAAGAAAGAAAATTAATAAAATTACATAATATCTAATTTGAT
TTTAAACTTATTAAATTTTCCTAATTTGTGGAACACCCCGAATCTATAAATAGGTCGTTTATCATTTGGA
AAATGGGGAGAAGGAATTGTTTTAGAGAAATTCTATGAAGAAAAAAGAAAGCATATTAGAAAGAATCTCT
ACGAGAGAAAACAATTTTTTGATTGCTTAGAGAATCTGTGTAAAAATTATTTTGATGATACATATTTCTC
TACAAAAGGTGGGTTATTACTTATTTTCGTTTTTTTATGTGTTATTTTCATTTTTATCTATTTACTTGTC
TCTTTTTTTCTTATCCTAAATCTAATCCCATAGAGGAAAAACCTTAGTTGAGGTTACATTAGGTAGGAAT
TTATTCCCAAGGATTCGCAATGTTTGTTCTTTTTTTAAAATTTGTTAAGATTGTATCAAATTCAAACAAA

ATAATTGATATATTTTAATTTTAAATTTAAAAAGAAGTAATGGAAAAAAAAAAAAAGATACTCTTTTTACA
TTTTCTTTTTTAAAATTTAGAAAGAAACATTATTAGATAGACAAATACTTTTCCGGTGGGGAGAGAGTTT
TATCAATTTCGGCCATCATCACTACTTTTCTTCCATCGACTCCTCAACTAATAATTTTGGTCTTCCCCAT
TATTATCTTCTTCTTCTTCTTCCATATCAACATCTTCTTATTTCTTCGTTCAGGTCAAATATTTCATCCC
TCACCATTTTTACATTTTCCATCTAATCATTTCAATACTTATAAACTGGATAATTTGTGTAGATTTTTTT
TTTTACATAGTTTTTACTTGGTGATCGGTATGTGTTCACTCCTACTTTTCTGTTAGTCAAAATCTTCAAA
ATTATTACAGTTCATTCCTTCTGGGTTTTTAGCTCTTCAAATATTTTTGTAGGTTTTGATCCTTTTCAAG
ATCAAGAACAAAGGATAATAATGGACATCCTTATTTCTGTCATTGCAAAAATTGCTGAATACGTTGGACG
CCAACTTGGTTATTTATTTTTCATTCGTTCCAACTTTCAAAAACTTAAGACTCAAGTAGAAAAGCTGAAG
ATTACAAAAGAATTTGTGAAACACAAGATCCATGCTGCAAGAAGAAATGCTGAAGACATAAAACCTGCCG
TTGAGGAATGGTTGAAAAAGGTTGATGACTTTGTTCGAGAATCTGACGAGTATTAGCCAACGAAGGTGGA
CATGGTAGACTCTGTTCCACCTATTTGGTCCAACGACACAAGTTAAGTAGAAAAGCAAGCAAAATGGTAG
ATGAGGTTCTTGAGATGAAAAATGAGGGGGAAAGTTTTGATACGGTATCCTATAAAAGTGTTATCCCATC
GGTTGATTGTTCACCGTCAAAAGTACCTGACTTTCTTGACTTTTGAGTCAATTGTGGAACAAATCATGGA
TGCATTCTCTGATGATAATATTCATAGGATTGGAGTGTACGAGATGGGGGGTGTTTGCAAAACAATGCTA
GTGAAGGAAATTTTAAGAAAAAATTGTGTAGAGTAAGAAGCCTTATGATGAGGTGGTACCATCCACGATCA
GCCAAACACCAGATTTTAAAAGTATTCAAGGACAACTAGCTGACAAGCTAGGTTTGAAATTCGAACAAGA
AACAATAGAAGGAAGGGCTCGTATTTTACGAAAGAGGTTGAAGATGGAGAGAAGTATCCTAGTTGTCTTG
GATGATGTCTAGGAGTATATTGATTTGGAAACAATAGGAATTCCAGGTGTTGAAGATCATACGGGATGCA
AGATCTTGCTTACCTCTACGAATAAACATTTGATCTCAAATCAAATGTGCACAAATAAAATTTTTGAGAT
AAAAGTTTTAGGAGAGGATGAGTCATGGAATTTATTTAAGGCAATGGCAGGTGAAATTGTGGAAGCAAGT
GATTTGAACCCTATAGCCATTCAAATTGTTAGAGAATGTGCATGTTTGCCTATTGCTATTACTACTGTTG
CTAAGGCATTACGAAATAAACCTTCTGACATTTGGATTGATGCCTTAGATCAACTTAAAAGTGTTGATGT
GGGTATGGCAAACATTGGACAAATGGACAAGAAAGTGTATTTGTCACTAAAATTGAGTTACGATGGCTTG
GGATATGAAGAGGTAAATTATTATTCTTGTTATGCAGCATGTTTCCAGAAGACTTTAGCATTAACATGGA
AGAGTTGCATGTTTATGCAATGCGCATATGGGTTTCTTACATGGTGTTGATACTGTGGTAAAAGGACGAC
GTAGGATTAAAAAATTGGTTGATGATCTTATATCTTCTTCTTTGCTTCAACAATATTCTGAGTATGGGTG
CAATTATGTGAAAATGCATGATATGGTTCGTGATGTAGCCCTATTAATTGCATCTAAGAATGATCACATA
CGTACATTGAGCTATGTGAAAAGATGGAATGAAGAAAGGGAAGAAGAGAGACTATCGAGTAATCATACTA
TAGTGTCCATTCATGGTTTAAATTATCCTCTTTCGAAGTTAATGTTACCCAAAGTTCAATTATTAAGGTT
AGATGGACAATTAAGATTAGAGGGACAATGGTTGAATAATAAATATGTGTCGGTGGTAGAAACATTTTTT
GAAGAAATGCAAGAGCTCAATGGTTTAGAATTAAAAATGGTGAAAATATCCTTATCGCCACCATCTCCTT
ACACCTTTGCAAACATTAGATTACTATGTTTACATGAGTGTGAATTAGGGAGCATTGATATGATTGGTGA
ACTAAAAAAGCTTGAAGTTCTTGATTTTAGTGAATCTAACATCACCCAAATTCCTTCGACCATGAGCCAA
TTGACCAAGCTGAAAGTGTTAAATTTATCTTCTTGTTACGCCCTTAAAATAATTCCACCAAATATTCTTT
CAAAGTTGACAAAACTGGAAGAATTAAGTCTGGAAACTTTTGATAGATGGGAAGGAGAAAAATGTGAAGG
AAGGAAAAATGCTAGCCTTTCTGAGCTCCGGTACTTACCACACCTTTATGCTTTAAAATTAACCATTCAA
GGCAAAGAGATTACGCCCAAAGACTTGTTTTCAAGAGAGTTAAATCTTGAAAATTCAACATTGTTATTGG
TTTTCGGATAGGAAGAATATATTATTCTGAAAATAAGACAAACTCCACAGGAATCAAGATGGAATCAGGA
AGGAGCTTGGATGATTGGATAAAAAATCTGTTAAAGAGAGATCAGAAAAAGTGCATTTGGAAGGATCAAT
TTGTTGGAAGGTTCTCCATTCAGAATTGGTAAATAAAAATGACTTTGTACATCTGAAGTATCTGGACCTT
TATGATAATTCAAAATTTCAACATTTTGTCCATGAAAATAAAAAGCCATTGCAAAAATGCTTATCCAAAT
TGGAGTACTTAATTCTAAGGAACTCGGAAAATTTGGAGAGCATAATTCATGGTTATCATGGCGAATCTAC
TTTCAACAATTTGAAGAATGTAATCATACGGAATTGCAATAAATTAAAAACTCTCTTTTTTAACTGCACG
TTGGATGACATTTTGAACCTAGAGGAAATTGTAATTAATTTTTGTGAGAAAATAGAAGTGACGATCATTG
TGAAGGAAAATGAGGAGACAACCAACCACATTGAGTTAACTCATTTAAAGTACTTGTATCTAATCAGTTT
ACCACAACTTCATAAATTTTGGAGAAATGTGGACAATTAACCGTGGAAACAAGCACAAGCAATACCATCC
GCATTGGTGAATCCCTTTTCAGTGAAGAGGTAATCGACACATTTTTCTATGTTTCTATATACATGTCTAA
ATTTAAGTACTTTTTTGTTCTTTTTATTGTTCCATCACAAGTTCCTTCTAACCTTTGTGGTTAGTAATGA
TATACAGGATCAAATTCTATCCTTATTTGTTTTAGAAGGCTCTTCTCTCAATGTAAACTACTGAAGTATA
TATAAATGCGTGCTAGGTGATGTTCCAGGTGATGTTCCATGAAAATTAAAAGAAGTTTATTAGATTTTTC
CTATCCAGGGAAGCTCATTTGAAGTAATCATGATTTTACACAATTTGTTATGGTAATTTTAAAACATTAT
ATACATTATATGTTGTTAATTGATGATATATGGTTAATTACGTTGTCTCAAAATTTGTAGATATCGCTTC
CTAATTTGGAGAAGTTAAAAATTTGTAGTGGGAAGAATTTGAAAGATGATATGGAGCAATAACTCATTCC
TAATTTCTTTTCCAAACTCAAGGAAGTAGACATTGATTCATGCAACGGTCTTCGAAAAGTATTGTTTTCT
TCAAATATGATGAGCATTCTTACTTGCCTTAAAATCTTAAGGGTAAAAGATTGTAAATTGTTGAAAGGGA
TATTTGAAGTGCGAGAGCCAATTAGTGTAGTTGAAGCGAGTTCTATCGTGCTCCAAAGTTTGAGTACGTT
GATTTTATACAATCTTCCAAACCTGAAGTACGTACGGAGCAAAAAATAATCCTTGTGAACTTCGGAGTTT
TGTAAATATGAAAAGTTTGTCGATTGATAAATGTTTGAGACTTAAAAGAGAATATTCAGTCAAAGCATAG
ATATCAAACAATTGATGAATGTTATTAAGAAGGAAGAGTCAGCAGATCATAATATGTTGGAATCAAAGCA

ATGGGAGACTTCTTCTTCTAAGGTACATATATATTCTACAACACAAGAAGTTTATTCAATTTAATTTTCA
GAAAATAAATTATTGTCATCAAGATCTTAGTTGAACAGAATTGGTTCGGTTGGTTGTATTTATATATATG
TGTGTGTCTTTGAGTTCTAAAGAAAACAAATTGCTGTCACCAATATGTCAGAATAATTTTTTAAATCACT
TTATTACTTCTTGTTTATTTTGTTCATCAACAGAGTCCTAGGAAAAATATTGTTATTCGAGGTAAATTAT
TTATGTGAAAAAAGAATATAAGCAACATAGACTTTTTAAGTTTAAGATATTTATCAATAGAAATAAATTA
ATTAAGTGGAAAATTATCTACTAATTATTACCATCAATTATTTAATTTTAGTTAGTCAGAGTTCAATTAA
TTTTATTTCAAACAACAATATATTTTTATAGGCAGTATACAAAAATGAAGGTAAGGAAAAGAAGACAAAA
AATAAAATGATATATTAATAAGAATTAATATTAGTAGATGTTTAAAATTATGATATGCATGTTAAACATA
AATTAGAATGATCTGATTAAAGTTAATCTGAATCAATAGAGTTTTTTAATTATATTAAATATAGTGAGTA
ATCAATTAATTGAATTTTTTTAGTACCAAATTAATTGAACTTATGAATTTTAACTAATTAAATATATTTA
AGATACCTTTTGCTGATTAATTACTAATTTTTGTGGATACATAAGTTATTGCATTCATTTAATAAATTTT
GATTAGGTTGGAGTTCTACGGTTGGAAGATGGTTCTAAGTTGTTTCGCAATCCTAAAAGTTTGAAGCTAT
GGTTATGTTGAGTTTAACTCAACTCATTTGCCAATGGAAATATTAGAAATCTTATACCGACTTAAAGACT
TTGAATTGGAAGGAGCATTTATTGAAGAAATTTTCCCCAGCAATATACTGATTTCAAGCTCTATGGATTT
ACAGAGTTTGACTCTATATAAACTACCCAAGCTTATGCTCACAAAATAATATTACCTCAGTTCTTCAACA
TTTGACTGATGTATCAATTTCTGAATGTGGTGGATTGAGTAGTTTAGTGTCATCATTGGTGTGTTTTACA
AAATTGAAACATCTTCATGTGAATAAATGTCATAGACTAACCCATTTGCTGAATCCTTTGGTGGCTACAA
CGCTTGTGCAACTTGAAGGTTTGACAGTAGAAGAATGCAAAAGGATGAGTAGTGTAATCGAGGAAGGATC
AACCGAAGAAGATGGAAATGATGAAATTGTTGTATTCAACAACCTACAACATTTAAGCATTACTTCTTGT
TCCAACATAATAAGCTTTTATCGTGGGAGATGCATCATTAAATTCCCATGTTTGAAACAAGTATACATTA
ACAGGTGTTCTGAAATGAAGGTATTTTCATTTGGATTAATCGTAAGCATGCCTCATTTGAAATATGAAGA
TGTTTATTTATTTAACAATCATGGTGATAAATGGTGTCATCCGAAACATTCCAAAGAGATGATGGTGAAA
GATGACATGAATGTGATCATTAGAGAAGCTTGGAATGACATCTATGATACAGACATTTCATATTTGTTTG
GAGAACAGGTTAGTGTATTTAATTACTTTTTCATGTCTAGCAATTTTAATTAATCTAATCTATATTATTT
GTGTGTTGGGTTATGAACTTTAATGAATTTATTTAATTAATGCAGGATTTGGAGGAAAACCAATATGAAC
ATTCTTCTTCTCCTTCGGACAATAATGTTGAGGAATAAAATAACAAGTTATATTGATATTCTATATCATT
TGATCCATAAATGAAGGTCAGAGCACTTAATTGAAACTAAATACGACAAAAATTTCGTGCTTTGAATTTT
GAATTTTTAGTGATAAACAAAACTGAAACCTATTACAGGAATGATCTTTAAAGACCCAGAGGCAAATGTG
TGTTACCAAAAAACAAAAACAAAATAATAATAAATAAATAAAAGGTTTCCCATATTTAATTTATACCCAA
AGCTAAGGTATGTCTGTGGATCCTTATCTCTAACCTTCAGAAATAACAATTTTTTTTTAAAAAAGTTAGT
TTTTCTTTGTTTCAAAAAATTCAGTGGGCTAGGTTGAATGCTTTTGATCACCAAGGGCGAGTGTGTCTGG
AATATGAAATCAAAATTTCTTGAAGCCTTGACCATTTTTGGATGGGTTATCTTAATGTTACATTATGATG
ATTATGGGAGGAGTTTGATGTTGCTTAGAGGTGGGGAACTTACTGAACAACAAGGCAATTCATCTTACGA
AGGTGACGTTTGTCATTGTCAATGTAAAAACTTTGGCTCCTAGTCATGGTAATTCTCTTTCATTTACACT
TAGCATCGTCACTATGTCTTGGCAAATTGATCTCTTGCCCAAATCTTCTCTACATGTCTTATCCTGTGTG
GTTTGTTGAGTTTTTTCTTTAAGATATGTTGAAATCATTTGATCAATCATTTAAAAAATATGGATATGAT
TCTTTGTCCTATCTACACAATCTGTTTATAGTTTATGCAGGGCCACTCAGGATTTTTGGTACTGCATTAT
GTTGGTGGGAGTTCAATGACATTGATTAATGAATCCGGTAATTGAATTTTAGCTACTACTTCAACCAATA
TTATTTACAAATGAAACACTTAATCATCATAACTGCTGCATTTTGGTATTTAGATTGTTTCATGATTAGA
ATATCTGTTTTAATTTGCTTTGCAGAGTATGGAGGAAAAAACCAATTATATCATAATCTTTCACACATGA
AGATATATAATCTTTTCAAACATATTATTTTCTAATTTAAAGAAATCTCCATAGAGTGTGAAATATGAAC
TGCCCTTTATGCAGAGACAAAGTGGAGATGATATTTATTGGCTGAAAGATAACAAATAAAGCTTCTTTGG
AACCAGATTCAAGCTGTGTTAGATACAACTTTAGTGAGAAGAGCATCAAATGCCCAAAGAGCAAAACAAA
ATATTTTCTTCAATAATGATTCTATTGCTACTCTTTGGATTGCCTGCTGAATGAAACGTAACAAACGCAC
TTTTACGGACGCATCAAATGGTGTTGCTAAGTTGTTGGGAATTAGATATAGTTTTGCAATGTTGACTTAG
CTGTTGGACCTTTTAGATCGAGACTTTTCAAACACACTACAGTCCTACTTCTATCCCTCCCTCAATTTGG
GTGCTTCTATGTAATATTCTGGACTTATCTCCATGTCCCTTCATGTTTATTTTGAATATAATATCAATGT
AGGAAGATATCTGACACTTGTTTGATAAAAATTTAGTTTAAAGAATACTCACTTCATCTCTGTACTTTTT
TTTTTTTTTATTTCGTTATCTACTTTTTATCGATATCTTAAAAAGTCAAGTAAAACTTTGAAAATTATAA
TTTCAAATAACAATTAGAAAATTCAATTTCGTCTCCAAAATTTGCAATTAGTCTCATTTATCTATTGAAT
TCTATCTCTCTTTAAAATTCTCTGCAATTTATTTCTTCCATAAGCTTCAATTTCAAAGCCCCCTTGGTTA
CTTAGAATCGTAAACAATTTAATTTGAAAGCTACCAGTACTAAAAGTTTTAGTTCGGTTAATTTATTAAT
TTGGTGTAGAATCAAGTGAAAATTTCCCAACGTTCATGTGCTTTTCTCCAAGTGTTTTGATTTTATATGT
ATGTAAGAAACCCATAAAGGACCCCATGATCAATTAACAAAAATCAATGTGTTTATATGTGAGGGTTGTT
GAACATTTATTTGGATGCACCATCAACATTTTGTTTTTGTTTTGTGCAGGGTTACTTGGGATTTGGAATC
AATGTAACTACAGATTTTTGTTCTTCCATTGGGGTGAGAGTTAAAATTTCTGCATTTTGGTAATTAGATT
GTTTCATGATTGGATGTGTCTATTTTTTGCTTTAATTTCTATGATATGAAGTAACAGTACAAATTATATC
ATCATCTTTAAAACATAAAGATAAAAAGTTTTAAATAGGTTAGTTTCTATTTCAATATATTGAGGTTGTA
TCATATCTTCTTTGACAAGTTAGTAGAGAAGTATTGGGGGGAGTGATGAGAAGAAAAATAATTTGCTCTT
GTGCATTGTCGTTGTGTTGGATCTTCGTTATAAAATGAAATATTGTTTAACATATTCTTGGAATGACCTT

TATTGACTATAAATTGCTAAGACTAAGAATGTGGAGCACGTATATTTCATGAATACAACAATTGTCTTCA
TGGTAGTTTTAGTGCTAGTTAGTACTAGTGTTTTGGATACTATTTTGAAGTTGGACGTAGAAGATGAGCT
AGAAATGAGAGGGGATATGGAGTATGGCTACACAAATATGTTTAATTTGTTTTACTGAGACAACGAATTT
TGATAGAGAGTTAAAAATTGGTGCATATACGTATTGTATATTTAATTCGTTTGGTTGAGAGACACCCTTT
GAAAAAAAAAATATATCAAAGGTCTATTAAATGTTTTTTCGAAGGTTCTTTGACCTTAAAATCTGGTCAA
TTTTCCAGTGAGCCTACTAGCTTTTACATTTAAATTATAGTCTATCGTTGTATATCACTTATAATTAAAG
TAAAATTTTATTATATTTGTAAATATGGTTTTGTTCATATACTATATATTAAAGGAAAATTATCAAAAGT
GGCAAATTTGACAAAATATTTACAAAATATAACAAAATTTTTTATTCTATCAATAATAGACATTAATAGA
CACTGATATGGTTCTATAAATGACATTGACGATAGATAATGATAGAACTTTATCGGTTTTTATCGTTAAT
AGAATCTAAAATTTTGTTATATTTCGTAAATATTTTAGTTTATTTTATTATATTTGAAAATTTTACTATA
TTAAAATTATTTAAATTAAAAAAAAAACGAAATGATTACTATAGAGTTAAACCTTTTTATAGATCGTATC
TAATCTAAACAAACAAAAATCGAAGATATTGAAAATTATAAATTTTAAGGAAGGAAGGAAATGATGAGTA
AGAGGCACCAAATGAGGATAGAAATGTAAGAAAAATAAAAAAGTGCTAAATAATCAAGGGGAGGAAATGT
ATATTCATGTGATTCAAAACTTAAACTTAGTACTTCACCCCAACAACAAAAGAACAATAGTGAATATGTG
GTAGGCCAATTAAATTAAAGAAATTGAGGAAAAGGGATTCTTTCAAATTTATTTAGAGGAATTGTTTCTT
TTTTCCTATAAAGTTATCTTTTATGACAAAATATATTTTTTTGATTATTTTAATAAAATATAATTTTTGG
CACAATCAATACCCATAATTTTTATTTCAATTTGGTATTGAGATATTTTTATGCAATATTAGTGAGATTT
AAGTTTTTTTTCTAAAAAAAATATTGATCCATATATTTTAAAAGTTTATACTTTTAACCTCGACTTTTCA
CTAAACATTAATGTCAACTAATCAATTAAGTTGTATATTTACTATTATTATATTTAAATTTAAAATTTTA
TTTCATATATTATTATTTTAAATTCATCAATGGGTATTAATGTCAAAATTGAAAATGATTATTAGTAAAG
AAAGAAAAAAAATTGTCAAAATTCGTAAAATCGATAAAATATTTATATTTTATAGAAAAATCAACTGCAAT
GAATTCTTTTTTCTTTTGGAGTGGTTTTATTTTTCTTTGAGGTGCGAATAATCTTTTTTTAAAAAAATAT
TTATAGGTGTGGTGCAGTCTTAAAACTATTTTGCATAAGTAACTATGGTTTTTCTTGTCTTATGCAATAA
AGTTTTCAGAAAAAACTGAATTGTTTCAAAATAAAATTGAAGATCAGAGAAGAAGGAGGAGAGAAGGATT
ACAAGTATTAGAAACCTTTTTTTAGTTATGTGAGCCCATTTCCCTTATTTGTTTTAATTAATTTAATTTG
ATTGAGATTTGGTTGTTAAGAACTTGACTTGGTTTAGATGTTTATTATGATATTTCACTATTTTGAGAAG
GTTTGGAGTACTATATGTTCAATTTTGTTCAAATTATTATATATGGTTGCGTGATGATTGATAAAGTTGG
AGTTCTTGTATGATTCTGCATTATCTATGGAAACAGGTGATATTGGCTAAATATGATCAAGATTCTTGTG
GTGGTATCCCTTCAAAAAGCAAATTTAGCAGAAGCAAGGCCCCCGACACTCAATAATTAAAATTGTTGAC
TGGTATAACTCCAACATTAGATGGAAAATAAGGAATGGTGAAAGAACATCCTTCTGGCATGATATCTGGC
ACATAAACAATCCTCTGAATATACATTATCCTATATTATATTCCCTATCAAAAGAAAAAGATTGTTGTAT
CAAGGATATGTGGAATAGTGAGACCTTTGGCTGGAATCTTCAACCAAGAAGACCTCTTCGAAGTTGGGAA
GTTGATCTATGGAATGAATTATTGTCCTCTTTTTTGGCCCCTACTGGAAATGGTGGTTGTGATTATCCTC
CTTGGAATCTTAATTCAAATGGTTCTTTCTCTGTGGCATATGTGAAAAAGGCCCTTTTCTCCTTGACTTT
TGTCAATGTAAATCAGATTGATCAACTGGGTTTCAAAAAACTGTGGAAGTCTTTTATCCCAAAAAAAAAT
GCAAATTTTTAATTTGGTCCATCATTCATGAGAGTATCAATACAGTTGATAAGCTGCAAAAAAAAGCTCC
TAAAAATGAATTTCGGGCCGAATTGGTGTGTCATGTGTAGAAGAAATGAGGAAAGTAGAGATCACCTCAT
CATAGATTGCCAATATGCTTCATATACCTGGGACAATTTTAATATTCTCTTTAGCTGGAAGCCTCCCTGT
CTTTCTGTTTCCTCTCTGTGCAAGTTGGTTTGCCAGACAAAACCAAAAGGAAAGAAAGAGATTATTTTTT
TCAACTTGGTCATTACAACCATTTGGTGCATTTGGCTTGAAAGAAATAACAGAATTTTTAACAGCAAGGA
TAAAGTTCCCTCTGATCTTTAGGAAGACATCAAGGCCCTCTCTAGACACTGGACCAGTAGAACAAACTTT
TTTAACGACTGTTCGGCTAGCTCTAATTGCTTTAAACCTTACTGCTTTTATTTGATTCTCTATTAATTAT
TGCTTTGTTGGGTGTGGGATTTTCTCTAGCCCTTTTTGTTATTACTCTGCGCTTCCTATACTTTTCTCCT
ATATTAATGAAGCTGAAATGATGAGGTTGCTAAGGGGGTGTCCACCTAGTGGAGATGTCTTGGTTCATCT
ACTGACCCAACCGTATCTTTTATAAAAAAAACTACTGAAGAATATAATTGCATGTTAGGTGATGTTCCAG
GTAAATTAAAAGAAGTTTATTAGATTTTTCCTATCCAAGGAAGCTTATTTGAAGTAATAATTTTACACAA
TTTGTTATGGTAATTTTACACAATTTCTCCCATCTGCCAAAATAATGATGTGTCCACGCAATATACAACT
CTTTGCCCACATGCATACATATTTTTGTCGGGAAAACAAAGATGTGTCCAGCGATAAAACTACGCCGACA
TATCATATCTTTGCCCACATTTTTTCTTACATTGCTAGAAAAAAGTGTCGGTAGAAACCAAAATTCTTAT
AGTGATATGATAACAATCAACTATGCAAATTGTTATATGATAGTCATCGTAAAATTGCCTATGATACTAA
TTGTTGTTTGAATATAACAATCAATGATATTGATTGTCTTTTGATTGCTCCTGGATTACTATCGACTACC
TTTTAAGCTAATATTGATTGATATTTGAATTAAAAATGCACAAGAACTAGAAAACCGGAACCAAACCCTA
AAAGTATATCTCACTTTCAATAAAATAATAATAAAAAAAGACAAAAATTAACCATAATGTTATTTATGTA
ATTTAATAAAAGAAAGGTAATTTAATAATTTTAGTTTAAAAATTAAAAAAACAAACCAACAAACAAAACA
TATCTCAAAAAATAAATAAAAACAAAAACAAAAGGCACACTCTTATTAAGCAAGTTGCCCCACCATTGAG
ATATATTTTCCCATAAGAAAAAATACGAAAAGGAAAGAAATCGAAGTGAAATGGAGTAGGAGAAGTTAAA
TTTGGAATTTTGAAAGATAGATTAAAGTGACTATTTAATTTTCTTTCTTTCACTCATTAATTTTACACAT
AAGTATGGTCTTAATATACCAAAATTACGATTAAATATCTCTGCCAAAAACTTATAGGATGTGTGTGAGG
GAGGAAAAGAGTTATGGGGGAAAAGAATTATAATAATCTTAGAATAAAATGATAATCCTAATGTTATGAT
AATATGTGTTTGGAGAAAATGTTGTTATTGGTAGTGTTATAATAATGTCTTTGGGAAACAAATATGAATG

GTAGTGTTATGATAGTATGTGTTTGGGGGAGGGATTATAATAGTAGTGCATGTTACAATAATATGTATTT
AGGGAAGAGTTATGATTGTAGTAATTTAAAAAACAATAATCGAATTTGAATTGGGTTATTTGGGTAGAGT
AATGTAGAGCTGTAAGAAAGAAAAAGAGAGAAGAAGATAGAGTTATTTGGAGATTATGATAACCCTAATA
TCCATTTATCATAACCCATGGGCCAAACACGGTTTGGCCTAATTTCCTAACCATTTTTTTTCCTTAAAAC
CCTAGCTACCCTAAACATACCAATAAGTTTTAAGTTGAAAACATAAGACGAAGACCGCACCCTTTGTGTT
AATGTATCAATTTCATACCAATTATATGTTCTATATAATGAAGAAGATGGATAACTTATCACATATTACT
TTGCTAATTAAGATGGGTAACGTATCACATATTACTTTTACTTTTATTCTTCCTTTGATAACTCCAAACA
ATACACTTTTGCAATGTTCAAGTATTCCATCAATTCTTTTTTTCTTTCAAAGGTGTGCAACGTGAGGACT
TTCCATCCCAAGTGGTCATTCTATTCTTCATTTCAATAATTTTGACATTGTTGAAATTCCTAGGAGCATA
AGCGAATTGACCCAGCTAAAAATGTTTAATTTATCTTCTTGTTATCAACTCTAAGACATTCCACCAAATG
CTCTTTCAAAGTTAAAGAAACTAGAGGAATTAAATCGGTAAACTTTTGATATATGGGAAAGAGAAAAGGG
GATATATGGAGGTTGCAGGGGAAATTCTAATCTTTTCGTGTTTCAGCAATTGTCTCATACCTTTTTGCTT
CAAATTTAACCATTCAAGATGAAGAGATTATGCCAAACCTCTTGTTTTCAAGAGAGTTTTAACTCAGAAA
AGGTCAACATTATTGCTATTGCTTGGATGATTGGATTAATAAACGTTATTAAGAAGGTGAGTAAGTGTTT
GTTGCAAAGGACCAATTTGTTTAAAGGATTTGTACTCACTAACTATTAGATATGGAAATGACTTATCACA
TTTGAAGCATCTCTTAATCCCTTCATGGAAGTTTAATATTTCAACATTTTATCGCTGTAAAGGACAAGCC
TTAAAATTGTTCTTTACTTTCCCCATATTAAATATCACATACATTCTTTTAACAACAATAGAAAAGAAAA
AGAAGGGTATAGGGAATTTAAACTTTAACTTTAATCAAAGGATCCAACTAAATTTAGCTACTTAAGATGG
GTCGCATATTACTTTTACTTTTCTTGCTTTGATGACTCAAAAGTTCCTCACTCCAAGTAACGAATTAAAA
TGTTCCTCACGGGGGTTTATGCCAATATATGTTCTATTCATACATAAATATAAACAACAAATAATTTTGG
TATTCTATCTCATTATTATCTTCTTCATTTTTCTCTGCTCCAGATCAGCATCATCTTATTTCTTCCATCA
GGTCACATATTTCTTCTCTATCATCACTTTATGCAATATATTTATGGTTACATTTTCCGTCACTTACACT
TTCCATCTCATTTCTGTACTTATAAGCTGATTTGTGTTGATTTTTTTCTTCTTCTTTTTTTGGATAGTTT
TTACTTGGTGATCTTTACGTGTTCACTTCTACTTTTCTGTTAAGGCAAAAGCTTCAAAATTATTATGGTT
CAGTCCCTGCGGGTTTTTATTTTTTTAAATATTTTTGTAGGTTTTAATTATTTTGAAGATCAAGAACAAG
GCATAATAATGGACATCCTTATTTCAGTCACCGCAAAAATTGCTGAATACACTGTTGAGCCTGTTTTACG
CCAACTTCGTTATGTATTTTTCATTCGTTCCAACTTTCGAGAACTTAAGACTCAAATAGAAAAGCTGAAG
ATTACAAGAGAATCTGTGCTACACAACATCCATTATGCAAGAAGAAATGCTGAAGACATAAAACCTGCCG
TTGAGGAATGGTTGAAAAAGGTTAATGACATTGTTGGAAAATCTGAGGAGATATTAGCCTATGAAGGTGG
ACATGGTAAACTGTGTTCCACCAATTTGGTCCAACGACACAAGTTAAGTAGAAAAGCAAGCAAAATGGCC
TATGAGGTTGGTGAGATGAACACCGAGGGGAAAAGTTTTGATACAGTATCCTACAAAATTGTTATCCCAT
CGGTTGGTTGTTCACCGACAAAAGTACCTGACTTTCTTGATTTTGACTCAAGAAAGTCAATTGTGAAACA
AATCATGGATGCACTCTCTGAAGATAATGTCCATAGGATCGGAGTGCACGGGATGGGGGGTGTTGGAAAA
ACAATGCTAGTGAATGAAATTTTAAGAAAAATTGGGGAGAGTAAGAAGCTTTTTGACGAGGTGGTAACAT
CTACGATCAGCCAAACATCAGATTTTAAAAGAATTCAAGGAGAACTAGCTGACAAGCTAGGTTTGAAATT
CGAACAAGAAACAATAAAAGGAAGGGCTTCTATTCTAGAAAAGAGGTTGAAGATGGAGAGAAGTATCCTA
GTCGTGTTGGATGATGTCTGGGAGAATATTGATTTGAAAGATATAGGAATTCCAAGTGTTGAAGATCATA
CGGGATGCAAGATCTTGTTTACCACTAGGAATAAAGATTTGATCTCAAATCAAATGTGCGCCAATAAAAT
TTTTGAGATAAAAGTTTTAGGAGAAGATGAGTCATGGAATTTATTTAAGACAATGGCAGGTGAAATTGTG
GAAGCAAGAGATTTGAAGCCTATAGCCATTCAAATTGTGAGAGAATGTGCAGGTTTGCCTATTGCTATTA
CTACTGTTGCTAAGGCATTACGAAATAAACCATCCGACATTTGGAATGATGCCTTAAATCAGCTTAAAAG
TGTTGATGTGGGTATTGCAAACATTGGAGAAATGGAAAGGAGAGTGTATTTGCCACTAAAACTGAGTTAT
GATTACTTGGGATATGAAGAGGTGAAGTTATTATTCTTGTTATGCAGCATGTTTCCAGAAGACTTTACCA
TTGACGAGGAAGAGTTGCATGTATATGCCATAGGCATGGGATTCTTACATGGTGTTAATACTGTGGAAAA
AGTACGATGTAGGATTAAAAAATTGGTTGAGGATCTTATATCTTCTTCTTTGCTTCAACAATATTCTGAG
TATGGATGCAATTATGTGAAAATGCATGATATGATTCGTGATGTAGCCCTATCAATTGCATCTAAGAATG
AACACGTACGTACATTGAGCTACGTGAAAAGATCGAATGAAGAATGGGAAGAAGAGAAACTATCGGGTAA
TCATACCGCAGTGTTCATTGATGGTTTACATTATCCTCTCCCGAAGTTAACGTTACCCAAAGTTCAATTA
TTAAGGTTAGTTGGACAATCTTGGGAACATAAGTTTGTGTCGGTGGTAGAAACTTTGTTTGAAGAAATGA
AAGAGCTCAAAGGTTTAGTATTAGAAAACGTAAATATATCATTGATGCAACGACCATTTGATCTTTACTC
CTTAGCAAACATCAGAGTATTACTTTTGCAAAGATGTCAATTATTAGGGAGCATAGATTGGATTGGTGAA
TTAAAAAAGCTTGAAATTCTTGATTTTAGTGAATCCAACATCACACAAATTCCTACAACCATGAGCCAAT
TGACACAGCTAAAAGTGTTGAATTTATCTTCTTGTGAAGAACTCGAGGTAATTCCACCAAATATTCTTTC
AAAGTTGACAAAATTGGAAGAATTAAATCTGGAAACTTTTGATAGATGGGAAGGAGAAGAATGGTATGAA
GGAAGGAAAAATGCTAGCCTTTCTGAACTCAAGTGCTTGCGACATCTTTATGCTTTAAATTTAACCATTC
AAGATGAAGAAATTATGCCAAAAGATTTGTTTTTAGCTGAGGAGTTGAAGCTTCAAAAATTCAACATTTG
TATTGGTTGCCAATCAATGTATACTTTTGGACCCCCGAACAGAATCAAAAACTTCATTGCAATGGAGATG
GAATCAGGAAGGTGCTTGGATGATTGGATAAAAAATTTGTTAAAGAGGTCGGACAATGTGTGTTTGAAAG
GATCAATTTGTTCAAAGGTTCTCCACTCAGAATTGGTAGGTACAAATGACTTCGTAAATTTGAAGTATCT
CTATCTTTATGATAATTCAAAATTTCAATATTTTATCAACGTTAGCAATACCATCAACATTGAAGAATCA

TTTTTTAGTGAAATGGTAAATTTCATCAACACATTTTTCTATGTTTCTATATACACATCAAAATTTATAA
ATCACCTGTTTGTACATTTTTATTGTTCCTTCCACAAGTTCCTTCTAACATTTGTTGTTAGTAATGATAT
TAGGGATAAGATTCTATTCTTCTTTGTTTTAGAAGGCTCTTCTCTTGGCGTAGAGAGACTCTTTCTTGTT
TCATACATTAATGATAATATAAAGTATATAAATGCATGTCAGGTGATATTCCAAGAAAATTTAAAGAAGT
TTATTAGATATTTCCTATCTAAGGAAGCACATTTGAAGTAATCATTTTACACAATTTGTTATGGTAATTT
TAAAACACTATATAAATTATATGTTGTTAGTTAGCATCAGTTGTGATGATATGGTTAATTACGTTGTCAC
AAACTGAACTTTTAAGGACGTAAATTTGTAGGTATCACTTCCTAATTTGGAGAAGTTGGAAATTGTGAAT
GCAGAGAGTTTGAAGATGATATGGAGCAATAACGTGCCAATTCTTAATTCCTTTTCCAAACTCGAGGAAA
TAAAAATTTGTTCATGCAACAATCTTCAAAAAGTATTATTTCCTTCAAATATGATGGACATTCTTACATG
CCTTAAAGTCTTAGATATTAGAGATTGTGAATTGTTGGAAGGGATATTTGAAGTGCAAGAGCCAATTAGT
GTTGTTGAGAGCAATAATGTACCCATTCTTAATTCCTTTTCCAAACTCGAGGAAATAAGAATTTGTTCAT
GCAACAATCTTCAAAAAGTATTATTTCCTTCAAATATGATGGGCATTCTTCCATGCCTTGAAGTCTTAGA
TATTAGAGGTTGTGAATTGTTGGAAGGGATATTTGAAGTGCAAGAGCCAATTAGTGTTGTTGAGAGCAAT
AATGTACCCATTCTTAATTCCTTTTCCAAACTCGAGGAAATAAGAATTTGGTCATGCAACAATCTTCAAA
AAGTATTATTTCCTTCAAATATGATGGACATTCTTACATGCCTTAAAGTCTTGGATATTAGAGATTGTGA
ATTGTTGGAAGGGATATTTGAAGTGCAAGAGCCAATNNNNNNNNNNNNNNNNNNNNNNNNNNNNNNNNNN
NNNNNNNNNNNNNNNNNNNNNNNNNNNNNNNNNNNNNNNNNNNNNNNNNNNNNNNNNNNNNNNNNNNNNN
NNNNNNNNNNNNNNNNNNNNNNNNNNNNNNNNNNNNNNNNNNNNNNNNNNNNNNNNNNNNNNNNNNNNNN
NNNNNNNNNNNNNNNNNNNNNNNNNNNNNNNNNNNNNNNNNNNNNNNNNNNNNNNNNNNNNNNNNNNNNN
NNNNNNNNNNNNNNNNNNNNNNNNNNNNNNNNNNNNNNNNNNNNNNNNNNNNNNNNNNNNNNNNNNNNNN
NNNNNNNNNNNNNNNNNNNNNNNNNNNNNNNNNNNNNNNNNNNNNNNNNNNNNNNNNNNNNNNNNNNNNN
NNNNNNNNNNNNNNNNNNNNNNNNNNNNNNNNNNNNNNNNNNNNNNNNNNNNNNNNNNNNNNNNNNNNNN
NNNNNNNNNNNNNNNNNNNNNNNNNNNNNNNNNNNNNNNNNNNNNNNNNNNNNNNNNNNNNNNNNNNNNN
NNNNNNNNNNNNNNNNNNNNNNNNNNNNNNNNNNNNNNNNNNNNNNNNNNNNNNNNNNNNNNNNNNNNNN
NNNNNNNNNNNNNNNNNNNNNNNNNNNNNNNNNNNNNNNNNNNNNNNNNNNNNNNNNNNNNNNNNNNNNN
NNNNNNNNNNNNNNNNNNNNNNNNNNNNNNNNNNNNNNNNNNNNNNNNNNNNNNNNNNNNNNNNNNNNNN
NNNNNNNNNNNNNNNNNNNNNNNNNNNNNNNNNNNNNNNNNNNNNNNNNNNNNNNNNNNNNNNNNNNNNN
NNNNNNNNNNNNNNNNNNNNNNNNNNNNNNNNNNNNNNNNNNNNNNNNNNNNNNNNNNNNNNNNNNNNNN
NNNNNNNNNNNNNNNNNNNNNNNNNNNNNNNNNNNNNNNNNNNNNNNNNNNNNNNNNNNNNNNNNNNNNN
NNNNNNNNNNNNNNNNNNNNNNNNNNNNNNNNNNNNNNNNNNNNNNNNNNNNNNNNNNNNNNNNNNNNNN
NNNNNNNNNNNNNNNNNNNNNNNNNNNNNNNNNNNNNNNNNNNNNNNNNNNNNNNNNNNNNNNNNNNNNN
NNNNNNNNNNNNNNNNNNNNNNNNNNNNNNNNNNNNNNNNNNNNNNNNNNNNNNNNNNNNNNNNNNNNNN
NNNNNNNNNNNNNNNNNNNNNNNNNNNNNNNNNNNNNNNNNNNNNNNNNNNNNNNNNNNNNNNNNNNNNN
NNNNNNNNNNNNNNNNNNNNNNNNNNNNNNNNNNNNNNNNNNNNNNNNNNNNNNNNNNNNNNNNNNNNNN
NNNNNNNNNNNNNNNNNNNNNNNNNNNNNNNNNNNNNNNNNNNNNNNNNNNNNNNNNNNNNNNNNNNNNN
NNNNNNNNNNNNNNNNNNNNNNNNNNNNNNNNNNNNNNNNNNNNNNNNNNNNNNNNNNNNNNNNNNNNNN
NNNNNNNNNNNNNNNNNNNNNNNNNNNNNNNNNNNNNNNNNNNNNNNNNNNNNNNNNNNNNNNNNNNNNN
NNNNNNNNNNNNNNNNNNNNNNNNNNNNNNNNNNNNNNNNNNNNNNNNNNNNNNNNNNNNNNNNNNNNNN
NNNNNNNNNNNNNNNNNNNNNNNNNNNNNNNNNNNNNNNNNNNNNNNNNNNNNNNNNNNNNNNNNNNNNN
NNNNNNNNNNNNNNNNNNNNNNNNNNNNNNNNNNNNNNNNNNNNNNNNNNNNNNNNNNNNNNNNNNNNNN
NNNNNNNNNNNNNNNNNNNNNNNNNNNNNNNNNNNNNNNNNNNNNNNNNNNNNNNNNNNNNNNNNNNNNN
NNNNNNNNNNNNNNNNNNNNNNNNNNNNNNNNNNNNNNNNNNNNNNNNNNNNNNNNNNNNNNNNNNNNNN
NNNNNNNNNNNNNNNNNNNNNNNNNNNNNNNNNNNNNNNNNNNNNNNNNNNNNNNNNNNNNNNNNNNNNN
NNNNNNNNNNNNNNNNNNNNNNNNNNNNNNNNNNNNNNNNNNNNNNNNNNNNNNNNNNNNNNNNNNNNNN
NNNNNNNNNNNNNNNNNNNNNNNNNNNNNNNNNNNNNNNNNNNNNNNNNNNNNNNNNNNNNNNNNNNNNN
NNNNNNNNNNNNNNNNNNNNNNNNNNNNNNNNNNNNNNNNNNNNNNNNNNNNNNNNNNNNNNNNNNNNNN
NNNNNNNNNNNNNNNNNNNNNNNNNNNNNNNNNNNNNNNNNNNNNNNNNNNNNNNNNNNNNNNNNNNNNN
NNNNNNNNNNNNNNNNNNNNNNNNNNNNNNNNNNNNNNNNNNNNNNNNNNNNNNNNNNNNNNNNNNNNNN
NNNNNNNNNNNNNNNNNNNNNNNNNNNNNNNNNNNNNNNNNNNNNNNNNNNNNNNNNNNNNNNNNNNNNN
NNNNNNNNNNNNNNNNNNNNNNNNNNNNNNNNNNNNNNNNNNNNNNNNNNNNNNNNNNNNNNNNNNNNNN
NNNNNNNNNNNNNNNNNNNNNNNNNNNNNNNNNNNNNNNNNNNNNNNNNNNNNNNNNNNNNNNNNNNNNN
NNNNNNNNNNNNNNNNNNNNNNNNNNNNNNNNNNNNNNNNNNNNNNTTAATATTCTATTTATTTTAATTT
TTATTTTTTTAATTTTGTAAAAAGTTGAATTTTTCTCTCTCTTACTTTACTCTTTTCTCCTTTCTTCTCT
ACCAAACCCTACACTTTACTCTTTTCTCCTTTCTTCTCTACCAAACCCTACCAAGGTTTTCTCTCAACAT
TTCTTCCCTTTCTTGCTTTCTCAATCTCATCACTTTCTCCCCTCTCTTCTCTGATCAGTCGCCAACGCCT
GTGAACTCGTCGCCGTGCTATCATGGGAGGCAGCTTCTTCCGTTGCGGCATCGCGCTCGTGCCTCACGTG
GAACACTCACCCTCCTCACCACCGGCACGTTACCGGCTCGGGATGTCCGGTAAATGAATATAAAAAATAT
TAAATATTATATTTCTTGCGTATTGTGTGGTTCTGGATTGAGAGAGTGGAAGAATTCAGTAATGTTGCGT
ATTGTGTGGTTCTGGATTGATAGAGTTTGGAGGAAAACCAATATGAAAATTCTTCTTCTTCGAACAATAA
TGTTGAGAAAGAATAAAGAATTATATGGATATTGTTGTACACTACTTAATATATCATTTCATCCACAAGG
AAAAGGTCAGACTCTTGAAATCCTCCATTTAATTTTATGAGAGAATATCATCCAATGTCAGATTGAAAAG

TCTCGATAGATTTGTTAAATTAATTTTTGATACAAGTCATAGAACGTTGATGTTTGATGATGGACTTTGC
CAGAACCTGGTATGTGGAAGTTGAATACAAATGTTACATGAGACGATCCTATCTTGGCAAATATGTAGGC
TAGGTTGGATGTTTCGTGTGATCAATTTAGGCAAGTGTCTCTAGTCGGTTTGAAAGCAATTTCTGATAGT
ATAAAATAAAGATTCTTGAAACCATGACCATTTTTGGAAGGGTTTTCTAATAATGTTATATTATGATATG
ATGATGGGGCATATGATTGTGCAGTTCGATTGGTCAGAGGTGGTGAACTTATTGAACAACAAGGCAATTG
AGGATCTTATGGAGATGGCACTTGTCATGGTAAGGTCTCTTCATTTACACTCATCATCATTACATGTCTA
GGTATGATAATGGAGAATTAATAGACAGAGAGTAATAACCAGAGATATAGAAAAGAATTTATAATGAACA
CAAATGCAATGATAAACAAACAAGTAAACATGCAAACAAATCAAATAGAAACAGAGTTAATGGGAAAACT
TAATTTATAAACATTTCAAACAGCTGCTAAGAAACATCCTATTGAAAAACTTCCCTGTAACAAGACTTTT
CAAATTTCAAAATGAATGGAAACAAATGCTAAACATTGTATTTTCTGTTAAATTAATGGACCCGTTAACT
AAAGGGTTCTTGAACAGAAAAAAAGACAGAAAACAAATGCTAGAATCATATAATTGAACAGACTACAGAG
AAAGGTCAGAAACAAAAGCCCACTTTAAACTAAATAAGTATAACAGAATTCTTCCAAAATCTGGTAACTT
ATATCTATATCCCCCTGTTTTCAAGAGAAGAGAAAGGAAAAAGAAAAGCTAAAAATGAATCAAACATATC
TAAACAATTGTAGTGGGTGCAGACGGTAATGCAATAATAAGAATTTTAGTCTAATAAAACAAAAATTGTT
TGGTAAACCGTTTGTTTTATGATTATCTTGATTTGTTTGGAAGTTGAGGTTATCAAATTAGATTGGTTTC
TTTTTGTTTTATGATACATATCCACCATGAAGACTTAGGTATTTGATTTTTTGTGTTTTGAGACAAAAAT
ATGAAGGGAAATTTGCAGCTATTAATTAATTGTTTTGTCGGGGAAATTTAAAACAAATTGGTAGGCTATT
AGGAAAATAATTGCCAGAAATAGAACAAAATGAGGGGACCAGAGGGTTTTGGTTTAAAAAGACATATTTT
TCGAACTACCTTGTAACTCAATGTCCCTTTCTAATAATAGTTACTAAGACATGTTAATATATAAATTTTA
AGTTCAATCCCAATATCTAAAATAAACTTGTCATCAACTATATTATGCTCTCGTTCCTCTTTGATGGGTG
ATATAATGGACAAAGACTTGTCGAAGGTCTGGCCATCAAAATAATTTTTCGATCCTACTTAAGCTAGATA
TAGTGGGTAGTACAAGGTCGAACCACATGGAAAACAGTTATTTTTCATTAACAATACTTAACGTTTCTAA
GTAAAACATAGTCTAAAGGAGGTTTGTCTGTCGTTTGCAAGAAAGTAAAAGTGCATCTACAAATATTAAA
GCAAGAGATTGAAATTAAATATTGATAATGCAATGAGAGAAATATTGAGAATTAGTGTTATATGATTGAG
AGGAGTCTAGCCATGATCGTACATGGAGCATTCATAACACCCTTTAGTATAATAATATATCTGATCCCAT
CAAATTAATTAGAAGTAACTACAAATTTAACTTCTGTAATATCAATTCAATTTGATGTCTCAATCAACTG
TTGAATCTTTTTTTTTCTTTGGGTATAAAACCAACAAGTTAAAATAGATATAACAATGAATTTAATTCAC
ATATAATAAATTCATCCAATATAACGTTCTTTCACCTTTTTCTCTCTTTCACAAAACTGTAATAATAATA
TCTACCACAAAAGGTAAACAATTAATATGATTCTTCAAGAAGGTTGTTTATTTGGTCAAATTTTCATGAA
AGTATTAATACAGTGTATGTTTTGCAAAGGAAGCTGCCAAATAACCTACTCAATCGTAGCAGATGCGTTC
TTTGCAAGTCGGCTGTCGAAGACTTGAACCATATTTTCACAACCTGTGCCAATTCGCAAATAGCCTCTGG
GTCAAACTGCACGACAAAATTGGTGGAAACTTTGATACAAACAATATCAAAGCCCTCTGTTGGTCTCTTG
GCTCGCTGAAACAATCAAACAGAAAGAACATCATTCTCTCGATGTAGGTGTGGATCTTCTTTTGTCCATT
TGGGTGGAAAGAAACAATAGGATTTTCATAGACATAGAAAGAAGCTTAGTCACATTTGGGAAGATATCGA
AACTTTGATTGGACCATGGTCGAGTAGAAACAAAATGTTCAAAGACTACAATCCAACATCAGTCTTTAAA
CTTTAGAGCTTTGTTAGATTAATGTTTGTTGTATATGGCTTCCCTGTAGCCAAAGAAATATACATTGTAA
CAATGGTTTGATGAAATGATAATGAAGTGGTATGGTGTGTTTCAATTGCAAAAAATATGATTCTTCAAGA
AAGGCCGAGAGGATAAGATTGTGATCGTGCCATGCGCTTGGGTTGTGATTTAATTACAAATATATTCATA
TAGTATTTGGAGCAAAACAATCCTAATTAATTAATATAATGCGTTATTTTATTTTACTGAATAAGTTAAA
TTTAACCACTAGAAAATTTTTACTAAGTGAACATTTTCATAGCACTTTCATAATCCCCACTTCATTAAAA
TGAATCAATAAAGTTCAAGGAAAGTGTAGAATGATGAATCTAAAAGAAACAAAAAACAAGGTATATAAGT
TTAAAGCAACGGTGGTAGCACTGTAATGATCCCGAGGGAGCACACATTTTTTCATAAATTATTTTATTAT
CATCAATAGAAGAATTTGAATTTCTTATGTTTTAATTGATAATATAATTTCATAATCAGCTATAAGTGGA
TTTTTCAAATGATTGGATCCATATATATATCAACTTCGATCAAATTTGTAAAGGTATAACTTTAAATCAC
TAAGAACAAAAGGATTGATGGTGGGCCTTATGCTATTATACACTTTGAGTACCCTCTCGGCCTCAACAAG
ATTCCCAAGTTCAATAATTGAAACTTTGAAAAATGTGTGCAATCACATGTATTCAATTTCTAGGTCGGTA
TCACATATTTTCATGTTTCCATACGTTTAATTTCCATATAGAAGTGAATCCATAATTTTATTATATTGCG
AAAAAATCTACCAAACATGGAAATTAGCTATGAAAATATTAGTAATATAAATAACAGATATGTTAGACTT
ATTTGAAAAAAATTAAAAAAAATATTCATTTATGGGTTCAAATTATTTTTAAATTTTTAGTTTTTATATA
TTATTAAAAATATATTCAAATTGATATTTTATTGATATTATATCATCAAAATTTTTGTAAAATTAAGATC
TTCAATATAACGCCCTAGGCCCAGGATTTGGAATTCGGATCCCAACATTCTCTTGCATCTTCTATGATTT
GGCAACATCATCATTACTTGTCTTAAATTCTTATCAGAGTGAAAGTTCTCTTTATAAACCAATACGAGTC
TTTTCAGCATGCTTTGTCCTCACTCACAGCATCCCTATCCCTATCACTCATAATTAAAGTAAAATTTTAT
TATATTTGTAAATATTTTTTGGTTTAGTTTTCCGTATATTAAAACCTACTACTAAAAAAACGAAATGATT
AAAAAAGAAGGGAAGGTATTAAAAATTATAAATTTTAAAAAAGGAAAAGAAAAAAAAAAAAAAAAAAAAA
AAAGAATGATGAGGGAGAGGCACCAAGTGAGGAAATACTACTCTTTGAATACATAACCTAATGGTTAAGA
AAAAAAAATCTCATATCAAATTCAAAGTGTCATGCTATTATTACTTAATATTTTATATGGTAAAGGCATA
GTTTTCTTTTTTTCTAAAAAAAAAAAACTTATTGGTGCCGAGCATGAGGTAATGCTAGACGTTTGGTAATT
TCTCCTCCGACTAGACTAAAAGATCCTATTGAAGCGGCTAATCCCATGCATATTGTCTGTATATGTGGTT
GGGCGGAAGAAATTTATATTCACGTGATTCAAAAGTTAGTGCTTCATCTCCCAACAAAAGGACAAAAGTG

AATGATTTTTTTTAAAGAAAAGTTATGTAGATGAATGATTTTTTTTATAAGGTTATCTTTCATTAGTAAA
CGTACTGCCTTGAGTTTAAGAGGGGACAGAGGAGACATGAATCAATTGATATCACATATGACGAGAATAA
TTTTCAAGATCGAACATAAAAGTATGGTTAAAAAAGACTTAAAAGAATTAAAAGTTAACACCTATACTAA
GGTATTAACATTAGTTTGTAATTTGTAGGATATATTAAATTTTAAATTGTTAACTCATTTTTGTAATTAA
TGTTTAATTTGATCATTGTCCTTCCAATTAATTGTAACATAGTTTTCTGCCCAATTTATCTCTTTTGCTT
TCGTTTTTCTTTAGATACCCTACTCGGCTACTCTTCAACTTTTCCTCATTTCATTTTCAGTTGGCCATAT
GAAGATTTTGTTTCAACCTTATCTTCATTCTCCCACTTCTTTTTTCCTTTTATTACTTTATATATTCGTT
TTTAAAATTTTTCATTACAATTTGATAATAAACGGAAACATTTGTCGTTTGATTGTTACTATCTGATTTT
TATTTGATATGGACTGTTTTCTATAACAACATGATATACTAATTTTCAAGGATATTGATTGCATGTCGTC
TAATTAATATCTGATTTGTATATTTGATATTTGATTGTTATTTTATACTATATTTGTTATTTAATAGTTG
TTTGATTGCTATTTATTATTGATTGTTCTAATAATATCAATCGTTGTTTGATTAAGATCTAATAATATTG
GTTGTCAACTATACAAATTGTTATATGATAATGATTGTAAAATTGTTGATGATATTGATTGTTGTTTGAA
TATAACAATCAATGATAATGAATATTGTTGTTTGATTGTTCTCAAATTACTCTCTACTGCTTTAAACTAA
TATTAATTAATATTTAAAAAAGCACAAGAACAAAACCCAAAAACTATCTCACTTCCAATAAAATAAAAAA
CACTTTGTTAGTTGTAAATTAAATACAAAATGACAGTTTGTTCAGTTTGTTCAATTTTAGTTTAAAAGTT
AAAAAATAAGAAAAACAAACAAAACATATCTGAAAAATAAAAAAACCAAACAAAACATATCTAAAAAGAT
ATACACTTTTCTCAAAATTTGAGAAATTGTTCTAAAATTCCTCTGTTTTTCTCTTCTTCTTCTAATTCTT
CTCTCTATTTTTTTGTTTCTTCTTCCAACTCTTCCCTTTTCCTCGCAGATCCAAGAAAAAGAACCATAGA
ATAGTTTCAAGATTTTTTCAGGGGAAGGGGGGTTGGATCATTTCAAAGTGAGTTTTGTTCTATTTTTGTT
TATGTTGATGATTATTAGATGACGAAGTTGTTTGGGCAACCTATTAATTAATTGAAGGGAAATTTGCAGC
TATTAATTAATTGTTTTGTTGGGGAAATTTAAAACAAATTGATAGGCTATTAGGAAAATAATTGCCAAAA
ATAGAACAAGTTGGTGTTCTATTTTTGATGTCTCAATCAACTGTTGAATCTTTTTTTTTTTTTTTGACAT
AAAACTCACTACAAGAAATATCAGTTACGATAGCATTTTCGATAGCATTTTCGAAATGCTGTTGTAGCCG
ATGTTATTGAAAGTCTATGACTTTTCATAATGTTTTTTTGCCGCTATGCAAAACGCTATAATATGTCCCC
TACAATAGCACAAATATGATGCTATAAATGTTTTTTATAACGTGAGAAAAAACACTATCAAAAAGCGCTA
TAAAATGTTTCAATAGCGTTTTCAATAACATTGAAAAAAATGCTATAAAAGACATTTTATAGCGTTTTTT
TCGTTCGAAAAGCGTTATCAAAGTATTTCACTATCTATTTTAAAACCATTTGAGCTCATTAACATTTCAT
AATCACATTATTAATAGAAGAAATAACAAAGAACTATACATATAATATCTCATTGAATAATATACGTTAT
CTTCTATGCATTACAATTTTGTTTATAAAGGAAAAAAGTACTTTGAGAACAAACCTAAACAAAAAACTAC
CTTGTTCTATAAAACTCAATACATCACAAGAAAACTGATAAACTGAAGCATTTCTTCATACAACAAATTT
CCCATGAAACCATTTCTCACAGATATCACAACAAATCTAAAATTCATCTGAAGCATAGTTCTCAACGCAT
GCCCTAGTTAGGTGTCAACGTCTTAACCTCTTCCCTGCGACTACCTTGCATACATTTCTTGCATTTCAGC
TTCCCTCTGAAATTATTATCATAACATTAGCAGAGCAACAAACATTATCATAACTAGTATCGGTAAACTT
TGTTAATAATACACGGCTTATACATTTCTAAAGGCAACACAAAAACTAATGAATCGGATAGTAAACTGTT
CAGATAAGCTTATAAAACCAAAAAACTGTCAACATTTGAAGAGTAAAATGCAAACAATTTGCTGTTTACT
ATATGTAATTTACTAACCATGATGGTATTTTGAATATTAATACACTTCATATTCATAGTGTCAACATCAG
TAAGTCTCTAATCTTGCAATGTTGCATTTAAGAGAGTGACACATGAAGAACTTTTATTGACAATTATATT
TAAACTAAAAAGAACATATCAAATGTTAGAAGATAAATATGAAGAAAACAATAATGCCATACAAACCTCT
GCAACGACTAAAAACATCTTTGCCAGTGCTTGTTTCATGGTGGTTGGTAACTTTTTAGTAAGCCCAAAAT
CAAGCAAAATTGGACAATGAGGAGGTTCCTTGCTGAGGAGAAAATTTCCTACACAAAGTACCAAAAGGGG
GCACTAAAACAGCTATTGCATTGGGATGGTTACATCCTTAACATTTCTATAAGCCAGTCGATACATCTAC
TAACATTAAAAAGAATTTAAAACAGTTTTAAAAGGTTAATCTATGAAATAGTTCTTANNNNNNNNNNNNN
NNNNNNNNNNNNNNNNNNNNNNNNNNNNNNNNNNNNNNNNNNNNNNNNNNNNNNNNNNNNNNNNNNNNNN
NNNNNNNNNNNNNNNNNNNNNNNNNNNNNNNNNNNNNNNNNNNNNNNNNNNNNNNNNNNNNNNNNNNNNN
NNNNNNNNNNNNNNNNNNNNNNNNNNNNNNNNNNNNNNNNNNNNNNNNNNNNNNNNNNNNNNNNNNNNNN
NNNNNNNNNNNNNNNNNNNNNNNNNNNNNNNNNNNNNNNNNNNNNNNNNNNNNNNNNNNNNNNNNNNNN
NNNNNNNNNNNNNNNNNNNNNNNNNNNNNNNNNNNNNNNNNNNNNNNNNNNNNNNNNNNNNNNNNNNNNNN
NNNNNNNNNNNNNNNNNNNNNNNNNNNNNNNNNNNNNNNNNNNNNNNNNNNNNNNNNNNNNNNNNNNNNN
NNNNNNNNNNNNNNNNNNNNNNNNNNNNNNNNNNNNNNNNNNNNNNNNNNNNNNNNNNNNNNNNNNNNNN
NNNNNNNNNNNNNNNNNNNNNNNNNNNNNNNNNNNNNTGTCAGCACAATATGAAACATTACCGTAGTTTA
CATCAAACACAAGTTGATATTTAGAGAGAGAAAAAAGAATAGATGAGAAAAGCCAAATAAAGAGAGAGAG
AGAGAAAAAAAGAGACAAATTCTACTTTACAGACCACTTCAATTGGGCAGCACTGACATTTCTTTTTCTT
TGTCTTCATATCATTTAACATAGAACGAATAATAAGCCACTGCACAAATTGCACCTCAAACCTCACAAAT
CTCACACATCAAAGCATGAACTAACATAAAGATAGATGCCAAAAACATGTTGTAAGACTTCTGCTGGTGC
AACAGCAAGACAATATCAATGTATATGTCATCAAATCATTGTATGTTTCTGCTGGTGCTAATGGTTAATT
CAAATAGATTATTTCAGAAAAAGGCAGAGACAAAAAGTATAGTTATGAATATAATTAAATTTGGGTAGGA
CATTTTGTGTTCTAATTCTAATTTGTTATGACAATCTAAAGACACAAAGCTAATTTACATGTATGTGTAC
TAGTTGCAACTGTAGCAATCCTTGATAAAGCCCCACAAGGGCAGAAAAAATTCTTAAGACTTGGAAACAT
GAAACAATGGTATTAGAACTGAAACATTGCATAATACCAATTGAATAAGCATAAATTATGCCTAATGTA
TTAGTCCTAAATTACATCTTCTTCTTGATTTTCTGTGATTGAGAGAAGGGAAGACATTAGTCATTTATTA

AGAAGCAACGACTCAATAGAAAACCTAAATACTAGTACAACAATAACAAATAAAACCAAGCATATATTTG
ATATCAGCAAAAAAATAGAGATGTAAGGTAAAGAAAAAGATTGATGCAAAAACTTTTGCTAATTCTATAA
CACGTTGATTGCACAAGCACACTTAGTTTGAGTTCATAACTTTTACAGTATAACTATAATCTCCAGACCG
CTTTTTCATTTTCTATGGCAATAAACAAATAATATGGCACACAAGCACAGAAGATAACAAAAATACTTAT
TATCTCTATCTCAAATATATGCACAAACCAATGGATACCAATAATAAGATAGACAATAATATAGAAAATT
GTAAGCAAAACCAAATACTTTATACCTCTGCGTAACAGCAAACAACCTTCGTTTGCACAGAAAACTAATA
TTATGGATGCTATAAGCTTGGTCTTTTCCTTCATTGCCAATCTATTTGAAAGCAAAACCAATTTGCTCCA
GTTTCCTTCACTAAAAAATGTTAATACAAGCTACTTGGAAGATTTTTTCGAATACGACACAAAATAAAAC
AATCTATCATTCATATATTCAGTACCAAGAGATGAAATTCCCACATCATTTCACAATAATTTATAAAATC
ATAACACAATAAATTTTCAAACATCAAGCTATAAATTTCTAGTAAAATTTACTTTATTGAACCCAGATTC
TATTCCCAACAACAATCATTTCTATTTGCAATTACTAAAATAGATGGATCTATATGAAAAAGAATAGAGC
AACTTAAAAAGAAAAAGAATAGAGCAACTTAAAAAGAAAAAAAAACTGAGACAGATTGAGAGGGAAAAATT
ACAATTTCATAAAGGGATTCAACGAAATGAGATCTGACAAGATCGCTCTTCACGCCTTGAAGTCCAAATC
GATAGAAGAATGGTGACGAATGGTGAGATTTGCATATGAAACTTTCAGTTCGACGGACGACGACTAACGA
GCGGCGTACGAGTAATGAACAAACGATGAAATGGTGAAGAGAGGCTTTTGTGGTCAAGGAATTGAGAAGG
AATTTTCTATTAGAGAGAAAAGGAAGACACTCACGGCTTCATGAAAACCAGACAATGCATCCGAAAGGAA
AAGAAAAAAGAGAAAAGAAAGGTAAAATAAAAAAAAAAAGCGTAAAGAAGGTTACCCATCCAATGTGAGT
AACTGGTGACTAGTTCTACGAGCAGGGAGCCATTCGGCAAGGAGTGGGGCGAGTCAGCGGCGATGTTGGA
TGGTAGTGGCGTCGTGGAAGGTGTCTGGGAGAATAGGAAAATTGAAGGAGGGGTCTGTGAGAATCGGAAA
ATCGGTGGAAGTCTGAAAAAAAAATGAAAAGGAGGCATTGGGGCAGGGAAGGGTAACACGCAAAACCTAAA
AAAAAAATGGGTTTTTTTTCGCGCTTTCCATATTTCGTTTTCCGTCTTTTTTTTCTTTTATTTTCTATAA
CTGAATATTAAAAAAGCTATGGTTGGATGCTATTAAAAGTCACATTTGTTGTAGTGAATGAATTGAATTT
ACATATTTATTAAATTCATCCAATATAATGTTCTTTCACCTTTTTCTCTCTTTCACAAAACTGTAATAAT
AATATATACAACAAATTAAAAGGTAAACCATTAATATGATTCTTCAAGAAGGTTGTTTATTTGGTCAATT
TTTCATGAAAGTATCAATACAGTGGATGTTTTGACAAAGAAGCTGCCAAATACCTACCTCAATCCTAGCA
GATGCGTTCTTTGCAGGTCGGTTGCCGAAGACTTGAACCATATTTTCACAACCATGCCAATTCGCAAGTA
GCCTCTGGGTCAAACTGCATGACCAAATTGGTGGAAACTTTTGATACAAACAGTATCAAAGTCCTCTGTT
TATCTCTTGGCTCGCTGAAACAATCAAACAGAAAGAACATCATTTCTCTCGATGCTGGTGCGGCTCTTCT
TTGGTCCATTTGGGTGGAAAGAAACAATAGGAATTTTAGGGCCATAGAAAGAAGCTTAGTCAACATTTGG
GAAGATATCAGAACTTTGATTAGATCATGGTTAAGTAGAAACAAAATGTTCAAAGATTACAATCCAACAT
CAATATCTTTAAACTTTAGAACTTTGTTAGATTAATATTTGTTGTATGGCTTCCCTCCAGCCAAAGAAAT
ATACATTGTAACAATGGTTTGATGAAATGATAATGAAGTGGTATGGTGTTAAAATCACCTTATCACTCTG
TGTTTCAATTTCAAAAAATATGATTCTTCAACAAAGGCCTGAGATGATAAGATTGTGATCGTGACATGTT
TTTGGGTTGTGATTTAATTACAAATCTATTCGTATAGAATTTGGAGAAAAACCATCCTAACTAATTAAAT
ATAATGCGTTATTTTATTTTACTGAATAAATTAAATTGGACTCCTAAATTTTTTTTACTAAGGGAACATT
TCCATAGTGCTTTCATAATCCCTATTTTATTAAGATGAATCAATAAAGTTCATGGAAAGTGTAGAATGAT
GAATCCAAAAGAAACAAAAAAACAAGGTCGATAAGTTTAAAACAACGGGGGTAGCACTCTAATGATCACA
AGGGAGTACACATTTTCCCATAAATTATTTTATTATCATCCACATGATTAGAATTCGAATTTCTTATCTT
TTAATTGATAATATAATTTCATAATTAGTTAAAAGTGGATTTTTCAAATGATTGGATCCATATATTAATG
TCAATCAAAGTTTTAATGGTAAAGATTAAAATTTTAAAATGTTTAATTAGTACAAAATTGAGAAATCACT
AAGAACAATTTCTCTTCTATGTATGAGATTTGAACCAACCTTTTTGTTTGGAGTAAATGTGCAAGATAAT
TTTTTATCCATGAAAATGAGATTTTATAAACATCATGCCAAATATAGAAATTAGATTTAAAGTATTAAAA
AGTATGTATAGTAACAATTTTTTTAAAAAATTGCAAATGTAATAAAATTTATTAGAATACTTACAAATAA
TTGAAGTGTGAACCATATTGCAAATGCTGATTATATCACTAATAGATCGTAAGAGTCTATAAAAAAAAAT
AATAAAAGCTAGTTTTATTTCGAACAAACTTTGTTATTTTTATAATTTTTTAAAATATTGTTATATATTT
AATTATATCCCTAAAATTATTACCAATTATAATTATTGAGATATTACGTTTTGTATTTAAGGTGTATAGT
GAAATTGATACCATTGGTCAAAAGGATTGATGGTGGGCCCTTCTGCTATTATACACTTTGAGTACCCTCT
CCTCGACAAGATTCCCAAGTTCAATAATTTAAACTTTGAAAAATGTGTGCAATCACATGTATTCAATTTC
TATGTCGGTATCACATATTTTCATGTTTCCATACGTTTAATTTCCATATAGAAGTGAATCCACAGTATTA
TTATATTTGTGAAAAATTTACCAAACATGGAAATTAGCTATAAAAATATCATTAATATAAATAATAGACA
TGTTAGACTTATTTGAAAAAAATTAAAAAAATATTCATTTATAGATTTAAATTTATTTTAAAATTTTTTG
TTTTTATATTTTATTTTAAAAATGTATATTAAAATTGATATTTTATCGATATTATATCATCAAAATTTTT
GTAAAATTAAGATCTTCAATATAACGCCACAGGCCCAGGATTTGGAATTTGGATCCTCGACATTCTCCTA
CATTCCCTGTGATCTGACAACATCATCCTTACTTGTCTTAAATTCTTACTAGAGTGAAAGTTCTCTCCAC
AAACTAACACGCGAGTCCTTTCAGCATCCTTACTTTATATATTCGTTTTTAACATTTTTCATTACAATTT
GATAATAAACGAAAATATTTGTCGTTTGATTGTTACTATCTGATTTTTATTTGATATCGACTGTTTTCTA
TTACAATATGATATACTAATTTTCAAGAATATTGATTGCATGTCGTCTAATTAATATCTGATTTGTATAT
TTGATATTTGATTGTTATTTTATACTATATTTGTTATTTAATAGTTGTTTGATTGCTATTTATTATTGAT
TGTTCTAATAATATCAATCGTTGTTTGATTAAGATCTAATAATATTGGTTGTCAACTATACAAATTGTTA
TATTATATGATTGTAAAATTGTTGATGATATTGATTGTTGTTTGAATATAACAATCAATGATATTGAATA

TTGTAGTTTGATTGCTCTCAAATTACTATCTACTTCTTTAAACTAATATTAATTGATATTTGAAAAAGCA
CAAAAACGAAATCCAAAAGCTATCTCACTTCCAATAAAATAAAAAACACTTTGTTAGTTGTAAATTAAAT
ACAAAATGACAGTTTGTTCAATTTTAGTTTAAAAGTTAAAAAATAAGAAAAACAAACAAAACATGTCTGA
AAAATAAAAAAACCTATTAAAATCTAATAGTACGCCAAGGGACTTTTGCACTATTAAAATTTTCTTCCGA
AGGGATGTCATCTCGAAATCTTTTGTTGTACTTTGCATCAATTAGCCTTTTCCATAAAGAACCAGACTCA
AAGTGGTACCTCCAAAGCCATTTACAAAAGAGAGCTTGATTAATATCATTTACATTTGTGATGCCTAACC
CTCCCAAATCCTTAGGTGTGGTGCCAATATATCCCAATTAACCAAATGGGAGTATGCTCATTTGAAGTAT
CTTTTAAGAAAAAATTTCTCCGATTGCATCTAAATTTCTTTATAGACATAGTTTGGAGCTTTGAATAATA
TTGAGAGTTGGTAATAAGTTGACAACCTACCTCCCTTGGAAATTTGACGATAGGGAGCCTACCACCCTTG
AAAGTTTGACTGTATTTCCAACCATATAATTTCTTGTGGATATTTTCAATGGTCTAGTTCCAAAATGATT
TTGAGTTGGGCTTGCCCCCTAATGGGACTCCTAAGTAATTGATAGGAAGGTACTGGGTTGATAAACCACA
CATTAGACGCAAATCGAATTCCTAATTCCATCGTCTAAGATGAGTTTCTGATCATGACTTATACAATTTA
TATAGGCACGCATAAAAGACGAGCAAAAAACGACGAAGTTACCTTAAAAAAAATGGCCCAATCAACCAAT
TATGATGACTTCGCCCTTACCTGCCTCTGCTAGAGCCTCCCCTTCAATTAAAATAATCGAATATATTTTA
ATTTTAAATTAAACAAAAATGGAAAAGATGAGTGGGAGACCCACCAAATGAGAAGTAAACGAAGATAAAT
AGAAATAATATGTAAGAAAGAAAGGGCTAAATAAGAGATAGAAATGATAATAACTTTTACTGCCATATTT
TATAAATAAGACTACACATTATTAAACCATCCTAATAAATTAAATAGAACATTTTTGGCATCTACTATAA
AGCCTTATTTTATTGCAAATACTTTTTAACACATTGCAAGTCTGATTGGTAAACTTAGGATAAGAATTAA
AATAATGTCGTTGTGAAAGAACTTGATATATTTAAAAAGAAGATGCTTTCCGATACATACAAAAGTTAAA
ACTTTTGTATGTTTGTTAACAATTTGTATAAACGAGGACAAAACCGATTTTATATTCTCAATTTTGAAAA
AACACACAACTGTAATCATGAAAAACTTTGGATGTTAATTAATGAAAACTTTTAGAAATAATGATGTAGA
CTAACAGCTACGATGGATGACTATTTAGAATTGCTATCTAAATGGAAAAATATATGGGAAAGGTATTTGG
TAATTGAAAGTCATGAATTTAATAAACAATAACAATTAGATTAATTGGTGAAGAGTTAAAAATGAGAATG
GATATTTAGTTTTAAATACCCTCATGAACATAAATGGCCATCATATGCAGTCTATTATTCCCAGTTTCAA
TAATTGAAATTTTGAAAAATGTGGCCATCACATGCATTCTATATTCTATGCATATATTCATCTGTTATTA
TTTGTCCAATATGGTATATTATATTCATCCACACAAACGTTATTTTATTGCCCACTAAAGGGTTATATTT
AATTTCTTTCATACATCATTGCCCACTAGAGTTTAAAATATAAAGAATAACAAATAATTTTGGCCATCAC
TAATTTTCTTCCATCGACTCCTCAACTAATAACTTTGGTCTTCTCATTCTTATCTTCTTTTTCATTTTTC
TCTGCAATATCTCTTCTCCATATCAAGATCATCTTGTTTCTTCAATCAGGTCAAATATTTCATCCCTCCC
CACATATTCAATATATTTCTCTTTACATTTTCCATCTCATTTTTATACTCATAAACAGAATAATTTGTGT
AGATTTTTTTTTTTTCGGATAGCTTTTACTTGGTGATCTGTATGTGTTCACTTCTACATTTTTTTCTTAGG
CAAAAGCTTTAAAGTAATTTTGGTTCATTCCTTGTGGGTTTTTAGCTCTTAAAGTATTTTTTTTTTTTTT
TAGGTTTTGATCCTTTTCAAGATCAAGATTTAATGGAAATCTTTATTTACATTTTCCATCTGATCATTTC
TATACTTATAAATTGGATAATTTGTTCAGATTTTTTTTAGATAGTTTTTACTTGGTGATCTGTATGTGTT
CATTCCTACTTTTCTGTTAGGCAAAATCTTCAAAATTATTATGGTTCATTCCTCGTGGGTTTTTAGCTCT
TCAAGTATTTTTGTAGGTTTTGATCCTTTTCAAGCTCAAGAACAAAAGATAATAATGGACATCCTTATTT
CAGTCACTGCAAAAATTGCTGAATACACTGTTAAGCCTGTTGGACGCCAACTTGGTTATGTATTTTTCAT
TCATTCTAACTTTCAAAAACTTAAGACTCAAGTAGAAAAGCTGAAGATTACAAGAGAGTCTGTGCAACAC
AAGATCCATAGTGCAAGAAGAAATGCTGAAGACATAAAACCTGCCGTTGAGGAATGGTTGAAAAAGGTCG
ATGACTTTGTTCGAGAATCTGACGAGATATTAGCCAATGAAGGTGGACATGGTAGATTCTGTTCCAGCAA
TTTGATCCAACGACACAAGTTAAGTAGAAAAGCAAGCCAAAAGGCATATGAGGTTCTTGAGATGAAAAAT
GAGGGGGAAAGTTTTGATACAGTATCCAATAAAAATGTTATCCCATTGGTTGATTGTTCACTGCCAAAAG
TACCTGACTTTCTTGACTTTGACTCAAGACAGTCGATTGTGAAACAAATCATGGATGCACTCTCTGATGA
TAATGTCCATAGGATTGGAGTGTATGGGATGGGGGGTGTTGGCAAAACAATGCTAGTGAAGGATATTTTA
AGAAAAATTGTGGAGAGTAAGAAGCCTTTTGATGAGGTGGTATTATCCACGGTCAGCCAAACACCAGATT
TTAGAAGTATCCAAGGACAACTAGCTGACAAGCTAGGTTTGAAATTCGAACAAGAAACAATAGAAGGAAG
GGCTACTATTCTACGAAAGAGGTTAAAGATGGAGAGAAGTATCCTAGTTGTGTTGGATGATGTTTGGGAG
TATATTGATTTGGAAACAATAGGAATTCCAAGTGTTGAAGATCATACGGGGTGCAAGATCTTGTTTACCA
CTAGGATTAAACATTTGATCTCAAATCAAATGTGCGCCAATAAAATTTTTGAGATAAAAGTTTTAGGAAA
AGATGAGTCATGGAATTTATTTAAGGCAATGGCAGGTGACATTGTTGATGCAAGTGATTTGAAGCCTATA
GCCATTCGAATTGTGAGACAATGTGCAGGTTTGCCTATTGCTATTACTACTGTTGCTAAGGCATTACGAA
ATAAACCTTCTGACATTTGGAATGATGCCTTAAATCAGCTTAAAAGTGTTGATGTGGGTATGGCAAACGT
TGGAGAAATGGAAAAGAAAGTGTATTTGTCACTAAAACTGAGTTATGATTGCTTGGGATATGAAGAGGTG
AAGTTATTATTCTTGTTATGCAGCATGTTTCCAGAAGACTTTCCCATTGACGTGCAAGAGTTGCATGTAT
ATGCCATGGGCATGGGATTCTTACATGGTGTTGATACTGTGGTAAAAGGACGATGTAGGATTAAAAAATT
GGTTGATGATCTTATATCTTCTTCTTTGCTTCAACAATATTCTGAGTATGGGTGCAATTATGTGAAAATG
CATGATATGGTTCGTGATGTAGCCCTATTAATTGCATCTAAGAATGAACACGTACGTACATTGAGCTATG
TGAAAAGATCGAATGAAGAATGGGAAGAAGATAAACTATTGGGTAATCATACCGCAGTGTTCATTGATGG
TTTACATTATCCTCTCCCGAAGTTAACGTTACCCAAAGTTCAATTATTAAGGTTAGTTGCACAATATTGT
TGGGAACATAATAAGCGTGTGTCGGTGGTAGAAACTTTTTTTGAAGAAATGAAAGAGCTCAAAGGTTTAG

TAGTAGAAAACGTAAATATATCATTGATGCAACGACCATCTGATCTTTACTCCTTAGCAAACATCAGAGT
ATTACGTTTGCAAAGATGTCAATTATTAGGGAGCATAGATTGGATTGGTGAATTAAAAAAGCTTGAAATT
CTTGATTTTAGTGAATCTAACATCACACAAATTCCTACAACCATGAGCCAATTGACACAGCTGAAAGTGT
TGAATTTATCTTCTTGTGAAGAACTCGAGGTAATTCCACCAAATATTCTTTCAAAGTTGACAAAAATTGGA
AGAATTAAATCTGGAAACTTTTGATAGATGGGAAGGAGAAGAATGGTATGAAGGAAGGAAAAATGCTAGC
CTTTCTGAACTCAAGTGCTTGCGACATCTTTATGCTTTAAATTTAACCATTCAAGATGAAGAAATTATGC
CAAAAGATTTGTTTTTAGCTGAGGAGTTGAAGCTTCAAAAATTCAACATTTGTATTGGTTGCCAAAGCAA
ATTAAAGTATACTTTTGAATCCACGAACAGAATCAAAAACTTCATTGCAATCAAGATGGAATCAGGAAGG
TGCTTGGATGATTGGATAAAAAATTTGTTAAAGAGGTCAGACAATGTGCATTTGGAAGGATCAATTTGTT
CAAAGTTTCTCCACTTAGAATTGGTAGGTGCAAATGACTTCGTAAATTTGAAGTAGCTCTACCTTTATGA
TAATTCAAAATTTCAACATTTTATCAACGTTAGCAATACCATCAACATTGAAGAATCATTTTTTATTGAA
ATGGTAAATTTCATCAACACATTTTTCTATGTTTCTGTATACACATCAAAATTTATAAATCACATGTTTG
TTCATTTTTATTGTTCCTTCCACAAGTTCCTTCTAACATTTATGGTTAGTAATGATATTAGGGATAAGAT
TCTATTCTTCTTTGTTTTAGAAGGCTCTTCTCTTGGCGTAGAGAGACTCTTTCTTGTTTCATACATTAAT
GATAATATATAATTTTAGACTTCTAAAGTATATAAATGCATGTTAGGTGATATTCCAAGAAAATTTAAAG
AAGTTTATTAGATATTTTCTATCTAAGGAAGCACATTTGAAGTAATCATTTTACACAATTTGTTATGGTA
ATTTTAAAACACTATATAAATTATATGTTGTTAGTTAGCATCAGTTGTGATGATATGGTTAATTACGTTG
TCACAAACTGAACTTTTAAGGACGTAAATTTGTAGGTATCGTTTCCTAATTTGGAGACGTTGGAAATTGT
GAATGCAGAGAGTTTGAAGATGATATGGAGCAATAACGTGCCAATTCTTAATTCCTTTTCCAAACTCGAG
GTAATAAGAATTTGTTCATGCAACAATCTTCAAAAAGTATTATTTCATCCAAATATGATGGGCATTCTTA
CATGCCTTAAAGTCTTGGAGATTAGAGATTGTAAATTGTTAGAAGGGATATTTGAAGTGCAAGAGCCAAT
TAGTGTTGTTGAAGCGAGTCCTATTGTGCTCCAAAATTTAAGTAGGTTGAAATTATATAATCTTCCAAAC
CTTGAGTATGTGTGGAGCAAAAATCCTTGTGAGCTTCTGAGTTTGGAAAATATAAAATTTTTGACCATTG
ATAAATGTCCAAGACTTAGAAGAGAATACTCAGTCAAAATTCTCAAGCCACTTGAAGATGTAAGCATAGA
TATCAAACATTTGATGGAGGTTATTGTGAAGGAAAAGTCAGCAGATCATAATATGTTGGAATCAAAGCAA
TGGGAGACTTCTTCTTCTTCTAGGGTACGTATATATTCTACAACAAAACATGTTTGTTCAATTTAAATTT
CAGAAAATAAATTGTTTTCAAGAATTAGTTGAACTGAATTGGTTCTGTTGGTTCTGTTAGTTGAACAGAG
TTTTAAAGAAAACAAATTATTAGGGGTTAATTAGTGTTATTAGGGGTTAATTATTTACCTGAATTAAAAA
AGAATAAGCAACGTAAACCGCTTAATTTTAATATGTTTAACAATAGAAATTTGGACCCGTTTGGATTGAC
TTGAATGACATGTTTTCCTGGAAAGAAACTCATTTTTGTTTGAACTCATTTTTATGAAAATTGGCTAAAA
TACATTTAAAAAATTAGGTGACTTTCAAATATTCAATTTGTTTTTAAAATAACTTATTTTTTGAATTAAA
CACTCCAAAATGTAATTCAAAAACACCCCATAAGTTAATTAGATATATAATTAATACATAAATTAATTAA
TTTTATTTAGTTTGAGTTAAATTAGTTTTAATGCAAACAATAATATATTTTTATAGCCAATATACAAGAA
TGAAAGTAAGAAAAGAAGACCAAAAAAAAAATGATATGTTAATTAGAATTTTACATAAAGAATGATCTTA
TTAAAGCTAATCTCTATCGATATAGTTTTTAAATTATATTAAATATAGTAAATAGTCCATTATAATAATT
GAATTTTTTTGTACCAAATTAATTAAACTAATTCTTTTTACTAATTATTGAATATTTAAGATATTTTTGG
CTAATTAATAAATAATTTGTGTGCAAATGTAAGTTATCGTTGCATTTATTTAATAGATTTTGATCATCAG
GATGGGGTTCTACGGCTGGGAGATGGTTCTAAGTTGTTTCCAAATCTTAAAACATTGAAGCTATATGGTT
TTGTTGATTATAACTCAACCCATTTACCAATGGAAATGTTGCAAATCTTATTCCAACTTGAAGACTTTGT
ATTAGTAGGAGCATCTATCGAAGAAATTTTCCCCAGCAACATACTGATTTCAAGCGATATGGTTTTAAGA
AGATTGAGTCTATCTAAACTACCCAAGCTTAAGCATTTGTGGAGTGAAGAATGCTCACAAAATAATATTA
CCTCAGTTCTTCAACATTTGACTGATGTATCCATTTCTGAATGTGGTGGATTGAGTAGTTTAGTATCATC
ATTGGTGTGTTTTACAAACTTGAAAGATCTTCATGTGAATAAATGTCATAGACTAACCCATTTGCTGAAT
GCTTCGGTGGCTACAACGCTTGTGCAACTTGAAGGTTTGACAGTAGAAGAATGCAAAAGGATGAGTAGTG
TAATTGAGGAAGGATCAACCGAAGAAGATGGAAATGATGAAATGGTTGTATTCAACAACCTACAACATTT
ATACATTTTTAATTGTTCCAACCTAACAAGCTTTTATTGTGGGAGATGCATTATTAAATTTCCATGTTTG
AGGCAAGTAGACATTTGGAACTGTCCTGAAATGAAGGTCTTTTCGCTTGGAATTGTAAGCACACCTCGAT
TGAAATATGAAAATTTTTCTTTAGAAAATTATTACGATGATGGACAGTGTCATCCGAAATATCCCAAAGA
TATGTTGGTGGAAGATATGAATGTCATCACCAGAGAATATTGGGAGGATAATGTTGATACCAGAATTTCA
AATTTATTTGCCGAACAGGTTAGTATATTTAATTACCTTTTCCATATTGGTAATAATTATTTTTTATTAT
TTGTGTGTTGGAGTATGAACTTTAATGAATTTATTTAATTAATGCAGAGTTTGGAGGAAAACCAATATGA
AAATTCTTCTTCTTCGAACAATAATGTTGAGAAAGAATAAAGAATTATATGGATATTGTTGTACACTACT
TAATATATCATTTCATCCACAAAGAAAAGGTCAGACTCTTAAAATCCTCCATTCTTTTTTATGAGAGAAT
ATCATCCAATGTCAGATTGAAAAGTCTCGATAGATTTGTTAAATTAATTTTTGATACAAGTCATAAAATG
TTGATGTTTGATGATGGACTTTGCCAGAACCTGGTATGTGGAAGTTGAATACAAATGTTACATGAGACGA
TCCTATCTCGGCAAATATGTAGGCTAGGTTGGATGTTTCGTGTGATCAATTTAGGCAAGTGTCTCTAGTC
GGTTTGAAAGCAATTTCTGATCGCTATGAAATAAAGATTCTTGAAACCATGACCATTTTTGGAAGGGTTT
TCTAATAATGTTATATTATGATATGATGATGGGGCATATGATTGTGGAGTTCGATTGGTCAGATTTGGTT
TAAAAAGACATATTTTTGGAACTACCTTGTAACTCGATGTCCCTTTCTAATAATAGTTACTAAGACACGT
TAATATATAAATTTTAAGTTCAATCCCAATATCTAAAATAAACTTGTCTACAACTATATTAAGCTCTTGT

TCCTCTTTGATGGGTGATATAATGGACAAAGACTTGTCGAAGGTCTGGCCATCAAAATAATTTTTCGTTC
CTACTTAAGCTAGATATAGTGGGTAGTACAAGGTCGAACCACATGGAAAACAGTTATTTTTCATTAAGAA
AACGTTTCTAAGTAAAACATAGTCTAAAGGAGGTTTGTCTGTTGTTTGCAAGAAAGTAAAAGTGCATTTA
CAAATATTAAAGCAAGAGATTGAAATTAAATATTGATAATGCAATGAGAGAAATATTGAGAATTAGTCTT
ATATGATTGAGAGGAGTCTAGCCATGATCGTACCTGGAGCATTCATAACACCCTTTAGTATAATAATATA
TCTGATCCCATCAAATTAATTAGAAGTAACTACAAATTTAACTTCTGTAATATCAATTCAATTTGATGTC
TCAATCAACTGTTGAATCTTTTTTTTTTCTTTGGATATAAAACCAACAAGTTAAAATAGATATAACAATG
AATTTAATTCACATGTAATAAATTCATCCAATATAATGTTCTTTCACCTTTTTCTCTCTTTCATAAAACT
GTAATAATAATATCTACCACAAAAGGTAAACCATTAATATGATTCTTCAAGAAGGTTGTTTATTTGGTCA
ATTTTTCATGAAAGTATCAATACAGTGTATGTTTTGCAAAGGAAGCTGCCAAATAACCTACTCAATCCTA
GCAGATGCGTTCTTTGCAAGTCGGTTGTCGAAGACTTGAACCATATTTTCACAACTTGTGCCAATTCGCA
AATAGCCTCTGGGTGAAACTGCAGTACCAAATTGGTGGAAACTTTGATACAAACAATATCAAAGCCCTCT
GTTGGTCTCTTAGCTTGCTGAAACAATCAAACAGAAAGAACATCATTCTCTCGATGCAGGTGTTGATCTT
CTTTGGTCCATTTGGGTGGAAAGAAACAATAGGATTTTCATAGACATAGAAAGAAGCTTAGTCACATTTG
GGAAGATATCGAAACTTTGATTGGACCATGGTCGAGTAGAAACAAAATGTTCAAAGACTACAATATATCC
AACATCAATCTTTAAACTTTAGAGCTTTGTTAGATTAATGTTTGTTGTATATGGCTTATGTTTGATTGAA
GTGATTTAGATCTTTTTTATTATTATTATTATTATTATTTTGTTGTGAAAAGTGAGGGAACACTATAGCC
ACCTAATTTTATGCTATCAATAGTAAGATATTTAGATAAAAACTACCAATGATAAGATATTTTATTCATT
TATTTTGTTAAAAAAAAAAAAGGTAAAATCATTTTATAATAATATGAGATCTTATGTTTTTTTAATCTTTT
TATTAAAATAGAAAATAAAGTCATTAATGAGAATATCTATTATTTAAAAAAATTCAAATCATTTTTGCCT
TATCACTTTAGGAAAAAAGCAAATTAATTTATTTATACAAAATCTCTTTTGATTTATCATGTTAGAAAAA
GCAAAATAATTTATTTTATACAAAATTCTTCTAATACCATATTTGATTTATCTTTATCACTTTAGGAAAA
AAACAAAAAGTTTATTTCGGACAAAATCTTTCAATATCATATTTTTACTATTTTAGAAAAAAAAAATTAAT
GAAATTACATAATATCTAATTTGATTTTATGCTTATTAAATTTTCCTAATTTGTGGCACACCCCGAGTCT
ATAAATAGAGTCCTTTGTTATTTGGAAAAGGGGAGAAGAAATTGTTTTAGACAAATTCTCTAAAGAAAAA
AAAACATATTAGAAAGAATCTCTACGAGAGAAAACAATTTTTTGATTGCTTAGAGAATCTCTGTAAAAAT
TATTTTGAAGATACATATTTCTTTACAAAAGGTGAGTTATTACTTTTTTTTTTTTGCATTATTTTCATTT
TTATCTATTTACTTGTCTCTTTTTTTTTTCTTACCCTAAATCTAATTGCATAGAGGAAAAAAACTTAGTT
GGAGGTTGCATTAGGTAGGAATTTATTCCCAAGGATCTGCACCTCATACAACAAGCAATCTTCTTCGGAA
TTGAGTCCTTATGTTTGTTCATTTTTTTTAAAACGTAAAGAGAAAGAAAAATGGCATAAGAATTTTTTTT
CGTTTTGCTACTTTTTATCGTTACTCTAAAAAAAAATTTTTTAACATTTTTTTTTCTCTGAAAAATACAAA
AAAAAAACTTTATAGGCAAAAGGTCTTATGAGAAAAATTATTATTATTATTATTATTATTATTATAAATA
TTTTTCTTTTCTTATTCTGGCCGCCCCCCCGGNNNNNNNNNNNNNNNNNNNNNNNNNNNNNNNNNNNNNN
NNNNNNNNNNNNNNNNNNNNNTATTCTTCCGGCGGCTGTTTTTCTTTATTTTATATATATATATATATA
TATAGAACTTTTTATTTATTTAGTTTGTTTTTCATATTTATGTATTTTTTTTATTTTTTGTTATTTTTAT
TCTTATTATCATTATTATTATTTCATTTTTTAATTTTTTTTAATTTTTAAATCTACTATCATCCATATGT
CCTCTTCTCTTCACTTATTCATTTAATTTCTCTTTTTTACATATTTATTTCTTTATTATTTCATTTAGTT
CTTTTTTCATTTTTTTTTATTATTTTTTTATTACTATCATTATATTTAGTATATGCATGCATTAATATAA
TAAATTTTTTGCCTAATTCATTATGTGGTGTAGTTGTTATTTCTATTTGACTTTTATTAATAATTTCTTG
AAATTTTAAAATACCTTTAACCATAATTGTATGTGATTTTGAATCTTTTAATTTTTTTTTCTTTAAACCAT
TTAGAAATTGTTTTTTGTTTCAAAATTATTTCTTTTAAACTCAAAATTAAATATACATTTCATAAGGTTT
CCTAATTTACATTTTTTAAAATAACCCACACCGATTTCACTAAAAAATCCTACGATGGAATTTAGAAATT
TCTGGGAGTCCGTTAGTTCTAGATTCTTGAGGTGAGGGATCAATATCTTTGGGAGTCGGAGATTTGATCC
CAAGATAAAATATATTTAATCAAAGTTTAAAAAAAAAAACTTTATTCGAAGACTAGCCTATGATAGAAGT
TGAGAAATTGTAATAATTTCTCATTCTTTTAAGGTGAAGAATTTTTCCTCAATATAGTCTAATTAATGGG
TGTATCCCACTTATTTTATGAGATCATTGCTCCAAATATTGGAGTAGTGAGCAATGAAATAAGACATAGT
TCTTTAAAAAAAAAATGAATTTTATTATAGACATTAAATTTGGCCACCGTTTTCTTAAACAGGTGTTACGG
GGTGCTAACACCTTCCCCGGTACGTGCGACAGCTTGGCGACTCCACTAGGAACATAAGGTTCCACCTTGA
GAGTTTGGTCTTTTCTTTTGTCTTAATCTTGTATATTTATTTATTTTTGTTTTATTTTCTTTTTAATGTG
TTGTTTATTATTGTTTCACACACAATCACAAGTCTCCTGCCCAGGCTCTACCCTATAGAATTAGAAACAA
GGATGGAGTAGTGTTGACCTTCGAGGAGCTTATGCCTCCGTGTAGGCACACGAAAAAATTCTCACTCAATT
TTGTCATACAATCACATGCGACGGTTGTGATCAAATTAAGGGCCTTTTTAGGACTTATATTCAACTTCAC
TCATTATGATTCGCATATCATGTTAATTCATATATTTTAATTTTTCTATCTTATTATATTATTCTCACAC
ACTAGCACAAGCCCTCTACTCGGGTTGTACCTATAGGATTAGATTCGAGAATGTAGTAGTGTTTGACCTT
CGAGGAGTTGTTTCTCCAGTGTAGGCACACGAAAAAATTCTCACTCAATTTGCTTGTATGATCAAGTGTGA
TTATACACCATTTTGAGGGCCTGTGTCAAATAGGATGCTAGCATTAACTTCACTAAGTTTGTTTGAGTCA
TTGCATTATTTGATGGTTTTTTTTCTCACATTTAGCATTTACATCTAGACTCATCCTAACTTAATCATTA
TTCTCTTTTTAAAAAAAGATAAAAATCCAAACTTGCAAAATAACTCAAGGTTCACCTCATCAACATCCAT
GTTGGATAAGAAGAAAGACTCAAATAATGGATGAGCAAACCAATGATTAGGTTCAAGCAGTTCGTCAAGA
TGTTGAAGGATTGAAAGATCAATTGGCAAAGATCTTGAAACTGCTTACTACTGGAAGAGGAAAGAGTGTT

GCAGGGACTTCATCACAAGTGGAAGTAGATCTGAACTAGGTACTACAGGACATGCTTGCAACCCTCTGAG
TTTTACTCCTCAAAGGTCGTCTAGTCTACGCATGGTAGATAAGACATATCTTACATCCTTTCCCGTGCCA
AATCCTAACATAACCACCCAACTGGCAGTCCATGCGAGCGATCCCATATCTACTCCAATTACGGAAAGCG
GTAAGAAAATTTCAGAAGAGTAGGGTAGTAGAAAAGACTAGAATTTCTAGAAGAAAGATTGCGCGTCATT
GAAGGTGCAGACATGTACGGGAGTATCGACGCAGCACAACTATGTTTGATATCAGATGTGGTGATTACCA
GATCACGCCACGCGTCCGCCTCGAAGTCGTGTGTTTGAACCCCAGGCGCGCCCTTATTTTTCCTTTCATT
TTTTTGCACGACCCGAAAGCGTGATTCGGCAACCCGATCCACGACCTACGCCTAGTCCGCCACGTGTCGA
GCCCTCGTCCGCGCGCGCCTCTCCCCTGTCGGCAATTTCACTTGAAGAAATCAAAGAATCATAAAAGGAA
GCATGCACTTCTCTTTGAAGCAAGCTTTTCCTCGGTACTTTCATACATTTTTTTTTACATAAATATGCTT
TGAAGACAAAGCAAAGCTTTTAAGTTGGGGGTGGGTGCTAATTTTTTTTTTCTTCTTTTAAGCATTTGCA
TGACTTGGGTAGATTAAAATTGACCAAATATTTTTTTTTTTTAGTATTAGTCTGAATTCATGAATGAAAT
TAATGCTATTTGCTTGAATGAATGCTTGCAAAAGAAAATGCATGGATGTTAACGTTTTTGCAAAGTTTCT
TATGATGATTGCCTATGATACACCTCTCTTAGCAATTTTTGTTTTGATTTTAAATAGGATAAAGAACCAT
TTATGAAATTGAGGTTAATTTTTAAACTTTTCATCTCTTTGTCTCATGAATTAGAAAAAAAGAAACTCCT
TTCTCAAGCCATGACGAACCTAGAAGTCTTTAGAAAAGGATAGAATGCTTAAATTCGCTTGTTGTAACCT
CGAAAGAACGGTAAATCATAGAACCTAATAGATTAAATCCAAGATTAGAATTAGCCAAGCCGGAGCTAAG
CAAAAAGCTTGCTACCAAAAATAGAATCCTAATAAATAAATAAATAAAGTAGCAAAATGCATACTTCTAG
CAAAATACTTTTATCAAAAAAAAAAAAAAAAAAAAGAAAAGAAAGAAAGAAAGAAAGAAAGAAAGAGTACG
ATTAAGTCGAACATTAGGATTACCCGATGTTAGTAAGGTACGAATCCCTACAGAAAAAAGCCTATCCTTT
GCCTTAGTAAAACCCATACAATTCTATCTTAGAATTTTCTAGCTAGTTTCAATGTTTTGACCGAGGGCTG
AGTTTAAGGAGGGATCCTTCCCGCTTCAGAGTAAAGGGAAAACAAGCATAGGGCATTTTTTAATTCTTTC
GAAGCACGTCTGTAGTGAGTCGAAATAGCAAGACTTAGATGTTTTATCTAAACATGATTTCAATAAATGA
ACTTTATTAAAAATCAACCTCAAATTGACTACTTGCTTCTCTTGAATATTTAATAAAAAAATTACTTTGT
TGAATTGTTAAGGCAAGAGTAACATCCTTTGCATATTTGTTTGAAAACATCATCAAAATTTTTGCATGAT
TGTTTGGTTTAATTATTGCAAAATAATGTTGAATCCCTTGCATGATGAATGACTGAATGTTAGCACATTT
TTAATTTTCTTGCTCGGGACAAACAAGTCTTAAGTTGGGGGTAACTGTTTTCCTTTGAAAGCTAAAGTGC
AAAGTCTAGTCAAGGCCGGATGGTTGAAATTCAAGAAGACAGGAGAAGATTCTAACGTTAACTAAAACCC
TTTACCAAATCATGAGGGTCCCACCATAAGTGTTGTTGACACATTTACGAAAAGATACAAGAATAAGGTG
TGTGATGTGACTACTTCAATGAATACATTTTTCCAAATCCTTCGTGGAGCTGGATATTTGTCACCAAGGT
TTAATAATGATGAGGGGGATAAGTTTGGATGCGTCAACGAGAAGCATTGTTTATTCCACCCCGAGATAGA
TGACCATTTCATTGAGGATTGTTGTGAGTTCAAGAATAAGGTACAAAAGTTGATGGATGCAAAAATTCTT
TTGGTAGGATAAATGAGCATGCAAGAAATCGAGATCGATATAATTATTGATGCTTCATTCTAATAAGAAA
ACTTCAAAAGATACATCAATTACGGTTATCTCCGAGAATACTATTTTGCCTAATCCATTGGTTTATCAGT
GTCCACCAAAATTTGAGTTGAACAATTGGGAGATGAAGAGGATGCTAAAAGTCTCTAAGGGATCACAAAA
GTAATAGCATTTTATACACCCTCTGATTATGCCTAAGGTCACAAGGTTTCTTTTCACTAGAAAATTTTTG
GCCTTTAATGTCGGTTTAAAACCGACATTAAAGGCCTTTAATGTCACTTGGCAACCGACATCTTTGTGAG
CGTTATTAAAGGCCTTTAATGTCGGTTGGGTTTTAATGTCTGTTTAAAAACGACATTAAAGGCCTTTAAT
GTCAGTTTTCAACCGACATCTTTGATGGTGTTATTGAAGCCCTTTAATGTCGGTTGGACTTTAATGTTGG
TTTAAAAACGACATTAAAGGCTTCTTTAATGTCGGTTTATCAGTTTTCAACTGACATCTTTGATAGTGCT
ATTGAAGCCCTTTAATGTCAGTTGAACTCTGATGTCGGTTTAAAACTGACATTAAAGCCTAGTTTTTGGA
TTCATTTTTACAAATTTATTTTAATTTAATTTTTACATTATTGTCCTATAGACCGACAATGGGTCAATGT
AGATAAATATGTTTTTCACACGTAACAAATCAATGAAATTCATATTATGTTAGAAACTATAATATTTGTC
TTTTACATTTGAATACACAAAATAAAATCTTAATATTATGTTTATATATACATTCTAAAATAGTACATGA
AAAAAGATTCTAATACAAGAATAAATAATTAAAAATCCTAAATGCCTTCTTAGTCTTCAATTTTCAACGG
TCGCTTGGCCATCTCTACACAATCGCTTAGCTTTCAACCCCATATGACTTTAATTATCTACAAACAATAT
CCAAATAAACCATTTATATAGAATAACAAAGCAGCTAAACTAGTACCTAATTCTCAAGCAGCCTCTCTAA
AAAACACATCATCTTGAGATCCGACATCACGAGCAAATCTAATATCAAGCAAGTTTATAAGGGAACTCAA
ATTTGATTTTCCAAGTTTATAAGGGAACTCAAATCACAAAAGAGTAAACTATACATAAACAAACTTGTAA
TTTAAGTCTGCAAACTTGGGTTTCTATAACAATTATGGAAACAACTTTTTCTGCTGCTGCAACAAGCATT
CTTCCATTATTTGGACTTTGTCGCACCATCCTAATTTAGTTGTTAAACATATTAGTACATTACGATGCAG
AGGTGATGTTTTATAAATAATACCAAGTGCAGTTTAAATATGATTATTGATTTCAGTACATATAAAAATG
TTGTTAGACATTAAACTCATTATGCTGGTTAAAAAACATTATAGTTCACAAAAGCACAATTGAATAAACA
ACAATTTCAATACATACCTGGTTCAATTATTTTGCAACTCGAGCAATGCTACAATGATTATATGCCTGTA
CAATTCAATAACAAGTAAAGAACTTGTGATGAGCTCTAATAAATGCTTCAAATTGATCAATGAAATATAT
TGTTCAATAAGGGAGCAAACTTGAAGTATATTTCACAGAAATATTAAAATGACAATTCAATAAAGAGAGC
AAATGTCACTTGTCATCATTTATATTAAAAAGAAGTGAACTACATCACAACCTTGTTTTTCCAAACGTCT
ACATGGAATTACTTGGTAACTAATATGCATGTTATTAGAGATATTCCATAACATGCTAAACATGCTCATA
TGCATGTTTAGAATAAAAGAGAGATATTCCATTGGAAGAGCAACTAATATATGCCTCTTTAGAAAGTTCA
TCATTGCTCAGAAGAGTTGGAAACAAAGTACATAAACACATGAATTCTTTAACAATACTACTTGATAGC
GAATTGCATAATTATGCAAGTGTTATAAGAAGAAAGTGTTCTAAGCTTTAAAACAAAAGTTGTGTTTATG

ACTAAACTCTGATTTGGAGAATAGGCAAGCTCATAGAACCAGCTCAAAAAGTTCCTGAAGCATGTTAGAC
AACCTACAAGAAATGGAAACGTGTAAAGTTGTGAATAATAATGAGCTTTATGATCAAAGCTTTAGAATGA
ACTTACCCACAACAAGCTTCTTCTTCTAAAACTTATCAAACAAACAAACAAAAAAATAAAAGCTTGAGAAA
TGAGGTACAAGATAAGCTTGAAAAATAGAAGAATGACTTGAAACTCTCTCTCTAAAACTTTTTTTCTTTT
TTCTTTTTCTATTTCTCTCTCTCCAAAACTTTTGAATATTTCCGGCATTGTGTACCCTTCCTTTCCTTTT
TTTTGTTCTTAACTTTTTGTTTGTTTTTTTGTTTGTTTGTTTGTTTTTTTTTTTTTTTTTTTTTTGCTATA
ATCCATTTTCTTCCATCTCTTAGTACAATGAAGAAGCTGTTTGGAATTAACATTAACAAGTAAATAAGGT
TAAGAACAATACATTAATAGATCCCGATATTTGGATATTCCTCTTGAGAATCTACTGCTCTTTTTGAAAG
GTATATAATTGTATGAGAAGTAGCTGATTGTATCACACTAGATGTTTTAAGAGTTTCTACTTACCTCCTT
AGAGATGCAAGGTATTCCTCTCTTGAAACATTCTGCATTCATTCAAGGTCCCTTGTATAATTAGTAACCT
GAAATATGCAATTGTTACATTTCTTATTACACAAAGAAAGTCGAGTAAAACAAAGTATGTAAGTAGGATA
TGGAAGATACTTCTCTAGTTAATATAATACTTTTTCCAAAACTCTGTAATTGTTCAATACACTTTTTGAA
AAGCTAGAAAGCGTCCCACAATTTAAATAGAAACAACTCTAGGATCGTAAGACAAAGGATAGAAAAGAAG
AATACATAGTTCAAAGTATGCATAAAAATAATTATCGAGAAACTATGGTAACGAGAGAATCAATTTCTAT
CTGGCATTTTTCCATTTCTTGAAGGGGAAGAGCATTAAGTGACATCTCACTGGAAAATTAATAAGAGTCC
CAAGACCCCAATATTTTAAGGCAGCCAAATCGTATGTCCTAGCAGCAGACTCTTCTTCATCATATGCTCC
TGTAAATTCACACGTTATTCATTGAAAGTGTGCGTAAAGAGCAAAATAATTCAATACTAGTCAATGTTAA
AAAGTACAAAGAGGAAATTACATTAGAATATTGACCCAGATACACTGGAATGATCAATCATGAGCCAGAA
GTTTCATGCACATTTTAAAGATAAAAGTGTTGTCAATACTTAGTACTCCAAAGCAAACCGGTGATTGGTT
TCACACTGATATTTGTGTGTGTGTGTGAGAGAGAAATGCCTTGCTTTCCCTTCTTGTTCTAGTTCCATGT
GCTTTTATCCCAAAGATGAGCTTCATAACGACTAGTCCATCTATGCCTGATATCATCAAACCATTCACAG
GTGCTACTTTTATGAATTAGAAATAGCACTAAGAAATTATCACTCACAATGGATACTAATGGAAATATTA
GAATCCAACATTATGTTTCTAAGCTTTTGCCCGCCACAAGCTCGACGAAAGTGCACATCTGGAGTTCCAT
CAGGAAGCAACACAGACTAAAAGGAAAAATATATATATATATATATATATTAATGAACATTTTATCCAAA
AAAAAAAAAAAAAAACAACAATACATCATCCAAAAGAATGTGACTGATACTAGAAATAAATTCAAAAATA
CTTTAAAAATCTATTACAGATACCTATTGAACCTTTCAGATGACAGCTAATCGGCGCTCTTGCAACATAT
CTAGCCACAAGCAGACTAGATTTGAGCCTGTGAGATAGATGCGGACGTGCAACGGTGATGGTGAGGGCTG
GGCATGAACGTGGTCAAAGAAAAGGACTACAGACGGTGGTGGCAAGCACAAAGACGATCGAAGAAAGGGA
AGAGTGACAACAGTGGAGGCGGGCACGAAATGGGCACGAGGGACGACTATGGTGGTGGCGGCGGCGACTG
TTCGGAGAAGAAAGAGAACATTGTGAGATGAGAATGAGAAGAGAAGAAGAAATTGGAAGGAAGAAATTGT
GTGGGTCATTTAAAAAGAGTAAAGTAAGAGAGAGAAATAGTTAGATTTTATTACAAATAAAAATAAAAAG
GATTTTTAATGTTGCTTTTCAAAAGGAGGATAAAGCTCCCTCTTTAATGTCAGTTTAAAACCGATATTAA
ACATCCGCTTTTTAAAAAGTGACATTAAAGCTCATTTTTCATTTTTTCCAACCGACATTAAAGGCTAGAT
TTCTTCTAGTGAAATATTAGGTATATAGTAATAGTTTAAAAAAATTGCAAAACTTCTATCGTTGATAGAC
TCTTATAGTTTACCAGTGATAGACCAACATTTTCCACATGGTCTATCAGTGATAGACTCCTATCATTGAT
AGATTTTGACAGATTTTGCTATATTTGTAATTTTTTTGAAATGTTGCTATATAATTAATTATTTTGAATA
TAATTACTACATTTGCAACTATCCATTTTTTTTCTAAGAGAAATTGTTTTAGTAATGTTTTCTTTAAAAT
GTGAGTCATCCATGCGGATTATTGTTGGGCATGACTTTACGTTACAAAGATTTCTTCTTTAATTGTTGTA
AGTGTTATAATCTTACAAATCTAAAAAACAAATTTGATGAATACAAATCTTATAACCACTAAATGGTCAT
AAATATAGTATTTTAAAATTGTTTGACCGTTACAGATAATTTTAGATCTAAAAGTATTTATTTGATTTTT
CTTACAAATTTTCTTTTATCATTTAAAGCAAATTAAAAGAAAGATTTTTTTCTCGGTTTTTCATTTTTTT
ATTTTCTCTAAGCAAAAGATTTTTCTTTGATTCGATTACTTTTCAGGTGAGTTCTCGTTTGTTAAGAACA
TTGTTTTAGCCTTTGGGGAACAACTAGCGTGAGACGATTAGTACCGAGGTCAAGGCAATATGGGCATTCC
ATGACACAACTAAACCAAACCAGTGGACTAGTTTGGATTGGATTGATTTTTCGGTTTTTTGGATTTTGGT
TGAACTCCTACAATTTAGTATTAATTTATCCAACACCTAACTAGTTTTCTACTTTAAAAATAAAATTTAG
AGAAACTTACATAAATCTAACAAACCTGTAAACTATTTACAGCCCGTGTAATAAAGCCCATAAGGTTAGC
CATTTTTTAAATATTTCAGGTTTGTCCTTCCATCTTTCTTTTCTTTCTCGCGATTTGTCTTTCTCCTGCA
ATTTCGTCTTCCCCATCTTCCCCTGTGATTTTGTCTCTCTTCTTTTCTCTTTTTCTTTTCTGTGATTTCT
TTCCATCATGTTTTTTATTTTTCAATTCTTTATTTATGTCGTTTAATCTCTCCATCGTTTTTCTTATTTT
CCTCTTCGTTTTTTTCGCTGCGATTTCTTTCCATTGTCTTTCTTCTTTTCTTTTCTTTTTTATGTGTTTA
CATTTGGGTAACCAAATCTAAACGACTGTGTACAACAAAATAGCAAAATTTAAAAGATCGTGTATAAAGA
ATCTTGAAAAAAAAAATCATTTAGATTGGAGTAGCCAAATGTAAACGATCGTTTAAAAAAATTAAATGAT
CATGTAAAAAAAATAAAAGATCGTGTATAAAAAATCTTGAACAAAAAATCATTTGGATTGGAGTAGCTAA
ATGTAAACGATCGTGTAAATAAATCTAAACGGTCGTGTCAAGAAATCTAAACGATCACGATCGTGTACCA
AAAGAATTAAAAAATCGTGTACCAAATTCTAAAAAAAAAAAAAATCATTTAGATTTGTGTCCCCAGATCT
AACCGATCGTGTAACAAAATTAAACGATGAATCATGAAAAAATAAATTATAGCCATATCTAAACGATCGC
GTATAAATTGTAGCCATATCTAAAAGATTGCGTATAAATTGTAGTCCTATCTAAACGATCACGTCCATAT
CTAAACAATCGCGTATAAATTATAGTCATATCTAAACGATCGCGTTGTAGTCATATCTAAACGATCACAT
ATAAATTATAGTCATATCTAAATGATCGCGTATATATTGAACCATATCTAAACGATCGCGTATAAATTAT
AGTCATATCTAAACGGTCGCGTATCAAACAATAACCAAATCTAAACGATCGCAAATATATTACGCGCACG

TTATTGACGGTGTGGTTGACTGACCATTTTTGGTATTTTCACGGTGGGCCTCTAGACTTTTTATGTTTTT
TGAATTGTGGGATAGAGTGTAATTATGTAACGACCCAACTTTCCGGACTAAGCTAAGGTCACTACGTACT
GCTACGACTCGACAGTAAAATACACAACTCATAAAAGACCTATTACGATTCATTAAAACATTCGAAATAT
CACAAAAACAGTTTCGAGCCCTATTTTAAATCGCGTTCCCAAAAGTTCAAAAATATAGTTCACAAAATCA
TCAAAACAAAATAACCAATCAAATGTTCTAACCATGACTCCATTTAATTCTCAAAAGAAGTAGAAATTAA
CAAATGTGTCAACGGAAGAATAAAGAAACTAGACGTGTCCATATGGCCTTCACGCACTCTTCTTACCCCT
CGGCGGTCTGCCCCTCGCCATACCATTACCTGAAAAGTTTTAAGAGAAAAATGGTGAGTATAAGACATAC
CCAGTAAGGGACCCACTATTGGACCCATTAGGGTAATAACAGTTAGCTTTCTATTAAGGGGTACCCTACA
TAAACAGTGTAGTGGTTCTGTAGAATGCACACATCAATCTAGTGATCCTGAAGGATGCACATATCAGTAT
CGTGATCCCGAAGGATGCACATATCAGTCTAGTGATCCCGAAGGATACATATATTAGTCGAGGAATCCCA
AAGGACATCGTGCCTATAAGGTACGCTACCCTATAGATAAAGCTAACCATTACCCTACAGTCTATACTAA
ATTGTCTACAACAGTCACATCATAATAACGTTGAAGTTAATGGTTCAGTTATAAGCTTAGCCAGTCAGAC
AGTTTAGGTTTAAACAACTGTCTCCTCAGTCGCTATATGTATATCTAATTCACACGTCATCGCGCATTTC
CTTAAATCCAGTTAACTAGTCAAATCATCACTTTGTAAATCCACCTGCCGGTAATTCGCCTCAGTTCAAA
CCTTAGCACATCTACAGGTAATCTATTTTATGCATAACGTCCATATTATGCATAACAGTCGTATTCGGCT
TACTGCACAGTTCAACGGCGTGTACACAATCTTCACATGAGGTTTTTGAACTCTTAGTCCCTCCACGACA
TAACTCCATAACCAGTATCCCGATAACAGACTTCAACTTTACATAACATTAAGATTTTCATAATCCATTG
ACATTACTATTTCAGTTAACGGCACCTCCCATCAACATAACTACAATACATAAAACATCTGGGCATCTGT
GCCCGTTGACAAAACAACTCATTAACGTATATAAATACATAGGCTACAACTAACAATCACATCACCTTTA
ATCAGTCAAGAGTGCATATTAGTGATGTTTTCTAGGCAAACATGCATCAATCGATTTATTACAGTTGCTA
TCGTATAATTCCCATGTGGGTTACTACATTTCCATTTTCGGTCGAAGGTTCAGTAGTAAGAAATCCCTTA
CCTGAACTGTAGCTAAAATCCTTGTTCAATTTGACGTCCAAACAAATCACCTAAACACAAACATAAGGGT
TTAATAATGACATCACCAAAAATCCACATTACAAAACGTTCCAAAGCAACATCTACCGGCTTACCCAAAT
ACGGGGGAGTTGGCTCCAACTACACTTGCCATAACACTCCTGAACTCGAGCGTCACCTCTCTAACACCTT
CAGAGGATCAACAATTAACCCAACCAATTAGTTCCACATTTTATTTGAATATCTACTTGAATCGGCTAAA
GCAACTTTAGGAAACTCATTAAGTAATCCTTACTAAAGACTCATCGTGACCCGACGTGGAAGGAAAGTGG
CTTATCTCAGCTCGAGAAACAAGGAGGTCGGCTTGGCTTGGTTCAGCTCGCGGCTCGGCTCGGCTTGACT
AAGCTCGCAGCTTGGCTTGCTTCAGCTCGCGGCTCAGCTTGGCTTGGCTCGACTTGGTTCAGCCGCGGCT
CGACTCGACTTGCTTAACTCGGCTCAGCTTGATCGTTTGTGTAACGACCTAACTTTTTATACTAAGCTGA
GGTCATTACTAAAAAGAAACAATAACAACATACACTTTCTTGAAAAGAGGGAAGACTAAATTTTCATTAA
AAACAGAATGCTGAAACACTGATACATAAACGCGGAAGCAAAACTGAGTCTTCATATGGCATGTCACGGA
TCCTTCTCTGTCGCTCGCCAGCTTTCCTCTAACTCTACCTTCGCCTGAAATATTAAACATAGAAAGAGTG
AGTATAAACAAATACTCACTAACGGACCTACTACTAGTCCCGCTAGGTGTCTGTTAACTTTCCATTAAAG
TCCTGTAAAGAAGTATCCCTAAACTGGCACATTCCCGAACACGTGTAATCTGTGATCCTGTAGGAACAAA
TCTGGTCTTCAGTGAACCCTAAGAAACATCTAGGACAATCTGGTCTTTAGTATACCCGAAGGAAACACTA
AGACAATCGAGCTACGAGTGATCCTGTCGAATCACTTGAATCATGTCTATGTCAATGTCACAGTCAAACT
GGCGGTCCCGTCGAACTACGCAGTCATAAGTGTGAGCGATCCCATCTGATCTCTCAAGTCATGTCTGTGT
CAATGTCATAATGGACTAGTAATCCCGTCGGACTACACAATCATAAAAAAAGGTGGTGATCCCGAGGGTC
ACCCATGTGGGTACAACTCTAATAGACAAAGTTAACAGAACACTCTATCCATAGCATGTAGCATAACATA
AAATCATCACATGACATGAGTATTAATCTTAACGTCCTTAATCATATGATTAATATATCATGCATCAACA
TAAACATCAACAGTCGTCAACAACATACTATCATTCATCATCATCAATCAATATAATGAATTATGCATGT
TAGCTACCATCAATGCATAATCATAATTACATGCGGTCTCTTAAATTCAGTTCGAAGGTTTAGTAGAAGA
ATCTCTTACTTGGACATTTTAGCGAAACAAAGTACTCCTTAGCTGACAGTAAAAAAATATCCAATTAAGT
TGATCCTAATCATAAAAGGAAAACTCAATATCTTAATTAATGAAATTAGGTTGAAACCAATTCAACCTTA
ATTGGGAAAATCTAAGATTTAAATCTAAAAAATTGGCCAATTAAATATTTGAAGAAACCCCAAAAAGATC
CAAAATTAAATTAATAAAATATTAATTTAATTTCATTGGCTTCCCAAGGTTACTCAGATGGAGGTTGAAA
AACCTTTTTCCTTTTGGACTNNNNNNNNNNNNNNNNNNNNNNNNNNNNNNNNNNNNNNNNNNNNNNNNNN
NNNNNNNNNNNNNNNNNNNNNNNNNNNNNNNNNNNNNNNNNNNNNNNNNNNNNNNNNNNNNNNNNNNNNN
NNNNNNNNNNNNNNNNNNNNNNNNNNNNNNNNNNNNNNNNNNNNNNNNNNNNNNNNNNNNNNNNNNNNNN
NNNNNNNNNNNNNNNNNNNNNNNNNNNNNNNNNNNNNNNNNNNNNNNNNNNNNNNNNNNNNNNNNNNNNN
NNNNNNNNNNNNNNNNNNNNNNNNNNNNNNNNNNNNNNNNNNNNNNNNNNNNNNNNNNNNNNNNNNNNNN
NNNNNNNNNNNNNNNNNNNNNNNNNNNNNNNNNNNNNNNNNNNNNNNNNNNNNNNNNNNNNNNNNNNNNN
NNNNNNNNNNNNNNNNNNNNNNNNNNNNNNNNNNNNNNNNNNNNNNNNNNNNNNNNNNNNNNNNNNNNNN
NNNNNNNNNNNNNNNNNNNNNNNNNNNNNNNNNNNNNNNNNNNNNNNNNNNNNNNNNNNNNNNNNNNNNN
NNNNNNNNNNNNNNNNNNNNNNNNNNNNNNNNNNNNNNNNNNNNNNNNNNNNNNNNNNNNNNNNNNNNNN
NNNNNNNNNNNNNNNNNNNNNNNNNNNNNNNNNNNNNNNNNNNNNNNNNNNNNNNNNNNNNNNNNNNNNN
NNNNNNNNNNNNNNNNNNNNNNNNNNNNNNNNNNNNNNNNNNNNNNNNNNNNNNNNNNNNNNNNNNNNNN
NNNNNNNNNNNNNNNNNNNNNNNNNNNNNNNNNNNNNNNNNNNNNNNNNNNNNNNNNNNNNNNNNNNNNN
NNNNNNNNNNNNNNNNNNNNNNNNNNNNNNNNNNNNNNNNNNNNNNNNNNNNNNNNNNNNNNNNNNNNNN
NNNNNNNNNNNNNNNNNNNNNNNNNNNNNNNNNNNNNNNNNNNNNNNNNNNNNNNNNNNNNNNNNNNNNN

NNNNNNNNNNNNNNNNNNNNNNNNNNNNNNNNNNNNNNNNNNNNNNNNNNNNNNNNNNNNNNNNNNNNNN
NNNNNNNNNNNNNNNNNNNNNNNNNNNNNNNNNNNNNNNNNNNNNNNNNNNNNNNNNNNNNNNNNNNNNN
NNNNNNNNNNNNNNNNNNNNNNNNNNNNNNNNNNNNNNNNNNNNNNNNNNNNNNNNNNNNNNNNNNNNNN
NNNNNNNNNNNNNNNNNNNNNNNNNNNNNNNNNNNNNNNNNNNNNNNNNNNNNNNNNNNNNNNNNNNNNN
NNNNNNNNNNNNNNNNNNNNNNNNNNNNNNNNNNNNNNNNNNNNNNNNNNNNNNNNNNNNNNNNNNNNNN
NNNNNNNNNNNNNNNNNNNNNNNNNNNNNNNNNNNNNNNNNNNNNNNNNNNNNNNNNNNNNNNNNNNNNN
NNNNNNNNNNNNNNNNNNNNNNNNNNNNNNNNNNNNNNNNNNNNNNNNNNNNNNNNNNNNNNNNNNNNNN
NNNNNNNNNNNNNNNNNNNNNNNNNNNNNNNNNNNNNNNNNNNNNNNNNNNNNNNNNNNNNNNNNNNNNN
NNNNNNNNNNNNNNNNNNNNNNNNNNNNNNNNNNNNNNNNNNNNNNNNNNNNNNNNNNNNNNNNNNNNNN
NNNNNNNNNNNNNNNNNNNNNNNNNNNNNNNNNNNNNNNNNNNNNNNNNNNNNNNNNNNNNNNNNNNNNN
NNNNNNNNNNNNNNNNNNNNNNNNNNNNNNNNNNNNNNNNNNNNNNNNNNNNNNNNNNNNNNNNNNNNNN
NNNNNNNNNNNNNNNNNNNNNNNNNNNNNNNNNNNNNNNNNNNNNNNNNNNNNNNNNNNNNNNNNNNNNN
NNNNNNNNNNNNNNNNNNNNNNNNNNNNNNNNNNNNNNNNNNNNNNNNNNNNNNNNNNNNNNNNNNNNNN
NNNNNNNNNNNNNNNNNNNNNNNNNNNNNNNNNNNNNNNNNNNNNNNNNNNNNNNNNNNNNNNNNNNNNN
NNNNNNNNNNNNNNNNNNNNNNNNNNNNNNNNNNNNNNNNNNNNNNNNNNNNNNNNNNNNNNNNNNNNNN
NNNNNNNNNNNNNNNNNNNNNNNNNNNNNNNNNNNNNNNNNNNNNNNNNNNNNNNNNNNNNNNNNNNNNN
NNNNNNNNNNNNNNNNNNNNNNNNNNNNNNNNNNNNNNNNNNNNNNNNNNNNNNNNNNNNNNNNNNNNNN
NNNNNNNNNNNNNNNNNNNNNNNNNNNNNNNNNNNNNNNNNNNNNNNNNNNNNNNNNNNNNNNNNNNNNN
NNNNNNNNNNNNNNNNNNNNNNNNNNNNNNNNNNNNNNNNNNNNNNNNNNNNNNNNNNNNNNNNNNNNNN
NNNNNNNNNNNNNNNNNNNNNNNNNNNNNNNNNNNNNNNNNNNNNNNNNNNNNNNNNNNNNNNNNNNNNN
NNNNNNNNNNNNNNNNNNNNNNNNNNNNNNNNNNNNNNNNNNNNNNNNNNNNNNNNNNNNNNNNNNNNNN
NNNNNNNNNNNNNNNNNNNNNNNNNNNNNNNNNNNNNNNNNNNNNNNNNNNNNNNNNNNNNNNNNNNNNN
NNNNNNNNNNNNNNNNNNNNNNNNNNNNNNNNNNNNNNNNNNNNNNNNNNNNNNNNNNNNNNNNNNNNNN
NNNNNNNNNNNNNNNNNNNNNNNNNNNNNNNNNNNNNNNNNNNNNNNNNNNNNNNNNNNNNNNNNNNNNN
NNNNNNNNNNNNNNNNNNNNNNNNNNNNNNNNNNNNNNNNNNNNNNNNNNNNNNNNNNNNNNNNNNNNNN
NNNNNNNNNNNNNNNNNNNNNNNNNNNNNNNNNNNNNNNNNNNNNNNNNNNNNNNNNNNNNNNNNNNNNN
NNNNNNNNNNNNNNNNNNNNNNNNNNNNNNNNNNNNNNNNNNNNNNNNNNNNNNNNNNNNNNNNNNNNNN
NNNNNNNNNNNNNNNNNNNNNNNNNNNNNNNNNNNNNNNNNNNNNNNNNNNNNNNNNNNNNNNNNNNNNN
NNNNNNNNNNNNNNNNNNNNNNNNNNNNNNNNNNNNNNNNNNNNNNNNNNNNNNNNNNNNNNNNNNNNNN
NNNNNNNNNNNNNNNNNNNNNNNNNNNNNNNNNNNNNNNNNNNNNNNNNNNNNNNNNNNNNNNNNNNNNN
NNNNNNNNNNNNNNNNNNNNNNNNNNNNNNNNNNNNNNNNNNNNNNNNNNNNNNNNNNNNNNNNNNNNNN
NNNNNNNNNNNNNNNNNNNNNNNNNNNNNNNNNNNNNNNNNNNNNNNNNNNNNNNNNNNNNNNNNNNNNN
NNNNNNNNNNNNNNNNNNNNNNNNNNNNNNNNNNNNNNNNNNNNNNNNNNNNNNNNNNNNNNNNNNNNNN
NNNNNNNNNNNNNNNNNNNNNNNNNNNNNNNNNNNNNNNNNNNNNNNNNNNNNNNNNNNNNNNNNNNNNN
NNNNNNNNNNNNNNNNNNNNNNNNNNNNNNNNNNNNNNNNNNNNNNNNNNNNNNNNNNNNNNNNNNNNNN
NNNNNNNNNNNNNNNNNNNNNNNNNNNNNNNNNNNNNNNNNNNNNNNNNNNNNNNNNNNNNNNNNNNNNN
NNNNNNNNNNNNNNNNNNNNNNNNNNNNNNNNNNNNNNNNNNNNNNNNNNNNNNNNNNNNNNNNNNNNNN
NNNNNNNNNNNNNNNNNNNNNNNNNNNNNNNNNNNNNNNNNNNNNNNNNNNNNNNNNNNNNNNNNNNNNN
NNNNNNNNNNNNNNNNNNNNNNNNNNNNNNNNNNNNNNNNNNNNNNNNNNNNNNNNNNNNNNNNNNNNNN
NNNNNNNNNNNNNNNNNNNNNNNNNNNNNNNNNNNNNNNNNNNNNNNNNNNNNNNNNNNNNNNNNNNNNN
NNNNNNNNNNNNNNNNNNNNNNNNNNNNNNNNNNNNNNNNNNNNNNNNNNNNNNNNNNNNNNNNNNNNNN
NNNNNNNNNNNNNNNNNNNNNNNNNNNNNNNNNNNNNNNNNNNNNNNNNNNNNNNNNNNNNNNNNNNNNN
NNNNNNNNNNNNNNNNNNNNNNNNNNNNNNNNNNNNNNNNNNNNNNNNNNNNNNNNNNNNNNNNNNNNNN
NNNNNNNNNNNNNNNNNNNNNNNNNNNNNNNNNNNNNNNNNNNNNNNNNNNNNNNNNNNNNNNNNNNNNN
NNNNNNNNNNNNNNNNNNNNNNNNNNNNNNNNNNNNNNNNNNNNNNNNNNNNNNNNNNNNNNNNNNNNNN
NNNNNNNNNNNNNNNNNNNNNNNNNNNNNNNNNNNNNNNNNNNNNNNNNNNNNNNNNNNNNNTTTTTTCA
ATTTTTTCATAAAAAAAGGTAGGGAAAATCAGAAGATCGGAATCTATGAAAAGTTTCTTAGAATTTAAAA
TGCAAAAGTAAAGAAAGAATTGTTTTTACTTTTTGATATTAGAACACAAATTGTTAAAGATTCTCTTAGT
CAAAAGAATTTAGAATAAGAATAGTTAAATTCTAGTTTTATCATTGAATTCAAACAAGGCTTAAGAATTT
CATAAAATAATCCACAACAGATTAGACATTAATCATCCAATTGCTCTCTTTTAATGAACAAGGGTTGGCT
CTTATAATTAATCCATATACTAAATTCTTCGACAACATTTGTATCCATAGTGGTTATCTATATTATGATT
GAAATGAAATGATGAGCAAGTTCAATTGGAAAAGTATTTGTGATTTAAAATAATCGTTTTGTTTAATTAG
AGTTATAATCGACGCCTAATTTTATTTGAGATAATGAATTATTAAGAAACTAAGGCATTATTGTCCTTCA
AAATGCTCTGTCCTCTCTCCTCTCTCCAAATCCCCTACAAGCAGTTATTCCCCTTGCCGGAAGATACAGA
GTCACTTGCAACAGCTACACAAAATGGCGGCTCCGCCCATCATTCTAATTTCAACTAAATTCACCCACTG
CATTAACTACCAGCTCAGCCTTTTTCTTGCTGTTAAAAGGCTGCTTTCATCTTCAGTCCCTACCACTGCT
TTTCACCCTCCTTCCTTGTCTTTTTCCAACATTTTCCCAAACAATGGCCTACTTCAAATCACTTCCTAGA
TCATGCAAAATCGAAAGAAAGGAATTTGTCCTTCACCTTGACAAATATTCGAAGCACACTCATTATTGGT
TAACCGAAACTGGAGCTCACAAGGCTTTCTCCATTGAAGCCTCCCCAAAAGACTTGGACTGGATAAGAAG
TACCCTGAAATCCTTGATTGCAACCCCAAGTACAAACCGATTCTTTCTTGAGACCCGTGACTCCGAACAA

TGTATCTGGATCAGGAAAACAAGAAATAATAAAGGATGTACTGCAGAGATCTTTAGAGTAGATCAAAAGA
ACAGAAAATCATGCATCTTAGTTCCGGAAGGACCTGAGAAAAGTGGCTGGGTCTCCTTTCTGTCCATGAT
TACACCAAAAGTAGAAGTGAAAGCAAAAACAAGACCAGCTTTTTTGCCAAGGTCCAGTCCCGATAGTCGT
CTTTCCCCTCCCATTGATTTCCACAAAAGATCATACGCAAGAGCTGTCACTGAAGGAAGATCTTCTGCTA
CAAGTGATTCAAGCGACTCTTATTCAAGTGATTCAAGCCATTCATCAGGTAATAGTCTTTGTGTCTCTCC
CTCATTTGATCTTCTTGAAAATACAGTGGTGATAATCAGAAGGTTCTTTCATGACGACTGGTACAAAATC
CTTCAAAACCTGAGGAAACAAACAGAGGAATCCTTCACGTATAACGCTTTCCATGCTGATAAAGCTTTGG
TCCATTTTAGTTCAAGTATACCTGCAAACCTTCTCTGTCAAAACAAAGGATGGACCACAGTAGGGAAGTA
CTCGGTAAGATTTGAGAAATGGTGTCCCATTCAACATGCCACTCCAAAACTCATTCCTAGCTATGGAGGA
TGGACGATTTTCCGAGGAATTCCGCTACACCTGTGGAATATGAAGACTTTTCAGCAAATTGGAAAAGCTT
GCGGAGGCTTGATTAAAGTGGCTGAGGAAACAAGATCAGCTAAAAACCTGATAGAAGCAAAGATAAAGAT
CAGATACAACTATTCAGGCTTCTTACCAGCAAATGTTAGGATCTTTGATAATGAAGGAAACAAATTCTCC
ATCCAAGTAGTTACTCATCCAGAAGGCAAATGGCTAATAGAAAGGAATGTCAGACTCCACGGCACCTTCA
AGAGACAAGCTGCTGCTGCCTTTGATGAATTCAATCCTGAATCCGAACAGTTCTTCTTCGAAGGAATGGA
GGCCATATCGCCGGATTTCCTTGCCACCAGCTCCGACGGACGTAAAAGCAGTACACCGGACCAGCCATCT
GCATTAAAATCGGTTATCATTAAACCTGACAAAATTGCCACGTCGCCGAGCTTATTAAATGAAGAGGCAG
TTAATGATAGTAATGTGCATGCAACGGCTAATAAATCCGGACTAGAGATATTACCTGGGATATCCAATGA
TGGCGTGTTGGATAAAGGAAAACAGAAGGTTGACATTCAGCTTCCACCATTTTCAGCATTAAATTTGGAT
AAATCTAAAAGGAAAGTCTCCTTCTACTCGCCCTGTAATAAAACCAACATCTTCAATCCGGATTCTGCTC
CTGCCAATCATTCTCCATCATTAAGTTCTCCTGAGAAAAAACAGAAAGTAAGTAGAGAGAGAAGTATCAA
GAAGAGATTGCCCTCCACTCAACCGAACTCAAAAGCCAATCAGAAGAAAGGAGAATTAAATATTCAACCA
ATCCAAATGATGGCACATGAAGGGGAAGCTTCTAAAAAAGGTCTTTTGCTCACTGTTGACCTGGGAGATC
TGCCAATCTTGGATCCAAACAAATCATTTGAAGACCACCACAGCTCTGATAACGCAGAGGTTATTGATAT
AACAAACACTGAAGTGGTTCCCGAAACACCTGAAATGAAAATGCAAGTTAACGAGAATTCAAATTCATCC
TCTGAAGTCAACTACAGAAAACCAAAACTTGCTCATAAAAGAAAATACTATTACAGGAAAAAAGAAGAAA
AAGAGAAAGATTCGGATTCAGAGGCCTTCAAAAAACAACTTGCTTTCTGGTTAAAGGAAAATGGTCTGAA
ACTCTCTGCGGTCAATGACTCTTCAAGGGCAACTACTTCATCAAATGTTTTGATAAACCATATCAGTTCG
GGGATAGTTTCAAAGGGGAAAGGGGATTTGGGGACATCCATTGTAAAATGAAACTGCTTACTTGGAATGC
AAGAGGTTTAGGCTCCCCTTCTAAAAGAGCCATAATAAAAAATACTATAATTTCATACTCCCCTGACTTT
GTGATTCTGACTGAAACTAGGCTTAAGATCACAACCAAGAGAATCATTAAATCCCTATGGCCCTCTAATA
GTATCAATTGGATCGCTAAAAATGCTTTGGATAGCTCTGGTGGGATTTTAATTCTTTGGGATGCTCAGAT
TCATTCAATTTTAAGCCACGAGGAAGGGTTTTTTAGCCTTTCAGCCAACTTCTTGTCCAACAACAATCTG
TCCTGGTGGCTAACAGGTCTTTATGGTCCAGTTAAAAGGAGGGAAAGAGCTCGTTTTTGGACTGATCTTC
ATAATCTTCAACATCTTAATTCACTCCCGTGGATTTTAGGAGGTGATCTTAATGTTACCAGATCGAGAGA
GGAATCAACATCTATTTCCAGCTCTACCCACAGCTCCAAAATGCTGAACAACTTCATCAACAATAATCTT
CTACTTGATCCCCCTCTCACAAACAATAGATTCACTTGGTCTAATCTTCGGAATCCTCCTACTTTTTCTC
GAATCGATAGATTCCTTTACAATTCTCCTTGGGAAAATCTCTTCAGTCCCCACACAACCAGGACCCTCCC
AAGATCAACTTCAGACCATTTTCCTCTGGTTTGTGAAGTTTCCAATCCCAAGCTCAGTTGGGGTCCAGTC
CCTTTCCGTTTAAACTCTATAGCCCTTAATGATCCAGATTTCAAAAGAAATTTGGGAAGATGGTGGGAAA
ATTCGATCCAAGATGGTCACCCTGGATTCTCCTTCATACAAAGGCTAAAGTCCTTAGCTAATTTTATCAA
ACCTTGGCAAAAAGAGAAGTTAAACTCTTTTGCCTTTGCTAAAGACAGTATTGTAAGGGAAGTGGATTCT
ATCGACAAGAAGGAATTGGATACCCCTTTGTCTCAAGAAGAAAGTGATCGCCGCCTAGCTCTTAAAGCTG
ATCTCAGCGAGTTATCTCTTAAGGAGTCCCAATTCTGGTACCAAAGGGCCAAAAAGCTTTGGCTTAGGGA
GGGAGATGAAAACTCCTCCTTCTTTCATAGAATTTGCTCAGCAAGACAAAAGAGAAATTTTATTCAAGAA
ATTCAGGATGAAGAAGGCTTGACTCAAAATACAAACAACGGTATTTCATCAGCTTTTATAAAATTCTTTT
CAAGGATTTACAGAGGCCCCACTAAAAGCGATCCTGTCTTTATAGACAATCTAGATTGGAATCCGATTGA
GCATTCTGAGGGGTTAAACCTTTGTGTCCCTTTTCTGGAAGAAGAGATTAAAGGGGTCATAAACTCTTTA
GAGGGTAAGAAGACCCCTGGTCCAGATGGTTTCCCTATCTCCTTCTTCAAATCATATTGGCATCTTCTAA
AAGAAGATATCATTGACATTTTCAAGGACTTCTATGACAAAGGTGTTATCAACAAGAATGTGAACAATAC
ATACATCGCTTTGATCCCAAAAAAGAAGGATTATTGTAATCCTAAAGACTTCAGACCGATCAGCCTAACA
ACATCCATTTATAAGATCATTGCTAAAACCCTTTCAAACAGGTTAAAGACCTCCCTTCCTGATACCATCT
CAGAAAACCAGCTAGCTTTTGTCAAGAATCGCCAAATTACTGATGCTATCCTAATGGCAAATGAAGCTGT
GGATTTCTGGGAGGTGAAGAAGATAAAGGGTTTATTTTGAAGCTTGACATTGAAAAGGCTTTCGACAACT
TAAATTGGGATTTCATTGATTTTGTCCTAGAGAAAAAGAACTATCCTATCCTTTGGAGAAAATGGATAAG
AGGATGCATAAGCAATGTCACATACTCAATTATTGTCAATGGAAGACCCCAAGGTCGTATTAAAGCCAAC
AGAGGTCTTAGACAAGGTGATCCTCTTTCTCCTTTCCTGTTTGTTGTTGCCATGGACTACCTTAGTCGTC
TTTTATCTCATTTGGAAAATTCTGGTGCAATTAAAGGGGTCTCGTTCAGTAATAATTGTAACATATCCCA
TATCCTCTTTGCTGATGACATTCTTCTTTTCATAGAAGATAATGATGTTTACCTGAATAACCTTCGAATG
GCATTATCTCTGTTTGAAAAAGCCTCGGGCCTTAAAATAAACTTATTGAAATCAGCTCTGGTGCCAATGA
ATGTGTCTGAATCTAGAGCTAAGGATTGTGCTTCTTTTTGGGGTATATCCTGTCATTCTCTCCCCCTCTC

CTACTTGGGAGTTCCTCTCGGTGGCAACCCAAAATCCAGACTTTTTTGGACCAATGTTGAAGAAAAGATC
CAAAAGAAGCTCAATAATTGGAAATATGCTCAGATTTCCAAGGGAGGAAGACTCACTTTAATTAAGTCAA
CCCTTAGCAGCCTTCCTATATACCAACTATCTGTTTTCCAAGCCCCTTCCCTGACATGTAAAAACATTGA
AAAATTCTGGAGAAATTTCCTATGGAAAGGTAACAACAGATCAGAAGGGTCTCACTTAATTAACTGGAAT
AAAGTCACGAAATCTAAAGAGGAGGGTGGTCTGGGTATCTCAAGACTTCATGTGACTAATAAAGCCCTCC
TAACAAAATGGCTCTGGCGTTATCTCTCAGAACCAACAGCTCTTTGGAGGAAACTGATTCAATGCAAATA
TAAAGGAAAATACCCAGGAGATATTCCATCAAACATCTCTTCTAGTACTCCTAAAGCCCCATGGAGATCT
ATAATTGACAATATTGACTGGTTCAGGAGCAAGCAAAGTTGGGATCTAAATAATGGAGATCAAATCTCCT
TCTGGTACTCTAATTGGTCTCTAGAAGGTTGTCTCTCAACTGCTTATCCGAGACTTTTTGCTCTTTCGCT
TGACAAAGAAATCACAGTTAAAGATGCATGGAACACACTCGATAGCCAATGGAACATAAGATTCAGAAGA
GAGCTGAACGATAGAGAAAGAAATACATGGGCAAAAATTTTAGAGATTCTTCCTTCTCCGAGATTTAGTA
GAGGTTCAAGCAAACCTACCTGGATCCCAGACAGCAAAAAATTCCTTTTCCATTGCATCTGCTAAAGTCTT
GATCTCTCGGCAGCTGAATCCATCCTCAGAGGACCCTCAATCAAAAATTCTCGAGAATATTTGGAAGTCC
ACCATTCCAATGAAAATCAAATTTTTCATGTGGTGCCTGATTCAGAGAAGAATAAATACGATGGAGGTTG
TTCAACAAAGAAGGCCCAATATTTCCCTGCAACCCAACTGGTGTGTTCTTTGCAAGAAGGATAGTGAATC
AGGAACCCACCTCTTTCTCCATTGCGAAGCCGTGAAGCCTTTATGGTTCTTTCTCCAGAATACTTTCAGA
TTAATTCCCAGCACCGCAGATCTGGAGGAAGTGTTTTCCTTCTTCCGGTCCCCAACTTGCTTCAGCCCGA
ATCGCAAAGTCGTGTTTTGTGGAATAATAGCCATTTTTTGGGTAATTTGGTGTGAAAGAAATACTAGAAT
TTTTGGATCCCCTAGCTCTCATAAAACATCTGCCAATATGTGGGAGGATTGCAAATTCCTTATAGGCAAT
TGGTGCACTAGGGACTCTTACTTTAAAAATTATTCAGCTGCTACAATTGCTTTAAACCTTAGCAACTTCT
GTACTTAACTCTTGGGCTTTTCTCTAGCCCTTTATTAATAAATTTGTGCGTCTAGATTGGTCTTAGGCTT
TTCCCTAAGTCCTATCTAGCTGTTTCCTTTCAAAAAAAAAAGAAACTAAGGCATTATTAAACAAGAGAAA
GAATTAATTTATAGATAAAATCAATTCTACAGTTTGAACCTTATCACACATATTAGAAAATGTGAGTGCG
TAGTATTTGAGCATCTTGAGGTTGCTTGCCTTAAATTGTTGAGACATAATAATGTCATATACATTTACTT
AGATAAAAATTATGTATTCTAACCTCAACATTTATATCACGAAAGTGAACAGACGTACAACAAAGGCCTT
GAGAGGTAACTCGCCTTAAATTAAAGTTAAAATATGAGATATTTATCGATTCAAATAAACTGTGAACCTT
TTAGTTCCAGTTACTCTATAAATAAACTTTTATGTTGCTTATTCTTGATATGCTCGTACTGAGTTAAAGT
TAGTTCAACAACATCTAAATTCATCTATTCCAATGTCATTCATTGCATTCCATTCCGAATCGATAGATTT
TATCACCTAAATTTGGATCAAACATTTAATCGAATTATACACCAAGAGTTAATACTAGTGGCCAGTCCGA
GTCGAATACAAGGAGCGCGTAAATTTCTCTAGCAAATTATTTTCAACCCGAAGTAACAAAACGAGGGGGT
TGATTGAATTAAAAATAAATTGTGAAAAATAAATTAAAAGTGTAAATCCTTTTTAGAAAACTTAGCTTGG
GTTTTGTAGATCTCCCAAAACATTATATTTGAACGTTAAGTTCTCATTATTGAATTTAACAACCGATGCT
CCTAAGCAATTTGAATAGAATAGCTAACCAAATTGTCCTATGCATCAAATTAATGCAAATTAAACCGATG
AAAGGTATACAAAGTCCAATTTACGCTAATTGCATTACAAATAAGGGTTAAATTAGGTTGAAAGCCTAAT
TTTCATGCACCTAATTAAACATTCATCAAAGTTCAATAGTAAGTCGTTTGGAAAAATACAAGATCTAATG
GATTTTTACAAGCTAACCTAACTTATTTCTTCTAATTGAATTACATACAACTTACTAAGAGAATTGCACA
TGATAAAAAAGCATTCGCCATCAAGTTCCCATGCATCTAACTATATGAACATGATCATTAAAGACAAAAT
TTGACTATCAATGTATTACAGTCAATTCAAATCTCACGAATTCATGAATTAAAGGGAGATACTACAAACT
GCAGTTAAGAAAAGAGTACCTTAACCTCTCAACAAAATGTTCATTGATGCAGAGATGTTAGGAAACTAAA
ATTAATAAAGACATCAGGAAATAGAGAGAAAACTAGTGGAATAAAGCAGGAATCATAAATGAAAAAACCT
AGGCGACAAAAAATATTTTTGTAAAAACCACACTAGTTCTTCTTTTCGAACACAAATAGTAAAGATAAAT
CAAGCAATATACATCTACGCTTCCTTACTTATAAGCATTATATCGACTCTATTTAAGGCGAGTAACCTCT
CAACGCCTAACAACCACATTTGTTTTCCTGTCGGGACACAAATGATTAGAGTTAAAATACTAGAATGGAG
TTTGGATAAACTCCATTTTGAGCAGTTTTAGCTATGTCCTTGTAACTTACTCTCTAGAATAGTTGGTGAC
TAACGCTGGCGAAGCGATTGAGTTTAACTTAGCTAAACACGTGAGACTTATCAACAAAAGAATCCTTATC
AAGTACAAAACATAAACTTAAACTACTTAAAACACATTAACAAGATGAATTGTAAAGAAATGGAAGAGAG
AGATAGGATAAAAGTGCATTATATTGATAATGTTTCCCTTTCGACCATGAAATGAAGAACATTCATACAA
TACATCATAGAGTATACAAAAATGGAAAGAATAAAAATGTTTTTAGCCAAAGAATGCAAGCTGCAGATTG
CATTGGTTTACATTCCTTGGCTCTTTTACATTTTCTTTTTTACATTTAGAAAGAAACATTATTAGATAGA
CAAATACTTTTCCGGTGGGGAGAGAGTTTTATCAATTTCGGCCATCATCACTACTTTTCTTTCATCGACT
CCTCAACTAATAATTTTGGTCTTCCATTTATTTTCTTCTTCATTATTCTCTGCAGTATCTCTTCTCCATA
TCAACATCTTCTTATTTCTTCGTTGAGGTCAAATATTTCATCCCTCACCATTTTTACATTTTCCATCTGA
TCATTTCAATACTTATAAACTGGGTAATTTGTGTAGATTTTTTTAGATAGTTTTTACTTGGTGATCGGTA
TGTGTTCACTCCTACTTTTCTGTTAGTCAAAATCTTCAAAATTATTATAGTTCATTCCTCGTGGGTTTTT
AGCTCTTCAAGTATTTTTGTAGGTTTTGATCCTTTTCAAGATCAAGAACAAAGGATAATAATGGACATCC
TTATTTCTGTCATTGCAAAAATTGCTGAATACACTGTTGAGCCCTTGGACGCCAATTTGGTTATTTATTT
TTCATTCGTTCCAACTTTCAAAAACTTAAGACTCAAGTAGAAAAGCTGAAGATTACAAAAGAATCTGTGA
AACACAAGATCCATGCTGCAAGAAGAAATGCTGAAGACATAAAACCTGCCGTTGAGGAAAGGTTGAAAAA
GGTTGATGACTTTGTTCGAGAATCTGACGAGATATTAGCCCACGAAGGTGGACATGGTAGACTCTGTTCC
ACCTATTTGGTCCAACGACACAAGTTAAATAGAAAAGCAAGCAAAATGGTAGATGAGGTTCTTGAGATGA

AAAATGAGGGGGAAAGTTTTGATACGGTATCCTATAAAAGTGTTATCTCATCGGTTGATTGTTCACCGTC
AAAAGTACCTGACTTTCTTGACTTTGAGTCAATTGTGGAACAAATCATGGATGCATTCTCTGATGATAAT
ATTCATAGGATTGGAGTGTACGGGATGGGGGGTGTTTGCAAAACAATGCTAGTGAAGGAAATTTTAAGAA
AAATTGTGGAGAGTAAGAAGCCTTGTGATGAGGTGGTACCATCCACGATCAGCCAAACACCAGATTTTAA
AAGTATTCAAGGACAACTAGCTGACAAGCTAGGTTTGAAATTCGAACAAGAAACAATAGAAGGAAGGGCT
CGTATTTTACAAAAGAGGTTGAAGATGGAGAGAAGTATCCTAGTTGTCTTGGATGATGTCTGGGAGTATA
TTGATTTGGAAACAATAGGAATTCCAGGTGTTGAAGATCATACGGGATGCAAGATCTTGCTTACCTCTAG
GAATAAACATTTGATCTCAAATCAAATGTGCACAAATAAATTTTTTGAGATAAAAGTTTTAGGAGAGGAT
GAGTAATGGAATTTATTTAAGGCAATGGCAGGTGAAATTGTGGAAGCAAGTGATTTGAAGCCTATAGCCA
TTCAAATTGTTAGAGAATGTGCAGGTTTGCCTATTGCTATTACTACTGTTGCTAAGGCATTACGAAATAA
ACCTTCTGACATTTGGATTGATGCCTTAGATCAACTTAAAAGTGTTGATGTGGGTATGGCAAACATTGGA
CAAATGGACAAGAAAGTGTATTTGTCACTAAAATGGAGTTACGATAGCTTGGGATATGAAGAGGTGAAGT
TATTATTCTTGTTATGTAGCATATGTTTCCAGAAGACTTTAACATTAACATGGAAGAGTTGCATGTTTAT
GCAATGAGCATGGGTTTCTTACATGGTGTTGATACTGTGGTAAAAGACGACGTAGGATTAAAAAATTGGT
TGATGATCTTATATCTTCTTCTTTTCTTCAACAATATTCTGAGTATGGGTGCAATTATGTGAAAATGCAT
GATATGGTTCGTGATGTAGCCCTATTAATTGCATCTAAGAATGATCACATACGTACATTGAGCTATGTGA
AAAGATGGAATGAAGAATGGGAAGAAGAGAGACTATCGGGTAATCACACTGTAGTGTCCATTCATGGTTT
ACATTATCCTCTTCCGAAGTTAATGTTACCCAAAGTTCATTTATTAAGGTTAGAGGGATAATTAAGGTTA
GAGGGACAATGGTTGAATAATAAATATGTATCGGTGGTAGAAACATTTTTTGAAGAAATGCAAGAGCTCA
ATGGTTTAGAATTAAAAATGGTGAAAATATCCTTATCGCCACCATCTCCTTACACCTTTGCAAACATTAG
ATTACTACGTTTACATGAGTGTGAATTAGGGAGCATTGATATAATTGGTGAACTAAAAAAGCTTGAAGTT
CTTGATTTTAGTGAATCTAACATCACCCAAATTCCTTCGACCATGAGCCAATTGACCAAGCTGAAAGTGT
TAAATTTATCTTCTTGTTACGCCCTTTAAATAATTCCACCAAATATTCTTTCAAAGTTGACAAAACAAGA
AGAATTAAGTCTGGAAACTTTTGATAGATGGGAAGGAGAAGAATATGAAGGAAGGAAAAATGCTAGCCTT
TCTGAGCTCCGGTACTTGCCACACCTTTATGCTTTAAAATTAACCATTCAAGATGAAGAGATTATGCCCA
AAGACTTGTTTTCAAGGGAGTTGAATCTTAAAAAATTCAACATTGTTATTGGTTTTCAGATAGGAAGAAT
ATATTATTTTGAAAATAAGATAAACTCCATTAGAATCAGGAAGGAGCTTGGATGATTGGATAAAAAATCT
GTTAAAGAGATCAGAAAAAGTGCATTTGGAAGGATCATTTTGTTGGAAGGTTCTCCATTCAGAATTGGTA
AATAAATATGACTTTGTACATCTGAAGTATCTGGACCTTTATGATAATTCAGAGTTTCAACATTTTGTCC
ATGAAAATAAAAAGCCATTGCAAAAATGCTTATCCAAATTGGAGTACTTAATTCTAAGGAACTCGGAAAA
TTTGGAGAGCATAATTCATGGTTATCATGGCGAATCTACTTTCAACAATTTGAAGAATGTAATCATACGG
AATTGCAATAAATTAAAAACTCTCTTTTTTAACTGCACGTTGGATGACATTTTGAATCTAGAGGAAATTG
TAATTAATTCTTGTGAGAAAATAGAAGTGACGATCATTGTGAAGGAAAATGAGGAGACAACCAACCACAT
TGAGTTAACTCATTTAAAGTACTTGTATCTAATCAGTTTACCACAACTTCATAAATTTTGCTCCAAAAGT
GAGAAATGTGGACAATTAACCGTGGAAACAAGCACAAGCAATACCATCAGCATTGGTGAATCCCTTTTCA
GTGAAGAGGTAAACTCTATCAACACATTTTTCTATGTTTCTATATACATGTCTAAATTTAATTACTTTAA
TTTTTTGTTCTTTTTATTGTTCCAACACAAGTTCCTTCTAACCTTTGTGGTTAGTAATGATATAAAGGAT
CAAATTCTATCCTTATTTGTTTTAGAAGGCTCTTCTCTCATTGAGAAACTACTGAAGTATATATAAATGC
ATGCTAGGTGATGTTCTAGGAAAATTAAAAGAAATTTATTAGATTTTTCCTATCCAAGGAAGCTCATTTG
AAGTAATCATGATTTTACACAATTTGTTATGGTAATTTTAAAACACTATATACATTATATGTTGTTAATT
GATGATATATGGTTAATTACGTTGTCTCAAAATTTGTAGGCATCGCTTCCTAATTTGGAGAAGTTAAAAA
TTTGTAGTGCGAAGAATTTGAAGATGATATGGAGCAATAATGTACTCATTCCTAATTTCTTTTCCAAACT
CAAGGAAGTAGACCAAGGAAACCTCTGCAAGCAATTTGATGAAGCTGAAATCCACTCTGTTCTAAGATCT
TTCATGTTCTAGTGATATGTAACAGTTGTAACCATAGTTGTAGAAAGAGCACTAAGCTAAAGTAACATGG
AAAAAGAACAAGCAACACAACAACGTTTGCCTAGGATAGAAGATTGTATTAATCACAAATACAATGACTG
GAGGTACAACACTCTGCACATTTATTATCGTACTGTCCTAGAACTATTGCAACAGAACTAAATATGTAAG
AACACAATAGCCATAACTCATAATAAGGATAAAATATCTCCCTCTTAATCTCAGATCTATAGATGATGTA
TTCAACTCAAGCCCCGAACGACTTGATGATTCTTCTATCGGTCTTTCGTATTATTTTTTATGAAGAACTG
CCAATTATTTTTTATGAAGAACTGCCAAGAGAAACTCACCGTCAAAAAGTTTTTATACTTTTTCTTCGCA
TAACTTCTCATACAGGATGTTACCAATATATAAGGAATAAATCCTAACCACCCGTGAGAATATTACAAAT
ATATAAGGAATAAATCCTAACCACATGTGGAACCCTTGAAAAGAAGATCTTAACATATTCTTTACCAACT
CCATAATCAATTAATTAATTAATTAATTTGCTAACCAATTAGTTAATTAATTTGCTCGTGATAAAATAAA
GTAGGTGGGACCATTTACTAACACTTCACAAACAGTAAGAGTCATGTGCTCGACGGTTAAAATATGGAAT
TTTTTAAAGCATCATGGAACTTGATCAAGGGGGAAGAAAAAATATTTTCAATGATTTCCACAAAAATGGT
ATCATTAATAGAGCTGTAAGTGAGACCTTTATTGCCCTCATTGCTAAAAAACAAAAATGCTCCCTTCCTT
TGGACTATAGACCGATCAGTCTTAGAACGACTCTTTATAAGCTCATTGTTAAAGTTCTTGTAGAGAGGCT
AAAGCTCACTCTTCCTGCCACAATCTCAGATAACCAATTGGCTTTTGTCAGTGGTAGACAAATTCCCGAT
GCCATCTTGATAGTGAACGAAGCTATCTATTATTGGAGGACTAAAAAAGTAAAATGGTTTGTTATCAAGC
TCGATATTGAGAAGACTTTCGACAAGATAAATTGGAGCTTCATTGATTATATGCTCATGAAAAAAAAACT
ATAGATGGAGACATTGGACAGAATCTTACATAAACAGTGTCAATTATTTAGTTATTATCAATGGCAGACC

TAGAGAAAAAATAAGTCCAACTAGAAGTATTCGGCAAGGAGATCCCATCTCTCCTTTTATCTTTGTCTTA
TTGATGGACTACCTTAGCTGGCTGCTTAAATTTCTTGAGATAAGTAACAAAATCAAAGGGGAAAGTATCA
ATGAAAAATAAAATCTAACTCATCTTCTCTTTGCAGATGACATCCTTCCATTTGTTGAAGATCACGATAA
GTCCATTGAAAACATTAAATGTGTCATACAGCTTTTCGAACTCGCATCTGGCCTCAATATTAACCTCAAC
AAATCCACGATTTCTCCTATAAAAATGGACACCAACAGATTGAGCTTTGTGGCTGCACAGTAGGGCATAA
ACACACATTTCCTTCCTTTAAATTATCTTGGAGTTCCTTTGGGAGGCAAACCTCTTTCAAAAGAATTTTG
GGCGAATATCTTTGATAAGATCCATAAGAAGTTCAACTACTGGAAGTATACAAATTTATCCAAAGAAGGC
AAAATTACTCTAATAAATTCTACTCTTTACAATCTACCCACCTACCAGCTATCTACCTTTAAGGCCCCAG
TCTCCTTATACAAAGACATAGAGAAATCATGGAGAAGCTTCTTCTAGAACCATTCGGTAGACAAACACAA
TCTTCACTTGATCAAATGGTCGACCATGACTTCTCCCAAAGAATTTGGTGGGTTGGGTATCAACCGCCTA
AGAGATACATATGTTGCGCTTATTTGTAAATGGTTGTGGAGGTACAATGTGGAAAAGCATTCTCTATGGA
AACAGGTGATATTGGCTAAATATGATCAAGATTCTTGTGGTGGTATCCCTTCAAAAAGCAAATTTAGCAG
AAGCAAGGCCCCTTGGCACTCAATAATTAAAATTGTTGACTNNNNNNNNNNNNNNNNNNNNNNNNNNNNN
NNNNNNNNNNNNNNNNNNNNNNNNNNNNNNNNNNNNNNNNNNNNNNNNNNNNNNNNNNNNNNNNNNNNNN
NNNNNNNNNNNNNNNNNNNNNNNNNNNNNNNNNNNNNNNNNNNNNNNNNNNNNNNNNNNNNNNNNNNNNN
NNNNNNNNNNNNNNNNNNNNNNNNNNNNNNNNNNNNNNNNNNNNNNNNNNNNNNNNNNNNNNNNNNNNNN
NNNNNNNNNNNNNNNNNNNNNNNNNNNNNNNNNNNNNNNNNNNNNNNNNNNNNNNNNNNNNNNNNNNNNN
NNNNNNNNNNNNNNNNNNNNNNNNNNNNNNNNNNNNNNNNNNNNNNNNNNNNNNNNNNNNNNNNNNNNNN
NNNNNNNNNNNNNNNNNNNNNNNNNNNNNNNNNNNNNNNNNNNNNNNNNNNNNNNNNNNNNNNNNNNNNN
NNNNNNNNNNNNNNNNNNNNNNNNNNNNNNNNNNNNNNNNNNNNNNNNNNNNNNNNNNNNNNNNNNNNNN
NNNNNNNNNNNNNNNNNNNNNNNNNNNNNNNNNNNNNNNNNNNNNNNNNNNNNNNNNNNNNNNNNNNNNN
NNNNNNNNNNNNNNNNNNNNNNNNNNNNNNNNNNNNNNNNNNNNNNNNNNNNNNNNNNNNNNNNNNNNNN
NNNNNNNNNNNNNNNNNNNNNNNNNNNNNNNNNNNNNNNNNNNNNNNNNNNNNNNNNNNNNNNNNNNNNN
NNNNNNNNNNNNNNNNNNNNNNNNNNNNNNNNNNNNNNNNNNNNNNNNNNNNNNNNNNNNNNNNNNNNNN
NNNNNNNNNNNNNNNNNNNNNNNNNNNNNNNNNNNNNNNNNNNNNNNNNNNNNNNNNNNNNNNNNNNNNN
NNNNNNNNNNNNNNNNNNNNNNNNNNNNNNNNNNNNNNNNNNNNNNNNNNNNNNNNNNNNNNNNNNNNNN
NNNNNNNNNNNNNNNNNNNNNNNNNNNNNNNNNNNNNNNNNNNNNNNNNNNNNNNNNNNNNNNNNNNNNN
NNNNNNNNNNNNNNNNNNNNNNNNNNNNNNNNNNNNNNNNNNNNNNNNNNNNNNNNNNNNNNNNNNNNNN
NNNNNNNNNNNNNNNNNNNNNNNNNNNNNNNNNNNNNNNNNNNNNNNNNNNNNNNNNNNNNNNNNNNNNN
NNNNNNNNNNNNNNNNNNNNNNNNNNNNNNNNNNNNNNNNNNNNNNNNNNNNNNNNNNNNNNNNNNNNNN
NNNNNNNNNNNNNNNNNNNNNNNNNNNNNNNNNNNNNNNNNNNNNNNNNNNNNNNNNNNNNNNNNNNNNN
NNNNNNNNNNNNNNNNNNNNNNNNNNNNNNNNNNNNNNNNNNNNNNNNNNNNNNNNNNNNNNNNNNNNNN
NNNNNNNNNNNNNNNNNNNNNNNNNNNNNNNNNNNNNNNNNNNNNNNNNNNNNNNNNNNNNNNNNNNNNN
NNNNNNNNNNNNNNNNNNNNNNNNNNNNNNNNNNNNNNNNNNNNNNNNNNNNNNNNNNNNNNNNNNNNNN
NNNNNNNNNNNNNNNNNNNNNNNNNNNNNNNNNNNNNNNNNNNNNNNNNNNNNNNNNNNNNNNNNNNNNN
NNNNNNNNNNNNNNNNNNNNNNNNNNNNNNNNNNNNNNNNNNNNNNNNNNNNNNNNNNNNNNNNNNNNNN
NNNNNNNNNNNNNNNNNNNNNNNNNNNNNNNNNNNNNNNNNNNNNNNNNNNNNNNNNNNNNNNNNNNNNN
NNNNNNNNNNNNNNNNNNNNNNNNNNNNNNNNNNNNNNNNNNNNNNNNNNNNNNNNNNNNNNNNNNNNNN
NNNNNNNNNNNNNNNNNNNNNNNNNNNNNNNNNNNNNNNNNNNNNNNNNNNNNNNNNNNNNNNNNNNNNN
NNNNNNNNNNNNNNNNNNNNNNNNNNNNNNNNNNNNNNNNNNNNNNNNNNNNNNNNNNNNNNNNNNNNNN
NNNNNNNNNNNNNNNNNNNNNNNNNNNNNNNNNNNNNNNNNNNNNNNNNNNNNNNNNNNNNNNNNNNNNN
NNNNNNNNNNNNNNNNNNNNNNNNNNNNNNNNNNNNNNNNNNNNNNNNNNNNNNNNNNNNNNNNNNNNNN
NNNNNNNNNNNNNNNNNNNNNNNNNNNNNNNNNNNNNNNNNNNNNNNNNNNNNNNNNNNNNNNNNNNNNN
NNNNNNNNNNNNNNNNNNNNNNNNNNNNNNNNNNNNNNNNNNNNNNNNNNNNNNNNNNNNNNNNNNNNNN
NNNNNNNNNNNNNNNNNNNNNNNNNNNNNNNNNNNNNCAACCACAAATTAGACACACTCTATACAAAAAT
ACATTTAACAAACAAAAATCTATTCCAAAAGAAAATCCTAAACTAATTTTCATAAAAAAAAACCCTAAAA
CATAAACTTAAACTAAATAAATTTTCATTTTCCACTTACCTAAAGTGGCAGATGGCGGCAAGGGACAACG
GCGAGGGAGGGCGGCGCAGAGGACGGCGGAACAACGGCGGAAGGGCGACGGAAATCGCGACGACGATCTC
GGCTTCTCCTTTCTTTTCCCCTCTCGGTTTCGGTTCTGTAAAATACAGAACTGAAACGATTTTAAAGGGG
ATTTCCCAACGCAGTGACGTGGCGTCGGGAAATCCCTCAAAATGCATCGGGAAAAGGGAAATTTCCGACG
CTCATTAAACGTTTTTCCCGACGTTTCATGCGGTGTCGGGAAAAACCCCTATTCCCGACGCCGCTCTCGA
CGCACTGTTCACGGCGTCGGGAATAGTTCGTTTTTTAAAATTAATTTACCCTTTTCCCGACGTATTCTTG
CCGACGCCTTCGTGGTGCGTCGGGAAAGATTGTTTTCTCGACGCATCTCCCGACGTCGTCCCTTATTCCT
GACGTTCTTTATGCCGACGTCCTTTTCGGCGTCGGGAATGCTCCATTTTCTTGTAGTGTACTGACCCAAC
CGTATCTTTTATAAAAAAAACCTACTGAAGAATATAAATGCATGTTAGGTGATGTTCCAGGTAAATTAAA
AGAAGTTTATTAGATTTTTCCTATCCAAGGAAGCTTATTGGAAGTAATAATTTTACACAATTTGTTATGG
TAATTTTACACAATTTCTTCCATTTGCCAAAATAATGATGTGTCCATGCAATATACAACTCTTTGCCCAC
GTACATATTTTTGTCGGGAAAACAAAGATGTGCTCAGCGATAAAACTACGCCGACATATCATATCTTTGC
CCACATTTTTTCTTACGTTGCTAGAAAAAAGTGTCGGTAGAAACCAAAATTCTTATAGTGATATGATAAC
AATCAACTATGCAAATTGTTATATGATAGTCATCGTAAAATTGCCTATGATACTAATTGTTGTTTGAATA
TAACAATCAATGATATTGATTGTCTTTTGATCGCTCCTAGATTACTATCGACTACCTTTTAAACTAATAT

TGATTGATATTTGAATTAAAATGCACAAGAACTAGAAAACCAGAACCAAACCCTAAAAGTATATCTCACT
TTCAATAAAATAATAATAACAAAAGACAAAAATTAACCATAATGTTATTTATGTAATTTAATAAAAGAAA
GGTAATTTATTAATTTTAGTTTAAAATTTAAAAAAAAAAACAAACAAAACATATCTCAAAAAAAAAAAAA
AAACAAAAACAAAAACAAAAGGCACACTCTTATTAAGCAAGTTGCCCCACCATTGAGATATATTTTCCCA
TAAGAAAAACTACGAAAAGGAAAGAAATCGAAGTGAAATGGAGAAGGAGAAGTTAAATTTGGTATTTTGA
AAGATAGATTAAAGTGACTATTTAATTTTCTTTCTTTCACTCATTAATTTTACATATAAGTATGGTCTTA
ATATACCAAAATTACGATTAAATATCTCTACCAAAAACTTATAAGATGTGTTTGAGGGAAGAAAAGAGTT
ATGGGGGAAAAGAATTATAATAATTCAATCATAATGTTATGATAATATGTGTTTGGAGAAAATGTTGTTA
TTGGTAGTGTTATAATAATGTCTTTGGGAAACAAATATGAATGGTAGTGTTATGATAGTATGTGTTTGGG
GGAGAGATTATTATAGTAGTGTTACAATAATATGTATTTAGAAAAGAGTTATGATTGTAGTAATTTAAAA
AACAATAATCGAATTTGAATTGGGTTATTTGGGTAGAGTAATGTAGAGCTGTAACAAAGAAAAAAAGAGA
AAAATATAGAGTTATTTGGAGATTATGATAACCCTAATACCCATTTATAATAACTCTTGGGCCAAACACG
GTTTGGCCCAATTTTCTAACCCTTTTTTTTCCTAAAACCCTAGCTACCCTAAACATACCAATAATTTTTA
AGTTGAAAACATAAGACGAAGACCGCACCCTTTGTGTTAATGTATCAATTTCATACCAATTATATGTTCT
ATATAATGCATTAAATGCACACGAGAGTGCTTTCTTCAATGGTTATTGAACACAAATTTACCGTAACATA
AAAATTGTAATACGTATTTAAAGTTTCAATTTAAAATTTGATGAAAAAGGAGATTTTCAAATTAGAAATA
CCACTTCCCAAGTTTAGCATTAAAATTTCCCCATATTAAATATCACATGCATTCTTCTTTGAAACAATTA
AAAAAAAAAAAAAAAAAGAAAAGAAGAAGAAGAAGAAGAAGAAGAAGAAAAGAAAAAGTAGGGTATAGAA
AATTTCAACTTTAATTAAAGGATCCAATTAAAGGTAGCTAATTAAGATGGGTAACGTATCACATATTACT
TTTACTTTTCTTCCTTTGATAACTCCAAACAATACACTTTTGCAATGTTCAAGTATTCCATCAAATTCTT
TTTTTCTTTCAAAGGTGTGCAACATGAGGACTTTCCATCCCAAGTGGTCATTCAATTCTTCATTTCAATA
ATTTTGACATTGTTGAAATTCCTAGGAGCATAAGCGAATTGACCCAGCTAAAAATGTTTAATTTATCTTC
TTGTTATCAACTCTAAGATATTCCACCAAATGCTCTTTCAAAGTTAAAGAAACTAGAGGAATTAAATCGG
TAAACTTTTGATATATGGGAAGGAGAAAAGGGGATATATGGAGGTTGGGGAAATTCTAATCTTTTCGTGT
TTCAGTAATTGTCTCACACCTTTTTGCTTCAAATTTAACCATTCAAGATGAAGAGAATATGCCAAACCTC
TTTGTTTTCAAGAGAGTTTAACTCAGAAAAGGTCAACATTATTGCTATTGCTTGGATGATTGGATTAATA
AACGTTGTTAAGAAGGTGAGTAAGTGTTTGTTGCAAAGGACCAATTTGTTTAAAGGATTTGTACTCACTA
ACTATTAGATATGGAAATGACTTCTCACATTTGAAGCATCTCTTCCTTTCATGGAAGTCTAATATTTCAA
CATTTTATGCCTGTAAAGGACAAGCCTTAAAATTGATCTTTAATTTCCCCATATTATATATCACATACAT
TCTTTTTCCAACAATAAAAAAGAAAAAGAAGGGTATAGGGAATTTGAACTTTAACTTTAATTAAAGGATC
CAACTAAATTTAGCTAATTAAGATGGGTCACATATTACTTTTACTTTTCTTGCTTTGATGACTCAAAAGT
TGCTGCTTCACTCCAATTAACAAATTAAAATGTTCCTCACGGGGGTTTATGCCAATATATGTTCTATTCA
TACATAAATATAAACAACAAATAATTTTGGTCTTCTATCTCATTATTATCTTCTTCATTTTTCTCTGCTC
CAGATCAACATCATCTTATTTCTTCCATCAGGTCACATATTTCTTCTCTGTCATCACTTTATGCAATATA
TTTATGGTTACATTTTCCATCACTTAAGCTTTCCATCTCATCGCTGTACTTATAAGCTGATTTGTGTTGA
TTTTTTTTTCTTCTTTTTTTGGATAGTTTTTACTCGTGTTCACTTCTACTTTTCTGTTAAGGCAAAAGCT
TCAAAATTATTATGGTTCAGTCCCTGCGGGTTTTTAGTTTTTAAATATTTTTGTAGGTTTTAATTATTTT
GAAGATCAAGAACAAGGCATAATAATGGACATCCTCATTTCAGTCACTGCAAAAATTGCTGAATACACTG
TTGAGCCAGTTGGACGCCAACTTGGTTATGTATTTTTCATTCATGCCAACTTTAAAAAACTCAAGACTCA
AGTAGAAATACTGAAAGACACAAAAGAATATGTGCAACAAAATATTCGTACTGCCAGAAGAAATGTAGAA
GACATAAAACCTGCAGTTGAAAAATGGTTGAAAAAGGTTGATGACATTGTTGGAAAATCTGAGGAGATAT
TAGCCTATGAAGGTGGACATGGTAGACTGTGTTCCACCGATTTGGTCCAACGACACAACTTAAGTAGAAA
AGCAAGCAAAATGGCCTATGAAGTTCTTGAGATGAACACCGAGGGGAAAAGTTTTGATACAGTATCCTAT
AAAATTGTTATCCCATCGGTTGATTGTTCACCGCCAAAAGTACCTGACTTTCTTGATTTTGACTCAAGAA
AGTCGATTGTGGAACAAATCATGGATGCACTCTCCGAAGATAATGTCCATAGGATCGGAGTGCACGGGAT
GGGGGGTGTCGGAAAAACAATGCTAGTGAAAGAAATTTTAAGAAAAATTGGGGAGAGTAAGAAGCTTTTT
GATGAGGTGGTAACATGTACGATCAGCCAAACACCAGATTTTAAAACTATTCAAGGACAACTAGCTGACA
AGCTAGGTTTGAAATTCCAACAAGAAACAATAGAAGGAAGGGCTCCTATTCTACGAAAGAGGTTGAAGAT
GGAGAGAAGTATCCTAGTTGTGCTGGATGATATCTGGGAGTACATTGATTTGGAAATAATAGGAATTCCA
AGTGTTGAAGATCATGCGGGATGCAAGATCTTGTTTACCTCTAGGAATAAACATTTGATCTCAAATGAAA
TGTGCGCCAATAAATTTTTTGAAATAAAAGTTTTAGGAGAAGATGAGTCATGGAATTTATTTAAGGCAAT
GGCAGGTGAAATTGTTGAGGCAAGTGATTTGAAGCCTATAGTCATTCAAATTGTTAGAGAATGTGCAGGT
TTGCCTATTGCTATTACTACTGTTGCTAGGGCATTACGAAATAAACCTTCCGACATTTGGAATGATGCCT
TAGATCAACTTAAAAGTGTTGATGTGGGTATGGCAAACATTGGAGAAATGGACAAGAAAGTGTATTTGTC
ACTAAAGTTGAGTTATGATTGCTTGGGATATGAAGAGGTCAAGTTACTATTCTTGCTATGCAGTATGTTT
CCAGAAGATTTTGACATTGATATGGAAGAGTTGCATGTATATGCCATCGGCATGGGATTCTTACATGGTG
TTGATACTGTCCTAAAAGGACGACGTAGGATCAAAAAATTGGTTGATGATCTTATATCTTCTTCGTTGCT
TCAACAATATTCTGAGTATGGGCGCAATTATGTGAAAATGCATGATATGGTTCGTGATGTAGCCCTATTA
ATTGCATCTAAGAATGATCACATACGTACATTGAGCTACGTGAAAAGACCGAATGAAGAATGGGAAGAAG
AGAGACTATCGGGTAATCATACCGCAGTATTCATTTATGGTTTACATTATCCTCTCCCGAAGTTAACGTT

ACCCAAAGTTCAATTATTAAGGTTTGTTGGACAATGGATGGAAGATAAGCGTGTGCCGGTGGTAGAAACT
TTGTTTGAAGAAATGAAAGAGCTCAAAGGTTTAGTATTAGAAAACGTGAATATATCCTTGATGCAACGAC
CATCTGATCTTTACTCCTTAGCAAACATTAGAGTATTACGTTTGCAAGAATGTGGATTAGAGAGCATAGA
TATGATTGGTGAATTAAAAAAACTTGAAATTCTTGATTTTAGTAAATCTAACATCACACAAATTCCTACA
ACCATGAGCCAATTGACACAACTCAAAGTGTTAAATTTATCTTCTTGTAATCAACTCAAGGTAATTCCAC
CAAATATTCTTTCAAAGTTGACAAAACTGGAAGAATTAAGTCTGGAAACTTTTGATAGATGGGAAGGAGA
AGAATGGTATGAAGGAAGGGAAAATGCTAGCCTTTCTGAACTCAAGTGCTTGCCACACCTTTATGCTTTA
AATTTAACCATTCAAGATGAAGAAATTATGCCGCCTAGCTCTTAAAGCTGATCTCAGCGAGTTATCTCTT
AAGGAGTCCCAATTCTGGTACCAAAGGGCCAAAAAGCTTTGGCTTAGGGAGGGAGATGAAAACTCCTCCT
TCTTTCATAGAATTTGCTCAGCAAGACAAAAGAGAAATTTTATTCAAGAAATTCAGGATGAAGAAGGCTT
GACTCAAAATACAAACAACGGTATTTCATCAGCTTTTATAAAATTCTTTTCAAGGATTTACAGAGGCCCC
ACTAAAAGCGATCCTGTCTTTATAGACAATCTAGATTGGAATCCGATTGAGCATTCTGAGGGGTTAAACC
TTTGTGTCCCTTTTCTGGAAGAAGAGATTAAAGGGGTCATAAACTCTTTAGAGGGTAAGAAGACCCCTGG
TCCAGATGGTTTCCCTATCTCCTTCTTCAAATCATATTGGCATCTTCTAAAAGAAGATATCATTGACATT
TTCAAGGACTTCTATGACAAAGGTGTTATCAACAAGAATGTGAACAATACATACATCGCTTTGATCCCAA
AAAAGAAGGATTATTGTAATCCTAAAGACTTCAGACCGATCAGCCTAACAACATCCATTTATAAGATCAT
TGCTAAAACCCTTTCAAACAGGTTAAAGACCTCCCTTCCTGATACCATCTCAGAAAACCAGCTAGCTTTT
GTCAAGAATCGCCAAATTACTGATGCTATCCTAATGGCAAATGAAGCTGTGGATTTCTGGAAGGTGAAGA
AGATTAAGGGTTTTATTTTGAAGCTTGACATTGAAAAGGCTTTCGACAACTTAAATTGGGATTTCATTGA
TTTTGTCCTAGAGAAAAAGAACTATCCTATCCTTTGGAGAAAATGGATAAGAGGATGCATAAGCAATGTC
ACATACTCAATTATTGTCAATGGAAGACCCCAAGGTCGTATTAAAGCCAACAGAGGTCTTAGACAAGGTG
ATCCTCTTTCTCCTTTCCTGTTTGTTGTTGCCATGGACTACCTTAGTCGTCTTTTATCTCATTTGGAAAA
TTCTGGTGCAATTAAAGGGGTCTCGTTCAGTAATAATTGTAACATATCCCATATCCTCTTTGCTGATGAC
ATTCTTCTTTTCATAGAAGATAATGATGTTTACCTGAATAACCTTCGAATGGCATTATCTCTGTTTGAAA
AAGCCTCGGGCCTTAAAATAAACTTATTGAAATCAGCTCTGGTGCCAATGAATGTGTCTGAATCTAGAGC
TAAGGATTGTGCTTCTTTTTGGGGTATATCCTGTCATTCTCTCCCCCTCTCCTACTTGGGAGTTCCTCTC
GGTGGCAACCCAAAATCCAGACTTTTTTGGACCAATGTTGAAGAAAAGATCCAAAAGAAGCTCAATAATT
GGAAATATGCTCAGATTTCCAAGGGAGGAAGACTCACTTTAATTAAGTCAACCCTTAGCAGCCTTCCTAT
ATACCAACTATCTGTTTTCCAAGCCCCTTCCCTGACATGTAAAAACATTGAAAAATTCTGGAGAAATTTC
CTATGGAAAGGTAACAACAGATCAGAAGGGTCTCACTTAATTAACTGGAATAAAGTCACGAAATCTAAAG
AGGAGGGTGGTCTGGGTATCTCAAGACTTCATGTGACTAATAAAGCCCTCCTAACAAAATGGCTCTGGCG
TTATCTCTCGGAACCTACAGCTCTTTGGAGGAAACTGATTCAATGCAAATATAAAGGAAAATACCCAGGA
GATATTCCATCAAACATCTCTTCTAGTACTCCTAAAGCCCCATGGAGATCTATAATTGACAATATTGACT
GGTTCAGGAGCAAGCAAAGTTGGGATCTAAATAATGGAGATCAAATCTCCTTCTGGTACTCTAATTGGTC
TCTAGAAGGTTGTCTCTCAACTGCTTATCCGAGACTTTTTGCTCTTTCGCTTGACAAAGAAATCACAGTT
AAAGATGCATGGAACACACTCGATAGCCATGGAACATAAGATTCAGAAAAGAGCTGAACGATAGAGAAAG
AAATACATGGGCAAAAATTTTAGAGATTCTTCCTTCTCCGAGATTTAGTAGAGGTTCAAGCAAACCTACC
TGGATCCCAGACAGCAAAAATTCCTTTTCCATTGCATCTGCTAAAGTCTTGATCTCTCGGCAGCTGAATC
CATCCTCAGAGGACCCTCAATCAAAAATTCTCGAGAATATTTGGAAGTCCACCATTCCAATGAAAATCAA
ATTTTTCATGTGGTGCCTGATTCAGAGAAGAATAAATACGATGGAGGTTGTTCAACAAAGAAGGCCCAAT
ATTTCCCTGCAACCCAACTGGTGTGTTCTTTGCAAGAAGGATAGTGAATCAGGAACCCACCTCTTTCTCC
ATTGCGAAGCCGTGAAGCCTTTATGGTTCTTTCTCCAGAATACTTTCAGATTAATTCCCAGCACCGCAGA
TCTGGAGGAAGTGTTTTCCTTCTTCCGGTCCCCAACTTGCTTCAGCCCGAATCGCAAAGTCGTGTTTTGT
GGAATAATAGCCATTTTTTGGGTAATTTGGTGTGAAAGAAATACTAGAATTTTTGGATCCCCTAGCTCTC
ATAAAACATCTGCCAATATGTGGGAGGATTGCAAATTCCTTATAGGCAATTGGTGCACTAGGGACTCTTA
CTTTAAAAATTATTCAGCTGCTACAATTGCTTTAAACCTTAGCAACTTCTGTACTTAACTCTTGGGCTTT
TCTCTAGCCCTTTATTAATAAATTTGTGCGTCTAGATTGGTCTTAGGCTTTTCCCTAAGTCCTATCTAGC
TGTTTCCTTTCAAAAAAAAGATGAAGAAATTATGCCAAAAGACTTGTTTTTAGCTGGGGAGTTGAATCTT
GAAAAATTCGTCATTAACATTGGTTGCCAACGTGACGGAAGATATATTTATGAAAACAACACCAGCTTCA
TTGGAATCAAGATGGAATCAGGAAGTTGCTTGGATGATTGGATAAAAATTTTGTTAAAAAGATCAGAAGA
AGTGCATTTGAAAGGATCAATTTGTTCAAAGATTCTTCACTCAGAATTGGTAGATGCAAATGACTTCGTA
CATCTGAAGTATCTCTACCTTTATGATGATTCAAAATTTCAACATTTTATCCATGAAAAGAATAAGCCTT
TGCGAAAATGCTTATCCAAATTGGAGTACTTAAATCTGAATAACTTGGGGAATTTGGAGAGTGTAATTCA
TGGTTATCATGGCGAATCTCCTTTGAACAATTTGAAGAATGTAATCATATCGAATTGCAATAAATTGAAA
ACTCTCTTTTTCAACTACAACTTGGATGACATTTTGAATCTTGAGCAACTTGAAGTTAATGTTTGTGAGA
AGATGGAAGTGATGATCACTGTGAAGGAAAACGAGGAGGCAACCAATCACATTGAGTTTACTCATTTAAA
GTCTTTATCTCTACGGTATTTATCACGACTTCAGAAATTTTGCTCTAAGATTGAGAAATTTGGACAATTA
AGTGAGGATAATTCAACAAACCCGAGAATAAGCACAGATAGCAATACCACCAACATTGGTGAATCATTTT
TCAGTGAAGAGGTAAATTCCAATTAACTCATTTTTCTATGTTTCTGTATTCTCATCTAAATTAATTAAAT
TATATTTGCGTTCTTTCTATCGTTCCTCTCACCCAAGTTCCTTCCAGCATTTGTAGTTAGTAATCATATC

AAGGATCAAATTCTATTCTTCTTTGTTTTAGAATGCTCTTCTCTTAACGTAGAGATGGCTCTTTCTTATT
TCATCCATTGATAATAATATATATCATTTTTAAACTACCTGAATTATATAAATGCATGGTAGGTGTTGTT
CCAGGAAAATTAAAAAGAAGTTTATTAGATATTTCCTATCTAAGAAAGCTCATCTGAAGTAATCATTTTA
CACCATTTGTTATGGTAATTTTGGAAGACTATATACATTATAAATTGTTAATTAGCATCAGTTGTGATGA
TGGTTAATTACATTGTCTCAAACTGAATTTTCAAATTACGCAAATTTGTAGGTATCGCTTCCTAATTTGG
AGAAGTTGAAAATTAGGAGCGCAACGAATTTGAAGATGATATGGAGCAATAATGTACTGGTTCCTAATTC
CTTTTCCAAACTGAAGGAAATAAACATTTATTCATGCAACAATCTTCAAAAAGTATTATTTTCTTCAAAT
ATGATGAACATTCTTACCTGCCTTAAAATCTTAATAATCGAAGATTGTAAATTGTTGGAAGGAATATTTG
AAGTGCAAGAGCCAATTAATATTGTTGAAGCAAGTCCCATCGTGCTCCAAAATTTGAATGAGTTGAAATT
ATATAATCTTCCAAACCTTGAATATGTGTGGAGCAAAAATCCTAGTGAGCTTCTGAGTTTGGAAAATATA
AAAAGTTTGACTATTGATGAATGTCCAAGACTTAGAAGAGAATATTCAGTCAAAATTCTCAAGCAACTTG
AAGCACTAAGCATTGATATCAAACAATTTGTGGAGGTTATATGGAAGAAAAAGTCGGCAGATTATGATAG
GTTGGAATCAAAGCAATTGGAAACTTCTTCTTCCAAGGTAAATATATATCCTTTACAACACATCATATTT
ATTTAATATTTCAAAAATTAAATTGCTATTCACTTATGTGCCAATATTTGTTAAATAATCACTTCATTAT
TTATTGTTTATTATGTTTGTCCTCGAAATCCTAGGGAAAATCTTGTGGAGTAAGACAAAAGGTTGCAAAT
ATTGTTATTGGAGGTTCAATTGTTTATGTGGAAAAAGAATGTTCATGTGGAAAAAGAATAAGCAATATAT
CTCTTAAATCTAAAGTTATGAATATAACCTTATGATTTGTTTCGTTTTTATCTAATTACATATATTTAAG
ATACTTTGGGCTAATTAATTAATTATAATTCTTGTGCATATAGAAGTTATTGCATTCATTTAATAAATTT
TGATCAGGTTGGGGTTTCTACAGGTGGGAGATAGTTCTAAATTGTTGCCGAATCTTAAAAAATTGAAGCT
ATATGGTTTTGTTGAGTATAACTCAACTCATTTGCCAATGGAAATGTTAGAAATCTTATACCAACTTGAA
GACTTTGAATTGGAAGGAGCATTTATTGAAGAAATTTTTCCCAGCAACATACTGATTCCAAGCTATATGG
TTTTACGGAGATTTGCTCTATCTAAACTACCCAAGCTTAAGCATTTGTGGGATGAAGAATTCTCACAAAA
CAATATTACCTCAGTTCTTCAAGATTTGCTTATTCTAAGCATTTCAGAATGTGGAAGATTGAGTAGTTTA
GTGCCATCATTAGTGTGTTTTACAAACTTGGTAGTTTTTGATGTGATAAAGTGTGATGGACTAACCCATT
TGCTGAATCCTTTGGTGGCTACAAAACTTGTGCATCTTGAACATTTGAGAATAGAAGAATGTAAAAGGAT
GAGTAGTGTAATTGAGAGAGGATCAGCTGAAGAAGATGGAAATGATGAAATTATCGTATTCAACAGCCTA
CAACTTTTAATCATTACTTCTTGTTCCAACCTAACAAGCTTTTATCGTGGGGGATGCATTATTAAATTTC
CATGTTTGGAAGAAGTATACATTCAAAAGTGTCCTGAAATGAAGGTCTTTTCGTTTGGAATCGTAAGCAC
ACCTCGTTTAAAATATGAAAATATTTGTTTAAAGAATGATGACGACGACGACGGCGACGACGACGATGAT
ACATTGCATCACCCAAAAGAATCCAAAGAGATGATGCTGGAAACTGATATGAATATCATCATTAGAAAAT
ATTGGGAGGACAATATCGATACCAGAATTCCAAATTTGTTTGAAGAACAGGTTAATATATTTAATTAACG
TTATCATCTCTTACGTAATTTTCCTTATTTGGTAATTAATGAATGTATTGATGCAGAATTTGGAGGAAAG
CCAATTTGAACATTCTTCTTCTTCGGATAAAGTAGAGGATCAATAAGGAATTATATTGAAATTCTTATGC
ACTACACTTTATATCATTTCATCCCCAAGGAAGGTCAGACTCTTGAAATCTCAATATCTTTATGAGAGAG
TATATAGAAAGCATTGGGTGTGGTTGAAAATGAGACAAGTTTGAGAGTAGAACTTTTTTCATCATATTAA
CATCACTCTCAAATATATGTTTTCAGTTATTCAAAATCTATTGTCCTGATAAGAAAATAATCTAATTTAG
AACTGTAAGCTCTAGCATTTAAATGTTAATTTTCAATAATTAAAAACATGTTTTTAGAGTGATTTTAAAC
TTGAGAAAAAAGTTTCGTGTATTGAATTTTTAATAAATGATATTGGATGATTGTTGTCAATGGATGTGCA
GGTTCTATTATTTCTGATTTGTAGGATTGTATCTTAAAAAAAGATATTTATGTTAAGATGAACTATCAAT
TTTTCGGTGATTTTACCAAAACTTATAAGTGTTTTTGTTCTTCTAATTAAATTCCATCATCGATTGTTAT
TTGCTGTAAAGAGATTCAGATCAATTGAAGTCGGAGGAAAACCAATTACATGCATCATCTTTTTTCACAC
AGAAATGAAAAAGTTCAAATACATCATTCCTTAGTTTAAAAGTACTTTAACTTCAGATCCATCTGTCTTT
CTCAAGCTCTGGAAGACTAGCATACCAAAAAAAATGTAAATTTTTTCTTTGATCCATTATTCATAATTGT
TTAAATACTGCAGATCGTCTCCAAAAAAAAAAAACTCGCAAAAAAATTGGTGCATTCTTTGCAAAGAAGC
CGATAAAGACATAAACCACCTGTTCATCTCCTGTCCATCTGCCCAAAAAATATGGGATAAAGGGGAAGAC
TTAACCAAATGGGAAATCGACTTCTAGGAAGCAGTCCCCCTTTTGCAAAAACATTTGCATTCTTTACAGA
AAAAGAGAGGAAAAAAAGCAATATTCTTCAATTTGGTGGATGTTTCTCTTTGGCTTATTTGGCTAGAAAG
CAACAATAGAATTTTCAAGAAGAAGGAAAACACACTCACTGAAATATGGGAAGATGTTAAAGCTCTCACT
GGCCTTTGGACGAGTGGATCCAATCTCTTCAGAGACTATTTTAGTAGTTCCATTGCTCTTAATATTTCTT
TCTGCTTTCATTTAATATGTTTTGACTGTGGGTTTCATTAGGCTTTCTTCTAGGCCCTTGTTTTGGGATT
CCTTTCTTGGTTTCTATACTTATTGTTTTATATTTAATGAAGCGGGAATGATGAGTGTGCTAAAAAGGTT
TCCACCTACTGGAAATGTCTTGGTGCATCTGTTGACTCATCTCATATCTTTTTCAAAAAAAAAAAAAAAT
ACTTTATACAAAAAAGGCTCAGTTTAATTTAGTTTTTCTCTTTCAAATTAAAATTAACAAATGTGATAGA
CTGATCCATTTACGTATGTCTTTGACAAACCTTTGTGTAACCTCTGAAAAGTTATTGCAATTGTAACTCT
CCAGCCCTGTTTTCTGTAACACCATTATAATATTAATATATTAGGGACTCGATGTGTACCATCTCTTGTA
GATGGACCAACACATTGATCCATGTGTTTCTTGCTCAGAAAAAAAAAAAACAACTGATCATCAAGGTTCAG
ATTATGTTGATGTCTCACACAATGGGCTTTGCCAGAACCTAGCACGTGGAAAGCTAATTGTCTGATGTTG
TATATGGGGATCCCCGTGATCTAGGAAAATCAGTGAGTTTGGTTGAATTGTTCACGATCGCATTGGACGA
GATAGGTTGTTTGGTTAGAAAGCAATTTCTCGTTAGTACGAAATTGAAACTCTTGAAGGCATGACCATTT
TTGGAAGGGTTGTCTAATGTCGTTATATTGATAATGATGGGGCATATGATTGTGGAGTTCGATTGCTTAG

ATGTGGTGGGAATTTGTAATACTTTAACGTGACCATACAAATATGGTTTATCTTTTGTGCAGGGCTATAG
ACTTAGGATTGGGAATATAATCAATGGAAGTTCAGACTTTTTTAGTCTTTTGCATTATATTGGTGGAGGT
TTAAGAATTGATATATATATACATATATATATATATGAATGAATCAGGTAATTAATTAATTGGTTTTGCT
ATCTATTTCAACCAAATAAATTATTTACAAAATGAAACTTAACATCAAAAACTGCTGCATTTTGGTATTT
GGATCGTTTCTGTTGGAAGCGTTTGATGAACACTCCAAATAAATAATCACCAAATAGAATATTTGATAGA
AAACAAAAACCTTTTTGAAAGACTTGTTGTGTGTATTCAACTCACACTTGGATGATTTAGATTACAACAT
TTCAATCCTATTTATAGGATTGTCATAAGAATAACTCATATCACAAATAAATACAACAAATTAAATCCTA
CATTTAACTAAGGTGAAACTCTTACTAAATTAACTCTAACATTTTTAACTCACATCCTTCTCTAACTTAC
ATAAATTAACTCACATATAACTTATTTATTTAAAACATGTTTAATTTTCAACGGTTTCTTGCATGATTAG
AATATGTGTTCTAATTTACTGTGGAATATGGAGGAAAAAACCAATTATATGATCACATCTTTCACACATG
AGGATAACTTTAAAGACTTGTGCAGAGACTGAAAATAAGCTGTAATAAAATGAAGAAAAGGGAAATGTTT
TGTTGCTACGATTATTATTTTTTTCTAAATTTCAATGTGTATTGCAATAACCCAGAAGCACTTTCATGGAC
ACATCAAATAGTACTGTTGCTAAGTTGTGGGAAGATATTTGCATGTTGACTTCTTGTTGCTGCTCCTTTA
GATCGAAGCTTTCAAAACCACTACCAGTCCTATCTCTGTCTCTCTCAATTTGGGTGCTTCTATGTAATGT
AATATTTCGGCCATTTATCTCTAGCTTGCTCTGTGTGTGACTCTATCCTTTTCCTGTTTATTTTGGATAT
CAACTGAAGGGATGGAGTGGAAGATGATGAGAGTGCTATGACTATGCCAACCTAGTTTAGTTGGGATTAT
TTGGTGTACCTACTGATCCACACTTCATTATATATGGAACATATGTACATATCAAGAACTGTTTAATAAA
CCGTACCATTTGGTTTTTCATTTTTTAAAATTAAGCTTATGAATAATTATCTTCTTTTGTTATCTACTTT
TATGATAAACCATATATGACTTTTTGTTTTGTTATCTACTTTTGCAATGTTTTAAAAAAATCAAGTTTTG
AGAACCATAATTGCAAATAACAATTATCTTCACCATGTTAATTCAATTTTGGCTTCAAAATCTAAAATTT
TAAAAAATAATTAAAGCCCAAGAATTAAGAATAAATTATCTTGCTTTGTAAGGAACACACAGGATGTTAC
ACAATTTGTTGTTGAGGATATCAAATTAGATTGATTCTATCGCCACGTGTTCGAGACGACGCAGAGCGGC
CGACGTCCTCAAAATGGGTTAGTTTCAAAACAAAATAGAAGTCACCACCAATCCTTTTTACGGTTTGATT
GGACACCGAAATAAAGTAAAATATTTATAAATAAAATCATAAAAAAAAATGGTATGCGAAAACTAGAGTTG
AGTTCGGGAGTTATTTGTGTATGAGGAAGGTATTAGCACCCCACAACAACCGTTTAGAAAACCGTGGCCA
AATTCCAAATCTTGAATTGAAAATATTCTTTAATTTCTTTATAAATTAATGTCTTATTTTACCCCTCATT
GCTCCGATATTTGAAGCAATGATCTCATAAAATAACTTTAACTTAGTTGGAGGTGTAACTTGACTCGGAT
GAACAAATGGCGGTTACTCTCTCTCGTCTCCCCTCACCTTCCGACCTTTCGTCTCCAACGCTGCCGCTTC
ATACCTGTGGACAGCATACATCCTATCAACTAAAAAATGATGATTACTACTCAGAAGCATTTAAGTTCGG
CAACCAATAATTTCAGCTGGAGCTTTGCATTTACTCTGCCGCCCAAAGGTAATCTCCTTAAAGTCTCTAC
TCTCCCTTTACTTTCTGTATTCTTCCATACCACCCTTCTCTTCCTGTTGTCCTCTTCTACACTTAAAGAA
TGGCTGGTATGAACCAACTTCCTCGTCACTGCTCTGTTGAGAAAAAAGATTCCGTGCTCTCCGTTGACAA
AAGGTCAAGAGAGTCAAACCTATTGATTACTGAAGTGGGTCCCTACAAATCCTTTTCCATAGCCATTACT
CTGGACTCCTTAGAATGGCTGAAAACGACCTTCAAAGCTCTACTGAACACACCAAGAACCACTCGATTCT
TTGTCGAAAAGAGGTATGTAGATTTCTGTTTGTGGGTCCAAAAAATACACAATAGAAGAGGATATATTGC
GGAGATCTACAGAGTTGATGATAGAGGTCGAAAATGTTGCATCCTTGTCCCGGAAGGCTTGGACAAAACA
GGTTGGGCCCTTTTCAACGACATGTTGACGTGCAAAAAAACATCAGACAAAAAGGAGATATCTACGAGAC
ACTACTACAACCAAGACAAAGGGAAAGAAAAAATCCAAAAGCCTTATGACTCCTCCACTGACTCAGAATC
CCCCAGGAAAACCTATGCTGAAGTTGTCTCAAGTTTTTCGAGCTCTGAGTCCGATTATTCTAAAGCCAAA
AGCACTTCCTCACTCAAGAGATTCTTACCGGCTCTGAAATCTGAAATTAGAAAAGAAGAAAGAAAGAATG
ACATTGATTGGGAAAAAACGATTATCCTGTCTAGGAGATGTTTTCATGATGACTGGGCTAAAATCATAGA
TCGTCTCAGAGAACAAACGGACAAAAAAGACTCGTGCTTTCGCTATGTTCCTTTCCATGCAGACAAGGCT
CTCCTCTTCATCAAAGACAAGGACTTAGCTAAACTGTTATGCAAAAATAATGGGTGGACTACCGTGGGTC
CTTTCTATGTGAAGTTTGAAAAGTGGTCAAAAAGTGTCCATGCAGATACAAAAGTGAATGGCAGAGTAGG
CACAAATCAAAAGAAGTGCTATGAACATGTGAGAAAACATTGGCAATTTGTTTGGTCTGCGGTTTAGTAG
GCACAAATCTTGTATAGACTATGACCCGGATCTATCTCTCTCTAAATTATAGAGCTTTGTTTTTTTAAAA
AATAAATAAATAAATAAGATATGGGTCGGTAGGTACATCAGAATATCTCCACTAGATGAACATCTTCGTA
GCACAATCATTATTCCCGTTTCATTAATAGATAAATGATAGGTACAGGAAGCAAGGAAATCAAAAACAAA
GTCTAGAGGGAAGTCCAAGGGAAACCCAATGTTAGTACAATTATACAAAAGGATTGATACTAAGAGCTAT
AGTGCTGGCCAAGTAGCTAGAAAAAAGAGGGGATCTACTTGTCCAGAGGCTCAAAAGAGCTTTGATGTCT
TTCCAAACCTCAGTGAGTGATTTTTCTTGTCGATTAAAAATTATGCTATTTCTTTCTAATCAAATAGACC
AAAGAGAGGGACAGCCTAAATTATAGAGCTTTGTTAAATTAAAGGATTCTATAGGTGAGCTAAAACACGT
CGTCTCCTGCTAAAAAAAACTATGAAAGAAAATTTGCAACTATTAATTGTTTTGTTTTAGATTTTTTTTT
TTAAAAAAAAATTGATGGACTATTAGGGAAATTATAATTGGCAAAAGAAATAGAAAAAAATGAGGGGCCA
AAGAGTTTTGGGATGAGTTTTGTTAAAAGAAAAAAGAAACCATAAACTAAACGTTAAAGTACAATCGCAC
TATCTATATATATAAAAAAAAAAAAAAAAAAAAAAAAGCCTTTTCTTCAACTACATTATGGTCTCGTTACTC
TTTGAATGGGTGATGGACAATGGCCTTGTCGAAGGTCTACCCGTCAAAATAATTTTTTATTTGTTCCTAT
ACTTACATTAGTTATAGTGGCAGTACGAGGTCGAACCACGTGAAACAGTTGCATTTTTTATTAAAAAAAT
ACTTAATAATGTTCCTAAGTAATGTAGCCAGAGGGGGTTAGTTTGTTGTTTGCAAGAAAGTATATAAGTG
CATAAAATTTAAACCAAGACAGATGAAATTAAATGTGGAAATATAATGGAATGAGAGAAATATTGAGAAT

TAGAGTAGTCTAGCGTAGATGGTACCTGGTGCACTCATAATACCACTAGTATAATGCACTACAAATTTTA
CAAATAAAATATCAATTCATTTTTATATCTCAATCAACTGTTAGAATCTTTATTTTACATATATTAAACT
CATCCAATGTTCTTCACCTTTTTCTTTCACAAAACTGTAATAACAATATACAACAAAAAGATATATATAT
CAATATGATTCTTCAAGAAAAAGGCAGAGAGGATGAGACTGTGTTTCCTGACATGTGCTTGGGTTGTACT
GATTTAATTACAAATCTATTCATATAGAATTTGGAGGAAAATTAAACCATCCTAATAAACTAATTGGACG
AGGCCGACTCTTCTACCAACATATTTGAAATTTAATATAAAGCGATAACATCTTTTCTAGTGGACATTTT
TGTAACACTCTTCTCATCCCTGCTTCATTAAGATGAATCATATAGAATGATAAAATCAAAAGAAAGGAGA
AGCAAGAACAAGAGAGAAAGCGTAAAAAACTTGAATGAACTTGAAATAGTATTAATTTTAAGTGGGTACA
ATATGAGATCAGCAATATTATTATTGTTCTATATATTATACAACATTTTTATGTTTTTATCTCTCTAATT
CTAAATAATAGTAATTTTGATAGGTGTTCTAAAACAAAGACTGCCCCTTAAATTTGTCACAAAAGTAAGT
TGTCCTATAATTATTTTTTGAATTCAAACTTCTAATCGAATCATCGACAAAATGTTAGAATTAGTGAATT
TTTATTTGGAGACTAAATTTTAAACTAACTTTTTGCCATCAAATTTTTATTATATGTTTGTATTTAATTT
ATGGTGTATAAAATGACATTGTTGGGGCCTAATTGGTTGAAATGATTGAATTGCATCTGTGAAAAGAGCC
CTTTTTGAAGAATCAACCACAACATCTGCCAGCTTTCTTTCTTAAGCTTTCAGCGATCTATGAAAATTAT
ATATGTCTTTAAAGTGCAAATTCTTCATCTGAACAATGATCCATCAAAGGATCAATACTGTGGATGTTAT
TTAAAAGAGTAATCCAAGAAGCTATCTCTGTCCAAACTAATGCATATCCTACAAAACCTCCAACCGAAGA
TCTAAATCACTTGTTCATTCATCGCACCAAGGCCCAAAGCTTATGAAAAAATTGCTATTGGAAACGGTGT
TGTTCTACCCCTGATAAGTACAAAAGAGTTATGTCTTACTCTTTGTGAGATTCAATGCAACAATGCCAAA
AACATCATAAAGTTCAATGCTGTAACGCCCCAAAAATTTGGGATGATTTAGTTTTAATTATCTTAAGTGT
AATTATTGAAATTTAGGGTGTTTTGAATTAATTGATTTATGAATATGTGATTTTTATTTGATATTAAAAC
AATATCTAATGGTGGTAGTGTTTGGGAGTTGTGTTTTAATTGGATGTATTTGAGGAAACTTGATTAATTA
TTTGTTGTTATGTAATTAATTAAGTTTTGAGGATAACATAATTATTTTATTGGGATAATAAAATTGGTAG
AAATATTTGTTTGAAGATAAAATTGATTGGGTTGAGGAGAGAGAAAATTTATTTATTGGTTAAAGATATT
GGTGATATTTAATTTGGGAAAAAGATTGATTCGTTGATTTTGAATTTAAAATGGAGATTTGTTAGGTTTA
AATGAAGAAAGAGATTAAGTGGTTTTATTTGAAATAAATTAGGGAAAAGATAAAAGAAAAATAATAATTA
TTATAATTATTAATTTTTCTTTAAAAGGCCATTGGGATCCGTGCACATATTAAACCATCATCTTCTTCAT
TGAGAAAGAGGAGCAAAACCCTAAATTTTTCCTTCTTCTCCAGCCGCCGCTGAAAGCACCGCCGTCGTCG
GCCTTCTCCGATCGTTCTTTCGCCGTATCCTTCCCCGCACCGTCGAAGTTTCCGTCAGCGTCCGTCGTCT
CCCATCGTCGAGCTAACCCGAGCCGACCCTCGTCGTGGCAGTCGTACGCCCAAGCATTCCGTTAGCCGCC
GTTGCAAGTCCAGCCGCTCGCACAGTCATGCCGTCGCATATCGGGCGCCGTCCGTCGAAGTCTGCGAAGC
CAAGCCGCGCCCCTTCTCCGCAACCCAAGCCGCACCCGCACCCAACCCGAACAGCCGTACCCGCGCCCGC
ATCCATCAGCTGAGTCGTCTGTGTGCGCAAGCGTTAGCCGACCCGCACGTCTGCGCCAGTTGCCGCGTGG
AGTTCAAGCCACGTCCGTGCCCGTTACCGCGTGAAGCCTGAGCCGCGCGCCCATGCCGAGCTGTACGCCT
GTGCCAACAGCCCCAGGGAGCCGCCTTCTGCCTTCCAGCCGAGCCCCAAGCCATTTTAACCACCTGATCA
TTTTTATCTCTTTCCACCTGTTTTTTTTTTTTTTTTTTGGTAAGTCTTGTTTGGGTTTTGGGTAATTCCCA
ACAAATCCTAGTTTTTGGACCCTAAATAATTTAATTTTTGATTAAATTAAATTATTTCTCTTAAAGGACA
GTTTGGACCAAGTGAAATTGGAGTGTGGGATTTCTCTAGCTAAGGGCTAATTTATTGCACCATCGACCTC
GGGTAATTATTTCAACTGAATTCTTGTGTCCTTGATCGTTTGATGGTTAAGTTATGTAAATTGGTGTTTT
CTAACTATTTAGAACTTCGCTGCTTGGAAAACGTGATTTATTGTTAGAATTCAATTGAGCAAATCTCCAG
GTAAAAGATTATACTACTAGACCTATGAGTGGAATTAAGAGATCGCATATATTTTTAGCTGTGCATATTG
ATAACTATATTACATAAAGACATGGCAATTAATAATGATATGATATGACATGACGATATGATATGACATG
ATGATATGATATGACATGACGATATGATATGACATGACGACATAATGATGATATGTTACGTGCATGCTAT
GGTTAGGGTGTATGTTAGCTTATCCTATTAGAGTCGACCTGCATGGGTGTCCTTCATGATCACCACTTTT
TTAGGATTGCGTAGTCCGACGGGACCGCCAGTCTGGCATTAACATAGACATGATTCGAGTGAATCGACGG
GATCGCTCGCAGCTCGATTGTCTTAGTGTTTCCTCCGGGTACGCTACAGATCAGTTTGTCCTAGGTGTTC
CTTTGGGATCACCGAAGACTAGTTATGTTCCTCTGGGATCACATGTTGCATGTGTTCGGGAACGTGCTAG
TTCTTGGGTAGCACTTTTTAGGACTCTAATAGGAAGTTAACAGGCACCTAGCGGGACTAGTAGTAGGTCC
CTTCCTGAGTATATCTTTATATTCACTCTTTCTATTTCTATTTTTCAGGCAGAGGCAGCGAGATAAGAGC
AAAGACAAGCTGGCGAGCAACCAGAAGTGACCGTGGCGAGCCATAGGGATCTTTTGCTTCCGCTTTATGT
TATGTCGGCTTTCAGTATTCACATTTTTTTTATTTTAATCTTTTATTCCTATTTTATTTCCTTAAAACTG
GACAGGGCCCGAGTTAGGATTTTGTTTTAGTTATTTCCGTACATTTTATTTATGATTTTAATAAAAGTTT
GCAGTTTGTCCATTTTCTGTTTAATTTAAATTATTTATTTTCCTTTTTATTTCTTTTATGTTAAAGTAAA
GTCCTTAACTTAGTATAAGAAGTTGGATCGTTATAAATGCAGCTGCACGTGACTACTTTATGGACAATCT
GGTTAGATAGAAACAACCAAATTTTAACAAACAAATTTTCCAAAAAAAAAAAGTTTGAGCTTGTGGAATCC
GTGGGAGAATTTTTGTAACCTTACATGTATACGATCTATCAAAAACACTCTCTTCAAGAACTCTAGCCGA
GCCACATTTTCCTTAAACATTAAAACTCACTAATTTTATAATTTTAAATAATTATTTAAATTTTATATTA
GGTATGGTTTTAATAAATATAATTTCATATTTTTTACTTAAAAATATTATATTTTCTTTATTCTAGATTA
TTGAAATAAACTTAAAAAATTGATGATTTATTGAATGGACCAAATTGCTTTGTAAACTAACAATATTAAG
TTTTATAAGGAAATGACAAAATTGACATAGAAAAAGTTTCTCTATATTCTAGTTATTTATATTTTATATG
ATATAAAGGAAATATTTGCGAGCGTAACAAAATAAATCAAAATATTTATGCAATATAACAAAATTTCATA

TTTATCACTCTATTGGTGTATATTAGTGATACTTATAAATACCAATAGAAGTCTATCAATGTCGATAACA
ATTAGTAACAACCGTATTTTCAACAACAAAAAGAAGCTCACCATCAACTCCTGGGAGAACATATGTAATT
CGATTGGATTATGGTCTAGCCAGACACACTCTTTTCAAAGACTATAAGCATGAACAATTTCTCTCAACTT
TAATGCTTTGATAAACTAATTCAATTTGTATTGGAGTTTAACTCTGGCCCCTTGTCTACCAACTTGTAAT
ACTTTCTATTCAATAAAATTAGGGTTGTTGGGTGCTAAAAGGAAAGCCCGCTTGCAAGGCTATCTGGATA
AACCTTATAACTCGTTCATTTTCTTGGTCAAAAAAATAAAAAAGAGTATCAATGTTTGTATCACTGATAA
AATCTGAAATTTCGTTGTATTTTGTAAATGTTTTTTACAATAATTCTCGTTTGAAAAAAACAATCTGGCA
TATCACATATACTTGCCACATTCATTTTAAGGGTGAAATAAATCGAAAAATTAAATGAAGCAAGAGTATT
CATGGAAGAGAAATAATTATCATAAATTTTAAGGGACCATTTGTTTTAATTATATTCACCTACTTCCTAC
CACACCGACATACACATAATGTGTGGATATGAACTTTAAGCATTCAATAAGAAATTGATGAGTAACCACT
GGGGGTTTGTTCTTCAATTATTGATCATTTCTGGCATTACTACGAGTCAAACATAGGGGCCTGGGGTGTA
TACATGATTGAGTTGATTGTAGGGTGGGAAATTATTAACGTATGGTTTTTGTTGTTGTTTTGTCTTGTCA
AATAAACCAAAGTAGTCGGCGATTTCAAAATTTTTCAACTTTAACTGTTGAAGGGTTGTTTCCAAAGAAC
TCTTTCGTAATTTTGTCTTTCTTTTGATCTTTTATGTCTCCTTGGCCTCAAATTCTCACTAAGGTCATTT
TCCCACTATAAAAATAGTGCTATACATTTTAGGAATCTTTTCTTCAAACTATTGAAGTTGTGAGAATGTC
AAATGATTGTATCATATAATTCAATTTAACTATTAATAGTATTGACAAACGCCAATACTACTTATTATCT
TCTTTTCATCCTTAACTTTTTTTTCAATATACATCGGTTTATCATTGACATGCAGGCTTTTATCAATGAT
ATATAATAGAAGCATATTACTGATAGCAGCTTATCTCAAATATAAGATGTCAACTGTGTATTCTATTATC
ACCGATAGATTTATCAACGACAATCTTGTTTCACTCGATAGACTTCTATCACAAGATATAGGTGGTACTT
CTATCACTAACTAATAAAAATCTAAGCTATGAATGTTAAAATTATATTGAATCAACAGCAATTCATATGA
ATTCCACAAAATATAGTGATACATGCACTTCTATCATTAATAGAAATCTAGCTAAGGACAACAACAATAA
TGATAGAGGTCTAGCTAAGGACAACAACAATAATTCACATGAGTGCCACCAACAATATAATAACTTCCGC
ATCAAGTAAAGAATTAAATGAACGCTATCAACATAATTTTCACGCATGTATTGAATCCTATCATAGACCT
TAGATAACCATTATTGTTAATATGAAACAATTGAATCTTATTCAACGTAACACATTTTAGGAGTAAAAAT
TAATTTTTTTCCCCAAATCTTTAAACTGAAAAGTCATGTAGCTTTCTAAGAAATCTAATAATTGATGAAT
AGTACTATTGGGATTTGAAGTCAGTCCATTAGTTAACTATGCCCAAGTGGACAAATGAAAAACTGTGCAT
GCAGTGCAAGGTACGTAGCAGAATACCTGTCTCTCCTTTAATTTGGTAGTTGCAATTAAGAAAGAAAATT
AATTTCGTACCATTGTATATAGGTTTGCTTTTCTCCCCTTTCCCATATACGAGTTCTAATCTAATTTTGT
TGGTAGTTTTTCTTCGAATGCAAGATCGATCATGTGGAGGCTTAAGGTAGGTGAGAGTGGGAATGATCCT
TACATCTATTCTGTGAACAACTTCGTAGGAAGACAAATATGGGAATTTGACCCAAATGCTGGAACTCCTG
AAGAGCGAGCCGAAGTTGAGCGTGTTCGCTACAATTTCACCAAAAATCGTCGCAAAGGTTTTCCAAGTGC
GGACTTATTTTGGCGCCTCCAAGTAACACCATTCTTCAAGCTTTTAAGAATATACCTATGTTTACTTTTT
TGTGGTTTCAATTTGATAATTTTAGTTTTGGAAAATAAAATTTAAAAGTTCTTATTCTACATTAATTAAT
TCAGTATTTCTATTTTCAAAAATATAGCAAGTGAGCTAATTTAGTTACAAACATAGCAAAATTTTGATGT
CTGTCAATGATACACCTAGATAAATATCTATCACTTATCGACATTAAAATTTTGCAATATTCGAAAATAT
TTTAGTAGTTTTGACCTTTAAAAATAGTTTACTTATTTTTCTTTTAATTATTTTAAAAAAGAATTATAAT
GGACAATAATTTTAGGAATAATTATCAATTGCATACGATATTTTAAAAACGTTGCAATTATAGCAAAGTC
AATCAAGTGTTGATAGACTTTTATGGTCTATCAATGATGACTTTTATCATTGATAGACTCTAATAGATTT
TTCTAAATTCGTAATATTTTAAAAAAATATTGCTATGTACTCACAGATTAAATCTAATATTGTTATAGAT
TTTGCTAATATTCGTTATCTTTGCCATGGTTGCTATTTTAAATATTACATGACATATTTAAAATAAATAT
GCTTCTGTCTTACCTTCCTTTTCTCTCCCTCTCCATCTCCTCCCTCCACAGTCTTCGAAATTTAATTATA
TCTGCAACATATTAAGTTATATCCAAAAGAAAAAAAATGAATACATTGCTATCTATACTCATTTGGTATC
TTTAATTTTAATTCAAGTTATTGGATTACAAATTCGTATGGATTTTGAATGGAAAAACAATAATTAATTG
AAATGATATGGGTGCAGTTTCTTAGAGAGAAAAAGTTCAAACAAAGTATACCACAAGAGAAAGTGAAGAA
ATGTTGCGTTATGTCTATAACCATCAGGTAAACCATATTCTCTTATCATCTAAATTTATAAATTTCAAAA
GTATATAGTGGTGAATAATGTGAAATACTATACAAAAAAAAAAGTTCACTTTTTTAAAGTGAATTTAGCT
CAACTAGCATATCAACTCTTCTCATTCTCTATTGTACTATTGTACTGAAAAAAATAAAAACAAAACTCAC
TTCGGGCATGTTATATAGAACTTTTTTGGAGGATCATATTCTTTTTACCCATATGTTTCACTAAAGAACC
ACACAGCTGGAGAGGCAGAGTAAATAATTGATAACTATAGTACTTTGCAGAACGAAGATGGAGGATGGGG
GTTGCATGTAGGTGGTCACAGTAACATGTTCTGCACTACCTTTAATTACATCTCTTTGCGTTTACTTGGG
GAAGGACCTGAGGTTGAAGAACTTTCGAAATCCAGAAATTGGATACGACACCATGGTGGTGTCACCTCGA
TACCTTCTTGGGGAAAGACATGGCTCTCGGTATAAATATCAATTTCATCTCCATATTGTAAATTTTTTTA
AAAAAATTTAATACCTATTTTTATAAAGTCTAATGTTAGTTATATAGTACACAGGTACATTCTGTTTCAT
TGATATACCAAATAGGATGTATAACAGTTGGCATATAATATACCAAAACATAAAAGTACACCTGCTCATA
CACAAATAAGGGTAAAATCATAAAAATAAAAAATTGGAAATCTAGTTTTTTTATTATTATTTATTTATTA
TTATTATTATTTTTGCTATATGTGCAATTTTAGAAGATCATCATTGCTATATTCATAAATACTTTAATAT
TTTTTTGTACTTTTAAGAAATTTTAACCTATTTTTCACTTTCTTATTGACTCCATCCCCTATTATATTAC
AAAATGTTTGTCTTACATATTTTGACTTTGAATAATGTAAAGATATTAAACGTGTTCGATTGGTCCGGAA
GCAATCCCATGCCTCCTGAATATTGGATGTTACCCAATTGGCTTCCCATTCACCCATGTAAGTTCAACTT
TCAACTAGAACCTAAACTAAATTAATTATAATATTCATCATATTATATACATCTATTCAAGCTTTTATAT

ATATATATATATATATATATACATACATATACATACATGAAACCTTTGATTTTGATCGATGATAACTTGATT
CATACAGCAAACATGATGTGTTACACTCGAATCACCTACATGTCCATGTCTTATTTGTATGGCAAAAGGT
TTCAAGCACCACTAACCTCTTTTGTATTACAATTAAGAGATGAACTTCACACTCAACCTTACCATCAAAT
TAATTGGAAGAAAGCTCGTCACATGTGTGCTATGGTATGATGCTATATATCCTTCACTACTCTATATATT
TAATTACTTAGCTAGGCCACACCAATTTATATATTATATATGTGTGTGTATGATTAATATTGCAGGAAGA
TTTGTACCTTCCACGTCCCATTGTTCAAGACTTGCTTTGGGACACTCTTTATTTACTTAGTGAACCACTC
ATAAGTCGATGGCCTTTTAATAAATTGATTCGTCAAAAAGCCTTGAATGAGACTATGAAACATATTCATT
ATGAGGATGAAAATAGTCGCTACGTTACCATTGGCTGCATCGAAAAGGTTTCATATCTCAACTCTAGCTC
ATCGCTAGTTTCTCTCTGTATATTTTGTTTTGATTAACATGCATCACTTTTGAATTAAAAGCTAACTATC
TTGGTATACCTATAGATCCCTCGATTTATAGTATCTTTAACATTTATAGGTTTTTTTTTTTTTTTTGGAA
AATGATACGATGGGTCAGTAGGAGCACTCGAAAATCTACACTTGGTAGACACCCCCTTTAACGCCCTCAT
CATCCCCACTTCATTAATATAATTGGAAAAAGTACAACACGTAGCAAGGATGACAAAACAAAAAGCAAAG
GGCTAGAGACAAACTCAAAGCAAGGAGAGAATAAAAAACATTAAGATTAATGGCAATAGTGCCATTAATG
TGTTAGTACATTTGTGTTAAGAGTAGGGACCTTTCTCTGGACAACATCAATTGTGTTAGTACATTTGTGC
AGTAGGAACCAAATGAAAAATCTACATTTTTTTGGGAATCTTGGATTTCCAAAGTTTCTTGAAAATCCCG
GCATTGTTTGCATTGTTCATGTCAGCTTCAAGTGCAATGGCCTTTTTAACGGATGCAATTGAGAAGGTGC
CATTTTGATTTAAAGCTACAGATGGGGATGCTTACATTACTTTGTATAATAAACTTGTTTAAATAGTTTT
AGTTTATATATCTTCAAATGTCCAATTTTATTTTATTTTTAATAAATATTAAATTTAGTTCCTCGTTCGA
AATACAAATTTGTCTACTTGGCATGTGTTAGCATGCCCTATTGCAAAGTTTCTTAAGATGTGAAGGGCTC
CCTGAGGCATTTACGTACACAAATTGCAACTCACATACAAGTGAAGTCTTACTGTCTGGTGTCTGGAGTG
TGGTTAAGATAGTAAATAAGAATGAGTCAGATTTAATTGAAGTTCCCTTTTTTGTTGAGGATGCAAAAGA
TTTGATGAGGAACTCTTGGAAAATGTTTTTTTTAAGCATTTTCATTTGTCTCTCAATGTGTTTTAAGCAT
TTTCAATTGTCTCTCAATGTGTTCTCGCTCACAGCCTAACAACAAAAGCCTTGAATGAGAGAATTTCTGA
TGTTCTAATTTTTCCATACCCTTAACGGTTCTCGTTACTCATATCTTGTGACGATGTCATTCTATAGTTC
TGTTTGCTATCTTGTTCCACTAATAATAATAAAAATTCTCAAAGTTATGGTTTTTTACCCACAGCCTCTA
TGCATGCTTGCTTGCTGGATTGAAGATCCAAATAGTGAAAGTGTGAAGAAGCATTTAGCCAGACTTCCTG
ATTATTTCTGGATGGCTGAAGATGGTATGAAAATACAAAGTTTTGGTAGCCAATCGTGGGATGCTGCTTT
CGCCATGGGAGCTCTGCTTTCTTGTAATATCACACATGAAATTGAGACTACCCTCAACAATGGCCATCAA
TTCATCAAGAACTCTCAGGTGATTAAATAAATGCTAATTAATTTTTGTAAATAACTCAGACGTTATTTTA
AATCTTTGGATGGAACTCTAAATCTTTTTTAGATTATTTAGGATAAAAAGTATTTCTCAAGTGCATTTTT
GCATTTTAAATAATTTTTGGACGGAACTCTCTAGTTAAATTTTCAGGACATTATTTTAAATGGCAAGACT
TCTAAAAATATTTATAAGTACAGTAAAATATCACGATCTATTTTGGTCGCCAAACTTCTAAAAAATATTT
ATAATAAAATATGAACAGTTTTAATGATATAGGACAATTGTTCCCACTCATTTATATAAAGTTCTAAACT
ATAGTAACATGACATGAAAATAAACAATTAATACATCAACATTTGTTAACCCAATTCGATGAATATGCAT
CTACATCTAAAGGCTATTTGCCCGGAATAGAAGATTGCATTAATCACGTCAAATACAACAAGTGTTGAAT
ACATAGTTATGATTGAAAAACAGCACTTCTACACAATTATTAATGTACTGTTCTAGAACTGTTTCAACAT
AACTCTCCTTAATTGCAAGCAACAGAACTCTCCTTAATTGCAGCACCAATCAATGATGTATTCAACTTCA
ATTCCAGTTCATTTAAAACTTTCTTCTATCAAATTTTTCTTCAAAAACGCCACAGAGGAACTTACCGTAA
TTGGTATTTATCCTTAATCCTTTCAAGCACGTCAAAAAAAGTCGATCGACTTAAGTTACAAATATATAAG
GGAAGATCTTAACCACTCGTGGGTCATTTTTTATGTCAATTCTTTAACTAATACGATGAAATAAAATAGG
GGAGATCAATTTGTTAACATATAATATTTATAAGTATAGTAAAATATCACAATCTATTTGGGTTGTTGGT
CACTATTTTTTATCACCAACCTCTAGTTATCGGTTCCTCCGATCAATCCCTACTTAACAATCAGTGACCA
AAAACTGGTGAGCCATCGTGGAAGTAATGCTTGGTCCTTTTGGTCGTTGATAGTAGTTATCAGTGGGCAT
TGGTGGCTTGCGACAGCGTGTAGTGGTCGCCATGGTGTGAGTGAACGTAGAAAGATATAGAAGGCCGTAA
TTTTTTTATATATTATGGAAATTTTGGAGGGATAGTCTTGGGGTAGTACCTATTTTTTAAAATTAAATAC
TTAAAAAATCAATCCAAACACACACTCAATGTTGCTTTGTGCCTTACCCAGACTTATAAGTTATATGTTG
AGAGAAATTGTAGGTTAGAAACAATCCTTCAGGTGACTATAAAAGTATGTTCCGCCATATGTCTAAAGGA
TCTTGGACGTTTTCAGACTGTGATCATGGATGGCAAGTTTCTGATTGCACTGCTGAGAACTTAAAGGTAT
GGCTTCAAATTTCACTTAATTCATTACTTTCCACACATTAATGCAGCCTATGACTATTAATAATATTTAA
TCAAACTTGTAAAAATTTTCTAGTGTTGCCTACTTCTCTCCTTGCTACCACCTGAAATAGTTGGCGAAAA
AATGGAACCAGAACGTCTCTACGATGCTGTTAATGTCATCCTAAGCATGCAAAGCAAAAATGGGGGTTTA
CCAGCCTGGGAACCAGCATCAAGTTACTACTGGATGGAGGTATATGAAAAAGAAAAATATTTATATGCAT
CCCGGGAGCTAACACCCATTATTTTTATAGTTCATGGTCCAACAATAGTTTGAGTTACAAAACTTTCCCT
TTTTCTTTTTCTTTTAACATTTGCATTTAAAAATGAGTAAAGAAACGTTGTCTTAACCCCTTTCTCTTCC
TTTAATATTATAATATATATTTGTTCTTTTCTTAAAAAAATATACGTTTGCACACGTACCCATACACTTT
TGCTAGGTGAACACCCTTTAACACCCTCATCAGTTTCATTAATATAATCAAAGATAGTACAGGAAGCAGG
AAGAGTACATGAAAAAGCTAGAGATAAGCCCATAACAAGGGACCCAAAAGGCTAAACAAAAGCATGAATA
TTTAAAGCAATAGAACTGGAGTAGTAATTTGCAAAGTTGCAAAGAGCTTTGATCTAGTAGATCAAAGACC
TGGCTGTGAGAGCAGGAACGTCATCCCAAATTCACAAGTGGATCTTTCCTCTTCCCTTGAATATTTCTTT
CGAGCCATAAGGACCAAAATAACACAGCAATGACGTTGAAAGTGATAGCCCATCTTTTGTTCTTCTGATT

GATAGAGCAAATCCCTTTGCATAAAGATTTTACGCTGTCCTGTATATTCTGCCAATGTATCAGTTTCCCA
GCCCTCTTCTAAAGTTCCGGACAGTTTCGCAAGAGATGAAAATATGGTCTAAGATTTCCCACTTCTTTTT
GCAAAGATAGCACAATTTGGATTGAGGTGCAAATTAGGAAGACGCCTTTGAAGTTTATTTATGGTATTAA
TACATTCATACAGGAGAGTCCAAATGAAGAACTTACATTTCTTAGGGATGGTGGACTTCCATAAATTTTG
AAAATATGGAAGAGTTGATTCTTTCACCATTATTCTGTTCACCAACCTGTAATACTCATTTGATGGATGC
TATGTTAAACTGCCCATCCGAATTAAGATTCCATAAGGGTATTGGTACCCCTGTTTATGTCTGGGGTATA
AAATATGCAACCTTAACTAAGTCCAGAAGTTTTCTTTAACTGTATCTAAATGCAAGATGTCTTAAATGTA
TGGATATCATAATTATTTTAGAATTATGTTTAAGTATGAAAAATCTAAATAAATAAGTCAAAAGTAAAAT
CTAAAAATATCCTAGCATTACTTCGGGTTTCAAGTTCAAAATCTAGAGAATTCCTTTAGGTAATGAAATA
CTCACAGAAATATCTAAGAAAGTCTAGAACAATCTTTCGCACTTGGGCACAAAAGATCTCTCACCTATCA
ATATTTATATGTCTTTAGAAAAACTAAAGATAAAGTTAGACAAACTGAAGAAAAAAAGTTGTGAGCAACT
ATTAAGAAAATAAAAGGTCCGCTTACAAAAAGTGTTCCTGGTGTGATATGACGGATGTGTCAACCTAGTC
AAAATATTTAGAAAATATTTGAGAGTACTAAATGATCTGTCTTACCGATTTTCTTTAAAAAAAAAAAGTGT
AGGTTAACTTTCTTCAACAGATATATTGTGTATCTTTAAACTCATTCTACCTTGTGCTTAATTAGTGGTT
GAATCCAGTGGAATTTCTTGAAGACCTAATCATCGAGCACGAGTACGATTTTTCATTAATTAATTTTCAT
TCTACTAAATTAATTTTTTTTTAATTTTATTTGATTAAACCTAATAATTAAGTTAATTAATATTATTCTG
AATTTATGAATATTATTTTCAGACATGTGGAGTGCACCTCATCGGCATTACAAGCCATACTTTTATTTAG
GAAACAATACCCATCACATAGAAAGGAAGAGATCAATAATTTTATCAACAAGGCTATTCAATTTCTTCTG
GACACACAACTGCCTGATGGTTCTTGGTAACCCTATTTATTATTACACATAATGGGTACTGACATTTTTG
TACAAATTACCCAAGTTTCACTTCAAAATCTGGTTTGACTCTCATTTTGAAAACAATTAAGAAAATCCTA
ATTGTCAAGCTTAGCATGTGGAAATATTATTGTATTAGATTAGCTGATCACCAAGTACAAAACAAGAGAG
TCTGTGTGAATGTGAGAGGGAGTGATGAAAGCAAAAGAGTTATACAACTCCTGGTCTTAGGCGATTATAT
GTAGATGTGCCTTTTGATCCCGCCGCCCTTTTATGATTTGTTAAAAAAAGTTAAGAGTTTTATTTTCAAA
ATCTAGGTCAAACGAAATTTTCCAACATGAGATTTTAGTCATCGTTACAAAAATTTTCTAGAGAAAGTTT
CAAGCATTGTGTGCGTATACATATATAGGTATGGAAATTGGGGGATTTGCTATATATATGGAACATGGTT
TGCACTCAAGGCATTGTCAATGGCGGGGAAGACTTATGAAAATTGTGAAGCACTTCGAAAAGGAGCTAAT
TTTCTACTTAACATACAAAATTCAGAAGGAGGGTTTGGAGAGAGCTACATGTCATGTAGCAAGAAGGTAA
CACATGTATATTTATTTTTTTAACTTACTTTCTACCATCAGTGTAGAGATACAAAAATTTAGTGTCAATT
ATATGAAAATTTTGTCATTTGTTTTCAGAGTTGCAATAATATTACTTTTAACTTTTCATGATAATTCATT
ATAGGGTATTATCGTAGAACCTCGTTCGAGATTCTACATGTTATTCAAAATTCTAAAAGATTTGTTGGTA
ATTTGAAATATTAATATGGATGTTATTTAAACAATATTCTTCATCTATATATATATATATATATATATAT
ATAATTAACTATATATATATNTGAAAAATAATTTGCTTCAAAATAACACTAATGAAATACTTAATTAACAGG
CAAATATAGACCCCAATCCAATTCATCGTGCTGCCAAGGTTTTGATCAATTCTCAAACTGAAGATGGTGA
TTTTCCACAAGAGGTGATGAGTGCCATTTTTCTTTCTTTCTTTTCTTTTTTTTTTTCTCTTTTTCTTTTT
TGGTTTCCTTCTAAAACATATATTCCTAACCAATATAATTAACAACTTCTGATTATTTACATGTGCAGGA
AATTACTGGAGCATTCTGCAAAAATTGCACATTACACTATGCAGCATATAGAGAAGTTTTTCCAGTGATG
GCATTGGGAGAATATTGTAACAACATTTCCCTGTTTTCTAATAAAAAACAATAAATTTGAGATTGGAATT
AATTAAACCTATAAACTACATATTAATAAATATGCACATTTGGACTCAAAAAGAGAAAAAAGATGGAGAC
TTTTATACTATTATTATGAAAATGTGATGAAGTTCAGATGATGTCTAGAGCTTCTCTCGATAAGTACATC
AAATTATAAAGAATTGGGCTAACCATCTTTTTTTCTATGTGGCAAGAGAATTAAGATATATATCAATAAA
TGTCAATGGGAATTTTTTTCTTCTCAAATTGACAAAGTATAGAGTACAATCTTTGCTTCCAAATCACTTA
TGCTTATTATCTTCGCGTATATGTATCTCAAAAGAAATTGCATAGCCACTTAACAAATAGTTGTACATGC
ATTATGAGAGAAGGTGATAGTGACAATAGCAGCTTATCAGAGAAGGCAGAAAAGAAAAAAATAGAGCAGG
AGATCGAAATGACTGATAAGAAAAAGGGTAAGCAAATTTGTTGTGTTGACTCTCCCCCAAAGTTGAACTC
TATCAACACAAAGAGGAAAGTTTCCTTTAGCTCCCCAAAGAACGAAACCTTTCACTACTCTGCCCAAGCT
GCTCCCATAAAGGCTCTAAAAATAGGAAGTCCAATGGGTTATGCAAAAATATGTAGGGAAGCAAAAACCA
AAAGGACAGAATTGCTCAAAAAGAAAAAAGTTTATAGAGTCAAGAGCCAAGACACCGAAGGAGAAACTTC
TCAGACAAATAGACAAAATGAAAAAGGCAGAGATGACCTAAATGAGCTTAATCTGGTTATGGACCTTGGC
CATATTTCTCCTCTTTCAGACATGGATTTCTCCAGCCCTGAAAGATCCTCGTACACCCCTTCACCAACAA
CTCCTACCGAGTCAAATATTGTGAAAGACAGTCTGGCAAATATGATCACGAGTGATCATGAAGAAAAAGG
AAGGCAGAAAAGGGAGATAAAAGGAGATGAGACTGACGATGATGAATCAAGCTTCAAGAGGAAATTAACA
AAATGGTTGAAAGAAAATAACCTTAGGCTTTCTGCTGTCTTTAATTCTCATTTTAACTCTACTTGTGATG
ATAGGGTGGAATCTGTTCCAAATGGGCCACAAAATTTAGATGTTGAAAATTTGACTGATGATGTAATAGA
TGGAAAGGAAAAAGACACTATGGGGTTGAATGTGTATCCATGAAGTTGATGTACTGGAATGTAAGAGGGC
TTAGCTCGGCCCAAAAGAGAGCCCAAATAAAAGCAACTATATTTGTCTATTGTCAAGACTTTGTCATTCT
AACTGAAACAAAACTTTCCTCTGTCTCAAAGAAAATTATAAAATCTCTATGGAGCCCAATTAGTATCAAA
TGGATTTTCCTTAAAGCCAATGGCCGATCAGGAGGCATTATTGTTATGTGGGATGATCAAAGACATTCAA
TTCAAAGTGTTTTTGAAGGAAAATTCTCTATCTCGGTCCAAATTTGTTACCAAAATGGTGTGTGCTGGTG
GCTCTCTGCCATTTATGGTCCAGCTAAAAGAAAGAAAAGGCCTCTGTTTTTGGGAGGAACTCGACAACCT
TAAAACTATCTGCCTTCCAAATTGGATTCTTGGGGGAGACTTTAATGTAATCAGCTGGAAGGAGGAGACA

TCTGCTAAAAATCCAGCTTCTCTAAGTATGAAGAGATTCAACACGTTCATCAGTAGTTGCAATTTAATCG
ACCCCCCCTCACAAATGCAAAGTTTACTTGGTCAAACCTCAGAGCACATGCCACCTTATCCAGATTTGAT
AGATTTCTCTACTCACCTGAATGGGAAAATACTTTCTCCGGCCATTATTCTAAAACTTTGACTCGAATTA
CATCAGACCATTTTCCCATAGTCCTTGAGTCCTCCTCGGTATCTTGGGGCCCCCCTCCTTTTATATTCAC
AAATGCCTATCTAAAAGATATAGATTACAAGAGAAATATAGAGAAGTGGTGGAACAATACTTGCCAATCA
GGCTATGCGGGTTACTCTTTCATGCGCAGGCTAAAACAACTAGCCAAAAAGATCAAAAAATTGGGGTAAA
GCCAAAAAAGGAAAAGCTGAAGAATTTAAAAGGGCCTGGATTAAAGAGATTGAGCTCATAGACAAATTAG
AGGCAGAAGGTAATTCAACTGAAATGCACAGAAACAAAAGACTTGCTCTAAAAGTCGATCTCTCCCAAGC
TACCTTCATAGAAACTCAGATGTGGGCGCAGAAATGTAAAAGAATATGGAATCACGAAGGAGATGAAAAC
TCTTCTTTTTTCCATAAAATATGTACAGCCAGACAAAGAAGAAGCATAATTTCTAAAGTGACCAACCACT
GCGGGCAGAGTTGTATGAATGACAGTGACATAGCCGAGACCTTCATTCATCATTTCAAAGAGATCTATGC
CGATAAAAGAATAAATCAATTATTTATTGATAATCTCGACCGGTGCCCTATTACCAACTACAGTCGTTGT
ATGCTTGACAGGCCCTTTAATGAATCTGAAATTTGGCTCACTTTAAAGTCCTTTGCAAAGAACAAAGCCC
CGGGTCCAGATGGTTTTACTATGGAGTTTTTGAAAAAATCTTGGCCGTTCATGAAGCAAAACATCCTAGA
CATTTTCAAGGACTTTCATAACAGCAAAATCATCAACAAAACAGTCAATGAAACTCTCATTACCCTTATA
GCCAAAAAAGATAAGTGTGAGATAGCATCAGACTTTCGCCCCATCAGCCTCACTACGGCTATCTACAAAT
TGATCACAAAGGTTTTGGCTGATAGATTGAAACAAACTTTGCCCGACACCATTTCTAAATTACAAATGGT
TTTTGTCAAAGGAAGACAGATAAATTCAACTTTAGAAAGTCTCCCCATGTATCAACTTTTGGTTTTCAAG
GCCCCAAATCAAAGCATCTTGGAGAAATTTTTTATGGAAAGGCACTTCCAGTGGACACAATATCAGCCTT
ATCAGATGGAATAAAATTATAACTCCAAAGGAGAAAGGAGGCTTGGGAATTCACTCTGTCCATAGCACAA
ACTTTGCCCTCCTCTACAAATGGCTCTGGAAATTCCTAACCGAAAAAGACCCTCTCTGGAAACGGCTGAT
CATTACCAAATATGATCAGGAAATGATGGGCAAATTTCCTATCCGTGGTAAATTCAGCAGTAATAAGGCA
CCGTGGAGAGCTGTGACAAATTGTATTAGGTGGTTTTATAAAAACATTGGCTGGAAGGTCAATGATGGGG
CTGCTATTTCTTTTTGGCTTGACAACTGGAATAGTAAAATTCCCCCCTTTCTCTGTCCGCTCCCCGCCTG
TACGCTCTGTCTACAAACAAAAAGGGTACTGTCAAAGATTTCTGGAATCCATCCCTAAAAGATTGGAATA
TCCATATCAACCGCCCTCTCCGCGATCATGAAACAAATTTGTGGCACAATATCAAATCCACTCTTACAAT
GCCACTGACAAACAGGGGTCTCTCAAAGCCTCTCTGGAAGCTAAATTCAAACAATATTTTCGACACTGCT
TCTGTTAAAAAGGACCTAGCCGGAGCTACTTCTTGCCCAGCAAATTTCCCTCCTAGTCTTTACAAAACTC
TGTGGAAGGTAGAATTCCCAAAAAAATGAAAATTCTTTATTTGGACTCTTATTCATGGCTGCATAAATAC
TGCTGATCGATTGGAAAAACGTCTTCCAAATTGGGCCCTCAGCCCAAACTGGTGTTATATGTGCAACAAG
AGTCAAGAGGATATAAACCATCTTTTCATTCACTGCCCCTATATACAGTTGTTATGGAACATAAGGTCCG
GTCTCTGTTGAATTGGAACAACACTCTTATTGACGTGAACTCCCTTGTTAAGGATATTTGCTCACTAGAT
ATAAAGACTCAAAAGGGCTGATAACTTTCAATACCATTGCTGTTCTACTATGGAAGATTTGGTTGGAAAG
AAACAGTAGAGTCTTCAAGCAGAAGAAAAAAGAAGTTCAAGACCTTTGGGAAGACATCCTTGCACAAACC
GGTTTATGAAGCAGCAAATCAAAATTATTTTCAAATTATAATTGTAGTTCCATAGCTTTAAACATTTCAG
CTTTTGTTTAAAAGGGCTTATCTCTAGCCCTTCTCCTTTTTGCTTCTTGTACTGATTGTTTATATATTAA
TGAAGCGGGAGTGATGAGGGTGATAAGGGGGTGTCCACCTAGTGGAGATGCCCGGGTGCACCCAACTGAC
CCATCGTATCCTTTTTTAAAAAAAAAAAAACAAATAATTGTACATGACGCTGTAAATATGTTCGGATGAT
AATAGTAAATCATTACGGAGCAGAATCAGCTGAGTCATTAATTTTGAGGAACACCTACAACTTTCCAACA
CTTACACCCACAAGTTGTGGGTGTCGAAAATTAACTGACTCTCATATTCTGCTCCAATTGAGGTGGGAGG
GAGGTGAGTGTGATGGTGATGGTGATGGTGAGAACGGAGGGAATTCTCAAATGGGTTTGAGAGAGATGCG
AAAAGGAGGCCGGAGATGAGTTTTCGGAGATATCGAATGGTGGTTGATGAAAAGAAATGTGCTATATATG
TTGCTGCAATGTCGACACTAACATCTCTAAAGACAAGCACATCATGTGATTTTATGACCTTAATTCCTTA
CTTTTGCAAGTGTAAGTCCATTCTTATATTTATATTTGTAAAATCGAGCATGAACTTATGTTAAAGAAAG
ATGATCATATATTTGAAGGGACCGTTGTTTTAAGTATATTCACTGACTTTTCTTCCATCCACTGACATTA
TATATATATAAATATGGGTCATGTGAACTTTAAGCATTTAATATAGTAAAACCAAATACTAGCTAGTGGC
CACTTGGGCAGCTACTGATATAGCTTAATTTTCTTTCAAAGTACAAACGTGTTGTATATTTTTTTCAGTA
AACGTATAAATTTTACATGAATATAATGTCACCAACAGTAATCAACAATAACTACTATAGTATTGAATCT
ATCCACAGATCTTAACTAATAATTACTGTTGTTACATAAAGAAGAAATTACGGTATAGCATTAATTTTCT
TGCATTGAATCAACGAGAGAAAAATAGTGGAAGAAGTTCTACTCACTGGTCATTATTAATATGATTTAGT
AGTGAGTGACGTTTTTTTTTAACAAGAAACGATTGGTCAGTAGGTGCACCCGGGCATTTCCACTAGGTGG
ACACCCCCTTAGCACCATCATCATTCCCGCTTCATTAATAATATATCAAGAAAAATACAGGGAAGCAAGA
AAACCCAAGAAAAGTACAGGGAAGTAGTGAGTGACGTTTAAACCGTAATGAAAAGAGGACAAATTTGAAA
TAATGTTAATATTGAGACAAATTGTTAGGGCTTGAAGAAGAGAGGGGTAAAGAATAAGAATCATAGTTTC
CATAACTTATAAGGGCTTTATGGAAGTGCATTTATAATAAATGACTCACCCCTGGTAAAAAGTATGAATT
CTAACCTTTTGTTGATGTGTTTGTTAATATCACATCTTGTTAGAATTTTCAATATTCGAAAATATTTTAG
AAGTTTTGACCTTTAAAAATAGTTTACTTATTTTATTAAAAAAAATTATTTTAAAGAAGAATTATAATGG
ATAATAATTTTAGGAATAATTATCAATTGTATATCAAGCGTATGGTCTATCAGTGATGATTTTTATTATT
GATAAACTCTAATAAATTTTGTTAAATTTGTAATTGTTTTTAAAAATATTGTTATATGCTAACACGTTAA
ATCTAATATTGTTATAGATTTTGCTAATATTTGTTATCTTTTCAATTGTTGCTATTTTACATATTACATG

ACATATTTAAAATAAATATGCTTCTATCTTACCTTCCTTTCCTCTCCCTCTCCATCTCCTCCCTCCACAG
TCTTCGAAATTTAATTATATCTACAACATATTAAGTTATATCCAAAGAAAGAATGAATACATTGCTATCT
ATACTCACATACATTGCTATCTATACTGACTTGGTATCTTTAATTTTAATTTAAGTTATTGGATTGCACA
TTCGTATGGAGTTTGAATGGAAAAACAATAACTAATTCAAATGATATGGGTGCAGTTTCTTAGAGAGAAA
AAGTTCAAACAAAGTATACCACAAGTGAAAGTAGAAGATGGAGAAGAAATAAGTTATGAAAAGGCATCAT
CAAATGCAATGAGAAGAGGAGCACATTTCCTAGCAGCCATACAAGCAAGTGATGGGCATTGGCCAAGTGA
AACATCAGGCCCTCTATTTTACATCTGTCCTATGCTAATTTGCATCTACATTATGGGTTGTATGGATAAA
GTATTCAGTCCCGAGCACAAGAAAGAAATGTTGCGTTACATTTATAACCACCAGGTAAAGTACTATATCC
TTATTATGAAATCTTACTATGTCAAAAGATTATTTCAATCATACAAAATCTCTCTTTGGTTCCGCTATTA
TAACAAACCATCCCCAAAGTTTTATATCACTCAAAACAAGTAGAGTTATGGTGTGTTTAGTTTAACTAAT
TTTGGAAATTATACATTCGGAAAAAAGTTGAAAATGCTTGGGGTCTACTTTAAATAAAAATGACTCCAAA
ACTTGAGATGATTTGAACCCAAAATTATACTTTTTATTGTACTTAGAGTCATCTCTGAATTATTATTATT
AATTTTTTTTCTTTTGACAAACACTCTGTAAAAACTAAGTAGGATAGAGACGTGTCGACGTAAACGTAGC
TCAAACTAATTGACATCTATTGAAGTTGAAATTTGAAAAGTAGGATCATATGGCACAAGTGACAATAACA
GTAACAATATTTTACAGAATGAAGATGGAGGATGGGCGTTACATATTGGTAGTCACAGTAGCATGTTTTG
CACTACCCTCAATTATATCTCTTTACGTTTACTTGGTGAGGGACCTGATTACGAGCCACTTGTCATAGCC
AGGAATTGGATCCGGCAACGCGGTGGTGTCACCTACATACCTTCGTGGGGAAAGACCTGGCTCTCGGTAT
ACCTTAAAATTTTTATGAAACGACTTATTCACGATTTATGCTTCGTTTGATTATTTACTAACGTTGGTAA
ATCAAACTATTAATTCGTTTAAGGGAATCATGATTTTAATCCCAACTAATCCTCTCAAATCGGTTGAGTA
GCCTGACATCTATTTATACAGGAGATCTCAAGTTCAAATTCTTCACCCAAGTTGTATTATAATGACTTTT
AAAGAAAACTTTTTCATATTAATATATATTTTTGACATATATATTTCAAATTTTGGTAGACCCAATTTCT
TATTACAATTATCATTATTCGTAAAACTTTATTAAATAATTTATATTCAACAAAAATTATTTAACACTAC
AACAAATTGGACTTTTCATCACTCTTAAGTAGTAGAATAATTGTTAACACGAGTTTAATTCAATTGACAT
CTATATATACAAAGATGAAAAAAAGAGACATAAAAATTTACTTTTCACGTGTTTTTTAGTGGAAAAATGA
AAACTTTCGTATCTATTGGAAATTTATGATGGCTTAAATTCAAATCTTATAAATTTTTTTAAAAATAAAT
TATAAATTAATTATGGATGTGAAAATAATAAAATTATAGGCCTATTTGATTCATATATATATATATATAT
ATATATATATATTGATAAAATTTGATTCATATAATTAAATTATCTCAATTTAGTATTGATTTATCTCAAT
TTGGTCGTCCTAGTTCGTTGATCCGTTACTTGTTAAAAAAACAATTACAAGAACAAATTTCAACAAATCT
TAAAATACAACATCAAAATGAAATTTTTTAACCTAATTTTAAATGTCCTTTAATTTCTTACCAAACCCCA
ACCCTTATTATATACATATGTTTTTTTTTTTAATTCTTAAATTCTTTTTTTGAGTGTGCAACTTAATTAC
ATATTTTGACTTCGAATGATGTAAAGATATTAAACGTGTTCGATTGGTGTGGAAGCAATCCAATGCCTCC
TGAATGTTGGATGTTGCCCAGTTGGATCCCCATACATCCATGTAAGGTCAACTTAATTATTATAATTATA
TCCTAATTAACCAATTTATTAGTTTCGTTCTATTATATATACATATTAATAATAAATTTTGTTGGTCAAT
CGATTATTTAACATGACTTGATGTGTGCAGCAAAAATGATGTGCTACACTCGAGTCACCTACCTGCCTTT
CTCTTACTTGTATGGTAAAAGATTTCAAGCACCATTGACACCTCTTATTTTACAATTAAGAGATGAACTT
CACACCCAACCCTATGATCAAATTGATTGGAAGAAAGCTCGACACATGTGTGCTATGGTACGATATATAT
GCATGTGTATTGTATTTCACCTTCTATATTAAGAGTAATTTTAAGGAAAATAATTAAGTGTTTATCAAGA
TTTGAAAATAATTGAAATGATTTTTACTCCCACAAACAATATGGTATATCATTGGTATAGAATCCAAATT
CCGAATCCTGGGTCTGAAATATCACAGGTATGATCTATCATTGATAAACTCATAATAGTGTTCAAATTAA
AGCTTTCTAGATTTATTTATTTTGCATTGTGCTATATATATTTTTGCTAATTCTTTGGTTTGATTTCTAT
ATTTTCAATTATCTTAATAAACTCTATTGTTGATAGACTTCTACAAATGATATAGTCTATATCATTGATT
GACTTCAAGATTAAATATAAAATTTTGCTATGTTTGTAAGTTCTTTAATTGTACTATAATTGTAGATATT
TTATGTCTGATTATTATACTTGCAACCGTCGTTATCATTAATGGTGCAGGAAGACCTCTACGTTAAACAT
CCCTCTTTTTATAACTTGCTTTGGGACACTCTTTACTTAGTCAATGAACCACTCATGAGCCAATGGCCTT
TTAACAAATTGATTCGACAAAGGGCCCTGGATGAAATTATGAGACGTATTCATTATGAGGATGAAAATAG
TCGCTACATCAGATTTAGGTTTGCTGCATTTCTTTCTTGGTTTAGAAGTTTGTCAAACTGATGGTGGGAT
ATTTATCTCACAAAAGAAGTATGCCAAGGATCTTCTAAAAAAAATTTGGTATGATCAATTGCAAGGCAGCA
ACTACACCAATGAATGTTAATGAGAAGCTGCAACAAAATGATGGTGCAGAGATGGCCGATGCGCAGCGGT
TTAGAAGCCTTGTTGGAGGCTTGAGTTATCTAACTTATACCCATCCCAATATTTCGTATTCTATTGGTGT
GATTTCCAGGTTTTTGCAATGTCCCTCAAGGGATCATTTCGGAGCAGCAAAGCGAGTTATGCGATACATT
GCTGGAACTATAGAATATGGTATTTGGTACTCTAAAGTTTCTGATTTCAAATTATGCAGGTTCACAGACA
GTGATTGGGCGAGCTCCTTGGATGATAAACGAAGTGTTTCAGCAGAATATGCTGCAGCAACTTCAGCAGC
ATGTTAGGCAATTTGGTTGCGAAGAATGCTAACAGAACTCCAACATGAGCAAGAGGGAGCAACTGTAATA
TTCTACGACAACAAATAACAATCTCAATGACGAAAAATTCGACATTTCATAATCGGACAAAGCACATTGA
TATTCACTTTCATTTTATTCGTGATTTGGTTGCAAAGGAAGAAGTTTCTCTGTCATATTGCAACACACAT
GAGCAGTGGGCTGATATTCTCACAAAAGCTTTGTCAAAGGAGAAGTTCCGTTACTTTAGAGCTATGATGG
GTATTAGCAAATTTGAATCAAGAGGGAGTATTGAAGACTTATTCAAATTACGTGATTATTTAAATTAATG
TTTTTTAATTTATTTTCTGTTAACAGGTTAAGTTAGTGGGTAAGTGGTATAAGAACGTGTTTTTTATTTT
TAGTAAGTGTCAGTTTTTTGTAAGCCTTTAATAAGGCATTTTAATTGATTATTCTGATATGAAAAAAAAA
AAAAAAAGAGTTTTTTCTGCCTTGACCTGCTGAATTTTTTGTTTCTGTATCCATAAGTGTCTAAAATTTC

TCCAACACCTTTGTTCTCTCATTGTTCACATTGATAGGGCTTATAGTAGATTTTGTGGTGTTGATTTTAA
GTGTTGATGCCCTTTCAAAAAGCTTGGGGGAAAACCTTAAAAGACTTGTTATAGTTGCAAATTTTTAAAA
TTGTTATTAATTCACTTGGTTATTATCATTATAATTATCCAACTATTAATTAATTAAAATCGTAAAATTT
ACAGTGTTGTTTACGACTCTCCTTGCTACCACCTGAAATAGTTGGTGAAAAAATGGAACCTTAACGTTTA
TATGATGCGGTTAATCTCCTTCTAGACATGCAGGGCAAAAATGGAGGTTTAGTTGCATGGGAACCAGCCT
CACGTTACCCTTGGCTTAAGGTATACGAAAAAACTTGCTTTCTAACTGGGGTGTGTTCGTGTCCAGTAGT
CAAACCAACCGCAGTTTGTCAAATGATAAACTTTCCATCTTTCTTCTAATATTATGACATTTTTTTCACA
TTTCTCTGATATTATATTATATATTGTCTATCTTTAACTCATTCTGCTTTTTGTATAGTGGCTGAATCCG
GTGGAATTTATGTAAGGCCTAATCGTGGAGGAACAGTATGGACTTTTCTCTTAATTTAAAAACTTTATTT
TTATTGTCTTATGATTTGTATTTAAAATACTCGGGTTGCCCTATCTTTTTGCAATACATCTAGTTGTCGT
CAAAACCACATTCTTTATCTTTTTACTCATAAGCTTCTTGGAAAACTTTTTGTCTTGTCACCAATATAGC
TTTGCTCCTCGGTATATTGGTCCTCTGTCAAACCATTATATAAGTTGAAATTTCAAGAATACACTAGTAT
CTTTTGTTTACACGGTACGTAACCTTCTTCATTGAATGTGATCATAGAAAGGAAAGATGTCCATCCATTT
TCCTTTTATCATTCTAGAATAAGTATATGCAAAACTTTCGAACTCCGTCATCGACACTGTAGATTTCAAC
AATGTATGTCTTGTTGTTTATCATTTTGACCCATGCACAATGTAGCCACCTAATTTTATCCTACATTTTT
TTTATTCTTTAAATTATTAGTTGTAGCAACGGTTAAGATATTTCATTAATTTATTTTGTTAAAAAAAGAA
AAAGGTAAAATCTTTTTGTAATAATATGAAATCTTATGTTTTTTTCTTATCTTTTTATTAAAATGGAAAA
TAAAGTCATTAATGTTTTTTTTGACTTATCACNNNNNNNNNNNNNNNNNNNNNNNNNNNNNNNNNNNNNN
NNNNNNNNNNNNNNNNNNNNNNNNNNNNNNNNNNNNNNNNNNNNNNNNNNNNNNNNNNNNNNNNNNNNNN
NNNNNNNNNNNNNNNNNNNNNNNNNNTCTTTTTTCGTTTTTAAAAAAAACTCTTTTTAAGGATGTCGGCC
GCTCCGCGTCGTCTCGGGTACGTGGCGACACACAATATTCCTTGTATCTTTTTTCAAGGAAAAAATAGAC
TTGTATGTGAGGTAACTATCAGGGTTTTGAATGTGGACCTCAACCAATCCAGTGATTCAGGTAGAAGGGA
AATCGGATAAGATTTGTATGAATATCCACCTTGGTCAAAAGTATGTTGTCAATTCCTCCTTGATCTTTAA
TTAATCTAGATTGGTTGTAGGTAGCAAACAAAAGTCATTTTTGGGTAGTTGAGTGAGTAAAGGAGTTGAT
GGATAAAAATACGGATGGAAGAGTGCCTAATAAATAGATCTTATTGCAGTAGGGTGTTGGTTGGGAAAGT
TAGGAAGTAGAAATGATTTATTTGGTGAAGATGAAGAATCTCTTGATGGAGTAGTACAAAAGACGAAAAT
TTAGTACTCATTTTTTTAAGATATGTTAGGCCAGGAGATACACCTAAAAATCTAGTACTCTTGGAAACAA
AAAAGTTTATTACCTGATTCAATAAACTTTTTTTTAGGTGTATCTACTGGCCTAACAAAAAATCTAGATT
AATTACCCGATTCAATAAACTTCTTTGTTTCCAAAAAAAAGTTTAAGATACGTTACGCCAGTAGATACAT
CTAAAAAAAGTTTTTTTTTTTTTTTTTTGTTTCCAATAGAATAATTTTTTTGAACAAGGACATCTTCTAA
TATAGTAAAATAAACCATCATATTTACAAAATATATCAAAATTTCAAATCTATCACTGATAGATAATGAT
ATCGATAGACATTATCAATATATTCTGTCAGTCTCTGTCATTGATGAAATCTAAAATTTTATATTATTTT
GTTAGTGTTTTCAACTGTTATTCCTTTTACATTAATTCTCTAATAATTATATTCATAAGTAAATTAATAA
CAGTGAAGTAGTAACTATATATTAGTGACTAAATATTAGTGAATAGTAACTATGAATTAATTTTATGAAT
TATAAATATATATAGTATCGAAACATTGTAGTTTATATTATAAGTCAATTTCTAAACATCGTAAACATTG
TTATGAAACACATGTTTCTAAAAACATGAAAACTATTCCGTTTTAATTGAATCTCTTATTTTGAAACTCG
ACCCTAAAAAAATTTGTTCAATTCTATTCAATAGGTATTCAAATTAGGCGATTTCCTATACATATGGAACA
TGGTTAGCACTTAAGGCATTGTCAATGTTGGGGAAGACATATGAAAATTGTGAAGCACTTCGTAAAGGAG
CTAAGTTTCTTCTTAAAATACAAAATTCAGAAGGAGGATTTGGAGAGAGCTACTTATCCTATACTTATAA
GGTAACAACATGTGCATATTTATTCATCTTTATTTACTGTATGGTAAGACACAATAAAGCATTTAGATTT
GTGTTAATAAAAATTGAGACGTTACCTAAAACAAGGTAAAACACTAGTAAGTACCAATTTTTTGTTTGAG
ATTTCAAGAATATAAATATTCTTTTTCAAGTTTTTAAATGTCTATAGAGTTGTATAAAGATGAAATTAAT
TTTTCTTTTAAGGATAAGTTGGGTCAATAGGTGGACTCGAACATCTTCATTAGGTGGATACTCCCTTAGC
ATTCTCATTATTTTCGCTTCATTAATGATCAAGAAATACTGATGAAATTGAAATTAACGTTTGGATGAAG
TCTATATTGAGAACATTTGTTAAAGAAGAAAAAGTTCAAAAATACTTTTTTTTTTACCAATAACTTGTGA
GAAATTATTTAAATGATAAAACTTCTAAAAATATTTAGAAATATAACAAAATATTATAATCTCTTTTTAT
CTATTGTCGTCAATCTTTATTTTTTTAAAAAAAAAAAAAAGATACGTTGGATTAGTAGGTGCAGCCAAACA
TCTCCATTAGGTGGACACTTCCTTATCACTCTCATCATTCTCCTTCATTAATGAATATCAATCAGTACAG
AGAAGCAAGGAAAGATGTAAAAAAGGGCTAGAGGTAAGCCAAACACCCAAAGGCATTATACAAAACCATG
AGGATTTAAAGCAATAGAAGAAGTCGAGTAGCTAGAATTTTTTTTTTATCTACTAGTCCAAAGGCCGGTA
AGAGCTTTAATATCTTCCCATATATCTCGTGGGGATTTGACTTTGTCACTGAAAATTCTATTGTTCCGTT
CGAGCCAAATACTCGAAAGGGTGATAGTTGGCCACAATGTTCAAGATGATGATGTTCTTTTTATATTTTT
TATTCCACTCACAAATATGTTTATAGAGATCAACTGAGTTGTGGAGTTGGATATTCCATTTATATAGAGA
ACCGATTTTTCTCCATAATGATTCTGCAAAGGAGCAAGAGATGAATAGGTGATTTATGTCTTCTTCTTCA
CATCTGCACATAACACACCATCTCGGTTTCAAATATAAATTAGGTAAATGTTTGACCAACTGATCGGCAG
TATTAATACAATCATATAGGAGAGACCAAATGAAGAATTTGCTCTTTTTGGGGATACAAGGCTTCTAAAA
ATTAGTAAAAATTGTATGGTCAAATATAACTCCTCCCTCTAGGATCACTGCAATGAATAGCTTTCTTCAC
CGAAGCAACAGAGAATTACCATCAGAATTCAACTTCTAGATAGGATTGTCACAACTATCACCAATTAATG
GGGTAGAGAGAGAAGTTGAGCTTGTTCCATAGAGAGATTTCCCAGCTTCTAAGCTGTCCTCTGGGTAAAA
GATCCCAATGAAGGGTATCAAGGTTCCATATCCTTAATTAAACTGTTATGCTTAGTAGAAATGGCATATA

ACTTGGGATAGTGCAAGGATAAATGGCAGTTTGAATGCCATAGGCTGTGCCAAAAGGAGAGATTGTCACC
ATTTCTGATTTTCCACATAATTTGTGTACGAAACCACTCAATACATTTAATAATTGAATGCCAAAGGAGC
TCTACCGCTACTGAATTTGCTTTTTGTAGGGATATCTCCTACAATAGGGCAATCATATTTGGCCAAAATG
ATCCTCTTCCATAAGGGGCTTTCTTCATAAATGAACCTCTAAAGGCACTTGTTAAGTAGTGCAAAATTTG
TATCTTTGATCTGACTAATCCCAAGACCACTTTTCTTCTTTGGCAATGAAATAACGAACCATCTTATGAG
GTGTAATTTGTGGTTTTCTTTTGGATGTTTCCAAAGGAAATTTCTCCAGGTCTGTTAAATGGTCTTGTAG
ATGGAGGTTGGAGCTTTGAAAACTGACATTTGATATGTGGGTAGGCTAGTGAGCGAGGATTTAATCAGAG
TGATTTTCCCTTCTTTTAACAGAAATGAATACTTCCAGCTATAAGTTTCTTTTGGATCTTTTCAGAAATA
TTAGTCCATCAGCTGTTGGTAAGAGGTTTCTCCCTAAGAGGCACTCCTAAATAATTAGTAGGGAGGAATT
GTGTGTTGATCATACCCCAAATATCAGCAACTCTGTTAACTCTTTGGGCATTAACGTTGATAGGAGAGAT
AATAGATTTGTTTAAGTTAATGCTGAGGCCTAAAGCAAGTTTAAAAAAATGAATGACATACCTCAAATTT
TCTAGTGAACCGTCAGGAGAATATCATCCACAAATAGGAGGTGGGTAAGATTTTAACTGTCATTGAATCT
AACCACCTTGATTTTATTAATTTTATCAAGAGCTTCGAGCAAACGGCTGAGGTAGTCCATAGCAAGAACA
AATATAAAGGGAAAGATTGGGTCTCCTAGGCGAATACCACGATTTGGTTGAATTTTGCCTCTTGGTCTCC
CATTAATAATAACATAATATTGTACATTGCTTATGCAAGAGTTGATCCATTTTCTCCATTTATGAGAGAA
GCCTTTTTTCATGAGCATATATCAATGAAGGGCCAATTTATTTTATCAAAAGCCTTCTCGATATAAAGTT
TTATCACATGCCCTTTAACCTTTTTCACTCTCCAGAAATCGATGGCTTCATTTGCGATCAAAATTGCATC
AGTTATCTGTCTTCCCCCAATGAAATCCATTTGATTCTTGGCAATAATATTTGGGAGAGTTTCTTTAAGT
CTCGGCTAAGACTTTGGCCATAAGCTTGTATAAGGATGTTGCTAAACTGGTAGGCTTGTATTCAGAAGCA
ACGGAGCATTTTTCTTTTTTCGCATTAAGAGCAATGTTAGTGACATTAACAGCTTTGTTGATGATGCAAT
TATCATGGAATTCACTAAAAATTTTAAAGATATCACCTTTAAAGAGATGTCAGGCTGAGTTATAGAACTT
CATAGTGAAATCGTCAGGCCCGTGACTTTTGTTGTTTGAGAATGATTTTAGAGCTCTAAGTATCTCCAAT
TCAGCAAAGAGAACGCAAAGATCATCTTGTTGTTGAGAGGAGATAGGAGACCAGTAAAGGTTTTCAATGA
GGCAAAGATTATCATCATTGTTGTTTATATAAATGTCATTAAAATGTTTAAGAAATGAGGCTCTTCTTTG
TCTGCCCATGCATATCTTATAAAGAAATATGTATTTTCATCACCATCTAAGTTCGAGAGCCTTTTGCATT
TTTTAGACCAAATTTGAGCTTCTTTATAGTCATATTGATTTAGCTCAGCTTTTAGTGAAGTTCTACACAA
ACTCAAGATTCAGTTAAAAGATTATCTGCTTCATCTTATCGATTTCATTAATTTCTTTAATCCAAGCCTT
TTATCCTCCTCATTGATGCTTTTGTTCCTTTTGCTCCATTTCTTTATGGTGAACGATAGTAACTTGAGCC
AGCTCCTCATGAAAGAGTAGCCTGGGTGGCCAGATTGGTGAGTGTTTGTCCATCATAATTCAATGTTGTT
TTTGAAGCCATTTTCTTAATAAGATGATTAGCAAATCTGTAAGGAGGACCCCAATTGAGATTGGAGGATT
CTAGCAAAAGCGGGAAGTGGTTCGAAGTAATTCTGAGAAAAAGTTTTGGAATAATGCATAGAGGAGATTC
TCTCAATCTGTAGTATAGAAAATCTGTCAATTCTCGAAAGTGTTGATTGGACTCTGAGATTAGACCAAGT
AGGGTTGGTGAGGCATATTTTAAAATTGTTAGTTGATTTAGCTCAATCATATAGATAACTATTATGCCCC
ATATCAATGGCACCCATGTTAGAGCTGCCATTGTTTACATCATTTTCAACATTTAAATTAACATTAAATT
CTATAAGAGTCAATTTCAGATTGTTAGTTCTCAGTCGGTCAACAAGTTTCTTCTTGAAAATTCCTTCGTC
TCCATCTTCATTTTTCTCTTCTTTTTCTTAGCCTTCTTGCTCACTAGAAAGCATCTGTCCCGCACTAATG
CATTGGTGTACTGAGGGAGAAGGGGACAATTGTTCTGGGCTTGATATGAACTCATCAGGCATATGGGAAA
GCGACCTAAGATTTGGGATAGTTGCAAATATAACAATTATATTCAAAATAATGAAATATATAGTAACACT
TTTAAAAAAAATTGTAAATATAGCAAAATTTGTCAAGATCTATCAATGATAGAAATCTATCGTTGATAGA
CCATGAAACAAATGTTGATCTATCACTGATAAACCACAAGAGTCTATCAATGATAGAACTCTATCACTGA
TAGACTTTGCTATATTTGTAATTTTTTAAAAATATTGCTACATACTTAATAATTATTCTAAAGATTCACT
GTAAGTTTTAATTCATGTTGATTTTTTAAATCTTCTTTGGCTGAATTTTAAGCCCACACTGTTTATTGCT
CATCTCATTCTATTATTTTCTTATGAATGAATACAATAGGTACAAAGGATGAGAATAAATAGAATACAAT
AAGAGTAAGAAAGGAAAACATATAGGAAATAAAATCTTTCATATTATTATTTTTAAATATACTCTAAGGG
CAAAGCCCCTACTAATTCTAACACGTATAATTAAATATGTTTTAAAAAATAGTAAAATATTGAAACTATT
TAAAAAATATAGTAAAATCTTTCGCAACTCTTCATAATGTATTTACATTTTTTTCTTGAACTTTTTGTTA
TATTTTATAAATAGCTTCAATATTTTGTTACTCATAACAATTTTCCTATCATTAATTTACTTAACAGGCA
AATATAGACCCTAGTCCAATTCATCGTGCTGCCAAGGTGTTGATCACTTCTCAAACTGAAGATGGTGATT
TTCCACAAGAGGTCAATACCAAAAATCCCCTTTTTTTTCCCCCTTTTTTTCCCCCTTTTTTTTCTCTTTA
AAGCATAAACATAATCAGCATATATAAGTATATATATATAACAACTTCTAAACGTTTCTTACTTAAATTC
AATTATGTTGCTATACATTGTATGTGCAGGGAATTACTGGATCATTCTTCAATAGTTGCTCCTTACACTA
TGCAGCATATACAGAAGTTTTTCCAGTGATGGCATTGGGAGAATACTATAACAGCATTTTCCTGTTTTCT
GAGATTTGAGTTAATTAAACCTATAAACTACATATTAATAAAGATGCAGACTTGGACTCAAAAAGAAAAA
AGAAAAAAAAGATGGAGACTTTTATATTTTTATTATGAAAATGGGATGAAGTTCAGAGGATGTCTAGAGC
TTCTCTCGATAAGTACATCAAACTATAAAGAAATGGGCTAACCATCCTTTTTTTATATGGCAAGAGAATT
AAGATGTATATCATAAAATGTCGATGTGAATTTTTTTCTTTTCAAATTGACAAAGTATAGAGTACAATAT
TTGCTAATTCCAACTGTCCAAGTCACTTATGCTTATTATCTTCGCATACATGTATCTCAAAGAAAAAATT
GCATATATAACTCTCATAAATTAATTCACATATAACTTATTTATTTAAAATATGTATAATTTTCAACATC
CTTCCTTAAATATGTTTCCTAAAAAACTTCAAGTAAGGTTCTTAACTTGTTAAACACATTAAATTTTTGC
GGTTTCGTAAAAATATCTGCAATTTGATCTTTAGTCTTCACATATTCAACTTGAATCTCCTTCCCTGAAA

TGCAATCTATGATGAGATGAAATCTGTTATCAATGTTGACTTATTGTTGACACAGGATTCTTTGCTAGAG
CAATTGTTGACTTATTGTCTACATAGATCACAATTGGATCATCTTGCAAAATTCCAACTGTCTTTAATAA
ATTTCTTAACCAAACTACACGACAGACAAATGAAGCTGCAGCAACATATCCTACCTCACAATTAGTTGAT
GATGTTACAGTAGGTTGCTTCTTAGAACACTCCAAGTAAATACAGTATTATCAACGAAGAAAACATATCC
ACTAGTGCTCTTTCAATCATTAATATCTCCAGCCTAGTCACTATCACAATAGCCTTCAAGCTTGAATTCT
TTAGATGAAGAATAAAATAACTCATAGTCAAGCGTACCCTTGAGGTAGCAAAGAATTCTCTTTGCCACTT
TCAAATGATTAATTGTAGGAGATTCCATAAATTGACTCACTAATCCAATGCTCAAAAGAATATATGGTCG
TGTGCAAGTCAAATATCTCAAACTCCCAACCAAACTTTTGAAATATAAAGGATCAACATAGTCTCCTTCT
TCATATTTGGACAGTTTGATTCTAGTTTCAATCGGAGTTGTCACAGGCTTAGAATAGATCATATTGAACT
TCTCTAGAATTTTTCTAGTATATCGTTCCTGAGAGATGAAAATATCTTCCTCTAATTGTCTCACCTCAAT
GTCAAAATAATATGACATCAGCCATATATCTATCATTTCAAATTCTTTAGGTCATCGTCTTCTTGAGATC
TTCAAACATTCTTGAACAATTTACTGTAAAAATTAAGTCATCCACATAAAACAAACCACAAAAATATCTC
CATGACCATTTGTCTTAATATAAAGAAAATGTTCATAAGGACACCTCAAATACTCATTATCAAGGAAATA
TTTGTTGATTCTGCTATTCCACATTCATGGTGCTTGTTTCAATCCGTAAAATGCCTTCTTCAATCTTAGA
ACTTTATCCTCTTGGCCTTTCATAGAATAACCAAGAGGTTGTTCTATGTAGACTTCTTCTTCTAGATATT
AATTCATAAATGTCGATTTGACGTCCATTTGAAAGGTTTTTCAATTATTTGGAGCAACAAGCGCAATTAA
CAACCTTATCGTTTTCAAATGAGCAATGAGAGCAAATACTTTATCATAATCAATGTCTTTTCTTTGAGAA
TATCTTTTTACAACTATTCTTGTTTTGTATCTCTCCACTTCTCTCTTTTCATTTCTTTTTATCTTGAACA
CCCATTTAACACCTACTGCTTTCTATCTATTTGGAAGAGCAGAAAGTTCCCACGTATCTCCTTTTTTTTT
TTTTTTTTTGCTTTTATCTCTTAATGCATAGCAATCTTCCATTTTCCATTTTGCGAAGCTTCTTCAAAAT
TCAAAGGTCTCCTTCACCAAATAGACAAAAAAGAGTAAGGTTATTAAAACTTTGACTTAACTCTTCAGTT
TCATCATCTATGTCTCGTAAAAACTTCTCGTGCCACGAGGCCCTTCACTTGAACTTGCAGATGATGAAGG
GTGTGTTTTATTGTGGAGTGATCAGCAATGTTGGTGGTGTTAATGAAGAAGCAATGTCACTAGACTCATC
ATTATCATCGGAAAAAATAAAAAAATTATAGTCTTCCGTTCGTCATTCCAACTTCATGATGCTTCTTAAT
CAAACACAACATCTCTCTGTTTATCATTCTCTTCTTTGTAACATGATTATAAAGCTTGTAGCCTTTTGAG
CTTGCATCATATCCAACAAGAACATATTTCTCATTTTTATCAACAAGCTTACTATGTTTTTTATCTGGTA
TATATGCATAAGCCATGAATTCAAATACTCTCAAATGAGCAATTGATGGTTTTCTTCCTGTCCATGCTTG
TTGAAGAGTTTTATTCCATAAGCTTCTAGTAGGGGAACGATTTGACAAGTACACTGCACACTCAACAACT
TGTGCTCAAAATTCTTTTGGTATATCTTCTTGCTCTTCAACATGCTTCGAGTCATGTTAAGTAATTGTTC
GATTCTTCCTTTCAACAACATCATTTTGTTGAGAATTAAATGGAACTATCATAGGTCAATGAATTCCATT
TTCTGCGCAAAAAGTTTTGAATTCATTTAAAGTGAATTCACCTCCTCTATCTGATCTCAAAGCTTTAATG
TAATAACTACTTTCTTTTACAACAAGAGTTTAAATCTCTAGAAAATGTCAAATACTTCTGATTTCTCCTT
GACAAAGTAAATCCAAGTTTTTTCGGTTGAAATGATCAATAAATAATATGAAATAAATATTCTTACCCAA
AGAACTTGGTTTGATCGGTCCACAGAAAATTTTATAAGCTTAGCTCTAGCCTCCCTACCTTGTAACCTTT
GCTCTATATAAATTTATTTTGTGTTCAGTCACATAGGATATTCACATCGTAGATACACCTAGGTGAACTG
AAACATGTTGTTTCCTGCTAAAAAAATATGAAAGAAAATTTGCAGCTATAAATTGTTTTGTTTTGGAATT
AAAAAGAAAAATTGATGGACTATTAGGAATATTATAATTGCCAAAAGAAATAGAACAAAATGAGGGGCTA
AAGAGTTGGGTGGAGTTTCGGTAAAAGAAAAAAAAAACCCTTAAAATAAACTTTAAGTTCAATCGCACTC
TCTATATAAAAAATTCTTTTCTTCAACATGGTCTCACTTGAGTTCTTAGGTTTATCGTATAGTAGACACC
ATCTTTTCAAAGACTATACGTCTAGTACTATAGTGTTAAATTTTTAAGGCTTTGTTTTGGACTTTCCTCT
AGTCCTGCTTGTATTTACTCCAAATGAATAAAATTCGGTGTCTTGTTAGAGTTCTAGGGTTCGCTCTCGA
GCTTCCCTTTAGTTCTACTTTTCACCTTTTTTCTTTTTTTTTTCATTTTTTTGGAAAAGATACTTATGAT
ATGGAAGCTAAAGAAAGCCACCAAATCACTGATTTTATTGAAAGTAAGAGGTTACACACGCGGGAGATAA
GTCCACAACGAAGAACAAAGCTTTAATATTTAAAGATATTGTGTGTATTTGTTGATGACCAAATGCCAAT
AAGATTGCAAATGTTTTCCCATAAGTTCTCAAAGCTCAACCTCTTTTCTGTAAATATTTGGTTGTTTCTA
TCTGTCCAGATAGACCATAGAGTGACCACAGCAATGTTGAATATTAAAGCTTGTCTACTTTTCACCTGGT
CTCCTTTTCAAAAAAAAAAAACAAAACACTATATTATAGTCTCGTTCCTCTTTGAATGGGTGATGGACAA
TGACCTTGTCGAAGGTTTAGCCGTCACAATGAATTCTTGTTCCTACTTAGACTAGTTATAGTGATAGTAT
GAGGTTGAACCACATGAAACAGTTGCATTTTTCATTAAAAATACTTAATGTTCGTACGTAATGTAGCTAG
AGGGGTGTTAGTTTGTTGTTTTGTAAGAAAGTATATAAGTGCATAAAATTTAAACCAAGAGAGATGAAAT
TAAATGTGGAAATATAATGCAATGAGAGAAATATTGAGAATTAGAGGAGTCTAGCGTAGATGGTACCTGG
TATAATTTTTAATTTTAATAATTCTAAAGCCTATAACAATCTTAAACCAATCCTTAATATGTATTAGTAT
TTTTTTTCTATTTAATATTCGTCGATTAACTTACTAATTTTATAATTTTAAATAATTATTTTAAACATTA
TATTAGGAATGTTTTTAATAAATATAATTTCGTATTTCTTTACTAGAAAATATCATTTTTTTGAATTCTA
GATTATTTAAATAAATTTAATAAATTAACTTTGAAAAATTGATGATTTATTGAATGGACCAAATTGCATT
GTAAACTAACAATATTAAGCTTTATAATGAAAGGACAAAATTGACATAGAAAGAGTTTCTCTATATCTAG
TTATTTATATATTGTATGATACATATAAAAAAAGAGATACTTTCAAACATAGCAAAATAAACCACACAAT
ATAATAAAATTTTATATTTATCACTCTATCGATGCATACTAGTGATACTCATACATACTAATAAAAGTCT
ATCAATGTCAACAACAAACGAAAACTATCGTATTTTCAACAACAAAAAAGAAGAGCACCACCAACTTTTA
GGAGAACATATGTAATTTGATCGGATTATGGTCTAGCCAGACACACTCTTTTCAAAGACTATAAGCATGC

ACAATTTCTCTCAACTTTAATGCTTTGATACACTAATTCAGTTTGTACCGGTGTTTAGTTCTAGCCTCTT
GGCTACCAACTTGTAATACTTTCTATTCAATAAAATTAGTGTTGTAGGGTGCTAGAAGGAAAGCCCGCTT
GCGAGGGCTATCTAGATACACCTTATAACTCATTTATTTTTTCAGTAAGAAAAAGATAGAAGTCTATCAA
TGTCTGTATCACTGATAAAATCTGAAATCTTGCTATATTTTGTAAATATTTTTTACAATAATTTTGACAT
ATCACATATACTCACATTCACTTTAAGGGTGAAATAAATTGAAAAATCAAATGTAAGAAGAGTATTCATC
GAAGAAAAATAATGATCATAAATTTTAAGGGACCATTTAATTTGTTTTGATTATATTCACCTACTTCCTA
CCACACCGGCATACACATAACGTGTGGATATGGACTTTAAGCATTCAATAAGAAATTGACGTGTAACCAC
TGGGGTTTCTTCTTCGATTATCGATCATTATTGGCATTACTACATATCCAAACATAGGGACCAGGGGTGT
ACATGATTGAGTTGATTGTATGGTGGAAAATTAGCAACGTATGGTTTTTGCTTTTGATTTGTCTTATCAA
ATAAATCGATTTTTTAAAAAAGGAATTAAACTCTGACACCAAAAGTATGTAGCACAAATTAATATATCTA
CTGTTACATTATTGCAAGTCATCAGATTGGTATATTTAGAATAAGAATTGAAATGATATTGATGTGATAG
AAGCGGATAATTAAATAGAAGAGATGGTTTGCGGTGCAACAACTTTGAATTTAGATGTCTGTTAATATTT
TATGAACGAGGACGATCGATTTTATTCATTTTTATATCAAATGTGCTCCTACTTTGAAGTTATTAATTGT
TCATAACTTATGCCAAAATTGGATGTTATATGGTTTTGGTAAAGATTCTCCATGTGGAGGCGAAGACAAC
GACGTATATTTCGAGCCCCCAACAAAACAATCTTTGTGCGACCATCTTTGAAAATCATTTTTGCATAGTC
AAACCAATTATGATTGTTGGTATCTATCTTCAACAACAACTTCGGCAACTAATTTAATTTTGGGATCTAA
TAACCATCTCCAATGACAACTTCGACAACTATCTCCACGAAATCAACTCCGACGAATAATTTTGGTCTCT
ATTTTTTGTGCATGATCAATTTGTATGTTCTATGTCTGTTAAAGTGTGTTGTAAAATTGTTGATTATATA
AATAATTATATTTTGTCTAAGTGCAATAAAGAATTTGAAAAGTATGTATGGTATTAGAATGACATAACAC
AAAGTTAAAAGGAAAAGGTAAAAAAAACTTAAAAACAAGAGAAATCATAAAATAGAGATTTCTTTTCTAG
AGCATTTTCTAGTCAGAAATGATGAAAATTCGAGATTTAATTTGTTGTTTGATAATCCCCCAAACTTGAA
GGAATTGAAATTCTAATTGTTGAAAAAAATAAAGTCATATTTCATATTGGCTTTTTAGACGAGGGAGGGG
ATTCAATTAGAAAAACTAAATTCACTTGCATAAATGGGTATTTGAGCCTAGAATATACTACACAATAATA
AAAGAAATCCATATATAGAACAAAATTCAATAAATGACTAAAAAATCTAGATGCAATAACTACAAGAAAT
TGGGGTTCTTCCGACGCAGAGAAAGACGTCGACAGAAAAATCGTCGAAAATGAAAATATCTTCTGACGCC
CAATGTAAAACGTCGGGAGAAAACGTTATTCCCAACGCTGTAAAATGAACGTCGAGAATATCATCAGGAA
TTATATATTTTCCCGACGCATACATGATGCATTGGGAAAGAACCGTCGGAAAACATATGACATTAATTAT
GTCATAGATGTATTTCTGACGCCATACTTCATGCGTCGGGGCCGACGTCGGAAATTACATGTTATCCTGA
CGCATCGTGTATGCGTCGAAAAAGAACCGTCGAAAATTAGGCCACGTTAATTATGGCAGACATGCGTTAG
GGCATCATGCGTCAAGGACGGCGTCGGGAATAAAGAGCAGTCCTGACGCTATTGTTGTGTATCGAGAACG
GCGTTGGGAATAAGGAGCATTCCTGACGCCCTGAGTGAGATCTCCCGACGTTTTCAAGCTGCGTCGGGAG
ATCCCCTATAAATTTCATTTTAGTTCAATGAAATTGAAACGAAGAGGCAAACAAAAAAAAAGAGAGAGAG
AGAAGCAAAACGAAGAAGGATTCGCCGTCGTGCCCTCGTCGTCGTTGCCCACACCCCATTGTCCCTTTCC
GCCGTCTGCCGCCATACATTGAGGTAAGTTTAAAATTGTTAATTTTTAGCTTTATTTAGATATTTTTAGG
GTTTTTTTTTTAATTTGTTTTGGAATAGATTTTTGTTTGTGAAATGTATTGTTGTATTGAATGTGTGTTG
AATTGAAATTTTGAATGTTGTTAAATTGATTTGAAGTTGGTTGAATAAAAATTTTGGGGGAGGGGGGGTT
TCAATGGAAATTTGAAATGGGGGTTTCAAAATAATTTTTTATTTTGAATTTGTTTCTATTTGGGATGGTT
TCAAGGGTGGTAGTAAGATAGCTTGTAAGGTAGCGTTGTTGGAAGGGGTGGGTAGGATGCGAAGATTGAA
GTATTTTGTTGTTAATAATTAGGTTGTGTTGGTTATCCTGCAGTTATGGTAGCCTTTGTAGTTTGAAGTT
AGTTTTAATGAAAATTTTGAGAGATTGTATATTAGTGAAAAATAGTGAAAATTCAAGAGACTAAGTTATG
GATATGTGAGATGAGAGAGATATGTTTCTAATCAAACTTGTTTATGTTTTAGGGACTTAAACGATGAACA
AGGGTTGGATGAAACTTAGAAATAAGCTCTCCCTTGAGTATAGAGAGGGAATGACCCAATTTTTAGAATT
TGCCAAGTTTTACGTTGATGCCTACGAACGATTAAGGTATCCATGCAAGAGATGTACACTACAAGAGATG
GGGGCACTCCCGACGCACAAATATGTCGGCAAAAATGAAAAGTTCGTCGTGAAAGAATATCCCGACGTAC
AAAACGGCATTGGAAAGAACGTCGGGAGAAATGCGTCGCGAGAGGCTTTCCCGACGCATACCTTGGCGTC
GGGAAAACCTCTTTTCCGACGTTTTTTCCCGACTTTTTTATATATTTTTTAAAATATTTTATTTTTACTT
TTTTCCTTATAATATTTTGCTCAAACATTCACAATGATGTTCGGATTGCCCAAAATATTTTCGCGATGTT
TTGGATTAAATTAAAACAAATTAAATATAAATTAAAATAAATTAATAAATATACAAACACAAATTAAAAA
AATAGAATTAAAATTAATAATACGAATAAGTTGACTACAACAAAATATAGTTCTCAAAATAAAAAAAACA
TAAACATAATGAAAAGTCTCCCAACGAGGTGCGTATGCGCGTCATTCTACACCTTCAGTACTAAAAATGG
TATAAAAATAACTAAGTTTAATACATAATCATATATTGCAAAAACTTAAGAATAATAGAGACAGATACGT
ATCGCAGAGCTAGGGATCATGTGGTGGTCCCTGTTGTGCACGAGTTAGTTCTTCTATCATCTTTTTCATA
GCTTCCACTTGTGAAGCTAATGCTTGGTGATTTCTATCTTGTACTTCAATCCGTTTCAAAGCTTCATGAA
GTTTAGCTTGTAATTCAATTTAAATTGGAGCTTATTAGAGGGTGTGGAACGACATCTACTCACTATTAGA
ATATCTCCCTACTACACAGAATAGGTGTATCATGGAGAGTCATTAAGCTTTAGAGAGGTACAGAAAACTT
TGAGAAAGGAACTAGTAGTAACCTTACTACACATAATGGGTGTATCATTAAAAGACCGGTTGTGCATCAT
GTCACTAACGACTTCATCCACGATGTGGATGAACACTTGTCACATGCAAGCGACGACAATGAATTATAGT
GACAAACACTTGTTAATAGGCACATTCCGATGACGATCGCCCTTGGAGCGGAGAAACCTATTTTTCCACA
CGTCGTTCGCTTCAGCCAGGTGATAAGCATGTGTGTACGAAAGACATTTTCTGTCCGCTACCTTAAGTGG
GCGGACGTTGACAAATAATACATTGAGGTCATCAAGGGCGACACTCCAGATAATTAAGTCCACTACACAT

TTATGATTTCATTTGAAACATATCTAAGTTAACCAATCTAATTTGTTATTCTTGTAATGTATAGCGCGGT
TTTTGTGCTTGATTTTAATGATCAAGCAATGAATAGGTTTGTTGAATATCAGATGCTCAGCACCTTTAAA
GAGTTTCGGGCTGACTGTCATAGACACTTCAAAAAGTACAGCGACCCCGAGGAGGCTCGTGCCAACCCAC
CCAACCTATTGGTAGGACGTGATGAGGATTGACGCTTCCTCTGCAACCACTATATAAGTCGTGCATTCCA
GGTGAACAATTGAACCATTTTGTCATGATTAATTATGATTTCAACTTTTAATATGTATAAGAACTAAACA
ATATGATTGTTTTCATGCAGGAGCAATCACTGACGAACAAGGTACTAGATAGAAACATCTTTACAATCAT
AGCAGCGGGTCAAAGTCATTTCTACAAGACAACACGAGCTCGCTGAGAAAAAAGGGGAGCCAATCGACCG
TGTTGAGTTGTTGCAGGAAACACACGTTCGAATCGGGACATTCGTGTCGCAGGACGCAGAGGATGCGCAA
TGTAAGTTATCGAAGCAACACTTATGCCCTTATTAATTATCCTCTTTTTGTGTTACCTAACTTAGTTTTT
AAATTTGTTGTAGAATCAAATGTTGGAATTCTAGTCCCAGCCTACCCCAAAGGGTAGTCAGTTGCTCTTT
GTGGATGAGATATGCAATCAAGTATTGGATAAACGACCAAATTACTTAAAAGGCCTTGGTTGGGGACCCA
AGCCGAAGGTCCGCAAAACAACGAGTGCGAGCAATTCCAGGACGTCATGTCTGCAGTCCACAGAAAAAGA
GATTGAATTACAAGCTAAACTTAATGAAGCTTTGAAACAGATTGAAATGCAAGATAGAAATCGCCGAGCT
TTAGCTTCAAAAGTGGAACAAATGCGGAAGCTGATACAAGACTTGACTCGGGCACAACAAGACCACCACA
TGATACATAGCTGTGCGGTACGTATATATGTCTCCATTATTCTTAAGTTTTTGCAATGATAGTATATAGT
GACAATATGGATATATGTACTAAACTAAGCTACTTTTGTATCATTGTAGGACCTGCAGTGTAGAATGGCG
CATACGAGGCTCGTTAGAAGATGTATGACTTTCTTTGCGCTTTTTTGTATTTTGAATACTATATTTTTTG
TATTATGAACACTGTTGTGTTTTTTGAACTTCTTCGCATTATCAATATTAAATTTTTTATGTTCAATTTG
TGTTTGTATTTTCGTAATTTACTAATTTATTTTGAATTTCCTTTAATTTAATACAAAACGAACTCGATTA
CTTCACAAAAATAGAGAACTTGTTTGAGGAAAATATTTTAGGAAAAAAATTTAATATTTAAAAAATACAT
TTAAAAATAAATTCTTGATGCAAACAACGTTGGGAATAGGTTGCACACGACAAGTTAGAGATAGTTTTTC
CCGACGCACATCCAATGTCACCTATACGTACGTCGAGAGAAATCATTCCCAATGCACAAAGGGAAGTTGG
AAATGATCACATTGGGAATTACTAATCTCGACGCGTTACACGCGTCGACAAATCAGACGTCGAGATAAAG
TTATTCCCGACGCCTTGTTGGTGATGCGTTTGAAATACTTCTCCTAACACATTTCTTCCAACGACAGTCC
CGACGTAGGAAACGATGGTCGGGATATCTTTCCTCACATTTTATTGCGTTGGGAGTCCCTCTGTTTCTTG
TAGTGAACCACCATTTTGGATGCTTTTTTCTAAATTTCTCAAATTAAAAGTGATCAGTGACATAACAAAA
TATTTCTTCTCAATGGTGCAAGAACGTGACAGTTGTCAAAGGAAAGCCATAAAGAAAGAAGAGAAGGAAA
CCAGAAAACGAAAGAAAGAGAAAGGTAATTGGAGGTACCTTGGTAGGATTGTATTTAATTTGTATCTACA
AGGGAAGATGAAACGATATGTTGAAGTAAATGCAATAGCTTAGAGTAAATGAAGAGTTCAGAAGGAGTCC
GACAAATGTCTTCTAGTCTTGACTTTCGCCTGAGTTGGAGGGGGAGTGTGAACGACGGAGGAGAAAAAAC
ATATCATTAAAAACTAATTATTGTCACAATTTTATTCATAATGGATTCAACCTAATGCTCAAAACGTTGT
TAAGAATTTTACATAATCACTTTTAGCATTCTAAAAAATAATTTTAAATACGGTCTGAAGGAAGGATCAA
ATTACATAAGTTCGTTCAAATAAATTGTAAAATAAACCATGAACTGATTAAACAAATTATGGAAAAAAAA
ATAATTAGGACTTTCTTCCACCGACATCATATCACTCCAAATATATTGTAGACTTGAAGCCTTTATGAGT
ATACAGTACTTGGCACTTGAGTAGGTATCCTGTTATGCATTTGCCAACTTGAGTAGGGCACCTATGGATG
CCAAGCTTTCCACGAGTCATCAAATGGTTCGGCCACGCCTTCACCTTTCAGAAGCTTAACAACTGTTAAA
ATTTTGGTTGGAAGGAATAATTGCACAATCAATTGATTGGGTCCATCTGAAAAAGTAATGTAATCCTATG
TAATTATTTGATTTTCTTTATTTCCATTATCAAGGGAATTTTTCTATATAAGTGGTGACCTGACCATTCT
ATTGAGTATAGAAAAATTAGAGAGCATTTCTTCTAAAATCAAGCAATCGTAAATTCCATTTCAATCTTCT
ATATGATTGTAAATAGGTGGAAGTGGGCATATATATATATGAGTCGAGAGATGGCAAACTAAACATATGA
GAAACTTTGCACGATAGCAAGAAAACGAATATTGATATGTATAAACTTCATGGCCATTATTACGTTGCTT
TGTTTTTTCTTCCCTTCACTACCTACAAGTTCTAGTCCTTTTGTTTGAAGTTTGAAGTTTTCTTTGCGAA
TCGAAGATCGATCATGTGGAGGCTTAAGATAGGTAAAGATGGAAATGATCCTTACATATATTCCATGAAC
AACTTTGTAGGAAGACAAATATGGGAATTTGATCCAAATGCAAGAACTCCTGAAGAGCAAGCTGAAGTTG
AACACTTACGCCAACGTTTTGCAAAAAATCGTTTCAATGGTCTTCCTAGTGGTGACTTGCTTTGGCGCCT
CCAGGTAATGTCATTTTCAAGTTTTTAATAATAAGAATAGGTACGTTTAACTTTTATAATTTCAAATTGA
CCTTTTTTTTTAAAAAAATATGATGGGTTAGTAAGTGCACCCGGATATCTTCACTAGGTGGACACTCTCT
TAGCACTCTCATTATTCCTACTTCATTAATAATATCACCAAAAAATGGAAAATACATTACTTTATATATG
AAAAATAATCTGAGCCCAATAAAGTAGAAATGATGATTGTGCTACGGAGGTGTCCGGAGGTGTCCACCTA
GTGGAGATGTCCTGGTGCACCTACTGATTCATTTCGTATTTAAAAAAAAAAAAAAAAAAAAACTCACGTTA
GTCTCCTTAAGACATTTTACTCACCTTAATGTTTGAATTTCATGAGTAATTTTCTCTCATGATAAAACAA
ATTAACTTTCTTGTAGAAGAATCACCTCATCCACAATATACGACGAACTATACTTTGGAAAAATAAACCA
AATGTGAATAAATTAAAAAAAAATGTTTTATGAGAGAAAGTGATTGTAAAAAAGTTCTTAATAAAAACAA
TTTGAAGAAGAAACATATTTATATATTATTCTTAGTCATTCTAATAGTTGTTTCTCTTCTTTTCAAGAGG
GTTATTTTCGAAAAGATAAATGAATTCATGAAACCATGCAACTCTATACAAATGGTCATATACCATTTAT
TCCAACGCAAATTGGAAAAGTAGTGATTTTAAAAAGTTCTTATTCCTATTTGTTACATTAATTCAGCATT
TTTAAAATTATTTTAAATATAACAAAATATATTTAAGTAAAGGGGAATTGTTAGTAATAAAACATTTGAC
AAAATATTTACAACTCCTAGAAAAATTAAATTAAGTATAATAAATTTTATCTTTGAGTGGTAATAGTTTA
TCGAATTTTTCTATTTTTGAAAATTTTCTTAGTTAAATAATAAAAATATTTTCTCTCTCTCTCATTCTTT
CTTATTTACCTCTTACTCTCCATCCCGTGTTCAAAATTTATATCTGCAATATATATAAAAGAAAAGAATG

GTTCGTTACGCAAGAAAAGGAATACATATCCATTACTGACTCACCTTGATATTTTTATGTTATTATATTA
CAAATTGTTATATGAAATTGAATGGAAAAACAATAATTAATTGAAATGATATGGGTGCAGTTTCTTAGAG
AGAAAAAGTTCAAACAAAGTATACCACAAGAGAAAGTTGAAGATGGAGAAGAAATAAGTTATGAAAAGGC
ATCAAATGCAATGAGAAGAGGAGCACATTTCCTAGCAGCCATACAAGCAAGTGATGGGCATTGGCCAAGT
GAAACATCAGGCCCTCAATTTTACTTGTGTCCTATGCTAATTTGCATCTACATTATGGGTATAATGGACA
CAATATTGAGTCCTGAGCACAAGAAAGAAATGTTGCGTTATGTCTATAACCATCAGGTAAACCATATTCT
CTTATCATCTAAATTTATAAATTTCAAGGTATATAGTGGTGAATAATGTAAAATACTATACAACAAAAAT
GCTCACTTTTTAAAAGTGAATTTAGCTCAACTAGCATATTAACTCTTCTCATTCTCTATTGTACTATTGT
ACTGAAAAAAATAAAAACAAAACTCACCTCGGGCATGTTATATAGAACTTTTTTGGAGGATCATATTCTT
TTTACCCATATGTTTCACTAAAGAACCACACAGGTGGAGAGGCATAGAGTAAATAATTGATAACTACTAA
TAGTACTTTGCAGAACGAAGATGGAGGATGGGGGTTGCATGTAGGTGGTCACAGTAACATGTTCTGCACT
ACCTTTAATTACATCTCTTTGCGTTTACTTGGGGAAGGACCTGAGGTTGAAGAACTTTCGAAATCCAGAA
ATTGGATACGACACCATGGTGGTGTCACCTCGATACCTTCTTGGGGAAAGACATGGCTCTCGGTATAAAT
ATCAATTTCATCTCCATATTGTAAATTTTTTAAAAAAAATTTTAATAACCTATTTTTATAAAGTCTAATGT
TAGTTATATAGTACACAGGTACATTCTGTTTCATTGATATACCAAATAGGATGTATAACAGTTGGCATAT
AATATACCAAAACATAAAAGCACACCTGCTCATACACAAATAAGGGTAAAATCATAAAAATAAAAAATTG
GAAATCTAGTTTTTTTTTTTCTATATGTGCAATCTTAGAATATCATCATTGCTATATATTCATAATTCCT
TTAATATTTCTTGTGTTAGAAATTTTAACCTATTTGTCACTTTCTTATTGACTCCATCCATGTAAAGATA
CTAAACGTGTTCGATTGGTCTGGAAGCAATCCCATGCCTCCTGAATATTGGATGTTACCCACTTGGCTTC
CCATTCACCCATGTAAGTTCAACTTTCAACTATATCCTAAACTAAATTAATTATAATATTCATCATATTA
TATACATCCATACAAGCCTTTTATATATATATATGAAACTTTTTTGATTTTGATCGATGATGACTTGATT
CATACAGCAAACATGATGTGTTACACTCGAATCACCTACATGCCCATGTCTTATTTGTATGGCAAAAGGT
TTCAAGCACCACTAACGTCTTTTGTTTTACAATTAAGAGATGAACTTCACACTCAACCTTACCATCAAAT
TGATTGGAAGAAAGCTCGTCACATGTGTGCTATGGTATGATGCTATATATCCTTCACTCCATATATTTAA
TTACTTAGCTAGGCCACACCCATTTATATATTATATATATGTGTGTATGATTAATGTTGCAGGAAGATTT
GTACTTTCCACATCCCATTGTTCAAGACTTGCTTTGGGACACTCTTTATTTACTTAGTGAACCACTCATG
ACTCGGTGGCCTTTTAATAAATTGATTCGTCAAAAAGCCTTGAATGAGACTATGAGACATATTCATTATG
AGGATGAAAATAGTCGCTACGTTACCATTGGCTGCGTTGAAAAGGTTCCATATCTCAACTCTTTGCTCAT
CGCAATTTTTCTCTTTATATTTTTCTTTTATTAACATGCATCACTTTTGAATTAGATCTAGCTATCTTGG
TATACCTATGGATCCCTCAATTTATAGTATCTTTAACATTCAACTAATATAATATATATAAGTTTTTCTT
TTTCGAAAAATGATATGATGAGTCAATAGGAGCATCCAGACATCTCCACTAGGTGGACATCCCTTAACAC
TCTCATCATCCCCGCTTCATTAATATAATTGGAAAAAGTACAATAACAAGAAACAAGGATGACAAAACAG
AGAGCATAAGAGTAGAGACAAGCCCAAAGCAAAGAGAGAATAAAAATCATTAAGATTACTGGCAATAGCT
CGAGAATAAAGGGGATCTAGAGGTCCAAAAACCCGTCAGAGCACAGATATCCTCCCAAAAGGTTGATGAT
CGAGGAGCTTTTTTCCTTGAATATACGATTGTTTCTTTCCATCCATATATTCCAAAGGGCCAATGAGATA
ATGTTAAATTTGATAATGTCCTTTTGGTGGCGTTGATCGATGTTGCAAAGCTCGGTGCAGCGGGAGGATA
CACTTAAATGTTGCATTTTTCAGCTGATCAGATTTTCAATTTTTCTCCAAATAGATCTAACTATTCTGCA
GTGACTGAATAGGTGACTGATATCTTCTAAGCTTTGTCTACATAGAGAGCACCCGCTTAGGCTAAGATTA
AGAGTAGGGACCTTTCTCTGGACAACATCAATTGTGTTAGTACATTTATGCAGTAGGAATAAACAAATGA
AAAATCTACATTTTTTGGGAATCTTGGATTTCCAAAGTTTTTTGAAAATCCCGGCATTGTTTGCATTGTT
CATGTCAGCTTCAGTGCAATGGCCTTTTTAACGGATGCAATTGAGAAGGTGCCATTTTGATTTAAGCTTC
AGATGGGGATGTCTTACATTACTTTGGATACTAAACTTGTTTAAATAGTTTTAGTTTATATATCTTCAAA
TGTCCAATTTTATTTTATTTTTAATAAATATTAAATTTAATTCCTCGTTTGAAACACAAATTCGTCTACT
TGGCATGTGTTAGCATGCCCTATTGCAAAGTTCTTAAGATGTGAAGGGCTCCCTGAGGCACTTACGTATA
CAAATTGCAACACTCATACAAGTGGAGTCTTACTGTCTGGTGTCTGGAGGGTGGTTAAGCTGGTAAATAA
GAATGAGTCAGATTTAATTGAAGTTCCCTTTTTTGTTGAGGATGCAAAAGATTTGATGAGGAACTCTTGG
AAAATGTTTTTCTTTAAGCATCTTCAGTTGTCTCTCAATGTGTTTTAAGCATTTTCAATTGTCTCTCAAT
GTGTTCTCGCTCACAGCCTAACAACAAAAGCCTTGAATGAGAGAATTTCTGATGTTCTAATTTTTCCATA
CCTTTAACGGTTCTCGTTACTCATATCTTGTGACGATGTCTTCTATAGTTCTGTTTGCTATCTTGTTCCA
CTAATAATAATAATAATCCTCAAAGTTATGGTTTTTTACCCACAGCCTCTATGTATGCTTGCTTGCTGGA
TTGAAGATCCAAATAGTGAAAGCGTGAAGAAGCATTTAGCCAGACTTCCTGATTATTTCTGGATGGCTGA
AGATGGTATGAAAATACAAAGTTTTGGTAGCCAATCATGGGATGCTGCTTTGGCCATGGGAGCTCTACTT
TCTTGTAATATCACACATGAAATTGAGACTGTCCTCAACAATGGCCATCAATTCATCAAGAACTCTCAGG
TGATTAATATATGCTAACTAATTTTTGTAAATAATTCAGACATTATTTTAAATCTTTGGATGGAACTCTA
AATATTTTTAGATTATTTAGGAAAGAATGTTTTTCAAGTATATTTTTGCACTTTAAATAATCTTTGGACG
GAACTCTTTGGTTAGATTTTCATAACATTGTTTTAATGACAAAACTTCTAAAAATATTTATAAGTATAAT
AAAATATCACGATCTATTTTTATCGTAAAACTTCTAAAAATATTTATAATATTTATAAGTATAGTAAAAT
ATCATAATCTATTTTGGTTGTTGGTCACTATTTTTTATCACCAACCTCTAGTTGTCGATTCCTCCGCTCA
ATCCCTGGTTAACAATCAGTAACCAAAAACTAGTGAGCCATCGTGGAAGTAACCTTAGTCCTTTTAGTCG
TTGATAGTATTATCAGTGGGCATTGGTCGATTGCGACAGCATGTAGGGGTCGCCATGGTATGAGTGAACG

TAGAAAGATTTAGAAGGCCAGGTAACTTTTTTATATATTATGGAAATTTTGGAGGGATAGTCTCATGGTA
GTATTTGTTTTTCAAAATTAAATATTTAAAAGATCAATCCAAACACACAGACTCAATATTGCTTTGTGCC
TTACCCAGACTTATAAGTTATATGTCGGGTGAAATTGTAGGTTAGAAACAATCCTTCAGGTGACTATAAA
AGTATGTTTCGCCATATGTCTAAAGGATCTTGGACCTTTTCAGACTGTGATCATGGATGGCAACTTTCTG
ATAGCACTGCTGAGAACTTAAAGGTGTGGCTTCAAATTTCACTTAATTCATTACTTTCCACACATTAATG
CATGCTATGACTATTAACAATATTTAATCAAACTTATAAAATTTCTAGTGTTGCCTACTTCTCTCCTTGC
TACCACCTGAAATAGTTGGCGAAAAAATGGAACCAGAACGTCTCTACGATGCTGTTAATGTCATCCTAAG
CCTGCAAAGCAAAAATGGGGGTTTACCACCCTGGGAACCAGCATCAAGTTACTACTGGATGGAGGTATTA
TGAAAAAGAAAAATATTTATATACATCCTGGGAGCTAACACCCATTTTTTTCATAGTTCAACAATAGTTT
GAATTACAAAACTTTCCCCTTTTCTTTTACTTTTAACATTTGCATTTAAAAGTGGGTAAAAAAATGTTGT
TTTAACCTCTTCCTCTTTTATTAATGTTATAATATATATATATAGTTGTTTTTTTAAAAAAAAAGATACG
TTTGGGTCAGTAGACGTACTCAGGTATTTCTATAGGTGAACATCCTTTAACGTCCTCATCATTCCCGTTT
CATTAATATAATCAAAGATAGTTCAGGAAGCAAGAAGAGTACAATAAAAGGCTAGAGATAAGCCCATAAC
AAAGACGCAAAAGGCTAAACAAAAACATGAATATTTAAAACAATAGATTTGGCGTTATAGTTTGCAAAGA
GCTTCGATCTAGTAGACCAAAGACTCGGCTGTGAGAGCATGAACATCATCCCAAATCTCACAAGTGGATC
TTTAATTTCCTCTTCCCTTGAATATTCTGTGATTTCTTTCGAGCCATAAGGACCAAAATAACACAGCAAT
GACGTTGAAAGTGATGGCCCCTCTTTTGCTCTTCTGATTGATAGAGCAATCCCTTTGCATAAAGATTTTA
CGCTGTGTCCTGTGTATTCTGCCAATGTATCAGTTTCCCAGCCCTCTTTCAAAGTTCCGGACAGTTTTGC
AAGAGATGAAAATATGGTCTAAGATTTCCGACTTCTTTTTTGATAGATAGCACCAATTTGGATTGAGGTG
CCAATTAGGAAGACGCCTTTGAAGTTTATTTATGGTATTAATACATTCATATAGGAGAGTCCAAATGAAG
AACTTACATAAATTTTGAAAAATGGAAGTGTTGATTCTTTCACCATCATTCTATTCATCAACCTGTAATG
CTCATTTGATGGATGCTACGTTAAACTGCCCATCCGAATTAAGCTTCCATAAGGGGTGTCAGCACCACTG
TTTATGCCTGGGGTATAGTATATACTGTTAACATAGGCAACCTTAGCTATGTCCAGAAGTTTTCTTGAAC
TGTATCTACATGGAAGATGTCTTAAATGTATAGATATCATAAATATTTTAGAATTGTGTTGAAGTATGAA
AAATCTAAATAAATAACTCAAAAGTAAAATCTAAAAATATCCTAGCATTTCTTCGGGCTTCGAGTTCAAA
TTCTAAAGAATTCCAATTTAGGTAATGAAATATTCACCGAAATATCTAAGAAAGTCTAGAACAATCTTTC
TCACTTGGGCACAAAAGATCTCTCACCTATCAATATTTATATGCCAGTAGAAAAGCTAAAGATAAAGTTA
GACAAACTGAAGGAAAAAGCTGTGAGCAAGTATTAAGAAAACAAAAGGTCCTCTTCAAAAAGTGTTTCAT
TGTAGGGAGAGATTGTGATACGACGGATGTGTCAACCTAATCAAAATATTTAGAAAATATTTGAGGGTAC
CAAATGATCTCTCGGTTTCTTTTTTAAAAAAATGTGTATGTAAACTTTCTTCAACATATATATTGTGTAT
CTTTAAACTCATCCTACCTTGTGCTTAATTAGTGGCTGAATCCAGTGGAATTTCTTGAAGACCTAATCAT
CGAGCACGAGTACGATTTTTCATTAATTAATTTTCATTCTACTAAATTAAATTATAATTTTTTTTTATTTT
ATTTGATTAAACCTAATAATTAAGTTAATTAATATTATTCTGAATTTATGAATATTATTTTCAGACATGT
GGAGTGCACCTCATCGGCGTTACAAGCCATACTTTTATTTAGGAAACAATACCCATCACATAGAAAGGAA
GAGATCAATAAATTTATCAACAAGGCTATTCGATTTCTTCTGGACACACAACTGCCTGATGGTTCTTGGT
AACCCTATTTATTATTACACATAATGGGTACTGACATTTTTGTACAAATTACCCAAGTTTCACTTCAAAA
TCTGGTTTGACTCTCATTTTGAAAACAATGAAGAAAAACTTAATTGTCAAGCTTAGCATGTGGAAATATT
ATTGTATTAGATTAGCTGATCACCAAGTACAAAACAAGAGAGTCTGTGTGAATGTGAGAGGGAGTGATGA
AAGCAAAAGAGTTATACAACTCTTGGTCTTAGGCGATGATATGTAGATGTGCCTTCTGATCCCACCATCC
TTTTATGATTTGTTAAAAAAAGTTAAGAGTTTTGTTTTAAAAATCTAGGTCAAACGAAATTTCCAACATG
AGATTTTAGTCTTCGTTACAAAAATTTTATAGAGAAAGTTTCAAGCATTGTGTGTGTATACATATATAGG
TATGGAAATTGGGGGATTTGCTATACATATGGAACATGGTTTGCACTTAAGGCATTGTCAATGGCGGGGA
AGACTTATGAAAATTGTGAAGCACTTCGAAAAGGAGCTAATTTTCTAATTAACATACAAAATTCAGAAGG
AGGGTTTGGAGAGAGCTACTTGTCATGTAGCAAGAAGGTAACACGTGTAGATTTATTTTTTAACTTACTT
TCTACCCTCAGTGTAGAGATAGTGTCAATTATACGCAAATTTTTAACTTTTCATGATATGATCTATATAC
ATTCGTTATAGGGTATTATCGTCGAGGTTCTAGATTTTATTCAAAATTCTAAAAGATTTATTGGTAATTT
GAAATGTTTATATGGATGTTATTCAAACAATATTCTTCTTATGTGTATATATATATAATTTAACCAATTG
TAAATTATTGTATCAATTATATGGGGGTAGCGTATATATGTATATACTTTGTATATTATATATTGATTGG
ATGGTTATTATTGGATGCACCACATAGAGATACATACCGTTGGATGGAAAACGATCAAATTTGGTGCAAA
CGGCATGGGGTTTGATGGGTTTGATATGTGCTGGACAGGCAAGTTCCTTTTCACTTATAACTTAAACATG
TTGTTCTTTATCCTTTAAAATCAATCATTTAACTTCTTACACCTTTTGAATGTAATTTTCGTATAATCAA
AACTTATCTTGAATGCTTAAAAGATGTTTTAGAGTAACGAAAAATATTTGTTTCTTCCACGAAGATCTGC
TCTTTATGGGAAGGGACTAGGAAAATTTTCCTTGTTAGCTAAGTGGGGATGGAGACAGACAATTTATTTT
CTTTCTCACAGCCTCTCCCTTTTTCTGTCTTTGCTTCCTATGATTCTTTTTTCAATTTATTATTTATTAT
TTTTTTATTTTTGTTATAAATTATTGTTATTGTATGTATTTTAATGACAAAGATAATGGTTATGTTTGAA
TCTAATGATTATTGAAGATTTAGATTACATAAATGAATGGTAATTATTTCCTTCTCCTAGAATATTTTAA
GCAATTTTCTCTAAATTTTAAATTTTCATGTTTTCAATTTAATTAAGGGAACGCAATCTCTACAAGTCTT
CTATTGAAAACTCCCATTCCGTTCTCTGCAAAATAAAACAAACAATTTTACAGAGATGGAGACTTAACAG
GAAGTGGAGATCCCAGATGGGAAACAAACCCCCAATAATCCACCCTTGATCTCCTCGAGCATCTCTATTT
TAGAATCGTCTTGAAAAAGAATTTGCTTCAAAATAACACTAATGAAATACTTAATTAACAGGCAAATATA

GACCCCAATCCAATTCATCGTGCTGCCAATCTTTTTCTTTTTCTTTTTCTTTTTTCTTTTTTTTTTTATT
TTTGGGTTTCCTTCTAAAACATATATTGCTAACCAATATAATTAACAACTTCTAATTGTTTACATGTGCA
GGAAATTACTGGAATATTCTTCAAAAATTGCACATTACACTATGCAGCATATAGAGAAGTTTTTCCAGTG
ATGGCATTGGGAGAATATTGTAACAAAATTTCCCTGTTTTCTAATGAAAAACAATAAATTTGAGATTTGA
ATTAATCAAACCTATAAACTACAAATTAATAAATATGCAGACTTGGACTCAATTAAAAGAGAAAAAAGAT
GAAGAGTACTTTTATACTTTTATTATGAAAATGTGATGAAGTTCAGATGATGTCTAGAGCTTCTCTCGAT
AAGTACATCAAATTATAAAGAATTGGGCTAACCATTTTTCTATATGGCAAGAGAATTAAGATGTATATCA
TAAAATGTCGATGGGAATTTTTTTCTTCTCAAATTGACAAAGTATAGAGTACAATCTTTGCTTCCAAATC
ACTTATGCTTATTATCTTCGCGTATATGTATCTCAAAAGAAATTGCATAACGACTTAACAAATAGTTGTA
CATGACGTTGTAAATATGTTCAGATGAAATAGTAAATCATATTGGAGCAGAATCAGCTGAGTCATTAATT
TTGAGGAACACGCCTACAACTTTCCAACACTTACACCCACAAGTTGTGGTGTCGAGAATTAACTGACTCT
CATATTCTGCTCCAATTGAGGTGGGAGGGAGGTGAGTGTGATGGTGATGGTGATGGTGGGGACAGAGGGA
ATTCTCAAATGGGTTTGAGAGAGATCGGAAAAGGAGGCCGGAGATGTGAGTTTTCGGAGATATCGAATGG
TGGTTGATGAAAAGAGATGTGCTCTATGTTGCTACAGTATCAACACTAACACCTCCAAAGACAAGCACAT
CATGTGATTTTATGACCTTAATTCCTTGCTTTTGCAAGTGCAAGTCCATTCTTATATTTATATTTGTAAA
ATCGAGCATGAACTAATGTTAAAGAAAGATGATCATATATTTGAAGGGACCGTTGTTTTAAGTATATTCA
CCGACTTTTCTTCCATAAATATGGGTCATGTGAACTTTAAGCATTTAATATAGTAAAACCAAATACTAGC
TAGTGGCCACTTGGGCAGCTACTGATATAGCTTAATTTTCTTTCAAAGTACAAACGTGTTGTATATTTTT
TTCAGTAAACGTATAAATTTTACATGAATATAATGTCACCAACAGTAATCAACAATAACTACTATAGTAT
TGAATCTATCCACAGATCTTAACTAATAATTACTGTTGTTACATAAAGAAGAAATTACTATATAGCATTA
ATTTTCATGTATTGAATCAACGAGAGAAAAATAGTGGAAGAAGTTCTACTTACTGTTCATTATTAATATG
ATTTAGTAGTGAGTGACGTTTAAACCGTAATGAAAAGAGGACAAATTTGAAATAATGTTAATATTGAGAC
AAATTGTTAGGGCTTGAAGAAGAGAGGGGCAAAGAATAAGAATCATAGTTTCCATAACTTATTAGGGCTT
GAAAACGGAGGGGGAGGGATAATTATAAAAGGTAAAAATGTTAAAGAAATTTCACTAATATAGCAAAATT
TCATATTGTCTATCAATAATAGAATTTAACTGTTGCTAATAATTTTATTCATTTGACTCTATTTGAAAAA
TACCCCAAATAAAAAATCAACTTTAACCCTATATTTCTTAAGCTGTTATGAATTTGAATTCAAAAAATTA
AAGGGATGGTTGCAAATGTAGTAATTAGATTCAAAATATTAGCAAAATCTTAGCAACATTTTAAAAATTT
GGAAATATAGCAAAATCTGTCAAAGTCCATCAACGATAAAAGCCTATCACTAAACCATGTTGTAAATTAA
ATATTGGTCTCAAAAAGTATAGAGGGGGTCATTGAGTGTGATGGCAGAGCTTCTCTCTCAACTCCTCAAG
CCGGCGGGCATTACGGTTCCCAAACTTCAGAGAAACTCAAACAAGTGCCATGGAAAACCTTTGCGCTTCC
CCTGAAGAAATTTACTCTGCTCAACAACCAAATTTATTCCAGCTGATACTCCCGCCAACCAAGGAAGATT
TCGATTATCTCAAACTATTAAGGTACATTTACAGCAGTCTTCAAACTCACTCTCTGTTTCCTGCTTTGCA
TTGCTTTCTTTCGCAGCATGTCTTCACTACGACAGCTCCCTCGCTCTTGCACCATCGAAAAAAAAGACTT
TGTCCTTTCAGTGGACAACCGATCACGCGATTTAAAATTGCTTATTACGGAGGTGGGGCCATACAAATCT
TTCTCAATTGCTGTCACTATGGAATCTTTGGAATGGTTAAAGTCTTCTTTCAAAACTCTGTTGGATACCC
CTCGAACAACCAGATTCTTCTTGGAAAAAAGATTCGAGGAATATTGCCTGTGGGTCCAAAAAACATACAA
CAGAAAAGGTTACATTGCTGAAATTTATCGAGTGGACGACAGAGGAAGGAAATGTTGTATTCTTATCCCA
GAAGGGTCAGAAAAATCAGGTTGGGCACAATTTGTCAGTCTTTTATATGGAAAGAAGGATTCGTCCACAA
AAGCAAACTACAGAACTCTTCTGAGCACCAAATTGAAAGACGGATTCTCCAGCTCTGAATCAGGGGACGA
CACAAAGAGGAGATCCTATGTCGAAGCGGTTATTAAAGGCAGCTCATCCGACGAGGAAACAAACTCATGG
ACTACAAATATAAAGAAAACAGGGAAATCCAACACTAACTTCTCTTTTGAATGGGAAAGAACAGTGGTCC
TTACTAGAAGATTTTTCCATGACGATTGGGAGAAAATAGTAGAGAAGCTAAATGAACAACTTGATACCAC
TGTTAGGTACAAGCCTTTTCATGCTGATAAAGCGTTGATTTGTTTCAAAAATGTGGAACAAGCCAACCTG
ATATGTAAAAATAAAGGTTGGACTACAGTGGGTCGTTTTTATGTCAAGTTCGAAGAATGGAACCAGAAAG
CCCATGCATTGTCGAAAGTCATCCCAAGTTATGAAGGATGGATTAAGGTGCGAGGAGTCCCCCTGCATGC
GTGGAATTTAGAGAGTTTTATTCAAATTGGAGATGCGTGTGGTGGTTTCATTAAAGTGGCAAAAGAAACT
AGGGAGCTCACAGATATCATAGAAGCATCTATCAGAATCAAAGACAACTACTCAGGGCTCATTCCGGCGT
TCATCAAACTGTTTGACAAAGAAGAACACAGTTTCATTATTCAAGTTATAGTCAAAACCGAAGGGAAATG
GCATCTGGAGAGAAGTCCAAGTATCCATGGTACTTTTACTAGAGAAGCGGCTAAAAGATTTGATGAATTT
AATTTGAACAGTGAACAATACTTCTTCGAAGATAATCTTGCAATCTCGCCGGAAAAAGCAGTGTCCATGG
TGATCGGAAAGGAAGATCAGAAAAAGAAGGGAAACGTTCAGATCCCCGAAAATAATAAAAAATTGGGAGC
AATGATGAATTTTTTGAATTATGACGGCGACAGCGACTCAAGTGAGAAAAGAAAGATGGAGACAGAAGTT
GATGAGCTGACAGTGTTTAGCCAAAAAGGAGGGAAGTTTTTTTATAAAGGGAAAAATTCAAAGTCAGCTG
GGCAAAAGAGAGGCAAAAGAAAAGTCTCTTTCGAATCTCCGAAATCCAAGACGATGCTTTATGACCCAAA
GAGTGCCCCACAGGCCCTTCTGAAGTTAGAAAGCCCACTTGAAAGCCCAAAAAAAAAATTGA
