# Supplementary material for: Interspecific and intraspecific gene variability in a 1-Mb region containing the highest density of NBS-LRR genes found in the melon genome
Source: BMC Genomics. 2014 Dec 17;15(1):1131. doi: 10.1186/1471-2164-15-1131 (PMC4378003; doi:10.1186/1471-2164-15-1131)
Supplement: Supplementary file 3 — Additional file 3: File S2: Protein sequences of the predicted TIR- and CC-NBS-LRR genes in the improved MELO3C004258-MELO3C004324 region. (PDF 29 KB) [file 12864_2014_6878_MOESM3_ESM.pdf]

>MELO3C004258
MDSSTVATESPTFKWSYDVFLSFRGEDTRTNFTSHLDMALRQKGVNVFIDDKLERGEQISETLFKSIQEALISIVTFSQNYASSSWCLDE
LVKIIECKKSKGQIVLPIFYKVDPLDIRKQTGRFREALVKHMPKFQTKTQIWREALTTMANLSGWDLGTRKEADLIGDVKKVLSTLNRTC
MSLYVAKFPVGIDSKLEYMKLRSNNLLEKSNKFHNRTQHEHESDTGIGKTTLAKALYNKIASQFEGCCFLSNVREASKRFNGLAQLQESL
LYEILMDDLKVVNLDRGINIIRNRLHSKKVFIVLDDVDKLEQLEALVGGRDWFGQGSRIIVTTRNKHLLNSHGFDEMHNVRGLNQDKAIE
LFSWHAFKKCCPSSNCVDLSKRATSYCKGHPLTLVVLGSFLCTRDQAKWSSILDEFENSLNKDIRDILRLSFDGLEDKVKDIFLDISCLL
VGEKVKYVKNMLSACHVNLDFGIIVLMDLSLITIENNKVQMHDLIRQMGHKIINDESSELGKRSRLWLVQDIWDIFFHNSGTDTIKTIKL
DLPNPTMLNVDSRGFRNLKNMRLLIVRYAIFSSKIEYLPNSLKWIKWHGFAQPSLPSRLIMKNLVGLDLQHSFIKKFGKRLEVNCISIAL
MGSLHF
>MELO3C004259
MGSSVVRVGSSFSDPNCNYDYDVFFSFRGEDTRSNFISHLHMALRLKEVNVFIDDKLKRGEQISESLLKSIERSRLSLVIFSKDYASSTW
CLDELVKIIECKKSKGQAVLPVFYKVDPSEVRKQTDWFGEALAKHEANKLLTNKIQPWKEALTFAAGLSGWDLANSKDEAELIQEIVKRV
LSIVNPMQLLHVAKHPVGVNSRLRKIEELVSHIGFEGVNMVGMYGIGGIGKTTLAKALYNKIATQFEGSCFLLDVRREASKHGLIQLQKT
LLNEILKEDLKVVNCDKGINIIRSRLCSKKVLIVLDDVDHRDQLEALVGERDWFCQGSKIIVTTRNKHLLSSHGFDEIHNILGLNEDKAI
ELFSWHAFKKNHPSSNYFDLSERVTSYCKGHPLALVVLGSFLCNRDQVEWCSILDEFENSLNKDIKDILQLSFDGLEDKVKDIFLDISCL
LVGEKVEYVKDTLSACHVNLDFGIIVLMDLSLITIENDQVQMHDLIKQMGHKIVCGESLELGKRSRLWLEKDVLEVFSSNSGTSAIKAIK
LEFHNPTRLIVDPQAFRNLKNLRLLIVRNARFCAKIKYLPESLKWIEWHGFSQPSLPSHFIVKNLVGLDLQHSFIKDFGNRLKVGEWLKH
VNLSYSTSLKKIPDFSAASNLEKLYLRDCTNLRTIHRSIFCLVKLTLLCLSGCCMIKKLPTSCFKLWSLKHLDLSGCTKLEKIPDFSSAL
NLEILHLSRCTNLRTIHNSVFSLHKLISLYLDFCSTLKTLPTSCFMLTSLNTLTLYSCQKLEEVPDLSSASNLNSLNVEKCTNLRGIHES
IGSLDRLQTLVSRKCTNLVKLPSILRLKSLKHLDLSWCSKLESFPIIDENMKSLRFLDLSFTAIKDLPSSIGYLTELPRLNLGNCTSLIS
LPKTISLLMSLLDLELRNCRSLQEIPNLPQNIQNLDAYGCELLTKSPDNIVDIISQKQDLTLGEISREFLLMGVEIPKWFSYKTTSNLVS
ASFRHYSDMERTLAACVSFKVNGDSSRRISCNIFICNRFHCSFSRPFLPSKSEYMWLVTTSLAWGSLDAQDWNKVVVLFEVDDEVNLSIR
SYGVHVTEEFNGTQTDVKWPVVNYGDFYQPEKLQNLDIEDILVKRLFDELSYLSNCKAVLHAGSYDPIVITDSNIQPMIFPLHVTYSGYT
VISGMEGMGKTALANSLRNKFKRKDNSNWGQCLNDSSRFYLLQGRKSRIFSGYANPSKRRISSKRYYYITFVNLDVIEAQNVNAWFTAQR
WIICCSPLESLRRCSHFVITSVDPSLWHTCGVDDVLSSTFQKKFSERRAYIFGIHLPDNFLM
>MELO3C004260
MDSSTESSTFKWSYDVFLSFRGEDTRTNFTSHLDMALRQKGVNVFIDDKLERGEQISESLFKSIQEALISIVIFSQNYASSSWCLDELVK
IIECKKSKGQIVLPIFYKVDPSDIRKQSGTFGEALAKHQAKFQTKTQIWREALTTAANLSGWDLGTRKEADLIGDLVKNVLSTLNRTCTP
LYVAKYPVGIDSKLEYMKLQSHNLFEKSNKFHYQTQHEYEFDTGVYLVGIYGIGGIGKTTLAKALYNKIASQFEGCCFLSNVREASKQFN
GLAQLQESLLYEILTVDLKVVNIDRGINIIRNRLCSKKVLIVLDDVDKLEQLETLVGGRDWFGQGSRIMVTTRNKHLLSSHGFDEIHNIL
GLNEDKAIELFSWHAFKKNSPSNLSKRATSYCKGHPLALVVLGSFLCTRDQVEWCSILDEFENSLNKDIKDILQLSFDGLEDKIKDIFLD
ISCLLVGEKVKYVKNMLSACHVNLDFGIIVLMDLSLMTIENDKVQMYDLIKQMGHKIVCGESLELGKRSRLWLVRDVWEVLVNNSGTDAV
KAIKLDFPNPTRLDVGPQAFRKMKNLRLFIVQNARFSTKIEYLPHSLKWTKWHGFPQPTLPSCFITKNLVGLDLQHSFIKTFGNILK
>MELO3C004262
MDSSTVIIEPPTFKWNYDVFLSYRGEDTRTNFTSHLDMALRQKGVNVFIDDKLERVFYKVDPSDIRKQSGSFGEALAKHQAKFKTKIQIW
REALTTAANLSGWDLGTRKEADLIGDIVKKVLSTLNRTCMPLYVAKYPVGIDSKLEYIKLRSHNMFEKNNKFHYRTQHEYEFDTGIYMVG
IYGIGGIGKTTLAKALYNKIASQFEGCCFLSNVREASKQFNGLAQLQESLLYEILMVDLKVVNLDRGINIIRNRLCSKKVLIVLDDVDKL
EQLEALVGGCDWFGKGSRIIVTTRNKHLLFSHGFDEIHNILGLNEDKAIELFSWHAFKKNRPSSNYLDLSKRATSYCKGHPLALVVLGSF
LCIRDQAEWCSILDEFENSLNKDIKDILQLSFDGLEDKIKDIFLDISCLLVGEKVEYVKDMLGACHVNLDFGVIVLMDLSLITIENDKVQ
MHDLIKQMGQKIVCGESLELGKRSRLWLVQDVWEVLVNNSGTDAIKAIKLDFPNPTRLGVNSQAFRKMKNLRLLIVQNARFSTKIEYLPD
SLKWIKWHGFPQPTLPSCFITKNLVGLDLQYSFMKTFGKRLEDCKRLKHVDLSHSTFLEKIPNFSAASNLEELYLINCKNLGMIDKSVFS
LDKLTILNLAGCSNLKKLPRGYFILRSLRYLNLSHCKKLEKIPDFSAASNLEELYLFNCTNLRMIDKSVFSLHKLTILNLDVCSNLKKLP
TSYYKLWSLQYLNLSYCKKLEKIPDLSAASNLQSLCLHECTNLRLIHESVGSLYKLIDMDLSGCTNLAKLPTYLRLKSLRYLGLSECCKL
ESFPSIAENMESLRELDMDFTAIKELPSSIGYLTQLYRLNLTGCTNLISLPNTIYLLRNLDKLLLSGCSRFEMFPHKWDPTIQPVCSPSK
MMEATSWSLEYPHLLPNESLCSHFTLLDLQSCNISNAKFLEILCDVAPFLSDLRLSENKFSSLPSCLHKFMSLWNLELKNCKFLQEIPNL
PQNIQNLDASGCKSLARSPDNIMDIISIKQDLAMDEISREFLLTGIEIPEWFSYKTASNLASASFRHYQDIERTLAVGVIFKVNGDSSER
GVRISCNIFICNKLHCSYSRPFLPSKSEYMWLLTTSLAWGSMEVNDWNKVMVWFEVHEVHGEVNATITRCGVHVTEELPAIQTDAKWPMV
NYADFYQLEKLQSLDIEHLLLKRFFEEMSCWSNCKAIMFHAANYDPETNSVCHRFNWSDDIDWRQPLDDPISFYWVQERQYRFMSYSGLD
NRGGLIHPYGNPGFDPPSN
>MELO3C004266
MDSSTVATKSPTFGWSYDVFLSFRGEDTRTNFTSHLDMALRQKGVNVFIDDKLERGEQISETLFKSIQEALISIVIFSQNYASSSWCLDE
LVNIIECKKSKGQIVLPVFYKVDPSDIRTQTGSFGEALAKHQAKFQIKTQIWREALTTAANLSGWDLGTRKEANLIGDLVKKVLSTLNRT
CTPLYVAKYPVAIDSILEYMKLRSHINLFEKSNKFHYQTQHEYEFDTDVNMVGIYGIGGIGKTTLAKALYNKIASQFEGCCFLSNVREAS
KQFNGLAQLQESLLYEILTIYLKVVNFDRGINIIRNRLCSKKVLIVLDDVDKLEQLEALVGGRDWFGQGSRIIVTTRNKHLLSSHGFDEI
HNILGLNEEKAIELFSWHAFKKNHPSSNYLNLSKRATSYCRGHPLALVVLGSFLCTRDQVEWCSILDEFENSLNKDIKDILQLSFDGLED
KVKHIFLDISCLLVGEKVKYVKNMLSACHVNLDFGIIVLMDLSLMTIENDKVQMHDLIKQMGHKIVCGESLELGKRSRLWLVQDVWDVLV
NNSGTDAVKAIKLDFPNPTKLDVDLQAFRKMKNLRLLIVQNARFCTKIEYLPDSLKWIKWHGFPQSTLPSCFITKNLVGLDLQHSFIKTF
EKRLKDCERLKHVDLSYSTLLEQIPDFSAASNLGELYLINCTNLGMIDKSLFSLNNLIVLNLDGCSNLKKFPRGYFMLSSLKELRLSYCK
KLEKIPDLSAASNLERLYLQECTNLRLIHESVGSLDKLDHLDLRQCTNLSKLPSHLRLKSLQNLELSRCCKLESFPTIDENMKSLRHLDL
DFTAIKELPSSIGYLTELCTLNLTSCTNLISLPNTIYLLRNLDELLLSGCSRFRIFPHKWDRSIQPDLTLGEISREFLLTGIEIPEWFSY

KTASNLVHEAHSEVNATITRCGVHVTEELHGIQMDVKWPMVNYADFYQLEKLQRLDIEDLLLKSFLESVSCWSNSKAMLHAGNYDPEAII
DSNIQPMIFPLHVTYNGETVICGMEGMGDTTLANSLCNKFKWMKDNLFEIKEHHYSEALDNSTSIFHIRGRELQRFSWSWAAHHRKRGDG
KRGTNITTHTISSKCYLMLFHEVENCNDIFDWVGTQRWIKTSGSSNGRELKLDMASNGNMLQHQLPRFSGKNFNQWSIQMKVLYGSQELW
DIVERGYTEVENQSELTNQQLVELRENRNKDKKALFFIYQAVDEFISERISTATSAKAAWDILRSTYQGEDKVKMIRLQALRSEFDCIKM
KETETIEEFFNHILVIVNSLRSNGEEVGDQRVVEKILRSMPRKFEHIVVAIEESKDLSTLSINSLMGSLQSHELRLKQFDVNPEEAFQMQ
TSFRGGSRGRRGGHGRRGGGRNYDNRSGANSENSQESSSLSRGRGSGRRRGFGRNQGGGRGNFSQIQCFNCRKYGHFQADCWALKNGVGN
TTMNMHKEQKKNDEGILFLACSVQDNVVKPTCEDGDNTRLQVKGQGDILVKTKKRTKRVTNVFYVPGLKHNLLSIGQLLQRGLKVSFEGD
ICAIKDQADVLISKHIEGFILALAFSIWSLKLQITILFVQKSYAWRASKPLELIHTDLCGPMRTTTNGGNRYFITFIDDFSRKLWIYFLK
EKSEALVCFKSFKAFTENQSGYKIKTLRSDRGGEYIVFGNFFKEQGIHHQMTARMTTQQNGVAERKNRTIMEMARSMLKAKNLPNEFWGD
AVACTVYILNRAPTKSVPGMTPYEAWCDEKPSVSHLKVFRSIAYSHIPNQLRGKLDDKSEKCIMVGYNENSKAYRLYNPVSRKIIINRDV
IFSEDESWNWNDDVDEAKSPFHVNINENEVAQELEQAKIQAVESSSSSTSSSTSNDEISPRRMRSIQEIYNNTNRINVDHFANFALFAGV
GPVTFDEAIQDEKWKIAMDQEIDAIRRNETWELMELPTNKQALGVKWVYRTKLKSDGNVEIYKARLVVKGYKQEYGVDYEEIFAPVTRIE
TIRLILSLAAQNGWKVHQMDIKSAFLNGHLKDEIFVAQPLGYVQRGEEEKVYKLKKALYGLKQAPRAWYSRIDSFFLKTGFRRCPYEHAL
YVKEDKYGKFLIVSLYMSDMGLIHYFLGIEVNQNEGEIVISQQKYAHDLLKKFRMENASPCNTPMDANLKLCKDDIGEAVDPSLYRSLVG
SLMYLTATRPDILFVVSMLSRFMTNPKRSHWEAGKRVLRYILGTINFGIYYKKVSESVLFGFCDSDWGGNVDDHRSTSGYVFSMGSGVFS
WTSKKQSVVTLSTTEAEYISLAAAGCQALWLRWMLKELKCTQKCETVLFCDNGSAIALSKNPVFHGRSKHIRIKYHFIKDLVKDGEVIVK
YCKTQDQVADIFTKAQKFDLFVKFRGKLGVRKQIGGFGEALAKHEANKLLTNKIQPWKEALTFAAGLSGWDLANCKDEAELIQEIVKRVL
SIVNPMQLLHVAKHSVGLNSRLRKIELVSHIGSEGVNMVGMCGIGGIGKTTLAKVLYNKIAYQFEGCCFLQDVRREASKHGLVELQKTLL
NDILKEDLKVVSRDRRIIIRSRLFKESSYSSR
>MELO3C004288
MQLDVAKYPVGIDIQVRNLLPHVMSNGTTMVGLYGIGGMGKTTLAKALYNKIADDFEGCCFLPNIREASNQYGGLVQLQRELLREILVDD
SIKVSNLPRGVTIIRNRLYSKKILLILDDVDTREQLQALVGGHDWFGHGSKVIATTRNKQLLVTHGFDKMQSVVGLDYDEALELFSWHCF
RNSHPLNDYLELSKRAVDYCKGLPLALEVLGSFLHSIDDPFNFKRILDEYEKYYLDKEIQDSLRISYDGLEDEGITKLMNLSLLTIGRFN
RVEMHDIIQQMGRTIHLSETSKSHKRKRLLIKDDAMNVLKGNKEARAVKVIKFNFPKPTELDIDSRAFEKVKNLVVLEVGNATSSKSTTL
EYLPSSLRWMNWPQFPFSSLPPTYTMENLVELKLPYSSIKHFGQGYMSCERLKEINLTDSNFLVEIPDLSTAINLKYLDLVGCENLVKVP
EGVICTSAAGCKSLARFPDNLADFISCGNSAECCKGGELKQLVLMNCDIPDWYRYKSMNDSLTFFLPADYPSWKWKALFAPCVKFEVTND
DWFQKLECKVFINDIQVWSSEEVYPNQKERSGMFGKVSPGEYMWLIVLDPHTHFQSYSDDIMDRRSPKIIDLNQPSFGINSSQSILGKIT
VSFQVTPWYKDVVSIKMCGVHVIMWE
>MELO3C004289
MGSTAAGAESSSSSPIFNWSYDVFLSFRGEDTRSNFTGHLYMFLRQKGVNVFIDDGLERGEQISETLFKTIQNSLISIVIFSENYASSTW
CLDELVEIMECKKSKGQKVLPIFYKVDPSDVRKQNGWFREGLAKHEANFMEKIPIWRDALTTAANLSGWHLGARKEAHLIQDIVKEVLSI
LNHTKPLNANEHLVGIDSKIEFLYRKEEMYKSECVNMLGIYGIGGIGKTTLAKALYDKMASQFEGCCYLRDVREASKLFDGLTQLQKKLL
FQILKYDLEVVDLDWGINIIKNRLRSKKVLILLDDVDKLEQLQALVGGHDWFGQGTKIIVTTRNKQLLVSHGFDKMYEVQGLSKHEAIEL
FRRHAFKNLQPSSNYLDLSERATRYCTGHPLALIVLGSFLCDRSDLAEWSGILDGFENSLRKDIKDILQLSFDGLEDEVKEIFLDISCLL
VGKRVSYVKKMLSECHSILDFGITKLKDLSLIRFEDDRVQMHDLIKQMGHKIVHDESHDQPGKRSRLWLEKDILEVFSNNSGSDAVKAIK
LVLTDPKRVIDLDPEAFRSMKNLRILMVDGNVRFCKKIKYLPNGLKWIKWHRFAHPSLPSCFITKDLVGLDLQHSFITNFGKGLQNCMRL
KLLDLRHSVILKKISESSAAPNLEELYLSNCSNLKTIPKSFLSLRKLVTLDLHHCVNLKKIPRSYISWEALEDLDLSHCKKLEKIPDISS
ASNLRSLSFEQCTNLVMIHDSIGSLTKLVTLKLQNCSNLKKLPRYISWNFLQDLNLSWCKKLEEIPDFSSTSNLKHLSLEQCTSLRVVHD
SIGSLSKLVSLNLEKCSNLEKLPSYLKLKSLQNLTLSGCCKLETFPEIDENMKSLYILRLDSTAIRELPPSIGYLTHLYMFDLKGCTNLI
SLPCTTHLLKSLGELHLSGSSRFEMFSYIWDPTINPVCSSSKIMETSLTSEFFHSRVPKESLCFKHFTLLDLEGCNISNVDFLEILCNVA
SSLSSILLSENNFSSLPSCLHKFMSLRNLELRNCKFLQEIPNLPLCIQRVDATGCVSLSRSPNNILDIISSQQINFAWLRNRPRGIREFV
LMNNGIPEWFSYQIASNAIMVTFQHNRDTKITLATSVTFRVDGDSDQGMALVSCNILIGCRLDRRYMRKFPKSASEYTWLVETSATYRRS
SLEMNDWNDVIVWFEAVKCAEVVTIRRCGVYFTEKVSGMQNDVKEPRAIYTYFNQPEKLRPRW
>MELO3C004290
MNRATGSSSSSRFRSSFDVFLSFRGEDTRPNFTSHLCMALRQRGINVFIDNKLSRGEEISTSLLKAIEESKISIVIISENYASSSWCLNE
LVKIITCNKLRGQVVLPIFYKVDPSQVGKQSGRFGEEFGKLEVRFSWDKMEAWREAMISVSHISGWTVLQKEDEANLIQKIVQEVSKRLN
RGAIQLRIAKYPIGIDRQINNILFQVTSDEKITMVGFYGIGGIGKTTLAKALYNKIANDFEGCCFLANVREASNQYRGLVELQKELLREI
LMDDLIKFSNLDVGISIIRDRLCSKKILLILDDVDTSEQLEALVGEHDSFGPGSMVIVTTRNKHVLVIHEFDILQSVQGLKDDEALKLFS
WHAFKQSCPSSDYLDLSKRAVRYCDGLPLALEVVGSFLHSIEQSKFKLILDEYENQYLDKGIQDLLRISYDGLEDEVKEIFLYISCCFVG
EDINEVKTKLXY
>MELO3C004291
MALRQRGINVFIDDKLSRGEEISASLLEAIEESKISIVIISENYASSSWCLNELEKIIMCNKLRSGEQLVLPIFYRVDPSQVRKQSGRFG
EEFGKLEVRFSSDKMEAWREAMISVSHMSGWPILQKDDEANLIQEIVQEVLKKLNHGTMQLRLPKYPVGIDKQVNNIHFQVMSTDEKTTM
VGLYGIGGIGKTTLAKALYNRIADDFEGCCFLPKIREASNQYDGLVQLQKKLLCEILMDNSININNLDIGINIIRNRLCSKKILLILDDV
DTREQLEALAGGHDWFGHGSKVIATTRNKQLLASHGFNKLEKVNELNVIEGLELFSWHAFRNSHPSSDYLDLSKRVVRYCDGLPLALEVV
GSFLYSIEQSKFKLILDEYETQYLDKGIQDPLQISYDGLEDEVKEIFLYISCCFVGEDINEVKKKLKACGCLCLEKGTTKLMNLSLLTLD
DFNQVEMHDLIQQMGRTIHLLETSTSHKRKRLLINDDAMDVLNGNKEARAVKVIKLNFPKPTELDIDSRAFEKVKNLVVLRMLHLQKCGE
WLKEIDLSFSEFLVEIPNLTAAINLEMLDLQGCINLVKIHESVGSLSKLVEFYLSSNIKGFEQFPSCLKLESLYTLTLYSCRIDERCPQF
SEEMNSLELLWIKDSVVINQLSPTIEYLTSLQQLWIINCMGLKSLPKIPKVPKGAVTMNASGCILLARFPDNILDFISCYDNYMEEKHEY
KVIKELILMNCDIPDWCQYKSTNNSITFLFPADYPTWERKAFIAFCVKFQVIDEEFKVDSRVFINDFEVYNGHFWTNEIVGRKRPRGEYL

WIEVIDPDILLDPYDDCEQNQPIIFDRVTVLFEVITPNAVNIKKCGVHVIMEE
>MELO3C004292
MYRASGSSSSHVRLPFDVFLNFRGEDTRSSFTSHLHMALCQKGVKVFIDDDKLPRGEEICTSLLKAIEESKISIVIISENYASSHWCLDE
LIKIIMCNKSNNRQVVFPVFYKVDPSQVRQQSGRFGEEFGKLQVRFSNKMQAWSEALTFISSMSGWDLKNYESEASLIQIIVQEVRKKLK
NSGTTQLDVAKYPVGINIQVNNLLLHVMPNGVTMVGLYGIGGMGKTTLAKALYNRISDDFEGCCFLANVREASNQHWGLVELQKALLRKI
LMDDSIKISNIGIGISTIRDLLCSKKILLVLDDVDTHEQLQALAGGHHWFGHGSKVIATTRNKQLLASHGFNILRRVNGLNAIEGLELFS
WHAFKNSHPSSDYLHLSKHAVHYCKGLPLALEVLGSFLNSIDDQSKFKHILDEYENSYLDKDIQDILRISYDELEQDVKEIFLYISCCFV
NEDKNKVQMMLQACDCHFRLEMGIKKLTDLSLINIDMFNCVEMHDLIQQMGHTIHLLEPSNSHKRKRFLFEKDVMDVLNGDTEARAVKAI
KLNFPQPTELDIDSRAFEKVKNLVVLKVHNVTSSKSLEYLPSSLRWIIWPKFPFSSLPSSYSMEKLIELTMPSSFIKHFGNGFMNCEWLK
RIDLSRSEFLEEISDLSSAINLEELDLSWCNNLVRVHESVGSLGKLATLDLSSHSNGFTQFPSNLKLKSLKELVMKECRIVKRYPHFSEE
MKSSLEELRIEYSCVTDLSPTIGHLTGLTHLTIVECKEFTTLPSTICHLSNLIALTVINSELSTFPFLYSRSLALFPHLICLDLSNCNIT
NLSFLESITHVAPSLTELYLTGNDFRSLPSCIVNFKYLRHFDIRNCRFLEEILKVPEGVIFMNAQGCKSLARFPDNIAGFISCDLEFVDR
KYRQLILMNCDIPEWFDYKSRNNSITFPTTFNYPGWRLKVLAACVKVQVHDCVTQYHNTAELECQVFFNDIPVWSSEDEEKCLVEESRWL
SLEASPNDYTWFIVLNPHRDFYLDLDDMMEGSPETDVSQLCFGINSMEMDHNIIPDDNWNSIGGSIWKNFTVLFTPRPELSDAKVSIKSC
GVHVIMEE
>MELO3C004294
MDRASGSFSSHRWRFDVFLSFRGEDTRFNFTSHLYTALRQRGINVFIDDSELTRGENFPSSLLRAIEESKISVVIISENYATSSWCLNEL
VYLIMCKKLRGQVVLPIFYKVNPSQVRTQNGAFGEAFAKLEVRFFDKMQAWREALTTVSFMSGWVLLQNDDEARLIQIIVRHVWKKLTCS
TVQLSVTKYPVGIDRQVKDLLSHVIIDETRMVGLYGIGGMGKTTLAKALYNRVADKFEGCCFLANIREASKQHDGLVRLQEKLLYDILMY
DFVRVGDVYKGINIIRNRLYSIRILLILDDIDTSEQLQVLAGGYDWFGHGSKVIVTTRNEQLLDIHGFYKLKEVPQLHFGEALELFSWHA
FHNSCPPSEYSTLPEDAVNYCKNLPLALEVLGSFLYSTDQSKFKGILEEFANSNLNKDIQKLLQSCGCLCWENGIKKLMNLSLLTINRIN
KEARAVKAIKMEFPNPTELDIIDSNAFSKVKNLAVLKVKNVTFSKISTLDSLPNSLRWMSWSGFPFSSFPSSYSMENIIQLKLPHSSIKR
FEKEAFTHCKWLKELDLSNSIFLEEIPDLSAATNLEKLSLSGCENLVKVHKSVGSLGKLVDLCISSHVYGFEQFPSPLKLKSLKRFSTYH
CTIVRGYPQFSKEMESSLEHLWFYRSSITELSSTIRYLTSLKILSITDCKELTTLPSTIYDLSKLTSIEVSQSDLSTFPFSYSCPSSLLH
LTRLDLYENKITNLDFLETIAHAAPSLRELNLSNNNFSILPSCIVNFKSLRFLETIDCKLLKKIPKIPEGLIYLDAQGCISLAKFPDNLA
DFISCDSVHISSLSLVLISLEHVDGQFKQLILMNCDIPDWFSYKSRNNPITLLVPSNDPSSELKVFAACVKFQVNHVDQDQYMDLECKVF
INDIQVWSYEEVPFHDESRSILIKASPHEYMWLLVLYPHINFRLNSDDIINRSQEINLHQPSFGINSIGRDNNNCNVDDDYRRNHIGESI
WRKFTVSFGVTSKFKDSELSIKTCGVHVIMEEWCD
>MELO3C004295
MALRQRGINVFIDNKISRGEEISASLLEAIEEPKIFIVIISENYASSRWCLNELIGFCLDGVIKELILMNCDIPDWYRYTSMNNSITFLL
PADHLSWKRGAFSSPCVKFEVTNDALQETWVLPTHKYVGENAKYVGKGYPDVQNGVGKNVKRNASREAFPTPYQIGVGKAFPTSGDASPS
TASGKPYSQRRLCRRISRRRENLFSNVFFPT
>MELO3C004301
MGSSALPVESSSSSSSPNFLYYYDYDVFFSFRGEDTRSSFISHLHMALRLKGVNVFIDDKLKRGDQISESLLKSIERSRLSLVIFSKNYA
SSTWCLDELVKIIEYKKSKSQAVLPVFYKVDPSEVRKQTGGFGEALAKHEANKLLTNKIQPWKEALTFAAGLSGWDLANCKDEAELIQEI
VKRVLSVLNPMQLLHVAKHPVGIDFRLRKIEELVSHIGSEGVNMVGMYGIGGIGKTTLAKALYNKIANQFEGCCFLQDVRREASKHGLVK
LQETLLNDILKEDLKVVSRDRGINIIRSRLCSKKVLIVLDDVDDREQLEALVGGRDWFGRGSKIIVTTRNEHLLFSHGFDDQKHKIQELN
QDHALELFSWHAFKKSHPSSNYLGLSERATNYCKGLSLALVVLGSFLRGRDQAEWNCILDEFETSLRKDIKDVLQLSFDGLEDKAKDIFL
DISCLLVGEEYNCAKKMLSACHLNIDFGIMILVDLSLVTIETDRVQMHELIQQMGRSIVHNESSEPGKRSRLWLVQDIWEVFVNNSGTDA
VKAIKLDLPNPTKLNVDPQAFRSMKNLRLLIIRNAQFCRKIKYLPNSLKWIEWRGFAHRSLPSCFITKNLVGLDLRHSSIKRFGKRLEGC
ERLKHVDLSYSTLLEQINDFSPASNLEELHLINCTNLGMIDKSVFSLYKLSVLNLDGCCNLQKLPRGYFMLSSLKELNLCYCKKLEKIPD
LSAASNLKRLYLQECTNLRVIHESVGSLDKLNHLDLRQCTKLVKLPSYLRLKSLSNLLLSGCCKLESFPTIAENMKSLRELDMDFTAIKE
LPSSIGYLTNLSILKLNGCTNLISLPNTIYLLRNLENLLLSGCSIFGMFPHTWDPTIPTIQQVCSPSKMMETASWSLEFPHLLVPNESLC
AHFTLLDLESCNISNAKFLELLCDVAPFLSDLRLSENKFSSLPSCLHKFMSLWNLELRNCKFLQEIPNLPENIQKMDASGCESLARNPDN
IVDIISKKQDLTLGEISREFLLTGIEIPEWFSYKTTSNLVTASFRHYPDMERTLAACVSFKVNGDSSKRGAQISCSIFICSKLHSSFSRP
FLPSKSEYMWLVTTSLAWGSMEVNDWNKVLVWFEVHEAHSEVNATITRCGVHVTEELHGIQMDVKWPMVNYADFYQLEKLQSLDIEDLLL
KSFLETVSCLSNSKAAMLHAGNYDPEAIIDSNIQPMIFPLHVTNNDGTYICGGMGGTALANSLCNKFKGMEGQCGEALDNSTSFFHIKRR
QLLSYSWSPAVHHRKCGDGERGTNITTHTISSKRYLILLREAKSYQDVHDWFYTHCWIKASYCSYDGRGDGVILIEGVDTSLL
>MELO3C4302-4303
MGSSTAVTESIAFEWSYDVFFSFRGEDTRTNFTSHLDMVLRQKGVNVFIDDKLQRVFYKVDPSDIRSNFGEALAKHQAKFQTKTQIWREA
LTSAANLSGWNLGAYSQFEGCCFLSNVRQASKQFNGLVQLQENLLYEILKDDLKFVNLDRGITIIRSRLHSKKVLIVLDDVDKLEQLEAL
VGGRDWFGQGSKIIVTTRNRHLLSSHGFDEMHNIRGLYQDKAIKLFSWHAFKESHPSSNYLGLVERATSYCKGHPLALVVLGSFLCTRDQ
TEWISILDEFENSLSNNIKDILQLSFDGLEDRVKDIFLDISCLLVGEEVNYVKNILSACHLNVDFGIIILMDLSLITVENGTVQMHDLIQ
QMGHKIVYGESPEPGKRSRLWLAQDIWEVFVNNSGTDTIKAIKLDLANPTRLHVDPQAFRSMKNLRVLIVQNARFSTKIKHLSDSLKWIK
WHGFAHRSLPSCFITKSLVGLDMQHSFIKKFGKRLEQIPHFPAASNLEELHLSECTNLRKIDKSVFSLDKLTILNLDGCSNLKKLPTSYF
MVRSLKHLKLSYCKKLERIPDLSLASNLESLYLEDCTNLRMIHESIGSLDKLVTLVLRRCFNLAKLPSHLHLKSLQYLGLSGCRKLENFP
TIAENLKSIKLLDLDFTAIKEVPPSTGYLTQLSRLNINGIVQEIPNLPDIIQKMDATGCESLARSPENLVDVISGKQDLTLGEISREFFL
TGIEIPEWFSYKSTTNSVTASIRHYPDMERTLAACVSFTVNGDSSTRGALISCNIFICNQLYCSFSRSFLPSKSEYMWLVTTSLARESME
VQDWNKVLIWFEAQDEVNVTMRSCGVHVTEELHGLQTDLNWPVVTFADFYQPEKLPDL
>MELO3C004309-4310

MGSSIVGVESSTSLSFKWSYDVFLSFKGDDTRSNFTSHLDMALRQKGVNVFIDDKLKRGEQISETLFKAIQETLISIVIFSQNYASSSWC
LDELVKIIECKKSKGQLVLPIFYKVDPSDVRKQTGCFGEALAKHQANFMEKTQIWRDALTTVANFSGWDLGTRKEADFIQDLVKEVLSRL
NCANGQLYVAKYPVGIDSQLEDMKLLSHQIRDVFDGVYMMGIYGIGGIGKTTLAKALYNKIANQFEGFCFLSNVREASKQFNGLVQLQEK
LLYEILKVDLKVDNLDEGINIIRSRLRSKKVLIVLDDVDKLKQLEALVGGRDWFGRGSKIIVTTRNSHLLSSHEFDEKYGIRELSHGHAL
ELFSWHAFKKSHPSSNYLDLSERATSYCKGHPLALAVLGSFLCTRDQTKWKTILDEFENSLSEDIEHIIQISFDGLEEKIKEIFLDISCL
FVGEKVNYVKSVLNTCHFSLDFGIIVLIDLSLITVENEEVQMHDLIRQMGQKIVNGESFEPGKRSRLWLVHDVLKVFADNSGTIAVKAIK
LDLSNPTRLDVDSQAFRNMKNLRLLIVRNAKFSTNVEYLPDSLKWIKWHGFSHRSLPLSFLKKNLVGLDLSHSFIKNLGKGFKDCKRLKH
GDLSYSSLLEKIPDFPATSNLEELYLNNCTNLRIIPKSVVSLGKLLTLDLDHCSNLIKLPSYLMLKSLKVLKLSYCKKLEKLPDFSTASN
LEKLYLKECTNLKMIHDSIGCLSKLVTLDLGKCSNLEKLPSYLTLKSLEYLNLAHCKKLEEVPDFSSALNLKSLYLEQCTNLRVIHESIG
SLNSLVTLDLRQCTNLEKLPSYLKLKSLTHFELSGCCKLEMFPKIAENMKSLMSLHLDSTAIRELPSSIGFLTALLLLNLNDFKCFPIDG
TQPPIQNCKFLQEIPNLPHCIQKLDATGCSLLGRSPDNIMDIISSKQDVALGDFTREFVLMNTGIPEWFKYQSISTSVRVSFRHDLNMER
TLATYATFQVVGDSHRGMALVSCKIFIGYRLQSCFMRKFPSSTSEYTWLVTTSSPTFSTSLEMNEWNHVTVWFEVVKCSEVTVTIKCCGV
HLTEEVHGIQNDVKGPGVIYTVFDQPDKLPSRDVVKSFAQEVSAKSDCNAILHAENFPVWNDSKMQRHMNFPLHVTSQGVTRIRGMEGMA
ETILANSICNKYERSQNLFSAKKVLNHSTAFLRGDGNGLSWEMVDSPISSDRLSSQKYLRIFDDRDRYGDLNDVACGTGNRFRSRFLRMD
DIKEDDIREEPRWKYMERSFQTDPIL
>MELO3C004311
MNLASGSSSSSRFRCSFDVFLSFRGEDTRSNFTSHLNMALRQRGINVFIDDKLSRGEEISASLLEAIEESKISIVIISENYASSSWCLNE
LEKIIMCNKLRWGVQLVLPIFYKVDPSQVRKQSGRFGEEFGKLEVRFSSDKMEAWREAMISVSHMSGWPILQNDDEANLIQEIVQEVLKK
LNRGTMLLRLPKYPVGIDRQVNNILFQVMSADEKITMIGIYGIGGIGKTTLAKALYNRIADDFEGCCFLAKIREASNQYDGLVQLQKKLL
CEILMDNSINVSNLDIGINIIRNRLCSKKILLILDDVDTREQLEVLAGGHDWFGPGSMVIATTRDKHLLAIHQFNILQSVQGLNDGYEAL
ELFSWHAFKRSCPSSDYLDLSKRAVRYCLGLPLALEVVGSFLFSTEQSKFKLILDEYENQYLDKGIQDPLRISYDGLENEGTTKLMNLSL
LTIDEHSNRIEMHDLIQQMGRTIHLSETSKSHKRKRLLIKDDVMDVLNGNKEARAVKVIKLNFPKPTELEIDSRAFEKVKKLVVLDIRNA
TSSRSSDLEYVPSSLRWMNWPHFPFSSLPSTYTMDNLMELKLPYSSIKHFGKAFMCGGCLKKINFRGSKFLVEIPDLSIAINLEELDLLG
CVNLVKIHESVGSLSKLVEFYLSSNIKGFEQFPSYLKLKSLKTLFLYRCRIDEWCPQFSEEMDSLEVPKGVLYMNATESASLARFPDNIL
DFISCYDNYAKRRYNPNVIKELILMNCDIPDWCQYKSTNNSITFLLPANHPTWERKVSIASCVKLQGIDKAFKVNSRVFINDFDVHLGQF
WKHEFEGGKGPRGEYLWIEVLDPYRFLYLYDHYEQNQPCFRIKSSRIIFDRITVLFEVITPNAVSIKKCGVHVIMEE
>MELO3C004312
MALRQRGINVFIDNKLSRGEEISASLLEAIEESKISIVVISENYASSSWCLNELVKILMCNELRGQVVLPIFYKVDPSQVGKQSGRFGEE
FAKLEVRFSSDKMEAWREAMISICHISGWTVLQKEDEANLIQKIVQEVLKKLNRGAIQLRVAKYPVGIDRQVNDILFHVMSADEKITMVG
LYGIGGMGKTTLAKALYNKIANDFEGCCFLANVREASNRYRGLVELQKELLREVLMDDSIKVSNVDIGISIIRDRLCSKKILLILDDVDT
REQLEALAGGHDWFGPGSMVIATTRNMPLLSSHGIFNKFKEVNGLNAIEGLELFSWHAFRNSDPSSDYLDLSKRAVHYCKGLSLALEVLG
SFLNSIDDQSKFERILDEYENFYLDKGIQDILRISYDELEQDEKISTKLKRREDINEVKTKLEACGCLCLEKGTTKLMNLSLLTIDEHSN
RIEMHDLIQQMGRTIHLLETSTSHKRKRLLIKDDVMDVLSGNKEARGVKVIKLDFPRPTQLDIDSRAFEKVRNLVVLEVGNATSSKSTDL
QYLPNSLRWINWPHFPFSSLPSTYTMDNLIQLKLPYNSIKHFGKAFMCGGCLKKINFRDSKFLVEIPDLSIAINLEKLDLLGCVNLVKIH
ESVGSLSKLVKFYLSSNIKGFEQFPSYLKLKSLKIFILYKCRIDEWCPQFSKEMDSLEVLVIHYSIVTNQLSPTIGYLTSLKFLSITDCM
ELKTLPRTIYRLKIPKVPKGVVTMNASGCVSLVKFPDNILDFISCDDNLEHAVHEEFRRLILMNCDIPDWCKYKSMNNSIEIFPADCLSS
KSRVFIAACAKFEVNIGDGEPNFLCSVFINDTEILHCEMLSSIGKITTKYDATQTPTGEYLSMVVLGSYIYPYRYGVVFYPYDVDRDPYG
DIMDSSGKVDLNQLCCRMKSSRGILDKVEVLFEVPQEFKDAISIKMCGVHVIVEE
>MELO3C004313
MALRQRGINVFIDDKLSRGEEISASLLEAIEESKISIVIISENYASSSWCLNELEKIIMCNKLRSGEQLVLPIFYRVDPSQVRKQSGRFG
EEFGKLEVRFSSDKMEAWREAMISVSHMSGWPVLQNDDEANLIQQIVQEVSKKLNRGILQLRLPKYPVGIDRQVNNILFQVMSADEKITM
VGLYGIGGIGKTTLAKALYNRIVDDFEGCCFLAKIREASNQYEGLVQLQKKLLCEILMDNSINVSNLDIGINIIRNRLCSKKILLILDDV
DTREQLEALAGRHDWFGPGSMVIATSRDKHLLAIHEFNIFQSVQGLKDDEALELFSWHAFKMSCPSSDYLYLSKRAVRYCDGLPLALEVV
GSFLYSIEQSKFKLILDEYENHYLDKGIQDPLRISYDGLEDEVKEIFLYISCCFVGKDINEVKIKLKACGCLCLEKGTTKLINLSLLTIV
GSNWIEMHDLIQQMGRTIHLSKTSKSHKRKRLLIKDDAMDVLNGNKEARAVKVIKIDFPQPTELDIDSRAFEKVKNLVVLEVENATSSKS
IDLEYVPNSLRWINWPHFPFSSLPSTYTMDNLIQFKLPYSSIKHFGKAFMCAEWLKEIDLSFSTFLEEIPDLTAAINLKKLDIGGCANLV
KVHESVGSLSKLVEFYLSSNIKGFEQFPTYLKLKSLKTLFSYRCRIDEWCPQFSEEMDSLEVLLIDDSTVINQLSPTIGYLTSLRELLIK
NCMELKTLPKIPKIPEGAVTMNASGCVSLARFPDNIPDFISCKFYEEVEYTKHGVFKNLVLMNCDIPDWCKYKSTNNSITFLFPVIDYLS
WKRKAFIAPCVKIQVTAYPFHLAYRVFINDFEVRKSFWMDDRSRGEYLWMQVLDPCVHFNPYGDDLNQPRFRINSSRGILDKITVLFEVI
TPEKWKYYYELLRYNFSRFNAVEKLGVSSNNSQQLVNCSEREAAPKIKYLKRSQEFPPITANNSDEHLQHFAILTHMEKVIKNTLAYWAL
KLTLMSTRAGKSF
>MELO3C004317
MDILISVIAKIAEYTVEPVGRQLGYVFFIRSNFQKLKTQVEKLKITRESVQHKIHSARRNAEDIKPAVEEWLKKVDDFVRESDEILANEG
GHGGLCSTYFVQRHKLSRKASKMVDEVLEMKNEGESFDMVSYKSVIPSVDCSLPKVPDFLDFESRKSIMEQIMDALSDGNVHRIGVYGMG
GVGKTMLVKDILRKIVESKKPFDEVVTSTISQTPDFRSIQGQLADTLGLKLEQETIEGRAPILRKRLKMERSILVVLDDVWENIDLETIG
IPSVEDHTGCKILFTTRNKHLISNQMCANKIFEIKVLGEDESWNLFKTMAGETVEASDLKPIAIQIARECAGLPIAITTVAKALRNKPSD
IWNDALNQLKSVDVGMANIGEMERKVYLPLKLSYDCLGYEEVKLLFLLCSMFPEDFPIDVEELHVYAMGMGFLHGVDTVEKGRCRIKKLV
DDLISSSLLQQYSEYGCNYVKMHDMVRDVALLIASQNDHIRILSYVKSLNEEWKEDRLSGNHTTVSIDGLHYPLPKLTFPKVQLLRLVAQ
SWWEHNESVSVVETFFEEMKELKGSIDWIGELKKLEILDFRGSNISQIPTTMSQLTQLKVLNLSFCEQLEVIPPNILSKLTKLEELNLET
FDGWEGEEWYEGRKNASLSELKCLRHLYALNLTIQDEEIMPENLFLVGKLKLQKFNIRIGCQSKLKYTFAYQNKNRIKNFIGIKMESGRC

LDDWIKNLLKRSDNVLLEGSVCSKVLHSELVGANNFVIEKEKSADHNMLESKQWETSSSSKDGVLRLGDGSKLFPNLKSLKLYGFVDYNS
THLPMEMLQILFQLEVFELEGAFIEEIFPSNILISSSMDLQSLILSKLPKLKHLWSEECSQNNITSVLQHLCSLGISDCGRLSSLVSSLV
CFTNLQHLHVNKCHRLTHLLNPSVATTLVQLEGLTVEECKRMSSVIEEGSTEEDGNDEMVVFSLGIVSTPRLKYENFYLKKDYDDERCHP
KYPKEMLVEDMNVMTREYWEDNVDTGIPNLFAEQSLEENRSENSSSSKNNVEKE
>MELO3C004318
MVHSSWFFSSSSIFVGFDPFQAQEQRMIMDILISVIAKIAEYTVEPVGRQLGYVFFIRSNFQKLKTQVEKLKITRESVQHKIHSARRNAE
DIKPAVEEWLKKVDDFVRESDEILANEGGHGGLCSTYLVQRHKLSRKASKMVDEVLEMKNEGESFDMVSYKSVIPSVDCSLPKEPDFLDF
ESTKSIMEQIMDALSDGNVHRIGVYGMGGVGKTMLVKDILRKIVESKKPFDEVVTSTISQTPDFRSIQGQLADTLGLKLEQETIEGRAPI
LRKRLKMERSILVVLDDVWENIDLETIGIPSVEDHTGCKILFTTRNKHLISNQMCANKIFEIKVLGEDESWNLFKTMAGETVEASDLKPI
AIQIARECAGLPIAITTVAKALRNKPSDIWNDALDQLKSVDVGIANIGEMERRVYLPLKLSYDYLGYEEVKLLFLLCSMFPEDFTIDEEE
LHVYAIGMGFLHGVNTVEKVIEKEKSADHNMLESKQWETSSSSKDGVLRLGDGSKLFPNLKSLKLYGFVDYNSTHLPMEMLQILFQLEVF
ELEGAFIEEIFPSNILISSSMDLQSLTLSKLPKLKHLWSEECSQNNITSVLQHLTDVSISECGGLSSLVSSLVCFTNLKDLHVIKCHRLT
HLLNPSMATTLVQLEYLTIEECKRMSSVIEGGSTEEDGNDEMVVFSLGIVSTPRLKYEKFTLMNDYDDKWCHPKYPKDMLVEDMNVITRE
YWEDNVDTGIPNLFAEQSLEENRSENSSSSKNNVEKE
>MELO3C004319
MDILISVIAKIAEYVGRQLGYLFFIRSNFQKLKTQVEKLKITKEFVKHKIHAARRNAEDIKPAVEEWLKKSKKPYDEVVPSTISQTPDFK
SIQGQLADKLGIPGVEDHTGCKILLTSTNKHLISNQMCTNKIFEIKVLGEDESWNLFKAMAGEIVEASDLNPIAIQIVRECACLPIAITT
VAKALRNKPSDIWIDALDQLKSVDVGMANIGQMDKKVYLSLKLSYDGLGYEEVMGFLHGVDTVVKGRRRIKKLVDDLISSSLLQQYSEYG
CNYVKMHDMVRDVALLIASKNDHIRTLSYVKRWNEEREEERLSSNHTIVSIHGLNYPLSKLMLPKVQLLRLDGQLRLEGQWLNNKYVSVV
ETFFEEMQELNGLELKMVKISLSPPSPYTFANIRLLCLHECELGSIDMIGELKKLEVLDFSESNITQIPSTMSQLTKLKVLNLSSCYALK
IIPPNILSKLTKLEELSLETFDRWEGEKCEGRKNASLSELRYLPHLYALKLTIQGKEITPKDLFSRELNLENSTLLLVFG
>MELO3C004320
MDILISVTAKIAEYTVEPVLRQLRYVFFIRSNFRELKTQIEKLKITRESVLHNIHYARRNAEDIKPAVEEWLKKVNDIVGKSEEILAYEG
GHGKLCSTNLVQRHKLSRKASKMAYEVGEMNTEGKSFDTVSYKIVIPSVGCSPTKVPDFLDFDSRKSIVKQIMDALSEDNVHRIGVHGMG
GVGKTMLVNEILRKIGESKKLFDEVVTSTISQTSDFKRIQGELADKLGLKFEQETIKGRASILEKRLKMERSILVVLDDVWENIDLKDIG
IPSVEDHTGCKILFTTRNKDLISNQMCANKIFEIKVLGEDESWNLFKTMAGEIVEARDLKPIAIQIVRECAGLPIAITTVAKALRNKPSD
IWNDALNQLKSVDVGIANIGEMERRVYLPLKLSYDYLGYEEVKLLFLLCSMFPEDFTIDEEELHVYAIGMGFLHGVNTVEKVRCRIKKLV
EDLISSSLLQQYSEYGCNYVKMHDMIRDVALSIASKNEHVRTLSYVKRSNEEWEEEKLSGNHTAVFIDGLHYPLPKLTLPKVQLLRLVGQ
SWEHKFVSVVETLFEEMKELKGSIDWIGELKKLEILDFSESNITQIPTTMSQLTQLKVLNLSSCEELEVIPPNILSKLTKLEELNLETFD
RWEGEEWYEGRKNASLSELKCLRHLYALNLTIQDEEIMPKDLFLAEELKLQKFNICIGCQSMYTFGPPNRIKNFIAMEMESGRCLDDWIK
NLLKRSDNVCLKGSICSKVLHSELVVANACELVAVLSWEAASSVAASRSCLTWNTHPPHHRHVTGSGCPFDWSEVVNLLNNKAIEDLMEM
ALVMVRSLHLHSSSLHV
>MELO3C004321
MVHSSWVFSSSSIFVGFDPFQAQEQKIIMDILISVTAKIAEYTVKPVGRQLGYVFFIHSNFQKLKTQVEKLKITRESVQHKIHSARRNAE
DIKPAVEEWLKKVDDFVRESDEILANEGGHGRFCSSNLIQRHKLSRKASQKAYEVLEMKNEGESFDTVSNKNVIPLVDCSLPKVPDFLDF
DSRQSIVKQIMDALSDDNVHRIGVYGMGGVGKTMLVKDILRKIVESKKPFDEVVLSTVSQTPDFRSIQGQLADKLGLKFEQETIEGRATI
LRKRLKMERSILVVLDDVWEYIDLETIGIPSVEDHTGCKILFTTRIKHLISNQMCANKIFEIKVLGKDESWNLFKAMAGDIVDASDLKPI
AIRIVRQCAGLPIAITTVAKALRNKPSDIWNDALNQLKSVDVGMANVGEMEKKVYLSLKLSYDCLGYEEVKLLFLLCSMFPEDFPIDVQE
LHVYAMGMGFLHGVDTVVKGRCRIKKLVDDLISSSLLQQYSEYGCNYVKMHDMVRDVALLIASKNEHVRTLSYVKRSNEEWEEDKLLGNH
TAVFIDGLHYPLPKLTLPKVQLLRLVAQYCWEHNKRVSVVETFFEEMKELKGLVVENVNISLMQRPSDLYSLANIRVLRLQRCQLLGSID
WIGELKKLEILDFSESNITQIPTTMSQLTQLKVLNLSSCEELEVIPPNILSKLTKLEELNLETFDRWEGEEWYEGRKNASLSELKCLRHL
YALNLTIQDEEIMPKDLFLAEELKLQKFNICIGCQSKLKYTFESTNRIKNFIAIKMESGRCLDDWIKNLLKRSDNVHLEGSICSKFLHLE
LVGANDFVNLK
>MELO3C004323
MDILISVIAKIAEYTVEPLDANLTQVEKLKITKESVKHKIHAARRNAEDIKPAVEERLKKVDDFVRESDEILAHEGGHGRLCSTYLVQRH
KLNRKASKMVDEVLEMKNEGESFDTVSYKSVISSVDCSPSKVPDFLDFESIVEQIMDAFSDDNIHRIGVYGMGGVCKTMLVKEILRKIVE
SKKPCDEVVPSTISQTPDFKSIQGQLADKLGLKFEQETIEGRARILQKRLKMERSILVVLDDVWEYIDLETIGIPGEIVEASDLKPIAIQ
IVRECAGLPIAITTVAKALRNKPSDIWIDALDQLKSVDVGMANIGQMDKKVYLSLKWSYDSLGYEEVKLLFLLCSICFQKTLTLTWKSCM
FMQ
>MELO3C004324
MDILISVTAKIAEYTVEPVGRQLGYVFFIHANFKKLKTQVEILKDTKEYVQQNIRTARRNVEDIKPAVEKWLKKVDDIVGKSEEILAYEG
GHGRLCSTDLVQRHNLSRKASKMAYEVLEMNTEGKSFDTVSYKIVIPSVDCSPPKVPDFLDFDSRKSIVEQIMDALSEDNVHRIGVHGMG
GVGKTMLVKEILRKIGESKKLFDEVVTCTISQTPDFKTIQGQLADKLGLKFQQETIEGRAPILRKRLKMERSILVVLDDIWEYIDLEIIG
IPSVEDHAGCKILFTSRNKHLISNEMCANKFFEIKVLGEDESWNLFKAMAGEIVEASDLKPIVIQIVRECAGLPIAITTVARALRNKPSD
IWNDALDQLKSVDVGMANIGEMDKKVYLSLKLSYDCLGYEEVKLLFLLCSMFPEDFDIDMEELHVYAIGMGFLHGVDTVLKGRRRIKKLV
DDLISSSLLQQYSEYGRNYVKMHDMVRDVALLIASKNDHIRTLSYVKRPNEEWEEERLSGNHTAVFIYGLHYPLPKLTLPKVQLLRFVGQ
WMEDKRVPVVETLFEEMKELKGLVLENVNISLMQRPSDLYSLANIRVLRLQECGLESIDMIGELKKLEILDFSKSNITQIPTTMSQLTQL
KVLNLSSCNQLKVIPPNILSKLTKLEELSLETFDRWEGEEWYEGRENASLSELKCLPHLYALNLTIQDEEIMPPSS
