# Supplementary material for: Interspecific and intraspecific gene variability in a 1-Mb region containing the highest density of NBS-LRR genes found in the melon genome
Source: BMC Genomics. 2014 Dec 17;15(1):1131. doi: 10.1186/1471-2164-15-1131 (PMC4378003; doi:10.1186/1471-2164-15-1131)
Supplement: Supplementary file 4 — Additional file 4: File S3: NBS domains of the predicted TIR- and CC-NBS-LRR proteins in the improved MELO3C004258-MELO3C004324 region. (PDF 18 KB) [file 12864_2014_6878_MOESM4_ESM.pdf]

>MELO3C004258
ESDTGIGKTTLAKALYNKIASQFEGCCFLSNVREASKRFNGLAQLQESLL
YEILMDDLKVVNLDRGINIIRNRLHSKKVFIVLDDVDKLEQLEALVGGRD
WFGQGSRIIVTTRNKHLLNSHGFDEMHNVRGLNQDKAIELFSWHAFKKCC
PSSNCVDLSKRATSYCKGHPLTLVVLGSFLCTRDQAKWSSILDEFENSLN
KDIRDILRLSFDGLEDKVKDIFLDISCLLVGEKVKYVKNMLSACHVN
>MELO3C004259
EELVSHIGFEGVNMVGMYGIGGIGKTTLAKALYNKIATQFEGSCFLLDVR
REASKHGLIQLQKTLLNEILKEDLKVVNCDKGINIIRSRLCSKKVLIVLD
DVDHRDQLEALVGERDWFCQGSKIIVTTRNKHLLSSHGFDEIHNILGLNE
DKAIELFSWHAFKKNHPSSNYFDLSERVTSYCKGHPLALVVLGSFLCNRD
QVEWCSILDEFENSLNKDIKDILQLSFDGLEDKVKDIFLDISCLLVGEKV
E
>MELO3C004260
TGVYLVGIYGIGGIGKTTLAKALYNKIASQFEGCCFLSNVREASKQFNGL
AQLQESLLYEILTVDLKVVNIDRGINIIRNRLCSKKVLIVLDDVDKLEQL
ETLVGGRDWFGQGSRIMVTTRNKHLLSSHGFDEIHNILGLNEDKAIELFS
WHAFKKNSPSNLSKRATSYCKGHPLALVVLGSFLCTRDQVEWCSILDEFE
NSLNKDIKDILQLSFDGLEDKIKDIFLDISCLLVGEKVKYV
>MELO3C004262
QHEYEFDTGIYMVGIYGIGGIGKTTLAKALYNKIASQFEGCCFLSNVREA
SKQFNGLAQLQESLLYEILMVDLKVVNLDRGINIIRNRLCSKKVLIVLDD
VDKLEQLEALVGGCDWFGKGSRIIVTTRNKHLLFSHGFDEIHNILGLNED
KAIELFSWHAFKKNRPSSNYLDLSKRATSYCKGHPLALVVLGSFLCIRDQ
AEWCSILDEFENSLNKDIKDILQLSFDGLEDKIKDIFLDISCLLVGEKVE
YVKDMLGACH
>MELO3C004266
QTQHEYEFDTDVNMVGIYGIGGIGKTTLAKALYNKIASQFEGCCFLSNVR
EASKQFNGLAQLQESLLYEILTIYLKVVNFDRGINIIRNRLCSKKVLIVL
DDVDKLEQLEALVGGRDWFGQGSRIIVTTRNKHLLSSHGFDEIHNILGLN
EEKAIELFSWHAFKKNHPSSNYLNLSKRATSYCRGHPLALVVLGSFLCTR
DQVEWCSILDEFENSLNKDIKDILQLSFDGLEDKVKHIFLDISCLLVGEK
VKYVKNMLSACHVN
>MELO3C004288
IDIQVRNLLPHVMSNGTTMVGLYGIGGMGKTTLAKALYNKIADDFEGCCF
LPNIREASNQYGGLVQLQRELLREILVDDSIKVSNLPRGVTIIRNRLYSK
KILLILDDVDTREQLQALVGGHDWFGHGSKVIATTRNKQLLVTHGFDKMQ
SVVGLDYDEALELFSWHCFRNSHPLNDYLELSKRAVDYCKGLPLALEVLG
SFLHSIDDPFNFKRILDEYEKYYLDKEIQDSLRISYDGLEDEGITKLMNL
SLLTIGRFNRVEMHDIIQQMGRTIH
>MELO3C004289
IDSKIEFLYRKEEMYKSECVNMLGIYGIGGIGKTTLAKALYDKMASQFEG
CCYLRDVREASKLFDGLTQLQKKLLFQILKYDLEVVDLDWGINIIKNRLR
SKKVLILLDDVDKLEQLQALVGGHDWFGQGTKIIVTTRNKQLLVSHGFDK
MYEVQGLSKHEAIELFRRHAFKNLQPSSNYLDLSERATRYCTGHPLALIV
LGSFLCDRSDLAEWSGILDGFENSLRKDIKDILQLSFDGLEDEVKEIFLD
ISCLLVGKRVSYVKKMLSECHSI
>MELO3C004290
IDRQINNILFQVTSDEKITMVGFYGIGGIGKTTLAKALYNKIANDFEGCC
FLANVREASNQYRGLVELQKELLREILMDDLIKFSNLDVGISIIRDRLCS
KKILLILDDVDTSEQLEALVGEHDSFGPGSMVIVTTRNKHVLVIHEFDIL
QSVQGLKDDEALKLFSWHAFKQSCPSSDYLDLSKRAVRYCDGLPLALEVV
GSFLHSIEQSKFKLILDEYENQYLDKGIQDLLRISYDGLEDEVKEIFLYI
SCCFVGEDINEVKTKL
>MELO3C004291
IDKQVNNIHFQVMSTDEKTTMVGLYGIGGIGKTTLAKALYNRIADDFEGC
CFLPKIREASNQYDGLVQLQKKLLCEILMDNSININNLDIGINIIRNRLC
SKKILLILDDVDTREQLEALAGGHDWFGHGSKVIATTRNKQLLASHGFNK
LEKVNELNVIEGLELFSWHAFRNSHPSSDYLDLSKRVVRYCDGLPLALEV
VGSFLYSIEQSKFKLILDEYETQYLDKGIQDPLQISYDGLEDEVKEIFLY

ISCCFVGEDINEVKKKLKACGCLC
>MELO3C004292
INIQVNNLLLHVMPNGVTMVGLYGIGGMGKTTLAKALYNRISDDFEGCCF
LANVREASNQHWGLVELQKALLRKILMDDSIKISNIGIGISTIRDLLCSK
KILLVLDDVDTHEQLQALAGGHHWFGHGSKVIATTRNKQLLASHGFNILR
RVNGLNAIEGLELFSWHAFKNSHPSSDYLHLSKHAVHYCKGLPLALEVLG
SFLNSIDDQSKFKHILDEYENSYLDKDIQDILRISYDELEQDVKEIFLYI
SCCFVNEDKNKVQMMLQACDCHFRLE
>MELO3C004294
GIDRQVKDLLSHVIIDETRMVGLYGIGGMGKTTLAKALYNRVADKFEGCC
FLANIREASKQHDGLVRLQEKLLYDILMYDFVRVGDVYKGINIIRNRLYS
IRILLILDDIDTSEQLQVLAGGYDWFGHGSKVIVTTRNEQLLDIHGFYKL
KEVPQLHFGEALELFSWHAFHNSCPPSEYSTLPEDAVNYCKNLPLALEVL
GSFLYSTDQSKFKGILEEFANSNLNKDIQKLLQSCGCLCWENGIKKLMNL
SL
>MELO3C004301
RLRKIEELVSHIGSEGVNMVGMYGIGGIGKTTLAKALYNKIANQFEGCCF
LQDVRREASKHGLVKLQETLLNDILKEDLKVVSRDRGINIIRSRLCSKKV
LIVLDDVDDREQLEALVGGRDWFGRGSKIIVTTRNEHLLFSHGFDDQKHK
IQELNQDHALELFSWHAFKKSHPSSNYLGLSERATNYCKGLSLALVVLGS
FLRGRDQAEWNCILDEFETSLRKDIKDVLQLSFDGLEDKAKDIFLDISCL
LVGEEYNCAKKMLSACHLNIDFGI
>MELO3C004302-3
NLGAYSQFEGCCFLSNVRQASKQFNGLVQLQENLLYEILKDDLKFVNLDR
GITIIRSRLHSKKVLIVLDDVDKLEQLEALVGGRDWFGQGSKIIVTTRNR
HLLSSHGFDEMHNIRGLYQDKAIKLFSWHAFKESHPSSNYLGLVERATSY
CKGHPLALVVLGSFLCTRDQTEWISILDEFENSLSNNIKDILQLSFDGLE
DRVKDIFLDISCLLVGEEVNYVKNILSACHLNV
>MELO3C004309-10
EDMKLLSHQIRDVFDGVYMMGIYGIGGIGKTTLAKALYNKIANQFEGFCF
LSNVREASKQFNGLVQLQEKLLYEILKVDLKVDNLDEGINIIRSRLRSKK
VLIVLDDVDKLKQLEALVGGRDWFGRGSKIIVTTRNSHLLSSHEFDEKYG
IRELSHGHALELFSWHAFKKSHPSSNYLDLSERATSYCKGHPLALAVLGS
FLCTRDQTKWKTILDEFENSLSEDIEHIIQISFDGLEEKIKEIFLDISCL
FVGEKVNYVKSVLNTCH
>MELO3C004311
IDRQVNNILFQVMSADEKITMIGIYGIGGIGKTTLAKALYNRIADDFEGC
CFLAKIREASNQYDGLVQLQKKLLCEILMDNSINVSNLDIGINIIRNRLC
SKKILLILDDVDTREQLEVLAGGHDWFGPGSMVIATTRDKHLLAIHQFNI
LQSVQGLNDGYEALELFSWHAFKRSCPSSDYLDLSKRAVRYCLGLPLALE
VVGSFLFSTEQSKFKLILDEYENQYLDKGIQDPLRISYDGLENEGTTKLM
NLSLLTIDEHSNRIEMHDLIQQMGRTIHL
>MELO3C004312
IDRQVNDILFHVMSADEKITMVGLYGIGGMGKTTLAKALYNKIANDFEGC
CFLANVREASNRYRGLVELQKELLREVLMDDSIKVSNVDIGISIIRDRLC
SKKILLILDDVDTREQLEALAGGHDWFGPGSMVIATTRNMPLLSSHGIFN
KFKEVNGLNAIEGLELFSWHAFRNSDPSSDYLDLSKRAVHYCKGLSLALE
VLGSFLNSIDDQSKFERILDEYENFYLDKGIQDILRISYDELEQDEKIST
K
>MELO3C004313
IDRQVNNILFQVMSADEKITMVGLYGIGGIGKTTLAKALYNRIVDDFEGC
CFLAKIREASNQYEGLVQLQKKLLCEILMDNSINVSNLDIGINIIRNRLC
SKKILLILDDVDTREQLEALAGRHDWFGPGSMVIATSRDKHLLAIHEFNI
FQSVQGLKDDEALELFSWHAFKMSCPSSDYLYLSKRAVRYCDGLPLALEV
VGSFLYSIEQSKFKLILDEYENHYLDKGIQDPLRISYDGLEDEVKEIFLY
ISCCFVGKDINEVKIKLKACGCLC
>MELO3C004317
RKSIMEQIMDALSDGNVHRIGVYGMGGVGKTMLVKDILRKIVESKKPFDE
VVTSTISQTPDFRSIQGQLADTLGLKLEQETIEGRAPILRKRLKMERSIL
VVLDDVWENIDLETIGIPSVEDHTGCKILFTTRNKHLISNQMCANKIFEI

KVLGEDESWNLFKTMAGETVEASDLKPIAIQIARECAGLPIAITTVAKAL
RNKPSDIWNDALNQLKSVDVGMANIGEMERKVYLPLKLSYDCLGYEEVKL
LFLLCSMFPEDFPIDVEELHVYAMGMGFLHGVDT
>MELO3C004318
TKSIMEQIMDALSDGNVHRIGVYGMGGVGKTMLVKDILRKIVESKKPFDE
VVTSTISQTPDFRSIQGQLADTLGLKLEQETIEGRAPILRKRLKMERSIL
VVLDDVWENIDLETIGIPSVEDHTGCKILFTTRNKHLISNQMCANKIFEI
KVLGEDESWNLFKTMAGETVEASDLKPIAIQIARECAGLPIAITTVAKAL
RNKPSDIWNDALDQLKSVDVGIANIGEMERRVYLPLKLSYDYLGYEEVKL
LFLLCSMFPEDFTIDEEELHVYAIGMGFLHGVNT
>MELO3C004319
IQGQLADKLGIPGVEDHTGCKILLTSTNKHLISNQMCTNKIFEIKVLGED
ESWNLFKAMAGEIVEASDLNPIAIQIVRECACLPIAITTVAKALRNKPSD
IWIDALDQLKSVDVGMANIGQMDKKVYLSLKLSYDGLGYEEVNYYSCYAA
CFQKTLALTWKSCMFMQCAYGFLTWC
>MELO3C004320
RKSIVKQIMDALSEDNVHRIGVHGMGGVGKTMLVNEILRKIGESKKLFDE
VVTSTISQTSDFKRIQGELADKLGLKFEQETIKGRASILEKRLKMERSIL
VVLDDVWENIDLKDIGIPSVEDHTGCKILFTTRNKDLISNQMCANKIFEI
KVLGEDESWNLFKTMAGEIVEARDLKPIAIQIVRECAGLPIAITTVAKAL
RNKPSDIWNDALNQLKSVDVGIANIGEMERRVYLPLKLSYDYLGYEEVKL
LFLLCSMFPEDFTIDEEELHVYAIGMGFLHGVNT
>MELO3C004321
RQSIVKQIMDALSDDNVHRIGVYGMGGVGKTMLVKDILRKIVESKKPFDE
VVLSTVSQTPDFRSIQGQLADKLGLKFEQETIEGRATILRKRLKMERSIL
VVLDDVWEYIDLETIGIPSVEDHTGCKILFTTRIKHLISNQMCANKIFEI
KVLGKDESWNLFKAMAGDIVDASDLKPIAIRIVRQCAGLPIAITTVAKAL
RNKPSDIWNDALNQLKSVDVGMANVGEMEKKVYLSLKLSYDCLGYEEVKL
LFLLCSMFPEDFPIDVQELHVYAMGMGFLHGVDT
>MELO3C004323
FESIVEQIMDAFSDDNIHRIGVYGMGGVCKTMLVKEILRKIVESKKPCDE
VVPSTISQTPDFKSIQGQLADKLGLKFEQETIEGRARILQKRLKMERSIL
VVLDDVWEYIDLETIGIPGEIVEASDLKPIAIQIVRECAGLPIAITTVAK
ALRNKPSDIWIDALDQLKSVDVGMANIGQMDKKVYLSLKWSYDSLGYEEV
KLLFLLCSICFQKTLTLTWKSCMFM
>MELO3C004324
RKSIVEQIMDALSEDNVHRIGVHGMGGVGKTMLVKEILRKIGESKKLFDE
VVTCTISQTPDFKTIQGQLADKLGLKFQQETIEGRAPILRKRLKMERSIL
VVLDDIWEYIDLEIIGIPSVEDHAGCKILFTSRNKHLISNEMCANKFFEI
KVLGEDESWNLFKAMAGEIVEASDLKPIVIQIVRECAGLPIAITTVARAL
RNKPSDIWNDALDQLKSVDVGMANIGEMDKKVYLSLKLSYDCLGYEEVKL
LFLLCSMFPEDFDIDMEELHVYAIGMGFLHGVDT
