# Supplementary material for: Interspecific and intraspecific gene variability in a 1-Mb region containing the highest density of NBS-LRR genes found in the melon genome
Source: BMC Genomics. 2014 Dec 17;15(1):1131. doi: 10.1186/1471-2164-15-1131 (PMC4378003; doi:10.1186/1471-2164-15-1131)
Supplement: Supplementary file 6 — Additional file 6: File S4: BLASTP alignment of the Vat (PI 161376 melon variety) and MELO3C004317 (DHL92 melon variety) protein sequences. (PDF 21 KB) [file 12864_2014_6878_MOESM6_ESM.pdf]

```
BLASTP
Query: Vat, PI 161376 melon variety
Subject: MELO3C004317 gene, DHL92 melon variety

> MELO3C004317
Length=1044

 Score = 1933 bits (5008),  Expect = 0.0, Method: Compositional matrix adjust.
 Identities = 951/1026 (93%), Positives = 978/1026 (96%), Gaps = 2/1026 (0%)

Query  1     MDILISVTAKIAEYTVEPVGRQLGYVFFIRSNFQKLKTQVEKLKITRESVQHKIHSARRN  60
             MDILISV AKIAEYTVEPVGRQLGYVFFIRSNFQKLKTQVEKLKITRESVQHKIHSARRN
Sbjct  1     MDILISVIAKIAEYTVEPVGRQLGYVFFIRSNFQKLKTQVEKLKITRESVQHKIHSARRN  60

Query  61    AEDIKPAVEEWLKKVDDFVRESDEILANEGGHGGLCSTYLVQRHKLSRKASKMVDEVLEM  120
             AEDIKPAVEEWLKKVDDFVRESDEILANEGGHGGLCSTY VQRHKLSRKASKMVDEVLEM
Sbjct  61    AEDIKPAVEEWLKKVDDFVRESDEILANEGGHGGLCSTYFVQRHKLSRKASKMVDEVLEM  120

Query  121   KNEGESFDMVSYKSVIPSVDCSLPKVPDFLDFESRKSIMEQIMDALSDGNVHRIGVYGMG  180
             KNEGESFDMVSYKSVIPSVDCSLPKVPDFLDFESRKSIMEQIMDALSDGNVHRIGVYGMG
Sbjct  121   KNEGESFDMVSYKSVIPSVDCSLPKVPDFLDFESRKSIMEQIMDALSDGNVHRIGVYGMG  180

Query  181   GVGKTMLVKDILRKIVESKKPFDEVVTSTISQTPDFRSIQGQLADKLGLKFEQETIEGRA  240
             GVGKTMLVKDILRKIVESKKPFDEVVTSTISQTPDFRSIQGQLAD LGLK EQETIEGRA
Sbjct  181   GVGKTMLVKDILRKIVESKKPFDEVVTSTISQTPDFRSIQGQLADTLGLKLEQETIEGRA  240

Query  241   TILRKRLKMERSILVVLDDVWEYIDLETIGIPSVEDHTGCKILFTTRIKHLISNQMCANK  300
              ILRKRLKMERSILVVLDDVWE IDLETIGIPSVEDHTGCKILFTTR KHLISNQMCANK
Sbjct  241   PILRKRLKMERSILVVLDDVWENIDLETIGIPSVEDHTGCKILFTTRNKHLISNQMCANK  300

Query  301   IFEIKVLGKDESWNLFKAMAGDIVDASDLKPIAIRIVRECAGLPIAITTVAKALRNKPSD  360
             IFEIKVLG+DESWNLFK MAG+ V+ASDLKPIAI+I RECAGLPIAITTVAKALRNKPSD
Sbjct  301   IFEIKVLGEDESWNLFKTMAGETVEASDLKPIAIQIARECAGLPIAITTVAKALRNKPSD  360

Query  361   IWNDALDQLKTVDVGMANIGEMEKKVYLSLKLSYDCLGYEEVKLLFLLCSMFPEDFSIDV  420
             IWNDAL+QLK+VDVGMANIGEME+KVYL LKLSYDCLGYEEVKLLFLLCSMFPEDF IDV
Sbjct  361   IWNDALNQLKSVDVGMANIGEMERKVYLPLKLSYDCLGYEEVKLLFLLCSMFPEDFPIDV  420

Query  421   EGLHVYAMGMGFLHGVDTVVKGRRRIKKLVDDLISSSLLQQYSEYGCNYVKMHDMVRDVA  480
             E LHVYAMGMGFLHGVDTV KGR RIKKLVDDLISSSLLQQYSEYGCNYVKMHDMVRDVA
Sbjct  421   EELHVYAMGMGFLHGVDTVEKGRCRIKKLVDDLISSSLLQQYSEYGCNYVKMHDMVRDVA  480

Query  481   LLIASKNEHVRTLSYVKRSNEEWEEEKLLGNHTAVFIDGLHYPLPKLTLPKVQLLRLVAK  540
             LLIAS+N+H+R LSYVK  NEEW+E++L GNHT V IDGLHYPLPKLT PKVQLLRLVA+
Sbjct  481   LLIASQNDHIRILSYVKSLNEEWKEDRLSGNHTTVSIDGLHYPLPKLTFPKVQLLRLVAQ  540

Query  541   YCWEHNKRVSVVETFFEEMKELKGSIDWIGELKKLEILDFSESNITQIPTTMSQLTQLKV  600
               WEHN+ VSVVETFFEEMKELKGSIDWIGELKKLEILDF  SNI+QIPTTMSQLTQLKV
Sbjct  541   SWWEHNESVSVVETFFEEMKELKGSIDWIGELKKLEILDFRGSNISQIPTTMSQLTQLKV  600

Query  601   LNLSSCEQLEVIPPNILSKLTKLEELDLETFDGWEGEEWYEGRKNASLSELKCLRHLYAL  660
             LNLS CEQLEVIPPNILSKLTKLEEL+LETFDGWEGEEWYEGRKNASLSELKCLRHLYAL
Sbjct  601   LNLSFCEQLEVIPPNILSKLTKLEELNLETFDGWEGEEWYEGRKNASLSELKCLRHLYAL  660

Query  661   NLTIQDEEIMPENLFLVGKLKLQKFNICIGCESKLKYTFAY--KNRIKNFIGIKMESGRC  718
             NLTIQDEEIMPENLFLVGKLKLQKFNI IGC+SKLKYTFAY  KNRIKNFIGIKMESGRC
Sbjct  661   NLTIQDEEIMPENLFLVGKLKLQKFNIRIGCQSKLKYTFAYQNKNRIKNFIGIKMESGRC  720

Query  719   LDDWIKNLLKRSDNVLLEGSVCSKVLHSELVGANNFVIEKEKSAHHNMLESKQWETSSSS  778
             LDDWIKNLLKRSDNVLLEGSVCSKVLHSELVGANNFVIEKEKSA HNMLESKQWETSSSS
```

```
Sbjct  721   LDDWIKNLLKRSDNVLLEGSVCSKVLHSELVGANNFVIEKEKSADHNMLESKQWETSSSS  780

Query  779   KDGVLRLGDGSKLFPNLKSLKLYGFVDYNSTHLPMEMLQILFQLVVFELEGAFLEEIFPS  838
             KDGVLRLGDGSKLFPNLKSLKLYGFVDYNSTHLPMEMLQILFQL VFELEGAF+EEIFPS
Sbjct  781   KDGVLRLGDGSKLFPNLKSLKLYGFVDYNSTHLPMEMLQILFQLEVFELEGAFIEEIFPS  840

Query  839   NILIPSYMVLRRLALSKLPKLKHLWSEECSQNNITSVLQHLISLRISECGRLSSLLSSIV  898
             NILI S M L+ L LSKLPKLKHLWSEECSQNNITSVLQHL SL IS+CGRLSSL+SS+V
Sbjct  841   NILISSSMDLQSLILSKLPKLKHLWSEECSQNNITSVLQHLCSLGISDCGRLSSLVSSLV  900

Query  899   CFTNLKHLRVYKCDGLTHLLNPSVATTLVQLESLTIEECKRMSSVIEGGSTEEDGNDEMV  958
             CFTNL+HL V KC  LTHLLNPSVATTLVQLE LT+EECKRMSSVIE GSTEEDGNDEMV
Sbjct  901   CFTNLQHLHVNKCHRLTHLLNPSVATTLVQLEGLTVEECKRMSSVIEEGSTEEDGNDEMV  960

Query  959   VFSLGIVSTPRLKYENFSLKNDYDDERCHPKYPKDMLVEDMNVITREYWEDNVDTGIPNL  1018
             VFSLGIVSTPRLKYENF LK DYDDERCHPKYPK+MLVEDMNV+TREYWEDNVDTGIPNL
Sbjct  961   VFSLGIVSTPRLKYENFYLKKDYDDERCHPKYPKEMLVEDMNVMTREYWEDNVDTGIPNL  1020

Query  1019  FAEQSLEENRSENSSSSKNNVEKE  1042
             FAEQSLEENRSENSSSSKNNVEKE
Sbjct  1021  FAEQSLEENRSENSSSSKNNVEKE  1044
```
